# ASAH1 facilitates TNBC by DUSP5 suppression-driven activation of MAP kinase pathway and represents a therapeutic vulnerability

**DOI:** 10.1038/s41419-024-06831-2

**Published:** 2024-06-26

**Authors:** Kiran Kumar Reddi, Suresh Chava, Siva Chander Chabattula, Yvonne J. K. Edwards, Kamaljeet Singh, Romi Gupta

**Affiliations:** 1 Department of Biochemistry and Molecular Genetics, The University of Alabama at Birmingham, Birmingham, AL USA; 2 Department of Pathology and Laboratory Medicine, Brown University, Providence, RI USA; 3 O’Neal Comprehensive Cancer Center at The University of Alabama at Birmingham, Birmingham, AL USA

**Keywords:** Breast cancer, Targeted therapies

## Abstract

Triple-negative breast cancer (TNBC) is a subtype of breast cancer that is prone to metastasis and therapy resistance. Owing to its aggressive nature and limited availability of targeted therapies, TNBC is associated with higher mortality as compared to other forms of breast cancer. In order to develop new therapeutic options for TNBC, we characterized the factors involved in TNBC growth and progression. Here, we demonstrate that N-acylsphingosine amidohydrolase 1 (ASAH1) is overexpressed in TNBC cells and is regulated via p53 and PI3K-AKT signaling pathways. Genetic knockdown or pharmacological inhibition of ASAH1 suppresses TNBC growth and progression. Mechanistically, ASAH1 inhibition stimulates dual-specificity phosphatase 5 (DUSP5) expression, suppressing the mitogen-activated protein kinase (MAPK) pathway. Furthermore, pharmacological cotargeting of the ASAH1 and MAPK pathways inhibits TNBC growth. Collectively, we unmasked a novel role of ASAH1 in driving TNBC and identified dual targeting of the ASAH1 and MAPK pathways as a potential new therapeutic approach for TNBC treatment.

## Introduction

Triple-negative breast cancer (TNBC) lacks hormone receptors (HR) and human epidermal growth receptors (HER2) expression and exhibits a heightened propensity for metastasis, recurrence, and significantly reduced overall patient survival as compared with other breast cancer subtypes [1, 2]. The lack of expression of HER2 and HR receptor markers limits the availability of TNBC-targeted therapies, and current treatments rely only on standard chemotherapeutic regimens that provide limited clinical benefits [3–5]. Thus, a comprehensive understanding of TNBC is imperative to develop more effective therapies to treat this breast cancer subtype.

Cancer cells display distinct metabolic pathways to promote cell proliferation, survival, and spread [6]. Metabolic deregulation in cancer cells not only drives tumor development and progression [7, 8] but also influences the efficacy and outcome of anticancer therapies [9, 10]. Deregulation of several metabolic pathways is as a key driver of cancer development and progression among different breast cancer subtypes, including TNBC [11–13]. Given the importance of metabolic enzymes and pathways in tumor development and growth, they have become promising therapeutic targets for cancer therapy [14]. Several metabolic enzyme inhibitors are currently under clinical consideration and development for the treatment of different cancer types [15].

Sphingolipids, a class of sphingosine-based lipids, play an important role in cell survival [14, 16, 17]. Ceramide and sphingosine, which are both interconvertible sphingolipid metabolites, are involved in cell survival [18]. Ceramide suppresses tumor growth by inducing cell cycle arrest and apoptosis [19], whereas sphingosine promotes tumor growth by regulating important cancer cell processes, including angiogenesis, cell survival, proliferation, migration, and inflammation [20]. The maintenance of specific sphingolipid levels is critical for tumor cell growth and proliferation [21]. Ceramide level is regulated by N-acylsphingosine amidohydrolase 1 (ASAH1), an enzyme involved in ceramide metabolism [22, 23]. ASAH1 converts ceramide to sphingosine and free fatty acids, thereby creating a favorable environment for tumor growth [24]. ASAH1 expression is associated with tumor growth, progression, and poor response to therapy, highlighting its potential role as a target for cancer therapy [25–27]. However, the involvement of ASAH1 in TNBC development and progression remains unreported, underscoring the need for further exploration and understanding of its role in TNBC.

In this study, we show that ASAH1 regulates TNBC growth and progression by modulating mitogen-activated protein kinase (MAPK) and pharmacological cotargeting of ASAH1 (via carmofur) and the MAPK pathway (via trametinib) effectively suppresses TNBC growth.

## Results

### ASAH1 overexpression in TNBC is regulated by p53 and PI3K-AKT signaling pathway

**Fig. 1:**
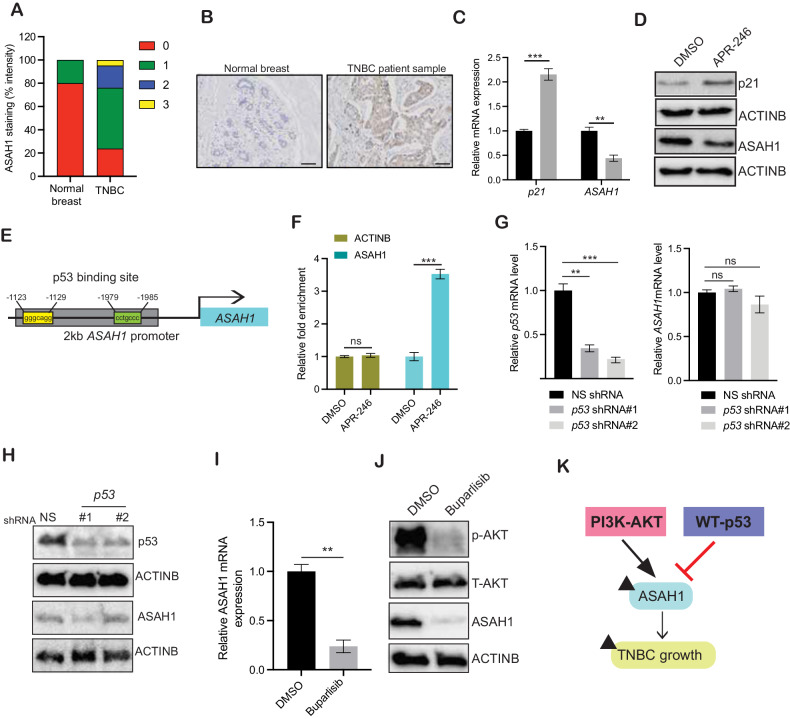
ASAH1 overexpression in TNBC is regulated via p53 and PI3K-AKT signaling pathway. **A** Analysis of immunohistochemical (IHC) staining data from a tissue microarray (TMA) with triple-negative breast cancer samples [21] and matched adjacent normal breast tissue samples [10]. The average intensity of ASAH1 staining in the TNBC and normal breast tissue samples is shown, which is categorized as follows: 0, no staining; +1, weak staining; +2, moderate staining; or +3, high staining. **B** Protein expression was measured via IHC staining in a TMA containing TNBC and matched normal adjacent breast tissue (20× magnification) samples. Representative images are shown. Scale bar, 50 μm. **C** Transcript levels of *ASAH1* and *CDKN1A* (p21) were evaluated by quantitative reverse-transcriptase–polymerase chain reaction (qRT-PCR) in MDA-MB-468 cells after treatment with 25 μM of APR-246 (Prima-1Met) for 24 h. **D** Protein levels for ASAH1 and p21 were evaluated by immunoblotting in MDA-MB-468 cells after treatment with 25 μM of APR-246 (Prima-1Met) for 24 h. ACTINB was used as a loading control. **E** Schematic diagram showing the p53 binding site on the *ASAH1* promoter region. **F** MDA-MB-468 cells after treatment with 25 μM of APR-246 (Prima-1Met) for 24 h were analyzed using CUT and RUN assay to evaluate the binding of p53 to the *ASAH1* promoter. **G** MDA-MB-468 cells expressing either nonspecific (NS) small hairpin RNA (shRNA) or mutant p53 targeting shRNAs were analyzed by performing a quantitative reverse-transcriptase–polymerase chain reaction (RT-qPCR). Mutant *p53* and *ASAH1* mRNA expression level in the knockdown cells is presented. **H** Immunoblotting of the shown protein was performed in mutant *p53* shRNA-expressing cells along with NS shRNA-expressing cells. ACTINB was used as the loading control. **I** *ASAH1* mRNA levels were evaluated by quantitative reverse-transcriptase–polymerase chain reaction (qRT-PCR) in MDA-MB-468 cells after treatment with 100 nM of Buparlisib for 24 h. **J** Immunoblotting of the shown protein was performed in MDA-MB-468 cells after treatment with 100 nM of Buparlisib for 24 h. ACTINB was used as the loading control. **K** Schematics showing PI3K-AKT signaling activates ASAH1 expression and WT p53 inhibits ASAH1 expression in TNBC cells. Data represent the mean ± standard error for three biological replicates. **P < 0.01, ***P < 0.001, ns not significant.

The identification and targeting of metabolic genes and associated pathways involved in tumor development may provide novel TNBC therapeutic targets. Therefore, we characterized metabolic genes that are significantly overexpressed in breast cancer, including TNBC. We observed ASAH1 to be overexpressed in breast cancer patient samples but not in normal adjacent breast tissue samples (Supplementary Fig. 1A–D). We also noticed that TNBC patient samples with higher ASAH1 expression were associated with poorer disease-free survival (DFS), relapse-free survival (RFS), and distant metastasis-free survival (DMFS) (Supplementary Fig. 1E–G). To confirm whether ASAH1 was overexpressed in TNBC, we performed an immunohistochemistry (IHC) to measure the ASAH1 protein levels using tissue microarray (TMA), which contained 21 TNBC cases and 10 adjacent normal breast tissue samples (US Biomax: BC081120f). We observed that ASAH1 protein expression was upregulated in the majority of patient-derived TNBC sample tissues, but not in the adjacent normal breast tissues (Fig. 1A, B and Supplementary Table 1), confirming that ASAH1 is overexpressed in the TNBC samples.

We then investigated the mechanism underlying the ASAH1 overexpression in TNBC. The mutations in the tumor suppressor *TP53* gene resulting in defective transcription function are noted in 80% of TNBCs [28]. To investigate whether p53 affects *ASAH1* mRNA expression, we treated the TNBC cell line MDA-MB-468 carrying p53 mutation [29] with APR-246. APR-246, also called (PRIMA-1^met^), has been shown to modify sequence-specific DNA-binding property of mutant p53 protein, thus restoring its wild-type transcription-associated activity and leading to antitumor activity [30, 31]. In our study, we observed that treatment of MDA-MB-468 cells with APR-246 led to upregulation of p53 target p21 and downregulation of *ASAH1* mRNA and protein levels (Fig. 1C, D and Supplemental Data 1). To determine whether p53 directly regulates the *ASAH1* transcription, we analyzed the promoter sequence of *ASAH1* using the in silico program PROMO, which predict putative transcription factor binding sites in the DNA sequence [32]. We identified a DNA-binding site for p53 on the *ASAH*1 promoter (Fig. 1E). To confirm the association between p53 and the endogenous *ASAH1* promoter, a CUT&RUN assay was performed in APR-246 treated MDA-MB-468 cells. Our results showed increased direct binding of p53 on *ASAH1* promoter in APR-246-treated MDA-MB-468 cells (Fig. 1F). In addition, we observed that the knockdown of mutant p53 in MDA-MB-468 cells did not alter *ASAH1* mRNA and protein expression level. (Fig. 1G–H and Supplemental Data 1). These results suggest that p53 inhibits ASAH1 expression in TNBC and that loss of wild-type and gain-of-function mutants of p53 plays distinct roles in TNBC tumor growth and progression.

PI3K-AKT signaling pathway is another frequently altered pathway reported in ∼25% of primary TNBC and higher in metatstic TNBC cases [33]. Activation of PI3K-AKT signaling pathways in TNBC is due to multiple genetic alterations, including activation of oncogenes such as PIK3CA, AKT among others and inactivation of tumor suppressor genes such as PTEN, TSC1, TSC2, LKB1 [34]. Based on the important role of PI3K-AKT signaling pathway in TNBC, PI3K and AKT inhibitors are used in phase II clinical trials in combination with first-line chemotherapy for the treatment of TNBC patients and have shown improved disease-free survival in patients [34]. To investigate whether inhibition of PI3K-AKT signaling pathway effects ASAH1 expression, we treated MDA-MB-468 cells with PI3K-AKT inhibitor Bupralisib and measured *ASAH1* mRNA and protein expression. Our result showed that inhibition of PI3K-AKT signaling pathway led to suppression of both *ASAH1* mRNA and protein levels in MDA-MB-468 cells (Fig. 1I, J and Supplemental Data 1). Collectively, our results show that both inactivation of p53 and activation of PI3K-AKT signaling pathway are the two major causes for the upregulation of ASAH1 expression in TNBC cells (Fig. 1K).

### Genetic inhibition of ASAH1 impairs TNBC growth and metastases

**Fig. 2:**
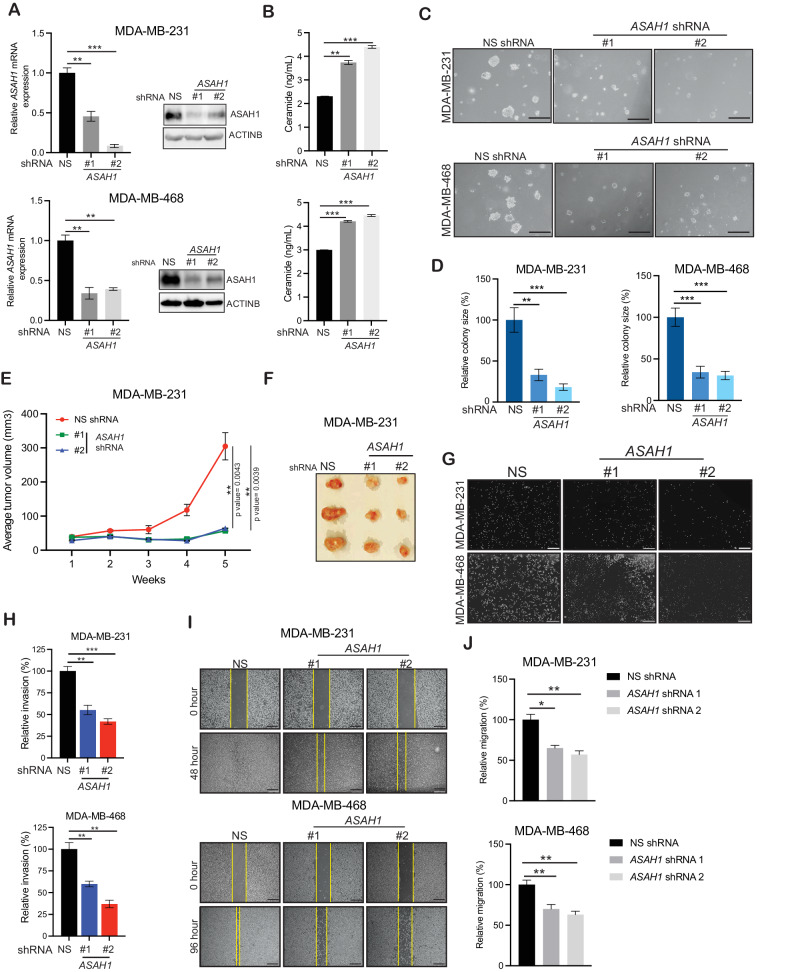
ASAH1 knockdown inhibits TNBC tumor growth and metastasis. **A** TNBC MDA-MB-231 and MDA-MB-468 cells expressing either nonspecific (NS) small hairpin RNA (shRNA) or *ASAH1* shRNAs were analyzed by performing a quantitative reverse-transcriptase–polymerase chain reaction (RT-qPCR), and immunoblotting to analyze *ASAH1* mRNA and protein expressions. The *ASAH1* mRNA expression level in *ASAH1* shRNA-expressing cells relative to NS shRNA-expressing cells is presented (left). The ASAH1 protein expression level in *ASAH1* shRNA-expressing cells relative to NS shRNA-expressing cells is presented (right). ACTINB was used as the loading control. **B** The ceramide level was measured in the indicated TNBC MDA-MB-231 and MDA-MB-468 cells expressing either NS small hairpin RNA (shRNA) or *ASAH1* shRNAs using the human ceramide antibody (ceramide-Ab) ELISA Kit (cat. no. MBS3804520). The relative ceramide level in each condition is shown. **C** TNBC MDA-MB-231 and MDA-MB-468 cells expressing either NS small hairpin RNA (shRNA) or *ASAH1* shRNAs were analyzed for their abilities to grow in soft-agar assay. Representative images are shown with a scale bar of 500 µm. **D** Relative colony sizes for the images are shown in (**C**). **E** MDA-MB-231 cells expressing either NS small hairpin RNA (shRNA) or *ASAH1* shRNAs were subcutaneously injected into the flanks of female NSG mice (*n* = 3). The average tumor volume was assessed weekly and plotted**. F** Representative tumor images at the experimental endpoint is shown. **G** Indicated TNBCs MDA-MB-231 and MDA-MB-468 cells expressing either NS small hairpin RNA (shRNA) or *ASAH1* shRNAs were analyzed for their invasive ability using Matrigel-based invasion assays. Representative images are shown with a scale bar of 200 µm. **H** Quantitation of the data presented in panel G and relative invasion percentage in each condition is plotted. **I** Migration was analyzed in a wound-healing assay for TNBC MDA-MB-231 and MDA-MB-468 cells expressing either NS small hairpin RNA (shRNA) or *ASAH1* shRNAs. Representative images are shown with a scale bar of 200 µm. **J** Quantitation of the data presented in (**I**) and relative migration percentage in each condition is plotted. Data represent the mean ± standard error for three biological replicates. *P < 0.05, **P < 0.01, ***P < 0.001, ns not significant.

We next investigated the role of ASAH1 in the TNBC growth and metastasis. We first knocked down ASAH1 in two different TNBC cell lines (MDA-MB-231 and MDA-MB-468) using two sequence-independent short hairpin RNAs (shRNAs) (Fig. 2A and Supplemental Data 2). ASAH1, a member of the acid ceramidase family of proteins, is involved in regulating intracellular ceramide levels by converting sphingosine into ceramide [24]. Therefore, we measured the ceramide levels in TNBC cells expressing either *ASAH1* shRNA or a nonspecific (NS) shRNA as a control. We observed that the *ASAH1* knockdown led to increased ceramide levels in the TNBC cells (Fig. 2B). Ceramide regulates cancer cell growth through its involvement in cell cycle arrest, migration, and metastasis [35]. We tested the effect of *ASAH1* knockdown on TNBC cell anchorage-independent growth by using a soft-agar assay. The anchorage-independent growth on soft agar is a reliable surrogate assay for in vitro tumorigenesis [36]. TNBC cells expressing nonspecific (NS) control shRNA were used as negative controls. The *ASAH1* knockdown in TNBC cells resulted in a significantly reduced cell ability to form colonies on soft agar (Fig. 2C, D). We also tested the effect of *ASAH1* knockdown in other TNBC subtypes and observed that ASAH1 knockdown in all TNBC subtypes resulted in a significantly reduced ability of the cells to form colonies on soft agar, confirming that ASAH1 is important for the growth of all TNBC subtypes (Supplementary Fig. 2 and Supplemental Data 2). We then tested the effects of *ASAH1* knockdown on TNBC tumor growth in vivo. We subcutaneously injected MDA-MB-231 TNBC cells expressing either ASAH1-specific or control NS shRNAs into the flanks of female NOD scid gamma (NSG) mice. Consistent with the results obtained in the cell culture experiments, *ASAH1* knockdown inhibited the subcutaneous TNBC tumor growth in vivo (Fig. 2E, F).

Furthermore, we investigated the role of ASAH1 in TNBC metastatic growth. We first analyzed the effects of *ASAH1* knockdown on TNBC cell migration and invasion in vitro. *ASAH1* knockdown in TNBC cells significantly inhibited the invasiveness of TNBC cells in a Matrigel invasion assay (Fig. 2G–H) and their migratory capacity in a wound-healing assay (Fig. 2I, J), as compared with the cells expressing a control nonspecific shRNA. Collectively, these results demonstrated that genetic inhibition of *ASAH1* can block the tumor growth of TNBC and metastasis.

### Pharmacological inhibition of ASAH1 inhibits TNBC growth and metastasis

**Fig. 3:**
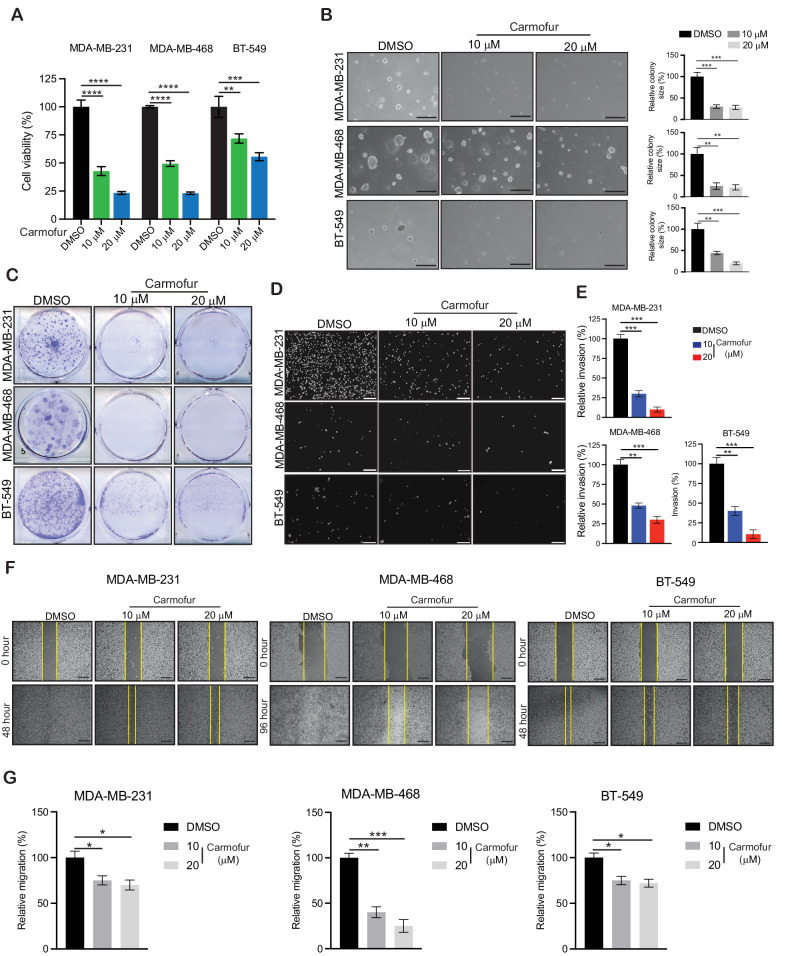
ASAH1 inhibitor carmofur inhibits TNBC growth and metastasis in vitro. **A** The indicated TNBC cancer cell lines were treated with different concentrations of ASAH1 inhibitor carmofur for 3 days, and survival was assessed by 3-(4,5-dimethylthiazol-2-yl)-2,5-diphenyltetrazolium bromide (MTT) assay. Cell survival in carmofur treatment condition is plotted relative to the control DMSO treatment. **B** The indicated TNBC cell lines were treated with different concentrations of carmofur and analyzed for their abilities to grow in soft-agar assay. Representative images are shown (left) with a scale bar of 500 µm. Relative colony sizes for the images are shown (right). **C** The indicated TNBC cell lines were treated with different concentrations of carmofur for 2–4 weeks. Cell survival was measured using clonogenic assay. Representative images are shown. **D** The indicated TNBC cell lines were treated with different concentrations of carmofur and analyzed for their invasive ability using Matrigel-based invasion assays. Representative images are shown at a scale bar of 200 µm. **E** Quantitation of the data presented in (**D**). **F** Migration was analyzed in a wound-healing assay for TNBC cancer cell lines treated with different concentrations of carmofur or control DMSO. Representative images are shown at a scale bar of 200 µm. **G** Quantitation of the data presented in (**F**). Data represent the mean ± standard error for three biological replicates. *P < 0.05, **P < 0.01, ***P < 0.001, ****P < 0.0001, ns not significant.

ASAH1 is pharmacologically targeted by carmofur (1-hexylcarbamoyl-5-fluorouracil) [37–39], a derivative of 5-fluorouracil [40]. Carmofur, a highly effective acid ceramidase inhibitor, can cross the blood–brain barrier and is used in the treatment of glioblastoma and colorectal cancer [37]. Carmofur inhibits acid ceramidase by modifying its catalytic site, thereby increasing the intracellular ceramide levels and affecting cancer cell viability [38, 41]. We also observed that the treatment with carmofur resulted in increased intracellular ceramide levels (Supplementary Fig. 3A) in TNBC cells as compared with the DMSO-treated control cells. We then measured the effect of carmofur on TNBC cell survival by using an MTT-based short-term survival assay, which showed that carmofur significantly inhibited TNBC cell survival (Fig. 3A) in a concentration-dependent manner. We then tested the effect of carmofur on TNBC cells in both soft agar and long-term survival clonogenic assays. We observed that carmofur treatment significantly inhibited the growth of TNBC cells in both soft agar (Fig. 3B and Supplementary Fig. 3B–E) and clonogenic assays (Fig. 3C). We also observed similar tumor-suppressive effect on growth of TNBC cells with another ASAH1 inhibitor, Ceranib-2 (Supplementary Fig. 4). In addition, we measured the effect of carmofur on the ability of TNBC cells to invade and migrate using Matrigel-based invasion assay and wound-healing based migration assays, respectively. We observed that carmofur treatment significantly inhibited both the invasive and migratory capabilities of TNBC cells (Fig. 3D–G).

**Fig. 4:**
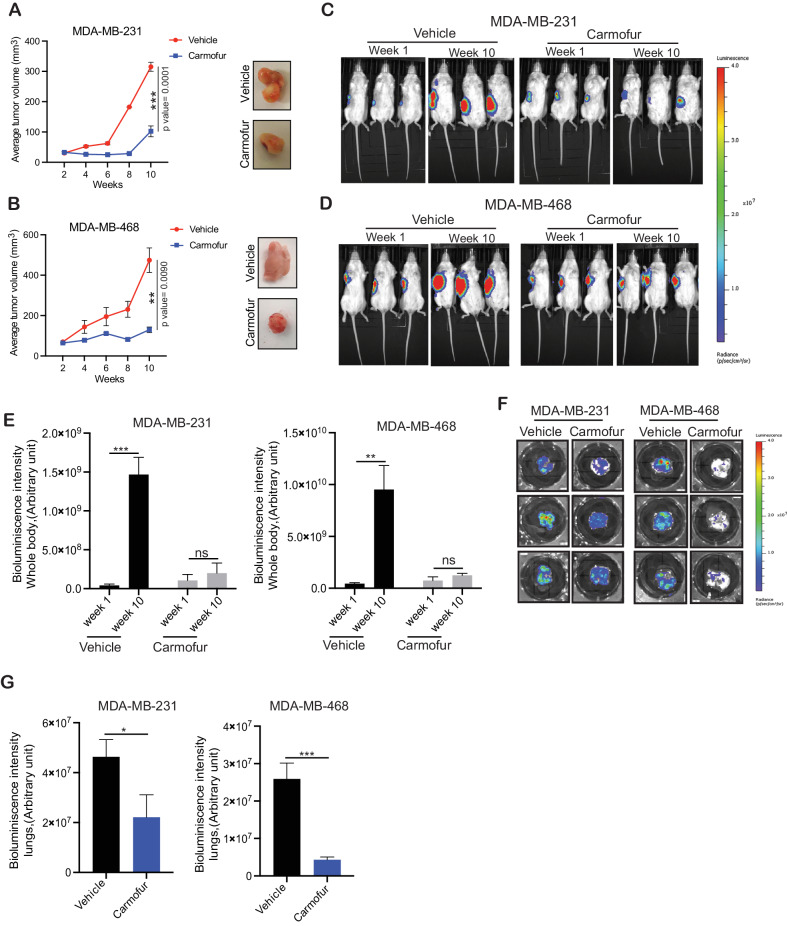
ASAH1 inhibitor carmofur inhibits TNBC tumor growth and spontaneous metastasis in a subcutaneous xenograft model of TNBC. **A**–**G** Firefly luciferase-labeled MDA-MB-231 and MDA-MB-468 cells were subcutaneously injected into the flanks of female NSG mice (*n* = 3). The mice were administered either vehicle or carmofur (20 mg/kg body weight) intraperitoneally every other day. The average tumor volume was assessed weekly and plotted (left). Tumor volume at week 1 and 10 are shown. Representative images of the tumors from mice after treatment with vehicle or carmofur at the endpoint is shown (right) (**A**, **B**). Mice injected with Firefly luciferase-labeled MDA-MB-231 and MDA-MB-468 cells subcutaneously and treated with either vehicle or carmofur (20 mg/kg body weight) intraperitoneally every other day and were assessed for tumor growth by IVIS imaging. Representative bioluminescence images of the mice at weeks 1 and 10 in the vehicle or carmofur treatment condition (**C**, **D**). Bioluminescence intensity from the mice at week 1 and 10 in the vehicle or carmofur treatment condition is presented (**E**). Bioluminescence images of the lungs obtained from vehicle-treated or carmofur-treated female NSG mice after either vehicle or carmofur treatment at the endpoint (**F**). Bioluminescence intensity measured from the lungs obtained from either the vehicle-treated or carmofur-treated female NSG mice at the endpoint, shown in (**F**) (**G**). Data represent the mean ± standard error for three biological replicates. *P < 0.05, **P < 0.01, ***P < 0.001, ns not significant.

Guided by the results obtained in the cell culture-based assays in vitro, we tested the effect of carmofur on TNBC cell growth and metastasis using series of clinically relevant complementary in vivo TNBC models of tumor growth and metastasis, including subcutaneous xenograft-based TNBC tumor growth and spontaneous metastasis models, orthotopic xenograft-based TNBC tumor growth and spontaneous metastasis models, and patient-derived xenografts (PDXs) models. We first started with the subcutaneous xenograft-based model for TNBC tumor growth and spontaneous metastasis and subcutaneously injected F-Luc-labeled MDA-MB-231 (*F-Luc*-MDA-MB-231) and MDA-MB-468 (*F-Luc*-MDA-MB-468) cells into the flanks of the female NSG mice. The mice were then treated with either vehicle as a control (30% PEG-300 and 5% tween 80) or carmofur (20 mg/kg), and tumor growth was measured weekly. We observed that the TNBC subcutaneous tumor growth was inhibited in the carmofur-treated group as compared with the control vehicle-treated group (Fig. 4A, B). We also monitored the tumor growth and spontaneous metastasis to the distal organs in each group by measuring the *F-Luc* activity via bioluminescence imaging using the Xenogen IVIS imaging instrument and observed significantly decreased bioluminescence intensity in the carmofur-treated group as compared with the control vehicle-treated group (Fig. 4C–E). In addition, spontaneous metastasis to the lungs was significantly inhibited in the carmofur-treated group as compared with the vehicle-treated group (Fig. 4F, G).

**Fig. 5:**
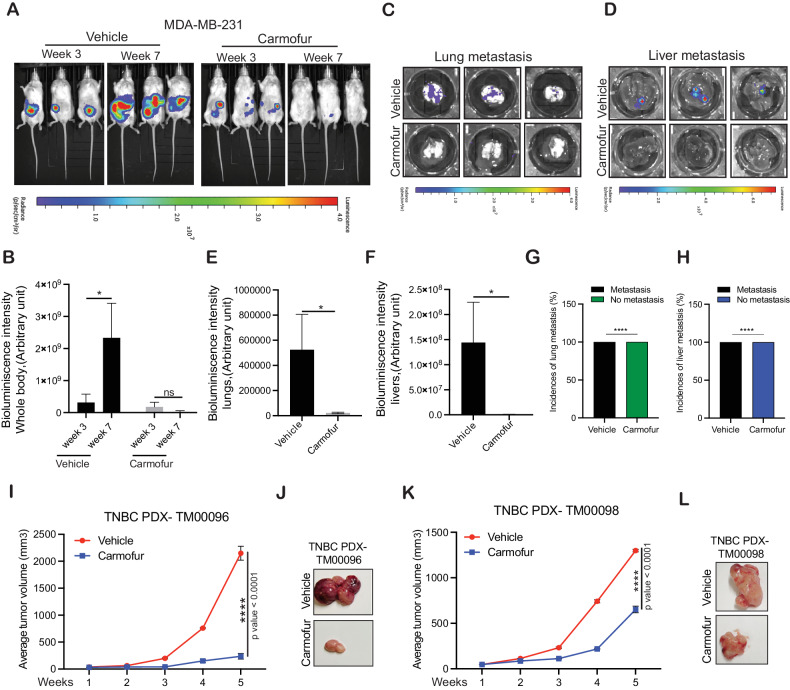
ASAH1 inhibitor carmofur inhibits TNBC tumor growth and spontaneous metastasis in an orthotopic xenograft model of TNBC. **A**–**F** Firefly luciferase-labeled MDA-MB-231 cells were injected orthotopically in the mammary fat pad of female NSG mice (*n* = 3). The mice were administered either vehicle or carmofur (20 mg/kg body weight) intraperitoneally every other day, and tumor growth was analyzed by IVIS imaging. **A** Bioluminescence images of the whole body of mice at week 3 and week 7 in vehicle or carmofur-treated group. **B** Bioluminescence intensity of the mice measured at weeks 3 and 7 after treatment with either vehicle or carmofur shown in (**A**) is plotted. **C** Representative bioluminescence images of the lungs from mice after week 7 of treatment with either vehicle or carmofur. **D** Representative bioluminescence images of the livers from mice after week 7 of treatment with either vehicle or carmofur. **E** Bioluminescence intensity of the lungs measured at week 7 in vehicle or carmofur-treated group shown in (**C**). **F** Bioluminescence intensity of the livers measured at week 7 in vehicle or carmofur-treated group shown in (**D**). **G** Incidence of lung metastasis in mice treated with either vehicle or carmofur. **H** Incidence of liver metastasis in mice treated with either vehicle or carmofur. **I**–**L** TNBC PDX (TM00096 and TM00098) were subcutaneously injected into the flanks of female NSG mice (*n* = 3). The mice were administered with either vehicle or carmofur (20-mg/kg body weight) intraperitoneally every other day. The average tumor volume was assessed weekly and plotted (**I**, **K**). Representative images of the tumors after treatment with vehicle or carmofur at the endpoint (**J**, **L**). Data represent the mean ± standard error for three biological replicates. *P < 0.05, ****P < 0.0001, ns not significant.

We then tested the effects of carmofur in an orthotopic xenograft model for TNBC tumor growth and spontaneous metastasis. This experimental model has the advantage of mirroring several aspects of breast cancer development as it occurs in humans. Further, this experimental approach also allows the modeling of the multistep metastatic process, which occurs in humans [42]. We engrafted the F-Luc-labeled MDA-MB-231 (*F-Luc-*MDA-MB-231) TNBC cells orthotopically into the mammary fat pad of female NSG mice. These mice were then treated with either vehicle as a control (30% PEG-300 and 5% tween 80) or carmofur (20 mg/kg), and tumor growth and metastasis to distal organs in each group were assessed by monitoring the *F-Luc* activity via bioluminescence imaging using the Xenogen IVIS instrument. Consistent with the results obtained using the subcutaneous xenograft-based model, carmofur treatment significantly inhibited orthotopic tumor growth (Fig. 5A, B). In addition, a reduction in spontaneous metastasis to the lungs and liver was observed in the carmofur-treated group as compared with the control vehicle-treated group (Fig. 5C–F). All three vehicle-treated mice developed lung and liver metastasis, whereas none of the three mice developed lung and liver metastasis under carmofur-treated conditions (Fig. 5G, H).

We then further validated our findings in the TNBC PDX-based models. The PDX cancer models mirror several characteristics of the cellular heterogeneity observed in cancer patients; therefore, they are considered a powerful tool for determining the efficacy of investigational cancer therapeutic agents [43, 44]. Two patient-derived xenograft models of TNBC were used. Both PDXs (TM00096 and TM00098) expressed higher *ASAH1* levels (Supplementary Fig. 5 and Supplemental Data 3). The TNBC PDXs were implanted into female NSG mice and treated with either vehicle as a control (30% PEG-300 and 5% tween 80) or carmofur (20 mg/kg). Consistent with the results obtained from subcutaneous xenograft and orthotopic xenograft-based models, carmofur treatment resulted in enhanced PDX tumor growth inhibition as compared with vehicle treatment (Fig. 5I–L). Collectively, these results demonstrated that ASAH1 is necessary for TNBC development and that its pharmacological inhibition leads to the impairment in TNBC tumor growth and metastasis.

### ASAH1 activates the MAPK pathway to promote TNBC growth and metastasis

**Fig. 6:**
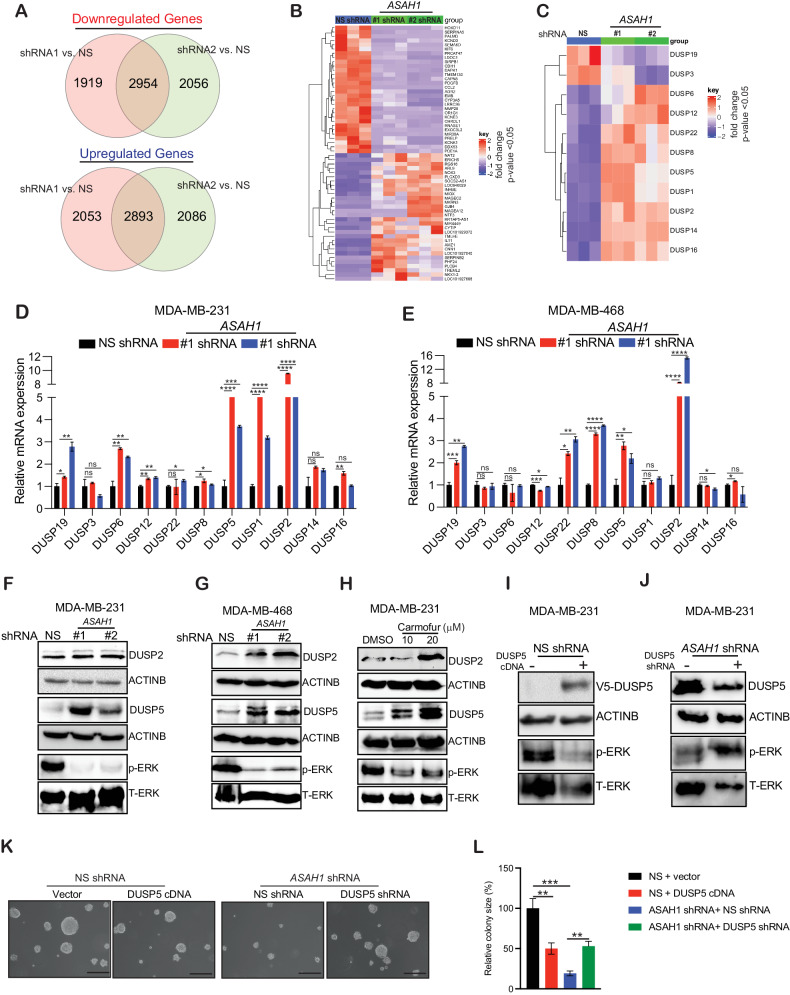
Inhibition of ASAH1 results in the activation of DUSP5 and inhibition of the MAPK pathway. **A** Venn diagram showing overlap among the most differentially expressed genes (upregulated and downregulated) in MDA-MB-231 cells expressing two different *ASAH1* shRNAs as compared with nonspecific (NS) shRNA. **B** Heatmaps showing the top upregulated and downregulated genes in MDA-MB-231 expressing NS or *ASAH1* shRNA. **C** Heatmaps showing the expression of dual-specificity phosphatases (DUSPs) in MDA-MB-231 expressing NS or *ASAH1* shRNA. **D**, **E** Quantitative reverse-transcriptase–polymerase chain reaction (qRT-PCR) was used to measure the mRNA levels of DUSPs identified by RNA sequencing in MDA-MB-231 expressing NS or *ASAH1* shRNA in TNBC cells. *Actin* mRNA was used as the internal control. **F** Indicated proteins were measured in MDA-MB-231 expressing NS or *ASAH1* shRNA via immunoblotting. ACTINB was used as a loading control. **G** Indicated proteins were measured in MDA-MB-468 expressing NS or *ASAH1* shRNA via immunoblotting. ACTINB was used as the loading control. **H** Indicated proteins were measured in MDA-MB-231 after treatment with DMSO or different concentrations of carmofur for 72 h via immunoblotting. ACTINB was used as the loading control. **I** MDA-MB-231 cells expressing NS shRNA with or without *DUSP5* cDNA were analyzed for the shown protein by immunoblotting. ACTINB was used as the loading control. **J** MDA-MB-231 cells expressing *ASAH1* shRNA with or without *DUSP5* shRNA were analyzed for the shown protein by immunoblotting. ACTINB was used as the loading control. **K** MDA-MB-231 cells expressing NS shRNA with or without *DUSP5* cDNA and MDA-MB-231 cells expressing *ASAH1* shRNA with or without *DUSP5* shRNA were analyzed using soft-agar assay. Representative images are shown. Scale bar, 500 μm. **L** Colony sizes plotted for the experiment shown in (**K**). Data represent the mean ± standard error for three biological replicates. *P < 0.05, **P < 0.01, ***P < 0.001, ****P < 0.0001, ns not significant.

We next characterized the mechanism underlying TNBC growth impairment upon ASAH1 inhibition. We analyzed MDA-MB-231 cells expressing either *ASAH1* or NS shRNA as a control using transcriptome-wide RNA sequencing. The RNA-Seq analysis identified several differentially expressed genes in cells expressing *ASAH1* or control NS shRNA, including 2954 common significantly upregulated and 2893 common significantly downregulated genes (*P* < 0.05) (Fig. 6A, B and Supplementary Table 2). We noted that several dual-specificity phosphatases (DUSP) genes were specifically altered in *ASAH1* shRNA-expressing MDA-MB-231 cells as compared with the control NS shRNA-expressing cells (Fig. 6C). We validated the differential expression of the identified DUSPs in several TNBC cells expressing *ASAH1* shRNA or, as a control, NS shRNA and carmofur-treated TNBC cells. We observed increased mRNA and protein expression of DUSP2 and DUSP5 in TNBC cells expressing *ASAH1* shRNA as compared with control NS shRNA-expressing cells and carmofur-treated cells (Fig. 6D–H and Supplemental Data 4). DUSPs are a subclass of protein tyrosine phosphatases that specifically interact with and inactivate MAPKs by dephosphorylating phosphothreonine and tyrosine residues. Hence, DUSPs are critical in the regulation of MAPK pathway activity [45].

We analyzed whether *DUSP2* and *DUSP5* upregulation in TNBC cells after *ASAH1* knockdown affects MAPK signaling. We measured the phosphorylated ERK1/2 level and found that TNBC cells expressing *ASAH1* shRNA or treated with the ASAH1 inhibitor carmofur showed significantly reduced p-ERK1/2 levels as compared with cells expressing the control NS shRNA (Fig. 6F–H and Supplemental Data 4). These results indicated that *ASAH1* downregulation in TNBC cells leads to inhibition of the MAPK pathway. Given that the protein expression level for DUPS5 was stronger than the DUSP2 in ASAH1 genetically and pharmacologically inhibited conditions (Fig. 6F–H and Supplemental Data 4), we focused on investigating the role of DUSP5 in regulating the MAPK pathway in TNBC cells expressing *ASAH1* shRNA. We either overexpressed DUSP5 in NS shRNA condition or generated *DUSP5* knockdown in MDA-MB-231 cells expressing *ASAH1* shRNA and checked the ERK1/2 phosphorylation level. We observed that DUSP5 overexpression in NS shRNA-expressing cells inhibited ERK1/2 phosphorylation, whereas *DUSP5* knockdown in the *ASAH1* shRNA-expressing TNBC cell line led to increased ERK1/2 phosphorylation (Fig. 6I, J and Supplemental Data 5). Furthermore, DUSP5 overexpression in NS shRNA-expressing cells inhibited TNBC tumor growth, whereas *DUSP5* knockdown in the *ASAH1* shRNA expressing partially restored ASAH1 inhibition induced tumor growth inhibition (Fig. 6K, L).

Further, to uncover the mechanism by which DUSP5 expression is regulated by ASAH1, we analyzed the promoter region of DUSP5 to identify the potential transcription factor binding site on DUSP5 promoter that regulates its expression in TNBC cells using the in silico program PROMO. PROMO predicts putative transcription factor binding sites in the DNA sequence [32]. We identified DNA-binding sites for multiple transcription factors on the promoter region of DUSP5 (Supplementary Fig. 6A). We observed that out of the identified transcription factors, transcription factor SP1 mRNA level was significantly upregulated in ASAH1 knockdown MDA-MB-231 cells (Supplementary Fig. 6B). We also noted the upregulation of the transcription factor SP1 mRNA level in ASAH1 inhibitor carmofur-treated MDA-MB-231 cells (Supplementary Fig. 6C). Consistent with the upregulated SP1 mRNA, SP1 protein levels was also upregulated in ASAH1 knockdown and carmofur treatment TNBC cells (Supplementary Fig. 7A–C and Supplemental Data 6).

Based on these results, we tested whether SP1 regulates DUSP5 expression in TNBC cells. We knockdown the transcription factor SP1 using shRNAs and noted that the loss of transcription factor SP1 results in the downregulation of DUSP5 (Supplementary Fig. 7D, E and Supplemental Data 6). To confirm the association between SP1 and *DUSP5* promoter, a CUT&RUN assay was performed in MDA-MB-231 cells expressing *SP1* shRNA and *ASAH1* shRNA. Our results showed decreased binding of SP1 on *DUSP5* promoter in SP1 shRNA-expressing MDA-MB-231 cells and increased binding of SP1 on *DUSP5* promoter in ASAH1 shRNA-expressing MDA-MB-231 cells (Supplementary Fig. 7F). Collectively, these results suggested that ASAH1 loss-mediated inhibition of TNBC growth occurs partly due to the inhibition of MAPK signaling in a DUSP5-dependent manner and one of the mechanisms by which ASAH1 regulate DUSP5 expression is via transcription factor SP1. These results provide an insight into the mechanism by which ASAH1 expression stimulates MAPK pathway to promote the growth of TNBC cells.

### Cotargeting MAPK pathway and ASAH1 leads to the potent TNBC tumor growth inhibition

**Fig. 7:**
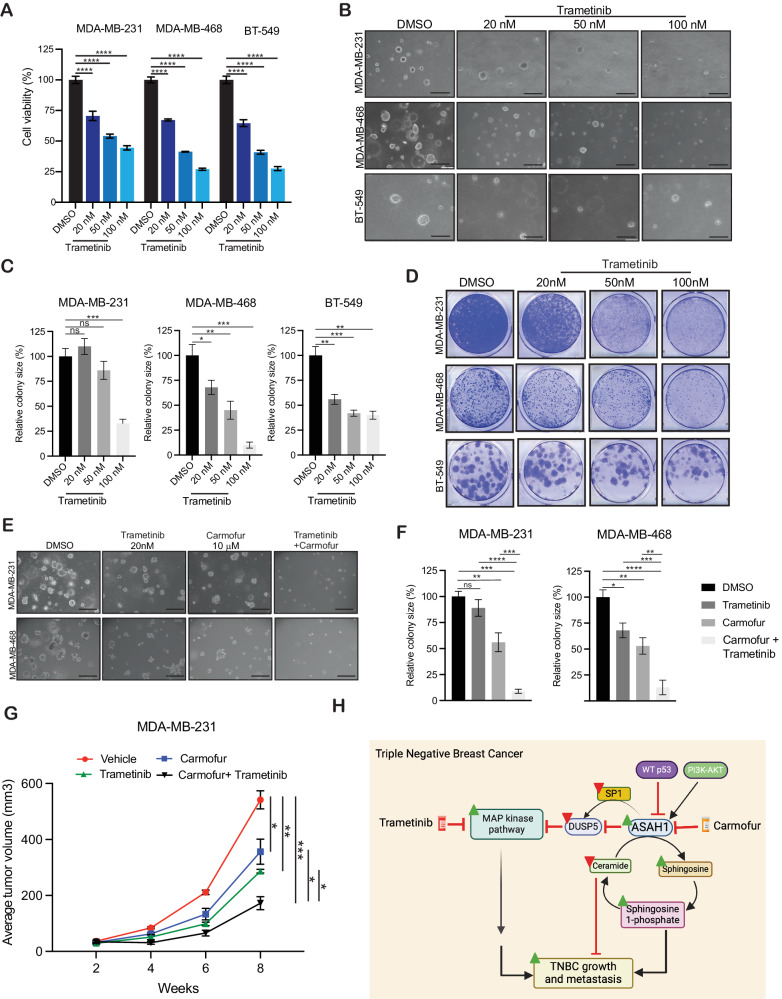
Carmofur in combination with MAPK pathway inhibitor trametinib causes potent TNBC tumor growth inhibition. **A** The indicated TNBC cancer cell lines were treated with various concentrations of trametinib for 3 days, and survival was assessed by the 3-(4,5-dimethylthiazol-2-yl)-2,5-diphenyltetrazolium bromide (MTT) assay. Cell survival is presented relative to the survival of DMSO-treated cells. **B** The indicated TNBC cell lines were treated with indicated concentrations of trametinib and analyzed for their abilities to grow in soft-agar assay. Representative images are shown with a scale bar of 500 µm. **C** Colony sizes plotted for the experiment shown in (**B**). **D** The indicated TNBC cell lines were treated with indicated concentrations of trametinib for 2–4 weeks. Cell survival was measured using a clonogenic assay. Representative images are shown. **E** TNBC cell lines MDA-MB-231 and MDA-MB-468 were treated with DMSO, 10 µM carmofur alone, 20 nM trametinib alone, or both in combination for 2–4 weeks and analyzed for their abilities to grow in soft-agar assay. Representative images are shown with a scale bar of 500 µm. **F** Colony sizes plotted for the experiment shown in (**E**). **G** MDA-MB-231 cells were subcutaneously injected into the flanks of female NSG mice (*n* = 3). Mice were treated with vehicle, 20-mg/kg carmofur alone, 2-mg/kg trametinib alone, or both in combination. The average tumor volume was assessed weekly and plotted. **H** The proposed model for the study—ASAH1 promotes TNBC tumor growth and progression by regulating DUSP5 expression, which inhibits the MAPK pathway. ASAH1 and MAPK pathway can both be targeted via small-molecule inhibitors, either alone or in combination, to provide an effective TNBC therapy. Data represent the mean ± standard error for three biological replicates. *P < 0.05, ***P < 0.001, ****P < 0.0001, ns not significant.

Based on our results that ASAH1 regulates MAPK pathway, we investigated whether the MAPK pathway inhibitor trametinib, an MEK inhibitor, can exert a tumor-suppressive effect in TNBC cells, similar to that observed for the ASAH1 inhibitor carmofur. Trametinib (GSK1120212, JTP-74057, Mekinist) is a highly specific and effective MEK1/2 inhibitor with an IC50 of 0.92 nM/1.8 nM in cell-free assays [46, 47]. Trametinib does not affect the kinase activities of c-Raf, B-Raf, and ERK1/2. Different TNBC cell lines (MDA-MB-231, MDA-MB-468, and BT-549) were treated with increasing concentrations of trametinib, and cell viability was measured using the MTT assay. The results showed that trametinib inhibited TNBC cell viability in short-term cell viability MTT assay (Fig. 7A). Trametinib treatment also significantly inhibited the TNBC growth in the soft-agar and clonogenic assays in a concentration-dependent manner (Fig. 7B–D).

We next investigated whether trametinib and the ASAH1 inhibitor carmofur can be combined to achieve even stronger TNBC tumor growth inhibition. We first performed a soft-agar assay, which demonstrated that their combined treatment using suboptimal concentrations of both trametinib and carmofur caused significant tumor growth inhibition in TNBC cells (Fig. 7E, F). We then analyzed the in vivo effect of the combined treatment in a subcutaneous xenograft model of TNBC tumor growth. We engrafted MDA-MB-231 cells subcutaneously into female NSG mice and then treated the mice group with either vehicle (30% PEG-300 and 5% tween 80), carmofur (10 mg/kg), trametinib (2 mg/kg), or a combination of trametinib (2 mg/kg) and carmofur (10 mg/kg). Tumor growth was measured weekly. Consistent with the results observed in cell culture experiments, the combined treatment of trametinib and carmofur significantly inhibited subcutaneous TNBC tumor growth as compared with the treatment using inhibitors alone (Fig. 7G) or in vehicle-treated conditions. In summary, these results provided evidence that combined treatment with the ASAH1 inhibitor carmofur and the MAPK pathway inhibitor trametinib can lead to significant inhibition of TNBC tumor growth. Thus, this combined treatment regimen can be used as an effective treatment option for TNBC patients.

## Discussion

TNBC presents unique challenges in treatment due to its aggressive nature, lack of expression of targetable receptors [48, 49] and lacks well-defined biomarkers for targeted treatment [50, 51]. For example, TNBC does not respond to hormonal or trastuzumab-based therapy [52]. This limits the treatment options, and chemotherapy remains the mainstay of systemic therapy, which becomes ineffective as tumors develop resistance over time [53, 54]. In addition, immunotherapy, particularly immune checkpoint inhibitors like pembrolizumab and atezolizumab, have shown promise in treating a subset of TNBC patients but not all patients respond to these treatments [55]. TNBC is highly heterogeneous, which makes it challenging to develop effective targeted therapies that works for all patients [2]. As a result, high mortality is observed in TNBC patients [56, 57]. Addressing these challenges will require advancing our understanding of TNBC biology to develop more effective treatment strategies and ensuring consistent care for TNBC patients. In the present study as summarized in Fig. 7H, we show that ASAH1 as a specific metabolic enzyme that is overexpressed in TNBC. We found that the ASAH1 → DUSP5 → MAPK-signaling axis is a crucial tumorigenic driver and a potential therapeutic target of clinical value for treating TNBC.

Metabolic deregulation is increasingly recognized as a critical factor in TNBC development and progression [11–13]. TNBC cells often strongly rely on the glycolytic pathway and dysregulated lipid metabolism to support aggressive tumor growth. Therefore, targeting the metabolic pathways essential for TNBC development offers potential novel therapeutic avenues for treating TNBC [15].

Ceramides, crucial bioactive lipids, play pivotal roles in cancer cell signaling and metabolism [14, 17, 18], with their upregulation linked to inhibiting tumor growth and progression [21]. Enzymes regulating ceramide levels, such as acid ceramidases, offer potential therapeutic targets. In humans, five ceramidases, including ASAH1, exhibit tissue-specific expression and functions [46]. ASAH1, specifically, maintains ceramide levels by hydrolyzing them into sphingosine and fatty acids within lysosomes [23, 24]. Given ceramides’ diverse functions in regulating cell growth, differentiation, and apoptosis, targeting ASAH1 holds promise for cancer therapy.

Previous studies have highlighted ASAH1’s significance in various cancers. In colorectal cancer (CRC), ASAH1 overexpression is linked to immunological cell death induction, enhancing the antitumor immune response [58]. In recurrent glioblastoma (GBM), elevated ASAH1 levels is observed and treatment of temozolomide (TMZ)-resistant GBM cells with carmofur resulted in decreased cell growth migration and invasion and increased apoptosis [39, 59]. ASAH1 also promotes melanoma tumor growth and metastasis and its pharmacological inhibition enhances the effectiveness of BRAF kinase inhibitor [22]. In addition, ASAH1-null cells lose the ability to form cancer-initiating cells in melanoma, suggesting its crucial role in malignancy maintenance [60]. In breast cancer, ASAH1 expression correlates with lymph node metastasis, although conflicting reports exist regarding its prognosis association [61, 62]. Despite these insights, ASAH1’s involvement in TNBC remains largely unexplored.

Our study results showed that ASAH1 inhibition via gene knockdown or carmofur suppresses TNBC growth and metastasis. Carmofur has been investigated for its anticancer properties in different cancer types, including glioblastoma [39], where it showed promising effects because of its ability to cross the blood–brain barrier. Carmofur’s efficacy in treating colorectal cancer, both as a standalone therapy and in combination with other compounds, has also been assessed in clinical trials [63]. Furthermore, the combination of carmofur with other drugs has also been investigated in metastatic cancers, including ovarian [64], colon [65, 66], gastrointestinal [67], and colorectal cancers [68], and has shown to improve the overall survival of these patients. Thus, its capability to penetrate the blood–brain barrier has led to further exploration of its potential for treating brain metastasis, along with other distal metastatic diseases. Our results too paves the way for employing carmofur for treating TNBC, including the metatstic disease.

We further find that ASAH1 modulates the MAPK pathway via DUSP5 and DUSP5 expression in regulated by transcription factor SP1. DUSP5, a member of the dual-specificity phosphatase family dephosphorylates both tyrosine and serine/threonine residues [69] and is repressed in several cancers, including breast, lung, and ovarian cancers [70, 71]. The tumor-suppressive effect of DUSP5 is attributed to its role in MAPK pathway inhibition [72], which causes cell cycle arrest and apoptosis [45]. Thus, our study also revealed a novel mechanism of DUSP5 regulation in TNBC. These findings highlight the need to understand the context-specific regulation of DUSP5 via ceramides to develop targeted therapeutic interventions.

Finally, in our study, we reported that a combination of carmofur and trametinib holds promise in treating TNBC. Trametinib has received FDA approval for treating certain cancers [73, 74] and exhibits high efficacy in improving the progression-free and overall survival of patients with melanoma [75–77]. Furthermore, several metabolic-based drugs are being developed and investigated in clinical trials [78]. For instance, a derivative of BPTES, CB-839, which inhibits glutaminase, is being investigated in several clinical (NCT03047993; NCT04250545; NCT02771626; (NCT03528642) [9]. Based on the success of the ongoing clinical trials exploring the combination of carmofur with trametinib, immunotherapy, radiation therapy, or chemotherapy will pave the way for effective future TNBC treatment.

One of the limitations of our in vivo study was that the survival of drug-treated mice compared with vehicle-treated mice was not assessed because death of mice was not used as an experimental endpoint. It is possible that drug treatment will also affect survival of the mice, and therefore, future study that compares the survival of the mice under different treatment condition can be performed.

In summary, our study underscores the importance of ASAH1 in TNBC and unveils novel insights. For the first time, we demonstrate in clinically relevant TNBC models that inhibiting ASAH1 suppresses TNBC growth and progression. We further reveal the role of ASH1 in stimulating the MAP kinase pathway via suppressing DUSP5 in TNBC, shedding light on its diverse functions in cancer. In addition, our study also introduces carmofur, and other ceramide-based therapies as novel therapeutic strategies for TNBC therapy that can be combined with trametinib, and potentially other approved therapeutic agents to potently inhibit TNBC tumor growth. These findings offer significant advancements in TNBC treatment, addressing an area lacking effective treatment options. Moreover, our study paves the way for swift translation into clinical practice, as several ceramide-based therapies, such as ceramide nanoliposomes, are approved by the United States Food and Drug Administration (U.S. FDA) for cancer therapy [79], thus bridging a critical therapeutic gap.

## Experimental methodology

### Cell culture and Inhibitors

TNBC cell lines (MDA-MB-231, MDA-MB-468, BT-549, DU445 and MDA-MB-453) were purchased from American Type Culture Collection (ATCC) and and maintained in a humidified atmosphere containing 5% CO_2_ at 37 °C in Dulbecco’s modified Eagle medium (Life Technologies, Carlsbad, CA, USA) or Roswell Park Memorial Institute (RPMI)-1640 medium (Life Technologies), as recommended. All media were supplemented with 10% fetal bovine serum (Life Technologies) and 1% penicillin/streptomycin (Life Technologies). The ASAH1 inhibitor carmofur and trametinib was purchased from Selleckchem and ASAH1 inhibitor Ceranib-2 was purchased from Cayman and dissolved for cell culture and in vivo experiments as suggested in the data sheet. Relevant information is provided in Supplementary Table 3. The treatment conditions are described in the corresponding figure legends.

### MTT assay

For this assay, 3 × 10^3^ MDA-MB-231, BT-549 and MDA-MB-468 cells were plated in a 100 µl volume in 96-well plates. After 24 h, inhibitors were added to 100 µl media at a range of concentrations as shown in figure and were then added to the cells. After 72 h of inhibitor treatment, cell viability was evaluated by adding 20 µl of 5 mg/ml MTT solution dissolved in 1 × PBS to each well, followed by incubation for 1 h in a 37 °C incubator. The MTT solution was gently removed, and 100 µl dimethyl sulfoxide was added to each well. After mixing by pipetting, absorbance was measured at 590 nm and 630 nm using the Biotek Synergy MX Multi-Format Microplate Reader (Winooski, VT, USA). The average measurement at 630 nm was subtracted from the average measurement at 590 nm, and the relative cell viability at each concentration was plotted with respect to vehicle-treated cells.

### Clonogenic assay

Clonogenic assays were performed by seeding 1 × 10^3^ MDA-MB-231, MDA-MB-468, and BT-549 cells in six-well plates in triplicate. After 24 h, the cells were treated with DMSO or inhibitor at different concentration. After 2–3 weeks of treatment, colonies were fixed in a solution containing 50% methanol and 10% acetic acid and then stained with 0.05% Coomassie blue (Sigma-Aldrich). After staining, the plates were scanned and the representative images were presented. Number of colonies were counted in each condition and plotted.

### Soft-agar assay

Soft-agar assays were performed by seeding 10 × 10^3^ MDA-MB-231, MDA-MB-468, and BT-549, DU445 and MDA-MB-453 cells onto 0.4% low-melting-point agarose (Sigma-Aldrich) layered on top of 0.8% agarose. The cells were treated with DMSO or inhibitor at different concentrations. After 3–4 weeks of treatment colonies formed were stained with 0.005% crystal violet and imaged under a microscope. Colony sizes were measured using ImageJ software (https://imagej.nih.gov/ij/; National Institutes of Health, Bethesda, MD, USA) and plotted as percent relative colony size compared with control colonies. Statistical analysis was performed by Student’s *t*-tests in GraphPad Prism 8.0 (GraphPad, San Diego, CA USA).

### Matrigel invasion assay

Invasion assays were performed in BioCoat Growth Factor Reduced Matrigel Invasion Chambers (Cat #354483, Corning, Corning, NY, USA) using TNBC treated with DMSO or inhibitor at different concentrations. The cells were serum-starved for 6 h, and 5 × 10^4^ cells/insert were seeded in triplicate in the top chamber containing low-serum medium. The cells were then incubated for 20 h to allow invasion toward the serum-rich medium in the bottom well. The number of cells invading the Matrigel was quantified by DAPI staining and imaging; 8–12 fields per membrane were counted, and nuclei quantification was performed using ImageJ software.

### Wound-healing assay

MDA-MB-231 and MDA-MB-468 cells were seeded in six-well plate at a density of 2 × 10^5^ cells per well and grown plates until fully confluent. A scratch was then created using a sterile 20-μl pipette tip, and the cell were then treated with DMSO or inhibitor at different concentrations. Cell migration into the wound was monitored at shown time points using light microscopy. Quantification of wound healing was performed using ImageJ software (https://imagej.nih.gov/ij/).

### RNA preparation, cDNA preparation, and quantitative polymerase chain reaction (qPCR)

Total RNA was extracted with TRIzol Reagent (Invitrogen, Carlsbad, CA, USA) and purified using the RNeasy Mini Kit (Qiagen, Hilden, Germany). Then, cDNA was generated using the M-MuLV First Strand cDNA Synthesis Kit (New England Biolabs, Ipswich, MA, USA) according to the manufacturer’s instructions. Next, qPCR was performed with gene-specific primers using Power SYBR-Green Master Mix (Applied Biosystems, Foster City, CA, USA) according to the manufacturer’s instructions. Beta-actin (*ACTB*) levels were used as a normalization control. The primer sequences are provided in Supplementary Table 3.

### Sample preparation for RNA sequencing

MDA-MB-231 cells expressing with NS shRNA or *ASAH1* shRNA used to prepare total RNA for gene expression analysis on an Illumina HiSeq 2500 system. Total RNA was extracted using TRIzol^®^ reagent (Invitrogen, Carlsbad, CA, USA) according to the manufacturer’s instructions and purified on RNAeasy mini columns (Qiagen, Hilden, Germany) according to the manufacturer’s instructions. Then, mRNA was purified from ~500 ng total RNA using oligo-dT beads and sheared by incubation at 94 °C. Following first-strand synthesis with random primers, second-strand synthesis was performed with dUTP to generate strand-specific libraries. The cDNA libraries were then end-repaired and A-tailed. Adapters were ligated, and second-strand digestion was performed using uracil-DNA-glycosylase. Indexed libraries that met appropriate cutoffs for both were measured by quantitative reverse transcription polymerase chain reaction (qRT-PCR) using a commercially available kit (KAPA Biosystems, Wilmington, MA, USA). The insert-size distribution was determined using LabChip GX (PerkinElmer, Waltham, MA, USA) or an Agilent Bioanalyzer (Agilent Technologies, Santa Clara, CA, USA). Samples with a yield ≥0.5 ng/μL were used for sequencing on the Illumina HiSeq 2500 system (Illumina, San Diego, CA, USA). Images were converted into nucleotide sequences by the base-calling pipeline RTA 1.18.64.0 and stored in FASTQ format. For data analysis, the reads were first mapped to the latest UCSC transcript set using Bowtie2 version 2.1.0, and the gene expression level was estimated using RSEM v1.2.15. The Trimmed Mean of the M-values method was used to normalize the raw count. Differentially expressed genes were identified using the edgeR program. Genes showing altered expression with *P* < 0.05 and more than 1.5-fold changes were considered differentially expressed. Clusterprofiler was used for the Gene Ontology and pathway enrichment analyses. RNA-sequencing data presented in this paper are submitted to Gene Expression Omnibus (Accession No. GSE247750) and are available publicly without restrictions.

### RNA-sequencing data analysis

RNA sequencing was carried out for 9 samples comprising three groups (control A (NS shRNA), treatment B (*ASAH1* shRNA#1), treatment C (*ASAH1* shRNA#2). The groups each have 3 biological replicates (Supplementary Table 2). Single-end 76 bp reads were sequenced utilizing the Illumina HiSeq 2500 sequencing instrument. Pre-alignment quality assessments of the raw fastq sequences were carried out using FastQC (version 0.11.7) (http://www.bioinformatics.babraham.ac.uk/projects/fastqc). Before trimming, the number of raw reads for the 9 samples ranges from 50.5 M to 72.4 M (Supplementary Table 2). Low-quality sequences were removed using the software Trimmomatic (version 0.36). The trimmed fastq sequences were aligned to the human hg38 reference genome (GenBank assembly accession: GCA_000001405.28). The alignments were carried out using STAR (version 2.7.1a) [80] with default parameters. Post-alignment quality assessments were carried out with RSeQC (version 2.6.3) [81] and MultiQC (version 1.4) [82]. Samtools (version 0.0.19) [83] and IGV (version 2.6.2) [84] were used for indexing and viewing the alignments respectively. Gene expression was quantified as gene level using the htseq-count function (version 0.12.3) [85]. The UCSC gene annotations for the human genome were used. The htseq-count default parameters were used, except for the strand parameter which was set to reverse to consider the strandedness of library. Differentially expressed genes were identified using DESeq2 (version 1.28) [86]. DESeq2 was run with default parameters [86]. Genes are differentially expressed (if the *P* value < 0.05). Genes are upregulated (if the *P* value < 0.05 and the log_2_ fold change >0) or downregulated (if the *P* value < 0.05 and the log_2_ fold change <0). The normalized gene expression data was used for downstream analyses such as the heatmaps. The complex heatmap package version 1.12.0 [87] was used to generate heatmaps. The volcano plot was generated with an R Bioconductor package EnhancedVolcano (https://github.com/kevinblighe/EnhancedVolcano). To determine the functions altered under conditions of treatment, over-representation enrichment analysis was performed using the WEB-based GEne SeT AnaLysis Toolkit (Webgestalt) [6]. The following parameters were used: Gene Ontology Biological Process for the functional database; the genome for the reference set; Homo sapiens for the organism of interest. The hypergeometric test was used for over-representation analysis for Biological Process in a list of gene supplied either for upregulated genes or for downregulated genes. The Benjamini and Hochberg method was used to calculate the FDR. Biological Processes are considered either as activated or deactivated depending on whether the differentially expressed genes entered were upregulated or downregulated, respectively.

### Immunoblotting

Whole-cell protein extracts were prepared using IP Lysis Buffer (Pierce Chemical, Rockford, IL, USA) containing Protease Inhibitor Cocktail (Roche, Basel, Switzerland) and Phosphatase Inhibitor Cocktail (Sigma-Aldrich, St. Louis, MO, USA). Lysed samples were centrifuged at 12,000 rpm for 40 min, and clarified supernatants were stored at −80 °C. Protein concentrations were determined using Bradford Protein Assay Reagent (Bio-Rad Laboratories, Hercules, CA, USA). Equal amounts of protein samples were electrophoresed on 10% or 12% sodium dodecyl sulfate (SDS)-polyacrylamide gels and transferred onto polyvinylidene difluoride membranes (Millipore, Burlington, MA, USA) using a wet-transfer apparatus (Bio-Rad). Membranes were blocked in 5% skim milk prepared in Tris-buffered saline containing 0.1% Tween-20 and probed with primary antibodies. After washing, the membranes were incubated with the appropriate horseradish peroxidase-conjugated secondary antibodies (1:2000) (GE Healthcare Life Sciences, Chicago, IL, USA). The blots were developed using Super Signal West Pico or Femto Chemiluminescent Substrate (Thermo Fisher Scientific, Waltham, MA, USA). All antibodies used for immunoblotting are listed in Supplementary Table 3.

### Immunohistochemistry (IHC)

Formalin-fixed, paraffin-embedded tissue microarray (TMA) slides containing breast cancer samples and matched normal breast tissues were obtained from US Biomax (#BC081120f). Briefly, following slide deparaffinization, antigen retrieval was performed in citrate buffer (pH 6.0) at 97 °C for 20 min, using the Lab Vision PT Module (Thermo Fisher Scientific). Endogenous peroxides were blocked by incubation in hydrogen peroxide for 30 min, followed by washing with 1 × Tris-buffered saline, and proteins were blocked by incubation with 0.3% BSA for 30 min. Slides were incubated in anti-ASAH1 antibody (dilution 1:100) followed by secondary anti-rabbit HRP-conjugated antibody (Dako, Jena, Germany). Slides were then stained using the Dako Liquid DAB+ Substrate Chromogen System and counterstained with Dako Automation Hematoxylin Histological Staining Reagent. ASAH1 staining was scored by Dr. Kamaljeet Singh, who was blinded to the identity of each slide. All antibodies used for immunohistochemistry analyses are listed in Supplementary Table 3.

### Mouse xenograft tumorigenesis experiments using MDA-MB-231 cells

MDA-MB-231 cells (5 × 10^6^) were injected subcutaneously into female 5–6-week-old NSG mice (stock no. 005557, Jackson Laboratory). Tumor volume was measured every week. Tumor size was calculated using the following formula: length × width^2^ × 0.5. When the tumor volumes reached 80–100 mm^3^, various treatments such as vehicle (0.5% methylcellulose in water) or carmofur or trametinib at the concentration shown in the figure legend were administered intraperitoneally every other day until the end of the experimental period. At the end of the experiment, tumor size was measured, mice were sacrificed subcutaneous tumors were harvested and imaged. All protocols were approved by the UAB Institutional Animal Care and Use Committee.

### Mouse xenograft tumorigenesis and spontaneous metastasis experiments using TNBC cells

MDA-MB-231 and MDA-MB-468 cells stably expressing firefly luciferase under the control of a cytomegalovirus promoter were generated by co-transfection of the transposon vector piggyBac GFP-Luc and the helper plasmid Act-PBase as described previously [88]. Cells with stable transposon integration were selected using blasticidin S (Thermo Fisher Scientific, Waltham, MA, USA). MDA-MB-231-*F*-*Luc* cells (5 × 10^6^) and MDA-MB-231-*F*-*Luc* cells (5 × 10^6^) were then injected subcutaneously into female 5–6-week-old NSG mice (stock no. 005557, Jackson Laboratory). Tumor volume was measured every week. Tumor size was calculated using the following formula: length × width^2^ × 0.5. When the tumor volumes reached 80–100 mm^3^, various treatments such as vehicle (0.5% methylcellulose in water) or carmofur at the concentration shown in the figure legend were administered intraperitoneally every other day until the end of the experimental period. To monitor spontaneous metastasis, imaging was performed every week using the Spectrum In Vivo Imaging System (IVIS) (PerkinElmer, Waltham, MA, USA). Total bioluminescence counts of the tumor-bearing areas were measured using in vivo imaging software (PerkinElmer). At the end of the experiment, whole-body imaging was performed and subcutaneous tumor size was measured. After that the mice were sacrificed and the lungs and liver were imaged using the IVIS Spectrum (PerkinElmer). Total luminescence counts of the liver and lungs were measured using the in vivo imaging software (PerkinElmer). In addition, at the end of the experiment, subcutaneous tumors were harvested and imaged. All protocols were approved by the UAB Institutional Animal Care and Use Committee.

### Orthotopic grafting of TNBC cells in the mouse mammary fat pad

MDA-MB-231-*F*-*Luc* cells (2.5 × 10^5^) (suspended in Matrigel^®^, 1:1 in 35 μl of PBS solution). were orthotopically into the mammary fat pad of 5–6-week-old recipient NSG female mice. Tumor volume was measured by imaging every week using the spectrum in vivo imaging system (PerkinElmer, Waltham, MA, USA). Total bioluminescence counts of the tumor-bearing areas were measured using the in vivo imaging software (PerkinElmer). After tumor became palpable, mice were treated with vehicle (0.5% methylcellulose in water) or carmofur (20 mg/kg body weight) was administered intraperitoneally every other day until the end of the experimental period. At the endpoint, all the animals were subjected to imaging of whole body, lung and liver to monitor the metastasis in the liver and lungs. The bioluminescence intensity at the initial week and the final week was plotted for the vehicle and carmofur-treated group. All protocols were approved by the UAB Institutional Animal Care and Use Committee.

### Mouse tumorigenesis experiment in the PDX model

TNBC PDXs (Stock No. TM00096 and TM00098- Jackson Laboratory) were obtained in donor NSG PDX-engrafted mice. After 6–8 weeks, the PDXs were harvested, and implanted into 5–6-week-old female NSG mice (stock no. 005557, Jackson Laboratory). In brief, F1-generation tumor tissues were minced to a size of 2 mm × 2 mm and subcutaneously implanted through a tiny incision in the right flank of anesthetized NSG mice. Tumor volume was measured every week. When the tumor volumes reached 80–100 mm^3^, the mice were randomized (*n* = 5 per group) and injected with vehicle (0.5% methylcellulose in water) or carmofur (20 mg/kg body weight) intraperitoneally every other day until the end of the experimental period. Tumor volume was measured every week and plotted. Subcutaneous tumors from individual groups were harvested and imaged. All protocols were approved by the UAB Institutional Animal Care and Use Committee.

### CUT&RUN assay

CUT&RUN assays were performed with MDA-MB-231 cells using the CUT&RUN Assay Kit (Cat#86652; Cell Signaling Technology Danvers, MA, USA) according to the manufacturer’s instructions. Briefly, 2 × 10^5^ cells were harvested, washed, bound to activated Concanavalin A‐coated magnetic beads, and permeabilized. The bead-cell complexes were incubated overnight with the appropriate antibody at 4 °C. Then, the complexes were washed three times, and the cells were resuspended in 100 μl pAG/MNase and incubated for 1 h at room temperature. The samples were then washed three times with digitonin buffer with protease inhibitors, resuspended in 150 μl digitonin buffer, and incubated 5 min on ice. MNase was activated by adding calcium chloride, and the samples were incubated at 4 °C for 30 min. The reaction was stopped by adding 150 μl stop buffer, and the samples were incubated at 37 °C for 10 min to release the DNA fragments. The DNA was extracted using the DNA purification columns included in the CUT&RUN Assay Kit. qPCR was then performed using ASAH1 promoter-specific primers/DUSP5 promoter-specific primers, and relative fold change was calculated as the ratio of immunoprecipitated DNA to IgG-precipitated DNA. The primer sequences and antibodies used for the CUT&RUN assays are listed in Supplementary Table 3.

### Bioinformatics analysis

The Human Protein Atlas is a publicly available database containing millions of high-resolution images showing the spatial distribution of proteins detected with 15,598 different antibodies (release 9.0, November 2011) in 46 different normal human tissue types and 20 different cancer types, as well as 47 different human cell lines. Samples containing normal and cancerous tissue were collected and paraffin-embedded following approval by the local ethics committee, and IHC staining of both normal and cancerous tissue was performed. The Human Tissue Atlas (HPA) dataset was used to show the expression of ASAH1 in normal breast tissue and breast cancer patient samples. Gene Expression Profiling Interactive Analysis (GEPIA), a web server for cancer and normal gene expression profiling and interactive analyses, was used to analyze *ASAH1* mRNA expression in breast cancer patient samples as compared to normal breast tissue and identify associations between overall survival (OS) and high and low ASAH1 expression levels. Km plotter was used to relapse-free survival (RFS) and distant metastasis-free survival (DMSF) among patients with high and low ASAH1 expression levels (https://kmplot.com/analysis/). Transcription factors binding with 100% sequence identity on the promoter region of *ASAH1* and *DUSP5* were identified using the PROMO tool (http://alggen.lsi.upc.es/cgi-bin/promo_v3/promo/promoinit.cgi?dirDB=TF_8.3). We selected a 2-kb upstream promoter region of the *ASAH1* and *DUSP5* gene to identify human transcription factors able to bind with 0% dissimilarity using PROMO.

### Statistical analysis

All experiments were conducted with at least three biological replicates. Results for individual experiments are expressed as the mean ± standard error of the mean. Measurements of tumor progression in mice and MTT assays were compared using the area under the curve method in the GraphPad Prism software, version 7.0, for Macintosh (GraphPad Software; https://www.graphpad.com). For the remaining experiments, *P* values were calculated using two-tailed unpaired Student’s t-tests in the GraphPad Prism software, version 7.0, for Macintosh.

### Supplementary information

Supplementary information

Raw western blot data

## Data Availability

The raw sequencing data and processed data for this paper have been deposited in the Gene Expression Omnibus (GEO) databased under the accession number: GSE247750) and available publicly without restrictions.
