# Supplementary material for: ASAH1 facilitates TNBC by DUSP5 suppression-driven activation of MAP kinase pathway and represents a therapeutic vulnerability
Source: Cell Death Dis. 2024 Jun 26;15(6):452. doi: 10.1038/s41419-024-06831-2 (PMC11208621; doi:10.1038/s41419-024-06831-2)
Supplement: Supplementary file 1 — Supplementary information [file 41419_2024_6831_MOESM1_ESM.pdf]

Supplemental information

Supplementary Figures

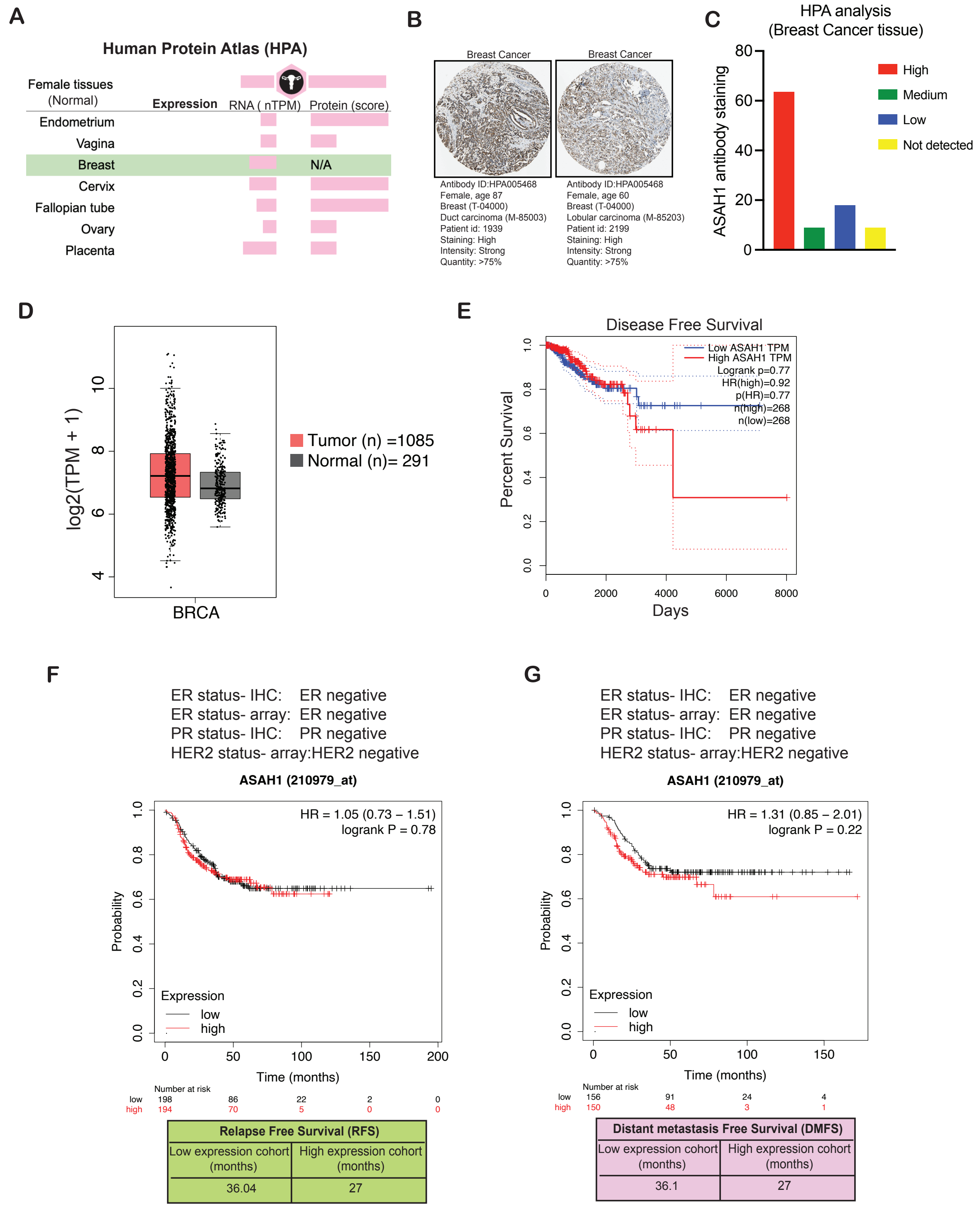

Supplementary Fig. 1. ASAH1 is overexpressed in patient derived samples of breast cancer. A. *ASAH1* mRNA and protein expression in normal tissue was analyzed by using Human Protein Atlas (HPA). **B–C.** Representative IHC images for the stained breast cancer patient samples using Human Protein Atlas (B) and ASAH1 protein expression is plotted in breast cancer patient samples and normal breast using Human Protein Atlas (C). **D**. *ASAH1* mRNA expression in normal tissue and breast cancer tumor tissue was analyzed by using GEPIA. **E**. Disease Free Survival plotted with patient sample expressing high or low ASAH1 using GEPIA. **F**. Relapse Free Survival (RFS) plotted with patient cohort expressing high or low ASAH1 using Km plotter. **G**. Distant metastasis Free Survival (DMFS) plotted with patient cohort expressing high or low ASAH1 using KM plotter.

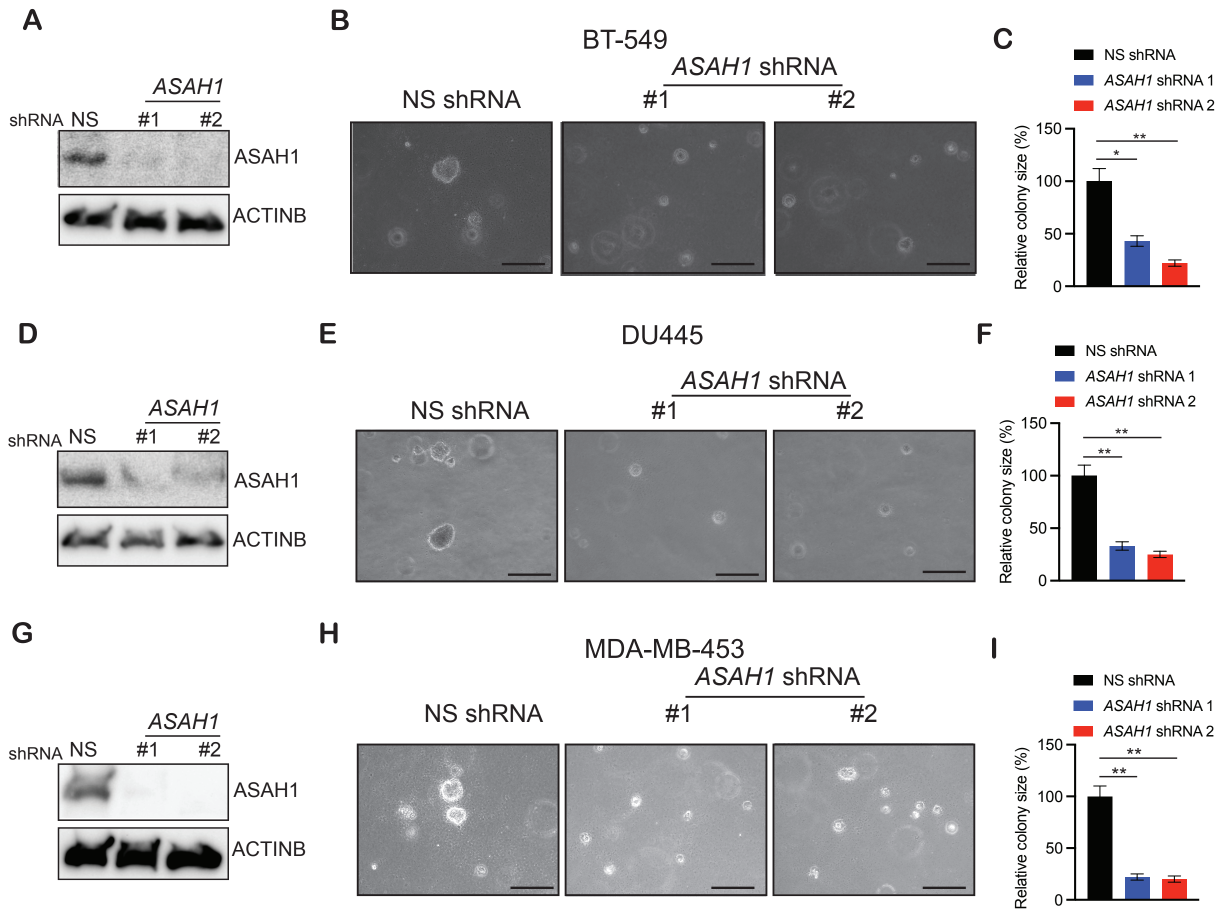

Supplementary Fig. 2. ASAH1 knockdown inhibits the growth of TNBC cells.

A, D, G. TNBC BT-549, DU445 and MDA-MB-453 cells expressing either nonspecific (NS) small hairpin RNA (shRNA) or *ASAH1* shRNAs were analyzed for the ASAH1 knockdown by performing immunoblotting. The ASAH1 protein expression level in *ASAH1* shRNA-expressing cells relative to NS shRNA-expressing cells is presented. ACTINB was used as the loading control. **B, E, H**. TNBC BT-549, DU445 and MDA-MB-453 cells expressing either NS small hairpin RNA (shRNA) or *ASAH1* shRNAs were analyzed for their abilities to grow in soft-agar assay. Representative images are shown with a scale bar of 500 µm. **C, F, I.** Relative colony sizes for the images are shown in panel B, E, H. Data represent the mean ± standard error for three biological replicates. *p<0.05, **p<0.01.

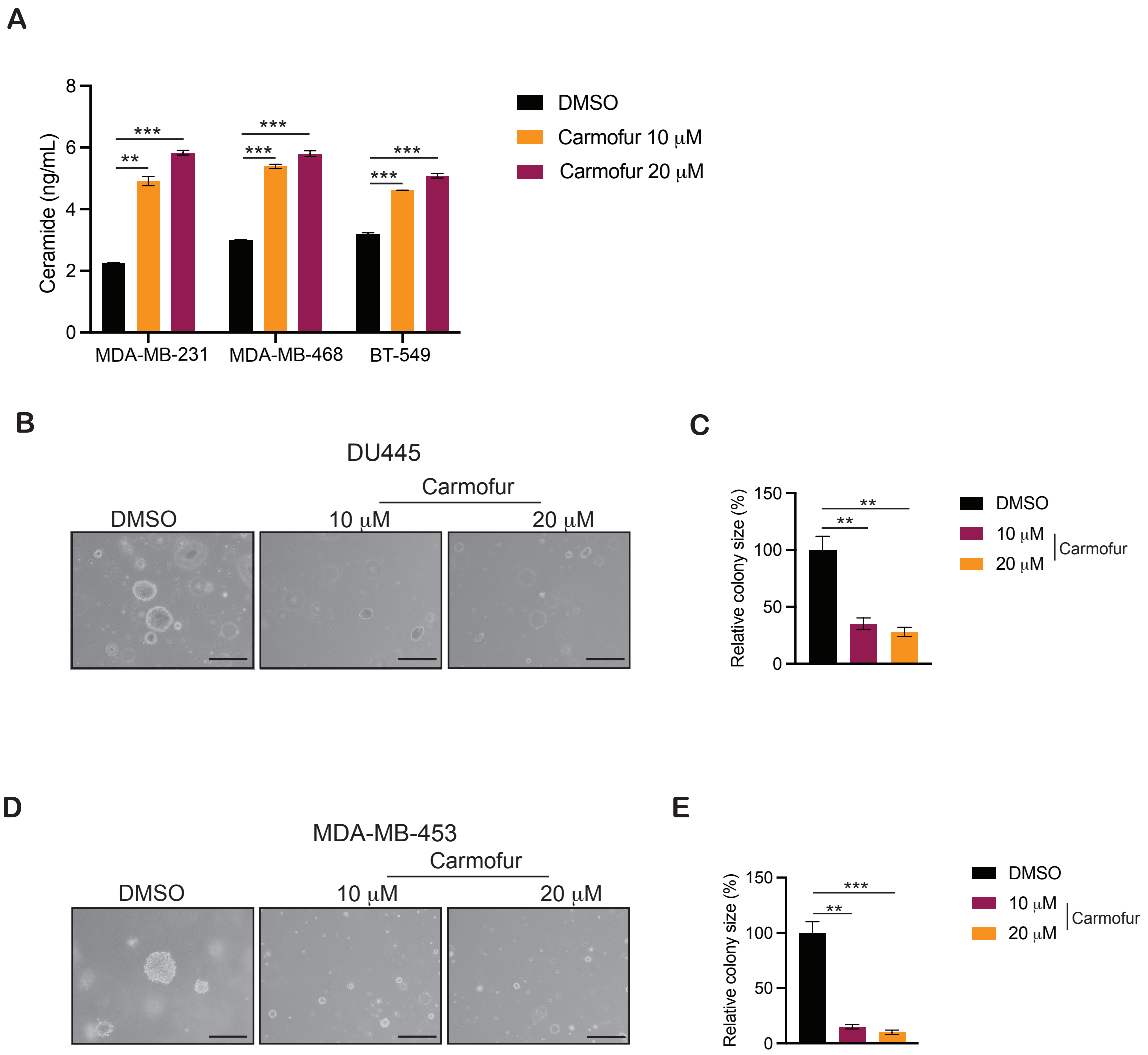

Supplementary Fig. 3. Carmofur treatment increases ceramide levels in TNBC cells and inhibits the growth of TNBC cells.

A. Ceramide levels was measured in indicated TNBC cells MDA-MB-231 and MDA-MB-468 cells upon treatment with either DMSO or different conc. of ceramide for 72 hours using Human ceramide antibody (ceramide-Ab) ELISA Kit (cat. no. MBS3804520). Relative levels in each condition is shown. **B and D.** The indicated TNBC cell lines were treated with different concentrations of carmofur and analyzed for their abilities to grow in soft-agar assay. Representative images are shown with a scale bar of 500 μm. **C and E**. Relative colony sizes for

the images are shown in panel B and D. Data represent the mean ± standard error for three biological replicates. $^{**}p<0.01$, $^{***}p<0.001$.

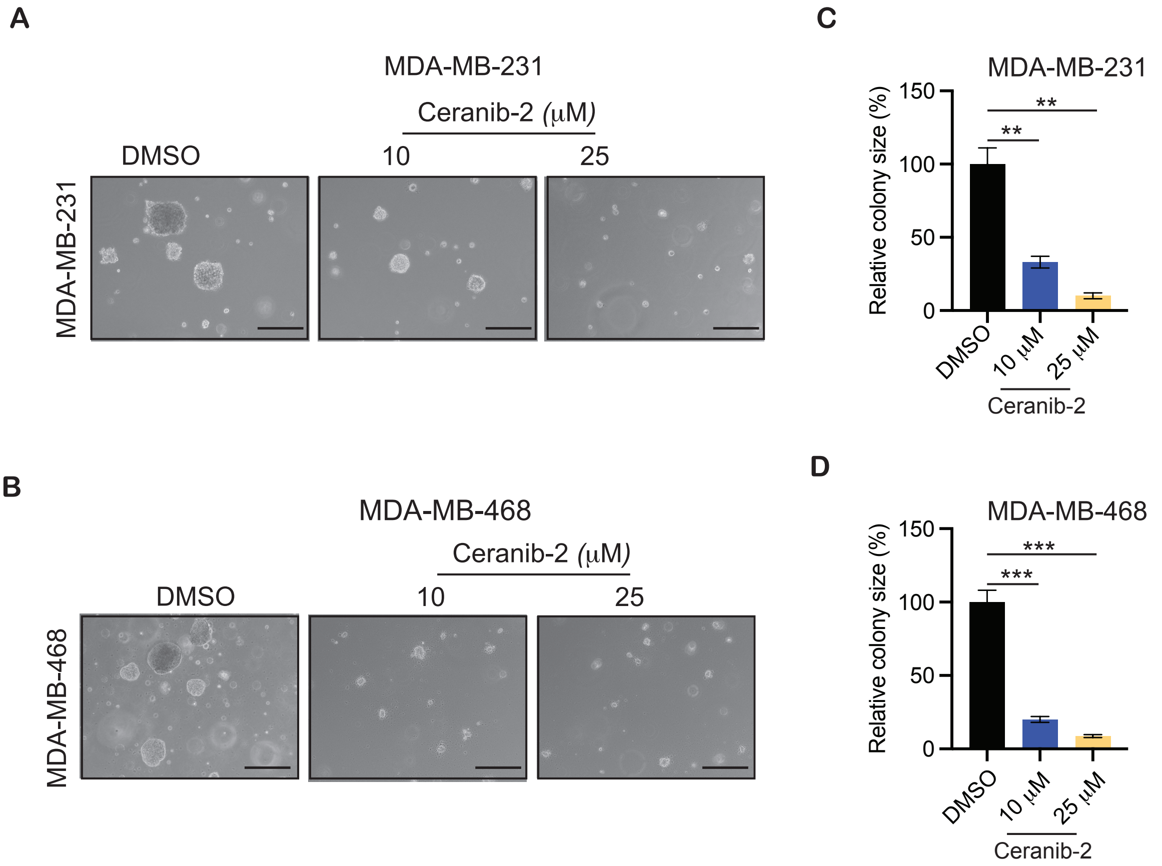

Supplementary Fig. 4. ASAH1 inhibitor ceranib-2 treatment inhibits the growth of TNBC cells.

A, B. The indicated TNBC cell lines were treated with different concentrations of ceranib-2 and analyzed for their abilities to grow in soft-agar assay. Representative images are shown with a scale bar of 500 μm. **C, D**. Relative colony sizes for the images are shown in panel A and B. Data represent the mean ± standard error for three biological replicates. **p<0.01, ***p<0.001.

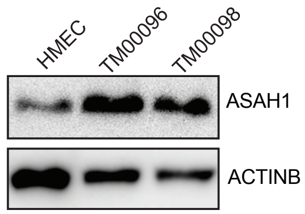

Supplementary Fig. 5. ASAH1 expression in TNBC PDXs.

ASAH1 proteins was measured in PDX's TNBC PDX (TM00096 and TM00098) along with human mammary epithelial cells (HMEC) as control via immunoblotting. ACTINB was used as the loading control.

A

Transcription factors	MATRIX_WIDTH
LCR-F1 [T01599];	6
c-Jun [T00133];	7
VDR [T00885];	4
STAT5A [T04683];	4
NF-1 [T00539];	5
Myf-3 [T00519];	8
MyoD [T00525];	8
c-Myc [T00140];	6
WT1 I [T01840];	7
Elk-1 [T00250];	5
R2 [T00712];	6
p300 [T01427];	5
YY1 [T00915];	7
TCF-4E [T02878];	7
Sp3 [T02338];	9
ETF [T00270];	9
Sp1 [T00759];	9
PKNOX1 [T04122];	11
AP-2alphaA [T00035];	6
MZF-1 [T00529];	7
HSF1 (long) [T01042];	8
HSF1 (short) [T02104];	8
Nkx2-1 [T00857];	7
c-Fos [T00123];	8

B

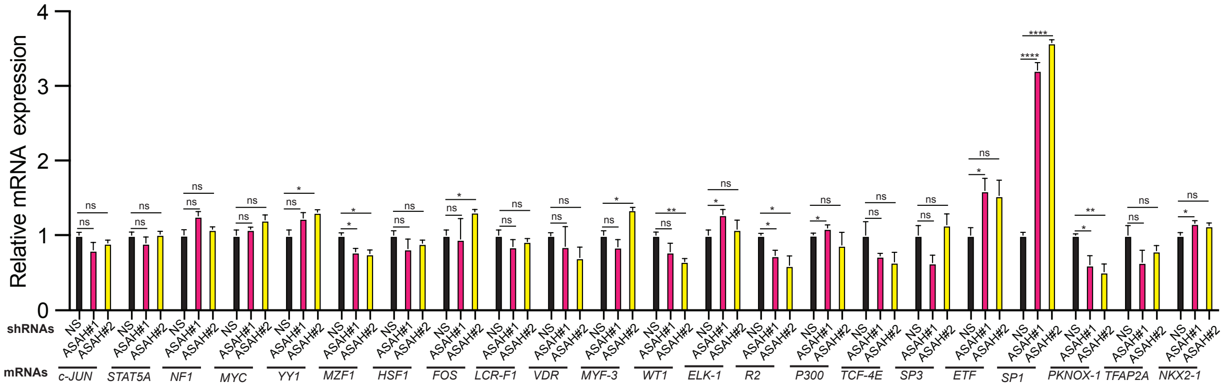

C

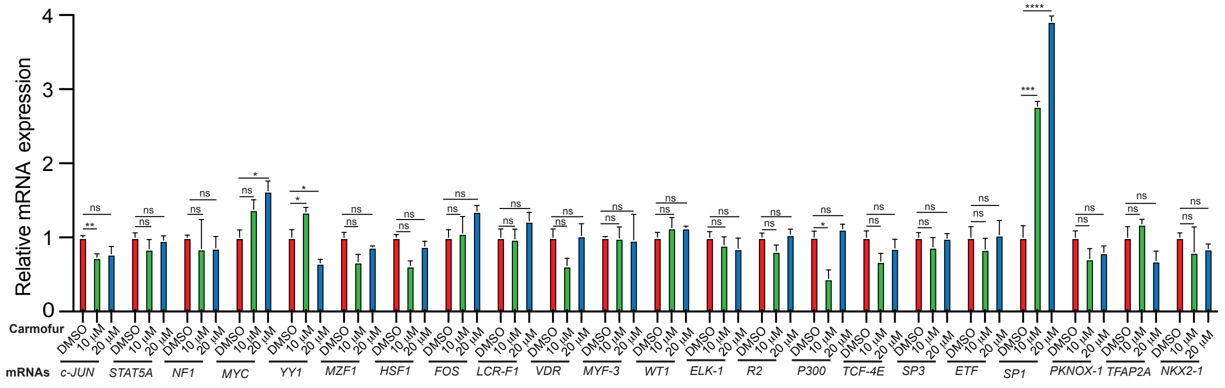

Supplementary Fig. 6. Analysis of the DUSP5 promoter region for transcription factor–binding sites.

A. Analysis of the DUSP5 promoter DNA sequence (~2 kb) for transcription factor consensus DNA-binding sites using the PROMO search tool for putative transcription factor identification with 0% dissimilarity. **B.** Quantitative reverse-transcriptase–polymerase chain reaction (qRT-PCR) was used to measure the mRNA levels of transcription factors identified by RNA sequencing in MDA-MB-231 expressing NS or *ASAH1* shRNA in TNBC cells. *Actin* mRNA was used as the internal control. **C.** Quantitative reverse-transcriptase–polymerase chain reaction (qRT-PCR) was used to measure the mRNA levels of transcription factors identified by RNA sequencing in MDA-MB-231 treated with DMSO or different concentrations of carmofur for 72 hours. Data represent the mean ± standard error for three biological replicates. *$p<0.05$, **$p<0.01$, ****$p<0.001$, ns= not significant.

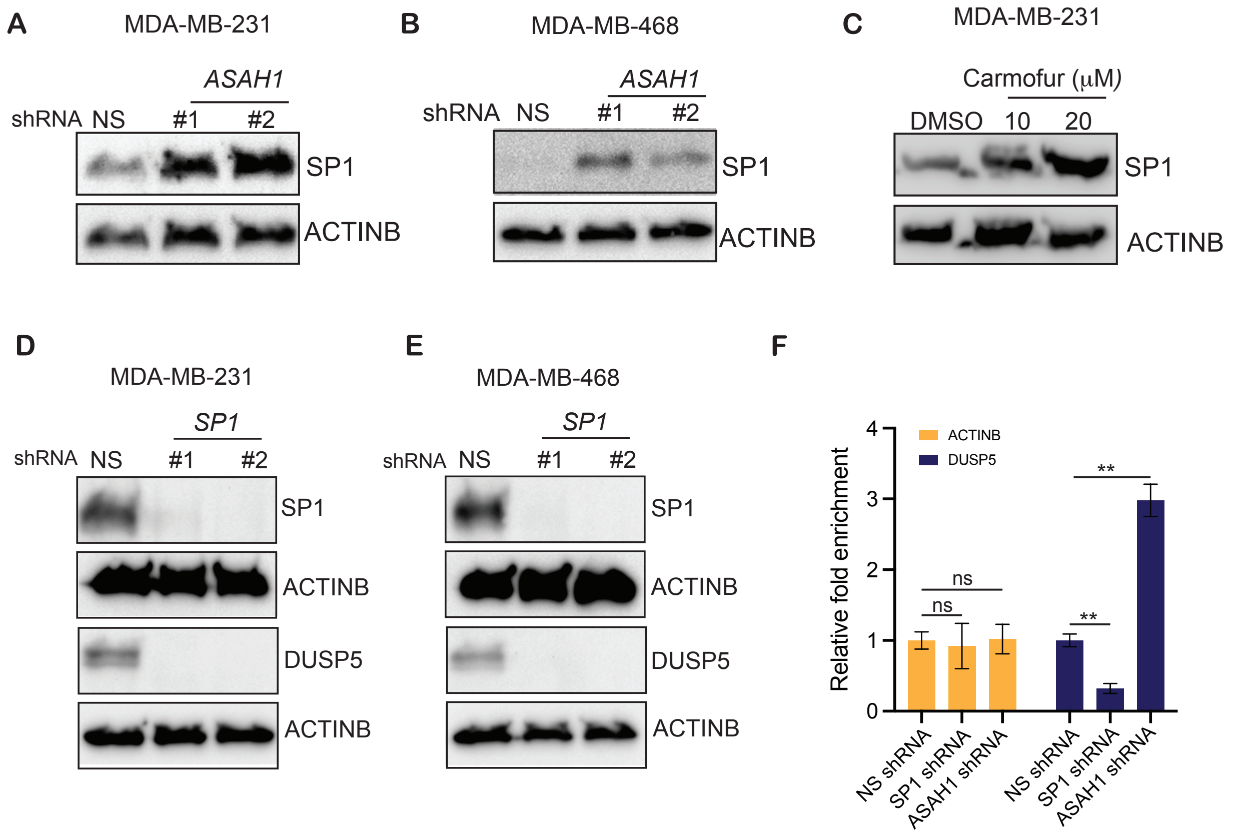

Supplementary Fig. 7. Transcription factor SP1 regulates DUSP5 expression in ASAH1 dependent manner in TNBC cells.

A-C. SP1 protein expression was measured in MDA-MB-231 expressing NS or *ASAH1* shRNA expressing cells (A), MDA-MB-468 expressing NS or *ASAH1* shRNA (B) and in MDA-MB-231 treated with DMSO or different concentrations of carmofur for 72 hours (C). ACTINB was used as loading control. **D.** MDA-MB-231 cells expressing either nonspecific (NS) small hairpin RNA (shRNA) or *SP1* shRNAs were analyzed for the SP1 and DUSP5 protein level by immunoblotting. ACTINB was used as the loading control. **E.** MDA-MB-468 cells expressing either nonspecific (NS) small hairpin RNA (shRNA) or *SP1* shRNAs were analyzed for the SP1 and DUSP5 protein level by immunoblotting. ACTINB was used as the loading control. **F.** MDA-MB-231 cells expressing (NS) small hairpin RNA (shRNA) or *SP1* shRNAs or ASAH1 shRNA were analyzed

using CUT&RUN assay to evaluate the binding of SP1 to the *DUSP5* promoter. Data represent the mean ± standard error for three biological replicates. **$p<0.01$, ns=not significant.

Supplementary Tables:

Supplementary Table 1. Summary of immunohistochemical staining for ASAH1 expression in human TNBC patient samples and matched, normal, adjacent breast tissues. Related to Fig. 1.

Supplementary Table 2: List of differentially regulated genes identified from RNA-sequencing analysis of *ASAH1* shRNA-expressing MDA-MB-231 cells along with NS-shRNA expressing MDA-MB-231cells as control related to Fig. 6.

Supplementary Table 3: List of Reagents, data and software used in this study with source and identifier.

Supplementary Table 1: Summary of immunohistochemical staining for ASAH1 expression in human

No.	Age	Sex	Organ/Anato	Pathology diagno	TNM	Grade
1	53	F	Breast	Invasive carcinon	T2N0M0	2
2	43	F	Breast	Invasive carcinon	T1N1M0	3
3	32	F	Breast	Invasive carcinon	T2N0M0	2
4	47	F	Breast	Invasive carcinon	T3N2M0	2
5	53	F	Breast	Invasive carcinon	T2N0M0	2
6	52	F	Breast	Invasive carcinon	T2N1M0	2
7	37	F	Breast	Invasive carcinon	T2N1M0	3
8	51	F	Breast	Invasive carcinon	T2N2M0	2
9	50	F	Breast	Invasive carcinon	T4N1M0	3
10	49	F	Breast	Invasive carcinon	T2N0M0	2
11	59	F	Breast	Invasive carcinon	T2N0M0	3
12	56	F	Breast	Invasive carcinon	T2N1M0	2
13	45	F	Breast	Invasive carcinon	T1N0M0	2
14	53	F	Breast	Invasive carcinon	T2N0M0	3
15	46	F	Breast	Invasive carcinon	T2N0M0	*
16	70	F	Breast	Invasive carcinon	T2N0M0	2--3
17	42	F	Breast	Invasive carcinon	T2N1M0	3
18	34	F	Breast	Invasive carcinon	T2N0M0	3
19	45	F	Breast	Invasive carcinon	T2N0M0	3
20	38	F	Breast	Invasive carcinon	T2N0M0	3
21	46	F	Breast	Invasive carcinon	T4N0M0	3
1	43	F	Breast	Adjacent normal	-	*
2	48	F	Breast	Adjacent normal	-	-
3	40	F	Breast	Adjacent normal	-	-
4	41	F	Breast	Adjacent normal	-	-
5	38	F	Breast	Adjacent normal	-	*
6	29	F	Breast	Adjacent normal	-	-
7	41	F	Breast	Adjacent normal	-	*
8	42	F	Breast	Adjacent normal	-	*
9	35	F	Breast	Adjacent normal	-	-
10	43	F	Breast	Adjacent normal	-	-

TNBC patient samples and matched, normal, adjacent breast tissues. Related to Fig. 1.

Stage	Type	Tissue ID.	ER	PR	HER2	Intensity
IIA	Malignant	Fmg020031	-	-	0	1+
IB	Malignant	Fmg080098	-	-	0	1+
IIA	Malignant	Fmg010613	-	-	0	2+
IIIA	Malignant	Fmg040661	-	-	0	2+
IIA	Malignant	Fmg010310	-	-	0	1+
IIB	Malignant	Fmg060191	-	-	0	1+
IIB	Malignant	Fmg010612	-	-	0	0
IIIA	Malignant	Fmg010643	-	-	0	1+
IIIB	Malignant	Fmg040675	-	-	0	1+
IIA	Malignant	Fmg010492	-	-	0	0
IIA	Malignant	Fmg040132	-	-	0	2+
IIB	Malignant	Fmg020026	-	-	0	1+
IA	Malignant	Fmg010547	-	-	0	2+
IIA	Malignant	Fmg020765	-	-	0	1+
IIA	Malignant	Fmg030512	-	-	0	0
IIA	Malignant	Fmg030520	-	-	0	1+
IIB	Malignant	Fmg130067	-	-	0	1+
IIA	Malignant	Fmg110075	-	-	0	1+
IIA	Malignant	Fmg110115	-	-	0	3+
IIA	Malignant	Fmg030644	-	-	0	0
IIIB	Malignant	Fmg021845	-	-	0	0
-	NAT	Fmg010001	+	+	0	0
-	NAT	Fmg110129	+	+	0	1+
-	NAT	Fmg021239	+	+	0	0
-	NAT	Fmg140091	+	+	0	1+
-	NAT	Fmg021401	+	+	0	0
-	NAT	Fmg140093	+	+	0	0
-	NAT	Fmg021705	*	*	*	0
-	NAT	Fmg020849	+	+	0	0
-	NAT	Fmg060379	*	*	*	0
-	NAT	Fmg100278	*	*	*	0

	Gene	baseMean	log2FoldChai	lfcSE	stat	pvalue
9	A4GALT	883.99891	-0.6452502	0.08213677	-7.8558023	3.97E-15
12	AAAS	965.213117	-0.3432377	0.0855955	-4.0099973	6.07E-05
21	AADAT	145.014334	-0.5354482	0.1964773	-2.7252422	0.00642543
23	AAGAB	1702.01106	0.30726933	0.06946585	4.42331488	9.72E-06
25	AAMDC	337.550502	-0.4520157	0.13692873	-3.3011023	0.00096306
28	AAR2	2187.64309	0.25535116	0.0677187	3.77076291	0.00016275
30	AARS	9618.258	1.68244702	0.05093168	33.0334096	2.69E-239
31	AARS2	736.157653	0.19164938	0.09127203	2.09976018	0.03574994
34	AASDHPPT	3886.46398	0.65891859	0.05606464	11.752838	6.83E-32
35	AASS	1679.21511	-0.6975612	0.09068699	-7.6919659	1.45E-14
36	AATBC	11.887751	-1.3547536	0.59997816	-2.2580048	0.02394536
37	AATF	6552.05714	0.59366169	0.0678928	8.74410357	2.25E-18
43	ABCA10	152.882544	1.48719769	0.18161836	8.18858693	2.64E-16
44	ABCA11P	344.749414	0.71644187	0.11510413	6.22429312	4.84E-10
48	ABCA2	2985.99066	-1.5559445	0.0705643	-22.050024	9.55E-108
52	ABCA6	77.034814	-2.1353667	0.26358483	-8.1012502	5.44E-16
53	ABCA7	998.78605	-1.1677744	0.08842664	-13.206138	8.09E-40
58	ABCB10	1271.06568	-0.7879467	0.07503204	-10.50147	8.50E-26
62	ABCB6	617.946826	-0.9046644	0.10363251	-8.7295428	2.56E-18
72	ABCC3	5177.38566	-0.7620171	0.05154283	-14.784154	1.85E-49
76	ABCC6	45.9228552	-1.3001739	0.30319332	-4.2882671	1.80E-05
81	ABCD1	838.874414	-1.0930882	0.0925171	-11.814985	3.27E-32
85	ABCE1	6369.99233	0.15345961	0.06036693	2.54211391	0.01101843
86	ABCF1	9345.04306	0.27593622	0.05360345	5.14773273	2.64E-07
87	ABCF2	5832.34761	0.57493793	0.06221265	9.24149561	2.43E-20
88	ABCF3	2816.89175	-0.2873582	0.06806148	-4.2220383	2.42E-05
90	ABCG2	157.214669	1.71456147	0.17635688	9.72211288	2.43E-22
97	ABHD11-AS1	8.69238347	-1.5768853	0.72232434	-2.183071	0.02903058
98	ABHD12	5525.20387	-0.2591084	0.05756211	-4.5013716	6.75E-06
101	ABHD14A	307.658797	-0.6551684	0.14283476	-4.5868976	4.50E-06
104	ABHD15	530.145308	-0.9484437	0.10317534	-9.1925419	3.84E-20
107	ABHD17A	667.071475	-0.814528	0.09752676	-8.3518404	6.72E-17
109	ABHD17C	1639.22211	-1.0058491	0.06997017	-14.375399	7.38E-47
110	ABHD2	3744.53134	-0.191178	0.09277325	-2.0607013	0.03933154
111	ABHD3	859.278193	-0.4419782	0.08786908	-5.0299632	4.91E-07
112	ABHD4	2099.25703	-0.5117056	0.07956544	-6.4312549	1.27E-10
120	ABL1	4205.78899	0.30184376	0.06685891	4.51463764	6.34E-06
121	ABL2	7545.12441	1.35707798	0.07550438	17.9734998	3.14E-72
122	ABLIM1	4826.56984	-0.3347745	0.07516547	-4.4538345	8.44E-06
123	ABLIM2	53.7205424	-2.6805368	0.33054386	-8.1094738	5.08E-16
130	ABTB1	1011.99836	-0.8365053	0.10686996	-7.8273195	4.98E-15
131	ABTB2	2127.24361	0.77654185	0.06635106	11.7035331	1.22E-31

132	ACAA1	1685.49553	-0.3669337	0.09604218	-3.8205478	0.00013316
133	ACAA2	1215.74635	-0.7182813	0.08286111	-8.6684969	4.38E-18
135	ACACB	208.926107	-1.5471646	0.16072452	-9.6261889	6.20E-22
136	ACAD10	516.917099	-0.3501112	0.10178407	-3.4397448	0.00058226
141	ACADM	1664.34224	0.45279747	0.0690973	6.5530417	5.64E-11
142	ACADS	499.194368	-0.803186	0.11073704	-7.2530926	4.07E-13
143	ACADSB	629.125669	-0.9559981	0.09277653	-10.304311	6.74E-25
149	ACAT1	6133.15021	-0.2395734	0.06589499	-3.6356854	0.00027724
150	ACAT2	2762.27307	-0.4723675	0.08409805	-5.6168668	1.94E-08
153	ACBD5	1946.23389	-0.338765	0.08848874	-3.8283408	0.00012901
156	ACCS	239.299254	-0.4781909	0.15479303	-3.0892276	0.00200678
164	ACHE	64.3912306	1.43466424	0.2678808	5.35560691	8.53E-08
165	ACIN1	5829.60548	-0.4695293	0.0564833	-8.3127103	9.35E-17
172	ACO1	9387.48916	-0.1877093	0.05120826	-3.6656064	0.00024675
173	ACO2	3559.85146	-0.2188437	0.05645604	-3.8763555	0.00010603
174	ACOT1	33.0383406	-1.4443255	0.38481671	-3.7532817	0.00017453
175	ACOT11	14.9842078	-1.5271996	0.53657627	-2.8461929	0.00442454
178	ACOT2	407.735483	-0.3367996	0.12159073	-2.7699447	0.00560658
184	ACOX1	1175.36477	-0.2902831	0.07400214	-3.9226309	8.76E-05
185	ACOX2	88.4892689	-0.4524808	0.20767269	-2.178817	0.02934527
186	ACOX3	652.735421	-0.9832361	0.09770234	-10.063588	8.00E-24
187	ACOXL	102.333273	1.14734729	0.2136626	5.36990225	7.88E-08
188	ACP1	2806.98394	0.39494694	0.06230559	6.3388686	2.31E-10
190	ACP5	17.9466033	-1.7778272	0.52574283	-3.3815529	0.00072077
200	ACSF2	1353.81708	-0.2934381	0.07494316	-3.9154757	9.02E-05
204	ACSL4	6586.66023	0.20492979	0.07106486	2.88370061	0.00393032
214	ACSS1	1040.05487	-1.8701998	0.08568919	-21.825389	1.33E-105
215	ACSS2	1516.68364	-0.8730081	0.0745877	-11.704452	1.21E-31
216	ACSS3	116.765352	0.81674958	0.21334991	3.82821608	0.00012908
220	ACTB	136308.08	-0.8870926	0.06146868	-14.43162	3.27E-47
223	ACTG1	90793.5857	-0.6583191	0.05293201	-12.437068	1.64E-35
239	ACTN4	27914.8263	-0.5880262	0.0694996	-8.4608585	2.65E-17
241	ACTR1A	6154.93227	-0.5386762	0.07752796	-6.9481535	3.70E-12
243	ACTR2	9239.19562	0.24974214	0.06199648	4.02832779	5.62E-05
249	ACTR5	634.135504	0.66922912	0.09602149	6.96957639	3.18E-12
250	ACTR6	890.53301	-0.4665411	0.07812238	-5.9719267	2.34E-09
254	ACTRT3	92.6472938	0.97179126	0.22122158	4.39284121	1.12E-05
256	ACVR1B	846.507416	0.2560479	0.08222549	3.11397224	0.00184587
258	ACVR2A	506.528768	0.49567599	0.0986131	5.0264721	5.00E-07
266	ADA	137.426622	-0.4602621	0.20473319	-2.2481068	0.02456938
270	ADAM10	10646.4096	-1.2955932	0.07010655	-18.480345	2.97E-76
273	ADAM15	5643.18118	0.26550115	0.05823094	4.55945154	5.13E-06
274	ADAM17	2258.92575	0.28473317	0.09177209	3.10261189	0.00191821

283	ADAM22	343.87984	-2.1251976	0.13076155	-16.252466	2.15E-59
285	ADAM28	835.190973	-1.3604284	0.08949064	-15.201907	3.43E-52
294	ADAM8	8310.33983	-0.7964064	0.05165341	-15.418275	1.23E-53
299	ADAMTS12	2108.22916	0.91726829	0.12181132	7.53023838	5.06E-14
301	ADAMTS14	187.793852	-1.0595678	0.16178352	-6.5492935	5.78E-11
302	ADAMTS15	3200.60486	-1.4891104	0.06799425	-21.900533	2.57E-106
313	ADAMTS6	1334.18101	3.59367046	0.11478357	31.3082297	3.61E-215
314	ADAMTS7	135.509476	-0.8350945	0.17513964	-4.768164	1.86E-06
317	ADAMTS9	1624.33775	0.80836238	0.08076839	10.0084008	1.40E-23
323	ADAMTSL4	639.347818	-0.2333391	0.08864856	-2.632182	0.00848384
333	ADAT2	328.360368	0.85349393	0.11897309	7.17384012	7.29E-13
336	ADCK2	1483.29869	-0.3929937	0.0785884	-5.0006586	5.71E-07
338	ADCK4	556.987662	-0.6156305	0.09822236	-6.267722	3.66E-10
339	ADCK5	760.100606	-0.5122616	0.09387273	-5.4569795	4.84E-08
353	ADD1	7867.96227	-0.5469632	0.04908344	-11.143539	7.70E-29
355	ADD3	3500.70451	-0.5983009	0.07149105	-8.3688921	5.82E-17
365	ADGRD1	410.441478	-0.589731	0.11087129	-5.3190594	1.04E-07
375	ADGRF5	11621.8879	-0.6786964	0.08985232	-7.5534654	4.24E-14
393	ADH5	4321.92099	-0.3928623	0.05737397	-6.847396	7.52E-12
397	ADI1	4338.81458	-0.3495206	0.07675276	-4.55385	5.27E-06
403	ADIRF	418.553859	-0.3802151	0.11408728	-3.3326682	0.00086017
405	ADM	1261.98666	-0.2958321	0.10102272	-2.9283723	0.00340742
406	ADM2	591.828518	3.66835806	0.12558935	29.2091499	1.48E-187
408	ADNP	5698.85039	0.24935431	0.05956994	4.18590858	2.84E-05
411	ADO	2614.01379	0.79903545	0.06698784	11.928068	8.45E-33
412	ADORA1	73.6323232	-1.5642963	0.29479011	-5.306475	1.12E-07
414	ADORA2A-AS	15.7330557	-1.1853709	0.51276703	-2.3117144	0.02079343
415	ADORA2B	4495.30684	1.72790684	0.05732405	30.1427897	1.33E-199
417	ADPGK	1966.69986	-0.5944203	0.06684124	-8.8930163	5.95E-19
422	ADPRM	530.372174	2.03084312	0.11776077	17.2454983	1.21E-66
429	ADRB1	8.09445554	-1.5249334	0.73095456	-2.0862219	0.03695853
430	ADRB2	2281.40791	0.52825706	0.06879117	7.67914009	1.60E-14
432	ADRBK1	3303.52973	-0.3336442	0.05614507	-5.9425374	2.81E-09
434	ADRM1	8524.51786	0.75535014	0.06677581	11.3117326	1.15E-29
438	ADTRP	224.076034	2.38561662	0.157302	15.1658377	5.95E-52
440	AEBP2	1228.45466	0.22462263	0.08005872	2.80572334	0.00502038
441	AEN	1554.55981	1.76695137	0.06822324	25.899551	6.74E-148
444	AFAP1-AS1	1100.01279	-0.353177	0.09105608	-3.8786751	0.00010503
446	AFAP1L2	2107.28993	-0.4833136	0.06089323	-7.9370658	2.07E-15
447	AFF1	2733.54137	0.20147983	0.0967682	2.08208722	0.0373345
450	AFF4	7289.38811	0.53860224	0.09068942	5.9389755	2.87E-09
452	AFG3L2	5458.19236	0.31802679	0.07394573	4.30081361	1.70E-05
454	AFMID	757.731375	-0.5667133	0.08863889	-6.3935062	1.62E-10

456	AFTPH	1986.7845	0.43830688	0.06961326	6.29631339	3.05E-10
460	AGAP11	61.665411	2.50716065	0.34772918	7.21009566	5.59E-13
462	AGAP2-AS1	582.473801	-0.5394063	0.09822321	-5.4916383	3.98E-08
468	AGAP9	257.926085	-0.4653721	0.14626732	-3.1816546	0.00146436
478	AGFG1	2916.43092	0.57608027	0.07782179	7.40255725	1.34E-13
480	AGGF1	1794.32137	-0.4585738	0.08391508	-5.4647358	4.64E-08
482	AGL	1302.04647	-0.3681865	0.09358882	-3.9340861	8.35E-05
483	AGMAT	205.704053	1.16613424	0.15595463	7.4773941	7.58E-14
486	AGO2	1035.95719	0.36577888	0.11594608	3.15473257	0.00160645
487	AGO3	487.137832	0.91048579	0.12442859	7.31733582	2.53E-13
495	AGPS	4808.89171	0.30232821	0.07106729	4.25411219	2.10E-05
496	AGR2	19.4722892	-1.5888226	0.46361834	-3.4270054	0.00061028
501	AGTPBP1	2887.31016	0.19594192	0.07591863	2.58094638	0.00985299
510	AHCYL1	7308.98344	-0.6171229	0.07785053	-7.9270233	2.24E-15
511	AHCYL2	756.240193	-0.4775192	0.08992142	-5.3104056	1.09E-07
513	AHI1	1014.98618	-0.2011085	0.10094013	-1.9923545	0.04633217
516	AHR	7833.64877	0.72298406	0.08944794	8.08273579	6.33E-16
517	AHRR	281.583742	1.81407224	0.13579615	13.35879	1.05E-40
519	AHSA2	536.028511	-0.5750283	0.10339851	-5.5612821	2.68E-08
533	AIMP1	2269.00496	0.47954821	0.06045325	7.93254575	2.15E-15
534	AIMP2	2730.32217	0.90755237	0.09324397	9.73309483	2.18E-22
535	AIP	3276.17023	-0.6299509	0.08627518	-7.301647	2.84E-13
540	AJUBA	11777.1057	0.24575958	0.05271887	4.6617005	3.14E-06
541	AK1	3254.30618	-0.418083	0.10501154	-3.9813055	6.85E-05
542	AK2	4291.65423	0.70887138	0.05767984	12.28976	1.03E-34
546	AK6	1841.4662	0.30034068	0.09138021	3.28671469	0.00101363
550	AKAP1	3130.74829	0.53259057	0.05715472	9.31839978	1.18E-20
552	AKAP11	2071.75526	0.26122479	0.08513124	3.06849491	0.0021514
560	AKAP5	95.0098648	-1.1850195	0.21481754	-5.5164	3.46E-08
564	AKAP8L	2336.9052	0.30992583	0.06479127	4.78345034	1.72E-06
567	AKIRIN1	2549.91496	0.35775198	0.05986283	5.97619605	2.28E-09
568	AKIRIN2	1733.21544	1.03899134	0.06846996	15.1744121	5.22E-52
571	AKR1A1	2062.43066	0.33583707	0.10420246	3.22292833	0.00126887
576	AKR1C2	474.091391	-3.3921565	0.13264118	-25.573932	2.98E-144
592	ALAD	1291.4303	-1.2793046	0.08073241	-15.846235	1.49E-56
597	ALDH16A1	1772.0225	-0.3409022	0.09442147	-3.6104315	0.00030569
601	ALDH1A3	1407.65559	-0.3448534	0.08075802	-4.2702064	1.95E-05
603	ALDH1L1	114.200211	-1.352894	0.20649656	-6.5516542	5.69E-11
607	ALDH2	2847.92008	-0.6141221	0.07437688	-8.2568951	1.50E-16
609	ALDH3A2	1945.81984	-1.5612624	0.07112655	-21.950488	8.57E-107
610	ALDH3B1	1760.47021	-1.2618961	0.0652272	-19.346164	2.20E-83
612	ALDH4A1	508.983486	-0.2896465	0.10551334	-2.7451173	0.00604893
613	ALDH5A1	221.340696	-1.5146466	0.14385953	-10.52865	6.37E-26

614	ALDH6A1	611.725038	-0.3611665	0.09882355	-3.6546597	0.00025752
615	ALDH7A1	2938.77748	-0.5590542	0.07980239	-7.0054819	2.46E-12
618	ALDOA	31824.4357	-0.7207429	0.05233393	-13.772001	3.76E-43
620	ALDOC	306.835866	-1.4407834	0.13161245	-10.947167	6.86E-28
625	ALG12	789.590288	-0.5809875	0.08904597	-6.5245788	6.82E-11
636	ALG9	1504.52077	-0.3395121	0.07371752	-4.6055826	4.11E-06
638	ALKBH1	481.626194	0.82866952	0.10006068	8.28166947	1.21E-16
640	ALKBH3	1083.60828	0.33123606	0.08757751	3.78220466	0.00015545
643	ALKBH5	3584.29592	-0.849311	0.06061163	-14.012342	1.31E-44
644	ALKBH6	634.939622	-0.3143567	0.09996522	-3.1446602	0.0016628
645	ALKBH7	683.826826	-0.9728765	0.12578318	-7.7345517	1.04E-14
646	ALKBH8	737.761817	0.28317844	0.0897856	3.15394039	0.00161082
648	ALMS1	645.722896	0.42467203	0.14163518	2.99835133	0.00271445
658	ALOX5	362.503519	-0.9909956	0.11254288	-8.8054931	1.30E-18
660	ALOXE3	103.990454	4.38381259	0.32563098	13.462517	2.60E-41
662	ALPK1	197.511004	-0.6268214	0.1614251	-3.8830476	0.00010316
663	ALPK2	385.736555	1.9915615	0.12371587	16.0978661	2.64E-58
665	ALPL	4.15378086	2.38757128	1.1311176	2.11080729	0.03478888
668	ALS2	2089.95203	0.3580729	0.07023646	5.09810552	3.43E-07
669	ALS2CL	1394.41386	-0.3051768	0.07401712	-4.1230565	3.74E-05
670	ALS2CR11	169.328438	-0.7155138	0.17114756	-4.1806835	2.91E-05
676	AMACR	65.8727476	-0.5949742	0.27784336	-2.1414017	0.03224166
679	AMBRA1	1135.44609	-0.2063476	0.08545443	-2.4147089	0.01574779
680	AMD1	6420.60581	0.68388936	0.05650503	12.1031589	1.02E-33
685	AMER1	291.926679	0.5241794	0.13807388	3.79636896	0.00014683
689	AMH	388.022593	-0.9853323	0.11036723	-8.9277612	4.35E-19
692	AMIGO1	239.507101	-0.5770261	0.15397496	-3.7475323	0.00017858
693	AMIGO2	5829.01228	0.81527432	0.06754286	12.0704735	1.51E-33
695	AMMECR1	513.64931	0.41092754	0.09944777	4.13209388	3.59E-05
696	AMMECR1L	2454.15795	0.93613659	0.07057471	13.2644771	3.72E-40
697	AMN	78.6987005	0.58220617	0.23812594	2.44495063	0.0144872
703	AMPD2	2794.38153	-0.7989023	0.06068264	-13.165251	1.39E-39
706	AMT	255.883809	-0.7756639	0.13422308	-5.7789158	7.52E-09
712	AMY2B	80.4093317	-1.1653144	0.27184464	-4.2866927	1.81E-05
713	AMZ1	59.2688494	4.53356899	0.43659965	10.3838127	2.94E-25
718	ANAPC11	3095.6204	-0.7010096	0.20968304	-3.3431867	0.00082822
719	ANAPC13	2010.8004	0.47597882	0.07463376	6.37752734	1.80E-10
720	ANAPC15	1114.03419	-0.4035304	0.10343137	-3.9014312	9.56E-05
723	ANAPC2	1565.25531	-0.720271	0.07335864	-9.818488	9.37E-23
724	ANAPC4	819.466138	-0.4119368	0.08245917	-4.9956461	5.86E-07
730	ANGPT1	156.536226	-2.7303531	0.22085205	-12.362815	4.15E-35
734	ANGPTL2	1942.9287	-1.5462454	0.06735289	-22.957372	1.24E-116
741	ANK1	677.976792	-0.861017	0.09294936	-9.2632914	1.98E-20

742	ANK2	592.138511	-0.5843737	0.1124998	-5.1944419	2.05E-07
744	ANKAR	76.7735356	-0.6671563	0.23521171	-2.8364079	0.00456241
745	ANKDD1A	59.2078442	-0.7201351	0.26520947	-2.7153447	0.00662068
750	ANKH	1288.31558	-0.5506425	0.07984844	-6.8960958	5.35E-12
762	ANKRD11	5892.00681	0.67619166	0.08422415	8.02847665	9.87E-16
764	ANKRD13A	3039.37041	0.53600908	0.08411304	6.37248493	1.86E-10
765	ANKRD13B	601.41696	1.0729412	0.11016712	9.73921436	2.05E-22
766	ANKRD13C	2264.60171	0.21514494	0.07311164	2.94269065	0.00325373
767	ANKRD13D	1705.02919	-0.7567515	0.10048327	-7.5311194	5.03E-14
769	ANKRD17	4186.9361	0.21210512	0.08589897	2.4692395	0.01354006
774	ANKRD2	30.7460643	-1.1178421	0.37904212	-2.9491238	0.00318676
791	ANKRD27	2594.58733	0.37514845	0.06173043	6.07720449	1.22E-09
792	ANKRD28	5411.47331	0.14772902	0.05954723	2.48087147	0.01310616
799	ANKRD31	11.554635	2.27070047	0.66732006	3.40271572	0.0006672
802	ANKRD34A	87.7006039	0.91703868	0.2372093	3.86594742	0.00011066
807	ANKRD36	101.758541	-0.6328765	0.19758159	-3.2031148	0.0013595
808	ANKRD36B	54.7541423	-1.0345061	0.29774983	-3.4744137	0.00051197
812	ANKRD37	224.51324	-1.4272131	0.16545929	-8.6257659	6.37E-18
821	ANKRD50	2095.23723	0.56061588	0.08492864	6.601023	4.08E-11
826	ANKRD6	460.790659	-0.2665383	0.10501587	-2.5380764	0.01114636
832	ANKRD65	53.3597444	-1.8214149	0.34784004	-5.2363578	1.64E-07
842	ANKZF1	1726.15281	-1.6691441	0.0739499	-22.571283	8.30E-113
847	ANO2	10.6055849	2.30995619	0.68928909	3.35121538	0.00080458
853	ANO8	964.229188	-1.6138848	0.08291724	-19.463803	2.23E-84
855	ANOS1	705.837867	0.34714426	0.11071587	3.1354516	0.0017159
864	ANTXR1	2796.7943	-0.474006	0.08395148	-5.6461901	1.64E-08
869	ANXA10	17.0305552	1.73472807	0.51541577	3.36568683	0.00076353
870	ANXA11	6893.09277	-0.1849591	0.04963675	-3.7262533	0.00019435
878	ANXA4	2476.78148	-0.1296563	0.06366108	-2.0366651	0.04168362
880	ANXA6	10885.3324	-0.5152444	0.04994422	-10.316396	5.94E-25
882	ANXA8	235.463857	-1.4848579	0.15863172	-9.36041	7.94E-21
883	ANXA8L1	160.213178	-1.4667423	0.17233556	-8.5109672	1.72E-17
888	AOC2	139.694044	1.95723946	0.2052653	9.53516976	1.50E-21
889	AOC3	27.107939	1.23584177	0.41716859	2.96245161	0.003052
893	AP1AR	1840.32536	0.18146841	0.06506324	2.78910792	0.00528534
898	AP1M1	4533.73724	-0.28356	0.05488088	-5.1668262	2.38E-07
901	AP1S2	4041.31406	-0.4115716	0.05518341	-7.4582486	8.77E-14
903	AP2A1	6212.19581	-0.2040408	0.05215675	-3.9120681	9.15E-05
904	AP2A2	4114.25254	-0.1846987	0.062247	-2.9671895	0.00300536
907	AP2S1	6062.65187	-0.3515729	0.10745886	-3.2716975	0.00106904
912	AP3M2	1393.58896	0.69443086	0.07062812	9.83221429	8.18E-23
921	AP5M1	1745.51286	-0.8686375	0.07408439	-11.724973	9.49E-32
922	AP5S1	716.38026	-0.2718822	0.08891659	-3.0577215	0.00223027

925	APBA1	20.9862102	-1.2350575	0.45257674	-2.728946	0.00635371
928	APBB1	1587.77662	-0.5757137	0.08179898	-7.0381534	1.95E-12
936	APCDD1L-AS:	197.948795	1.25508002	0.15079313	8.32319119	8.56E-17
938	APEH	2560.54049	-0.5997096	0.06613585	-9.0678441	1.21E-19
940	APEX1	4970.91227	-0.4886865	0.05456247	-8.9564586	3.35E-19
942	APH1A	6730.01236	-0.1425769	0.05616607	-2.5384886	0.01113324
943	APH1B	730.901773	-0.4548975	0.0842046	-5.4022884	6.58E-08
949	APLN	357.591578	-2.6659129	0.1322988	-20.150697	2.65E-90
952	APLP2	46156.352	-0.5347487	0.04786034	-11.173108	5.52E-29
953	APMAP	8483.98063	-0.480578	0.07482523	-6.4226731	1.34E-10
965	APOBEC3B	524.046654	-0.2657957	0.11100584	-2.3944298	0.01664623
967	APOBEC3C	398.81854	-0.7884475	0.10796473	-7.3028242	2.82E-13
984	APOL1	909.246225	-0.9625064	0.08151462	-11.807777	3.56E-32
995	APOPT1	1008.33969	0.4023246	0.08653236	4.64941186	3.33E-06
997	APPBP2	2133.87936	0.79010992	0.07728547	10.2232654	1.56E-24
1001	APTR	339.070061	0.70004672	0.13921664	5.02847027	4.94E-07
1002	APTX	1423.7173	0.30703695	0.07576243	4.05262819	5.06E-05
1009	AQP3	597.616428	-1.5130355	0.09576419	-15.799596	3.13E-56
1012	AQP5	41.2093652	-2.0075781	0.33700562	-5.9571058	2.57E-09
1013	AQP6	12.2922192	-3.3100179	0.79902009	-4.1425966	3.43E-05
1021	ARAF	2950.43926	-0.4482198	0.0614176	-7.2979047	2.92E-13
1022	ARAP1	4699.09243	-0.2101179	0.05797646	-3.6241931	0.00028987
1023	ARAP2	337.090275	0.45334351	0.13760877	3.29443754	0.00098619
1024	ARAP3	1595.89104	-0.1715167	0.06655085	-2.5772278	0.00995963
1025	ARC	17.2728742	3.0973355	0.60619858	5.10944036	3.23E-07
1026	ARCN1	14106.4152	0.3309747	0.06555535	5.04878242	4.45E-07
1027	AREG	1798.29265	1.08588467	0.07137569	15.2136477	2.87E-52
1028	AREL1	2305.00132	-0.573369	0.07358959	-7.791442	6.62E-15
1029	ARF1	16131.8154	-0.3424977	0.04615834	-7.4200619	1.17E-13
1030	ARF3	4991.8412	-0.3574306	0.06768685	-5.2806505	1.29E-07
1031	ARF4	14642.6697	0.19121481	0.05070445	3.77116414	0.00016249
1032	ARF5	3804.38807	-0.3426916	0.084067	-4.0764103	4.57E-05
1033	ARF6	10047.6345	0.2474392	0.05962273	4.15008161	3.32E-05
1034	ARFGAP1	5246.80308	0.20007806	0.05647626	3.54269318	0.00039606
1036	ARFGAP3	2408.14798	0.24809619	0.05949684	4.16990531	3.05E-05
1037	ARFGEF1	1965.9094	0.40354777	0.06840683	5.89923241	3.65E-09
1038	ARFGEF2	5679.86188	0.18911281	0.08060517	2.34616235	0.01896784
1050	ARHGAP11A	2287.87783	-0.2744172	0.07811389	-3.5130394	0.00044301
1063	ARHGAP25	87.9437022	-1.09062	0.22360794	-4.8773759	1.08E-06
1067	ARHGAP27	1690.70314	-0.2926842	0.08037176	-3.6416304	0.00027092
1069	ARHGAP29	11652.3842	0.655782	0.0817649	8.02033638	1.05E-15
1073	ARHGAP32	3755.51235	0.16926956	0.07238993	2.33830263	0.01937155
1074	ARHGAP33	312.901859	-0.3554289	0.12085626	-2.940923	0.00327236

1077	ARHGAP39	1314.38022	0.9181235	0.07197807	12.7556005	2.90E-37
1083	ARHGAP5-AS	401.305657	0.66997109	0.11411952	5.87078456	4.34E-09
1087	ARHGDIA	21459.3424	0.25499001	0.05122398	4.97794177	6.43E-07
1088	ARHGDIB	842.522039	-1.5498804	0.0932951	-16.612666	5.64E-62
1090	ARHGEF1	3395.90639	-0.369626	0.06697936	-5.5185049	3.42E-08
1096	ARHGEF16	131.088201	0.3578075	0.18116743	1.97501002	0.04826701
1097	ARHGEF17	1868.52738	-0.5448083	0.07956807	-6.8470721	7.54E-12
1098	ARHGEF18	4736.19079	0.27638766	0.05773484	4.78719027	1.69E-06
1100	ARHGEF2	5043.46818	1.49425826	0.0533734	27.9963115	1.80E-172
1101	ARHGEF25	124.127697	-0.4614022	0.18938017	-2.4363807	0.01483506
1110	ARHGEF37	144.508897	-0.7161667	0.19017917	-3.7657472	0.00016605
1114	ARHGEF4	1026.39429	-0.7631217	0.08070468	-9.4557302	3.21E-21
1121	ARHGEF9	450.486109	-0.5736508	0.11678861	-4.9118727	9.02E-07
1123	ARID1A	2853.38495	0.77127079	0.09451739	8.1600938	3.35E-16
1127	ARID3B	123.976636	1.18083392	0.18704418	6.31312844	2.73E-10
1133	ARIH1	2392.81361	0.2976075	0.06832094	4.35602144	1.32E-05
1140	ARL13B	1549.15499	0.71318892	0.07280003	9.79654666	1.17E-22
1142	ARL14EP	1380.60826	0.87209244	0.07240043	12.0454041	2.05E-33
1143	ARL14EPL	31.6023412	0.70485465	0.35155164	2.00498184	0.04496499
1145	ARL16	965.311327	0.27243879	0.08205407	3.32023492	0.00089942
1148	ARL2	3742.72998	-0.4840778	0.07007388	-6.9081054	4.91E-12
1151	ARL3	1336.96938	-0.1723363	0.078193	-2.2039861	0.02752532
1152	ARL4A	1496.74175	0.81352607	0.06968257	11.6747423	1.72E-31
1154	ARL4D	972.772952	0.83618882	0.13593061	6.15158604	7.67E-10
1156	ARL5B	802.344923	0.96006599	0.14969086	6.41365821	1.42E-10
1159	ARL6IP1	11356.571	-0.3106549	0.06222305	-4.992602	5.96E-07
1160	ARL6IP4	4419.05219	-0.1707064	0.0760599	-2.2443678	0.02480875
1161	ARL6IP5	5156.1324	-0.7275038	0.05483533	-13.267064	3.59E-40
1165	ARL9	2.03106574	4.48456447	1.72907887	2.59361475	0.00949728
1174	ARMC6	3612.76987	0.6125898	0.07259815	8.4380913	3.23E-17
1176	ARMC8	1427.201	0.24551903	0.06812595	3.60389858	0.00031348
1178	ARMCX1	1686.133	-0.6692013	0.07219953	-9.2687754	1.88E-20
1186	ARMT1	1749.81579	0.30372801	0.07364062	4.12446281	3.72E-05
1189	ARNTL	435.843344	0.72048215	0.11621863	6.19936908	5.67E-10
1193	ARPC1B	7137.66521	-0.3941412	0.06979367	-5.6472342	1.63E-08
1195	ARPC3	4833.39403	0.55224048	0.0702463	7.86148832	3.80E-15
1199	ARPC5L	3393.69194	0.65011947	0.08214948	7.91385982	2.50E-15
1200	ARPIN	953.072639	-0.9747288	0.07843612	-12.427039	1.86E-35
1204	ARRB1	2826.07054	-0.8353265	0.06209567	-13.45225	2.99E-41
1205	ARRB2	1495.65943	0.22146974	0.0687031	3.22357711	0.001266
1211	ARRDC4	275.145037	1.73211774	0.14828343	11.6811282	1.59E-31
1214	ARSB	726.354692	-0.5275522	0.08475185	-6.2246682	4.83E-10
1216	ARSE	255.727454	-0.7709488	0.13781371	-5.5941376	2.22E-08

1220	ARSI	494.162869	-0.5083696	0.10399991	-4.8881733	1.02E-06
1221	ARSJ	513.088923	-1.5071127	0.10780087	-13.980525	2.05E-44
1232	ASAH1	2907.80175	-1.7559221	0.06137967	-28.607551	5.41E-180
1235	ASAP1	6451.9403	0.38755471	0.09221051	4.20293445	2.63E-05
1243	ASB12	7.5664082	-1.7062743	0.81116377	-2.1034894	0.03542301
1244	ASB13	1341.37562	-0.4775311	0.0829426	-5.7573679	8.54E-09
1255	ASB6	2003.86908	0.47468956	0.06177402	7.68429181	1.54E-14
1256	ASB7	650.820119	0.37875517	0.10457894	3.62171563	0.00029266
1257	ASB8	634.674879	-0.3910532	0.0942525	-4.1489953	3.34E-05
1258	ASB9	152.361896	-1.5349897	0.18995164	-8.0809498	6.43E-16
1261	ASCC2	1223.07231	-0.1672622	0.07612776	-2.1971256	0.02801148
1262	ASCC3	1787.20653	0.62863292	0.08088908	7.77154296	7.75E-15
1269	ASF1B	2576.62319	-0.4445201	0.06728259	-6.6067628	3.93E-11
1270	ASGR1	63.145139	1.62231863	0.31175476	5.20382955	1.95E-07
1272	ASH1L	2307.24902	0.24155894	0.11148656	2.16670901	0.03025705
1274	ASH2L	1284.99958	-0.4960364	0.0829402	-5.9806513	2.22E-09
1275	ASIC1	515.295295	-1.8226164	0.10802121	-16.872765	7.14E-64
1284	ASMTL-AS1	266.666133	-0.295089	0.13957525	-2.1141926	0.03449882
1286	ASNS	4620.70234	2.41912394	0.05583163	43.3289173	0
1287	ASNSD1	2928.86593	-0.2974162	0.0646594	-4.5997375	4.23E-06
1292	ASPHD1	792.10943	0.3320379	0.11508223	2.88522313	0.00391136
1293	ASPHD2	219.938456	0.9354474	0.14048532	6.65868454	2.76E-11
1294	ASPM	3854.47749	-0.230251	0.09147453	-2.5171047	0.01183237
1299	ASS1	998.885668	0.31910333	0.09145005	3.48937281	0.00048416
1303	ASTN2	75.2788195	-0.7526403	0.24443421	-3.0791118	0.00207619
1306	ASXL1	10558.8594	0.1848803	0.05949088	3.10770821	0.00188544
1310	ATAD1	2458.22143	0.25812966	0.07406185	3.48532557	0.00049154
1313	ATAD3A	2114.22388	0.2135678	0.09003625	2.37202023	0.01769112
1323	ATF3	1475.62877	3.52512471	0.09629995	36.6056747	2.32E-293
1324	ATF4	6180.22819	0.60426598	0.06155642	9.81645694	9.56E-23
1329	ATF7IP	2523.52162	0.2677922	0.0809533	3.30798363	0.0009397
1331	ATG10	167.573861	-0.366157	0.16100418	-2.2742083	0.02295347
1332	ATG101	1818.99467	0.5990948	0.09632025	6.21982219	4.98E-10
1333	ATG12	1796.60953	0.23605254	0.08291969	2.84676099	0.00441665
1335	ATG14	1299.00357	0.36933449	0.07958472	4.64077149	3.47E-06
1337	ATG16L2	539.284624	-1.06115	0.113233	-9.3713846	7.16E-21
1339	ATG2B	1323.48918	0.35976565	0.09384246	3.83371915	0.00012622
1345	ATG5	2324.83696	0.29578281	0.08417993	3.51369766	0.00044192
1347	ATG9A	3716.48156	-0.5901129	0.05667767	-10.411735	2.19E-25
1349	ATHL1	437.752062	-0.4336907	0.11579574	-3.7453078	0.00018017
1350	ATIC	5087.58696	0.54920119	0.06046509	9.08294738	1.06E-19
1351	ATL1	417.416383	-0.4328034	0.11023514	-3.9261838	8.63E-05
1359	ATOH8	203.692525	-3.4014925	0.19823928	-17.158519	5.43E-66

1370	ATP13A1	2967.23304	-0.1571631	0.06553895	-2.3980102	0.01648441
1372	ATP13A3	10026.4657	0.7000568	0.08281912	8.45283985	2.84E-17
1377	ATP1A1	49986.0868	-0.7779714	0.0465256	-16.721362	9.16E-63
1390	ATP2B1	16010.2377	1.06121297	0.06067371	17.4904908	1.69E-68
1399	ATP5A1	21449.2605	-0.5132701	0.06522219	-7.8695631	3.56E-15
1401	ATP5C1	6087.57404	-0.2700734	0.05345811	-5.0520568	4.37E-07
1405	ATP5F1	4891.93608	-0.2155326	0.05914134	-3.6443641	0.00026805
1408	ATP5G3	7759.29192	-0.698686	0.08523241	-8.1974216	2.46E-16
1412	ATP5J2	2395.39291	-0.3097391	0.11139675	-2.7805037	0.00542746
1416	ATP5O	4433.00971	0.16701107	0.08145753	2.05028395	0.04033673
1419	ATP6AP1	4752.15018	-0.3259123	0.05265088	-6.1900636	6.01E-10
1423	ATP6V0A2	1566.7508	0.69754121	0.06622123	10.5334982	6.05E-26
1424	ATP6V0A4	892.894433	-0.3315426	0.08866726	-3.7391771	0.00018462
1425	ATP6V0B	4554.88613	0.50287617	0.08228278	6.11156062	9.87E-10
1428	ATP6V0D1	2549.03677	-0.2336082	0.08332443	-2.8035976	0.00505359
1430	ATP6V0E1	8421.28144	-0.2190324	0.07463695	-2.9346377	0.00333937
1431	ATP6V0E2	2283.08008	-0.8368209	0.09392356	-8.9095954	5.12E-19
1436	ATP6V1B2	4656.67158	0.43500644	0.07033881	6.18444372	6.23E-10
1437	ATP6V1C1	7111.99011	0.25301514	0.05103479	4.9576993	7.13E-07
1439	ATP6V1D	3217.00876	-0.1968809	0.06115477	-3.2193881	0.00128464
1452	ATP8B1	1140.10213	-0.8304506	0.08969957	-9.2581337	2.08E-20
1454	ATP8B3	244.758563	-1.100706	0.14665164	-7.5055828	6.12E-14
1457	ATP9A	719.657062	-0.3126482	0.09549744	-3.273891	0.00106078
1458	ATP9B	799.187397	-0.8868756	0.09075746	-9.7719305	1.49E-22
1459	ATPAF1	1361.23985	0.21759648	0.08748698	2.48718695	0.01287577
1460	ATPAF2	330.357638	-0.4232188	0.11947095	-3.5424411	0.00039644
1461	ATPIF1	2889.33973	0.3107222	0.11526032	2.69582974	0.00702135
1462	ATR	1327.79642	0.16846836	0.0788482	2.13661659	0.03262919
1463	ATRAID	1998.8212	-0.1317646	0.06691192	-1.9692251	0.04892725
1465	ATRN	2454.60891	-0.5865878	0.0939406	-6.2442418	4.26E-10
1466	ATRNL1	696.43141	-0.971278	0.09491031	-10.233641	1.40E-24
1468	ATXN1	1262.90616	0.23639078	0.08238988	2.86917263	0.00411547
1472	ATXN2L	6778.27586	0.92579523	0.08723614	10.6125198	2.61E-26
1473	ATXN3	819.099448	0.47701336	0.08720783	5.46984528	4.50E-08
1475	ATXN7	1601.58532	0.52577082	0.09591395	5.48169271	4.21E-08
1478	ATXN7L3	3743.64477	0.16627955	0.05390933	3.08442978	0.00203943
1479	ATXN7L3B	2000.16459	0.15430352	0.06346696	2.43124162	0.01504718
1481	AUH	524.615204	-0.2730297	0.09354545	-2.9186847	0.00351512
1482	AUNIP	264.369754	0.63321888	0.12804039	4.94546181	7.60E-07
1484	AURKA	5305.54437	-0.1313502	0.05165571	-2.5428008	0.01099679
1503	AXL	38637.1208	0.38013467	0.04799783	7.91982995	2.38E-15
1507	AZIN1	11276.7554	0.34810171	0.04897075	7.10835966	1.17E-12
1513	B3GALNT2	1974.2253	0.27599856	0.0721958	3.8229172	0.00013188

1516	B3GALT4	42.294574	-2.3244423	0.34820149	-6.6755666	2.46E-11
1517	B3GALT5	86.4771106	1.99777167	0.25934444	7.70315997	1.33E-14
1522	B3GAT3	1404.36874	-0.496583	0.09955141	-4.988207	6.09E-07
1523	B3GLCT	1248.43606	-0.860414	0.08147568	-10.560378	4.55E-26
1526	B3GNT4	57.3246901	-1.0758762	0.2674988	-4.021985	5.77E-05
1527	B3GNT5	2007.23214	0.32929559	0.07905908	4.16518362	3.11E-05
1532	B3GNTL1	479.982159	-0.2733612	0.10221097	-2.6744796	0.00748453
1539	B4GALT2	3008.2278	0.54580649	0.06519187	8.37230869	5.65E-17
1546	B4GAT1	1393.88789	-1.5496865	0.0881037	-17.589347	2.97E-69
1554	BACE1	2966.32691	-0.2251367	0.07371623	-3.0540989	0.00225738
1556	BACE2	3519.97628	-0.5532691	0.05467942	-10.118416	4.58E-24
1557	BACH1	3490.12295	0.73752046	0.09531462	7.73774759	1.01E-14
1561	BAG1	2814.87387	-0.9333504	0.09625565	-9.6965783	3.12E-22
1562	BAG2	3134.15988	0.6041662	0.05640584	10.7110568	9.03E-27
1564	BAG4	897.229337	0.2831574	0.08726159	3.24492606	0.00117481
1575	BAIAP2-AS1	381.573394	-1.0529305	0.1180083	-8.9225122	4.56E-19
1578	BAIAP3	328.30484	-0.811741	0.11614864	-6.9888119	2.77E-12
1579	BAK1	1316.59329	0.62727307	0.07565123	8.29164397	1.12E-16
1585	BANP	629.44881	0.51095053	0.09110137	5.60859327	2.04E-08
1586	BAP1	5171.50917	-0.281254	0.05252273	-5.3549018	8.56E-08
1596	BATF3	510.735267	0.55741507	0.10822351	5.15059136	2.60E-07
1598	BAZ1A	3131.59768	0.33880493	0.06203812	5.46123762	4.73E-08
1607	BBS10	979.374983	0.41278201	0.1117498	3.69380551	0.00022092
1608	BBS12	386.022582	0.39292195	0.12200618	3.22050851	0.00127963
1609	BBS2	1108.5092	-0.4696765	0.07403624	-6.3438727	2.24E-10
1610	BBS4	172.983533	-0.3368181	0.16006813	-2.1042173	0.0353595
1612	BBS7	1291.02438	0.38718512	0.07841222	4.93781588	7.90E-07
1613	BBS9	324.649623	-0.3447453	0.1176192	-2.9310291	0.00337841
1615	BCAM	1681.03399	-0.8213722	0.08193733	-10.024396	1.19E-23
1620	BCAR3	7016.72596	0.64742493	0.0554708	11.6714549	1.78E-31
1625	BCAS4	571.984429	-0.4114364	0.09514132	-4.324476	1.53E-05
1626	BCAT1	6244.38537	0.11517692	0.05525143	2.08459622	0.03710597
1627	BCAT2	1628.26487	-0.3753159	0.07349515	-5.1066753	3.28E-07
1631	BCHE	3.52612226	-3.2860362	1.44084466	-2.2806318	0.02257024
1633	BCKDHB	564.882272	-0.5366055	0.12214674	-4.3931215	1.12E-05
1638	BCL2	376.748157	-0.2824429	0.12341658	-2.2885328	0.02210651
1640	BCL2L1	20409.1227	0.19784539	0.05069906	3.90234865	9.53E-05
1642	BCL2L11	1082.3355	-0.3613106	0.09253994	-3.9043751	9.45E-05
1643	BCL2L12	1567.87936	-0.5273831	0.11095201	-4.7532539	2.00E-06
1650	BCL6	1396.68833	0.46047695	0.07872158	5.8494377	4.93E-09
1653	BCL7B	3253.00581	0.48779046	0.06521591	7.47962381	7.45E-14
1654	BCL7C	774.01601	-0.4760868	0.1084434	-4.390187	1.13E-05
1660	BCOR	1102.8245	0.52603775	0.0890434	5.90765536	3.47E-09

1663	BCR	1970.59041	-0.8457372	0.07951864	-10.63571	2.03E-26
1668	BDH1	1015.23485	-0.306295	0.08341225	-3.6720625	0.0002406
1669	BDH2	473.723327	-0.928708	0.11951979	-7.7703278	7.83E-15
1670	BDKRB1	64.9471373	0.57663369	0.26040538	2.21436933	0.02680338
1671	BDKRB2	90.7252234	1.01463497	0.21901656	4.63268603	3.61E-06
1685	BEND6	216.273233	0.79352025	0.14891763	5.32858482	9.90E-08
1686	BEND7	890.055431	0.26150179	0.08361753	3.12735624	0.00176386
1694	BEX2	468.194302	1.02516471	0.12933747	7.92627759	2.26E-15
1714	BICC1	1166.1738	-0.7334182	0.08020272	-9.1445548	5.99E-20
1717	BID	1563.30458	-0.4602476	0.069832	-6.5907829	4.38E-11
1724	BIRC3	8016.6591	0.82001624	0.06228849	13.1648119	1.40E-39
1725	BIRC5	4016.9818	-0.4720779	0.0792235	-5.9588114	2.54E-09
1726	BIRC6	2677.70198	0.17655625	0.08702181	2.02887353	0.04247117
1728	BIRC7	6.90554329	-2.8681925	0.91881218	-3.1216309	0.00179852
1744	BLOC1S3	580.880595	0.41867259	0.10112785	4.14003252	3.47E-05
1750	BLVRB	2463.5377	0.47452803	0.08333934	5.69392591	1.24E-08
1751	BLZF1	1198.49715	0.83528308	0.08693612	9.60800976	7.40E-22
1752	BMF	369.232072	-0.5219524	0.12270246	-4.2538053	2.10E-05
1753	BMI1	113.093492	0.53660312	0.25409152	2.11184979	0.03469934
1754	BMP1	2268.98738	-0.5404656	0.05986617	-9.0278971	1.75E-19
1762	BMP6	6.59128921	2.53141424	1.11773089	2.26477972	0.0235262
1768	BMPR1A	540.362405	-0.7455581	0.09978145	-7.4719106	7.90E-14
1771	BMPR2	2762.23721	-0.5701538	0.10212702	-5.5827913	2.37E-08
1783	BNIP1	729.104415	0.78486851	0.09024108	8.69746357	3.39E-18
1785	BNIP3	6886.50403	-1.3260015	0.05341046	-24.826628	4.63E-136
1789	BOD1	4824.95764	1.13264967	0.05787898	19.569276	2.83E-85
1794	BOLA1	119.548158	-1.279317	0.21529589	-5.9421337	2.81E-09
1798	BOLA3-AS1	38.1701665	-1.0257782	0.34006407	-3.0164264	0.00255773
1800	BOP1	5059.16868	0.3453277	0.08882307	3.88781533	0.00010115
1804	BPHL	898.851286	-0.3527237	0.08042261	-4.385878	1.16E-05
1821	BRAF	356.611234	0.57688689	0.1265802	4.55748134	5.18E-06
1822	BRAP	1503.31301	0.59124511	0.07893227	7.49053741	6.86E-14
1823	BRAT1	2158.48184	-0.6449778	0.09378763	-6.8770024	6.11E-12
1830	BRD2	10969.1518	0.54592435	0.04891657	11.1603166	6.38E-29
1832	BRD4	3882.40627	0.35437571	0.08162106	4.3417193	1.41E-05
1835	BRD8	3258.72295	-0.3691807	0.05803935	-6.3608687	2.01E-10
1843	BRF2	362.870889	0.61741752	0.11806349	5.22953827	1.70E-07
1850	BRIP1	1106.08302	-0.5472059	0.11048898	-4.9525838	7.32E-07
1856	BRPF1	1287.85959	0.18554002	0.07732552	2.39946703	0.01641896
1857	BRPF3	5628.5687	0.60864248	0.09225511	6.59738516	4.18E-11
1861	BRWD1	1729.71038	0.21980069	0.06875016	3.19709354	0.0013882
1867	BSG	22226.1673	-0.1551092	0.05185942	-2.9909545	0.00278107
1874	BST2	4513.91844	-1.1585601	0.10497603	-11.036424	2.55E-28

1878	BTBD10	2274.7811	0.41524869	0.06505271	6.3832654	1.73E-10
1884	BTBD2	2402.76869	-0.7278471	0.06018999	-12.092495	1.16E-33
1890	BTC	47.1130484	-0.9537728	0.33377423	-2.8575386	0.00426941
1891	BTD	836.106386	-0.8342353	0.09295366	-8.9747437	2.84E-19
1893	BTF3L4	1459.8234	0.93812703	0.06981455	13.4374141	3.65E-41
1896	BTG2	61.529797	1.85913608	0.29018124	6.40680997	1.49E-10
1897	BTG3	1600.29023	0.97193618	0.07924457	12.2650188	1.40E-34
1906	BTN3A2	956.638968	-0.8848635	0.09507313	-9.3071884	1.31E-20
1915	BUB1B	2728.96672	-0.2564734	0.09026519	-2.8413321	0.00449255
1917	BUD13	1244.03794	-0.2429302	0.07860507	-3.0905157	0.00199809
1921	BYSL	3172.494	0.77645316	0.06227817	12.4675011	1.12E-35
1922	BZRAP1	612.926214	-1.3823926	0.09631952	-14.352154	1.03E-46
1926	C10orf10	225.021201	-1.4480655	0.1560255	-9.2809542	1.68E-20
1944	C10orf54	1856.22235	-0.9877811	0.07097621	-13.917072	4.99E-44
1952	C10orf88	713.639205	0.37744717	0.08477773	4.45219724	8.50E-06
1968	C11orf49	973.475679	-0.4633705	0.08445687	-5.4864751	4.10E-08
1972	C11orf57	2087.93653	0.59295747	0.06920075	8.56865635	1.05E-17
1973	C11orf58	7569.91824	0.23919025	0.0495305	4.82915065	1.37E-06
1978	C11orf71	238.682155	-0.4712773	0.13144832	-3.5852668	0.00033673
1980	C11orf73	775.872145	0.21573992	0.1078105	2.00110307	0.04538128
1982	C11orf80	973.63418	-0.2538633	0.08329142	-3.0478929	0.00230452
1985	C11orf86	98.8221136	-2.7654217	0.26715616	-10.351331	4.13E-25
1991	C11orf96	39.6153435	0.72907487	0.37057481	1.96741613	0.04913526
1995	C12orf29	1816.12282	0.70943528	0.074601	9.50972859	1.91E-21
1996	C12orf4	949.651443	0.63832482	0.07976984	8.00208258	1.22E-15
2001	C12orf49	3411.08154	0.54689703	0.06076901	8.99960387	2.27E-19
2008	C12orf66	160.712692	1.35954634	0.18684635	7.27628003	3.43E-13
2012	C12orf75	5197.87558	0.27268331	0.05715648	4.77082065	1.83E-06
2019	C14orf119	3438.5633	0.33590952	0.05737501	5.85463103	4.78E-09
2021	C14orf142	686.624375	0.38985768	0.08999159	4.33215702	1.48E-05
2022	C14orf159	582.469621	-0.4659224	0.09306344	-5.0065025	5.54E-07
2023	C14orf166	5614.02512	0.13851285	0.0550965	2.51400475	0.01193688
2032	C14orf79	299.545361	0.96559917	0.13731207	7.03215067	2.03E-12
2034	C14orf93	224.453847	-0.4454653	0.13749368	-3.2398964	0.00119573
2041	C15orf41	301.835388	0.354841	0.12881265	2.75470624	0.00587449
2043	C15orf48	8233.41573	-0.4718884	0.10802218	-4.3684393	1.25E-05
2044	C15orf52	4126.42013	-0.6714592	0.06562381	-10.231943	1.43E-24
2058	C16orf52	436.565707	0.42959365	0.11615799	3.69835636	0.000217
2061	C16orf59	374.935003	-0.6469713	0.1163045	-5.5627367	2.66E-08
2065	C16orf72	1745.32091	0.4360763	0.06969242	6.25715578	3.92E-10
2076	C16orf95	133.122571	-0.7525785	0.19525582	-3.8543205	0.00011605
2083	C17orf107	89.5262632	1.16919008	0.2214227	5.28035323	1.29E-07
2088	C17orf51	1821.42889	1.05848643	0.07460865	14.1871802	1.10E-45

2090	C17orf58	246.978932	-0.27702	0.1398225	-1.9812263	0.0475659
2092	C17orf62	1475.49003	-0.5501259	0.10552537	-5.21321	1.86E-07
2099	C17orf80	1149.94234	0.51876824	0.09396551	5.52083661	3.37E-08
2103	C17orf96	914.037773	0.63725624	0.0944766	6.74512242	1.53E-11
2115	C18orf8	2416.30214	0.55052717	0.06364851	8.64949019	5.17E-18
2124	C19orf44	373.910134	0.90392099	0.1323535	6.82959627	8.52E-12
2127	C19orf48	3108.69622	0.63200856	0.06588658	9.59237215	8.61E-22
2130	C19orf54	1188.95495	0.93625894	0.07591653	12.332741	6.04E-35
2132	C19orf60	871.982767	-0.5978963	0.15823109	-3.7786268	0.0001577
2133	C19orf66	822.654253	-0.7366129	0.09331924	-7.8934731	2.94E-15
2148	C1QBP	8372.11644	0.29847597	0.05710378	5.22690383	1.72E-07
2150	C1QL1	96.9265991	-2.015394	0.2647617	-7.6121054	2.70E-14
2161	C1QTNF6	251.172825	-0.3691576	0.13223116	-2.7917594	0.00524223
2167	C1R	1273.79668	-2.6729319	0.0848751	-31.492534	1.10E-217
2168	C1RL	216.340911	-0.3980259	0.1436666	-2.7704831	0.00559732
2170	C1S	561.73356	-2.102208	0.12111795	-17.356701	1.76E-67
2174	C1orf106	104.943903	-0.5749214	0.2117549	-2.7150325	0.00662693
2175	C1orf109	1069.22833	1.22447808	0.08651882	14.1527368	1.80E-45
2176	C1orf110	324.876712	0.59478609	0.15200242	3.91300414	9.12E-05
2202	C1orf198	1067.62649	0.56514948	0.08461992	6.67868155	2.41E-11
2204	C1orf21	1260.13849	-1.0094561	0.08209626	-12.296006	9.52E-35
2206	C1orf216	1097.59131	0.23440648	0.07537338	3.10993733	0.00187127
2208	C1orf226	524.911793	-0.472268	0.09798139	-4.8199767	1.44E-06
2215	C1orf43	9766.87729	0.67226166	0.05448851	12.337677	5.68E-35
2216	C1orf50	224.017632	0.93942903	0.14621906	6.4248054	1.32E-10
2228	C2	1881.77085	-0.7378143	0.06971615	-10.583119	3.57E-26
2249	C21orf58	270.2181	-0.7135721	0.13483348	-5.2922468	1.21E-07
2250	C21orf59	1352.11124	0.33483879	0.09655985	3.4676814	0.00052497
2253	C21orf91	388.58569	1.22537935	0.127053	9.64463121	5.18E-22
2265	C2CD2L	1660.0993	0.23366601	0.06405856	3.64769361	0.0002646
2281	C2orf49	1171.16947	0.28954672	0.08480853	3.41412271	0.00063988
2287	C2orf68	1186.76345	-0.3898254	0.07770651	-5.0166373	5.26E-07
2298	C2orf82	40.1295251	-0.9092117	0.33200188	-2.7385738	0.00617063
2302	C3	11139.041	-0.956947	0.06781176	-14.111814	3.21E-45
2303	C3AR1	44.9410704	-0.6634678	0.30095345	-2.2045528	0.02748549
2306	C3orf17	1127.92955	0.44598025	0.07946464	5.61231093	2.00E-08
2307	C3orf18	616.583933	-0.2020674	0.09017398	-2.2408621	0.02503501
2308	C3orf20	9.90749985	2.36985956	0.84449396	2.80624809	0.00501221
2334	C4orf26	221.019116	-0.3854615	0.1459234	-2.6415329	0.00825318
2335	C4orf27	778.136204	-0.571163	0.09169649	-6.2288433	4.70E-10
2337	C4orf3	4394.02554	-1.5035054	0.09973108	-15.075596	2.34E-51
2338	C4orf32	1057.53873	0.72463225	0.07714889	9.39264669	5.85E-21
2339	C4orf33	296.864176	-0.2947364	0.13993728	-2.1062038	0.03518665

2346	C5	385.698778	-0.8353708	0.11920114	-7.0080771	2.42E-12
2349	C5orf15	3780.42916	-0.4862449	0.07391688	-6.5782652	4.76E-11
2351	C5orf22	1255.38443	-0.253525	0.10401284	-2.4374398	0.01479168
2352	C5orf24	2622.4273	-0.3511331	0.07094334	-4.9494868	7.44E-07
2355	C5orf34	647.084475	0.90008511	0.09465422	9.50919199	1.92E-21
2358	C5orf45	282.246478	-0.5659529	0.14425631	-3.9232452	8.74E-05
2376	C6orf106	7395.78711	0.44328774	0.05178187	8.56067446	1.12E-17
2379	C6orf132	2847.60637	0.1370645	0.06750318	2.0304896	0.0423068
2388	C6orf223	32.386266	2.01454119	0.38138176	5.28221692	1.28E-07
2393	C6orf48	8749.9834	1.40400481	0.07524754	18.6584813	1.08E-77
2394	C6orf52	591.897574	0.26150217	0.11605273	2.25330481	0.02423994
2401	C7orf13	746.355157	1.0605918	0.10505273	10.0958042	5.77E-24
2408	C7orf49	2751.00861	0.2352303	0.07075331	3.32465447	0.00088528
2421	C7orf73	2379.0948	-0.4514288	0.06693358	-6.7444295	1.54E-11
2429	C8orf33	2690.4339	0.4546736	0.057708	7.8788664	3.30E-15
2437	C8orf46	398.771003	1.74096688	0.11378014	15.3011486	7.51E-53
2441	C8orf59	1148.23986	-0.4506173	0.08129767	-5.5428065	2.98E-08
2444	C8orf82	1198.01984	-0.6570496	0.08554217	-7.6810025	1.58E-14
2463	C9orf16	2216.42329	-0.6115142	0.12963574	-4.7171725	2.39E-06
2471	C9orf3	2054.75814	-0.5396943	0.06523215	-8.27344	1.30E-16
2472	C9orf40	1754.16471	0.20481478	0.08396474	2.43929525	0.01471594
2474	C9orf43	41.4572563	2.03119696	0.34683742	5.85633747	4.73E-09
2481	C9orf69	3491.48713	-0.5197583	0.0730176	-7.1182611	1.09E-12
2484	C9orf84	521.519859	3.45864853	0.12871469	26.8706582	4.84E-159
2485	C9orf85	300.171384	0.50827934	0.12871464	3.94888524	7.85E-05
2486	C9orf89	1848.58412	-0.7332752	0.07731131	-9.4847083	2.43E-21
2488	C9orf91	938.901533	1.17012618	0.08375988	13.9700076	2.38E-44
2492	CA11	169.99389	-0.4886807	0.16885936	-2.8940104	0.00380356
2493	CA12	452.21713	-1.3730988	0.1196844	-11.472664	1.81E-30
2501	CA5B	420.767078	0.24258745	0.11402171	2.12755487	0.03337401
2502	CA5BP1	654.87435	0.62346443	0.0869796	7.16793838	7.61E-13
2506	CA9	178.350288	-1.0519477	0.16833475	-6.2491416	4.13E-10
2507	CAAP1	1256.86169	-0.4287551	0.08050428	-5.3258668	1.00E-07
2510	CABIN1	1482.53189	-0.6690897	0.06979595	-9.5863688	9.12E-22
2512	CABLES2	1215.5235	-0.734949	0.08861649	-8.2935924	1.10E-16
2520	CACFD1	831.12199	-0.5886995	0.11221509	-5.2461706	1.55E-07
2538	CACNA2D1	278.139125	0.71921504	0.13574755	5.29818046	1.17E-07
2543	CACNB1	651.884776	1.17343997	0.09345536	12.5561541	3.68E-36
2545	CACNB3	147.783106	-0.3423148	0.16506496	-2.073819	0.03809613
2552	CACNG6	20.2198219	-2.0429446	0.53961048	-3.7859616	0.00015312
2553	CACNG7	30.1529218	-2.4624787	0.41799186	-5.8912121	3.83E-09
2554	CACNG8	13.4820535	-1.4186742	0.56501779	-2.5108487	0.01204413
2556	CACTIN-AS1	61.6505218	-0.7024913	0.28750179	-2.4434328	0.01454828

2559	CAD	5115.49642	0.40922387	0.05265551	7.77172028	7.74E-15
2565	CADM4	251.608634	-1.1973972	0.13626195	-8.7874656	1.53E-18
2567	CADPS2	220.156343	-1.0798585	0.14338815	-7.5310165	5.03E-14
2570	CALB1	25.2723116	1.9816892	0.42630794	4.64849236	3.34E-06
2575	CALCOCO2	4378.42519	0.5117473	0.0543327	9.418771	4.56E-21
2576	CALCR	30.0552957	-1.2552338	0.38503771	-3.2600283	0.00111401
2578	CALD1	10425.9347	-0.3564496	0.06451704	-5.5248903	3.30E-08
2580	CALHM2	907.964895	-0.3759184	0.09407331	-3.9960155	6.44E-05
2581	CALHM3	157.016139	-0.5954964	0.16431242	-3.6241714	0.00028989
2582	CALM1	10238.6113	-0.5524801	0.05467386	-10.105014	5.25E-24
2584	CALM3	14648.6475	-0.3489386	0.04656172	-7.4941093	6.68E-14
2591	CALR	48354.0972	-0.3762451	0.04986833	-7.544771	4.53E-14
2593	CALU	23327.4415	-0.5063656	0.07666664	-6.6047719	3.98E-11
2596	CAMK1D	680.704581	-0.618704	0.10061994	-6.1489201	7.80E-10
2600	CAMK2D	1300.85095	-0.2501531	0.09058326	-2.7615822	0.0057522
2601	CAMK2G	1712.82538	-0.8652544	0.06866357	-12.601362	2.08E-36
2602	CAMK2N1	973.726155	0.8667494	0.0864497	10.0260543	1.17E-23
2603	CAMK2N2	761.450766	-0.3925679	0.09940045	-3.9493578	7.84E-05
2605	CAMKK1	505.708295	0.96576827	0.10402519	9.2839851	1.63E-20
2609	CAMLG	1884.94339	0.19644967	0.068133	2.88332642	0.00393499
2611	CAMSAP1	2733.1077	0.38837409	0.06165482	6.29916868	2.99E-10
2612	CAMSAP2	2946.09885	0.71887111	0.10977208	6.54876124	5.80E-11
2619	CANT1	4608.12762	-0.4001909	0.05598881	-7.1476947	8.82E-13
2621	CAP1	13665.5892	0.41403696	0.06365003	6.50489851	7.77E-11
2624	CAPN1	9417.47535	-0.5830578	0.051762	-11.264206	1.97E-29
2625	CAPN10	544.23057	-0.4558809	0.10610127	-4.2966579	1.73E-05
2634	CAPN5	827.593649	-1.4478779	0.10631465	-13.6188	3.10E-42
2636	CAPN7	1992.34323	0.23627241	0.07134222	3.31181759	0.00092692
2637	CAPN8	14.1995907	-3.7448005	0.74082853	-5.0548816	4.31E-07
2639	CAPNS1	10333.2636	-0.3638705	0.05493092	-6.6241466	3.49E-11
2641	CAPRIN1	12615.3174	0.36181497	0.06736643	5.37084964	7.84E-08
2643	CAPS	96.835759	-1.0908181	0.21983222	-4.9620486	6.98E-07
2650	CARD10	1485.26509	-0.6627875	0.07663738	-8.6483573	5.22E-18
2651	CARD11	1037.21709	-0.8917889	0.08693793	-10.257765	1.09E-24
2652	CARD14	17.1312086	-1.9052477	0.53514218	-3.5602645	0.00037048
2657	CARD8	661.67758	-0.373605	0.08755215	-4.2672285	1.98E-05
2659	CARD9	42.2967268	1.06037428	0.30482365	3.47864839	0.00050395
2661	CARHSP1	2924.72727	-0.7517009	0.05989726	-12.549838	3.98E-36
2662	CARKD	1431.97073	0.37050232	0.09608373	3.85603577	0.00011524
2663	CARM1	4195.41554	0.23197701	0.05269209	4.40250124	1.07E-05
2666	CARS	4756.00115	0.7808243	0.05267654	14.8229986	1.04E-49
2682	CASC4	3131.18135	-0.5669833	0.07089049	-7.9980169	1.26E-15
2685	CASC8	283.809258	-0.2694144	0.13064334	-2.0622127	0.03918749

2692	CASP10	336.588572	-0.8709618	0.12751432	-6.8303057	8.47E-12
2698	CASP3	1521.17063	0.32268829	0.0711435	4.53573824	5.74E-06
2699	CASP4	1158.2747	0.2612585	0.0769428	3.39548986	0.00068506
2701	CASP6	742.135138	-0.9496544	0.08938893	-10.623848	2.31E-26
2712	CAT	1422.95042	-1.1846863	0.09009113	-13.149866	1.70E-39
2724	CAV1	31146.9766	-0.6437291	0.05994922	-10.737907	6.76E-27
2727	CBARP	165.257557	1.35616317	0.17003366	7.9758512	1.51E-15
2732	CBLB	635.918808	0.21721055	0.09932889	2.18678131	0.02875849
2734	CBLL1	1576.8011	0.54503778	0.09371408	5.81596498	6.03E-09
2742	CBR4	647.031637	-0.4668331	0.08939395	-5.222201	1.77E-07
2744	CBWD1	341.015519	0.30919768	0.11344381	2.72555796	0.00641929
2745	CBWD2	244.690436	0.36132691	0.14180815	2.54799829	0.0108343
2747	CBWD5	155.160437	0.79544305	0.18672535	4.25996275	2.04E-05
2749	CBX1	4437.15222	0.26473575	0.06420298	4.1234186	3.73E-05
2753	CBX4	973.707475	0.40386982	0.07793451	5.18216909	2.19E-07
2754	CBX5	2758.18858	-0.8257879	0.07599294	-10.86664	1.66E-27
2756	CBX7	555.806667	-0.6171699	0.09539094	-6.4699006	9.81E-11
2760	CC2D1A	1575.46193	-0.4965651	0.07406106	-6.7048064	2.02E-11
2761	CC2D1B	2847.67083	0.2175192	0.06269419	3.46952704	0.00052138
2762	CC2D2A	689.070748	-0.4015821	0.09289579	-4.3229311	1.54E-05
2764	CCAR1	5356.05396	0.32496095	0.05423781	5.99140992	2.08E-09
2768	CCBE1	919.893252	-0.2960299	0.08798481	-3.3645564	0.00076667
2769	CCBL1	660.621617	-0.372581	0.09476064	-3.9318118	8.43E-05
2770	CCBL2	1745.84475	-0.3582868	0.06327765	-5.6621382	1.49E-08
2772	CCDC102A	614.629121	-0.6541283	0.13242309	-4.939685	7.82E-07
2776	CCDC106	611.251281	-0.9209819	0.10621106	-8.6712429	4.27E-18
2777	CCDC107	818.776591	-0.6864956	0.09792264	-7.0105911	2.37E-12
2779	CCDC109B	1090.53024	-0.8747724	0.07793599	-11.224242	3.10E-29
2781	CCDC112	738.108499	0.96220996	0.08646168	11.1287444	9.09E-29
2783	CCDC114	49.951228	-0.6050465	0.2948619	-2.0519655	0.04017302
2784	CCDC115	854.077917	-0.3296907	0.08178173	-4.0313487	5.55E-05
2788	CCDC120	697.121367	-0.5298029	0.0897116	-5.9056234	3.51E-09
2789	CCDC121	40.7989726	-0.9783506	0.37139114	-2.6342864	0.00843143
2790	CCDC122	276.342587	1.42745597	0.13826748	10.3238737	5.50E-25
2792	CCDC125	447.862425	-0.2078292	0.10228643	-2.0318359	0.04217027
2799	CCDC134	81.5149407	-0.8785924	0.21971016	-3.9988703	6.36E-05
2801	CCDC137	1900.2169	0.33132052	0.08085547	4.09768834	4.17E-05
2812	CCDC146	246.043363	-0.8992866	0.13879506	-6.4792408	9.22E-11
2816	CCDC15	869.329374	1.18831739	0.1095858	10.8437168	2.14E-27
2819	CCDC152	16.5728912	2.06353544	0.57571651	3.58429086	0.000338
2821	CCDC154	59.8616251	0.75044015	0.25782275	2.91068244	0.0036064
2825	CCDC159	63.4506179	-0.8996833	0.2481401	-3.6257071	0.00028817
2830	CCDC167	714.805704	-0.6799359	0.12734851	-5.339174	9.34E-08

2832	CCDC169	19.7756296	1.02515994	0.44548109	2.3012423	0.02137794
2834	CCDC17	76.7469399	0.49866318	0.24875686	2.00462079	0.0450036
2839	CCDC174	992.02085	1.21720476	0.08070248	15.0826193	2.11E-51
2861	CCDC3	979.55375	-1.2681415	0.08179273	-15.50433	3.24E-54
2862	CCDC30	10.7074925	1.90169181	0.65906004	2.88546062	0.00390841
2864	CCDC34	887.258219	-0.1834527	0.08996173	-2.0392309	0.04142699
2873	CCDC43	1399.30368	0.18562373	0.06710171	2.7663041	0.00566956
2875	CCDC50	8172.56922	0.62183821	0.0551502	11.2753572	1.74E-29
2876	CCDC51	898.561414	0.69737558	0.09518551	7.32648915	2.36E-13
2884	CCDC61	294.766453	-0.4050263	0.16216321	-2.4976459	0.0125021
2885	CCDC62	30.7306823	1.30360429	0.38890661	3.35197255	0.00080238
2893	CCDC69	4770.4621	-0.3562488	0.06751489	-5.2765966	1.32E-07
2897	CCDC71L	942.024789	-0.4229461	0.09397691	-4.5005324	6.78E-06
2901	CCDC77	462.49926	-0.3449686	0.09944974	-3.4687738	0.00052284
2904	CCDC8	505.001101	-0.4816351	0.1029652	-4.6776496	2.90E-06
2905	CCDC80	3573.83267	-0.5114875	0.05555721	-9.2065006	3.37E-20
2913	CCDC86	3015.59094	0.32883638	0.05635895	5.83467925	5.39E-09
2916	CCDC88B	303.021584	-1.4608331	0.16634028	-8.7821967	1.60E-18
2917	CCDC88C	1263.11038	0.53266139	0.08107444	6.57002887	5.03E-11
2920	CCDC90B	1831.79801	0.65205124	0.06993927	9.32310628	1.13E-20
2923	CCDC93	2569.66586	0.20895977	0.07234196	2.88850009	0.00387084
2924	CCDC94	732.681195	-0.2423308	0.09931911	-2.4399208	0.01469048
2926	CCDC97	902.637213	0.31809895	0.08570318	3.71163518	0.00020592
2929	CCHCR1	1672.01777	-0.4171521	0.07892065	-5.2857154	1.25E-07
2945	CCL2	3237.42895	-5.890971	0.106365	-55.384489	0
2946	CCL20	70.9073831	0.57389585	0.24044236	2.38683335	0.01699419
2954	CCL28	411.948684	-1.0998851	0.11328454	-9.7090481	2.76E-22
2967	CCNA2	4381.87133	-0.6195025	0.06872833	-9.0137854	1.99E-19
2968	CCNB1	8303.40141	-0.2647825	0.06738654	-3.9293083	8.52E-05
2969	CCNB1IP1	1354.42662	0.91990439	0.07913949	11.6238358	3.12E-31
2970	CCNB2	2026.5647	-0.6272083	0.06821554	-9.1945073	3.77E-20
2974	CCND2	23.8748036	2.3374323	0.46390624	5.03858775	4.69E-07
2978	CCNE1	731.694745	0.44520441	0.09031405	4.92951439	8.24E-07
2980	CCNF	1193.3457	-0.4647679	0.07154293	-6.4963496	8.23E-11
2982	CCNG2	1738.72193	-0.7385305	0.06715296	-10.997736	3.92E-28
2983	CCNH	2302.89405	0.75541464	0.06623703	11.4047173	3.96E-30
2984	CCNI	5814.61005	-0.170428	0.08674403	-1.9647228	0.04944633
2988	CCNK	2431.8399	0.38492104	0.07053327	5.45729714	4.83E-08
2992	CCNT1	1329.59023	0.26336432	0.07542991	3.49151042	0.0004803
2993	CCNT2	1625.79668	0.60734896	0.0894418	6.79043735	1.12E-11
3000	CCR1	18.4717282	-2.3514969	0.51583649	-4.5586091	5.15E-06
3010	CCRL2	2345.53161	-0.2506542	0.06654521	-3.7666755	0.00016544
3011	CCS	1158.55634	-0.7074486	0.09701374	-7.292252	3.05E-13

3015	CCT2	9457.25891	0.16045615	0.06200038	2.58798654	0.00965388
3017	CCT4	10419.5079	0.40242319	0.0638113	6.30645623	2.85E-10
3019	CCT6A	12715.8386	0.18379044	0.05540639	3.31713446	0.00090946
3021	CCT6P1	159.834887	1.32810666	0.17298651	7.67751599	1.62E-14
3023	CCT7	15143.7242	0.39718746	0.07064101	5.6226184	1.88E-08
3024	CCT8	11569.4621	0.50614228	0.08338327	6.07007021	1.28E-09
3037	CD177	183.103205	1.46864551	0.1555557	9.44128404	3.68E-21
3038	CD180	17.0761171	-3.7847827	0.69763761	-5.4251415	5.79E-08
3059	CD274	1271.81804	1.55541138	0.09376287	16.5887782	8.40E-62
3060	CD276	2959.9627	-0.1661011	0.05766044	-2.8806778	0.00396821
3062	CD2AP	6708.99521	0.56511961	0.06184133	9.13821939	6.35E-20
3074	CD34	128.372541	-1.9710061	0.19848338	-9.9303333	3.07E-23
3080	CD3EAP	1426.20539	0.84675074	0.07613427	11.1218075	9.83E-29
3086	CD46	7537.78192	-1.1570537	0.06796886	-17.023291	5.52E-65
3087	CD47	6283.30152	-0.5846564	0.05558099	-10.518999	7.06E-26
3092	CD55	11021.5555	0.25403813	0.04756239	5.34115601	9.24E-08
3093	CD58	1726.33072	-0.6848752	0.07076995	-9.6774848	3.76E-22
3094	CD59	19285.4446	-0.1925532	0.046208	-4.1670973	3.09E-05
3103	CD74	18116.3299	-0.6317409	0.04902724	-12.885508	5.43E-38
3107	CD81	9870.38298	-0.4108835	0.08253035	-4.9785746	6.41E-07
3115	CD9	7507.70847	-0.456996	0.06138785	-7.444404	9.74E-14
3118	CD99	13240.0748	-0.5382867	0.09479669	-5.6783277	1.36E-08
3119	CD99L2	3200.22476	-0.7685019	0.05756577	-13.349981	1.18E-40
3121	CDA	3830.0993	0.5890088	0.06086317	9.67758943	3.75E-22
3123	CDAN1	545.65586	-0.845312	0.10502855	-8.0484027	8.39E-16
3124	CDC123	4520.9686	0.12411985	0.06003619	2.06741734	0.03869485
3125	CDC14A	335.548451	0.55990137	0.14009361	3.99662329	6.43E-05
3129	CDC20	5035.71604	0.31183295	0.06625955	4.70623401	2.52E-06
3132	CDC25A	1346.33689	0.42548935	0.07527188	5.65269992	1.58E-08
3133	CDC25B	6781.2937	-0.4368973	0.06582769	-6.6369839	3.20E-11
3134	CDC25C	754.341761	-0.2066543	0.08961892	-2.3059226	0.02111496
3137	CDC34	4637.94662	0.60326415	0.08761231	6.8856096	5.75E-12
3141	CDC40	580.928643	-0.3020369	0.11095369	-2.7221891	0.0064851
3145	CDC42BPG	292.571909	-1.891587	0.14643364	-12.917708	3.58E-38
3146	CDC42EP1	4885.74611	0.52240171	0.06410149	8.14960316	3.65E-16
3147	CDC42EP2	4885.9215	0.83938854	0.07055772	11.8964802	1.23E-32
3148	CDC42EP3	10309.7183	0.90342244	0.07094294	12.7344937	3.80E-37
3154	CDC45	1140.56065	-0.4229084	0.07828707	-5.4020214	6.59E-08
3155	CDC5L	3283.35267	0.18516565	0.05961105	3.10623054	0.00189489
3156	CDC6	3625.20999	0.13639407	0.06380269	2.1377481	0.03253719
3159	CDCA2	1109.75883	-0.6540505	0.084477	-7.7423506	9.76E-15
3160	CDCA3	1089.80311	-0.454919	0.0752587	-6.0447365	1.50E-09
3161	CDCA4	2246.22036	0.17664734	0.06892382	2.56293584	0.01037912

3163	CDCA7	925.088536	-0.9741967	0.08071309	-12.069873	1.52E-33
3164	CDCA7L	3819.44368	-0.6408556	0.05501426	-11.648901	2.32E-31
3166	CDCP1	23838.8862	0.70230936	0.08045492	8.72922865	2.56E-18
3168	CDH1	244.881257	-3.4551079	0.19209874	-17.986104	2.50E-72
3178	CDH2	71.2867382	1.19026804	0.24996202	4.76179563	1.92E-06
3182	CDH24	821.463616	-0.547459	0.08413938	-6.5065733	7.69E-11
3185	CDH4	1071.43396	0.21678136	0.07448504	2.91040125	0.00360965
3196	CDIP1	1283.2794	0.63699174	0.07647732	8.32915879	8.14E-17
3199	CDK1	4960.95176	-0.2025148	0.06064824	-3.3391703	0.00084029
3200	CDK10	1562.34734	-0.3013412	0.07074346	-4.2596337	2.05E-05
3206	CDK15	94.6108678	0.68166156	0.21510524	3.16896769	0.00152981
3208	CDK17	2112.21099	0.58043515	0.07428627	7.81349143	5.56E-15
3210	CDK19	739.69429	-0.5534722	0.10096212	-5.4819788	4.21E-08
3212	CDK20	181.882529	0.96945512	0.15836081	6.12181219	9.25E-10
3214	CDK2AP2	1848.42963	-0.3506018	0.07815203	-4.4861506	7.25E-06
3216	CDK4	7830.32723	0.58146143	0.07109531	8.17861821	2.87E-16
3217	CDK5	1090.62893	-0.7632553	0.12590986	-6.0619187	1.35E-09
3218	CDK5R1	677.218902	0.78725935	0.09406169	8.36960649	5.78E-17
3220	CDK5RAP1	1499.41468	-0.2408604	0.07716869	-3.1212196	0.00180104
3223	CDK6	3012.40384	1.62832724	0.08447236	19.2764508	8.47E-83
3224	CDK7	2136.23575	0.49817654	0.07221787	6.89824494	5.26E-12
3225	CDK8	484.97805	0.42389148	0.11150912	3.80140649	0.00014388
3228	CDKL1	238.125631	1.86610789	0.14772585	12.6322367	1.40E-36
3230	CDKL3	140.612135	0.75330018	0.1967924	3.82789269	0.00012925
3233	CDKN1A	3267.71883	2.03286759	0.05994956	33.9096316	4.80E-252
3235	CDKN1C	631.271897	-0.9433709	0.09889535	-9.5390831	1.44E-21
3238	CDKN2AIP	1261.35299	0.66582904	0.07384789	9.01622271	1.95E-19
3242	CDKN2C	1080.89712	-1.2477264	0.07901595	-15.790818	3.60E-56
3247	CDON	1078.44611	-2.1378244	0.11271835	-18.966072	3.25E-80
3251	CDR2L	1912.08036	0.56581837	0.06418768	8.81506169	1.20E-18
3261	CDS2	2447.84314	-0.4887195	0.06288488	-7.771653	7.75E-15
3264	CDV3	9124.16457	0.64046194	0.05130653	12.4830481	9.24E-36
3272	CDYL	2875.90755	0.36673779	0.05835369	6.28474005	3.28E-10
3274	CEACAM1	52.9600577	-1.2914745	0.30029659	-4.3006632	1.70E-05
3288	CEBPA-AS1	2.78266616	3.9001283	1.57172059	2.4814387	0.01308532
3289	CEBPB	6667.74743	0.82516764	0.08533672	9.66954972	4.06E-22
3291	CEBPD	1647.52245	-1.1664744	0.09061534	-12.872813	6.40E-38
3293	CEBPG	3956.56561	2.1064257	0.06128254	34.3723602	6.53E-259
3294	CEBPZ	3585.71977	0.65550735	0.06265961	10.4614011	1.30E-25
3301	CECR6	60.1601291	-1.1082429	0.26133438	-4.2407083	2.23E-05
3309	CELF1	5564.28073	0.4086705	0.05352963	7.6344725	2.27E-14
3314	CELF4	138.703416	1.08537901	0.18016215	6.02445621	1.70E-09
3318	CELSR1	914.567625	-0.931654	0.09649944	-9.6545009	4.70E-22

3319	CELSR2	952.301576	-1.7239344	0.10527937	-16.374855	2.89E-60
3322	CEMIP	4787.99053	-0.6856353	0.08804261	-7.7875402	6.83E-15
3325	CENPA	738.332302	-0.3710381	0.09346176	-3.969946	7.19E-05
3326	CENPB	5125.39369	-0.6759768	0.0731637	-9.2392371	2.48E-20
3329	CENPC	571.792923	-0.4496838	0.11941439	-3.7657423	0.00016605
3332	CENPH	638.523499	0.38345787	0.11191401	3.42636166	0.00061173
3335	CENPK	1867.77264	-0.1912972	0.06989978	-2.7367349	0.00620523
3337	CENPM	1172.95285	-0.2306812	0.11739868	-1.9649386	0.04942134
3341	CENPQ	1371.69525	0.22778001	0.07829566	2.90922884	0.00362322
3343	CENPU	2905.55365	-0.5085096	0.05935023	-8.5679468	1.05E-17
3347	CENPW	879.507756	-0.3786119	0.15652446	-2.4188675	0.01556891
3348	CEP104	1179.75833	0.33717668	0.08300167	4.06228784	4.86E-05
3353	CEP131	617.261158	-0.7378301	0.11116544	-6.6372258	3.20E-11
3362	CEP192	1483.3149	-0.212213	0.08287493	-2.5606422	0.01044789
3364	CEP290	1275.1994	0.42181816	0.07907345	5.33451093	9.58E-08
3369	CEP44	601.344292	-0.2644059	0.10829177	-2.4416063	0.01462208
3370	CEP55	5127.83418	-0.2235441	0.06691486	-3.3407237	0.0008356
3374	CEP68	356.636839	-0.4151779	0.12843284	-3.2326463	0.00122649
3375	CEP70	485.649406	-0.28658	0.11429348	-2.5074042	0.01216215
3376	CEP72	749.220013	0.28126888	0.08392911	3.35126734	0.00080443
3378	CEP78	805.404179	-0.4518488	0.08416868	-5.3683728	7.95E-08
3379	CEP83	524.23693	0.76709917	0.10010874	7.66265936	1.82E-14
3381	CEP85	1209.44025	0.3574284	0.09178734	3.89409236	9.86E-05
3386	CEPT1	1600.57529	-0.4354	0.0778954	-5.5895468	2.28E-08
3402	CES2	1067.5665	-0.4494301	0.07648292	-5.8762155	4.20E-09
3403	CES3	47.5598894	-1.8375627	0.31192989	-5.8909476	3.84E-09
3409	CETN3	1165.99221	-0.640295	0.09403535	-6.8090882	9.82E-12
3413	CFAP20	1055.62721	-0.3605715	0.08390165	-4.2975501	1.73E-05
3417	CFAP44	379.937002	-0.8626033	0.11232696	-7.6793968	1.60E-14
3434	CFB	6274.42087	-0.4918842	0.05524927	-8.902999	5.44E-19
3439	CFH	58.2501094	-1.1440032	0.26597422	-4.3011808	1.70E-05
3445	CFI	47.6234684	-3.0541499	0.36977009	-8.2595916	1.46E-16
3448	CFL2	5059.34095	0.35208731	0.05537233	6.35854227	2.04E-10
3451	CFP	397.049424	-0.6082568	0.13409423	-4.53604	5.73E-06
3462	CGNL1	170.952023	-1.9570448	0.18311141	-10.687727	1.16E-26
3464	CGRRF1	750.423409	0.62731757	0.09727284	6.44905144	1.13E-10
3469	CHAC1	781.686869	3.30595053	0.10687723	30.932226	4.41E-210
3470	CHAC2	422.335908	1.00987029	0.11018032	9.16561367	4.93E-20
3477	CHCHD1	1655.06001	-0.2533808	0.11144616	-2.273571	0.02299179
3480	CHCHD3	4601.37017	0.18398298	0.0737396	2.49503621	0.01259443
3481	CHCHD4	1233.20979	0.94488205	0.07249875	13.03308	7.93E-39
3483	CHCHD6	387.504464	-0.5699792	0.11965597	-4.7634831	1.90E-06
3485	CHD1	2399.26159	0.53168413	0.10595506	5.01801567	5.22E-07

3504	CHI3L2	16.5198455	-1.4115172	0.51282677	-2.7524249	0.00591557
3507	CHIC1	681.374445	0.3395287	0.10356808	3.27831411	0.00104429
3508	CHIC2	1459.8067	0.6444935	0.09310261	6.92239971	4.44E-12
3511	CHKA	3411.11993	-0.3569399	0.06148022	-5.8057673	6.41E-09
3518	CHML	1419.89854	0.56436579	0.0908098	6.21481142	5.14E-10
3519	CHMP1A	2943.44513	-0.1528326	0.06934688	-2.2038853	0.02753241
3520	CHMP1B	3439.93612	0.59622703	0.06776495	8.79845735	1.39E-18
3522	CHMP2A	2666.01812	-0.526133	0.1080622	-4.8687981	1.12E-06
3523	CHMP2B	2593.78005	0.25362858	0.06524371	3.88740263	0.00010132
3525	CHMP4A	1300.41147	0.24384789	0.08728067	2.79383622	0.00520869
3526	CHMP4B	8670.26834	0.28777886	0.07351293	3.9146697	9.05E-05
3527	CHMP4C	431.269612	1.32702065	0.10886228	12.1899034	3.52E-34
3535	CHORDC1	1998.11643	0.17812197	0.06835785	2.60572801	0.00916792
3539	CHPF2	4397.23501	-0.8241906	0.05604869	-14.704904	6.00E-49
3543	CHRDL1	43.1638525	-2.7922815	0.38678261	-7.2192528	5.23E-13
3558	CHRNA5	279.053833	-0.4271031	0.12625706	-3.3828056	0.00071749
3574	CHST14	1124.71931	-0.5918618	0.07694545	-7.6919669	1.45E-14
3575	CHST15	4475.41643	-0.5821505	0.07085921	-8.2155937	2.11E-16
3577	CHST3	3055.9144	-0.2684499	0.06726752	-3.990781	6.59E-05
3578	CHST4	87.4823971	-1.3655085	0.23401741	-5.8350722	5.38E-09
3581	CHST7	745.897513	-0.5442156	0.10299497	-5.2839046	1.26E-07
3585	CHSY3	11.3452513	-1.2820929	0.63856009	-2.0077875	0.04466588
3586	CHTF18	1053.73251	-0.9870588	0.07929457	-12.448	1.43E-35
3588	CHTOP	4087.62098	0.74751218	0.06836017	10.934909	7.85E-28
3592	CIAO1	3987.59592	0.19942964	0.05631413	3.54137815	0.00039804
3593	CIAPIN1	2083.17852	0.42519383	0.09472575	4.48868266	7.17E-06
3595	CIB1	2321.34139	-0.2270046	0.10130569	-2.2407885	0.02503978
3606	CILP2	142.328416	0.40168551	0.17717592	2.26715633	0.02338068
3607	CINP	836.939815	-0.2325097	0.10342338	-2.2481347	0.02456761
3608	CIPC	775.199278	0.64504552	0.08319084	7.75380417	8.92E-15
3609	CIR1	1298.20034	-0.6361514	0.07664771	-8.2996793	1.04E-16
3610	CIRBP	4391.88385	0.5424992	0.07603393	7.13496161	9.68E-13
3612	CIRH1A	2852.88343	0.70400462	0.05856348	12.0212233	2.75E-33
3618	CIT	2448.84642	-0.2233722	0.09455518	-2.3623478	0.0181596
3620	CITED2	6131.23479	-0.2323994	0.05029449	-4.6207733	3.82E-06
3622	CIZ1	6022.82193	-0.1953865	0.05414521	-3.6085646	0.0003079
3625	CKAP4	11806.1518	-0.1827653	0.04733929	-3.8607515	0.00011304
3626	CKAP5	11249.4103	0.16687852	0.06089911	2.74024549	0.00613933
3628	CKLF	661.894909	-0.4069884	0.10199282	-3.990363	6.60E-05
3634	CKMT2-AS1	179.898502	0.76678248	0.15612308	4.91139724	9.04E-07
3636	CKS2	2560.19401	-0.3514445	0.08160502	-4.3066526	1.66E-05
3639	CLASRP	1358.14377	-0.5721232	0.08089502	-7.0724164	1.52E-12
3645	CLCC1	1334.57495	-1.116908	0.07018063	-15.914762	5.01E-57

3648	CLCN2	322.907314	-0.6775465	0.12071485	-5.6127847	1.99E-08
3649	CLCN3	2824.26043	-0.5105519	0.07934742	-6.434386	1.24E-10
3651	CLCN5	417.823622	-0.5111264	0.11191526	-4.5670841	4.95E-06
3652	CLCN6	927.057216	0.5785184	0.08154477	7.09448817	1.30E-12
3656	CLDN1	2178.97733	0.82380764	0.07664675	10.7481092	6.05E-27
3659	CLDN11	632.479452	-0.7868335	0.08883045	-8.8577005	8.17E-19
3662	CLDN15	409.207088	0.66658744	0.10529515	6.33065672	2.44E-10
3667	CLDN2	189.913251	-1.6308817	0.15829929	-10.302521	6.86E-25
3670	CLDN23	527.113895	-0.8780653	0.10086087	-8.705708	3.16E-18
3673	CLDN3	189.278176	-1.0518874	0.15878452	-6.6246216	3.48E-11
3675	CLDN4	7476.61367	0.15567783	0.06775656	2.29760527	0.02158427
3701	CLEC3B	40.7648069	-1.193669	0.33082059	-3.6082063	0.00030832
3717	CLIC1	30971.7189	0.54020743	0.08409656	6.42365692	1.33E-10
3718	CLIC2	289.193137	-0.4880746	0.12248792	-3.9846757	6.76E-05
3720	CLIC4	14589.7498	0.57885746	0.06761578	8.56098169	1.12E-17
3721	CLIC5	59.4017754	1.23551824	0.2996571	4.12310683	3.74E-05
3722	CLIC6	1787.0036	0.16502719	0.07487564	2.204017	0.02752315
3723	CLINT1	4939.53859	0.25047313	0.07209837	3.47404685	0.00051267
3727	CLIP3	71.2900972	-0.7331046	0.25549046	-2.8694011	0.0041125
3729	CLK1	2921.94485	0.27644228	0.05992283	4.61330514	3.96E-06
3733	CLK4	963.88962	0.28821869	0.07727647	3.72970838	0.0001917
3736	CLMN	511.718464	-0.7554062	0.10230704	-7.3837169	1.54E-13
3738	CLN3	1595.46369	-0.5225516	0.08930825	-5.8511011	4.88E-09
3743	CLNS1A	4036.31559	0.21659154	0.07368141	2.93956851	0.0032867
3753	CLPX	1822.10932	0.1589367	0.06988335	2.27431431	0.0229471
3762	CLSTN3	456.077727	-0.7205695	0.10178011	-7.0796692	1.44E-12
3766	CLTCL1	515.457283	0.46553759	0.10424619	4.46575137	7.98E-06
3767	CLU	3830.41027	-1.1040136	0.06631885	-16.647055	3.18E-62
3768	CLUAP1	719.288725	0.8712582	0.08513472	10.233876	1.40E-24
3769	CLUH	6393.11462	0.73182143	0.05291436	13.8302986	1.67E-43
3779	CMAS	1878.6941	-0.3447761	0.08826341	-3.9062172	9.38E-05
3780	CMBL	1544.58612	-0.5670227	0.10845336	-5.2282636	1.71E-07
3784	CMIP	3066.35113	-0.2115122	0.06623588	-3.193317	0.00140648
3786	CMPK1	6630.85144	0.20775712	0.05424215	3.83017872	0.00012805
3794	CMTM6	4703.77817	-0.6840436	0.06377134	-10.726505	7.64E-27
3795	CMTM7	2890.2523	-0.3276206	0.08531893	-3.8399521	0.00012306
3796	CMTM8	259.473755	-0.7980752	0.13402725	-5.9545746	2.61E-09
3798	CMTR2	1547.51469	1.51837441	0.06916176	21.9539577	7.94E-107
3802	CNBP	12089.615	0.81468314	0.0512952	15.8822494	8.41E-57
3804	CNDP2	7595.85957	0.68945073	0.06792704	10.1498717	3.32E-24
3807	CNGA1	211.277399	-2.1287391	0.15518546	-13.717387	7.99E-43
3811	CNGB1	52.824481	-1.206466	0.3077364	-3.9204529	8.84E-05
3820	CNN1	14.7368705	6.36934976	1.26350873	5.04100178	4.63E-07

3821	CNN2	7310.02071	-0.7574824	0.05917628	-12.800439	1.63E-37
3822	CNN3	11854.5363	-0.4516444	0.04694107	-9.6215204	6.49E-22
3824	CNNM2	416.156858	-0.9279114	0.11017573	-8.4221042	3.70E-17
3825	CNNM3	1191.92747	-0.7387487	0.07474254	-9.8839132	4.89E-23
3830	CNOT2	2026.24825	0.19224581	0.06391121	3.00801392	0.00262961
3833	CNOT6	2009.42192	-0.3186546	0.07798981	-4.0858495	4.39E-05
3842	CNPY4	568.024075	-1.0766804	0.11735183	-9.1748071	4.52E-20
3846	CNST	1628.96616	0.97567415	0.06846781	14.2501155	4.48E-46
3861	CNTNAP1	814.170348	-1.0255357	0.09563866	-10.723025	7.94E-27
3870	COA1	1913.6691	-0.3442514	0.07409463	-4.6461049	3.38E-06
3873	COA5	636.324579	-0.2351576	0.089411	-2.6300748	0.00853661
3875	COA7	869.283585	0.94307452	0.09837206	9.58681246	9.08E-22
3877	COBL	97.9119721	-0.5052312	0.24603499	-2.0534932	0.04002477
3880	COG1	1556.86015	1.22972072	0.07201747	17.0753103	2.27E-65
3882	COG3	1018.31009	0.21695715	0.07983293	2.71763996	0.00657493
3883	COG4	1339.92207	-0.1973607	0.07156045	-2.7579586	0.00581636
3884	COG5	1937.29897	0.26831483	0.08904265	3.01332945	0.00258398
3885	COG6	626.764331	-0.8578245	0.10893588	-7.8745816	3.42E-15
3886	COG7	415.202911	-0.7476074	0.11957049	-6.2524409	4.04E-10
3887	COG8	340.451192	-0.6158246	0.14522013	-4.2406283	2.23E-05
3893	COL13A1	3523.04194	-0.7804231	0.05733491	-13.611656	3.41E-42
3895	COL15A1	60.5908406	1.16678034	0.25546919	4.56720569	4.94E-06
3915	COL4A1	1815.02341	-0.8527079	0.07480336	-11.399326	4.21E-30
3916	COL4A2	6646.50787	-0.2576574	0.07203043	-3.5770637	0.00034748
3918	COL4A3	16.8399857	-2.5060848	0.5790826	-4.3276811	1.51E-05
3919	COL4A3BP	1699.85183	-0.2504356	0.07635115	-3.2800506	0.00103788
3920	COL4A4	132.358572	-0.9056842	0.19720387	-4.5926291	4.38E-06
3927	COL6A2	5524.49901	0.38123787	0.05027318	7.58332545	3.37E-14
3933	COL7A1	8069.06518	0.30122163	0.06897938	4.36683603	1.26E-05
3934	COL8A1	1304.62867	-0.753109	0.10865831	-6.9309843	4.18E-12
3938	COL9A3	19.0296861	-1.5201683	0.50396292	-3.016429	0.00255771
3948	COMMD10	1166.61538	-0.2867201	0.07651718	-3.7471341	0.00017887
3952	COMMD4	1657.95176	-0.5883615	0.1229926	-4.7837144	1.72E-06
3953	COMMD5	1060.99283	-0.2950273	0.10503609	-2.8088182	0.00497237
3957	COMMD9	930.996774	-0.583229	0.08203427	-7.1095773	1.16E-12
3964	COPE	4738.83739	-0.209716	0.07178278	-2.921536	0.0034831
3965	COPG1	5344.247	-0.4317633	0.05409029	-7.982269	1.44E-15
3969	COPS2	3111.6253	0.22580526	0.0657592	3.43381984	0.00059514
3970	COPS3	3634.37691	0.49038275	0.06258312	7.83570296	4.66E-15
3976	COPS8	1940.51138	-0.2229585	0.0623393	-3.5765316	0.00034818
3977	COPZ1	4292.2442	0.13368598	0.0520616	2.56784218	0.01023337
3979	COQ10A	416.503283	-0.3700456	0.11704381	-3.1615992	0.00156905
3980	COQ10B	1749.87433	0.16346804	0.06724453	2.43094935	0.01505932

3981	COQ2	1337.34896	-0.7317073	0.08025392	-9.117403	7.69E-20
3987	COQ9	1202.24791	-0.2107073	0.07769958	-2.7118204	0.00669148
3990	CORO1B	6015.37814	-0.2885387	0.06493406	-4.4435641	8.85E-06
3992	CORO2A	1110.82513	-0.4008017	0.075008	-5.3434529	9.12E-08
3994	CORO6	43.2461591	-0.6392495	0.30753049	-2.0786542	0.03764914
4003	COX15	1280.86167	-0.1465444	0.06899326	-2.1240387	0.0336669
4006	COX18	989.439741	-0.4846449	0.08121006	-5.9677933	2.40E-09
4007	COX19	691.577486	0.75510069	0.09472766	7.97127991	1.57E-15
4008	COX20	1283.68694	-0.2191287	0.08305689	-2.6382964	0.00833237
4011	COX5A	2612.14948	-0.4665219	0.07268745	-6.4181907	1.38E-10
4015	COX6B1	3976.87335	-0.3931012	0.08252808	-4.7632417	1.91E-06
4023	COX7C	3277.7348	0.40218332	0.09114449	4.41259074	1.02E-05
4024	COX8A	6927.46129	-0.2795334	0.07645308	-3.6562739	0.00025591
4030	CPA4	694.773957	0.56191974	0.0897166	6.26327531	3.77E-10
4031	CPA5	5.32200099	-2.0111519	0.96980162	-2.0737766	0.03810007
4037	CPD	11763.9999	-0.549049	0.07015208	-7.8265529	5.01E-15
4038	CPE	1608.58169	-1.0612265	0.06769129	-15.677446	2.16E-55
4041	CPEB2	1906.76815	0.20140559	0.08898784	2.2632933	0.02361761
4044	CPEB4	2249.22591	0.75332979	0.09946189	7.57405453	3.62E-14
4050	CPM	344.043562	-0.3898562	0.11209917	-3.4777796	0.00050559
4054	CPNE2	1197.11755	-0.9182388	0.07231936	-12.696997	6.14E-37
4059	CPNE7	586.361817	0.91549989	0.10984524	8.33445248	7.79E-17
4065	CPQ	225.503818	-1.2372891	0.14994779	-8.2514663	1.56E-16
4066	CPS1	113.841529	-1.1746307	0.19853154	-5.9165951	3.29E-09
4069	CPSF2	4441.95351	0.41114244	0.06257839	6.57003824	5.03E-11
4070	CPSF3	2378.63666	0.33499234	0.06190993	5.41096273	6.27E-08
4075	CPSF7	4505.89168	0.41554258	0.08090169	5.13638925	2.80E-07
4076	CPT1A	2756.12205	0.58642538	0.06878379	8.52563291	1.52E-17
4081	CPVL	144.079267	-0.8441922	0.1716044	-4.9194092	8.68E-07
4088	CR2	27.1550636	2.70960905	0.45739937	5.92394575	3.14E-09
4090	CRABP2	305.850542	0.60908059	0.14993342	4.06234053	4.86E-05
4091	CRACR2A	244.431638	0.57786732	0.13640332	4.23646091	2.27E-05
4092	CRACR2B	549.747405	-0.6612033	0.10425478	-6.342187	2.27E-10
4094	CRAMP1L	1061.761	0.3515173	0.10939961	3.21314948	0.00131288
4095	CRAT	1936.6283	-0.1908939	0.06885527	-2.7723929	0.00556458
4103	CRCP	2711.79149	0.8281343	0.06217288	13.3198643	1.77E-40
4108	CREB3L2	2951.92298	-0.8521169	0.07739849	-11.009476	3.44E-28
4109	CREB3L3	18.4079946	-1.9490795	0.49803735	-3.9135207	9.10E-05
4111	CREB5	110.00991	1.80324401	0.20970905	8.59878948	8.06E-18
4112	CREBBP	2391.74844	0.68880245	0.09896932	6.95975764	3.41E-12
4113	CREBL2	2351.03397	-0.128654	0.06309867	-2.0389343	0.04145659
4116	CREG1	4350.8866	-0.4911662	0.06182443	-7.9445326	1.95E-15
4120	CREM	1102.57646	0.41347904	0.08040986	5.14214377	2.72E-07

4121	CRH	3.05144364	-3.1005199	1.40414308	-2.2081225	0.02723574
4131	CRIPT	772.113858	-0.4063149	0.09828549	-4.1340275	3.56E-05
4136	CRISPLD2	814.923038	-1.5129112	0.08510996	-17.77596	1.09E-70
4144	CRNDE	284.798713	0.70351051	0.14685581	4.79048478	1.66E-06
4150	CROT	330.858792	-0.7211241	0.12940985	-5.5724047	2.51E-08
4154	CRTAM	73.2812826	-0.5258853	0.24953816	-2.1074344	0.03507994
4155	CRTAP	9043.51184	-0.3009393	0.05050541	-5.9585561	2.54E-09
4156	CRTC1	817.159755	-0.2215147	0.08676671	-2.5529915	0.01068021
4161	CRY1	2662.62702	0.74264193	0.05826449	12.7460479	3.28E-37
4162	CRY2	482.116237	0.60379892	0.09778089	6.17501987	6.62E-10
4179	CRYL1	407.396966	-0.70109	0.11213979	-6.2519291	4.05E-10
4181	CRYM-AS1	14.8981993	1.16049441	0.52933916	2.19234567	0.02835456
4183	CRYZL1	770.069989	0.28598195	0.09467999	3.02051098	0.00252349
4193	CSF1	43923.8349	-1.2955756	0.0520752	-24.878938	1.26E-136
4195	CSF2	7523.73758	1.94510984	0.06565916	29.6243484	7.26E-193
4197	CSF2RB	5.21940371	2.10416764	0.93523005	2.2498931	0.02445573
4201	CSGALNACT2	2451.12411	0.95054102	0.06162619	15.4243031	1.12E-53
4215	CSNK1A1	6572.8262	0.22952178	0.05413593	4.2397307	2.24E-05
4223	CSNK1G3	2189.17087	0.28661857	0.07382563	3.88237211	0.00010344
4225	CSNK2A2	914.825422	0.51500801	0.08759229	5.87960455	4.11E-09
4227	CSNK2B	7236.21746	0.5113154	0.0569523	8.97795816	2.76E-19
4228	CSPG4	1864.68885	0.98508106	0.06721777	14.6550685	1.25E-48
4231	CSPP1	989.807806	0.50444117	0.08489663	5.94182773	2.82E-09
4232	CSRNP1	1262.20423	1.41552542	0.07202519	19.6531992	5.43E-86
4233	CSRNP2	796.306037	0.63893435	0.08786845	7.27148781	3.56E-13
4237	CSRP2BP	882.678052	-0.4996693	0.08097343	-6.170781	6.80E-10
4239	CST1	8870.00645	-0.6894209	0.07037375	-9.7965641	1.16E-22
4242	CST2	573.632725	-0.8660558	0.11835261	-7.3175892	2.52E-13
4243	CST3	13058.0272	-0.659151	0.10319186	-6.387626	1.68E-10
4244	CST4	1182.83249	-0.3362054	0.08953293	-3.7551034	0.00017327
4247	CST7	1665.77572	-0.3796861	0.07504675	-5.0593274	4.21E-07
4253	CSTF1	1782.20884	-0.7318238	0.06664687	-10.980618	4.74E-28
4254	CSTF2	1456.96844	0.37151104	0.07202415	5.15814513	2.49E-07
4256	CSTF3	1685.743	0.24038576	0.06998382	3.43487602	0.00059282
4293	CTAGE5	2332.54385	0.31603349	0.06844639	4.61724127	3.89E-06
4302	CTBP1	6386.851	-0.4969558	0.06684011	-7.4349946	1.05E-13
4304	CTBP1-AS2	598.042704	-0.5811862	0.10489658	-5.5405642	3.01E-08
4305	CTBP2	2969.57731	0.66436996	0.0637088	10.4282283	1.84E-25
4306	CTBS	2102.36404	-0.75012	0.08611712	-8.7104632	3.03E-18
4320	CTDNEP1	4865.26692	0.14252631	0.05654437	2.52061017	0.01171516
4322	CTDSP1	2182.16454	-0.6362431	0.06561628	-9.6964211	3.12E-22
4324	CTDSPL	3187.63619	-1.1136748	0.05725589	-19.450835	2.87E-84
4327	CTGF	6102.22785	-0.1187864	0.05026174	-2.3633567	0.01811023

4329	CTH	2332.51364	2.74699822	0.0673677	40.7761912	0
4330	CTHRC1	132.812483	-1.1795139	0.19487004	-6.0528234	1.42E-09
4331	CTIF	1851.4086	-0.6679562	0.06781842	-9.8491851	6.91E-23
4339	CTNNBL1	3015.85328	0.49325089	0.09145919	5.39312537	6.92E-08
4343	CTPS1	3019.08073	0.19818586	0.06691481	2.96176388	0.00305882
4344	CTPS2	652.716872	-0.5468675	0.09506039	-5.7528427	8.78E-09
4345	CTR9	2156.27822	-0.6188428	0.06741786	-9.1792129	4.34E-20
4350	CTSA	9997.98009	-0.1709146	0.05673617	-3.0124446	0.00259153
4351	CTSB	21112.9964	-0.4796672	0.05414187	-8.8594516	8.04E-19
4353	CTSD	109558.61	-0.795817	0.05447282	-14.609434	2.45E-48
4355	CTSF	2693.79835	-0.6081696	0.09516283	-6.3908313	1.65E-10
4357	CTSH	2510.36437	-0.8298942	0.09417945	-8.8118397	1.23E-18
4358	CTSK	79.5517628	-1.0846375	0.24588293	-4.4111947	1.03E-05
4363	CTSO	300.082089	-1.3283241	0.15489659	-8.5755543	9.86E-18
4365	CTSV	278.708915	-1.2627177	0.14849728	-8.5033051	1.84E-17
4367	CTSZ	14548.7141	-0.4844779	0.07282747	-6.6524065	2.88E-11
4370	CTTNBP2NL	1559.07199	0.54657377	0.09975449	5.47918969	4.27E-08
4373	CTXN1	536.594839	0.69666336	0.15273177	4.56135205	5.08E-06
4378	CUEDC2	2259.32406	-0.3646859	0.07036001	-5.1831422	2.18E-07
4380	CUL2	2818.32181	0.1478282	0.0677251	2.18276832	0.02905288
4382	CUL4A	4592.70138	0.12158981	0.05197684	2.33930735	0.01931953
4392	CWC15	2483.76903	-0.2411689	0.063968	-3.7701495	0.00016315
4395	CWC27	1202.11953	-0.3854454	0.07537311	-5.1138311	3.16E-07
4399	CX3CL1	1093.98273	-0.6370171	0.08678652	-7.3400464	2.14E-13
4404	CXCL1	8196.18043	-1.1974834	0.0513716	-23.310222	3.49E-120
4410	CXCL16	485.076145	0.93319401	0.10219547	9.13146142	6.76E-20
4416	CXCL8	27611.9158	0.80847112	0.04957214	16.3089812	8.52E-60
4422	CXCR4	319.913276	-1.5478957	0.13832889	-11.189967	4.57E-29
4425	CXXC1	1298.48603	-0.6621389	0.08349335	-7.930439	2.18E-15
4446	CYB561A3	1697.00601	-0.2908124	0.06960801	-4.1778581	2.94E-05
4447	CYB561D1	432.171089	-0.7329722	0.10396548	-7.05015	1.79E-12
4455	CYB5R3	6744.03051	-0.4266155	0.06066142	-7.032732	2.03E-12
4458	CYBA	3711.63585	-0.3980955	0.11319954	-3.5167588	0.00043685
4460	CYBRD1	7220.38488	-0.7720496	0.07764154	-9.94377	2.68E-23
4462	CYCS	6466.23729	0.52649738	0.05641921	9.33188188	1.04E-20
4464	CYFIP1	4904.89335	-0.5249797	0.05534183	-9.4861286	2.40E-21
4470	CYLD	3693.59952	0.99267376	0.05587556	17.7657951	1.30E-70
4477	CYP1A1	351.619692	2.32486994	0.13139762	17.6933942	4.71E-70
4479	CYP1B1	11229.9487	-0.1730887	0.06008335	-2.8808093	0.00396656
4481	CYP20A1	1449.67102	-0.1542558	0.06812496	-2.264306	0.0235553
4486	CYP26B1	401.708014	0.52000335	0.11473877	4.53206325	5.84E-06
4488	CYP27A1	490.431553	-0.3282855	0.11540808	-2.8445624	0.00444725
4507	CYP2S1	200.512008	-0.6711531	0.15873972	-4.2280099	2.36E-05

4508	CYP2U1	1815.16715	-0.5613668	0.07343419	-7.6444881	2.10E-14
4513	CYP3A5	20.0932772	-1.81647	0.4660207	-3.8978311	9.71E-05
4535	CYP51A1	2871.63046	-0.7533836	0.06766726	-11.13365	8.60E-29
4545	CYSTM1	1843.15817	0.85150369	0.07342096	11.5975566	4.24E-31
4547	CYTH2	3411.53062	-0.6760687	0.08691018	-7.7789356	7.31E-15
4549	CYTH4	158.329329	-0.740773	0.1676992	-4.4172722	1.00E-05
4550	CYTIP	5.45731674	4.92126131	1.3900912	3.54024348	0.00039976
4554	D2HGDH	415.805863	-0.5659412	0.12865461	-4.3989186	1.09E-05
4560	DAB2IP	3333.89419	-0.4462048	0.06311392	-7.0698317	1.55E-12
4565	DACT3	11.2666303	2.71571716	0.73431559	3.69829702	0.00021705
4568	DAG1	5932.43458	-1.0780014	0.10312348	-10.453501	1.41E-25
4570	DAGLB	2577.07441	0.78590812	0.07437851	10.5663339	4.27E-26
4578	DAP	16140.4995	-0.3180727	0.04533823	-7.0155511	2.29E-12
4579	DAP3	5668.3109	0.67448825	0.06996842	9.63989594	5.42E-22
4580	DAPK1	18.8227534	-3.2060501	0.58955957	-5.4380427	5.39E-08
4581	DAPK2	285.802439	-1.0177887	0.13940695	-7.3008464	2.86E-13
4582	DAPK3	2875.86824	-0.3086437	0.06976897	-4.4237963	9.70E-06
4587	DARS2	1179.30686	-0.3180588	0.08818337	-3.6067894	0.00031001
4594	DAZAP1	6885.23288	0.33648057	0.0573667	5.86543412	4.48E-09
4595	DAZAP2	4267.73974	-0.1670013	0.07681995	-2.1739316	0.02971027
4601	DBH-AS1	513.221568	-1.1755554	0.11178372	-10.516338	7.26E-26
4602	DBI	2590.13773	-0.721719	0.0661539	-10.909697	1.04E-27
4605	DBN1	4493.56234	0.67149817	0.06586274	10.1954185	2.08E-24
4606	DBNDD1	428.914801	-0.5087221	0.11060227	-4.5995626	4.23E-06
4609	DBP	442.657866	-0.715089	0.12613474	-5.669247	1.43E-08
4619	DCAF13	3061.28495	0.72590172	0.07221159	10.0524267	8.96E-24
4621	DCAF15	1552.90613	-0.4558595	0.06903384	-6.6034215	4.02E-11
4624	DCAF4	878.802794	0.348234	0.08799507	3.95742637	7.58E-05
4628	DCAF6	2881.03574	-0.6739507	0.07096467	-9.4969898	2.16E-21
4630	DCAF8	3583.85432	-0.3877729	0.05881658	-6.5929174	4.31E-11
4633	DCAKD	583.464857	-0.6543314	0.10928101	-5.9876039	2.13E-09
4636	DCBLD2	13976.149	0.62615358	0.05631794	11.1181902	1.02E-28
4644	DCHS1	33.0167526	-1.3282444	0.37052924	-3.5847224	0.00033744
4648	DCLK2	360.10387	0.46776103	0.11983917	3.90323993	9.49E-05
4651	DCLRE1B	1221.4987	0.18301159	0.07981133	2.29305288	0.02184496
4654	DCP1A	1495.53151	0.53818131	0.07760098	6.93523818	4.06E-12
4657	DCPS	914.077917	-0.5497849	0.08797181	-6.2495575	4.12E-10
4672	DCUN1D2	538.521145	0.33262866	0.09594436	3.46689122	0.00052651
4673	DCUN1D3	343.43302	0.35768071	0.12473463	2.86753327	0.00413685
4675	DCUN1D5	3542.99169	0.30820458	0.0798779	3.85844634	0.00011411
4685	DDHD1	1291.87461	0.39566868	0.09154631	4.32206023	1.55E-05
4686	DDHD2	1455.73291	-0.5599142	0.07514491	-7.451127	9.25E-14
4688	DDI2	104.326326	0.98261249	0.21656434	4.53727747	5.70E-06

4689	DDIAS	883.02345	0.59549661	0.09989737	5.96108372	2.51E-09
4690	DDIT3	1480.30235	2.47574481	0.07746433	31.9598029	3.95E-224
4691	DDIT4	6413.31848	-0.3690448	0.06470674	-5.7033444	1.17E-08
4693	DDN	45.1872889	1.20730779	0.3141174	3.84349219	0.0001213
4697	DDR2	23.2742959	1.57970072	0.43452213	3.63548971	0.00027745
4698	DDRGK1	2726.44981	-0.3447214	0.09498547	-3.6292015	0.0002843
4702	DDX10	3201.16449	0.82823682	0.05788508	14.3082948	1.94E-46
4703	DDX11	965.046805	-0.2827424	0.08522007	-3.3177916	0.00090732
4711	DDX12P	222.81121	-0.8315681	0.14012435	-5.9345009	2.95E-09
4712	DDX17	6021.31882	-0.5747178	0.05890765	-9.7562515	1.73E-22
4713	DDX18	4769.96336	0.4919928	0.05167975	9.52003008	1.73E-21
4714	DDX19A	1164.40828	0.46236894	0.07636108	6.05503437	1.40E-09
4717	DDX21	14790.583	0.95485031	0.06444853	14.8157031	1.16E-49
4718	DDX23	2472.73352	-0.6376331	0.06496704	-9.8147156	9.73E-23
4719	DDX24	8401.27404	0.36966949	0.052604	7.02740276	2.10E-12
4725	DDX31	1157.85824	0.99585399	0.07621112	13.0670431	5.08E-39
4731	DDX41	2701.38172	-0.3486466	0.06446974	-5.4079101	6.38E-08
4735	DDX47	3962.38306	0.34785979	0.06809769	5.10824676	3.25E-07
4739	DDX51	959.4545	0.38664258	0.09088583	4.25415686	2.10E-05
4740	DDX52	2751.07968	0.4980218	0.05915886	8.41838066	3.82E-17
4741	DDX53	17.8452287	-4.386025	0.80501799	-5.4483565	5.08E-08
4742	DDX54	4949.89393	-0.4004294	0.05869031	-6.8227508	8.93E-12
4744	DDX56	3621.34457	0.24285309	0.06984819	3.47687011	0.0005073
4747	DDX6	5265.33664	0.24325702	0.08325869	2.92170116	0.00348125
4749	DDX60L	1953.41014	0.29743699	0.07236858	4.11002957	3.96E-05
4750	DEAF1	1529.91496	-0.2705886	0.06908044	-3.9170073	8.97E-05
4753	DECR1	1678.31344	-0.3634139	0.07984707	-4.5513744	5.33E-06
4754	DECR2	412.946303	-0.7425656	0.10915697	-6.802732	1.03E-11
4756	DEDD2	1252.71911	-0.1568037	0.07618288	-2.0582536	0.0395658
4757	DEF6	348.103751	-0.7150042	0.12646567	-5.6537411	1.57E-08
4811	DEGS1	6572.19924	-0.861409	0.05498173	-15.667187	2.54E-55
4813	DEK	20890.7838	0.21832227	0.04597006	4.74922732	2.04E-06
4814	DENND1A	1892.91055	0.21332542	0.07037054	3.03145939	0.00243375
4815	DENND1B	517.791658	0.28288455	0.12398174	2.281663	0.02250924
4818	DENND2C	126.774442	0.5843979	0.19342709	3.02128261	0.00251706
4820	DENND3	1198.34824	-0.4745525	0.08034218	-5.9066418	3.49E-09
4827	DENND6A	1909.66085	-0.138292	0.07001004	-1.975317	0.04823218
4828	DENND6B	105.365875	-0.9518143	0.1939402	-4.9077721	9.21E-07
4830	DEPDC1	2375.90374	0.36509025	0.08423108	4.33438904	1.46E-05
4832	DEPDC1B	818.516349	-0.7508928	0.09105326	-8.2467428	1.63E-16
4834	DEPDC5	252.768517	-0.2687761	0.13367234	-2.0107085	0.04435625
4842	DESI1	1985.54283	0.89069073	0.06629646	13.4349666	3.77E-41
4843	DESI2	2849.3509	0.13276508	0.06061287	2.1903776	0.02849686

4846	DFFA	1169.77475	0.27501606	0.07183603	3.82838599	0.00012899
4849	DFNB31	452.529702	-0.4846691	0.10333414	-4.6903098	2.73E-06
4851	DGAT1	1752.74182	-0.3688537	0.06745921	-5.4678037	4.56E-08
4856	DGCR14	450.203852	-0.8395518	0.11083952	-7.5744806	3.61E-14
4857	DGCR2	3472.64604	-0.656615	0.05482068	-11.977507	4.66E-33
4863	DGKA	488.065008	0.76344781	0.11595112	6.58422112	4.57E-11
4865	DGKD	2095.00375	0.36175979	0.06344634	5.70182237	1.19E-08
4869	DGKI	147.47338	1.19037705	0.21659791	5.49579194	3.89E-08
4871	DGKQ	1023.79333	-0.2845574	0.084096	-3.3837207	0.00071511
4875	DHCR24	3983.24531	-0.4738028	0.06962728	-6.8048433	1.01E-11
4876	DHCR7	3610.60934	-0.7365055	0.06951492	-10.594927	3.15E-26
4877	DHDDS	1341.61869	0.7345899	0.06944823	10.5775177	3.79E-26
4879	DHFR	1039.69542	-0.5310334	0.08907806	-5.9614382	2.50E-09
4882	DHODH	333.065789	0.37699395	0.11522713	3.27174655	0.00106885
4883	DHPS	2307.75848	-0.2869903	0.0667455	-4.2997706	1.71E-05
4884	DHRS1	869.093812	-0.3432416	0.08682425	-3.9532914	7.71E-05
4886	DHRS12	233.68063	-0.6129537	0.14139872	-4.3349306	1.46E-05
4887	DHRS13	716.842127	-0.9455192	0.12727256	-7.429089	1.09E-13
4889	DHRS3	1081.34346	-0.8915113	0.0857615	-10.395238	2.61E-25
4890	DHRS4	147.109709	-0.9833872	0.1823683	-5.3923145	6.96E-08
4893	DHRS4L2	77.8856911	-1.0892487	0.23799459	-4.5767794	4.72E-06
4894	DHRS7	1059.6399	-0.6769314	0.08570417	-7.8984654	2.82E-15
4898	DHRSX	1418.88344	-0.4754742	0.08276178	-5.7450934	9.19E-09
4899	DHTKD1	1104.07599	-0.2658846	0.07345894	-3.6194999	0.00029517
4904	DHX32	3482.64098	-0.2268659	0.05799294	-3.9119566	9.16E-05
4905	DHX33	2335.01738	0.96256571	0.07653382	12.5769977	2.83E-36
4906	DHX34	1693.91828	0.53585597	0.07382102	7.25885388	3.90E-13
4908	DHX36	2000.75771	0.54632253	0.06770414	8.06926373	7.07E-16
4909	DHX37	1656.21917	0.86252605	0.06488527	13.293095	2.54E-40
4911	DHX40	1559.32837	-0.4360886	0.08508142	-5.1255439	2.97E-07
4912	DHX57	859.387925	-0.2739672	0.09730355	-2.8155935	0.00486872
4916	DIABLO	2327.30312	0.3297422	0.07940639	4.15259029	3.29E-05
4917	DIAPH1	10780.0761	0.33012733	0.05824097	5.66830093	1.44E-08
4918	DIAPH2	708.712413	0.24335586	0.09711379	2.50588367	0.01221458
4926	DIEXF	3241.54307	0.9860422	0.06428034	15.3397166	4.15E-53
4927	DIMT1	1555.22042	0.78781473	0.08203951	9.60286951	7.77E-22
4935	DIP2B	1764.29	-0.3071854	0.10352284	-2.9673198	0.00300408
4936	DIP2C	1185.56058	-0.7546796	0.09325523	-8.0926257	5.84E-16
4943	DIS3	2408.75156	0.20778414	0.08687598	2.39173282	0.01676904
4945	DIS3L2	655.173409	-0.3953702	0.09318104	-4.2430327	2.21E-05
4952	DIXDC1	191.131718	-1.1145371	0.16155087	-6.8989853	5.24E-12
4953	DKC1	7810.21601	0.97638143	0.07074299	13.8018122	2.49E-43
4971	DLC1	4582.45854	0.72879938	0.06484965	11.2382928	2.64E-29

4974	DLEU1	515.970955	0.39287243	0.10339101	3.79987021	0.00014477
4983	DLG3	1001.89996	-0.4391677	0.07934519	-5.5348999	3.11E-08
5004	DLL4	4.59868487	3.08661249	1.15952572	2.66196121	0.00776868
5005	DLST	4311.28675	0.33016676	0.06516088	5.06694737	4.04E-07
5010	DLX3	13.1141711	1.38485933	0.66240656	2.09064857	0.03655958
5022	DMKN	171.190161	2.42430599	0.18411718	13.1671905	1.36E-39
5035	DMTN	811.340029	-0.7426784	0.08476116	-8.7620132	1.92E-18
5036	DMWD	1450.77749	0.44670013	0.07424777	6.01634386	1.78E-09
5038	DMXL2	1128.86808	-0.5732824	0.10287711	-5.5724968	2.51E-08
5039	DNA2	825.893134	-0.7565184	0.1034526	-7.3127055	2.62E-13
5041	DNAAF2	936.14537	0.69358634	0.07964654	8.70830524	3.08E-18
5042	DNAAF3	112.384127	-0.7072084	0.1926682	-3.6706028	0.00024198
5043	DNAAF5	3251.31605	-0.5048481	0.06139068	-8.2235301	1.98E-16
5048	DNAH14	634.295134	0.84491782	0.112449	7.51378674	5.74E-14
5051	DNAH2	4434.32905	-1.3083765	0.08401357	-15.573395	1.10E-54
5072	DNAJB4	818.099568	0.3791495	0.09442707	4.01526278	5.94E-05
5079	DNAJB9	1264.33697	0.17638461	0.07585868	2.32517379	0.02006267
5086	DNAJC15	1066.37758	-0.5219269	0.0739512	-7.0577209	1.69E-12
5087	DNAJC16	1022.64669	0.44109002	0.08140332	5.41857552	6.01E-08
5089	DNAJC18	578.776652	0.36917234	0.11135994	3.31512704	0.00091601
5093	DNAJC22	77.9443074	-0.777138	0.24111696	-3.2230748	0.00126822
5103	DNAJC4	454.115816	-0.5771831	0.10681	-5.4038297	6.52E-08
5107	DNAJC6	539.840248	0.68209633	0.0958201	7.11850991	1.09E-12
5109	DNAJC8	4833.94199	0.52987398	0.05503368	9.62817677	6.08E-22
5116	DNASE1L1	1366.74782	-0.1386293	0.06776289	-2.0458	0.04077605
5119	DNASE2	1873.45124	-0.2793257	0.06369098	-4.3856395	1.16E-05
5123	DNHD1	1164.66292	0.27023844	0.07681666	3.51796661	0.00043487
5125	DNM1	905.335515	-0.3828299	0.08564919	-4.4697428	7.83E-06
5130	DNM2	7765.23604	-0.157575	0.07408264	-2.1270162	0.03341874
5131	DNM3	308.442509	-1.9383321	0.12850985	-15.08314	2.09E-51
5137	DNMT3A	328.666823	-0.3657451	0.12010368	-3.045245	0.00232491
5144	DNTTIP2	5432.0217	0.36724764	0.05855341	6.27201087	3.56E-10
5145	DOC2A	131.957198	-0.560172	0.18790024	-2.98122	0.00287102
5148	DOCK1	1753.97626	-0.6441134	0.07122651	-9.0431692	1.52E-19
5149	DOCK10	312.294623	-0.7659671	0.14698647	-5.21114	1.88E-07
5156	DOCK6	925.386365	0.46110481	0.09178013	5.02401582	5.06E-07
5157	DOCK7	2951.29951	0.69537981	0.08950108	7.7695131	7.88E-15
5162	DOHH	1348.15228	0.48650826	0.10236044	4.75289358	2.01E-06
5163	DOK1	803.08969	0.21945089	0.10230003	2.14516941	0.0319393
5166	DOK4	320.1283	-0.8587919	0.12638167	-6.7952256	1.08E-11
5170	DOLK	1159.02249	-0.2045228	0.07731413	-2.6453482	0.00816069
5173	DOPEY1	423.345758	0.45576333	0.1317493	3.45932252	0.00054154
5186	DPH2	1249.44221	0.44551456	0.08874193	5.02033915	5.16E-07

5187	DPH3	2184.90615	0.53226319	0.06090332	8.73947732	2.34E-18
5189	DPH5	1130.13913	0.45767917	0.07576625	6.04067346	1.53E-09
5190	DPH6	143.581694	-0.5422443	0.1851389	-2.9288511	0.00340217
5193	DPM1	2259.6732	0.18511476	0.06228787	2.97192298	0.00295941
5195	DPM3	719.36938	-0.6849023	0.16053679	-4.266326	1.99E-05
5200	DPP4	387.45401	-0.4875026	0.13560813	-3.5949362	0.00032447
5204	DPP9	6099.52086	-0.1236032	0.06164484	-2.0050854	0.04495391
5223	DPY30	1183.56447	-0.2065768	0.08521532	-2.4241741	0.01534325
5224	DPYD	1979.34015	-0.8897645	0.07093742	-12.542951	4.35E-36
5228	DPYSL2	4303.62739	-1.3311371	0.07161459	-18.587512	4.06E-77
5236	DRAM2	1197.62294	-0.4587997	0.07760362	-5.9120914	3.38E-09
5237	DRAP1	12081.4318	0.56548125	0.10540139	5.36502636	8.09E-08
5254	DSC2	24.5079674	-0.8561542	0.40262064	-2.1264538	0.03346549
5261	DSCC1	637.723483	-0.6882793	0.09109397	-7.5557057	4.17E-14
5263	DSCR3	2835.70718	0.26497654	0.06141442	4.31456561	1.60E-05
5278	DST	12914.1983	0.20017685	0.08117202	2.46608194	0.01366001
5288	DTNBP1	1482.60804	1.0539163	0.08603791	12.2494407	1.69E-34
5289	DTWD1	435.076445	0.51178869	0.11232682	4.55624648	5.21E-06
5290	DTWD2	177.432591	-0.9612527	0.17190402	-5.5917987	2.25E-08
5292	DTX2	1725.45778	-0.6954918	0.08120535	-8.5646054	1.08E-17
5298	DUOX1	40.4572724	-1.6300873	0.36506649	-4.4651794	8.00E-06
5303	DUS1L	3457.17309	0.3022083	0.0679554	4.44715687	8.70E-06
5306	DUS4L	591.592275	1.09717478	0.10464662	10.4845697	1.02E-25
5307	DUSP1	2274.60375	1.72400479	0.06931193	24.8731317	1.45E-136
5310	DUSP12	1681.69963	0.37994481	0.08907212	4.2655861	1.99E-05
5312	DUSP14	2266.88504	0.62100252	0.05918633	10.492331	9.37E-26
5314	DUSP16	796.234617	0.5567648	0.08524127	6.53163416	6.51E-11
5316	DUSP19	19.8436968	-1.6252406	0.49656894	-3.2729405	0.00106435
5317	DUSP2	71.1034092	2.1403893	0.27769227	7.70777425	1.28E-14
5319	DUSP22	1146.90576	0.65602364	0.09075695	7.22835723	4.89E-13
5324	DUSP3	4856.94926	-0.2430865	0.05723974	-4.2468128	2.17E-05
5326	DUSP5	7544.99611	1.52140363	0.05443417	27.9494222	6.70E-172
5328	DUSP6	2940.64596	0.19328608	0.05708632	3.3858564	0.00070957
5330	DUSP8	464.091785	0.96253939	0.10415244	9.24164017	2.43E-20
5337	DVL1	6421.29321	0.25793131	0.05725624	4.5048594	6.64E-06
5339	DVL3	2556.69187	-0.2089857	0.07960221	-2.6253755	0.00865535
5340	DXO	1154.33584	0.55826298	0.09145592	6.10417552	1.03E-09
5345	DYNC1H1	23608.6786	0.48045574	0.0718312	6.68867751	2.25E-11
5348	DYNC1LI1	3578.91078	0.73036979	0.06406331	11.400751	4.15E-30
5358	DYRK1A	2028.92608	0.91326233	0.06896526	13.2423523	5.00E-40
5359	DYRK1B	629.02395	-1.0966922	0.09262971	-11.83953	2.44E-32
5360	DYRK2	560.228804	0.24430542	0.09643279	2.53342687	0.01129533
5363	DYSF	2906.91629	-0.2902399	0.06253842	-4.6409851	3.47E-06

5371	E2F1	3655.2622	-0.6527978	0.05913834	-11.038487	2.49E-28
5373	E2F3	2607.0285	0.59720383	0.07369682	8.1035228	5.34E-16
5374	E2F4	2371.46697	0.31358945	0.06842351	4.58306582	4.58E-06
5375	E2F5	1515.92859	1.12729822	0.06738575	16.7290293	8.05E-63
5376	E2F6	496.757531	0.43700463	0.10901915	4.00851249	6.11E-05
5377	E2F7	5670.05967	1.31629403	0.07490585	17.572646	3.99E-69
5378	E2F8	744.744575	-0.5989238	0.09995418	-5.991983	2.07E-09
5380	EAF1	1401.84878	0.99816777	0.07688553	12.9825182	1.54E-38
5389	EBI3	339.162553	-0.8952053	0.11604852	-7.7140608	1.22E-14
5390	EBLN1	7.77506485	-2.1331917	0.7832638	-2.7234652	0.0064601
5392	EBLN3	2918.07896	0.37913245	0.08577959	4.41984453	9.88E-06
5393	EBNA1BP2	5453.04933	0.6445771	0.07266007	8.8711328	7.24E-19
5394	EBP	2787.48477	-0.5752817	0.09609149	-5.9868127	2.14E-09
5395	EBPL	801.250499	-0.802258	0.12469604	-6.4337083	1.25E-10
5401	ECH1	2435.88836	-0.7394812	0.08789671	-8.4130703	3.99E-17
5404	ECHDC3	1070.24116	-0.6830671	0.08765811	-7.7924002	6.57E-15
5405	ECHS1	5302.434	-0.426098	0.07782473	-5.4750981	4.37E-08
5407	ECI2	3626.0942	-0.2232864	0.06040586	-3.696436	0.00021865
5409	ECM2	24.3692605	3.39035388	0.5339196	6.3499333	2.15E-10
5412	ECSIT	1275.59357	-0.21043	0.08368778	-2.5144654	0.0119213
5420	EDC4	2159.76487	-0.3374613	0.07401672	-4.5592572	5.13E-06
5423	EDEM1	4271.2536	-0.4446347	0.06882917	-6.4599756	1.05E-10
5427	EDIL3	5063.04096	-0.7643224	0.08523378	-8.9673646	3.04E-19
5428	EDN1	1517.54658	1.18834453	0.07221556	16.4555199	7.66E-61
5429	EDN2	192.898445	-2.1527746	0.17221192	-12.500729	7.40E-36
5434	EDRF1	695.408708	-0.3356989	0.11233479	-2.9883784	0.00280462
5439	EEF1A2	4079.55608	0.41309441	0.10758751	3.83961313	0.00012323
5440	EEF1B2	2418.21987	0.42984736	0.07877423	5.45670034	4.85E-08
5445	EEF1G	16210.1448	0.17169185	0.07819188	2.19577576	0.028108
5450	EEPD1	246.868094	-0.9318994	0.13989701	-6.6613249	2.71E-11
5466	EFEMP1	2819.95884	-1.7262618	0.07319864	-23.583249	5.73E-123
5467	EFEMP2	461.078599	-0.653139	0.11782465	-5.5433134	2.97E-08
5469	EFHC1	574.36642	0.49961801	0.10160291	4.9173592	8.77E-07
5471	EFHD1	325.369687	-0.9842285	0.11814543	-8.330653	8.04E-17
5477	EFNA5	994.597112	-1.0589273	0.08903682	-11.893139	1.28E-32
5479	EFNB2	1725.00695	0.34599973	0.08950601	3.86565904	0.00011079
5480	EFNB3	90.691033	-1.2879563	0.2766924	-4.654831	3.24E-06
5487	EGF	160.839552	-0.6581737	0.17326036	-3.7987555	0.00014542
5495	EGFR	5664.4109	1.17169023	0.21587156	5.42771924	5.71E-08
5497	EGLN1	3826.51853	-0.9612876	0.0586959	-16.377423	2.77E-60
5501	EGR1	1083.79808	2.8144367	0.11268545	24.9760427	1.11E-137
5503	EGR3	5.0430402	2.4047353	1.00084513	2.4027047	0.01627432
5508	EHD2	8392.7656	-0.813439	0.05317463	-15.297502	7.94E-53

5510	EHD4	3303.87739	0.29285844	0.06168914	4.74732546	2.06E-06
5517	EHMT2	2425.33533	-1.0000125	0.0641631	-15.585477	9.14E-55
5518	EI24	6599.33238	-0.3196982	0.05173868	-6.1790945	6.45E-10
5519	EID1	5237.5043	-0.2569155	0.06374764	-4.0301957	5.57E-05
5523	EIF1	22258.4197	1.09048276	0.05811464	18.7643386	1.48E-78
5528	EIF1B	1486.55192	0.80925055	0.07014496	11.5368304	8.60E-31
5530	EIF2A	3521.8318	0.4103452	0.07134499	5.7515628	8.84E-09
5531	EIF2AK1	5427.54606	-0.6764481	0.06779367	-9.9780427	1.90E-23
5536	EIF2B2	2046.09958	0.50036049	0.06520709	7.67340625	1.67E-14
5537	EIF2B3	1305.99721	0.4791183	0.10659066	4.49493718	6.96E-06
5538	EIF2B4	1266.57524	0.21136371	0.07306131	2.89296373	0.00381625
5542	EIF2S1	6515.39131	0.11999796	0.05372776	2.23344406	0.02551968
5544	EIF2S3	8717.50868	0.30464494	0.05561415	5.47783159	4.31E-08
5549	EIF3D	7001.29817	0.38563334	0.04984768	7.73623442	1.02E-14
5550	EIF3E	12809.2592	0.27537804	0.04975898	5.5342379	3.13E-08
5552	EIF3G	3900.55052	-0.5761572	0.06136765	-9.3886142	6.08E-21
5553	EIF3H	8267.94731	0.23022466	0.06126729	3.7577089	0.00017148
5554	EIF3I	7792.35556	0.78308617	0.05557768	14.0899411	4.38E-45
5556	EIF3J	3548.03191	1.01130054	0.05996637	16.8644628	8.21E-64
5559	EIF3L	5071.12033	-0.2940442	0.05970037	-4.9253339	8.42E-07
5565	EIF4E	803.454483	0.2373329	0.09248182	2.56626538	0.01028001
5569	EIF4EBP1	2986.56604	0.8662637	0.06485308	13.3573259	1.07E-40
5570	EIF4EBP2	3678.2113	-0.4902595	0.06404698	-7.6546866	1.94E-14
5574	EIF4G2	26955.8257	0.21526404	0.05234364	4.11251535	3.91E-05
5578	EIF5A	16027.2401	0.38558587	0.08565665	4.50152867	6.75E-06
5579	EIF5A2	1223.22823	0.60639199	0.07759207	7.81512799	5.49E-15
5580	EIF5AL1	583.015042	0.41816173	0.11709352	3.57117727	0.00035538
5581	EIF5B	14878.9377	0.51294455	0.05624361	9.12005083	7.51E-20
5582	EIF6	12523.927	0.8528596	0.05809208	14.6811692	8.51E-49
5584	ELAC2	4682.68372	0.53663042	0.0562829	9.53451917	1.51E-21
5591	ELF1	1983.77793	0.85120792	0.08409738	10.1216937	4.43E-24
5593	ELF3	935.557935	-1.6070707	0.0870906	-18.45286	4.95E-76
5594	ELF4	2317.22896	0.24329327	0.08338516	2.91770451	0.00352618
5598	ELFN2	930.422274	-0.668807	0.08017983	-8.3413374	7.35E-17
5601	ELK3	8188.6769	0.43823604	0.06159013	7.11536122	1.12E-12
5603	ELL	1209.83916	0.76605594	0.07506785	10.2048477	1.89E-24
5604	ELL2	3216.38629	0.62501831	0.07487303	8.34770972	6.96E-17
5608	ELMO2	2543.6091	0.39705457	0.0620763	6.3962341	1.59E-10
5612	ELMOD3	424.534818	-0.2469369	0.1060295	-2.3289455	0.01986195
5615	ELOF1	1479.17066	0.72738285	0.08222807	8.84591933	9.08E-19
5616	ELOVL1	4377.72062	0.54448228	0.05501585	9.89682646	4.30E-23
5619	ELOVL3	17.1014349	-1.2906866	0.5034967	-2.563446	0.01036388
5623	ELOVL7	763.998433	0.21418597	0.10277834	2.08396013	0.03716379

5624	ELP2	1753.44442	0.62904137	0.06850457	9.18247265	4.21E-20
5625	ELP3	1306.78697	0.6369961	0.07138407	8.92350544	4.52E-19
5626	ELP4	627.31084	-0.3381275	0.10586769	-3.1938682	0.0014038
5630	EMB	232.565989	-2.1484615	0.19163832	-11.211023	3.60E-29
5632	EMC1	4293.47302	0.12855845	0.05511547	2.33252951	0.01967285
5633	EMC10	3126.49761	-0.2421013	0.07399298	-3.2719504	0.00106808
5635	EMC3	1499.46505	-0.1671534	0.06772217	-2.4682221	0.0135786
5636	EMC3-AS1	460.757139	0.66589051	0.12099999	5.50322798	3.73E-08
5637	EMC4	2223.79768	-0.3764561	0.06586347	-5.7157038	1.09E-08
5638	EMC6	1443.35229	-0.2546196	0.06746596	-3.7740457	0.00016062
5643	EMD	2441.04962	0.4002002	0.06873683	5.82220928	5.81E-09
5644	EME1	397.903472	-0.4924207	0.11685689	-4.2138778	2.51E-05
5646	EMG1	1407.03719	0.81547435	0.07342254	11.1065935	1.17E-28
5651	EML1	91.8149424	-1.5378458	0.23947539	-6.4217279	1.35E-10
5652	EML2	1273.66872	-0.2022303	0.08228405	-2.4577098	0.01398261
5654	EML3	1278.73925	-0.5825992	0.08851497	-6.5819286	4.64E-11
5655	EML4	2943.63921	0.25065977	0.07595573	3.30007712	0.00096658
5659	EMP2	1016.04565	-0.531143	0.09175882	-5.788468	7.10E-09
5660	EMP3	6947.12793	-0.1834899	0.05822893	-3.1511812	0.00162612
5666	ENAH	3113.67163	0.35764719	0.07856536	4.55222484	5.31E-06
5669	ENDOD1	1937.4479	-1.3429736	0.07175842	-18.715204	3.72E-78
5670	ENDOG	944.953789	-0.7435495	0.14041713	-5.2952907	1.19E-07
5674	ENGASE	706.896039	-0.3889567	0.09599138	-4.0519959	5.08E-05
5677	ENKUR	16.286159	1.6641522	0.5133753	3.24158995	0.00118865
5680	ENO2	4889.968	-0.9973848	0.06154569	-16.205601	4.60E-59
5683	ENOPH1	2846.06161	0.23343752	0.06027291	3.87300879	0.0001075
5692	ENPP4	583.402185	0.80696287	0.10897407	7.40509064	1.31E-13
5705	ENTPD4	2403.77362	-0.4461251	0.0739641	-6.0316435	1.62E-09
5707	ENTPD6	3645.51585	-0.460765	0.06143709	-7.4997854	6.39E-14
5708	ENTPD7	614.941023	0.72581023	0.09618084	7.54630778	4.48E-14
5711	EOGT	473.300798	-0.565681	0.11408854	-4.9582631	7.11E-07
5715	EP400	2850.99857	0.52348098	0.078689	6.65253087	2.88E-11
5720	EPB41L2	3402.75535	0.25618915	0.0885556	2.89297512	0.00381612
5721	EPB41L3	211.42729	5.49798366	0.3021624	18.1954591	5.61E-74
5722	EPB41L4A	15.4949411	-1.4940145	0.65989553	-2.2640167	0.02357309
5723	EPB41L4A-A!	768.045827	0.75740403	0.145808	5.19453019	2.05E-07
5726	EPB41L5	1166.61099	0.38850096	0.08605957	4.51432621	6.35E-06
5729	EPC2	972.364122	0.68660307	0.09402965	7.30198444	2.84E-13
5730	EPCAM	809.564912	0.21070947	0.09483317	2.22189638	0.02629031
5731	EPDR1	2306.68505	-0.3073103	0.06560887	-4.683975	2.81E-06
5732	EPG5	7753.91441	0.84483485	0.06339632	13.3262438	1.63E-40
5737	EPHA2	16121.5773	0.38368054	0.04906588	7.81970093	5.29E-15
5746	EPHB2	4089.68096	0.25918995	0.05584829	4.64096494	3.47E-06

5747	EPHB3	27.483593	0.85747659	0.38284256	2.23976299	0.02510631
5750	EPHX1	4350.20281	-1.2545715	0.06689178	-18.75524	1.75E-78
5751	EPHX2	113.635943	-0.8129913	0.22720824	-3.5781768	0.000346
5753	EPHX4	782.108782	-0.7240604	0.09798579	-7.3894425	1.47E-13
5756	EPN1	7844.61184	-0.2313322	0.05788827	-3.9961851	6.44E-05
5757	EPN2	1620.36162	0.30794984	0.07228152	4.26042281	2.04E-05
5760	EPN3	63.7287476	-2.7344	0.31061242	-8.8032541	1.33E-18
5762	EPOR	573.343107	-1.0493158	0.09851841	-10.650962	1.73E-26
5765	EPPK1	20.1058263	1.30140562	0.49952477	2.60528747	0.00917972
5766	EPRS	8726.25105	0.59758916	0.06479389	9.22292524	2.89E-20
5767	EPS15	4232.85101	0.7160336	0.07205119	9.9378453	2.85E-23
5768	EPS15L1	2945.32857	0.5849176	0.07120516	8.21453946	2.13E-16
5769	EPS8	2951.47757	0.20828121	0.07013859	2.96956646	0.0029822
5770	EPS8L1	411.878526	-0.3310036	0.13040069	-2.5383574	0.01113742
5771	EPS8L2	5764.97278	-0.8974927	0.05523274	-16.24929	2.26E-59
5774	EPT1	2059.62743	0.57222173	0.07951149	7.19671775	6.17E-13
5780	ERAP2	560.580363	-0.5963874	0.10801245	-5.5214695	3.36E-08
5782	ERBB2	2650.59003	-0.6880463	0.06323935	-10.880035	1.44E-27
5783	ERBB2IP	6647.32169	0.33846795	0.09279051	3.64765681	0.00026464
5784	ERBB3	238.642334	-0.8627837	0.14495902	-5.9519147	2.65E-09
5790	ERCC2	3109.11653	-0.3191382	0.06814171	-4.6834486	2.82E-06
5794	ERCC6	462.043574	0.45695071	0.13420861	3.40477943	0.00066218
5796	ERCC6L	1291.31201	-0.9597313	0.0771927	-12.432929	1.73E-35
5799	EREG	551.939196	1.57651743	0.11318943	13.9281335	4.27E-44
5804	ERGIC3	7864.77175	-0.2362252	0.08373202	-2.8212056	0.00478435
5805	ERH	6325.18097	-0.2771203	0.06590133	-4.2050791	2.61E-05
5812	ERICH2	309.760412	0.48804924	0.12496955	3.90534507	9.41E-05
5816	ERICH5	1.79038096	4.33375697	1.98751478	2.18049044	0.02922113
5820	ERLEC1	2535.30474	-0.5908745	0.06293146	-9.3891755	6.05E-21
5826	ERMP1	615.455084	-0.98953	0.0987281	-10.022779	1.21E-23
5827	ERN1	408.172406	2.19272309	0.15945363	13.7514781	4.99E-43
5829	ERO1A	6986.93773	-0.652648	0.06957384	-9.3806521	6.56E-21
5834	ERRFI1	15870.2957	1.13290469	0.05766769	19.6453981	6.33E-86
5835	ERV3-1	125.790635	0.59915651	0.20320757	2.94849506	0.00319325
5839	ERVMER34-1	117.359573	1.09328453	0.20121766	5.43334278	5.53E-08
5844	ESCO1	1657.83203	0.34446844	0.08232513	4.18424424	2.86E-05
5846	ESD	1838.86503	0.15559679	0.06850779	2.27122773	0.0231332
5847	ESF1	3007.89956	0.70880565	0.06932968	10.2236974	1.55E-24
5849	ESPL1	1801.30217	-0.3616833	0.06929127	-5.2197525	1.79E-07
5854	ESR2	128.403582	0.88446407	0.2045807	4.32330159	1.54E-05
5857	ESRP2	542.202813	0.6871489	0.10373634	6.62399398	3.50E-11
5858	ESRRA	3358.31172	0.41580575	0.07907582	5.25831763	1.45E-07
5865	ETAA1	596.387638	-0.215087	0.09907607	-2.1709281	0.0299366

5866	ETF1	8125.82073	0.37765729	0.05095733	7.41124533	1.25E-13
5867	ETFA	3688.8851	-0.2549238	0.06143568	-4.1494418	3.33E-05
5870	ETHE1	649.907589	0.67797106	0.10137768	6.68757739	2.27E-11
5872	ETNK2	1036.2008	-0.2412193	0.08490785	-2.8409535	0.00449789
5874	ETS1	14946.4623	0.7386946	0.05114186	14.4440297	2.73E-47
5875	ETS2	4858.98776	1.31837455	0.0626418	21.0462446	2.48E-98
5880	ETV4	2443.31865	0.86845359	0.06774554	12.8193468	1.28E-37
5881	ETV5	2619.19757	0.17691333	0.07154182	2.47286596	0.01340344
5884	EVA1A	1080.14271	0.74130633	0.08954495	8.27859408	1.25E-16
5888	EVC	1864.95349	-0.2777918	0.09901783	-2.8054723	0.00502429
5894	EVL	305.798331	-0.9115565	0.12651608	-7.2050643	5.80E-13
5895	EVPL	1610.34067	-0.8623269	0.07096356	-12.151686	5.62E-34
5908	EXOC2	2445.57729	-0.2491162	0.06973522	-3.5723159	0.00035384
5909	EXOC3	2385.01024	-0.2696485	0.06292974	-4.284914	1.83E-05
5911	EXOC3L1	13.9139732	-1.0955438	0.55339132	-1.9796909	0.04773827
5912	EXOC3L2	10.3965826	-4.9485574	1.16586943	-4.2445211	2.19E-05
5914	EXOC4	3003.46758	-0.601427	0.06060864	-9.9231243	3.30E-23
5917	EXOC6B	340.69223	0.77509474	0.14292846	5.42295601	5.86E-08
5918	EXOC7	6430.60021	0.21503767	0.05347495	4.0212782	5.79E-05
5920	EXOG	385.286983	0.46425425	0.1256154	3.6958388	0.00021916
5923	EXOSC2	2092.8952	0.39963418	0.07825013	5.10713741	3.27E-07
5925	EXOSC4	1200.59002	0.41514124	0.13037933	3.18410308	0.00145203
5926	EXOSC5	1874.72734	0.38534306	0.0916066	4.20649891	2.59E-05
5928	EXOSC7	1653.67134	0.26910385	0.08828189	3.04823404	0.00230191
5929	EXOSC8	1886.34423	0.40728326	0.08073455	5.04472079	4.54E-07
5931	EXPH5	425.893469	1.37786591	0.11781821	11.6948463	1.35E-31
5940	EYA3	550.311172	0.66236173	0.10639095	6.22573401	4.79E-10
5942	EYS	7.42752794	1.86191018	0.78635123	2.36778441	0.01789496
5952	F12	152.659931	1.08647184	0.17560256	6.18710695	6.13E-10
5957	F2RL1	6075.94753	0.40203095	0.06074436	6.61840758	3.63E-11
5960	F3	22446.5668	0.21056196	0.04779579	4.40545024	1.06E-05
5963	F8	249.429172	-0.5016693	0.14797766	-3.3901693	0.00069849
5964	F8A1	236.252266	-0.5788009	0.14974776	-3.8651727	0.00011101
5978	FABP3	72.6459923	-0.9555332	0.24177688	-3.952128	7.75E-05
5985	FADD	3384.03572	-0.7184721	0.06769518	-10.613341	2.58E-26
5987	FADS2	4067.25085	-1.2421393	0.06597019	-18.828798	4.39E-79
5988	FADS3	3755.45399	0.49844954	0.06524167	7.64004888	2.17E-14
5990	FAF1	1756.62511	0.36209845	0.06772165	5.34686416	8.95E-08
5991	FAF2	3826.89	0.39789345	0.06285605	6.33023357	2.45E-10
5992	FAH	1310.19203	-0.2291834	0.08841682	-2.5920786	0.0095398
6002	FAM102A	3612.37269	-0.9284295	0.05481457	-16.937641	2.37E-64
6003	FAM102B	1605.81271	-1.6415577	0.09663454	-16.987277	1.02E-64
6005	FAM104A	2066.75911	0.29099849	0.06711565	4.33577681	1.45E-05

6007	FAM105A	583.395836	-0.5331472	0.09396949	-5.6736197	1.40E-08
6012	FAM107B	4074.08383	0.76147319	0.05607329	13.5799621	5.27E-42
6015	FAM110A	469.790568	-0.4009663	0.10469775	-3.8297511	0.00012827
6019	FAM111A	2501.60266	-0.4712932	0.06138319	-7.6778872	1.62E-14
6021	FAM114A1	3819.05376	-1.2203612	0.06904214	-17.675601	6.46E-70
6022	FAM114A2	498.813064	0.3095716	0.09842063	3.14539358	0.00165864
6025	FAM118A	652.625422	0.54700594	0.0877052	6.23686988	4.46E-10
6028	FAM120AOS	985.330965	0.35144977	0.07823764	4.49208043	7.05E-06
6030	FAM120C	528.559735	-0.3320569	0.12815315	-2.5910948	0.00956711
6032	FAM122B	2094.61036	-0.6088664	0.06519145	-9.3396662	9.66E-21
6036	FAM126A	2656.9373	0.47208233	0.07749795	6.091546	1.12E-09
6038	FAM127A	3035.89832	-1.2041221	0.07935077	-15.174674	5.20E-52
6039	FAM127B	1854.18141	-0.2835684	0.06914988	-4.1007796	4.12E-05
6040	FAM127C	976.837698	-1.1152927	0.08052798	-13.849755	1.28E-43
6048	FAM132B	804.952282	0.44003071	0.0878666	5.00794054	5.50E-07
6049	FAM133A	44.0781296	-0.9633693	0.30342728	-3.1749593	0.00149858
6055	FAM134C	1879.25179	-0.6316061	0.0730577	-8.645305	5.37E-18
6056	FAM135A	761.321306	0.79474664	0.11237228	7.07244365	1.52E-12
6058	FAM136A	2905.16163	0.44961966	0.06514453	6.90187846	5.13E-12
6067	FAM13B	2823.7445	0.88071167	0.08776346	10.0350611	1.07E-23
6068	FAM13C	56.1411596	-1.3415316	0.27655477	-4.8508713	1.23E-06
6069	FAM149A	47.6077059	-0.905721	0.30416183	-2.9777602	0.00290363
6090	FAM160B1	1681.68387	-0.8285581	0.08346015	-9.9275888	3.16E-23
6091	FAM160B2	1205.44066	-0.4070898	0.07410531	-5.4933962	3.94E-08
6100	FAM167A	585.130441	0.59946593	0.10917809	5.49071633	4.00E-08
6103	FAM168A	2203.19498	-0.6370396	0.09138363	-6.9710466	3.15E-12
6105	FAM169A	453.514949	0.5831732	0.15381389	3.79142096	0.00014979
6111	FAM171A2	275.072043	-0.8279768	0.13330498	-6.2111469	5.26E-10
6113	FAM172A	389.072872	-1.144828	0.11375514	-10.063967	7.97E-24
6115	FAM173A	610.072086	-0.8842031	0.17233972	-5.1305821	2.89E-07
6124	FAM179A	84.5478958	-1.3489479	0.26043891	-5.1795174	2.22E-07
6125	FAM179B	744.312575	0.52337849	0.10668054	4.90603542	9.29E-07
6146	FAM189B	2227.95583	-0.3956112	0.07238836	-5.4651216	4.63E-08
6147	FAM192A	1679.84602	-0.4402415	0.07150712	-6.1566113	7.43E-10
6149	FAM193B	1904.68297	-0.3224367	0.06268616	-5.1436655	2.69E-07
6153	FAM196B	346.634857	1.91583336	0.13846155	13.8365735	1.53E-43
6157	FAM198B	84.5750615	-1.5917461	0.24160195	-6.5883	4.45E-11
6164	FAM200A	488.591316	0.74960559	0.10055637	7.45458087	9.02E-14
6165	FAM200B	1294.81207	0.2912385	0.07153566	4.07123541	4.68E-05
6173	FAM208A	2988.28926	0.13047564	0.062805	2.07747213	0.037758
6174	FAM208B	4294.03407	0.86786963	0.09327865	9.30405401	1.35E-20
6179	FAM20C	3872.51002	-1.7249374	0.06338122	-27.215277	4.28E-163
6180	FAM210A	2601.29935	0.36485299	0.06440339	5.6651211	1.47E-08

6183	FAM212B	135.87693	1.15449811	0.1838035	6.28115424	3.36E-10
6185	FAM213A	2935.24296	-0.3149351	0.07420305	-4.2442341	2.19E-05
6193	FAM217B	576.22019	-0.8852525	0.10823958	-8.1786392	2.87E-16
6196	FAM219B	1186.02138	-0.361125	0.07300289	-4.9467214	7.55E-07
6200	FAM220A	1234.81681	0.20304742	0.07160701	2.8355801	0.00457425
6204	FAM222A-AS	5.22226563	3.93946162	1.39064835	2.83282371	0.00461388
6227	FAM24B	203.366331	1.31327041	0.17038945	7.707463	1.28E-14
6235	FAM26F	57.2921157	-0.9114242	0.29791732	-3.0593193	0.00221841
6241	FAM32A	2790.70446	-0.3373301	0.06319027	-5.3383229	9.38E-08
6247	FAM3C	2281.3782	-0.7989253	0.07072285	-11.296566	1.36E-29
6252	FAM43A	586.513706	-0.923029	0.09422851	-9.7956447	1.18E-22
6254	FAM45A	798.64256	-0.5032608	0.08666964	-5.8066558	6.37E-09
6255	FAM45B	160.992971	-0.4196864	0.16186236	-2.5928599	0.00951815
6258	FAM46C	410.489223	-1.6535485	0.11683065	-14.153379	1.78E-45
6268	FAM50B	207.370442	-0.5356142	0.14855575	-3.6054759	0.00031158
6269	FAM53A	150.910699	0.41537557	0.17947756	2.31435936	0.02064802
6270	FAM53B	659.874627	-0.7733735	0.10133999	-7.6314739	2.32E-14
6273	FAM57A	1405.59168	-0.4607053	0.07656336	-6.0173083	1.77E-09
6276	FAM60A	1263.98329	1.14957477	0.10020277	11.4724849	1.81E-30
6277	FAM63A	485.254988	-0.7416362	0.11587942	-6.4000684	1.55E-10
6279	FAM64A	975.382718	-0.4510419	0.08943116	-5.0434531	4.57E-07
6282	FAM65C	160.723679	-0.8704179	0.16074231	-5.4149896	6.13E-08
6299	FAM72A	101.823041	0.63745125	0.21369654	2.98297421	0.00285462
6300	FAM72B	122.690641	0.48066963	0.2023252	2.37572798	0.01751437
6301	FAM72C	63.7848693	0.65932898	0.30610849	2.1539062	0.03124753
6303	FAM73A	1137.92569	0.28380897	0.0913726	3.10606203	0.00189597
6310	FAM76A	415.45442	0.95804495	0.11355897	8.43654169	3.27E-17
6314	FAM81A	75.3399998	-0.858965	0.2719379	-3.1586809	0.00158485
6321	FAM83D	2330.006	-0.6391505	0.06198857	-10.31078	6.30E-25
6324	FAM83G	1298.59881	1.24653246	0.1013927	12.2941045	9.74E-35
6328	FAM84B	2109.66929	1.06722725	0.06200224	17.2127207	2.13E-66
6335	FAM86EP	114.686316	1.74101134	0.21153103	8.23052459	1.86E-16
6337	FAM86HP	60.4850065	2.52052604	0.30105895	8.37220108	5.66E-17
6341	FAM89A	432.479012	0.50858165	0.10564725	4.81395998	1.48E-06
6350	FAM91A1	4886.08447	0.17883534	0.07849585	2.27827771	0.02271004
6357	FAM96A	1348.16594	-0.6403594	0.09845336	-6.5041904	7.81E-11
6359	FAM98A	2084.10445	0.42120483	0.06342051	6.64146122	3.11E-11
6360	FAM98B	1326.37204	-0.1835989	0.09230183	-1.9891143	0.04668859
6373	FANCE	1416.35607	0.63067446	0.07824672	8.06007573	7.62E-16
6375	FANCG	1519.48597	-0.5992758	0.08013086	-7.4787144	7.51E-14
6379	FANK1	106.888112	-0.6710868	0.21006345	-3.1946862	0.00139983
6382	FAR1	3801.87856	0.29052891	0.06877703	4.22421446	2.40E-05
6386	FARP1	1069.06685	-0.4053211	0.08249929	-4.9130259	8.97E-07

6388	FARS2	560.349138	-0.4140773	0.12136754	-3.4117636	0.00064544
6389	FARSA	4596.02809	0.24819438	0.08232536	3.01479858	0.0025715
6390	FARSB	1910.98902	0.49002899	0.07084842	6.9165837	4.63E-12
6391	FAS	227.460846	-0.527193	0.13574946	-3.8835731	0.00010293
6395	FASTK	3638.89321	-0.3020367	0.08327584	-3.6269432	0.0002868
6397	FASTKD2	1537.9971	0.41713964	0.07605315	5.48484352	4.14E-08
6398	FASTKD3	724.746024	0.289184	0.10928609	2.64611892	0.00814212
6400	FAT1	5591.35566	-1.0182858	0.09269979	-10.984769	4.52E-28
6402	FAT3	210.881964	1.36043679	0.14722232	9.24069682	2.45E-20
6407	FAXDC2	272.713175	-0.8806324	0.13462535	-6.5413569	6.10E-11
6409	FBL	10055.4662	0.30915053	0.10061824	3.07250973	0.00212267
6410	FBLIM1	758.424259	0.67153681	0.08439993	7.9566035	1.77E-15
6415	FBLN7	444.985519	-1.0644628	0.12446501	-8.5523057	1.21E-17
6419	FBP1	1377.24059	-0.467764	0.0859213	-5.4440979	5.21E-08
6421	FBRS	2829.11595	0.62325223	0.08869121	7.02721548	2.11E-12
6422	FBRSL1	1528.50584	0.55365585	0.07974818	6.94255158	3.85E-12
6423	FBXL12	866.624579	0.22792501	0.08446378	2.69849404	0.0069654
6424	FBXL13	256.5635	1.21349854	0.14855163	8.16886736	3.11E-16
6426	FBXL15	595.339023	-0.8947692	0.13413831	-6.6704974	2.55E-11
6428	FBXL17	554.146607	-1.0483135	0.1170515	-8.956002	3.37E-19
6431	FBXL19-AS1	139.567673	0.53374696	0.210753	2.5325711	0.01132294
6433	FBXL20	519.106752	-0.6784424	0.11884996	-5.7083942	1.14E-08
6438	FBXL5	2177.69421	-0.3307042	0.06243445	-5.296823	1.18E-07
6441	FBXL8	99.5353144	-1.1696567	0.21107976	-5.5413021	3.00E-08
6443	FBXO11	1877.48181	0.21936707	0.06775883	3.23746864	0.00120595
6446	FBXO17	1068.02706	0.27980969	0.10390143	2.6930301	0.00708059
6453	FBXO25	599.413885	-0.1911979	0.09435076	-2.0264589	0.04271778
6456	FBXO3	1131.67398	-0.6749291	0.07447684	-9.0622685	1.28E-19
6458	FBXO30	1033.56941	0.39496792	0.08467006	4.66478862	3.09E-06
6462	FBXO34	1986.67201	0.18153118	0.06178126	2.93828857	0.0033003
6469	FBXO42	1045.30594	0.3285554	0.07744989	4.24216746	2.21E-05
6470	FBXO43	351.1015	-0.3814732	0.11859464	-3.2166142	0.00129713
6472	FBXO45	2180.08343	0.2564245	0.06167714	4.15752868	3.22E-05
6477	FBXO6	132.532737	0.72999828	0.18265452	3.99660665	6.43E-05
6479	FBXO8	843.576994	-0.6069534	0.08518379	-7.1252215	1.04E-12
6480	FBXO9	1349.27107	-1.0151179	0.0696623	-14.571983	4.23E-48
6481	FBXW10	23.5751658	1.99845132	0.43911544	4.55108414	5.34E-06
6487	FBXW5	5723.36883	-0.2766737	0.05868338	-4.7146866	2.42E-06
6490	FBXW9	441.274583	-0.3251328	0.10721445	-3.0325466	0.002425
6496	FCF1	1981.26708	0.29859837	0.06879958	4.34011918	1.42E-05
6497	FCGBP	363.795306	-1.6496234	0.12905856	-12.781976	2.07E-37
6501	FCGR2A	10.0205074	-1.4994987	0.65225031	-2.298962	0.0215071
6506	FCGRT	1603.67973	-0.490501	0.08746268	-5.6081174	2.05E-08

6524	FDFT1	2894.11199	-0.6125895	0.06705822	-9.1351892	6.53E-20
6525	FDPS	4757.52978	-0.4545752	0.08017228	-5.6699792	1.43E-08
6530	FDXR	220.497603	-0.5673019	0.1779054	-3.1887841	0.00142873
6532	FEM1A	1624.90758	0.36638701	0.06519393	5.6199563	1.91E-08
6533	FEM1B	1687.46158	0.19592787	0.06902413	2.83854178	0.00453202
6534	FEM1C	2196.83571	0.40928937	0.07884683	5.19094254	2.09E-07
6545	FERMT2	3531.38041	0.24473518	0.0700498	3.49373133	0.00047632
6551	FEZ2	3683.92811	0.43000275	0.06688778	6.42871875	1.29E-10
6558	FFAR4	26.2272835	-2.9726933	0.48430123	-6.1381082	8.35E-10
6561	FGD1	974.544657	-0.2556848	0.07556279	-3.3837392	0.00071506
6563	FGD3	22.1718605	-1.3016569	0.44985008	-2.8935348	0.00380932
6566	FGD5-AS1	4730.54472	-0.4231839	0.06606474	-6.4055941	1.50E-10
6597	FGFBP1	32.3649997	-2.621203	0.41977355	-6.2443264	4.26E-10
6600	FGFR1	4505.81359	0.60401377	0.06939218	8.70434936	3.19E-18
6601	FGFR1OP	2019.35266	-0.211109	0.07467835	-2.8269101	0.00469995
6613	FHAD1	33.860806	0.73221677	0.36536109	2.00409075	0.04506034
6614	FHDC1	119.411089	-0.8881954	0.19463769	-4.5633267	5.03E-06
6617	FHL2	1636.82808	1.12073409	0.08004933	14.0005426	1.55E-44
6618	FHL3	1143.01287	0.51026614	0.07786875	6.55289985	5.64E-11
6620	FHOD1	1056.83997	-1.1528439	0.08861064	-13.010221	1.07E-38
6621	FHOD3	1980.63811	-0.3490117	0.07148276	-4.8824602	1.05E-06
6625	FICD	270.970105	1.12114725	0.13858862	8.08974947	5.98E-16
6626	FIG4	483.754125	-0.273663	0.09939261	-2.7533536	0.00589881
6629	FIGN	433.483604	0.42760099	0.14333314	2.98326674	0.00285189
6630	FIGNL1	1465.52787	0.27991968	0.07984949	3.50559134	0.00045559
6636	FIS1	2532.22279	-0.3586866	0.10008707	-3.5837451	0.0003387
6640	FJX1	3490.86655	0.25930627	0.0706734	3.66907885	0.00024343
6644	FKBP15	1307.58014	-0.187764	0.08145401	-2.3051541	0.02115795
6651	FKBP4	5528.79849	0.47763352	0.06207692	7.69422078	1.42E-14
6654	FKBP7	374.517124	-0.4870266	0.11391853	-4.2752186	1.91E-05
6656	FKBP9	3715.91064	-0.1682219	0.05419136	-3.1042207	0.00190781
6657	FKBP9P1	20.0191605	-1.5690896	0.48601029	-3.228511	0.00124436
6658	FKBPL	615.034078	0.44073385	0.09213239	4.78370165	1.72E-06
6662	FLAD1	923.46567	0.8383414	0.08902683	9.41672763	4.65E-21
6667	FLI1	56.6368321	-1.9778195	0.30103914	-6.5699746	5.03E-11
6669	FLJ10038	322.898516	-0.4249166	0.12968799	-3.276453	0.0010512
6709	FLJ42627	41.9674432	1.05352756	0.31010003	3.39737973	0.00068034
6720	FLNB	49686.7163	0.62495149	0.06861305	9.10834723	8.36E-20
6721	FLNC	13119.1044	0.7498698	0.08032532	9.33541053	1.01E-20
6723	FLOT2	4627.46554	-0.5690494	0.05136022	-11.079574	1.58E-28
6738	FMNL1	5200.58353	-0.1519433	0.05337117	-2.846918	0.00441447
6753	FN3K	407.184591	-1.1001595	0.12131626	-9.0685248	1.21E-19
6755	FNBP1	3195.41964	-0.5253974	0.06145868	-8.5487909	1.24E-17

6757	FNBP4	2302.60024	0.20264685	0.06802615	2.97895519	0.00289233
6760	FNDC3B	7573.6491	0.31119104	0.09188381	3.38678843	0.00070716
6774	FOLR1	126.188463	-1.7539595	0.19954369	-8.789852	1.50E-18
6778	FOS	133.52784	1.27802778	0.1989618	6.42348312	1.33E-10
6779	FOSB	53.2871412	2.95918807	0.3363831	8.79707703	1.40E-18
6780	FOSL1	13499.326	0.5795565	0.05572217	10.4008251	2.46E-25
6781	FOSL2	5485.19803	0.6719112	0.07247877	9.27045578	1.85E-20
6787	FOXC1	724.806906	0.55721623	0.09966075	5.59113035	2.26E-08
6814	FOXJ3	2216.79425	0.48573854	0.06921656	7.01766322	2.26E-12
6816	FOXK2	5207.17871	0.56525417	0.0559179	10.1086452	5.06E-24
6820	FOXM1	5932.90999	-0.2806651	0.06497427	-4.3196345	1.56E-05
6822	FOXN2	1073.43819	0.7630444	0.11853768	6.43714625	1.22E-10
6824	FOXN3-AS1	193.356699	1.34603014	0.16925808	7.95253089	1.83E-15
6828	FOXO3	1411.65212	0.95575337	0.07427252	12.8681968	6.80E-38
6830	FOXO4	309.368289	-0.5607525	0.1253179	-4.4746399	7.65E-06
6838	FOXQ1	756.554668	-2.146373	0.09900454	-21.679541	3.20E-104
6842	FOXRED2	1414.28228	-0.8822123	0.08719076	-10.118186	4.59E-24
6847	FPR1	157.413438	-0.5826636	0.18041075	-3.2296503	0.00123942
6848	FPR2	7.38668445	-1.7140615	0.81373725	-2.1064065	0.03516905
6851	FRAS1	1616.20111	-0.9881384	0.09133006	-10.819422	2.79E-27
6852	FRAT1	80.0724057	-0.8121453	0.2455493	-3.3074635	0.00094145
6853	FRAT2	620.988777	-0.3154425	0.11860478	-2.6596106	0.0078231
6857	FRG1	508.386347	-0.3441535	0.11410826	-3.0160262	0.00256111
6861	FRG1HP	337.28027	-0.3241498	0.11883761	-2.7276698	0.00637834
6870	FRMD3	452.073736	-1.2280102	0.10791929	-11.378968	5.32E-30
6872	FRMD4B	227.705714	-1.0098047	0.15723733	-6.4221692	1.34E-10
6874	FRMD6	10010.5224	0.82038761	0.0698463	11.7456127	7.44E-32
6883	FRMPD3	22.017325	1.06220059	0.45444035	2.33738175	0.01941934
6886	FRRS1	69.5475653	-0.6578962	0.24261581	-2.7116791	0.00669434
6890	FRY	73.0907886	-1.2729209	0.28332612	-4.4927764	7.03E-06
6896	FSCN1	1052.53686	-1.5640485	0.08247885	-18.963025	3.45E-80
6900	FSD1L	454.436925	0.38356176	0.11827403	3.2429921	0.00118281
6908	FSTL3	4056.54145	1.05975267	0.09881662	10.7244373	7.82E-27
6917	FTL	72483.7935	0.36614265	0.07247317	5.05211316	4.37E-07
6922	FTSJ1	4239.50646	0.57631705	0.05541215	10.4005544	2.46E-25
6928	FUCA1	1842.49972	-0.5441032	0.06603583	-8.2395153	1.73E-16
6930	FUK	206.057772	-0.7721715	0.1497477	-5.156483	2.52E-07
6935	FURIN	5702.00433	-0.3963544	0.06355566	-6.2363359	4.48E-10
6936	FUS	13870.6618	0.18077364	0.06490076	2.78538572	0.00534641
6937	FUT1	145.557692	1.89759396	0.18565454	10.221102	1.60E-24
6938	FUT10	234.997085	-0.403636	0.15249223	-2.6469279	0.00812267
6939	FUT11	538.069028	-1.1535859	0.12200961	-9.4548769	3.23E-21
6946	FUT8	5584.27306	-1.053305	0.05755916	-18.299522	8.35E-75

6947	FUT8-AS1	156.64069	-0.4422039	0.16205374	-2.7287487	0.00635751
6952	FXR2	3638.52225	0.17163875	0.05583564	3.07399988	0.0021121
6963	FYN	2759.03326	0.74806157	0.06020275	12.4257046	1.90E-35
6965	FZD1	1454.75645	-1.4203293	0.07460201	-19.038753	8.14E-81
6968	FZD2	2404.27114	-0.4218418	0.07334575	-5.7514146	8.85E-09
6970	FZD4	566.711762	-0.4473813	0.09599547	-4.6604421	3.16E-06
6972	FZD6	2906.53826	-0.6848352	0.07210545	-9.4976894	2.15E-21
6977	G0S2	8875.0192	0.64145257	0.11175571	5.73977471	9.48E-09
6979	G3BP1	9425.74024	0.17039864	0.05494136	3.10146397	0.00192566
6985	GAA	2026.82485	-0.5490404	0.06502581	-8.4434218	3.08E-17
6991	GABARAPL1	1621.67634	0.47760137	0.06753594	7.07181022	1.53E-12
6992	GABARAPL2	1415.34903	-0.2389487	0.07222152	-3.3085518	0.0009378
6994	GABBR1	886.592819	0.22431542	0.09304016	2.41095275	0.01591091
6997	GABPB1	1031.95617	0.19084004	0.08090599	2.35878765	0.01833474
6998	GABPB1-AS1	338.73216	0.7873371	0.12965407	6.0725983	1.26E-09
7021	GACAT2	23.8497098	0.8737219	0.41098415	2.12592601	0.03350942
7025	GADD45A	8552.86103	3.08161777	0.09932218	31.0264829	2.37E-211
7053	GAL	106.781776	0.94912695	0.20185336	4.70206166	2.58E-06
7059	GALE	5220.25758	0.55109915	0.08020403	6.87121505	6.37E-12
7060	GALK1	706.794036	-0.8540893	0.1050618	-8.1293989	4.31E-16
7062	GALM	542.165207	-0.3444336	0.10353973	-3.3265845	0.00087917
7064	GALNT1	4405.1871	-0.3108565	0.07130768	-4.359369	1.30E-05
7065	GALNT10	2872.79318	-0.679369	0.0783481	-8.6711608	4.28E-18
7066	GALNT11	2144.05511	-0.6129893	0.06849937	-8.9488312	3.59E-19
7073	GALNT2	7621.18427	-0.8887909	0.0588923	-15.091802	1.83E-51
7074	GALNT3	1393.05365	0.36359094	0.08197523	4.4353754	9.19E-06
7076	GALNT5	795.202199	-0.9678172	0.08429577	-11.481207	1.64E-30
7087	GALT	824.280575	-0.2360253	0.09886541	-2.3873391	0.01697083
7088	GAMT	1520.35342	-0.3476542	0.07976268	-4.3586069	1.31E-05
7089	GAN	211.179496	0.4349094	0.14544864	2.99012349	0.00278865
7091	GANC	502.688632	-0.3332617	0.10352432	-3.2191634	0.00128565
7093	GAPDH	55214.1781	-0.2849995	0.05051295	-5.6421089	1.68E-08
7098	GAR1	1938.19772	0.69271442	0.0798287	8.6775111	4.05E-18
7101	GARNL3	151.424953	0.79912394	0.19496707	4.09876373	4.15E-05
7102	GARS	14917.4936	0.93127274	0.04698437	19.820905	1.97E-87
7103	GART	5813.05443	1.00581648	0.05546801	18.1332704	1.74E-73
7106	GAS2L1	1755.14506	-0.9374375	0.06976377	-13.437312	3.65E-41
7109	GAS2L3	1823.5094	1.29130888	0.11323712	11.4035825	4.01E-30
7110	GAS5	5986.41468	1.56795387	0.07913498	19.8136629	2.27E-87
7112	GAS6	12253.351	-0.9233174	0.06669249	-13.844398	1.38E-43
7120	GATA2	551.893649	0.35344223	0.09981384	3.54101428	0.00039859
7126	GATA6	2246.30288	0.58015959	0.0706133	8.2160097	2.10E-16
7129	GATAD2A	5554.68087	0.37545418	0.06038571	6.2175996	5.05E-10

7130	GATAD2B	1922.19888	0.33643691	0.08911402	3.77535334	0.00015978
7132	GATC	2732.05983	0.41493191	0.0647582	6.40740295	1.48E-10
7134	GATS	601.620127	-0.3482225	0.10293901	-3.3828046	0.0007175
7135	GATSL2	485.462388	-1.1295906	0.12087551	-9.3450743	9.18E-21
7136	GATSL3	224.037463	-0.390389	0.13734071	-2.8424853	0.00447633
7141	GBAS	1784.20301	-0.4409657	0.07185147	-6.1371843	8.40E-10
7146	GBP1	1432.26458	-0.5963275	0.07865802	-7.581267	3.42E-14
7148	GBP2	3528.72178	-1.5938076	0.06130217	-25.999206	5.06E-149
7149	GBP3	2787.80511	-0.2063336	0.07678701	-2.68709	0.00720775
7150	GBP4	676.786163	-1.9556176	0.11898517	-16.43581	1.06E-60
7157	GCA	603.387176	-1.3062014	0.09546395	-13.682666	1.29E-42
7159	GCC1	1336.87137	0.51316467	0.07549598	6.79724522	1.07E-11
7165	GCH1	454.298465	0.47389997	0.11032223	4.29559828	1.74E-05
7166	GCHFR	252.524752	-1.4347831	0.14809534	-9.6882398	3.38E-22
7168	GCKR	73.4023943	2.22206062	0.27368579	8.11902093	4.70E-16
7173	GCN1	9599.20916	0.19529567	0.05631168	3.46812022	0.00052411
7175	GCNT2	3231.16694	0.71748178	0.07151921	10.0320155	1.10E-23
7186	GDAP1	172.875342	1.38192613	0.1577732	8.75894088	1.97E-18
7188	GDAP2	882.65155	0.39859656	0.07881035	5.05766757	4.24E-07
7192	GDF11	339.653603	0.67752283	0.13455874	5.03514547	4.77E-07
7196	GDF5	364.992759	1.12773293	0.12776347	8.82672425	1.08E-18
7208	GDPD5	1279.95381	-0.5609544	0.08071868	-6.9494994	3.67E-12
7210	GEM	2087.4649	1.44591272	0.07151109	20.2194207	6.61E-91
7213	GEMIN5	2612.67927	0.96122273	0.06785755	14.1653031	1.50E-45
7215	GEMIN7	1321.84585	0.20215381	0.09413933	2.14738942	0.03176229
7217	GEMIN8P4	35.2348356	-0.8551034	0.33052379	-2.5871162	0.0096783
7219	GET4	3793.32344	0.5336262	0.09952641	5.36165405	8.25E-08
7220	GFAP	284.339756	-0.9891659	0.12977875	-7.6219401	2.50E-14
7222	GFI1	14.6570176	-1.3195911	0.55657287	-2.3709225	0.01774375
7224	GFM1	2372.61459	0.377593	0.06869479	5.49667594	3.87E-08
7226	GFOD1	134.467791	0.81150863	0.18235694	4.45011109	8.58E-06
7227	GFOD2	814.157326	0.22906362	0.08524568	2.68709932	0.00720755
7228	GFPT1	5706.3603	0.99645975	0.0589373	16.9071159	3.99E-64
7236	GGA1	1161.8412	0.21544391	0.07089733	3.03881553	0.0023751
7240	GGCT	1738.44823	0.34168338	0.08657618	3.94662128	7.93E-05
7242	GGH	1986.95282	-0.4373329	0.09191042	-4.7582519	1.95E-06
7245	GGNBP2	4733.41435	0.68187541	0.05841271	11.6734073	1.74E-31
7247	GGT1	520.610397	-0.9546201	0.11058661	-8.6323299	6.01E-18
7249	GGT5	773.329199	-0.7643065	0.08376294	-9.1246377	7.20E-20
7251	GGT7	190.309798	-0.5426888	0.16646581	-3.2600617	0.00111388
7258	GHDC	1634.0396	-0.221033	0.07585173	-2.9140138	0.00356814
7270	GIGYF1	3424.25814	-0.2806931	0.05731775	-4.8971405	9.72E-07
7291	GIPR	123.436169	-0.5652943	0.18195068	-3.1068547	0.00189089

7296	GJA3	450.048105	1.32029729	0.10713207	12.3240157	6.73E-35
7304	GJB3	835.850213	1.37722547	0.09110738	15.1165081	1.26E-51
7305	GJB4	30.3633748	4.13914737	0.55142239	7.50630999	6.08E-14
7318	GK5	363.35793	-0.8780605	0.11707354	-7.5000763	6.38E-14
7319	GKAP1	414.679553	0.42668601	0.10499316	4.06394087	4.83E-05
7323	GLB1	3912.37467	-0.3585467	0.0620777	-5.7757735	7.66E-09
7327	GLCCI1	193.489856	-0.8317481	0.17563944	-4.7355428	2.18E-06
7328	GLCE	2127.18453	-1.0603339	0.09252459	-11.460023	2.09E-30
7331	GLE1	2596.74852	-0.2415476	0.05907848	-4.0885891	4.34E-05
7335	GLI3	541.369251	0.6062133	0.10244863	5.91724175	3.27E-09
7336	GLI4	430.559379	-0.270587	0.11549741	-2.342797	0.01913979
7341	GLIPR2	350.227492	-0.8947139	0.12141857	-7.3688394	1.72E-13
7347	GLMN	658.067699	-0.4975544	0.09185734	-5.416599	6.07E-08
7349	GLO1	11551.7747	0.40064568	0.05789299	6.92045257	4.50E-12
7359	GLRX	877.736689	-0.194158	0.08603385	-2.2567623	0.02402293
7360	GLRX2	850.784952	0.67323946	0.09786257	6.87943789	6.01E-12
7361	GLRX3	3893.18691	0.3995421	0.08310135	4.8078896	1.53E-06
7367	GLT8D1	1857.58439	-0.64536	0.06819643	-9.4632526	2.99E-21
7388	GM2A	1907.23964	-0.6457406	0.07141644	-9.0419039	1.54E-19
7393	GMEB1	517.431472	0.52078299	0.10738395	4.8497284	1.24E-06
7394	GMEB2	1825.05761	0.16238974	0.06422191	2.52857213	0.01145276
7396	GMFG	73.1150314	-1.3372873	0.26583937	-5.0304335	4.89E-07
7404	GMPR2	2169.74511	-0.2469238	0.06762675	-3.651274	0.00026094
7408	GNA13	4368.14446	0.2084363	0.07890599	2.64157767	0.00825209
7413	GNAI2	11912.7058	-0.4071068	0.05105234	-7.9743029	1.53E-15
7424	GNB1	17164.5236	0.1495633	0.04641765	3.22212123	0.00127245
7426	GNB2	9623.24572	-0.2932503	0.05211542	-5.6269384	1.83E-08
7427	GNB2L1	36492.3146	0.23385862	0.05423502	4.31194831	1.62E-05
7431	GNE	2744.02502	-0.5890754	0.07290796	-8.0797124	6.49E-16
7434	GNG12	7030.65178	0.60915654	0.08384216	7.26551595	3.72E-13
7437	GNG2	83.7092313	-1.2635727	0.22904081	-5.5168018	3.45E-08
7440	GNG5	3474.03721	0.24218904	0.06231729	3.88638588	0.00010175
7441	GNG7	123.349213	-2.5497572	0.21081289	-12.094883	1.12E-33
7445	GNL1	2963.10655	0.2217331	0.06548405	3.38606285	0.00070903
7446	GNL2	3654.86843	1.10759136	0.06462943	17.1375686	7.78E-66
7447	GNL3	5808.40689	0.58478886	0.05688895	10.2794797	8.72E-25
7448	GNL3L	1883.17438	0.2565999	0.07640925	3.35823069	0.00078443
7451	GNPAT	2354.28931	-0.5342223	0.06228357	-8.5772599	9.72E-18
7454	GNPNAT1	3600.71045	0.65720045	0.06560047	10.0182273	1.27E-23
7457	GNRH1	79.7039279	-1.0255185	0.23867607	-4.296696	1.73E-05
7461	GNS	9000.18296	-0.1915803	0.05996927	-3.194641	0.00140005
7467	GOLGA2P5	163.900574	-1.3557643	0.19302402	-7.0238115	2.16E-12
7471	GOLGA3	3645.71043	-0.3288635	0.05771541	-5.6980197	1.21E-08

7479	GOLGA6L10	31.9097914	-1.0151024	0.36733905	-2.7633938	0.00572037
7509	GOLIM4	1604.72102	-0.9559269	0.08852052	-10.79893	3.48E-27
7510	GOLM1	8860.03573	-0.5436683	0.05185573	-10.484247	1.02E-25
7515	GON4L	1245.80051	0.32888751	0.07709104	4.26622216	1.99E-05
7516	GOPC	2062.92806	0.52737323	0.06983703	7.55148455	4.30E-14
7518	GORASP1	1447.82497	0.28822364	0.06768154	4.25852682	2.06E-05
7519	GORASP2	5349.96822	0.13495006	0.06808291	1.98214287	0.04746325
7520	GOSR1	1923.0512	0.34302511	0.06609324	5.1900179	2.10E-07
7524	GOT2	4898.7754	0.22685557	0.05262373	4.31089861	1.63E-05
7532	GPAA1	5449.76198	-0.1648435	0.0684567	-2.4079967	0.01604033
7533	GPALPP1	526.952366	0.29016294	0.12216597	2.37515361	0.01754165
7534	GPAM	446.191242	-0.3401114	0.11834284	-2.8739503	0.00405373
7535	GPANK1	1000.00514	0.49024136	0.07718198	6.35175926	2.13E-10
7536	GPAT2	1636.37922	-0.206719	0.08461888	-2.4429423	0.01456807
7538	GPAT4	3330.16121	-0.4954522	0.06445252	-7.687088	1.51E-14
7541	GPATCH2	716.997854	0.58103702	0.10456337	5.55679313	2.75E-08
7544	GPATCH4	2238.21292	0.71455123	0.06480446	11.0262659	2.85E-28
7547	GPBP1	4123.54396	0.30610407	0.05638545	5.42877751	5.67E-08
7548	GPBP1L1	3320.04306	0.48727447	0.06479618	7.52011116	5.47E-14
7561	GPD1L	994.604017	-0.3781261	0.07762664	-4.8710868	1.11E-06
7563	GPER1	358.412869	-1.1238546	0.11912996	-9.4338539	3.95E-21
7567	GPI	13687.8929	-0.3920965	0.04973258	-7.8840973	3.17E-15
7574	GPN2	809.304961	0.58757167	0.10117858	5.80727312	6.35E-09
7577	GPR1	543.25074	0.19456193	0.09588087	2.02920485	0.04243743
7587	GPR137B	158.79602	-0.9579127	0.17493376	-5.4758592	4.35E-08
7593	GPR146	90.7926932	-0.8133453	0.21625999	-3.7609603	0.00016926
7606	GPR160	125.471884	0.42737614	0.18546138	2.30439425	0.02120052
7626	GPR3	302.14139	1.57120119	0.13185494	11.9161347	9.75E-33
7631	GPR35	156.326357	0.7151318	0.16929807	4.22409897	2.40E-05
7634	GPR39	379.475078	-1.0299598	0.1152677	-8.9353727	4.06E-19
7639	GPR55	53.1604243	-2.2946898	0.3301546	-6.9503495	3.64E-12
7643	GPR63	75.1503302	1.63233518	0.26046098	6.26710059	3.68E-10
7645	GPR68	1478.13653	-0.2081357	0.0725964	-2.8670247	0.00414351
7657	GPRASP1	68.3252563	0.77127638	0.25392787	3.03738367	0.00238641
7661	GPRC5C	570.687596	-0.8404828	0.09374881	-8.9652637	3.10E-19
7664	GPRIN1	606.629886	-0.2207366	0.10266956	-2.1499712	0.03155749
7665	GPRIN2	90.6568159	-1.9305622	0.23754689	-8.1270785	4.40E-16
7667	GPS1	2956.25449	-0.3580898	0.07068732	-5.065828	4.07E-07
7669	GPSM1	1443.04238	0.85526467	0.07006428	12.2068567	2.86E-34
7670	GPSM2	2790.82143	-0.3694725	0.05718684	-6.4607964	1.04E-10
7673	GPT2	1566.78093	0.79127946	0.07053748	11.2178577	3.33E-29
7674	GPX1	6918.94681	-0.4861829	0.10994182	-4.4221835	9.77E-06
7677	GPX4	13345.7924	-0.4122498	0.0919628	-4.4827888	7.37E-06

7680	GPX7	86.9000695	-0.6618586	0.24040428	-2.7531065	0.00590327
7683	GRAMD1B	67.9758376	1.8697603	0.27724478	6.74407754	1.54E-11
7684	GRAMD1C	221.315526	-0.9476579	0.14756985	-6.4217582	1.35E-10
7687	GRAMD4	1113.04218	-0.41944	0.07760536	-5.4047813	6.49E-08
7692	GRB10	4867.30452	0.38031221	0.05226222	7.2770011	3.41E-13
7700	GRHL1	41.6215239	1.27167892	0.34079827	3.73147122	0.00019036
7703	GRHPR	2479.82446	-0.5952907	0.08080066	-7.3673995	1.74E-13
7718	GRIK4	508.198204	-1.2831772	0.11064344	-11.597408	4.25E-31
7727	GRINA	10671.9625	0.5851848	0.05718101	10.2339014	1.40E-24
7730	GRIPAP1	1974.64882	-0.2078611	0.07087393	-2.9328287	0.00335889
7733	GRK5	1031.60605	-0.5992608	0.0815193	-7.351153	1.97E-13
7741	GRM5-AS1	2.31116425	3.63813511	1.63981697	2.21862267	0.02651241
7751	GRPEL2	987.681554	0.65526342	0.08208482	7.9827601	1.43E-15
7756	GRWD1	2393.428	0.28811429	0.0796981	3.61507098	0.00030027
7759	GS1-124K5.1	571.683061	0.47587699	0.09707398	4.90220929	9.48E-07
7761	GS1-259H13.	105.257872	0.42221497	0.19586078	2.15568924	0.03110794
7765	GSAP	417.598459	-0.6504771	0.10821355	-6.0110502	1.84E-09
7770	GSDMC	58.2803917	-0.9570061	0.27055178	-3.5372381	0.00040433
7771	GSDMD	1653.53961	-1.1134756	0.1122826	-9.916724	3.52E-23
7772	GSE1	1362.7284	0.8182788	0.07734315	10.5798485	3.70E-26
7778	GSK3B	1202.26214	-0.3708888	0.07768463	-4.7742875	1.80E-06
7779	GSKIP	1054.99526	0.15597315	0.07368886	2.11664485	0.03429
7780	GSN	12112.8472	-1.3169537	0.06155045	-21.396331	1.45E-101
7782	GSPT1	8437.58804	0.22978129	0.05625719	4.08447902	4.42E-05
7783	GSPT2	457.191892	0.58088762	0.10023011	5.79554009	6.81E-09
7793	GSTK1	2994.48247	-0.5873587	0.08172724	-7.1868165	6.63E-13
7794	GSTM1	466.199548	-0.8084186	0.11918505	-6.7828864	1.18E-11
7795	GSTM2	302.146872	-1.250236	0.12941644	-9.6605655	4.43E-22
7798	GSTM4	1158.27704	-1.4831749	0.08538873	-17.369681	1.40E-67
7816	GTF2E1	470.18628	0.59555441	0.10796338	5.51626297	3.46E-08
7819	GTF2F2	2037.48332	0.59261234	0.06282835	9.43224361	4.01E-21
7820	GTF2H1	4305.0004	0.39769223	0.05895823	6.74532153	1.53E-11
7825	GTF2H3	2407.75101	0.45664979	0.0642973	7.10216163	1.23E-12
7837	GTF3C1	3461.15778	-0.394128	0.07455956	-5.2860827	1.25E-07
7838	GTF3C2	1897.99886	-0.1831653	0.07324952	-2.500566	0.0123995
7840	GTF3C3	1043.08652	-0.2429023	0.08780448	-2.7664001	0.0056679
7841	GTF3C4	1571.17667	0.6814452	0.08012864	8.50438986	1.83E-17
7842	GTF3C5	2519.58275	-0.2745104	0.06646142	-4.1303718	3.62E-05
7844	GTPBP1	1637.89052	0.64176534	0.06447865	9.95314489	2.44E-23
7845	GTPBP10	1954.48793	1.4169587	0.07112331	19.9225627	2.59E-88
7846	GTPBP2	5821.2711	1.55587861	0.05445672	28.5709223	1.54E-179
7848	GTPBP4	6754.36004	0.82558015	0.06663656	12.3892964	2.99E-35
7852	GTSE1	1830.50556	-0.4618533	0.0820066	-5.6319041	1.78E-08

7861	GUCD1	2520.30753	-0.2093977	0.06371466	-3.2864918	0.00101444
7871	GUF1	1507.10294	0.16199282	0.08110884	1.99722761	0.04580046
7872	GUK1	4808.94254	-0.3151097	0.0887751	-3.5495279	0.00038592
7874	GUSB	3055.3513	-0.2487239	0.07049661	-3.5281683	0.00041845
7875	GUSBP1	95.0292825	-0.551898	0.20398151	-2.7056275	0.00681755
7886	GXYLT2	261.324138	-1.7056382	0.13680306	-12.467837	1.12E-35
7888	GYG2	309.20124	-2.483546	0.13898152	-17.869613	2.03E-71
7890	GYLTL1B	11.3857402	1.4114724	0.6515598	2.16629754	0.03028846
7897	GZF1	1207.39568	0.57707365	0.08186744	7.04887879	1.80E-12
7902	GZMM	10.2692689	1.95084505	0.67280959	2.89955002	0.00373699
7904	H1F0	14164.6724	0.83006557	0.0505222	16.4297192	1.17E-60
7907	H1FX	3739.00188	-0.589671	0.07461399	-7.9029546	2.72E-15
7908	H1FX-AS1	74.1693321	-0.6327198	0.24846735	-2.5464907	0.01088121
7913	H2AFV	6793.16564	-0.5472656	0.05686927	-9.6232225	6.38E-22
7914	H2AFX	5963.41872	-0.7414704	0.08453531	-8.7711315	1.77E-18
7925	H6PD	3205.52307	-0.6364386	0.0741505	-8.5830654	9.24E-18
7929	HACD1	1768.11018	-0.2043504	0.08167903	-2.5018709	0.0123539
7930	HACD2	263.565578	-0.5545719	0.22614183	-2.4523189	0.01419388
7931	HACD3	5555.93055	-0.8188822	0.05602032	-14.617591	2.17E-48
7935	HADH	1933.49788	-0.7197411	0.06331853	-11.36699	6.11E-30
7938	HAGH	1636.09142	-0.3227053	0.08909637	-3.6219803	0.00029236
7940	HAGLR	582.718847	-0.5085404	0.11485946	-4.4275014	9.53E-06
7957	HARBI1	213.60403	0.97587435	0.15120281	6.45407533	1.09E-10
7958	HARS	3564.34698	-0.2480581	0.0716153	-3.4637581	0.00053269
7965	HAUS1	1464.44011	-0.2814475	0.07565178	-3.7203031	0.00019898
7966	HAUS2	1222.39286	0.30949794	0.07253868	4.26666081	1.98E-05
7968	HAUS4	1352.71314	-0.4263591	0.09292917	-4.5880009	4.48E-06
7969	HAUS5	814.202341	-0.5651253	0.08361673	-6.7585196	1.39E-11
7976	HAX1	2301.75291	0.41707661	0.06565949	6.35211427	2.12E-10
7991	HCAR1	158.294356	-0.8866505	0.16901707	-5.2459229	1.56E-07
7996	HCFC1	3529.01407	-0.5148882	0.07423348	-6.9360645	4.03E-12
7998	HCFC1R1	1979.95957	-0.6200079	0.07625072	-8.1311747	4.25E-16
7999	HCFC2	490.065023	0.43505855	0.10906891	3.98884122	6.64E-05
8002	HCG17	14.6400679	1.88715653	0.54285572	3.47635011	0.00050829
8003	HCG18	2781.81626	1.25885649	0.07066732	17.8138423	5.52E-71
8010	HCG4	14.1131407	1.40108402	0.58133019	2.41013464	0.01594663
8019	HCN4	18.9081754	3.58011098	0.64407844	5.55850151	2.72E-08
8029	HDAC2	7224.44313	0.12238011	0.05326415	2.29760752	0.02158414
8030	HDAC3	3589.9818	-0.6163432	0.07001693	-8.802774	1.33E-18
8032	HDAC5	963.646606	-0.7397258	0.08753374	-8.4507505	2.89E-17
8033	HDAC6	2272.6451	-0.6685635	0.06491291	-10.299392	7.09E-25
8034	HDAC7	2327.44117	-0.61532	0.0606232	-10.14991	3.32E-24
8035	HDAC8	918.065852	-0.5094607	0.07832395	-6.5045326	7.79E-11

8036	HDAC9	5151.60574	1.27202693	0.07505623	16.9476531	2.00E-64
8039	HDDC3	312.639846	-0.4785532	0.13825164	-3.4614647	0.00053724
8040	HDGF	24868.0521	-0.3458325	0.06429927	-5.3784822	7.51E-08
8042	HDGFRP2	2980.79774	-0.8401853	0.06490251	-12.945343	2.50E-38
8043	HDGFRP3	1015.8247	-0.2187396	0.07869972	-2.779421	0.00544559
8049	HEATR1	1866.92172	0.20570842	0.06654866	3.09109792	0.00199418
8050	HEATR3	686.878699	-0.5355007	0.0965643	-5.5455348	2.93E-08
8053	HEATR5B	1182.73565	0.65327806	0.09108714	7.17201205	7.39E-13
8056	HEBP1	2314.35788	-0.8032748	0.07794353	-10.305856	6.63E-25
8065	HECW2	249.388443	3.18831007	0.18720768	17.0308726	4.85E-65
8067	HEIH	739.523718	0.75506625	0.0945609	7.98497304	1.41E-15
8069	HELLS	1551.14297	-0.5245321	0.06952734	-7.5442561	4.55E-14
8072	HELZ	1084.54828	0.34073088	0.10627737	3.20605289	0.00134569
8073	HELZ2	1818.88833	-0.8470313	0.09126319	-9.2811929	1.68E-20
8085	HERC2P2	773.794156	-0.7196398	0.09915717	-7.2575672	3.94E-13
8091	HERC4	4188.22452	0.31146178	0.05258033	5.92354139	3.15E-09
8094	HERPUD1	1959.69304	0.73569437	0.06923978	10.6253135	2.27E-26
8099	HES4	394.890633	0.76749084	0.17963398	4.27252587	1.93E-05
8101	HES6	312.175376	-0.586792	0.14598098	-4.0196466	5.83E-05
8102	HES7	60.6806111	2.78589689	0.30721609	9.06819983	1.21E-19
8106	HEXB	8057.31755	-0.1561657	0.05419352	-2.881631	0.00395623
8107	HEXDC	740.404357	-0.6302077	0.09426003	-6.6858424	2.30E-11
8108	HEXIM1	5316.69443	-1.1628793	0.06067763	-19.164878	7.27E-82
8109	HEXIM2	319.328023	-1.2519421	0.15807105	-7.9201229	2.37E-15
8110	HEY1	32.1681201	2.71674464	0.41255223	6.58521382	4.54E-11
8113	HFE	582.418259	-0.5946668	0.10364927	-5.7372982	9.62E-09
8121	HGS	6711.8076	0.39971632	0.05491095	7.27935589	3.35E-13
8122	HGSNAT	2632.66371	-0.3675347	0.08738211	-4.2060637	2.60E-05
8126	HHIP	49.0063851	-1.8218067	0.30388296	-5.9950934	2.03E-09
8127	HHIP-AS1	96.6935873	-1.131507	0.21181478	-5.3419645	9.19E-08
8132	HHLA3	50.3973673	1.23488976	0.29732201	4.15337484	3.28E-05
8133	HIAT1	4000.0477	-0.5601183	0.06224303	-8.9988924	2.28E-19
8134	HIATL1	3579.72651	-0.5767384	0.06104708	-9.4474364	3.47E-21
8137	HIBCH	765.464913	-0.629156	0.095452	-6.5913343	4.36E-11
8144	HIF1A-AS2	118.395173	0.70590827	0.22051171	3.20122798	0.00136843
8145	HIF1AN	2451.87658	-0.1699744	0.06656317	-2.5535802	0.01066217
8147	HIGD1A	3148.48544	0.14757838	0.05912209	2.49616302	0.01255449
8150	HIGD2A	2568.6026	-0.1543772	0.06089457	-2.5351549	0.01123976
8154	HINFP	1332.64647	0.48054488	0.07097079	6.77102312	1.28E-11
8159	HIP1R	3347.04486	0.66090093	0.05934431	11.1367196	8.31E-29
8160	HIPK1	2186.52436	0.21083919	0.08324524	2.53274764	0.01131724
8166	HIRIP3	725.439928	-0.5335784	0.11229563	-4.7515506	2.02E-06
8240	HIVEP1	1407.89593	1.14473844	0.09550187	11.9865558	4.18E-33

8241	HIVEP2	1645.41298	0.38091831	0.11934445	3.1917555	0.00141411
8243	HJURP	2494.58695	-0.3776345	0.06574507	-5.743921	9.25E-09
8244	HK1	12499.9777	-0.5842488	0.05027782	-11.620408	3.25E-31
8245	HK2	2561.33294	0.19045269	0.06003638	3.17228776	0.00151243
8248	HKR1	649.234475	0.28553675	0.10082619	2.83196993	0.00462622
8252	HLA-DMA	1420.93816	-0.4407921	0.08547406	-5.1570274	2.51E-07
8253	HLA-DMB	712.582213	-0.722514	0.09588158	-7.5354827	4.87E-14
8255	HLA-DOB	60.2951525	0.92119801	0.28765019	3.20249405	0.00136243
8256	HLA-DPA1	3202.32936	-0.3360818	0.06070776	-5.5360598	3.09E-08
8257	HLA-DPB1	1781.44387	-0.8278358	0.11591275	-7.1418877	9.21E-13
8261	HLA-DQB1	224.544327	-0.8977963	0.14338167	-6.2615838	3.81E-10
8262	HLA-DQB2	64.9535991	-0.8771625	0.25772231	-3.4035178	0.00066524
8263	HLA-DRA	6089.63294	-0.4544211	0.06109643	-7.4377678	1.02E-13
8269	HLA-E	13141.521	0.16839301	0.0469491	3.58671445	0.00033487
8275	HLA-L	259.689211	0.48524376	0.13354143	3.63365707	0.00027943
8277	HLF	20.2552247	1.18300854	0.43763942	2.70315807	0.00686841
8280	HLX	214.987604	1.71598677	0.15876199	10.8085494	3.14E-27
8285	HMBS	2532.5731	-0.2931076	0.08920304	-3.2858481	0.00101676
8286	HMCES	1580.21766	-0.5848112	0.08762615	-6.6739346	2.49E-11
8290	HMG20B	3830.92269	-0.8721701	0.07653498	-11.395707	4.39E-30
8291	HMGA1	56980.5653	1.03680048	0.06598026	15.713799	1.22E-55
8295	HMGB2	6689.04432	-0.8063312	0.0575191	-14.018494	1.20E-44
8296	HMGB3	1267.95329	-0.5597982	0.09118412	-6.1392066	8.29E-10
8302	HMGCS1	1570.38609	-0.6566803	0.07672186	-8.5592329	1.14E-17
8304	HMGN1	2985.74864	0.18202601	0.08744904	2.0815094	0.0373873
8305	HMGN2	1836.36295	0.21356557	0.06386714	3.34390377	0.00082608
8307	HMGN3	1153.27704	-0.552082	0.10132585	-5.44858	5.08E-08
8309	HMGN4	5423.46206	0.56138217	0.06244013	8.99072752	2.46E-19
8312	HMGXB4	1167.24667	-0.3977938	0.08002777	-4.9706974	6.67E-07
8324	HN1	7374.30178	0.33604403	0.07766924	4.32660365	1.51E-05
8325	HN1L	5640.55096	0.16941903	0.05825461	2.90825125	0.00363456
8333	HNMT	1331.4199	-1.2077002	0.07424772	-16.265822	1.73E-59
8342	HNRNPAB	9983.54867	-0.4122757	0.05581589	-7.3863513	1.51E-13
8343	HNRNPC	12232.8194	0.37500503	0.06819268	5.49919798	3.82E-08
8353	HNRNPH3	8650.31623	0.36397596	0.06665763	5.46037984	4.75E-08
8354	HNRNPK	19671.4052	0.40042096	0.06223656	6.43385468	1.24E-10
8357	HNRNPLL	1535.39907	0.23024211	0.06622744	3.47653654	0.00050794
8359	HNRNPR	8166.7516	0.16399444	0.05050042	3.24738776	0.0011647
8360	HNRNPU	27411.0991	0.36493104	0.04798018	7.6058708	2.83E-14
8366	HOMER1	402.495927	0.47773553	0.12516641	3.81680305	0.00013519
8389	HOXA13	18.4446928	-1.3722559	0.47157771	-2.9099252	0.00361515
8407	HOXB8	37.3704771	1.47518496	0.38272722	3.854403	0.00011601
8408	HOXB9	770.001488	2.38836221	0.09803642	24.3619886	4.33E-131

8425	HOXD11	3.92646285	-5.4005157	1.48606998	-3.6340925	0.00027896
8437	HPCAL1	11208.9405	0.33829386	0.05605006	6.03556651	1.58E-09
8451	HPS6	1284.83252	-0.4659752	0.08963784	-5.1984209	2.01E-07
8452	HPSE	993.941309	-1.5809933	0.09155165	-17.268867	8.07E-67
8463	HRAT17	11.8999826	3.26269703	0.76689798	4.25440819	2.10E-05
8466	HRAT92	21.8308245	-0.8565359	0.41830456	-2.0476371	0.04059557
8468	HRCT1	13.8167979	-1.628359	0.58885968	-2.765275	0.00568748
8470	HRH1	2995.86106	1.41872647	0.06988012	20.3022915	1.23E-91
8476	HRSP12	639.141376	-0.2014804	0.09919423	-2.0311709	0.04223766
8483	HS3ST3B1	824.398669	-0.8862042	0.0843581	-10.505265	8.17E-26
8492	HSBP1L1	949.018411	0.33168562	0.12890172	2.57316681	0.01007726
8493	HSCB	297.394958	0.62566078	0.11982532	5.2214406	1.78E-07
8494	HSD11B1	77.6889489	-0.8933455	0.23872555	-3.7421446	0.00018246
8495	HSD11B1L	212.459617	-0.768876	0.15788702	-4.8697856	1.12E-06
8499	HSD17B11	3583.79067	-0.7754272	0.05668281	-13.680114	1.33E-42
8505	HSD17B4	3220.50074	-0.7149918	0.06154379	-11.617612	3.35E-31
8512	HSD3B7	1757.34143	-0.3873228	0.07818696	-4.9538028	7.28E-07
8515	HSDL1	1401.47379	0.21289203	0.06936129	3.06932039	0.00214546
8517	HSF1	4786.68909	0.13703089	0.05301157	2.58492436	0.00974004
8518	HSF2	1570.86944	0.54656975	0.0706609	7.7351084	1.03E-14
8520	HSF4	313.958618	-0.5748545	0.12372455	-4.6462443	3.38E-06
8529	HSP90AB1	58292.8641	0.36545803	0.06191021	5.90303369	3.57E-09
8535	HSPA13	2485.74639	0.18430901	0.08290559	2.22311913	0.02620777
8538	HSPA1B	1407.21154	-1.4036958	0.09840068	-14.265102	3.61E-46
8541	HSPA4	13320.3266	0.59737527	0.05201549	11.4845657	1.58E-30
8543	HSPA5	32269.5987	0.4742893	0.06168982	7.68829101	1.49E-14
8547	HSPA9	23203.368	0.89955768	0.04903342	18.3458091	3.57E-75
8555	HSPB8	1710.28818	2.06639035	0.07281671	28.3779677	3.78E-177
8558	HSPBP1	1691.58443	-0.3235768	0.09151022	-3.5359633	0.00040629
8560	HSPE1	2582.4085	-0.9402598	0.09045319	-10.394988	2.61E-25
8562	HSPG2	5526.87145	-1.0573917	0.08482318	-12.465835	1.15E-35
8571	HTR1D	283.31287	-0.5140543	0.13920732	-3.6927247	0.00022186
8589	HTRA1	997.793377	-1.7756359	0.08284362	-21.433587	6.50E-102
8590	HTRA2	1714.71709	0.48239208	0.09211569	5.23680699	1.63E-07
8591	HTRA3	180.648395	-1.167597	0.16997833	-6.8690935	6.46E-12
8593	HTT	2754.45606	-0.5207397	0.07810559	-6.6671248	2.61E-11
8598	HUS1B	6.33826146	1.72860279	0.81295347	2.1263244	0.03347626
8599	HUWE1	21219.1616	0.36000268	0.07360832	4.89078762	1.00E-06
8600	HVCN1	146.049064	-1.3293218	0.18872223	-7.0438005	1.87E-12
8602	HYAL2	1270.28155	-0.4606831	0.07072895	-6.5133595	7.35E-11
8610	HYLS1	1509.64515	-0.3149638	0.09971774	-3.1585531	0.00158554
8612	HYOU1	11164.2223	0.26059126	0.05242599	4.97064991	6.67E-07
8617	IARS	16645.0085	0.90285108	0.0507809	17.7793427	1.02E-70

8622	IBTK	3215.6043	0.29977339	0.07813959	3.83638298	0.00012486
8629	ICAM5	149.997656	0.89215284	0.17555492	5.08190173	3.74E-07
8636	ICT1	1124.17744	0.61926094	0.08313687	7.44869175	9.43E-14
8637	ID1	2259.54434	-2.2252624	0.08009634	-27.782322	7.09E-170
8638	ID2	193.531729	-1.8727128	0.16255353	-11.520592	1.04E-30
8640	ID3	865.879558	-0.5254472	0.11565499	-4.5432295	5.54E-06
8643	IDH1	2155.36384	-1.2077893	0.0669713	-18.03443	1.05E-72
8644	IDH1-AS1	12.5988394	-2.0320026	0.65683718	-3.0936169	0.00197733
8645	IDH2	875.51606	-0.9537636	0.11408615	-8.3600299	6.27E-17
8647	IDH3B	3081.93091	-0.2882341	0.07774811	-3.7072811	0.0002095
8648	IDH3G	1520.35815	-0.5670312	0.07785906	-7.2827897	3.27E-13
8652	IDNK	297.806632	0.59498748	0.12774617	4.65757596	3.20E-06
8657	IER2	2860.92313	0.19495185	0.07573966	2.57397321	0.01005381
8658	IER3	53904.2695	1.01691358	0.04734341	21.4795156	2.42E-102
8661	IER5L	1156.5225	-1.6467659	0.07734589	-21.290931	1.38E-100
8668	IFI30	2087.97237	0.29071318	0.08134504	3.57382777	0.0003518
8674	IFIT1	2491.62634	-0.6312761	0.0616486	-10.23991	1.31E-24
8679	IFITM1	648.860787	-1.6589609	0.10856864	-15.280295	1.03E-52
8681	IFITM2	664.588833	-1.0438941	0.1073486	-9.7243386	2.37E-22
8682	IFITM3	4382.74915	-0.8626574	0.06177046	-13.965533	2.53E-44
8699	IFNAR1	3041.9936	0.45976426	0.08329561	5.5196696	3.40E-08
8714	IFRD1	3133.25241	0.26705183	0.06396384	4.17504352	2.98E-05
8715	IFRD2	5923.62783	0.27554687	0.08590874	3.20743696	0.00133923
8716	IFT122	315.186276	-0.5089028	0.12800907	-3.9755215	7.02E-05
8717	IFT140	467.743404	-0.3233474	0.11351928	-2.848392	0.00439408
8724	IFT52	1855.60301	0.18053255	0.07162264	2.52060748	0.01171525
8725	IFT57	1637.68165	0.29617082	0.07750748	3.82119032	0.00013281
8728	IFT80	805.621405	-0.5355568	0.0882841	-6.0662875	1.31E-09
8736	IGF1R	1155.37461	-0.8106926	0.07754155	-10.454944	1.39E-25
8740	IGF2BP2	3386.26999	0.51587265	0.07629596	6.76146724	1.37E-11
8745	IGFBP1	274.191794	0.49820375	0.14152429	3.52027034	0.00043111
8748	IGFBP4	56561.8623	-1.506086	0.08228725	-18.302786	7.86E-75
8750	IGFBP6	2785.65588	-0.7617165	0.09092398	-8.3775093	5.41E-17
8751	IGFBP7	11894.5383	-0.6625838	0.07860383	-8.429408	3.47E-17
8758	IGFLR1	98.5967043	-1.1109911	0.22593846	-4.9172287	8.78E-07
8773	IGSF3	262.29909	0.39729838	0.13938822	2.85030103	0.00436779
8780	IK	3077.66377	-0.4893218	0.05918163	-8.2681376	1.36E-16
8784	IKBKE	2419.16381	-0.5179469	0.06639691	-7.8007687	6.15E-15
8787	IKZF2	117.620953	-0.7210758	0.19296935	-3.7367372	0.00018642
8792	IL10RA	6.28182626	3.50879272	1.13696181	3.08611308	0.00202792
8795	IL11	1144.61576	4.57112299	0.1179559	38.7528129	0
8796	IL11RA	127.573961	-0.8489805	0.18434805	-4.6053134	4.12E-06
8797	IL12A	188.333164	1.83592614	0.16396584	11.1970039	4.22E-29

8811	IL17D	26.2879792	1.01348627	0.41000428	2.47189191	0.01344001
8813	IL17RA	820.688345	-0.5078771	0.08268299	-6.1424624	8.13E-10
8815	IL17RC	1308.53784	-0.5561947	0.09587699	-5.8011287	6.59E-09
8816	IL17RD	1048.47905	-0.7950926	0.092289	-8.6152472	6.98E-18
8817	IL17RE	262.255216	-0.7688107	0.14232363	-5.4018483	6.60E-08
8824	IL1A	16200.0742	2.85567303	0.06147467	46.4528382	0
8827	IL1R1	1110.06648	-0.2613035	0.09649805	-2.7078628	0.0067718
8830	IL1RAPL1	406.75231	1.3542097	0.12474518	10.8558079	1.87E-27
8833	IL1RL2	109.307662	2.26556407	0.22392663	10.117439	4.62E-24
8834	IL1RN	174.164792	2.77253496	0.19521083	14.2027722	8.81E-46
8838	IL20RB	115.139533	1.99553748	0.21587375	9.24400233	2.37E-20
8848	IL24	9821.12074	5.22439383	0.06233861	83.8067057	0
8852	IL27RA	628.813429	-0.4733444	0.0968952	-4.8851171	1.03E-06
8855	IL2RG	47.4809547	2.23981258	0.33808118	6.62507323	3.47E-11
8859	IL32	2268.86262	0.63635652	0.09887623	6.43588962	1.23E-10
8873	IL6	7406.09757	1.68785978	0.05933008	28.4486334	5.07E-178
8875	IL6ST	16139.9644	0.62749062	0.08870726	7.07372307	1.51E-12
8877	IL7R	1277.42177	1.16004416	0.07930415	14.6277861	1.87E-48
8881	ILDR2	1057.06552	-0.5146097	0.09980107	-5.1563545	2.52E-07
8882	ILF2	8435.50144	0.15590569	0.06322001	2.46608133	0.01366003
8885	ILK	7241.72909	-0.1223582	0.06212801	-1.9694537	0.04890102
8887	ILVBL	2378.96658	-0.2389039	0.08492312	-2.8131788	0.00490544
8891	IMP3	1542.64035	-1.3477687	0.11831761	-11.391108	4.63E-30
8892	IMP4	1829.89148	0.45204965	0.0887531	5.09333902	3.52E-07
8894	IMPA2	1358.23386	-1.0673226	0.10515002	-10.150474	3.30E-24
8898	IMPDH2	8616.12211	0.18285264	0.06459791	2.83062766	0.00464568
8904	INAFM2	504.161937	0.25906499	0.1089557	2.37770945	0.01742055
8906	INCENP	2252.60508	-0.7130607	0.07171185	-9.9434155	2.69E-23
8909	INF2	5235.5908	-0.5588952	0.05312845	-10.519695	7.01E-26
8911	ING2	578.269475	-0.3884481	0.09693261	-4.007404	6.14E-05
8913	ING4	461.948603	-0.6669018	0.10553457	-6.3192736	2.63E-10
8916	INHA	17.6986916	-1.3849764	0.49804199	-2.7808427	0.0054218
8921	INHBE	9.04825137	5.67623613	1.32524342	4.28316494	1.84E-05
8929	INO80D	459.400331	0.31022407	0.11461991	2.70654626	0.00679871
8932	INPP4A	1232.75471	-0.2741263	0.08856435	-3.0952213	0.00196666
8935	INPP5B	288.127574	0.56262395	0.15129937	3.71861404	0.00020032
8937	INPP5E	1237.9232	-0.4988903	0.07051164	-7.0752899	1.49E-12
8939	INPP5J	66.2673369	-1.2917344	0.25269298	-5.1118728	3.19E-07
8941	INPPL1	5850.3299	-0.3132707	0.07847914	-3.9917708	6.56E-05
8945	INSIG1	2515.32191	-0.9034418	0.06318334	-14.298734	2.23E-46
8953	INSR	1586.39013	-0.6351279	0.06685921	-9.4994824	2.11E-21
8955	INTS1	3243.19298	-0.786107	0.0707085	-11.117574	1.03E-28
8957	INTS12	664.687441	0.52988334	0.08602275	6.15980442	7.28E-10

8959	INTS3	3012.16479	-0.1832572	0.07243605	-2.5299167	0.01140896
8960	INTS4	709.279787	-0.3734408	0.09146046	-4.0830848	4.44E-05
8962	INTS5	1128.57809	-0.2917555	0.07530562	-3.8742858	0.00010694
8963	INTS6	1001.65472	0.66113332	0.0965447	6.84795081	7.49E-12
8965	INTS7	2133.26277	0.62959661	0.07242114	8.69354689	3.51E-18
8966	INTS8	1136.51166	-0.3035049	0.08533353	-3.5566893	0.00037556
8969	INVS	438.745409	-0.3633988	0.10946359	-3.3198142	0.00090077
8974	IPMK	1669.42505	0.22808973	0.09623658	2.3700939	0.01778357
8978	IPO4	2860.0341	0.40703557	0.0601126	6.77121856	1.28E-11
8979	IPO5	10675.8352	0.52912496	0.04902407	10.7931673	3.71E-27
8980	IPO5P1	327.379772	0.62196795	0.11889176	5.23137963	1.68E-07
8981	IPO7	10097.9312	0.35505489	0.08372141	4.24090908	2.23E-05
8983	IPO9	6385.59733	0.28973814	0.06854289	4.22710704	2.37E-05
8986	IPPK	853.356082	0.51782726	0.0949922	5.45126113	5.00E-08
8990	IQCB1	586.731302	0.44053139	0.10187774	4.32411841	1.53E-05
8992	IQCD	143.058183	-1.6149609	0.18571332	-8.6959885	3.44E-18
8993	IQCE	1157.69371	-0.7880983	0.07517548	-10.483449	1.03E-25
9012	IQSEC2	1381.21092	-0.6505302	0.08993748	-7.2331386	4.72E-13
9016	IRAK1	8857.23506	0.30115447	0.04874804	6.17777607	6.50E-10
9018	IRAK2	2400.86239	1.28351561	0.06588667	19.4806569	1.60E-84
9019	IRAK3	460.024795	-0.8131414	0.10978006	-7.4070046	1.29E-13
9020	IRAK4	436.080442	0.25589033	0.106433	2.40423867	0.01620619
9022	IRF1	3196.87477	0.45332215	0.06496596	6.9778409	3.00E-12
9026	IRF2BPL	1840.92178	-0.8323868	0.07125404	-11.68196	1.58E-31
9039	IRS2	2250.83755	0.55517126	0.06198763	8.95616224	3.36E-19
9052	ISG20L2	1196.22674	0.87415224	0.07626214	11.4624659	2.04E-30
9064	IST1	3873.58837	0.42629882	0.06707398	6.35565084	2.08E-10
9068	ISYNA1	579.426044	-1.2203655	0.10128237	-12.049141	1.96E-33
9070	ITFG1	1493.78629	-0.5478765	0.07399364	-7.4043733	1.32E-13
9073	ITFG3	2158.84073	-0.7025503	0.06302118	-11.147845	7.34E-29
9075	ITGA10	86.256803	-0.9209634	0.22655112	-4.0651462	4.80E-05
9078	ITGA2B	13.6941923	-1.43234	0.5714901	-2.5063252	0.01219934
9079	ITGA3	50303.1368	0.38052876	0.04804868	7.91965011	2.38E-15
9081	ITGA5	6995.21878	0.82920309	0.06173229	13.4322431	3.91E-41
9097	ITGB2-AS1	68.6436645	-1.1278944	0.26073427	-4.3258387	1.52E-05
9098	ITGB3	582.411489	-1.1897024	0.11476048	-10.36683	3.51E-25
9100	ITGB4	7514.26158	-1.2115595	0.06397301	-18.938604	5.48E-80
9101	ITGB5	8940.58965	-0.8146044	0.0499449	-16.310063	8.37E-60
9102	ITGB6	44.6121453	-1.0339476	0.30992807	-3.3360888	0.00084966
9117	ITM2B	6975.35746	-0.7335828	0.05555399	-13.204862	8.23E-40
9118	ITM2C	3177.19737	-0.4963952	0.0623862	-7.9568111	1.77E-15
9123	ITPKB	224.817368	-1.67397	0.14631317	-11.441006	2.61E-30
9125	ITPKC	824.400294	0.62574328	0.08910276	7.02271458	2.18E-12

9129	ITPR3	23343.844	0.8069175	0.07768765	10.3866898	2.85E-25
9130	ITPRIP	3344.82102	0.92175991	0.08397091	10.9771339	4.92E-28
9131	ITPRIPL1	3.64186952	3.35603416	1.38880839	2.41648465	0.01567119
9132	ITPRIPL2	2804.63618	-0.2144141	0.06849686	-3.1302767	0.00174642
9137	IVNS1ABP	3716.67817	0.16932417	0.05684267	2.97882129	0.00289359
9144	IZUMO4	56.8508774	-0.8709527	0.26623802	-3.2713312	0.00107042
9147	JADE3	721.382477	0.61222208	0.10435927	5.86648481	4.45E-09
9148	JAG1	1581.53711	0.43479776	0.08547304	5.08695779	3.64E-07
9150	JAGN1	2199.07413	0.61013349	0.07626572	8.00010134	1.24E-15
9151	JAK1	6414.87115	0.32662851	0.07043957	4.63700338	3.53E-06
9153	JAK3	41.6054334	-1.1268732	0.32028623	-3.5183316	0.00043427
9160	JARID2	2506.24774	0.84745839	0.07123784	11.8961826	1.24E-32
9162	JAZF1	255.201469	0.38546982	0.1282594	3.00539232	0.00265238
9165	JDP2	632.95859	0.7264525	0.10929224	6.64688064	2.99E-11
9167	JKAMP	3112.38824	-0.4735088	0.06153555	-7.6948818	1.42E-14
9170	JMJD4	1199.37442	0.50272031	0.08705607	5.77467252	7.71E-09
9171	JMJD6	1921.95787	0.39781826	0.07831098	5.07998078	3.77E-07
9172	JMJD7	90.9468823	-0.6458141	0.20929491	-3.0856655	0.00203097
9174	JMJD8	1652.57291	-0.7613921	0.07233249	-10.526281	6.54E-26
9176	JOSD1	3213.211	0.67983099	0.07507589	9.0552503	1.36E-19
9178	JPH1	163.666513	2.67998983	0.19051496	14.0670837	6.05E-45
9179	JPH2	291.573399	-0.7857053	0.13861948	-5.6680723	1.44E-08
9184	JRKL	1519.93389	0.35522914	0.07562207	4.69742676	2.63E-06
9188	JUN	8502.69004	1.29566832	0.05043723	25.6887294	1.56E-145
9190	JUND	7253.97424	-0.2785081	0.06978462	-3.9909665	6.58E-05
9191	JUP	4179.1062	-1.0923464	0.05481025	-19.929601	2.25E-88
9193	KALRN	82.4636442	0.54460927	0.21695647	2.51022368	0.01206547
9194	KANK1	642.501878	-0.291786	0.09075557	-3.2150748	0.00130411
9195	KANK2	3943.11023	-0.6504415	0.05874848	-11.071631	1.72E-28
9204	KARS	4998.19248	0.38708381	0.05507474	7.0283365	2.09E-12
9205	KAT2A	2923.46968	-0.5199039	0.05659272	-9.1867633	4.05E-20
9206	KAT2B	1453.7144	-0.7205405	0.08746023	-8.238493	1.74E-16
9208	KAT6A	1953.25166	0.27959292	0.08764353	3.19011485	0.00142216
9210	KAT7	1526.55124	0.58230358	0.08650737	6.73125965	1.68E-11
9218	KAZALD1	188.882083	-0.8713391	0.17038549	-5.1139281	3.16E-07
9219	KAZN	476.879372	-0.3417219	0.11759062	-2.9060304	0.00366046
9224	KBTBD2	3462.05585	0.32783489	0.05965634	5.495391	3.90E-08
9225	KBTBD3	162.567004	-0.7742241	0.17161004	-4.5115318	6.44E-06
9226	KBTBD4	585.160428	-1.1037938	0.09524185	-11.589377	4.67E-31
9227	KBTBD6	460.054718	-0.6635866	0.11272429	-5.8868109	3.94E-09
9228	KBTBD7	119.963412	-1.7118077	0.20676503	-8.2789999	1.24E-16
9234	KCMF1	2408.51883	0.19022234	0.07293197	2.6082162	0.00910154
9246	KCNAB2	791.397054	0.72237555	0.0919164	7.85905004	3.87E-15

9247	KCNAB3	64.5222658	0.72484542	0.25671506	2.8235407	0.00474964
9252	KCNC3	35.6309758	0.82384273	0.37353433	2.20553418	0.02741663
9255	KCND1	93.8421646	0.52936474	0.22898004	2.31183794	0.02078662
9257	KCND3	7.48827168	-5.3342291	1.35664336	-3.9319317	8.43E-05
9262	KCNE3	17.6609552	-2.8204828	0.5966006	-4.7275897	2.27E-06
9286	KCNJ11	19.2211192	1.80848844	0.48364476	3.73929092	0.00018454
9289	KCNJ14	130.001789	0.73732902	0.1826042	4.0378535	5.39E-05
9301	KCNK1	9.65067259	-1.538084	0.72191833	-2.1305512	0.03312613
9312	KCNK5	41.2150527	-1.3782998	0.34038375	-4.0492526	5.14E-05
9328	KCNN4	1804.32026	-0.5776686	0.07141388	-8.0890236	6.01E-16
9332	KCNQ1OT1	352.860824	-0.5215796	0.16730462	-3.1175444	0.00182364
9336	KCNQ5	665.483664	1.36671207	0.12327904	11.08633	1.46E-28
9338	KCNQ5-IT1	26.5342218	2.00130348	0.45400031	4.40815441	1.04E-05
9352	KCTD12	927.458121	-0.9056655	0.0871582	-10.391053	2.72E-25
9356	KCTD16	1.96208461	3.36490593	1.70846754	1.96954631	0.04889039
9357	KCTD17	431.796169	0.23176258	0.11106693	2.08669298	0.0369159
9358	KCTD18	601.183487	-0.4321909	0.09689145	-4.4605685	8.17E-06
9360	KCTD2	1893.39593	0.17736055	0.06819993	2.60059722	0.00930616
9366	KCTD5	2926.47288	0.63016442	0.07279895	8.65622901	4.88E-18
9370	KCTD9	1574.53786	0.23218012	0.07886577	2.94399119	0.00324009
9371	KDELC1	203.707305	-1.0794395	0.16645885	-6.4847229	8.89E-11
9372	KDELC2	5224.13784	-0.8904553	0.06491419	-13.71742	7.99E-43
9373	KDELR1	7422.7621	-0.401663	0.07782734	-5.1609502	2.46E-07
9375	KDELR3	797.627508	-1.0746652	0.09295819	-11.560736	6.51E-31
9378	KDM1B	839.947834	0.24712123	0.1039119	2.3781803	0.01739832
9381	KDM3A	2320.62272	-0.4980507	0.07048833	-7.0657185	1.60E-12
9382	KDM3B	3459.30634	-0.3055222	0.06966565	-4.3855506	1.16E-05
9383	KDM4A	1378.45316	0.22842158	0.07620067	2.99763235	0.00272086
9385	KDM4B	2213.12971	-0.6467018	0.06851847	-9.4383566	3.79E-21
9390	KDM5B	3925.37533	0.67994448	0.0589192	11.5402879	8.26E-31
9393	KDM6A	1693.63865	0.38326976	0.09061212	4.22978458	2.34E-05
9394	KDM6B	1253.16898	1.78255986	0.10013868	17.8009132	6.95E-71
9395	KDM7A	676.582195	1.2045798	0.10053154	11.9821081	4.41E-33
9400	KEL	21.869536	2.42230207	0.47893997	5.05763193	4.24E-07
9404	KHDC1	575.038933	-0.3015078	0.11293421	-2.6697649	0.00759044
9407	KHDRBS1	9577.25461	0.44177901	0.05444248	8.11460044	4.87E-16
9409	KHDRBS3	84.8988837	1.00130199	0.22474096	4.45536046	8.38E-06
9410	KHK	285.237257	-1.4991232	0.14198034	-10.558667	4.63E-26
9412	KHSRP	7731.64131	0.64178471	0.10502322	6.11088422	9.91E-10
9413	KIAA0020	2994.40166	0.72419712	0.06048129	11.9739038	4.87E-33
9416	KIAA0100	10361.5719	-0.3789936	0.07707389	-4.9172764	8.78E-07
9420	KIAA0195	1950.65655	-0.6784647	0.07219901	-9.3971474	5.61E-21
9421	KIAA0196	903.042322	-0.3136599	0.09685496	-3.2384493	0.00120181

9428	KIAA0368	8080.04656	0.2133586	0.04991352	4.27456547	1.92E-05
9431	KIAA0430	1284.4473	-0.1717393	0.08059084	-2.1310024	0.03308894
9432	KIAA0513	462.459945	0.57711375	0.10770159	5.35845163	8.39E-08
9435	KIAA0753	1013.62191	0.40075769	0.08912203	4.49673004	6.90E-06
9439	KIAA0895L	241.305388	-0.7500843	0.14261195	-5.2596178	1.44E-07
9440	KIAA0907	2603.59479	0.20539348	0.06450824	3.1839882	0.00145261
9442	KIAA0930	2406.62554	-0.7388761	0.06466175	-11.426789	3.07E-30
9445	KIAA1033	4548.94661	0.50188971	0.08604113	5.83313689	5.44E-09
9446	KIAA1107	173.143616	-0.5854497	0.18893166	-3.0987382	0.00194347
9450	KIAA1161	495.969269	-0.3354861	0.09902143	-3.3880149	0.000704
9454	KIAA1211L	90.7654179	-1.6364962	0.2325707	-7.0365536	1.97E-12
9455	KIAA1217	2900.37695	-0.5170813	0.0914466	-5.6544622	1.56E-08
9457	KIAA1279	3342.16204	-0.485092	0.06159941	-7.8749457	3.41E-15
9458	KIAA1324	132.103329	-0.881997	0.19586756	-4.5030273	6.70E-06
9468	KIAA1524	2433.49406	0.17838241	0.07394704	2.41229962	0.01585225
9472	KIAA1586	923.577686	0.18599521	0.08683353	2.14197448	0.03219554
9478	KIAA1715	2745.5429	-0.8717063	0.06311007	-13.812476	2.14E-43
9481	KIAA1841	431.233222	-0.4772839	0.12320436	-3.8739209	0.0001071
9482	KIAA1875	111.454011	0.39255206	0.19820362	1.98054944	0.04764182
9490	KIDINS220	2514.54764	0.39042504	0.08631116	4.52345954	6.08E-06
9494	KIF13B	1701.59759	-0.3639045	0.07008292	-5.1924844	2.08E-07
9496	KIF15	1268.51738	-0.451391	0.07445033	-6.0629825	1.34E-09
9498	KIF17	52.0474848	1.88727546	0.30129535	6.2638719	3.76E-10
9500	KIF18B	1366.92934	-0.7146196	0.07468753	-9.5681257	1.09E-21
9505	KIF20A	2310.3891	-0.7936741	0.0787506	-10.078324	6.89E-24
9509	KIF22	2218.41422	-0.466036	0.07350645	-6.3400696	2.30E-10
9510	KIF23	4174.27585	-0.1458521	0.05615359	-2.5973773	0.00939387
9517	KIF2A	2977.01969	0.21283876	0.07926038	2.68531094	0.00724624
9523	KIF4A	3326.05181	-0.177168	0.06501977	-2.7248329	0.0064334
9528	KIF6	3.01914845	-3.9735991	1.58833847	-2.5017332	0.0123587
9532	KIFAP3	971.213244	0.56720305	0.08632476	6.57057202	5.01E-11
9533	KIFC1	3378.23795	-0.2687385	0.05549015	-4.8429953	1.28E-06
9536	KIN	660.081088	0.25869237	0.08634466	2.99604369	0.00273507
9553	KIRREL	4726.47209	-0.8252788	0.09132136	-9.0370837	1.61E-19
9562	KIZ	314.227031	-0.4030544	0.11998656	-3.3591627	0.00078179
9571	KLF10	3918.17473	2.15223247	0.06628962	32.4671093	3.11E-231
9572	KLF11	1149.87622	0.67044265	0.07436784	9.01522273	1.96E-19
9578	KLF17	35.1132682	-1.5022934	0.36815912	-4.0805547	4.49E-05
9582	KLF4	3568.93894	1.33973183	0.06445695	20.78491	5.93E-96
9583	KLF5	2013.28775	0.48964482	0.07551815	6.48380325	8.94E-11
9594	KLHDC7B	6.55069384	4.22311968	1.22742641	3.44062963	0.00058036
9596	KLHDC8B	629.111385	-0.8179751	0.09706821	-8.4268074	3.55E-17
9597	KLHDC9	46.8133468	-0.9297041	0.29049536	-3.2004095	0.00137232

9606	KLHL18	1313.51295	0.66095537	0.08521592	7.75624286	8.75E-15
9609	KLHL21	3961.21544	1.10881372	0.06548061	16.9334664	2.55E-64
9610	KLHL22	728.964464	-0.5341903	0.09270949	-5.76198	8.31E-09
9612	KLHL24	1502.14107	-0.1703013	0.07739971	-2.2002834	0.02778679
9614	KLHL26	372.763201	-0.2653321	0.11229338	-2.3628475	0.01813513
9616	KLHL29	1424.62622	1.90115836	0.08131341	23.3806261	6.73E-121
9624	KLHL36	1056.00095	-0.7681189	0.0770883	-9.9641436	2.19E-23
9629	KLHL42	838.953213	-0.8608212	0.08676471	-9.9213284	3.36E-23
9635	KLHL8	997.975651	0.36984628	0.08538562	4.33148206	1.48E-05
9657	KLRC1	10.6569443	-3.2942286	0.78843282	-4.1781983	2.94E-05
9658	KLRC2	137.452116	-0.6568846	0.18852329	-3.4843682	0.0004933
9659	KLRC3	104.168926	-1.065519	0.22600586	-4.7145637	2.42E-06
9669	KMT2A	4698.56095	0.39836291	0.0933503	4.26739824	1.98E-05
9676	KNDC1	134.615571	-1.0765653	0.19700934	-5.464539	4.64E-08
9678	KNOP1	1006.33867	0.32948634	0.07611036	4.32906021	1.50E-05
9683	KPNA3	2703.29095	0.29910993	0.07768539	3.8502726	0.00011799
9684	KPNA4	4127.57425	0.73640413	0.05645839	13.0433078	6.94E-39
9685	KPNA5	113.203244	0.58769241	0.21981457	2.67358264	0.00750458
9686	KPNA6	4527.05823	0.6756151	0.06708	10.0717816	7.36E-24
9687	KPNA7	8.95045381	-2.7989568	0.82227141	-3.403933	0.00066423
9688	KPNB1	21321.206	0.25263004	0.05362354	4.71117814	2.46E-06
9690	KPTN	461.819208	-0.4036452	0.11380629	-3.5467737	0.00038998
9694	KRBOX1	290.487144	-0.4115542	0.14332365	-2.8715021	0.00408526
9696	KRBOX4	400.291544	0.96776517	0.11208831	8.63395263	5.93E-18
9702	KRR1	3399.8223	0.54975048	0.06299336	8.72711847	2.61E-18
9704	KRT10	1448.00741	0.40836034	0.08254224	4.94728953	7.53E-07
9708	KRT15	112.793568	-0.7777078	0.207005	-3.7569516	0.000172
9717	KRT19	7734.99971	-1.0014599	0.10091673	-9.9236266	3.29E-23
9746	KRT7	19332.3958	-0.7662762	0.07695785	-9.9570898	2.35E-23
9756	KRT79	14.0076436	3.24829348	0.71147536	4.5655741	4.98E-06
9757	KRT8	8928.52605	-1.4977537	0.04954712	-30.228876	9.89E-201
9758	KRT80	7510.65072	-0.5645255	0.06291424	-8.9729374	2.89E-19
9759	KRT81	1267.50885	-0.8309634	0.0932398	-8.9121106	5.01E-19
9764	KRT86	124.50406	-1.1757137	0.21026479	-5.5915861	2.25E-08
9847	KRTAP5-AS1	4.2993742	4.56612229	1.44231602	3.16582651	0.00154643
9867	KTN1	16145.3965	0.14567682	0.0509317	2.86023871	0.00423322
9868	KTN1-AS1	79.1536899	0.80190335	0.25857779	3.10120742	0.00192733
9873	L1CAM	2459.93829	0.34384843	0.07567508	4.5437472	5.53E-06
9883	LACC1	737.050868	0.94035561	0.09932656	9.46731244	2.87E-21
9886	LACTB	2860.60738	-0.1669083	0.06198903	-2.692546	0.00709088
9898	LAMA3	2383.9952	-0.1977216	0.08436541	-2.3436331	0.01909695
9899	LAMA4	78.0522905	-1.419702	0.24237997	-5.8573405	4.70E-09
9900	LAMA5	9725.24561	-1.4151028	0.06759637	-20.934598	2.59E-97

9903	LAMB2	11861.7629	-0.277357	0.06290704	-4.4089979	1.04E-05
9905	LAMB3	37052.9412	1.15474347	0.06725986	17.1683902	4.58E-66
9908	LAMC2	11060.5495	1.63870785	0.06054861	27.0643344	2.59E-161
9911	LAMP2	10947.0457	-0.2907291	0.07034656	-4.1328115	3.58E-05
9912	LAMP3	135.304016	1.35663308	0.18849786	7.19707427	6.15E-13
9917	LAMTOR3	1491.53957	0.37884967	0.09053664	4.18449015	2.86E-05
9918	LAMTOR4	1332.64385	-0.3644106	0.13942417	-2.6136829	0.00895721
9919	LAMTOR5	3851.53379	-0.6544065	0.07540828	-8.6781784	4.02E-18
9921	LANCL1	917.935953	-1.2211664	0.10239944	-11.925518	8.71E-33
9934	LARP4B	4620.1907	0.15044955	0.05201206	2.89258968	0.0038208
9936	LARP7	2247.72224	-0.3296148	0.0652104	-5.0546351	4.31E-07
9937	LARS	9718.93783	0.64029369	0.0516112	12.4061002	2.42E-35
9940	LAS1L	2651.54807	0.22775195	0.07115446	3.20081083	0.00137041
9941	LASP1	21424.4862	-0.6120815	0.04844043	-12.635757	1.34E-36
9944	LATS1	1216.04615	0.30397582	0.10046186	3.02578344	0.0024799
9947	LAYN	3731.24064	-0.8415183	0.05641166	-14.917454	2.54E-50
9948	LBH	669.164146	-3.0402997	0.10727463	-28.341275	1.07E-176
9955	LBX2-AS1	385.009347	2.30201051	0.12532666	18.368083	2.37E-75
9957	LCA5	84.5168056	0.8615823	0.22232816	3.87527293	0.00010651
9984	LCMT2	576.377646	0.23608741	0.09436968	2.50172947	0.01235883
9989	LCN2	5241.71511	-0.7626347	0.06496111	-11.739865	7.96E-32
10001	LDB1	1406.97348	-0.1435832	0.07228647	-1.9863089	0.04699904
10007	LDHB	19110.1498	-0.4718036	0.09682253	-4.8728697	1.10E-06
10009	LDHD	52.9959031	-1.6729343	0.34071504	-4.9100689	9.10E-07
10013	LDLRAD3	1780.86975	0.3087934	0.08053885	3.83409252	0.00012603
10017	LDOC1	364.291988	-2.1673228	0.12778977	-16.960064	1.62E-64
10018	LDOC1L	1673.27522	-0.6363787	0.07463444	-8.5266093	1.51E-17
10022	LEF1	29.2680782	1.59582074	0.45124159	3.53651075	0.00040545
10033	LENG1	365.954978	-0.5090188	0.14908395	-3.4143096	0.00063944
10040	LEPROT	4514.12132	0.76504751	0.06037311	12.6719917	8.45E-37
10046	LFNG	373.479241	-1.5658779	0.11703747	-13.379287	7.99E-41
10055	LGALS3BP	24989.8056	-0.4638713	0.05878443	-7.8910571	3.00E-15
10061	LGALS9	50.2027475	-0.9955677	0.29755649	-3.3458108	0.00082042
10070	LGR4	1264.23404	-1.4618482	0.09372068	-15.597926	7.52E-55
10078	LHFPL2	4395.35335	0.27692332	0.05971818	4.63716958	3.53E-06
10084	LHPP	155.101353	-0.8318668	0.17908226	-4.6451656	3.40E-06
10099	LIG1	2172.0581	-1.0834873	0.07414897	-14.612305	2.34E-48
10101	LIG4	870.550934	0.78091658	0.09124786	8.55819093	1.15E-17
10115	LIMA1	11102.6214	0.91878252	0.06132983	14.981006	9.77E-51
10117	LIMD1	2603.30688	-0.3591852	0.06551154	-5.4827773	4.19E-08
10120	LIME1	457.621494	-0.7250519	0.14027236	-5.1688866	2.35E-07
10121	LIMK1	6315.58282	-0.2021306	0.05500124	-3.6750191	0.00023783
10124	LIMS2	1104.19842	-0.1839069	0.08574758	-2.1447471	0.03197307

10134	LIN7B	87.1395955	-0.7265761	0.26790074	-2.7121095	0.00668565
10136	LIN9	696.778053	-0.2573876	0.09388086	-2.7416413	0.00611331
10137	LINC-PINT	953.037278	0.55456027	0.08347211	6.64365971	3.06E-11
10145	LINC00094	836.92347	-0.358889	0.0793541	-4.5226272	6.11E-06
10154	LINC00152	4361.39989	0.19811425	0.08738299	2.26719467	0.02337834
10162	LINC00173	112.304084	-0.7260345	0.19388342	-3.7446962	0.00018061
10164	LINC00176	74.1142649	0.6902026	0.23244122	2.96936398	0.00298417
10204	LINC00294	376.027889	-0.439741	0.12288295	-3.5785352	0.00034553
10215	LINC00310	33.5128578	0.77488091	0.34441386	2.24985401	0.02445821
10236	LINC00341	39.2680504	-0.7291872	0.34431096	-2.1178158	0.03419067
10239	LINC00346	48.685075	-0.5731791	0.28240577	-2.0296297	0.0423942
10297	LINC00460	22.5626329	1.72468237	0.50049576	3.44594805	0.00056906
10362	LINC00565	19.1279209	1.6421237	0.54443967	3.01617204	0.00255988
10411	LINC00638	152.628235	0.97610381	0.17206841	5.67276591	1.41E-08
10424	LINC00657	10744.5579	-0.2829784	0.05248705	-5.3913955	6.99E-08
10428	LINC00662	203.812058	1.63932939	0.15422089	10.6297489	2.17E-26
10431	LINC00665	124.566375	0.7302583	0.20288588	3.599355	0.00031901
10432	LINC00667	733.965631	0.43431544	0.08607962	5.04550827	4.52E-07
10434	LINC00669	88.0969927	-0.9262949	0.22117907	-4.1879864	2.81E-05
10437	LINC00672	37.0110089	-1.1523663	0.33951424	-3.394162	0.00068839
10439	LINC00674	1616.24162	-0.6381477	0.07955411	-8.0215557	1.04E-15
10458	LINC00702	46.3694645	2.25263692	0.34199878	6.58668107	4.50E-11
10463	LINC00707	15.7167637	1.53681984	0.59629155	2.57729602	0.00995766
10474	LINC00842	43.6517806	-1.0365636	0.30510254	-3.3974269	0.00068023
10489	LINC00865	40.9544794	1.05757978	0.34169654	3.09508485	0.00196757
10500	LINC00883	620.907261	-0.4639116	0.0924179	-5.0197155	5.17E-07
10523	LINC00909	281.563456	0.86318607	0.13492159	6.39768683	1.58E-10
10534	LINC00926	48.9581064	-0.8905038	0.30982453	-2.8742197	0.00405027
10550	LINC00944	25.8812052	2.09628153	0.42167376	4.97133505	6.65E-07
10578	LINC00997	232.920629	-0.3986172	0.14209394	-2.8053073	0.00502686
10579	LINC00998	843.577247	0.3290806	0.08323909	3.95343828	7.70E-05
10584	LINC01003	423.465117	-0.2730794	0.13589315	-2.0095159	0.04448245
10639	LINC01088	203.684178	0.61132704	0.14263793	4.2858659	1.82E-05
10662	LINC01116	622.152678	-0.6403243	0.09874017	-6.4849419	8.88E-11
10670	LINC01124	76.4394558	-0.7425728	0.23968345	-3.0981397	0.0019474
10674	LINC01128	443.331862	0.77769984	0.11325364	6.86688598	6.56E-12
10681	LINC01138	155.767292	0.68220495	0.18786189	3.63141753	0.00028187
10705	LINC01179	7.20525381	2.99577994	0.93204833	3.21418947	0.00130813
10723	LINC01204	79.6019164	3.29786426	0.29725482	11.0944012	1.34E-28
10739	LINC01224	522.761116	0.58901386	0.11685341	5.04062194	4.64E-07
10748	LINC01234	29.8023709	-0.8675474	0.37364989	-2.321819	0.02024268
10759	LINC01252	29.0207125	-1.0624788	0.3826022	-2.7769803	0.00548665
10790	LINC01296	15.2609749	3.42148658	0.67841889	5.04332449	4.58E-07

10832	LINC01356	62.6941593	1.38600682	0.31325872	4.42447967	9.67E-06
10928	LINC01503	85.8704534	-0.9107926	0.24441173	-3.7264684	0.00019418
10945	LINC01521	109.418294	-0.5599628	0.19629515	-2.8526575	0.00433553
10952	LINC01530	13.5198255	-1.371734	0.60953625	-2.2504552	0.02442006
10979	LINC01561	31.0597691	-1.4900709	0.37446692	-3.9791791	6.92E-05
10992	LINC01578	1804.649	-0.7836165	0.09684964	-8.0910628	5.91E-16
10996	LINC01583	14.3590231	2.26647306	0.62289527	3.63861014	0.00027411
11030	LIPA	3935.32294	-0.5600655	0.06143086	-9.1170069	7.72E-20
11032	LIPE	172.132238	-0.5226616	0.16022776	-3.2619914	0.00110633
11036	LIPH	761.758038	-1.3550269	0.09504788	-14.256256	4.10E-46
11042	LIPT1	284.075307	1.26928991	0.13859149	9.15849801	5.26E-20
11044	LITAF	8402.44669	-0.6722393	0.0707663	-9.4994268	2.11E-21
11046	LIX1L	1607.36759	-0.4842483	0.06730855	-7.1944548	6.27E-13
11051	LLGL1	2561.90402	-0.691471	0.06692658	-10.331785	5.06E-25
11052	LLGL2	378.608298	-1.2716009	0.11126674	-11.4284	3.02E-30
11053	LLPH	721.269771	0.6131064	0.09829215	6.237593	4.44E-10
11057	LMAN2	5651.97345	-0.1851803	0.06745481	-2.7452495	0.00604649
11061	LMBRD1	1344.63084	-0.4252809	0.0730944	-5.8182414	5.95E-09
11063	LMCD1	255.457191	0.7781108	0.1315373	5.91551454	3.31E-09
11065	LMF1	121.743769	-0.6645462	0.19008474	-3.4960522	0.0004722
11067	LMF2	3537.84734	-0.3222406	0.06807875	-4.7333507	2.21E-06
11068	LMLN	1234.83757	0.47795161	0.08730491	5.47451021	4.39E-08
11071	LMNB1	2955.18381	-0.9809231	0.07004956	-14.003273	1.49E-44
11074	LMNTD2	320.344823	-1.264822	0.1270836	-9.9526767	2.45E-23
11079	LMO7	2455.08041	-0.224618	0.07712738	-2.9122985	0.0035878
11092	LNX1	172.168531	-1.4254193	0.16246763	-8.7735586	1.73E-18
11109	LOC1001283!	190.823354	0.94878969	0.16772663	5.65676223	1.54E-08
11138	LOC1001295!	235.708345	-0.6134649	0.15392855	-3.9853876	6.74E-05
11151	LOC1001300!	118.670365	-1.342925	0.21478967	-6.2522794	4.05E-10
11156	LOC1001303!	47.3722655	2.51211934	0.33607353	7.47490976	7.73E-14
11166	LOC1001307(	108.255085	0.96295186	0.21365861	4.50696493	6.58E-06
11256	LOC1002886:	376.587899	-0.3965356	0.14072529	-2.8177991	0.00483541
11259	LOC1002887!	111.453648	1.64212996	0.21709724	7.56402971	3.91E-14
11276	LOC1002941·	1442.16289	0.55311772	0.07474775	7.39979106	1.36E-13
11279	LOC1003350:	22.2085819	1.05525624	0.43430106	2.42978047	0.01510797
11280	LOC1003792:	66.906175	0.93437694	0.25746809	3.62909799	0.00028441
11326	LOC1005061(	294.441984	-1.0006763	0.17571166	-5.6949908	1.23E-08
11331	LOC1005061:	95.8600279	-0.9143636	0.2088609	-4.3778591	1.20E-05
11360	LOC1005064	18.3194337	1.18645286	0.49203445	2.41132072	0.01589486
11363	LOC1005065·	1181.1499	0.48279635	0.07966892	6.06003388	1.36E-09
11366	LOC1005066(	111.099668	0.53804234	0.19342484	2.78166108	0.00540815
11382	LOC1005068·	198.885825	0.55660718	0.1480724	3.7590205	0.00017058
11386	LOC1005068!	42.0285069	-0.9705532	0.3437824	-2.8231613	0.00475526

11389	LOC1005070(	385.546008	-0.4247881	0.11811788	-3.5963064	0.00032277
11390	LOC1005070(	7.11963104	-1.8648908	0.79051789	-2.3590747	0.01832057
11403	LOC1005072!	29.8348952	2.93566992	0.44914337	6.53615333	6.31E-11
11419	LOC1005074:	39.9951686	1.671348	0.34435124	4.85361402	1.21E-06
11437	LOC1005076	136.084114	-1.1269921	0.18315982	-6.153053	7.60E-10
11506	LOC1019269!	2.65896512	3.82225423	1.5702296	2.43420086	0.01492471
11517	LOC1019270:	220.786116	-0.4457411	0.13950681	-3.1951209	0.00139772
11518	LOC1019270	9.05121994	5.66326746	1.31709143	4.29982865	1.71E-05
11585	LOC1019273:	14.5077693	-4.0660189	0.81683567	-4.9777685	6.43E-07
11621	LOC1019274!	37.7218577	-0.8556049	0.33874516	-2.5258072	0.01154328
11625	LOC1019274!	27.6254186	1.83053284	0.43025612	4.25451896	2.09E-05
11637	LOC1019275	26.1887348	0.92291781	0.38536887	2.39489456	0.01662515
11662	LOC1019276(	3.74011458	5.37842909	1.55770413	3.45279248	0.00055482
11675	LOC1019277	162.152714	0.61228127	0.16848273	3.63408917	0.00027896
11683	LOC1019277!	212.629246	0.91359764	0.15107792	6.04719507	1.47E-09
11721	LOC1019279:	9.42801909	-2.336542	0.77258302	-3.0243248	0.00249189
11745	LOC1019280(	129.006738	1.08819735	0.19242804	5.65508711	1.56E-08
11748	LOC1019281(	28.0210759	-1.889246	0.41174466	-4.5883922	4.47E-06
11785	LOC1019282	7.73489477	2.55194235	0.85275243	2.99259465	0.00276617
11822	LOC1019284	16.5968745	2.43044878	0.55378923	4.3887614	1.14E-05
11888	LOC1019287!	29.6216277	3.08593285	0.46963626	6.57089994	5.00E-11
11896	LOC1019288	182.919072	2.18037655	0.17806944	12.2445299	1.80E-34
11989	LOC1019293	1.40910511	3.97476573	1.96107467	2.02683038	0.04267976
12029	LOC1019295!	78.3472644	-1.431657	0.23953936	-5.9767087	2.28E-09
12057	LOC1019296	2.82177482	2.98498807	1.47727536	2.02060371	0.0433208
12063	LOC1019297(	194.162374	2.85892116	0.18316189	15.6087116	6.35E-55
12064	LOC1019297(	216.527305	1.84939626	0.16143105	11.4562612	2.19E-30
12110	LOC1026064(	221.909285	1.32065014	0.15103741	8.74386096	2.25E-18
12132	LOC1027237(	17.3442319	1.09753028	0.55227213	1.98729978	0.04688919
12170	LOC1027243:	25.5161757	-1.3954382	0.39868668	-3.5000873	0.00046511
12179	LOC1027245:	31.6628351	1.68172157	0.3740437	4.49605638	6.92E-06
12180	LOC1027245!	3.58430198	2.65774584	1.29679281	2.04947608	0.04041558
12205	LOC1027249:	110.806536	-0.4909452	0.21095864	-2.327211	0.01995404
12217	LOC1033449:	105.340368	-0.5317652	0.21050881	-2.5260946	0.01153384
12219	LOC1036110!	169.217251	-0.3345607	0.16398615	-2.0401767	0.04133273
12251	LOC146880	882.367445	-0.3651879	0.08193038	-4.4572956	8.30E-06
12252	LOC148413	552.85018	0.33418176	0.10850467	3.07988376	0.00207081
12264	LOC152225	464.937951	2.19026269	0.11493205	19.0570228	5.75E-81
12266	LOC153684	33.228899	-0.8529991	0.34683017	-2.4594143	0.01391639
12284	LOC254896	28.4364858	-1.2684556	0.38322989	-3.3099077	0.00093327
12291	LOC283140	34.9775434	0.77566115	0.33188842	2.33711422	0.01943325
12300	LOC283575	9.47248903	2.46390455	0.7926091	3.10859988	0.00187976
12303	LOC283710	5.05598104	-2.1919461	1.02532175	-2.1378129	0.03253193

12310	LOC284080	40.3251312	1.33930469	0.31757201	4.21732603	2.47E-05
12385	LOC374443	521.915299	1.22790323	0.123542	9.93915634	2.81E-23
12409	LOC389906	296.513575	0.50155145	0.12522631	4.00516032	6.20E-05
12418	LOC399815	61.0001635	0.94139388	0.25395342	3.70695491	0.00020977
12430	LOC400706	10.7965771	-2.5412453	0.7168585	-3.5449748	0.00039265
12513	LOC642846	97.2105805	-0.7009617	0.24093403	-2.9093511	0.0036218
12549	LOC646029	14.4827164	4.8570642	0.96257431	5.04591089	4.51E-07
12569	LOC652276	121.692832	2.20099608	0.2146866	10.2521354	1.16E-24
12586	LOC728323	59.6622953	1.13003243	0.27930898	4.04581494	5.21E-05
12587	LOC728392	194.507977	-0.6961941	0.14992902	-4.643491	3.43E-06
12588	LOC728485	33.219793	1.92445436	0.37936719	5.07280125	3.92E-07
12589	LOC728554	299.806264	-0.6360963	0.15784519	-4.0298748	5.58E-05
12594	LOC728743	234.249407	-1.7539449	0.15563497	-11.269606	1.85E-29
12617	LOC730101	508.164397	-0.3862298	0.11444251	-3.3748804	0.00073848
12618	LOC730102	278.047453	-1.4329062	0.16251906	-8.8168503	1.18E-18
12636	LONP2	1113.71789	-0.6395528	0.09224988	-6.9328309	4.13E-12
12639	LONRF3	446.693938	1.14346491	0.12263641	9.3240247	1.12E-20
12643	LOXL1	340.045889	-0.3240492	0.11370987	-2.8497898	0.00437481
12644	LOXL1-AS1	430.563873	0.59827167	0.10735036	5.57307579	2.50E-08
12646	LOXL3	108.44163	1.59234903	0.20061354	7.9373955	2.06E-15
12647	LOXL4	5483.97962	-1.3233673	0.05383474	-24.582033	1.97E-133
12649	LPAL2	104.38393	-0.5482671	0.2046044	-2.6796449	0.00737003
12656	LPCAT1	4786.75121	-1.2067798	0.0548309	-22.009118	2.36E-107
12659	LPCAT4	1487.15967	-1.0382631	0.07142802	-14.535795	7.19E-48
12663	LPIN3	994.764989	-0.8801491	0.08176053	-10.764963	5.04E-27
12670	LPPR2	2043.50836	-0.6428928	0.06796182	-9.4596168	3.09E-21
12677	LRCH1	930.140636	0.23733075	0.0836055	2.83869782	0.0045298
12680	LRCH4	2444.37174	-0.6365381	0.06076631	-10.475182	1.12E-25
12684	LRFN3	771.699783	-0.7755016	0.10655368	-7.2780368	3.39E-13
12687	LRG1	168.609552	-2.159989	0.17606083	-12.268425	1.34E-34
12691	LRIG2	790.806901	0.77756612	0.09502608	8.18265985	2.78E-16
12692	LRIG3	409.322989	0.43840393	0.10660235	4.11251641	3.91E-05
12697	LRP1	3454.65395	-0.4317254	0.08152	-5.2959443	1.18E-07
12699	LRP10	8752.4967	-0.3825041	0.057445	-6.6586143	2.76E-11
12700	LRP11	2378.65091	-0.3689317	0.06677859	-5.5247006	3.30E-08
12704	LRP2BP	132.738403	-0.6593194	0.17639367	-3.7377722	0.00018566
12705	LRP3	1131.91294	-0.2496677	0.07521685	-3.3193049	0.00090242
12706	LRP4	204.901858	-0.4759211	0.1513096	-3.1453462	0.0016589
12708	LRP5	7250.701	-1.462453	0.06247598	-23.408243	3.52E-121
12713	LRPPRC	9006.04013	0.50464299	0.05027834	10.0369866	1.05E-23
12714	LRR1	2170.57516	0.31756835	0.06775336	4.68712328	2.77E-06
12720	LRRC15	245.100139	-0.442621	0.16170318	-2.7372437	0.00619564
12728	LRRC20	1550.11057	-0.3957454	0.07140029	-5.5426295	2.98E-08

12730	LRRC24	212.854477	-0.4337248	0.1802153	-2.4067032	0.01609724
12733	LRRC27	296.799762	-0.5206774	0.1270091	-4.0995283	4.14E-05
12736	LRRC3	126.821385	-0.5054752	0.19599969	-2.5789592	0.00990985
12742	LRRC36	7.44313296	-1.518358	0.73569147	-2.0638515	0.0390318
12747	LRRC37A4P	185.360844	-0.4803767	0.16408814	-2.9275527	0.00341641
12749	LRRC37A6P	36.546686	-1.7255715	0.36203257	-4.7663432	1.88E-06
12758	LRRC40	1108.75559	0.38572123	0.08688271	4.43956282	9.01E-06
12759	LRRC41	2314.29649	0.19060638	0.0638488	2.98527748	0.00283321
12760	LRRC42	2991.06825	0.48664988	0.06971908	6.9801537	2.95E-12
12762	LRRC45	863.370086	-1.6095755	0.12838616	-12.536986	4.68E-36
12766	LRRC49	194.846655	0.76464372	0.14475745	5.28224089	1.28E-07
12781	LRRC70	37.0931997	2.35475363	0.38542846	6.10944414	1.00E-09
12788	LRRC75A-AS	13892.1394	0.59698191	0.06469335	9.22787175	2.76E-20
12789	LRRC75B	132.656218	-0.3920066	0.18880539	-2.076247	0.03787112
12792	LRRC8C	673.734047	0.40465258	0.10803425	3.74559538	0.00017997
12796	LRRCC1	336.686772	-0.6351994	0.12374275	-5.1332255	2.85E-07
12798	LRRFIP1	5536.12992	0.2213101	0.07186014	3.07973402	0.00207186
12799	LRRFIP2	2640.00508	0.3503228	0.0588796	5.94981647	2.68E-09
12803	LRRK1	1092.25466	-0.4877495	0.11493001	-4.2438825	2.20E-05
12805	LRRN1	38.094009	-1.1628306	0.32524041	-3.5752956	0.00034983
12818	LRWD1	1460.26627	0.16699982	0.08399222	1.98827724	0.04678104
12821	LSG1	2114.07105	0.25566879	0.06329808	4.03912404	5.37E-05
12822	LSM1	1265.56555	0.18437051	0.07974737	2.3119321	0.02078143
12823	LSM10	868.961297	0.31385741	0.08777352	3.57576415	0.00034921
12827	LSM14B	3526.05627	0.3361062	0.06502302	5.16903366	2.35E-07
12828	LSM2	1460.75182	-0.145714	0.07380777	-1.974237	0.0483548
12829	LSM3	1925.69156	-0.1930017	0.0709679	-2.7195631	0.00653682
12842	LTA	71.6790708	2.92552163	0.28884449	10.1283622	4.14E-24
12848	LTBP2	1694.75122	-0.6524678	0.07413433	-8.8011551	1.35E-18
12849	LTBP3	3918.75103	-1.344218	0.07492964	-17.939736	5.77E-72
12851	LTBR	4106.97779	-0.1993473	0.06137595	-3.2479709	0.00116231
12861	LUM	118.385386	-0.4338302	0.18161336	-2.3887569	0.01690548
12864	LURAP1L	407.028915	1.44785328	0.11172634	12.9589252	2.09E-38
12865	LURAP1L-AS	61.5020474	2.75357418	0.31895879	8.63300936	5.98E-18
12866	LUZP1	2990.79471	0.6531865	0.09046105	7.22063827	5.17E-13
12870	LVCAT1	13.4587714	-4.282264	0.85876474	-4.9865392	6.15E-07
12874	LXN	328.864824	-0.6279628	0.12375077	-5.0744151	3.89E-07
12899	LYPD3	23.7662175	-2.3181908	0.4577892	-5.0638828	4.11E-07
12901	LYPD5	4.5266117	2.53421498	1.09610964	2.31200866	0.02077721
12910	LYRM1	1690.79049	0.61961148	0.06528987	9.49016201	2.31E-21
12912	LYRM4	1491.0689	0.33894564	0.08194798	4.13610703	3.53E-05
12913	LYRM5	298.645257	0.64313422	0.13066355	4.92206316	8.56E-07
12914	LYRM7	783.520392	0.90402332	0.09579366	9.43719414	3.83E-21

12915	LYRM9	56.1736123	-1.183113	0.27855175	-4.2473725	2.16E-05
12918	LYSMD3	1736.20747	0.38039335	0.08490791	4.4800698	7.46E-06
12927	LZIC	802.242098	0.438013	0.10378015	4.22058544	2.44E-05
12928	LZTFL1	1183.69073	0.71780541	0.0896602	8.00584233	1.19E-15
12932	LZTS2	3819.90811	-0.5678464	0.06486908	-8.7537302	2.06E-18
12949	MAD2L1BP	1471.33615	0.36308199	0.08728458	4.15974955	3.19E-05
12952	MADD	3175.75731	-0.3536831	0.06998225	-5.0538965	4.33E-07
12959	MAFB	36.4618376	-1.2219259	0.33854385	-3.6093578	0.00030696
12960	MAFF	4545.4477	1.59247421	0.05670527	28.0833555	1.56E-173
12961	MAFG	4915.16737	0.66621771	0.06064354	10.9857978	4.47E-28
12964	MAFK	3126.33834	0.65674213	0.06023777	10.902498	1.12E-27
12971	MAGEA12	3.80876771	4.38588265	1.47228625	2.97896055	0.00289228
12993	MAGEC2	7.58033625	5.3822012	1.35494015	3.97227965	7.12E-05
12995	MAGED1	3239.86475	-1.1449345	0.06919176	-16.547266	1.68E-61
12996	MAGED2	6392.8332	-0.7422659	0.06721545	-11.043083	2.37E-28
12999	MAGEE1	272.957933	-1.6499824	0.14549065	-11.340814	8.24E-30
13002	MAGEH1	74.068258	-1.2162482	0.2439139	-4.9863835	6.15E-07
13011	MAGOH	1237.71176	0.33483172	0.08040213	4.16446322	3.12E-05
13015	MAK	25.3128789	1.47750972	0.40683444	3.63172233	0.00028154
13016	MAK16	1045.773	0.83847629	0.08207417	10.2160806	1.68E-24
13018	MAL2	246.27655	0.79473467	0.1300578	6.11062677	9.92E-10
13024	MAMDC2	596.833084	-0.4850035	0.09659349	-5.0210785	5.14E-07
13027	MAML1	2210.27218	0.39812546	0.07375234	5.39814001	6.73E-08
13032	MAN1A1	157.136743	-1.1565962	0.19577727	-5.9077145	3.47E-09
13037	MAN2A1	2488.57199	0.34454245	0.08957328	3.8464871	0.00011982
13038	MAN2A2	1985.22699	-0.5983149	0.08162228	-7.3302886	2.30E-13
13040	MAN2B2	1977.04924	-0.4850325	0.07527194	-6.4437368	1.17E-10
13041	MAN2C1	1069.27015	-0.4890247	0.07699575	-6.3513205	2.13E-10
13042	MANBA	504.909407	-0.9965808	0.10496485	-9.4944233	2.21E-21
13044	MANEA	390.828709	-0.4727214	0.13657142	-3.4613496	0.00053747
13048	MANSC1	98.3890157	-0.8726752	0.20560837	-4.2443565	2.19E-05
13054	MAP1B	13012.296	0.52562814	0.07285563	7.21465384	5.41E-13
13056	MAP1LC3B	6040.86259	1.44014299	0.05300142	27.171782	1.40E-162
13058	MAP1LC3C	16.8685602	-1.4337991	0.51732284	-2.771575	0.00557858
13059	MAP1S	1952.87047	-0.3673037	0.06231407	-5.8943942	3.76E-09
13063	MAP2K3	4847.0907	0.43528207	0.06641653	6.55382107	5.61E-11
13067	MAP2K6	14.1111171	-4.0182014	0.79356995	-5.0634495	4.12E-07
13069	MAP3K1	1748.82131	-1.0027283	0.09492837	-10.562999	4.42E-26
13070	MAP3K10	1370.51002	0.79769731	0.07384111	10.8028891	3.34E-27
13071	MAP3K11	3886.48483	-0.7969069	0.06017233	-13.243742	4.90E-40
13072	MAP3K12	320.155556	-0.7855657	0.12217198	-6.429999	1.28E-10
13074	MAP3K14	1515.68776	0.51142419	0.06979205	7.3278291	2.34E-13
13076	MAP3K15	286.390517	-1.3320377	0.128806	-10.341426	4.58E-25

13078	MAP3K2	2527.33382	0.23929062	0.06692776	3.57535664	0.00034975
13081	MAP3K5	642.928937	-0.5191188	0.10190907	-5.0939414	3.51E-07
13082	MAP3K6	592.645274	-0.6931134	0.09688823	-7.1537414	8.44E-13
13085	MAP3K8	514.148215	-1.3642612	0.10419774	-13.093002	3.61E-39
13089	MAP4K2	786.07113	-0.6193596	0.09521285	-6.5050001	7.77E-11
13092	MAP4K5	4357.90705	0.76013875	0.07523386	10.1036792	5.32E-24
13096	MAP7D1	9371.8693	0.39126005	0.05805721	6.73921586	1.59E-11
13100	MAPK1	4334.56423	-0.2538076	0.06860436	-3.6995838	0.00021595
13102	MAPK11	263.257284	-0.8794374	0.15167729	-5.7980821	6.71E-09
13107	MAPK1IP1L	6110.87312	0.45701448	0.05330678	8.57329032	1.01E-17
13108	MAPK3	1379.10469	-0.3241169	0.06801393	-4.7654489	1.88E-06
13110	MAPK6	2318.5247	0.52996262	0.08893453	5.95902006	2.54E-09
13111	MAPK7	888.240848	-0.6164024	0.08534626	-7.222371	5.11E-13
13112	MAPK8	2043.21501	0.74220956	0.06450099	11.5069491	1.22E-30
13115	MAPK8IP3	2215.7993	-0.6660001	0.06382384	-10.434974	1.72E-25
13117	MAPKAP1	3959.26671	-0.3006077	0.05900245	-5.0948334	3.49E-07
13118	MAPKAPK2	7552.73587	0.22980586	0.04961938	4.63137317	3.63E-06
13121	MAPKAPK5-A	347.724862	-0.3117091	0.12834065	-2.4287636	0.01515041
13124	MAPRE2	1328.21304	0.49339297	0.09299585	5.30553764	1.12E-07
13129	1-Mar	177.069802	-0.5930215	0.1662123	-3.567856	0.00035991
13134	2-Mar	527.015375	-0.710695	0.11127551	-6.3868053	1.69E-10
13142	MARCKS	13634.7901	-0.1954888	0.04679624	-4.1774479	2.95E-05
13145	MARK1	105.003874	-1.1593536	0.21233823	-5.4599383	4.76E-08
13146	MARK2	2782.44987	0.23053997	0.0623965	3.69475794	0.0002201
13148	MARK3	3103.21076	0.46286902	0.05815118	7.95975247	1.72E-15
13150	MARS	6605.59406	0.94913135	0.05026316	18.8832406	1.57E-79
13151	MARS2	795.512216	0.66916685	0.08353557	8.01056215	1.14E-15
13152	MARVELD1	1142.15326	-0.6892162	0.08156925	-8.449461	2.93E-17
13158	MASP2	5.74336171	2.20546683	1.06960949	2.06193649	0.03921378
13160	MAST2	2660.97839	0.17248289	0.0764087	2.25737249	0.02398481
13166	MAT2B	3336.61909	-0.1795526	0.06512806	-2.7569157	0.00583494
13168	MATN1	13.1848475	1.89420054	0.61620862	3.07395982	0.00211238
13170	MATN2	6004.73874	-0.6170067	0.0661807	-9.323061	1.13E-20
13171	MATN3	151.761864	0.5930872	0.19487001	3.04350167	0.00233842
13173	MATR3	12314.5245	0.28666069	0.05316676	5.39172807	6.98E-08
13175	MAVS	4982.30841	-0.7087352	0.06856586	-10.336561	4.82E-25
13176	MAX	3396.58991	0.66310132	0.06494871	10.2096144	1.80E-24
13178	MB	169.677666	-0.9659895	0.16128107	-5.9894784	2.11E-09
13179	MB21D1	1905.30693	0.87081342	0.07370539	11.8147866	3.27E-32
13180	MB21D2	480.495302	0.8427446	0.10335658	8.15375837	3.53E-16
13182	MBD2	2611.74504	-0.2186838	0.06289121	-3.4771763	0.00050672
13190	MBD5	290.004634	-0.4417135	0.14639399	-3.0172924	0.00255044
13195	MBLAC1	95.6778094	-0.6576383	0.22914684	-2.8699428	0.00410546

13201	MBOAT1	142.482079	-0.5761801	0.18321539	-3.1448237	0.00166187
13202	MBOAT2	1101.57681	-0.380758	0.0784668	-4.8524725	1.22E-06
13204	MBOAT7	12321.8953	-0.6274009	0.06045018	-10.378808	3.10E-25
13207	MBTPS1	4651.72542	-0.5107244	0.05410066	-9.4402626	3.72E-21
13216	MCC	567.897519	1.03165673	0.11226766	9.18926003	3.96E-20
13218	MCCC2	2356.36385	-0.2179073	0.06575338	-3.3140096	0.00091968
13231	MCL1	10165.0684	0.46411579	0.05395874	8.60130839	7.88E-18
13235	MCM3AP	2965.22616	0.20967194	0.06121367	3.42524693	0.00061424
13236	MCM3AP-AS	219.371129	0.99767207	0.1638516	6.08887613	1.14E-09
13238	MCM5	2593.36048	-1.1995224	0.0595243	-20.151808	2.59E-90
13239	MCM6	4003.20029	-0.9053186	0.06181516	-14.645576	1.44E-48
13248	MCOLN3	605.093742	-0.6789353	0.101693	-6.6763227	2.45E-11
13249	MCPH1	490.295877	0.63552352	0.1140927	5.57023823	2.54E-08
13251	MCRS1	1647.74027	-0.6120638	0.07127877	-8.5869012	8.93E-18
13255	MCTS2P	179.937983	0.70165293	0.16085363	4.3620584	1.29E-05
13256	MCU	1171.84665	-0.2628051	0.0798437	-3.2914941	0.00099657
13260	MDFIC	1744.9467	-0.4060322	0.08334174	-4.8718955	1.11E-06
13263	MDH1	5459.14376	-0.4054462	0.09149631	-4.4312844	9.37E-06
13268	MDM2	1917.79024	0.43484752	0.07393258	5.88167693	4.06E-09
13270	MDN1	2147.72687	0.54411288	0.09265526	5.87244444	4.29E-09
13274	ME2	2182.4473	0.34378063	0.06040061	5.69167446	1.26E-08
13276	MEA1	5414.14683	0.57761474	0.08009518	7.21160409	5.53E-13
13277	MEAF6	1698.20399	0.59749197	0.06762911	8.83483478	1.00E-18
13279	MECOM	2383.16073	0.41188451	0.07435936	5.53910768	3.04E-08
13282	MED1	3460.48689	0.47681101	0.10267277	4.64398713	3.42E-06
13283	MED10	2933.12821	0.92664718	0.09282646	9.98257576	1.82E-23
13287	MED13	3391.18999	0.33435749	0.09099878	3.67430759	0.0002385
13288	MED13L	3277.93136	0.67389287	0.08347356	8.07312958	6.85E-16
13289	MED14	6878.31581	-0.3639837	0.08729833	-4.1694227	3.05E-05
13290	MED14OS	46.2538846	-1.0423128	0.30606658	-3.40551	0.00066041
13291	MED15	3313.37263	0.25964949	0.06211503	4.18013952	2.91E-05
13293	MED16	2519.87418	-0.1925839	0.07057216	-2.7288931	0.00635473
13294	MED17	2394.17209	0.29756173	0.07023086	4.23690843	2.27E-05
13296	MED19	585.638969	0.32257536	0.10896024	2.96048686	0.00307153
13299	MED22	2867.49895	0.43698896	0.05897598	7.4096091	1.27E-13
13301	MED24	2626.84209	-0.2442636	0.05819634	-4.1972329	2.70E-05
13303	MED26	528.011655	0.29955857	0.1073723	2.78990566	0.00527234
13313	MED8	1514.80193	0.7268759	0.0680205	10.6861305	1.18E-26
13330	MEGF8	1554.73025	-0.9157233	0.08035506	-11.395963	4.38E-30
13331	MEGF9	1917.20292	-1.6036023	0.09265229	-17.307747	4.11E-67
13332	MEI1	49.9708916	1.54425487	0.29868679	5.17014777	2.34E-07
13343	MELK	4340.83146	-0.1944373	0.05923312	-3.2825765	0.00102863
13345	MEN1	2796.56313	-0.188733	0.06134582	-3.0765415	0.00209417

13353	MERTK	1168.79847	1.5337322	0.07691238	19.9412909	1.78E-88
13358	MEST	1158.83154	-0.3273001	0.0715072	-4.5771626	4.71E-06
13366	METTL1	439.0761	0.61830968	0.1143398	5.40765052	6.39E-08
13367	METTL10	961.128008	-0.1885984	0.08600905	-2.1927736	0.02832369
13370	METTL13	2586.83458	0.52538359	0.0669953	7.84209679	4.43E-15
13374	METTL17	1683.6711	-0.7126472	0.06745632	-10.564573	4.35E-26
13381	METTL22	308.623112	0.60703034	0.15487885	3.91938814	8.88E-05
13384	METTL25	97.6324727	-0.949031	0.20120088	-4.7168332	2.40E-06
13385	METTL2A	780.260725	0.62606937	0.08535026	7.33529505	2.21E-13
13386	METTL2B	921.650629	0.5338936	0.09204249	5.8005125	6.61E-09
13390	METTL6	795.852059	0.71151246	0.08386709	8.4838102	2.18E-17
13391	METTL7A	481.04418	-2.32773	0.11378294	-20.457637	5.14E-93
13393	METTL8	1289.48286	0.91229648	0.07887391	11.5665178	6.09E-31
13394	METTL9	2381.71571	-0.3423334	0.05801856	-5.9004122	3.63E-09
13395	MEX3A	264.947207	2.14949421	0.14139247	15.2023247	3.41E-52
13397	MEX3C	1414.19776	0.64074834	0.09285788	6.90031195	5.19E-12
13401	MFAP3	1776.8557	0.1750852	0.0836408	2.09329905	0.03632247
13402	MFAP3L	107.070997	0.90454898	0.19512488	4.63574386	3.56E-06
13405	MFF	1460.18582	-0.8051772	0.08234401	-9.7782129	1.40E-22
13406	MFGE8	3698.00998	-0.238948	0.05609003	-4.2600795	2.04E-05
13409	MFI2-AS1	53.5159676	-0.9495672	0.28439175	-3.3389408	0.00084098
13411	MFN2	5538.13793	0.13036795	0.056296	2.31575849	0.02057146
13418	MFSD2A	337.514877	1.82371575	0.13940108	13.0825081	4.15E-39
13420	MFSD3	1413.286	-0.6230804	0.10666591	-5.8414198	5.18E-09
13423	MFSD6	2367.34797	-0.6855697	0.06810928	-10.065731	7.83E-24
13425	MFSD7	512.102475	-0.5731366	0.1199358	-4.778695	1.76E-06
13428	MGA	1912.30841	0.47512041	0.08804723	5.39619938	6.81E-08
13437	MGAT4B	11813.7223	0.20982834	0.06844602	3.065603	0.00217232
13442	MGAT5B	489.412599	-0.5939794	0.11561958	-5.1373602	2.79E-07
13443	MGC12916	298.505444	-1.0128158	0.1247395	-8.1194477	4.68E-16
13459	MGC72080	283.979521	0.92300144	0.12965265	7.1190325	1.09E-12
13462	MGME1	2114.85806	0.56386301	0.06533898	8.62981038	6.15E-18
13467	MGST2	379.471096	-1.0190761	0.11409443	-8.9318651	4.19E-19
13468	MGST3	2498.9869	-0.4513378	0.08586162	-5.2565715	1.47E-07
13475	MIATNB	31.8542319	-0.7264609	0.34927738	-2.0798967	0.037535
13478	MICA	2009.60575	0.34573365	0.06154266	5.61778841	1.93E-08
13483	MICALL1	3120.60389	0.74700737	0.05840067	12.7910749	1.84E-37
13484	MICALL2	565.433992	-0.8901593	0.09988337	-8.9119878	5.01E-19
13486	MICU1	2984.90815	-0.1363531	0.06422135	-2.1231736	0.03373931
13490	MID1IP1	2546.70615	0.38675586	0.0733924	5.26969893	1.37E-07
13493	MIDN	1215.13862	0.40766318	0.07007335	5.81766331	5.97E-09
13496	MIEN1	1606.99158	-0.482338	0.10562954	-4.5663176	4.96E-06
13497	MIER1	2475.29386	0.70445167	0.07514503	9.37456078	6.95E-21

13498	MIER2	2021.65129	0.54563087	0.06901614	7.90584505	2.66E-15
13500	MIF	9708.85808	-0.4148821	0.07036827	-5.8958693	3.73E-09
13503	MIIP	806.314864	0.60983652	0.10572896	5.76792315	8.03E-09
13506	MINA	2839.54892	1.08920078	0.06159946	17.6819854	5.77E-70
13507	MINK1	4455.13689	-0.3164275	0.06840237	-4.6259725	3.73E-06
13511	MINPP1	1659.73506	-0.823507	0.0657243	-12.52972	5.13E-36
13513	MIOX	6.99948076	3.75392899	1.10569264	3.39509269	0.00068605
13516	MIPEPP3	22.5930338	1.1378019	0.43084833	2.64084093	0.00827005
13553	MIR1204	17.507434	1.56984055	0.50353309	3.11765119	0.00182298
13734	MIR17HG	233.678787	0.73522539	0.145913	5.03879285	4.68E-07
13807	MIR210HG	466.008279	-3.158007	0.1409529	-22.404698	3.54E-111
13829	MIR222	3.59746781	3.33726427	1.34692708	2.47768741	0.01322369
13835	MIR22HG	1044.83208	0.43979024	0.07879343	5.5815599	2.38E-08
13875	MIR30A	98.9974929	-10.055403	1.19731704	-8.3982796	4.53E-17
14326	MIR4449	1.36421655	3.90366386	1.96625313	1.98533129	0.04710762
14525	MIR4712	15.1805158	1.48143422	0.55986403	2.64606074	0.00814352
14661	MIR5047	187.746248	-0.3937722	0.17188951	-2.2908449	0.02197239
15269	MIR7111	22.9014488	1.00166624	0.50757595	1.97343124	0.04844645
15434	MIS12	1481.64102	0.39947274	0.07374412	5.41701099	6.06E-08
15437	MISP	296.779937	0.74830904	0.13431227	5.57141238	2.53E-08
15439	MITF	886.147236	0.81287139	0.08400029	9.67700725	3.78E-22
15441	MKI67	12279.59	-0.2401588	0.10372706	-2.3152954	0.02059677
15444	MKL2	773.425964	-0.6347979	0.10673174	-5.9476022	2.72E-09
15445	MKLN1	5387.09166	0.29747102	0.06352171	4.68298153	2.83E-06
15446	MKLN1-AS	468.731451	0.84263821	0.1184704	7.11264735	1.14E-12
15447	MKNK1	1561.13432	0.57131482	0.07202026	7.93269583	2.14E-15
15449	MKNK2	3828.88739	0.21959212	0.06043056	3.63379229	0.00027929
15453	MKRN3	6.76840597	5.21977574	1.35328673	3.85710996	0.00011474
15456	MKS1	311.677045	-0.3136172	0.12167669	-2.5774635	0.00995284
15457	MKX	183.868605	1.84137324	0.16681811	11.0382097	2.50E-28
15462	MLF1	732.079031	0.53664561	0.08781616	6.1110122	9.90E-10
15463	MLF2	4417.59423	-0.356594	0.05750905	-6.2006592	5.62E-10
15465	MLH3	921.736226	0.24280011	0.08223881	2.95237868	0.00315336
15469	MLKL	592.619344	-0.6907433	0.10532667	-6.5581047	5.45E-11
15470	MLLT1	4005.49149	0.16149667	0.05541717	2.91419901	0.00356603
15474	MLLT3	552.807272	-0.8489225	0.09843263	-8.6244009	6.44E-18
15482	MLX	2623.33484	-0.1937818	0.07469502	-2.5943071	0.00947818
15487	MMAB	538.539833	-0.3109505	0.0959711	-3.2400432	0.00119512
15488	MMACHC	403.241105	0.63626902	0.11225915	5.66785894	1.45E-08
15489	MMADHC	6055.34435	0.45655922	0.05963658	7.65569055	1.92E-14
15495	MMP1	283.683277	3.76322734	0.17443147	21.5742457	3.14E-103
15497	MMP11	169.293723	-1.8551397	0.17224068	-10.770625	4.74E-27
15500	MMP14	14319.6873	-0.2539761	0.05060925	-5.0183744	5.21E-07

15501	MMP15	617.773701	-1.9466787	0.10614002	-18.340666	3.92E-75
15510	MMP24	145.63923	-1.3343891	0.17204326	-7.7561253	8.76E-15
15511	MMP24-AS1	5831.27856	-0.2590332	0.08346472	-3.1035049	0.00191243
15516	MMP28	11.4329901	-3.7386117	0.83970957	-4.4522676	8.50E-06
15518	MMP7	7.1105404	-3.3203939	0.98007505	-3.3878975	0.00070431
15522	MMRN2	32.0560381	-1.0038634	0.34678992	-2.8947308	0.00379484
15526	MNAT1	1758.97289	0.19499126	0.07033225	2.77243044	0.00556394
15529	MNS1	154.647175	-0.4408617	0.16227934	-2.7166843	0.00659395
15533	MOAP1	1859.43691	0.51264104	0.06889928	7.44044076	1.00E-13
15537	MOB3A	3328.8491	-0.2442366	0.06097299	-4.005652	6.18E-05
15539	MOB3C	554.048002	0.54456025	0.0951263	5.72460222	1.04E-08
15542	MOCOS	864.57299	0.31758069	0.1040515	3.05214907	0.00227209
15550	MOGS	2452.44929	-0.3540378	0.06216645	-5.6949986	1.23E-08
15552	MON1A	444.247063	0.27106633	0.11355289	2.38713723	0.01698015
15556	MORC2	2304.27748	0.24880839	0.06249472	3.98127053	6.85E-05
15558	MORC3	2076.09692	0.75670472	0.08233178	9.19091875	3.90E-20
15559	MORC4	5924.87505	-0.4791934	0.05522934	-8.6764284	4.08E-18
15569	MOSPD1	2710.25606	0.64751316	0.07150513	9.05547862	1.36E-19
15571	MOSPD3	728.789992	-0.599559	0.09535528	-6.2876322	3.22E-10
15572	MOV10	1938.88937	-0.7015045	0.06543273	-10.721003	8.11E-27
15574	MOXD1	328.91456	-0.5399118	0.11828035	-4.5646787	5.00E-06
15578	MPC2	2656.24004	-0.2933246	0.08138663	-3.6040881	0.00031325
15583	MPHOSPH10	1016.42702	0.29997718	0.07837206	3.82760372	0.0001294
15584	MPHOSPH6	1050.19636	0.32638594	0.07971262	4.09453306	4.23E-05
15589	MPLKIP	1520.45586	0.31829304	0.08531843	3.73064834	0.00019099
15590	MPND	250.927152	-0.6932548	0.13804916	-5.0217968	5.12E-07
15594	MPP3	615.199458	0.98886944	0.10245787	9.65147331	4.85E-22
15597	MPP6	1061.06258	0.36338898	0.08078922	4.49798848	6.86E-06
15599	MPPE1	92.0956673	-0.9122608	0.21104101	-4.3226707	1.54E-05
15609	MPZL2	295.72617	-2.765933	0.14499231	-19.076411	3.97E-81
15610	MPZL3	138.081545	0.85886217	0.17668477	4.86098591	1.17E-06
15611	MR1	875.661539	0.5599303	0.08877664	6.30718037	2.84E-10
15614	MRAS	815.865397	0.49653215	0.08867711	5.59932729	2.15E-08
15616	MRC2	2696.83352	-0.7614022	0.0693585	-10.977778	4.89E-28
15617	MRE11A	1701.68306	-0.2920372	0.08108307	-3.6017041	0.00031614
15632	MRI1	704.079524	-0.8940705	0.0898466	-9.9510776	2.49E-23
15646	MRPL10	1806.5889	0.76270883	0.06707977	11.3701764	5.89E-30
15658	MRPL21	1529.24403	0.35227234	0.09641387	3.65375168	0.00025844
15659	MRPL22	1035.92163	-0.2396004	0.07931968	-3.0206925	0.00252197
15660	MRPL23	148.700092	-0.628824	0.16492823	-3.812713	0.00013745
15662	MRPL24	1993.79867	0.30789249	0.06857122	4.49011249	7.12E-06
15667	MRPL32	1678.32776	0.26043702	0.06839358	3.80791613	0.00014014
15671	MRPL36	1364.5913	0.35262977	0.07610229	4.63362885	3.59E-06

15672	MRPL37	3412.69881	0.29681515	0.07006384	4.23635276	2.27E-05
15673	MRPL38	1778.40002	-0.339598	0.12104298	-2.8055982	0.00502233
15675	MRPL4	2540.24527	-0.4416594	0.06379455	-6.9231526	4.42E-12
15680	MRPL43	1940.25007	-0.1948665	0.06544919	-2.9773702	0.00290733
15682	MRPL45	2113.49429	0.19880631	0.0794095	2.50355829	0.01229514
15690	MRPL52	1568.158	0.30487872	0.11315409	2.69436759	0.00705223
15693	MRPL55	636.375544	-0.5257042	0.14606841	-3.5990269	0.00031941
15696	MRPS10	4390.15051	0.61599909	0.05244641	11.7453042	7.47E-32
15708	MRPS22	1343.57146	-0.5295293	0.07950525	-6.6603057	2.73E-11
15711	MRPS25	2078.88359	0.82230281	0.07314052	11.2427808	2.51E-29
15713	MRPS27	2772.39243	0.13351899	0.06053171	2.20576918	0.02740017
15715	MRPS30	1597.91241	0.49956625	0.07532441	6.63219566	3.31E-11
15716	MRPS31	629.185394	0.237496	0.09059922	2.62139133	0.00875717
15720	MRPS35	2936.22014	0.31495873	0.06730485	4.67958462	2.87E-06
15726	MRRF	1202.72487	0.68089765	0.07607287	8.95059764	3.54E-19
15729	MRTO4	3102.09338	0.91805597	0.06927329	13.2526684	4.35E-40
15749	MSANTD3	3529.52709	0.34939991	0.06160986	5.67116857	1.42E-08
15751	MSANTD4	1622.11836	0.32202788	0.06712277	4.7975949	1.61E-06
15755	MSH2	2892.01306	0.29471654	0.06724864	4.3824904	1.17E-05
15764	MSL2	1028.62564	0.51988805	0.07761206	6.69854757	2.11E-11
15774	MSRB1	458.409765	-0.4396139	0.12145874	-3.6194501	0.00029523
15775	MSRB2	971.777422	-0.3391006	0.09938349	-3.4120418	0.00064478
15777	MSS51	60.7952243	-0.8625988	0.32714825	-2.6367215	0.00837115
15778	MST1	63.1491285	-1.1417324	0.26348246	-4.3332386	1.47E-05
15780	MST1P2	20.9895104	-1.0766711	0.52558737	-2.0485102	0.04051003
15781	MST1R	1015.52004	-0.6450049	0.08590112	-7.5086903	5.97E-14
15786	MSX2	70.375667	1.26699904	0.28083045	4.51161568	6.43E-06
15788	MT1A	93.8926186	-1.6226496	0.26909651	-6.0299911	1.64E-09
15792	MT1F	114.693534	-0.7350658	0.22565025	-3.2575447	0.00112381
15799	MT1M	4.43060217	-2.1104557	1.07364328	-1.9656954	0.0493338
15805	MTA2	5486.92977	0.21652156	0.05831533	3.71294408	0.00020486
15806	MTA3	1167.77185	-0.3283876	0.08125533	-4.0414286	5.31E-05
15809	MTCH1	24887.8121	0.35206222	0.0734445	4.79358211	1.64E-06
15810	MTCH2	6160.38819	-0.406331	0.07778519	-5.2237577	1.75E-07
15816	MTERF3	1079.75235	0.31907811	0.08223224	3.88020679	0.00010437
15818	MTF1	811.898135	0.85540212	0.1062563	8.05036633	8.25E-16
15820	MTFMT	441.443419	-0.3870438	0.10991269	-3.5213746	0.00042932
15825	MTG1	1130.77897	-0.2748576	0.08777815	-3.131276	0.00174049
15826	MTG2	1992.69292	0.44506128	0.07338906	6.06440917	1.32E-09
15829	MTHFD2	2434.1176	1.33575972	0.06108959	21.8655849	5.53E-106
15840	MTMR10	759.905551	-0.2863935	0.10089279	-2.8385923	0.0045313
15841	MTMR11	514.907104	-0.3751346	0.10697449	-3.5067674	0.00045359
15844	MTMR2	3536.93174	0.18906523	0.06811232	2.77578604	0.00550684

15846	MTMR4	1719.47577	-0.7429523	0.07244744	-10.255052	1.12E-24
15849	MTMR8	41.0323052	-0.9130569	0.31505965	-2.8980445	0.00375497
15850	MTMR9	646.749033	0.64055035	0.09358545	6.84455054	7.67E-12
15851	MTMR9LP	99.2172331	-1.0649218	0.21807427	-4.8832986	1.04E-06
15854	MTO1	1761.37675	0.28667504	0.07199132	3.98207807	6.83E-05
15855	MTOR	2661.46808	0.71056973	0.09986017	7.11564678	1.11E-12
15857	MTPAP	1628.80947	0.59413408	0.06862692	8.65744936	4.82E-18
15861	MTRF1L	389.751824	0.57529992	0.13301005	4.3252366	1.52E-05
15872	MTRR	2641.1246	0.72264584	0.07187862	10.0536968	8.85E-24
15873	MTSS1	30.6984442	0.94851707	0.35796063	2.64978041	0.00805441
15874	MTSS1L	4072.74291	0.2196907	0.06010239	3.65527385	0.00025691
15881	MTX1	548.331845	-0.2849862	0.11194383	-2.5457967	0.01090287
15882	MTX2	2618.15355	-0.4104846	0.06978838	-5.8818468	4.06E-09
15884	MUC1	617.202946	-1.9322566	0.11613631	-16.637833	3.71E-62
15892	MUC20	20.4656912	1.03961801	0.52604096	1.97630621	0.04812011
15907	MUSK	45.7001812	2.18790334	0.32803604	6.6697041	2.56E-11
15912	MVB12B	506.141066	-0.6097102	0.09858248	-6.1847719	6.22E-10
15913	MVD	862.717343	-0.5439033	0.11261171	-4.8298999	1.37E-06
15915	MVP	6418.10526	-0.2147394	0.06914224	-3.1057625	0.00189789
15918	MXD1	786.416463	1.55111879	0.09819671	15.7960369	3.31E-56
15919	MXD3	647.107504	-1.0227908	0.11917096	-8.5825505	9.28E-18
15920	MXD4	1467.32277	-1.6057869	0.07451117	-21.550954	5.19E-103
15921	MXI1	2061.45821	-0.3299445	0.0935716	-3.5261178	0.0004217
15923	MXRA7	3061.99048	-0.4163541	0.05932057	-7.0187151	2.24E-12
15924	MXRA8	1984.77981	-0.8064729	0.06792414	-11.87314	1.63E-32
15929	MYBBP1A	3900.46825	0.51948221	0.06078454	8.54628816	1.27E-17
15930	MYBL1	2787.80558	0.18665144	0.07529365	2.47897998	0.01317587
15937	MYC	11118.1171	1.40453204	0.05022237	27.9662614	4.18E-172
15939	MYCBP2	1925.69246	0.25278797	0.07791076	3.24458369	0.00117622
15949	MYEF2	1576.31702	-0.3355236	0.06946458	-4.8301397	1.36E-06
15950	MYEOV	6807.3473	0.50453417	0.05050984	9.98882885	1.71E-23
15951	MYEOV2	761.508823	-0.43424	0.14392394	-3.017149	0.00255164
15959	MYH15	925.773944	-0.5590501	0.08116273	-6.8880152	5.66E-12
15978	MYL6	19119.6856	-0.6183751	0.10410468	-5.9399352	2.85E-09
15981	MYL9	7338.54587	-0.3220042	0.07364554	-4.3723521	1.23E-05
15983	MYLK	4228.74665	-0.3429523	0.08847459	-3.8762804	0.00010607
15987	MYLK3	7.84870697	-2.0883306	0.8717653	-2.3955193	0.01659684
15990	MYNN	1107.06615	0.44656457	0.07469963	5.9781364	2.26E-09
15991	MYO10	5553.34367	-0.2790759	0.06607884	-4.2233779	2.41E-05
15993	MYO15B	88.5292022	-0.6404845	0.21537433	-2.9738199	0.00294118
15996	MYO18A	4673.67305	-1.5279338	0.08646692	-17.670733	7.05E-70
16002	MYO1D	34.6792254	1.42300544	0.36575587	3.89058811	0.0001
16029	MYPN	140.662782	3.1942821	0.23239084	13.7453015	5.43E-43

16040	MZF1	564.774432	-0.7487817	0.10375036	-7.2171481	5.31E-13
16042	MZT1	1695.67853	0.42899416	0.07564794	5.67092976	1.42E-08
16043	MZT2A	987.113439	-0.5147175	0.14518057	-3.5453608	0.00039208
16044	MZT2B	3270.27849	-0.4375383	0.12533765	-3.4908766	0.00048144
16046	N4BP2	392.980337	0.67921955	0.13353695	5.08637898	3.65E-07
16048	N4BP2L2	1340.36878	0.23048546	0.07693602	2.99580686	0.0027372
16053	NAA10	2392.78788	0.20793646	0.07323788	2.83919273	0.00452278
16055	NAA15	5690.51887	0.68728303	0.05537725	12.4109274	2.28E-35
16058	NAA25	2506.50613	0.17088451	0.07245576	2.35846685	0.0183506
16059	NAA30	1588.0249	0.45744837	0.06777268	6.74974602	1.48E-11
16060	NAA35	1542.10997	0.43638151	0.07075983	6.1670792	6.96E-10
16063	NAA50	6244.74044	0.45877313	0.05459221	8.40363766	4.33E-17
16064	NAA60	1369.38213	0.65741637	0.08484684	7.74827168	9.32E-15
16065	NAAA	818.05201	-0.3592467	0.08424256	-4.2644322	2.00E-05
16076	NACA	9487.65103	0.21851368	0.05788582	3.77490872	0.00016007
16078	NACAD	88.0672796	-0.8034411	0.22010454	-3.6502706	0.00026196
16084	NADSYN1	1317.54808	-0.3975794	0.08087894	-4.9157351	8.84E-07
16086	NAF1	681.702725	0.74075565	0.09120215	8.12212955	4.58E-16
16087	NAGA	950.673739	-0.3054274	0.08169395	-3.738679	0.00018499
16090	NAGPA	434.123631	-0.763498	0.10620276	-7.1890601	6.52E-13
16092	NAGS	507.08163	1.67908877	0.10229926	16.4134981	1.53E-60
16095	NALCN	72.4082542	-1.1495724	0.24647992	-4.6639595	3.10E-06
16098	NAMPT	25001.0323	1.07709308	0.05412385	19.9005256	4.03E-88
16101	NANOS1	1096.1381	-2.1631966	0.11269481	-19.195176	4.06E-82
16104	NANP	875.803349	-0.3714875	0.08314598	-4.4678948	7.90E-06
16105	NANS	2166.95528	0.22561226	0.0779742	2.89342175	0.00381069
16109	NAP1L4	5469.83247	-0.1811739	0.05680437	-3.1894355	0.00142551
16115	NAPEPLD	289.824128	-0.5657056	0.13414471	-4.2171294	2.47E-05
16120	NARF	1370.27325	-1.0535016	0.07991444	-13.182869	1.10E-39
16123	NARS	10520.2795	0.63306616	0.0523391	12.095472	1.12E-33
16124	NARS2	1011.28392	0.76509377	0.07791695	9.81934992	9.29E-23
16126	NAT1	289.765943	-0.897026	0.1268862	-7.0695314	1.55E-12
16127	NAT10	3493.88547	0.41267864	0.05431945	7.59725315	3.02E-14
16128	NAT14	1408.09579	-0.6079159	0.11855771	-5.1275945	2.93E-07
16130	NAT2	3.21055828	4.12494976	1.52743414	2.70057455	0.00692198
16131	NAT6	297.142064	-0.4969289	0.1234242	-4.0261867	5.67E-05
16135	NAT9	2152.09002	0.78170873	0.0758531	10.3055608	6.65E-25
16136	NATD1	731.494075	0.91565141	0.09444428	9.69514911	3.16E-22
16142	NAV3	1080.68942	0.74293007	0.09641649	7.70542564	1.30E-14
16147	NBEAL2	1585.7787	-0.6222191	0.0693158	-8.976585	2.79E-19
16150	NBN	2506.34163	-0.1751601	0.07256309	-2.4139002	0.01578278
16151	NBPF1	317.229631	0.71391026	0.13301912	5.36697458	8.01E-08
16156	NBPF14	197.107552	-0.4974929	0.15811316	-3.1464357	0.00165274

16175	NCAPD2	5052.94667	-0.7274586	0.05660715	-12.851003	8.49E-38
16176	NCAPD3	4253.50969	-0.3908265	0.07384755	-5.2923422	1.21E-07
16179	NCAPH	2650.72147	-0.5030434	0.06298586	-7.9866084	1.39E-15
16181	NCBP1	2515.8108	0.21214529	0.0585857	3.62111078	0.00029334
16182	NCBP2	4000.86864	0.19146602	0.0609158	3.14312556	0.00167154
16183	NCBP2-AS2	575.053191	-0.6337423	0.11458389	-5.5308154	3.19E-08
16192	NCK1	1158.90025	0.21107763	0.07131141	2.95994171	0.00307697
16198	NCKAP5L	1253.11181	0.27160878	0.07567048	3.58936224	0.00033149
16199	NCKIPSD	1868.50904	-0.4767619	0.06263324	-7.6119631	2.70E-14
16200	NCL	31887.9714	0.44767261	0.06027887	7.42669253	1.11E-13
16201	NCLN	5046.86804	-0.5118889	0.07383301	-6.9330636	4.12E-12
16205	NCOA3	2679.5967	0.21951377	0.0815849	2.69061767	0.00713199
16208	NCOA6	3147.24423	0.25188246	0.08504166	2.96187134	0.00305776
16213	NCOR2	4339.50044	0.35972991	0.08415638	4.27454108	1.92E-05
16221	NCSTN	5198.39228	-0.1803983	0.06196881	-2.9111143	0.00360142
16224	NDE1	974.206357	-0.2527051	0.07764711	-3.2545327	0.00113579
16225	NDEL1	1825.89131	0.21924869	0.0791432	2.77027819	0.00560084
16233	NDP	23.212305	-1.273157	0.42549046	-2.9922104	0.00276965
16237	NDRG4	59.6613793	-2.2060802	0.28576992	-7.7197776	1.17E-14
16238	NDST1	7547.19579	-0.196904	0.07790032	-2.5276399	0.01148321
16239	NDST2	748.401772	-0.4294896	0.09036541	-4.7528093	2.01E-06
16245	NDUFA12	2315.49383	-0.3043248	0.06567891	-4.6335238	3.59E-06
16247	NDUFA2	1592.36073	-0.4062058	0.09288431	-4.3732444	1.22E-05
16248	NDUFA3	570.123896	-0.2445071	0.11425898	-2.1399375	0.03235982
16249	NDUFA4	2685.93332	-0.2238019	0.06670703	-3.354997	0.00079366
16252	NDUFA6	1689.74343	0.23357286	0.0826822	2.82494718	0.00472884
16255	NDUFA8	2623.66342	-0.4750975	0.10409771	-4.5639579	5.02E-06
16256	NDUFA9	1717.20767	-0.6419047	0.08294378	-7.7390337	1.00E-14
16261	NDUFAF4	1042.41263	0.86097088	0.07538605	11.4208245	3.29E-30
16263	NDUFAF5	529.278628	0.24588474	0.10757619	2.28567984	0.022273
16267	NDUFB10	1410.07471	-0.3686478	0.07971015	-4.6248546	3.75E-06
16272	NDUFB4	1926.74669	-0.2788516	0.0623204	-4.4744827	7.66E-06
16273	NDUFB5	2391.23903	-0.7777388	0.07158774	-10.864134	1.71E-27
16274	NDUFB6	2022.65897	-0.4343023	0.08634631	-5.029773	4.91E-07
16275	NDUFB7	1998.93401	-0.572954	0.12277623	-4.6666525	3.06E-06
16278	NDUFC1	1023.01984	-0.650589	0.13432721	-4.8433148	1.28E-06
16283	NDUFS3	1907.34335	-0.9206542	0.0948254	-9.708941	2.76E-22
16286	NDUFS6	2140.82616	-0.4303877	0.09974084	-4.31506	1.60E-05
16289	NDUFV1	5915.85146	-0.4077222	0.10561502	-3.860457	0.00011318
16292	NDUFV3	1378.16468	0.73319333	0.06988251	10.4918007	9.42E-26
16294	NEB	27.1829779	0.90087498	0.41243037	2.18430803	0.02893962
16299	NECAB3	1087.60478	-0.359781	0.08890224	-4.0469282	5.19E-05
16301	NECAP2	4077.041	0.39151644	0.0627877	6.23555979	4.50E-10

16302	NEDD1	2831.5978	0.2595933	0.06709331	3.86913834	0.00010922
16304	NEDD4L	13301.6173	0.92155186	0.06082382	15.1511668	7.44E-52
16311	NEGR1	110.008141	0.86279054	0.21364727	4.0383879	5.38E-05
16313	NEIL1	168.30847	-1.465711	0.16795312	-8.7269056	2.62E-18
16319	NEK2	1749.3857	-0.2052125	0.06916797	-2.9668718	0.00300846
16323	NEK6	4347.83265	-0.404524	0.05405109	-7.4841041	7.20E-14
16326	NEK9	2690.82513	0.36596097	0.06485532	5.64272883	1.67E-08
16327	NELFA	1073.6801	-0.7051194	0.09348573	-7.542535	4.61E-14
16328	NELFB	4766.51143	-0.7184316	0.05599632	-12.82998	1.11E-37
16330	NELFE	4456.49455	0.5157961	0.06223149	8.28834493	1.15E-16
16338	NES	639.524282	-0.9955571	0.10061727	-9.8944949	4.40E-23
16348	NEURL1B	196.748577	-0.5830169	0.15696866	-3.7142251	0.00020383
16359	NEXN	896.960044	0.55897993	0.08575997	6.5179587	7.13E-11
16361	NF1	1603.48599	0.77290157	0.12559632	6.15385516	7.56E-10
16363	NF2	261.891449	0.32637916	0.13747219	2.37414679	0.01758956
16366	NFAT5	3327.76879	0.26242086	0.10215121	2.5689452	0.01020086
16367	NFATC1	103.025341	1.70082316	0.21735339	7.82515137	5.07E-15
16368	NFATC2	2015.73542	0.87934309	0.06938088	12.6741409	8.23E-37
16370	NFATC3	1059.99757	0.18214041	0.08981415	2.02797016	0.04256329
16372	NFE2	16.7163682	-3.4048549	0.65053264	-5.2339494	1.66E-07
16374	NFE2L2	7329.40253	0.20207343	0.06054778	3.33742117	0.0008456
16382	NFIL3	1706.92304	1.31750257	0.06832419	19.2831063	7.45E-83
16384	NFKB1	3127.43824	0.24174068	0.0588981	4.1043883	4.05E-05
16387	NFKBIB	1521.46052	1.23021991	0.09004142	13.6628229	1.69E-42
16389	NFKBIE	3267.87025	1.44494819	0.0947304	15.2532681	1.57E-52
16390	NFKBIL1	1335.96886	0.92875889	0.09181455	10.1155963	4.71E-24
16393	NFS1	871.924421	-0.5821786	0.0849595	-6.8524246	7.26E-12
16395	NFX1	2494.77996	0.51557179	0.06573347	7.8433681	4.39E-15
16396	NFXL1	1179.48199	0.8309583	0.07345901	11.3118631	1.15E-29
16398	NFYB	1517.45035	-0.4127919	0.07176174	-5.7522566	8.81E-09
16399	NFYC	2229.45773	1.07826041	0.08476213	12.7210161	4.52E-37
16402	NGDN	1422.59837	0.3120065	0.09747683	3.20082716	0.00137034
16403	NGEF	487.658929	-0.3621807	0.10554696	-3.431465	0.00060033
16404	NGF	450.449339	1.22869363	0.12705029	9.67092338	4.01E-22
16406	NGFRAP1	5730.6697	-0.8014594	0.19086898	-4.1990031	2.68E-05
16407	NGLY1	1599.52403	0.23890872	0.06960254	3.43247136	0.00059811
16410	NHEJ1	451.151948	0.24482906	0.10437841	2.34559097	0.01899694
16414	NHLRC2	1484.34137	0.49637402	0.11366941	4.36682161	1.26E-05
16415	NHLRC3	674.160332	-0.5266478	0.08853566	-5.9484262	2.71E-09
16418	NHS	758.997643	0.27956793	0.09815656	2.84818381	0.00439695
16422	NICN1	807.632583	-0.3565114	0.08063047	-4.4215466	9.80E-06
16425	NIF3L1	1440.62138	-0.4275213	0.0705706	-6.0580647	1.38E-09
16426	NIFK	2478.63852	0.87429869	0.07286794	11.9984004	3.62E-33

16430	NINJ1	1144.36259	-0.9722909	0.07859128	-12.371486	3.73E-35
16431	NINJ2	203.282476	-1.1871461	0.15913198	-7.4601353	8.64E-14
16432	NINL	1122.42832	-0.4317089	0.09355107	-4.6146869	3.94E-06
16433	NIP7	2092.21138	0.86770119	0.06717901	12.9162546	3.64E-38
16442	NIPSNAP1	1156.49467	-1.0332007	0.07730624	-13.365036	9.68E-41
16443	NIPSNAP3A	1015.27231	-0.4074166	0.09242744	-4.4079619	1.04E-05
16445	NISCH	2701.90297	-0.3265027	0.05975022	-5.4644606	4.64E-08
16448	NKAIN1	7.06814265	2.91395209	0.94064614	3.09781963	0.0019495
16452	NKAP	667.547182	-1.0650886	0.10021721	-10.627801	2.21E-26
16454	NKAPP1	18.0722728	-1.9579956	0.53358117	-3.6695365	0.00024299
16459	NKIRAS1	1138.06626	0.57088906	0.08050422	7.09141791	1.33E-12
16465	NKX1-2	2.19965534	4.62196417	1.76129207	2.62418951	0.00868554
16471	NKX2-5	23.131335	-1.269293	0.43448924	-2.9213452	0.00348523
16474	NKX3-1	1181.18766	0.61458303	0.08678723	7.08149112	1.43E-12
16482	NLGN2	1927.63063	-0.578674	0.06570724	-8.8068524	1.29E-18
16488	NLN	2136.42441	0.41918928	0.07441703	5.63297498	1.77E-08
16491	NLRC5	1279.36583	-0.8524705	0.08059114	-10.577721	3.78E-26
16494	NLRP11	10.2223517	-1.7288645	0.66195197	-2.6117673	0.00900755
16506	NLRX1	857.10758	-0.4743681	0.08501788	-5.5796271	2.41E-08
16507	NMB	347.137921	-1.1098251	0.13471364	-8.2384016	1.75E-16
16509	NMD3	2236.06405	0.97087439	0.06609874	14.6882425	7.67E-49
16510	NME1	3150.28602	0.4985981	0.12230162	4.07679056	4.57E-05
16513	NME3	542.602024	-1.03859	0.14153883	-7.3378449	2.17E-13
16514	NME4	1278.57983	-0.576959	0.08702763	-6.6296069	3.37E-11
16516	NME6	1076.76354	0.61800435	0.07378003	8.37630935	5.46E-17
16517	NME7	2216.75731	0.52502913	0.07681164	6.83528064	8.18E-12
16520	NMI	902.683251	-0.3604149	0.08293244	-4.3458855	1.39E-05
16521	NMNAT1	475.605199	0.42920884	0.10985591	3.90701635	9.34E-05
16524	NMRAL1	1046.36799	-0.4238743	0.09248779	-4.5830306	4.58E-06
16534	NNMT	2293.43682	-1.3400684	0.09076698	-14.763831	2.51E-49
16535	NNT	4801.18183	-0.3415239	0.06857647	-4.98019	6.35E-07
16536	NNT-AS1	850.057367	0.6579782	0.101594	6.47654554	9.38E-11
16537	NOA1	1162.2225	-0.4017472	0.07744749	-5.1873496	2.13E-07
16540	NOC2L	3512.8222	0.30104218	0.0691432	4.35389404	1.34E-05
16541	NOC3L	2000.13632	0.95853622	0.08103531	11.8286241	2.78E-32
16543	NOCT	931.019923	1.72545561	0.1197832	14.404821	4.83E-47
16544	NOD1	348.273533	-0.3534594	0.11913481	-2.9668861	0.00300832
16545	NOD2	198.809873	0.82519272	0.14766377	5.58832208	2.29E-08
16548	NOL10	1954.62386	0.50194494	0.06668669	7.52691337	5.20E-14
16549	NOL11	2616.20023	0.6211112	0.07856378	7.90582111	2.66E-15
16554	NOL6	4333.37497	0.68776759	0.05875757	11.7051737	1.20E-31
16555	NOL7	5105.70731	0.42880486	0.07541915	5.68562316	1.30E-08
16558	NOLC1	7600.37185	0.39231485	0.05934195	6.61108768	3.82E-11

16563	NONO	10596.5455	-0.4892834	0.05819368	-8.4078438	4.18E-17
16565	NOP14	4662.74362	0.26958205	0.05389877	5.0016362	5.68E-07
16567	NOP16	2089.71525	0.84181897	0.08520964	9.87938653	5.11E-23
16568	NOP2	2360.35758	0.57472111	0.06466834	8.88720925	6.27E-19
16569	NOP56	6839.63253	0.63697719	0.05724917	11.1264001	9.33E-29
16570	NOP58	4582.98928	0.99630665	0.05388735	18.4886931	2.55E-76
16576	NOSIP	1543.49532	-0.4889075	0.10066063	-4.8569888	1.19E-06
16577	NOSTRIN	71.0300643	-0.5993817	0.23321764	-2.570053	0.0101683
16578	NOTCH1	2700.45635	0.95784176	0.07792412	12.2919799	1.00E-34
16585	NOV	408.910137	0.69308842	0.10858689	6.38280028	1.74E-10
16589	NOX3	2.88823632	3.94923129	1.58508231	2.49149919	0.01272053
16592	NOXA1	1035.30297	-0.9284348	0.10706057	-8.6720524	4.24E-18
16597	NPAS2	2878.26804	-1.0899835	0.07903886	-13.790476	2.91E-43
16604	NPC1	10335.5556	1.17108519	0.06397057	18.306624	7.33E-75
16606	NPC2	7384.14234	-0.8055254	0.05698085	-14.136775	2.25E-45
16634	NPL	12.5668377	1.76139336	0.65354952	2.69511844	0.00703636
16636	NPM1	15580.8732	0.6284658	0.05474208	11.4804875	1.65E-30
16641	NPPA-AS1	54.4322857	0.80002517	0.27333846	2.9268665	0.00342396
16644	NPR1	24.5703481	-1.720217	0.44769198	-3.8424119	0.00012183
16648	NPRL3	1529.36295	-0.4514864	0.06816848	-6.6230955	3.52E-11
16652	NPTN	5330.87563	-0.5606643	0.05825059	-9.6250405	6.27E-22
16656	NPTXR	383.352923	-0.2875861	0.12562505	-2.2892414	0.02206533
16669	NR1D1	1510.84946	2.05728354	0.08039265	25.5904426	1.95E-144
16670	NR1D2	4372.31553	0.86686511	0.07169765	12.0905644	1.18E-33
16671	NR1H2	2951.2973	-0.8562862	0.05694617	-15.036764	4.22E-51
16672	NR1H3	426.975395	-1.0137611	0.12182024	-8.3217787	8.67E-17
16676	NR2C1	962.067232	-0.3678837	0.09166812	-4.0132126	5.99E-05
16678	NR2C2AP	989.219906	0.46806733	0.09303423	5.03113015	4.88E-07
16683	NR2F2	7006.0377	-1.0165016	0.05150974	-19.734165	1.10E-86
16685	NR2F6	2646.70935	-1.0746128	0.09295658	-11.560374	6.54E-31
16688	NR4A1	61.3142056	1.37500231	0.2649087	5.19047625	2.10E-07
16701	NRBP2	1507.13122	-0.2918575	0.08652144	-3.373239	0.00074289
16703	NRD1	7557.90809	0.18355411	0.05117364	3.58688807	0.00033465
16705	NREP	450.938565	-1.1978291	0.11196249	-10.698485	1.03E-26
16708	NRG1	3947.30789	1.67811164	0.0611516	27.4418266	8.70E-166
16711	NRG2	19.6614562	-1.8726603	0.51042749	-3.6688076	0.00024368
16715	NRGN	935.982561	-0.4490721	0.10617111	-4.2297011	2.34E-05
16716	NRIP1	1787.19263	0.57053507	0.08739958	6.52789276	6.67E-11
16722	NRM	1405.33383	-0.3498212	0.07790278	-4.490485	7.11E-06
16727	NRP2	2152.61616	-1.115222	0.084495	-13.198675	8.93E-40
16728	NRROS	337.222572	-0.3899312	0.12116433	-3.218201	0.00128997
16730	NRSN2	2613.476	-0.1503906	0.06251037	-2.4058507	0.01613486
16732	NRTN	78.059088	-2.3371133	0.31070029	-7.5220828	5.39E-14

16736	NSA2	2529.51728	-0.1778557	0.06757081	-2.632138	0.00848494
16738	NSDHL	1132.11606	-0.4653873	0.08056573	-5.7764924	7.63E-09
16744	NSMAF	2622.79775	0.88346827	0.06311501	13.9977525	1.61E-44
16746	NSMCE2	226.621509	-0.3665021	0.13602834	-2.6943069	0.00705352
16747	NSMCE4A	1259.90294	-0.2799136	0.07779929	-3.5978936	0.0003208
16749	NSRP1	2220.34995	0.55987909	0.06874777	8.14396039	3.83E-16
16750	NSUN2	7154.71646	0.38971538	0.04967254	7.84569072	4.31E-15
16752	NSUN4	618.239208	1.0097568	0.09340166	10.8109081	3.06E-27
16753	NSUN5	1116.57103	0.50911962	0.10818762	4.70589524	2.53E-06
16754	NSUN5P1	234.751574	-0.4158173	0.14118565	-2.945181	0.00322766
16755	NSUN5P2	288.832344	-0.570336	0.15421461	-3.6983266	0.00021703
16756	NSUN6	437.135182	0.60171696	0.10306377	5.83829777	5.27E-09
16762	NT5C2	6541.64774	-0.2659386	0.07297633	-3.6441764	0.00026825
16763	NT5C3A	1711.83302	0.69015131	0.07358423	9.37906563	6.66E-21
16765	NT5DC1	584.413256	-0.6032529	0.10569148	-5.7076774	1.15E-08
16767	NT5DC3	1374.60369	-0.7158886	0.07515909	-9.5249771	1.65E-21
16771	NTF3	8.16720065	1.77472846	0.83963205	2.11369784	0.03454108
16773	NTHL1	537.502011	-0.7677156	0.16103702	-4.7673237	1.87E-06
16776	NTMT1	1890.1546	0.4341935	0.07813896	5.55668389	2.75E-08
16793	NUB1	5397.97659	0.25320436	0.06018464	4.20712573	2.59E-05
16795	NUBP2	1391.48477	-0.6950312	0.09732838	-7.1410943	9.26E-13
16796	NUBPL	355.931982	-0.468611	0.11305835	-4.1448598	3.40E-05
16797	NUCB1	5718.61168	-0.6516881	0.05235249	-12.448082	1.43E-35
16802	NUDCD1	5364.07052	0.50193341	0.06961028	7.2106221	5.57E-13
16803	NUDCD2	1734.06038	-0.3310868	0.08069969	-4.1027019	4.08E-05
16804	NUDCD3	4388.0965	-0.2438048	0.06477559	-3.7638383	0.00016733
16805	NUDT1	739.49684	-0.3929896	0.11233395	-3.4984043	0.00046805
16808	NUDT12	557.234453	-0.4221691	0.10077219	-4.1893416	2.80E-05
16810	NUDT14	933.317995	-0.3337698	0.13439623	-2.4834759	0.01301071
16812	NUDT16	1000.39799	0.17997011	0.07556677	2.38160374	0.01723743
16813	NUDT16L1	489.684637	-0.9229423	0.13452244	-6.86088	6.84E-12
16818	NUDT2	485.76772	-0.3027451	0.11042808	-2.7415589	0.00611484
16820	NUDT22	1406.8932	-0.4052235	0.10528958	-3.8486573	0.00011877
16822	NUDT4	612.819488	0.20936131	0.09488173	2.20655037	0.02734549
16825	NUDT5	2573.80469	0.17172468	0.06938435	2.47497723	0.01332447
16826	NUDT6	555.084184	-0.461334	0.09887982	-4.6656032	3.08E-06
16831	NUF2	1798.34085	-0.2027763	0.07785985	-2.6043759	0.00920418
16832	NUFIP1	541.698697	0.64055043	0.09751888	6.56847611	5.08E-11
16833	NUFIP2	5835.54472	0.36194727	0.08349057	4.33518723	1.46E-05
16835	NUMA1	8460.65121	-0.6553555	0.07622414	-8.5977421	8.13E-18
16837	NUMBL	910.642332	-0.2938054	0.08356873	-3.5157334	0.00043854
16839	NUP133	3018.13423	-0.1790901	0.06203436	-2.8869499	0.00388996
16840	NUP153	7725.87335	0.70176021	0.0705383	9.94864101	2.56E-23

16842	NUP160	3310.61183	0.40271278	0.06508984	6.18702961	6.13E-10
16845	NUP210	1962.80559	-0.2405482	0.08071197	-2.9803283	0.0028794
16849	NUP35	1154.55315	0.4238476	0.07883262	5.37655071	7.59E-08
16851	NUP43	2607.90417	0.40176917	0.05897557	6.81246742	9.59E-12
16853	NUP50-AS1	800.336418	1.2352347	0.08333528	14.8224699	1.05E-49
16855	NUP62	3328.917	-0.3804561	0.06487868	-5.864117	4.52E-09
16858	NUP88	3102.19636	0.28897218	0.05606218	5.15449375	2.54E-07
16859	NUP93	2427.47914	0.19618421	0.06809799	2.88091031	0.00396528
16860	NUP98	6757.5977	0.43509552	0.06938898	6.27038348	3.60E-10
16861	NUPL1	2737.45894	0.43458208	0.08036236	5.40778146	6.38E-08
16862	NUPL2	877.509143	0.43652826	0.08893691	4.90829127	9.19E-07
16863	NUPR1	1391.18922	1.39446139	0.10010937	13.929379	4.20E-44
16866	NUSAP1	3992.32715	-0.3552831	0.08349926	-4.2549246	2.09E-05
16872	NUTM2B-AS	91.5525107	1.23881199	0.26267901	4.71606766	2.40E-06
16877	NWD1	55.9129198	-1.3882684	0.31084009	-4.4661819	7.96E-06
16879	NXF1	2906.76492	0.14952348	0.06149885	2.4313215	0.01504386
16895	NXPH4	56.7329299	0.54899556	0.26359982	2.08268565	0.03727989
16899	NYAP2	32.0953438	0.72419121	0.34470124	2.10092427	0.03564761
16903	OAF	3244.25254	0.5573473	0.05584814	9.97969373	1.87E-23
16904	OARD1	2291.87598	0.6688883	0.08795436	7.60494806	2.85E-14
16905	OAS1	77.528888	-1.4959494	0.25765055	-5.8061177	6.39E-09
16906	OAS2	869.30418	-0.1840904	0.08439495	-2.1812966	0.02916149
16909	OAT	6677.68618	-0.5570611	0.05729972	-9.7218814	2.43E-22
16911	OAZ2	2279.81889	-0.5328603	0.06842309	-7.7877257	6.82E-15
16913	OBFC1	1087.39137	-0.1535109	0.07511533	-2.0436689	0.04098626
16917	OBSL1	2504.54675	-1.0832078	0.08763736	-12.360115	4.30E-35
16920	OCEL1	755.749732	-0.5558376	0.10403906	-5.3425858	9.16E-08
16924	OCLN	156.899362	0.35400043	0.16975603	2.08534818	0.03703771
16930	ODC1	6260.00801	1.7819619	0.07792034	22.869022	9.45E-116
16933	ODF2L	1678.25014	0.37825302	0.08831209	4.28313954	1.84E-05
16942	OGFOD1	2015.70275	0.92507967	0.07442918	12.4289919	1.82E-35
16944	OGFOD3	1470.78547	-0.519852	0.09986084	-5.2057642	1.93E-07
16949	OGG1	755.254969	-0.1779309	0.08836178	-2.0136631	0.04404492
16951	OGT	10539.2083	0.56020864	0.06018958	9.30740235	1.31E-20
16952	OIP5	547.439559	-0.3607868	0.11838124	-3.0476692	0.00230624
16953	OIP5-AS1	741.043631	-0.2126337	0.08782497	-2.4211081	0.01547327
16955	OLA1	5867.78548	0.43762658	0.05405823	8.09546629	5.70E-16
16958	OLFM2	20.5612334	2.4024548	0.48878289	4.91517773	8.87E-07
16962	OLFML2A	1839.37534	-1.0159426	0.07041239	-14.428463	3.43E-47
16975	ONECUT2	121.899153	0.96987088	0.22922269	4.23112945	2.33E-05
16985	OPHN1	1037.4343	0.41575218	0.08121822	5.11895216	3.07E-07
16986	OPLAH	592.095447	-0.6733311	0.09697197	-6.9435638	3.82E-12
16996	OPRL1	160.233763	-0.5277539	0.16445613	-3.209086	0.00133158

17072	OR1G1	3.56673794	-5.264524	1.5052886	-3.497352	0.0004699
17402	ORAI3	1157.64575	-0.7914298	0.07939663	-9.9680531	2.10E-23
17403	ORAOV1	1549.45756	0.75765178	0.07803746	9.70882211	2.77E-22
17407	ORC4	2390.76588	0.35892899	0.07495774	4.7884177	1.68E-06
17408	ORC5	1174.87445	0.58992021	0.07827067	7.5369257	4.81E-14
17409	ORC6	965.313015	0.48541907	0.08077481	6.00953561	1.86E-09
17416	OSBP	4129.51292	0.56316539	0.06695666	8.41089386	4.07E-17
17418	OSBPL10	2130.06582	0.33786626	0.06599877	5.11928081	3.07E-07
17424	OSBPL5	566.768714	-0.9989949	0.1001437	-9.975614	1.95E-23
17426	OSBPL7	408.268825	-0.8056752	0.10678801	-7.544622	4.54E-14
17427	OSBPL8	5149.53383	0.27581051	0.0954066	2.89089538	0.00384146
17428	OSBPL9	4562.08025	0.25237228	0.07169368	3.52014666	0.00043131
17433	OSGEP	1105.6816	-0.2849756	0.10087957	-2.8249089	0.00472941
17434	OSGEPL1	437.969716	-0.8461109	0.10347881	-8.176659	2.92E-16
17436	OSGIN1	887.950511	1.39490918	0.08665744	16.0968205	2.69E-58
17443	OST4	1857.8819	-0.5455519	0.0726769	-7.5065379	6.07E-14
17447	OSTM1	2932.50425	0.37714571	0.06897618	5.46776762	4.56E-08
17462	OTUB2	2100.80205	1.49400171	0.06922275	21.5825241	2.62E-103
17465	OTUD4	2785.31512	0.28558744	0.08260917	3.45709109	0.00054604
17466	OTUD5	2226.26684	-0.3867032	0.06694084	-5.7767899	7.61E-09
17468	OTUD6B	1029.8235	0.58178859	0.08549863	6.8046537	1.01E-11
17471	OTUD7B	1782.5402	0.46299261	0.06819505	6.7892409	1.13E-11
17486	OXA1L	3300.46668	-0.1390134	0.06287833	-2.2108315	0.02704751
17487	OXCT1	464.998516	0.38827004	0.11344544	3.42252665	0.00062042
17496	OXSR1	6014.57484	0.65646652	0.05149319	12.7486097	3.17E-37
17498	OXTR	167.02283	-0.4437387	0.16856315	-2.6324776	0.00847646
17499	P2RX1	4.82622304	-2.1447534	1.02386147	-2.0947692	0.03619152
17510	P2RY11	102.941333	0.74761879	0.2273933	3.28777841	0.00100981
17514	P2RY2	682.813871	-0.3738758	0.09053326	-4.1297065	3.63E-05
17516	P2RY6	71.2971608	-1.1689881	0.26199908	-4.4618021	8.13E-06
17519	P3H2	3097.49125	-0.9641288	0.05987276	-16.102963	2.43E-58
17522	P3H4	386.780073	-0.9957851	0.12460967	-7.9912347	1.34E-15
17523	P4HA1	3956.2185	-1.0131237	0.05950696	-17.025298	5.33E-65
17528	P4HTM	953.208533	-0.6265601	0.09313672	-6.7273151	1.73E-11
17529	PA2G4	5311.72427	0.43239075	0.06064985	7.12929621	1.01E-12
17532	PABPC1	46116.4453	0.24912443	0.04969054	5.01351788	5.34E-07
17533	PABPC1L	1263.8564	0.70039305	0.07097802	9.86774498	5.74E-23
17539	PABPC4	8835.06501	0.61895867	0.04911372	12.6025608	2.04E-36
17540	PABPC4L	57.2354372	-0.5644151	0.26573553	-2.123973	0.03367241
17548	PACRGL	616.059383	0.3347077	0.08968541	3.73201954	0.00018995
17552	PACSIN2	4504.87777	0.28098727	0.05204352	5.39908233	6.70E-08
17553	PACSIN3	1284.61219	-0.4167955	0.10430133	-3.9960709	6.44E-05
17554	PADI1	185.001608	-0.4450713	0.15586976	-2.8554051	0.0042982

17563	PAFAH1B3	2739.39995	-0.3574659	0.11602441	-3.0809541	0.00206338
17564	PAFAH2	279.625267	0.77871595	0.13051855	5.96632378	2.43E-09
17571	PAGE5	57.7105184	-1.0531558	0.26170545	-4.0242029	5.72E-05
17574	PAICS	11817.6724	0.26348608	0.0613115	4.29749862	1.73E-05
17576	PAIP2	4054.49717	0.32950559	0.05749288	5.73124193	9.97E-09
17577	PAIP2B	59.8757568	0.96166857	0.26128037	3.68060018	0.00023269
17578	PAK1	1735.31542	-0.1705456	0.06636957	-2.569635	0.01018057
17579	PAK1IP1	2400.58913	1.10236881	0.06338647	17.391233	9.61E-68
17580	PAK2	8043.65128	0.30862934	0.07248763	4.25768253	2.07E-05
17582	PAK4	2705.81459	-0.556537	0.06886518	-8.0815445	6.40E-16
17588	PALM	254.35404	-0.7585153	0.14296255	-5.3056921	1.12E-07
17592	PALMD	2.96327236	-4.992786	1.58722951	-3.145598	0.00165748
17593	PAM	7714.53217	-0.7452595	0.04911946	-15.172388	5.39E-52
17600	PANK1	376.781902	-0.9919978	0.11755825	-8.4383513	3.22E-17
17602	PANK3	1694.46339	0.48295418	0.09968202	4.84494767	1.27E-06
17605	PANX1	2059.61437	0.4188007	0.0783109	5.34792357	8.90E-08
17608	PAOX	485.745024	-0.7658163	0.11469841	-6.6767819	2.44E-11
17613	PAPLN	126.346498	-0.4321248	0.18001284	-2.4005221	0.0163717
17620	PAPSS1	1987.48101	-0.7202834	0.062377	-11.54726	7.62E-31
17621	PAPSS2	3589.4055	-0.9133194	0.07304845	-12.502926	7.20E-36
17622	PAQR3	1770.77698	0.43593068	0.09136355	4.77138494	1.83E-06
17623	PAQR4	1066.20143	-0.3839201	0.08061041	-4.7626617	1.91E-06
17627	PAQR8	927.619238	-0.5423171	0.07920142	-6.8473154	7.52E-12
17633	PARD6A	215.428026	-0.7213578	0.16265226	-4.4349693	9.21E-06
17637	PARG	1125.09191	-0.450237	0.08783738	-5.1258018	2.96E-07
17644	PARP1	6606.37695	-0.2694494	0.05651312	-4.7679094	1.86E-06
17645	PARP10	1722.18512	-1.3125849	0.09509257	-13.803233	2.44E-43
17647	PARP12	3467.54989	0.54495953	0.05581484	9.76370338	1.61E-22
17651	PARP2	1565.54262	-0.44798	0.06662257	-6.7241478	1.77E-11
17652	PARP3	1060.27609	-0.1599669	0.07994229	-2.0010295	0.04538921
17654	PARP6	2255.22922	0.44051406	0.06726351	6.54907914	5.79E-11
17656	PARP9	541.171196	-0.5388847	0.10008643	-5.3841935	7.28E-08
17657	PARPBP	1260.28896	-0.3487708	0.07387219	-4.7212731	2.34E-06
17658	PARS2	312.220121	0.60256733	0.11805299	5.10421066	3.32E-07
17661	PARVA	5153.16898	-0.2381629	0.06060588	-3.9296995	8.51E-05
17674	PAWR	2591.63466	0.32828836	0.08506522	3.85925494	0.00011373
17682	PAX8	1715.15117	0.81155699	0.06940582	11.6929239	1.39E-31
17683	PAX8-AS1	6936.18965	0.92146477	0.06227846	14.795883	1.56E-49
17685	PAXBP1	1390.9185	0.14595867	0.07042486	2.07254454	0.03821469
17687	PAXIP1	1711.05913	-0.2497304	0.07445923	-3.3539215	0.00079675
17689	PAXIP1-AS2	50.0314015	-0.8831182	0.28532626	-3.0951172	0.00196735
17690	PBDC1	2589.61739	0.34201604	0.09859522	3.46889053	0.00052261
17691	PBK	3018.52178	-0.4314348	0.05957658	-7.2416849	4.43E-13

17695	PBX1	599.333085	-1.7820243	0.11304292	-15.76414	5.49E-56
17697	PBX3	752.191726	-0.3323335	0.10443612	-3.1821703	0.00146176
17699	PBXIP1	1613.14561	-1.0015663	0.0896548	-11.171362	5.63E-29
17716	PCBP2	9042.22367	-0.346987	0.09614912	-3.6088416	0.00030757
17720	PCCA	150.792005	-0.7729687	0.16262684	-4.7530204	2.00E-06
17730	PCDH18	11.3598739	-2.7473919	0.69449561	-3.9559529	7.62E-05
17755	PCDHB10	80.3306388	-1.8504965	0.2387531	-7.7506701	9.14E-15
17756	PCDHB11	71.0892444	-1.7959625	0.25184556	-7.1312059	9.95E-13
17757	PCDHB12	40.3942488	-1.8692185	0.34453123	-5.4253966	5.78E-08
17758	PCDHB13	99.0283824	-1.2084247	0.22350711	-5.4066502	6.42E-08
17759	PCDHB14	231.042145	-1.1012067	0.14789422	-7.4459074	9.63E-14
17761	PCDHB16	25.8937417	-1.1368546	0.40736241	-2.7907696	0.00525829
17772	PCDHB9	53.8101626	-1.5963791	0.32083547	-4.975694	6.50E-07
17774	PCDHGA10	12.1606579	-2.1920493	0.74243813	-2.952501	0.00315211
17779	PCDHGA4	52.9798756	-1.3469973	0.29273907	-4.6013582	4.20E-06
17782	PCDHGA7	16.2761182	-1.1271952	0.52729782	-2.1376823	0.03254253
17785	PCDHGB1	160.169221	-1.1389529	0.19998022	-5.6953275	1.23E-08
17786	PCDHGB2	249.867649	-1.1815273	0.18181541	-6.498499	8.11E-11
17793	PCDHGC3	66.3153632	-1.3800815	0.25403051	-5.4327393	5.55E-08
17795	PCDHGC5	13.3332275	-1.2839697	0.56874463	-2.2575505	0.0239737
17796	PCED1A	2043.14519	-0.1678655	0.06566186	-2.5565147	0.01057266
17799	PCF11	1320.24388	0.35263428	0.09184099	3.83961747	0.00012323
17804	PCGF5	4391.19314	-0.2012611	0.06750679	-2.9813466	0.00286984
17807	PCIF1	2403.97621	-0.4885251	0.06211878	-7.8643715	3.71E-15
17809	PCK2	1734.44877	1.37528204	0.08308463	16.5527859	1.53E-61
17812	PCMT1	4732.27647	0.24547691	0.05735947	4.27962262	1.87E-05
17813	PCMTD1	665.604169	-0.6099567	0.10298652	-5.9226852	3.17E-09
17814	PCMTD2	2166.06142	-0.1979806	0.06441004	-3.073754	0.00211384
17815	PCNA	7558.97718	-1.0012609	0.06766997	-14.796237	1.55E-49
17821	PCNXL2	569.048243	1.09693662	0.10321635	10.6275476	2.22E-26
17823	PCNXL4	7296.82768	0.15822959	0.05974897	2.6482396	0.00809122
17824	PCOLCE	64.7513887	-0.7620529	0.26618642	-2.8628543	0.00419844
17833	PCSK4	175.697341	-0.5987981	0.15927312	-3.7595678	0.00017021
17836	PCSK7	1145.98652	0.1793466	0.07727155	2.32099121	0.02028732
17837	PCSK9	81.6396684	0.6449389	0.2414634	2.67095919	0.00756348
17839	PCYOX1	3268.59256	-1.0635627	0.07873947	-13.507364	1.41E-41
17840	PCYOX1L	194.368253	-0.5170033	0.15507549	-3.3338814	0.00085643
17841	PCYT1A	939.865216	0.27164167	0.09938105	2.73333461	0.00626966
17844	PCYT2	2237.31357	-0.471394	0.06611553	-7.1298529	1.00E-12
17849	PDCD11	2521.09522	0.62588473	0.06199533	10.095676	5.77E-24
17850	PDCD1LG2	195.368466	0.90827419	0.16512908	5.50038898	3.79E-08
17851	PDCD2	3832.83825	0.705655	0.06259865	11.2726868	1.79E-29
17852	PDCD2L	522.842428	0.59249429	0.12648312	4.68437436	2.81E-06

17853	PDCD4	2464.65885	-0.2011505	0.06279077	-3.2035048	0.00135766
17862	PDCL3	630.252737	-0.7559246	0.10869698	-6.9544208	3.54E-12
17863	PDCL3P4	135.742358	2.19340052	0.20041833	10.9441114	7.09E-28
17866	PDE11A	114.359714	-0.4761197	0.19330954	-2.462991	0.01377834
17868	PDE1A	12.1581629	-3.8557752	0.86514106	-4.4568168	8.32E-06
17874	PDE4A	1878.23207	-0.4299592	0.06871635	-6.2570142	3.92E-10
17875	PDE4B	80.1978779	-1.3461254	0.23608671	-5.701826	1.19E-08
17881	PDE6B	5.72728928	2.57845071	1.06918026	2.41161459	0.01588206
17887	PDE7B	857.601712	-1.6325634	0.08530343	-19.13831	1.21E-81
17888	PDE8A	2667.20775	-0.5638564	0.07522605	-7.4954937	6.60E-14
17889	PDE8B	58.3886895	-1.4576696	0.29456056	-4.9486246	7.47E-07
17892	PDGFA	379.121757	-0.2897019	0.13386864	-2.1640759	0.03045852
17893	PDGFB	127.279325	-2.9865699	0.2257667	-13.228567	6.00E-40
17897	PDGFRB	661.752123	-1.0384252	0.10937592	-9.4940931	2.22E-21
17898	PDGFRL	138.645654	-1.2446499	0.18387515	-6.7689949	1.30E-11
17901	PDHB	5701.77401	-0.5141618	0.06297835	-8.1641034	3.24E-16
17902	PDHX	2205.61018	0.25417209	0.06196003	4.10219426	4.09E-05
17904	PDIA3	9916.00197	-0.409992	0.05233172	-7.8344833	4.71E-15
17907	PDIA5	1182.86566	-0.4230838	0.08066087	-5.2452179	1.56E-07
17909	PDIK1L	611.014348	0.81022842	0.09470712	8.55509488	1.18E-17
17912	PDK2	1200.77506	-0.3984023	0.07768493	-5.1284368	2.92E-07
17913	PDK3	656.109675	-1.3284475	0.09713224	-13.67669	1.40E-42
17915	PDLIM1	5975.42068	-0.2677373	0.08974139	-2.9834317	0.00285036
17917	PDLIM3	143.804839	-0.5638966	0.16837136	-3.3491245	0.00081067
17921	PDP1	7982.04333	0.14929801	0.05893373	2.53332036	0.01129876
17922	PDP2	1746.88771	0.36240479	0.08076455	4.48717639	7.22E-06
17926	PDRG1	1786.42539	0.8201529	0.07983435	10.2731838	9.31E-25
17930	PDSS2	501.439159	-0.7346308	0.11829138	-6.2103497	5.29E-10
17938	PDZD11	1589.0526	-0.4665612	0.06867712	-6.7935471	1.09E-11
17943	PDZD8	1772.67497	0.17733682	0.08015054	2.21254678	0.02692891
17951	PEA15	18182.0403	0.82526008	0.05162385	15.9860246	1.60E-57
17953	PEAR1	288.960669	-0.3324921	0.15417758	-2.1565526	0.03104054
17954	PEBP1	6176.77062	-0.3939534	0.06068116	-6.4921858	8.46E-11
17956	PECAM1	80.4696186	-0.574799	0.24027171	-2.3922874	0.01674373
17957	PECR	329.637263	-1.3626699	0.13247123	-10.286535	8.10E-25
17958	PEF1	2259.09811	0.41921223	0.08887765	4.71673412	2.40E-06
17959	PEG10	2293.33737	-0.9100771	0.07970409	-11.418198	3.39E-30
17964	PELI3	828.148712	-0.9699478	0.0885811	-10.949828	6.66E-28
17965	PELO	2690.18487	0.13575328	0.06821094	1.99019804	0.04656912
17966	PELP1	2517.55128	0.29723497	0.07582542	3.9199911	8.86E-05
17969	PEPD	3922.98757	-0.4733023	0.08750443	-5.4088956	6.34E-08
17970	PER1	460.758023	1.21878333	0.1164797	10.4634832	1.27E-25
17971	PER2	305.833799	2.14903668	0.14244305	15.0869883	1.97E-51

17972	PER3	962.500916	0.99756253	0.09504118	10.4961081	9.00E-26
17974	PERM1	9.37093306	1.36767307	0.67185982	2.03565243	0.04178527
17975	PERP	5104.29884	-0.1196733	0.05100779	-2.3461775	0.01896707
17976	PES1	4205.59572	0.45605342	0.07993455	5.70533528	1.16E-08
17977	PET100	701.099999	-0.4940416	0.16201044	-3.0494429	0.00229266
17980	PEX10	1005.45669	0.37612117	0.07545534	4.98468607	6.21E-07
17981	PEX11A	159.553807	-0.7626182	0.16209564	-4.7047425	2.54E-06
17998	PFAS	2176.03753	0.37203223	0.06435573	5.78087231	7.43E-09
18000	PFDN2	4719.60465	1.37909532	0.09398114	14.6741703	9.44E-49
18001	PFDN4	2158.1121	0.25482693	0.0716671	3.55570298	0.00037697
18002	PFDN5	2433.46524	-0.4263128	0.08969181	-4.7530847	2.00E-06
18005	PFKFB2	1407.21837	-0.4536347	0.07989659	-5.6777724	1.36E-08
18007	PFKFB4	1222.77008	-1.7583402	0.07651573	-22.980115	7.37E-117
18008	PFKL	5865.09251	-0.7845285	0.06387813	-12.281645	1.14E-34
18010	PFKP	13706.3435	-0.5256931	0.05820709	-9.0314283	1.69E-19
18011	PFN1	28946.7967	-0.3796577	0.07979407	-4.7579691	1.96E-06
18019	PGAM1	2335.17446	-1.053908	0.1067582	-9.8719162	5.51E-23
18023	PGAM5	1555.30768	0.24520916	0.06716849	3.65065754	0.00026157
18025	PGAP2	415.088494	-0.5154544	0.11131378	-4.630643	3.65E-06
18026	PGAP3	275.095515	-0.7489757	0.1368273	-5.4738757	4.40E-08
18034	PGD	4421.35653	0.31270239	0.05951676	5.25402232	1.49E-07
18035	PGF	145.868532	-2.2994646	0.1976017	-11.636867	2.68E-31
18036	PGGT1B	634.502786	0.70943318	0.10184292	6.96595463	3.26E-12
18037	PGK1	30907.7439	-0.9206885	0.0436766	-21.079674	1.22E-98
18045	PGM2	3325.05423	-0.1970016	0.05849431	-3.3678766	0.00075749
18046	PGM2L1	756.215254	0.39222022	0.09237631	4.24589615	2.18E-05
18047	PGM3	3938.76466	0.51799998	0.06365049	8.13819346	4.01E-16
18054	PGPEP1	1469.10495	-0.341003	0.07118688	-4.7902501	1.67E-06
18057	PGRMC1	3146.16178	-0.2798263	0.0579245	-4.83088	1.36E-06
18063	PHACTR4	1678.8846	0.61046091	0.07012262	8.70561981	3.16E-18
18068	PHC2	6609.14775	0.68358732	0.05078733	13.4598001	2.70E-41
18077	PHF14	1246.01647	-0.2091038	0.07494561	-2.7900736	0.00526961
18078	PHF19	5190.14528	-0.2865129	0.05183552	-5.5273471	3.25E-08
18084	PHF23	2489.93285	0.62580967	0.06423657	9.74226455	1.99E-22
18085	PHF24	15.2597095	7.39063148	1.26767846	5.83005212	5.54E-09
18086	PHF3	6451.63873	0.32107836	0.07458462	4.30488731	1.67E-05
18087	PHF5A	1241.17626	0.74226872	0.07634103	9.7230645	2.40E-22
18097	PHKB	1481.16324	-0.5326799	0.06845886	-7.7810221	7.19E-15
18100	PHLDA1	37720.0701	1.26021326	0.05800176	21.7271552	1.14E-104
18101	PHLDA2	2971.71808	0.14420749	0.06391277	2.25631742	0.02405076
18102	PHLDA3	1365.94992	-0.4081914	0.09774566	-4.1760566	2.97E-05
18103	PHLDB1	2542.88695	-0.6217504	0.06193963	-10.038007	1.04E-23
18106	PHLPP1	704.294635	-0.9262457	0.11611262	-7.9771322	1.50E-15

18107	PHLPP2	721.67748	0.24567832	0.1038735	2.36516845	0.01802187
18115	PHTF1	1447.20692	-0.8053432	0.07258396	-11.095334	1.32E-28
18117	PHYH	1176.76597	-0.2202897	0.07808131	-2.8212854	0.00478316
18118	PHYHD1	1138.86981	-0.2813005	0.07229304	-3.8911143	9.98E-05
18123	PI16	147.033526	-0.3389957	0.17173343	-1.9739647	0.04838576
18126	PI4K2B	2315.65429	-0.3254629	0.06785013	-4.7967916	1.61E-06
18134	PIAS3	1529.62603	-0.5825285	0.07548343	-7.7173035	1.19E-14
18137	PICALM	12292.6363	0.69127076	0.08914777	7.75421299	8.89E-15
18139	PID1	331.115201	1.76950666	0.13163445	13.4425805	3.40E-41
18140	PIDD1	615.821825	-0.4741074	0.10682466	-4.4381829	9.07E-06
18143	PIF1	375.484525	-0.310022	0.12629179	-2.4548075	0.014096
18145	PIGA	749.876829	0.26252309	0.09002626	2.91607253	0.00354468
18146	PIGB	386.481021	-0.6801719	0.11667784	-5.8294866	5.56E-09
18150	PIGG	1979.49807	-0.4423694	0.0715378	-6.183716	6.26E-10
18153	PIGL	170.715923	0.37908828	0.16205179	2.33930329	0.01931974
18154	PIGM	348.466827	0.86226619	0.12856173	6.70702071	1.99E-11
18158	PIGQ	1301.76985	-0.2077066	0.07346512	-2.8272821	0.0046945
18160	PIGS	2393.28926	-0.4972545	0.06096356	-8.1565858	3.45E-16
18162	PIGU	1113.05995	-0.2993653	0.09458548	-3.165024	0.0015507
18164	PIGW	1000.85476	0.77534038	0.07862319	9.86147123	6.11E-23
18165	PIGX	1347.30074	-0.6084709	0.07526407	-8.0844805	6.24E-16
18167	PIGZ	117.010936	-1.2059278	0.19352336	-6.231433	4.62E-10
18168	PIH1D1	3735.29491	-0.6567622	0.08760278	-7.4970475	6.53E-14
18171	PIK3AP1	144.910307	-0.6151774	0.17513	-3.51269	0.00044359
18172	PIK3C2A	2619.93805	0.28035397	0.09825724	2.85326526	0.00432725
18173	PIK3C2B	196.552514	-1.5966441	0.15429755	-10.347826	4.28E-25
18175	PIK3C3	2015.39393	-0.4977645	0.06806844	-7.3127065	2.62E-13
18178	PIK3CD	1094.76962	0.65097822	0.07351975	8.85446689	8.41E-19
18180	PIK3CD-AS2	16.5802476	-2.1253935	0.53458108	-3.9758114	7.01E-05
18184	PIK3R1	578.862688	0.75540584	0.11185107	6.75367535	1.44E-11
18185	PIK3R2	3457.91363	-0.3538492	0.06132062	-5.7704759	7.90E-09
18194	PIM2	902.40506	-0.8843708	0.08821959	-10.024654	1.19E-23
18195	PIM3	4428.28523	0.28133879	0.09544929	2.94752115	0.00320333
18196	PIN1	2206.86858	-0.2315368	0.07153087	-3.2368789	0.00120845
18198	PIN4	925.162786	-0.3055946	0.08466763	-3.6093439	0.00030697
18202	PINLYP	42.2224945	1.7493521	0.33146607	5.27762045	1.31E-07
18203	PINX1	501.810011	0.53071657	0.0977689	5.42827589	5.69E-08
18207	PIP4K2C	1191.06839	0.6130311	0.09130843	6.71385016	1.90E-11
18208	PIP5K1A	4530.89953	0.76560692	0.07311456	10.4713329	1.17E-25
18215	PIR	108.950725	-0.9318771	0.1963862	-4.7451251	2.08E-06
18218	PISD	1323.76102	0.76728168	0.07343768	10.448066	1.50E-25
18220	PITHD1	2028.59277	0.9905445	0.09027563	10.9724461	5.19E-28
18221	PITPNA	2978.6583	0.15907649	0.05828955	2.72907382	0.00635125

18223	PITPNB	4150.25654	0.11016039	0.05390217	2.04370971	0.04098223
18226	PITPNM2	935.687397	-0.7856307	0.08585314	-9.1508679	5.65E-20
18227	PITPNM3	2485.29985	-0.759017	0.06989004	-10.860159	1.78E-27
18228	PITRM1	5237.91432	0.13650555	0.05646549	2.41750382	0.01562737
18244	PKD1P6	452.983812	0.36720205	0.10365995	3.54237152	0.00039655
18259	PKMYT1	1880.32363	0.34268566	0.08939883	3.83322309	0.00012648
18263	PKN3	1415.11654	-0.6559114	0.0700954	-9.3574092	8.17E-21
18269	PKP3	1311.03217	-0.535101	0.08457701	-6.3267908	2.50E-10
18275	PLA2G15	1348.58391	-0.3153764	0.0786323	-4.0107738	6.05E-05
18294	PLA2R1	454.563534	-1.2909533	0.10917175	-11.824976	2.90E-32
18295	PLAA	2607.02488	0.36579348	0.06037177	6.0590152	1.37E-09
18298	PLAC8	2242.67548	-1.3036896	0.07488093	-17.410167	6.91E-68
18303	PLAGL2	1683.74444	0.3845259	0.09047358	4.25014566	2.14E-05
18308	PLBD1	292.218644	-0.4335173	0.12476292	-3.4747288	0.00051137
18313	PLCB3	3227.0374	-0.5511138	0.06554138	-8.4086397	4.15E-17
18314	PLCB4	47.9084163	9.04439634	1.21021596	7.47337386	7.82E-14
18315	PLCD1	367.690988	-0.692758	0.11261946	-6.1513173	7.68E-10
18317	PLCD4	23.6793256	-1.3755758	0.45129757	-3.0480462	0.00230335
18328	PLCXD1	488.069378	0.47672924	0.09780331	4.87436718	1.09E-06
18329	PLCXD2	23.4298503	1.25185712	0.46479654	2.69334433	0.00707392
18331	PLCXD3	8.39945639	3.98150743	1.02723032	3.8759637	0.0001062
18334	PLD2	470.417121	-0.577579	0.10763482	-5.366098	8.05E-08
18335	PLD3	6126.29487	-0.526716	0.06649505	-7.9211305	2.35E-15
18338	PLD6	1446.1944	1.98845151	0.07303922	27.2244363	3.34E-163
18342	PLEKHA1	1035.12887	-0.1980891	0.09333507	-2.1223438	0.03380888
18343	PLEKHA2	1531.77644	-1.2880941	0.08581411	-15.010284	6.29E-51
18347	PLEKHA6	875.112252	-0.1730931	0.07892513	-2.1931307	0.02829797
18348	PLEKHA7	728.537521	1.05612197	0.11032489	9.57283542	1.04E-21
18351	PLEKHB1	81.6122367	-1.397125	0.23626623	-5.9133506	3.35E-09
18354	PLEKHF1	585.536871	0.39429301	0.09923418	3.97335886	7.09E-05
18355	PLEKHF2	1115.9777	0.37458988	0.07882539	4.75214744	2.01E-06
18359	PLEKHG4	1184.4602	-0.5459946	0.07185962	-7.5980717	3.01E-14
18364	PLEKHH1	354.932688	-0.9184623	0.11421095	-8.0418055	8.85E-16
18365	PLEKHH2	164.191056	-0.5849622	0.20859995	-2.8042297	0.0050437
18366	PLEKHH3	712.526132	-1.0971291	0.09797762	-11.197752	4.18E-29
18370	PLEKHM2	4182.62557	0.52075184	0.06650282	7.83052218	4.86E-15
18371	PLEKHM3	233.892484	-0.6033803	0.1592801	-3.7881714	0.00015176
18382	PLIN1	15.2978394	-1.5530604	0.51918355	-2.9913513	0.00277746
18383	PLIN2	6468.0907	1.89351488	0.07135324	26.5371963	3.61E-155
18384	PLIN3	5494.31104	-0.659553	0.06167701	-10.693662	1.09E-26
18387	PLK1	3478.9096	-0.4752495	0.05612872	-8.4671349	2.52E-17
18392	PLLP	387.823978	-1.2182754	0.11291219	-10.789583	3.86E-27
18398	PLP2	16200.5675	0.21592036	0.05604321	3.85274769	0.0001168

18399	PLRG1	3625.44335	0.63971778	0.05371482	11.9095202	1.06E-32
18400	PLS1	1292.82954	0.2303113	0.08885451	2.5920046	0.00954185
18403	PLSCR1	1586.41097	0.14306012	0.07037105	2.03294011	0.04205857
18410	PLXDC1	17.2842448	-1.4885503	0.49540533	-3.0047119	0.00265833
18411	PLXDC2	5.18969002	3.86143602	1.29258465	2.98737574	0.00281384
18412	PLXNA1	7009.19718	-0.4680845	0.06515467	-7.1842055	6.76E-13
18416	PLXNB1	388.010352	-0.6646267	0.1202774	-5.5257822	3.28E-08
18417	PLXNB2	9809.71682	-0.5789589	0.05718938	-10.123539	4.34E-24
18418	PLXNB3	1124.3792	-1.0465063	0.07424996	-14.094369	4.11E-45
18420	PLXND1	1892.45659	-0.9336619	0.07217333	-12.936384	2.81E-38
18423	PMAIP1	9731.77688	1.04908732	0.05269945	19.9069898	3.54E-88
18428	PMEPA1	2496.7868	-0.1406229	0.06289966	-2.2356711	0.02537333
18429	PMF1	569.178433	-0.5289859	0.13140136	-4.0257262	5.68E-05
18432	PML	2783.5443	-0.3411774	0.05979227	-5.7060449	1.16E-08
18434	PMM2	1618.94187	0.21943807	0.06986293	3.14098017	0.00168383
18438	PMPCB	3673.44001	-0.1298881	0.06447523	-2.0145431	0.04395255
18441	PMS2CL	195.14227	0.45974738	0.16277049	2.82451304	0.00473525
18452	PNKD	1685.45644	-0.2962619	0.08508285	-3.4820396	0.00049761
18453	PNKP	1282.86937	-0.4325133	0.08687422	-4.9786158	6.40E-07
18464	PNMAL1	1326.84312	-0.5506929	0.07863668	-7.003003	2.51E-12
18468	PNO1	1674.93297	1.45863373	0.0901393	16.1819956	6.76E-59
18470	PNP	2347.7515	0.49828056	0.0672311	7.4114593	1.25E-13
18476	PNPLA6	5759.13271	-0.7829108	0.06381669	-12.26812	1.34E-34
18480	PNPT1	1819.42443	0.70257202	0.06961251	10.0926122	5.96E-24
18482	PNRC2	1884.9229	0.9353084	0.06796462	13.761696	4.33E-43
18483	POC1A	1682.17175	-0.1903494	0.08480887	-2.244452	0.02480333
18484	POC1B	1342.20094	0.30951155	0.09794927	3.15991684	0.00157814
18486	POC5	573.915924	1.00760107	0.10665516	9.44727898	3.48E-21
18488	PODNL1	55.5897767	-2.203389	0.29788739	-7.3967179	1.40E-13
18489	PODXL	17257.6759	-1.0415709	0.05733733	-18.165668	9.65E-74
18490	PODXL2	415.503376	-0.3881651	0.1053934	-3.683012	0.00023049
18494	POGK	2614.54506	0.42957398	0.07747427	5.54473089	2.94E-08
18498	POLA2	1467.73682	-0.2074254	0.07041709	-2.9456681	0.00322258
18500	POLD1	2829.34814	-0.7028598	0.06174203	-11.383814	5.03E-30
18502	POLD3	1300.35463	-0.3761275	0.08881413	-4.2349964	2.29E-05
18505	POLDIP3	3348.13802	0.51314149	0.05871946	8.73886564	2.35E-18
18506	POLE	2883.43699	-0.5880812	0.07556295	-7.782666	7.10E-15
18507	POLE2	908.821965	-0.4576987	0.07871449	-5.8146696	6.08E-09
18508	POLE3	4541.00462	0.34748399	0.05738226	6.05559995	1.40E-09
18510	POLG	2890.89335	-0.8474214	0.05766002	-14.696862	6.75E-49
18511	POLG2	599.800383	0.36623793	0.11427615	3.2048502	0.00135133
18514	POLK	1360.507	0.58756645	0.10425242	5.63599831	1.74E-08
18519	POLR1A	3089.30586	0.49181955	0.0901765	5.45396578	4.93E-08

18520	POLR1B	2135.17761	0.69413684	0.07544446	9.20063315	3.56E-20
18521	POLR1C	1621.85156	0.35412386	0.09526486	3.71725595	0.0002014
18523	POLR1E	1305.51448	0.35166683	0.07458247	4.71514048	2.42E-06
18527	POLR2D	1364.88945	0.35177857	0.07327044	4.80109821	1.58E-06
18528	POLR2E	6141.60213	-0.6840881	0.05615786	-12.18152	3.90E-34
18529	POLR2F	1445.73336	-0.6742554	0.1232548	-5.4704187	4.49E-08
18530	POLR2G	1871.58379	0.19470535	0.09543926	2.04009696	0.04134067
18532	POLR2I	801.909595	-0.2520266	0.11038221	-2.2832177	0.02241755
18540	POLR3A	1613.89103	0.46897381	0.08189888	5.72625413	1.03E-08
18542	POLR3C	1990.97019	1.22334898	0.0626886	19.5146948	8.24E-85
18543	POLR3D	868.954853	1.39821012	0.08639152	16.1845758	6.48E-59
18544	POLR3E	1379.43786	0.76959717	0.08359567	9.20618467	3.38E-20
18545	POLR3F	796.326302	0.77073973	0.08336683	9.24516011	2.35E-20
18546	POLR3G	491.746277	1.21988004	0.10436474	11.6886228	1.46E-31
18548	POLR3H	2252.22264	0.50041952	0.07391932	6.7698066	1.29E-11
18550	POLRMT	1610.44912	0.26160684	0.07897521	3.31251859	0.0009246
18562	POMGNT2	958.996465	-0.7831215	0.09319146	-8.4033615	4.34E-17
18565	POMT1	1630.59761	-0.8205512	0.068862	-11.915878	9.78E-33
18567	POMZP3	276.757997	-0.4864712	0.12359285	-3.9360787	8.28E-05
18569	PON2	902.655245	-0.5518825	0.08608188	-6.411134	1.44E-10
18573	POP5	347.71037	-0.5685463	0.15057112	-3.7759322	0.00015941
18577	POR	2418.72128	-0.4685839	0.06229498	-7.5220177	5.39E-14
18580	POT1	1522.27115	0.21050776	0.06788824	3.10079845	0.00193
18599	POU2F1	1012.19513	0.43229206	0.0904332	4.78023641	1.75E-06
18621	PP7080	510.035998	-0.2337178	0.10318778	-2.2649758	0.02351416
18623	PPA2	1963.65033	-0.6935936	0.07479706	-9.2730065	1.81E-20
18626	PPAP2A	1149.26606	-0.4752807	0.08472304	-5.6098163	2.03E-08
18629	PPAPDC1A	805.138702	-0.3802444	0.09156248	-4.1528404	3.28E-05
18631	PPAPDC2	128.530286	-0.4012816	0.17690537	-2.2683405	0.02330846
18638	PPAT	1647.27177	0.43463768	0.07069967	6.147662	7.86E-10
18642	PPCS	1183.17785	0.60055149	0.07853993	7.6464484	2.07E-14
18650	PPFIA4	625.345542	-3.0638688	0.11400792	-26.874174	4.40E-159
18653	PPHLN1	1568.64922	-0.1943566	0.07604523	-2.5558027	0.01059432
18663	PPIC	1236.06822	-0.3854956	0.09521279	-4.0487796	5.15E-05
18665	PPIE	1685.41529	0.41065938	0.07809073	5.25874657	1.45E-07
18668	PPIG	2735.26199	-0.2465437	0.08165636	-3.0192834	0.00253373
18670	PPIL1	3449.85046	0.65084765	0.05961032	10.9183718	9.42E-28
18673	PPIL4	1453.1584	0.57364297	0.07882366	7.27754782	3.40E-13
18674	PPIL6	34.4863975	1.85459774	0.42396434	4.37441921	1.22E-05
18683	PPM1G	8032.21607	0.20755234	0.07119277	2.91535679	0.00355282
18688	PPM1M	611.821362	-0.2548642	0.09423233	-2.7046363	0.00683792
18689	PPM1N	45.5222063	-0.599205	0.28568879	-2.0974046	0.03595777
18692	PPP1CA	9733.0851	-0.3112966	0.08602628	-3.6186226	0.00029618

18693	PPP1CB	10776.9394	0.16864094	0.05644063	2.98793513	0.00280869
18694	PPP1CC	9235.32739	-0.5268818	0.0496756	-10.606451	2.78E-26
18695	PPP1R10	4988.90952	1.02800957	0.05824219	17.6506002	1.01E-69
18696	PPP1R11	2749.63765	0.19238871	0.06888928	2.79272351	0.00522663
18701	PPP1R13L	2916.25085	-0.5176268	0.06386001	-8.1056499	5.25E-16
18706	PPP1R15A	21308.518	2.82215953	0.07013969	40.236271	0
18707	PPP1R15B	2785.1416	0.93956641	0.07149729	13.1412878	1.91E-39
18711	PPP1R18	9035.31157	0.61174081	0.04906551	12.4678366	1.12E-35
18715	PPP1R2	1707.8597	0.42132251	0.07811313	5.39374766	6.90E-08
18717	PPP1R26	2309.04221	0.23439585	0.06571314	3.56695542	0.00036115
18729	PPP1R3D	475.960864	-0.4879842	0.1026182	-4.7553377	1.98E-06
18730	PPP1R3E	467.909485	-0.4056668	0.11098131	-3.6552714	0.00025691
18732	PPP1R3G	36.7338761	-1.8546812	0.37860423	-4.8987332	9.65E-07
18734	PPP1R7	1388.84653	0.43866034	0.09262	4.73612961	2.18E-06
18735	PPP1R8	1798.73356	0.32529225	0.07518026	4.32683077	1.51E-05
18737	PPP1R9B	4436.96616	0.26465153	0.07580379	3.49127025	0.00048073
18738	PPP2CA	10548.0316	0.22674371	0.05262939	4.30830956	1.65E-05
18739	PPP2CB	3632.30139	0.20673293	0.06075767	3.40258177	0.00066752
18740	PPP2R1A	11301.1286	-0.2385044	0.06152128	-3.8767792	0.00010585
18742	PPP2R2A	3261.01488	0.6791195	0.06642552	10.223774	1.55E-24
18745	PPP2R2C	101.797856	1.29908687	0.21092428	6.15902012	7.32E-10
18746	PPP2R2D	1608.36721	-0.545674	0.08277979	-6.5918749	4.34E-11
18750	PPP2R4	6565.29665	-0.4290401	0.06121565	-7.0086666	2.41E-12
18751	PPP2R5A	992.032363	-0.4741449	0.08812394	-5.3804326	7.43E-08
18755	PPP2R5E	2032.54119	0.26318058	0.09877881	2.66434243	0.0077139
18757	PPP3CB	3363.78878	-0.1973443	0.05510142	-3.5814732	0.00034166
18759	PPP3CC	598.371725	0.46003284	0.0917288	5.01514091	5.30E-07
18760	PPP3R1	3117.66329	0.38345305	0.0611641	6.2692503	3.63E-10
18762	PPP4C	3694.54547	-0.1734876	0.06913045	-2.509569	0.01208786
18767	PPP4R3A	3270.74141	0.4279863	0.06624154	6.46099594	1.04E-10
18768	PPP4R3B	2651.48656	0.25908396	0.07196245	3.60026605	0.00031789
18775	PPP6R2	1334.3964	-0.3730384	0.07840603	-4.7577773	1.96E-06
18776	PPP6R3	7495.34754	0.1782645	0.06157105	2.89526484	0.00378839
18777	PPRC1	3366.65626	1.09768104	0.06478914	16.9423621	2.19E-64
18781	PPTC7	1683.08426	0.27327729	0.09079318	3.00988769	0.00261344
18787	PQLC2	1107.55775	0.21622255	0.07564386	2.85842814	0.00425746
18789	PQLC3	420.215618	-1.1611933	0.11639596	-9.9762341	1.94E-23
18821	PRC1	7151.61417	-0.2033296	0.05474923	-3.7138345	0.00020414
18823	PRCAT47	59.0066311	-2.974702	0.32442158	-9.1692483	4.76E-20
18826	PRCP	5308.1968	-0.1924853	0.05302581	-3.6300304	0.00028339
18835	PRDM2	694.3809	0.45666171	0.12562529	3.63510966	0.00027786
18836	PRDM4	3191.95674	0.83308587	0.06536723	12.7447016	3.34E-37
18844	PRDX3	12006.0413	-0.2793169	0.05354283	-5.2167003	1.82E-07

18846	PRDX5	7300.49522	-0.4313829	0.10293046	-4.1910126	2.78E-05
18847	PRDX6	9909.24849	0.28682038	0.05911225	4.8521312	1.22E-06
18851	PRELP	9.99525876	-2.1035065	0.70359299	-2.9896638	0.00279285
18852	PREP	1488.30726	-0.6644754	0.07072177	-9.3956275	5.69E-21
18853	PREPL	970.239435	0.42861185	0.09210136	4.65369733	3.26E-06
18870	PRICKLE4	243.957795	-0.675395	0.15261205	-4.4255682	9.62E-06
18871	PRIM1	645.075798	-0.5135133	0.09199996	-5.581669	2.38E-08
18872	PRIM2	1078.70693	0.23515803	0.07671016	3.06553959	0.00217278
18876	PRKAA2	851.738258	0.21986358	0.08620669	2.55042362	0.01075921
18878	PRKAB2	576.754459	0.3992878	0.11007506	3.62741374	0.00028627
18888	PRKAR2A	867.119197	0.47165753	0.1164121	4.05161958	5.09E-05
18890	PRKAR2B	182.642032	-1.3610686	0.17055389	-7.9802848	1.46E-15
18895	PRKCDBP	2821.27811	-1.4145922	0.1187323	-11.914132	9.99E-33
18896	PRKCE	1298.05304	1.69974614	0.0840348	20.2266937	5.70E-91
18897	PRKCG	25.3552685	-0.7618598	0.38835737	-1.9617493	0.04979168
18898	PRKCH	691.897499	1.03482707	0.08787	11.7767957	5.14E-32
18902	PRKCSH	12279.1238	-0.2375116	0.05506603	-4.3132153	1.61E-05
18903	PRKCZ	394.033624	0.24485108	0.11293274	2.16811423	0.03015
18907	PRKDC	10417.0362	0.31001394	0.08245775	3.75967021	0.00017014
18909	PRKG1-AS1	62.3868059	1.19934148	0.264165	4.54012252	5.62E-06
18921	PRLR	179.674417	-0.5741461	0.16953077	-3.3866775	0.00070744
18925	PRMT1	8596.61016	0.68624883	0.05598889	12.2568743	1.54E-34
18926	PRMT2	3220.24723	-0.3278255	0.06919655	-4.7375985	2.16E-06
18927	PRMT3	1700.84108	0.92049682	0.06934296	13.2745527	3.25E-40
18928	PRMT5	5614.2694	0.72754035	0.05315977	13.6859196	1.23E-42
18930	PRMT6	699.526068	-1.1751674	0.0951431	-12.351578	4.78E-35
18931	PRMT7	778.987184	-0.4271465	0.08372812	-5.101589	3.37E-07
18941	PROCA1	21.4423833	-0.8324814	0.42125102	-1.9762123	0.04813074
18955	PROS1	1235.09848	-1.1498995	0.08089679	-14.214402	7.46E-46
18956	PROSC	897.660996	0.81422217	0.08272794	9.8421664	7.41E-23
18957	PROSER1	1408.83113	0.75057323	0.12456314	6.02564482	1.68E-09
18958	PROSER2	2344.33794	0.30834313	0.06837387	4.50966307	6.49E-06
18966	PRPF19	4586.69575	0.48757034	0.06211963	7.84889258	4.20E-15
18968	PRPF31	3772.49572	-0.2748107	0.06396614	-4.2961903	1.74E-05
18983	PRPSAP1	1751.27047	-0.3114387	0.06790027	-4.5867081	4.50E-06
18984	PRPSAP2	1051.72317	0.32114371	0.09105243	3.5270195	0.00042027
18986	PRR12	1880.91333	-0.2202031	0.0705936	-3.1193069	0.00181277
18988	PRR14	1035.22104	-0.1900602	0.07973154	-2.3837518	0.01713715
18989	PRR14L	1032.66033	0.17737213	0.08171514	2.17061528	0.02996027
18994	PRR19	196.079182	-0.3304084	0.14358608	-2.3011174	0.021385
19012	PRR3	877.57883	0.81104247	0.09078048	8.93410655	4.10E-19
19022	PRR5L	665.378642	-0.3383977	0.08682178	-3.8976123	9.71E-05
19028	PRRC2B	9085.49548	0.28876232	0.07004235	4.12268167	3.74E-05

19030	PRRG1	972.945742	0.3046431	0.10226295	2.97901731	0.00289174
19035	PRRT2	18.5173363	-1.4240319	0.52290425	-2.7233129	0.00646308
19047	PRSS22	111.002924	-0.6675607	0.2338111	-2.8551283	0.00430195
19048	PRSS23	13024.8978	-1.2482068	0.04858998	-25.688565	1.57E-145
19071	PRTFDC1	554.452859	-0.5191522	0.10109903	-5.1350855	2.82E-07
19072	PRTG	440.099865	-0.5248185	0.12742824	-4.1185412	3.81E-05
19074	PRUNE	552.079311	0.27292322	0.09854002	2.76966878	0.00561133
19079	PSAP	49271.6502	-0.1757035	0.0582641	-3.0156398	0.00256438
19081	PSAT1	5117.11417	1.68557789	0.05794764	29.0879458	5.10E-186
19082	PSCA	17.2975126	1.56655751	0.51386961	3.04855058	0.00229948
19090	PSG1	490.702552	1.35015821	0.11463964	11.777411	5.10E-32
19091	PSG10P	33.8073141	3.06829925	0.44035818	6.96773537	3.22E-12
19092	PSG11	92.0290778	3.08830193	0.26417418	11.6904005	1.43E-31
19093	PSG2	68.4595458	3.5860702	0.34390906	10.4273793	1.86E-25
19095	PSG4	548.96493	1.49933169	0.09857145	15.2106082	3.01E-52
19096	PSG5	558.656309	0.78287275	0.09812941	7.97796266	1.49E-15
19097	PSG6	61.0384876	2.81421385	0.33693133	8.35248486	6.68E-17
19098	PSG7	214.75668	3.41030625	0.20274541	16.8206333	1.72E-63
19099	PSG8	102.504743	2.2013893	0.23161525	9.5045093	2.01E-21
19100	PSG9	396.394398	1.90129044	0.11941864	15.9212199	4.51E-57
19101	PSIP1	1790.47469	-0.5487757	0.06939387	-7.9081292	2.61E-15
19102	PSKH1	1337.14725	-0.8405064	0.06988937	-12.02624	2.59E-33
19104	PSMA1	5459.31349	-0.3244168	0.06335543	-5.1205832	3.05E-07
19105	PSMA2	5670.57688	-0.1313235	0.06446624	-2.0370892	0.0416411
19108	PSMA4	4743.49979	-0.2669064	0.05894132	-4.5283409	5.94E-06
19111	PSMA7	13413.7721	0.39942351	0.09370687	4.26247837	2.02E-05
19116	PSMB2	5631.30666	0.49708034	0.05861526	8.48039179	2.24E-17
19128	PSMC3IP	1011.62895	0.18118669	0.09103994	1.99018909	0.04657011
19129	PSMC4	4405.9229	0.41895263	0.05511646	7.60122526	2.93E-14
19130	PSMC5	5690.49779	0.45388927	0.06387536	7.10585814	1.20E-12
19132	PSMD1	5838.9067	-0.3059235	0.05383334	-5.6827886	1.33E-08
19134	PSMD11	5967.68842	0.24356493	0.05772879	4.21912437	2.45E-05
19135	PSMD12	4287.08445	0.6395602	0.05915821	10.811014	3.05E-27
19137	PSMD14	7072.49048	0.54336869	0.05702112	9.52925286	1.58E-21
19138	PSMD2	26203.4778	0.5856244	0.04643074	12.6128608	1.79E-36
19139	PSMD3	5320.06206	-0.3160336	0.06458774	-4.8930902	9.93E-07
19145	PSMD7	4411.15584	0.36982183	0.05626401	6.57297279	4.93E-11
19150	PSME3	8599.05291	0.42978548	0.0526465	8.16361005	3.25E-16
19151	PSME4	3654.05115	0.61397174	0.07190768	8.53833335	1.36E-17
19152	PSMF1	3729.03004	-0.185713	0.05405386	-3.4357032	0.00059102
19153	PSMG1	2588.65961	0.81370223	0.06579582	12.3670816	3.94E-35
19157	PSMG4	978.848194	0.41356857	0.12825428	3.22459868	0.00126149
19158	PSORS1C1	206.344358	0.66810153	0.15608843	4.28027584	1.87E-05

19162	PSPH	1436.50021	0.63000058	0.07242456	8.69871507	3.36E-18
19164	PSRC1	1317.63001	-0.6692432	0.07367564	-9.0836431	1.05E-19
19170	PTBP1	13684.6875	0.25078644	0.07279942	3.44489593	0.00057128
19185	PTDSS1	5627.3634	0.12085027	0.05112568	2.36378784	0.01808917
19187	PTEN	3061.26198	-0.1918043	0.06215934	-3.085688	0.00203082
19200	PTGES	2787.76616	-1.3245216	0.06900265	-19.195229	4.06E-82
19201	PTGES2	4098.11902	-0.194687	0.08855609	-2.1984596	0.02791637
19220	PTK2B	1065.15544	-1.1343727	0.08068423	-14.05941	6.74E-45
19224	PTMS	4281.14636	-1.1705768	0.11065443	-10.578671	3.74E-26
19225	PTN	122.046603	-1.0283388	0.19793998	-5.1952054	2.04E-07
19226	PTOV1	3600.67646	-0.7967724	0.08108297	-9.8266297	8.65E-23
19229	PTP4A1	8555.09261	0.24231289	0.06197614	3.90977689	9.24E-05
19230	PTP4A2	10327.6762	0.17875185	0.05883761	3.03805412	0.00238111
19236	PTPN12	9857.22398	0.32446139	0.06125849	5.29659486	1.18E-07
19240	PTPN2	1716.40514	0.71667632	0.06800713	10.5382531	5.76E-26
19245	PTPN3	898.81861	-1.2714413	0.10171805	-12.499663	7.50E-36
19248	PTPN6	158.875956	-1.2173681	0.1873412	-6.4981335	8.13E-11
19250	PTPN9	1625.64748	-0.2970206	0.07568176	-3.9245995	8.69E-05
19251	PTPRA	3438.68114	-0.56965	0.06672256	-8.5375926	1.37E-17
19254	PTPRCAP	75.4750878	-0.7353579	0.26480153	-2.7770154	0.00548606
19258	PTPRE	2432.84513	-0.8898146	0.07377105	-12.061839	1.68E-33
19259	PTPRF	14961.478	0.23515716	0.07218883	3.25752826	0.00112387
19263	PTPRJ	4926.42139	-1.0391509	0.08551187	-12.152124	5.59E-34
19264	PTPRK	5043.6812	0.4159764	0.06352957	6.54776044	5.84E-11
19270	PTPRR	609.314534	2.10795724	0.10169494	20.7282416	1.93E-95
19271	PTPRS	2327.29577	-0.5583229	0.06269959	-8.9047296	5.35E-19
19276	PTRF	21094.7216	-1.3729723	0.04944993	-27.7649	1.15E-169
19278	PTRH2	1607.45389	0.57139884	0.08338439	6.85258764	7.25E-12
19288	PUM1	4045.101	0.35194317	0.06846634	5.14038246	2.74E-07
19289	PUM2	4699.136	0.20603976	0.07032515	2.9298162	0.00339163
19290	PURA	1654.12391	0.40160811	0.06526375	6.1536169	7.57E-10
19293	PUS1	891.427995	0.49050594	0.10696486	4.58567364	4.53E-06
19295	PUS3	448.119246	0.599312	0.1105643	5.42048363	5.94E-08
19296	PUS7	1645.27646	0.55292012	0.06474087	8.54051044	1.34E-17
19297	PUS7L	627.213577	0.84182681	0.10901822	7.72189126	1.15E-14
19298	PUSL1	763.022342	0.21325517	0.10181843	2.09446535	0.03621855
19300	PVR	8027.91462	0.84861453	0.05278808	16.075875	3.77E-58
19303	PVRL1	458.804785	-0.6366009	0.10718432	-5.9393094	2.86E-09
19308	PVT1	885.37224	1.10916638	0.09180683	12.0815239	1.32E-33
19313	PWP1	3514.16572	0.82622317	0.06348541	13.0143781	1.01E-38
19314	PWP2	177.01081	0.73626284	0.17236422	4.27155257	1.94E-05
19321	PXDC1	3021.19342	0.15149166	0.06008833	2.5211495	0.01169721
19326	PXMP4	453.326893	-0.6786396	0.1066946	-6.3605803	2.01E-10

19327	PXN	16697.5571	0.14461481	0.0478685	3.02108521	0.0025187
19330	PXYLP1	102.132236	-1.1214887	0.21984214	-5.1013362	3.37E-07
19331	PYCARD	295.231839	-0.9548923	0.16284548	-5.863794	4.52E-09
19333	PYCR1	4871.78148	0.96400448	0.05567514	17.3148107	3.64E-67
19338	PYGB	12350.6939	0.18498208	0.05121365	3.61196829	0.00030388
19339	PYGL	5738.96512	0.61158447	0.05359861	11.4104547	3.71E-30
19341	PYGO1	943.707374	0.43580959	0.10931009	3.98691083	6.69E-05
19344	PYROXD1	257.305628	0.98719442	0.13827865	7.13916717	9.39E-13
19345	PYROXD2	399.226997	-1.0198721	0.10806566	-9.4375221	3.82E-21
19350	QARS	5285.47401	-0.7913509	0.06197249	-12.76939	2.43E-37
19352	QKI	4590.69935	0.35159335	0.07148995	4.91808081	8.74E-07
19360	QRSL1	579.740121	0.21694584	0.09150034	2.37098397	0.0177408
19361	QSER1	2829.33084	0.44375749	0.10372367	4.2782665	1.88E-05
19362	QSOX1	17319.5465	0.35295395	0.07072444	4.99055106	6.02E-07
19363	QSOX2	5859.93652	0.18590333	0.05535425	3.35842929	0.00078387
19365	QTRTD1	957.339445	0.73935906	0.08391803	8.81049113	1.25E-18
19366	R3HCC1	763.030627	-0.5595816	0.10696654	-5.2313706	1.68E-07
19367	R3HCC1L	994.692188	1.11857244	0.079701	14.0346098	9.57E-45
19372	RAB10	6801.78222	0.30862672	0.05238433	5.89158458	3.83E-09
19373	RAB11A	4406.33556	-0.4680562	0.05778649	-8.099751	5.51E-16
19377	RAB11FIP2	1299.56384	0.29981942	0.07684725	3.90149821	9.56E-05
19378	RAB11FIP3	1054.89061	-0.8298187	0.07671185	-10.817346	2.85E-27
19384	RAB15	479.357647	1.08448485	0.10770082	10.069421	7.54E-24
19389	RAB1B	11115	-0.2332873	0.04772218	-4.8884461	1.02E-06
19390	RAB20	326.029077	-1.2859895	0.12512831	-10.277366	8.91E-25
19391	RAB21	1923.89747	0.48370847	0.06830004	7.0821106	1.42E-12
19392	RAB22A	4148.33027	-0.2887067	0.05281702	-5.4661666	4.60E-08
19396	RAB26	75.7890926	-0.7397576	0.22799228	-3.2446608	0.00117591
19397	RAB27A	1073.73933	-1.1514601	0.09235129	-12.468262	1.11E-35
19398	RAB27B	987.490561	-0.8235441	0.11420135	-7.211334	5.54E-13
19399	RAB28	1395.56458	0.32704804	0.07781324	4.20298708	2.63E-05
19400	RAB29	2919.93147	0.56963489	0.05674078	10.0392497	1.02E-23
19404	RAB30-AS1	464.892832	0.42571471	0.13358487	3.18684829	0.00143832
19405	RAB31	4110.33614	-0.2823693	0.05307047	-5.3206493	1.03E-07
19408	RAB33B	468.626675	0.52756168	0.11705267	4.50704526	6.57E-06
19410	RAB35	3144.5921	0.66174121	0.08887616	7.44565517	9.65E-14
19413	RAB38	245.501607	-1.3706376	0.14249545	-9.6188166	6.66E-22
19416	RAB3A	94.4433112	-1.9406178	0.22346137	-8.6843546	3.81E-18
19419	RAB3D	690.376013	-1.488782	0.10766497	-13.827914	1.73E-43
19427	RAB40C	509.222401	-0.7760467	0.10688574	-7.2605259	3.86E-13
19435	RAB5A	4519.85964	0.39306797	0.06960819	5.64686353	1.63E-08
19436	RAB5B	2778.20128	-1.3870947	0.06594287	-21.034793	3.15E-98
19443	RAB7B	22.0143173	-1.196996	0.46477083	-2.5754541	0.01001085

19445	RAB8B	2224.28518	0.54849143	0.08486213	6.46332383	1.02E-10
19446	RAB9A	2232.84146	0.46444725	0.06695089	6.93713372	4.00E-12
19450	RABEP1	4178.70498	0.38783316	0.06011278	6.45175869	1.11E-10
19451	RABEP2	349.252504	-0.8297951	0.12619128	-6.5756931	4.84E-11
19452	RABEPK	987.845535	1.05958912	0.09076943	11.6734141	1.74E-31
19455	RABGEF1	3567.25549	0.45051438	0.05955637	7.56450399	3.89E-14
19456	RABGGTA	842.261221	-0.4717846	0.08910416	-5.2947534	1.19E-07
19457	RABGGTB	2714.77291	0.76757433	0.06951991	11.0410718	2.42E-28
19458	RABIF	1305.21944	0.51017869	0.07648827	6.67002511	2.56E-11
19464	RAC2	10029.3679	-0.3836298	0.05859475	-6.5471699	5.86E-11
19466	RACGAP1	4206.82992	-0.2845982	0.06013693	-4.7325034	2.22E-06
19468	RAD1	2214.12316	0.35129116	0.06011298	5.84384909	5.10E-09
19470	RAD18	2024.65168	0.39719548	0.07864625	5.05040591	4.41E-07
19474	RAD23A	8285.68667	0.46585348	0.05322061	8.75325265	2.07E-18
19475	RAD23B	12009.5554	0.49998771	0.05963772	8.38374886	5.13E-17
19476	RAD50	4625.25122	0.54064444	0.08427175	6.41548882	1.40E-10
19478	RAD51-AS1	41.8593461	-1.0120686	0.33105913	-3.057063	0.00223517
19488	RAD54L2	382.627635	0.42273294	0.14924519	2.8324728	0.00461895
19490	RAD9B	39.2867322	1.63287793	0.35932424	4.54430214	5.51E-06
19492	RAE1	2739.32811	0.87999764	0.06381582	13.7896479	2.94E-43
19495	RAET1G	157.400357	-1.0009577	0.17126834	-5.8443821	5.08E-09
19503	RAI14	6003.90831	0.3867875	0.06933516	5.57851848	2.43E-08
19505	RALA	4704.03459	0.82620244	0.05663626	14.5878707	3.36E-48
19512	RALGDS	1966.25733	-0.3511139	0.06299302	-5.5738536	2.49E-08
19515	RALY	6056.01991	-0.1902723	0.05331583	-3.5687768	0.00035865
19518	RAMP1	1094.45579	-1.1641754	0.10287474	-11.316436	1.09E-29
19525	RANBP17	200.028334	-0.3749788	0.14965735	-2.5055823	0.01222499
19531	RANGAP1	12065.4513	0.43326714	0.07642365	5.66928061	1.43E-08
19534	RAP1B	1210.75474	0.49203166	0.0757266	6.49747484	8.17E-11
19535	RAP1GAP	124.287752	0.69586389	0.18855482	3.69051238	0.0002238
19538	RAP2A	1607.62692	0.254093	0.07659877	3.31719439	0.00090926
19539	RAP2B	2779.25012	0.51198489	0.06717248	7.62194438	2.50E-14
19540	RAP2C	2350.01531	0.32824967	0.07292637	4.50111078	6.76E-06
19543	RAPGEF2	1789.36218	0.27744996	0.10250401	2.70672301	0.00679509
19545	RAPGEF4	41.4756884	-1.8669788	0.32672127	-5.7142861	1.10E-08
19550	RAPH1	4108.08009	0.19706755	0.08690335	2.26766348	0.02334973
19552	RARA	1418.95094	0.54907348	0.08186251	6.70726396	1.98E-11
19557	RARRES2	3360.42437	-2.0066057	0.11958964	-16.779093	3.47E-63
19558	RARRES3	1372.52604	-1.5924341	0.13230305	-12.036261	2.29E-33
19563	RASA3	2637.00193	0.31778652	0.06384904	4.97715426	6.45E-07
19568	RASAL2	3368.80626	0.74002414	0.08546022	8.65928234	4.75E-18
19572	RASD2	213.888494	-0.8474171	0.14096677	-6.0114674	1.84E-09
19573	RASEF	32.9259722	-1.1732779	0.37032908	-3.1682035	0.00153384

19582	RASGRP3	479.329789	1.29558046	0.1141973	11.3451061	7.84E-30
19587	RASL11A	61.1400251	-0.7583034	0.27246645	-2.7831076	0.00538409
19590	RASSF1	2385.12497	0.90892793	0.08256062	11.0092194	3.45E-28
19593	RASSF2	120.689112	-1.3073279	0.19391573	-6.7417322	1.57E-11
19594	RASSF3	2725.48353	-0.4483296	0.05807501	-7.719837	1.16E-14
19595	RASSF4	688.997562	-1.5452449	0.10347003	-14.934227	1.97E-50
19599	RASSF8	642.105046	0.79173881	0.11209419	7.06315627	1.63E-12
19615	RBBP8	3203.13622	0.21679168	0.06539313	3.31520574	0.00091576
19617	RBBP9	1068.52396	-0.4062218	0.09538064	-4.2589547	2.05E-05
19618	RBCK1	3849.47468	0.66570563	0.05982828	11.1269398	9.28E-29
19626	RBL2	1296.32291	-0.7057082	0.09543333	-7.3947768	1.42E-13
19637	RBM18	1570.33824	0.8679823	0.07471397	11.6174032	3.36E-31
19639	RBM20	33.9356697	1.98305359	0.42073738	4.71328124	2.44E-06
19640	RBM22	2257.97537	0.83177657	0.06545919	12.7067952	5.42E-37
19641	RBM23	2805.65003	-0.4916732	0.05727054	-8.5850978	9.08E-18
19642	RBM24	512.423641	0.97620303	0.10916585	8.94238487	3.81E-19
19645	RBM26-AS1	90.8751828	1.12280072	0.21033028	5.33827426	9.38E-08
19646	RBM27	1709.52338	0.24786187	0.09627375	2.57455299	0.01003697
19647	RBM28	1480.33559	0.64767932	0.06990086	9.2656848	1.94E-20
19650	RBM34	2267.51686	0.39743988	0.06680941	5.94886061	2.70E-09
19651	RBM38	1121.63167	1.04646356	0.07552712	13.8554681	1.18E-43
19652	RBM39	11901.7152	0.12866633	0.04802747	2.67901566	0.00738389
19658	RBM45	402.524805	-0.2385449	0.11938284	-1.9981507	0.04570033
19662	RBM4B	405.918555	-0.4594031	0.11038035	-4.1620007	3.15E-05
19663	RBM5	4030.86494	0.27903016	0.05626851	4.95890435	7.09E-07
19665	RBM6	2991.4681	-0.1634083	0.05900595	-2.7693529	0.00561678
19675	RBMXL1	861.78772	-0.6149897	0.08118283	-7.5753667	3.58E-14
19693	RBP7	14.6878778	-1.0957821	0.52956951	-2.0691941	0.03852787
19696	RBPMS	334.894949	1.84633133	0.13216638	13.9697506	2.38E-44
19697	RBPMS-AS1	12.8196944	1.99586169	0.64221431	3.10778139	0.00188497
19698	RBPMS2	238.584469	-0.4470519	0.13655399	-3.2738104	0.00106108
19700	RBX1	1761.00924	-0.4496522	0.09998004	-4.4974197	6.88E-06
19701	RC3H1	1071.66907	0.24021517	0.09903581	2.42553857	0.01528569
19703	RCAN1	2341.54149	0.6783799	0.06656688	10.190953	2.18E-24
19705	RCAN3	470.125799	-0.4339809	0.10951465	-3.9627654	7.41E-05
19708	RCBTB2	96.8827278	-0.4242895	0.20856422	-2.0343348	0.04191785
19709	RCC1	4038.95292	0.71753296	0.05746578	12.4862659	8.87E-36
19710	RCC2	4490.08947	0.55982645	0.06611643	8.46728206	2.51E-17
19711	RCCD1	1719.781	-0.4347676	0.09540917	-4.5568745	5.19E-06
19714	RCL1	389.358795	1.07023079	0.12280847	8.71463363	2.92E-18
19717	RCN3	341.299917	-1.4687617	0.14986558	-9.8005275	1.12E-22
19721	RCSD1	859.891134	-0.6600675	0.08680445	-7.6040746	2.87E-14
19738	RECK	561.639136	-0.9093779	0.09741129	-9.3354471	1.01E-20

19740	RECQL4	3409.18901	-0.8399832	0.06438699	-13.045852	6.71E-39
19743	REEP2	429.885097	-0.8076479	0.12853784	-6.2833474	3.31E-10
19744	REEP3	4142.18136	-0.397403	0.08037189	-4.9445517	7.63E-07
19745	REEP4	1529.21892	-0.4162325	0.09321646	-4.4652253	8.00E-06
19747	REEP6	357.600058	-0.6718891	0.11429937	-5.8783272	4.14E-09
19754	REL	227.043652	1.11559381	0.20771817	5.37070887	7.84E-08
19757	RELL1	781.511325	-0.4360225	0.10478886	-4.1609622	3.17E-05
19759	RELN	431.992082	3.76708239	0.15454179	24.3758175	3.09E-131
19766	REPIN1	11065.784	-0.5727949	0.06053097	-9.4628413	3.00E-21
19767	REPS1	1648.32501	0.49077426	0.07253146	6.76636421	1.32E-11
19776	REST	1453.86433	0.44057772	0.09115086	4.8335002	1.34E-06
19782	REV3L	1284.94297	0.59895874	0.09756939	6.13879769	8.31E-10
19783	REXO1	2223.06061	0.27489592	0.06391847	4.30072717	1.70E-05
19786	REXO4	1868.56268	0.22837641	0.08025222	2.84573317	0.00443093
19787	RFC1	4245.3644	0.52539029	0.06139939	8.55693086	1.16E-17
19791	RFC5	1451.41924	-0.2326275	0.0690333	-3.3697859	0.00075227
19792	RFESD	282.391279	1.10421305	0.12677221	8.7102139	3.03E-18
19793	RFFL	177.443039	0.66775916	0.15393337	4.33797533	1.44E-05
19794	RFK	2961.11933	0.59511901	0.05883207	10.1155551	4.71E-24
19795	RFNG	1643.87349	0.25980885	0.07092813	3.66298758	0.00024929
19804	RFT1	671.902052	-0.360939	0.0930229	-3.8801094	0.00010441
19805	RFTN1	3217.08118	0.98321919	0.05663627	17.3602407	1.65E-67
19808	RFWD3	2504.63044	0.23810488	0.06604219	3.60534503	0.00031174
19809	RFX1	785.611079	-0.2999612	0.08695493	-3.4496174	0.00056138
19819	RFXAP	212.471598	0.49515904	0.16182028	3.05993192	0.00221387
19823	RGL1	647.144225	0.48480572	0.0987486	4.90949469	9.13E-07
19825	RGL3	166.546967	-0.7243586	0.15480639	-4.6791261	2.88E-06
19828	RGMB	1664.19611	0.26695349	0.07271729	3.67111417	0.0002415
19830	RGN	54.5508115	-2.0601864	0.30979333	-6.6501961	2.93E-11
19844	RGS12	798.805197	-0.9609458	0.08678541	-11.072665	1.70E-28
19846	RGS14	161.40209	-0.7882499	0.18542679	-4.2510035	2.13E-05
19847	RGS16	43.116029	5.00330868	0.59760475	8.37227055	5.65E-17
19850	RGS19	964.823341	-0.2741455	0.09739864	-2.8146743	0.00488267
19856	RGS4	397.519084	-0.5316512	0.10891756	-4.8812259	1.05E-06
19857	RGS5	169.130702	0.6430104	0.16635717	3.86523996	0.00011098
19862	RGS9	122.954548	-0.6609595	0.19821191	-3.3346103	0.00085419
19867	RHBDD2	2376.16643	-0.1451266	0.06559794	-2.2123647	0.02694148
19869	RHBDF1	1504.97242	-0.4932976	0.06690816	-7.3727578	1.67E-13
19872	RHBDL2	374.228843	0.25654184	0.12100219	2.12014217	0.03399406
19885	RHOBTB2	417.757913	-1.0886107	0.11206469	-9.7141269	2.62E-22
19890	RHOG	1803.52093	-0.3281801	0.07318442	-4.4842891	7.32E-06
19892	RHOJ	40.1888327	-3.126036	0.39698495	-7.8744445	3.42E-15
19896	RHOU	596.037889	-0.7731036	0.0985722	-7.8430189	4.40E-15

19897	RHOV	28.6912302	-2.5710053	0.46521713	-5.5264631	3.27E-08
19903	RHPN1	732.229036	-0.5688748	0.1032566	-5.5093307	3.60E-08
19907	RIBC2	48.7681112	-1.0597818	0.31043448	-3.4138662	0.00064048
19910	RIC8A	8737.89132	0.31070525	0.05259433	5.90758029	3.47E-09
19911	RIC8B	640.77834	-0.6313509	0.09961179	-6.3381144	2.33E-10
19912	RICTOR	2696.751	0.28574341	0.08426968	3.39082107	0.00069684
19915	RILP	534.315607	-0.5460809	0.10789893	-5.0610411	4.17E-07
19917	RILPL2	1042.03386	0.21325021	0.09216952	2.31367392	0.02068562
19919	RIMBP3	7.23155163	-2.0872979	0.96789148	-2.1565413	0.03104142
19922	RIMKLA	24.3997275	-1.7500323	0.42105538	-4.1562995	3.23E-05
19925	RIMS2	292.551546	0.5242889	0.12472787	4.20346218	2.63E-05
19928	RIN1	1268.15675	-0.3939239	0.07688723	-5.1233989	3.00E-07
19929	RIN2	2490.67689	-0.5261648	0.06115354	-8.6039954	7.70E-18
19930	RIN3	1918.79492	0.1784201	0.06530556	2.7320813	0.00629356
19931	RING1	2242.06179	0.35910794	0.06810823	5.27260718	1.34E-07
19934	RIOK1	1364.06964	0.85854557	0.07147658	12.0115647	3.09E-33
19935	RIOK2	1151.38953	0.49709114	0.08343752	5.95764518	2.56E-09
19936	RIOK3	4989.44587	0.79386251	0.06460911	12.2871603	1.06E-34
19937	RIPK1	2150.70944	0.27070002	0.06393177	4.23420201	2.29E-05
19944	RIT1	2042.03909	0.97102948	0.06438134	15.0824679	2.11E-51
19946	RITA1	2076.14946	-0.349638	0.07114498	-4.914443	8.90E-07
19948	RLF	1442.87461	0.48846805	0.08389256	5.82254298	5.80E-09
19954	RMDN1	1373.7083	-0.3322149	0.07016283	-4.734913	2.19E-06
19955	RMDN2	126.633782	-0.50922	0.19332275	-2.6340409	0.00843753
19957	RMDN3	1320.45515	-0.2076526	0.09480212	-2.1903798	0.02849671
19958	RMI1	1422.59645	0.17131555	0.0816898	2.09714747	0.03598052
19959	RMI2	708.520541	-0.4508281	0.09431025	-4.7802663	1.75E-06
19989	RNASE1	23.1423895	-3.2668219	0.55932187	-5.8406832	5.20E-09
19996	RNASE4	331.597018	-1.5798437	0.13773277	-11.470354	1.86E-30
20002	RNASEH1-AS	457.763038	1.15881569	0.10734928	10.7948153	3.64E-27
20006	RNASEH2C	3222.24573	-0.5325174	0.09160828	-5.8129832	6.14E-09
20010	RNASET2	1932.31752	-0.9608794	0.09095937	-10.563831	4.38E-26
20013	RND3	6461.72448	1.03832937	0.0578305	17.9547004	4.41E-72
20015	RNF103	1722.28012	-0.3462724	0.06892611	-5.0238196	5.07E-07
20017	RNF11	3760.2614	0.57580494	0.06063127	9.4968315	2.16E-21
20018	RNF111	1257.94552	0.43877294	0.09781801	4.48560462	7.27E-06
20026	RNF123	1673.29877	-0.22587	0.07008919	-3.2226088	0.00127029
20028	RNF126	3103.94341	0.28221501	0.06955804	4.05725951	4.97E-05
20032	RNF130	1678.67507	-0.7112396	0.07326184	-9.7081866	2.78E-22
20034	RNF135	928.248757	-0.8195909	0.09098209	-9.0082667	2.09E-19
20035	RNF138	1862.29078	0.77523579	0.08295503	9.34525357	9.17E-21
20041	RNF144A	120.4706	-1.5012733	0.2373849	-6.3242156	2.55E-10
20043	RNF144B	440.405563	0.45802305	0.10903459	4.20071322	2.66E-05

20044	RNF145	4806.57867	-0.8890829	0.05879519	-15.121695	1.17E-51
20047	RNF149	3600.5544	0.20396666	0.06541424	3.11807725	0.00182035
20053	RNF165	28.9659842	-0.9842655	0.39748453	-2.4762359	0.01327758
20055	RNF167	2357.38118	0.58591239	0.06373285	9.19325533	3.81E-20
20057	RNF169	1345.59722	0.62397789	0.07835014	7.96396689	1.67E-15
20059	RNF170	797.916401	-0.3077199	0.08122471	-3.7885016	0.00015156
20063	RNF182	503.615943	0.62748047	0.10714229	5.85651534	4.73E-09
20067	RNF187	4546.1894	-0.3823181	0.08917423	-4.287316	1.81E-05
20072	RNF207	621.19245	-0.381637	0.09453793	-4.0368671	5.42E-05
20073	RNF208	304.068174	-0.3769208	0.13089148	-2.8796433	0.00398125
20081	RNF216P1	658.282386	0.67430416	0.1025513	6.57528658	4.86E-11
20082	RNF217	821.426305	0.21305152	0.084698	2.51542563	0.01188888
20084	RNF219	720.499767	0.52399326	0.10488436	4.99591419	5.86E-07
20088	RNF223	12.3564533	2.7040251	0.68632443	3.93986427	8.15E-05
20093	RNF26	3630.05979	-0.8042825	0.05693861	-14.125433	2.65E-45
20097	RNF38	1354.76588	0.25830154	0.07975104	3.23884846	0.00120013
20101	RNF41	1278.40603	0.64521054	0.07380597	8.74198302	2.29E-18
20106	RNF6	2623.24926	0.93239719	0.074462	12.5217854	5.67E-36
20108	RNF8	2771.8102	0.15482689	0.06770183	2.28689374	0.02220203
20110	RNFT2	115.412958	-0.9771168	0.23498131	-4.1582747	3.21E-05
20115	RNMTL1	1387.08404	0.35589766	0.09715289	3.66327413	0.00024901
20116	RNPC3	382.457882	-0.6688322	0.11984716	-5.5807101	2.40E-08
20117	RNPEP	3146.3146	-0.57609	0.0563426	-10.224768	1.54E-24
20118	RNPEPL1	1721.56674	-0.6641254	0.07068478	-9.3955936	5.69E-21
20119	RNPS1	2598.11479	-0.567468	0.05945912	-9.5438336	1.38E-21
20129	RNU11	7.24259594	1.95535632	0.78278602	2.49794488	0.01249156
20211	ROCK1	3424.64384	0.38183309	0.08411832	4.5392382	5.65E-06
20213	ROCK2	2079.77035	0.30129423	0.12864962	2.34197526	0.01918199
20214	ROGDI	212.826292	-0.6415285	0.15868128	-4.0428747	5.28E-05
20221	ROR1	1996.17292	0.1722884	0.08481914	2.03124437	0.04223021
20229	RORC	15.7932135	-1.9200867	0.64572656	-2.9735291	0.00294397
20233	RP1L1	41.3945021	-0.7337311	0.30287001	-2.422594	0.01541014
20235	RP9	435.079005	0.86531723	0.11582013	7.47121635	7.95E-14
20238	RPA2	2317.80378	0.40690707	0.068213	5.96524227	2.44E-09
20239	RPA3	1218.66525	-0.4527506	0.10662501	-4.2461951	2.17E-05
20240	RPA4	21.6740441	2.86061289	0.51145607	5.59307646	2.23E-08
20241	RPAIN	1796.7492	0.50017247	0.08580308	5.82930664	5.57E-09
20243	RPAP2	916.287133	0.91071193	0.08815646	10.3306315	5.12E-25
20244	RPAP3	1616.10907	0.33126028	0.07302356	4.53634814	5.72E-06
20250	RPF2	1913.42021	1.00274898	0.06787573	14.7733077	2.18E-49
20251	RPGR	619.041592	0.68205024	0.10513005	6.48768098	8.72E-11
20256	RPIA	1484.1599	0.46250859	0.09934649	4.65550993	3.23E-06
20257	RPL10	6971.5946	0.36239966	0.05505771	6.5821784	4.64E-11

20258	RPL10A	11089.3433	0.58954966	0.08000997	7.36845232	1.73E-13
20260	RPL11	15355.1067	0.46299683	0.06845252	6.76376603	1.34E-11
20261	RPL12	9305.25063	0.36576116	0.06437989	5.68129511	1.34E-08
20271	RPL15	13351.1463	-0.206281	0.05463294	-3.7757627	0.00015952
20282	RPL22L1	2316.64287	1.04159483	0.06427015	16.2065094	4.54E-59
20286	RPL23AP53	223.718141	0.81050157	0.15005226	5.40146194	6.61E-08
20288	RPL23AP7	234.058556	1.17209284	0.14670456	7.98947769	1.36E-15
20292	RPL24	12107.6153	0.2239462	0.08654833	2.58752763	0.00966674
20293	RPL26	11124.6036	0.40450021	0.09819224	4.11947208	3.80E-05
20294	RPL26L1	678.187401	0.49873057	0.09047867	5.51213436	3.55E-08
20295	RPL27	12805.3425	0.41823031	0.05994773	6.97658281	3.02E-12
20298	RPL29	8719.67786	-0.130097	0.06620636	-1.9650221	0.04941168
20301	RPL30	17341.8934	0.23419892	0.05821995	4.02265736	5.75E-05
20314	RPL37	15363.3083	0.20002304	0.09297424	2.1513814	0.03144611
20320	RPL4	12070.6247	-0.3473182	0.06620823	-5.2458472	1.56E-07
20321	RPL41	119.470379	0.51774197	0.18825736	2.75018177	0.00595622
20322	RPL5	21254.2657	0.13373697	0.05137791	2.60300546	0.00924105
20326	RPL7L1	2785.73282	1.02460148	0.07921325	12.934724	2.87E-38
20327	RPL8	41390.8989	0.34000341	0.08796768	3.86509455	0.00011105
20335	RPP14	1307.54576	0.6787753	0.07418951	9.14920885	5.74E-20
20337	RPP25	1522.62217	0.39747903	0.08312207	4.7818714	1.74E-06
20341	RPP40	573.898589	1.37296173	0.10306941	13.3207491	1.75E-40
20343	RPRD1A	2760.14244	0.14795828	0.07387638	2.00278201	0.04520069
20366	RPS2	8848.01815	0.26481855	0.09787899	2.70557096	0.00681871
20368	RPS21	8633.66457	0.24763371	0.11685613	2.11913325	0.03407921
20369	RPS23	6633.31528	0.20298286	0.07863336	2.58138364	0.00984052
20376	RPS27L	2086.67034	-0.4576435	0.07361199	-6.2169696	5.07E-10
20387	RPS6KA1	1932.51862	0.20844429	0.06444532	3.23443639	0.00121883
20388	RPS6KA2	1263.27049	0.26084088	0.07808629	3.34041833	0.00083652
20390	RPS6KA2-IT1	7.62614805	3.05153663	0.88771788	3.43750722	0.0005871
20392	RPS6KA4	5367.62865	-0.290525	0.06080168	-4.7782403	1.77E-06
20393	RPS6KA5	308.101806	0.61321467	0.12301913	4.98470988	6.21E-07
20396	RPS6KB2	2374.13431	-0.2099238	0.08872249	-2.3660722	0.01797794
20401	RPS8	14756.3554	0.45765446	0.0532813	8.58940048	8.74E-18
20403	RPSA	3782.91383	0.22451563	0.06281974	3.57396604	0.00035161
20404	RPSAP52	43.1798781	2.71149933	0.4028966	6.7300129	1.70E-11
20412	RPUSD4	1439.97386	-0.3635998	0.08543215	-4.2560072	2.08E-05
20415	RRAGA	2773.39969	-0.7468821	0.08427819	-8.862105	7.85E-19
20416	RRAGB	686.955993	-0.6627741	0.09224932	-7.1845967	6.74E-13
20417	RRAGC	5437.70014	1.15973976	0.05787943	20.0371656	2.61E-89
20422	RREB1	1888.45604	0.64925848	0.07987516	8.12841515	4.35E-16
20427	RRN3	1491.20206	0.71352814	0.07798411	9.14966055	5.71E-20
20433	RRP12	3673.85696	1.82384227	0.05933283	30.7391759	1.71E-207

20434	RRP15	2037.9698	0.50985621	0.06448223	7.90692616	2.64E-15
20435	RRP1B	2528.0817	0.3836992	0.07329321	5.23512602	1.65E-07
20436	RRP36	2905.79358	0.42098427	0.05853355	7.19218732	6.38E-13
20437	RRP7A	3551.78578	0.53277012	0.07130895	7.47129348	7.94E-14
20439	RRP8	552.986006	0.45285964	0.09549357	4.74230494	2.11E-06
20440	RRP9	1682.32114	1.13972447	0.08268892	13.7832788	3.21E-43
20441	RRS1	1076.79347	0.47600305	0.07982758	5.9628896	2.48E-09
20444	RSAD1	1280.61227	-0.3633401	0.08761872	-4.1468322	3.37E-05
20448	RSC1A1	93.781917	1.01550139	0.23253803	4.36703367	1.26E-05
20451	RSL1D1	6634.72132	0.8149323	0.05453437	14.9434636	1.72E-50
20452	RSL24D1	3294.66462	1.13540252	0.0891611	12.734281	3.81E-37
20465	RSPRY1	1205.74084	0.24670521	0.0778383	3.16945777	0.00152724
20466	RSRC1	640.325115	-0.5556066	0.11298242	-4.9176373	8.76E-07
20467	RSRC2	4168.01589	0.93376356	0.05732468	16.2890316	1.18E-59
20475	RTEL1	113.769008	-1.0047567	0.191961	-5.2341712	1.66E-07
20477	RTF1	2666.6566	-0.4391229	0.06211134	-7.0699306	1.55E-12
20479	RTKN	743.610883	-0.2925127	0.09433637	-3.1007418	0.00193037
20483	RTN2	566.05183	-0.3945507	0.09298516	-4.2431575	2.20E-05
20484	RTN3	9639.87944	-0.3529288	0.05212837	-6.7703795	1.28E-11
20485	RTN4	27719.3099	0.57160985	0.06095124	9.3781491	6.71E-21
20486	RTN4IP1	317.026781	-0.3157944	0.1261804	-2.5027219	0.01232424
20496	RUFY1	3223.84353	-0.8779117	0.06231619	-14.088018	4.50E-45
20500	RUNDC1	1148.15585	0.33710203	0.0717189	4.70032381	2.60E-06
20504	RUNX1	3395.82042	0.53016227	0.06826261	7.76651049	8.07E-15
20512	RUVBL1	2708.97913	0.46206408	0.06420089	7.1971602	6.15E-13
20527	RYBP	3374.89054	0.55492737	0.07008033	7.91844633	2.40E-15
20534	S100A11	12005.2812	0.20217556	0.05088228	3.973398	7.09E-05
20537	S100A14	13.4789514	2.08153199	0.60022972	3.46789226	0.00052456
20538	S100A16	9252.34583	0.36309233	0.05984667	6.06704318	1.30E-09
20540	S100A3	1507.14926	-0.5272943	0.07055224	-7.4738141	7.79E-14
20543	S100A6	51252.3016	-0.3412676	0.0724467	-4.7106024	2.47E-06
20552	S100PBP	1042.18179	0.31910856	0.07619428	4.18809068	2.81E-05
20553	S100Z	6.89534361	2.90804365	0.90847904	3.20100247	0.0013695
20558	S1PR5	31.8355547	-2.6225743	0.42329402	-6.1956328	5.81E-10
20559	SAA1	8734.50496	-0.523807	0.20916171	-2.5043158	0.01226885
20560	SAA2	2365.81096	-0.5206659	0.06705967	-7.7642181	8.22E-15
20565	SAC3D1	1444.92634	-1.0065522	0.12227229	-8.2320551	1.84E-16
20567	SACS	2047.03718	0.30980037	0.08638892	3.58611241	0.00033564
20575	SALL2	109.459192	-2.4528891	0.2288233	-10.719578	8.24E-27
20590	SAMD4A	3622.55931	0.33696993	0.06223518	5.4144607	6.15E-08
20595	SAMD9	1331.64938	0.54202537	0.11532991	4.69978142	2.60E-06
20598	SAMM50	1509.885	-0.2991836	0.07739813	-3.8655142	0.00011086
20604	SAP30	904.235019	-1.1063601	0.10216639	-10.829003	2.51E-27

20610	SAPCD2	1175.40766	-0.5053576	0.07577282	-6.6693784	2.57E-11
20611	SAR1A	6734.12145	-0.1888364	0.04928957	-3.8311639	0.00012754
20613	SARAF	4821.74693	-0.4939917	0.0679903	-7.2656209	3.71E-13
20614	SARDH	747.373899	-1.2713119	0.09031457	-14.076487	5.30E-45
20617	SARS	14895.8429	1.10220061	0.06889784	15.9976072	1.33E-57
20618	SARS2	655.584438	-0.3579692	0.09185807	-3.896981	9.74E-05
20619	SART1	4985.14341	-0.5605286	0.0899473	-6.2317449	4.61E-10
20624	SAT1	3384.57343	0.5343027	0.08846126	6.03996267	1.54E-09
20626	SATB1	303.531746	0.33797875	0.12606464	2.68099569	0.00734035
20635	SBDS	6475.60687	0.57287878	0.0529729	10.8145639	2.94E-27
20636	SBDSP1	1496.85691	0.58208043	0.07262748	8.01460361	1.10E-15
20651	SCAF4	1819.5496	0.36634002	0.07625328	4.80425245	1.55E-06
20652	SCAF8	2434.63351	0.60693499	0.07651784	7.93194106	2.16E-15
20655	SCAMP1-AS1	291.949397	1.35372718	0.13206329	10.2505938	1.18E-24
20656	SCAMP2	1526.79829	-0.3711701	0.10562592	-3.5140055	0.0004414
20659	SCAMP5	524.192316	-0.6007005	0.09969874	-6.0251567	1.69E-09
20663	SCAPER	438.750209	-0.3518924	0.11459456	-3.07076	0.00213515
20664	SCARA3	615.22339	-1.4404531	0.10726124	-13.42939	4.07E-41
20665	SCARA5	13.7126209	-4.753228	0.97736717	-4.8632982	1.15E-06
20666	SCARB1	2193.94883	0.26780745	0.0634116	4.22331964	2.41E-05
20667	SCARB2	5845.10426	-0.469455	0.06808667	-6.8949616	5.39E-12
20668	SCARF1	162.25824	-0.7373476	0.17381509	-4.2421383	2.21E-05
20669	SCARF2	529.777973	-1.0546371	0.10180425	-10.35946	3.79E-25
20696	SCD	14007.5717	-2.2344493	0.06373889	-35.056294	3.13E-269
20700	SCFD1	3378.3104	0.65799001	0.05969778	11.0220178	2.99E-28
20702	SCG2	256.307169	-1.0421944	0.13198083	-7.896559	2.87E-15
20725	SCMH1	2430.19959	0.52286283	0.07587602	6.89101594	5.54E-12
20726	SCML1	1691.5308	0.38838216	0.08051735	4.82358351	1.41E-06
20727	SCML2	383.932054	0.57559175	0.14117993	4.07700831	4.56E-05
20732	SCN1B	257.251771	-1.792523	0.14024648	-12.781234	2.09E-37
20739	SCN5A	995.254211	-0.6922775	0.10176638	-6.8026147	1.03E-11
20742	SCN9A	64.5502141	0.55067683	0.26003545	2.11769907	0.03420056
20744	SCNN1A	1064.66802	-1.5608779	0.08332055	-18.733408	2.64E-78
20748	SCO1	1508.4034	0.62804301	0.06713182	9.35537031	8.33E-21
20754	SCPEP1	2000.45084	-0.4443985	0.07653429	-5.8065278	6.38E-09
20758	SCRN2	908.814707	-0.4243272	0.09245372	-4.5896172	4.44E-06
20767	SCX	166.950525	-0.4514851	0.19416624	-2.3252505	0.02005857
20769	SCYL2	1762.19528	0.69041791	0.08730711	7.9079233	2.62E-15
20770	SCYL3	618.510771	1.05355385	0.08979433	11.7329665	8.64E-32
20771	SDAD1	3206.85163	0.4829795	0.06234971	7.74629921	9.46E-15
20773	SDC2	1698.70561	-2.6634827	0.07555151	-35.253863	2.99E-272
20774	SDC3	2902.55186	-0.2753799	0.06325096	-4.3537665	1.34E-05
20775	SDC4	15292.0255	-0.0992265	0.04952574	-2.0035343	0.04511997

20777	SDCBP	13660.2288	0.4267768	0.05003522	8.52952813	1.47E-17
20780	SDCCAG3	3640.94929	0.43406796	0.06511238	6.66644266	2.62E-11
20782	SDE2	1377.94811	0.67236332	0.07760362	8.66407129	4.55E-18
20783	SDF2	1949.87597	-0.5640561	0.10404785	-5.421122	5.92E-08
20786	SDHA	6555.35351	-0.9685533	0.07288073	-13.289567	2.66E-40
20789	SDHAF3	581.634042	-0.4357661	0.09815901	-4.4393901	9.02E-06
20796	SDHD	3778.18384	-0.411715	0.05748435	-7.1622111	7.94E-13
20797	SDK1	131.037347	-0.9890204	0.20107038	-4.9187771	8.71E-07
20799	SDPR	1836.47554	-1.3319426	0.0763068	-17.455096	3.15E-68
20800	SDR16C5	25.9576614	-1.1780507	0.42204847	-2.7912688	0.00525018
20808	SEC11A	3451.97948	-0.6331324	0.07446932	-8.5019233	1.86E-17
20809	SEC11C	586.401396	0.30644956	0.10084505	3.03881597	0.0023751
20815	SEC14L4	31.0470831	-0.8781469	0.38427545	-2.2852017	0.02230101
20818	SEC16A	4962.65361	0.13155677	0.05956817	2.20850771	0.0272089
20819	SEC16B	55.4399114	-2.2831227	0.3040223	-7.5097211	5.93E-14
20823	SEC22C	2597.86798	-0.3174934	0.06150357	-5.1621939	2.44E-07
20825	SEC23B	3945.2967	0.23031425	0.0565251	4.07454863	4.61E-05
20830	SEC24C	5346.59997	-0.4072525	0.0529999	-7.6840234	1.54E-14
20833	SEC31B	475.376068	-0.5203513	0.10973286	-4.7419822	2.12E-06
20835	SEC61A2	745.364587	0.28340308	0.10148978	2.79242974	0.00523138
20839	SEC63	3461.77498	0.30948808	0.06558686	4.71875103	2.37E-06
20840	SECISBP2	1728.98629	0.23457007	0.06493826	3.61220129	0.00030361
20843	SEH1L	4574.26175	0.3465754	0.05665048	6.11778428	9.49E-10
20848	SELENBP1	481.327608	-1.4628882	0.1069535	-13.677797	1.38E-42
20852	SELO	968.816102	-0.7735969	0.09971771	-7.7578691	8.64E-15
20858	SEMA3B	1919.04193	-1.7876978	0.08332312	-21.455004	4.10E-102
20861	SEMA3D	34.7249248	1.13837643	0.38669649	2.94384988	0.00324157
20863	SEMA3F	215.605098	-0.4303685	0.17105619	-2.5159482	0.01187126
20864	SEMA3G	42.1526294	-1.0917133	0.32347347	-3.37497	0.00073824
20866	SEMA4B	3008.15022	-0.6558128	0.05869596	-11.173049	5.53E-29
20869	SEMA4F	623.790675	-0.7515411	0.10212443	-7.3590722	1.85E-13
20870	SEMA4G	95.2070505	-1.621329	0.22564305	-7.1853707	6.70E-13
20875	SEMA6B	2660.97607	-1.7822749	0.08119341	-21.950979	8.48E-107
20876	SEMA6C	122.544149	-0.7193629	0.18932134	-3.7996928	0.00014488
20877	SEMA6D	3.8359078	-3.4066838	1.39664448	-2.4391918	0.01472015
20878	SEMA7A	1475.39868	0.65395052	0.07256023	9.01251963	2.01E-19
20887	SENP6	4048.77414	0.22994838	0.0585359	3.9283308	8.55E-05
20890	15-Sep	10427.2245	-0.5356961	0.04795441	-11.170945	5.66E-29
20892	SEPHS2	2479.27043	0.14782352	0.06922071	2.13553895	0.03271701
20893	SEPN1	3187.6482	-0.3558803	0.08788824	-4.0492369	5.14E-05
20894	SEPP1	33.9952393	-1.9953513	0.4177468	-4.7764611	1.78E-06
20897	1-Sep	96.563703	-0.9407895	0.24268249	-3.8766271	0.00010591
20903	3-Sep	70.5696115	-0.7711643	0.29349257	-2.6275429	0.0086004

20904	4-Sep	192.108532	-0.8486454	0.14734233	-5.7596848	8.43E-09
20908	6-Sep	721.597417	-0.6514958	0.09219085	-7.0668158	1.59E-12
20909	7-Sep	6954.84505	-0.3844539	0.05511716	-6.9752123	3.05E-12
20911	SEPT7P2	227.887887	0.32844285	0.1407438	2.33362218	0.01961551
20913	8-Sep	2780.40098	-0.3849387	0.05979178	-6.4379862	1.21E-10
20916	SERAC1	707.557727	0.23656185	0.08947378	2.64392385	0.00819511
20917	SERBP1	10545.6585	0.84930953	0.0490134	17.3281098	2.89E-67
20922	SERGEF	556.220405	-0.7397028	0.10353545	-7.1444393	9.04E-13
20924	SERHL2	15.4265188	-1.0168908	0.49806186	-2.0416959	0.04118171
20926	SERINC2	4307.34011	0.97298789	0.06385228	15.2381071	1.98E-52
20932	SERPINA1	6693.35432	-0.4439403	0.05580875	-7.9546716	1.80E-15
20938	SERPINA3	118.307065	-1.8807254	0.2073201	-9.071602	1.17E-19
20940	SERPINA5	47.1576285	-3.9984194	0.48145878	-8.3048011	1.00E-16
20944	SERPINB1	4256.86017	-0.2825458	0.05460981	-5.173902	2.29E-07
20949	SERPINB2	19.8340354	6.80609405	1.24178169	5.4809103	4.23E-08
20952	SERPINB5	2.95516173	3.04723706	1.42482162	2.13867968	0.03246162
20960	SERPINE1	36365.1916	0.95444758	0.04446659	21.4643748	3.35E-102
20961	SERPINE2	923.777788	0.77884309	0.0894969	8.70245915	3.25E-18
20970	SERTAD2	4154.67781	0.53410469	0.06530531	8.17857933	2.87E-16
20975	SESN1	241.655739	-0.5469306	0.13853926	-3.9478385	7.89E-05
20976	SESN2	1151.23142	2.7631556	0.08549202	32.3206256	3.59E-229
20977	SESN3	34.0762077	-1.2829179	0.34595044	-3.7083863	0.00020858
20978	SESTD1	605.272584	-1.006058	0.0953429	-10.551997	4.97E-26
20979	SET	17253.2949	0.3831343	0.05879483	6.51646253	7.20E-11
20985	SETD4	782.084051	0.73515894	0.08741471	8.41001434	4.10E-17
20986	SETD5	5521.41015	0.40198657	0.07621659	5.27426609	1.33E-07
20989	SETD8	1543.72769	0.83225335	0.07155415	11.6310989	2.86E-31
20999	SF1	13858.219	0.4975549	0.06511145	7.64158822	2.15E-14
21000	SF3A1	3801.45232	0.31840376	0.05920522	5.37796763	7.53E-08
21001	SF3A2	3899.22601	0.59143317	0.06421715	9.20989507	3.26E-20
21002	SF3A3	3522.53439	0.57512561	0.0610336	9.42309821	4.38E-21
21006	SF3B4	3398.33684	0.74018546	0.07479233	9.89654179	4.31E-23
21008	SF3B6	2651.97765	0.36724422	0.08129703	4.51731388	6.26E-06
21009	SFI1	587.833799	-0.3983335	0.0912415	-4.3657057	1.27E-05
21010	SFMBT1	981.646535	0.25101934	0.07659822	3.27709096	0.00104883
21012	SFN	3515.31185	0.355723	0.10463528	3.39964699	0.00067473
21020	SFT2D1	3701.79894	-0.2076768	0.06799354	-3.0543601	0.00225541
21023	SFTA1P	723.2273	1.66210136	0.0901816	18.4306038	7.46E-76
21033	SFXN3	2734.53976	-0.5171956	0.07225231	-7.1581875	8.18E-13
21035	SFXN5	415.147735	-0.5369593	0.11127377	-4.8255696	1.40E-06
21053	SGOL2	2563.87792	-0.2715183	0.06548751	-4.1461083	3.38E-05
21054	SGPL1	2991.96614	-0.4959891	0.06133985	-8.0859198	6.17E-16
21059	SGSM2	2851.0227	-0.6161499	0.0597311	-10.315395	6.00E-25

21060	SGSM3	1727.56414	-0.6150517	0.06975681	-8.817085	1.17E-18
21061	SGTA	4904.30532	0.39550484	0.06524445	6.06189233	1.35E-09
21062	SGTB	1216.28407	1.10185434	0.09377364	11.7501504	7.05E-32
21063	SH2B1	1158.34568	-0.7231463	0.07760484	-9.3183146	1.18E-20
21065	SH2B3	3277.77249	0.39357166	0.07038923	5.59136198	2.25E-08
21069	SH2D3A	1467.44628	-0.4342976	0.10100174	-4.2999023	1.71E-05
21073	SH2D5	2138.70166	2.03887223	0.07276331	28.0206068	9.12E-173
21077	SH3BGRL	2028.91274	-2.4059883	0.07270454	-33.092684	3.79E-240
21080	SH3BP1	463.032795	-0.8489092	0.10971559	-7.7373615	1.02E-14
21083	SH3BP5	996.264815	0.9437585	0.08101994	11.6484716	2.34E-31
21085	SH3BP5L	1833.73366	-0.3075781	0.06979881	-4.406639	1.05E-05
21093	SH3GLB1	6482.16214	-0.4542833	0.06747858	-6.732259	1.67E-11
21094	SH3GLB2	3323.43009	-0.3905038	0.06334031	-6.1651696	7.04E-10
21095	SH3KBP1	9326.84859	0.39378956	0.06376921	6.17523042	6.61E-10
21099	SH3RF1	1655.89829	0.65133838	0.07216223	9.02602864	1.78E-19
21103	SH3TC1	146.84702	-0.7578458	0.17390681	-4.3577694	1.31E-05
21104	SH3TC2	1466.01829	-0.9068368	0.08804076	-10.300193	7.03E-25
21105	SH3YL1	406.121865	0.72959447	0.14482622	5.03772353	4.71E-07
21112	SHB	2167.20489	-0.5186531	0.0657291	-7.8907685	3.00E-15
21116	SHC3	633.966268	-0.4054138	0.09154223	-4.4287075	9.48E-06
21123	SHFM1	2477.33892	-0.5185331	0.10292031	-5.0381995	4.70E-07
21124	SHH	106.653772	-0.5516802	0.19346015	-2.8516478	0.00434933
21127	SHISA4	413.159645	-0.409269	0.11339165	-3.60934	0.00030698
21133	SHKBP1	2610.14559	-0.3364714	0.06536952	-5.147221	2.64E-07
21134	SHMT1	1110.02729	-0.6909123	0.07537328	-9.1665418	4.88E-20
21139	SHPK	503.646626	-0.4940526	0.11077531	-4.4599529	8.20E-06
21142	SHROOM1	715.182748	-0.3608813	0.09275629	-3.8906396	1.00E-04
21148	SIAE	1422.6062	-0.5424845	0.07434464	-7.2968876	2.95E-13
21149	SIAH1	892.39188	0.19114131	0.09210296	2.07530039	0.0379587
21150	SIAH2	777.176475	0.22614208	0.08255327	2.73934745	0.00615613
21154	SIGIRR	194.626115	-1.6163188	0.17600183	-9.1835341	4.17E-20
21171	SIK2	1421.90194	0.33687535	0.08272628	4.07216865	4.66E-05
21177	SIMC1	676.752361	0.26586355	0.09758127	2.72453465	0.00643922
21180	SIPA1	2859.42885	-0.7757707	0.0758439	-10.228518	1.48E-24
21182	SIPA1L2	15.0838766	1.87988339	0.57153755	3.28916864	0.00100484
21185	SIRPB1	24.0361627	-4.547518	0.69147522	-6.5765452	4.82E-11
21191	SIRT2	1270.10409	-0.145527	0.06969767	-2.0879749	0.0368001
21192	SIRT3	683.591097	-0.7333191	0.1012553	-7.2422787	4.41E-13
21204	SIX5	925.37792	-0.6421718	0.08992257	-7.1413864	9.24E-13
21207	SKA2	3231.7344	0.25432184	0.06066637	4.19213876	2.76E-05
21213	SKIL	2126.8747	0.42362904	0.06990076	6.06043532	1.36E-09
21216	SKIV2L2	1508.49096	0.17949314	0.06785069	2.64541358	0.00815911
21221	SKP2	771.805467	-1.8844056	0.09745277	-19.336603	2.64E-83

21236	SLC10A5	19.4371505	1.28192978	0.48270057	2.65574534	0.00791333
21240	SLC11A2	2321.94259	0.17413742	0.06453143	2.69848989	0.00696549
21244	SLC12A4	1964.1711	-0.5442475	0.06534161	-8.3292631	8.13E-17
21247	SLC12A7	2652.87857	0.30673867	0.06152435	4.98564657	6.18E-07
21249	SLC12A9	933.150392	-0.6440054	0.07793833	-8.2630122	1.42E-16
21263	SLC16A1	178.179827	2.05456496	0.17590626	11.6798856	1.62E-31
21271	SLC16A2	99.6145607	-0.9450539	0.20300303	-4.6553685	3.23E-06
21272	SLC16A3	13211.6231	-0.8317129	0.06023041	-13.808854	2.25E-43
21273	SLC16A4	80.3888035	-1.5621993	0.23832497	-6.5549123	5.57E-11
21283	SLC17A5	4289.08435	-0.6857832	0.06432501	-10.661222	1.55E-26
21287	SLC17A9	2652.41078	0.26853083	0.06614236	4.05989183	4.91E-05
21291	SLC18B1	835.94525	-0.3806324	0.0932914	-4.0800373	4.50E-05
21298	SLC1A4	572.351324	0.38172252	0.09290679	4.10866105	3.98E-05
21299	SLC1A5	11568.7745	1.44083982	0.0471149	30.5814023	2.16E-205
21301	SLC1A7	20.9112058	-1.1652227	0.43749802	-2.6633783	0.00773604
21302	SLC20A1	10396.3102	0.45739156	0.05455536	8.38398988	5.12E-17
21310	SLC22A15	549.481521	1.4191966	0.10383277	13.6680993	1.57E-42
21313	SLC22A18	457.12458	-0.3222343	0.10594696	-3.041468	0.00235428
21316	SLC22A20	35.6962801	-1.430343	0.34383889	-4.1599222	3.18E-05
21330	SLC23A3	64.4601218	0.62681749	0.2525551	2.48190387	0.01306825
21336	SLC25A1	2526.60871	-0.6750735	0.08074529	-8.3605309	6.24E-17
21337	SLC25A10	1790.72692	-0.9461144	0.08854766	-10.684805	1.20E-26
21338	SLC25A11	1717.13487	-0.333199	0.08173654	-4.0764992	4.57E-05
21339	SLC25A12	847.680739	-0.594925	0.08080702	-7.3622938	1.81E-13
21346	SLC25A19	411.829596	0.68367468	0.11523013	5.93312417	2.97E-09
21351	SLC25A22	4295.33052	1.09632035	0.06703148	16.3553051	3.99E-60
21352	SLC25A23	1946.43817	-0.4910077	0.08517525	-5.7646756	8.18E-09
21354	SLC25A25	1721.13701	1.156171	0.08271855	13.9771667	2.15E-44
21364	SLC25A32	3048.53091	0.78391968	0.062116	12.6202538	1.63E-36
21365	SLC25A33	330.875023	0.59584268	0.12205035	4.88194148	1.05E-06
21367	SLC25A35	124.655162	-0.5373247	0.17725858	-3.0313044	0.002435
21370	SLC25A38	1875.56957	0.33313614	0.07335989	4.54112125	5.60E-06
21373	SLC25A4	1774.71928	-0.923677	0.08638768	-10.69223	1.11E-26
21376	SLC25A42	200.550653	-1.0087363	0.16594712	-6.0786613	1.21E-09
21378	SLC25A44	1684.46841	0.28891429	0.07960187	3.62949126	0.00028398
21380	SLC25A46	2270.8661	-0.2105542	0.0685731	-3.0705069	0.00213696
21393	SLC26A2	1346.27956	-0.6242368	0.1051092	-5.9389357	2.87E-09
21398	SLC26A6	1169.8214	-0.7145376	0.0716614	-9.9710242	2.04E-23
21399	SLC26A7	38.3954569	0.920614	0.32034918	2.87378289	0.00405588
21402	SLC27A1	1082.08824	-1.0553411	0.08224719	-12.831334	1.09E-37
21404	SLC27A3	193.993497	-1.1108173	0.17265741	-6.4336499	1.25E-10
21411	SLC29A1	6145.34674	0.20520802	0.05367609	3.82308061	0.00013179
21417	SLC2A10	133.257729	-1.6962963	0.19984506	-8.4880569	2.10E-17

21419	SLC2A12	382.076808	-1.6154632	0.13449664	-12.011179	3.10E-33
21420	SLC2A13	167.099224	-1.0766546	0.17384544	-6.1931716	5.90E-10
21425	SLC2A4RG	5010.42786	-0.6348064	0.07083276	-8.9620445	3.19E-19
21426	SLC2A5	44.0957795	-1.1928752	0.30937838	-3.8557161	0.00011539
21440	SLC30A9	3219.76712	-0.2840754	0.07361576	-3.8588931	0.0001139
21442	SLC31A2	2399.80802	0.25635699	0.06093978	4.20672631	2.59E-05
21451	SLC35A4	4152.55663	-0.3971158	0.05702318	-6.9641122	3.30E-12
21453	SLC35B1	1895.37009	-0.2373488	0.08267406	-2.8708981	0.00409307
21454	SLC35B2	3836.44115	-0.4103539	0.06069085	-6.7613802	1.37E-11
21455	SLC35B3	1356.89085	0.25160642	0.07790305	3.22973783	0.00123904
21464	SLC35E2B	2326.89968	0.17935949	0.07147417	2.50943084	0.01209259
21468	SLC35F2	5263.77121	0.60106718	0.06373605	9.43056872	4.08E-21
21479	SLC36A1	1033.94609	0.69790781	0.0978083	7.135466	9.65E-13
21483	SLC37A1	358.611791	-0.7872488	0.11965669	-6.5792291	4.73E-11
21485	SLC37A3	2897.63224	-0.363193	0.07467971	-4.8633427	1.15E-06
21486	SLC37A4	2359.77031	-0.4855142	0.06652987	-7.2976882	2.93E-13
21487	SLC38A1	12471.4922	0.441889	0.06012092	7.35000364	1.98E-13
21490	SLC38A2	24665.4904	0.28056045	0.07477815	3.7519039	0.0001755
21492	SLC38A4	696.500031	-1.6145275	0.09974071	-16.187246	6.20E-59
21494	SLC38A6	530.203236	-0.7590505	0.1035868	-7.3276764	2.34E-13
21498	SLC39A1	12087.3126	0.33703969	0.06436072	5.23672989	1.63E-07
21499	SLC39A10	3962.71577	-0.7075848	0.08482458	-8.3417423	7.32E-17
21500	SLC39A11	466.077264	-0.3750702	0.1144264	-3.2778292	0.00104609
21503	SLC39A13	4294.23492	-0.2420387	0.06824351	-3.5466922	0.0003901
21504	SLC39A14	7430.11449	0.84279419	0.06692947	12.5922736	2.33E-36
21512	SLC39A9	3068.47136	-0.4530147	0.07038154	-6.4365554	1.22E-10
21514	SLC3A2	15141.0102	1.79708627	0.06927724	25.940501	2.33E-148
21515	SLC40A1	202.743901	-2.9635588	0.17929703	-16.528767	2.28E-61
21519	SLC43A1	1026.71658	1.26419484	0.08230495	15.3598886	3.04E-53
21520	SLC43A2	318.146405	-0.9386229	0.12053288	-7.7872764	6.85E-15
21521	SLC43A3	18253.8527	0.46170586	0.06036521	7.64854247	2.03E-14
21522	SLC44A1	2757.47245	-1.0516279	0.07460853	-14.095277	4.06E-45
21523	SLC44A2	8320.95433	-0.4942754	0.04806918	-10.282584	8.44E-25
21527	SLC45A1	88.19255	-0.6637656	0.24233365	-2.7390567	0.00616157
21531	SLC46A1	1091.53244	-0.2393061	0.09580967	-2.4977236	0.01249936
21534	SLC47A1	148.514934	-1.9214555	0.18385524	-10.450915	1.45E-25
21541	SLC4A2	10141.6277	-0.7531314	0.04794306	-15.708871	1.31E-55
21544	SLC4A5	33.6169824	1.14906744	0.39751691	2.89061275	0.00384492
21545	SLC4A7	1397.70237	0.60361559	0.095668	6.30948306	2.80E-10
21572	SLC6A14	10.0746296	-3.8693601	0.95700952	-4.0431783	5.27E-05
21583	SLC6A6	3584.01243	-0.267578	0.07723896	-3.464288	0.00053164
21585	SLC6A8	191.249382	-0.4858653	0.15850053	-3.0653859	0.00217389
21586	SLC6A9	1230.25713	1.23068764	0.07284285	16.8951053	4.89E-64

21587	SLC7A1	7512.72516	0.83974011	0.08786941	9.55668324	1.22E-21
21589	SLC7A11	3298.26793	1.56565251	0.06243048	25.0783372	8.57E-139
21590	SLC7A11-AS1	5.69433891	2.95061057	1.01734712	2.90029874	0.00372807
21593	SLC7A2	1121.19649	0.95348801	0.08947129	10.6569162	1.62E-26
21596	SLC7A5	12288.1205	1.05919264	0.05717209	18.5263923	1.26E-76
21599	SLC7A6	2233.77357	0.3430039	0.07590645	4.51877155	6.22E-06
21608	SLC8B1	3158.26691	-0.4674141	0.07427607	-6.2929299	3.12E-10
21609	SLC9A1	1337.724	1.04585823	0.07568882	13.8178689	1.99E-43
21616	SLC9A6	1511.13821	-0.9576918	0.0856698	-11.178873	5.17E-29
21617	SLC9A7	2474.36518	-0.4489285	0.06178444	-7.2660439	3.70E-13
21618	SLC9A7P1	33.5410084	-0.958259	0.34439388	-2.7824508	0.005395
21620	SLC9A9	13.1735655	1.43311918	0.59448134	2.41070506	0.01592172
21625	SLC9C2	9.96729744	-1.6044586	0.65534488	-2.448266	0.01435456
21633	SLCO3A1	1313.8558	-0.6133567	0.07164596	-8.5609385	1.12E-17
21634	SLCO4A1	1208.56033	-2.0330895	0.07629822	-26.646617	1.96E-156
21635	SLCO4A1-AS1	587.551039	-1.2377463	0.09788636	-12.644727	1.20E-36
21641	SLF2	2391.62098	-0.1684211	0.08203654	-2.0530011	0.04007247
21643	SLFN12	938.6024	0.48323218	0.0866544	5.57654537	2.45E-08
21644	SLFN12L	58.4027261	0.53900205	0.26435856	2.03890525	0.04145948
21645	SLFN13	727.395506	-0.7490745	0.10221204	-7.3286333	2.33E-13
21647	SLFN5	1596.7742	1.34504711	0.08853436	15.1923748	3.97E-52
21649	SLFNL1-AS1	50.7195411	1.06568471	0.2883616	3.69565397	0.00021932
21663	SLMAP	3894.00389	-0.173457	0.06726392	-2.5787523	0.00991579
21664	SLMO1	405.8633	1.42167472	0.14431988	9.85085822	6.80E-23
21665	SLMO2	4816.19092	0.50748673	0.05188762	9.78049715	1.37E-22
21670	SLU7	2431.64948	0.54839405	0.05920028	9.26336965	1.98E-20
21680	SMAD1	339.872375	-0.8000702	0.11942466	-6.6993723	2.09E-11
21683	SMAD2	3340.1036	0.20487749	0.06658932	3.0767318	0.00209284
21688	SMAD6	245.160101	-2.0412117	0.14371211	-14.203477	8.72E-46
21693	SMAP2	943.215092	0.55883811	0.07927996	7.04892025	1.80E-12
21696	SMARCA4	6019.85872	-0.5799881	0.05372501	-10.795495	3.62E-27
21701	SMARCB1	2499.76851	-0.480638	0.07700187	-6.2418997	4.32E-10
21702	SMARCC1	3386.87231	0.24165909	0.07510044	3.21781214	0.00129172
21708	SMC1A	7292.07799	-0.576776	0.0693864	-8.3125214	9.37E-17
21713	SMC4	8633.65311	0.24620743	0.0598178	4.11595565	3.86E-05
21721	SMCO4	356.851377	-0.7898679	0.11630754	-6.7912017	1.11E-11
21725	SMCR8	2516.13795	0.48452714	0.09586185	5.05443132	4.32E-07
21727	SMG1	3772.08048	0.39659464	0.10209936	3.88439894	0.00010258
21734	SMG6	2081.08929	-0.2540819	0.06615548	-3.8406781	0.00012269
21735	SMG7	3669.04282	0.8707411	0.09459039	9.20538667	3.40E-20
21738	SMG9	3455.9374	0.70730615	0.07449179	9.49508818	2.20E-21
21741	SMIM10L1	738.304094	-0.5149159	0.09025497	-5.7051256	1.16E-08
21745	SMIM12	1904.23765	0.47527025	0.09050193	5.25149317	1.51E-07

21746	SMIM13	3329.86778	0.68646137	0.06750954	10.1683615	2.74E-24
21747	SMIM14	296.687911	-0.7483743	0.13234784	-5.6546018	1.56E-08
21748	SMIM15	4632.96781	0.26530932	0.06119697	4.33533397	1.46E-05
21753	SMIM2-AS1	72.0754175	1.42409619	0.26997488	5.27492113	1.33E-07
21760	SMIM3	520.175251	0.3605922	0.09450012	3.81578556	0.00013575
21761	SMIM4	387.914807	0.45096803	0.15839375	2.84713282	0.0044115
21764	SMIM7	2602.8183	-0.2069837	0.06976079	-2.9670494	0.00300673
21767	SMKR1	96.879159	0.51775726	0.19938694	2.59674615	0.00941114
21775	SMOX	1137.7162	1.05511167	0.07943322	13.2830028	2.91E-40
21776	SMPD1	2508.90582	-0.6493843	0.06686078	-9.7124849	2.67E-22
21780	SMPDL3A	340.940762	-0.8880058	0.11638898	-7.6296379	2.35E-14
21791	SMURF1	3246.8798	0.5516881	0.05642797	9.77685569	1.42E-22
21792	SMURF2	7321.1984	0.21767275	0.06572471	3.31188625	0.00092669
21797	SMYD5	1520.87806	0.37177091	0.0684276	5.43305468	5.54E-08
21798	SNAI1	36.6801755	0.68967133	0.33985136	2.02933228	0.04242446
21805	SNAP29	1553.87595	0.47198224	0.06999766	6.74282875	1.55E-11
21806	SNAP47	1675.69936	-0.2575997	0.08113864	-3.1748092	0.00149935
21808	SNAPC1	6775.26708	1.30548628	0.053794	24.2682524	4.24E-130
21813	SNAPIN	697.540593	-0.8009871	0.08704131	-9.2023786	3.50E-20
21846	SNCG	521.49193	-1.3553942	0.12179443	-11.12854	9.11E-29
21849	SNED1	60.8414181	-1.1404178	0.29726607	-3.8363537	0.00012487
21851	SNHG1	6279.66307	1.86936129	0.05825533	32.0891008	6.26E-226
21854	SNHG12	474.041368	0.71487972	0.10190106	7.01542994	2.29E-12
21855	SNHG15	2062.34836	1.98455976	0.1081637	18.3477422	3.44E-75
21857	SNHG17	1162.80735	1.20866351	0.11642193	10.3817511	3.00E-25
21865	SNHG3	2402.5685	0.92489094	0.076051	12.1614562	4.99E-34
21866	SNHG4	224.610168	0.63299887	0.13579598	4.66139636	3.14E-06
21870	SNHG8	1762.88531	0.74029293	0.13115253	5.64451888	1.66E-08
21873	SNN	902.823189	-0.9702261	0.08191156	-11.844801	2.29E-32
21947	SNORA67	75.7826133	0.80988438	0.22840071	3.54589261	0.00039129
21950	SNORA70	72.8451783	0.59255159	0.26209933	2.26079018	0.02377225
21957	SNORA71A	31.4151341	1.25137224	0.40537025	3.08698589	0.00202197
21959	SNORA71C	58.681248	1.86863825	0.29200209	6.39940036	1.56E-10
21963	SNORA73A	204.741589	2.87161415	1.02043773	2.81410031	0.0048914
21964	SNORA73B	259.812871	3.04160719	0.96254931	3.15994948	0.00157796
21967	SNORA75	5.34528302	1.8971092	0.93491063	2.02918775	0.04243917
21981	SNORA9	24.2661394	1.14713423	0.4451065	2.57721294	0.00996006
22141	SNORD17	48.150892	2.13224232	0.45498314	4.68642053	2.78E-06
22155	SNORD24	20.3500295	1.55357004	0.48274156	3.2182231	0.00128987
22189	SNORD47	133.363454	0.99346826	0.18481977	5.37533547	7.64E-08
22230	SNORD76	121.066747	0.62556602	0.19341524	3.23431614	0.00121934
22261	SNPH	529.851479	-0.9536778	0.09849698	-9.6823055	3.59E-22
22262	SNRK	1097.16265	0.29713363	0.08503494	3.49425343	0.00047539

22264	SNRNP200	11512.7393	0.17586713	0.05865094	2.99853887	0.00271278
22265	SNRNP25	672.78887	-0.7022713	0.11960019	-5.8718247	4.31E-09
22267	SNRNP35	721.718708	-0.2541137	0.09634047	-2.637663	0.00834795
22268	SNRNP40	1970.00595	0.17142951	0.06494485	2.63961674	0.00829998
22269	SNRNP48	800.934023	0.21545187	0.08324437	2.58818539	0.0096483
22270	SNRNP70	8254.56767	-0.6058089	0.07688157	-7.879768	3.28E-15
22272	SNRPA1	882.566063	-0.3105644	0.08184417	-3.7945815	0.00014789
22284	SNTA1	1369.99379	-1.0221005	0.10016512	-10.204155	1.90E-24
22286	SNTB2	1249.60294	-0.5969287	0.08439735	-7.0728367	1.52E-12
22290	SNU13	4565.69858	0.39347343	0.06844453	5.74879265	8.99E-09
22293	SNW1	3754.0522	0.30300111	0.06014552	5.03780015	4.71E-07
22294	SNX1	2957.9214	-0.3809131	0.0574654	-6.6285651	3.39E-11
22299	SNX14	2801.93742	-0.3422771	0.06422469	-5.3293698	9.86E-08
22301	SNX16	892.36023	0.57952234	0.09095797	6.37131996	1.87E-10
22309	SNX24	1637.86185	0.38700957	0.06598378	5.86522315	4.49E-09
22311	SNX27	1079.71289	-0.3002025	0.09392693	-3.1961283	0.00139285
22315	SNX3	5980.80487	-0.2418837	0.06458939	-3.7449451	0.00018043
22319	SNX33	965.039491	-0.9904043	0.09046676	-10.947715	6.81E-28
22320	SNX4	982.988604	-0.3800196	0.08929077	-4.2559783	2.08E-05
22323	SNX7	2203.24175	-0.9572666	0.06528402	-14.663108	1.11E-48
22324	SNX8	4372.19852	0.2880089	0.06513175	4.4219436	9.78E-06
22326	SOAT1	7906.3165	0.58945742	0.05194644	11.3474077	7.64E-30
22329	SOCS1	259.813617	-0.6108214	0.1546239	-3.9503682	7.80E-05
22330	SOCS2	327.524965	2.27231689	0.13274751	17.1175859	1.10E-65
22331	SOCS2-AS1	10.2117913	4.34738162	1.03834268	4.1868467	2.83E-05
22332	SOCS3	871.426735	-0.7658202	0.08459146	-9.053162	1.39E-19
22333	SOCS4	1958.01106	0.70237145	0.09408403	7.46536312	8.31E-14
22344	SON	10416.7602	0.20574638	0.05305858	3.87772118	0.00010544
22346	SORBS2	339.184034	-1.4809654	0.14257837	-10.387027	2.84E-25
22347	SORBS3	2532.31252	-0.5303921	0.07911439	-6.7041163	2.03E-11
22353	SORL1	1347.57781	-1.448558	0.0864176	-16.762302	4.60E-63
22354	SORT1	7162.52338	-0.7762518	0.05344683	-14.523813	8.56E-48
22355	SOS1	1461.42469	0.23695325	0.09814175	2.41439806	0.01576123
22361	SOWAHC	1395.24351	0.72676164	0.07744215	9.38457485	6.32E-21
22367	SOX13	1625.59209	-0.2930008	0.0673258	-4.3519836	1.35E-05
22378	SOX4	107.355253	-0.8945751	0.20421507	-4.3805538	1.18E-05
22391	SP2-AS1	107.584803	1.2377876	0.20951241	5.90794399	3.46E-09
22393	SP4	249.354116	0.70736494	0.14554427	4.86013604	1.17E-06
22394	SP5	145.504487	-1.602559	0.19528662	-8.206189	2.28E-16
22395	SP6	196.630101	-0.4301328	0.14653208	-2.9354171	0.003331
22405	SPACA6P	391.762881	-1.3816102	0.12316924	-11.217169	3.36E-29
22414	SPAG5	3715.1007	-0.6868686	0.05922222	-11.598157	4.21E-31
22417	SPAG7	1391.77586	-0.2862529	0.09043712	-3.165215	0.00154968

22419	SPAG9	7115.76463	0.76370347	0.06536752	11.6832257	1.55E-31
22445	SPATA20	3479.14824	-0.3733185	0.06417257	-5.817416	5.98E-09
22484	SPC24	301.324352	-0.821204	0.12675582	-6.4786297	9.26E-11
22485	SPC25	1002.64735	-0.740294	0.08005682	-9.2471071	2.31E-20
22489	SPDEF	79.8592083	-0.9619627	0.2254617	-4.2666344	1.98E-05
22503	SPECC1L	1569.93916	0.25794622	0.07608395	3.39028405	0.0006982
22509	SPEN	2699.10152	0.46909021	0.102958	4.55613152	5.21E-06
22516	SPG7	2271.83592	-0.2043579	0.07193197	-2.840988	0.0044974
22518	SPHK1	1121.19612	2.11259788	0.13745455	15.3694289	2.62E-53
22519	SPHK2	1075.05126	-0.4513645	0.0838173	-5.3850991	7.24E-08
22525	SPIDR	1555.5159	-0.7060938	0.06569386	-10.748247	6.04E-27
22541	SPINT1	765.654756	-1.2623263	0.08829984	-14.295907	2.32E-46
22545	SPIRE1	4472.15405	0.85869539	0.06510794	13.1887971	1.02E-39
22546	SPIRE2	456.72327	-0.6703144	0.13603691	-4.9274453	8.33E-07
22549	SPNS2	140.891752	-0.919192	0.20045566	-4.5855126	4.53E-06
22553	SPOCK1	139.43559	3.17358017	0.23300999	13.6199318	3.05E-42
22563	SPPL2B	1948.02131	-0.3039811	0.07254627	-4.1901688	2.79E-05
22565	SPPL3	2482.34211	-0.4761363	0.05979029	-7.9634382	1.67E-15
22566	SPR	1517.01369	-0.6904256	0.11446696	-6.0316581	1.62E-09
22569	SPRED3	387.769176	1.431279	0.13236233	10.8133407	2.98E-27
22583	SPRTN	878.814664	0.33204419	0.09667117	3.4347799	0.00059304
22584	SPRY1	338.708896	-1.4072374	0.12669355	-11.107411	1.15E-28
22585	SPRY2	2469.15709	0.32482246	0.06044092	5.37421463	7.69E-08
22587	SPRY4	2803.95035	0.63713563	0.06651407	9.5789596	9.80E-22
22592	SPSB1	995.822718	0.25688999	0.09018519	2.8484721	0.00439297
22597	SPTAN1	17068.7599	0.2217393	0.04927487	4.50004853	6.79E-06
22598	SPTB	50.4144774	-1.1071616	0.31667998	-3.4961528	0.00047202
22600	SPTBN2	827.036169	-0.2123044	0.0898228	-2.3635911	0.01809878
22603	SPTLC1	3753.01579	-0.2323336	0.05901236	-3.9370324	8.25E-05
22604	SPTLC2	1858.91832	-0.7705111	0.07094816	-10.860198	1.78E-27
22614	SQSTM1	51776.8566	1.03572396	0.06907378	14.9944599	7.98E-51
22616	SRBD1	650.591231	0.5918069	0.09332987	6.34102375	2.28E-10
22617	SRC	2784.10912	0.26651136	0.06804186	3.91687377	8.97E-05
22619	SRCIN1	32.2415264	1.67797948	0.36458917	4.60238438	4.18E-06
22620	SRD5A1	1036.65102	-0.799247	0.07915184	-10.097643	5.66E-24
22623	SRD5A3	484.537843	-0.3307224	0.10549162	-3.1350585	0.0017182
22625	SREBF1	4635.38175	-0.9529509	0.05589009	-17.050445	3.47E-65
22626	SREBF2	5562.8689	-0.6024989	0.05524966	-10.905025	1.09E-27
22629	SRF	3618.09423	0.46560488	0.06461589	7.20573287	5.77E-13
22630	SRFBP1	975.764905	1.13207286	0.0942926	12.0059572	3.31E-33
22632	SRGAP2	2143.5572	-0.178922	0.08548629	-2.0929907	0.03634998
22639	SRGN	1103.24407	-0.5236798	0.08196306	-6.3892171	1.67E-10
22640	SRI	3466.4099	-0.4139659	0.05887125	-7.0317159	2.04E-12

22642	SRM	4797.61196	0.44409904	0.07682895	5.7803607	7.45E-09
22645	SRP14-AS1	37.7421114	1.64805395	0.35129736	4.69133596	2.71E-06
22646	SRP19	1769.84645	0.16031243	0.06440941	2.48895969	0.01281175
22648	SRP68	5312.88972	0.23298709	0.05516324	4.22359332	2.40E-05
22649	SRP72	5878.57623	0.12158047	0.05961237	2.03951759	0.0413984
22651	SRPK1	11819.6266	0.84829293	0.06072748	13.9688479	2.42E-44
22652	SRPK2	2291.20335	0.56694446	0.06763576	8.38231779	5.19E-17
22654	SRPR	7256.99935	0.27205901	0.06234599	4.36369734	1.28E-05
22655	SRPRB	1417.12224	0.47461802	0.07599697	6.24522329	4.23E-10
22659	SRRD	735.990394	0.79724798	0.08536465	9.33932212	9.70E-21
22674	SRSF5	6156.02366	-0.3241909	0.06838602	-4.7406024	2.13E-06
22675	SRSF6	8394.77725	-0.1141094	0.0497613	-2.2931347	0.02184025
22676	SRSF7	4287.49721	-0.476491	0.07465061	-6.3829478	1.74E-10
22681	SS18	3878.06872	0.4312026	0.07570849	5.69556476	1.23E-08
22686	SSBP2	191.007261	-1.00079	0.19053249	-5.2525948	1.50E-07
22694	SSH2	798.036953	-0.3292835	0.09366673	-3.5154803	0.00043896
22695	SSH3	1122.46944	-0.4900944	0.07752472	-6.3217822	2.59E-10
22697	SSNA1	2691.71366	-0.3069069	0.09275518	-3.3087843	0.00093702
22698	SSPN	141.917675	-0.5908083	0.17277074	-3.4196088	0.00062711
22701	SSR2	5294.45419	-0.4730058	0.05963417	-7.9317911	2.16E-15
22702	SSR3	6772.45666	0.14664575	0.05217994	2.81038554	0.00494822
22705	SSRP1	13362.2914	0.12866647	0.05489701	2.34377919	0.01908947
22736	ST3GAL1	1610.38124	0.90533671	0.09249904	9.78752578	1.27E-22
22738	ST3GAL3	559.34504	-0.1885119	0.09428051	-1.9994787	0.04555659
22739	ST3GAL4	2628.5839	-0.6065794	0.06273473	-9.668957	4.09E-22
22741	ST3GAL5	555.862802	-1.627451	0.10273078	-15.841902	1.60E-56
22743	ST3GAL6	662.492354	-0.2346683	0.09275024	-2.5301099	0.01140268
22745	ST5	2181.41227	-1.2045309	0.06494535	-18.546837	8.65E-77
22749	ST6GALNAC2	71.496481	-1.2547474	0.24723057	-5.0752115	3.87E-07
22754	ST7	680.785259	-0.2573681	0.08576511	-3.000848	0.00269229
22759	ST7L	572.259513	0.7453194	0.09979174	7.46874835	8.10E-14
22760	ST8SIA1	70.2771233	-0.8233126	0.24932446	-3.3021732	0.00095939
22763	ST8SIA4	119.12076	-0.6583385	0.18691201	-3.522184	0.00042801
22772	STAG1	1490.80749	0.25692485	0.07520316	3.41641017	0.00063453
22780	STAG3L5P-P\	254.861354	-0.3126853	0.13122783	-2.382767	0.01718307
22781	STAM	2689.77318	0.37833898	0.07785599	4.85947191	1.18E-06
22790	STARD13	799.132139	0.26725087	0.08463551	3.15766844	0.00159036
22792	STARD3	2265.05244	-0.581115	0.06664354	-8.7197492	2.79E-18
22801	STARD9	591.846532	-0.7441561	0.11470219	-6.4877233	8.71E-11
22802	STAT1	5546.87408	-0.1534154	0.06040958	-2.5395875	0.01109833
22803	STAT2	2831.26158	-0.2646034	0.06142496	-4.3077509	1.65E-05
22806	STAT5A	1175.30266	-0.2097297	0.08230502	-2.5482002	0.01082803
22814	STC1	4328.05113	-0.5140899	0.08245205	-6.2350162	4.52E-10

22820	STEAP3	3408.23261	-0.8812986	0.06097573	-14.453269	2.39E-47
22824	STIL	1691.86706	0.48136811	0.09766175	4.92893174	8.27E-07
22825	STIM1	3725.67905	-0.1505531	0.05725908	-2.6293322	0.00855527
22827	STIP1	12051.5243	0.18787335	0.063531	2.95719179	0.00310455
22829	STK11	4254.22428	0.23323256	0.07113367	3.27879266	0.00104252
22830	STK11IP	707.475743	-0.2102274	0.10489267	-2.0042145	0.04504709
22831	STK16	1071.90007	-0.4145381	0.08245502	-5.0274459	4.97E-07
22833	STK17B	2105.53926	0.18248455	0.06930409	2.63309935	0.00846096
22834	STK19	472.804966	0.72430602	0.10932782	6.62508455	3.47E-11
22836	STK25	3481.7053	-0.1585118	0.05620443	-2.8202729	0.00479828
22837	STK26	971.75858	0.60769105	0.07819575	7.77140809	7.76E-15
22844	STK35	1396.69539	0.39520465	0.07019628	5.62999442	1.80E-08
22845	STK36	621.076862	-0.3620357	0.09503876	-3.809348	0.00013933
22848	STK39	2080.44002	-0.2412176	0.06928452	-3.4815506	0.00049852
22849	STK4	3467.06235	0.60960089	0.06328431	9.63273323	5.82E-22
22851	STK40	6510.34535	1.0079319	0.05260327	19.1610136	7.83E-82
22855	STMN3	1066.7315	-1.727225	0.08868229	-19.476549	1.74E-84
22858	STOM	871.843966	1.03546927	0.08201532	12.6253146	1.53E-36
22860	STOML2	5825.05527	0.29937163	0.07851933	3.81271247	0.00013745
22867	STPG1	987.062825	1.70741404	0.10908929	15.6515277	3.24E-55
22873	STRADA	701.685451	-0.2440122	0.1127868	-2.1634821	0.03050412
22874	STRADB	1596.0514	-0.5129518	0.07466057	-6.8704516	6.40E-12
22883	STS	4867.44179	-0.765945	0.07248867	-10.56641	4.27E-26
22887	STX10	2409.23371	-0.5142069	0.08936037	-5.754306	8.70E-09
22889	STX12	1240.80078	0.60334375	0.06970396	8.65580284	4.89E-18
22894	STX18	1361.41535	0.32807688	0.08398691	3.90628587	9.37E-05
22898	STX1A	1038.83036	0.33749726	0.07948879	4.24584731	2.18E-05
22901	STX3	6673.69071	0.44565619	0.05304301	8.40178955	4.40E-17
22909	STXBP3	2133.02972	-0.3630028	0.06896222	-5.263793	1.41E-07
22916	STYX	1253.31731	0.39931389	0.0876012	4.55831538	5.16E-06
22921	SUCLG2	2530.18131	-0.3348085	0.06273045	-5.337257	9.44E-08
22924	SUCO	3417.73651	0.25717919	0.06620435	3.88462667	0.00010249
22926	SUFU	880.933757	-0.5026083	0.09526922	-5.2756635	1.32E-07
22935	SULT1A1	15.0622547	-1.0601485	0.53094273	-1.9967285	0.04585468
22949	SUMF1	1228.65918	-0.76263	0.09036126	-8.4397899	3.18E-17
22950	SUMF2	7110.90923	-0.1336402	0.05450197	-2.4520247	0.01420549
22951	SUMO1	4974.06212	-0.2749141	0.06017416	-4.568641	4.91E-06
22958	SUN2	4981.2291	-0.7042688	0.05712785	-12.327941	6.41E-35
22959	SUN3	251.493335	2.011578	0.1591401	12.6402961	1.27E-36
22961	SUOX	205.342684	-1.1951043	0.15971016	-7.4829568	7.27E-14
22967	SUPT4H1	2868.01313	-0.1172246	0.05933008	-1.9758035	0.04817703
22968	SUPT5H	8004.01192	0.56976964	0.05095926	11.1808859	5.06E-29
22975	SURF6	2443.45148	0.31150691	0.09553517	3.26065147	0.00111157

22976	SUSD1	1772.61459	-0.5123914	0.07995696	-6.4083405	1.47E-10
22977	SUSD2	47.8552342	-1.6653387	0.30623583	-5.4380922	5.39E-08
22979	SUSD4	75.3723971	1.63096675	0.24715822	6.59887736	4.14E-11
22980	SUSD5	111.301678	-0.3865899	0.1869033	-2.068395	0.0386029
22982	SUV39H1	1243.35662	-0.6599122	0.08548493	-7.719632	1.17E-14
22983	SUV39H2	1257.66995	0.37246218	0.0780385	4.77280014	1.82E-06
22985	SUV420H2	197.002937	-0.4861753	0.16554967	-2.9367338	0.00331689
22996	SVIP	600.173791	0.35782289	0.09517211	3.75974517	0.00017009
22998	SVOPL	9.59279693	-1.7078395	0.69010414	-2.4747562	0.01333271
22999	SWAP70	5317.08886	0.32363446	0.06016615	5.37901199	7.49E-08
23000	SWI5	394.365161	0.39734455	0.11100024	3.57967297	0.00034402
23002	SWT1	264.139595	0.47287723	0.14218434	3.32580396	0.00088164
23003	SYAP1	4481.96809	0.26615505	0.05438798	4.89363759	9.90E-07
23004	SYBU	92.0705033	1.99316216	0.24697987	8.07014006	7.02E-16
23011	SYCP2	52.0451439	-1.0370856	0.29505392	-3.5149021	0.00043992
23012	SYCP2L	137.735538	-0.8295404	0.17168292	-4.8318166	1.35E-06
23015	SYDE2	273.251042	0.6474198	0.15800521	4.09745863	4.18E-05
23033	SYNGR2	7336.54776	-0.6482117	0.06720863	-9.6447678	5.17E-22
23036	SYNJ1	611.230954	0.97095402	0.11457969	8.47404984	2.37E-17
23037	SYNJ2	7473.86111	-0.2922977	0.05391268	-5.4216869	5.90E-08
23041	SYNM	1412.17987	-1.0393685	0.07618441	-13.642799	2.23E-42
23042	SYNPO	1024.23266	-1.7398999	0.10131006	-17.174008	4.16E-66
23043	SYNPO2	24.5475724	-1.2072245	0.40252182	-2.9991528	0.00270731
23047	SYNRG	1537.02894	0.26579589	0.07873185	3.37596398	0.00073558
23052	SYS1	2445.27662	0.45985703	0.06056345	7.59297886	3.13E-14
23054	SYT1	156.478523	1.61495963	0.173574	9.30415639	1.35E-20
23057	SYT12	885.999297	-2.5862557	0.09449725	-27.368581	6.49E-165
23059	SYT14	60.552559	3.3039935	0.34616446	9.54457743	1.37E-21
23061	SYT15	12.0197024	-2.4836355	0.69261165	-3.5858991	0.00033592
23070	SYT8	24.9598342	-2.2500514	0.4984319	-4.5142605	6.35E-06
23072	SYTL1	282.03825	-0.5552584	0.1353134	-4.1034988	4.07E-05
23073	SYTL2	362.511312	0.93053885	0.11451428	8.12596314	4.44E-16
23077	SYVN1	2920.02359	-0.4159655	0.06198372	-6.710883	1.93E-11
23079	SZT2	1071.39954	0.4776278	0.10548348	4.52798679	5.95E-06
23088	TAB1	882.647006	-0.4072308	0.08715196	-4.6726525	2.97E-06
23096	TACC3	6330.3512	-0.5503614	0.07138953	-7.7092735	1.27E-14
23102	TADA1	557.835946	-0.5220645	0.10117464	-5.1600329	2.47E-07
23105	TADA3	4737.21204	-0.2481702	0.08011076	-3.097838	0.00194938
23109	TAF12	210.798413	1.45286749	0.14703879	9.88084469	5.04E-23
23110	TAF13	1257.91575	0.38446549	0.09198859	4.17949123	2.92E-05
23111	TAF15	6871.10864	0.40246296	0.07399117	5.43933757	5.35E-08
23112	TAF1A	819.841768	0.89099873	0.08674129	10.2719104	9.43E-25
23114	TAF1B	1429.4634	0.57511486	0.0726749	7.9135276	2.50E-15

23116	TAF1D	2037.60442	0.81991594	0.06652022	12.3258148	6.58E-35
23118	TAF2	1860.86785	0.26714281	0.08988019	2.97221022	0.00295664
23121	TAF4B	735.499787	1.30698794	0.10150448	12.87616	6.13E-38
23123	TAF5L	1388.85619	0.50533719	0.07056196	7.16160953	7.97E-13
23125	TAF6L	695.613794	-0.9665802	0.10676714	-9.0531619	1.39E-19
23137	TALDO1	7881.95087	-0.235306	0.0788731	-2.9833492	0.00285113
23138	TAMM41	363.755745	0.44741462	0.11297603	3.96026132	7.49E-05
23139	TANC1	3159.34782	-0.4025097	0.08374548	-4.806345	1.54E-06
23147	TAP1	3621.53026	0.25690118	0.06287883	4.08565475	4.40E-05
23148	TAP2	5969.64227	0.76711234	0.05763143	13.3106596	2.01E-40
23149	TAPBP	9704.92589	-0.218136	0.06382853	-3.4175318	0.00063192
23150	TAPBPL	128.808052	-0.8006116	0.18156698	-4.409456	1.04E-05
23153	TARBP1	1072.84339	-0.9013287	0.08841426	-10.194381	2.10E-24
23154	TARBP2	705.439316	-0.213087	0.0917667	-2.3220511	0.02023018
23159	TARS	27320.1774	0.57881867	0.04620131	12.528188	5.23E-36
23170	TAS2R19	14.9230847	1.49277332	0.54104858	2.7590375	0.00579719
23184	TAS2R5	48.0451019	2.31845813	0.3243954	7.14701306	8.87E-13
23193	TATDN1	432.406126	0.79610267	0.14900364	5.34284034	9.15E-08
23196	TAX1BP1	8695.85673	0.5118898	0.05289755	9.67700365	3.78E-22
23198	TAZ	1182.80675	-0.239163	0.07973446	-2.9994937	0.00270429
23200	TBC1D1	2744.90917	-0.4807554	0.0605153	-7.9443605	1.95E-15
23204	TBC1D12	1294.97192	-0.3571417	0.07142736	-5.0000689	5.73E-07
23209	TBC1D17	2151.64136	-0.6995491	0.06861979	-10.194567	2.10E-24
23211	TBC1D2	3535.23064	-0.4023296	0.05954536	-6.7566906	1.41E-11
23214	TBC1D22A	862.431596	-0.4627164	0.08287917	-5.5830247	2.36E-08
23216	TBC1D22B	2023.41666	0.82643379	0.07349625	11.2445702	2.46E-29
23217	TBC1D23	2492.63751	0.1552695	0.05966987	2.60214249	0.00926434
23223	TBC1D2B	1081.20557	-0.6592793	0.09280035	-7.1042759	1.21E-12
23225	TBC1D30	26.274528	2.19504521	0.43696862	5.02334743	5.08E-07
23240	TBC1D5	1271.76374	-0.3431711	0.09210101	-3.7260298	0.00019452
23242	TBC1D8	795.58871	-0.403807	0.08759847	-4.6097495	4.03E-06
23249	TBCCD1	218.05327	-0.4557848	0.15451661	-2.9497465	0.00318035
23250	TBCD	3539.25517	-0.43014	0.08187221	-5.2537981	1.49E-07
23252	TBCEL	1738.49002	0.33278967	0.09963645	3.34003955	0.00083766
23253	TBCK	261.414784	-0.5107822	0.15452851	-3.3054235	0.00094833
23257	TBL1XR1	3409.38735	0.25605297	0.06403842	3.9984271	6.38E-05
23259	TBL2	2653.98343	0.25939762	0.058827	4.40949934	1.04E-05
23262	TBPL1	610.99615	0.52092604	0.09628349	5.41033604	6.29E-08
23278	TBX4	9.83762769	2.27868163	0.82000764	2.77885415	0.0054551
23282	TBXA2R	127.142557	-0.9043348	0.20022815	-4.5165218	6.29E-06
23283	TBXAS1	123.359563	-0.4356413	0.19345407	-2.2519108	0.02432791
23285	TCAF1	782.627225	-0.9870148	0.09258293	-10.660872	1.55E-26
23288	TCAIM	1304.45919	0.68997232	0.07537695	9.15362472	5.51E-20

23291	TCEA1	2769.2501	0.86523393	0.05913766	14.6308439	1.79E-48
23292	TCEA2	1356.55669	-0.6458583	0.11206949	-5.7630166	8.26E-09
23294	TCEAL1	921.712486	-0.6857854	0.08438848	-8.1265285	4.42E-16
23304	TCEB1	2637.20698	0.17774967	0.0698269	2.54557589	0.01090976
23306	TCEB3	2747.45287	0.99094061	0.06269366	15.8060747	2.83E-56
23307	TCEB3-AS1	64.7117566	1.38849495	0.25605922	5.4225541	5.88E-08
23317	TCF19	3779.0528	-0.4784612	0.06121607	-7.8159405	5.46E-15
23318	TCF20	2047.70221	0.54328752	0.08043869	6.75405688	1.44E-11
23322	TCF25	2576.31338	-0.4514537	0.08296089	-5.4417647	5.28E-08
23329	TCHH	17.0245801	-1.5992666	0.50811011	-3.1474804	0.00164684
23332	TCIRG1	5699.75276	-1.2658149	0.05314854	-23.816549	2.25E-125
23337	TCN2	303.396877	-0.5717864	0.12366701	-4.6235964	3.77E-06
23338	TCOF1	5663.83077	0.5649802	0.06907683	8.17901152	2.86E-16
23345	TCP11L1	518.393415	0.44308858	0.11639745	3.80668641	0.00014084
23346	TCP11L2	581.015284	1.94408407	0.1245933	15.60344	6.90E-55
23354	TCTN1	527.268176	-0.2442953	0.10476589	-2.331821	0.01971011
23355	TCTN2	687.206413	-0.793939	0.09270709	-8.5639508	1.09E-17
23357	TDG	668.76139	0.43872898	0.09337526	4.69855674	2.62E-06
23363	TDP2	2246.56832	0.18597147	0.06623415	2.80778831	0.0049883
23375	TDRP	1080.52707	0.24387408	0.07484232	3.25850498	0.00112001
23379	TEAD4	469.17652	0.31233962	0.12391374	2.52062135	0.01171478
23381	TECPR1	909.050117	-0.8554088	0.08031601	-10.650539	1.73E-26
23383	TECR	2968.75857	-0.6666117	0.06694959	-9.95692	2.35E-23
23388	TEF	475.943648	0.71547925	0.1099467	6.5075103	7.64E-11
23397	TELO2	1417.18038	-0.2109576	0.0867591	-2.4315324	0.0150351
23405	TEPP	41.0381809	-1.0321906	0.3604362	-2.8637262	0.0041869
23407	TERF1	363.776581	-0.2667672	0.11374541	-2.345301	0.01901172
23408	TERF2	973.084855	0.8129826	0.08104919	10.0307305	1.12E-23
23409	TERF2IP	2155.83313	0.82271914	0.07511064	10.9534304	6.40E-28
23410	TERT	82.4630496	0.6196778	0.22793363	2.71867643	0.00655437
23411	TES	5597.15777	0.42580401	0.05519203	7.71495462	1.21E-14
23414	TESK1	1471.02891	0.35068529	0.07374064	4.75565831	1.98E-06
23417	TET1	68.0879232	-1.2186215	0.26010339	-4.6851425	2.80E-06
23420	TET3	1001.3495	1.31096623	0.09128444	14.3613334	9.05E-47
23421	TEX10	3496.43363	0.68895185	0.05464261	12.6083262	1.90E-36
23436	TEX264	2730.55554	-0.5521206	0.07164359	-7.7064901	1.29E-14
23446	TEX40	67.8447411	-0.8226779	0.25370002	-3.2427191	0.00118395
23458	TFAP2E	258.288146	1.48357805	0.14263165	10.4014643	2.44E-25
23461	TFB2M	1113.81812	0.39944595	0.07600729	5.25536369	1.48E-07
23465	TFDP2	1529.80499	-0.491357	0.08027983	-6.1205534	9.33E-10
23474	TFIP11	1473.19168	0.35224007	0.07579618	4.64720101	3.36E-06
23475	TFPI	4108.13635	-0.8046101	0.06497696	-12.383007	3.23E-35
23476	TFPI2	12626.8949	-0.9675159	0.04590906	-21.07462	1.36E-98

23484	TGFB1	4095.69562	-0.4018922	0.0596216	-6.7407157	1.58E-11
23485	TGFB1I1	1261.36814	-0.7610626	0.0757663	-10.04487	9.68E-24
23487	TGFB2-AS1	68.1764299	1.21176305	0.25517088	4.74882967	2.05E-06
23489	TGFB3	57.7095676	-1.1644104	0.26315994	-4.424725	9.66E-06
23490	TGFBI	8501.75881	-1.033733	0.0523291	-19.754457	7.34E-87
23496	TGIF1	3009.31089	0.63517511	0.06722268	9.44882143	3.43E-21
23497	TGIF2	1156.36388	0.71896468	0.07768051	9.25540587	2.13E-20
23502	TGM2	25336.9031	-0.9601193	0.0616837	-15.565204	1.25E-54
23508	TGOLN2	6952.43349	-0.5588554	0.06125109	-9.1240075	7.24E-20
23509	TGS1	673.492894	0.50316892	0.09462851	5.317308	1.05E-07
23513	THAP1	484.243861	0.52071553	0.11283722	4.61474958	3.94E-06
23514	THAP10	295.449733	0.53930572	0.13545317	3.98149212	6.85E-05
23519	THAP5	1255.67159	0.37255005	0.07951536	4.68525875	2.80E-06
23523	THAP8	242.639733	-0.3945231	0.15979325	-2.4689598	0.01355064
23525	THAP9-AS1	1617.40026	1.19407782	0.06964016	17.146397	6.69E-66
23526	THBD	4239.8835	-0.7220561	0.05803985	-12.440697	1.57E-35
23527	THBS1	22681.3908	0.27118914	0.0730517	3.71229065	0.00020539
23529	THBS3	529.93174	-0.5972023	0.10076487	-5.926691	3.09E-09
23538	THEM6	1344.28881	-1.5435561	0.11332271	-13.620889	3.01E-42
23542	THNSL1	533.78301	0.19288899	0.09555134	2.0186947	0.04351896
23544	THOC1	2028.33117	0.3257712	0.06323937	5.1513988	2.59E-07
23546	THOC3	487.008847	-0.6569347	0.11698083	-5.6157464	1.96E-08
23551	THOP1	3565.25738	-0.6118832	0.07874423	-7.7705156	7.82E-15
23553	THRA	679.470526	-0.8582096	0.10419096	-8.2368915	1.77E-16
23555	THRAP3	6491.24265	0.31943676	0.07922406	4.0320675	5.53E-05
23560	THSD1	66.1161523	3.4486785	0.32748391	10.5308334	6.23E-26
23567	THUMPD1	1352.22861	0.247424	0.07243462	3.41582543	0.00063589
23573	TIA1	1101.77402	-0.3941471	0.07799017	-5.0538042	4.33E-07
23576	TIAM1	449.947947	0.68320313	0.11372522	6.0074898	1.88E-09
23578	TICAM1	1381.16418	0.42933486	0.07345256	5.84506359	5.06E-09
23580	TICRR	591.138622	-0.3816655	0.11315586	-3.372919	0.00074376
23584	TIGAR	557.472735	0.49068926	0.09399954	5.22012416	1.79E-07
23586	TIGD2	485.105064	0.30736618	0.09714959	3.16384451	0.001557
23589	TIGD5	1049.81472	0.23054891	0.07624414	3.02382465	0.00249601
23594	TIMELESS	2152.7185	-0.7109664	0.06628937	-10.725196	7.75E-27
23595	TIMM10	1285.34774	0.32150645	0.11401882	2.81976647	0.00480586
23596	TIMM10B	480.657051	-0.2063588	0.1025334	-2.0126008	0.04415664
23598	TIMM17A	4064.39388	0.22503236	0.06338874	3.55003702	0.00038518
23601	TIMM22	1905.7726	0.62727493	0.06910005	9.0777783	1.11E-19
23604	TIMM44	2921.23998	0.78810131	0.07874027	10.0088725	1.39E-23
23606	TIMM8A	609.405362	0.79378533	0.104185	7.61899823	2.56E-14
23607	TIMM8B	1431.25429	0.23591558	0.09196606	2.56524609	0.01031027
23608	TIMM9	1985.93144	1.09772898	0.06986447	15.7122627	1.25E-55

23610	TIMP1	8106.56979	-0.3289434	0.08294724	-3.9656943	7.32E-05
23611	TIMP2	34799.8878	-0.4961625	0.04472151	-11.094494	1.33E-28
23615	TINAGL1	8486.60085	0.59797186	0.05001575	11.9556709	6.06E-33
23618	TIPARP	3277.13953	1.22136447	0.06841789	17.8515359	2.81E-71
23620	TIPIN	615.918376	0.44306794	0.10246795	4.32396618	1.53E-05
23622	TIRAP	278.797492	-0.3579435	0.15673464	-2.2837548	0.02238595
23624	TJAP1	2033.36495	0.61463437	0.06193132	9.9244506	3.26E-23
23627	TJP3	115.008202	-3.4159882	0.24842309	-13.750687	5.04E-43
23630	TKFC	418.81069	-0.4121507	0.10655126	-3.8680978	0.00010969
23638	TLE1	3961.61697	0.40678955	0.05707702	7.12702844	1.03E-12
23644	TLK2	1226.24287	0.54050271	0.07569585	7.14045336	9.30E-13
23649	TLR1	41.7256193	-0.9291581	0.3222897	-2.8829903	0.0039392
23652	TLR3	141.474886	-1.7007629	0.19499407	-8.7221258	2.73E-18
23658	TLR8-AS1	456.475412	0.52828362	0.10648234	4.96123244	7.00E-07
23667	TM4SF1	3783.54093	-0.2223975	0.05902184	-3.768054	0.00016453
23678	TM7SF2	450.123397	-1.1322542	0.12337448	-9.1773783	4.42E-20
23679	TM7SF3	1773.64833	-0.7551608	0.06671089	-11.319902	1.05E-29
23680	TM9SF1	3089.86861	-0.6211564	0.05892602	-10.541292	5.57E-26
23682	TM9SF3	11084.7785	-0.2524208	0.05221133	-4.8345981	1.33E-06
23683	TM9SF4	4816.25371	-0.3215744	0.06010758	-5.3499798	8.80E-08
23684	TMA16	1257.45308	0.4646812	0.07672115	6.05675494	1.39E-09
23687	TMBIM4	1281.56232	-0.5587868	0.07804665	-7.1596519	8.09E-13
23694	TMC5	450.373029	-0.6597273	0.11180833	-5.9005203	3.62E-09
23695	TMC6	2216.03594	-0.5752212	0.06651411	-8.6481088	5.24E-18
23697	TMC8	385.721211	-1.0639534	0.12790394	-8.3183784	8.92E-17
23699	TMCC1-AS1	138.607742	1.6221564	0.18642983	8.70116315	3.28E-18
23700	TMCC2	494.852297	-0.6534559	0.09984573	-6.5446554	5.96E-11
23708	TMCO6	422.14201	-0.2193022	0.10448541	-2.0988789	0.03582758
23709	TMED1	1643.46554	-0.2713735	0.1060169	-2.559719	0.01047568
23710	TMED10	6402.81703	-0.7059403	0.05747536	-12.282485	1.12E-34
23714	TMED3	2287.08664	-0.5007207	0.06935001	-7.2201964	5.19E-13
23725	TMEM101	1325.6746	-1.0572949	0.11968633	-8.8338817	1.01E-18
23727	TMEM104	1486.84527	-0.505353	0.08322288	-6.072285	1.26E-09
23731	TMEM106C	2718.66767	-0.4388289	0.05999359	-7.3145959	2.58E-13
23735	TMEM109	4762.45344	-0.4807215	0.05298312	-9.0731078	1.16E-19
23737	TMEM110	803.073953	-0.8684951	0.08325976	-10.431151	1.79E-25
23740	TMEM115	2400.1513	-0.4929498	0.0677653	-7.2743695	3.48E-13
23741	TMEM116	871.325573	0.34327791	0.08057585	4.26030789	2.04E-05
23742	TMEM117	26.7207225	-1.1576913	0.43689868	-2.6497935	0.0080541
23744	TMEM120A	904.140128	-0.4516735	0.09926037	-4.550391	5.35E-06
23751	TMEM127	3186.76593	-0.376522	0.06195553	-6.0772939	1.22E-09
23753	TMEM129	1845.22804	-0.7351653	0.06807244	-10.799749	3.45E-27
23754	TMEM130	17.9813052	-2.8172996	0.57174465	-4.9275488	8.33E-07

23761	TMEM133	402.326375	-0.3799441	0.13227426	-2.8723966	0.00407371
23762	TMEM134	597.700904	-0.8173254	0.11102766	-7.361457	1.82E-13
23763	TMEM135	624.637319	-1.026225	0.10024604	-10.237063	1.35E-24
23764	TMEM136	1149.16215	0.61385181	0.07795559	7.87437878	3.42E-15
23766	TMEM139	105.489725	-0.7301869	0.20719976	-3.524072	0.00042497
23769	TMEM143	221.122335	-1.3226375	0.1459994	-9.0591981	1.31E-19
23772	TMEM147	3729.35537	0.25347273	0.07978908	3.1767845	0.00148918
23774	TMEM14A	935.30754	-0.7488671	0.10730696	-6.9787372	2.98E-12
23789	TMEM161A	1173.14403	-0.4180752	0.10210594	-4.0945237	4.23E-05
23790	TMEM161B	1239.54768	0.19557133	0.07961487	2.45646738	0.01403105
23793	TMEM164	2160.85438	-0.2259049	0.06412529	-3.522867	0.00042691
23794	TMEM165	4648.85049	-0.6152474	0.06287806	-9.7847714	1.31E-22
23796	TMEM167B	1845.506	0.4195515	0.07115153	5.8965914	3.71E-09
23798	TMEM169	12.6707339	-1.2108116	0.60287301	-2.0084024	0.04460055
23800	TMEM170A	1015.50129	0.41333573	0.07593966	5.44294935	5.24E-08
23803	TMEM173	1720.0195	-1.3563779	0.07560336	-17.94071	5.67E-72
23805	TMEM175	620.937062	-0.5619426	0.09946067	-5.6498974	1.61E-08
23812	TMEM179B	1401.34635	-0.4527664	0.10068608	-4.4968125	6.90E-06
23813	TMEM18	930.303568	-0.2662175	0.11127906	-2.392341	0.01674128
23815	TMEM181	1672.59693	-0.5633349	0.07008441	-8.0379497	9.14E-16
23825	TMEM187	372.684253	-0.6340741	0.12033621	-5.2691881	1.37E-07
23826	TMEM189	1051.00181	-0.5944269	0.08592257	-6.9181692	4.58E-12
23828	TMEM19	1280.02511	-0.6569047	0.07850616	-8.3675564	5.88E-17
23830	TMEM191A	7.21818038	-2.1550297	0.79995782	-2.6939291	0.00706152
23833	TMEM192	1970.61492	0.66501469	0.07047954	9.43557133	3.89E-21
23836	TMEM198B	240.141387	-0.4560086	0.14581453	-3.1273194	0.00176408
23842	TMEM201	1040.5384	0.45908922	0.07532211	6.09501246	1.09E-09
23847	TMEM206	1069.56303	0.56241174	0.08126125	6.92103273	4.48E-12
23850	TMEM209	1322.39968	0.55572906	0.07573644	7.33767069	2.17E-13
23856	TMEM214	2511.21172	-0.4173412	0.07303684	-5.7141191	1.10E-08
23860	TMEM218	569.723091	-0.3619509	0.11068184	-3.2701923	0.00107474
23864	TMEM221	28.0431517	-1.5218968	0.3978556	-3.8252491	0.00013064
23865	TMEM222	1211.95858	0.48225902	0.08360835	5.76807254	8.02E-09
23866	TMEM223	463.24711	-0.6186011	0.12779703	-4.8404966	1.30E-06
23869	TMEM229B	227.550034	-0.7687303	0.13767658	-5.5835955	2.36E-08
23870	TMEM230	3263.07765	-0.1629991	0.06798403	-2.3976088	0.01650248
23871	TMEM231	359.398168	0.59363152	0.13803252	4.30066434	1.70E-05
23876	TMEM236	67.929883	1.95692742	0.27784801	7.04315791	1.88E-12
23877	TMEM237	1327.10238	-0.596883	0.07016997	-8.5062457	1.80E-17
23882	TMEM242	213.986683	-0.5696062	0.1436739	-3.9645768	7.35E-05
23885	TMEM245	5994.87948	-1.0869336	0.0629326	-17.271391	7.73E-67
23889	TMEM248	5268.31034	-0.726977	0.05786727	-12.562835	3.38E-36
23891	TMEM25	980.732575	-0.5909577	0.08072907	-7.320259	2.47E-13

23898	TMEM255B	364.633838	-0.2631657	0.10863117	-2.4225616	0.01541151
23902	TMEM258	1399.26866	-0.1926275	0.08261688	-2.3315761	0.019723
23907	TMEM261	360.148861	0.34389118	0.13604122	2.52784554	0.01147648
23911	TMEM27	42.6907357	1.5891824	0.32630719	4.8702035	1.11E-06
23918	TMEM37	70.9645882	-1.6971701	0.2690385	-6.3082798	2.82E-10
23920	TMEM38B	1728.79571	0.43625004	0.06484484	6.72759867	1.72E-11
23928	TMEM44	898.26222	-0.4946274	0.09311802	-5.3118333	1.09E-07
23931	TMEM45B	263.84571	0.86314214	0.13703003	6.2989269	3.00E-10
23933	TMEM5	522.379233	0.53104858	0.09814572	5.4108174	6.27E-08
23935	TMEM50A	4809.16836	0.30640426	0.05544032	5.52674057	3.26E-08
23936	TMEM50B	823.190713	-0.2717448	0.07910844	-3.4350922	0.00059235
23937	TMEM51	1166.49185	0.3327534	0.08355031	3.98267126	6.81E-05
23941	TMEM53	152.257143	-0.7901998	0.16542977	-4.776648	1.78E-06
23943	TMEM55A	1161.77413	-0.6986641	0.0901944	-7.7462028	9.47E-15
23944	TMEM55B	1893.72066	0.32990497	0.06574097	5.0182555	5.21E-07
23945	TMEM56	1145.81738	-1.2394667	0.09078493	-13.65278	1.94E-42
23947	TMEM57	943.837786	0.69894511	0.09165912	7.62548391	2.43E-14
23950	TMEM60	975.029208	-0.2800487	0.09336747	-2.9994246	0.0027049
23951	TMEM61	9.54255427	-1.6621284	0.71052966	-2.3392809	0.0193209
23952	TMEM62	769.398589	-0.1990067	0.08216116	-2.4221512	0.01542893
23956	TMEM64	2186.3164	0.20994378	0.07587921	2.76681549	0.00566068
23960	TMEM69	2056.68547	0.87789924	0.06869103	12.7804055	2.11E-37
23968	TMEM79	218.300865	1.08371469	0.15947348	6.79557958	1.08E-11
23969	TMEM80	565.508832	-0.3915112	0.1059738	-3.6944148	0.00022039
23980	TMEM8B	299.858012	-1.0504609	0.14364455	-7.3129184	2.61E-13
23983	TMEM91	109.878659	-0.8821036	0.24623705	-3.5823349	0.00034054
23984	TMEM92	756.787937	-0.4352277	0.09291523	-4.6841378	2.81E-06
23987	TMEM97	1375.43248	-1.009167	0.08889454	-11.352406	7.21E-30
23991	TMEM9B-AS	35.2290115	0.93611294	0.36046593	2.59695263	0.00940549
23993	TMIE	67.3261226	1.61556928	0.26366266	6.12741032	8.93E-10
23997	TMLHE	2.94877243	5.00106421	1.63853393	3.05215785	0.00227203
24000	TMOD2	215.756541	-0.5825222	0.16248133	-3.5851643	0.00033687
24001	TMOD3	4582.46019	0.21331734	0.06737941	3.16591268	0.00154597
24004	TMPO-AS1	184.403314	-0.8506673	0.16347708	-5.2035879	1.95E-07
24023	TMPRSS9	28.4007841	-1.7627442	0.40827325	-4.3175599	1.58E-05
24027	TMSB4X	25096.5393	-1.0821714	0.08709803	-12.424751	1.92E-35
24032	TMTC4	509.549706	-0.5913031	0.10375871	-5.6988288	1.21E-08
24034	TMUB2	1795.80404	-0.2097265	0.07134767	-2.9395009	0.00328741
24036	TMX2	3043.80718	0.14919956	0.06847783	2.178801	0.02934645
24038	TMX3	2592.52522	0.30436884	0.0610426	4.98617085	6.16E-07
24039	TMX4	1210.83077	-0.7260743	0.07195719	-10.090366	6.09E-24
24041	TNF	170.285601	3.68988511	0.21627285	17.06125	2.88E-65
24042	TNFAIP1	2717.19459	-0.1359094	0.06623774	-2.0518428	0.04018494

24043	TNFAIP2	11623.2732	-1.4330265	0.04985934	-28.741386	1.16E-181
24046	TNFAIP8	619.326101	-0.471102	0.10156666	-4.6383529	3.51E-06
24047	TNFAIP8L1	811.334464	-1.2479595	0.08276986	-15.077462	2.28E-51
24050	TNFAIP8L3	36.7822561	-1.6317513	0.35004519	-4.6615446	3.14E-06
24051	TNFRSF10A	1083.38472	1.14687449	0.08575463	13.3739079	8.59E-41
24052	TNFRSF10B	6641.82471	0.50560051	0.05684732	8.89400827	5.89E-19
24055	TNFRSF11A	263.294651	-0.5210285	0.14355057	-3.6295811	0.00028388
24057	TNFRSF12A	5725.68378	0.69735111	0.05996661	11.6289896	2.94E-31
24060	TNFRSF14	621.647948	-0.3836994	0.11196187	-3.4270542	0.00061017
24062	TNFRSF18	18.4397912	-1.2537268	0.48941133	-2.5617037	0.01041601
24063	TNFRSF19	212.266696	-0.4283292	0.14586869	-2.9364029	0.00332043
24064	TNFRSF1A	3363.04654	-0.7935526	0.05721365	-13.869986	9.63E-44
24066	TNFRSF21	6593.2376	-0.1921738	0.05123003	-3.7511953	0.00017599
24071	TNFRSF9	487.064887	0.94427115	0.11498779	8.21192505	2.18E-16
24074	TNFSF12	486.202443	-0.7032252	0.12295517	-5.719363	1.07E-08
24077	TNFSF13B	8.90252546	-1.4238519	0.69452295	-2.0501149	0.04035322
24087	TNIP3	180.352634	-0.3212609	0.15732803	-2.0419816	0.04115336
24090	TNKS	1058.31884	0.33010203	0.08961746	3.68345674	0.00023009
24102	TNNT1	3631.54012	-0.4990103	0.09984915	-4.9976425	5.80E-07
24107	TNPO1	8614.88424	0.22163856	0.07994558	2.77236808	0.00556501
24109	TNPO3	3756.35085	0.3448	0.07006881	4.92087723	8.62E-07
24114	TNRC6B	941.762585	-0.3267775	0.11714189	-2.7895875	0.00527752
24115	TNRC6C	676.935379	0.26065512	0.08870565	2.93842744	0.00329882
24117	TNS1	167.661113	-1.8751758	0.18447147	-10.165126	2.84E-24
24118	TNS2	962.420947	-1.3352647	0.10226546	-13.056849	5.81E-39
24119	TNS3	6012.83057	-1.2519969	0.06723792	-18.6204	2.20E-77
24120	TNS4	247.112446	-0.7635605	0.14670984	-5.2045624	1.94E-07
24125	TOB2	1216.30834	0.50943094	0.08650754	5.88886187	3.89E-09
24127	TOE1	705.497962	0.60400425	0.08768908	6.88802162	5.66E-12
24128	TOLLIP	4581.38238	0.31593629	0.0701992	4.5005683	6.78E-06
24131	TOM1L1	1634.24062	1.05139327	0.06698823	15.6951951	1.63E-55
24136	TOMM34	5484.88531	1.10984242	0.0537977	20.629924	1.48E-94
24139	TOMM5	3495.60579	0.29432304	0.09631741	3.05576149	0.0022449
24140	TOMM6	7374.76681	0.29741488	0.06453057	4.60889866	4.05E-06
24142	TOMM70A	4539.87952	0.19936288	0.05582859	3.57098196	0.00035565
24143	TONSL	2417.80403	-0.5448985	0.06123694	-8.8982002	5.68E-19
24145	TOP1	8547.99296	0.67489051	0.05401203	12.4951897	7.93E-36
24149	TOP2A	16546.1284	-0.3678565	0.04964068	-7.4103843	1.26E-13
24150	TOP2B	4283.13643	-0.6225118	0.05301086	-11.743098	7.66E-32
24151	TOP3A	1733.41212	0.54609763	0.07639725	7.14813199	8.80E-13
24155	TOPORS	1485.28983	0.24133494	0.09116572	2.64721158	0.00811586
24158	TOR1AIP1	2259.05944	-0.483828	0.07334042	-6.5970165	4.20E-11
24164	TOX	239.379048	1.23276571	0.15428352	7.99026178	1.35E-15

24167	TOX4	2448.00582	0.31403005	0.06104323	5.14438824	2.68E-07
24168	TP53	3242.02443	-0.6353714	0.05682695	-11.180811	5.06E-29
24169	TP53AIP1	32.817435	2.35321497	0.41859661	5.62167704	1.89E-08
24171	TP53BP2	3172.66352	0.6305109	0.06904941	9.13130007	6.77E-20
24173	TP53I13	1175.63009	-0.7365973	0.09886123	-7.4508206	9.28E-14
24177	TP53RK	1511.6536	0.59065843	0.07515605	7.85909313	3.87E-15
24184	TP63	32.2647937	-1.1160444	0.39363334	-2.8352385	0.00457915
24186	TP73-AS1	995.376588	-0.6143526	0.07786804	-7.8896626	3.03E-15
24187	TPBG	3623.66367	0.2927821	0.06503506	4.50191179	6.73E-06
24189	TPCN1	3811.66368	-0.3534754	0.06497277	-5.440362	5.32E-08
24192	TPD52L1	753.260475	-0.5233893	0.09418574	-5.5569907	2.74E-08
24193	TPD52L2	11471.4398	0.54326108	0.04829152	11.249617	2.33E-29
24195	TPGS1	580.82528	-1.5395154	0.12934436	-11.902454	1.15E-32
24202	TPK1	303.727408	-0.2622292	0.12097461	-2.1676384	0.03018621
24203	TPM1	6105.18486	-0.2846059	0.08416662	-3.3814579	0.00072102
24204	TPM2	2429.45578	-0.638472	0.13290097	-4.8041184	1.55E-06
24211	TPP2	1825.53834	0.27445538	0.08152376	3.36656913	0.0007611
24220	TPRG1L	1717.57952	0.15062033	0.07536204	1.99862333	0.04564912
24222	TPRN	736.323153	-0.3323893	0.10522959	-3.1587052	0.00158472
24229	TPST1	1027.82624	-0.6449877	0.07838313	-8.2286537	1.89E-16
24241	TRA2A	2204.58555	-0.1732455	0.07616083	-2.2747325	0.02292198
24242	TRA2B	4470.68849	-0.4749887	0.0527752	-9.0002252	2.25E-19
24244	TRABD2A	186.265915	0.58073255	0.15373597	3.77746689	0.00015843
24246	TRADD	494.216672	-1.0007754	0.13782949	-7.2609672	3.84E-13
24247	TRAF1	4833.82868	2.35047577	0.05569764	42.2006321	0
24248	TRAF2	2680.32009	0.3637656	0.10231614	3.55531004	0.00037753
24254	TRAF4	2574.45741	0.42684719	0.07348174	5.80888783	6.29E-09
24256	TRAF6	658.665231	0.23950649	0.09010507	2.65808012	0.00785872
24258	TRAFD1	1199.20655	-0.656136	0.07380747	-8.8898321	6.12E-19
24260	TRAK1	3359.1342	-0.2384442	0.06738233	-3.538675	0.00040214
24261	TRAK2	2836.9836	-0.1929236	0.08396437	-2.2976838	0.02157979
24264	TRAM2	11351.1396	0.2481139	0.05656402	4.38642645	1.15E-05
24265	TRAM2-AS1	501.763977	0.71869911	0.09907593	7.25402304	4.05E-13
24266	TRANK1	607.86515	-0.6353431	0.1090089	-5.8283604	5.60E-09
24268	TRAPPC1	3323.91994	-0.3335558	0.07131676	-4.677103	2.91E-06
24269	TRAPPC10	1040.38915	0.33429177	0.11373965	2.93909621	0.00329171
24271	TRAPPC12	701.204776	-0.8111307	0.08764349	-9.2548875	2.14E-20
24274	TRAPPC2B	250.929419	-0.7329042	0.13098676	-5.5952543	2.20E-08
24276	TRAPPC3	2663.99598	0.5831099	0.07384959	7.89591242	2.88E-15
24279	TRAPPC5	1044.56125	-0.3525131	0.09926822	-3.5511178	0.0003836
24282	TRAPPC8	2717.26109	0.2630065	0.07596582	3.46216914	0.00053584
24283	TRAPPC9	508.158122	-0.8344539	0.10981783	-7.5985282	3.00E-14
24291	TREML2	9.62546731	5.73282226	1.32148419	4.3381694	1.44E-05

24297	TREX1	364.341353	-0.6150977	0.11974929	-5.1365458	2.80E-07
24304	TRIAP1	1341.41734	0.49050069	0.10771102	4.55385807	5.27E-06
24305	TRIB1	457.529589	0.28966951	0.10664395	2.71623001	0.006603
24306	TRIB2	2539.81643	-1.4031761	0.06240887	-22.483598	6.01E-112
24307	TRIB3	3155.30385	1.89555844	0.06981146	27.1525414	2.36E-162
24310	TRIM11	1613.80454	0.5118868	0.08484428	6.03325088	1.61E-09
24312	TRIM14	1129.26317	-0.3311905	0.09755469	-3.394921	0.00068648
24319	TRIM22	109.429562	-1.5988414	0.20952536	-7.6307771	2.33E-14
24321	TRIM24	2774.7057	0.30379745	0.0702496	4.32454333	1.53E-05
24325	TRIM28	13590.4951	-0.4695044	0.06502754	-7.2200865	5.20E-13
24331	TRIM33	3439.36886	0.17743545	0.07631073	2.32517042	0.02006285
24333	TRIM35	874.873294	0.3047331	0.08476604	3.59499026	0.0003244
24334	TRIM36	453.798741	1.15242055	0.12195102	9.44986418	3.39E-21
24337	TRIM39	1013.64215	0.89660233	0.0857736	10.4531272	1.42E-25
24348	TRIM47	1648.81419	-0.4332099	0.06418977	-6.7488937	1.49E-11
24359	TRIM52	125.19711	-0.3928482	0.19176796	-2.04856	0.04050516
24387	TRIM8	10639.2756	-0.2863283	0.05175698	-5.5321681	3.16E-08
24391	TRIO	14071.3765	0.500082	0.09140133	5.4712771	4.47E-08
24392	TRIOBP	3164.58838	-0.4131787	0.0590796	-6.9935934	2.68E-12
24395	TRIP12	6705.08602	0.1504562	0.06025782	2.49687433	0.01252934
24399	TRIQK	886.107412	-0.6417641	0.08560694	-7.4966365	6.55E-14
24400	TRIT1	909.657708	0.75985088	0.08575535	8.86068174	7.95E-19
24401	TRMT1	2254.87995	0.91441378	0.06685557	13.6774507	1.38E-42
24402	TRMT10A	297.690178	0.50136466	0.12984852	3.86115034	0.00011285
24405	TRMT11	911.394513	0.22925863	0.08284766	2.76723131	0.00565346
24408	TRMT13	593.02816	0.80383313	0.09559448	8.40878212	4.14E-17
24410	TRMT2A	1317.72708	-0.2744075	0.07450942	-3.6828563	0.00023064
24411	TRMT2B	813.126101	-0.4842237	0.08220034	-5.8907758	3.84E-09
24412	TRMT44	384.121827	0.23587379	0.1097833	2.14853986	0.03167089
24414	TRMT6	1764.80358	0.57951951	0.06968183	8.31665217	9.05E-17
24415	TRMT61A	1239.35423	0.84072202	0.0895814	9.385006	6.29E-21
24418	TRNAU1AP	822.655257	0.82168523	0.0864587	9.50378932	2.02E-21
24419	TRNP1	170.891813	1.88836025	0.17177418	10.9932719	4.12E-28
24420	TRNT1	719.076023	0.50537494	0.08951627	5.64562134	1.65E-08
24422	TROAP	1141.45345	-0.3015625	0.07578586	-3.9791395	6.92E-05
24423	TROVE2	1529.52397	0.61521909	0.07134297	8.62340171	6.50E-18
24438	TRPM4	241.292336	-0.5415155	0.13787861	-3.9274802	8.58E-05
24444	TRPT1	850.453823	-0.4758524	0.11417621	-4.1677021	3.08E-05
24445	TRPV1	318.604396	-0.2744841	0.12095714	-2.269267	0.0232521
24453	TRUB2	1382.09212	-0.4158243	0.07983181	-5.2087548	1.90E-07
24455	TSACC	59.8843169	1.61676512	0.26982625	5.99187486	2.07E-09
24457	TSC2	2212.71756	-0.3969534	0.06434022	-6.1696003	6.85E-10
24458	TSC22D1	7904.3609	1.34987378	0.05796664	23.2870814	5.99E-120

24460	TSC22D2	1356.87456	0.7411437	0.07777707	9.52907766	1.59E-21
24461	TSC22D3	656.747334	0.25515021	0.10077456	2.53189109	0.01134492
24462	TSC22D4	4846.89931	-1.2018214	0.05952202	-20.191204	1.17E-90
24463	TSEN15	2930.27633	0.77442898	0.06520609	11.8766356	1.57E-32
24469	TSG101	3097.12282	-0.2749835	0.06173321	-4.4543857	8.41E-06
24470	TSGA10	178.333317	0.36304501	0.15353156	2.36462794	0.01804819
24475	TSHZ1	244.762589	-0.7353988	0.13556574	-5.4246658	5.81E-08
24480	TSKU	1258.59779	-0.5124522	0.07144241	-7.1729411	7.34E-13
24488	TSPAN1	69.2374617	-0.7787866	0.26368931	-2.9534252	0.00314269
24494	TSPAN15	1089.54105	-1.0244652	0.07981132	-12.83609	1.03E-37
24499	TSPAN2	450.605079	-0.2606306	0.10328499	-2.5234119	0.01162222
24509	TSPAN9	1662.53834	-0.8112713	0.07026283	-11.546236	7.71E-31
24513	TSPO	4603.28685	-0.3055038	0.09456962	-3.2304643	0.00123589
24522	TSPYL1	2259.04579	-0.4301509	0.06157089	-6.9862712	2.82E-12
24527	TSR1	5951.2471	0.20359799	0.05061021	4.0228641	5.75E-05
24528	TSR2	2203.98984	-0.5942445	0.09080784	-6.5439776	5.99E-11
24532	TSSC4	1846.67296	0.54879993	0.09464591	5.79845373	6.69E-09
24537	TSSK6	115.283079	1.12229693	0.21079397	5.32414144	1.01E-07
24538	TST	808.065399	-0.4232317	0.11645796	-3.6342013	0.00027884
24540	TSTD1	244.105997	-0.552804	0.17177839	-3.2181231	0.00129032
24541	TSTD2	1288.05229	0.60952297	0.08505532	7.16619448	7.71E-13
24545	TTC1	3058.28843	-0.418336	0.05698117	-7.3416544	2.11E-13
24546	TTC12	1185.55193	-0.4188998	0.07460136	-5.6151765	1.96E-08
24547	TTC13	1442.29292	-0.3212204	0.07359723	-4.364571	1.27E-05
24566	TTC30A	271.051089	-0.8643514	0.14446779	-5.9830042	2.19E-09
24567	TTC30B	111.105583	-0.9780472	0.21346399	-4.5817902	4.61E-06
24568	TTC31	652.692108	-0.2254709	0.09553914	-2.3599846	0.01827569
24570	TTC33	1113.53731	0.16782288	0.07755436	2.16393876	0.03046905
24574	TTC38	1859.84583	-0.1534084	0.06342638	-2.4186841	0.01557676
24577	TTC39B	110.914835	0.61233854	0.20356806	3.00802861	0.00262948
24583	TTC5	241.751743	-0.6436036	0.14817061	-4.3436654	1.40E-05
24585	TTC7A	1718.98902	-0.699083	0.07204125	-9.7039268	2.90E-22
24588	TTC9	177.352978	-0.7533566	0.16875494	-4.4642046	8.04E-06
24592	TTF2	1194.15942	0.36973671	0.08367548	4.41869833	9.93E-06
24597	TTLL1	181.597845	-0.3931258	0.17906048	-2.195492	0.02812832
24599	TTLL11	184.966793	0.78603367	0.15080387	5.2122912	1.87E-07
24652	TTYH3	4350.27171	-0.45366	0.06096543	-7.4412663	9.97E-14
24653	TUB	211.976964	-1.0605221	0.15956875	-6.6461768	3.01E-11
24655	TUBA1A	2039.09756	-0.252518	0.07253701	-3.4812306	0.00049912
24657	TUBA1C	16579.9765	0.19042577	0.06126211	3.10837781	0.00188117
24670	TUBB3	1508.92684	-0.7984486	0.08766207	-9.1082554	8.37E-20
24672	TUBB4B	21293.6386	-0.4855176	0.05839159	-8.3148557	9.19E-17
24673	TUBB6	10242.3436	-0.2308679	0.06807788	-3.3912323	0.00069579

24677	TUBE1	666.691801	1.73331026	0.10684055	16.2233371	3.45E-59
24680	TUBGCP2	4963.85038	-0.3612588	0.0566436	-6.3777512	1.80E-10
24681	TUBGCP3	1179.55945	-0.8289669	0.08703123	-9.5249362	1.65E-21
24683	TUBGCP5	597.681635	-0.5917129	0.10234398	-5.7816098	7.40E-09
24684	TUBGCP6	955.362168	-0.8172604	0.1021526	-8.0003883	1.24E-15
24686	TUFT1	2010.54125	0.30611111	0.07034242	4.35172856	1.35E-05
24690	TULP3	813.941516	1.05590672	0.09993489	10.565947	4.29E-26
24691	TULP4	797.089842	0.37811995	0.10853441	3.48387147	0.00049422
24693	TUSC1	422.002525	-0.9372004	0.12503013	-7.4957966	6.59E-14
24701	TVP23B	760.063473	0.37359902	0.09643836	3.87396706	0.00010708
24708	TWISTNB	2005.55687	0.47752263	0.06620347	7.2129552	5.48E-13
24709	TWSG1	6130.52133	-0.3648486	0.06460098	-5.6477259	1.63E-08
24711	TXLNA	4104.97628	0.577286	0.05839211	9.88637063	4.77E-23
24713	TXLNG	2522.31602	0.67557222	0.06826889	9.89575507	4.34E-23
24716	TXN2	1905.31339	-0.2223416	0.08488703	-2.6192651	0.00881195
24718	TXNDC12	1889.41954	0.23336699	0.06985646	3.34066461	0.00083578
24720	TXNDC15	1341.34636	-0.9440718	0.07685479	-12.283839	1.11E-34
24726	TXNDC9	2538.61378	0.43860303	0.06365189	6.89065239	5.55E-12
24727	TXNIP	13054.2497	1.61352907	0.04706997	34.2793705	1.59E-257
24730	TXNL4B	573.051368	1.47912265	0.09872464	14.982305	9.58E-51
24733	TXNRD3	312.70899	-0.6907167	0.12121079	-5.6984752	1.21E-08
24735	TYK2	2843.05023	-0.3133883	0.06870357	-4.5614564	5.08E-06
24737	TYMS	9775.06939	-0.2429655	0.05630581	-4.3151062	1.60E-05
24744	TYSND1	1429.0773	-0.2148784	0.08740095	-2.4585358	0.01395049
24747	TYW3	1044.28395	0.38253337	0.08066063	4.74250392	2.11E-06
24748	TYW5	654.016435	0.70138976	0.08907518	7.87413284	3.43E-15
24752	U2SURP	3763.38815	0.41675358	0.06230792	6.68861293	2.25E-11
24753	UACA	6850.70197	-0.6803231	0.07037301	-9.6673862	4.15E-22
24754	UAP1	7087.6143	0.69382338	0.0538559	12.8829583	5.61E-38
24760	UBA52	9987.15613	-0.2094135	0.08344337	-2.5096486	0.01208514
24761	UBA6	4708.9394	0.12802366	0.06469082	1.97900817	0.04781509
24762	UBA6-AS1	551.096306	1.62535747	0.1178883	13.7872669	3.04E-43
24763	UBA7	714.156171	-1.6274963	0.10557018	-15.416251	1.27E-53
24764	UBAC1	1561.44687	-0.6870258	0.09165333	-7.4959174	6.58E-14
24769	UBAP1	3958.66159	0.9343897	0.06156532	15.1772081	5.01E-52
24776	UBC	24714.3265	0.34089178	0.05960787	5.71890563	1.07E-08
24779	UBE2B	3878.74171	0.35943847	0.05968181	6.02257972	1.72E-09
24782	UBE2D2	6280.7817	0.41062288	0.05228842	7.85303676	4.06E-15
24786	UBE2E1	6270.90418	0.58596701	0.06867003	8.53308175	1.42E-17
24789	UBE2E3	1946.40257	-0.2684411	0.06444657	-4.1653278	3.11E-05
24791	UBE2F	1097.57469	0.36630125	0.08747906	4.18730225	2.82E-05
24793	UBE2G1	2099.63396	0.22928741	0.06966901	3.29109589	0.00099798
24794	UBE2G2	2895.86548	0.24980802	0.07231655	3.45436852	0.00055158

24795	UBE2H	12389.517	0.1238168	0.04940925	2.50594376	0.0122125
24798	UBE2J2	2828.10524	0.82641521	0.07734076	10.6853776	1.19E-26
24800	UBE2L3	3886.65354	0.13247694	0.0609621	2.17310351	0.02977253
24802	UBE2M	4446.09217	0.23114137	0.05893538	3.92194587	8.78E-05
24807	UBE2Q1	3860.02085	0.30579482	0.05619531	5.44164278	5.28E-08
24811	UBE2Q2P1	48.5620494	-1.1783185	0.28849161	-4.0844117	4.42E-05
24816	UBE2T	1475.79427	-0.4324222	0.06703281	-6.4509039	1.11E-10
24819	UBE2V2	1937.66051	0.13168429	0.06329299	2.08055094	0.03747503
24820	UBE2W	1186.1919	0.3327132	0.07492862	4.4404023	8.98E-06
24821	UBE2Z	8148.40711	0.39367023	0.05719226	6.88327798	5.85E-12
24829	UBIAD1	1261.01633	1.11913949	0.08501902	13.1634015	1.43E-39
24839	UBOX5	514.091646	-0.3978579	0.09667023	-4.1156195	3.86E-05
24841	UBP1	3866.36056	0.30458085	0.05889503	5.17158842	2.32E-07
24842	UBQLN1	8789.06479	0.27745626	0.05713359	4.85627179	1.20E-06
24843	UBQLN2	1777.77837	-0.2644695	0.07516524	-3.5185076	0.00043398
24848	UBR2	3406.32811	0.34759832	0.07653305	4.54180661	5.58E-06
24850	UBR4	12124.8992	0.77858489	0.06293111	12.3720194	3.70E-35
24851	UBR5	5225.47109	0.30157321	0.0648154	4.65280212	3.27E-06
24854	UBTD1	1634.01537	-0.2650079	0.0926956	-2.8589052	0.00425106
24868	UCA1	19000.9365	0.8515354	0.10136364	8.40079709	4.43E-17
24871	UCHL3	1975.27711	0.76551363	0.0742267	10.3131849	6.14E-25
24872	UCHL5	2932.04763	0.28874346	0.06634719	4.35200758	1.35E-05
24874	UCK2	2961.33794	0.17468503	0.0644075	2.71218486	0.00668413
24876	UCKL1-AS1	25.9471648	1.42384987	0.39630717	3.59279361	0.00032715
24884	UEVLD	743.606544	0.51453585	0.12087408	4.25679214	2.07E-05
24888	UFM1	2992.93878	0.61465078	0.06017204	10.2148894	1.70E-24
24889	UFSP1	93.584065	1.20572514	0.21818961	5.5260428	3.28E-08
24897	UGP2	3294.29537	-0.5532778	0.05516115	-10.030207	1.12E-23
24922	UHRF1BP1	1832.29954	0.8010416	0.08899219	9.00125774	2.23E-19
24923	UHRF1BP1L	1472.73047	0.27684895	0.07890186	3.50877614	0.00045017
24930	ULK2	570.846537	-0.6457381	0.10006402	-6.4532496	1.09E-10
24932	ULK4	166.915954	0.9669053	0.15796961	6.12083116	9.31E-10
24936	UMAD1	590.464859	-0.4972915	0.09212334	-5.398106	6.73E-08
24941	UNC119	694.78782	-1.0034123	0.11472005	-8.7466164	2.20E-18
24946	UNC13D	9714.42994	-0.1312231	0.05338692	-2.4579631	0.01397275
24947	UNC45A	2814.18544	-0.6782865	0.07852941	-8.6373567	5.75E-18
24959	UNC93B1	2115.27807	-0.7934384	0.06484527	-12.235871	2.00E-34
24967	UPF1	7221.80161	0.41230826	0.05072337	8.12856544	4.34E-16
24968	UPF2	2322.69939	0.25277316	0.06892561	3.66733277	0.00024509
24970	UPF3B	1551.57051	0.50238704	0.07626359	6.58750868	4.47E-11
24978	UPP1	8004.50915	1.99361322	0.08547007	23.3252791	2.46E-120
24983	UQCC3	1050.74627	-0.7299193	0.26421268	-2.7626201	0.00573395
24984	UQCR10	1943.87286	-0.1555488	0.06506166	-2.3907901	0.01681216

24988	UQCRC1	6560.3386	-0.1457384	0.0613381	-2.3759853	0.01750216
24990	UQCRFS1	2865.65877	-0.3716666	0.08024337	-4.6317425	3.63E-06
24991	UQCRH	3860.7381	0.28322335	0.0862832	3.28248532	0.00102896
24996	URB1	2253.25616	1.12273842	0.08788289	12.7753926	2.25E-37
24998	URB2	1428.58963	0.74873636	0.07138891	10.4881332	9.79E-26
24999	URGCP	2689.17579	0.35528604	0.06869097	5.17223818	2.31E-07
25004	UROD	3118.75425	0.24503673	0.08812408	2.78058762	0.00542606
25005	UROS	2740.77982	-0.3421891	0.07793434	-4.3907358	1.13E-05
25008	USF1	1268.55529	-0.412359	0.07606311	-5.4212745	5.92E-08
25014	USMG5	4124.1762	-0.2869967	0.08758641	-3.276726	0.00105018
25016	USP1	4876.80006	0.18628203	0.05474839	3.40251152	0.0006677
25017	USP10	3357.26113	0.61785707	0.05912309	10.4503509	1.46E-25
25018	USP11	2988.46948	-0.2659162	0.05702341	-4.6632806	3.11E-06
25023	USP14	7115.54922	0.21889779	0.05541086	3.95044941	7.80E-05
25024	USP15	1994.52245	0.27213362	0.06713577	4.05348163	5.05E-05
25058	USP21	971.067741	-0.5475929	0.07902978	-6.9289444	4.24E-12
25060	USP24	3714.23502	0.50421158	0.08511546	5.92385458	3.14E-09
25061	USP25	1533.95475	0.79281255	0.08146075	9.73244893	2.19E-22
25067	USP3	1268.91468	0.55605108	0.08050067	6.90740908	4.94E-12
25071	USP31	865.105952	0.69107642	0.08623685	8.01370193	1.11E-15
25072	USP32	3504.42516	0.47867507	0.07618779	6.28283173	3.32E-10
25074	USP32P2	289.197111	-0.3073531	0.12946878	-2.3739553	0.01759868
25075	USP33	2696.57863	0.53292704	0.06703546	7.94992711	1.87E-15
25077	USP35	476.943926	-0.8381361	0.10119025	-8.2827752	1.20E-16
25078	USP36	2567.14993	1.39201227	0.07053055	19.7363019	1.05E-86
25079	USP37	837.350797	0.20739471	0.10159204	2.04144648	0.04120647
25080	USP38	1633.25538	0.27499393	0.08060363	3.41168195	0.00064563
25081	USP39	3829.2938	0.21659484	0.05578163	3.88290621	0.00010322
25086	USP44	136.421809	0.42859543	0.18482141	2.31897071	0.02039662
25087	USP45	664.365275	0.58388059	0.09699494	6.01970126	1.75E-09
25090	USP47	3590.73595	0.47134032	0.05435766	8.67109249	4.28E-18
25092	USP49	1031.39844	1.22831817	0.11090957	11.0749526	1.66E-28
25093	USP5	3102.78937	-0.5576407	0.06226535	-8.9558752	3.37E-19
25096	USP53	2043.9893	0.58033169	0.0921282	6.29917557	2.99E-10
25104	USPL1	583.437752	0.4435845	0.11205529	3.95862174	7.54E-05
25108	UTP11L	1429.99184	0.32682244	0.06661113	4.90642389	9.28E-07
25109	UTP14A	1443.8166	0.2434604	0.07201399	3.38073734	0.00072292
25111	UTP15	1279.91715	0.79007173	0.09380443	8.42254154	3.68E-17
25112	UTP18	4043.44384	0.15787121	0.07147784	2.2086736	0.02719735
25113	UTP20	2241.24315	0.35055596	0.08368021	4.18923399	2.80E-05
25114	UTP23	1189.78998	0.78690238	0.08459665	9.30181514	1.38E-20
25116	UTP6	2738.30915	0.55147515	0.07062294	7.80872519	5.78E-15
25122	UVRAG	1604.33922	0.62874628	0.07113387	8.83891551	9.67E-19

25124	UXS1	4404.89428	-0.4784301	0.05291741	-9.0410722	1.55E-19
25129	VAMP1	125.051937	-0.8604762	0.1962767	-4.3839959	1.17E-05
25134	VAMP7	3114.03164	0.20371116	0.06034602	3.37571824	0.00073623
25135	VAMP8	4030.72332	-0.2879275	0.09836834	-2.9270341	0.00342211
25136	VANGL1	2518.04119	-0.8822751	0.07051306	-12.512221	6.40E-36
25140	VARS	9467.32853	0.8814612	0.06529085	13.500531	1.55E-41
25142	VASH1	80.5022205	-1.5536675	0.23509122	-6.6087858	3.87E-11
25144	VASN	1246.41339	-1.3998842	0.07803721	-17.938677	5.88E-72
25145	VASP	4952.16056	0.32674565	0.07082524	4.61340707	3.96E-06
25157	VCL	12805.8277	-0.3084586	0.08150142	-3.7847018	0.00015389
25159	VCPIP1	1149.93884	0.30950636	0.09127435	3.39094578	0.00069652
25160	VCPKMT	339.473894	0.4506122	0.127657	3.52986662	0.00041577
25167	VDAC1	9156.1829	-0.4066267	0.04965589	-8.1888923	2.64E-16
25169	VDAC3	3714.87368	-0.5372022	0.09193963	-5.8429878	5.13E-09
25171	VEGFA	17634.9537	1.37615282	0.06171649	22.2979747	3.87E-110
25172	VEGFB	5767.66458	-0.36365	0.06729025	-5.4042003	6.51E-08
25179	VEZT	3945.70901	0.51040431	0.0633347	8.05884104	7.70E-16
25183	VGLL3	498.849509	1.16970731	0.12651714	9.24544504	2.34E-20
25184	VGLL4	1513.82773	-0.2072483	0.07016191	-2.9538579	0.00313828
25189	VIM	61947.3139	-0.2695659	0.04756941	-5.6667906	1.45E-08
25198	VKORC1	1868.39699	-0.498615	0.09597204	-5.1954192	2.04E-07
25200	VLDLR	838.180948	0.44768666	0.08540986	5.24162717	1.59E-07
25201	VLDLR-AS1	35.6315968	3.3143246	0.43851129	7.55812837	4.09E-14
25203	VMAC	48.2315577	-0.5731825	0.28428769	-2.016206	0.04377844
25205	VMP1	4533.46113	-0.2219177	0.05972829	-3.7154531	0.00020284
25224	VPS16	748.299812	-0.7134354	0.08825914	-8.0834162	6.30E-16
25228	VPS26B	1894.10842	-0.7553337	0.06252418	-12.080665	1.34E-33
25232	VPS33B	611.487427	-0.356057	0.10107094	-3.5228429	0.00042694
25234	VPS36	983.569774	-0.3042244	0.08751558	-3.4762317	0.00050851
25235	VPS37A	1534.31146	0.30336688	0.09095363	3.33540148	0.00085176
25236	VPS37B	2995.04664	0.453043	0.05810572	7.79687489	6.35E-15
25237	VPS37C	1490.30193	0.86989566	0.07074668	12.2959213	9.53E-35
25238	VPS37D	98.8218323	-0.8576835	0.23255604	-3.6880726	0.00022596
25240	VPS41	1793.41483	-0.4427445	0.06813654	-6.497901	8.14E-11
25242	VPS4A	2977.77807	-0.3066497	0.06446212	-4.7570525	1.96E-06
25244	VPS50	1213.83591	0.19927895	0.07138968	2.79142517	0.00524765
25245	VPS51	3015.29579	-0.3952568	0.08240161	-4.7967118	1.61E-06
25247	VPS53	2128.06893	-0.1347351	0.06864554	-1.9627659	0.04967339
25248	VPS54	897.566921	-0.3639368	0.09472011	-3.8422337	0.00012192
25251	VPS9D1	474.867009	-0.4388875	0.10509999	-4.1759038	2.97E-05
25252	VPS9D1-AS1	399.852116	1.38701365	0.11999759	11.5586795	6.67E-31
25255	VRK3	1026.21286	-0.2636824	0.09037671	-2.9175923	0.00352745
25258	VSIG10	739.104691	-1.1735943	0.09288582	-12.634805	1.36E-36

25269	VSTM4	23.021208	1.20890938	0.44118074	2.74016806	0.00614078
25271	VSX1	28.3306373	-1.3055911	0.39602467	-3.2967418	0.00097813
25277	VTN	11.2901967	-3.0864783	0.7430464	-4.1538164	3.27E-05
25282	VWA1	1370.24355	-1.81862	0.10036606	-18.119869	2.22E-73
25290	VWA8	509.741115	-0.2721867	0.10652403	-2.5551673	0.01061368
25297	VWDE	193.724485	1.20691655	0.16473797	7.32628045	2.37E-13
25299	WAC	6233.93924	0.32330412	0.08423046	3.83832768	0.00012388
25301	WAPL	3878.61479	0.45057172	0.08526198	5.28455593	1.26E-07
25302	WARS	5673.37137	1.25846247	0.07197741	17.4841309	1.89E-68
25307	WASF2	4837.05657	0.46136855	0.05703378	8.08939087	6.00E-16
25319	WBP1L	1592.01018	-0.502312	0.06496017	-7.7326145	1.05E-14
25322	WBP4	1137.14535	0.45520427	0.08823789	5.15883009	2.48E-07
25329	WDFY1	2942.54708	0.20152382	0.07114879	2.83242786	0.0046196
25330	WDFY2	289.26327	-0.5117893	0.13368547	-3.8283089	0.00012903
25334	WDHD1	1987.56474	0.22245979	0.06773693	3.28417292	0.00102282
25337	WDR11	3239.69676	-0.1967056	0.05811349	-3.3848531	0.00071216
25339	WDR12	1897.67524	0.32189976	0.06353993	5.06610173	4.06E-07
25340	WDR13	2521.94624	-0.2264096	0.07101465	-3.1882098	0.00143157
25344	WDR20	964.145857	0.54537973	0.08983849	6.07066892	1.27E-09
25345	WDR24	576.002479	-0.3886102	0.10619926	-3.6592556	0.00025295
25349	WDR3	3140.23689	1.08005351	0.06322171	17.0835854	1.97E-65
25352	WDR34	4575.08718	-0.6340711	0.08074489	-7.8527711	4.07E-15
25353	WDR35	1209.07774	0.84459007	0.08846816	9.54682546	1.34E-21
25354	WDR36	2705.5759	0.59343499	0.06913744	8.58341007	9.21E-18
25357	WDR4	235.701509	0.50577969	0.13943883	3.62725144	0.00028645
25359	WDR43	3674.95943	1.30658613	0.07388436	17.6842038	5.55E-70
25363	WDR46	4219.97423	0.74230907	0.06088398	12.1921897	3.42E-34
25364	WDR47	1247.11378	0.27498258	0.0814501	3.37608638	0.00073525
25368	WDR53	680.600555	0.71653441	0.10756712	6.66127707	2.71E-11
25369	WDR54	811.602304	-0.2895045	0.10063364	-2.8768162	0.0040171
25373	WDR6	7111.59721	-0.6008028	0.04987864	-12.045292	2.05E-33
25374	WDR60	1824.8984	-0.8840148	0.0680527	-12.99015	1.39E-38
25375	WDR61	737.958585	-0.784912	0.09894919	-7.9324747	2.15E-15
25376	WDR62	2228.13899	0.12724322	0.06173624	2.06107813	0.03929559
25380	WDR7	447.663371	-0.6709862	0.13294273	-5.0471822	4.48E-07
25383	WDR73	849.283684	0.21174596	0.08233424	2.57178504	0.01011757
25384	WDR74	1625.98135	0.21402363	0.07653914	2.79626405	0.00516971
25385	WDR75	2887.37773	0.75824923	0.05970849	12.6991869	5.97E-37
25387	WDR77	2928.82455	0.27092004	0.08391511	3.22850117	0.00124441
25388	WDR78	47.7693494	2.73495482	0.34048502	8.03252604	9.55E-16
25390	WDR82	5048.6922	0.16183873	0.05962096	2.71446049	0.00663838
25392	WDR83OS	3050.84096	-0.3318958	0.09222464	-3.598776	0.00031972
25397	WDR89	844.890686	0.28231952	0.0797478	3.54015441	0.00039989

25398	WDR90	565.465599	-0.585839	0.09965563	-5.8786345	4.14E-09
25399	WDR91	363.205487	-0.2503294	0.11286661	-2.2179223	0.02656013
25402	WDSUB1	1359.77775	0.69944174	0.07238321	9.6630379	4.33E-22
25407	WEE2-AS1	19.5547329	1.84848985	0.47084313	3.92591443	8.64E-05
25423	WFS1	1109.04603	-0.7848277	0.07509458	-10.45119	1.45E-25
25426	WHAMMP2	22.0700842	-1.8994549	0.46863878	-4.0531322	5.05E-05
25427	WHAMMP3	54.5975218	-1.5467563	0.31049088	-4.981648	6.30E-07
25429	WHSC1L1	1457.73586	0.55488655	0.0979653	5.66411293	1.48E-08
25431	WIBG	429.253734	-0.3805611	0.11817185	-3.2204039	0.0012801
25433	WIPF1	1536.57836	-0.1651857	0.08094645	-2.0406793	0.04128272
25434	WIPF2	2668.08694	0.31193576	0.09179323	3.39824344	0.0006782
25437	WIPI2	3337.31712	-0.9310817	0.05897627	-15.787395	3.80E-56
25439	WISP2	135.527704	-4.5341136	0.30103804	-15.061597	2.90E-51
25441	WIZ	2218.21497	0.46590035	0.09269105	5.0263791	5.00E-07
25445	WNK3	282.421448	0.31137898	0.15070805	2.06610714	0.03881836
25461	WNT7B	1027.5439	-0.3837458	0.07792817	-4.9243534	8.46E-07
25469	WRN	648.992428	0.5104992	0.10802917	4.72556804	2.29E-06
25492	XAB2	1969.82162	-0.423544	0.06630871	-6.3874567	1.69E-10
25500	XBP1	4978.02959	0.93989481	0.07914877	11.8750393	1.60E-32
25527	XPC	721.176034	-0.6154721	0.09019536	-6.8237664	8.87E-12
25532	XPO4	1434.30273	0.40238573	0.10262778	3.92082656	8.82E-05
25533	XPO5	5608.42915	1.09471842	0.07416303	14.7609719	2.61E-49
25534	XPO6	5147.77855	0.41720001	0.05742589	7.26501586	3.73E-13
25536	XPOT	5291.44	0.60954925	0.07314616	8.33330521	7.86E-17
25540	XRCC3	1777.8103	0.57469759	0.07608172	7.55368809	4.23E-14
25543	XRCC6	12273.5693	0.1336229	0.04834361	2.7640238	0.00570934
25547	XRN2	8026.13426	0.3203401	0.06345986	5.04791719	4.47E-07
25548	XRRA1	486.146183	1.03558959	0.11360513	9.11569375	7.82E-20
25549	XXYLT1	1533.63387	-0.2624633	0.06982079	-3.7590997	0.00017053
25554	XYLT2	1131.97001	-0.3548997	0.07601133	-4.6690365	3.03E-06
25555	YAE1D1	532.732917	0.23676935	0.1082056	2.18814318	0.02865917
25557	YAP1	9457.30827	0.26562047	0.07239297	3.66914726	0.00024336
25558	YARS	7743.76107	1.74731163	0.05451627	32.0511948	2.11E-225
25565	YDJC	1489.3102	0.7279468	0.12373593	5.88306721	4.03E-09
25568	YES1	4839.046	0.49121774	0.07257759	6.76817341	1.30E-11
25569	YIF1A	2347.67154	-0.2351789	0.08528673	-2.7575091	0.00582436
25570	YIF1B	2198.12401	-0.2117089	0.07619017	-2.7786905	0.00545785
25579	YKT6	5010.82492	0.18676641	0.05393091	3.46306777	0.00053405
25582	YOD1	1301.83526	0.199953	0.08396511	2.38138195	0.01724782
25583	YPEL1	48.0170643	-1.0372315	0.28967054	-3.5807285	0.00034264
25584	YPEL2	306.916479	-0.3502315	0.12341726	-2.8377838	0.00454279
25585	YPEL3	652.022068	-0.9268463	0.10400649	-8.9114275	5.04E-19
25588	YRDC	2062.28199	1.50761681	0.07870687	19.1548311	8.82E-82

25591	YTHDF1	4630.44156	0.24213934	0.05405296	4.47966871	7.48E-06
25592	YTHDF2	3566.31991	0.88102623	0.06230309	14.1409711	2.12E-45
25593	YTHDF3	2584.13062	0.20990019	0.08427541	2.49064573	0.01275112
25594	YTHDF3-AS1	19.0360049	-0.9865118	0.48664489	-2.0271699	0.04264504
25600	YWHAH	6737.63058	-0.4857064	0.05444474	-8.921089	4.62E-19
25602	YWHAZ	35528.4024	0.37986265	0.04552487	8.34406957	7.18E-17
25603	YY1	4245.15211	0.2874729	0.05681923	5.05942975	4.21E-07
25604	YY1AP1	2235.12482	0.4240261	0.06650324	6.37602152	1.82E-10
25609	ZAK	2723.41377	-0.5360336	0.08047966	-6.6604851	2.73E-11
25620	ZBED4	1466.5786	0.36835989	0.08204944	4.48948694	7.14E-06
25622	ZBED5-AS1	748.212028	-0.4251441	0.09519852	-4.4658686	7.97E-06
25626	ZBED9	128.038058	0.45420245	0.18508901	2.45396772	0.01412897
25629	ZBTB10	1168.41875	0.57525306	0.09345896	6.15514062	7.50E-10
25630	ZBTB11	1523.68298	0.56328852	0.09219683	6.10963028	9.99E-10
25635	ZBTB17	1295.47946	0.665905	0.0741822	8.97661416	2.79E-19
25637	ZBTB2	1660.8488	0.95445421	0.07346289	12.9923307	1.35E-38
25642	ZBTB21	1643.2664	1.7282153	0.10355783	16.6884071	1.59E-62
25646	ZBTB26	212.121474	0.61829485	0.15688809	3.94099301	8.11E-05
25647	ZBTB3	81.1866891	-0.5157857	0.22223901	-2.3208605	0.02029438
25655	ZBTB40	1740.15247	0.5429753	0.06476971	8.38316739	5.15E-17
25658	ZBTB43	1373.16827	0.90552085	0.07770807	11.652854	2.22E-31
25660	ZBTB45	663.365649	-0.6992902	0.08912919	-7.8458042	4.30E-15
25663	ZBTB47	1639.9203	0.17735986	0.0705589	2.51364265	0.01194914
25669	ZBTB7B	1709.74284	0.3233965	0.07218953	4.47982555	7.47E-06
25671	ZBTB8A	490.373671	0.79528579	0.09914987	8.02104699	1.05E-15
25673	ZBTB8OS	825.676296	0.64738985	0.08696588	7.44418245	9.75E-14
25674	ZBTB9	729.280643	0.84046038	0.09070395	9.26597311	1.93E-20
25681	ZC3H12B	16.1098763	-1.4121871	0.57365361	-2.4617419	0.01382641
25682	ZC3H12C	5858.01305	0.45853118	0.0801534	5.72067018	1.06E-08
25683	ZC3H12D	47.9055146	-1.231057	0.3167259	-3.8868215	0.00010157
25686	ZC3H15	5377.46582	0.68128225	0.06371707	10.6923033	1.11E-26
25691	ZC3H7A	2891.16602	0.43849629	0.06332593	6.92443541	4.38E-12
25693	ZC3H8	641.092227	1.86040333	0.09909584	18.7737779	1.24E-78
25696	ZC3HC1	1075.65873	0.53227312	0.09500428	5.60262262	2.11E-08
25697	ZC4H2	393.991739	-0.4928757	0.1061509	-4.6431612	3.43E-06
25699	ZCCHC11	1691.41261	0.40907242	0.09500229	4.30592167	1.66E-05
25705	ZCCHC18	48.1310931	1.00317908	0.29245803	3.43016429	0.00060322
25706	ZCCHC2	399.591684	0.39314727	0.11951715	3.28946318	0.00100379
25707	ZCCHC24	1950.34676	-1.2969123	0.06566206	-19.751319	7.81E-87
25710	ZCCHC5	31.4892873	1.16136714	0.36165037	3.21129807	0.00132137
25713	ZCCHC8	1524.48799	0.57896569	0.08598185	6.73358028	1.66E-11
25716	ZCWPW1	231.990447	0.68960709	0.13977558	4.93367363	8.07E-07
25719	ZDHHC1	483.400538	-0.7759709	0.10500304	-7.3899852	1.47E-13

25720	ZDHHC11	44.1188069	1.17465769	0.29896816	3.9290394	8.53E-05
25721	ZDHHC12	1132.11035	-0.7956328	0.1103639	-7.2091759	5.63E-13
25725	ZDHHC16	2739.36571	-0.253885	0.0633129	-4.0100049	6.07E-05
25727	ZDHHC18	1245.78756	0.1551503	0.07207055	2.15275592	0.03133787
25729	ZDHHC2	2086.57474	-0.6606019	0.09388479	-7.0363043	1.97E-12
25731	ZDHHC21	440.701529	0.47864277	0.11180105	4.28120088	1.86E-05
25734	ZDHHC24	435.140836	-0.2931107	0.12991091	-2.2562441	0.02405535
25738	ZDHHC6	1717.05857	-0.4679693	0.06674328	-7.0114816	2.36E-12
25742	ZDHHC9	2299.19652	0.24121544	0.08119623	2.97077159	0.00297053
25743	ZEB1	2296.86842	0.54663145	0.09157442	5.96925909	2.38E-09
25745	ZEB2	623.211208	1.25858255	0.11242445	11.1949183	4.32E-29
25747	ZER1	2002.96574	-0.1965892	0.06262763	-3.1390169	0.00169516
25748	ZFAND1	2686.65187	0.83816598	0.06221462	13.47217	2.28E-41
25749	ZFAND2A	874.365429	0.64338907	0.08089093	7.95378503	1.81E-15
25750	ZFAND2B	656.797823	-0.3026453	0.10238798	-2.9558676	0.00311791
25751	ZFAND3	10453.7273	0.74911307	0.06841806	10.949055	6.71E-28
25752	ZFAND4	197.461614	-0.4856382	0.14337885	-3.3870977	0.00070636
25755	ZFAS1	4359.59417	1.64351082	0.08801481	18.6731163	8.19E-78
25763	ZFP1	443.174708	0.84020657	0.1077413	7.79837048	6.27E-15
25768	ZFP30	612.130646	0.34540172	0.10150432	3.40282778	0.00066692
25770	ZFP36L1	13817.0715	1.32871024	0.07697812	17.2608826	9.27E-67
25778	ZFP69	149.834776	0.85636533	0.16903061	5.06633284	4.06E-07
25779	ZFP69B	199.65013	2.51383716	0.19198233	13.0941067	3.56E-39
25780	ZFP82	584.147304	0.81086665	0.09450879	8.57980128	9.50E-18
25782	ZFP91	6079.04462	0.39334723	0.07025055	5.599205	2.15E-08
25785	ZFPL1	1536.69891	-0.2331749	0.06979877	-3.3406741	0.00083575
25789	ZFR	7655.90003	0.25763377	0.07086508	3.63555298	0.00027738
25794	ZFYVE1	1340.93288	0.54902014	0.07127189	7.70317937	1.33E-14
25796	ZFYVE19	1582.38211	-0.6051656	0.10369526	-5.8360007	5.35E-09
25797	ZFYVE21	1033.32728	-0.8045156	0.08571558	-9.3858736	6.24E-21
25801	ZFYVE9	1235.18404	0.27130084	0.07745497	3.5026912	0.00046058
25803	ZG16B	37.6044236	-1.4574397	0.33963198	-4.2912322	1.78E-05
25807	ZHX1	2255.69978	0.44835932	0.06546274	6.84907627	7.43E-12
25810	ZHX3	1653.55325	-0.1652063	0.07917086	-2.0867055	0.03691477
25820	ZKSCAN1	2062.49006	0.21840311	0.07522638	2.90327833	0.00369278
25826	ZKSCAN8	2170.3872	0.86832397	0.2328431	3.72922355	0.00019207
25828	ZMAT2	3608.39638	0.15132999	0.07032526	2.15185838	0.03140851
25831	ZMAT5	504.640291	0.38794911	0.09928377	3.90747763	9.33E-05
25835	ZMPSTE24	4169.44309	0.51095021	0.0569488	8.97209754	2.91E-19
25836	ZMYM1	1102.76574	0.48025325	0.0800452	5.99977574	1.98E-09
25838	ZMYM3	2260.88997	-1.2243254	0.08764759	-13.968729	2.42E-44
25839	ZMYM4	2492.82851	0.38874856	0.078999	4.92093017	8.61E-07
25840	ZMYM5	578.532658	0.97367629	0.1010523	9.63537032	5.67E-22

25841	ZMYM6	654.463882	0.29753336	0.08910827	3.33900947	0.00084078
25848	ZMYND8	5397.50018	-0.2952136	0.05927507	-4.9804015	6.35E-07
25849	ZNF10	281.907031	0.54174315	0.1290222	4.1988368	2.68E-05
25850	ZNF100	453.761884	0.36777991	0.11758454	3.1277913	0.00176125
25855	ZNF114	4703.39398	1.2043057	0.05819554	20.694125	3.91E-95
25858	ZNF121	236.239878	0.8795645	0.20857859	4.21694527	2.48E-05
25859	ZNF124	258.799436	1.27392573	0.14132499	9.01415754	1.98E-19
25860	ZNF131	2394.41493	0.61076863	0.07047001	8.66707163	4.43E-18
25863	ZNF134	638.762091	0.61188973	0.09292723	6.58461175	4.56E-11
25865	ZNF136	227.506652	0.31066861	0.14174305	2.19177313	0.02839589
25868	ZNF14	255.069153	0.89044927	0.13826281	6.44026612	1.19E-10
25869	ZNF140	695.703837	1.03294895	0.09637983	10.7174807	8.43E-27
25871	ZNF142	794.474774	0.41637803	0.09613799	4.3310457	1.48E-05
25873	ZNF146	6017.14218	1.00276881	0.05718713	17.534868	7.76E-69
25878	ZNF16	316.67787	0.54422669	0.12898511	4.21929869	2.45E-05
25884	ZNF175	649.8549	0.30677441	0.11002297	2.78827604	0.00529894
25889	ZNF182	790.856877	0.45875539	0.08648499	5.30445133	1.13E-07
25892	ZNF189	587.148508	0.36267591	0.09508026	3.81441879	0.0001365
25895	ZNF195	1070.10061	0.81697488	0.07365693	11.0916233	1.38E-28
25896	ZNF197	667.242861	0.47209284	0.08891333	5.30958429	1.10E-07
25900	ZNF200	366.408512	0.26982165	0.11408976	2.36499439	0.01803034
25901	ZNF202	940.981665	0.36147891	0.08423109	4.29151416	1.77E-05
25905	ZNF207	3471.43406	-0.228024	0.08891752	-2.5644441	0.01033412
25907	ZNF211	743.461009	-0.4006743	0.08448935	-4.7423059	2.11E-06
25913	ZNF217	3560.85084	0.31077394	0.07713943	4.02873029	5.61E-05
25918	ZNF223	178.666672	-0.3921882	0.15885971	-2.4687707	0.0135578
25924	ZNF23	1054.89131	1.22224504	0.07794898	15.680064	2.07E-55
25926	ZNF232	472.68759	1.04378289	0.10802924	9.66204066	4.37E-22
25933	ZNF248	440.913154	-0.6550333	0.10781821	-6.0753499	1.24E-09
25934	ZNF25	262.01055	-0.6234927	0.1315921	-4.7380708	2.16E-06
25935	ZNF250	363.225541	0.27966269	0.11228495	2.49065164	0.01275091
25936	ZNF251	987.22861	0.26214349	0.07791035	3.36468124	0.00076632
25943	ZNF26	355.300268	0.83411265	0.11678277	7.14242935	9.17E-13
25944	ZNF260	659.659641	0.58531649	0.09808847	5.96723038	2.41E-09
25945	ZNF263	1046.79669	0.17815731	0.07552284	2.35898588	0.01832495
25946	ZNF264	722.943309	0.32147637	0.09253996	3.47391937	0.00051292
25948	ZNF267	490.031576	0.71085213	0.10446665	6.8045842	1.01E-11
25950	ZNF271P	1213.98137	0.73416589	0.07625799	9.6273962	6.13E-22
25952	ZNF274	2058.25388	0.55690181	0.06496272	8.57263685	1.01E-17
25954	ZNF276	826.879887	0.37389599	0.08781737	4.25765399	2.07E-05
25956	ZNF28	548.018696	0.87381291	0.11572577	7.55071994	4.33E-14
25960	ZNF280D	480.187041	-0.3924619	0.1015605	-3.8643164	0.0001114
25961	ZNF281	1880.82825	0.30780494	0.07891479	3.90047205	9.60E-05

25966	ZNF286A	351.354911	0.83597688	0.11970372	6.98371667	2.87E-12
25967	ZNF286B	39.8389949	0.7652631	0.31436498	2.43431407	0.01492004
25971	ZNF296	181.176101	0.58377107	0.14843739	3.93277653	8.40E-05
25972	ZNF3	1064.05784	0.58767279	0.07976647	7.36741647	1.74E-13
25975	ZNF300	9.3206201	3.72181679	0.92095621	4.04125273	5.32E-05
25981	ZNF317	1888.19945	0.65346241	0.07546256	8.65942494	4.74E-18
25982	ZNF318	1415.14057	-0.284455	0.07343582	-3.8735183	0.00010728
25984	ZNF32	752.309569	-0.4765513	0.09651577	-4.9375489	7.91E-07
25991	ZNF324	455.830841	0.49282691	0.10822784	4.55360569	5.27E-06
25995	ZNF330	2500.22803	0.94248577	0.07603756	12.3950027	2.78E-35
25996	ZNF331	1049.83007	0.30067544	0.07853843	3.82838601	0.00012899
25998	ZNF334	259.973292	-0.2785794	0.12633997	-2.2049981	0.02745423
26006	ZNF341	180.796139	-0.9405265	0.16165615	-5.8180683	5.95E-09
26008	ZNF343	614.114184	0.40397844	0.08882074	4.54824437	5.41E-06
26010	ZNF346	578.077078	0.38610297	0.09450725	4.08543228	4.40E-05
26011	ZNF347	794.376302	0.66315617	0.09490871	6.98730605	2.80E-12
26012	ZNF35	338.315252	0.65173094	0.11644223	5.59703239	2.18E-08
26013	ZNF350	478.321279	0.64846822	0.10019809	6.47186176	9.68E-11
26015	ZNF354A	863.578228	1.2365183	0.09749469	12.6829293	7.35E-37
26018	ZNF358	2423.57798	-0.78339	0.08843536	-8.8583348	8.12E-19
26023	ZNF37A	1403.2628	0.60683748	0.09828207	6.17444764	6.64E-10
26024	ZNF37BP	811.727049	-0.1733536	0.08536839	-2.0306535	0.04229015
26029	ZNF385B	23.3006296	-1.8543108	0.43645702	-4.2485531	2.15E-05
26036	ZNF395	4202.52715	-0.5074909	0.05494625	-9.2361344	2.56E-20
26042	ZNF408	590.804248	0.30025614	0.09043717	3.32005232	0.00090001
26044	ZNF410	1733.54276	0.28330623	0.07639825	3.7082816	0.00020867
26045	ZNF414	333.669033	-0.5756497	0.1283401	-4.4853459	7.28E-06
26050	ZNF419	473.821672	0.82226179	0.11386294	7.22150491	5.14E-13
26051	ZNF420	339.37988	0.32441144	0.12186477	2.66206082	0.00776638
26054	ZNF426	295.527952	0.63826034	0.15846626	4.02773635	5.63E-05
26058	ZNF430	354.862875	0.70105341	0.15505245	4.5213951	6.14E-06
26059	ZNF431	298.532782	0.34927436	0.13615603	2.56525077	0.01031013
26062	ZNF436	549.537723	0.24392241	0.09430036	2.58665403	0.00969128
26066	ZNF44	300.868667	0.3664741	0.12219273	2.99914803	0.00270736
26067	ZNF440	544.355675	0.30716284	0.10658541	2.88184697	0.00395352
26069	ZNF442	42.7479549	1.26837602	0.32591918	3.89168883	9.95E-05
26073	ZNF446	345.096453	-0.3483765	0.11755629	-2.9634869	0.00304175
26078	ZNF460	129.196517	1.63759324	0.26888158	6.09038827	1.13E-09
26081	ZNF467	166.089479	-2.2696439	0.18353202	-12.366474	3.97E-35
26082	ZNF468	928.94437	0.93559581	0.09844799	9.50345281	2.03E-21
26086	ZNF473	870.690118	0.87096407	0.08696645	10.0149433	1.31E-23
26089	ZNF48	412.65475	0.46707115	0.11210145	4.16650402	3.09E-05
26092	ZNF484	202.428912	0.83525835	0.14855401	5.62259026	1.88E-08

26094	ZNF486	544.504772	0.2929824	0.10906004	2.6864322	0.00722196
26096	ZNF488	307.859222	-2.2036263	0.14659808	-15.031754	4.55E-51
26102	ZNF497	115.206163	-0.7786765	0.19368354	-4.0203547	5.81E-05
26103	ZNF500	232.630228	-0.311352	0.14319577	-2.1743101	0.02968185
26105	ZNF502	445.228456	1.01607117	0.10566425	9.61603515	6.84E-22
26106	ZNF503	65.4109792	2.40116638	0.29381029	8.1725061	3.02E-16
26108	ZNF503-AS2	11.1618856	1.51522103	0.62029396	2.44274672	0.01457596
26109	ZNF506	508.126606	0.86469901	0.10777046	8.02352509	1.03E-15
26110	ZNF507	1350.26099	0.45071652	0.10408227	4.33038717	1.49E-05
26112	ZNF511	1525.99912	-0.2692575	0.08735826	-3.0822212	0.00205462
26115	ZNF513	262.57902	-0.3266503	0.12778757	-2.5561979	0.01058229
26117	ZNF516	570.739872	0.28465048	0.10005239	2.84501445	0.00444094
26120	ZNF518B	1079.4066	0.43687442	0.08937269	4.88823181	1.02E-06
26123	ZNF524	432.457837	-0.2652048	0.10702033	-2.4780787	0.0132092
26127	ZNF528	839.912378	0.55278586	0.09340426	5.91820803	3.25E-09
26128	ZNF528-AS1	645.89516	0.68301615	0.11980308	5.70115672	1.19E-08
26130	ZNF529-AS1	167.799099	0.82605185	0.17564759	4.70289327	2.57E-06
26132	ZNF532	999.019543	0.49513994	0.11526903	4.29551588	1.74E-05
26137	ZNF542P	177.815271	1.09004008	0.15311294	7.11918977	1.09E-12
26139	ZNF544	1432.41183	0.30334509	0.07519268	4.0342369	5.48E-05
26143	ZNF549	388.992013	0.38905059	0.11110792	3.50155588	0.00046255
26144	ZNF550	411.706347	0.59788811	0.11606658	5.1512512	2.59E-07
26145	ZNF551	206.805014	0.40035734	0.16206133	2.47040638	0.01349596
26148	ZNF555	287.810582	0.6348763	0.12520624	5.07064407	3.96E-07
26151	ZNF558	1313.21295	0.7101454	0.08470929	8.3833237	5.15E-17
26157	ZNF562	971.572399	0.55497768	0.09089437	6.10574288	1.02E-09
26158	ZNF563	162.362652	0.52113642	0.15927417	3.27194549	0.0010681
26161	ZNF566	502.345731	0.95228046	0.10828558	8.79415793	1.44E-18
26162	ZNF567	400.159611	0.38270588	0.12161183	3.14694603	0.00164985
26163	ZNF568	322.175707	0.32283386	0.12480121	2.58678466	0.00968761
26165	ZNF57	230.025535	0.91631955	0.15883829	5.76888317	7.98E-09
26166	ZNF570	137.634603	0.62951879	0.19008878	3.31170937	0.00092728
26167	ZNF571	117.453078	0.97461202	0.19380798	5.02875081	4.94E-07
26172	ZNF575	38.9378275	-1.5664014	0.33186266	-4.720029	2.36E-06
26173	ZNF576	417.69803	-0.3362598	0.12688013	-2.6502165	0.00804402
26175	ZNF578	14.4737783	1.96445503	0.55218783	3.55758482	0.00037428
26177	ZNF580	1184.32055	-0.5227031	0.09832177	-5.3162498	1.06E-07
26182	ZNF584	987.163746	0.76118649	0.08323558	9.14496479	5.96E-20
26185	ZNF586	249.882409	0.51493718	0.13489476	3.81732526	0.00013491
26189	ZNF592	1635.5029	-0.181505	0.0702143	-2.5850151	0.00973747
26190	ZNF593	838.862753	1.4206023	0.13880435	10.2345663	1.39E-24
26191	ZNF594	271.966151	0.42370701	0.13890106	3.05042308	0.00228519
26197	ZNF600	595.079742	1.12560995	0.10402075	10.8210136	2.74E-27

26200	ZNF607	343.185503	0.27509905	0.13201148	2.08390248	0.03716904
26202	ZNF609	2074.8795	0.46294545	0.09052475	5.11402061	3.15E-07
26206	ZNF614	522.819402	0.40590879	0.11767833	3.44930783	0.00056203
26208	ZNF616	288.543376	1.13496331	0.12400124	9.15283856	5.55E-20
26213	ZNF622	2953.14492	0.44868859	0.10512473	4.2681544	1.97E-05
26214	ZNF623	898.155605	0.61523512	0.09313609	6.60576517	3.95E-11
26224	ZNF638	2318.53222	0.36916683	0.06207312	5.94728973	2.73E-09
26225	ZNF639	2323.37144	0.36877903	0.05992888	6.15361155	7.57E-10
26227	ZNF644	2125.63568	0.19265366	0.09449673	2.03873356	0.04147663
26233	ZNF652	720.851022	-0.5364773	0.10500139	-5.1092399	3.23E-07
26234	ZNF653	448.811719	-0.6557168	0.12073685	-5.4309587	5.61E-08
26235	ZNF654	783.065719	0.31921426	0.10930674	2.92035296	0.00349635
26236	ZNF655	3656.66717	0.87455062	0.07024562	12.449895	1.40E-35
26244	ZNF665	76.2592311	1.67830925	0.24076655	6.97069106	3.15E-12
26248	ZNF669	333.399634	0.77928794	0.12313626	6.3286632	2.47E-10
26249	ZNF670	344.775055	1.13454744	0.12479302	9.09143384	9.77E-20
26252	ZNF672	970.40975	-0.4457319	0.09380063	-4.7519069	2.02E-06
26254	ZNF674-AS1	363.739798	0.73809259	0.11812914	6.24818404	4.15E-10
26255	ZNF675	411.586706	1.11012459	0.12468495	8.90343726	5.41E-19
26261	ZNF681	208.800522	1.03502953	0.15662099	6.6084983	3.88E-11
26265	ZNF687	1260.37925	0.23742829	0.0737941	3.21744284	0.00129339
26266	ZNF688	176.066674	-0.8719384	0.16134727	-5.4041101	6.51E-08
26268	ZNF69	281.45534	-0.3626363	0.13260412	-2.734729	0.00624316
26269	ZNF691	233.424635	0.40940475	0.14764223	2.77295159	0.00555504
26272	ZNF696	621.122975	0.52430332	0.09731149	5.38788683	7.13E-08
26273	ZNF697	1693.09893	1.15859168	0.0730912	15.851315	1.38E-56
26274	ZNF699	69.9205558	0.73848679	0.25197871	2.93075079	0.00338144
26281	ZNF704	105.762948	-1.2567328	0.20684305	-6.0757797	1.23E-09
26288	ZNF707	606.473195	0.27589211	0.09831231	2.80628247	0.00501167
26299	ZNF720	140.002072	0.53162432	0.1908856	2.78504149	0.00535209
26300	ZNF721	661.894862	0.27921251	0.10082369	2.76931452	0.00561744
26311	ZNF737	184.508879	-0.7955525	0.1652946	-4.8129369	1.49E-06
26315	ZNF746	1033.77616	0.3322233	0.08313985	3.99595758	6.44E-05
26317	ZNF749	284.982209	0.47423258	0.13859492	3.42171687	0.00062227
26327	ZNF767P	384.824891	-0.2609136	0.11672041	-2.2353727	0.02539289
26329	ZNF77	146.270069	0.34742462	0.16752718	2.07384032	0.03809414
26330	ZNF770	2218.64791	0.64899728	0.07718764	8.40804672	4.17E-17
26331	ZNF771	143.26282	-1.7888314	0.1919213	-9.3206507	1.16E-20
26335	ZNF775	621.225376	-0.7723173	0.10677489	-7.2331357	4.72E-13
26336	ZNF776	822.683327	0.19698124	0.0909145	2.1666648	0.03026042
26338	ZNF778	131.256704	0.79410119	0.2039894	3.89285526	9.91E-05
26342	ZNF782	115.598502	0.9005409	0.20512938	4.3901118	1.13E-05
26343	ZNF783	858.173431	0.49521034	0.08540221	5.79856563	6.69E-09

26344	ZNF784	143.80968	-0.7031957	0.1739615	-4.0422489	5.29E-05
26350	ZNF79	258.073133	0.68097347	0.13855982	4.91465307	8.89E-07
26351	ZNF790	316.667007	0.56950843	0.11910583	4.78153291	1.74E-06
26352	ZNF790-AS1	40.4959552	1.4377609	0.34614893	4.15359046	3.27E-05
26354	ZNF792	325.77151	-0.5737032	0.13653686	-4.2018192	2.65E-05
26358	ZNF8	179.119498	0.46245432	0.15936337	2.90188596	0.00370924
26360	ZNF800	946.502572	0.52250705	0.09237194	5.65655627	1.54E-08
26366	ZNF81	179.732001	0.58177937	0.17009319	3.42035656	0.00062539
26375	ZNF823	346.589309	0.3784419	0.11318198	3.3436587	0.00082681
26385	ZNF837	147.940469	-0.6824222	0.17144215	-3.980481	6.88E-05
26387	ZNF84	1139.3474	0.45966945	0.0756824	6.07366416	1.25E-09
26388	ZNF841	838.525946	0.63484329	0.10755639	5.902423	3.58E-09
26394	ZNF85	342.43001	0.61032311	0.13130714	4.64805728	3.35E-06
26399	ZNF862	792.139925	-0.6298028	0.09976461	-6.3128874	2.74E-10
26400	ZNF865	1030.51112	-0.5812	0.07402875	-7.8510036	4.13E-15
26403	ZNF879	119.869573	1.25859363	0.18677365	6.73860376	1.60E-11
26410	ZNF91	540.47236	0.31243636	0.10074894	3.10113788	0.00192779
26411	ZNF92	331.808228	-0.370092	0.11740767	-3.1521962	0.00162047
26415	ZNFX1	4581.40502	0.73224128	0.07849557	9.3284412	1.07E-20
26416	ZNHIT1	1721.16774	-0.2736878	0.11625724	-2.3541569	0.01856477
26417	ZNHIT2	794.042918	0.56011417	0.14274758	3.92380839	8.72E-05
26419	ZNHIT6	3056.04579	0.71576306	0.08428233	8.49244461	2.02E-17
26436	ZPR1	3169.02302	0.95803986	0.05564998	17.2154559	2.03E-66
26437	ZRANB1	2419.41123	0.18059363	0.06299524	2.86678211	0.00414668
26438	ZRANB2	2953.78747	0.25682616	0.07031594	3.65246004	0.00025974
26445	ZSCAN12	562.270935	1.54530852	0.1042934	14.8169346	1.14E-49
26446	ZSCAN12P1	48.5005147	3.71513399	0.40911773	9.08084325	1.08E-19
26447	ZSCAN16	285.965151	0.45876136	0.12740009	3.60095013	0.00031706
26448	ZSCAN16-AS	162.903112	-0.5951804	0.18169211	-3.2757636	0.00105377
26450	ZSCAN2	138.975695	-0.5159311	0.17370715	-2.97012	0.00297683
26451	ZSCAN20	188.26427	0.9309259	0.15671968	5.94007006	2.85E-09
26452	ZSCAN21	433.070704	0.70699796	0.10360084	6.82424906	8.84E-12
26455	ZSCAN25	711.433301	0.18357201	0.0867436	2.11626002	0.0343227
26464	ZSCAN9	406.219309	0.40047788	0.10487781	3.81851872	0.00013426
26465	ZSWIM1	335.080727	-0.2979219	0.12553899	-2.3731422	0.01763747
26470	ZSWIM6	1762.33814	0.81691937	0.1084728	7.53109862	5.03E-14
26471	ZSWIM7	605.620893	0.3444774	0.11256857	3.06015613	0.00221222
26476	ZWILCH	1906.37643	0.55246842	0.07035981	7.85204502	4.09E-15
26477	ZWINT	4470.08396	-0.3407133	0.06962748	-4.8933741	9.91E-07
26478	ZXDA	145.08831	0.54819371	0.18761001	2.9219854	0.00347808
26479	ZXDB	737.836883	0.37008562	0.08739374	4.23469253	2.29E-05
26483	ZYX	10739.5627	0.59867914	0.0578928	10.3411678	4.59E-25
26485	ZZZ3	2621.9686	0.7915169	0.07446082	10.6299788	2.16E-26

padj	CtrlA_1	CtrlA_2	CtrlA_3	TrtB_1	TrtB_2	TrtB_3
3.22E-14	1063.32249	1106.01784	1069.14323	652.531055	722.115081	690.863766
0.00018827	1140.76921	1055.96735	1042.36848	829.035357	811.275007	911.863302
0.01407494	191.523638	154.07702	168.0346	143.075457	122.092872	91.2824169
3.37E-05	1524.86306	1565.30476	1474.45745	1854.63232	1820.14769	1972.66107
0.0024705	450.02822	376.8508	344.378604	266.093606	274.708963	313.24282
0.000473	1983.2639	1972.57841	2024.72461	2516.52345	2383.22074	2245.54746
2.19E-236	4618.54543	4549.6883	4542.47381	14365.0433	15112.2059	14521.5912
0.06751384	676.088908	717.390455	671.215134	720.725899	808.062037	823.463487
1.51E-30	2903.20531	3084.48454	3053.24408	4787.01061	4757.60581	4733.23353
1.12E-13	2051.29142	1895.04921	2286.93244	1276.98188	1378.36427	1186.67142
0.04694927	20.9315451	11.7765875	18.4653407	6.685769	4.81945549	8.64780792
2.29E-17	5328.12481	5430.9696	4911.78062	7484.04982	7744.06173	8413.35624
2.33E-15	63.8412127	85.3802594	91.4034363	228.6533	233.743591	214.273463
2.65E-09	253.271696	277.731189	251.128633	437.249293	417.686142	431.429528
1.60E-105	4243.87078	4478.0474	4649.57278	1497.61226	1611.30462	1435.53611
4.72E-15	132.915312	109.914817	132.950453	34.7659988	30.5232181	21.139086
2.46E-38	1396.13406	1398.46977	1358.12581	552.24452	669.904313	617.837832
1.37E-24	1547.88776	1609.46696	1673.88313	893.218739	938.187335	963.750149
2.59E-17	877.031741	746.831924	794.932916	401.14614	426.521811	461.216422
7.85E-48	6523.31604	6402.53807	6616.13156	3826.93418	3869.21952	3826.17457
6.03E-05	71.1672535	54.9574083	70.1682946	24.0687684	27.3102478	27.8651588
7.25E-31	1091.58008	1169.80769	1160.54666	595.033441	510.862282	505.416329
0.02312253	5637.91168	6184.6712	6271.75296	6626.93424	6769.72848	6728.95543
1.10E-06	8802.76131	8107.19911	8452.50969	10284.0499	10216.4424	10207.2959
2.79E-19	4389.34502	4699.8398	4964.40684	7086.91514	6978.57155	6875.00729
7.92E-05	3297.76494	3030.50852	2964.61045	2436.29423	2547.08223	2625.09014
3.17E-21	71.1672535	79.4919656	70.1682946	225.978992	265.873295	230.608211
0.05596366	14.6520816	15.7021167	9.23267034	1.3371538	6.42594065	4.80433773
2.38E-05	6237.60045	6015.87345	5808.27291	4942.12045	4937.53215	5209.82384
1.62E-05	444.795334	353.297625	331.452865	243.361992	248.201958	224.843006
4.35E-19	693.880721	716.409073	685.987406	339.637065	346.197553	398.760032
6.15E-16	850.86731	913.666914	789.393314	462.655215	509.255797	476.590303
2.89E-45	2167.4615	2129.59957	2265.6973	1135.24358	1085.18073	1052.14996
0.07354074	3691.27799	3836.22338	4446.45403	3772.11087	3629.04998	3092.07176
1.99E-06	984.829199	932.313177	1049.75462	760.840513	689.985378	737.946276
7.27E-10	2388.2893	2354.33612	2658.08579	1818.52917	1778.37908	1597.92273
2.25E-05	3563.59556	3733.17824	4002.36259	4827.12522	4674.06858	4434.40373
2.50E-70	3948.73599	4227.79491	4531.3946	11478.1282	11147.4005	9937.29217
2.94E-05	5182.65058	5190.53094	5776.88183	4463.41939	4441.93148	3904.00484
4.42E-15	95.2385304	101.082376	81.247499	20.057307	11.2453961	13.4521457
4.01E-14	1409.73957	1346.4565	1139.31152	652.531055	719.705353	804.246136
2.66E-30	1500.79179	1595.72761	1606.48464	2749.18821	2729.41829	2581.8511

0.00039131	2023.03384	1974.54117	1701.58114	1339.82811	1433.78801	1640.2009
4.35E-17	1561.49327	1580.02549	1396.90302	887.870124	899.631691	968.554487
7.86E-21	308.740291	289.507776	334.222666	121.680996	110.044234	89.3606818
0.00155424	630.039509	544.667172	564.116158	447.946523	455.438544	459.294687
3.34E-10	1395.08748	1418.09741	1405.21243	1821.20348	1943.0438	2003.40883
2.86E-12	672.949176	670.284105	562.269624	351.67145	371.901315	366.090535
1.02E-23	793.305561	825.342507	870.640813	438.586447	419.292628	427.586058
0.00077846	6980.6703	6822.56969	6122.1837	5538.49104	5549.603	5785.3835
9.23E-08	3486.14884	3201.26904	2948.91491	2110.0287	2360.72995	2466.54699
0.00038018	2001.05572	2105.06502	2412.49676	1811.8434	1761.51098	1585.43145
0.00487613	329.671836	250.252484	255.744968	201.910224	183.942551	214.273463
3.76E-07	31.3973177	31.4042333	41.5470165	90.9264585	108.437749	82.634609
8.46E-16	6643.67243	6665.54853	6996.51758	5067.81291	4866.04356	4738.03787
0.00069833	9803.28917	10210.3014	9981.4399	8605.92186	9030.0531	8693.92956
0.0003166	3696.51087	3875.47867	3915.57549	3284.04973	3301.32701	3286.16701
0.00050455	55.4685946	51.0318792	39.7004825	9.36007661	22.4907923	20.1782185
0.01002563	16.7452361	23.553175	26.774744	5.3486152	8.8356684	8.64780792
0.01241338	474.099497	463.212442	430.242438	316.905451	352.623493	409.329575
0.00026533	1285.19687	1301.31292	1290.72731	1108.5005	1054.65751	1011.79353
0.05648743	103.611148	101.082376	101.559374	78.8920743	69.8821046	75.9085362
1.14E-22	886.450937	884.225445	831.863597	409.169063	423.308841	481.394641
3.48E-07	77.446717	52.9946438	60.9356242	141.738303	150.206363	130.677986
1.30E-09	2375.73037	2547.66843	2352.4844	3154.34582	3154.33362	3257.34098
0.00189208	29.3041632	31.4042333	23.0816758	4.0114614	6.42594065	13.4521457
0.00027286	1573.00562	1405.33944	1493.84606	1235.53011	1187.19254	1227.98872
0.00900148	5518.60188	6458.47686	6380.69847	7090.92661	7196.25029	6875.00729
2.07E-103	1636.84683	1612.41111	1656.34106	402.483294	473.913123	458.33382
2.63E-30	2038.7325	1945.0997	1903.77662	1034.95704	1036.98617	1140.54978
0.00038026	74.3069852	91.2685531	87.7103682	144.41261	121.28963	181.603966
1.29E-45	185558.148	177261.195	168008.749	94802.8673	91222.6535	100994.866
4.22E-34	114350.078	111703.877	107415.657	69997.3272	68144.6909	73149.8854
2.50E-16	32897.0629	31373.8105	36304.7063	23281.1848	22616.0981	21016.095
2.42E-11	7323.94764	7879.51842	6672.45085	4708.11853	4970.4651	5375.09305
0.00017506	7953.98715	8436.94356	8933.53182	9967.14443	10395.5655	9748.00126
2.09E-11	462.587148	506.393263	501.333999	722.063052	797.619884	814.815679
1.22E-08	1019.36625	1050.07905	1030.36601	742.120359	737.37669	763.889699
3.85E-05	54.4220174	73.6036719	59.0890902	132.378226	107.634506	128.756251
0.00451641	748.302739	771.366481	793.086382	962.750737	915.696543	887.841613
2.02E-06	401.885667	437.696502	420.0865	617.765056	577.531416	584.207468
0.04800979	191.523638	167.816372	119.101447	106.972304	118.076659	121.069311
2.61E-74	13920.5241	15051.4602	16416.6111	6174.97625	6358.46828	5956.41792
1.84E-05	5222.42051	5220.95379	4935.78556	5943.64864	6126.33117	6409.9474
0.00467935	1789.64711	2071.69802	2247.23196	2563.32384	2592.86705	2288.7865

1.20E-57	534.800978	548.592701	594.58397	135.052534	134.944754	115.304106
1.61E-50	1227.63512	1192.37948	1181.7818	512.129906	436.963964	460.255555
6.00E-52	10377.8601	10743.1919	10526.1675	5880.80242	6177.7387	6156.27837
3.80E-13	1155.42129	1534.8819	1686.80887	2959.12136	2866.77277	2446.36877
3.42E-10	248.03881	232.587603	278.826644	139.063995	114.863689	113.38237
4.07E-104	4781.81149	4626.23612	4749.28562	1758.35725	1739.02019	1548.91848
2.23E-212	168.498938	213.94134	228.046957	2726.4566	2427.39908	2240.74312
7.04E-06	189.430484	166.83499	165.264799	93.6007661	97.995595	99.9302248
1.95E-22	1061.22934	1230.65339	1248.25703	2135.43462	2060.31722	2010.13491
0.01820086	716.905421	690.893133	665.675531	584.336211	591.18654	587.090071
5.03E-12	215.594915	248.28972	237.279628	431.900678	415.276415	421.820853
2.29E-06	1738.36482	1744.89771	1571.40049	1222.15857	1264.30382	1358.66671
2.03E-09	671.902599	686.967604	663.828997	422.540601	420.09587	476.590303
2.20E-07	904.24275	953.903588	825.400728	587.010519	638.577852	651.468197
1.45E-27	9346.98148	9242.65842	9438.55889	6273.92563	6457.26711	6448.3821
5.34E-16	3879.66189	4304.34273	4462.14957	2886.91506	2800.90688	2670.25091
4.55E-07	488.751579	504.430498	486.561727	335.625604	349.410523	297.868939
3.20E-13	13037.2129	13863.9876	16014.99	9499.1406	9356.16959	7959.82675
4.80E-11	5019.38452	4939.29707	4765.90443	3598.28088	3787.28877	3821.37023
1.88E-05	5012.05848	5007.99384	4568.32528	3574.21211	3673.22833	4197.06944
0.00222668	506.543392	464.193824	451.47758	331.614143	360.655919	396.838297
0.00789887	1514.39729	1410.24635	1250.10356	1052.34004	1070.72236	1274.11037
6.99E-185	87.9124896	78.5105833	92.3267034	1115.18627	1132.57204	1044.46302
9.20E-05	5093.69151	5107.11345	5421.42402	6255.20548	6403.44986	5912.21801
1.92E-31	1782.32107	2000.05711	1941.63057	3255.96951	3309.35944	3394.74504
4.86E-07	92.0987986	149.170108	87.7103682	45.4632292	28.9167329	38.4347019
0.04129393	24.0712769	20.6090281	20.3118747	13.371538	6.42594065	9.60867546
6.63E-197	2117.22579	2108.99055	2030.26421	6688.44331	6947.24509	7079.67208
6.23E-18	2293.05077	2346.48506	2460.50665	1517.66956	1561.50358	1620.98355
8.23E-65	230.246997	186.462635	208.65835	922.636123	853.043622	781.185315
0.06959804	10.4657726	13.7393521	12.0024714	4.0114614	6.42594065	1.92173509
1.24E-13	1964.42551	1738.02804	1901.93009	2737.15383	2655.51997	2691.39
1.45E-08	3593.9463	3692.94156	3764.1597	2929.70398	2946.29379	2894.13305
2.25E-28	6792.2864	6237.66585	6001.23572	10198.472	10688.749	11228.6981
2.73E-50	70.1206762	69.6781427	75.7078968	398.471833	375.917528	354.560125
0.01123498	1048.67041	1168.82631	1179.93527	1362.55972	1346.23457	1264.50169
1.75E-145	704.346494	703.651103	709.992349	2409.55115	2388.0402	2411.77754
0.00031385	1153.32814	1184.52843	1362.74214	997.516735	996.824044	905.137229
1.72E-14	2501.31964	2437.75361	2431.88537	1755.68294	1736.61046	1780.48756
0.07018793	2289.91104	2477.0089	2859.358	3146.32289	3026.61805	2602.02932
1.47E-08	5266.37676	5946.19531	6617.9781	9114.04031	9016.39798	7775.34019
5.72E-05	4928.3323	5175.81021	4475.99858	5705.63527	6013.07397	6450.30384
9.22E-10	873.89201	957.829117	884.489818	577.650442	624.119486	628.407375

1.69E-09	1610.6824	1662.4616	1787.44498	2318.62469	2333.4197	2208.07362
3.89E-12	14.6520816	8.83244063	31.3910791	109.646612	103.618293	101.85196
1.83E-07	687.601258	727.204278	653.67306	506.781291	469.89691	449.686012
0.00364169	326.532104	303.247128	269.593974	188.538686	213.662527	245.982092
9.69E-13	2201.99855	2286.62074	2533.44474	3710.6018	3513.38305	3252.53664
2.11E-07	1867.09383	2099.17672	2262.00423	1577.84149	1486.80202	1473.00995
0.00025393	1293.56949	1543.71435	1562.16782	1202.10127	1151.84986	1058.87604
5.61E-13	133.961889	138.374903	108.94551	266.093606	280.331661	306.516747
0.00396639	742.023275	987.270586	985.125925	1230.1815	1216.10927	1055.03257
1.80E-12	317.112909	362.130066	333.299399	740.783206	601.628694	567.87272
6.94E-05	4055.48687	4167.93059	4693.8896	5521.10804	5287.74591	5127.18923
0.0016232	30.3507405	26.4973219	30.4678121	12.0343842	8.8356684	8.64780792
0.0208854	2444.80447	2709.59651	2918.44709	3132.95136	3132.64607	2985.41547
1.86E-14	8169.58207	8724.48857	9649.98704	6018.52926	5972.91184	5318.40187
4.76E-07	838.308383	856.746741	944.502176	635.148055	611.267605	651.468197
0.08509282	964.944231	1066.76255	1223.32882	986.819505	967.907311	880.154673
5.45E-15	5261.14387	6009.00377	6460.09944	10349.5704	10220.4586	8701.6165
3.32E-39	113.030344	124.635551	135.720254	439.9236	421.702355	454.490349
1.25E-07	598.642191	674.209634	653.67306	405.157602	462.667727	421.820853
1.78E-14	1849.30201	1891.12368	1945.32364	2627.50722	2650.70052	2650.07269
2.85E-21	2037.68592	2001.03849	1660.03413	3318.81573	3455.54959	3908.80918
2.02E-12	4256.4297	4131.61945	3556.42461	2382.80807	2542.26277	2787.47675
1.15E-05	10429.1424	10691.1787	11208.4618	12767.1445	13000.4812	12566.2258
0.0002109	3939.3168	3958.89617	3273.9049	2389.49384	2795.28418	3168.94117
2.52E-33	3282.06628	3358.2902	3136.33811	5177.45952	5393.77394	5401.99735
0.00259281	1678.70992	1772.37642	1502.15546	1900.09555	1937.42111	2258.03873
1.38E-19	2626.90891	2532.9477	2519.59574	3686.53303	3767.20771	3651.29668
0.00519931	1688.12912	1956.87629	2008.1058	2302.57885	2374.38507	2100.45646
1.60E-07	121.402962	127.579698	147.722725	50.8118444	61.0464362	61.495523
6.55E-06	2137.11076	2091.32566	2034.88054	2477.74599	2578.40869	2701.95954
1.19E-08	2207.23143	2223.81227	2273.08344	2898.94944	2779.21933	2917.19387
2.42E-50	1173.21311	1098.16678	1131.92538	2380.13377	2248.27599	2367.57763
0.00319188	1936.16793	1957.85767	1578.78663	2087.29708	2210.52358	2603.95105
7.30E-142	826.796033	907.77862	864.177944	73.543459	81.9307433	90.3215494
7.81E-55	1827.32389	1896.03059	1769.9029	689.971361	797.619884	767.73317
0.00085165	2103.62029	2080.53046	1759.74697	1446.80041	1515.71875	1725.71811
6.50E-05	1434.85742	1653.62916	1636.02918	1254.25027	1269.92652	1197.24096
3.37E-10	185.244174	147.207344	161.571731	54.8233058	73.8983175	62.4563905
1.34E-15	3663.0204	3498.62787	3177.88513	2126.07454	2290.84784	2331.06467
1.37E-104	2889.59981	3027.56437	2807.65505	927.984738	1011.28241	1010.83266
2.29E-81	2515.97173	2485.84135	2454.04378	1016.23689	1033.7732	1056.9543
0.01331374	518.055742	581.959699	577.965163	488.061137	465.077455	422.78172
1.03E-24	311.880023	333.669979	337.915734	116.332381	114.863689	113.38237

0.00072594	631.086086	723.278749	707.222548	566.953212	532.549832	509.2598
1.63E-11	3677.67248	3510.40446	3319.14499	2188.92077	2318.15809	2618.36406
1.29E-41	40587.3126	39739.1131	38512.2378	23695.7025	23400.8661	25011.3822
1.23E-26	487.705002	438.677884	420.0865	163.132764	176.713368	154.699675
4.01E-10	944.012686	951.940823	945.425443	589.684826	618.496788	687.981163
1.49E-05	1708.01408	1625.16908	1707.12075	1390.63995	1351.85726	1244.32347
1.09E-15	341.184186	353.297625	345.301871	643.170978	597.612481	609.190024
0.00045317	993.201817	1016.71205	871.56408	1147.27796	1206.47036	1266.42343
4.78E-43	4790.1841	4473.14049	4562.78568	2655.58745	2508.52658	2515.55124
0.00409028	706.439648	744.86916	661.982463	526.838598	552.630896	616.876965
8.15E-14	1008.90048	935.257324	775.544308	421.203447	429.734781	532.320621
0.00397663	628.992931	651.637842	716.455218	788.920743	830.552829	810.011342
0.00642304	515.962588	560.369289	575.195362	899.904508	750.228571	572.677058
1.34E-17	486.658424	465.175206	495.794397	238.013377	248.201958	241.177754
8.38E-40	11.5123498	4.90691146	12.0024714	217.95607	186.352279	191.212642
0.00030862	217.688069	244.364191	256.668235	167.144225	169.484185	129.717119
1.44E-56	145.474239	158.002549	160.648464	660.553978	620.906516	568.833588
0.06588652	3.13973177	0	0.92326703	8.02292281	8.03242582	4.80433773
1.41E-06	1704.87435	1898.97473	1889.92762	2405.53969	2363.13968	2277.25609
0.0001191	1576.14535	1561.37923	1490.15299	1172.68388	1265.10707	1301.01466
9.40E-05	221.874378	199.220605	210.504884	135.052534	144.583665	104.734563
0.06159011	83.7261806	96.1754646	59.0890902	40.114614	59.439951	56.6911852
0.03206331	1180.53915	1179.62151	1287.95751	1101.81473	1098.03261	964.711017
2.41E-32	4719.01685	4934.39016	5125.97857	7796.94381	7845.27029	8102.03515
0.00042932	244.899078	207.071664	264.977639	378.414526	333.345671	322.851496
4.59E-18	528.521515	527.983673	490.254795	260.744991	255.431141	265.199443
0.00051551	244.899078	280.675335	333.299399	201.910224	178.319853	197.938715
3.55E-32	3948.73599	4562.44627	4163.01106	7197.89891	7462.12358	7639.85786
0.00011474	428.050098	453.398619	441.321642	599.044903	610.464362	549.616237
1.14E-38	1656.7318	1623.20631	1772.6727	3337.53589	3306.14647	3028.65451
0.02967555	66.9809444	67.7153781	55.396022	74.8806128	99.6020801	107.617165
4.19E-38	3430.68025	3570.26878	3644.13498	2075.2627	2057.90749	1988.03495
3.70E-08	323.392372	343.483802	300.985053	201.910224	179.123096	186.408304
6.07E-05	103.611148	149.170108	81.247499	45.4632292	48.1945549	54.7694501
4.56E-24	3.13973177	5.88829375	5.5396022	120.343842	109.240991	111.460635
0.0021508	4303.52568	4112.97319	3084.63516	1957.59316	2290.0446	2824.95059
1.02E-09	1706.96751	1769.43227	1570.47722	2299.90454	2246.6695	2471.35133
0.00028754	1395.08748	1300.33154	1114.38331	867.812817	963.087855	1043.50216
1.25E-21	1985.35706	2021.64752	1840.0712	1133.90642	1191.20875	1219.34092
2.35E-06	904.24275	949.978059	950.965045	730.085975	701.230774	680.294223
1.04E-33	240.712769	289.507776	288.059315	34.7659988	55.4237381	30.7477615
2.27E-114	2794.36128	2902.92882	2983.99905	1013.56258	966.300826	996.419646
2.29E-19	841.448115	864.597799	916.804165	494.746906	499.616886	450.646879

8.67E-07	626.899777	745.850542	758.925502	474.689599	514.075252	432.390396
0.01030674	77.446717	99.1196115	106.175709	56.1604596	60.2431936	61.495523
0.01446194	76.4001398	66.7339958	77.5544308	50.8118444	48.1945549	35.5520992
3.45E-11	1580.33166	1441.65059	1572.32376	1056.3515	1097.22937	982.006632
8.41E-15	4253.28997	4374.02087	4978.25585	7672.58851	7574.57754	6499.30808
1.05E-09	2435.38528	2321.9505	2685.7838	3675.8358	3867.61303	3249.65404
2.69E-21	400.839089	351.33486	409.007296	894.555893	817.700948	735.063673
0.00757596	1960.2392	2074.64216	2250.92503	2508.50053	2455.51257	2337.79074
3.78E-13	2243.86164	2308.21115	1877.00188	1168.67242	1216.10927	1416.31876
0.02788538	3730.00134	3660.55595	4246.10509	4800.38215	4674.87183	4009.70027
0.00743357	49.1891311	41.2180563	35.0841473	26.743076	16.8680942	15.3738807
6.50E-09	2192.57935	2221.84951	2361.71707	2955.1099	2947.90027	2888.36784
0.02706027	4874.95686	5146.36874	5381.72354	5706.97242	5795.39523	5563.42309
0.00176333	4.18630903	4.90691146	2.7698011	21.3944608	12.0486387	24.0216887
0.00032953	54.4220174	57.9015552	69.2450275	132.378226	122.896115	89.3606818
0.00340074	118.26323	126.598316	126.487584	80.2292281	85.9469562	73.0259335
0.00137749	77.446717	84.3988771	58.1658231	45.4632292	36.1459162	26.9042913
6.25E-17	368.395194	329.74445	284.366246	112.320919	110.044234	142.208397
2.45E-10	1555.2138	1679.1451	1843.76427	2575.35822	2623.39027	2294.5517
0.02336143	469.913188	500.504969	539.187948	417.191986	430.538024	407.40784
6.99E-07	116.170076	67.7153781	64.6286924	30.7545374	16.8680942	24.0216887
1.46E-110	2735.75295	2670.34122	2476.20218	795.606512	835.372285	843.641706
0.00209487	2.09315451	2.94414688	5.5396022	18.7201532	20.8843071	13.4521457
2.34E-82	1466.25474	1460.29685	1435.68024	453.295138	463.47097	506.377197
0.00421337	556.779101	582.941081	722.918087	811.652357	806.455552	754.281024
7.84E-08	3166.94278	3033.45266	3554.57808	2441.64284	2453.90609	2130.24335
0.00199381	9.41919531	9.81382292	4.61633517	21.3944608	28.1134904	28.8260264
0.00055768	7340.69288	7268.11725	7392.59914	6489.2074	6331.15803	6536.78192
0.07743715	2685.51724	2534.91046	2546.37048	2281.18438	2361.53319	2451.17311
9.07E-24	12911.6236	12703.9938	12814.9464	8674.11671	9034.87256	9172.4416
9.42E-20	341.184186	305.209893	392.388489	141.738303	118.879902	113.38237
1.64E-16	244.899078	226.699309	232.663293	93.6007661	73.0950749	90.3215494
1.86E-20	43.9562448	52.9946438	73.8613627	225.978992	208.843071	232.529946
0.00714714	23.0246997	8.83244063	16.6188066	42.7889216	37.7524013	33.6303641
0.01176763	1658.82495	1731.15836	1782.82864	1969.62755	1937.42111	1962.09153
9.99E-07	5056.01473	4808.77323	5067.81275	4137.15386	4098.94689	4033.72196
6.45E-13	4461.55885	4687.08183	4692.04307	3484.62281	3474.02417	3448.55362
0.00027641	6612.27511	6831.40213	6507.18605	5825.97911	5677.31857	5819.01386
0.00704714	4456.32596	4544.78139	4133.46651	3738.68203	3856.36763	3955.89169
0.00272248	7408.7204	7227.88058	5759.33976	4793.69638	5058.82178	6127.45234
1.09E-21	1017.27309	1081.48329	1094.07144	1712.89402	1701.26779	1754.54414
2.07E-30	2083.73532	2341.57815	2341.4052	1232.8558	1247.43573	1226.06699
0.0053754	790.165829	773.329246	790.316581	600.382057	661.871887	682.215958

0.01393147	34.5370495	31.4042333	23.0816758	8.02292281	14.4583665	14.4130132
1.30E-11	1879.65275	1978.4667	1847.45733	1155.30088	1281.17192	1384.61013
7.77E-16	119.309807	124.635551	107.098976	271.442222	273.102478	292.103734
1.33E-18	3116.70707	3224.82221	2915.67729	1995.03347	2020.15509	2090.84778
3.57E-18	5995.8411	5653.74338	5764.87936	4137.15386	4079.66907	4194.18684
0.02334213	7389.88201	6789.20269	7010.36659	6281.94856	6451.64442	6457.02991
2.94E-07	854.007042	826.32389	854.945273	625.787979	625.725971	598.620481
3.17E-88	628.992931	600.605963	625.975049	86.9149971	101.208565	101.85196
1.05E-27	54095.4853	55545.2563	54205.9308	36512.3217	38256.8377	38322.2804
7.67E-10	10513.9151	10036.5967	9104.33622	6760.64962	6823.54573	7664.84042
0.03372001	546.313328	534.853349	634.284452	498.758368	494.79743	435.272999
2.00E-12	491.891311	527.983673	494.87113	298.185298	285.954359	294.025469
7.88E-31	1181.58572	1220.83957	1202.09368	613.753595	589.580055	647.624726
1.22E-05	805.864488	880.299916	916.804165	1199.42696	1076.34506	1171.29754
2.31E-23	1513.35071	1477.96173	1697.88808	2808.02298	2751.10584	2554.94681
2.00E-06	249.085387	296.377452	230.816758	375.740218	409.653717	472.746833
0.00015882	1319.73392	1267.94592	1231.63822	1531.0411	1513.30902	1678.6356
1.62E-54	852.960464	888.150974	914.95763	304.871067	317.28082	307.477615
1.33E-08	65.9343672	64.7712313	67.3984935	17.3829994	19.277822	12.4912781
0.00010984	13.6055043	34.3483802	19.3886077	0	1.60648516	4.80433773
2.07E-12	3543.71059	3295.48174	3379.15734	2421.58553	2517.36225	2545.33813
0.00081029	4929.37888	5221.93517	4974.56278	4187.9657	4458.79957	4421.91245
0.0025273	260.597737	300.302981	289.905849	458.643754	347.000795	366.090535
0.02108988	1652.54549	1706.62381	1713.58361	1466.85772	1491.62147	1544.11415
1.34E-06	6.27946354	1.96276458	2.7698011	29.4173836	35.3426736	27.8651588
1.81E-06	12263.7923	11976.7895	13242.4191	16485.7692	15963.6431	14706.0778
1.36E-50	1167.98022	1200.23054	1088.53183	2377.45946	2412.13747	2543.4164
5.29E-14	2618.5363	2697.81992	2950.76144	1957.59316	1825.77039	1779.5267
8.53E-13	18284.7513	17799.3306	18027.7121	14326.2658	14050.3192	14302.5134
5.56E-07	5536.39369	5298.48299	5982.77038	4581.08892	4374.4591	4177.85209
0.0004724	13310.3696	13723.65	13982.8792	16072.5887	15377.276	15389.2546
0.00014413	4560.98369	4432.90381	3770.62257	3211.84343	3251.52597	3598.44896
0.00010658	9581.41479	9167.09199	8819.96997	10411.0795	10935.3445	11370.9065
0.00108437	5003.68587	4935.37155	4714.20147	5464.94758	5575.30676	5787.30523
9.83E-05	2164.32177	2245.40268	2193.68247	2660.93606	2611.34163	2573.20329
1.86E-08	1651.49891	1655.59193	1768.97964	2317.28754	2260.32462	2141.77376
0.03798024	4810.06907	5438.82066	5673.47592	6308.69163	6323.1256	5524.98839
0.00120426	2415.50031	2436.77223	2658.08579	2233.04685	2075.57883	1908.28295
4.18E-06	114.076921	134.449374	111.715311	46.800383	61.0464362	59.5737879
0.0007613	1856.62805	1937.24864	1793.90785	1406.6858	1488.4085	1661.33999
8.97E-15	8084.80931	9035.58676	10023.9102	14621.7768	14833.4808	13314.7416
0.03869772	3397.18978	3432.87526	3774.31563	4127.79378	4115.01175	3685.88791
0.00761735	338.044454	364.09283	350.841473	288.825221	281.938146	253.669032

8.10E-36	946.10584	882.26268	900.185358	1761.03156	1694.0386	1702.65729
2.19E-08	312.9266	281.656718	334.222666	490.735445	472.306638	515.985872
2.56E-06	20294.1796	19136.9547	19274.1226	23023.1141	23057.8815	23969.8018
3.36E-60	1277.87083	1185.50981	1303.65305	426.552062	398.40832	463.138157
1.58E-07	3856.63719	3933.38023	3699.531	2787.96567	2941.47433	3156.44989
0.0883382	126.635848	105.989288	112.638578	136.389688	140.567452	164.30835
4.81E-11	2082.68874	2153.15275	2417.11309	1482.90357	1598.45274	1476.85342
6.44E-06	4222.93923	4183.63271	4444.6075	5232.28282	5319.87562	5013.80686
6.58E-170	2687.6104	2606.55137	2635.00411	7323.59137	7588.23267	7419.81919
0.03033258	150.707125	149.170108	131.103919	105.63515	89.9631691	118.186708
0.00048222	178.964711	182.537106	177.26727	120.343842	125.305843	82.634609
3.90E-20	1292.52291	1283.64804	1294.42038	817.000972	716.492383	754.281024
3.53E-06	513.869433	505.41188	594.58397	399.808986	351.017008	338.225376
2.94E-15	2037.68592	1917.621	2367.25667	3888.44325	3683.67048	3225.63235
1.53E-09	80.5864488	79.4919656	67.3984935	184.527225	164.664729	167.190953
4.52E-05	2152.80942	2045.2007	2240.76909	2734.47952	2674.7978	2508.82516
1.54E-21	1108.32532	1178.64013	1232.56149	1980.32478	1930.99517	1864.08304
4.78E-32	948.198995	1008.861	968.507118	1841.26078	1801.67311	1715.14857
0.08287093	20.9315451	26.4973219	24.9282099	32.0916912	40.9653717	44.1999071
0.00232222	929.360604	834.174948	861.408143	1020.24835	1052.24778	1094.42814
3.18E-11	4728.43605	4310.23103	4057.75861	3074.11659	3114.97473	3170.8629
0.05326839	1497.65205	1358.23309	1395.05649	1227.50719	1209.68333	1333.68415
3.68E-30	1087.39377	1095.22264	1075.60609	1837.24932	1898.86546	1986.11322
4.14E-09	746.209584	803.752097	547.497351	1096.46612	1227.35466	1415.3579
8.12E-10	392.466471	605.512874	635.207719	1107.16335	1125.34286	948.376268
2.39E-06	12596.6039	13300.6742	11829.8205	9763.89705	10168.2478	10480.1823
0.04843522	5108.34359	4645.86377	4287.6521	4087.67917	4008.98372	4375.79081
1.11E-38	6379.93496	6668.49267	6240.36188	3865.71164	3901.34922	3880.94402
0.02020557	0	0	0	4.0114614	2.40972774	5.76520528
3.03E-16	2956.58075	2999.10428	2618.38531	4170.5827	4359.19749	4572.76865
0.00087146	1278.91741	1306.21983	1334.12086	1509.64664	1567.12628	1567.17497
2.18E-19	2124.55183	2102.12087	1986.87066	1243.55303	1282.7784	1376.92319
0.00011846	1444.27661	1628.11322	1626.79651	1892.07263	1974.37027	1933.2655
3.09E-09	293.041632	366.055595	327.759797	562.94175	555.040624	510.220667
7.80E-08	8601.81847	8310.34525	7409.21795	6063.99249	6006.64803	6433.96909
3.08E-14	3976.99358	4126.71254	3656.13745	5569.24558	5544.78354	6126.49148
2.06E-14	2871.80799	2677.21089	2379.25915	3900.47764	4113.40526	4419.99071
4.76E-34	1270.54479	1267.94592	1253.79663	615.090748	649.020006	662.03774
9.60E-40	3699.6506	3635.04001	3530.57314	2031.13662	1951.87947	2108.1434
0.00318556	1429.62453	1344.49374	1371.05155	1580.51579	1652.26999	1596.00099
3.42E-30	107.797457	154.07702	120.024714	414.517678	434.554237	419.899118
2.64E-09	825.749456	871.467475	875.257148	599.044903	592.793025	593.816144
1.05E-07	316.066332	311.098187	342.53207	164.469918	203.220373	196.977847

3.96E-06	598.642191	589.810757	553.96022	383.763141	396.801835	441.999071
7.41E-43	707.486226	759.589894	807.858655	286.150913	261.857082	255.590767
2.31E-177	4436.44099	4512.39578	4517.5456	1267.6218	1364.70915	1348.09717
8.58E-05	5318.70562	5205.25168	6244.97822	7901.24181	7602.69103	6438.77343
0.06695294	7.3260408	17.6648813	9.23267034	6.685769	1.60648516	2.88260264
4.19E-08	1582.42481	1653.62916	1450.45251	1060.36296	1106.06503	1195.31923
1.19E-13	1647.3126	1678.16372	1704.35094	2364.08792	2297.27378	2332.02554
0.00081764	502.357083	587.847993	606.586441	754.154744	772.719364	681.25509
0.00010705	721.09173	755.664365	685.064139	524.16429	539.779015	582.285733
5.53E-15	261.644314	205.108899	211.428151	92.2636123	75.5048027	68.2215958
0.05415653	1324.96681	1233.59754	1323.96493	1152.62658	1199.24117	1104.03681
6.16E-14	1307.17499	1394.54424	1507.69507	2271.82431	2224.17871	2017.82185
2.36E-10	3107.28788	2786.14433	3017.23667	2191.59508	2125.37987	2232.09531
8.27E-07	45.002822	24.5345573	24.0049429	76.2177666	89.9631691	119.147576
0.05808406	1795.92657	2046.18208	2499.28386	2666.28468	2581.62166	2254.19526
1.16E-08	1418.11218	1634.00152	1460.60845	1021.5855	1061.08345	1114.60635
4.44E-62	757.721934	803.752097	846.63587	243.361992	224.10468	216.195198
0.06539252	328.625259	283.619482	270.517241	235.339069	261.857082	220.038668
0	1484.04655	1423.00432	1460.60845	7715.37743	7764.94604	7876.23128
1.53E-05	3111.47418	3300.38865	3278.52124	2533.90645	2576.8022	2772.10287
0.0089615	814.237106	676.172399	615.819112	815.663819	865.09226	965.671884
1.68E-10	152.80028	158.002549	142.183123	291.499529	276.315448	298.829807
0.02465777	3854.54404	3950.06372	4677.27079	3765.4251	3698.12885	3181.43245
0.00130895	941.919531	911.704149	815.244791	1038.9685	1089.19694	1196.2801
0.00503317	114.076921	87.343024	82.170766	58.8347672	60.2431936	49.0042449
0.00460568	9686.07251	9644.04378	10317.5091	11559.6946	11512.8759	10632.9603
0.0013277	2099.43398	2306.24839	2310.93739	2698.37637	2832.23334	2502.09909
0.03563904	2105.71344	2042.25655	1729.27915	2106.01724	2269.16029	2432.91663
3.20E-290	272.110087	201.18337	235.433094	2698.37637	2754.31881	2692.35087
1.27E-21	4926.23915	5105.15068	4684.65693	7176.50445	7372.16041	7816.65749
0.00241578	2199.90539	2133.5251	2535.29127	2814.70875	2849.90468	2607.79452
0.0452025	180.011288	191.369547	192.96281	167.144225	136.551239	137.404059
2.72E-09	1578.2385	1472.07344	1292.57385	1992.35916	2153.49336	2425.22969
0.01000903	1565.67958	1615.35525	1765.28657	2100.66862	1945.45353	1787.21364
1.27E-05	1103.09243	1161.95663	1134.69518	1528.36679	1519.73496	1346.17543
8.50E-20	797.49187	743.887777	648.133458	324.928374	349.410523	371.85574
0.00037271	1042.39095	1195.32363	1238.10109	1581.85295	1539.01279	1344.2537
0.00120146	2053.38458	2251.29098	1959.17265	2400.19107	2466.75797	2818.22451
3.43E-24	4346.43535	4626.23612	4425.21889	2975.16721	2961.5554	2964.27638
0.00051959	536.894133	464.193824	505.950335	397.134679	334.148914	388.190489
1.16E-18	4214.56661	4207.18589	3971.89478	5907.54549	5874.91624	6349.41275
0.0002617	488.751579	471.0635	478.252323	374.403064	371.098073	320.929761
3.61E-64	372.581503	348.390714	396.081557	29.4173836	30.5232181	45.1607747

0.03342246	3044.49324	3040.32234	3303.44945	2686.34199	2839.46253	2889.32871
2.68E-16	6911.5962	7588.04788	8419.27208	13021.2037	12867.9462	11350.7283
5.54E-61	63103.3757	63410.054	62930.8043	35719.3895	37461.6275	37291.2695
1.23E-66	9707.00406	10573.4128	10839.155	21858.4532	22215.2801	20868.1214
2.89E-14	25763.5923	26426.6624	23488.8366	16834.7664	17586.9963	18594.7088
1.78E-06	6815.3111	6698.91552	6453.63657	5483.66774	5435.54255	5638.37076
0.00075384	5409.75784	5251.37664	5104.74343	4733.52446	4359.19749	4493.01665
2.17E-15	10422.8629	9938.45847	8448.81663	5559.8855	5821.90223	6363.82576
0.01205706	2610.16368	2371.01962	2969.22678	2484.43176	2106.10205	1831.41354
0.07520813	4469.93146	4305.32411	3756.77356	4506.20831	4489.32279	5070.49804
3.27E-09	5328.12481	5168.94053	5362.33493	4248.13763	4241.92407	4163.43908
9.84E-25	1196.2378	1210.04437	1180.85854	1893.40978	1970.35405	1949.60025
0.00053157	971.223694	996.103026	1013.7472	846.418356	718.902111	810.972209
5.28E-09	4177.93641	3741.0293	3390.23655	5157.40221	5189.75032	5672.96199
0.01129519	2940.88209	2803.80921	2522.36554	2222.34962	2245.06302	2559.75114
0.0077552	9809.56863	9147.46434	8223.53947	7504.10713	7627.59156	8215.41752
5.38E-18	3129.266	3094.29837	2561.14275	1521.68103	1604.87868	1787.21364
3.39E-09	4075.37184	4122.78701	3684.75873	5063.80144	5317.46589	5675.8446
2.83E-06	6339.11845	6572.31721	6559.81227	7628.46243	7891.05512	7681.17517
0.00322748	3548.94348	3351.42053	3406.85535	3019.29328	2879.62466	3095.91523
2.40E-19	1336.47916	1455.38994	1585.2495	846.418356	847.420924	769.654905
4.56E-13	315.019754	321.893392	361.920677	180.515763	146.993392	142.208397
0.00270412	766.094552	784.124451	842.942802	632.473748	695.608076	596.698746
1.96E-21	1082.16088	983.345056	1047.90808	560.267443	596.809238	524.63368
0.0266245	1229.72828	1343.51236	1205.78675	1335.81665	1454.67232	1597.92273
0.00108524	412.351439	355.26039	368.383546	279.465144	285.151116	281.534191
0.01525896	2850.87645	2670.34122	2220.45722	2705.06214	3178.4309	3710.87046
0.06223741	1196.2378	1238.50445	1315.65552	1433.42887	1467.5242	1315.42767
0.08944598	2167.4615	2103.10225	2002.5662	1838.58648	1922.96274	1958.24806
2.34E-09	2678.1912	2991.25323	3170.49899	2035.14808	2132.60905	1719.95291
2.09E-23	859.239928	929.36903	975.893255	490.735445	445.799633	477.551171
0.00937873	1111.46505	1126.62687	1239.02436	1428.08026	1400.05182	1272.18863
4.32E-25	4590.28785	4229.75768	5202.60974	9574.02121	8973.82612	8099.15255
2.06E-07	658.297095	678.135164	715.531951	1017.57404	929.351667	915.706772
1.93E-07	1238.10089	1242.42998	1455.99211	2033.81093	1956.69893	1682.47907
0.00495009	3561.50241	3519.2369	3503.79839	3941.9294	3904.56219	4030.83936
0.03071717	1854.5349	1906.82579	1916.70236	2164.852	2118.15069	2039.9218
0.00812119	573.524337	572.145876	577.041896	469.340984	477.126093	478.512038
3.00E-06	213.50176	196.276458	212.351418	319.579758	334.148914	310.360218
0.02308307	5538.48684	5638.04127	5465.74084	4990.25798	5057.21529	5143.52398
1.97E-14	32490.9909	34170.75	34064.8605	44138.1098	43899.6168	43058.3965
7.97E-12	9662.00124	10034.6339	10072.8433	12452.9133	12754.689	12683.4516
0.00038776	1648.35918	1821.44553	1886.23455	2195.60654	2166.34524	2127.36075

1.51E-10	71.1672535	63.789849	75.7078968	18.7201532	12.8518813	11.5304106
1.03E-13	27.2110087	44.1622031	32.3143462	151.098379	152.616091	111.460635
2.44E-06	1793.83342	1727.23283	1412.59856	1093.79181	1158.2758	1240.48
7.42E-25	1571.95904	1533.90052	1723.73955	903.915969	917.303028	839.798236
0.00017953	77.446717	73.6036719	81.247499	44.1260754	32.9329458	34.5912317
0.00010019	1609.63582	1830.27797	1896.39049	2261.12708	2306.10945	2139.85203
0.01619478	558.872255	501.486351	517.029539	415.854832	436.963964	449.686012
5.19E-16	2575.62663	2428.92117	2336.78886	3429.7995	3669.21211	3609.0185
2.19E-67	2066.99008	2233.6261	1936.09097	660.553978	696.411318	769.654905
0.00543416	3113.56734	3023.63884	3453.94197	2782.61706	2839.46253	2584.7337
6.61E-23	4125.60755	4167.93059	4266.41696	2846.80044	2841.06901	2872.0331
7.96E-14	2242.81506	2689.96886	2916.60056	4466.09369	4604.98972	4020.26981
4.04E-21	3901.64001	3919.64087	3267.44203	1736.96279	1904.48816	2159.06938
1.54E-25	2427.01266	2526.07802	2508.51653	3786.81956	3812.99253	3743.53996
0.00297323	761.908243	830.249419	832.786864	1056.3515	932.564637	969.515354
4.80E-18	521.195474	478.914558	544.72755	254.059222	261.857082	228.686476
1.83E-11	416.537748	405.310887	433.012239	236.676223	236.153319	242.138622
1.01E-15	1008.90048	1074.61361	1019.28681	1671.44225	1612.9111	1512.40552
9.66E-08	520.148897	500.504969	537.341414	731.423129	769.506393	717.768057
3.77E-07	5681.86793	5550.69824	5789.80757	4669.34107	4731.90205	4605.43815
1.08E-06	418.630903	433.770973	388.695421	565.616058	587.97357	669.72468
2.15E-07	2813.19967	2772.40497	2709.78874	3625.02395	3550.33221	3318.83651
0.00062991	737.836966	860.67227	919.573966	1239.54157	1146.22716	972.397957
0.00321624	335.951299	308.15404	356.381075	468.00383	459.454757	388.190489
1.26E-09	1263.21875	1300.33154	1297.19018	937.344814	898.828449	954.141474
0.06685406	193.616793	174.686048	209.581617	167.144225	142.97718	149.895337
3.12E-06	1029.83202	1153.12419	1173.4724	1440.11464	1499.6539	1449.94913
0.00784077	348.510227	363.111448	378.539484	283.476606	300.412726	273.847251
1.67E-22	2239.67533	1993.18743	2204.76168	1309.07357	1210.48657	1129.01937
3.81E-30	5199.39581	5632.15297	5570.99328	8647.37363	8476.61896	8573.82112
5.17E-05	669.809444	638.879872	649.979992	486.723984	466.68394	519.829343
0.0698463	5808.50378	6125.78827	6049.24561	6653.67731	6558.47568	6270.62161
1.36E-06	1919.42269	1841.07318	1756.97717	1387.96565	1367.11887	1497.03164
0.04450166	2.09315451	8.83244063	8.3094033	0	0	1.92173509
3.85E-05	609.107964	714.446308	683.217605	469.340984	522.107678	391.073091
0.04365463	363.162308	414.143327	461.633517	357.020065	334.952157	329.577568
0.00028659	19371.0984	19188.968	18479.1897	21181.8534	21828.1172	22405.5094
0.00028444	1160.65418	1155.08696	1335.04413	973.447967	999.233772	870.545997
7.55E-06	1991.63652	1953.93214	1612.02424	1112.51196	1269.92652	1467.24474
2.47E-08	1151.23498	1113.8689	1261.18277	1642.02487	1671.54781	1540.27068
5.52E-13	2812.15309	2769.46083	2546.37048	3657.11565	3839.49954	3893.4353
3.90E-05	964.944231	948.996676	790.316581	591.02198	637.77461	711.041984
1.77E-08	905.289327	875.393004	929.729903	1389.3028	1352.66051	1164.57147

3.39E-25	2292.00419	2688.0061	2618.38531	1357.21111	1450.6561	1417.27963
0.00068221	1179.49257	1121.71996	1070.06649	846.418356	925.335454	948.376268
6.21E-14	627.946354	626.121902	613.972577	267.43076	354.229978	352.63839
0.0520513	50.2357083	46.1249677	60.0123572	70.8691514	68.2756194	94.1650196
1.32E-05	54.4220174	71.6409073	54.472755	112.320919	123.699358	127.795384
4.32E-07	139.194775	170.760519	163.418265	298.185298	257.037626	269.042913
0.00432694	867.612546	772.347864	790.316581	956.064968	991.201346	962.789282
1.87E-14	318.159486	344.465184	264.977639	548.233058	653.036219	680.294223
6.68E-19	1368.92305	1457.3527	1543.70248	862.464202	908.46736	856.132984
2.62E-10	1859.76779	1835.18489	1738.51182	1280.99334	1302.85947	1362.51018
4.21E-38	5457.90039	5803.89487	6128.64657	10532.7605	10304.7991	9871.95317
1.31E-08	4914.7268	4838.2147	4253.49122	3258.64381	3191.28278	3645.53147
0.07867978	2360.03171	2491.72964	2687.63034	3066.09367	2947.09703	2513.6295
0.00440961	14.6520816	8.83244063	12.9257385	1.3371538	0.80324258	2.88260264
0.00011104	535.847556	476.951794	478.252323	675.262669	618.496788	700.472441
6.00E-08	2258.51372	2065.80972	1864.07614	2741.16529	2800.90688	3050.75446
9.33E-21	826.796033	849.877065	908.494761	1393.31426	1633.79541	1578.70538
6.95E-05	440.609025	424.938532	440.398375	288.825221	274.708963	345.912317
0.0657378	81.633026	120.710022	75.7078968	92.2636123	142.97718	165.269218
1.90E-18	2728.42691	2647.76942	2690.40014	1870.67817	1853.88388	1822.76574
0.04619826	1.04657726	4.90691146	0	1.3371538	21.6875497	10.569543
5.84E-13	660.390249	692.855898	678.60127	402.483294	432.144509	375.699211
1.11E-07	2830.99148	3389.69444	3680.1424	2377.45946	2315.74836	1979.38715
3.40E-17	549.45306	556.443759	502.257266	882.521509	922.122484	961.828414
1.02E-133	9900.62085	9984.58344	9651.83357	3900.47764	3826.64766	4054.86105
3.05E-83	3143.91808	2894.09638	3030.1624	6608.21408	6512.69085	6760.66406
1.45E-08	188.383906	185.481253	134.796987	69.5319976	79.5210156	59.5737879
0.00608277	64.8877899	40.236674	48.9331528	21.3944608	25.7037626	27.8651588
0.00030318	4677.15376	4753.81582	3941.42697	5249.66582	5499.80196	6233.14777
3.97E-05	1032.97175	991.196115	998.974931	803.629434	753.441542	812.893944
1.85E-05	254.318273	294.414688	308.371189	463.992369	431.341266	387.229621
5.09E-13	1297.7558	1137.42208	1164.23973	1834.57501	1865.93252	1719.95291
3.92E-11	2693.88986	2837.17621	2370.94974	1545.74979	1621.74677	1881.37866
1.21E-27	8925.21085	8824.58957	9003.70011	13209.7424	13173.9816	12677.6864
4.81E-05	3270.55393	3200.28765	3748.46416	4734.86161	4167.22251	4173.04775
1.13E-09	3744.65343	3549.65975	3728.15228	2787.96567	2885.24735	2856.65922
7.24E-07	263.737469	287.545012	307.447922	427.889216	421.702355	468.903363
2.90E-06	1155.42129	1306.21983	1474.45745	1013.56258	878.747384	808.089607
0.03330108	1154.37471	1165.88216	1295.34365	1351.86249	1404.87128	1354.82324
2.51E-10	4029.32244	4282.75232	5061.34988	7180.51591	7076.56714	6140.90449
0.00346769	1531.14253	1594.74622	1666.497	1914.80424	1862.71955	1808.35272
0.00656767	23362.7441	22650.3033	24243.1458	21569.628	20959.0087	20572.1742
4.68E-27	6946.13325	6349.54343	5411.26808	2559.31237	2604.11245	3213.14108

9.82E-10	1859.76779	1988.28052	2000.71966	2600.76414	2673.99455	2525.15991
2.73E-32	3028.79458	3039.34096	2922.14016	1801.14617	1837.01578	1788.1745
0.00970729	55.4685946	44.1622031	86.7871012	32.0916912	35.3426736	28.8260264
3.04E-18	1090.5335	1108.96199	1019.28681	538.872982	640.184338	618.7987
1.17E-39	1016.22652	968.624322	1017.44027	1978.98763	1892.43952	1885.22213
8.47E-10	19.8849679	31.4042333	28.621278	84.2406895	115.666932	89.3606818
3.39E-33	1008.90048	1182.56566	1049.75462	2138.10893	2054.69452	2167.71718
1.53E-19	1281.01056	1200.23054	1246.4105	591.02198	675.527011	745.633216
0.01016302	2666.67885	2963.77452	3283.13757	2444.31715	2706.9275	2308.96471
0.00485635	1435.904	1265.98316	1346.12334	1101.81473	1171.93093	1142.47151
2.91E-34	2384.10299	2228.71918	2401.41755	4121.10801	3881.26815	4018.34808
4.02E-45	865.519391	868.523328	921.4205	362.36868	334.952157	324.773231
1.95E-19	348.510227	337.595508	301.90832	114.995227	106.831263	140.286662
1.77E-42	2608.07052	2395.55417	2401.41755	1219.48427	1218.519	1294.28859
2.96E-05	621.666891	610.419786	631.514651	780.89782	822.520404	814.815679
1.88E-07	1161.70076	1097.1854	1128.23232	774.212051	793.603671	885.919878
1.01E-16	1639.98656	1624.18769	1726.50935	2663.61037	2439.44772	2433.8775
5.27E-06	6979.62373	6958.98183	6895.88148	8004.20265	8299.10235	8281.71738
0.00093119	267.923778	276.749806	287.136048	200.57307	200.810645	198.899582
0.08350921	750.395893	751.738836	654.596327	735.43459	828.143102	934.924123
0.00553499	1046.57726	1112.88752	1016.517	927.984738	902.844662	834.993898
6.37E-24	182.104443	203.146134	132.027186	22.7316146	24.0972774	28.8260264
0.08979874	39.7699358	34.3483802	15.6955396	44.1260754	44.178342	59.5737879
2.36E-20	1285.19687	1367.06553	1480.92032	2303.916	2275.58623	2184.05193
1.04E-14	721.09173	771.366481	734.920559	1199.42696	1152.6531	1118.44982
2.44E-18	2668.77201	2824.41824	2820.58079	4193.31432	4051.55558	3907.84831
2.42E-12	75.3535625	80.4733479	113.561845	247.373453	226.514408	220.999536
6.95E-06	4878.09659	4669.41694	4579.40449	5534.47958	5644.38562	5881.47025
2.40E-08	3019.37539	3003.02981	3094.7911	3927.22071	3711.78397	3875.17881
5.00E-05	613.294273	612.38255	558.576555	759.503359	767.899908	808.089607
2.23E-06	690.74099	685.986222	649.056725	508.118444	474.716366	486.198979
0.02485821	5546.85946	5326.94308	5161.06272	5876.79095	5802.62441	5969.87007
1.36E-11	194.66337	233.568985	180.960339	375.740218	384.753197	427.586058
0.00302148	263.737469	248.28972	263.131105	204.584532	181.532823	185.447436
0.0129611	254.318273	242.401426	298.215252	324.928374	346.197553	344.951449
4.29E-05	9920.50582	10837.4046	7948.40589	6352.81771	6564.09838	7777.26192
2.12E-23	5043.4558	4750.87167	5414.96115	3169.05451	3221.80599	3158.37163
0.00061956	323.392372	380.776329	410.85383	512.129906	506.042826	486.198979
1.24E-07	450.02822	485.784234	436.705307	288.825221	273.102478	315.164555
2.17E-09	1466.25474	1530.95638	1453.22231	1982.99909	1929.38868	2109.10426
0.00034404	174.778402	178.611577	147.722725	94.9379199	83.5372285	119.147576
5.57E-07	51.2822856	48.0877323	65.5519594	129.703919	125.305843	117.225841
4.18E-44	1147.04867	1122.70134	1275.03177	2440.30569	2547.08223	2396.40366

0.08717088	273.156664	286.563629	253.898434	196.561609	225.711165	245.982092
7.88E-07	1904.77061	1857.75668	1500.30893	1101.81473	1162.29202	1325.99721
1.56E-07	867.612546	937.220089	1029.44274	1430.75457	1405.67452	1228.94959
9.51E-11	701.206762	779.21754	667.522065	1033.61989	1104.45855	1198.20183
5.11E-17	2005.24202	2013.79646	1863.15287	2905.63521	2817.77498	2892.21131
5.40E-11	218.734647	266.935983	294.522184	486.723984	453.832059	522.711945
1.08E-20	2578.76636	2416.1632	2322.93986	3663.80141	3746.3234	3924.18306
1.50E-33	807.957642	783.143069	855.86854	1607.25887	1557.48737	1522.01419
0.00045943	1205.657	1150.18005	797.702717	550.907366	711.672927	815.776547
2.41E-14	1036.11148	980.40091	1064.52689	679.274131	609.661119	565.950985
7.34E-07	7756.18405	7589.02926	7184.86406	8991.02216	9133.6714	9577.9277
2.06E-13	178.964711	179.592959	108.022243	38.7774602	42.5718568	33.6303641
0.01168909	270.016932	279.693953	300.061786	216.618916	232.137106	208.508258
7.03E-215	2213.5109	2275.82553	2121.66764	316.905451	352.623493	362.247065
0.01239595	248.03881	225.717927	263.131105	200.57307	184.745794	175.838761
1.24E-65	974.363426	942.127	816.168058	212.607454	188.762007	236.373416
0.01447382	103.611148	138.374903	135.720254	70.8691514	90.7664117	90.3215494
6.78E-44	576.664069	650.656459	694.296809	1480.22926	1506.88308	1506.64031
0.00027539	251.178542	210.997193	313.910791	399.808986	416.8829	356.48186
1.48E-10	841.448115	805.714862	934.346238	1323.78226	1266.71355	1233.75393
2.35E-33	1765.57583	1608.48558	1672.95987	907.927431	801.636096	804.246136
0.00457669	1002.62101	1053.0232	971.27692	1172.68388	1208.88009	1177.06274
5.50E-06	633.17924	580.978317	616.742379	438.586447	456.241786	423.742588
1.41E-33	7889.09936	7485.98412	7220.87147	12057.1158	11949.8399	11998.3531
7.57E-10	148.61397	161.928078	151.415794	260.744991	319.690547	301.71241
5.88E-25	2282.585	2412.23767	2361.71707	1410.69726	1339.80863	1483.57949
5.24E-07	371.534926	316.005098	318.527127	217.95607	193.581462	203.70392
0.00141077	1271.59137	1245.37413	1073.75956	1366.57118	1494.0312	1661.33999
6.61E-21	206.17572	231.606221	259.438036	577.650442	564.679535	491.964184
0.00074448	1507.07125	1519.17979	1551.08862	1803.82048	1800.86987	1778.56583
0.00169589	1050.76357	996.103026	1112.53678	1382.61703	1286.79462	1198.20183
2.12E-06	1431.71769	1280.70389	1324.88819	1056.3515	1011.28241	1015.637
0.01355962	65.9343672	50.0504969	41.5470165	24.0687684	26.5070052	32.6694966
1.20E-43	14207.2863	14114.2401	15786.0197	7898.5675	7716.75148	7111.38071
0.05320281	50.2357083	53.9760261	61.8588913	26.743076	39.3588865	37.4738343
9.47E-08	897.963286	1013.76791	953.734846	1260.93603	1298.84325	1342.33196
0.04885033	645.738168	669.302723	665.675531	550.907366	595.202753	572.677058
0.0112195	2.09315451	0	7.38613627	21.3944608	20.8843071	7.68694037
0.01774642	233.386728	238.475897	277.903377	207.258839	190.368492	178.721364
2.58E-09	974.363426	963.717411	854.945273	601.71921	651.429734	622.64217
1.04E-49	7075.90883	6759.76123	5659.62692	2012.41647	2314.94512	2541.49466
7.00E-20	785.97952	796.882421	807.858655	1370.58265	1326.1535	1257.77562
0.06656241	288.855323	331.707215	358.227609	303.533913	258.644111	240.216887

1.60E-11	475.146075	489.709764	518.876073	278.127991	304.428938	247.903827
2.84E-10	3985.36619	4602.68295	4643.10991	3254.63235	3059.55099	3137.23254
0.03025079	1196.2378	1352.3448	1544.62575	1240.87873	1147.03041	1051.1891
2.95E-06	2773.42973	2915.68679	3128.02871	2394.84246	2326.99376	2195.58234
2.37E-20	416.537748	459.286913	477.329056	870.487124	845.011196	813.854812
0.00026469	371.534926	336.614126	305.601388	189.87584	245.79223	244.060357
1.08E-16	6175.85239	6243.55414	6384.39154	8660.74517	8638.07072	8272.10871
0.0784402	2706.44879	2569.25884	2859.358	3088.82528	3004.12726	2857.62008
5.52E-07	15.6986589	13.7393521	9.23267034	53.486152	47.3913123	54.7694501
9.74E-76	5249.63152	4665.49142	4486.15452	11902.006	12713.7236	13482.8934
0.04744889	613.294273	506.393263	496.717664	611.079287	608.054634	715.846322
8.27E-23	507.58997	522.095379	422.856301	962.750737	975.136494	1087.70206
0.0022877	2636.32811	2586.92372	2360.79381	2864.18344	2920.59003	3137.23254
9.55E-11	2821.57228	2783.20018	2642.39025	1937.53586	1971.1573	2118.71294
2.69E-14	2288.86446	2260.12342	2262.9275	3044.6992	3163.16929	3122.81953
3.60E-51	186.290752	182.537106	182.806873	593.696288	640.98758	606.307422
1.39E-07	1378.34225	1345.47512	1257.4897	911.938892	967.104068	1029.08914
1.22E-13	1573.00562	1409.26497	1416.29163	917.287507	873.927929	998.341381
8.92E-06	2944.02182	2961.81176	2134.59338	1524.35533	1680.38348	2053.37395
1.17E-15	2528.53065	2439.71638	2334.94233	1707.5454	1665.12187	1652.69218
0.03010621	1660.91811	1700.73551	1529.85347	1763.70586	1800.06663	2069.7087
2.37E-08	15.6986589	10.7952052	22.1584088	69.5319976	64.2594065	66.2998607
7.43E-12	4253.28997	4239.5715	3851.87006	2697.03922	2826.61064	3080.54135
1.35E-156	81.633026	81.4547302	96.9430385	1006.87681	934.974365	927.237182
0.00023959	259.55116	260.066307	225.277156	324.928374	352.623493	378.581813
2.97E-20	2424.9195	2386.72173	2117.05131	1329.13088	1404.87128	1428.81004
8.56E-43	591.31615	602.568727	539.187948	1341.16526	1295.63028	1263.54082
0.00873461	200.942833	202.164752	194.809344	112.320919	155.025818	154.699675
3.71E-29	641.551858	611.401168	703.52948	270.105068	266.676537	220.038668
0.06352274	387.233585	348.390714	419.163233	485.38683	443.389905	441.038204
5.25E-12	522.242051	518.16985	506.873602	780.89782	796.816641	804.246136
2.27E-09	237.573037	274.787042	210.504884	106.972304	117.273417	122.991046
4.39E-07	1378.34225	1502.49629	1447.68271	1010.88827	1050.6413	1151.11932
1.15E-20	1742.55113	1844.01733	1872.38554	1165.99811	1132.57204	1137.66718
9.90E-16	1487.18628	1410.24635	1658.18759	926.647584	956.661915	854.211249
6.66E-07	1032.97175	1078.53914	887.259619	574.976134	673.117283	739.868011
5.08E-07	190.477061	218.848251	219.737554	382.425987	342.18134	315.164555
9.83E-35	378.860967	417.087474	404.390961	938.681968	908.46736	863.819924
0.07146207	156.986589	162.90946	175.420736	135.052534	133.338269	122.991046
0.00044682	41.8630903	40.236674	15.6955396	6.685769	7.22918323	9.60867546
1.94E-08	61.7480582	46.1249677	45.2400847	9.36007661	8.8356684	9.60867546
0.02506405	24.0712769	18.6462635	16.6188066	5.3486152	10.4421536	5.76520528
0.02979726	84.7727578	79.4919656	66.4752264	33.428845	63.4561639	42.278172

6.16E-14	4310.85172	4427.01552	4447.3773	5792.55027	5962.46968	5752.714
1.56E-17	371.534926	331.707215	348.071672	149.761226	146.19015	162.386615
3.78E-13	302.460827	280.675335	314.834059	131.041072	147.796635	144.130132
1.22E-05	11.5123498	11.7765875	7.38613627	44.1260754	39.3588865	37.4738343
5.49E-20	3561.50241	3566.34325	3699.531	5256.35159	5113.44227	5073.38065
0.00282855	34.5370495	40.236674	51.7029539	22.7316146	17.6713368	13.4521457
1.53E-07	11258.0316	11337.9096	12522.2708	9547.27814	9152.94922	8737.1686
0.00019911	1053.9033	914.648296	1108.84371	782.234973	818.504191	769.654905
0.00081029	204.082565	178.611577	183.73014	128.366765	115.666932	131.638854
7.55E-23	12595.5573	12231.9489	11698.7166	8290.35357	8095.07874	8520.01253
4.95E-13	16670.9291	16321.3689	16244.8835	12712.3212	12941.8445	13000.5379
3.42E-13	55626.6278	55171.3497	53077.6985	40854.0601	42165.4161	43229.4309
2.39E-10	25350.1943	26374.6491	30411.4928	20073.3529	19627.2325	18127.7271
4.20E-09	815.283683	748.794689	908.494761	537.535828	519.69795	554.420574
0.01270908	1269.49821	1423.98571	1542.77921	1268.95896	1138.19474	1161.68886
5.59E-35	2239.67533	2131.56234	2262.00423	1248.90165	1227.35466	1167.45407
1.64E-22	692.834144	720.334602	655.519594	1262.27319	1176.75038	1334.64502
0.0002392	925.174295	904.834473	765.388371	627.125133	665.8881	680.294223
1.90E-19	371.534926	332.688597	324.989996	633.810902	685.165922	686.059428
0.00901103	1817.9047	1771.39504	1684.03907	1932.18724	2025.77779	2078.3565
1.66E-09	2368.40433	2312.13668	2423.57596	3044.6992	3225.01897	3024.81104
3.43E-10	2003.14887	2072.6794	2602.68977	3983.38117	3841.90927	3172.78464
6.06E-12	5199.39581	5354.42178	5178.60479	3844.31718	3962.39566	4109.6305
4.55E-10	11350.1304	11249.5852	12551.8153	16081.9488	15865.6475	14894.4078
3.83E-28	11211.9822	11551.851	11125.3678	7296.84829	7610.72346	7708.07946
5.81E-05	617.480582	673.228252	601.046839	409.169063	481.142306	483.316376
1.02E-40	1132.39659	1144.29175	1352.5862	493.409753	420.09587	422.78172
0.002386	1698.59489	1844.99871	1944.40037	2134.09747	2162.32903	2169.63892
1.76E-06	25.1178542	27.4787042	26.774744	1.3371538	1.60648516	2.88260264
2.11E-10	12057.6166	11033.6811	11797.5062	9044.50831	9048.52768	9017.74192
3.47E-07	10681.3675	10539.0644	11901.8353	14731.4234	14435.0724	13403.1414
2.77E-06	148.61397	125.616933	122.794515	48.1375368	66.6691343	69.1824633
5.16E-17	1917.32953	1779.2461	1769.9029	1071.06019	1196.0282	1178.02361
1.64E-23	1261.12559	1369.0283	1410.75203	747.468975	673.920526	761.007097
0.00101838	19.8849679	36.3111448	24.9282099	8.02292281	8.8356684	4.80433773
6.58E-05	731.557503	743.887777	763.541837	597.707749	570.302233	563.068382
0.00135836	29.3041632	24.5345573	28.621278	52.1489982	58.6367085	60.5346554
1.06E-34	3772.91101	3565.36187	3670.90973	2176.88639	2130.19933	2232.09531
0.00034181	1309.26815	1260.09486	1180.85854	1437.44034	1572.74897	1831.41354
3.69E-05	3858.73035	3920.62226	3797.39731	4524.92846	4513.42007	4557.39477
4.47E-48	3586.62026	3451.52152	3460.40484	6050.62095	5978.53453	6008.30477
1.07E-14	3542.66401	3606.57992	4066.99128	2492.45468	2569.57302	2508.82516
0.07330203	340.137608	314.042333	276.98011	255.396376	253.021413	263.277708

5.38E-11	409.211707	414.143327	481.945392	244.699146	251.414928	218.116933
2.04E-05	1336.47916	1366.08415	1355.35601	1593.88733	1710.10346	1765.11368
0.00180707	1058.08961	1001.99132	1102.38084	1214.13565	1278.76219	1294.28859
3.83E-25	984.829199	923.480737	1024.82641	517.478521	514.075252	488.120714
5.11E-38	1959.19262	1878.36571	2087.50676	960.076429	877.140899	775.42011
1.16E-25	36504.6147	36947.0805	40493.5688	24999.4275	24494.8825	23442.2855
1.27E-14	103.611148	85.3802594	90.4801693	219.293223	260.250596	232.529946
0.05548096	576.664069	628.084667	560.423089	687.297054	731.753992	631.289978
2.99E-08	1206.70358	1178.64013	1459.68518	1969.62755	1881.99737	1764.15282
7.53E-07	755.628779	770.385099	726.611156	536.198674	529.336861	564.02925
0.01406321	299.321095	312.079569	302.831587	365.042988	371.901315	394.916562
0.02277136	196.756524	207.071664	239.126162	254.059222	272.299235	298.829807
6.78E-05	106.75088	111.877581	120.024714	231.327608	158.238789	202.743052
0.00011896	4252.24339	3796.96809	4046.67941	4689.39838	4831.50413	5006.11992
9.24E-07	802.724756	868.523328	842.942802	1109.83765	1089.19694	1129.01937
2.95E-26	3422.30763	3332.77426	3823.24879	2056.54255	2043.44913	1870.80911
5.68E-10	682.368372	646.73093	687.83394	455.969446	420.899113	441.038204
1.24E-10	1900.5843	1856.7753	1779.13557	1236.86727	1388.00318	1291.40598
0.00140216	2568.30059	2685.06195	2647.92985	2963.13282	3006.53698	3215.06281
5.20E-05	724.231462	790.012745	837.4032	607.067826	589.580055	586.129203
1.08E-08	4692.85242	4719.46744	4849.92173	6160.26756	5832.34439	5881.47025
0.00200141	1038.20464	950.959441	1051.60115	862.464202	852.240379	763.889699
0.00025609	762.95482	781.180304	693.373542	554.918827	563.07305	608.229157
7.18E-08	1961.28578	1979.44808	1945.32364	1506.97233	1549.45494	1532.58374
3.09E-06	805.864488	789.031363	662.90573	398.471833	462.667727	568.833588
4.25E-17	857.146773	804.733479	740.460161	386.437448	413.66993	465.059892
1.57E-11	1117.74451	996.103026	917.727432	604.393518	618.496788	658.194269
5.99E-28	1429.62453	1358.23309	1443.06637	810.315203	748.622086	753.320156
1.71E-27	488.751579	490.691146	521.645874	1000.19104	979.95595	947.415401
0.07494191	62.7946354	70.659525	48.0098858	36.1031526	46.5880697	35.5520992
0.00017294	990.062085	948.015294	917.727432	731.423129	769.506393	767.73317
1.79E-08	797.49187	793.938274	878.950216	574.976134	569.49899	567.87272
0.01810145	35.5836267	50.0504969	76.6311638	29.4173836	32.9329458	20.1782185
8.41E-24	161.172898	134.449374	154.185595	402.483294	432.947751	372.816608
0.0782489	467.820034	492.653911	480.098858	415.854832	432.947751	397.799164
0.00019693	104.657726	110.896199	101.559374	56.1604596	61.0464362	54.7694501
0.00013225	1806.39235	1706.62381	1539.08615	1996.37062	2124.57663	2228.25184
5.35E-10	330.718413	293.433305	336.992467	165.807071	163.861487	185.447436
3.77E-26	436.422716	583.922464	568.732493	1254.25027	1208.07684	1164.57147
0.00093425	2.09315451	7.85105833	9.23267034	17.3829994	32.1297033	30.7477615
0.00831279	45.002822	40.236674	48.9331528	66.85769	75.5048027	82.634609
0.00080574	82.6796033	82.4361125	83.094033	40.114614	43.3750994	49.0042449
4.09E-07	980.64289	947.033912	714.608684	509.455598	531.746589	605.346554

0.04237011	11.5123498	10.7952052	16.6188066	28.0802298	25.7037626	25.9434238
0.08293356	62.7946354	67.7153781	59.0890902	114.995227	72.2918323	83.5954765
9.45E-50	635.272395	580.978317	574.272095	1402.67434	1364.70915	1394.21881
1.59E-52	1413.92587	1315.05227	1421.83123	596.370595	562.269807	567.87272
0.00895703	4.18630903	2.94414688	6.46286924	14.7086918	22.4907923	13.4521457
0.07700835	1019.36625	873.43024	936.192772	873.161432	796.013398	825.385222
0.01253733	1297.7558	1306.21983	1323.96493	1497.61226	1502.06363	1468.20561
3.39E-28	6115.15091	6617.46079	6579.20088	10028.6535	9817.23083	9877.71838
1.69E-12	701.206762	718.371838	637.97752	1059.02581	1050.6413	1224.14525
0.02590868	373.628081	373.906653	262.207838	219.293223	266.676537	272.886383
0.00208976	14.6520816	23.553175	14.7722725	52.1489982	46.5880697	32.6694966
5.68E-07	5144.9738	5132.62939	5789.80757	4276.21786	4233.89165	4045.25237
2.39E-05	1134.48975	1145.27313	960.197715	766.189128	827.339859	818.65915
0.00140568	518.055742	507.374645	526.262209	419.866293	405.637504	397.799164
1.07E-05	595.502459	595.699051	576.118629	411.843371	457.848272	392.994827
3.84E-19	4284.68729	4207.18589	4113.15464	2894.93798	2981.63646	2961.39378
2.69E-08	2671.91174	2623.23487	2725.48428	3326.83866	3364.78317	3381.2929
1.64E-17	521.195474	442.603414	372.999882	127.029611	172.697155	181.603966
2.99E-10	981.689467	1020.63758	1092.2249	1592.55018	1477.16311	1414.39703
1.33E-19	1433.81084	1503.47767	1337.81393	2223.68677	2228.19492	2263.80394
0.00887388	2247.00137	2357.28027	2547.29375	2753.19968	2885.24735	2627.97274
0.03005756	852.960464	774.310628	757.078968	620.439364	651.429734	739.868011
0.0005893	751.44247	812.584538	845.712603	1020.24835	1034.57645	951.258871
5.42E-07	1992.6831	1955.89491	1790.21478	1355.87395	1400.05182	1537.38807
0	6351.67737	6249.44243	6495.18358	123.01815	85.1437137	120.108443
0.0343549	64.8877899	51.0318792	55.396022	84.2406895	93.979382	75.9085362
3.59E-21	546.313328	527.983673	610.279509	267.43076	260.250596	259.434238
2.15E-18	4967.05566	5233.71176	5722.40908	3535.43465	3486.87605	3345.7408
0.00025859	9266.39503	9319.20624	8607.61856	7123.0183	7380.99608	8123.17424
6.58E-30	940.872954	895.02065	971.27692	1902.76986	1714.92291	1701.69642
4.28E-19	2459.45655	2361.20579	2556.52642	1667.43079	1568.73276	1546.03588
1.90E-06	7.3260408	6.86967604	9.23267034	48.1375368	31.3264607	40.356437
3.25E-06	639.458704	631.028814	588.121101	850.429817	795.210156	885.919878
4.80E-10	1354.27097	1387.67456	1409.82876	992.16812	992.807831	1023.32394
7.16E-27	2111.9929	2182.59422	2225.07355	1353.19965	1289.20434	1270.2669
7.97E-29	1655.68522	1816.53862	1668.34353	2884.24075	2950.31	2842.2462
0.0902659	5778.15304	5860.81505	6829.40625	6006.49487	5421.08418	4991.7069
2.20E-07	2144.4368	1986.31776	2195.52901	2865.5206	2795.28418	2603.95105
0.0012995	1136.5829	1203.17469	1285.18771	1450.81187	1469.13068	1432.65351
7.03E-11	1196.2378	1283.64804	1383.97728	2071.25124	2060.31722	1759.34848
1.84E-05	30.3507405	31.4042333	30.4678121	8.02292281	4.81945549	5.76520528
0.00048058	2658.30623	2485.84135	2506.67	2041.83385	2228.19492	2152.3433
2.16E-12	1589.75085	1387.67456	1336.89066	804.966588	868.305231	963.750149

0.02050461	9200.46067	9144.5202	8453.43296	9622.15875	9799.5595	10523.4214
1.59E-09	9105.22214	9405.56789	8418.34881	11483.4768	11623.7234	12480.7086
0.00234476	12227.1621	11944.4039	11553.7637	12942.3116	13594.8807	14032.5096
1.25E-13	102.564571	83.4174948	86.7871012	248.710607	208.843071	228.686476
8.95E-08	13746.7923	13435.1236	12038.4789	16202.2926	17147.6226	18292.0355
6.78E-09	9952.94971	10274.0912	8457.12603	12815.282	13209.3243	14707.9995
4.46E-20	103.611148	94.2127	94.1732374	262.082145	270.69275	273.847251
2.61E-07	30.3507405	23.553175	41.5470165	2.6743076	2.40972774	1.92173509
4.98E-60	632.132663	579.996934	723.841354	1957.59316	1955.89569	1781.44843
0.00907317	3194.15379	3087.42869	3108.6401	2826.74313	2822.59443	2720.21602
7.08E-19	5035.08318	5691.03591	5507.28786	8041.64296	8070.98146	7907.93991
4.20E-22	192.570215	229.643456	192.039543	49.4746906	54.6204955	51.8868475
1.84E-27	1090.5335	957.829117	1009.13087	1888.06117	1817.73796	1793.93971
3.54E-63	9529.08592	10613.6495	11081.9742	4657.30669	4772.86742	4571.80779
1.14E-24	7359.53127	7813.76581	7445.22536	4952.81768	4940.74512	5187.72388
4.05E-07	9977.02099	9962.01164	10221.4893	12042.4071	12100.0462	11826.3578
4.84E-21	2039.77907	2152.17137	2192.75921	1353.19965	1362.29942	1257.77562
9.94E-05	20778.7449	20494.2064	20439.2856	17857.689	17775.7583	18366.9832
1.57E-36	22141.3884	21383.3388	22534.1785	14463.9927	14104.1365	14070.9444
2.55E-06	12377.8692	11378.1463	10046.9919	7973.44811	8376.21364	9069.62877
7.13E-13	9278.95396	8534.10041	8250.31421	6224.45094	6340.79694	6417.63434
6.56E-08	17044.5572	16371.4194	13632.961	9838.77767	10482.3157	12070.4181
3.73E-39	4114.0952	4069.79236	3917.42202	2333.33338	2400.08883	2366.61677
4.84E-21	3232.87715	3016.76917	2928.60303	4578.41461	4570.45029	4653.48153
7.17E-15	650.971054	702.669721	747.846297	414.517678	397.605078	360.32533
0.07246379	4458.41911	4379.90917	4142.69918	4642.598	4596.95729	4905.22882
0.00019872	230.246997	278.712571	302.831587	446.609369	398.40832	356.48186
9.38E-06	4711.69081	4467.25219	4303.34764	5428.84443	5359.2345	5943.92664
7.56E-08	1169.0268	1163.9194	1118.07638	1466.85772	1636.20514	1523.93593
1.94E-10	8090.0422	7921.71786	7391.67587	5541.16535	5589.76513	6153.39577
0.04187207	813.190529	831.230801	778.314109	750.143282	698.821046	654.350799
3.70E-11	4005.25116	3778.32182	3265.5955	5237.63144	5430.72309	6110.15673
0.01418829	587.129841	605.512874	730.304224	544.221597	524.517406	493.885919
1.04E-36	395.606203	494.616675	491.178062	131.041072	118.076659	124.912781
3.20E-15	4165.37748	4020.72325	3848.177	5563.89697	5604.22349	6112.07846
2.78E-31	3627.43677	3639.94692	3244.36036	6084.04979	6053.2361	6666.49904
1.06E-35	6847.75499	6878.50848	7820.07178	14137.7271	13444.6743	12729.5733
2.94E-07	1336.47916	1351.36342	1232.56149	957.402121	951.842459	1013.71526
0.0046275	3089.49606	2942.18411	3186.19453	3530.08603	3509.36684	3442.78842
0.06208178	3410.79528	3569.2874	3385.62021	3576.88642	3835.48333	3973.1873
7.69E-14	1317.64077	1370.00968	1383.05402	893.218739	912.483573	782.146183
7.89E-09	1209.84331	1293.46186	1276.87831	914.6132	905.254389	938.767593
0.02188662	2147.57653	2184.55698	1997.02659	2287.87015	2368.76237	2491.52955

3.57E-32	1273.68452	1210.04437	1193.78427	640.496671	628.135699	604.385687
4.95E-30	4688.66611	4734.18818	4541.55054	2944.41267	3018.58562	2989.25894
2.60E-17	17174.3328	17279.198	19988.7313	31472.589	30330.4399	26788.0263
2.00E-70	399.792512	474.989029	468.096386	56.1604596	33.7361884	36.5129668
7.25E-06	35.5836267	52.0132615	42.4702836	105.63515	103.618293	88.3998143
4.50E-10	1007.8539	927.406266	989.74226	683.285592	645.003793	675.489885
0.0083192	1006.80732	971.568469	996.205129	1135.24358	1188.79902	1129.98023
7.40E-16	974.363426	1041.24661	998.051664	1520.34387	1510.09605	1655.57478
0.00217899	5105.20386	5282.78088	5539.6022	4439.35062	4609.80918	4788.96385
6.79E-05	1774.99503	1733.12113	1668.34353	1355.87395	1373.54481	1468.20561
0.00379549	72.2138307	67.7153781	77.5544308	131.041072	122.092872	97.0476222
4.46E-14	1567.77273	1744.89771	1765.28657	2563.32384	2629.81621	2402.16887
1.93E-07	861.333082	874.411622	902.031892	649.856747	629.742184	520.79021
4.97E-09	133.961889	120.710022	115.408379	216.618916	249.0052	255.590767
2.55E-05	2194.67251	2143.33893	1879.77168	1585.86441	1621.74677	1665.18346
2.53E-15	6601.80934	6408.42637	5811.96598	9057.87985	9034.87256	10067.0093
7.12E-09	1424.39165	1495.62661	1200.24714	693.982823	754.244784	975.28056
5.31E-16	495.031043	469.100736	524.415675	909.264585	816.094463	849.406911
0.00441517	1694.40858	1571.19305	1612.02424	1267.6218	1437.80422	1413.43616
8.66E-81	1376.24909	1417.11603	1621.25691	4908.6916	4546.35301	4204.75638
3.40E-11	1696.50173	1878.36571	1738.51182	2433.61992	2424.18611	2646.22922
0.00042137	419.67748	368.999742	452.400847	592.359134	542.188743	534.242356
3.82E-35	106.75088	97.1568469	104.329175	351.67145	405.637504	363.207933
0.00038063	106.75088	124.635551	82.170766	199.235916	159.845274	171.034423
4.78E-249	1314.50103	1233.59754	1302.72978	5196.17967	5262.04215	5297.26278
1.79E-20	835.168651	869.504711	786.623513	427.889216	402.424533	466.02076
2.11E-18	932.500336	982.363674	1010.9774	1544.41264	1591.22355	1506.64031
1.86E-54	1553.12065	1553.52817	1458.76191	617.765056	635.364882	666.842077
3.11E-78	1673.47703	1704.66104	1890.85089	457.3066	417.686142	326.694966
1.23E-17	1514.39729	1511.32873	1600.02177	2257.11562	2308.51918	2281.09956
6.16E-14	2918.90397	2801.84644	2852.89513	2107.35439	2055.49777	1950.56112
2.41E-34	7137.65689	6948.18663	7307.65857	11256.1607	11236.5605	10858.7641
1.82E-09	2437.47843	2545.70567	2554.67988	3181.08889	3277.22973	3259.26272
5.72E-05	94.1919531	67.7153781	63.7054253	33.428845	33.7361884	24.9825562
0.02702348	0	0.98138229	0	4.0114614	4.01621291	7.68694037
5.22E-21	5108.34359	4990.32895	4337.50852	7946.70504	8170.58354	9453.01492
1.84E-36	2477.24837	2340.59677	2023.80134	954.727814	1019.31484	1069.44558
7.30E-256	1447.41635	1569.23028	1457.83865	6268.57702	6470.11899	6526.21238
2.07E-24	2687.6104	2894.09638	2774.41744	4209.36017	4376.06558	4572.76865
7.33E-05	76.4001398	92.2499354	77.5544308	41.4517678	37.7524013	35.5520992
1.74E-13	4933.56519	4659.60312	4750.20889	6430.37263	6250.03053	6361.90403
8.90E-09	74.3069852	92.2499354	99.7128396	184.527225	191.171734	190.251774
6.02E-21	1193.09807	1189.43534	1215.01942	688.634207	655.445947	545.772766

1.65E-58	1328.10654	1455.38994	1601.8683	462.655215	474.716366	391.073091
5.45E-14	5399.29207	5671.40826	6641.98304	3816.23695	3842.71251	3356.31034
0.00022061	888.544091	767.440952	842.942802	627.125133	626.529214	677.41162
2.85E-19	6693.90814	6308.32537	5915.37189	3715.95041	3866.00655	4252.79976
0.00048222	582.943532	647.712313	746.92303	518.815675	433.750994	500.611992
0.00162681	625.8532	544.667172	494.87113	653.868209	751.835056	760.046229
0.01362927	1947.68028	1918.60238	2107.81864	1750.33433	1792.0342	1690.16601
0.09022948	1327.05996	1376.87936	1098.68777	930.659045	1056.26399	1248.16694
0.0083451	1175.30626	1294.44324	1322.11839	1441.4518	1552.66791	1444.18392
1.02E-16	3427.54052	3447.59599	3366.23161	2302.57885	2450.69312	2438.68183
0.03172436	1153.32814	1057.93011	774.621041	603.056364	774.325849	913.785037
0.00015271	952.385304	1084.42743	1088.53183	1370.58265	1318.12108	1264.50169
1.94E-10	861.333082	720.334602	733.997292	447.946523	429.734781	510.220667
0.02202126	1499.74521	1590.8207	1684.96234	1421.39449	1444.23016	1258.73649
4.19E-07	1029.83202	1113.8689	1124.53925	1528.36679	1484.39229	1370.19712
0.0299313	619.573736	669.302723	675.831469	625.787979	514.075252	503.494594
0.00216855	5479.87852	5202.30753	5887.67387	4926.0746	4741.54096	4529.52961
0.00309353	407.118553	389.60877	429.319171	260.744991	344.591068	308.438482
0.02528321	509.683124	542.704407	544.72755	500.095522	391.98238	424.703456
0.00209478	697.020453	675.191017	658.289395	799.617973	822.520404	842.680838
3.51E-07	907.382482	955.866352	927.883369	695.319976	701.230774	644.742124
1.41E-13	361.069154	399.422593	402.544427	683.285592	665.8881	633.211713
0.00029593	1007.8539	1007.87961	1164.23973	1408.02295	1428.16531	1240.48
1.07E-07	1822.091	1745.8791	1949.01671	1453.48618	1340.61187	1292.36685
2.12E-08	1224.49539	1185.50981	1287.03425	910.601738	910.877088	886.880745
1.95E-08	70.1206762	84.3988771	68.3217605	22.7316146	22.4907923	17.2956158
6.21E-11	1392.99433	1516.23564	1353.50947	858.45274	851.437137	1023.32394
5.79E-05	1283.10372	1115.83167	1163.31646	895.893047	920.515999	955.102341
1.24E-13	469.913188	487.746999	513.336471	260.744991	263.463567	284.416794
5.70E-18	7669.31814	7143.4817	7190.40366	5160.07652	5213.8476	5269.39762
5.71E-05	90.0056441	74.5850542	76.6311638	32.0916912	37.7524013	38.4347019
1.31E-15	87.9124896	79.4919656	87.7103682	8.02292281	7.22918323	15.3738807
1.15E-09	4307.71199	4606.60848	4421.52582	5579.94281	5669.28614	5770.97048
2.04E-05	514.91601	497.560822	426.54937	296.848144	280.331661	366.090535
1.96E-25	246.992233	252.215249	315.757326	74.8806128	71.4885898	64.3781256
6.50E-10	597.595614	595.699051	574.272095	998.853889	916.499786	819.620017
2.54E-207	160.12632	134.449374	136.643521	1461.5091	1374.34806	1423.04484
5.54E-19	257.458005	290.489158	291.752383	574.976134	538.975772	580.363998
0.04527299	2041.87223	1872.47741	1488.30646	1393.31426	1493.22796	1641.16177
0.0260819	4584.00839	4322.00761	4022.67447	4594.46046	4838.73331	5246.3368
2.34E-37	814.237106	839.08186	875.257148	1635.3391	1615.32083	1620.02268
7.19E-06	499.217352	458.30553	433.935506	280.802298	311.658122	341.107979
2.10E-06	1787.55395	1768.45089	2328.47946	2969.81859	2943.08082	2598.18585

0.01304106	25.1178542	19.6276458	27.698011	5.3486152	8.8356684	12.4912781
0.0026659	541.080442	605.512874	657.366128	776.886358	804.045824	703.355044
2.88E-11	1161.70076	1233.59754	1024.82641	1619.29325	1807.29581	1912.12642
3.17E-08	3966.5278	3852.90688	3674.60279	2945.74982	2926.21272	3100.71957
2.81E-09	1025.64571	1175.69599	1232.56149	1822.54063	1685.20294	1577.74451
0.05327635	3294.6252	3088.41007	2918.44709	2659.59891	2821.79119	2877.7983
1.42E-17	2855.06276	2810.67888	2553.75662	4014.13571	4082.0788	4323.90396
4.35E-06	3522.77905	3199.30627	2722.71448	1894.74694	2207.31061	2449.25138
0.00030365	2319.2152	2269.93724	2508.51653	2860.17198	2839.46253	2765.3768
0.01162152	1294.61607	1193.36087	1086.6853	1341.16526	1389.60967	1497.03164
0.00027363	8555.76908	7439.85915	7432.29962	9120.72608	9347.33392	10125.6222
8.43E-33	241.759346	264.973219	230.816758	617.765056	609.661119	622.64217
0.01955262	1782.32107	1874.44018	1965.63551	2150.14331	2052.2848	2163.87371
2.50E-47	5429.64281	5779.36032	5653.16405	3131.6142	3222.60924	3167.01943
3.65E-12	58.6083264	68.6967604	98.7895726	12.0343842	11.2453961	9.60867546
0.00188376	329.671836	308.15404	322.220195	246.036299	248.201958	220.038668
1.12E-13	1345.89835	1397.48838	1311.96245	906.590277	856.256592	930.119785
1.87E-15	5140.78749	5196.41924	5758.41649	3757.40218	3635.47592	3363.99728
0.00020321	3353.23353	3137.47919	3525.9568	2840.11467	2806.52958	2672.17265
2.69E-08	143.381084	118.747257	116.331646	50.8118444	56.2269807	39.3955694
5.47E-07	971.223694	911.704149	773.697774	588.347672	619.30003	611.11176
0.08236207	12.5589271	24.5345573	11.0792044	8.02292281	8.03242582	3.84347019
3.69E-34	1472.5342	1395.52562	1335.04413	699.331438	701.230774	718.728925
1.41E-26	3264.27446	3021.67608	2872.28374	4918.05168	5107.01633	5342.42356
0.00108929	3792.79598	3758.69418	3586.89243	4222.7317	4214.61383	4349.84738
2.52E-05	1837.78966	1958.83905	1540.93268	2235.72116	2367.95913	2557.82941
0.04885431	2754.59134	2600.66307	2158.59832	1948.23309	2096.46314	2369.49937
0.04594274	132.915312	125.616933	110.792044	144.41261	180.729581	159.504013
0.04800979	934.59349	952.922205	829.093796	672.588362	793.603671	838.837368
7.04E-14	583.990109	608.457021	620.435447	968.099352	933.36788	936.845858
9.42E-16	1661.96468	1520.16117	1556.62822	1051.00289	1033.7732	965.671884
6.61E-12	3795.93571	3617.37513	3317.29845	4861.89122	5146.37522	5612.42734
6.37E-32	2210.37117	2095.25119	2205.68494	3522.06311	3552.74194	3531.18823
0.03648836	2541.08958	2434.80947	2935.0659	2465.71161	2305.30621	2011.09577
1.39E-05	6741.00411	6561.522	6569.04495	5653.48627	5572.09379	5690.25761
0.00085714	6631.1135	6336.78546	6326.22572	5471.63335	5752.02013	5619.15341
0.00033589	12444.8502	12769.7464	12443.7931	11052.9133	10935.3445	11190.2634
0.0135011	10095.2842	10546.9155	11154.9123	12093.219	12258.285	11347.8457
0.00020353	805.864488	751.738836	703.52948	616.427902	517.288223	576.520528
3.54E-06	119.309807	134.449374	145.876191	217.95607	236.956562	224.843006
5.58E-05	2767.15027	2752.77733	3087.40496	2440.30569	2256.30841	2057.21742
1.03E-11	1672.43046	1634.00152	1569.55396	1000.19104	1095.62288	1177.06274
2.67E-55	1829.41705	1790.0413	1860.38307	845.081202	832.159315	850.367779

9.44E-08	425.956944	389.60877	375.769683	260.744991	244.185745	241.177754
7.13E-10	3028.79458	3323.94182	3603.51123	2344.03061	2441.05421	2204.23015
1.78E-05	491.891311	465.175206	513.336471	375.740218	319.690547	341.107979
8.79E-12	780.746634	711.502162	738.613627	1135.24358	1127.75258	1068.48471
1.04E-25	1455.78896	1600.63452	1660.03413	2953.77275	2767.97394	2635.65968
8.51E-18	809.00422	802.770715	791.239848	462.655215	474.716366	454.490349
1.37E-09	317.112909	307.172657	324.989996	496.08406	514.075252	495.807654
1.04E-23	270.016932	302.265746	288.059315	100.286535	92.3728969	86.4780792
3.17E-17	707.486226	704.632486	636.130986	370.391603	360.655919	383.386151
2.11E-10	281.529282	245.345573	240.972696	108.309458	126.912328	132.599721
0.04273174	7393.02174	7288.72628	6543.19347	7476.0269	7952.9048	8205.80885
0.00085819	65.9343672	46.1249677	58.1658231	22.7316146	20.8843071	30.7477615
7.62E-10	26885.5232	26309.8779	22527.7156	33884.8145	35878.4364	40343.9457
0.00020822	332.811568	326.800303	352.688007	248.710607	242.57926	231.569079
1.08E-16	10680.3209	12152.4569	12271.1421	17340.2105	18162.9213	16931.447
0.0001191	32.443895	24.5345573	48.9331528	89.5893047	90.7664117	70.1433309
0.05326839	1729.99221	1687.97754	1640.64552	1743.64856	1907.70113	2012.05664
0.00137917	4277.36125	4387.76023	4869.31034	5459.59897	5613.05916	5030.14161
0.00937315	73.260408	108.933434	84.0173001	60.171921	46.5880697	54.7694501
1.44E-05	2567.25401	2754.74009	2606.38284	3186.43751	3172.00495	3244.8497
0.00055045	836.215228	877.355769	890.029421	1060.36296	1078.75479	1040.61955
1.11E-12	680.275217	623.177755	623.205248	405.157602	379.130499	359.364462
2.45E-08	2052.338	1890.14229	1702.50441	1270.29611	1245.026	1412.47529
0.00764874	3765.58497	3892.16217	3547.19194	4034.19302	4267.62784	4711.13358
0.04519492	1689.17569	1717.41901	1756.97717	2003.05639	1961.51838	1804.50925
9.76E-12	565.151719	585.885228	551.190419	350.334296	334.148914	349.755787
2.79E-05	450.02822	397.459828	451.47758	611.079287	622.513001	560.18578
1.91E-60	5219.28078	5423.11854	5048.42414	2273.16146	2458.72554	2559.75114
2.08E-23	517.009165	513.262939	494.87113	942.69343	914.8933	933.002388
5.86E-42	4647.8496	4899.0604	4870.2336	7875.83589	8118.37277	7947.33548
0.00028247	2210.37117	2147.26445	1952.70978	1492.26364	1641.02459	1828.53094
7.29E-07	1735.22509	1681.10787	2113.35824	1378.60557	1266.71355	1092.5064
0.00350701	3134.49888	3339.64394	3396.69942	2909.64667	2931.03218	2686.58566
0.00037781	5920.48754	6302.43708	6240.36188	6975.93138	7089.41903	7256.47171
1.31E-25	5448.4812	5829.41081	6113.8743	3721.29903	3568.80679	3540.79691
0.00036391	3448.47206	3289.59344	2916.60056	2366.76223	2527.8044	2792.28109
1.35E-08	356.882845	312.079569	318.527127	197.898763	178.319853	193.134377
1.29E-104	800.631602	803.752097	798.625984	2290.54446	2357.51698	2234.01705
4.43E-55	8990.09864	8790.24119	8514.36859	15084.432	15372.4565	15786.0929
4.83E-23	6085.84675	5957.97189	5403.88195	8861.31824	9426.85494	9839.28368
2.69E-41	350.603381	329.74445	351.76474	77.5549204	81.1275007	76.8694037
0.00026755	88.9590668	52.0132615	80.3242319	29.4173836	29.7199755	36.5129668
1.88E-06	0	0.98138229	0	32.0916912	31.3264607	24.0216887

4.62E-36	9197.32093	8751.96728	9604.74695	5625.40604	5309.43346	5371.24958
8.21E-21	13582.4796	13843.3786	13656.966	10167.7175	9958.60153	9918.07481
3.46E-16	541.080442	527.983673	568.732493	268.767914	281.938146	308.438482
6.60E-22	1511.25756	1462.25961	1501.2322	846.418356	901.238177	929.158917
0.00623879	1938.26108	1873.4588	1860.38307	2239.73262	2112.52799	2133.12595
0.00013891	2181.067	2144.32031	2363.56361	1885.38686	1841.032	1641.16177
5.10E-19	835.168651	821.416978	657.366128	344.985681	373.5078	375.699211
1.72E-44	1054.94987	1112.88752	1125.46251	2202.29231	2176.7874	2101.41732
1.36E-25	1053.9033	1046.15352	1173.4724	573.638981	547.811441	490.042449
1.24E-05	2151.76284	2080.53046	2187.2196	1799.80902	1706.08724	1556.60543
0.0183031	668.762867	687.948987	708.145815	577.650442	605.644907	569.794455
1.14E-20	572.47776	573.127258	637.97752	1199.42696	1211.28981	1021.4022
0.07469648	116.170076	130.523845	96.9430385	102.960843	85.1437137	55.7303177
1.48E-63	918.894832	909.741385	962.967516	2229.03539	2240.24356	2080.27824
0.01437253	907.382482	929.36903	986.049192	1143.2665	1052.24778	1091.54553
0.01284448	1488.23286	1420.06018	1388.59362	1210.12419	1274.74598	1257.77562
0.00613867	1621.14817	1739.9908	1911.16276	2166.18916	2273.17651	1912.12642
2.78E-14	804.817911	762.534041	853.098739	498.758368	452.225573	389.151356
2.23E-09	502.357083	504.430498	552.113686	346.322834	314.067849	271.925516
7.33E-05	350.603381	495.598057	389.618688	275.453683	278.725176	252.708165
1.12E-40	4523.3069	4539.87448	4298.73131	2559.31237	2602.50596	2614.52059
1.78E-05	38.7233585	35.3297625	37.8539484	86.9149971	81.1275007	83.5954765
8.44E-29	2208.27801	2296.43456	2503.9002	1302.3878	1330.97296	1248.16694
0.00095882	6901.13043	6957.01907	7857.00246	6100.09564	6364.89422	5698.90542
5.10E-05	24.0712769	25.5159396	36.0074143	5.3486152	2.40972774	7.68694037
0.00265106	1795.92657	1741.95357	2003.48946	1535.05256	1592.0268	1530.662
1.58E-05	164.312629	146.225961	207.735083	86.9149971	99.6020801	89.3606818
2.56E-13	4726.34289	4849.99129	4819.45392	6256.54263	6235.57216	6259.0912
4.31E-05	6813.21794	7833.39345	7044.52747	8502.96102	8934.46724	9285.82397
2.72E-11	1399.27379	1611.42972	1901.00682	998.853889	1007.2662	909.941567
0.00608277	29.3041632	23.553175	31.3910791	10.6972304	4.81945549	14.4130132
0.00051624	1202.51727	1323.88471	1319.34859	1057.68866	1059.47697	1036.77608
6.54E-06	2150.71626	2145.30169	1681.26927	1135.24358	1269.92652	1565.25323
0.01114008	1244.38036	1241.4486	1024.82641	851.766971	959.071642	1044.46302
7.90E-12	1114.60478	1179.62151	1056.21749	744.794667	751.835056	738.907143
0.00805971	5288.35488	5267.07876	4695.73613	4177.26847	4331.88724	4672.69888
1.21E-14	6341.2116	5989.37613	6086.17629	4468.768	4588.92487	4591.02514
0.00158506	2698.07617	2908.81711	2998.77133	3345.55881	3465.99174	3252.53664
3.76E-14	3067.51794	2849.93418	3148.34059	4357.78424	4210.59761	4172.08689
0.00096063	2100.48055	2109.97193	2061.65529	1771.72879	1832.19633	1767.03542
0.02161492	4048.16083	4137.50774	4095.61256	4454.05931	4512.61682	4505.50793
0.00388316	529.568092	454.380001	425.626103	362.36868	363.868889	363.207933
0.03073846	1602.30978	1662.4616	1684.96234	1924.16432	1836.21254	1789.13537

8.54E-19	1653.59207	1773.3578	1582.4797	969.436506	976.742979	1068.48471
0.01459878	1337.52573	1306.21983	1227.02189	1085.76889	1082.771	1174.18014
3.07E-05	6829.96318	6823.55108	6193.27526	5265.71167	5288.54916	5691.21848
4.00E-07	1282.05714	1242.42998	1270.41544	929.321892	1000.03701	940.689328
0.07072003	55.4685946	56.9201729	45.2400847	36.1031526	27.3102478	38.4347019
0.06399014	1342.75862	1326.82886	1367.35848	1220.82142	1203.25739	1224.14525
1.25E-08	1209.84331	1168.82631	1083.9155	822.349588	808.062037	843.641706
1.32E-14	524.335206	524.058144	496.717664	818.338126	843.404711	942.611063
0.01790165	1309.26815	1403.37668	1428.2941	1251.57596	1225.74818	1083.85859
7.88E-10	3189.96748	2860.72938	3037.54854	2349.37923	2121.36366	2113.9086
7.20E-06	4814.25538	4670.39833	4064.22148	3241.26081	3343.89887	3727.20521
3.53E-05	3121.93996	2799.88368	2551.91008	3496.65719	3557.56139	4138.45652
0.00072195	8199.93281	7732.31108	6859.87406	6011.84349	6127.93766	6632.86867
2.08E-09	569.338028	590.79214	524.415675	811.652357	834.569042	837.876501
0.07146207	4.18630903	11.7765875	9.23267034	4.0114614	0.80324258	1.92173509
4.03E-14	12720.1	14296.7772	14910.7626	9541.92952	9920.84913	9193.58068
1.09E-53	2136.06418	2239.51439	2150.28892	1021.5855	1039.3959	1064.64124
0.04635235	1583.47139	1824.38968	1912.08603	2122.06308	2127.7896	1870.80911
2.74E-13	1509.1644	1622.22493	1891.77415	2977.84151	2996.09483	2498.25562
0.00136256	386.187008	395.497064	389.618688	294.173836	307.641909	291.142867
1.69E-35	1582.42481	1573.15581	1540.00941	843.744048	806.455552	836.915633
7.09E-16	468.866611	379.794947	372.076615	736.771744	782.358274	778.302713
1.40E-15	296.181364	300.302981	351.76474	145.749764	124.5026	134.521457
1.68E-08	143.381084	166.83499	161.571731	80.2292281	61.8496788	69.1824633
2.99E-10	3587.66684	3874.49729	3975.58785	5169.43659	5138.34279	4906.18969
2.81E-07	2146.52995	2138.43201	2027.49441	2614.13568	2629.81621	2715.41169
1.17E-06	3673.48617	3729.25271	4177.78333	5586.62858	5184.93086	4683.26842
1.45E-16	2174.78754	2130.58096	2304.47452	3382.99912	3440.28798	3103.60218
3.41E-06	189.430484	172.723283	192.039543	109.646612	93.979382	106.656298
1.61E-08	7.3260408	5.88829375	8.3094033	53.486152	51.4075252	36.5129668
0.0001527	256.411428	264.973219	206.811816	320.916912	357.442949	428.546926
7.47E-05	178.964711	204.127517	205.888549	275.453683	310.051637	292.103734
1.27E-09	712.719112	686.967604	622.281981	405.157602	412.063444	459.294687
0.00329102	815.283683	953.903588	1030.36601	1168.67242	1330.97296	1071.36731
0.01233049	2086.87505	2016.74061	2092.1231	1727.60271	1777.57583	1918.85249
5.53E-39	1870.23356	1984.35499	2008.1058	3387.01058	3466.79498	3554.24905
6.30E-27	3731.04792	3642.89107	4021.7512	2207.64093	2200.88467	1907.32208
0.00027489	23.0246997	31.4042333	33.2376132	8.02292281	8.03242582	6.72607283
7.85E-17	50.2357083	41.2180563	55.396022	176.504302	179.123096	157.582278
2.24E-11	1865.00067	1641.85257	1985.02412	3266.66674	3017.78238	2574.16416
0.07705275	2347.47279	2515.28281	2502.05366	2295.89308	2210.52358	2234.97791
1.63E-14	5000.54613	5327.92446	4924.70636	3579.56072	3502.13766	3770.44425
1.13E-06	987.968931	923.480737	926.036835	1232.8558	1205.66712	1339.44936

0.05277642	5.23288628	3.92552917	7.38613627	0	0.80324258	0.96086755
0.00011386	909.475636	948.015294	784.776979	647.18244	695.608076	647.624726
8.27E-69	1193.09807	1201.21193	1227.94515	415.854832	440.176935	411.25131
6.34E-06	236.52646	223.755163	191.116276	314.231143	335.755399	407.40784
1.18E-07	396.65278	434.752355	402.544427	278.127991	257.840869	215.23433
0.06637458	102.564571	82.4361125	74.7846297	54.8233058	53.0140104	72.065066
1.32E-08	10012.6046	9750.03307	10185.4819	8238.20457	8140.86356	7933.88333
0.02246068	887.497514	832.212183	918.650699	779.560666	771.112878	713.924587
9.13E-36	1946.6337	2034.40549	1994.25679	3358.93035	3324.62105	3316.91477
3.59E-09	386.187008	374.888035	386.848887	580.32475	573.515203	590.933541
2.24E-09	518.055742	524.058144	473.635988	284.81376	318.084062	325.734098
0.0547666	8.37261806	7.85105833	11.0792044	28.0802298	17.6713368	16.3347483
0.0060109	720.045153	725.241514	638.900787	790.257896	841.798226	904.176361
2.85E-134	59857.9396	63008.6687	64390.4895	25757.5937	25774.448	24753.8697
3.51E-190	3261.13473	3171.82757	2875.05354	11900.6688	11528.9408	12404.8
0.04781865	3.13973177	0.98138229	1.84653407	8.02292281	9.63891098	7.68694037
5.48E-52	1634.75368	1678.16372	1701.58114	3254.63235	3306.14647	3131.46733
7.36E-05	5812.69008	6207.243	6133.26291	7006.68592	7053.27311	7223.80221
0.00030933	1855.58148	1979.44808	2080.12063	2497.8033	2465.95473	2256.117
2.08E-08	748.302739	815.528685	697.06661	1064.37443	1112.49098	1051.1891
2.96E-18	6120.3838	5929.51181	5850.74319	8539.06417	8128.01168	8849.5901
5.13E-47	1220.30908	1231.63478	1302.72978	2487.10607	2542.26277	2404.0906
1.45E-08	846.681001	788.04998	818.014592	1256.92457	1111.68773	1117.48896
5.96E-84	697.020453	697.76281	670.291867	1850.62086	1833.80281	1823.7266
2.51E-12	592.362727	671.265488	605.663174	942.69343	994.414316	971.437089
3.68E-09	1004.71417	1078.53914	1017.44027	744.794667	711.672927	738.907143
1.54E-21	11875.5121	10848.1999	10127.3161	6685.769	6588.19565	7095.04596
1.80E-12	729.464348	663.414429	826.323995	457.3066	394.392108	370.894873
9.57E-10	17181.6588	17083.9029	13708.6689	9005.73085	9785.90437	11582.2974
0.00050114	1424.39165	1345.47512	1191.93774	1006.87681	1018.51159	1109.80202
1.72E-06	1984.31048	1876.40294	1792.98458	1382.61703	1425.75558	1532.58374
8.62E-27	2233.39587	2192.40804	2245.38543	1400.00003	1332.57944	1289.48425
1.04E-06	1295.66264	1283.64804	1235.33129	1555.10987	1702.07103	1669.9878
0.00157984	1524.86306	1548.62126	1561.24455	1853.29517	1722.95534	1903.47861
1.41E-05	1942.44739	2120.76713	2170.6008	2582.04399	2657.12646	2522.27731
7.64E-13	7798.04714	7629.26594	7004.82699	5042.40698	5247.58379	5598.97519
1.40E-07	700.160185	708.558015	742.306695	490.735445	523.714163	422.78172
2.89E-24	2303.51654	2208.11016	2379.25915	3773.44803	3660.37644	3492.75353
3.05E-17	2387.24272	2663.47154	2857.51147	1652.7221	1608.09165	1445.14479
0.02442507	4715.87712	4466.27081	4690.19653	5276.4089	5009.82398	5033.02421
4.05E-21	2690.75013	2635.99284	2643.31352	1629.99048	1693.23536	1799.70491
3.00E-82	4247.01051	4453.51284	4379.97881	2017.76509	2014.53239	2013.01751
0.0364096	6252.25253	6480.06727	6327.14898	5856.73365	5833.95087	5863.21377

0	600.735345	589.810757	623.205248	4113.08509	4084.48853	3983.75685
7.51E-09	173.731825	210.01581	168.957867	77.5549204	71.4885898	95.1258871
9.26E-22	2289.91104	2198.29633	2332.17253	1391.97711	1493.22796	1402.86662
3.08E-07	2707.49536	2553.55672	2258.31116	3174.40312	3506.95711	3894.39617
0.00716125	2930.41632	2844.04588	2664.54866	3095.51105	3197.70872	3382.25376
4.30E-08	713.765689	820.435596	789.393314	546.895905	531.746589	514.064137
4.90E-19	2511.78542	2640.89975	2684.86053	1667.43079	1784.80502	1647.88784
0.00615415	10428.0958	10412.4661	10924.0955	9795.98875	9456.57491	8970.65941
8.39E-18	24431.2995	23838.7573	25500.6355	18000.7645	17959.7009	16946.8209
9.91E-47	144426.615	140052.085	132620.847	78362.5613	79089.6743	82799.8782
9.38E-10	3501.8475	3432.87526	2827.96692	1984.33624	2073.1691	2342.59508
1.27E-17	3409.7487	3340.62532	2891.67235	1662.08217	1718.13588	2039.9218
3.55E-05	127.682425	85.3802594	110.792044	54.8233058	45.7848272	52.8477151
9.55E-17	419.67748	385.683241	484.715193	153.772687	204.826858	151.817072
1.75E-16	454.21453	368.999742	357.304342	159.121302	151.009605	181.603966
1.75E-10	18318.2417	16954.3605	15638.297	11419.2935	12110.4884	12851.6034
1.95E-07	1189.95834	1195.32363	1413.52183	2036.48524	1877.98116	1641.16177
1.82E-05	417.584326	492.653911	319.450394	593.696288	626.529214	769.654905
9.19E-07	2658.30623	2555.51949	2419.8829	1873.35247	1974.37027	2074.51303
0.05599458	2555.74166	2650.71357	2813.19465	3071.44228	3007.34023	2811.49844
0.03860515	4317.13118	4423.08999	4457.53324	4769.62761	4801.78415	4787.04212
0.00047409	2805.87363	2653.65772	2613.76897	2253.10415	2220.1625	2356.04722
1.31E-06	1398.22722	1345.47512	1345.20007	980.133736	1067.50939	1076.17165
1.53E-12	1360.55043	1305.23845	1328.58126	895.893047	906.860875	766.772302
6.65E-118	11671.4296	11323.1889	11253.7019	4864.56553	4974.48131	5089.71539
7.52E-19	318.159486	334.651362	348.071672	605.730672	646.610278	657.233402
4.81E-58	19381.5642	20795.4908	20036.7412	35132.379	35145.0759	35180.2435
8.73E-28	477.239229	475.970412	472.712721	199.235916	145.386907	148.93447
1.81E-14	1569.86589	1644.79672	1564.01436	903.915969	1027.34726	1080.97599
9.51E-05	1851.39517	1928.4162	1826.22219	1444.1261	1559.89709	1571.97931
1.20E-11	554.685946	536.816114	527.185476	324.928374	314.067849	335.342774
1.35E-11	7690.24968	7440.84054	8066.58407	6018.52926	5738.365	5509.61451
0.00118913	4686.57296	4451.55008	3526.88007	2800.00006	3148.71092	3656.10101
3.68E-22	8324.4755	9125.87393	9868.80132	5629.4175	5429.11661	4944.62439
1.23E-19	5170.09165	5417.23025	5311.55525	7409.16921	7515.13759	7974.23977
2.93E-20	5668.26242	5962.87881	5731.64175	3983.38117	3949.54377	4133.65218
9.87E-69	2461.54971	2460.32541	2489.12792	5021.01252	4951.18727	4778.39431
3.56E-68	105.704303	118.747257	125.564317	609.742133	549.417926	600.542217
0.00907054	11097.9052	12400.7466	12209.2833	10555.4921	10746.5825	10369.6826
0.04624019	1571.95904	1532.91914	1477.22725	1366.57118	1374.34806	1375.00146
2.08E-05	296.181364	342.50242	349.918206	501.432675	466.68394	453.529482
0.01007199	551.546214	605.512874	482.868659	403.820448	443.389905	455.451217
7.73E-05	255.364851	214.922722	266.824173	175.167148	143.780422	147.012735

1.61E-13	2008.38176	2201.24048	2280.46957	1494.93795	1477.16311	1428.81004
0.00029165	27.2110087	34.3483802	32.3143462	9.36007661	9.63891098	7.68694037
1.62E-27	3507.08039	3855.85102	3453.94197	2089.97139	2193.65549	2129.28248
8.90E-30	1392.99433	1307.20121	1245.48723	2282.52154	2343.05861	2487.68608
5.82E-14	4459.46569	4256.255	3880.49134	2307.92746	2763.15448	2801.88977
3.46E-05	206.17572	174.686048	213.274685	117.669534	113.257204	124.912781
0.00109365	0	0	0.92326703	9.36007661	12.8518813	9.60867546
3.75E-05	551.546214	518.16985	421.009767	311.556836	334.148914	358.403595
1.04E-11	3697.55745	3843.09305	3998.66952	2722.44514	2940.67109	2800.9289
0.00061956	4.18630903	4.90691146	0	18.7201532	22.4907923	17.2956158
2.24E-24	7644.20028	7341.72093	9164.34858	4201.33724	3976.05078	3266.94966
7.00E-25	1965.47209	1940.19279	1774.51924	3021.96759	3404.9453	3355.34947
1.52E-11	17922.6355	17983.8305	17829.2097	14521.4903	14199.7224	14386.1089
6.91E-21	4474.11777	4529.07928	4100.2289	6598.85401	6824.34897	7483.23645
2.44E-07	27.2110087	38.2739094	36.0074143	5.3486152	3.21297033	2.88260264
2.03E-12	378.860967	368.999742	397.004825	223.304685	182.336066	164.30835
3.36E-05	3354.28011	3230.7105	2964.61045	2484.43176	2547.08223	2674.09438
0.00086262	1221.35566	1259.11348	1444.91291	1068.38589	1082.771	999.302248
2.25E-08	6430.17067	5895.16343	5934.76049	7573.63913	7711.93203	7765.73151
0.0571263	4344.34219	4300.4172	4893.31528	4356.44708	3878.05518	3833.86151
1.17E-24	792.258983	670.284105	673.984935	300.859605	332.542429	309.39935
1.85E-26	3393.00347	3155.14407	3126.18218	1948.23309	1890.83304	2027.43052
3.05E-23	3631.62308	3503.53478	3267.44203	5245.65436	5572.89703	5740.22272
1.53E-05	479.332384	480.877323	549.343885	370.391603	343.787825	349.755787
6.90E-08	579.8038	577.052788	493.947863	319.579758	301.215968	384.347019
1.27E-22	2372.59064	2389.66588	2162.29139	3562.17773	3863.59682	4017.38721
2.42E-10	1843.02255	1828.31521	1718.19995	1295.70203	1295.63028	1336.56676
0.00023186	846.681001	740.94363	733.997292	958.739275	1000.84026	991.615308
2.65E-20	3465.2173	3333.75565	3826.94185	2221.01246	2271.57002	2167.71718
2.59E-10	3918.38525	4071.75513	4194.40213	3190.44897	3076.41909	3051.71533
1.11E-08	794.352138	716.409073	630.591384	447.946523	442.586662	468.903363
1.91E-27	10626.9455	10921.8035	11418.0434	17516.7148	17120.3124	16253.0745
0.00093285	55.4685946	39.2552917	47.0866187	16.0458456	15.2616091	24.9825562
0.00028561	325.485527	272.824277	307.447922	446.609369	410.456959	397.799164
0.04320963	1128.21028	1127.60825	1175.31893	1354.5368	1347.03781	1196.2801
2.64E-11	1163.79391	1183.54704	1309.19265	1857.30663	1756.69153	1702.65729
2.27E-09	1095.76639	1125.64549	1040.52195	679.274131	748.622086	794.637461
0.00141471	487.705002	457.324148	483.791926	623.113671	576.728174	602.463952
0.00942505	272.110087	312.079569	318.527127	398.471833	409.653717	349.755787
0.00033868	3295.67178	3336.69979	2867.66741	3741.35633	3789.6985	4226.85634
5.22E-05	1057.04303	1043.20938	1245.48723	1529.70395	1508.48957	1367.31452
6.80E-13	1650.45233	1754.71154	1794.83111	1248.90165	1147.83365	1137.66718
2.03E-05	54.4220174	67.7153781	87.7103682	136.389688	136.551239	143.169264

1.30E-08	661.436826	646.73093	801.395785	1055.01435	1109.27801	1024.2848
2.62E-221	462.587148	456.342766	434.858773	2505.82622	2420.1699	2602.02932
5.70E-08	7539.54256	7181.75561	6969.74284	5319.19782	5499.80196	5969.87007
0.00035899	19.8849679	30.422851	31.3910791	60.171921	56.2269807	73.0259335
0.00077881	8.37261806	13.7393521	12.9257385	32.0916912	41.7686142	30.7477615
0.00079578	3389.86374	3033.45266	2731.94715	2194.26939	2349.48455	2659.68137
7.53E-45	2259.5603	2376.90791	2284.16264	4036.86732	4071.63665	4177.85209
0.0023406	1095.76639	1062.83702	1017.44027	929.321892	878.747384	806.167871
1.51E-08	272.110087	277.731189	305.601388	164.469918	162.255001	154.699675
2.28E-21	7216.15019	6911.87548	7484.92584	4966.18922	4898.17326	4650.59892
2.14E-20	3949.78257	3949.08234	3995.89972	5482.33058	5633.14022	5609.54474
7.41E-09	945.059263	1029.47002	964.81405	1323.78226	1399.24858	1324.07548
4.94E-48	9497.68861	10001.2669	10699.7417	20209.7425	19848.9274	18486.1307
1.29E-21	2848.78329	3046.21063	3136.33811	1925.50147	1960.71514	1918.85249
1.40E-11	7435.93141	7370.18101	7189.48039	9163.515	9482.27868	9766.25774
1.51E-37	729.464348	784.124451	806.01212	1535.05256	1564.71655	1527.7794
2.86E-07	3173.22224	2921.57508	2987.69212	2287.87015	2438.64448	2399.28626
1.34E-06	3658.83409	3544.75284	3260.0559	4270.86924	4372.85261	4666.93367
6.94E-05	851.913887	873.43024	770.927973	1036.2942	1044.21536	1179.94535
3.56E-16	2306.65627	2213.99845	2322.01659	3239.92366	3268.39406	3155.48902
2.31E-07	20.9315451	35.3297625	46.1633517	0	0.80324258	3.84347019
5.65E-11	5765.59411	5671.40826	5463.89431	4103.72501	4241.12083	4453.62108
0.00136657	3486.14884	3269.9658	3199.12027	3693.2188	3879.66167	4199.95205
0.00805647	4402.95052	4743.02062	5318.01811	6101.43279	5769.69146	5256.90635
0.0001258	1644.17287	1751.76739	1861.30634	2176.88639	2213.73655	2072.5913
0.00027127	1695.45516	1639.88981	1686.80887	1333.14234	1443.42692	1380.76666
1.90E-05	2034.54619	1877.38432	1757.90043	1379.94272	1512.50578	1507.60118
6.48E-11	548.406483	518.16985	484.715193	304.871067	304.428938	317.08629
0.07394013	1358.45728	1296.40601	1311.03919	1125.8835	1257.07464	1167.45407
7.52E-08	432.236407	466.156589	401.62116	234.001915	263.463567	291.142867
1.28E-53	8312.96315	8506.62171	8614.08143	4722.82722	4800.98091	4475.72103
7.68E-06	19233.9968	19578.5767	19128.2464	22160.6499	22407.2551	22835.9781
0.00581727	1761.38952	1670.31266	1826.22219	2104.68008	2058.71074	1936.14811
0.04438628	414.444594	440.640649	546.574084	554.918827	616.890303	533.281488
0.0059996	110.937189	79.4919656	113.561845	159.121302	148.599878	148.93447
1.78E-08	1330.19969	1469.12929	1382.13075	1010.88827	1052.24778	945.493666
0.08830154	1874.41987	2044.21931	2081.96716	1872.01532	1795.24717	1790.09624
3.60E-06	139.194775	134.449374	143.10639	72.2063052	73.0950749	70.1433309
4.96E-05	1877.5596	2024.59167	2326.63293	2726.4566	2717.36965	2582.81196
1.45E-15	953.431881	1081.48329	1043.29175	627.125133	573.515203	632.250846
0.08185857	275.249819	256.140778	298.215252	215.281762	235.350076	236.373416
1.20E-39	1414.97245	1344.49374	1415.36836	2519.19776	2537.44332	2681.78132
0.05502366	2658.30623	2735.11245	2759.64516	3064.75651	3017.78238	2860.50269

0.00038018	1049.71699	1047.13491	1079.29916	1278.31903	1309.28541	1254.89302
1.01E-05	501.310506	529.946438	553.036953	363.705834	384.753197	382.425283
2.08E-07	2033.49961	1926.45344	1969.32858	1472.20633	1528.57063	1586.39232
2.74E-13	576.664069	586.866611	570.579027	298.185298	307.641909	361.286197
1.07E-31	4223.98581	4280.78956	4245.18182	2652.91314	2686.84644	2746.15945
2.73E-10	370.488349	394.515681	319.450394	661.891131	567.089263	614.95523
5.74E-08	1841.97597	1778.26471	1882.54148	2289.20731	2386.43371	2391.59932
1.79E-07	65.9343672	120.710022	82.170766	232.664761	195.99119	187.369172
0.00187803	1184.72545	1115.83167	1074.68283	878.510047	901.238177	987.771838
6.39E-11	4550.51791	4416.22031	4928.39943	3345.55881	3510.97332	3147.80208
5.20E-25	4481.44381	4295.51029	4757.59503	2854.82336	2731.82802	2542.45553
6.22E-25	996.341549	1029.47002	996.205129	1656.73356	1658.69593	1712.26597
1.29E-08	1161.70076	1173.73322	1349.8164	877.172893	866.698746	809.050474
0.00272239	301.41425	288.526394	279.749911	370.391603	366.278617	392.033959
5.74E-05	2626.90891	2574.16575	2412.49676	1996.37062	2139.03499	2097.57385
0.00023558	1024.59913	971.568469	923.267034	715.377283	792.800428	786.950521
4.95E-05	309.786868	258.103543	280.673178	175.167148	182.336066	196.016979
7.98E-13	965.990808	974.512616	893.722489	402.483294	466.68394	597.659614
4.06E-24	1526.95622	1378.84212	1311.96245	732.760283	763.080453	774.459242
3.10E-07	205.129142	212.959957	169.881134	84.2406895	107.634506	102.812827
1.70E-05	103.611148	117.765875	97.8663056	40.114614	57.0302233	50.92598
2.32E-14	1355.31755	1313.08951	1246.4105	750.143282	813.684735	879.193805
4.50E-08	1769.76214	1686.01478	1497.53913	1161.98665	1171.93093	1226.06699
0.00082428	1243.33378	1196.30501	1178.08874	988.156659	1004.85647	1013.71526
0.00027649	3830.47276	3835.242	3602.58797	3202.48335	3184.05359	3241.00623
7.57E-35	1462.06843	1569.23028	1718.19995	3140.97428	3173.61144	2946.0199
2.75E-12	1466.25474	1308.1826	1373.82135	2071.25124	2028.99076	1915.00902
6.06E-15	1577.19193	1724.28869	1578.78663	2323.97331	2390.44992	2409.85581
7.89E-39	1158.56102	1199.24916	1167.9328	2143.45754	2125.37987	2142.73463
1.23E-06	1664.05784	1759.61845	1953.63304	1390.63995	1354.26699	1233.75393
0.01091879	837.261806	936.238706	1047.90808	775.549204	779.145304	780.224448
0.00010547	2230.25613	2086.41875	1873.30881	2491.11753	2561.54059	2721.17689
6.94E-08	8960.79447	9880.55691	9815.25184	11768.2906	12170.7316	12084.8311
0.02537452	603.875077	638.879872	702.606213	810.315203	780.751789	715.846322
1.99E-51	2121.4121	2247.36545	2154.90526	4454.05931	4399.35962	4072.15666
9.80E-21	1162.74733	1047.13491	1212.24962	2011.07932	2052.2848	1845.82656
0.00704507	1677.66334	1966.69011	2209.37801	1599.23595	1692.43212	1440.34045
5.06E-15	1359.50386	1528.01223	1581.55643	837.058279	970.317039	836.915633
0.03394575	1945.58712	2302.32286	2457.73684	2583.38114	2635.43891	2528.04251
7.26E-05	706.439648	786.087216	741.383428	561.604596	593.596268	541.929296
3.38E-11	258.504582	248.28972	276.056843	128.366765	102.011808	133.560589
8.61E-42	5411.851	5495.74083	4886.85241	9741.16544	10271.8661	11053.8203
5.11E-28	3245.43607	3515.31137	3583.19936	5951.67157	5737.56176	5461.57113

0.00042371	414.444594	458.30553	466.249852	573.638981	624.922728	558.264044
1.45E-07	1148.09525	1104.05508	1208.55655	825.023895	852.240379	873.4286
0.01677515	0	0.98138229	1.84653407	9.36007661	9.63891098	5.76520528
1.66E-06	3629.52993	3741.0293	4088.22643	4859.21691	4893.35381	4656.36413
0.068912	9.41919531	7.85105833	4.61633517	13.371538	8.8356684	34.5912317
4.09E-38	50.2357083	53.9760261	57.2425561	272.779375	322.903518	270.003781
1.96E-17	1022.50598	1059.89288	965.737317	593.696288	606.448149	619.759567
9.34E-09	1201.47069	1209.06298	1270.41544	1771.72879	1662.71214	1589.27492
1.18E-07	1240.19405	1320.94056	1486.45992	1000.19104	926.941939	798.480931
1.86E-12	917.848254	1047.13491	1146.69766	635.148055	637.77461	570.755323
3.10E-17	739.930121	688.930369	716.455218	1176.69534	1119.72016	1175.14101
0.00068579	142.334507	136.412139	138.490055	90.9264585	73.8983175	92.2432845
1.76E-15	3713.25611	4022.68601	3706.91714	2698.37637	2672.38807	2694.2726
4.29E-13	423.863789	414.143327	520.722607	882.521509	787.17773	777.341845
5.46E-53	6076.42755	6079.6633	6794.3221	2725.11945	2677.20752	2253.2344
0.00018434	646.784745	719.35322	764.465104	988.156659	887.583053	902.254626
0.0399672	1144.95552	1258.1321	1157.77686	1338.49095	1302.05622	1384.61013
1.14E-11	1247.52009	1239.48583	1284.26444	871.824278	863.485775	891.685083
2.70E-07	800.631602	913.666914	887.259619	1187.39258	1177.55362	1169.3758
0.00236065	468.866611	475.970412	567.809226	714.04013	628.135699	617.837832
0.0031907	104.657726	99.1196115	91.4034363	64.1833824	65.0626491	43.2390396
2.92E-07	573.524337	551.536848	506.873602	367.717295	379.130499	345.912317
7.42E-12	407.118553	412.180563	422.856301	691.308515	637.77461	667.802945
7.72E-21	3972.80727	4050.16472	3845.4072	5690.92658	5594.58458	5849.76162
0.07594026	1434.85742	1442.63197	1419.9847	1303.72496	1321.33405	1277.95384
3.97E-05	2076.40928	2067.77249	2016.4152	1744.98571	1669.94133	1665.18346
0.0011841	1014.13336	1054.98596	1096.84124	1318.43365	1290.00759	1213.57571
2.74E-05	1094.71981	997.084409	984.202658	755.491897	775.129091	825.385222
0.06359435	7757.23063	7793.15678	9013.85605	7597.7079	7491.04032	6938.42455
9.41E-50	483.518693	490.691146	494.87113	119.006688	130.928541	131.638854
0.00557872	392.466471	357.223154	360.074143	290.162375	267.47978	304.595012
1.97E-09	4745.18128	4557.53936	4928.39943	6277.93709	6178.54194	5904.53107
0.00675959	181.057865	133.467992	156.955396	113.658073	107.634506	98.9693573
1.66E-18	2031.40646	2179.65007	2208.45474	1334.47949	1431.37828	1338.48849
7.97E-07	417.584326	331.707215	429.319171	256.73353	238.563047	199.86045
2.04E-06	716.905421	811.603155	809.705189	996.179582	1149.44013	1068.48471
6.25E-14	2072.22297	2172.78039	2513.13287	3864.37448	3780.86283	3304.42349
7.55E-06	1170.07337	1224.7651	976.816522	1424.0688	1575.1587	1718.03117
0.06105161	771.327438	800.80795	656.442861	802.292281	859.469562	928.19805
6.81E-11	395.606203	451.435854	391.465222	217.95607	219.285225	245.021224
0.01755808	1246.47351	1310.14536	1167.00953	1072.39735	1072.32885	1085.78033
0.00145245	329.671836	353.297625	388.695421	482.712522	563.876292	421.820853
2.08E-06	1024.59913	1120.73858	1031.28928	1306.39926	1437.80422	1575.82278

2.38E-17	1739.4114	1790.0413	1828.99199	2586.05545	2588.85084	2576.08589
8.09E-09	981.689467	963.717411	914.034363	1266.28465	1350.25078	1304.85813
0.00788876	190.477061	155.058402	163.418265	139.063995	102.011808	111.460635
0.00694759	2193.62593	2082.49322	2070.88796	2350.71638	2427.39908	2432.91663
6.61E-05	1025.64571	972.549851	665.675531	446.609369	563.876292	641.859521
0.00089937	432.236407	421.994385	501.333999	355.682911	345.39431	268.082045
0.08285905	6028.285	6427.07263	6625.36423	5927.6028	6004.2383	5584.56218
0.03128234	1335.43258	1303.27568	1167.9328	1045.65427	1078.75479	1170.33667
1.16E-34	2408.17427	2679.17366	2623.00164	1421.39449	1346.23457	1398.06228
3.63E-75	6595.52987	5911.84693	5965.22831	2580.70684	2293.25757	2475.1948
1.72E-08	1407.64641	1309.16398	1441.21984	1017.57404	969.513796	1040.61955
3.57E-07	10555.7782	10495.8836	8181.99245	12852.7223	14104.9397	16297.2745
0.06365626	29.3041632	27.4787042	37.8539484	18.7201532	19.277822	14.4130132
3.15E-13	774.46717	805.714862	778.314109	514.804213	465.880697	487.159846
5.39E-05	2519.11146	2552.57534	2653.46946	3207.83197	3090.07421	2991.18067
0.0281066	11600.2623	11450.7686	13005.1394	14819.6756	14307.3569	12301.9872
4.09E-33	1061.22934	948.015294	885.413085	1884.04971	2024.17131	2092.76952
1.86E-05	334.904722	367.036977	372.999882	542.884443	528.533619	464.099025
1.06E-07	194.66337	263.010454	244.665764	125.692457	111.650719	124.912781
1.05E-16	2283.63157	2132.54372	1983.17759	1331.80519	1232.17412	1389.41447
2.80E-05	46.0493993	69.6781427	66.4752264	29.4173836	17.6713368	13.4521457
3.03E-05	3238.11003	3069.76381	2984.92232	3598.28088	3800.9439	4051.01758
1.63E-24	376.767812	334.651362	418.239966	826.361049	796.013398	797.520064
3.25E-134	1020.41283	1119.75719	1032.21254	3352.24458	3549.52897	3573.46641
6.63E-05	1543.70145	1448.52026	1395.97976	1698.18533	1900.47195	2103.33906
1.51E-24	1783.36765	1793.96683	1780.98211	2782.61706	2733.43451	2726.9421
3.83E-10	653.064208	605.512874	673.984935	960.076429	927.745182	957.024076
0.00271246	27.2110087	22.5717927	39.7004825	13.371538	10.4421536	5.76520528
1.00E-13	29.3041632	29.4414688	20.3118747	112.320919	98.7988375	136.443192
3.42E-12	963.897654	855.765358	854.022006	1311.74788	1361.49618	1534.50547
7.16E-05	5075.8997	5421.15578	5301.39931	4305.63524	4545.54977	4492.05578
2.35E-169	4076.41842	3843.09305	3777.08544	11293.601	11124.1065	11155.6722
0.0018654	2770.29	2691.93163	2768.87783	3144.98574	3079.63206	3188.15852
2.79E-19	335.951299	310.116804	298.215252	632.473748	607.251392	600.542217
2.35E-05	5693.38028	6129.71379	5723.33234	6923.78238	6859.69165	7197.85879
0.01853327	2720.05429	2565.33331	2935.0659	2565.99814	2344.6651	2209.03449
5.52E-09	961.804499	1001.99132	839.249734	1303.72496	1346.23457	1473.00995
1.38E-10	18629.0752	19256.6833	21252.6839	28439.9242	28718.332	25355.3728
8.31E-29	2504.45938	2793.99538	2776.26397	4424.64193	4467.63524	4506.46879
1.53E-38	1489.27944	1330.75439	1401.51936	2715.75937	2602.50596	2633.73794
5.44E-31	854.007042	825.342507	890.952688	414.517678	399.211563	390.112224
0.0236432	477.239229	543.68579	517.029539	616.427902	606.448149	600.542217
1.27E-05	3056.00559	3236.5988	3303.44945	2584.7183	2698.09183	2562.63375

4.59E-27	4602.84678	4532.02342	4272.87983	2790.63998	2861.15008	2872.0331
4.64E-15	1891.1651	2120.76713	2211.22455	3194.46043	3182.44711	3042.10665
1.65E-05	2206.18486	2098.19534	2044.11321	2472.39738	2702.10804	2705.80301
4.89E-61	953.431881	951.940823	951.888312	2031.13662	2098.06962	2109.10426
0.00018939	459.447416	412.180563	397.004825	521.489982	583.154114	607.268289
2.93E-67	2997.39726	3183.60415	3563.81075	8341.16541	8268.57914	7665.80129
1.08E-08	899.009864	813.56592	975.893255	637.822363	569.49899	572.677058
4.49E-37	874.938587	955.866352	973.123454	1944.22163	1874.76819	1788.1745
9.53E-14	446.888489	439.659267	435.78204	243.361992	223.301438	245.982092
0.01414224	14.6520816	8.83244063	14.7722725	1.3371538	3.21297033	3.84347019
3.42E-05	2287.81788	2506.45037	2815.04119	3357.59319	3466.79498	3074.77615
7.57E-18	4519.1206	4373.03949	3874.95174	6379.56078	6588.9989	6982.62446
1.11E-08	3546.85032	3530.0321	2933.21937	2088.63424	2117.34745	2508.82516
7.16E-10	1119.83766	1098.16678	837.4032	574.976134	528.533619	648.585594
3.72E-16	3224.50453	3157.10683	2762.41497	1678.12802	1784.00177	2009.17404
5.25E-14	1420.20534	1331.73577	1206.71001	794.269358	810.471765	858.054719
2.00E-07	6510.75712	6216.07544	5515.59726	4297.61232	4425.06338	4849.49851
0.00062392	3893.2674	4005.02113	3822.32552	3233.23789	3296.50755	3506.20568
1.21E-09	5.23288628	1.96276458	5.5396022	44.1260754	48.9977975	40.356437
0.02482864	1429.62453	1375.89797	1302.72978	1099.14042	1161.48877	1284.67991
1.84E-05	2290.95762	2456.39988	2486.35812	1933.5244	2018.54861	1772.80062
6.06E-10	4535.86583	5085.52304	5148.13698	3753.39072	3608.96892	3495.63613
3.24E-18	5784.4325	6227.85202	7106.38636	3931.23217	3869.21952	3459.12317
4.45E-59	934.59349	961.754646	879.873483	2167.52631	2046.6621	2114.86947
1.95E-34	305.600559	353.297625	285.289513	76.2177666	70.6853472	66.2998607
0.00661805	775.513747	781.180304	770.004706	671.251208	664.281615	510.220667
0.00036429	3976.99358	3703.73677	2819.65752	4464.75654	4570.45029	4941.74179
2.20E-07	2229.20956	2053.05175	1902.85336	2615.47283	2775.20312	2933.52862
0.05433727	16054.4951	15749.223	13940.4089	15821.2038	16984.5644	18710.9737
1.65E-10	336.997877	343.483802	291.752383	164.469918	163.861487	180.643099
1.14E-120	4164.33091	4234.66459	4596.02329	1297.03919	1392.01939	1235.67566
1.38E-07	630.039509	506.393263	553.036953	387.774602	346.197553	343.029714
3.45E-06	435.376139	505.41188	484.715193	719.388745	654.642704	646.663859
7.31E-16	427.003521	431.808208	438.551841	216.618916	229.727378	208.508258
2.89E-31	1477.76709	1276.77836	1277.80157	652.531055	646.610278	636.094316
0.00032986	1471.48762	1475.01758	1607.40791	2138.10893	1939.02759	1718.99204
1.19E-05	97.3316849	137.393521	153.262328	33.428845	69.8821046	52.8477151
0.00042541	183.15102	216.885486	192.039543	114.995227	144.583665	113.38237
2.57E-07	2791.22154	3415.21038	4241.48875	8369.24564	8705.5431	6463.75599
1.59E-58	4882.2829	5139.49906	5146.29045	2623.49576	2653.91349	2513.6295
2.59E-135	325.485527	235.53175	249.282099	2033.81093	1842.63848	1816.03966
0.03302644	1.04657726	0.98138229	2.7698011	6.685769	12.0486387	6.72607283
3.79E-51	10778.6992	10431.1124	10879.7787	6304.68017	6025.1226	5937.20057

7.75E-06	2819.47913	2995.17875	3094.7911	3579.56072	3711.78397	3622.47065
4.53E-53	3148.10439	3176.73448	3375.46428	1639.35056	1639.41811	1572.94017
3.50E-09	7551.05491	7210.2157	7223.64127	5797.89888	5908.65243	5904.53107
0.00017376	5682.91451	5973.67401	5454.66164	4551.67154	4751.17987	5010.92425
1.37E-76	15011.0576	13949.3679	13716.0551	30146.1324	29290.2407	31437.6644
1.78E-29	1087.39377	1060.87426	1090.37837	1978.98763	1868.34224	1833.33528
4.33E-08	3076.93714	2782.2188	3211.12274	4202.6744	3951.15026	3906.88744
2.64E-22	6711.69995	6234.7217	7084.22795	4261.50916	4303.77375	3969.34383
1.29E-13	1779.18134	1654.61054	1652.64799	2353.39069	2424.98935	2411.77754
2.45E-05	1174.25968	1118.77581	983.279391	1330.46803	1494.83444	1734.36592
0.00875928	1173.21311	1173.73322	1172.54913	1401.33718	1289.20434	1389.41447
0.04969855	6347.49106	6050.22183	6339.15145	6601.52831	6923.14781	6830.80739
1.97E-07	8079.57642	7592.95479	7725.89854	10016.6191	9389.90578	9500.09743
8.05E-14	6209.34287	6031.57557	5973.53771	7895.89319	7928.00428	7969.43543
1.45E-07	11230.8205	11611.7153	11926.7635	14135.0528	13952.3236	13998.8793
7.26E-20	4789.13753	4643.90101	4576.63469	3034.00197	3052.32181	3307.30609
0.00049619	7699.66888	7172.92317	7953.02223	9293.21892	8813.17761	8675.67308
1.63E-43	5883.85734	5445.69034	5853.51299	9937.72705	9680.67959	9952.66605
5.09E-62	2274.21238	2330.78294	2454.04378	4686.72407	4866.8468	4675.58148
3.32E-06	5502.90322	5348.53349	5905.21595	4610.50631	4551.17247	4508.39053
0.0217032	769.234284	713.464926	728.45769	931.996199	886.77981	790.793991
3.38E-39	2201.99855	2111.93469	2034.88054	3800.1911	3731.86503	4038.5263
1.49E-13	4185.26245	4127.69392	4577.55795	3092.83674	3095.69691	2990.2198
0.00012448	24366.4117	24822.1023	25653.8978	29822.5412	29225.9813	27844.0198
2.38E-05	15014.1973	14384.1203	12298.8402	17001.9106	17558.0796	19906.293
4.40E-14	923.081141	964.698793	1019.28681	1545.74979	1463.50798	1423.04484
0.00097868	568.291451	484.802852	445.937977	620.439364	642.594065	736.024541
8.34E-19	12977.558	11820.7497	11987.6992	17297.4216	17366.1046	17824.093
3.53E-47	9503.96807	8680.32637	8596.53935	15973.6393	15981.3144	16407.7742
1.87E-20	3978.04015	3789.11703	3700.45427	5458.26182	5610.64943	5559.57962
6.41E-23	1275.77768	1455.38994	1513.23467	2545.94084	2714.95993	2397.36453
4.32E-74	1474.62736	1343.51236	1409.82876	455.969446	488.37149	441.038204
0.00814571	2051.29142	2022.6289	2290.62551	2670.29614	2596.08002	2272.45175
6.70E-16	1177.39941	1106.99923	1143.92785	711.365822	746.212358	696.628971
7.58E-12	6638.43954	6931.50313	7290.1165	9794.65159	9504.76947	8972.58115
2.78E-23	926.220872	854.783976	905.72496	1577.84149	1506.07984	1488.38383
6.36E-16	2272.11922	2640.89975	2675.62786	4000.76417	3888.49734	3820.40936
9.06E-10	2114.08606	2191.42666	2282.31611	2844.12613	2947.09703	2882.60264
0.03960224	469.913188	430.826826	481.022125	397.134679	388.76941	379.542681
9.43E-18	1226.58855	1094.24126	1022.05661	1855.96948	1802.47635	1873.69172
5.82E-22	3620.11073	3450.54014	3612.7439	5253.67728	5234.7319	5094.51973
0.02186221	15.6986589	26.4973219	30.4678121	10.6972304	9.63891098	9.60867546
0.06993309	610.154541	739.962248	771.85124	803.629434	847.420924	810.972209

4.76E-19	1366.8299	1351.36342	1414.4451	2044.50816	2118.15069	2225.36924
4.77E-18	1045.53068	992.177497	1029.44274	1642.02487	1556.68412	1574.86191
0.00350129	687.601258	671.265488	739.536894	619.10221	553.434139	492.925051
6.93E-28	321.299218	386.664623	432.088972	81.5663819	108.437749	65.3389932
0.03925143	4023.04298	4081.56895	4202.71154	4472.77946	4590.53135	4390.20382
0.00272086	3512.31327	3423.06143	3234.20442	2626.17006	2888.46032	3074.77615
0.0279487	1580.33166	1557.4537	1618.48711	1450.81187	1374.34806	1415.3579
1.72E-07	385.140431	311.098187	372.076615	601.71921	587.170327	507.338065
5.32E-08	2630.04865	2498.59932	2411.57349	1876.02678	1975.97675	1950.56112
0.00046705	1612.77555	1538.80743	1560.32129	1311.74788	1327.75999	1308.7016
2.89E-08	2151.76284	1949.02523	2213.07108	2762.55975	2772.79339	2797.08543
8.20E-05	486.658424	476.951794	430.242438	362.36868	328.526216	302.673277
2.17E-27	996.341549	1047.13491	1014.67047	1787.77463	1714.92291	1881.37866
7.71E-10	163.266052	137.393521	108.94551	50.8118444	48.1945549	42.278172
0.02871424	1382.52856	1395.52562	1314.73226	1073.7345	1217.71575	1257.77562
2.77E-10	1629.52079	1532.91914	1438.45004	994.842428	953.448944	1123.25416
0.00247883	2518.06488	2611.45828	2935.0659	3241.26081	3320.60483	3035.38058
3.51E-08	1202.51727	1104.05508	1297.19018	829.035357	885.173325	778.302713
0.00401052	7795.95399	7166.0535	7201.48286	6634.95716	6345.61639	6538.70365
1.90E-05	2502.36622	2728.24277	2957.22431	3563.51488	3657.96672	3272.71486
3.40E-76	2701.2159	2688.0061	2946.1451	1132.56927	1098.83585	1057.91517
5.15E-07	1260.07902	1323.88471	968.507118	587.010519	682.756194	847.485176
0.00015922	819.469992	804.733479	782.007178	573.638981	583.154114	678.372488
0.00300528	7.3260408	9.81382292	6.46286924	20.057307	28.1134904	25.9434238
2.54E-57	6788.10009	6373.0966	6389.93114	3143.64859	3213.77357	3431.25801
0.00032055	2589.23213	2658.56463	2604.5363	2945.74982	3158.34983	3119.93692
9.51E-13	400.839089	388.627388	481.945392	780.89782	738.983175	709.120249
8.53E-09	2646.79388	2677.21089	2992.30846	2076.59985	2096.46314	1933.2655
4.76E-13	4417.6026	4200.31621	4054.06555	2968.48144	3102.12285	3130.50647
3.38E-13	439.562448	454.380001	494.87113	799.617973	771.916121	729.298468
2.82E-06	496.07762	589.810757	609.356242	371.728757	400.014806	372.816608
1.75E-10	2209.32459	2295.45318	2511.28633	3516.7145	3496.51496	3076.69788
0.00875928	3013.09592	2856.80385	3431.78356	3971.34679	3807.36984	3336.13212
4.60E-72	11.5123498	4.90691146	11.0792044	410.506217	424.112083	406.446972
0.04627004	12.5589271	16.683499	39.7004825	6.685769	14.4583665	2.88260264
8.67E-07	601.781923	659.4889	453.324114	826.361049	922.925726	1144.39325
2.25E-05	920.987986	1010.82376	1099.61104	1297.03919	1375.95454	1295.24945
2.02E-12	676.088908	746.831924	810.628456	1268.95896	1216.10927	1115.56722
0.05111037	771.327438	795.901039	684.140872	871.824278	813.684735	920.51111
1.04E-05	2425.96608	2608.51413	2617.46204	2120.72593	2070.75938	1996.68276
5.10E-39	5323.93851	5490.83392	5823.04518	10120.9171	10372.2715	9392.48027
4.25E-14	13528.0576	14280.0937	14162.9163	18163.8972	18436.0237	18158.4749
1.27E-05	3654.64778	3650.74213	3863.87254	4435.33916	4434.70229	4498.78185

0.04896278	14.6520816	19.6276458	24.0049429	38.7774602	31.3264607	36.5129668
1.62E-76	6366.32945	6188.59673	5843.35706	2405.53969	2594.47354	2702.92041
0.00095505	144.427661	148.188726	142.183123	69.5319976	65.0626491	112.421503
1.07E-12	1022.50598	1020.63758	878.026949	632.473748	580.744387	558.264044
0.00019904	8652.05418	8484.04991	8284.47509	6890.35354	7164.92383	7591.81448
6.77E-05	1365.78332	1466.18514	1512.3114	1782.42602	1856.29361	1739.17026
1.37E-17	106.75088	116.784493	108.022243	22.7316146	18.4745794	9.60867546
2.89E-25	780.746634	814.547302	724.764622	365.042988	368.688345	386.268754
0.01957415	4.18630903	12.7579698	17.5420736	32.0916912	28.1134904	25.9434238
3.30E-19	6568.31886	6917.76378	7344.58925	10845.6545	10755.4182	9925.76176
3.89E-22	2959.72048	3213.04562	3434.55337	5497.03928	5288.54916	5004.19818
1.89E-15	2287.81788	2312.13668	2465.12298	3788.15672	3503.74414	3314.99304
0.00699743	2507.59911	2808.71612	2899.05849	3155.68297	3192.88926	3144.91948
0.02334815	504.450238	465.175206	409.930563	312.893989	369.491588	409.329575
1.26E-57	7679.78391	7183.71838	7642.80451	4059.59894	3996.93509	4026.99589
4.28E-12	1491.37259	1684.05201	1792.06131	2507.16338	2497.28119	2385.83412
1.56E-07	686.554681	613.363932	721.99482	488.061137	439.373692	414.133913
2.56E-26	3180.54828	3387.73167	3242.51382	2045.84532	1955.09244	2091.80865
0.00074448	5484.06483	5516.34986	6609.66869	8105.82634	7564.93863	6603.08178
1.37E-08	290.948477	334.651362	300.061786	147.086918	172.697155	186.408304
1.04E-05	3206.71272	3630.1331	3518.57067	2741.16529	2852.31441	2705.80301
0.00175187	345.370495	359.185919	463.480051	538.872982	583.957357	481.394641
4.44E-34	1599.17005	1728.21422	1790.21478	857.115586	879.550627	893.606818
1.52E-42	238.619615	294.414688	297.291985	858.45274	852.240379	770.615772
0.01074709	9202.55382	8750.9859	7571.71294	6727.22077	7131.99088	7804.16621
8.50E-05	7523.8439	6664.56714	6604.12909	5787.20165	5653.22129	5718.12277
0.00028344	251.178542	258.103543	264.054372	351.67145	335.755399	397.799164
0.05627645	0	0	0	0	4.01621291	6.72607283
7.23E-20	2933.55605	3172.80895	3037.54854	1980.32478	2061.12046	2026.46966
1.70E-22	762.95482	808.659008	884.489818	407.831909	423.308841	405.486105
1.70E-41	117.216653	162.90946	158.80193	699.331438	763.080453	547.694501
7.80E-20	8276.33295	8141.54749	9202.20253	5644.12619	5550.40624	5107.01101
6.91E-84	9904.80716	9557.68214	10357.2096	22579.1791	21975.9138	20846.9823
0.00744773	83.7261806	103.045141	112.638578	168.481379	163.861487	122.991046
2.50E-07	75.3535625	71.6409073	76.6311638	187.201532	135.747996	157.582278
9.26E-05	1331.24627	1525.06808	1523.39061	1964.27893	1861.9163	1741.09199
0.04552139	1824.18416	1676.20095	1721.89302	1865.32955	1971.96054	1973.62194
2.30E-23	2443.75789	2160.02242	2245.38543	3899.14048	3631.45971	3667.63142
7.62E-07	1938.26108	1993.18743	2144.74932	1588.53872	1556.68412	1586.39232
5.19E-05	96.2851076	69.6781427	104.329175	181.852917	175.106883	143.169264
2.11E-10	444.795334	405.310887	397.004825	680.611285	707.656714	617.837832
6.25E-07	3092.63579	2906.85435	2637.77392	3548.80619	3921.43028	4042.36977
0.0575306	600.735345	649.675077	672.138401	553.581674	587.170327	515.025005

9.10E-13	6862.40707	7279.89384	7062.06954	9176.88654	9176.24325	9197.42415
0.00010686	4195.72822	4033.48122	3814.93938	3298.75843	3354.34102	3436.06235
1.39E-10	555.732523	448.491707	496.717664	778.223512	796.816641	823.463487
0.01017252	1165.88706	1094.24126	1110.69024	877.172893	943.006791	1026.20654
1.08E-45	10904.2884	11108.2662	11590.6943	18789.6852	18996.6871	18289.1529
3.33E-96	2754.59134	2685.06195	2903.67482	7073.54361	7151.2687	6585.78616
3.63E-36	1738.36482	1661.48022	1785.59844	3325.5015	3131.84283	3017.1241
0.02761673	2243.86164	2546.68705	2584.22443	2806.68583	2785.64527	2748.08118
1.12E-15	830.982342	835.15633	761.695303	1252.91311	1321.33405	1478.77515
0.01123959	1931.98162	1928.4162	2270.31364	1773.06594	1817.73796	1468.20561
4.03E-12	422.817212	381.757712	395.15829	192.550147	212.859284	229.647344
1.34E-32	2023.03384	2019.68476	2191.83594	1119.19773	1163.8985	1144.39325
0.00097458	2643.65415	2578.09128	2746.71943	2277.17292	2344.6651	2083.16084
6.11E-05	2686.56382	2645.80666	2490.97446	2134.09747	2159.9193	2192.69974
0.08746886	25.1178542	15.7021167	16.6188066	5.3486152	12.0486387	8.64780792
7.22E-05	25.1178542	22.5717927	12.9257385	0	0.80324258	0.96086755
4.49E-22	3589.75999	3658.59318	3612.7439	2434.95707	2452.2996	2272.45175
2.64E-07	201.989411	259.084925	291.752383	446.609369	444.99639	399.720899
0.00018004	5898.50942	5838.24325	6121.26043	6836.86738	7103.07415	6785.64661
0.00062528	299.321095	370.962506	300.985053	439.9236	420.09587	480.433773
1.35E-06	1894.30484	1836.16627	1688.6554	2187.58362	2486.03579	2464.62526
0.00361403	1108.32532	1139.38484	842.019535	1164.66096	1355.07024	1594.07926
8.46E-05	1772.90187	1651.6664	1456.91538	1924.16432	2190.44252	2252.27353
0.0055302	1600.21663	1485.81279	1416.29163	1664.75648	1748.6591	2006.29144
1.85E-06	1632.66052	1750.78601	1482.76686	2111.36585	2069.95613	2270.53001
2.94E-30	206.17572	239.457279	263.131105	633.810902	616.08706	596.698746
2.63E-09	401.885667	433.770973	441.321642	724.73736	694.804833	605.346554
0.0360213	2.09315451	2.94414688	4.61633517	6.685769	12.8518813	15.3738807
3.33E-09	109.890612	100.100994	84.0173001	193.887301	203.220373	224.843006
2.19E-10	5057.06131	5184.64265	5459.27797	7207.25899	6947.24509	6600.19918
3.64E-05	20198.9411	21061.4454	21171.4364	24378.9881	23972.7748	23895.815
0.00183872	293.041632	261.04769	324.989996	177.841456	222.498195	217.156066
0.0003303	301.41425	282.6381	263.131105	220.630377	186.352279	163.347483
0.00023665	103.611148	90.2871708	93.2499704	56.1604596	52.2107678	40.356437
4.29E-25	4395.62448	4261.16191	3976.51111	2426.93415	2567.96653	2676.01612
4.11E-77	5597.09517	5480.03872	6071.40401	2517.86061	2408.9245	2328.18207
1.67E-13	3137.63862	3237.58018	2966.45698	4216.04593	4345.54237	4629.45984
3.93E-07	1529.04937	1606.52281	1475.38072	2013.75362	1936.61786	1978.42628
1.37E-09	3259.04158	3231.69189	3413.31822	4570.39169	4350.36182	4136.53479
0.0202912	1541.6083	1380.80488	1323.96493	1103.15189	1249.04221	1262.57996
1.50E-62	4783.90464	4649.7893	4776.06037	2463.0373	2491.65849	2509.78603
6.52E-63	2150.71626	2504.48761	2640.54372	803.629434	827.339859	708.159382
4.93E-05	1952.91316	1792.00406	1830.83853	2353.39069	2249.88247	2221.52577

6.74E-08	663.529981	722.297367	683.217605	490.735445	480.339064	460.255555
1.72E-40	2937.74236	3042.2851	3088.32823	5111.93898	5022.67586	5241.53247
0.00037839	546.313328	520.132615	540.111215	367.717295	416.8829	427.586058
1.25E-13	2935.64921	2892.13361	2894.44215	2072.58839	2181.60685	2033.19573
4.82E-68	5211.95474	5145.38736	5671.62939	2418.91123	2299.68351	2166.75632
0.00408229	427.003521	449.47309	458.863716	561.604596	530.943346	564.990117
2.45E-09	545.266751	511.300174	534.571613	778.223512	776.735576	769.654905
2.48E-05	872.845432	855.765358	871.56408	1046.99143	1140.60447	1124.21503
0.0203389	565.151719	539.760261	661.059196	512.129906	501.223371	392.033959
1.14E-19	2485.62099	2500.56208	2599.91997	1732.95133	1606.48516	1642.12264
5.96E-09	1998.96256	2281.71383	2395.87795	3127.60274	3141.48174	2995.98501
2.41E-50	4506.56167	4272.9385	3925.73143	1730.27702	1783.19853	1996.68276
0.00013059	2106.76002	1949.02523	2048.72955	1735.62563	1602.46895	1682.47907
4.50E-42	1360.55043	1345.47512	1300.88325	653.868209	601.628694	598.620481
2.21E-06	670.856022	719.35322	658.289395	936.007661	874.731171	970.476222
0.00372263	52.3288628	66.7339958	56.3192891	25.4059222	32.9329458	30.7477615
5.29E-17	2391.42903	2161.98519	2295.24185	1560.45849	1419.32964	1447.06652
1.03E-11	465.726879	591.773522	611.202776	1010.88827	992.807831	895.528553
3.32E-11	2501.31964	2378.87068	2490.97446	3174.40312	3364.78317	3520.61869
1.51E-22	1893.25826	1924.49067	2142.90279	3941.9294	3806.56659	3233.31929
4.74E-06	69.074099	83.4174948	88.6336352	34.7659988	32.1297033	28.8260264
0.00682738	61.7480582	65.7526136	59.0890902	26.743076	28.1134904	44.1999071
4.31E-22	1949.77343	2248.34683	2256.46463	1234.19296	1262.69734	1138.62804
1.81E-07	1417.06561	1317.01504	1390.44015	1009.55112	1032.96996	1065.60211
1.84E-07	453.167952	431.808208	511.489937	689.971361	771.916121	652.429064
2.07E-11	2420.7332	2624.21625	2997.84806	1830.56355	1757.49477	1588.31405
0.00043739	275.249819	399.422593	412.700364	611.079287	547.008198	475.629436
2.87E-09	331.76499	375.869418	349.918206	176.504302	204.023616	212.351728
1.13E-22	536.894133	542.704407	527.185476	255.396376	252.218171	220.038668
1.20E-06	931.453759	843.007389	601.970106	343.648527	403.227776	537.124958
9.37E-07	159.079743	103.045141	103.405908	38.7774602	53.0140104	49.9651124
3.63E-06	557.825678	631.028814	640.747321	977.459428	879.550627	779.26358
2.11E-07	2558.88139	2563.37055	2475.27892	1806.49478	1879.58764	2084.12171
4.02E-09	2059.66404	1849.90562	1894.54395	1401.33718	1426.55882	1447.06652
1.12E-06	2149.66969	2059.92143	2140.13298	1698.18533	1690.02239	1690.16601
5.38E-42	122.449539	147.207344	165.264799	557.593135	584.760599	502.533727
2.66E-10	109.890612	121.691404	148.645992	48.1375368	42.5718568	36.5129668
6.63E-13	368.395194	354.279007	369.306814	640.496671	583.154114	615.916097
0.00014714	1201.47069	1177.65875	1116.22984	1400.00003	1441.82043	1431.69264
0.07091708	2809.01336	2807.73474	2940.6055	3237.24935	3163.16929	2971.96332
1.58E-19	2735.75295	2982.42078	3399.46922	5943.64864	5759.24931	4943.66353
1.30E-160	6182.13186	6054.14736	5601.46109	1819.86632	1781.59205	1795.86144
7.06E-08	2288.86446	2326.85741	2206.60821	2900.28659	2816.16849	3069.01094

1.86E-09	88.9590668	73.6036719	90.4801693	172.49284	183.139309	206.586522
7.23E-05	3388.81716	3374.9737	3003.38766	2457.68869	2644.27458	2742.31598
2.53E-15	697.020453	721.315985	822.630927	433.237831	411.260202	371.85574
2.99E-06	1331.24627	1345.47512	1322.11839	1083.09458	1032.96996	1001.22398
0.01032931	1149.14183	1146.25452	1150.39072	1274.30757	1320.5308	1368.27539
0.01040987	0	0	1.84653407	10.6972304	16.8680942	1.92173509
1.00E-13	116.170076	133.467992	100.636107	279.465144	257.037626	333.421039
0.00534825	95.2385304	74.5850542	55.396022	32.0916912	39.3588865	47.0825098
4.11E-07	3245.43607	3055.04307	3045.85794	2456.35153	2385.63047	2555.90767
2.67E-28	2826.80517	2855.82247	3008.92726	1706.20825	1740.62667	1549.87935
1.56E-21	768.187707	791.97551	745.999763	385.100295	418.489385	409.329575
3.16E-08	892.7304	976.47538	938.962573	687.297054	669.904313	626.48564
0.02024757	180.011288	185.481253	186.499941	149.761226	142.173937	122.030178
6.74E-44	645.738168	634.954343	589.967635	181.852917	195.187947	215.23433
0.00086659	262.690891	249.271102	226.200423	151.098379	179.123096	175.838761
0.04105069	126.635848	147.207344	115.408379	155.109841	196.794432	164.30835
1.78E-13	866.565969	795.901039	833.710131	494.746906	436.963964	531.359753
9.29E-09	1737.31825	1615.35525	1533.54654	1153.96373	1175.94714	1217.41918
3.71E-29	690.74099	810.621773	853.098739	1850.62086	1814.52499	1564.29237
8.85E-10	567.244873	588.829375	667.522065	346.322834	402.424533	339.186244
1.86E-06	1172.16653	1173.73322	1037.75215	762.177666	857.059835	849.406911
2.75E-07	214.548338	206.090281	202.19548	116.332381	106.028021	119.147576
0.00672716	85.8193351	90.2871708	63.7054253	110.983765	123.699358	136.443192
0.0353267	95.2385304	98.1382292	112.638578	172.49284	141.370694	116.264973
0.0598187	40.816513	73.6036719	34.1608802	70.8691514	73.8983175	89.3606818
0.00462951	920.987986	1039.28385	1118.07638	1260.93603	1309.28541	1178.98448
3.07E-16	258.504582	300.302981	288.982582	510.792752	561.466565	572.677058
0.003919	102.564571	81.4547302	106.175709	70.8691514	55.4237381	35.5520992
9.59E-24	2841.45725	2755.72148	2916.60056	1801.14617	1800.06663	1865.04391
2.40E-33	665.623135	776.273393	866.947745	1859.98094	1932.60165	1690.16601
1.43E-64	1367.87647	1393.56285	1328.58126	2836.10321	2857.93711	2873.95483
1.66E-15	52.3288628	49.0691146	56.3192891	199.235916	148.599878	182.564834
5.19E-16	12.5589271	21.5904104	19.3886077	106.972304	94.7826246	107.617165
5.67E-06	364.208885	344.465184	360.99741	533.524367	479.535821	512.142402
0.04476743	4154.91171	4585.01807	5008.72366	5364.66105	5328.71129	4874.48106
4.57E-10	1625.33448	1791.02268	1513.23467	970.773659	1000.03701	1188.59315
1.88E-10	1756.15664	1738.02804	1852.07367	2366.76223	2424.98935	2366.61677
0.08569468	1371.01621	1276.77836	1582.4797	1289.01626	1257.07464	1181.86708
6.53E-15	1093.67323	1192.37948	1050.67788	1643.36202	1731.79101	1786.25277
5.55E-13	1904.77061	1914.67685	1673.88313	1176.69534	1187.19254	1259.69735
0.0034932	132.915312	114.821728	146.799458	70.8691514	77.9145304	98.0084897
7.86E-05	3122.98653	3541.80869	3595.20183	4155.87401	4180.07439	4215.32593
3.52E-06	1252.75298	1171.77046	1227.94515	980.133736	928.548424	853.250381

0.00170963	712.719112	671.265488	539.187948	441.260754	498.010401	499.651124
0.00611145	4385.15871	4310.23103	3913.72896	4498.18539	4968.85861	5500.00584
3.00E-11	1608.58924	1478.94311	1680.346	2239.73262	2213.73655	2244.58659
0.00030801	253.271696	280.675335	271.440508	188.538686	186.352279	184.486569
0.00080202	4172.70352	4168.91198	3717.99635	2949.76128	3237.0676	3586.91855
1.90E-07	1225.54197	1388.65594	1338.7372	1688.82525	1820.14769	1766.07455
0.01752445	607.014809	612.38255	735.843826	853.104125	823.323646	716.80719
8.24E-27	7048.69783	7159.18382	8248.46768	4028.8444	3855.56439	3207.37587
2.81E-19	119.309807	116.784493	119.101447	292.836682	326.116488	291.142867
3.59E-10	332.811568	352.316243	373.923149	208.595993	197.597675	171.034423
0.00513473	10103.6568	9153.35264	7692.66093	10114.2313	10838.1522	12430.7434
1.48E-14	582.943532	568.220347	603.81664	919.961815	915.696543	959.906679
1.16E-16	637.365549	620.233608	552.113686	243.361992	293.986785	322.851496
2.36E-07	1693.362	1627.13184	1478.15052	1079.08312	1136.58825	1249.12781
1.40E-11	2201.99855	2017.72199	2459.58338	3703.91603	3447.51716	3143.95861
2.52E-11	1298.80238	1254.20657	1166.08626	1703.53394	1806.49257	1941.91331
0.01514858	829.935765	824.361125	742.306695	914.6132	952.645702	935.88499
2.74E-15	153.846857	145.244579	164.341532	382.425987	387.966167	305.55588
1.56E-10	854.007042	850.858447	618.588913	401.14614	391.98238	455.451217
3.58E-18	648.877899	783.143069	808.781922	370.391603	383.146711	330.538436
0.02368991	98.3782622	99.1196115	144.029657	179.178609	183.139309	133.560589
5.54E-08	590.269573	640.842637	681.371071	454.632292	347.804038	399.720899
5.12E-07	2388.2893	2409.29353	2483.58832	1869.34101	1977.58324	1938.06984
1.40E-07	147.567393	136.412139	131.103919	49.4746906	68.2756194	64.3781256
0.00304473	1724.75932	1665.40575	1814.21972	2044.50816	2058.71074	1957.28719
0.01537276	996.341549	1050.07905	851.252205	1040.30566	1210.48657	1259.69735
0.07907935	666.669713	622.196373	627.821583	561.604596	530.140104	588.050938
1.40E-18	1383.57513	1427.91123	1364.58868	867.812817	899.631691	846.524308
1.14E-05	878.078319	877.355769	924.190301	1172.68388	1259.48437	1089.6238
0.0076764	1852.44174	1891.12368	1841.91773	2127.4117	2137.42851	2069.7087
7.29E-05	919.941409	910.722767	951.888312	1104.48904	1200.04442	1184.74968
0.00325427	399.792512	368.018359	423.779569	311.556836	318.084062	285.377661
0.0001034	1918.37611	2009.87093	2032.11074	2356.065	2379.20453	2384.87325
0.00019872	109.890612	85.3802594	103.405908	173.829994	152.616091	170.073556
7.08E-12	951.338727	1050.07905	1054.37095	653.868209	671.510798	680.294223
1.70E-46	1782.32107	1843.03594	1789.29151	905.253123	881.157112	894.567686
1.91E-05	8.37261806	9.81382292	10.1559374	36.1031526	43.3750994	33.6303641
9.02E-06	6540.06128	6199.39194	6076.02035	4983.57222	5197.78275	5343.38443
0.00579791	518.055742	499.523587	455.170648	386.437448	379.130499	409.329575
4.84E-05	1660.91811	1855.79391	1813.29645	2194.26939	2203.2944	2160.03024
5.83E-36	515.962588	599.62458	541.957749	153.772687	178.319853	193.134377
0.04260723	16.7452361	17.6648813	10.1559374	4.0114614	4.81945549	6.72607283
9.68E-08	1991.63652	1915.65823	1715.43015	1271.63326	1269.12328	1458.59694

7.27E-19	3619.06415	3542.79007	3338.53359	2215.66385	2228.19492	2420.42535
6.88E-08	5823.15586	5691.03591	4992.10485	3746.70495	3947.13405	4345.04305
0.00355899	265.830623	292.451923	232.663293	149.761226	157.435546	224.843006
9.08E-08	1447.41635	1415.15326	1395.97976	1846.6094	1798.46014	1845.82656
0.01024195	1492.41917	1601.6159	1623.10345	1846.6094	1801.67311	1759.34848
8.83E-07	1755.11006	1823.4083	2082.89043	2483.09461	2592.06381	2444.44704
0.00128912	3035.07405	3190.47383	3469.63751	3953.96379	3882.0714	3657.06188
7.38E-10	3241.24976	3084.48454	3094.7911	3956.6381	4226.66246	4499.74272
4.49E-09	54.4220174	36.3111448	48.9331528	5.3486152	5.62269807	6.72607283
0.00187803	1072.74169	1040.26523	1068.21996	897.2302	872.321444	896.489421
0.00874673	24.0712769	31.4042333	39.7004825	8.02292281	14.4583665	15.3738807
8.53E-10	5200.44239	5268.06014	5788.8843	4094.36494	4168.829	3862.68754
2.34E-09	60.7014809	61.8270844	45.2400847	6.685769	12.0486387	7.68694037
3.21E-17	3368.93219	3482.92575	3874.02847	5589.30289	5492.57277	5227.11945
0.0105774	2008.38176	2324.89465	2168.75426	1805.15763	1904.48816	1904.43948
0.0829877	16.7452361	27.4787042	32.3143462	33.428845	48.9977975	44.1999071
1.81E-05	151.753702	159.965314	155.108862	72.2063052	99.6020801	77.8302713
5.62E-43	1014.13336	1129.57102	950.965045	2190.25793	2228.99816	2307.04298
3.34E-10	1004.71417	911.704149	913.111096	1334.47949	1313.30162	1380.76666
3.14E-37	1425.43822	1569.23028	1379.36095	640.496671	624.119486	702.394176
4.07E-06	2168.50808	2113.89746	2371.87301	1799.80902	1711.70994	1718.03117
5.17E-15	145.474239	186.462635	179.113805	389.111756	371.098073	354.560125
0.01300893	557.825678	517.188468	513.336471	442.597908	430.538024	441.038204
0.00672162	309.786868	362.130066	434.858773	566.953212	489.977975	437.194734
0.0012364	1272.63794	1284.62942	1413.52183	1635.3391	1685.20294	1501.83598
0.00093606	3176.36197	2860.72938	2502.05366	2027.12516	2147.06742	2479.99914
0.00068946	3395.09662	3217.95253	2922.14016	3776.12233	3712.58721	3921.30046
0.04195268	1328.10654	1345.47512	1504.002	1226.17004	1277.1557	1164.57147
1.11E-13	4669.82772	4732.22541	4466.76591	6109.45572	6395.41743	6799.09876
6.37E-05	418.630903	426.901297	467.173119	295.51099	328.526216	310.360218
0.00465651	3888.03451	3995.20731	3913.72896	3477.93704	3464.38525	3556.17079
0.00313602	35.5836267	27.4787042	27.698011	4.0114614	12.8518813	12.4912781
6.54E-06	547.359905	523.076762	495.794397	696.65713	698.017803	729.298468
5.60E-20	662.483404	651.637842	673.984935	1120.53489	1134.98177	1297.17119
2.99E-10	84.7727578	89.3057886	97.8663056	20.057307	30.5232181	17.2956158
0.00268124	391.419894	387.646005	331.452865	268.767914	255.431141	302.673277
0.00179517	30.3507405	23.553175	27.698011	64.1833824	52.2107678	53.8085826
9.26E-19	37318.8518	38058.0053	41892.3184	61645.4645	63289.8927	55915.7651
1.19E-19	9448.49948	9199.4776	10703.4347	17202.4836	17311.4841	14849.2471
2.92E-27	5524.88134	5494.75945	5565.45368	3738.68203	3745.52016	3695.49658
0.01000536	5419.17704	5358.34731	5643.00811	4978.2236	4866.8468	4937.89832
1.32E-18	573.524337	540.741643	555.806754	216.618916	278.725176	277.690721
1.19E-16	3903.73317	3672.33254	3737.38495	2646.22737	2697.28859	2515.55124

0.00680349	2177.92727	2115.86022	2129.05378	2543.26653	2551.09844	2298.39517
0.00185989	6081.66044	6649.84641	7545.86147	8958.93047	8756.95062	7448.64522
1.53E-17	207.222297	193.332311	185.576674	44.1260754	63.4561639	63.4172581
7.63E-10	79.5398715	92.2499354	61.8588913	204.584532	167.8777	195.056112
1.44E-17	15.6986589	8.83244063	12.0024714	97.6122275	92.3728969	93.204152
3.84E-24	10809.0499	10497.8464	11160.4519	16909.647	15983.7241	15635.2367
2.15E-19	3960.24834	4144.37742	4584.94409	6911.748	6972.94885	6336.92147
1.06E-07	594.455882	546.629937	615.819112	954.727814	832.962557	804.246136
1.50E-11	1826.27731	1807.70618	1906.54642	2650.23883	2687.64968	2422.34708
7.28E-23	4243.87078	4063.90407	4290.42191	6366.18925	6254.04674	6024.63952
5.28E-05	6304.5814	6374.07799	6842.33199	5617.38312	5425.1004	5033.98508
7.01E-10	669.809444	772.347864	943.578909	1454.82334	1366.31563	1233.75393
1.53E-14	114.076921	110.896199	103.405908	239.35053	266.676537	325.734098
1.95E-36	934.59349	981.382292	965.737317	1863.9924	1954.2892	1769.91802
2.68E-05	374.674658	337.595508	393.311756	260.744991	255.431141	234.451681
4.90E-102	1336.47916	1173.73322	1191.93774	287.488067	278.725176	270.964648
6.62E-23	1761.38952	1740.97219	2000.71966	969.436506	1071.5256	941.650196
0.00312481	191.523638	178.611577	198.502412	102.960843	151.812848	121.069311
0.06653615	12.5589271	7.85105833	13.8490055	2.6743076	6.42594065	0.96086755
4.90E-26	1948.72685	2194.3708	2300.78145	1167.33527	1106.86828	979.12403
0.00241888	127.682425	92.2499354	86.7871012	52.1489982	56.2269807	65.3389932
0.01688655	698.06703	705.613868	662.90573	508.118444	493.994188	657.233402
0.00608919	605.968232	599.62458	501.333999	433.237831	424.915326	485.238111
0.01397863	403.978821	374.888035	347.148405	295.51099	310.051637	292.103734
1.06E-28	597.595614	634.954343	666.598798	280.802298	258.644111	273.847251
7.69E-10	263.737469	311.098187	336.0692	163.132764	131.731783	160.46488
1.64E-30	6544.24759	7451.63574	7717.58914	12993.1235	12930.5991	12425.9391
0.0387802	13.6055043	7.85105833	21.2351418	29.4173836	32.1297033	27.8651588
0.01460323	72.2138307	88.3244063	95.0965045	50.8118444	57.0302233	53.8085826
2.48E-05	76.4001398	106.97067	126.487584	48.1375368	49.8010401	30.7477615
3.28E-78	1624.2879	1504.45905	1587.09603	565.616058	519.69795	514.064137
0.0029918	358.975999	425.919915	397.928092	532.187213	554.237381	457.372952
1.34E-25	2784.94208	2855.82247	2252.77156	4938.10899	5492.57277	6015.03084
1.78E-06	65545.0404	66569.1236	57892.5361	78921.4916	78084.0146	87890.5545
3.85E-24	3442.1926	3455.44705	3314.52865	5027.69829	4992.15264	5205.0195
1.54E-15	2241.76848	2095.25119	2220.45722	1512.32095	1514.11227	1471.08821
1.05E-06	250.131964	277.731189	250.205366	171.155687	147.796635	139.325794
2.46E-09	6205.15656	6381.92904	6852.48792	5026.36114	5058.01854	4688.07276
0.01189323	14031.4613	12469.4434	12511.1916	14149.7615	14834.284	15227.8289
2.36E-23	53.3754401	69.6781427	61.8588913	217.95607	246.595473	223.882138
0.01748889	231.293574	292.451923	280.673178	183.190071	227.317651	195.056112
3.93E-20	665.623135	765.478188	795.856183	348.997142	367.08186	285.377661
6.92E-73	7448.49034	7332.88848	7827.45791	3702.57887	3690.89966	3503.32307

0.0139364	186.290752	173.704666	181.883606	124.355303	128.518813	145.091
0.00511191	3405.56239	3383.80614	3476.10038	3872.39741	3752.74934	3940.51781
4.83E-34	2115.13264	2036.36826	2029.34094	3352.24458	3539.89006	3481.22312
7.88E-79	2093.15451	2198.29633	2058.88549	838.395433	771.112878	768.694037
4.33E-08	2726.33375	2588.88649	2942.45204	2134.09747	2085.21774	1948.63938
1.16E-05	634.225818	628.084667	698.913145	480.038215	479.535821	479.472906
2.64E-20	3411.84186	3521.19966	3820.47899	2174.21208	2368.76237	2142.73463
4.63E-08	7587.68511	7492.8538	5724.25561	9438.96868	10765.8603	12240.4917
0.00469625	8532.74438	9219.10525	8858.74719	9738.49113	9977.87935	10227.4742
2.90E-16	2387.24272	2390.64726	2449.42744	1585.86441	1718.13588	1629.63136
1.03E-11	1366.8299	1324.86609	1372.89808	1958.93032	1833.80281	1872.73085
0.00241123	1565.67958	1580.02549	1452.29904	1276.98188	1284.38489	1332.72329
0.03235495	819.469992	746.831924	885.413085	1005.53966	963.891098	898.411156
0.03679898	991.108662	945.071147	956.504647	1053.6772	1068.31263	1177.06274
6.68E-09	252.225119	212.959957	279.749911	433.237831	411.260202	442.959939
0.06370597	15.6986589	17.6648813	17.5420736	22.7316146	37.7524013	31.708629
1.41E-208	2016.75437	1810.65033	1596.3287	13641.6431	14942.7217	17309.068
9.56E-06	78.4932943	74.5850542	66.4752264	121.680996	148.599878	150.856205
4.08E-11	4494.00274	4274.90126	3940.5037	5646.8005	6187.37761	6777.95967
3.77E-15	958.664767	930.350413	842.019535	478.701061	465.077455	565.950985
0.0022729	638.412127	609.438403	571.502294	476.026753	442.586662	515.025005
4.45E-05	4497.14247	5024.67733	5109.35976	4086.34202	4006.574	3707.02699
4.25E-17	3517.54616	3215.98977	3874.95174	2281.18438	2183.21334	2163.87371
3.81E-18	2643.65415	2609.49551	2530.67494	1580.51579	1745.44613	1754.54414
8.29E-50	9358.49383	10041.5036	10290.7344	5392.74128	5444.37822	5199.25429
3.19E-05	1181.58572	1185.50981	1287.03425	1627.31618	1639.41811	1437.45785
3.37E-29	1044.4841	1016.71205	1095.91797	533.524367	535.762802	544.811899
0.0343193	942.966108	952.922205	780.160644	736.771744	752.638299	780.224448
4.47E-05	1800.11288	1726.25145	1583.40296	1289.01626	1301.25298	1422.08397
0.00658382	177.918134	195.295076	165.264799	255.396376	247.398715	225.803873
0.00322955	572.47776	513.262939	594.58397	461.318061	440.176935	434.312131
8.02E-08	63059.4195	58904.5279	59987.429	49473.3535	49041.9758	50818.3628
4.03E-17	1643.12629	1382.76765	1420.90796	2338.682	2389.64668	2454.05571
0.00013166	123.496116	94.2127	113.561845	216.618916	208.843071	151.817072
2.24E-85	10278.4352	10292.7375	10219.6428	19312.5123	19393.4889	20008.1449
1.42E-71	3806.40148	3849.96273	3937.7339	7870.48727	7959.33074	7454.41043
1.17E-39	2392.47561	2333.72709	2192.75921	1192.74119	1181.56984	1237.5974
8.06E-29	863.426237	1095.22264	1212.24962	2832.09175	2555.11465	2382.95152
2.57E-85	3279.97312	3073.68934	2707.94221	8642.02501	8683.05231	9531.80606
4.84E-42	16639.5318	16447.9672	15054.7923	7970.77381	8412.35956	8994.6811
0.00109063	479.332384	509.337409	463.480051	651.193901	576.728174	631.289978
1.87E-15	1811.62523	1736.06527	1851.1504	2866.85775	2671.58483	2540.53379
2.76E-09	4942.98438	4618.38507	4943.1717	6569.43662	6178.54194	6075.5655

0.00046483	1729.99221	1551.5654	1811.44992	2377.45946	1996.86106	2065.86522
8.44E-10	2276.30553	2272.88139	2474.35565	3195.79758	3141.48174	3031.53711
0.00188376	672.949176	612.38255	734.920559	553.581674	533.353074	502.533727
1.09E-19	752.489048	593.736287	650.903259	328.939835	285.954359	300.751542
0.01012889	255.364851	265.954601	241.895963	184.527225	192.77822	203.70392
4.51E-09	1971.75155	2058.94005	2130.90031	1583.1901	1532.58685	1427.84917
2.60E-13	1622.19475	1715.45625	1831.7618	1168.67242	1080.36127	1175.14101
1.35E-146	5366.84817	5061.96986	5476.82004	1722.2541	1773.55962	1770.87889
0.01562233	2770.29	3003.02981	3183.42473	2750.52537	2600.09624	2419.46448
6.12E-59	949.245572	1041.24661	1236.25456	284.81376	261.857082	287.299396
4.32E-41	857.146773	860.67227	863.254677	322.254066	366.278617	350.716654
6.72E-11	1106.23216	1050.07905	1150.39072	1506.97233	1639.41811	1568.13584
5.84E-05	354.78969	363.111448	422.856301	513.46706	522.910921	548.655369
4.37E-21	361.069154	396.478446	347.148405	140.401149	117.273417	152.77794
4.09E-15	34.5370495	19.6276458	24.0049429	109.646612	118.076659	134.521457
0.00140889	8595.53901	9142.55743	9115.41542	10039.3507	10676.7004	10025.692
1.55E-22	2348.51936	2412.23767	2567.60562	4272.20639	4046.73613	3739.69649
2.01E-17	94.1919531	96.1754646	96.9430385	256.73353	243.382502	249.825562
1.73E-06	759.815089	758.608512	764.465104	1033.61989	974.333252	1005.06745
1.93E-06	235.479883	236.513132	311.14099	421.203447	418.489385	415.09478
1.11E-17	233.386728	237.494515	215.121219	560.267443	444.99639	498.690257
2.40E-11	1473.58078	1478.94311	1621.25691	1099.14042	1030.56023	976.241427
8.04E-89	1094.71981	1092.27849	1175.31893	3091.49959	3181.64387	2889.32871
5.70E-44	1652.54549	1805.74342	1860.38307	3433.81096	3542.29978	3381.2929
0.0607327	1326.01338	1200.23054	1164.23973	1301.05065	1355.07024	1584.47058
0.02054429	45.002822	49.0691146	42.4702836	22.7316146	28.1134904	24.0216887
3.64E-07	3396.1432	3215.00839	2690.40014	4114.42225	4291.72511	5052.24156
1.91E-13	410.258285	368.018359	356.381075	188.538686	183.942551	198.899582
0.035733	21.9781224	15.7021167	24.9282099	8.02292281	4.81945549	12.4912781
1.78E-07	1935.12135	2089.3629	2167.831	2620.82145	2774.39988	2648.15096
2.99E-05	91.0522214	88.3244063	112.638578	184.527225	167.8777	162.386615
0.01562233	737.836966	762.534041	750.616098	822.349588	884.370082	927.237182
2.50E-62	3666.16013	3859.77655	3905.41955	7587.01067	7904.71025	7315.08463
0.00568849	1085.30062	1062.83702	1078.3759	1236.86727	1264.30382	1243.36261
0.00024178	1686.03596	1471.09206	1445.83617	1833.23786	1889.22655	2105.26079
7.37E-06	2370.49749	2464.25093	2025.64787	1564.46995	1663.51539	1833.33528
3.73E-30	3751.97947	3559.47357	3597.97163	5579.94281	5987.3702	5923.74842
5.91E-17	721.09173	627.103284	715.531951	318.242605	377.524013	364.1688
8.00E-19	1001.57443	982.363674	937.116039	570.964673	574.318446	573.637925
0.00282855	208.268874	202.164752	266.824173	152.435533	167.074457	145.091
0.00823414	1880.69933	1691.90307	1704.35094	1501.62372	1445.0334	1580.62711
3.80E-06	3736.28081	3759.67556	3776.16217	2977.84151	3109.35203	3186.23678
0.00461837	152.80028	132.486609	156.955396	96.2750737	101.208565	100.891092

1.66E-33	265.830623	263.010454	242.81923	672.588362	620.906516	635.133448
5.71E-50	468.866611	422.975768	502.257266	1174.02104	1216.10927	1230.87133
4.53E-13	3.13973177	2.94414688	3.69306814	54.8233058	61.8496788	55.7303177
4.75E-13	450.02822	449.47309	512.413204	252.722068	257.037626	258.47337
0.00015171	369.441772	343.483802	348.071672	489.398291	470.700153	466.981628
3.77E-08	4258.52286	4316.11932	4613.56537	3439.15958	3541.49654	3305.38436
8.18E-06	257.458005	225.717927	258.514769	148.424072	108.437749	162.386615
4.27E-29	2609.1171	2787.12571	3227.74155	1458.8348	1412.9037	1267.38429
0.0001374	2738.89268	2845.02726	2857.51147	2345.36777	2375.18831	2418.50361
1.67E-08	435.376139	393.534299	458.863716	659.216824	679.543224	621.681303
0.03828172	456.307684	442.603414	510.56667	431.900678	366.278617	375.699211
1.24E-12	463.633725	479.895941	422.856301	254.059222	257.037626	223.882138
2.73E-07	791.212406	780.198922	740.460161	530.850059	525.320648	580.363998
2.92E-11	9577.22848	9774.56763	10519.7046	13423.687	13264.748	12750.7123
0.04707558	1004.71417	901.890326	902.955159	830.37251	784.768002	841.719971
3.86E-11	697.020453	646.73093	625.051782	980.133736	991.201346	1164.57147
5.83E-06	3496.61462	3515.31137	3063.40002	4113.08509	4298.15105	4872.55933
3.64E-20	2324.44809	2264.04895	2209.37801	1449.47472	1384.79021	1513.36639
1.67E-18	2277.35211	2219.88674	2481.74179	1528.36679	1480.37608	1455.71433
4.77E-06	424.910366	393.534299	454.247381	664.565439	573.515203	593.816144
0.02393634	1681.84965	1738.02804	1746.82123	1949.57024	1957.50217	1876.57432
1.98E-06	117.216653	104.026523	95.0965045	26.743076	51.4075252	44.1999071
0.00073465	2399.80165	2372.98238	2297.08838	1858.64378	2049.07183	2040.88267
0.0177462	3628.48335	4081.56895	4448.30057	4698.75846	4834.7171	4517.03834
1.29E-14	14077.5107	13235.903	13436.3051	10099.5227	10277.4888	10349.5043
0.00319999	16343.3504	16274.2625	16212.5691	17667.8132	18341.2411	18147.9054
8.73E-08	10728.4635	10326.1045	10734.8258	8936.19885	8462.1606	8551.72116
5.45E-05	35110.5738	33272.7852	32241.4081	39062.274	38672.1141	40594.7321
5.58E-15	3089.49606	3233.65465	3565.65728	2217.001	2248.27599	2110.06513
2.62E-12	4982.75432	5645.89232	6072.32728	9040.49685	8651.72585	7790.71407
1.60E-07	117.216653	129.542463	107.098976	56.1604596	48.9977975	43.2390396
0.00030482	3271.60051	3017.75055	3259.13263	3774.78518	3651.54078	3869.41361
2.65E-32	218.734647	196.276458	216.967753	36.1031526	34.539431	37.4738343
0.00186427	2779.70919	2782.2188	2648.85312	3024.6419	3174.41468	3368.80162
5.14E-64	2455.27024	2297.41595	2201.06861	4833.81099	5005.80777	5135.83704
1.32E-23	4566.21657	4808.77323	4569.24855	6691.11762	7099.86118	7115.22418
0.00204599	1683.94281	1722.32592	1742.20489	2093.98285	2184.81982	1871.76998
9.41E-17	2847.73672	2836.19482	2671.9348	1926.83863	1902.07843	1940.95244
1.77E-22	2602.83764	2879.37564	2898.13522	4516.90554	4271.64405	4435.36459
5.81E-05	125.589271	92.2499354	103.405908	46.800383	50.6042826	59.5737879
0.0034932	9121.96737	9750.03307	9919.58101	8357.21126	8771.40899	8080.89607
1.44E-11	281.529282	205.108899	221.584088	78.8920743	86.7501988	109.5389
5.87E-08	4074.32526	3974.59828	4125.15711	3356.25604	3151.92389	3192.00199

0.01264343	36.630204	52.9946438	38.7772154	20.057307	25.7037626	17.2956158
6.05E-26	1984.31048	2013.79646	2352.4844	1149.95227	1104.45855	1023.32394
1.63E-24	10477.2849	10233.8545	10820.6896	7128.36691	7366.53772	7133.48066
6.61E-05	1071.69511	1091.29711	1149.46746	1418.72018	1437.80422	1305.819
3.25E-13	1566.72615	1773.3578	1729.27915	2421.58553	2440.25096	2446.36877
6.82E-05	1305.08184	1278.74113	1328.58126	1553.77272	1616.12407	1604.6488
0.08699427	4998.45298	4836.25193	5461.1245	5899.52257	5530.32517	5374.13219
8.87E-07	1652.54549	1656.57331	1775.44251	2196.94369	2143.85445	2112.94773
5.47E-05	4566.21657	4403.46234	4573.86489	5288.44328	5338.3502	5222.31512
0.03258233	5987.46849	5897.12619	5401.11215	4880.61137	5062.03475	5470.21894
0.03537773	412.351439	467.137971	540.111215	631.136594	527.730376	583.246601
0.00925451	447.935066	514.244321	534.571613	379.751679	434.554237	366.090535
1.20E-09	858.193351	808.659008	828.170529	1167.33527	1136.58825	1201.08443
0.02983438	1817.9047	1765.50674	1682.19254	1347.85103	1584.79761	1620.02268
1.17E-13	3968.62096	4024.64878	3699.531	2650.23883	2768.77718	2869.15049
1.28E-07	506.543392	577.052788	638.900787	878.510047	894.812236	806.167871
5.24E-27	1755.11006	1622.22493	1706.19748	2865.5206	2730.22153	2750.00292
2.56E-07	3778.1439	3547.69699	3738.30822	4539.63715	4629.087	4508.39053
4.10E-13	2622.72261	2736.09383	2932.2961	3980.70687	3858.77736	3789.6616
4.31E-06	1136.5829	1076.57637	1158.70013	869.149971	847.420924	879.193805
4.77E-20	495.031043	453.398619	526.262209	219.293223	229.727378	226.764741
2.59E-14	16009.4923	15332.1355	15269.9135	11694.7471	11755.4552	12065.6138
3.15E-08	619.573736	727.204278	595.507237	899.904508	1015.29862	998.341381
0.07863677	504.450238	511.300174	502.257266	617.765056	560.663322	563.068382
1.99E-07	194.66337	212.959957	218.814287	129.703919	94.7826246	101.85196
0.00049074	120.356385	112.858964	112.638578	76.2177666	55.4237381	67.2607283
0.04203244	107.797457	102.063758	111.715311	140.401149	163.058244	127.795384
2.22E-31	143.381084	151.132873	162.494998	413.180524	474.716366	467.942495
7.86E-05	100.471417	122.672786	131.103919	203.247378	191.171734	189.290907
4.30E-18	528.521515	481.858705	519.79934	238.013377	267.47978	241.177754
2.39E-11	78.4932943	83.4174948	101.559374	24.0687684	11.2453961	20.1782185
2.03E-09	25.1178542	39.2552917	45.2400847	113.658073	122.896115	104.734563
0.00943781	1597.07689	1655.59193	1503.07873	1329.13088	1374.34806	1409.59269
0.00571329	41.8630903	61.8270844	48.0098858	76.2177666	85.9469562	96.0867546
3.30E-18	758.768511	715.427691	721.99482	419.866293	401.621291	406.446972
0.06036045	605.968232	695.800045	658.289395	520.152829	549.417926	610.150892
3.83E-15	144.427661	147.207344	138.490055	38.7774602	28.9167329	46.1216422
1.66E-06	3443.23918	3365.15988	3159.41979	2429.60846	2577.60544	2762.4942
6.87E-33	1066.46222	982.363674	1034.05908	1843.93509	1858.70333	1872.73085
6.03E-10	3125.07969	3188.51107	3127.10544	2386.81953	2454.70933	2462.70352
6.43E-28	1196.2378	1163.9194	1082.99223	2005.7307	1964.73135	1987.07409
3.38E-05	8623.7966	8859.91933	6740.77261	5153.39075	5527.91545	6607.88612
2.59E-05	16417.6574	16144.7201	13159.325	10718.6249	11017.2752	12617.1518

0.01301715	114.076921	79.4919656	125.564317	68.1948438	61.0464362	73.0259335
9.57E-11	37.6767812	21.5904104	28.621278	109.646612	118.076659	92.2432845
7.71E-10	260.597737	298.340217	313.910791	163.132764	142.97718	148.93447
2.90E-07	1261.12559	1313.08951	1244.56396	978.796582	899.631691	981.045765
2.41E-12	4239.68447	4209.14865	4241.48875	5380.70689	5522.29275	5610.5056
0.00054705	16.7452361	30.422851	25.8514769	61.5090748	69.078862	46.1216422
1.25E-12	3118.80023	3092.3356	2745.79616	1826.55209	1967.14108	2128.32162
8.90E-30	712.719112	690.893133	758.002235	302.196759	322.100275	263.277708
2.08E-23	8585.07324	8120.93847	8901.21747	13192.3594	12496.8481	12735.3385
0.0077985	2190.4862	1966.69011	2193.68247	1799.80902	1827.37687	1869.84825
1.41E-12	1314.50103	1161.95663	1252.87336	817.000972	836.175527	807.128739
0.05151417	0	0	0.92326703	2.6743076	6.42594065	3.84347019
1.21E-14	803.771333	743.887777	755.232434	1199.42696	1270.72976	1153.04106
0.00083772	2257.46714	2181.61283	2029.34094	2456.35153	2555.11465	2880.6809
3.70E-06	505.496815	461.249677	469.94292	624.450825	677.133496	691.824633
0.05958971	94.1919531	86.3616417	89.5569023	113.658073	113.257204	134.521457
9.64E-09	483.518693	504.430498	541.034482	336.962758	314.871092	324.773231
0.00110533	65.9343672	82.4361125	83.094033	32.0916912	40.9653717	45.1607747
4.78E-22	2517.0183	2350.41059	1920.39543	946.704891	1000.03701	1186.67142
6.09E-25	977.503158	968.624322	1010.05413	1862.65524	1717.33264	1640.2009
6.84E-06	1314.50103	1341.54959	1408.90549	1107.16335	1049.83805	991.615308
0.06502424	981.689467	989.23335	1022.05661	1127.22065	1102.85206	1106.91941
2.05E-99	17529.1225	17961.2587	16375.9874	6596.1797	6975.35858	7239.1761
0.00013958	7383.60255	7879.51842	8037.03953	9027.12531	8973.02288	9325.21954
3.36E-08	369.441772	370.962506	359.150876	537.535828	542.991985	563.068382
4.59E-12	3823.14672	3683.12774	3283.13757	2278.51008	2294.06081	2604.91192
7.40E-11	628.992931	640.842637	512.413204	320.916912	346.197553	347.834052
5.68E-21	463.633725	394.515681	419.163233	171.155687	179.926338	184.486569
9.99E-66	1790.69369	1689.94031	1642.49205	556.255981	616.890303	653.389932
1.60E-07	375.721235	397.459828	350.841473	545.558751	541.3855	610.150892
4.84E-20	1674.52361	1627.13184	1573.24703	2456.35153	2433.82502	2459.82092
9.50E-11	3580.3408	3770.47077	3793.70424	4996.94375	4988.93967	4699.60317
8.32E-12	1991.63652	2091.32566	2004.41273	2897.61229	2791.26797	2670.25091
5.41E-07	3564.64214	4006.9839	4220.25361	3023.30474	3059.55099	2892.21131
0.0257169	1944.54054	1966.69011	2142.90279	1810.50625	1841.83524	1681.51821
0.01253519	1084.25404	1061.85564	1244.56396	978.796582	964.694341	924.35458
1.74E-16	1132.39659	1227.70925	1260.2595	1949.57024	2055.49777	1801.62665
0.00011556	2828.89833	2779.27465	2667.31846	2261.12708	2176.7874	2404.0906
3.36E-22	1274.7311	1273.83421	1289.80405	2003.05639	2016.13888	1969.77847
3.02E-86	1013.08678	1084.42743	1095.91797	2943.07552	2888.46032	2701.95954
6.43E-177	2849.82987	3006.95534	3006.15746	8807.83209	8727.23065	8529.62121
7.53E-34	4928.3323	5109.07621	4583.09756	8310.41087	8422.80171	9172.4416
8.50E-08	1896.39799	2184.55698	2281.39284	1543.07549	1567.12628	1510.48378

0.0025945	2810.05993	2650.71357	2651.62292	2241.06977	2371.97534	2396.40366
0.08420268	1306.12842	1429.874	1529.85347	1640.68771	1621.74677	1514.32725
0.00105839	5693.38028	5584.06524	4722.51088	3919.19779	4248.35001	4686.15102
0.00114199	3497.66119	3271.92856	3188.96433	2614.13568	2800.90688	2958.51118
0.01484848	120.356385	102.063758	116.331646	78.8920743	74.7015601	77.8302713
2.91E-34	400.839089	406.292269	391.465222	133.71538	121.28963	114.343238
1.58E-69	551.546214	517.188468	506.873602	84.2406895	96.3891098	98.9693573
0.05813189	7.3260408	1.96276458	9.23267034	21.3944608	16.8680942	11.5304106
1.21E-11	945.059263	979.419527	984.202658	1418.72018	1563.11006	1353.86237
0.00859497	2.09315451	4.90691146	5.5396022	16.0458456	12.8518813	20.1782185
6.75E-59	10585.0824	9950.23506	10062.6874	17943.2669	18307.5049	18139.2575
2.24E-14	4966.00908	4259.19915	4253.49122	2977.84151	2933.44191	3044.02839
0.02285922	102.564571	78.5105833	88.6336352	64.1833824	45.7848272	65.3389932
8.08E-21	8195.7465	7694.03717	8306.6335	5666.85781	5501.40844	5394.31041
1.81E-17	8166.44234	7472.24477	6752.77509	4222.7317	4276.4635	4889.85494
8.97E-17	3722.6753	3806.78191	4171.32046	2644.89022	2558.32762	2329.14293
0.02563419	1994.77625	1947.06247	1740.35836	1548.4241	1617.73056	1760.30935
0.02912137	231.293574	257.12216	451.47758	238.013377	230.530621	172.956158
8.83E-47	6984.85661	7009.03233	7279.96056	4093.02778	4106.97932	3861.72667
1.21E-28	2421.77977	2365.13132	2430.03883	1466.85772	1424.95234	1492.2273
0.00081693	1878.60618	1924.49067	1654.49452	1350.52534	1415.31343	1593.11839
3.30E-05	658.297095	618.270844	774.621041	517.478521	487.568247	440.077336
6.30E-10	137.101621	158.002549	135.720254	315.568297	264.266809	270.964648
0.00143021	4173.7501	3734.15962	3705.07061	3119.57982	3214.57681	3438.94495
0.00057035	1670.3373	1580.02549	1573.24703	1242.21588	1314.10486	1406.71009
6.60E-05	1080.06773	1056.94873	1137.46499	1367.90834	1358.28321	1333.68415
1.62E-05	1672.43046	1514.27288	1469.84112	1051.00289	1122.12989	1286.60164
8.70E-11	969.13054	973.531234	974.969988	629.79944	694.001591	643.781256
1.20E-09	2038.7325	1981.41085	1892.69742	2686.34199	2523.78819	2687.54653
6.66E-07	192.570215	195.295076	227.12369	120.343842	102.011808	112.421503
2.63E-11	4012.5772	4158.11677	4281.18924	3143.64859	2934.24515	2644.30749
3.71E-15	2502.36622	2400.46109	2295.24185	1506.97233	1483.58905	1691.12688
0.00020481	398.745935	392.552917	459.786983	534.86152	600.022208	554.420574
0.00136882	7.3260408	5.88829375	5.5396022	22.7316146	23.2940349	23.0608211
4.26E-69	1547.88776	1631.05737	1738.51182	4016.81002	4041.91667	3714.71393
0.03241287	3.13973177	10.7952052	9.23267034	21.3944608	25.7037626	14.4130132
1.27E-07	2.09315451	4.90691146	1.84653407	24.0687684	44.9815846	35.5520992
0.04273174	7156.49528	6652.79056	6945.7379	7540.21028	7528.79272	7522.63202
1.37E-17	4485.63012	4403.46234	4149.16205	2714.42222	2727.81181	3059.40227
2.73E-16	1287.29003	1202.19331	1129.15558	684.622746	715.68914	762.928832
1.07E-23	2699.12275	2787.12571	2884.28621	1755.68294	1813.72175	1695.93122
4.83E-23	2748.31188	2816.56718	2885.20948	1814.51771	1857.09685	1842.94395
4.56E-10	1110.41847	1077.55776	1046.98482	770.200589	751.835056	751.398421

1.27E-62	2755.63792	3098.2239	3194.50394	7657.87982	7404.29012	6799.09876
0.00144159	369.441772	396.478446	328.683064	221.967531	269.086265	290.181999
3.33E-07	28145.6022	28664.214	26698.1128	20527.9852	21676.3043	23496.0941
7.27E-37	3997.92512	3800.89362	3676.44933	2118.05162	2065.13668	2226.33011
0.01209283	1070.64853	1062.83702	1142.08132	969.436506	904.451147	945.493666
0.00484815	1703.82777	1718.40039	1780.98211	1980.32478	2082.80801	1935.18724
1.37E-07	738.883543	863.616417	837.4032	534.86152	571.908718	574.598793
5.10E-12	827.84261	920.53659	1010.05413	1406.6858	1534.19333	1397.10141
1.01E-23	3098.91526	3003.02981	2727.33082	1621.96756	1621.74677	1813.15706
3.13E-63	36.630204	56.9201729	53.549488	505.444137	454.635301	389.151356
1.19E-14	540.033865	579.996934	532.725078	854.441279	932.564637	997.380513
3.43E-13	1760.34295	1823.4083	1904.69989	1298.37634	1274.74598	1245.28434
0.00336903	913.661945	887.169592	1068.21996	1322.44511	1257.87788	1057.91517
1.95E-19	2107.8066	2295.45318	2607.3061	1387.96565	1291.61407	1223.18439
2.77E-12	892.7304	960.773264	1032.21254	635.148055	589.580055	532.320621
1.61E-08	3762.44524	3748.88035	3701.37754	4702.76992	4631.49673	4582.37733
3.78E-25	1506.02467	1499.55214	1406.13569	2446.99146	2345.46834	2553.98594
6.44E-05	318.159486	341.521038	217.89102	446.609369	427.325053	617.837832
0.00018119	432.236407	368.018359	325.913263	221.967531	243.382502	281.534191
1.32E-18	14.6520816	17.6648813	13.8490055	100.286535	100.405323	117.225841
0.00905039	8648.91445	8607.70408	8224.46274	7454.63244	7517.54732	7890.64429
1.41E-10	974.363426	900.908944	823.554194	587.010519	569.49899	587.090071
7.31E-80	7377.32308	7583.14097	7097.15369	3107.54543	3357.55399	3377.44943
1.96E-14	489.798156	503.449116	357.304342	172.49284	181.532823	211.39086
2.72E-10	7.3260408	7.85105833	10.1559374	57.4976134	50.6042826	59.5737879
4.70E-08	654.110786	683.042075	765.388371	463.992369	490.781217	437.194734
2.37E-12	5838.85452	5920.67937	5607.92396	7369.0546	7756.11037	7778.22279
8.47E-05	2856.10933	2741.00074	3296.98658	2501.81476	2226.58844	2173.48239
1.06E-08	72.2138307	85.3802594	72.0148286	18.7201532	21.6875497	24.0216887
4.03E-07	133.961889	128.56108	134.796987	70.8691514	61.0464362	50.92598
0.00010517	34.5370495	32.3856156	23.0816758	80.2292281	61.0464362	71.1041984
2.45E-18	4542.1453	4904.94869	4854.53806	3227.88928	3357.55399	3113.21085
4.21E-20	4074.32526	4389.72299	4391.05801	2941.73836	2879.62466	2801.88977
2.61E-10	1029.83202	893.057886	867.871012	591.02198	610.464362	600.542217
0.00342165	85.8193351	105.989288	77.5544308	175.167148	153.419333	112.421503
0.02242539	2767.15027	2521.17111	2502.05366	2285.19585	2343.05861	2292.62997
0.02600822	3014.1425	3072.70796	2876.90008	3314.80427	3365.58642	3246.77144
0.0235434	2694.93644	2733.14968	2685.7838	2536.58076	2369.56562	2391.59932
8.02E-11	1153.32814	1067.74393	1118.07638	1539.06402	1573.55222	1544.11415
1.57E-27	2579.81294	2642.86251	2561.14275	3928.55787	4217.02355	4152.86954
0.02368076	1885.93222	1965.70873	2225.99682	2504.48907	2336.63267	2200.38668
7.60E-06	952.385304	835.15633	791.239848	518.815675	660.265402	594.777011
9.60E-32	817.376838	860.67227	951.888312	1992.35916	2101.28259	1723.79638

0.00352504	1274.7311	1365.10277	1647.10839	2000.38209	2041.0394	1544.11415
4.53E-08	2818.43255	2701.74545	2934.14263	2262.46423	2150.28039	2100.45646
6.84E-30	15094.7838	14980.8007	14919.9953	9658.2619	10049.3679	10296.6566
0.00375601	2370.49749	2412.23767	2397.72449	2638.20445	2808.93931	2740.39424
0.01042858	621.666891	517.188468	615.819112	734.097437	690.78862	715.846322
1.05E-06	1755.11006	1676.20095	1479.99706	1153.96373	1192.01199	1268.34516
3.66E-13	949.245572	832.212183	878.026949	580.32475	519.69795	515.985872
0.00340712	40.816513	52.9946438	31.3910791	72.2063052	68.2756194	96.0867546
1.44E-07	3668.25329	3447.59599	3606.28103	2833.4289	2918.98354	2739.43337
6.31E-12	2476.20179	2391.62865	1972.09838	1120.53489	1217.71575	1510.48378
2.11E-09	295.134786	267.917366	312.987524	159.121302	147.796635	164.30835
0.00175894	81.633026	95.1940823	75.7078968	41.4517678	40.9653717	54.7694501
7.48E-13	7461.04926	6718.54317	6946.66116	4991.59514	5252.40324	5167.54566
0.00092661	12547.4147	12272.1856	12307.1496	13839.5418	13752.3162	14130.5181
0.0007835	239.666192	210.997193	198.502412	314.231143	293.986785	300.751542
0.0149538	12.5589271	10.7952052	13.8490055	26.743076	29.7199755	27.8651588
5.47E-26	105.704303	81.4547302	113.561845	338.299912	330.935944	319.968893
0.00260006	2866.57511	2996.16014	2505.74673	2159.50339	2156.70633	2510.7469
1.52E-10	1944.54054	1998.09435	1749.59103	1153.96373	1256.2714	1378.84493
8.78E-29	5388.8263	4933.40878	4546.16687	2570.00961	2717.36965	2829.75492
6.22E-54	37605.614	34728.1752	39694.9429	80518.0533	76328.9296	73007.677
4.39E-43	8959.7479	8411.42762	8164.45038	4808.40507	4886.92787	4903.30709
4.46E-09	1694.40858	1414.17188	1426.44757	978.796582	1022.52781	1071.36731
1.09E-16	1816.85812	1987.29914	1961.01918	1236.86727	1278.76219	1141.51065
0.07027243	2894.83269	2996.16014	2505.74673	2888.25221	3214.57681	3414.92326
0.00214588	1681.84965	1690.92169	1731.12569	1917.47855	2014.53239	1982.26975
2.30E-07	1472.5342	1398.46977	1244.56396	870.487124	874.731171	1058.87604
2.64E-18	4570.40288	4381.87193	4192.5556	6407.64101	6207.45867	6780.84228
2.65E-06	1319.73392	1329.77301	1328.58126	1085.76889	1005.65971	933.963255
5.12E-05	7129.28427	6438.84922	5991.07978	8073.73465	7793.05953	8819.80321
0.00837015	5361.61529	5038.41669	5528.523	6022.54072	6034.76152	5857.44856
9.68E-58	1772.90187	1900.9375	1898.23702	829.035357	783.161517	804.246136
1.09E-12	11868.1861	11014.0535	11321.1004	8377.26856	8450.9152	8869.76832
1.76E-07	10815.3294	11166.1677	9977.74683	12967.7176	13761.9552	14707.9995
2.16E-07	8118.29978	7076.74771	7503.39118	9293.21892	9988.3215	9921.91828
7.16E-10	16511.8494	16433.2465	17928.9225	23210.3157	22853.0547	21091.0426
0.00136807	1397.18064	1435.76229	1407.98223	1623.30471	1691.62888	1656.53565
0.00294888	7646.29344	7526.2208	7936.40342	8617.95625	8666.18421	8607.45148
2.16E-13	23689.2762	23587.5234	24612.4526	30463.0379	31494.3384	30619.9661
0.00039691	279.436128	365.074213	363.767211	477.363907	464.274212	465.059892
0.00833081	31.3973177	21.5904104	26.774744	10.6972304	9.63891098	10.569543
0.00034398	27.2110087	10.7952052	21.2351418	58.8347672	41.7686142	64.3781256
9.11E-129	245.945655	240.438662	253.898434	1355.87395	1318.92432	1204.9279

0.00078244	9.41919531	4.90691146	9.23267034	0	0	0
8.34E-09	10372.6272	9787.3256	9543.81133	12372.6841	12272.7434	12904.4511
8.50E-07	1573.00562	1531.93776	1370.12828	989.493813	1073.13209	1171.29754
5.54E-65	1377.29567	1462.25961	1628.64305	506.781291	489.977975	498.690257
6.94E-05	0	1.96276458	4.61633517	20.057307	26.5070052	18.2564834
0.07564348	29.3041632	25.5159396	29.5445451	14.7086918	13.6551239	18.2564834
0.0125754	21.9781224	23.553175	17.5420736	2.6743076	5.62269807	11.5304106
1.53E-89	1557.30696	1596.70899	1736.66529	4547.66008	4389.72071	4147.10433
0.07833635	753.535625	662.433047	635.207719	599.044903	573.515203	611.11176
1.32E-24	1081.11431	1043.20938	1082.99223	612.416441	575.121688	551.537972
0.02131033	918.894832	899.927562	704.452747	926.647584	996.020801	1248.16694
7.56E-07	232.340151	239.457279	229.893491	358.357219	353.426736	370.894873
0.00052567	94.1919531	96.1754646	113.561845	48.1375368	65.0626491	49.0042449
4.33E-06	296.181364	275.768424	233.58656	136.389688	169.484185	163.347483
4.47E-41	4577.72892	4623.29198	4372.59267	2640.87876	2641.06161	2647.19009
7.06E-30	3975.947	3851.9255	4176.86006	2507.16338	2413.74396	2397.36453
2.88E-06	1994.77625	1926.45344	2057.96222	1394.65141	1526.16091	1644.04437
0.00518637	1251.7064	1329.77301	1313.80899	1488.25218	1534.99657	1490.30556
0.02066065	4677.15376	4536.93034	4464.91938	5012.9896	4967.25212	5060.88937
8.12E-14	1268.45164	1226.72786	1334.12086	1868.00386	1808.09905	1919.81336
1.24E-05	390.373317	377.832182	358.227609	254.059222	231.333864	271.925516
1.82E-08	52508.8741	52290.9927	48050.5095	62392.9335	65279.5246	69234.3502
0.05096478	2127.69156	2291.52765	2562.06602	2638.20445	2797.69391	2497.29475
1.39E-44	2329.68097	1971.59702	1828.99199	732.760283	795.210156	785.028785
3.24E-29	10766.1402	10715.7132	10325.8185	15474.8809	16189.3542	16450.0524
1.16E-13	27173.3319	28180.3925	25687.1354	35677.9377	37244.752	39654.0428
3.01E-73	16729.5375	15855.2123	16002.9875	29687.4887	30490.2852	30454.6969
1.50E-174	642.598436	639.861254	695.220076	2706.39929	2850.70792	2726.9421
0.00111034	2020.94068	1939.21141	1684.03907	1400.00003	1465.11447	1640.2009
4.07E-24	3731.04792	3403.43379	3054.16735	1659.40787	1704.48076	1941.91331
2.97E-34	6919.96882	7186.66252	8291.86123	3653.10418	3833.0736	3276.55833
0.00063229	347.463649	288.526394	364.690478	225.978992	252.218171	220.999536
9.38E-100	1551.02749	1586.89517	1492.92279	468.00383	430.538024	457.372952
6.98E-07	1496.60548	1514.27288	1283.34118	1863.9924	1927.7822	2202.30842
4.14E-11	261.644314	262.029072	225.277156	127.029611	115.666932	92.2432845
1.59E-10	3027.748	3255.24506	3451.17217	2436.29423	2253.89868	2102.37819
0.06366997	2.09315451	2.94414688	3.69306814	10.6972304	8.03242582	10.569543
3.91E-06	17904.8437	18063.3225	19785.6125	25095.7025	24918.9946	21546.4939
1.25E-11	196.756524	184.499871	244.665764	90.9264585	83.5372285	75.9085362
4.31E-10	1450.55608	1489.73832	1472.61092	1076.40881	1036.98617	1095.389
0.00391963	1622.19475	1509.36596	1886.23455	1478.8921	1324.54702	1236.63653
2.65E-06	9768.75212	10411.4847	10297.1972	11943.4577	12413.3109	12151.131
7.82E-69	11335.4783	11652.9333	11812.2784	21734.0979	22302.8335	21032.4297

0.00036892	2590.27871	2972.60696	3083.71189	3618.33819	3657.96672	3370.72335
1.54E-06	97.3316849	117.765875	100.636107	173.829994	195.187947	215.23433
6.92E-13	945.059263	875.393004	838.326467	1404.01149	1280.36868	1401.90575
2.44E-167	3937.22364	3775.37768	3455.78851	784.909281	752.638299	851.328646
2.14E-29	299.321095	308.15404	303.754854	92.2636123	87.5534414	70.1433309
1.97E-05	1075.88142	1127.60825	863.254677	669.914054	670.707556	787.911388
8.40E-71	2895.87927	3118.83292	3008.004	1343.83957	1311.69514	1253.93215
0.00480849	32.443895	11.7765875	16.6188066	4.0114614	4.01621291	6.72607283
5.74E-16	1217.16935	1151.16143	1098.68777	520.152829	555.843866	710.081117
0.00059885	3622.20389	3473.11193	3074.47922	2630.18153	2779.21933	2912.38953
2.32E-12	1936.16793	1802.79927	1705.27421	1286.34196	1214.50278	1177.06274
1.18E-05	231.293574	264.973219	216.044486	350.334296	363.868889	360.32533
0.02127077	2818.43255	2720.39171	2466.96951	2936.38975	2953.52297	3269.83226
3.58E-100	36402.0502	34870.4756	35693.5035	73830.9471	71482.1638	71146.4766
1.91E-98	1760.34295	1760.59983	1737.58856	577.650442	573.515203	529.438018
0.00096941	2060.71062	1817.52	1760.67023	2143.45754	2371.1721	2374.30371
1.96E-23	3063.33163	2999.10428	3021.853	1938.87301	1892.43952	2034.1566
4.93E-51	972.270272	891.095121	1091.30163	330.276989	303.625696	304.595012
3.11E-21	1000.52786	816.510067	866.024478	468.00383	418.489385	417.977383
9.04E-43	5872.34499	5587.00939	5501.74825	3278.70112	3019.38886	3037.30231
1.57E-07	2335.96044	2639.91837	2707.01894	3627.69826	3745.52016	3195.84546
9.62E-05	2794.36128	2881.33841	2857.51147	3305.4442	3323.01456	3637.84453
0.0033538	5619.07329	5719.496	4743.74602	5990.44903	6446.02172	7022.9809
0.00021584	346.417072	373.906653	388.695421	284.81376	266.676537	230.608211
0.00996543	508.636547	530.92782	522.569141	393.123217	470.700153	380.503548
0.02442507	1821.04443	1673.25681	1726.50935	1896.08409	1932.60165	2084.12171
0.00039036	1531.14253	1542.73296	1338.7372	1769.05448	1841.83524	1802.58752
6.94E-09	915.7551	948.996676	997.128396	643.170978	701.230774	627.446508
2.21E-24	1427.53138	1426.92985	1560.32129	842.406895	850.633894	824.424355
8.54E-11	2779.70919	2577.1099	3001.54113	4248.13763	3931.06919	3780.05293
0.00117475	239.666192	240.438662	203.118747	288.825221	310.854879	362.247065
6.55E-73	88686.9568	87343.024	74975.746	27459.7905	28521.5376	32384.1189
4.98E-16	3795.93571	3560.45495	3160.34306	1893.40978	2012.12267	2291.6691
3.26E-16	15428.6419	15078.9389	13233.1864	8565.80725	9063.78929	9996.86595
3.45E-06	142.334507	156.039784	106.175709	60.171921	58.6367085	68.2215958
0.00991209	230.246997	244.364191	204.042014	323.59122	304.428938	267.121178
1.22E-15	3699.6506	3500.59064	3585.96916	2497.8033	2555.91789	2626.051
4.92E-14	2778.66262	2781.23742	2988.61539	1946.89593	2074.77559	1944.79591
0.00053641	148.61397	135.430756	154.185595	101.623689	89.9631691	75.9085362
0.00492415	3.13973177	0	0	17.3829994	10.4421536	6.72607283
0	113.030344	86.3616417	79.4009649	2115.37731	2209.72034	2263.80394
1.49E-05	164.312629	170.760519	156.032129	97.6122275	78.717773	98.0084897
8.09E-28	73.260408	83.4174948	90.4801693	275.453683	293.183542	314.203688

0.0276889	16.7452361	23.553175	12.0024714	33.428845	29.7199755	42.278172
4.37E-09	930.407181	987.270586	972.200187	696.65713	685.165922	652.429064
3.26E-08	1611.72898	1632.03875	1431.0639	986.819505	995.217559	1194.35836
6.84E-17	1204.61042	1367.06553	1418.13816	791.59505	798.423126	711.041984
2.94E-07	344.323918	354.279007	295.445451	169.818533	208.843071	200.821317
0	3781.28363	3881.36696	4133.46651	29629.9911	28787.4109	26986.9259
0.01475962	1150.18841	1119.75719	1359.04907	1063.03727	1030.56023	937.806725
3.31E-26	208.268874	249.271102	227.12369	649.856747	572.711961	533.281488
6.67E-23	38.7233585	44.1622031	30.4678121	164.469918	179.123096	198.899582
3.35E-44	34.5370495	56.9201729	41.5470165	320.916912	298.002998	293.064602
2.73E-19	48.1425538	50.0504969	40.6237495	187.201532	207.236586	157.582278
0	527.474937	501.486351	506.873602	18963.5152	19055.3238	19372.0506
4.02E-06	724.231462	710.520779	757.078968	528.175751	485.961762	566.911852
2.10E-10	13.6055043	22.5717927	13.8490055	64.1833824	89.9631691	80.7128739
7.07E-10	1938.26108	1806.7248	1587.09603	2492.45468	2671.58483	3117.05432
2.06E-175	3429.63367	3420.11729	3672.75626	11796.3708	11122.5	10995.2073
1.02E-11	11170.1191	12703.0124	14177.6886	20358.1666	20555.7809	17875.019
7.62E-47	777.606902	754.682982	835.556666	1841.26078	1781.59205	1673.83127
1.05E-06	1180.53915	1200.23054	1348.89314	919.961815	931.761395	761.007097
0.0281066	8064.92434	8304.45695	7574.48275	8465.52071	8828.43921	9375.18465
0.08940716	7969.68581	7520.3325	7159.01258	6712.51208	6876.55974	7212.2718
0.01099562	2721.10087	2645.80666	2362.64034	2023.1137	2123.77339	2397.36453
9.23E-29	2536.90327	2313.11806	1796.67765	818.338126	838.585255	952.219739
1.45E-06	1663.01126	1584.9324	1392.28669	1933.5244	2149.47715	2256.117
4.81E-23	1964.42551	1853.83115	1699.73461	814.326665	792.800428	1024.2848
0.01046315	8348.54678	8224.96499	7641.88124	8767.71747	8961.77748	9751.84473
0.0351533	501.310506	405.310887	469.94292	568.290365	542.991985	537.124958
3.69E-22	2605.97737	2840.12035	2947.99164	1722.2541	1754.2818	1645.00524
1.13E-24	6158.06058	6234.7217	6322.53265	4113.08509	4324.65806	4260.4867
0.00019025	681.321794	628.084667	656.442861	508.118444	469.093668	526.555415
1.47E-09	591.31615	566.257582	545.650817	334.28845	381.540226	352.63839
0.01204897	24.0712769	26.4973219	25.8514769	10.6972304	5.62269807	13.4521457
6.16E-05	0	0	0.92326703	10.6972304	22.4907923	20.1782185
0.01481106	406.071976	368.999742	454.247381	537.535828	514.878495	474.668568
0.0047871	1261.12559	1341.54959	1445.83617	1131.23212	1194.42172	1022.36307
0.00057399	207.222297	276.749806	212.351418	389.111756	315.674335	327.655833
1.01E-11	1430.67111	1469.12929	1451.37578	1000.19104	1039.3959	1036.77608
1.32E-06	81.633026	100.100994	100.636107	37.4403064	39.3588865	38.4347019
0.00020247	6117.24407	6215.09405	7113.7725	5499.71358	5343.16965	4812.98554
8.62E-45	3256.94842	3395.58273	3180.65493	1759.6944	1701.26779	1797.78318
2.60E-20	1978.03102	1871.49603	1939.78404	1255.58742	1228.15791	1245.28434
1.93E-27	3763.49182	4363.22567	4189.7858	2374.78515	2413.74396	2354.12549
3.94E-09	542.127019	536.816114	553.036953	780.89782	786.374487	788.872256

0.02385316	2964.95337	3258.18921	3385.62021	2861.50913	2928.62245	2674.09438
0.00014035	768.187707	774.310628	858.638341	627.125133	634.561639	592.855276
0.00031908	1184.72545	1285.6108	1257.4897	992.16812	1028.1505	1023.32394
4.78E-11	692.834144	761.552658	872.487347	1248.90165	1261.8941	1172.25841
3.51E-17	1601.2632	1707.60519	1716.35342	2604.7756	2741.46693	2428.11229
0.00103155	1233.91459	1230.65339	1302.72978	1032.28273	1093.21315	926.276315
0.00232505	472.006343	470.082118	537.341414	403.820448	378.327256	370.894873
0.03580771	1388.80802	1516.23564	1705.27421	1948.23309	1837.01578	1620.98355
8.02E-11	2396.66192	2530.98493	2452.19724	3158.35728	3326.22753	3295.77568
6.43E-26	8590.30612	8721.54443	8908.60361	12438.2047	12859.9137	12536.4389
7.18E-07	274.203241	237.494515	262.207838	391.786064	391.179137	407.40784
7.33E-05	8116.20663	8581.20676	9884.49686	11983.5724	11634.1656	10387.939
7.76E-05	5751.9886	5467.28075	6018.77779	7294.17398	7287.81994	6493.54288
2.27E-07	744.11643	699.725574	663.828997	927.984738	982.365677	1102.11508
5.18E-05	540.033865	455.361383	499.487465	641.833824	670.707556	712.963719
3.44E-17	217.688069	209.034428	222.507355	54.8233058	75.5048027	78.7911388
1.65E-24	1442.18346	1440.6692	1516.92774	842.406895	879.550627	824.424355
3.31E-12	1700.68804	1513.29149	1846.53407	1123.20919	1085.98397	1017.55873
3.53E-09	7985.38447	7857.92801	7967.7945	9568.6726	9912.8167	9850.81409
1.71E-82	1455.78896	1391.60009	1349.8164	3300.09558	3534.26736	3373.60596
9.38E-13	614.34085	538.778878	603.81664	359.694372	324.510003	319.008025
0.03289562	392.466471	385.683241	415.470165	458.643754	506.846069	457.372952
1.97E-11	2828.89833	2668.37845	2601.7665	3546.13188	3672.42508	3863.6484
3.40E-30	2278.39869	2505.46899	2291.54878	1279.65619	1336.59566	1353.86237
3.58E-18	1810.57865	1834.2035	1822.52912	2763.89691	2685.23995	2588.57717
4.15E-29	836.215228	817.491449	878.026949	1612.60748	1544.63548	1488.38383
1.17E-09	3389.86374	3447.59599	3080.94209	4308.30955	4377.67207	4637.14678
4.57E-32	811.097374	861.653652	760.772036	334.28845	339.771612	368.973138
9.56E-13	1772.90187	1715.45625	1831.7618	1267.6218	1235.38709	1139.58891
1.39E-27	2731.56664	2688.0061	2605.45957	1603.24741	1634.59865	1690.16601
0.00015098	109.890612	122.672786	107.098976	48.1375368	58.6367085	71.1041984
0.02535462	19.8849679	15.7021167	24.0049429	10.6972304	8.03242582	3.84347019
1.97E-14	42449.1735	44129.8175	44543.9413	56232.6659	58161.1888	56302.0339
1.25E-39	4791.23068	4977.57098	5345.71613	8966.95339	9218.01187	8671.82961
5.14E-05	114.076921	74.5850542	94.1732374	41.4517678	43.3750994	44.1999071
5.43E-24	736.790389	793.938274	895.569023	394.460371	351.820251	321.890628
5.19E-78	9977.02099	10437.0007	11069.9717	4746.89599	4532.69789	4321.98222
4.74E-58	11343.8509	11672.561	11180.7638	6546.70501	6384.97528	6514.68197
0.00220073	64.8877899	50.0504969	64.6286924	29.4173836	24.0972774	34.5912317
2.50E-38	8409.24826	8702.89816	9018.47239	5391.40413	5205.81517	5124.30663
1.48E-14	3858.73035	3661.53733	3639.51865	2513.84915	2668.37186	2721.17689
5.30E-29	333.858145	343.483802	348.071672	114.995227	97.995595	110.499768
1.45E-11	619.573736	662.433047	664.752264	925.31043	1014.49538	1059.8369

4.43E-24	15554.2312	16965.1557	18421.0239	30385.483	31492.7319	27244.4384
8.94E-27	2140.25049	2227.7378	2564.83582	4401.91031	4666.8394	4067.35232
0.03191823	0	1.96276458	0	4.0114614	7.22918323	8.64780792
0.00428596	2834.13121	3007.93672	3197.27374	2532.5693	2693.27238	2562.63375
0.00680557	3486.14884	3610.50545	3401.31575	3868.38595	3927.85622	4005.8568
0.00272562	81.633026	63.789849	74.7846297	42.7889216	37.7524013	40.356437
2.24E-08	519.102319	587.847993	601.970106	965.425044	860.272805	793.676593
1.50E-06	1210.88989	1357.25171	1467.07132	1771.72879	1913.32383	1768.95715
1.05E-14	1852.44174	1792.00406	1580.63316	2575.35822	2660.33943	2733.66817
1.29E-05	5286.26172	5697.90559	6088.02282	7457.30675	7225.97026	6733.75977
0.00118265	54.4220174	51.0318792	66.4752264	21.3944608	31.3264607	24.9825562
2.79E-31	1733.13194	1752.74877	1883.46475	3381.66196	3268.39406	3018.08496
0.00629116	217.688069	232.587603	214.197952	280.802298	294.790027	291.142867
1.82E-10	512.822856	411.19918	505.950335	802.292281	738.179933	827.306958
1.10E-13	3475.68307	3658.59318	3717.99635	2699.71352	2590.45733	2531.88598
3.79E-08	990.062085	1058.91149	929.729903	1339.82811	1351.85726	1525.85766
1.55E-06	1773.94845	1706.62381	1496.61586	2171.53777	2151.88688	2231.13444
0.00493023	115.123498	115.80311	102.482641	65.5205362	69.8821046	76.8694037
1.06E-24	2216.65063	2025.57305	1994.25679	1231.51865	1208.88009	1238.55827
1.49E-18	2523.29777	2299.37871	2585.14769	4165.23409	4049.14585	3657.06188
2.23E-43	38.7233585	47.10635	46.1633517	307.545374	271.495993	270.964648
6.94E-08	341.184186	421.013003	345.301871	204.584532	204.826858	232.529946
9.77E-06	1237.05432	1388.65594	1376.59115	1640.68771	1747.05262	1729.56158
3.96E-143	5021.47768	4786.20144	4959.79051	11954.155	12182.7802	12111.7354
0.00020309	8331.80154	8210.24425	7315.96798	6358.16632	6372.1234	6935.54195
2.64E-86	5808.50378	5593.87906	5666.08979	2702.38783	2689.25616	2614.52059
0.02510555	72.2138307	59.8643198	69.2450275	97.6122275	98.7988375	97.0476222
0.00327086	692.834144	689.911751	739.536894	573.638981	600.825451	558.264044
3.18E-27	4843.55955	4667.45418	4942.24843	3054.05928	3173.61144	2977.72853
1.39E-11	4413.41629	4162.0423	4418.75602	5729.70404	5674.90884	5590.32739
4.58E-19	3441.14602	3502.5534	3390.23655	2396.17961	2386.43371	2424.26882
1.56E-15	1649.40576	1836.16627	1941.63057	1128.55781	1151.84986	1014.67613
0.00354412	1637.89341	1713.49348	1939.78404	2274.49862	2198.47495	1955.36546
1.04E-10	1113.5582	1192.37948	1359.97234	1878.70109	1861.11306	1753.58327
1.31E-06	257.458005	260.066307	217.89102	105.63515	146.19015	146.051867
0.00842871	523.288628	487.746999	586.274566	459.980907	427.325053	376.660078
1.79E-07	3084.26318	3159.0696	2970.15005	3737.34487	3843.51575	3977.99164
2.28E-05	215.594915	211.978575	187.423208	120.343842	103.618293	136.443192
9.77E-30	785.97952	817.491449	792.163115	382.425987	383.146711	349.755787
1.99E-08	511.776279	599.62458	577.965163	382.425987	330.132701	358.403595
1.12E-15	173.731825	170.760519	207.735083	53.486152	65.0626491	49.0042449
0.01942133	2405.03454	2106.0464	2237.07602	2693.02775	2541.45953	2468.46873
3.14E-14	565.151719	571.164494	653.67306	1012.22543	947.826246	998.341381

0.01067715	57.5617491	46.1249677	42.4702836	84.2406895	87.5534414	69.1824633
0.05309248	17.7918134	23.553175	36.0074143	37.4403064	58.6367085	40.356437
0.04128956	81.633026	65.7526136	82.170766	135.052534	110.044234	88.3998143
0.000256	21.9781224	12.7579698	9.23267034	0	0	0.96086755
8.49E-06	29.3041632	27.4787042	36.9306814	1.3371538	8.03242582	2.88260264
0.00053142	7.3260408	10.7952052	7.38613627	32.0916912	24.0972774	33.6303641
0.00016839	104.657726	102.063758	86.7871012	147.086918	175.106883	164.30835
0.0630979	7.3260408	19.6276458	16.6188066	1.3371538	7.22918323	5.76520528
0.00016097	56.5151719	70.659525	50.7796869	25.4059222	16.0648516	27.8651588
5.19E-15	2271.07265	2130.58096	2084.73696	1371.9198	1471.54041	1496.07077
0.00446509	411.304862	385.683241	446.861244	383.763141	252.218171	237.334284
2.71E-27	352.696536	322.874774	438.551841	1036.2942	976.742979	865.741659
3.60E-05	6.27946354	10.7952052	14.7722725	28.0802298	47.3913123	51.8868475
4.24E-24	1127.16371	1219.85819	1277.80157	681.948438	625.725971	632.250846
0.08939686	0	0	0.92326703	2.6743076	2.40972774	5.76520528
0.06952507	411.304862	414.143327	366.537012	454.632292	442.586662	501.572859
2.85E-05	653.064208	733.092572	684.140872	536.198674	517.288223	483.316376
0.01982252	1769.76214	1828.31521	1736.66529	1901.4327	2041.0394	2083.16084
4.83E-17	2319.2152	2339.61538	2235.22949	3342.8845	3446.71392	3875.17881
0.00754911	1418.11218	1394.54424	1530.77674	1751.67148	1779.18232	1572.94017
5.17E-10	276.296396	316.98648	235.433094	144.41261	130.928541	118.186708
2.69E-41	6595.52987	6576.24274	7186.71059	3788.15672	3729.45531	3468.73184
1.03E-06	8389.36329	7928.58754	9024.01199	7056.16061	6116.69226	6021.75691
1.36E-29	1114.60478	1001.00994	1126.38578	550.907366	498.010401	494.846786
0.03511635	693.880721	739.962248	867.871012	984.145197	894.812236	859.015587
1.07E-11	2638.42126	2656.60186	2856.5882	1949.57024	2011.31942	1811.23533
3.97E-05	3661.97382	3701.774	4106.69177	3191.78612	3149.51416	2944.09816
0.00643651	1192.0515	1323.88471	1294.42038	1416.04588	1521.34145	1522.97506
4.58E-20	2550.50878	2868.58044	2683.93727	1718.24263	1731.79101	1725.71811
1.71E-29	3074.84398	2899.98467	3076.32576	4892.64576	4931.90945	4676.54235
7.67E-05	1479.86024	1362.15862	1565.86089	2068.57693	1980.79621	1704.57903
5.34E-69	610.154541	484.802852	598.277038	2017.76509	2021.76158	1786.25277
1.01E-31	384.093853	419.050239	423.779569	1008.21397	966.300826	858.054719
1.73E-06	6.27946354	5.88829375	8.3094033	41.4517678	33.7361884	35.5520992
0.01641005	698.06703	665.377194	542.881016	498.758368	525.320648	519.829343
4.24E-15	7805.37318	8420.26006	8140.44544	10940.5924	10839.7586	11317.098
2.92E-05	63.8412127	47.10635	59.0890902	109.646612	122.092872	107.617165
7.55E-25	422.817212	453.398619	390.541955	135.052534	170.287427	139.325794
5.30E-09	5768.73384	5387.78878	6959.5869	10571.5379	9488.70462	8213.49579
1.11E-31	2346.42621	2259.14204	2171.52406	3657.11565	3765.60122	3766.60078
3.45E-06	10932.546	11406.6064	12802.944	9388.15684	9359.38256	8279.79565
6.73E-20	2264.79318	2439.71638	2501.13039	1453.48618	1577.56843	1467.24474
0.00303556	907.382482	987.270586	1106.99717	821.012434	839.388498	756.202759

6.39E-05	7464.189	7404.52939	7583.71542	8608.59617	8883.86295	8535.38642
0.06303377	1298.80238	1322.90333	1461.53171	1190.06688	1270.72976	1162.64973
3.70E-07	349.556804	394.515681	369.306814	538.872982	527.730376	594.777011
2.43E-05	844.587846	820.435596	955.58138	1170.00958	1202.45414	1088.66293
6.21E-07	341.184186	282.6381	285.289513	169.818533	181.532823	187.369172
0.00361497	2406.08111	2322.93188	2526.0586	2824.06883	2853.92089	2688.5074
6.22E-29	3073.7974	3086.44731	2870.43721	1771.72879	1779.98556	1857.35697
2.71E-08	3395.09662	3734.15962	4165.78086	5511.74797	5611.45268	4875.44193
0.00473386	161.172898	213.94134	246.512298	156.446995	125.305843	135.482324
0.00185268	545.266751	541.723025	574.272095	426.552062	459.454757	428.546926
1.32E-11	149.660548	120.710022	141.259856	44.1260754	36.9491588	51.8868475
7.49E-08	3292.53205	3123.73983	3824.17205	2584.7183	2401.69532	2175.40413
2.78E-14	3792.79598	4049.18334	3858.33293	2682.33052	2887.65708	2782.67241
2.37E-05	176.871556	182.537106	152.339061	112.320919	76.3080453	92.2432845
0.03225398	2074.31612	2371.01962	2402.34082	2636.8673	2591.26057	2525.15991
0.06151512	806.911065	855.765358	929.729903	974.785121	1024.93753	949.337136
7.47E-42	3380.44454	3670.36977	3601.6647	1932.18724	1978.38648	1910.20468
0.00031946	501.310506	463.212442	541.957749	362.36868	402.424533	316.125423
0.08730108	104.657726	98.1382292	87.7103682	106.972304	140.567452	130.677986
2.16E-05	2040.82565	2115.86022	2370.94974	2972.4929	3022.60183	2564.55548
8.76E-07	1830.46362	1946.08108	1970.25185	1460.17195	1552.66791	1449.94913
7.08E-09	1410.78614	1461.27823	1522.46734	1101.81473	1087.59046	1027.16741
2.08E-09	15.6986589	25.5159396	24.9282099	85.5778433	82.7339859	77.8302713
1.36E-20	1716.3867	1680.12648	1695.11827	1112.51196	1004.85647	992.576176
9.83E-23	2935.64921	2772.40497	3084.63516	1686.15094	1824.96715	1558.52716
1.29E-09	2689.70355	2585.94234	2447.58091	1779.75171	1808.90229	1998.6045
0.02000219	4313.99145	4443.69902	4401.21395	3838.96856	4095.73392	3952.04822
0.01570003	2453.17709	2872.50597	2945.22184	3301.43273	3197.70872	3092.07176
0.01408896	3354.28011	3732.19686	3503.79839	3066.09367	3067.58342	3232.35843
0.02563849	9.41919531	5.88829375	1.84653407	0	0	0.96086755
2.98E-10	778.653479	721.315985	848.482404	1128.55781	1170.32444	1179.94535
4.93E-06	3633.71624	3722.38303	3719.84288	3036.67628	3028.22453	3128.58473
0.00646928	605.968232	590.79214	605.663174	730.085975	704.443744	723.533262
1.75E-18	5632.6788	5850.01984	6642.90631	3645.08126	3611.37865	2976.76766
0.0020397	369.441772	357.223154	347.148405	275.453683	284.347874	251.747297
2.42E-228	1383.57513	1535.86329	1397.82629	6465.13863	6571.32756	6155.3175
2.12E-18	884.357782	869.504711	909.418028	1385.29134	1465.11447	1385.571
0.00014179	60.7014809	46.1249677	49.8564198	10.6972304	19.277822	24.0216887
7.63E-94	1910.00349	2067.77249	2083.8137	5323.20928	5086.13203	4942.70266
5.20E-10	1669.29072	1588.85793	1764.3633	2473.73453	2393.66289	2189.81714
0.00154985	1.04657726	0.98138229	0	17.3829994	11.2453961	8.64780792
3.33E-16	789.119252	833.193566	789.393314	411.843371	465.880697	485.238111
0.00342947	62.7946354	65.7526136	55.396022	33.428845	28.9167329	34.5912317

6.92E-14	1029.83202	918.573825	1104.22737	1638.01341	1643.43432	1546.99675
1.60E-62	2388.2893	2689.96886	2450.35071	5390.06697	5290.95889	5557.65789
4.08E-08	931.453759	843.007389	815.244791	570.964673	619.30003	593.816144
0.05374535	1558.35354	1588.85793	1620.33364	1537.72687	1381.57724	1325.99721
0.03645147	395.606203	398.44121	424.702836	361.031526	320.49379	336.303641
1.31E-118	589.222996	597.661816	617.665646	2317.28754	2349.48455	2076.43477
3.02E-22	1319.73392	1359.21447	1314.73226	750.143282	773.522606	818.65915
4.57E-22	1131.35001	1023.58173	1094.07144	566.953212	620.103273	597.659614
5.02E-05	888.544091	804.733479	919.573966	1119.19773	1176.75038	1079.05425
9.50E-05	23.0246997	15.7021167	19.3886077	1.3371538	1.60648516	2.88260264
0.00133186	183.15102	156.039784	163.418265	128.366765	89.9631691	103.773695
9.02E-06	111.983766	134.449374	176.344003	66.85769	69.078862	66.2998607
6.58E-05	3698.60403	3995.20731	4464.91938	5846.03642	5501.40844	4685.19016
2.11E-07	175.824979	196.276458	178.190538	62.8462286	95.5858672	98.9693573
5.07E-05	908.429059	860.67227	905.72496	1137.91788	1097.22937	1128.0585
0.00034943	2319.2152	2311.1553	2638.69718	3153.00866	2997.70131	2799.96803
2.05E-37	3036.12062	3116.87016	3134.49158	5288.44328	5207.42166	4982.09823
0.01623552	71.1672535	87.343024	111.715311	157.784149	134.944754	116.264973
1.05E-22	3671.39302	3301.37003	3482.56325	5904.87118	5385.74151	5416.41036
0.00175656	18.8383906	12.7579698	15.6955396	0	1.60648516	4.80433773
9.17E-06	18909.5579	19060.4069	20407.8945	22984.3367	23726.1794	22838.8607
0.00106919	562.011987	525.039526	493.024596	361.031526	388.76941	441.038204
0.009317	364.208885	353.297625	278.826644	229.990454	246.595473	270.003781
5.84E-17	246.992233	285.582247	278.826644	545.558751	512.468767	532.320621
2.64E-17	2630.04865	2843.0645	2806.73178	4010.12425	4200.15546	3908.80918
2.98E-06	1372.06278	1160.97525	1201.17041	1636.67625	1609.69813	1707.46163
0.00049761	167.452361	144.263197	116.331646	81.5663819	88.356684	78.7911388
4.48E-22	11143.9546	10885.4924	8925.22242	4559.69446	5066.05096	5829.5834
3.24E-22	25843.1322	24918.2778	22291.3593	13144.2219	14371.6163	15425.7676
1.79E-05	5.23288628	2.94414688	0	28.0802298	20.8843071	26.9042913
5.06E-198	13494.5672	13043.552	13025.4513	4643.93515	4695.75613	4667.89454
3.08E-18	8794.38869	8807.90607	9279.75696	6318.05171	6218.70407	5645.09684
5.27E-18	1698.59489	1675.21957	1497.53913	833.046818	885.976568	1014.67613
1.06E-07	185.244174	199.220605	132.950453	81.5663819	73.0950749	74.9476686
0.00383231	0	0.98138229	0	6.685769	10.4421536	7.68694037
0.00963113	15398.2912	14900.3273	15692.7698	17253.2955	17170.9167	16456.7785
0.0046984	64.8877899	67.7153781	40.6237495	100.286535	85.1437137	116.264973
1.97E-05	2080.59559	2146.28307	2278.62304	2725.11945	2966.37485	2562.63375
3.50E-20	496.07762	461.249677	556.730021	1000.19104	1000.03701	908.019831
0.01539323	3172.17567	2964.7559	2941.52877	2715.75937	2736.64748	2632.77708
0.03820547	2420.7332	2481.91582	2738.41002	2270.48715	2392.85965	1999.56536
2.36E-08	100.471417	126.598316	112.638578	50.8118444	39.3588865	38.4347019
3.41E-95	13204.6653	14171.1603	15062.1784	5353.96382	5530.32517	5029.18074

3.58E-05	12164.3675	13507.7459	13325.5131	10481.9486	11250.2156	10440.7868
3.06E-64	23203.6644	24241.124	21463.1887	47995.7985	51300.694	54113.1776
7.48E-159	5193.11635	5261.19047	5676.24572	16793.3146	17395.8246	16043.6054
0.00011442	11530.1416	11709.8535	12894.3474	10507.3546	9861.40917	9179.16767
4.27E-12	77.446717	63.789849	86.7871012	192.550147	209.646314	181.603966
9.26E-05	1191.00492	1234.57892	1462.45498	1762.36871	1688.41591	1610.41401
0.01914072	1699.64147	1622.22493	1180.85854	996.179582	1118.11367	1378.84493
4.01E-17	5014.15164	4843.12161	4274.72637	2949.76128	2884.44411	3142.99774
1.98E-31	1225.54197	1276.77836	1351.66294	577.650442	603.235179	472.746833
0.00876859	4402.95052	4319.06347	4415.98622	4900.66868	4892.55056	4789.92472
1.76E-06	2621.67603	2464.25093	2423.57596	2039.15955	2002.48376	1935.18724
6.13E-34	7591.87142	7688.14887	7514.47039	11401.9105	12077.5555	12039.6704
0.00342518	2573.53347	2440.69776	2318.32352	2675.64476	2935.85164	2965.23725
3.66E-35	25899.6474	25168.5303	26637.1772	17098.1857	16933.9601	16809.4169
0.00591889	938.779799	1105.03646	1218.71248	1426.74311	1330.97296	1276.0321
1.09E-48	4730.5292	4726.33712	4911.78062	2648.90168	2631.4227	2738.47251
4.17E-174	1186.81861	1214.95128	1175.31893	159.121302	141.370694	137.404059
2.02E-73	132.915312	130.523845	126.487584	607.067826	624.119486	688.942031
0.00031785	55.4685946	61.8270844	62.7821583	109.646612	121.28963	96.0867546
0.02563849	503.403661	550.555466	533.648346	616.427902	605.644907	648.585594
1.74E-30	6597.62303	6464.36516	6718.6142	4191.97717	3803.35362	3674.3575
0.08622029	1412.8793	1460.29685	1557.55149	1327.79372	1358.28321	1325.03635
4.27E-06	24252.3348	23209.6912	19164.2538	14340.9745	15884.9253	17808.7191
3.57E-06	99.4248394	90.2871708	53.549488	18.7201532	32.9329458	23.0608211
0.0003722	1538.46857	1488.75694	1744.05143	2032.47378	2004.89348	1876.57432
1.03E-62	636.318972	571.164494	582.581498	119.006688	137.354481	139.325794
1.44E-16	2047.10511	1920.56515	2141.05625	1337.1538	1349.44754	1244.32347
0.00110821	15.6986589	10.7952052	17.5420736	21.3944608	50.6042826	59.5737879
0.00169498	496.07762	414.143327	382.232552	246.036299	308.445151	348.794919
2.32E-35	3219.27164	3305.29556	3506.56819	5825.97911	5729.52933	5498.0841
2.54E-39	533.754401	554.480995	585.351299	197.898763	183.942551	185.447436
2.45E-14	29354.3989	29818.3196	27750.6372	20053.2956	20840.1288	22122.0535
0.00213365	66.9809444	61.8270844	72.0148286	28.0802298	28.1134904	44.1999071
3.74E-53	1651.49891	1917.621	1995.18006	691.308515	700.427531	629.368243
1.29E-05	3839.89196	3948.10096	4134.38978	4927.41176	4890.94408	4631.38157
1.24E-05	235.479883	174.686048	186.499941	106.972304	116.470174	110.499768
9.52E-47	2953.44102	3009.89949	2897.21195	1262.27319	1434.59125	1474.93168
1.10E-16	582.943532	642.805401	694.296809	1131.23212	1118.91692	1053.11083
4.25E-49	7202.54468	7722.49725	8119.21029	14719.389	14791.7121	14060.3748
1.92E-07	2827.85175	2930.40752	3014.46687	2404.20253	2253.09544	2189.81714
9.89E-07	659.343672	541.723025	510.56667	318.242605	308.445151	407.40784
0.00067489	6877.05916	6635.12568	6764.77756	5637.44042	6062.07176	5917.02235
0.06110963	1260.07902	1150.18005	1117.15311	954.727814	1070.72236	1072.32818

0.01458784	131.868734	106.97067	87.7103682	52.1489982	53.817253	90.3215494
0.01344882	731.557503	753.7016	790.316581	657.87967	665.8881	581.324866
1.86E-10	746.209584	790.012745	779.237377	1124.54635	1072.32885	1205.88877
2.17E-05	946.10584	924.462119	950.041778	746.131821	729.344264	725.454998
0.04594274	4386.20528	4256.255	3546.26868	4527.60277	4445.94769	5006.11992
0.00052069	155.940011	140.337668	123.717783	85.5778433	82.7339859	85.5172116
0.00700112	60.7014809	49.0691146	60.0123572	98.9493813	87.5534414	88.3998143
0.00095388	478.285806	381.757712	438.551841	328.939835	326.919731	301.71241
0.04781865	21.9781224	29.4414688	23.0816758	36.1031526	48.1945549	42.278172
0.06486337	57.5617491	52.0132615	36.9306814	37.4403064	30.5232181	21.139086
0.07858597	56.5151719	52.9946438	65.5519594	34.7659988	40.9653717	41.3173045
0.00152126	11.5123498	15.7021167	4.61633517	25.4059222	47.3913123	30.7477615
0.00608707	16.7452361	2.94414688	8.3094033	28.0802298	24.0972774	34.5912317
6.77E-08	99.4248394	94.2127	115.408379	188.538686	219.285225	198.899582
3.11E-07	11469.4402	11723.5929	12188.9714	9898.94959	9735.30009	9451.09319
3.61E-25	94.1919531	111.877581	91.4034363	299.522451	316.477577	309.39935
0.00088587	111.983766	98.1382292	72.0148286	141.738303	163.058244	160.46488
1.84E-06	635.272395	646.73093	591.814169	838.395433	859.469562	832.111295
9.12E-05	122.449539	109.914817	112.638578	73.543459	56.2269807	53.8085826
0.00181452	56.5151719	47.10635	49.8564198	18.7201532	20.0810645	29.7868939
8.89E-15	1959.19262	1836.16627	2105.04884	1341.16526	1244.22276	1211.65398
2.69E-10	18.8383906	10.7952052	18.4653407	89.5893047	77.1112878	63.4172581
0.0210898	9.41919531	2.94414688	12.0024714	12.0343842	28.1134904	29.7868939
0.00179512	59.6549036	66.7339958	49.8564198	28.0802298	29.7199755	27.8651588
0.00478735	20.9315451	34.3483802	24.9282099	42.7889216	69.8821046	52.8477151
2.09E-06	730.510925	695.800045	732.150758	528.175751	493.994188	544.811899
8.98E-10	223.967533	186.462635	190.193009	334.28845	366.278617	388.190489
0.0092478	73.260408	58.8829375	58.1658231	36.1031526	24.0972774	43.2390396
2.65E-06	11.5123498	7.85105833	10.1559374	40.114614	43.3750994	42.278172
0.01124246	261.644314	245.345573	286.21278	220.630377	199.20416	184.486569
0.00023548	776.560325	749.776071	718.301752	910.601738	926.138697	980.084897
0.08205764	484.56527	491.672528	411.777097	421.203447	316.477577	415.09478
6.09E-05	164.312629	168.797754	150.492527	247.373453	244.185745	246.942959
5.16E-10	765.047975	753.7016	752.462633	534.86152	486.765004	440.077336
0.00474279	86.8659123	105.007905	95.0965045	53.486152	48.9977975	69.1824633
4.20E-11	302.460827	320.912009	357.304342	516.141367	575.121688	588.050938
0.00078984	150.707125	102.063758	106.175709	205.921685	191.974977	177.760496
0.00328004	0	0.98138229	3.69306814	13.371538	13.6551239	11.5304106
2.48E-27	16.7452361	8.83244063	18.4653407	143.075457	150.206363	140.286662
1.88E-06	363.162308	418.068856	469.94292	655.205362	649.823249	580.363998
0.0403075	37.6767812	46.1249677	31.3910791	22.7316146	16.8680942	24.0216887
0.01217646	49.1891311	38.2739094	30.4678121	17.3829994	17.6713368	21.139086
1.86E-06	2.09315451	2.94414688	2.7698011	28.0802298	34.539431	21.139086

3.35E-05	41.8630903	17.6648813	44.3168176	98.9493813	81.1275007	92.2432845
0.0005573	115.123498	134.449374	87.7103682	49.4746906	60.2431936	68.2215958
0.00984264	124.542694	144.263197	121.871248	96.2750737	90.7664117	78.7911388
0.04776493	14.6520816	14.7207344	29.5445451	5.3486152	12.0486387	4.80433773
0.00021269	51.2822856	41.2180563	45.2400847	16.0458456	20.0810645	12.4912781
5.12E-15	2476.20179	2371.01962	2004.41273	1235.53011	1281.17192	1459.5578
0.00077004	8.37261806	4.90691146	1.84653407	18.7201532	32.1297033	20.1782185
8.57E-19	4484.58355	4759.70412	4823.14698	3253.2952	3244.29679	3046.91099
0.00281183	187.337329	193.332311	228.046957	139.063995	138.960967	146.051867
1.57E-44	1017.27309	1106.01784	1164.23973	415.854832	453.028816	414.133913
5.91E-19	177.918134	176.648813	146.799458	363.705834	428.128296	411.25131
2.60E-20	10126.6815	9814.8043	11029.348	6946.514	6422.72768	6074.60463
4.35E-12	1934.07477	1846.96147	1840.99447	1377.26841	1314.10486	1330.80155
7.76E-24	2987.97807	3320.01629	3186.19453	1909.45563	1951.87947	2015.90011
6.12E-29	546.313328	536.816114	523.492408	219.293223	220.89171	224.843006
2.44E-09	614.34085	555.462377	539.187948	950.716352	818.504191	849.406911
0.01331001	6030.37815	6260.23764	5751.95362	5202.86544	4995.36562	5671.04026
2.96E-08	1462.06843	1552.54679	1608.33117	1163.32381	1153.45635	1128.0585
1.69E-08	191.523638	198.239223	174.497469	334.28845	313.264607	320.929761
0.00127854	131.868734	154.07702	161.571731	92.2636123	85.9469562	104.734563
8.27E-06	4129.79386	3953.98925	3712.45674	3038.01344	3069.1899	3323.64084
2.00E-07	1015.17994	968.624322	1109.76697	1549.76126	1428.16531	1337.52762
5.42E-43	4072.23211	4043.29504	3657.06072	1892.07263	2007.30321	2059.13915
3.38E-22	478.285806	461.249677	416.393432	197.898763	175.106883	193.134377
0.0082731	2540.043	2533.92908	2862.1278	2330.65907	2352.69752	2111.026
1.77E-17	241.759346	247.308338	262.207838	101.623689	85.9469562	94.1650196
7.40E-08	116.170076	126.598316	146.799458	282.139452	257.037626	216.195198
0.00020763	300.367673	262.029072	289.905849	224.641839	166.271214	171.034423
2.23E-09	150.707125	159.965314	198.502412	81.5663819	67.4723769	53.8085826
5.71E-13	15.6986589	12.7579698	13.8490055	92.2636123	79.5210156	70.1433309
2.33E-05	73.260408	62.8084667	83.094033	171.155687	133.338269	125.873649
0.01084815	452.121375	345.446567	487.484994	303.533913	336.558642	334.381906
2.96E-13	58.6083264	52.0132615	51.7029539	161.79561	143.780422	200.821317
9.89E-13	1108.32532	1179.62151	1215.94268	1805.15763	1669.13808	1674.79213
0.03083073	18.8383906	8.83244063	15.6955396	29.4173836	29.7199755	30.7477615
0.00079597	52.3288628	37.2925271	48.9331528	74.8806128	99.6020801	88.3998143
5.97E-08	458.400839	418.068856	303.754854	160.458456	199.20416	226.764741
4.11E-05	125.589271	119.72864	129.257385	77.5549204	63.4561639	59.5737879
0.0323283	14.6520816	6.86967604	12.0024714	25.4059222	19.277822	31.708629
7.20E-09	1025.64571	911.704149	1020.21007	1329.13088	1404.06803	1396.14055
0.01202012	94.1919531	83.4174948	95.0965045	117.669534	144.583665	131.638854
0.00049384	174.778402	164.872225	144.029657	231.327608	240.972774	237.334284
0.01068603	50.2357083	41.2180563	75.7078968	24.0687684	27.3102478	33.6303641

0.0008952	439.562448	413.161945	473.635988	327.602681	356.639706	302.673277
0.03677878	9.41919531	12.7579698	11.0792044	4.0114614	1.60648516	3.84347019
3.71E-10	6.27946354	6.86967604	7.38613627	60.171921	42.5718568	55.7303177
4.68E-06	17.7918134	24.5345573	14.7722725	72.2063052	53.0140104	57.6520528
4.10E-09	165.359207	207.071664	187.423208	84.2406895	81.1275007	91.2824169
0.03049847	0	0.98138229	0	5.3486152	4.81945549	4.80433773
0.00348905	242.805924	259.084925	260.361304	201.910224	175.106883	185.447436
5.74E-05	0	0.98138229	0	17.3829994	22.4907923	13.4521457
2.56E-06	36.630204	24.5345573	21.2351418	0	0.80324258	3.84347019
0.02410303	61.7480582	40.236674	43.3935506	30.7545374	23.2940349	26.9042913
6.94E-05	17.7918134	9.81382292	9.23267034	30.7545374	48.1945549	49.9651124
0.03368111	21.9781224	17.6648813	14.7722725	33.428845	33.7361884	35.5520992
0.00148562	0	0	0	4.0114614	4.01621291	14.4130132
0.00078244	146.520816	121.691404	116.331646	204.584532	179.123096	204.664787
7.77E-09	148.61397	149.170108	144.952924	282.139452	306.838666	244.060357
0.00594454	9.41919531	22.5717927	15.6955396	1.3371538	5.62269807	1.92173509
7.47E-08	88.9590668	89.3057886	69.2450275	192.550147	178.319853	155.660543
1.61E-05	47.0959766	50.0504969	36.0074143	8.02292281	14.4583665	12.4912781
0.00653679	0	2.94414688	3.69306814	12.0343842	10.4421536	17.2956158
3.92E-05	4.18630903	5.88829375	5.5396022	25.4059222	34.539431	24.0216887
2.98E-10	10.4657726	3.92552917	4.61633517	49.4746906	60.2431936	49.0042449
4.34E-33	74.3069852	67.7153781	57.2425561	280.802298	334.952157	282.495059
0.07901714	0	0	0	2.6743076	4.81945549	0.96086755
1.18E-08	116.170076	109.914817	115.408379	50.8118444	34.539431	43.2390396
0.08008802	0	0	1.84653407	4.0114614	7.22918323	3.84347019
3.18E-53	45.002822	39.2552917	56.3192891	359.694372	338.968369	325.734098
4.45E-29	101.517994	103.045141	78.4776979	315.568297	332.542429	368.01227
2.29E-17	116.170076	119.72864	144.029657	316.905451	296.396513	338.225376
0.08603639	17.7918134	8.83244063	6.46286924	33.428845	24.0972774	13.4521457
0.0012603	40.816513	35.3297625	35.0841473	12.0343842	14.4583665	15.3738807
2.44E-05	13.6055043	19.6276458	12.0024714	45.4632292	47.3913123	51.8868475
0.07533946	0	2.94414688	0	6.685769	8.03242582	3.84347019
0.03977257	155.940011	111.877581	121.871248	77.5549204	94.7826246	102.812827
0.02408613	124.542694	101.082376	147.722725	86.9149971	84.3404711	87.4389467
0.07687303	195.709947	165.853607	204.965281	149.761226	160.648516	138.364927
2.90E-05	1012.04021	1024.56311	942.655641	790.257896	750.228571	774.459242
0.00502151	550.499637	461.249677	457.940449	574.976134	640.184338	632.250846
5.59E-79	177.918134	153.095638	170.804401	758.166205	798.423126	731.220203
0.02859793	36.630204	43.1808208	48.0098858	28.0802298	23.2940349	20.1782185
0.00240062	41.8630903	46.1249677	32.3143462	18.7201532	15.2616091	16.3347483
0.03880364	26.1644314	26.4973219	24.9282099	38.7774602	42.5718568	50.92598
0.00459557	1.04657726	0.98138229	6.46286924	21.3944608	14.4583665	12.4912781
0.06207836	6.27946354	9.81382292	8.3094033	4.0114614	0	1.92173509

8.08E-05	19.8849679	26.4973219	22.1584088	56.1604596	58.6367085	58.6129203
3.85E-22	364.208885	286.563629	288.059315	663.228285	730.950749	798.480931
0.0001919	262.690891	246.326955	228.046957	350.334296	363.065647	328.616701
0.00059953	38.7233585	46.1249677	40.6237495	77.5549204	85.1437137	77.8302713
0.00107536	13.6055043	25.5159396	16.6188066	1.3371538	4.81945549	2.88260264
0.00834356	137.101621	88.3244063	136.643521	60.171921	75.5048027	85.5172116
1.84E-06	0	0.98138229	1.84653407	20.057307	36.1459162	27.8651588
1.73E-23	38.7233585	54.9574083	36.9306814	187.201532	195.187947	217.156066
0.00016323	32.443895	45.1435854	35.0841473	65.5205362	82.7339859	97.0476222
1.25E-05	256.411428	241.420044	225.277156	137.726841	158.238789	147.973602
1.61E-06	13.6055043	16.683499	11.0792044	62.8462286	44.178342	50.92598
0.00017397	444.795334	332.688597	318.527127	211.270301	227.317651	264.238575
3.61E-28	383.047276	354.279007	349.918206	84.2406895	114.863689	119.147576
0.00193374	541.080442	593.736287	589.967635	493.409753	447.406118	383.386151
1.21E-17	408.16513	368.999742	444.091443	110.983765	172.697155	163.347483
2.69E-11	1295.66264	1298.36877	1477.22725	855.778433	946.219761	809.050474
1.32E-19	242.805924	292.451923	300.061786	595.033441	677.133496	572.677058
0.00992552	379.907544	369.981124	383.155819	318.242605	298.80624	290.181999
1.17E-07	367.348617	316.98648	343.455337	525.501444	523.714163	506.377197
1.72E-14	58.6083264	52.9946438	50.7796869	159.121302	168.680942	160.46488
4.29E-131	7888.05279	7781.38019	7841.30692	3139.63712	3221.00275	3032.49798
0.01595859	124.542694	115.80311	130.180652	101.623689	81.1275007	73.0259335
3.90E-105	6813.21794	6670.45544	6559.81227	2809.36014	2949.50676	2918.15474
2.87E-46	2062.80377	1932.34173	2009.02907	941.356276	1008.87268	968.554487
8.69E-26	1259.03244	1294.44324	1310.11592	752.81759	689.182135	662.998607
3.76E-20	2545.27589	2563.37055	2367.25667	1557.78418	1572.74897	1654.61392
0.01023953	813.190529	831.230801	914.95763	1032.28273	978.349464	1010.83266
1.79E-24	2972.27941	2890.17085	3063.40002	1890.73547	1917.34004	1932.30464
2.39E-12	976.456581	1081.48329	867.871012	517.478521	586.367085	600.542217
3.26E-33	283.622437	266.935983	274.210309	72.2063052	57.0302233	57.6520528
2.45E-15	536.894133	578.03417	631.514651	1044.31712	1020.11808	933.963255
0.00012448	329.671836	362.130066	349.918206	485.38683	457.045029	471.785965
5.14E-07	3561.50241	4084.5131	4257.18429	2951.09844	3114.97473	2758.65073
1.68E-10	9924.69213	10292.7375	9504.11085	7336.96291	7594.65861	7861.81827
1.53E-07	2535.85669	2810.67888	2694.0932	2128.74885	2024.17131	2078.3565
0.00053429	172.685247	151.132873	163.418265	109.646612	104.421536	95.1258871
0.00232862	1248.56667	1272.85283	1167.9328	1030.94558	1030.56023	1040.61955
0.00408239	216.641492	235.53175	262.207838	181.852917	177.516611	155.660543
6.93E-119	10169.5912	10904.1386	10853.9272	3650.42988	4059.58801	3866.53101
1.48E-22	7234.98858	7588.04788	7518.16346	10416.4281	10683.1263	10595.4864
1.03E-05	1983.2639	1970.61564	1842.841	2452.34007	2297.27378	2477.11653
0.01360988	273.156664	238.475897	335.145933	217.95607	223.301438	182.564834
1.39E-07	1754.06348	1799.85512	1729.27915	1382.61703	1375.1513	1259.69735

0.03268931	251.178542	283.619482	198.502412	183.190071	150.206363	210.429993
0.00013127	363.162308	343.483802	345.301871	217.95607	265.873295	245.021224
0.02099846	124.542694	162.90946	158.80193	109.646612	115.666932	89.3606818
0.07302605	10.4657726	11.7765875	11.0792044	2.6743076	4.81945549	3.84347019
0.00791767	223.967533	210.01581	213.274685	171.155687	167.8777	125.873649
7.10E-06	46.0493993	56.9201729	65.5519594	17.3829994	20.8843071	12.4912781
3.13E-05	864.472814	996.103026	1022.97987	1230.1815	1232.97736	1305.819
0.00668023	2070.12981	2156.0969	2257.3879	2489.78038	2485.23255	2427.15142
1.94E-11	2524.34434	2587.9051	2364.48687	3306.78135	3453.9431	3708.94873
1.25E-34	1409.73957	1376.87936	1117.15311	374.403064	392.785622	509.2598
5.52E-07	147.567393	141.31905	144.029657	252.722068	244.185745	239.256019
5.34E-09	9.41919531	12.7579698	13.8490055	78.8920743	58.6367085	49.0042449
3.16E-19	11474.673	11356.5559	10347.0536	15885.3872	16684.1517	17605.0152
0.07109968	149.660548	167.816372	132.950453	128.366765	97.995595	119.147576
0.00051908	540.033865	555.462377	641.670588	839.732587	772.719364	692.785501
1.19E-06	390.373317	399.422593	436.705307	292.836682	258.644111	242.138622
0.00502335	4911.58707	4941.25984	5479.58985	6390.25801	5879.7357	5614.34907
1.38E-08	2342.2399	2298.39733	2324.78639	2870.86921	2985.65268	3018.08496
7.24E-05	1143.90894	1271.87145	1407.98223	988.156659	981.562435	760.046229
0.00096465	50.2357083	52.9946438	54.472755	26.743076	24.90052	19.2173509
0.08585555	1360.55043	1469.12929	1299.95998	1445.46326	1511.70254	1674.79213
0.00016757	1870.23356	1930.37897	1978.56125	2348.04207	2238.63707	2318.57339
0.04128383	1233.91459	1229.67201	1093.14817	1295.70203	1353.46375	1387.49274
0.0009633	828.889187	758.608512	739.536894	905.253123	973.530009	1007.95006
9.88E-07	3298.81151	3055.04307	3001.54113	3758.73933	4001.75454	4040.44803
0.08848078	1614.86871	1523.10532	1467.99458	1331.80519	1386.3967	1440.34045
0.01429621	2163.27519	1913.69547	2085.66023	1821.20348	1803.2796	1767.03542
6.00E-23	18.8383906	14.7207344	16.6188066	124.355303	137.354481	118.186708
1.39E-17	1928.84188	2136.46925	2151.21219	1276.98188	1361.49618	1313.50594
4.53E-70	5773.96673	5823.52252	5275.54783	2055.20539	2184.01658	2400.24713
0.00294368	4558.89053	4308.26826	4301.50111	3891.11756	3638.68889	3943.40041
0.03419488	144.427661	134.449374	129.257385	102.960843	101.208565	98.0084897
6.10E-37	218.734647	209.034428	227.12369	605.730672	613.677332	567.87272
5.88E-17	8.37261806	19.6276458	19.3886077	96.2750737	110.044234	115.304106
3.62E-12	2222.93009	2181.61283	2569.45215	3812.22549	3906.97192	3251.57578
2.46E-06	27.2110087	27.4787042	22.1584088	1.3371538	1.60648516	0.96086755
1.60E-06	411.304862	362.130066	422.856301	276.790837	247.398715	252.708165
1.68E-06	35.5836267	47.10635	36.0074143	8.02292281	7.22918323	8.64780792
0.04128003	3.13973177	0	0.92326703	8.02292281	6.42594065	8.64780792
2.82E-20	1334.386	1307.20121	1356.27927	2095.32001	2009.71294	2041.84354
0.00011289	1409.73957	1303.27568	1240.87089	1540.40118	1700.46455	1751.66154
3.37E-06	229.200419	261.04769	208.65835	382.425987	350.213766	360.32533
4.63E-20	492.937888	548.592701	595.507237	1002.86535	1071.5256	989.693573

7.14E-05	72.2138307	71.6409073	90.4801693	29.4173836	32.9329458	40.356437
2.62E-05	1409.73957	1437.72506	1676.65293	2024.45085	2011.31942	1857.35697
7.97E-05	659.343672	619.252226	766.311638	891.881585	998.430529	878.232937
1.01E-14	835.168651	900.908944	945.425443	1617.9561	1376.75778	1425.92744
2.10E-17	4723.20316	4591.88774	4376.28574	2900.28659	3111.76176	3216.02368
0.00010248	1397.18064	1308.1826	1158.70013	1569.81856	1643.43432	1750.70067
1.77E-06	3279.97312	3671.35115	3737.38495	2800.00006	2861.95332	2703.88128
0.00085485	53.3754401	52.0132615	47.0866187	25.4059222	16.8680942	24.0216887
5.96E-171	2278.39869	2243.43992	2271.2369	6597.51685	6828.36519	7053.72866
8.15E-27	3719.53557	3943.19405	3739.23149	5791.21311	5975.32156	6322.50846
2.00E-26	2493.9936	2430.88394	2358.024	3705.25318	3822.63145	3947.24388
0.00680349	0	0.98138229	0	5.3486152	8.8356684	7.68694037
0.00021861	0	0.98138229	0	21.3944608	14.4583665	8.64780792
9.87E-60	4576.68234	4660.5845	4150.08532	1973.63901	1976.77999	2101.41732
4.37E-27	8160.16287	8156.26823	7692.66093	4542.31146	4627.48051	5178.11521
1.62E-28	378.860967	435.733738	430.242438	109.646612	142.97718	140.286662
2.46E-06	99.4248394	104.026523	106.175709	49.4746906	35.3426736	49.9651124
0.00010047	1122.9774	1054.00458	1110.69024	1268.95896	1432.18152	1437.45785
0.00078903	10.4657726	16.683499	12.9257385	36.1031526	35.3426736	40.356437
2.48E-23	708.532803	749.776071	789.393314	1412.03441	1295.63028	1319.27114
5.31E-09	178.964711	178.611577	182.806873	311.556836	317.28082	308.438482
2.07E-06	659.343672	687.948987	739.536894	521.489982	498.010401	474.668568
3.00E-07	1870.23356	1802.79927	2047.80628	2552.62661	2598.48975	2389.67759
1.77E-08	177.918134	249.271102	222.507355	112.320919	102.011808	78.7911388
0.0003547	1959.19262	2230.68195	2387.56855	2849.47475	2968.78458	2535.72946
1.64E-12	2303.51654	2301.34147	2566.68235	1651.38494	1649.05702	1439.37958
6.72E-10	2231.30271	2227.7378	2455.89031	1751.67148	1636.20514	1559.48803
1.20E-09	1286.24345	1234.57892	1228.86842	845.081202	920.515999	900.332891
2.71E-20	656.20394	670.284105	693.373542	308.882528	331.739186	368.973138
0.00144199	390.373317	431.808208	539.187948	346.322834	326.919731	310.360218
7.23E-05	115.123498	127.579698	138.490055	74.8806128	69.8821046	64.3781256
3.77E-12	11394.0866	9968.88132	10636.9595	16651.5763	14814.2029	14608.0693
4.18E-160	3355.32669	3211.08286	3194.50394	8874.68978	8702.33013	8907.24216
0.0123575	20.9315451	28.4600865	24.0049429	13.371538	9.63891098	4.80433773
1.91E-08	2199.90539	2203.20325	2195.52901	1727.60271	1659.49917	1731.48332
3.32E-10	4409.22998	3964.78446	3992.20665	5414.13574	5457.2301	5844.95729
1.68E-06	20.9315451	27.4787042	31.3910791	1.3371538	1.60648516	1.92173509
7.22E-25	2104.66686	2277.7883	2616.53877	1194.07834	1231.37088	1068.48471
5.81E-26	1061.22934	968.624322	974.969988	1691.49956	1746.24937	1780.48756
1.50E-38	5184.74373	4778.35038	4836.07272	2870.86921	2786.44852	2862.42442
7.32E-10	420.724057	394.515681	401.62116	231.327608	254.627898	218.116933
1.67E-12	1215.0762	1226.72786	1306.42285	1810.50625	1800.06663	1735.32679
7.04E-24	415.491171	420.031621	393.311756	173.829994	163.861487	151.817072

0.00096465	2250.1411	2336.67124	2363.56361	2910.98382	2685.23995	2617.4032
1.44E-06	775.513747	702.669721	792.163115	568.290365	541.3855	477.551171
5.81E-12	731.557503	740.94363	721.99482	473.352446	415.276415	472.746833
1.08E-37	788.072674	711.502162	723.841354	280.802298	302.019211	278.651588
4.55E-10	918.894832	949.978059	984.202658	685.9599	615.283818	562.107515
7.65E-23	3013.09592	3243.46847	3447.4791	5801.91034	5577.71649	5063.77197
9.88E-11	8461.57712	7964.89868	7904.08908	10159.6946	10736.1403	11004.816
0.00061682	4438.53415	4687.08183	5015.18653	4143.83963	3959.98593	3762.75731
3.31E-08	356.882845	378.813565	288.059315	176.504302	172.697155	206.586522
9.73E-17	5199.39581	5102.20654	5148.13698	7334.2886	6955.27751	6925.93327
7.13E-06	1554.16723	1508.38458	1536.31634	1243.55303	1203.25739	1228.94959
1.31E-08	1725.8059	1843.03594	2121.66764	2832.09175	2859.54359	2529.00338
3.58E-12	1026.69229	1112.88752	1084.83876	697.994284	664.281615	742.750613
2.50E-29	1503.93152	1485.81279	1596.3287	2568.67245	2583.22814	2521.31644
2.70E-24	2728.42691	2627.1604	2801.19218	1667.43079	1714.11967	1756.46587
1.44E-06	4464.69858	4163.02368	4481.53818	3614.32672	3543.10303	3488.91006
1.32E-05	6818.45083	6927.5776	7110.07943	8192.74134	8142.47005	8125.09597
0.03090324	415.491171	410.217798	329.606331	316.905451	298.002998	316.125423
4.88E-07	1041.34437	1022.60035	1243.64069	1635.3391	1563.91331	1462.44041
0.00099025	242.805924	211.978575	185.576674	128.366765	148.599878	145.091
9.61E-10	663.529981	656.544753	640.747321	410.506217	347.804038	442.959939
9.53E-05	14649.9884	14291.8703	14727.9557	12846.0366	12652.6771	12640.2126
2.17E-07	127.682425	142.300432	166.188066	57.4976134	73.8983175	62.4563905
0.00062775	2552.60193	2519.20834	2606.38284	3155.68297	2964.76837	2896.05479
1.44E-14	2693.88986	2529.02217	2607.3061	3552.81765	3604.14946	3632.07933
1.48E-77	4459.46569	4571.27872	4493.54065	8583.19025	8687.06852	8839.02056
9.69E-15	618.527159	632.991578	590.890902	1001.5282	980.759192	948.376268
2.76E-16	1337.52573	1424.96709	1465.22478	914.6132	894.812236	815.776547
0.07333588	6.27946354	0	0	10.6972304	8.8356684	8.64780792
0.04701632	2433.29212	2344.5223	2726.40755	2923.01821	2868.37926	2670.25091
0.01287917	3559.40925	3476.05608	3599.81816	3052.72213	3341.48914	2990.2198
0.00511191	7.3260408	7.85105833	1.84653407	16.0458456	24.90052	21.139086
1.33E-19	7357.43812	7584.12235	6870.03	4535.62569	4644.34861	5036.86768
0.00560964	100.471417	136.412139	126.487584	155.109841	171.09067	220.999536
3.11E-07	10566.244	11384.0346	11333.1028	13601.5285	13406.9219	13595.3149
7.39E-24	6241.78676	5817.63423	6484.10438	3953.96379	3855.56439	3540.79691
2.65E-23	2594.46502	2517.24558	2774.41744	4302.96093	4191.31979	3999.13073
1.10E-08	227.107265	234.550368	211.428151	121.680996	120.486387	102.812827
7.26E-31	1251.7064	1400.43253	1389.51689	2366.76223	2453.10284	2570.32069
3.09E-15	352.696536	316.005098	362.843944	627.125133	611.267605	613.033495
0.00136522	2940.88209	2683.09919	2803.96198	2430.94561	2411.33423	2400.24713
0.00606881	303.507405	299.321599	397.928092	262.082145	241.776017	235.412549
0.0093583	114.076921	141.31905	96.9430385	61.5090748	74.7015601	85.5172116

0.00408856	170.592093	158.002549	180.960339	139.063995	104.421536	101.85196
4.71E-06	1231.82143	1307.20121	1202.09368	909.264585	979.95595	979.12403
4.80E-24	15382.5925	15117.2128	14383.5771	9226.36123	9563.40618	10258.2219
4.50E-20	5420.22361	5501.62913	5475.89678	3927.22071	3871.62924	3713.75307
4.47E-19	316.066332	389.60877	411.777097	790.257896	740.58966	759.085362
0.00236942	2419.68662	2540.79875	2638.69718	2249.09269	2180.80361	2109.10426
7.69E-17	8172.7218	8813.79436	8646.39577	11531.6144	11912.0875	11913.7967
0.00163302	2658.30623	2805.77197	2787.34317	3151.67151	3298.11404	3090.15003
6.06E-09	129.77558	132.486609	176.344003	302.196759	314.067849	261.355973
3.12E-88	3614.87785	3637.98416	3588.73896	1567.14425	1612.10786	1539.30981
5.89E-47	5087.41205	5389.75155	5186.9142	2632.85583	2853.11765	2869.15049
1.50E-10	737.836966	727.204278	771.85124	449.283677	514.878495	429.507793
1.19E-07	347.463649	376.8508	426.54937	623.113671	620.103273	547.694501
8.69E-17	2020.94068	2082.49322	1873.30881	1301.05065	1293.22056	1315.42767
4.40E-05	128.729003	125.616933	156.955396	212.607454	238.563047	217.156066
0.00255171	1192.0515	1284.62942	1357.20254	1079.08312	1085.18073	1032.93261
4.29E-06	1792.78684	1986.31776	2187.2196	1482.90357	1520.53821	1499.91424
3.25E-05	6492.9653	6644.9395	5528.523	4333.71547	4490.12603	5264.59329
2.05E-08	1648.35918	1538.80743	1702.50441	2344.03061	2163.93551	2109.10426
2.17E-08	1682.89623	1593.76484	1963.78898	2689.01629	2635.43891	2321.45599
6.08E-08	1906.86376	1942.15556	1923.16523	2410.8883	2494.06822	2417.54275
3.85E-12	4642.61671	4507.48887	3886.03095	6192.35925	6310.27372	6946.11149
1.04E-17	1359.50386	1403.37668	1291.65058	2052.53108	2015.33564	2066.82609
1.41E-07	1953.95974	1970.61564	2208.45474	2842.78898	2737.45072	2585.69457
1.25E-05	2717.96114	2663.47154	3297.90984	4343.07555	4261.2019	3479.30139
2.53E-22	2171.64781	2109.97193	1788.36824	3473.92557	3849.13845	4205.71725
0.00067645	2785.98866	2907.83573	3302.52618	4154.53686	3827.4509	3368.80162
5.89E-15	2355.84541	2514.30143	2706.09568	4222.7317	4259.59541	3609.0185
9.85E-05	7272.66536	7278.91246	8669.47745	6446.41847	6160.06736	5442.35378
0.00174771	53.3754401	70.659525	63.7054253	22.7316146	35.3426736	31.708629
9.42E-05	3154.38385	2861.71076	3032.00894	3653.10418	3645.91808	3533.10997
0.013932	2556.78824	2896.05914	2611.92244	2285.19585	2352.69752	2416.58188
7.45E-05	2036.63934	2113.89746	2290.62551	2750.52537	2614.5546	2558.79028
0.00718913	545.266751	459.286913	555.806754	659.216824	602.431936	691.824633
9.20E-13	2457.3634	2495.65517	2356.17747	3346.89596	3302.13025	3246.77144
8.78E-05	2799.59416	2910.77988	2834.42979	2425.59699	2375.99156	2414.66014
0.01174452	432.236407	465.175206	520.722607	619.10221	578.334659	552.498839
1.99E-25	1185.77203	1117.79443	1119.92291	1905.44417	1890.02979	1869.84825
8.76E-29	1885.93222	2007.90817	2203.83841	1041.64281	1085.98397	1103.07594
2.86E-65	2612.25683	2757.68424	3283.13757	972.110813	922.925726	955.102341
9.83E-07	27.2110087	19.6276458	29.5445451	76.2177666	67.4723769	79.7520064
0.00262854	4534.81925	4512.39578	4848.99846	4147.85109	4065.21071	3935.71347
0.00507196	3020.42196	2963.77452	2958.14758	2487.10607	2698.89507	2651.03356

2.10E-86	589.222996	593.736287	617.665646	1714.23117	1804.88608	1693.04862
1.70E-05	1273.68452	1280.70389	1314.73226	1020.24835	1020.11808	1043.50216
2.86E-07	376.767812	358.204537	304.678121	533.524367	511.665524	549.616237
0.05471289	1077.97457	1006.89823	986.972459	881.184355	849.027409	964.711017
3.57E-14	2151.76284	2087.40013	2127.20725	2853.48621	3095.69691	3205.45414
7.12E-25	2118.27237	2108.00916	2050.57608	1222.15857	1282.7784	1320.23201
0.00026865	282.575859	264.973219	187.423208	357.020065	363.868889	395.877429
8.93E-06	125.589271	130.523845	130.180652	62.8462286	67.4723769	69.1824633
1.59E-12	610.154541	600.605963	627.821583	1000.19104	921.319241	921.471977
3.27E-08	739.930121	749.776071	767.234905	1184.71827	1110.08125	978.163162
2.07E-16	636.318972	592.754904	581.658231	996.179582	977.546222	990.65444
6.43E-91	807.957642	782.161687	813.398257	177.841456	150.206363	154.699675
1.27E-29	869.705701	845.951536	967.583851	1711.55687	1698.05482	1644.04437
1.84E-08	2671.91174	2658.56463	2658.08579	2111.36585	2100.47935	2089.88691
1.60E-50	93.1453759	98.1382292	100.636107	451.957985	434.554237	411.25131
3.35E-11	1013.08678	1054.98596	1246.4105	1753.00863	1802.47635	1615.21835
0.06850834	1510.21098	1652.64778	1842.841	1924.16432	1908.50437	1822.76574
1.30E-05	70.1206762	74.5850542	78.4776979	149.761226	139.764209	129.717119
1.84E-21	1929.88846	1938.23003	1704.35094	1048.32858	1016.10187	1124.21503
6.78E-05	4007.34432	3998.15146	4007.90219	3284.04973	3392.09342	3498.51874
0.00218016	65.9343672	85.3802594	60.0123572	37.4403064	32.9329458	39.3955694
0.04090757	5333.3577	5452.56001	5081.66175	5637.44042	5903.83297	5819.97473
1.23E-37	120.356385	167.816372	156.955396	528.175751	496.403915	555.381442
2.59E-08	1883.83906	1771.39504	1489.22973	1002.86535	1085.18073	1247.20608
1.11E-22	2809.01336	2903.9102	3044.01141	1863.9924	1865.12927	1718.03117
6.70E-06	697.020453	574.108641	569.65576	365.042988	435.357479	431.429528
3.03E-07	1439.04373	1649.70363	1708.96728	2386.81953	2247.47274	2041.84354
0.00524439	11450.6018	11229.9576	10190.0983	12208.2142	12254.2688	13549.1933
1.16E-06	531.661247	565.2762	667.522065	409.169063	382.343469	380.503548
4.08E-15	405.025398	411.19918	380.386018	203.247378	185.549036	205.625655
7.39E-12	219.781224	182.537106	186.499941	373.06591	371.098073	370.894873
6.04E-17	1742.55113	1657.55469	1720.96975	2529.89499	2612.94812	2425.22969
4.43E-18	501.310506	540.741643	483.791926	242.024838	261.053839	247.903827
6.30E-07	3039.26035	3036.39681	2587.9175	1961.60463	2092.44693	2276.29522
0.07052783	37.6767812	44.1622031	36.9306814	28.0802298	24.0972774	20.1782185
9.19E-08	1747.78402	1780.22748	1780.05884	2298.56738	2226.58844	2224.40837
5.20E-36	2356.89198	2364.14994	2272.16017	3800.1911	3940.70811	3989.52205
5.27E-18	679.22864	761.552658	760.772036	415.854832	387.966167	387.229621
0.06410218	3059.14532	3188.51107	3133.56831	2746.51391	3015.37265	2766.33767
5.89E-07	2270.02607	2233.6261	2122.59091	2655.58745	2919.78678	3078.61962
2.97E-08	1063.32249	1040.26523	1030.36601	1387.96565	1382.38048	1386.53187
1.78E-05	2024.08041	1968.65288	1630.48958	1182.04396	1340.61187	1496.07077
8.25E-20	1834.64993	1775.32057	2035.80381	3199.80905	3100.51637	2905.66346

2.19E-14	1616.96186	1628.11322	1687.73214	2290.54446	2363.13968	2543.4164
1.89E-08	11710.1529	11330.0586	10248.2641	8004.20265	8078.21064	8882.2596
3.95E-08	679.22864	655.563371	583.504765	854.441279	997.627286	1067.52384
4.32E-68	1780.22791	1784.15301	1883.46475	3814.89979	3984.88645	3789.6616
1.36E-05	4725.29632	4749.89029	5351.25573	3884.43179	4105.37283	3914.57438
1.36E-34	2102.57371	2176.70592	2082.89043	1211.46134	1195.22496	1189.55402
0.00180943	0	1.96276458	0.92326703	10.6972304	21.6875497	6.72607283
0.01778057	12.5589271	12.7579698	16.6188066	38.7774602	24.0972774	30.7477615
0.00446408	10.4657726	6.86967604	9.23267034	21.3944608	22.4907923	34.5912317
1.90E-06	167.452361	158.983931	198.502412	315.568297	286.757602	274.808118
6.10E-109	937.733222	825.342507	749.692831	100.286535	85.9469562	97.0476222
0.02727777	0	0.98138229	0.92326703	6.685769	7.22918323	5.76520528
1.12E-07	870.752278	912.685531	877.103682	1176.69534	1161.48877	1270.2669
4.19E-16	221.874378	218.848251	153.262328	0	0	0
0.08638408	0	0	0	4.0114614	3.21297033	0.96086755
0.01752535	3.13973177	8.83244063	12.0024714	17.3829994	25.7037626	24.0216887
0.0434185	206.17572	235.53175	195.732611	197.898763	151.812848	139.325794
0.08862132	10.4657726	22.5717927	12.9257385	17.3829994	24.0972774	49.9651124
2.72E-07	1221.35566	1349.40065	1260.2595	1758.35725	1650.66351	1649.80958
1.18E-07	216.641492	228.662074	220.660821	322.254066	378.327256	414.133913
4.86E-21	598.642191	662.433047	666.598798	1147.27796	1144.62068	1097.31074
0.04095336	13088.4952	11748.1274	15058.4853	12435.5303	11725.7352	9621.16674
1.40E-08	857.146773	932.313177	1032.21254	635.148055	646.610278	537.124958
1.04E-05	4782.85806	4563.42766	5151.83005	6109.45572	5845.99951	5868.97897
7.73E-12	288.855323	353.297625	364.690478	584.336211	646.610278	574.598793
1.78E-14	1238.10089	1336.64268	1193.78427	1851.95801	1856.29361	1890.02646
0.00078322	3620.11073	3578.11984	3417.93456	4004.77563	4046.73613	4305.64748
0.00034042	1.04657726	0	0	14.7086918	10.4421536	14.4130132
0.02108207	342.230763	369.981124	324.989996	264.756453	277.921933	290.181999
4.59E-27	72.2138307	89.3057886	78.4776979	312.893989	275.512205	274.808118
5.30E-09	622.713468	593.736287	577.965163	822.349588	876.337657	899.372023
3.07E-09	5007.87217	4768.53656	5103.82016	3964.66102	3879.66167	3781.0138
0.00736236	809.00422	849.877065	875.257148	976.122275	1050.6413	969.515354
3.23E-10	772.374016	693.83728	727.534423	465.329523	405.637504	491.003316
0.00823031	3823.14672	3669.38839	3850.9468	4292.2637	4244.3338	4152.86954
6.32E-17	700.160185	692.855898	736.767093	421.203447	371.901315	393.955694
0.02016732	2805.87363	2637.9556	2949.83817	2626.17006	2421.77638	2298.39517
0.00302035	620.620313	587.847993	582.581498	459.980907	498.813643	481.394641
6.95E-08	299.321095	304.22851	342.53207	497.421214	466.68394	509.2598
1.48E-13	5197.30266	5218.00965	4902.54795	6764.66108	6904.67323	7344.87153
4.72E-101	38.7233585	44.1622031	34.1608802	509.455598	531.746589	543.851031
8.19E-26	288.855323	265.954601	240.972696	73.543459	71.4885898	74.9476686
2.10E-06	15109.4359	15583.3694	16038.9949	12930.2773	13331.4171	12924.6294

3.29E-73	939.826377	1043.20938	963.890783	224.641839	265.070052	270.003781
6.92E-14	206.17572	207.071664	212.351418	85.5778433	86.7501988	75.9085362
0.00466715	6654.1382	6623.34909	5786.1145	4888.6343	5166.45628	5868.97897
2.96E-05	19.8849679	16.683499	27.698011	0	2.40972774	1.92173509
0.0018532	10.4657726	13.7393521	14.7722725	0	0.80324258	2.88260264
0.00871776	40.816513	45.1435854	42.4702836	21.3944608	23.2940349	19.2173509
0.01233049	1564.633	1747.84186	1607.40791	1900.09555	1865.93252	1867.92651
0.01440882	191.523638	168.797754	174.497469	125.692457	130.928541	136.443192
7.34E-13	1482.99997	1514.27288	1600.94504	2108.69154	2174.37767	2275.33435
0.0001916	3491.38173	3654.66765	3679.21913	3162.36874	2927.01597	3058.4414
5.05E-08	461.54057	439.659267	451.47758	657.87967	684.36268	629.368243
0.00546591	705.393071	743.887777	858.638341	1009.55112	1008.06944	861.898189
5.97E-08	2714.8214	2832.26929	2711.63528	2071.25124	2182.41009	2202.30842
0.03433427	440.609025	401.385357	368.383546	447.946523	513.27201	493.885919
0.0002109	2197.81224	2034.40549	2086.5835	2503.15192	2499.69091	2504.02083
4.41E-19	1384.62171	1572.17443	1672.95987	2656.9246	2690.86265	2479.03827
4.07E-17	6693.90814	6960.9446	7048.22054	4813.75368	5107.01633	4925.40704
1.48E-18	2008.38176	2048.14484	2276.77651	3440.49673	3305.34322	3182.39331
1.79E-09	936.686645	868.523328	830.017063	557.593135	553.434139	626.48564
1.38E-25	2434.3387	2456.39988	2315.55372	1433.42887	1490.81823	1502.79684
1.80E-05	386.187008	411.19918	372.076615	262.082145	259.447354	282.495059
0.00087096	3047.63297	3004.0112	2727.33082	2221.01246	2308.51918	2628.93361
0.00038102	938.779799	915.629678	881.720017	1072.39735	1163.8985	1126.13676
0.00013398	935.640068	932.313177	930.65317	1085.76889	1193.61848	1223.18439
0.00054848	1377.29567	1457.3527	1225.17535	1703.53394	1590.42031	1768.95715
2.07E-06	338.044454	308.15404	283.442979	197.898763	179.123096	198.899582
6.20E-21	409.211707	378.813565	449.631045	762.177666	840.994983	850.367779
2.42E-05	951.338727	869.504711	962.967516	1223.49573	1218.519	1140.54978
5.21E-05	121.402962	112.858964	126.487584	66.85769	68.2756194	56.6911852
3.90E-79	499.217352	520.132615	526.262209	80.2292281	70.6853472	77.8302713
4.52E-06	95.2385304	102.063758	97.8663056	165.807071	198.400918	169.112688
1.58E-09	711.672535	647.712313	762.61857	1075.07166	1020.11808	1036.77608
1.02E-07	706.439648	635.935725	687.83394	985.482351	983.972162	895.528553
8.88E-27	3257.995	3526.10657	3394.85288	1914.80424	1950.27299	2136.96942
0.00087858	1740.45798	1848.92424	2027.49441	1612.60748	1532.58685	1448.02739
3.43E-22	863.426237	954.88497	928.806636	486.723984	504.436341	486.198979
1.17E-28	1384.62171	1302.2943	1332.27433	2285.19585	2199.27819	2335.86901
0.00072817	1488.23286	1366.08415	1179.012	1589.87587	1714.11967	1838.13962
0.0060081	1176.35284	1123.68272	1064.52689	965.425044	918.909513	966.632752
0.00040321	190.477061	177.630195	174.497469	109.646612	118.879902	121.069311
2.50E-05	1871.28014	1785.13439	1689.57867	2204.96662	2147.06742	2264.76481
0.00041077	1577.19193	1482.86864	1524.31387	1754.34579	1849.86767	1881.37866
1.31E-05	1284.15029	1158.0311	1155.00706	1504.29803	1504.47336	1581.58798

7.47E-05	3160.66332	3169.8648	2861.20454	3575.54926	3736.68449	3972.22644
0.01123794	2159.08888	2108.00916	1696.96481	1309.07357	1568.73276	1828.53094
2.87E-11	2972.27941	2988.30908	2820.58079	2073.92555	2149.47715	2236.89965
0.00683338	2150.71626	2072.6794	1989.64046	1838.58648	1811.31202	1778.56583
0.02552707	2049.19827	2010.85232	1847.45733	2096.65716	2226.58844	2450.21224
0.01532235	1561.49327	1455.38994	1194.70754	1563.13279	1653.87648	1980.34801
0.00088685	850.86731	799.826568	605.663174	426.552062	535.762802	599.581349
1.64E-30	3459.98441	3453.48428	3488.10285	5234.95713	5306.22049	5398.15388
1.66E-10	1577.19193	1695.8286	1490.15299	1072.39735	1074.73857	1151.11932
4.86E-28	1604.40293	1439.68782	1465.22478	2512.51199	2754.31881	2697.1552
0.05306633	2587.13898	2582.01681	2762.41497	2914.99529	2910.95112	2876.83743
2.00E-10	1274.7311	1392.58147	1307.34612	1750.33433	1925.37247	1937.10897
0.01873561	589.222996	567.238965	577.965163	643.170978	693.198348	704.315912
1.06E-05	2542.13616	2772.40497	2538.06108	3132.95136	3222.60924	3409.15805
3.75E-18	911.568791	884.225445	974.969988	1497.61226	1439.41071	1508.56205
1.33E-38	2213.5109	2226.75642	2003.48946	3895.12902	4021.83561	4251.83889
6.83E-08	3240.20319	2982.42078	3088.32823	4046.2274	3817.00875	4002.9742
6.13E-06	1408.69299	1492.68247	1425.5243	1755.68294	1822.55742	1827.57007
4.03E-05	2453.17709	2593.7934	2745.79616	3174.40312	3300.52377	3084.38482
1.29E-10	845.634424	821.416978	867.871012	1244.89019	1232.17412	1159.76713
0.00082431	582.943532	514.244321	487.484994	357.020065	368.688345	440.077336
0.00170838	1197.28438	1111.90614	949.118511	822.349588	850.633894	899.372023
0.01797634	118.26323	57.9015552	60.0123572	34.7659988	45.7848272	48.0433773
4.98E-05	70.1206762	93.2313177	96.9430385	41.4517678	37.7524013	39.3955694
0.07549195	26.1644314	42.1994385	17.5420736	6.685769	11.2453961	22.0999536
4.46E-13	1191.00492	1267.94592	1260.2595	763.51482	861.87929	748.515819
2.28E-05	26.1644314	41.2180563	56.3192891	92.2636123	109.240991	97.0476222
8.62E-09	174.778402	155.058402	95.0965045	48.1375368	39.3588865	50.92598
0.00284995	154.893434	152.114255	122.794515	80.2292281	66.6691343	111.460635
0.09008803	4.18630903	12.7579698	4.61633517	1.3371538	0.80324258	2.88260264
0.00058654	4999.49956	4911.81837	5314.32505	6035.91226	5939.97889	5720.0445
0.00016611	1327.05996	1230.65339	1339.66047	1105.82619	1028.1505	975.28056
6.25E-06	23219.363	22327.4285	20055.2065	26170.7742	27586.5632	29967.537
7.47E-07	7439.07114	7258.30343	6372.38907	5007.64098	5174.48871	5710.43583
0.00031199	892.7304	1005.91685	985.125925	1139.25504	1221.73197	1233.75393
7.06E-15	529.568092	539.760261	662.90573	1101.81473	1065.90291	971.437089
0.00117023	474.099497	515.225703	511.489937	369.054449	359.852677	418.93825
0.00427199	1290.42976	1278.74113	1147.62092	961.413583	997.627286	1108.84115
7.02E-09	1739.4114	1728.21422	1599.0985	2151.48047	2321.37106	2416.58188
8.68E-104	1388.80802	1353.32618	1401.51936	3493.98288	3413.78097	3553.28819
0.01024162	732.60408	889.132356	884.489818	653.868209	713.279412	686.059428
0.0012315	568.291451	548.592701	625.051782	488.061137	443.389905	416.055648
0.01221631	3136.59204	3257.20783	3519.49393	3927.22071	3781.66607	3599.40983

1.68E-23	2028.26672	2156.0969	2273.08344	1295.70203	1311.69514	1252.01041
0.00863523	52.3288628	60.8457021	48.0098858	24.0687684	27.3102478	33.6303641
4.89E-11	506.543392	479.895941	529.03201	819.67528	801.636096	743.711481
4.06E-06	124.542694	141.31905	137.566788	53.486152	58.6367085	79.7520064
0.00021029	1516.49045	1693.86584	1549.24208	1985.67339	1945.45353	1877.53519
7.57E-12	1926.74873	1812.61309	2315.55372	3528.74888	3426.63285	2958.51118
4.78E-17	1341.71204	1246.35551	1306.42285	1978.98763	1996.86106	1902.51774
5.15E-05	274.203241	293.433305	371.153348	461.318061	496.403915	441.999071
1.25E-22	1935.12135	1925.47206	2117.05131	3407.06788	3382.45451	3079.58049
0.01735236	20.9315451	17.6648813	24.0049429	44.1260754	36.1459162	41.3173045
0.00072455	3818.96041	3569.2874	3901.72648	4385.86447	4463.61903	4296.99967
0.02290203	600.735345	664.395812	544.72755	447.946523	527.730376	504.455462
2.05E-08	3158.57016	2951.01655	2856.5882	2180.89785	2185.62306	2376.22544
2.22E-60	1017.27309	898.946179	1012.82394	300.859605	232.137106	241.177754
0.08814142	19.8849679	8.83244063	11.0792044	45.4632292	19.277822	18.2564834
1.57E-10	16.7452361	17.6648813	14.7722725	86.9149971	69.8821046	68.2215958
3.38E-09	640.505281	585.885228	609.356242	390.44891	408.047231	402.603502
5.25E-06	1067.5088	1072.65084	932.499704	623.113671	661.871887	818.65915
0.00463357	7454.7698	6627.27462	6605.97563	5681.5665	5898.21028	6240.83471
1.71E-54	373.628081	382.739094	442.244909	1240.87873	1171.12768	1107.88028
9.00E-17	936.686645	901.890326	767.234905	365.042988	436.160722	475.629436
7.74E-101	2303.51654	2224.79366	2100.4325	708.691514	733.360477	733.141938
0.00115052	2114.08606	2174.74316	2598.07343	1901.4327	1908.50437	1671.90953
1.49E-11	3574.06133	3463.29811	3468.71425	2521.87207	2645.88106	2698.11607
3.65E-31	2557.83482	2576.12852	2444.81111	1390.63995	1420.13288	1519.13159
1.22E-16	3391.95689	3073.68934	3152.03365	4635.91223	4593.74432	4555.47304
0.02719479	2411.314	2609.49551	2800.26891	3063.41936	3044.28938	2798.0463
1.50E-169	5987.46849	6024.70589	6275.44603	16410.8886	16085.7359	15924.4578
0.00297597	1679.7565	1694.84722	1897.31375	2108.69154	2200.88467	1972.66107
5.24E-06	1773.94845	1745.8791	1756.97717	1410.69726	1448.24637	1322.15374
2.37E-22	5696.52001	5525.1823	5667.01305	7902.57896	8135.24087	7917.54858
0.00607071	1027.73887	903.853091	696.143343	570.964673	611.267605	759.085362
3.64E-11	1062.27592	1127.60825	1120.84618	711.365822	754.244784	778.302713
1.47E-08	25339.7285	24593.4402	19537.2537	13522.6364	14630.2604	17094.7945
4.21E-05	8725.31459	8294.64313	7444.30209	6332.7604	6300.63481	6933.62022
0.00031664	4293.05991	4693.9515	5198.91667	3857.68872	3984.0832	3344.77993
0.03363138	10.4657726	16.683499	10.1559374	8.02292281	0.80324258	0.96086755
1.17E-08	923.081141	910.722767	976.816522	1287.67911	1289.20434	1254.89302
7.88E-05	5859.78606	5932.45595	6473.94844	5059.78998	5238.74812	4755.33349
0.0069075	104.657726	113.840346	104.329175	78.8920743	69.8821046	59.5737879
5.24E-68	6285.74301	6742.09634	7791.4505	2516.52345	2468.36445	2237.86052
0.00029989	15.6986589	17.6648813	23.0816758	52.1489982	61.0464362	38.4347019
1.84E-41	16.7452361	31.4042333	34.1608802	276.790837	241.776017	243.099489

3.70E-12	769.234284	685.986222	672.138401	387.774602	435.357479	438.155601
6.84E-08	1387.76144	1403.37668	1542.77921	2028.46232	1968.74757	1842.94395
0.00107411	1293.56949	1276.77836	915.880898	696.65713	760.670725	979.12403
0.0013018	4238.63789	3952.02649	3100.3307	2319.96184	2759.13827	3251.57578
1.50E-06	257.458005	302.265746	345.301871	517.478521	502.026614	433.351263
0.00647345	1274.7311	1136.44069	1288.88078	1460.17195	1437.80422	1444.18392
0.01022624	2290.95762	2290.54627	2081.96716	2481.75745	2473.98715	2737.51164
5.80E-34	4226.07896	4429.95967	4422.44909	7177.8416	7098.25469	6788.52922
0.03682668	2190.4862	2382.7962	2498.36059	2769.24552	2659.53619	2538.61206
9.23E-11	1284.15029	1370.00968	1359.97234	1835.91217	1807.29581	1870.80911
3.77E-09	1271.59137	1308.1826	1348.89314	1862.65524	1726.97155	1734.36592
4.02E-16	5023.57083	5377.97496	5378.03047	7204.58468	7250.87078	7233.41089
7.35E-14	1067.5088	996.103026	1121.76945	1809.16909	1669.94133	1551.80109
6.66E-05	946.10584	944.089765	868.794279	704.680053	707.656714	736.985408
0.00046558	9287.32658	8459.51536	8566.07154	10108.8827	10056.5971	10447.5128
0.0007373	124.542694	102.063758	108.94551	65.5205362	56.2269807	71.1041984
3.47E-06	1545.79461	1489.73832	1461.53171	1052.34004	1122.12989	1233.75393
3.99E-15	480.378961	542.704407	506.873602	882.521509	836.97877	840.759103
0.00053254	1018.31967	1044.19076	1086.6853	903.915969	816.897706	834.03303
4.52E-12	550.499637	562.332053	525.338942	327.602681	303.625696	335.342774
8.79E-59	234.433306	241.420044	248.358832	755.491897	796.013398	766.772302
1.14E-05	92.0987986	112.858964	95.0965045	42.7889216	52.2107678	39.3955694
4.62E-86	15233.9786	16683.499	16317.8216	34176.314	34190.0205	33404.5603
4.11E-80	1825.23074	1949.02523	1601.8683	385.100295	353.426736	462.17729
2.76E-05	959.711345	981.382292	1022.05661	782.234973	788.784215	720.65066
0.00874876	2105.71344	2039.3124	1851.1504	2215.66385	2286.83163	2503.05996
0.00355147	5922.5807	5916.75384	5600.53783	5041.06983	5028.29856	5309.75406
8.09E-05	303.507405	364.09283	368.383546	252.722068	227.317651	222.921271
3.33E-38	1886.97879	1960.80182	1701.58114	883.858662	894.812236	893.606818
2.64E-32	8001.08313	8533.11903	8212.46027	12589.303	13079.199	12706.5124
1.24E-21	722.138307	746.831924	779.237377	1235.53011	1286.79462	1297.17119
1.05E-11	372.581503	368.999742	389.618688	195.224455	191.171734	220.999536
2.30E-13	3004.7233	2965.73729	3022.77627	3948.61517	3975.24754	4046.21324
1.22E-06	1914.1898	1771.39504	1419.06143	978.796582	1109.27801	1255.85388
0.01505945	0	0.98138229	0	4.0114614	5.62269807	8.64780792
0.00017653	329.671836	347.409331	364.690478	263.419299	239.366289	238.295152
1.01E-23	1611.72898	1634.00152	1505.84853	2531.23215	2707.73074	2921.99821
4.09E-21	457.354261	511.300174	551.190419	944.030583	967.104068	957.984944
1.02E-13	779.700056	790.012745	854.945273	1408.02295	1461.9015	1189.55402
2.99E-18	1922.56242	1877.38432	1969.32858	1235.53011	1302.05622	1207.81051
0.0321255	2510.73884	2587.9051	2875.05354	2386.81953	2408.9245	2268.60828
3.54E-07	209.315451	252.215249	258.514769	413.180524	413.66993	356.48186
0.00407001	198.849679	228.662074	264.054372	165.807071	159.042031	166.230086

2.43E-36	6090.03306	6255.33073	6553.34941	3881.75748	3779.25635	3757.95297
5.23E-07	4431.20811	4859.80511	5185.06766	3805.53972	3759.17528	3480.26225
1.17E-14	3006.81646	3103.13081	3214.81581	2202.29231	2259.52138	2117.75207
0.00081942	2286.77131	2357.28027	2347.86807	2729.13091	2655.51997	2718.29429
0.00411066	3903.73317	3689.01604	3618.28351	4099.71355	4306.18348	4388.28208
1.48E-07	691.787567	767.440952	642.593856	390.44891	479.535821	478.512038
0.00719998	1071.69511	1061.85564	1089.4551	1243.55303	1262.69734	1224.14525
0.00091768	1100.99927	1118.77581	1185.47487	1408.02295	1402.46155	1302.93639
2.06E-13	2193.62593	2133.5251	2197.37554	1543.07549	1574.35546	1569.0967
8.12E-13	28189.5584	26439.4203	26314.957	34727.2214	36925.0615	38731.6099
2.68E-11	5993.74795	6170.93185	5638.39178	3841.64287	4102.15986	4534.33395
0.01547309	2311.88916	2431.86532	2683.014	2992.55021	3022.60183	2635.65968
0.00715969	2833.08463	2705.67098	3078.17229	3693.2188	3530.25115	3043.06752
6.39E-05	3755.1192	3482.92575	4163.01106	5226.93421	4964.03915	4444.97327
0.00830345	5321.84535	5397.60261	5847.05012	4998.28091	4927.89324	4697.68143
0.00287814	1036.11148	1045.17214	1094.9947	915.950354	889.189538	863.819924
0.01240221	1713.24697	1621.24355	1724.66282	2127.4117	1971.96054	1796.82231
0.00654416	32.443895	37.2925271	28.621278	16.0458456	15.2616091	9.60867546
9.13E-14	102.564571	98.1382292	94.1732374	17.3829994	21.6875497	24.0216887
0.02399158	7591.87142	7676.37229	8913.21994	7369.0546	7178.57895	6554.07753
7.56E-06	793.305561	873.43024	908.494761	656.542516	632.151912	626.48564
1.31E-05	2675.05147	2580.05405	2421.72943	2075.2627	2044.25237	2096.61299
4.20E-05	1962.33236	1866.58912	1617.56384	1317.09649	1296.43353	1494.14903
0.06179603	677.135485	644.768166	533.648346	512.129906	494.79743	558.264044
0.00206855	3087.40291	2830.30653	2763.33823	2517.86061	2429.00557	2487.68608
0.010637	1665.10442	1500.53352	1496.61586	1711.55687	1777.57583	1987.07409
1.80E-05	3399.28293	3137.47919	2622.07838	1973.63901	2163.13227	2446.36877
7.89E-14	2191.53278	2147.26445	1942.55384	1268.95896	1292.41731	1460.51867
6.66E-29	740.976698	750.757453	728.45769	1377.26841	1345.43132	1311.5842
0.04394461	473.05292	486.765617	494.87113	513.46706	569.49899	638.016051
1.36E-05	1732.08536	1544.69573	1492.92279	1211.46134	1252.25518	1227.02786
2.68E-05	2149.66969	2104.08363	2084.73696	1712.89402	1738.21695	1770.87889
3.03E-26	3039.26035	3133.55366	2893.51888	1642.02487	1771.14989	1867.92651
1.99E-06	2579.81294	2279.75106	2117.05131	1651.38494	1680.38348	1827.57007
1.13E-05	2607.02395	2624.21625	1944.40037	1382.61703	1609.69813	1825.64834
4.92E-06	1333.33943	1425.94847	992.512061	703.342899	771.112878	911.863302
3.59E-21	2618.5363	2722.35448	2148.44239	1283.66765	1278.76219	1392.29707
5.38E-05	2777.61604	2433.82808	2164.13793	1711.55687	1743.83964	2013.97838
0.00033624	7385.6957	7173.90455	5682.70859	4535.62569	4996.16886	5721.00537
1.51E-24	1026.69229	1028.48864	1051.60115	1671.44225	1714.11967	1776.64409
0.05580634	19.8849679	10.7952052	25.8514769	41.4517678	30.5232181	34.5912317
0.00016251	1284.15029	1193.36087	1189.16794	949.379199	869.911716	1039.65869
2.47E-09	3717.44242	3547.69699	3318.22172	4577.07746	4584.90866	4716.89879

0.00032546	2403.98796	2648.75081	2678.39766	3130.27705	3139.87525	2988.29807
3.40E-50	9129.29341	8889.3608	9554.89053	18213.3719	17635.1909	16387.596
0.00016804	63.8412127	75.5664365	95.0965045	123.01815	157.435546	145.091
2.65E-17	236.52646	274.787042	229.893491	90.9264585	88.356684	89.3606818
0.00705165	1799.0663	1922.52791	1901.00682	1557.78418	1625.76299	1690.16601
5.34E-13	4925.19257	5004.06831	4933.93903	3635.72118	3759.97852	3828.09631
7.99E-08	2256.42057	2349.42921	2446.65764	3096.8482	3095.69691	2899.89826
3.47E-13	1382.52856	1409.26497	1201.17041	803.629434	766.293423	879.193805
3.17E-36	6020.95896	6068.86809	5700.25067	3551.4805	3598.52677	3658.98362
1.03E-15	3846.17142	3646.8166	3513.95433	5102.5789	5159.2271	5470.21894
5.94E-22	799.585024	921.517972	835.556666	403.820448	419.292628	457.372952
0.00058377	265.830623	212.959957	230.816758	141.738303	168.680942	160.46488
4.18E-10	732.60408	674.209634	770.004706	1044.31712	1108.47476	1052.14996
4.08E-09	992.15524	1131.53378	1426.44757	2163.51485	2162.32903	1744.93546
0.03546636	207.222297	263.991837	226.200423	280.802298	292.3803	300.751542
0.02155133	2821.57228	2876.4315	3376.38754	4082.33055	3695.71912	3114.17172
4.07E-14	43.9562448	44.1622031	57.2425561	152.435533	174.30364	146.051867
2.26E-35	1343.8052	1423.00432	1490.15299	2694.36491	2616.96433	2526.12078
0.07881782	942.966108	964.698793	1068.21996	1227.50719	1099.63909	1056.9543
7.08E-07	30.3507405	32.3856156	29.5445451	0	3.21297033	4.80433773
0.00219148	6671.93001	6511.47151	7265.18829	7982.80819	7843.66381	7701.35338
7.66E-81	991.108662	943.108382	998.051664	2471.06022	2476.39688	2361.81243
0.00012875	2814.24624	2840.12035	2941.52877	3477.93704	3406.55179	3284.24527
5.61E-41	944.012686	978.438145	807.858655	2011.07932	2110.11826	2277.25609
7.44E-51	1882.79249	1852.84977	1533.54654	4476.79093	4549.56598	5311.6758
6.79E-23	977.503158	957.829117	827.247262	1651.38494	1696.44833	1905.40034
4.64E-11	1089.48692	1015.73067	1028.51948	746.131821	681.952952	669.72468
3.54E-14	2006.2886	2004.96402	2148.44239	3000.57313	3001.71753	2806.6941
2.25E-28	838.308383	874.411622	834.633399	1501.62372	1551.06143	1476.85342
4.31E-08	1742.55113	1675.21957	1784.67518	1231.51865	1329.36647	1341.37109
1.25E-35	1464.16158	1275.79698	1557.55149	3179.75174	3037.0602	2862.42442
0.00342518	1414.97245	1285.6108	1109.76697	1490.92649	1542.22576	1692.08775
0.00159817	589.222996	547.611319	507.796869	450.620831	418.489385	412.212177
5.15E-21	320.252641	231.606221	256.668235	657.87967	604.038421	632.250846
8.72E-05	7953.98715	7896.20192	5999.38919	3572.87496	3992.91887	4968.64608
0.00159249	1491.37259	1493.66385	1419.9847	1652.7221	1750.26559	1789.13537
0.03802574	383.047276	421.013003	433.935506	500.095522	493.190945	475.629436
4.31E-05	1037.15806	1265.98316	1388.59362	1917.47855	1767.13368	1529.70113
1.40E-08	772.374016	833.193566	781.083911	560.267443	539.779015	558.264044
0.00997069	665.623135	669.302723	720.148286	899.904508	856.256592	742.750613
3.39E-05	925.174295	895.02065	901.108625	707.354361	720.508596	696.628971
7.28E-09	1669.29072	1705.64242	1583.40296	1212.7985	1212.8963	1259.69735
8.34E-32	1879.65275	1681.10787	1690.50194	3096.8482	3136.66228	3387.0581

9.36E-34	1600.21663	1443.61335	1503.07873	786.246435	751.835056	781.185315
6.36E-13	275.249819	317.967863	254.821701	116.332381	118.879902	136.443192
1.43E-05	1330.19969	1340.56821	1201.17041	845.081202	985.578648	1031.97174
1.05E-36	1514.39729	1527.03085	1403.36589	2682.33052	2643.47134	2782.67241
3.06E-39	1582.42481	1482.86864	1593.5589	782.234973	730.147507	767.73317
3.60E-05	1233.91459	1201.21193	1040.52195	822.349588	873.124686	920.51111
2.11E-07	2929.36974	2992.23461	3096.63763	2417.57407	2428.20232	2347.39942
0.00474662	1.04657726	2.94414688	0.92326703	18.7201532	12.0486387	6.72607283
3.68E-25	941.919531	921.517972	850.328938	386.437448	440.980177	464.099025
0.00068844	36.630204	20.6090281	28.621278	10.6972304	8.03242582	3.84347019
8.98E-12	907.382482	948.996676	890.952688	1335.81665	1292.41731	1452.83173
0.01858904	0	0	0	1.3371538	3.21297033	8.64780792
0.00806256	43.9562448	26.4973219	27.698011	13.371538	12.8518813	14.4130132
9.65E-12	880.171473	905.815855	1011.90067	1502.96087	1455.47556	1330.80155
1.33E-17	2377.82353	2260.12342	2289.70224	1528.36679	1506.07984	1603.68794
8.45E-08	1760.34295	1772.37642	1949.01671	2575.35822	2461.13527	2300.31691
6.21E-25	1573.00562	1741.95357	1624.94998	931.996199	951.039217	853.250381
0.01923223	13.6055043	15.7021167	17.5420736	5.3486152	2.40972774	6.72607283
1.13E-07	1036.11148	1010.82376	944.502176	696.65713	701.230774	753.320156
1.56E-15	499.217352	489.709764	433.935506	215.281762	191.974977	252.708165
3.19E-47	1448.46292	1558.43508	1527.08367	2842.78898	3037.86344	3001.75022
0.00014397	3081.12344	2599.68169	2155.82852	3230.56358	3600.93649	4233.58241
1.56E-12	775.513747	794.919656	621.358714	303.533913	331.739186	428.546926
2.04E-10	1675.57019	1429.874	1489.22973	978.796582	1006.46295	1091.54553
5.03E-16	835.168651	851.839829	861.408143	1315.75934	1321.33405	1275.07123
5.20E-11	1835.69651	1949.02523	1671.11333	2492.45468	2623.39027	2728.86383
4.72E-05	1076.928	1008.861	959.274448	788.920743	780.751789	801.363534
0.00028163	452.121375	371.943889	393.311756	536.198674	568.695748	531.359753
1.65E-05	1249.61324	1253.22519	1096.84124	827.698203	880.353869	970.476222
1.06E-47	3428.58709	3510.40446	2923.9867	1303.72496	1192.01199	1401.90575
2.53E-06	4970.19539	5409.37919	5721.48581	4186.62855	4400.16286	4119.23917
5.45E-10	591.31615	651.637842	733.074025	1116.52342	1040.19914	967.593619
8.99E-07	1406.59983	1298.36877	1264.87584	1005.53966	1016.90511	981.045765
4.56E-05	3273.69366	3215.00839	2957.22431	3694.55595	3830.66387	4105.78703
6.18E-31	1249.61324	1351.36342	1475.38072	2656.9246	2776.00636	2491.52955
1.89E-45	366.30204	434.752355	493.947863	1569.81856	1468.32744	1252.97128
0.00705165	392.466471	411.19918	366.537012	331.614143	285.151116	302.673277
1.08E-07	146.520816	129.542463	154.185595	252.722068	251.414928	258.47337
3.89E-13	1675.57019	1580.02549	1596.3287	2390.831	2239.44032	2245.54746
2.19E-14	1916.28296	2045.2007	2220.45722	3344.22166	3225.01897	2946.0199
2.61E-30	3171.12909	3370.06679	3417.93456	5276.4089	5498.19547	5266.51502
6.30E-08	4661.4551	4411.3134	3987.59032	5567.90843	5748.80716	6257.16946
2.30E-10	6454.24194	6315.19505	6950.35423	8609.93332	8867.7981	8404.70843

3.88E-16	12034.5919	12100.4437	12990.3672	9128.749	8844.50407	8480.61697
2.28E-06	4221.89265	4266.06882	4199.94174	4939.44614	5133.52334	5215.58904
6.89E-22	1586.61112	1555.49093	1350.73967	2516.52345	2633.83243	2895.09392
6.55E-18	1851.39517	1838.12903	1999.7964	2773.25698	2865.96953	2833.59839
1.75E-27	5498.71691	5381.90049	5184.14439	7993.50542	8333.64178	8645.88618
2.25E-74	3144.96466	3045.22925	2993.23172	6041.26087	6154.44466	6118.80454
4.61E-06	1988.49679	1852.84977	1567.70742	1227.50719	1212.8963	1411.51443
0.02149014	83.7261806	91.2685531	81.247499	61.5090748	54.6204955	53.8085826
2.45E-33	1704.87435	1814.57586	1985.02412	3705.25318	3668.40887	3324.60171
9.84E-10	304.553982	301.284364	330.529598	529.512905	500.420128	487.159846
0.02633086	0	0	0.92326703	5.3486152	2.40972774	8.64780792
4.23E-17	1420.20534	1422.02294	1233.48476	623.113671	697.214561	815.776547
1.01E-41	3854.54404	3734.15962	4157.47145	1974.97616	1880.39088	1668.06606
6.13E-73	6278.41696	6063.96118	6726.00034	14823.687	14613.3923	13507.876
8.48E-44	9890.15508	9224.01216	9069.25207	5291.11759	5379.31557	5451.00159
0.01528972	10.4657726	4.90691146	1.84653407	26.743076	16.0648516	15.3738807
3.39E-29	12502.4119	12400.7466	11818.7413	18417.9565	18596.6722	19748.7107
0.00793311	48.1425538	36.3111448	35.0841473	66.85769	73.8983175	66.2998607
0.00036046	38.7233585	48.0877323	25.8514769	14.7086918	10.4421536	9.60867546
2.13E-10	1832.55678	1743.91633	1722.81629	1314.42219	1273.94273	1288.52338
7.94E-21	6268.99777	6187.61535	6600.43602	4482.13954	4249.9565	4196.10858
0.04358292	438.515871	402.36674	420.0865	403.820448	317.28082	318.047158
4.85E-142	603.875077	530.92782	620.435447	2509.83768	2401.69532	2398.3254
2.79E-32	2833.08463	3107.05634	3349.6128	5583.95427	5822.70547	5537.47967
1.86E-49	3794.88913	3807.76329	3804.78345	2106.01724	2139.03499	2055.29568
7.86E-16	574.570914	575.090023	561.346357	295.51099	240.169532	315.164555
0.00018591	1030.8786	1043.20938	1179.012	829.035357	900.434934	789.833123
1.97E-06	897.963286	856.746741	737.69036	1103.15189	1140.60447	1199.1627
1.21E-84	9563.62297	9113.11596	9455.17769	4595.79761	4629.087	4679.42495
1.36E-29	3852.45088	3723.36442	3196.35047	1535.05256	1701.26779	1871.76998
8.85E-07	29.3041632	34.3483802	38.7772154	82.9035357	97.995595	84.5563441
0.00194445	1576.14535	1581.00687	1817.91279	1422.73164	1392.01939	1252.97128
0.00092614	7005.78816	6980.57224	7247.64622	8000.19119	8213.95864	7899.2921
1.75E-25	635.272395	577.052788	670.291867	290.162375	271.495993	261.355973
2.83E-163	1859.76779	1827.33383	1951.78651	5850.04788	5941.58538	6253.32599
0.00069008	26.1644314	31.4042333	36.0074143	4.0114614	13.6551239	6.72607283
7.67E-05	1110.41847	1141.34761	989.74226	763.51482	707.656714	903.215494
3.92E-10	1371.01621	1380.80488	1562.16782	2208.97808	2270.76678	1929.42203
2.50E-05	1558.35354	1481.88726	1682.19254	1283.66765	1235.38709	1190.51489
2.71E-38	2661.44596	2945.12826	3226.81828	1429.41741	1356.67672	1296.21032
0.0032395	415.491171	377.832182	355.457808	276.790837	288.364087	309.39935
0.03276198	2869.71484	2640.89975	2740.25656	2436.29423	2521.37846	2472.3122
4.04E-13	142.334507	129.542463	120.024714	25.4059222	38.5556439	12.4912781

0.01820104	2871.80799	2628.14178	2556.52642	2393.5053	2391.25317	2335.86901
3.76E-08	1416.01903	1267.94592	1256.56643	938.681968	943.006791	970.476222
5.83E-43	1756.15664	1898.97473	1877.00188	3317.47858	3419.40367	3467.77098
0.01532328	251.178542	269.88013	244.665764	196.561609	192.77822	204.664787
0.00088989	1374.15594	1460.29685	1312.88572	1089.78035	1136.58825	1185.71055
3.35E-15	1888.02537	1691.90307	1803.14052	2726.4566	2561.54059	2651.03356
3.48E-14	6314.00059	6094.38403	6175.73319	8028.27142	8136.04411	8179.86542
5.34E-26	390.373317	421.994385	419.163233	799.617973	858.66632	819.620017
9.39E-06	982.736044	988.251968	795.856183	1199.42696	1265.91031	1467.24474
0.00752112	291.995055	274.787042	239.126162	197.898763	209.646314	195.056112
0.00061956	415.491171	310.116804	312.064257	203.247378	239.366289	252.708165
2.64E-08	345.370495	347.409331	348.071672	550.907366	523.714163	507.338065
0.00075427	6678.20948	6899.11751	7848.69305	6129.51302	6009.861	5684.4924
7.92E-20	1296.70922	1401.41391	1232.56149	2085.95993	2077.98856	2176.36499
5.56E-08	670.856022	670.284105	770.927973	501.432675	463.47097	429.507793
2.05E-20	1733.13194	1626.15046	1765.28657	1081.75742	1050.6413	990.65444
0.0654657	8.37261806	0	2.7698011	16.0458456	11.2453961	10.569543
7.07E-06	782.839788	739.962248	511.489937	330.276989	404.031019	456.412085
1.28E-07	1747.78402	1582.96964	1495.69259	2076.59985	2180.80361	2257.07787
8.44E-05	4785.9978	4793.07111	5196.14687	5943.64864	5992.18966	5676.80546
6.34E-12	1787.55395	1851.86838	1523.39061	998.853889	1016.90511	1170.33667
0.00010882	398.745935	436.71512	404.390961	299.522451	306.035424	290.181999
3.69E-34	7091.60749	6865.75051	7011.28985	4349.76131	4448.35742	4544.90349
3.87E-12	4046.06768	4540.85586	4730.82028	6463.80147	6228.34298	6174.53485
0.0001296	2065.94351	1963.74597	1770.82617	1445.46326	1586.4041	1571.97931
0.00048559	4837.28008	4881.39552	4560.01588	3776.12233	4050.75234	4223.01287
0.00126789	934.59349	825.342507	762.61857	556.255981	658.658917	699.511574
9.08E-05	578.757223	678.135164	657.366128	473.352446	476.322851	479.472906
0.02688491	1150.18841	1136.44069	838.326467	714.04013	804.849067	956.063209
0.03481518	925.174295	936.238706	950.965045	1097.80327	1045.82184	1046.38476
4.38E-11	667.71629	702.669721	554.883487	299.522451	316.477577	396.838297
0.01345054	555.732523	559.387906	496.717664	395.797525	431.341266	475.629436
0.00035168	1695.45516	1742.93495	1372.89808	1135.24358	1153.45635	1341.37109
0.05296616	576.664069	535.834731	591.814169	663.228285	623.316243	686.059428
0.02746982	2541.08958	2441.67914	2281.39284	2655.58745	2658.73295	2864.34616
1.13E-05	620.620313	612.38255	696.143343	463.992369	472.306638	465.059892
0.01962396	1898.49114	1788.07854	2086.5835	1684.81379	1719.74237	1612.33574
3.02E-10	405.025398	410.217798	454.247381	659.216824	654.642704	666.842077
4.94E-05	4603.89335	5172.86606	5542.372	6967.90846	6744.82796	5981.40048
7.92E-17	9697.58486	10049.3547	11302.635	6657.68877	6960.90021	6095.74371
0.00119337	1066.46222	995.121644	949.118511	796.943665	814.487978	841.719971
0.00891589	3241.24976	3179.67863	3198.197	2693.02775	2826.61064	2970.04159
3.51E-22	5542.67315	5970.72986	6132.33964	10181.089	9699.95742	8828.45102

3.34E-09	2773.42973	2784.18156	2995.07826	3879.08318	3850.74494	3581.15335
0.00677663	2075.3627	2045.2007	2256.46463	1870.67817	1895.65249	1633.47483
3.36E-07	952.385304	971.568469	1032.21254	1389.3028	1326.95674	1254.89302
6.07E-11	2201.99855	2234.60748	2302.62798	3019.29328	2929.4257	2959.47204
4.49E-48	485.611847	470.082118	475.482522	1140.59219	1096.42612	1133.8237
2.27E-08	3730.00134	3564.38048	3999.59279	2947.08698	2879.62466	2852.81575
1.06E-06	2770.29	2778.29327	2827.04366	3481.9485	3389.68369	3365.91902
0.00906879	2236.5356	2376.90791	2177.98693	2456.35153	2645.88106	2671.21178
1.99E-09	5462.0867	5687.11038	6086.17629	8206.11288	7882.21945	7221.88048
2.86E-07	2178.97385	2229.70057	2574.06849	3250.62089	3229.03518	2962.35465
3.60E-06	728.417771	717.390455	788.470047	1089.78035	983.972162	957.024076
1.50E-42	840.401537	783.143069	678.60127	1878.70109	1929.38868	2236.89965
6.92E-05	4358.99428	4176.76303	4905.31775	3756.06503	3574.42949	3182.39331
8.96E-06	40.816513	44.1622031	77.5544308	148.424072	122.092872	116.264973
2.79E-05	82.6796033	65.7526136	95.0965045	28.0802298	41.7686142	22.0999536
0.0307139	2797.50101	2849.93418	2622.07838	3044.6992	3026.61805	3099.7587
0.07012206	50.2357083	43.1808208	44.3168176	77.5549204	62.6529214	62.4563905
0.06734191	24.0712769	26.4973219	22.1584088	40.114614	44.178342	35.5520992
2.60E-22	2688.65697	2603.60722	2584.22443	3869.7231	3884.48112	3834.82238
2.18E-13	1915.23638	1803.78065	1593.5589	2611.46137	2768.77718	3058.4414
3.17E-08	102.564571	100.100994	139.413322	49.4746906	36.1459162	37.4738343
0.05617972	888.544091	945.071147	939.88584	834.383972	845.011196	762.928832
3.17E-21	7629.5482	8316.23354	7907.78214	5336.58082	5332.7275	5543.24488
5.44E-14	2714.8214	2617.34657	2757.79863	1789.11179	1813.72175	1986.11322
0.0763	1153.32814	1100.12955	1180.85854	1052.34004	1024.93753	1012.75439
1.07E-33	3576.15449	3538.86454	3097.5609	1472.20633	1568.73276	1773.76149
4.02E-07	985.875776	889.132356	826.323995	558.930289	601.628694	672.607283
0.06972514	123.496116	128.56108	160.648464	179.178609	177.516611	171.995291
1.69E-113	2866.57511	2987.3277	2610.0759	8925.50162	9660.59853	10509.9692
6.16E-05	1400.32037	1441.65059	1533.54654	2073.92555	1936.61786	1683.43994
4.65E-34	1376.24909	1292.48048	1502.15546	2687.67914	2669.1751	2566.47722
8.19E-07	1851.39517	1854.81253	1494.76933	1135.24358	1175.94714	1312.54507
0.08132593	798.538447	826.32389	782.930445	659.216824	714.082655	750.437554
1.53E-19	8040.85306	8520.36106	8991.69764	12752.4358	12773.9668	12155.9353
0.00553837	624.806622	673.228252	550.267152	435.912139	459.454757	540.968429
0.03153307	768.187707	809.640391	806.935388	723.400206	692.395105	645.702991
4.94E-15	5134.50802	4837.23332	4981.94891	6747.27808	6621.1286	6884.61597
3.48E-06	7.3260408	6.86967604	5.5396022	32.0916912	36.9491588	34.5912317
1.35E-45	2508.64568	2332.74571	2539.90761	1267.6218	1192.01199	1195.31923
7.63E-05	96.2851076	61.8270844	88.6336352	191.212994	169.484185	123.951913
1.27E-06	868.659123	922.499354	878.026949	1137.91788	1263.50058	1154.00192
2.50E-11	747.256161	737.018101	702.606213	418.52914	481.142306	466.02076
0.00333556	181.057865	176.648813	210.504884	124.355303	138.157724	130.677986

0.00127271	6.27946354	5.88829375	9.23267034	0	0	0
2.91E-22	1563.58642	1454.40856	1385.82382	839.732587	863.485775	838.837368
3.60E-21	1211.93646	1118.77581	1126.38578	1845.27225	1904.48816	2089.88691
6.40E-06	1908.95692	2108.00916	2265.6973	2713.08506	2660.33943	2688.5074
3.62E-13	938.779799	999.047173	877.103682	1394.65141	1423.34585	1416.31876
9.73E-09	850.86731	783.143069	779.237377	1155.30088	1136.58825	1086.7412
3.79E-16	3345.90749	3103.13081	3550.88501	4988.92083	5014.64344	4773.58997
1.27E-06	1866.04725	1901.91888	1877.00188	2428.2713	2458.72554	2248.43006
2.70E-22	694.927299	785.105833	786.623513	375.740218	386.359682	371.85574
3.42E-13	513.869433	538.778878	505.027067	304.871067	298.80624	288.260264
0.00881375	4090.02392	4703.76532	5180.45133	5971.72887	5943.19186	5008.04165
0.00117512	4393.53132	3813.65159	4284.8823	5069.15006	5107.81958	4703.44664
0.010637	1320.7805	1204.15607	1120.84618	915.950354	946.219761	1126.13676
2.57E-15	569.338028	545.648554	573.348828	311.556836	306.035424	321.890628
1.46E-56	479.332384	493.635293	495.794397	1210.12419	1284.38489	1364.43192
4.53E-13	2286.77131	2280.73245	2047.80628	1508.30949	1474.75338	1548.91848
2.08E-07	2372.59064	2698.8013	2580.53136	3356.25604	3196.90547	3389.9407
3.98E-101	1065.41565	1124.66411	1111.61351	3175.74028	3192.88926	2934.48949
0.00146366	2315.02889	2460.32541	2755.02883	3130.27705	3218.59302	2832.63753
3.75E-08	2607.02395	2467.19508	2490.97446	2036.48524	1905.2914	1850.63089
6.40E-11	768.187707	880.299916	824.477461	1297.03919	1238.60006	1170.33667
7.08E-11	1444.27661	1540.7702	1512.3114	2056.54255	2144.65769	1996.68276
0.05247985	3476.72965	3310.20247	3592.43203	3118.24266	3261.16488	3044.02839
0.00164871	367.348617	386.664623	452.400847	556.255981	508.452554	518.868475
8.85E-36	4596.56731	4690.02597	4719.74108	7481.37552	7401.88039	7197.85879
0.01818776	182.104443	223.755163	171.727668	139.063995	136.551239	148.93447
0.06827574	5.23288628	6.86967604	11.0792044	4.0114614	0.80324258	0.96086755
0.00258341	93.1453759	61.8270844	74.7846297	156.446995	113.257204	118.186708
0.00011587	771.327438	726.222896	813.398257	613.753595	587.97357	584.207468
2.84E-05	85.8193351	99.1196115	112.638578	30.7545374	51.4075252	48.0433773
1.33E-56	3935.13049	4206.2045	4146.39225	2068.57693	2101.28259	2127.36075
1.13E-14	533.754401	559.387906	454.247381	239.35053	261.053839	272.886383
3.43E-63	5125.08883	5488.87116	5262.62209	2533.90645	2684.43671	2642.38575
1.07E-10	1230.77485	1189.43534	1049.75462	763.51482	698.821046	786.950521
6.88E-12	4622.73174	4256.255	4684.65693	6231.13671	6133.56035	5942.00491
2.15E-06	40523.4714	42722.5153	43184.8922	50924.1654	49882.9708	49460.657
7.73E-22	948.198995	974.512616	965.737317	1557.78418	1529.37388	1607.53141
5.52E-35	7051.83756	6979.59086	6873.72307	10682.5217	10521.6746	10901.0423
0.06399014	59.6549036	74.5850542	71.0915616	42.7889216	53.0140104	42.278172
0.00054603	563.058564	546.629937	525.338942	685.9599	678.739981	696.628971
2.99E-07	4029.32244	4083.53172	4090.07296	4897.99437	4907.81217	5020.53293
0.0001991	1625.33448	1475.01758	1308.26939	1021.5855	1036.98617	1240.48
0.00976778	215.594915	204.127517	221.584088	136.389688	157.435546	174.877893

0.00500417	3441.14602	3260.15197	2532.52147	2154.15477	2306.10945	2742.31598
1.26E-08	206.17572	202.164752	208.65835	381.088833	360.655919	319.008025
0.00017797	78.4932943	79.4919656	75.7078968	38.7774602	40.1621291	33.6303641
5.79E-05	10371.5806	10497.8464	11352.4914	13300.6689	13136.2292	12247.2177
4.87E-08	3578.24764	3669.38839	3536.11274	4333.71547	4570.45029	4639.06851
0.0006606	37.6767812	37.2925271	47.0866187	70.8691514	82.7339859	83.5954765
0.02151101	1866.04725	1817.52	1829.91526	1611.27033	1586.4041	1700.73556
6.89E-66	1564.633	1507.4032	1504.92527	3259.98097	3172.8082	3393.78417
6.85E-05	6553.66678	7239.65717	7763.75249	9178.22369	9056.56011	8470.04742
5.50E-15	3410.79528	3192.4366	3062.47675	2135.43462	2138.23175	2295.51257
4.88E-07	327.578681	308.15404	321.296928	204.584532	159.845274	204.664787
0.00408112	8.37261806	2.94414688	6.46286924	0	0	0
2.49E-50	9667.23412	9758.86551	9568.73954	5674.88073	5853.22869	5764.24441
3.02E-16	500.263929	491.672528	512.413204	242.024838	233.743591	280.573324
4.88E-06	1197.28438	1474.0362	1567.70742	2017.76509	2060.31722	1849.67003
3.91E-07	1616.96186	1792.98545	1878.84841	2305.25315	2494.06822	2269.56914
1.50E-10	640.505281	649.675077	546.574084	335.625604	351.017008	391.073091
0.033209	140.241352	154.07702	142.183123	94.9379199	113.257204	113.38237
1.58E-29	2485.62099	2447.56744	2490.05119	1464.18341	1531.7836	1505.67945
1.90E-34	4348.5285	4744.98338	4974.56278	2484.43176	2624.99676	2358.92983
6.94E-06	1351.13124	1522.12393	1638.79898	2222.34962	1999.27079	1890.98733
7.22E-06	1208.79673	1178.64013	1238.10109	854.441279	963.087855	954.141474
4.80E-11	1086.34719	1082.46467	1130.07885	755.491897	730.147507	781.185315
3.19E-05	283.622437	290.489158	229.893491	164.469918	140.567452	183.525701
1.23E-06	1220.30908	1297.38739	1377.51441	1012.22543	973.530009	869.58513
7.05E-06	6905.31674	7232.78749	7525.54959	6018.52926	6076.53013	5879.54852
8.45E-42	2562.02112	2626.17901	2183.52653	887.870124	1008.87268	1064.64124
2.12E-21	2841.45725	2829.32515	2791.03624	4055.58748	4061.99774	4225.89547
1.09E-10	1832.55678	1803.78065	1785.59844	1306.39926	1363.9059	1301.01466
0.08351522	1092.62666	1083.44605	1178.08874	1053.6772	987.185133	966.632752
3.43E-10	1878.60618	1850.887	2009.02907	2685.00483	2615.35785	2492.49042
3.23E-07	600.735345	632.991578	687.83394	455.969446	431.341266	438.155601
8.75E-06	1395.08748	1410.24635	1432.91044	1075.07166	1170.32444	1078.09339
1.37E-06	256.411428	238.475897	249.282099	375.740218	386.359682	367.051403
0.00025821	5403.47838	5515.36848	5810.11944	4920.72599	4745.55717	4523.76441
0.00033773	2020.94068	2341.57815	2529.75167	2941.73836	2935.04839	2780.75068
3.00E-30	1261.12559	1175.69599	1297.19018	2226.36108	2164.73876	2165.79545
6.61E-48	4960.7762	4603.66433	4813.91431	9551.2896	9088.68981	8598.80367
0.07166204	1292.52291	1374.91659	1295.34365	1429.41741	1456.2788	1497.03164
0.0020754	1800.11288	1800.83651	1973.02165	1628.65333	1583.99437	1479.73602
0.00478735	55.4685946	73.6036719	65.5519594	36.1031526	37.7524013	31.708629
0.00140528	2435.38528	2408.31214	2011.79887	2588.72976	2851.51116	3241.9671
3.11E-12	3362.65273	3593.82195	3441.9395	2579.36968	2524.59143	2608.75539

2.82E-54	899.009864	850.858447	1033.13581	292.836682	257.840869	262.31684
0.00363571	783.886365	859.690888	866.947745	751.480436	591.989783	659.155137
1.07E-27	2296.1905	2223.81227	1937.01424	1017.57404	1037.78942	1166.4932
0.00085636	10303.5531	8890.34218	11176.1474	8744.98586	8119.17601	7019.13743
7.55E-06	188.383906	189.406782	192.96281	112.320919	114.060447	107.617165
0.00023325	15.6986589	20.6090281	23.0816758	2.6743076	3.21297033	2.88260264
7.22E-14	121.402962	127.579698	129.257385	30.7545374	39.3588865	33.6303641
6.79E-12	101.517994	110.896199	119.101447	29.4173836	32.9329458	32.6694966
2.61E-07	65.9343672	59.8643198	64.6286924	18.7201532	21.6875497	11.5304106
2.87E-07	118.26323	131.505227	164.341532	65.5205362	62.6529214	51.8868475
7.06E-13	276.296396	322.874774	345.301871	152.435533	141.370694	147.973602
0.01171759	36.630204	28.4600865	41.5470165	18.7201532	18.4745794	11.5304106
2.59E-06	60.7014809	72.6222896	108.94551	29.4173836	28.1134904	23.0608211
0.00736041	11.5123498	20.6090281	26.774744	10.6972304	2.40972774	0.96086755
1.52E-05	62.7946354	89.3057886	76.6311638	26.743076	34.539431	27.8651588
0.06208536	28.2575859	23.553175	14.7722725	14.7086918	9.63891098	6.72607283
5.96E-08	174.778402	229.643456	256.668235	101.623689	115.666932	82.634609
4.74E-10	297.227941	344.465184	396.081557	192.550147	152.616091	116.264973
2.51E-07	83.7261806	105.007905	98.7895726	36.1031526	37.7524013	36.5129668
0.04699969	12.5589271	21.5904104	22.1584088	10.6972304	7.22918323	5.76520528
0.02226391	2249.09453	2082.49322	2153.98199	1918.8157	1877.98116	1976.50454
0.00036429	1125.07055	1138.40346	1215.01942	1541.73833	1599.25598	1301.97553
0.00675769	4377.83267	4686.10044	5028.11227	3980.70687	4204.17167	4070.23493
3.01E-14	2777.61604	2838.15759	2806.73178	1950.9074	1961.51838	2088.92605
9.03E-60	1008.90048	1005.91685	882.643284	2372.11084	2451.49636	2685.62479
6.25E-05	4454.23281	4337.70973	4203.6348	4947.46906	5174.48871	5276.1237
1.62E-08	755.628779	791.97551	862.33141	576.313288	531.746589	475.629436
0.00511451	2304.56312	2363.16856	2279.54631	1920.15286	2070.75938	2058.17828
6.59E-48	10485.6575	10226.9849	9532.73212	4922.06314	4782.50633	5403.91908
3.69E-25	361.069154	336.614126	388.695421	822.349588	779.145304	726.415865
0.01742746	6496.10503	7050.25038	7146.08684	7528.1759	7932.02049	7628.32745
0.00955563	96.2851076	77.5292011	71.0915616	48.1375368	57.0302233	38.4347019
0.00049292	225.01411	214.922722	195.732611	139.063995	152.616091	126.834516
0.0403874	1083.20746	1058.91149	1084.83876	1178.0325	1294.82704	1176.10188
0.01635573	64.8877899	49.0691146	76.6311638	102.960843	86.7501988	109.5389
4.60E-40	4024.08955	4369.11396	4869.31034	2176.88639	2114.93772	2057.21742
0.00221731	204.082565	234.550368	246.512298	175.167148	159.845274	146.051867
0.01376066	749.349316	941.145618	864.177944	1034.95704	1081.96776	967.593619
6.85E-12	2474.10864	2740.01936	2584.22443	1874.68963	1867.539	1883.30039
8.28E-23	2007.33518	2013.79646	1929.6281	2975.16721	3167.1855	3033.45884
1.75E-07	117.216653	143.281815	146.799458	259.407837	283.544631	221.960403
3.49E-28	2869.71484	3045.22925	2827.96692	4583.76323	4704.5918	4965.76348
1.04E-05	451.074798	425.919915	375.769683	552.24452	600.825451	731.220203

0.00339709	2725.28718	2612.43966	2574.06849	2210.31523	2306.91269	2358.92983
2.32E-11	884.357782	791.97551	700.759679	445.272216	473.913123	485.238111
1.27E-26	45.002822	58.8829375	42.4702836	208.595993	219.285225	240.216887
0.028337	137.101621	145.244579	116.331646	104.297996	94.7826246	88.3998143
2.90E-05	12.5589271	29.4414688	26.774744	0	3.21297033	0.96086755
2.17E-09	2165.36834	2100.1581	2198.29881	1698.18533	1562.30682	1545.07501
5.74E-08	119.309807	122.672786	103.405908	42.7889216	40.1621291	52.8477151
0.0323073	0	4.90691146	0	10.6972304	7.22918323	11.5304106
1.20E-79	1251.7064	1288.55495	1350.73967	413.180524	417.686142	423.742588
4.90E-13	3038.21378	3079.57763	3427.16723	2194.26939	2249.07923	2014.93924
2.96E-06	76.4001398	75.5664365	103.405908	40.114614	24.0972774	30.7477615
0.05842071	436.422716	432.789591	381.309285	339.637065	291.577057	392.994827
1.83E-38	215.594915	211.978575	251.128633	26.743076	31.3264607	26.9042913
2.72E-20	810.050797	872.448857	984.202658	469.340984	394.392108	440.077336
8.13E-11	192.570215	207.071664	187.423208	68.1948438	93.979382	82.634609
2.85E-15	6916.82909	6855.93669	6353.92373	4454.05931	4773.67066	4856.22458
0.00012984	1988.49679	2065.80972	1979.48452	2438.96853	2336.63267	2424.26882
3.79E-14	11565.7253	11268.2315	11112.442	8481.56656	8269.38238	8798.66412
6.68E-07	1395.08748	1414.17188	1257.4897	981.47089	996.824044	1052.14996
1.13E-16	442.70218	426.901297	462.556784	740.783206	773.522606	819.620017
1.21E-06	1449.5095	1301.31292	1347.0466	1032.28273	1067.50939	1006.98919
4.66E-41	974.363426	922.499354	916.804165	401.14614	380.736984	341.107979
0.00671977	7093.70065	6686.15755	5808.27291	4996.94375	5257.2227	6010.2265
0.00211013	184.197597	161.928078	168.957867	114.995227	121.28963	111.460635
0.02364762	7183.70629	7633.19147	7889.3168	8548.42425	8538.46864	8099.15255
2.54E-05	1403.4601	1585.91378	1594.48217	2051.19393	2017.74537	1828.53094
1.40E-23	1381.48198	1291.4991	1204.86348	2176.88639	2224.17871	2439.6427
2.88E-09	565.151719	670.284105	639.824054	426.552062	339.771612	367.051403
6.88E-11	1878.60618	1829.29659	1826.22219	1305.06211	1396.03561	1299.09292
0.05226672	1533.23568	1656.57331	1799.44745	1954.91886	1908.50437	1783.37017
8.60E-56	13020.4677	12721.6586	13612.6491	23719.7713	23224.956	22792.7391
0.05946866	285.715591	315.023716	366.537012	246.036299	300.412726	220.038668
4.92E-10	7443.25745	6645.92088	6955.89383	5336.58082	5274.09079	5404.87995
0.03389878	100.471417	93.2313177	94.1732374	80.2292281	66.6691343	48.0433773
1.23E-23	441.655602	464.193824	520.722607	161.79561	199.20416	190.251774
8.94E-06	2027.22015	2018.70337	1756.0539	2394.84246	2492.46173	2865.30702
6.85E-29	2824.71202	2900.96605	3250.82323	1702.19679	1577.56843	1503.75771
1.20E-26	1053.9033	1073.63223	1159.62339	573.638981	527.730376	580.363998
0.08549474	2610.16368	2548.64981	2535.29127	2652.91314	2776.00636	3018.08496
0.00026802	2423.87293	2272.88139	2083.8137	2639.5416	2777.61285	2907.5852
2.84E-07	4859.2582	4709.65362	4117.77097	2973.83005	3268.39406	3609.0185
2.02E-24	302.460827	243.382808	285.289513	656.542516	676.330254	600.542217
8.89E-50	116.170076	95.1940823	125.564317	534.86152	492.387703	470.825098

1.45E-24	605.968232	637.89849	681.371071	1378.60557	1318.12108	1153.04106
0.07760988	5.23288628	7.85105833	2.7698011	10.6972304	15.2616091	14.4130132
0.03798024	5332.31112	5269.04152	5347.56266	4861.89122	4966.44888	4848.53764
5.64E-08	3856.63719	3558.49219	3226.81828	4607.832	4767.24472	5216.54991
0.00551021	929.360604	896.002032	637.054253	468.00383	557.450352	718.728925
2.48E-06	892.7304	857.728123	876.180415	1107.16335	1143.01419	1155.92366
9.44E-06	212.455183	197.257841	193.886077	108.309458	125.305843	120.108443
3.66E-08	1856.62805	1949.02523	1888.08108	2350.71638	2517.36225	2494.41215
3.90E-47	2871.80799	2718.42895	2276.77651	6152.24464	6949.65482	7348.715
0.00103495	1969.6584	2091.32566	1843.76427	2313.27608	2290.0446	2440.60357
7.55E-06	3008.90961	2873.48735	2490.97446	1962.94178	1987.22215	2277.25609
6.58E-08	1490.32601	1674.23819	1716.35342	1157.97519	1227.35466	1177.06274
1.36E-114	1841.97597	1896.03059	1921.3187	581.661903	537.369287	558.264044
2.77E-33	7644.20028	7473.22615	7151.62644	4080.9934	4252.36623	4588.14253
1.84E-18	16822.6828	16276.2253	15435.1783	10744.0308	11389.1766	11570.767
7.38E-06	35809.6874	33146.1869	29248.1764	24021.968	24500.5052	26954.2564
7.42E-22	3467.31045	3331.79288	2660.85559	1361.22257	1514.11227	1675.753
0.0007363	1455.78896	1419.07879	1394.13322	1724.9284	1641.02459	1696.89209
1.33E-05	489.798156	450.454472	525.338942	340.974219	353.426736	330.538436
2.01E-07	341.184186	385.683241	307.447922	211.270301	198.400918	206.586522
6.39E-07	3989.5525	3773.41491	4068.83782	4855.20545	4774.47391	5066.65457
5.67E-30	259.55116	259.084925	209.581617	45.4632292	50.6042826	50.92598
2.14E-11	460.493993	529.946438	456.093915	735.43459	836.175527	788.872256
1.68E-96	40671.0388	40315.1845	40360.6184	21200.5735	21440.9542	21458.094
0.00197948	3403.46924	3595.78472	3655.21419	3090.16243	3092.48394	3113.21085
7.18E-05	620.620313	655.563371	685.064139	887.870124	896.418721	791.754858
3.51E-15	3162.75647	3183.60415	3369.00141	4800.38215	4739.13123	4377.71254
6.35E-06	1715.34012	1562.36061	1648.95492	1279.65619	1290.00759	1318.31027
5.23E-06	3329.16225	3498.62787	3521.34047	2877.55498	2828.21713	2822.06798
3.17E-17	1267.40506	1341.54959	1376.59115	2063.22831	2073.97235	1950.56112
8.68E-40	5184.74373	5025.65872	5006.87712	8067.04888	8097.48847	8273.06958
0.01174135	1345.89835	1267.94592	1393.20995	1170.00958	1122.93313	1176.10188
1.51E-07	5759.31464	5655.70615	5698.40413	4589.11184	4662.82319	4775.51171
2.61E-21	1948.72685	2015.75923	1911.16276	2943.07552	2956.73594	3164.13683
2.76E-08	0	0	0	38.7774602	29.7199755	23.0608211
5.62E-05	5627.44591	5436.8579	6144.34211	7425.21506	7524.7765	6551.19493
3.14E-21	969.13054	907.77862	911.264562	1485.57787	1544.63548	1628.67049
5.73E-14	1792.78684	1750.78601	1711.73708	1216.80996	1237.79682	1177.06274
1.75E-102	23071.7956	22521.7422	21066.1839	51426.9352	52751.3501	55482.4139
0.04711113	2942.97525	2791.05124	2736.56349	3083.47666	3016.97914	3259.26272
9.58E-05	1710.10724	1589.83931	1376.59115	1087.10604	1155.06283	1276.99297
1.46E-22	3085.30975	3080.55901	3086.48169	1906.78132	2070.75938	2027.43052
1.26E-14	845.634424	900.908944	1022.97987	488.061137	545.401713	422.78172

0.03625231	601.781923	669.302723	708.145815	839.732587	809.668522	701.433309
2.46E-27	1778.13476	1828.31521	1913.00929	1089.78035	1011.28241	1062.71951
0.01074576	1313.45446	1239.48583	1246.4105	1071.06019	1036.18293	1154.00192
0.00029934	1270.54479	1264.02039	1215.01942	1017.57404	1032.16672	1033.89348
0.08852838	165.359207	158.983931	168.957867	128.366765	146.19015	114.343238
6.15E-06	2445.85105	2543.7429	2736.56349	2072.58839	2081.20153	2013.97838
9.30E-14	1798.01973	1827.33383	1873.30881	1318.43365	1215.30603	1145.35412
7.02E-14	9039.28777	8563.54188	10602.7986	16211.6527	15637.5266	13701.0103
1.09E-39	139.194775	174.686048	136.643521	517.478521	513.27201	505.416329
3.15E-05	803.771333	687.948987	656.442861	520.152829	487.568247	539.046694
0.0289305	444.795334	400.403975	405.314228	283.476606	366.278617	352.63839
0.00818527	680.275217	686.967604	675.831469	885.195816	808.062037	762.928832
2.77E-08	450.02822	502.467733	474.559255	312.893989	310.854879	268.082045
3.40E-09	2229.20956	2430.88394	2184.4498	1629.99048	1743.0364	1659.41825
0.03860515	159.079743	152.114255	134.796987	191.212994	211.252799	175.838761
1.22E-10	242.805924	218.848251	280.673178	431.900678	480.339064	436.233866
0.01056775	1437.99715	1394.54424	1356.27927	1174.02104	1265.91031	1181.86708
3.02E-15	2900.06558	2743.94489	2760.56843	2005.7307	1971.96054	1977.46541
0.00384039	1271.59137	1194.34225	1215.01942	1117.86058	998.430529	881.11554
8.21E-22	737.836966	775.292011	701.682946	1267.6218	1256.2714	1266.42343
5.38E-15	1697.54831	1607.50419	1578.78663	1025.59697	1048.23157	1126.13676
2.54E-09	182.104443	158.002549	149.569259	72.2063052	69.078862	71.1041984
4.85E-13	4837.28008	4795.03388	4085.45662	2636.8673	2904.52518	3152.60642
0.00120566	181.057865	160.946696	182.806873	123.01815	99.6020801	122.030178
0.00982632	2120.36552	2291.52765	2683.93727	3094.1739	2958.34243	2571.28155
6.60E-24	306.647136	285.582247	292.67565	105.63515	90.7664117	98.0084897
1.86E-12	2286.77131	2357.28027	2436.5017	1652.7221	1753.47856	1605.60967
8.75E-18	835.168651	860.67227	860.484875	1317.09649	1366.31563	1328.87982
0.00021561	21.9781224	32.3856156	26.774744	5.3486152	7.22918323	5.76520528
8.99E-11	382.000699	436.71512	472.712721	739.446052	778.342062	663.959475
3.89E-08	3773.95759	4021.70463	3844.48393	3051.38497	3140.67849	2915.27214
1.67E-22	1140.76921	1170.78907	1197.47734	652.531055	578.334659	674.529018
0.00746929	4241.77762	4270.97573	3483.48652	4467.43085	4682.90425	5423.13643
0.0030506	2491.90045	2202.22186	2454.96704	2075.2627	1975.97675	2040.88267
0.00085485	1081.11431	1014.74929	975.893255	780.89782	864.289018	834.03303
5.65E-07	16.7452361	20.6090281	20.3118747	78.8920743	61.0464362	55.7303177
2.57E-07	416.537748	391.571534	422.856301	607.067826	589.580055	583.246601
1.17E-10	879.124896	899.927562	1043.29175	1541.73833	1421.73937	1360.58845
1.86E-24	3178.45513	3271.92856	3614.59044	6045.27233	5727.92285	5347.2279
7.83E-06	128.729003	143.281815	156.955396	72.2063052	74.7015601	77.8302713
2.36E-24	995.294971	986.289203	957.427914	1719.57979	1700.46455	1583.50972
9.41E-27	1458.9287	1383.74903	1234.40802	2520.53491	2585.63787	2988.29807
0.01392778	2832.03806	2733.14968	2878.74661	3112.89405	3107.74555	3207.37587

0.0763	3990.59908	3929.4547	4055.91208	4313.65816	4384.09801	4227.8172
6.32E-19	1199.37754	1097.1854	1257.4897	669.914054	696.411318	693.746369
3.16E-26	2925.18343	3133.55366	3313.60538	1863.9924	1884.4071	1791.05711
0.03183623	4994.26667	4859.80511	5114.89937	5629.4175	5552.01272	5277.08457
0.00108537	369.441772	403.348122	413.623631	516.141367	520.501193	494.846786
0.00037334	1737.31825	1683.07063	1557.55149	1870.67817	2106.10205	2327.2212
9.68E-20	1715.34012	1785.13439	1692.34847	1109.83765	1066.70615	1121.33243
1.40E-09	1521.72333	1625.16908	1511.38813	968.099352	1081.96776	1157.84539
0.00018772	1506.02467	1588.85793	1393.20995	1160.6495	1198.43793	1244.32347
6.45E-31	626.899777	644.768166	667.522065	238.013377	281.134904	269.042913
7.24E-09	2316.07547	2246.38407	2271.2369	3013.94467	2959.94891	2834.55926
4.97E-66	3257.995	3347.495	2968.30351	1331.80519	1222.53521	1327.91895
7.06E-05	1405.55326	1334.67992	1638.79898	2036.48524	1869.94873	1817.00053
0.00137608	351.649958	333.669979	324.066729	229.990454	265.070052	248.864695
3.85E-16	3790.70282	3667.42562	4046.67941	2674.3076	2510.93631	2672.17265
5.77E-13	0	0	0	114.995227	95.5858672	76.8694037
4.14E-09	465.726879	436.71512	458.863716	295.51099	265.873295	283.455926
0.00553291	47.0959766	26.4973219	29.5445451	10.6972304	17.6713368	10.569543
4.24E-06	407.118553	420.031621	397.928092	561.604596	581.547629	560.18578
0.01536014	8.37261806	9.81382292	23.0816758	36.1031526	35.3426736	27.8651588
0.000317	2.09315451	0.98138229	0	20.057307	12.8518813	14.4130132
3.55E-07	581.896955	521.113997	585.351299	403.820448	376.720771	353.599257
1.95E-14	7434.88483	7394.71557	6870.03	4811.07938	4865.24032	5381.81913
1.03E-160	568.291451	580.978317	597.353771	2250.42985	2312.53539	2367.57763
0.06422073	1028.78544	1102.09231	1185.47487	1021.5855	1003.24998	869.58513
2.75E-49	2043.96538	2165.91072	2305.39778	978.796582	873.124686	823.463487
0.0546691	915.7551	908.760002	957.427914	831.709664	824.126889	812.893944
1.30E-20	428.050098	450.454472	540.111215	1004.2025	1049.03481	899.372023
1.71E-08	120.356385	110.896199	122.794515	53.486152	46.5880697	35.5520992
0.00021766	496.07762	490.691146	531.801811	629.79944	644.20055	720.65066
7.58E-06	937.733222	1043.20938	935.269505	1242.21588	1259.48437	1277.95384
2.29E-13	1406.59983	1411.22774	1400.59609	965.425044	987.988375	934.924123
7.56E-15	477.239229	468.119353	447.784511	247.373453	254.627898	234.451681
0.01127752	177.918134	181.555724	228.970224	175.167148	119.683145	101.85196
8.03E-28	997.388126	1032.41417	884.489818	439.9236	443.389905	477.551171
3.91E-14	3330.20883	3308.23971	3666.29339	5117.2876	4961.62943	4712.09445
0.00044293	265.830623	241.420044	337.915734	195.224455	177.516611	185.447436
0.00656	23.0246997	23.553175	22.1584088	6.685769	9.63891098	6.72607283
9.79E-153	2705.40221	2985.36493	2540.83088	9833.42905	10104.7917	10638.7255
1.85E-25	6843.56868	6861.82498	6486.87418	4007.44994	4337.50994	4428.63852
2.37E-16	4158.05144	4017.7791	3966.35518	2872.20636	2914.96733	2944.09816
6.68E-26	551.546214	562.332053	514.259738	225.978992	229.727378	243.099489
0.00034615	15723.7767	14235.9315	15011.3987	17518.0519	17059.2659	17654.9803

2.39E-31	2818.43255	2863.67353	2821.50406	4431.3277	4441.93148	4375.79081
0.02029315	1068.55538	1206.11884	1293.49711	1430.75457	1442.62368	1315.42767
0.07806891	1531.14253	1475.01758	1520.6208	1569.81856	1702.87427	1718.99204
0.00630274	30.3507405	20.6090281	25.8514769	6.685769	9.63891098	10.569543
0.00663804	2.09315451	0	0	12.0343842	11.2453961	5.76520528
4.67E-12	7653.61948	8292.68037	8463.5889	5879.46526	6179.34518	5586.48392
1.52E-07	454.21453	498.542204	471.789454	340.974219	291.577057	270.964648
6.29E-23	11219.3082	11897.2975	12142.808	7648.51974	8157.73166	7792.6358
1.53E-43	1507.07125	1507.4032	1533.54654	707.354361	751.031814	739.868011
8.15E-37	2364.21802	2492.71102	2596.2269	1309.07357	1355.87348	1236.63653
4.09E-86	6218.76206	6550.7268	6256.05742	12847.3737	13031.0044	13486.7369
0.04944043	2717.96114	2594.77478	2544.52395	2314.61323	2356.71373	2452.13398
0.00017685	777.606902	692.855898	548.420618	433.237831	459.454757	503.494594
5.61E-08	3159.61674	3010.88087	3161.26632	2511.17484	2457.11906	2401.208
0.00413919	1512.30414	1526.04946	1452.29904	1703.53394	1827.37687	1692.08775
0.08116374	4023.04298	3746.91759	3746.61762	3460.55404	3372.8156	3690.69225
0.01064882	135.008466	164.872225	192.039543	234.001915	220.088467	224.843006
0.00134269	2012.56807	1855.79391	1707.12075	1452.14903	1455.47556	1629.63136
2.55E-06	1530.09595	1573.15581	1320.27186	1049.66573	1103.65531	1120.37156
1.66E-11	1463.11501	1608.48558	1660.95739	1037.63135	1101.24558	1089.6238
3.70E-57	837.261806	836.137713	1005.4378	2580.70684	2491.65849	2298.39517
9.09E-13	2001.05572	1962.76458	1872.38554	2898.94944	2642.66809	2708.68561
3.26E-33	7112.53904	7668.52123	7076.84181	4010.12425	4252.36623	4434.40373
8.52E-23	1362.64359	1350.38203	1440.29657	2325.31046	2293.25757	2144.65636
1.48E-41	1246.47351	1362.15862	1275.03177	2430.94561	2456.31581	2538.61206
0.04842993	1929.88846	1808.68756	1643.41532	1472.20633	1553.47115	1685.36168
0.00390457	1062.27592	1209.06298	1321.19513	1632.66479	1433.78801	1394.21881
4.22E-20	362.115731	354.279007	426.54937	783.572127	794.406913	722.572395
1.01E-12	83.7261806	93.2313177	97.8663056	16.0458456	22.4907923	20.1782185
7.89E-72	22459.5479	22753.3484	24473.9625	11621.2037	11332.1463	10905.8467
0.00065469	490.844733	448.491707	474.559255	353.008603	363.868889	362.247065
1.37E-07	2110.94633	2139.4134	2432.80863	3062.0822	3118.99094	2823.02885
0.00751026	1580.33166	1528.99361	1608.33117	1373.25695	1306.87568	1408.63182
1.00E-28	3485.10227	3637.98416	3395.77615	2085.95993	2182.41009	2188.85627
7.51E-05	1358.45728	1466.18514	1577.86336	1234.19296	1108.47476	1056.9543
2.39E-17	2654.11992	2857.78523	2765.18477	3879.08318	3947.93729	3984.71772
5.66E-14	3307.18413	3372.02955	3705.99387	2459.02584	2311.73215	2144.65636
3.01E-08	1035.06491	1055.96735	1066.37342	736.771744	787.17773	771.57664
7.39E-09	3913.15236	4142.41465	3934.04083	5069.15006	4949.58079	5237.689
2.81E-47	3747.79316	3663.5001	3740.15475	2016.42793	2077.98856	2099.49559
0.00338219	575.617491	545.648554	453.324114	617.765056	681.952952	724.49413
8.30E-08	933.546913	1087.37158	1239.02436	1699.52248	1698.85806	1504.71858
2.24E-07	2331.77413	2493.6924	2875.97681	3765.4251	3785.68229	3283.28441

4.04E-19	1576.14535	1604.56005	1711.73708	2725.11945	2761.548	2431.95576
0.00057699	1448.46292	1572.17443	1252.87336	1702.19679	1790.42771	1964.97413
9.00E-06	1135.53632	1194.34225	1114.38331	1387.96565	1502.86687	1497.9925
6.03E-06	1146.0021	1180.6029	1269.49217	1553.77272	1506.88308	1532.58374
9.33E-33	7858.74862	7560.56918	7294.73283	4668.00392	4636.31618	4831.24202
2.05E-07	1991.63652	1894.06782	1450.45251	964.08789	1126.94934	1247.20608
0.07687982	1905.81718	1819.48277	1513.23467	1896.08409	1935.81462	2159.06938
0.04421033	934.59349	912.685531	771.85124	637.822363	759.867482	794.637461
5.01E-08	1241.24063	1369.0283	1449.52924	1937.53586	1928.58544	1757.42674
8.83E-83	1209.84331	1193.36087	1179.012	2812.03444	2807.33282	2744.23771
3.56E-57	459.447416	486.765617	486.561727	1278.31903	1192.01199	1310.62333
3.85E-19	1038.20464	918.573825	1102.38084	1771.72879	1757.49477	1688.24428
2.70E-19	560.96541	595.699051	608.432975	997.516735	996.824044	1018.5196
3.15E-30	272.110087	303.247128	310.217723	696.65713	705.246987	662.998607
8.08E-11	1974.89128	1867.5705	1756.97717	2500.47761	2620.1773	2793.24196
0.00238106	1464.16158	1541.75158	1392.28669	1617.9561	1801.67311	1844.86569
4.02E-16	1234.96116	1137.42208	1271.33871	639.159517	768.703151	702.394176
2.22E-31	2121.4121	2105.06502	2021.9548	1143.2665	1163.8985	1227.98872
0.000252	324.43895	317.967863	325.913263	234.001915	220.89171	237.334284
8.25E-10	1115.65136	1010.82376	1094.9947	695.319976	723.721566	775.42011
0.00046405	484.56527	410.217798	351.76474	266.093606	249.808443	323.812363
4.04E-13	2826.80517	2836.19482	2760.56843	2023.1137	1957.50217	2108.1434
0.00470297	1398.22722	1393.56285	1443.98964	1597.89879	1687.61266	1612.33574
6.65E-06	900.056441	806.696244	875.257148	1278.31903	1111.68773	1101.15421
0.04617969	511.776279	540.741643	601.046839	463.992369	474.716366	467.942495
2.10E-19	2450.03736	2578.09128	2254.6181	1445.46326	1498.04741	1555.64456
9.60E-08	1361.59701	1417.11603	1235.33129	902.578816	991.201346	987.771838
0.00010539	924.127718	930.350413	881.720017	637.822363	758.260997	698.550706
0.04583102	133.961889	150.151491	155.108862	105.63515	114.863689	111.460635
4.24E-09	1354.27097	1452.44579	1395.97976	1921.49001	1955.89569	1803.54838
1.59E-13	962.851076	985.307821	874.333881	1398.66288	1414.51019	1463.40127
1.25E-156	1106.23216	1120.73858	1124.53925	125.692457	122.092872	152.77794
0.022301	1614.86871	1622.22493	1784.67518	1486.91503	1515.71875	1387.49274
0.00016126	1485.09313	1474.0362	1242.71743	1032.28273	1017.70835	1164.57147
6.23E-07	1571.95904	1408.28359	1363.66541	1849.28371	1914.93031	2004.3697
0.0060313	3081.12344	2685.06195	3135.41485	2695.70206	2451.49636	2362.7733
1.69E-26	2683.42409	2645.80666	2721.79122	4397.89885	4190.51655	4059.66538
2.40E-12	1082.16088	1157.04972	1263.0293	1773.06594	1753.47856	1690.16601
4.18E-05	16.7452361	4.90691146	23.0816758	52.1489982	56.2269807	53.8085826
0.00820195	7950.84742	7455.56127	6964.20324	8053.67734	8629.23505	9139.7721
0.01488924	667.71629	668.321341	661.059196	545.558751	527.730376	600.542217
0.06788484	52.3288628	54.9574083	57.2425561	36.1031526	36.9491588	35.5520992
0.0008267	11548.98	11252.5294	9539.19499	8060.36311	8498.30651	9499.13656

0.00662678	9787.59051	10620.5192	10036.8359	11089.0165	11357.0469	11770.6274
4.60E-25	10591.3618	11003.2583	11114.2886	7577.65059	7577.79051	7547.61458
7.45E-68	3183.68802	3225.80359	3437.32317	6839.54169	6719.92744	6527.17324
0.01165721	2552.60193	2722.35448	2426.34576	2845.46329	2886.85384	3064.20661
4.56E-15	3607.5518	3385.76891	3306.21925	2463.0373	2326.99376	2407.93407
0	5412.89757	5412.32334	5015.18653	34688.4439	36746.7416	40575.5148
5.72E-38	1765.57583	1926.45344	2033.03401	3731.99626	3739.89746	3513.89262
2.91E-34	7188.93918	7059.08283	7197.7898	10754.728	11160.2524	10851.0772
3.07E-07	1427.53138	1519.17979	1435.68024	1837.24932	1902.07843	2125.43901
0.0009935	2140.25049	2057.95867	2166.90773	2548.61514	2571.98275	2368.5385
7.47E-06	523.288628	564.294818	577.965163	414.517678	395.19535	380.503548
0.00072455	588.176418	478.914558	533.648346	394.460371	409.653717	402.603502
3.77E-06	60.7014809	62.8084667	49.8564198	9.36007661	13.6551239	24.0216887
8.16E-06	1272.63794	1160.97525	1105.15064	1543.07549	1475.55662	1775.68323
5.12E-05	1638.93998	1493.66385	1655.41779	2112.70301	1992.0416	1899.63514
0.00130047	4354.80797	3805.80053	3937.7339	4456.73362	5064.44448	5002.27645
5.54E-05	9923.64555	9812.84154	9430.24948	10999.4272	11547.4154	11574.6105
0.00176394	3491.38173	3400.48964	3227.74155	3802.86541	3849.13845	4022.19155
0.00031615	12421.8255	12503.7918	11777.1943	9885.57805	10167.4446	11050.9377
2.30E-23	2472.01548	2386.72173	2661.77886	4106.39932	4086.09501	3853.07886
3.96E-09	60.7014809	66.7339958	48.9331528	156.446995	140.567452	137.404059
2.60E-10	2020.94068	1985.33638	1722.81629	1259.59888	1298.04001	1363.47105
1.59E-11	7694.43599	7726.42278	7186.71059	5329.89505	5607.43646	5846.87902
3.30E-07	1063.32249	1247.33689	1147.62092	871.824278	812.07825	810.011342
0.01666891	1675.57019	1804.76203	2058.88549	2468.38592	2202.49116	1985.15235
0.0009438	3534.2914	3586.95228	3659.83052	3110.21974	3164.77577	3126.663
2.13E-06	526.42836	479.895941	505.027067	695.319976	685.969165	697.589839
2.01E-09	2601.79106	2688.0061	2825.19712	3604.96665	3532.66087	3453.35796
0.02514629	4219.7995	3851.9255	3681.06566	3345.55881	3516.59602	3552.32732
6.02E-10	2747.2653	2692.91301	2925.83323	3851.00295	3862.79357	3544.64038
0.00088291	2228.16298	2451.49297	2561.14275	2928.36682	2964.76837	2774.98547
7.39E-06	1552.07407	1476.98035	1492.92279	1069.72304	1179.96335	1234.7148
0.00870648	6669.83686	7102.26365	7324.27738	8168.67257	8134.43762	7572.59713
1.39E-62	2016.75437	2171.79901	2242.61563	4659.981	4672.4621	4436.32546
0.00620372	1344.85178	1632.03875	1595.40543	1843.93509	1962.32163	1719.95291
0.00968257	1028.78544	1054.98596	989.74226	1198.08981	1148.63689	1225.10612
2.69E-22	534.800978	641.824019	565.962692	254.059222	259.447354	265.199443
0.00058457	7445.35061	7540.94153	7978.87371	6576.12239	6792.21927	6576.17749
5.36E-19	114.076921	105.989288	95.0965045	9.36007661	16.0648516	13.4521457
0.00079373	5546.85946	5763.6582	5677.16899	4832.47384	4992.15264	5036.86768
0.00077984	519.102319	538.778878	696.143343	879.847201	813.684735	718.728925
9.28E-36	2312.93574	2182.59422	2389.41508	4151.86255	4213.81058	3901.12224
7.74E-07	13038.2595	13675.5622	12781.7088	10649.0929	10727.3047	11164.32

9.02E-05	9390.93773	8413.39039	7351.97539	5399.42705	6254.04674	6993.194
4.71E-06	9045.56723	9033.624	8705.48486	10479.2743	10614.0475	11577.4931
0.0065922	14.6520816	21.5904104	12.9257385	1.3371538	5.62269807	3.84347019
6.81E-20	1793.83342	1841.07318	1840.99447	1159.31235	1199.24117	1095.389
1.20E-05	730.510925	871.467475	878.950216	1108.5005	1146.22716	1085.78033
3.33E-05	256.411428	323.856156	321.296928	163.132764	194.384705	204.664787
1.12E-07	743.069852	768.422335	765.388371	530.850059	563.07305	499.651124
0.0052446	972.270272	980.40091	1018.36354	1215.4728	1171.12768	1114.60635
0.02262151	768.187707	763.515423	827.247262	964.08789	924.532211	862.859057
0.00080081	450.02822	535.834731	505.950335	679.274131	700.427531	589.011806
0.00015942	636.318972	721.315985	821.70766	1029.60843	1111.68773	882.076408
1.23E-14	221.874378	271.842895	294.522184	102.960843	97.995595	106.656298
2.26E-31	4620.63859	4312.19379	3380.08061	1366.57118	1471.54041	1776.64409
6.99E-89	559.918832	600.605963	671.215134	1997.70778	2021.76158	1937.10897
0.09083152	32.443895	32.3856156	30.4678121	21.3944608	15.2616091	20.1782185
1.13E-30	458.400839	458.30553	445.937977	897.2302	932.564637	958.945811
5.42E-05	13880.7542	12844.3314	13141.783	10921.8722	11403.6349	11482.3672
0.0579096	383.047276	335.632744	364.690478	413.180524	455.438544	412.212177
0.0004928	8478.32236	9273.08128	10152.2443	12184.1454	11980.3631	10434.0607
2.00E-05	33.4904722	34.3483802	45.2400847	90.9264585	77.1112878	93.204152
0.00186037	189.430484	206.090281	247.435565	161.79561	130.125298	143.169264
3.74E-33	6916.82909	6295.5674	6557.04247	10662.4644	10473.48	10674.2776
8.11E-06	3723.72188	3635.04001	3398.54595	2701.05068	2830.62686	3032.49798
1.00E-38	1122.9774	1181.58428	1221.48229	2279.84723	2241.0468	2158.10851
4.14E-41	4144.44594	4267.0502	4272.87983	6856.92469	6951.2613	7193.05445
1.19E-33	1016.22652	956.847735	936.192772	417.191986	405.637504	465.059892
1.39E-06	858.193351	915.629678	906.648227	653.868209	655.445947	684.137693
0.08815188	27.2110087	28.4600865	26.774744	16.0458456	17.6713368	12.4912781
2.85E-44	1589.75085	1729.1956	1790.21478	762.177666	798.423126	740.828878
9.92E-22	616.434004	654.581989	679.524537	1186.05542	1116.50719	1132.86284
8.84E-09	980.64289	914.648296	1253.79663	1968.29039	1824.1639	1511.44465
2.30E-05	2133.97103	2168.85486	1983.17759	2517.86061	2543.86926	2718.29429
3.39E-14	4004.20458	3858.79517	3597.04836	5204.20259	5425.1004	5430.82337
5.82E-05	4346.43535	4014.83496	4032.8304	3320.15289	3341.48914	3579.23161
1.63E-05	1955.00632	1964.72735	1901.00682	1504.29803	1541.42251	1641.16177
0.00114678	975.410003	978.438145	851.252205	1176.69534	1085.18073	1243.36261
0.00444211	2021.98726	1902.90026	2147.51912	1735.62563	1757.49477	1719.95291
0.03462935	1146.0021	1048.11629	1114.38331	1006.87681	968.710553	927.237182
0.05756989	980.64289	948.996676	974.969988	1183.38111	1068.31263	1039.65869
0.04237941	215.594915	220.811016	219.737554	163.132764	177.516611	179.682231
4.35E-18	677.135485	634.954343	599.200305	1179.36965	1138.99798	1035.81522
0.00029186	720.045153	758.608512	749.692831	593.696288	578.334659	591.894409
0.00011927	7867.12124	7802.9706	8865.21006	10368.2906	10209.2132	9400.16721

0.00680349	746.209584	899.927562	964.81405	1081.75742	1119.72016	1025.24567
0.01414703	34.5370495	16.683499	30.4678121	5.3486152	14.4583665	9.60867546
0.00977506	161.172898	133.467992	115.408379	64.1833824	80.3242582	111.460635
3.96E-143	18568.3737	18281.1893	18152.3532	7516.14151	7790.6498	7840.67918
1.17E-06	642.598436	638.879872	677.678003	470.678138	482.748792	414.133913
0.00012137	466.773457	501.486351	587.197833	401.14614	363.065647	320.929761
0.01242237	469.913188	487.746999	541.957749	597.707749	604.038421	611.11176
0.00609614	51492.6476	54828.8473	50487.9345	44021.7774	46759.1604	48039.5339
2.28E-183	2374.6838	2376.90791	2526.98187	8041.64296	7638.83695	7743.63156
0.00552511	5.23288628	12.7579698	8.3094033	20.057307	30.5232181	26.9042913
1.13E-30	276.296396	244.364191	307.447922	740.783206	664.281615	711.041984
2.12E-11	8.37261806	4.90691146	8.3094033	66.85769	68.2756194	46.1216422
3.09E-30	21.9781224	18.6462635	17.5420736	177.841456	166.271214	149.895337
2.91E-24	11.5123498	10.7952052	9.23267034	151.098379	125.305843	102.812827
1.42E-50	277.342973	289.507776	293.598917	815.663819	834.569042	783.10705
1.25E-14	424.910366	391.571534	417.316699	661.891131	731.753992	724.49413
6.12E-16	24.0712769	14.7207344	7.38613627	104.297996	114.863689	100.891092
1.06E-61	30.3507405	36.3111448	43.3935506	447.946523	395.19535	335.342774
2.48E-20	29.3041632	37.2925271	42.4702836	192.550147	155.829061	157.582278
2.42E-55	166.405784	165.853607	168.957867	676.599823	595.202753	605.346554
2.15E-14	2144.4368	2018.70337	2219.53395	1413.37157	1474.75338	1472.04908
6.01E-32	1692.31542	1733.12113	1724.66282	934.670507	983.972162	954.141474
1.26E-06	6148.64138	5894.18204	6165.57725	5102.5789	4918.25433	4526.64701
0.07736619	6265.85804	5889.27513	5633.77544	5193.50536	5372.88963	5668.15766
2.11E-05	5430.68939	5138.51768	4977.33258	4212.03447	4392.93368	4309.49095
6.72E-05	12481.4804	12083.7602	10143.9349	13854.2505	14801.3511	17117.8553
2.13E-16	4461.55885	4820.54982	4726.20395	6839.54169	6478.15142	6461.83425
0.08549474	1039.25122	881.281298	926.960102	1000.19104	1098.83585	1123.25416
2.24E-13	3843.03169	3815.61435	3654.29092	4999.61806	4978.49752	5144.48484
8.11E-12	5148.11353	4588.9436	4673.57772	6398.28094	6570.52432	6763.54666
6.40E-08	6622.74088	6482.03004	6265.29009	5105.25321	5243.56757	5314.5584
8.02E-05	5416.0373	5327.92446	5651.31751	6521.29909	6686.19125	6203.36088
5.34E-26	3302.99782	3276.83547	3475.17712	5319.19782	5333.53074	5014.76773
1.97E-20	5883.85734	5688.09176	5700.25067	7988.15681	8436.45683	8738.12947
4.86E-35	21178.5374	20718.9429	20976.627	30797.3263	32079.099	31470.3339
3.87E-06	5948.74513	5526.16369	6222.81981	4926.0746	4650.77455	4645.79459
2.94E-10	3950.82914	3772.43353	3822.32552	5008.97814	4817.849	5094.51973
2.86E-15	7273.71194	7262.22896	7445.22536	10090.1626	10032.4998	9490.48876
1.30E-16	2679.23778	2913.72402	3068.93962	4567.71738	4530.28816	4164.39995
0.00157573	4025.13613	3917.67811	3963.58538	3504.68011	3527.03818	3436.06235
9.88E-34	1995.82283	1816.53862	1819.75932	3380.32481	3275.62325	3243.88884
0.00317511	951.338727	864.597799	705.376014	952.053506	1109.27801	1290.44511
6.23E-05	181.057865	158.002549	140.336589	231.327608	250.611685	276.729853

3.36E-17	1134.48975	1168.82631	1078.3759	1817.19202	1706.89049	1713.22684
1.16E-18	1542.65488	1685.0334	1624.94998	1018.9112	993.611073	1040.61955
0.00152651	11913.1889	12053.3373	13524.9388	15715.5686	15149.1551	13751.9363
0.03637542	5479.87852	5344.60796	5353.10226	5761.79573	5883.75191	5941.04404
0.00493023	3340.6746	3114.90739	3334.84053	2959.12136	2806.52958	2811.49844
4.11E-80	4226.07896	3925.52917	3802.93691	1564.46995	1546.24197	1661.33999
0.05398431	4750.41417	4404.44373	3970.97151	3518.05165	3694.91588	4249.91716
2.48E-43	1411.83272	1459.31547	1517.851	693.982823	679.543224	628.407375
6.16E-25	6696.00129	6082.60744	5009.64693	2390.831	2482.01958	3025.7719
8.64E-07	188.383906	166.83499	136.643521	78.8920743	82.7339859	78.7911388
1.15E-21	4801.69645	4719.46744	4189.7858	2599.42699	2432.21854	2861.46355
0.00027886	7414.99987	7746.05043	8350.02705	9563.32398	9084.6736	9171.48073
0.00570136	9791.77682	10041.5036	9234.51687	10705.2533	10724.0917	11468.915
5.12E-07	8552.62934	8500.73341	9204.04906	11379.1788	11121.6968	10385.0564
9.36E-25	1301.94211	1246.35551	1347.0466	2159.50339	2167.14849	2076.43477
1.97E-34	1247.52009	1232.61616	1328.58126	600.382057	524.517406	459.294687
4.75E-10	264.784046	206.090281	196.655878	85.5778433	91.5696543	108.578033
0.00026334	1760.34295	1797.89236	1816.98952	1535.05256	1506.07984	1337.52762
1.31E-16	3992.69224	3925.52917	4405.83029	2838.77752	2756.72854	2712.52908
0.01217646	123.496116	80.4733479	79.4009649	50.8118444	51.4075252	67.2607283
3.93E-32	3120.89338	3155.14407	3206.50641	1704.8711	1843.44172	1566.2141
0.00284995	13003.7224	13230.9961	14998.473	16485.7692	16901.8304	15148.0769
1.33E-32	6412.37885	6183.68982	7282.73036	3506.01727	3260.36164	2913.3504
3.45E-10	4130.84043	4268.03159	4564.63221	5839.35065	5949.6178	5509.61451
2.46E-93	210.362029	243.382808	233.58656	1020.24835	966.300826	982.006632
5.62E-18	2770.29	2761.60977	2786.41991	1865.32955	1963.12487	1817.00053
3.89E-167	31291.6134	30241.2953	29783.6712	11503.5341	11759.4714	11988.7444
4.64E-11	1379.38882	1315.05227	1188.24467	1795.79755	1920.55301	2045.68701
1.14E-06	3344.86091	3483.90714	3832.48146	4567.71738	4657.20049	4384.43861
0.00786736	4144.44594	4199.33483	4745.59255	5224.2599	5035.52774	4845.65504
4.09E-09	1450.55608	1426.92985	1397.82629	1908.11847	1842.63848	1898.67427
1.63E-05	857.146773	730.148425	637.97752	1032.28273	1022.52781	1068.48471
2.67E-07	344.323918	347.409331	374.846416	593.696288	507.649312	520.79021
1.28E-16	1316.59419	1347.43789	1336.89066	1954.91886	1927.7822	1988.03495
8.99E-14	393.513049	511.300174	443.168176	782.234973	839.388498	793.676593
0.06831953	722.138307	758.608512	641.670588	751.480436	809.668522	894.567686
2.04E-56	5596.04859	5673.37103	5926.45109	10495.3202	10379.5006	10096.7962
1.47E-08	563.058564	555.462377	553.96022	385.100295	325.313246	369.934005
3.11E-32	604.921655	559.387906	520.722607	1129.89496	1228.96115	1268.34516
2.98E-37	2554.69508	2509.39452	2541.75414	4265.52062	4451.57039	4762.05956
6.47E-05	133.961889	109.914817	155.108862	201.910224	240.169532	220.999536
0.024396	2919.95055	2859.748	2807.65505	3189.11182	3063.56721	3287.12788
1.13E-09	580.850378	512.281556	580.734964	353.008603	351.017008	342.068847

0.00600111	16103.6843	15598.0901	15884.8093	17178.4149	17525.1466	17895.1972
1.39E-06	120.356385	144.263197	153.262328	80.2292281	57.0302233	57.6520528
2.27E-08	455.261107	401.385357	312.987524	183.190071	187.958764	230.608211
2.54E-65	3393.00347	3185.56692	3329.30092	6314.04025	6455.66063	6553.11667
0.00084728	11900.63	11639.194	11142.9098	12944.9859	13241.454	13234.9896
7.48E-29	4618.54543	4533.98619	4472.30551	6733.90654	7127.97467	6947.07236
0.00020634	787.026097	698.744192	919.573966	1144.60365	1119.72016	992.576176
6.42E-12	163.266052	163.890843	189.269742	370.391603	338.968369	318.047158
4.62E-20	544.220174	538.778878	522.569141	254.059222	271.495993	264.238575
6.81E-36	6826.82345	6808.83034	6466.5623	3719.96187	3784.87904	4105.78703
3.43E-06	3735.23423	4082.55033	4283.03577	5246.99151	5353.61181	4842.77243
0.03573108	554.685946	523.076762	530.878544	632.473748	628.135699	609.190024
6.29E-05	2140.25049	2299.37871	2750.41249	3556.82911	3359.96372	2869.15049
2.41E-06	14953.4958	16309.5923	14374.3444	18189.3032	19281.0349	20809.5085
0.00204482	5377.31395	5705.75664	5366.95127	6121.4901	6209.8684	6378.23877
1.28E-17	672.949176	717.390455	761.695303	1171.34673	1247.43573	1173.21927
7.18E-07	970.177117	937.220089	823.554194	545.558751	618.496788	683.176826
3.52E-43	633.17924	602.568727	647.210191	1322.44511	1408.88749	1353.86237
1.94E-08	5923.62727	6150.32282	6156.34458	7591.02213	7658.91802	7330.45851
4.78E-15	4960.7762	5028.60286	5354.02553	3727.9848	3718.20991	3648.41407
0.0002875	1080.06773	1209.06298	1204.86348	1450.81187	1455.47556	1397.10141
5.01E-26	1391.94775	1346.4565	1314.73226	731.423129	775.129091	769.654905
1.07E-22	307.693714	278.712571	334.222666	671.251208	633.758397	650.507329
3.96E-06	12192.625	11790.3269	12051.4046	10251.9582	10103.1852	10300.5001
1.34E-23	470.959766	483.82147	434.858773	171.155687	198.400918	196.977847
9.61E-12	1581.37824	1530.95638	1700.65788	2223.68677	2270.76678	2235.93878
2.10E-07	4560.98369	4556.55798	4570.17182	3670.48718	3733.47152	3798.30941
0.00297558	100.471417	97.1568469	86.7871012	58.8347672	58.6367085	52.8477151
2.90E-34	1400.32037	1452.44579	1584.32623	740.783206	631.348669	633.211713
3.86E-12	1069.60196	1265.98316	1448.60598	750.143282	738.179933	652.429064
8.58E-05	1240.19405	1317.99642	1157.77686	1496.2751	1547.84845	1613.29661
1.45E-22	2349.56594	2364.14994	2338.6354	3522.06311	3530.25115	3414.92326
0.0035814	445.841911	387.646005	359.150876	455.969446	522.910921	617.837832
4.51E-07	4434.34784	4579.12977	4521.23866	3674.49864	3724.63585	3728.16608
2.33E-05	326.532104	407.273651	417.316699	538.872982	546.204955	575.55966
7.07E-13	2378.8701	2219.88674	2706.09568	4133.1424	3921.43028	3508.12741
8.42E-21	343.27734	377.832182	340.685535	135.052534	125.305843	150.856205
3.81E-17	152.80028	138.374903	158.80193	37.4403064	41.7686142	37.4738343
6.05E-42	1065.41565	1044.19076	939.88584	406.494755	322.100275	364.1688
2.71E-12	703.299917	604.531492	622.281981	359.694372	368.688345	396.838297
7.82E-08	3765.58497	3844.07444	4111.3081	5395.41559	5253.20648	4749.56828
4.21E-96	3922.57156	3902.95737	4229.48628	1577.84149	1483.58905	1552.76196
0.02118491	30.3507405	32.3856156	28.621278	16.0458456	6.42594065	18.2564834

5.93E-10	1652.54549	1821.44553	1944.40037	2707.73645	2804.9231	2414.66014
2.61E-11	1947.68028	1906.82579	1775.44251	2586.05545	2516.55901	2664.48571
6.39E-10	3532.19824	3523.16243	3805.70671	4724.16438	4860.42086	4626.57724
2.88E-10	458.400839	474.007647	410.85383	223.304685	252.218171	276.729853
3.73E-30	702.253339	609.438403	612.126043	1256.92457	1330.97296	1415.3579
2.95E-13	2918.90397	2954.94208	3169.57573	4110.41078	4139.10902	4110.59136
5.17E-07	984.829199	1051.06043	900.185358	714.04013	697.214561	706.237647
4.46E-27	2094.20109	2067.77249	1867.76921	3314.80427	3388.88045	3555.20992
1.56E-10	1017.27309	1102.09231	1112.53678	1472.20633	1603.27219	1523.93593
3.46E-10	11683.9885	11578.3483	10804.9941	8485.57802	8534.45243	9088.84612
8.30E-06	4556.79738	4586.98083	4722.51088	3588.9208	3937.49514	3848.27452
2.55E-08	1925.70215	1938.23003	1974.86819	2438.96853	2517.36225	2489.60781
1.80E-06	1641.03314	1708.58657	1890.85089	2412.22546	2334.22294	2160.99111
2.11E-17	7097.88696	6793.12822	6988.20818	9456.35168	9422.03548	9956.50952
4.74E-16	9677.69989	9663.67143	10503.0858	14522.8274	14220.6067	13469.4413
8.02E-10	3531.15166	3688.03465	4083.61009	5912.89411	5636.3532	4899.46362
0.0053865	47.0959766	48.0877323	72.0148286	33.428845	26.5070052	24.0216887
0.01041883	264.784046	369.981124	344.378604	496.08406	445.799633	374.738343
1.97E-05	17.7918134	21.5904104	18.4653407	40.114614	61.8496788	75.9085362
1.01E-41	2032.45303	1897.01197	1858.53654	3507.35442	3498.92469	3641.688
2.55E-08	230.246997	205.108899	194.809344	106.972304	114.060447	93.204152
1.14E-07	4813.2088	5210.15859	5586.68882	6899.71361	7006.68504	6506.99502
1.35E-46	3366.83904	3338.66256	3471.48405	6134.86164	6127.93766	5784.42263
1.17E-07	2208.27801	2174.74316	2232.45969	1672.7794	1747.05262	1762.23108
0.00098708	6667.7437	6405.48222	6291.14157	5669.53212	5569.68406	5732.53578
2.13E-28	1618.00844	1583.95102	1339.66047	643.170978	630.545427	751.398421
0.02539321	223.967533	205.108899	247.435565	185.864378	168.680942	169.112688
6.90E-08	10832.0746	10634.2585	9337.92278	13156.2562	13415.7576	15016.438
4.76E-10	1026.69229	977.456763	1017.44027	1337.1538	1468.32744	1437.45785
0.00063751	84.7727578	105.989288	93.2499704	168.481379	155.829061	137.404059
0.0023446	1359.50386	1504.45905	1533.54654	1801.14617	1784.80502	1662.30086
1.91E-13	2350.61252	2351.39197	2174.29386	3128.93989	3241.88706	3428.37541
2.39E-05	1976.98444	2091.32566	2179.83347	2771.91983	2629.81621	2450.21224
0.01480678	1450.55608	1534.8819	1865.92268	2080.61131	2031.40049	1772.80062
5.36E-08	66.9809444	59.8643198	68.3217605	18.7201532	17.6713368	17.2956158
0.04590207	3542.66401	3641.90969	4295.96151	4694.74699	4425.06338	4048.13497
1.22E-10	1199.37754	1037.32108	1219.63575	1699.52248	1715.72615	1642.12264
2.13E-61	5973.86298	5757.76991	4417.83276	1137.91788	1355.07024	1520.09246
5.33E-32	2271.07265	2264.04895	1651.72472	599.044903	649.823249	799.441799
2.57E-06	2223.97667	2396.53556	2420.80616	2983.19013	2944.6873	2852.81575
4.70E-17	2337.00701	2425.97703	2803.96198	4483.47669	4290.11863	3872.29621
9.62E-09	271.06351	286.563629	267.74744	151.098379	159.845274	147.012735
0.00380495	43.9562448	45.1435854	48.9331528	12.0343842	27.3102478	20.1782185

1.55E-28	288.855323	305.209893	239.126162	685.9599	689.985378	666.842077
0.01197261	87.9124896	65.7526136	75.7078968	56.1604596	36.1459162	45.1607747
6.31E-27	1770.80872	1698.77275	1506.7718	2886.91506	3133.44931	3314.03217
9.72E-11	171.63867	171.741901	170.804401	82.9035357	67.4723769	59.5737879
9.13E-14	3067.51794	3156.12545	3212.04601	2315.95038	2293.25757	2308.00385
8.53E-49	982.736044	1029.47002	1065.45016	347.659988	315.674335	392.994827
1.09E-11	433.282984	459.286913	516.106272	889.207278	836.97877	717.768057
0.00236032	2880.18061	2947.09102	3057.86042	3631.70972	3447.51716	3254.45838
6.81E-05	1172.16653	1133.49655	1347.96987	936.007661	975.939737	845.563441
1.74E-27	3016.23565	3029.52713	2887.05601	4551.67154	4696.55937	4915.79837
1.03E-12	1453.69581	1641.85257	1721.89302	1087.10604	975.939737	897.450288
7.07E-30	1155.42129	1060.87426	1120.84618	1977.65047	2147.06742	1960.16979
9.07E-06	14.6520816	6.86967604	19.3886077	64.1833824	61.0464362	37.4738343
1.50E-35	1582.42481	1574.1372	1716.35342	2886.91506	2934.24515	2853.77661
8.82E-17	3275.78681	3344.55085	3217.58561	2330.65907	2343.86185	2321.45599
4.04E-18	323.392372	351.33486	361.920677	664.565439	742.999388	630.32911
4.11E-07	59.6549036	55.9387906	56.3192891	117.669534	126.912328	128.756251
0.02123767	1395.08748	1539.78882	1751.43756	1989.68486	1885.21034	1695.93122
2.24E-19	1181.58572	1167.84493	1111.61351	1793.12325	1764.72395	1863.12217
1.39E-08	2043.96538	1934.3045	1896.39049	2461.70015	2598.48975	2670.25091
4.16E-42	722.138307	750.757453	721.071553	1568.48141	1487.60526	1479.73602
0.01598668	11480.9525	11137.7076	11497.4444	12232.283	12532.1908	12529.7128
0.08404448	414.444594	427.882679	461.633517	419.866293	350.213766	341.107979
0.00010153	490.844733	462.231059	455.170648	361.031526	316.477577	349.755787
2.81E-06	3779.19047	3617.37513	3530.57314	4454.05931	4404.17908	4399.8125
0.01243127	3263.22789	3051.11755	3167.72919	2844.12613	2837.0528	2785.55502
2.72E-13	1066.46222	1003.95408	1057.14075	695.319976	686.772407	661.076872
0.07221153	23.0246997	22.5717927	14.7722725	8.02292281	12.0486387	7.68694037
8.57E-43	144.427661	136.412139	155.108862	578.987596	489.977975	504.455462
0.00460568	4.18630903	1.96276458	9.23267034	14.7086918	20.8843071	25.9434238
0.00270451	266.877201	275.768424	281.596445	221.967531	200.810645	184.486569
2.43E-05	2248.04795	2080.53046	1773.59597	1383.95418	1437.80422	1642.12264
0.03116853	1057.04303	872.448857	1015.59374	1276.98188	1082.771	1125.1759
3.19E-23	1881.74591	1793.96683	1730.20242	2761.2226	2910.14787	2971.96332
0.00022696	509.683124	582.941081	529.955277	383.763141	432.947751	381.464416
0.07783191	93.1453759	123.654169	116.331646	80.2292281	84.3404711	83.5954765
2.32E-34	3011.00277	3114.90739	3041.24161	4839.15961	5185.73411	5041.67202
2.37E-16	3790.70282	3637.00277	3464.09791	5117.2876	5250.79676	5680.64893
1.86E-05	2183.16016	1956.87629	1792.98458	1370.58265	1391.21615	1623.86615
2.94E-17	271.06351	245.345573	239.126162	468.00383	538.975772	573.637925
1.49E-21	574.570914	498.542204	431.165705	175.167148	159.845274	208.508258
2.19E-13	1069.60196	1046.15352	1046.98482	613.753595	665.084858	717.768057
1.19E-19	705.393071	761.552658	733.997292	374.403064	412.063444	382.425283

1.99E-37	4386.20528	4594.83189	4144.54571	2385.48238	2426.59584	2517.47297
1.84E-09	572.47776	579.996934	491.178062	274.116529	301.215968	360.32533
3.02E-06	4350.62166	4668.43556	5104.74343	3809.55118	3640.29538	3279.44094
2.80E-05	1801.15946	1824.38968	1622.18018	1191.40404	1260.28761	1475.89255
2.09E-08	435.376139	417.087474	466.249852	267.43076	277.921933	281.534191
3.47E-07	120.356385	115.80311	192.96281	353.008603	334.148914	245.982092
0.00010197	905.289327	848.895682	938.039306	758.166205	650.626491	588.050938
6.58E-129	53.3754401	59.8643198	63.7054253	834.383972	866.698746	713.924587
3.65E-20	13183.7337	13861.0435	12658.9143	8683.47678	8652.52909	9355.00643
8.27E-11	1397.18064	1298.36877	1415.36836	1984.33624	1962.32163	1832.37441
5.16E-06	1161.70076	1150.18005	1385.82382	1785.10032	1638.61487	1601.7662
4.47E-09	941.919531	1093.25987	1030.36601	1600.5731	1681.98997	1361.54931
5.72E-05	1974.89128	2110.95331	1950.86324	2405.53969	2455.51257	2440.60357
0.01003757	1805.34577	1730.17698	1632.33612	1829.2264	2120.56042	2093.73038
1.11E-16	3353.23353	3406.37793	3680.1424	5130.65913	5013.84019	4887.93321
0.00196611	1536.37541	1545.67711	1620.33364	1371.9198	1324.54702	1309.66247
3.05E-17	187.337329	175.66743	174.497469	399.808986	367.885102	389.151356
4.88E-05	127.682425	145.244579	137.566788	228.6533	206.433343	219.077801
6.79E-23	2267.93292	2389.66588	2418.03636	3562.17773	3543.90627	3584.99682
0.00070506	1497.65205	1487.77555	1506.7718	1674.11656	1833.80281	1863.12217
0.00031206	755.628779	754.682982	759.848769	537.535828	628.938941	594.777011
1.17E-65	2129.78472	2152.17137	2202.91514	4205.3487	4343.13264	4269.13451
0.00086689	2186.29989	2288.5835	2417.11309	2801.33721	2665.15889	2669.29004
0.00150185	857.146773	849.877065	890.952688	755.491897	692.395105	667.802945
0.00533876	143.381084	176.648813	207.735083	264.756453	240.169532	242.138622
3.57E-06	509.683124	525.039526	580.734964	815.663819	725.328051	726.415865
1.06E-05	207.222297	209.034428	205.888549	128.366765	122.896115	125.873649
0.00068453	1403.4601	1560.39784	1566.78416	1817.19202	1779.98556	1857.35697
1.78E-10	100.471417	78.5105833	84.9405671	24.0687684	24.90052	14.4130132
3.15E-27	1048.67041	1105.03646	1014.67047	516.141367	546.204955	562.107515
7.03E-05	220.827801	228.662074	163.418265	121.680996	106.028021	127.795384
5.19E-16	4.18630903	1.96276458	1.84653407	56.1604596	90.7664117	103.773695
0.0109487	1052.85672	1144.29175	974.969988	783.572127	891.599266	941.650196
4.10E-06	463.633725	447.510325	498.564198	326.265527	330.132701	319.008025
0.00033027	136.055043	124.635551	136.643521	187.201532	236.153319	194.095244
0.00221183	143.381084	161.928078	148.645992	72.2063052	106.831263	104.734563
0.05228545	2492.94703	2573.18437	2421.72943	2198.28085	2198.47495	2372.38197
1.21E-12	1773.94845	1743.91633	1761.5935	1260.93603	1273.94273	1215.49745
0.06452456	367.348617	319.930627	337.915734	363.705834	414.473172	441.999071
3.42E-21	543.173596	600.605963	559.499822	275.453683	248.201958	279.612456
2.57E-05	2034.54619	1922.52791	2061.65529	1715.56833	1544.63548	1542.19241
2.79E-14	71.1672535	79.4919656	65.5519594	9.36007661	8.8356684	6.72607283
3.55E-14	733.650657	740.94363	781.083911	454.632292	464.274212	401.642634

1.51E-07	56.5151719	55.9387906	36.0074143	2.6743076	10.4421536	10.569543
1.66E-07	900.056441	887.169592	840.173001	520.152829	579.941144	665.88121
0.00169723	80.5864488	48.0877323	69.2450275	29.4173836	29.7199755	35.5520992
1.77E-08	7871.30755	7978.63803	7554.17087	9467.04891	9638.10774	9918.07481
1.30E-09	754.582202	737.018101	844.789336	508.118444	529.336861	470.825098
0.00183516	2236.5356	2405.368	2647.00659	3146.32289	3039.46993	2705.80301
1.71E-06	653.064208	661.451665	587.197833	439.9236	394.392108	469.86423
0.04110718	1082.16088	905.815855	909.418028	1077.74596	1112.49098	1164.57147
0.05946866	12.5589271	3.92552917	18.4653407	2.6743076	0	5.76520528
0.00010392	35.5836267	43.1808208	34.1608802	10.6972304	11.2453961	11.5304106
8.56E-05	227.107265	245.345573	246.512298	365.042988	349.410523	321.890628
1.25E-06	1450.55608	1515.25426	1353.50947	1123.20919	1064.29642	1102.11508
7.52E-17	2892.73954	2899.98467	3025.54607	2044.50816	1990.43512	2090.84778
0.01381143	1727.89905	1848.92424	1822.52912	2051.19393	1989.63187	2072.5913
5.80E-07	2014.66122	2032.44273	1847.45733	2479.08315	2469.97094	2608.75539
7.15E-32	982.736044	944.089765	981.432857	1778.41456	1693.23536	1804.50925
1.32E-08	941.919531	884.225445	1036.82888	1387.96565	1314.10486	1343.29283
2.60E-33	3381.49112	3758.69418	3808.47651	6451.76709	6371.32016	6164.92618
7.54E-05	1859.76779	1971.59702	2016.4152	2354.72784	2368.76237	2332.9864
9.45E-50	1367.87647	1392.58147	1379.36095	2628.84437	2673.99455	2809.57671
3.49E-06	2452.13051	2259.14204	2271.2369	1738.29994	1810.50878	1925.57856
2.88E-08	1233.91459	1149.19866	1218.71248	1775.74025	1761.51098	1518.17072
8.21E-06	1487.18628	1567.26752	1540.93268	1184.71827	1251.45194	1210.69311
0.01811237	129.77558	152.114255	162.494998	121.680996	89.9631691	103.773695
0.05502366	1532.1891	1422.02294	1296.26692	1089.78035	1269.92652	1312.54507
0.06792063	1219.2625	1423.00432	1370.12828	1571.15572	1506.88308	1445.14479
6.65E-06	871.798855	783.143069	797.702717	645.845286	584.760599	567.87272
2.60E-08	49.1891311	42.1994385	34.1608802	4.0114614	1.60648516	7.68694037
3.80E-29	559.918832	481.858705	448.707778	176.504302	172.697155	149.895337
6.32E-26	304.553982	264.973219	280.673178	617.765056	618.496788	660.116004
3.04E-08	4126.65412	3876.46005	3432.70683	2341.35631	2680.42049	2875.87657
7.17E-25	2735.75295	2583.97957	2341.4052	1228.84434	1238.60006	1465.32301
3.49E-70	4011.53063	4434.86658	4247.95162	8696.84832	8608.35075	8770.79896
2.05E-06	1884.88564	1924.49067	1971.17512	1593.88733	1509.29281	1449.94913
2.66E-20	2847.73672	3104.11219	3106.79357	4455.39646	4543.94328	4503.58619
2.55E-05	993.201817	995.121644	1213.17288	1556.44702	1432.98477	1356.74498
0.003195	1749.87717	1757.65568	1905.62316	1508.30949	1579.97816	1538.34894
0.00015589	2987.97807	2798.9023	2619.30857	3314.80427	3355.14426	3547.52298
3.61E-21	2169.55465	2122.7299	1963.78898	1216.80996	1282.7784	1316.38854
2.26E-18	1243.33378	1222.80234	1092.2249	623.113671	677.936739	710.081117
1.08E-19	1289.38318	1315.05227	1513.23467	2499.14045	2303.69972	2253.2344
1.42E-09	176.871556	160.946696	193.886077	78.8920743	40.1621291	72.065066
8.66E-05	341.184186	381.757712	389.618688	522.827136	528.533619	478.512038

5.31E-50	6435.40355	6130.69518	6166.50052	3265.32958	3524.62845	3316.91477
0.00445946	3488.242	3441.7077	3111.4099	3789.49387	3833.87684	3938.59607
0.02738262	30.3507405	31.4042333	53.549488	18.7201532	17.6713368	22.0999536
4.32E-19	1901.63088	1792.00406	1961.01918	2849.47475	2816.16849	2823.98972
1.40E-14	1054.94987	1028.48864	1092.2249	1711.55687	1673.95754	1512.40552
0.00044242	878.078319	866.560564	903.878426	708.691514	726.934536	703.355044
2.37E-08	358.975999	404.329504	422.856301	636.485209	627.332456	571.71619
6.05E-05	5608.60752	5168.94053	4661.57525	3586.24649	3878.85843	4372.9082
0.00016907	714.812266	650.656459	743.229962	537.535828	543.795228	537.124958
0.00910066	372.581503	342.50242	313.910791	294.173836	251.414928	249.825562
2.89E-10	493.984465	462.231059	565.962692	764.851974	830.552829	832.111295
0.02476677	717.951998	776.273393	789.393314	853.104125	911.68033	880.154673
2.35E-06	515.962588	622.196373	632.437918	917.287507	828.946344	806.167871
0.00024815	3.13973177	1.96276458	4.61633517	26.743076	13.6551239	24.0216887
9.94E-44	4760.87994	4482.95431	4606.17923	2648.90168	2665.96213	2615.48146
0.00303175	1219.2625	1159.99387	1319.34859	1556.44702	1451.45934	1422.08397
2.33E-17	948.198995	1007.87961	1034.98234	1565.8071	1594.43652	1519.13159
1.50E-34	1712.20039	1773.3578	1923.16523	3641.0698	3465.99174	3223.71062
0.04382391	2677.14462	2688.98748	2505.74673	2804.01152	2857.13386	3097.83697
0.0001031	203.035988	136.412139	120.024714	74.8806128	70.6853472	87.4389467
0.0007045	1298.80238	1259.11348	1095.91797	1397.32572	1554.2744	1717.07031
1.12E-07	442.70218	477.933176	488.408261	299.522451	322.903518	263.277708
2.28E-23	3776.05074	3825.42817	3696.7612	2532.5693	2489.24876	2557.82941
6.81E-20	2074.31612	2196.33357	2062.57855	1305.06211	1289.20434	1401.90575
1.71E-20	3179.50171	3112.94463	3015.39013	2096.65716	2073.1691	2111.026
0.02589284	3.13973177	2.94414688	2.7698011	14.7086918	11.2453961	8.64780792
2.01E-05	2709.58852	2998.1229	3212.04601	4097.03925	4016.21291	3514.85348
0.03836183	1533.23568	1838.12903	2215.84088	2619.4843	2321.37106	1950.56112
0.00016514	272.110087	250.252484	254.821701	168.481379	138.157724	193.134377
0.07833435	1714.29355	1882.29124	2032.11074	2237.05831	2160.72254	1950.56112
0.00691314	24.0712769	14.7207344	36.0074143	9.36007661	9.63891098	0.96086755
0.03141436	54.4220174	52.9946438	48.0098858	29.4173836	33.7361884	29.7868939
5.86E-13	335.951299	273.805659	315.757326	569.627519	588.776812	526.555415
1.27E-08	2105.71344	1960.80182	1915.7791	2533.90645	2653.11025	2737.51164
7.18E-05	1459.97527	1518.19841	1249.1803	930.659045	980.759192	1173.21927
1.05E-07	5.23288628	4.90691146	5.5396022	40.114614	32.9329458	41.3173045
2.77E-08	1625.33448	1497.58938	1344.2768	2020.43939	2048.26858	2244.58659
7.84E-24	660.390249	598.643198	648.133458	1283.66765	1175.94714	1130.9411
2.04E-05	1381.48198	1413.1905	1496.61586	1892.07263	1800.06663	1713.22684
9.19E-48	1249.61324	1315.05227	1257.4897	2504.48907	2483.62606	2670.25091
5.07E-10	440.609025	456.342766	528.108743	787.583589	794.406913	707.198514
1.19E-05	1242.2872	1392.58147	1111.61351	1601.91025	1657.89269	1898.67427
2.77E-10	6252.25253	5902.0331	6148.03518	7732.76043	7678.99908	8115.4873

1.25E-12	9409.77612	9248.54672	7908.70541	12454.2505	13228.6021	14286.1787
8.41E-11	14095.3025	12391.9142	12252.6768	17072.7797	17886.6058	18431.3613
6.45E-08	8677.17204	8075.79488	7644.65104	10312.1301	10199.5743	10922.1814
0.00046416	14855.1176	14337.9953	13718.8249	12416.8102	12073.5392	12704.5907
2.51E-57	1564.633	1533.90052	1446.75944	3118.24266	3053.9283	3182.39331
2.95E-07	147.567393	154.07702	185.576674	284.81376	306.035424	264.238575
1.14E-14	130.822157	146.225961	153.262328	359.694372	316.477577	297.868939
0.02052702	11826.323	11782.4758	9903.88547	11999.6182	12792.4414	14340.9481
0.00012091	10596.5947	9959.0675	8172.75978	11647.9468	12279.1693	14092.0834
1.64E-07	593.409305	524.058144	569.65576	784.909281	807.258795	789.833123
1.99E-11	11475.7196	10437.0007	10971.1822	15323.7826	14488.0864	14136.2833
0.09022104	9871.31669	8711.7306	8753.49475	8513.65825	8027.60636	8440.26053
0.00017905	16755.7019	15950.4064	15110.1883	18118.434	18983.8352	19132.7946
0.06016026	14590.3335	15770.8134	12541.6594	15005.54	16005.4117	18266.0921
6.66E-07	13853.5431	14236.9129	12460.4119	10464.5656	10320.8639	11087.4506
0.01312421	93.1453759	108.933434	93.2499704	127.029611	139.764209	154.699675
0.01969553	21025.7371	19923.0419	19859.4739	22520.3443	21637.7487	22559.2483
8.32E-37	1667.19757	1926.45344	1914.85583	3657.11565	3994.52536	3554.24905
0.00033035	39776.2152	37547.6865	32287.5714	42435.913	45342.2405	50955.7669
6.41E-19	1037.15806	1001.00994	977.739789	1668.76794	1628.97596	1531.62287
6.60E-06	1403.4601	1331.73577	1210.40308	1621.96756	1730.98776	1837.17875
5.48E-39	340.137608	289.507776	328.683064	871.824278	798.423126	814.815679
0.08321966	2414.45373	2626.17901	2813.19465	2991.21305	2939.86785	2775.94634
0.01484921	8680.31177	8505.64032	6931.88889	8727.60286	9415.60954	10827.0555
0.06467247	9049.75354	8147.43579	6489.64398	8136.58088	9258.174	10720.3992
0.02086143	6635.29981	6183.68982	5688.2482	6477.17301	7257.29672	7558.18412
2.77E-09	2550.50878	2232.64471	2460.50665	1807.83194	1773.55962	1694.97035
0.00307507	1783.36765	1804.76203	1792.06131	2024.45085	2031.40049	2159.06938
0.00217016	1109.37189	1100.12955	1237.17783	1385.29134	1343.82484	1403.82749
0.00156667	1.04657726	1.96276458	1.84653407	16.0458456	10.4421536	14.4130132
6.71E-06	6031.42473	5991.33889	5697.48087	4708.11853	4678.0848	5099.32407
2.48E-06	232.340151	234.550368	264.054372	365.042988	390.375895	362.247065
0.036168	2700.16932	2656.60186	2286.00918	2035.14808	2155.09985	2411.77754
8.51E-17	12787.0809	11967.957	12548.1223	17535.4349	16589.369	17110.1684
0.00096905	3599.17919	3540.82731	3326.53112	4122.44517	3898.93949	4209.56072
1.05E-10	5.23288628	18.6462635	10.1559374	90.9264585	75.5048027	58.6129203
6.89E-05	1681.84965	1643.81534	1537.23961	1194.07834	1189.60226	1393.25794
8.20E-18	3704.88349	3447.59599	3278.52124	1889.39832	2022.56482	2297.4343
4.66E-12	781.793211	884.225445	858.638341	541.547289	516.48498	539.046694
3.10E-87	3532.19824	3307.25832	3248.97669	7659.21697	7503.8922	7374.65842
3.79E-15	1475.67393	1364.12139	1569.55396	2444.31715	2318.96133	2158.10851
6.38E-19	1091.58008	1212.98851	1085.76203	1783.76317	1822.55742	1950.56112
9.55E-205	1623.24133	1630.07599	1603.71484	5503.72504	5870.09679	5812.28779

2.17E-14	1718.47986	1602.59728	1723.73955	2384.14523	2388.0402	2410.81667
7.04E-07	2047.10511	2174.74316	2358.94727	2877.55498	2968.78458	2741.35511
4.42E-12	2575.62663	2432.8467	2446.65764	3321.49004	3308.55619	3349.58427
5.86E-13	3023.5617	3003.02981	2686.70707	3982.04402	4209.79437	4405.5777
7.93E-06	445.841911	482.840088	470.866187	663.228285	640.184338	614.95523
1.10E-41	1114.60478	1032.41417	1007.28433	2107.35439	2369.56562	2462.70352
1.28E-08	952.385304	899.927562	849.405671	1298.37634	1239.4033	1221.26265
0.00010798	1462.06843	1545.67711	1316.57879	1077.74596	1079.55803	1202.0453
4.31E-05	73.260408	57.9015552	54.472755	153.772687	115.666932	107.617165
7.45E-49	4818.44169	4707.69085	4897.93161	8791.78624	8288.6602	8303.81734
1.06E-35	2171.64781	2168.85486	1845.6108	4183.95424	4388.91747	5009.00252
0.00379034	1049.71699	1090.31573	1167.00953	1346.51388	1326.95674	1253.93215
3.44E-06	699.113608	750.757453	832.786864	588.347672	506.846069	464.099025
6.65E-58	2964.95337	2747.87042	2881.51641	5432.85589	5547.19327	5433.70598
7.07E-07	153.846857	141.31905	159.725197	78.8920743	69.078862	79.7520064
1.04E-11	3104.14814	3003.02981	3097.5609	2365.42507	2251.48896	2178.28673
0.00470323	897.963286	796.882421	763.541837	641.833824	681.149709	680.294223
7.26E-05	646.784745	622.196373	661.059196	474.689599	499.616886	491.964184
8.05E-11	10509.7288	11172.056	10756.0609	8493.60094	8297.49587	8610.33408
7.98E-20	23201.5712	22652.2661	21045.872	31719.9625	32937.7653	34758.4226
0.02558154	367.348617	354.279007	333.299399	272.779375	260.250596	314.203688
1.67E-43	4410.27656	4077.64342	4040.21654	2253.10415	2278.7992	2283.02129
9.64E-06	1026.69229	1011.80514	1005.4378	1293.02773	1298.04001	1253.93215
6.39E-14	2693.88986	2693.89439	2945.22184	4224.06886	4004.16427	3813.68329
4.27E-12	2405.03454	2213.99845	2218.61068	3104.87113	3082.84503	3228.51496
1.99E-14	2600.74448	2674.26675	2923.06343	4200.00009	4039.50694	3811.76156
0.00021766	11535.3745	10981.6678	10977.645	13126.8389	12667.1355	12743.0254
0.00140987	3.13973177	4.90691146	7.38613627	16.0458456	22.4907923	26.9042913
6.91E-09	8551.58277	8084.62732	7648.34411	10255.9697	10279.0953	10694.4558
5.76E-13	1845.1157	1760.59983	1734.81876	1196.75265	1220.92872	1284.67991
9.19E-06	63418.3955	54459.8475	53982.5002	43755.6838	44572.7341	47324.6484
9.12E-05	947.152418	933.29456	903.878426	1123.20919	1199.24117	1146.31498
0.00342385	1.04657726	0.98138229	2.7698011	12.0343842	12.0486387	12.4912781
3.16E-09	49.1891311	59.8643198	55.396022	6.685769	6.42594065	13.4521457
0.02547839	11461.0675	11078.8247	8370.33893	5922.25418	6944.03212	8630.5123
6.50E-14	2909.48477	2739.03798	2720.86795	1842.59794	2016.94212	1965.935
1.64E-15	2082.68874	2088.38152	1620.33364	834.383972	920.515999	1123.25416
0.00092847	1653.59207	1893.08644	1934.24444	2444.31715	2274.78299	2082.19997
1.40E-25	192.570215	165.853607	198.502412	25.4059222	36.9491588	37.4738343
2.76E-07	3165.8962	3114.90739	3320.99152	4191.97717	4088.50474	3853.07886
9.66E-06	985.875776	1002.9727	1262.10604	1734.28848	1640.22135	1364.43192
0.00032995	1716.3867	1656.57331	1627.71978	1263.61034	1332.57944	1462.44041
4.42E-26	1313.45446	1287.57357	1107.92044	510.792752	593.596268	612.072627

1.57E-10	1406.59983	1410.24635	1323.96493	927.984738	1001.6435	982.006632
0.00037641	7084.28145	7299.52149	7138.70071	6291.30863	6288.58617	6302.33024
2.62E-12	5849.32029	5847.07569	5225.69141	3813.56264	4056.37504	4138.45652
1.96E-43	1028.78544	1062.83702	1080.22243	415.854832	425.718568	470.825098
7.16E-56	9741.54111	9863.87342	8799.6581	19061.1274	20312.3984	21596.459
0.00029257	702.253339	785.105833	721.99482	580.32475	585.563842	558.264044
2.53E-09	6532.73524	5984.46922	5311.55525	3674.49864	3985.68969	4421.91245
8.12E-09	2873.90115	2903.9102	2520.519	3586.24649	4000.9513	4421.91245
0.01589816	238.619615	276.749806	288.059315	350.334296	339.771612	327.655833
5.15E-26	5203.58212	5238.61867	5179.52806	7516.14151	7703.09636	8012.67447
9.40E-15	1237.05432	1182.56566	1178.08874	1747.66002	1739.02019	1896.75254
5.94E-06	1530.09595	1555.49093	1681.26927	2155.49193	2087.62747	1907.32208
1.79E-14	1760.34295	1985.33638	2042.26668	3021.96759	3016.17589	2781.71155
1.76E-23	154.893434	158.983931	179.113805	387.774602	428.931539	441.999071
0.00120025	1633.7071	1546.65849	1984.10086	1420.05734	1384.79021	1191.47576
8.87E-09	662.483404	595.699051	637.054253	413.180524	402.424533	434.312131
0.00516282	514.91601	445.54756	515.183005	401.14614	404.031019	351.677522
1.29E-39	891.683823	852.821212	954.658113	326.265527	367.08186	298.829807
4.47E-06	33.4904722	29.4414688	16.6188066	0	0.80324258	1.92173509
7.88E-05	1984.31048	1933.32311	2056.11568	2368.09938	2466.75797	2355.08636
3.48E-11	6607.04222	6581.14965	7173.78485	5051.76706	5106.21309	4550.6687
7.29E-05	232.340151	198.239223	179.113805	116.332381	134.141511	113.38237
5.86E-24	742.023275	738.980866	666.598798	330.276989	355.836464	344.951449
3.73E-266	22016.8458	22624.7874	24668.7719	5129.32198	4872.4695	4733.23353
5.48E-27	2586.0924	2563.37055	2713.48181	4141.16532	4253.16947	4012.58287
2.35E-14	338.044454	343.483802	352.688007	171.155687	156.632303	175.838761
3.57E-11	1919.42269	1912.71409	2149.36565	2991.21305	2928.62245	2679.85959
5.40E-06	1362.64359	1454.40856	1575.09356	2041.83385	1892.43952	1822.76574
0.00014386	266.877201	309.135422	347.148405	518.815675	471.503395	390.112224
5.88E-36	417.584326	390.590152	391.465222	102.960843	118.879902	122.030178
6.48E-11	1231.82143	1141.34761	1312.88572	837.058279	786.374487	662.03774
0.06487525	56.5151719	46.1249677	54.472755	82.9035357	86.7501988	60.5346554
2.43E-76	1529.04937	1541.75158	1698.81134	548.233058	534.959559	535.203223
9.86E-20	1189.95834	1207.10022	1158.70013	1821.20348	1806.49257	1866.96564
3.16E-08	2459.45655	2281.71383	2180.75673	1595.22448	1686.80942	1798.74405
1.60E-05	1084.25404	1109.94337	931.576437	758.166205	769.506393	799.441799
0.0399672	241.759346	179.592959	158.80193	120.343842	144.583665	156.62141
2.15E-14	1245.42694	1310.14536	1487.38319	2287.87015	2232.21113	2010.13491
1.89E-30	397.699358	407.273651	401.62116	831.709664	832.962557	839.798236
7.46E-14	2527.48408	2726.28001	2770.72437	3731.99626	3812.18929	3672.43576
3.83E-269	2919.95055	3052.09893	2830.73673	466.666676	468.290425	454.490349
4.56E-05	3074.84398	3147.29301	3311.75885	2701.05068	2657.12646	2523.23818
0.08308813	15887.0428	16123.1297	15446.2575	14401.1464	14826.2516	15068.3249

1.40E-16	11261.1713	11937.5342	11763.3453	15624.6422	15504.9916	15869.6884
1.60E-10	3199.38667	3168.88342	2924.90996	4110.41078	4059.58801	4382.51688
4.52E-17	1009.94705	1116.81305	1058.98729	1774.40309	1707.69373	1599.84446
2.66E-07	2487.71414	2432.8467	2061.65529	1391.97711	1525.35766	1799.70491
8.24E-39	9123.01395	8888.37942	8023.19052	4158.54832	4396.14665	4742.84221
3.13E-05	706.439648	635.935725	666.598798	458.643754	505.239584	516.94674
5.47E-12	4234.45158	4469.21496	4237.79569	3205.15766	3201.72493	3320.75824
3.42E-06	158.033166	184.499871	179.113805	104.297996	93.979382	66.2998607
2.27E-66	2450.03736	2719.41033	2714.40508	1087.10604	1061.08345	986.81097
0.01170245	36.630204	44.1622031	27.698011	10.6972304	14.4583665	22.0999536
1.77E-16	4438.53415	4227.79491	3928.50123	2587.3926	2610.53839	2919.11561
0.00568849	567.244873	515.225703	489.331528	692.645669	621.709758	632.250846
0.04399503	43.9562448	33.3669979	44.3168176	12.0343842	25.7037626	26.9042913
0.05273013	4468.88489	4806.81047	4933.01576	5190.83105	5249.19027	5127.18923
4.43E-13	86.8659123	83.4174948	105.252442	20.057307	16.8680942	20.1782185
1.02E-06	2869.71484	2799.88368	2980.30599	2245.08123	2343.86185	2348.36028
0.00014519	3500.80092	3759.67556	3632.13251	4257.4977	4256.38244	4265.29104
1.19E-13	6235.5073	5913.80969	6139.72577	4583.76323	4569.64705	4637.14678
7.94E-06	551.546214	547.611319	579.811697	422.540601	404.834261	345.912317
0.01166635	646.784745	646.73093	721.071553	898.567354	824.930131	734.102806
8.86E-06	3117.75365	2902.92882	3253.59303	3917.86064	3877.25194	3701.26179
0.00084665	1567.77273	1574.1372	1622.18018	1921.49001	1853.08064	1835.25701
5.08E-09	3920.4784	3949.08234	4211.02094	5168.09944	5106.21309	5090.67626
4.60E-41	744.11643	685.00484	687.83394	274.116529	244.185745	252.708165
6.83E-14	1250.65982	1301.31292	1115.30658	700.668592	652.232976	792.715726
5.97E-100	3135.54546	3104.11219	2691.3234	822.349588	858.66632	902.254626
0.00755059	23.0246997	12.7579698	29.5445451	34.7659988	50.6042826	57.6520528
0.02473307	213.50176	253.196631	273.287042	224.641839	174.30364	154.699675
0.00193339	61.7480582	51.0318792	58.1658231	36.1031526	20.8843071	24.9825562
1.05E-27	3615.92442	3671.35115	3753.08049	2364.08792	2381.61425	2262.84307
1.33E-12	751.44247	779.21754	820.784393	410.506217	481.142306	499.651124
4.63E-12	136.055043	128.56108	167.111333	42.7889216	50.6042826	46.1216422
1.37E-104	4385.15871	4175.78165	3811.24632	1133.90642	1155.86607	1303.89726
0.00042388	140.241352	165.853607	151.415794	86.9149971	85.1437137	105.69543
0.03011139	11.5123498	5.88829375	3.69306814	0	0	1.92173509
2.17E-18	1117.74451	1222.80234	1099.61104	1795.79755	1803.2796	1813.15706
0.00025951	3561.50241	3809.72606	3808.47651	4392.55024	4453.17687	4267.21277
1.07E-27	12177.973	12509.6801	12335.7708	8468.19502	8421.99847	8649.72965
0.06237835	2432.24555	2397.51694	2230.61315	2475.07169	2601.70272	2738.47251
0.00016097	3484.05569	3284.68653	3965.43191	2964.46998	2892.47654	2534.76859
6.77E-06	45.002822	48.0877323	70.1682946	14.7086918	19.277822	6.72607283
0.00031629	143.381084	123.654169	111.715311	90.9264585	53.0140104	56.6911852
0.01842002	63.8412127	98.1382292	103.405908	72.2063052	47.3913123	38.4347019

4.14E-08	236.52646	252.215249	252.975167	131.041072	140.567452	139.325794
1.07E-11	927.26745	838.100477	878.950216	589.684826	571.908718	523.672813
2.01E-11	7619.08243	7895.22054	8112.74743	6110.79287	6152.03493	5839.19208
0.03915012	204.082565	195.295076	207.735083	229.990454	250.611685	279.612456
6.98E-10	3138.68519	3048.1734	3260.97916	2396.17961	2431.41529	2406.9732
0.0176279	633.17924	653.600606	661.059196	799.617973	783.96476	713.924587
2.03E-65	7382.55597	7472.24477	7728.66834	13613.5628	13628.6169	13448.3022
6.20E-12	727.371194	698.744192	663.828997	378.414526	414.473172	454.490349
0.07663196	20.9315451	22.5717927	18.4653407	10.6972304	11.2453961	8.64780792
9.35E-51	2940.88209	2730.20554	3051.39755	5721.68111	5865.27733	5534.59707
1.50E-14	7959.22004	7660.67017	7523.70306	5729.70404	5462.04955	5824.77907
1.28E-18	208.268874	170.760519	180.037072	44.1260754	48.9977975	57.6520528
9.04E-16	108.844035	64.7712313	93.2499704	1.3371538	3.21297033	11.5304106
9.65E-07	4703.31819	4612.49677	4697.58267	3957.97525	3750.33961	3819.4485
1.94E-07	0	0	0.92326703	40.114614	43.3750994	34.5912317
0.06196008	0	0.98138229	0.92326703	2.6743076	6.42594065	6.72607283
4.92E-100	24499.327	25083.15	24687.2372	47574.5951	47840.3249	48506.5155
3.26E-17	704.346494	711.502162	625.975049	1137.91788	1120.5234	1242.40174
2.53E-15	3428.58709	3220.89668	3530.57314	5157.40221	4910.2219	4680.38582
0.0002406	258.504582	311.098187	291.752383	188.538686	204.023616	196.016979
2.68E-226	283.622437	283.619482	318.527127	2023.1137	1996.05782	2002.44797
0.00059653	52.3288628	43.1808208	49.8564198	17.3829994	22.4907923	19.2173509
8.10E-25	779.700056	789.031363	856.791807	387.774602	408.047231	410.290442
4.23E-10	15026.7563	15395.9254	14507.2949	18677.3643	19219.1853	20693.2435
3.81E-16	616.434004	559.387906	587.197833	936.007661	1020.11808	973.358825
5.75E-07	4567.26315	4581.09254	5119.5157	6732.56939	6365.69746	5762.32268
6.06E-30	1131.35001	1141.34761	1058.06402	2003.05639	1902.07843	2026.46966
1.65E-13	11577.2376	10751.043	12144.6546	16975.1675	16269.6785	15431.5328
3.34E-07	3306.13755	3366.14126	3475.17712	4391.21308	4192.12303	4077.92187
3.72E-19	3265.32104	2914.70541	3152.03365	4757.59322	4550.36922	4755.33349
5.27E-20	2951.34786	2679.17366	2859.358	4165.23409	4223.44949	4256.64323
5.83E-22	2492.94703	2422.0515	2718.09815	4492.83677	4335.10021	3928.9874
2.22E-05	2378.8701	2490.74826	2081.04389	2870.86921	2921.39327	3168.94117
4.34E-05	685.508103	647.712313	673.061668	500.095522	518.091465	502.533727
0.00267595	869.705701	928.387648	891.875955	1052.34004	1081.96776	1065.60211
0.00178166	3216.13191	3358.2902	2681.16747	3451.19396	3904.56219	4480.52537
0.00543162	4118.28151	4120.82424	3665.37012	3393.69635	3363.17669	3549.44472
6.42E-74	341.184186	346.427949	354.534541	1088.44319	1142.21095	1066.56298
5.62E-12	3063.33163	3081.5404	3512.1078	2271.82431	2243.45653	2234.97791
5.35E-06	468.866611	491.672528	516.106272	307.545374	350.213766	356.48186
0.00010829	2631.09522	2911.76126	2868.59067	2353.39069	2303.69972	2314.72992
5.32E-15	3537.43113	3426.98696	3539.80581	2508.50053	2569.57302	2369.49937
9.15E-24	3441.14602	3346.51362	3563.81075	2271.82431	2269.96354	2212.87796

1.21E-17	2175.83412	2057.95867	2039.49688	1315.75934	1349.44754	1426.88831
7.12E-09	4288.8736	4390.70437	4030.98387	5380.70689	5414.65824	5919.90495
1.55E-30	705.393071	760.571276	853.098739	1646.03633	1785.60826	1546.99675
1.38E-19	1523.81649	1408.28359	1398.74956	847.75551	902.844662	868.624262
1.06E-07	2915.76424	2652.67633	2928.60303	3959.3124	3653.14726	3557.13166
5.74E-05	1702.7812	1803.78065	1556.62822	1103.15189	1210.48657	1427.84917
3.40E-170	857.146773	866.560564	789.393314	3318.81573	3386.47072	3613.82284
3.23E-237	3245.43607	3468.20502	3524.11027	665.902593	624.119486	645.702991
7.98E-14	601.781923	551.536848	635.207719	306.20822	346.197553	337.264509
4.97E-30	669.809444	679.116546	697.06661	1236.86727	1319.72756	1375.00146
3.62E-05	2096.29425	2091.32566	1898.23702	1650.04779	1642.63108	1623.86615
1.04E-10	7138.70347	7358.40442	7982.56677	5720.34396	5545.58678	5147.36745
3.81E-09	3882.80162	3878.42282	3550.88501	2860.17198	2824.20092	2944.09816
3.59E-09	7840.95681	7769.6036	8571.61114	11039.5418	10674.2907	10065.0875
1.93E-18	1246.47351	1291.4991	1325.81146	2081.94847	2085.21774	1904.43948
4.48E-05	187.337329	191.369547	174.497469	119.006688	115.666932	93.204152
1.07E-23	1796.97315	1868.55188	2067.19489	1104.48904	1024.93753	933.963255
1.91E-06	299.321095	253.196631	362.843944	557.593135	518.894708	444.881674
2.46E-14	2621.67603	2437.75361	2600.84323	1744.98571	1838.62227	1759.34848
3.29E-05	693.880721	708.558015	763.541837	560.267443	541.3855	536.164091
1.91E-06	3368.93219	2775.34912	2612.84571	1834.57501	2040.23616	2232.09531
0.0098727	126.635848	136.412139	117.254913	89.5893047	88.356684	81.6737414
0.00085485	489.798156	476.951794	450.554312	316.905451	381.540226	363.207933
1.10E-06	3008.90961	2808.71612	2920.29363	2388.15669	2343.05861	2191.73887
5.49E-19	1349.03808	1338.60545	1422.7545	873.161432	832.962557	843.641706
2.86E-05	542.127019	581.959699	642.593856	429.22637	442.586662	383.386151
0.00029987	845.634424	790.994127	779.237377	573.638981	643.397308	658.194269
2.09E-12	1641.03314	1659.51746	1756.0539	1228.84434	1122.12989	1128.0585
0.07122678	857.146773	876.374387	768.158172	902.578816	922.925726	1027.16741
0.01353305	720.045153	713.464926	714.608684	850.429817	806.455552	858.054719
4.71E-19	343.27734	283.619482	255.744968	81.5663819	103.618293	99.9302248
0.00014663	1150.18841	1274.8156	1342.43027	1662.08217	1600.05922	1501.83598
0.01409996	669.809444	610.419786	565.039425	732.760283	774.325849	708.159382
2.19E-23	3696.51087	3742.01068	3392.08308	2068.57693	1974.37027	2283.02129
0.00257105	2.09315451	6.86967604	10.1559374	24.0687684	23.2940349	24.0216887
2.87E-10	37.6767812	48.0877323	52.6262209	1.3371538	1.60648516	2.88260264
0.06932886	1364.73674	1311.12674	1327.65799	1191.40404	1221.73197	1203.96704
3.10E-12	918.894832	878.337151	765.388371	492.072599	506.846069	540.007561
6.33E-12	1175.30626	1142.32899	1070.06649	657.87967	729.344264	777.341845
8.98E-05	2919.95055	3031.4899	2887.97928	3658.4528	3392.09342	3500.44047
7.18E-09	1748.8306	1799.85512	1898.23702	2574.02107	2407.31802	2332.9864
0.0175568	1400.32037	1432.81815	1413.52183	1549.76126	1654.67972	1599.84446
2.74E-81	1112.51162	1262.05763	1270.41544	315.568297	330.132701	340.147111

0.01706486	7.3260408	9.81382292	16.6188066	26.743076	22.4907923	33.6303641
0.01514858	2100.48055	2145.30169	2299.85818	2444.31715	2487.64228	2454.05571
7.40E-16	2378.8701	2297.41595	2313.70719	1640.68771	1626.56623	1527.7794
2.47E-06	2263.74661	2409.29353	2442.0413	2936.38975	2968.78458	2897.01565
1.27E-15	1119.83766	1175.69599	1118.07638	732.760283	721.311838	731.220203
3.47E-30	75.3535625	69.6781427	62.7821583	294.173836	312.461364	254.6299
1.19E-05	124.542694	135.430756	132.950453	73.543459	70.6853472	60.5346554
7.84E-42	17790.7668	15777.6831	17185.6926	9513.84929	9633.28828	9368.45858
3.30E-10	118.26323	116.784493	123.717783	49.4746906	33.7361884	40.356437
2.59E-25	5152.29984	5269.04152	5445.42897	3416.42796	3383.25775	3068.05008
0.00015426	2379.91668	2310.17391	2525.13534	3031.32767	2831.4301	2836.481
0.00014208	870.752278	1014.74929	952.811579	680.611285	726.131294	770.615772
0.00012648	478.285806	494.616675	517.952806	641.833824	639.381095	662.03774
1.17E-202	6298.30193	6171.91323	6216.35694	16802.6747	17093.8054	16829.5951
0.0167107	32.443895	29.4414688	24.9282099	12.0343842	11.2453961	15.3738807
4.73E-16	9188.94832	8445.776	8651.01211	12145.368	11849.4346	12097.3224
5.23E-41	304.553982	294.414688	299.138519	787.583589	862.682533	748.515819
0.00564465	501.310506	502.467733	517.952806	425.214909	371.098073	424.703456
0.00010242	56.5151719	52.9946438	47.0866187	16.0458456	18.4745794	23.0608211
0.02699445	54.4220174	49.0691146	48.9331528	68.1948438	73.8983175	92.2432845
5.72E-16	3324.97595	3183.60415	2816.88772	1825.21494	1962.32163	2046.64787
2.02E-25	2513.87857	2383.77759	2180.75673	1093.79181	1249.04221	1323.11461
0.0001441	1956.05289	2005.9454	1785.59844	1397.32572	1530.17712	1627.70962
1.30E-12	1053.9033	1019.6562	986.972459	676.599823	681.149709	667.802945
1.52E-08	318.159486	319.930627	311.14099	465.329523	492.387703	564.02925
2.27E-58	2801.68732	2829.32515	2584.22443	5575.93135	5793.78874	6187.02613
4.02E-08	2305.6097	2109.97193	2405.11062	1740.97425	1655.48296	1461.47954
7.76E-43	1165.88706	1064.79979	970.353652	2273.16146	2373.58183	2479.03827
4.43E-35	2133.97103	2350.41059	2234.30622	3915.18633	3828.25414	3829.05717
4.08E-06	280.482705	258.103543	251.128633	430.563524	395.998593	368.973138
0.00581871	142.334507	153.095638	146.799458	106.972304	98.7988375	99.9302248
1.99E-05	1720.57301	1694.84722	1565.86089	2033.81093	2022.56482	2215.76056
1.87E-25	2489.80729	2327.8388	2157.67506	1144.60365	1183.17632	1345.21457
6.45E-09	243.852501	316.98648	243.742497	123.01815	132.535026	143.169264
0.00079501	1462.06843	1567.26752	1516.00447	2019.10224	1828.18012	1714.1877
0.0051665	2268.97949	2489.76687	2550.06355	2080.61131	2128.59284	2107.18253
1.47E-08	1406.59983	1674.23819	1816.06626	1133.90642	1090.80343	956.063209
2.83E-22	1472.5342	1430.85538	1456.91538	895.893047	873.927929	888.80248
0.00925824	26.1644314	27.4787042	25.8514769	50.8118444	43.3750994	56.6911852
3.12E-36	1524.86306	1393.56285	1467.07132	667.239747	693.198348	746.594084
7.16E-10	285.715591	289.507776	218.814287	140.401149	113.257204	116.264973
0.00038757	5931.99989	5544.80995	5650.39425	6625.59708	6558.47568	6560.80361
1.99E-16	208.268874	182.537106	217.89102	78.8920743	56.2269807	55.7303177

7.17E-32	533.754401	526.020908	667.522065	196.561609	187.958764	180.643099
3.21E-09	196.756524	237.494515	246.512298	104.297996	120.486387	97.0476222
3.39E-18	6431.21724	6227.85202	5630.08237	3753.39072	3874.84221	4145.1826
0.0003422	63.8412127	64.7712313	55.396022	25.4059222	22.4907923	32.6694966
0.00033812	3261.13473	3496.66511	3847.25373	2968.48144	2922.99975	2822.06798
8.45E-05	2221.88352	2223.81227	2116.12804	2603.43845	2571.98275	2661.6031
2.17E-11	4765.06625	4561.46489	4836.07272	3571.5378	3674.03157	3507.16654
0.00933244	2154.90257	2111.93469	1889.92762	1601.91025	1749.46234	1864.08304
8.54E-11	4443.76703	4301.39859	4391.98128	3183.7632	3219.39627	3478.34052
0.00312429	1160.65418	1239.48583	1313.80899	1539.06402	1469.13068	1419.20137
0.0251532	2064.89693	2136.46925	2345.09827	2488.44322	2540.65629	2385.83412
4.92E-20	4178.98299	3999.13284	4369.82287	6453.10424	6580.96647	6000.61783
6.59E-12	720.045153	759.589894	884.489818	1337.1538	1320.5308	1181.86708
2.82E-10	438.515871	424.938532	498.564198	266.093606	269.889507	253.669032
4.47E-06	3180.54828	3177.71586	3420.70436	2670.29614	2626.60324	2309.92558
2.08E-12	2827.85175	2753.75871	2680.2442	1888.06117	1929.38868	2079.31737
1.43E-12	10159.1254	10504.7161	11063.5089	14663.2286	14823.8418	13614.5323
0.00050717	20334.9961	22248.9179	24237.6062	28060.1725	27854.8462	25256.4035
3.42E-57	1008.90048	1005.91685	1133.77192	361.031526	349.410523	319.968893
1.67E-12	654.110786	645.749548	696.143343	434.574985	371.098073	379.542681
6.98E-07	10983.8283	10964.003	10101.4646	12740.4014	13432.6257	14301.5526
6.68E-16	4441.67388	5021.73319	5278.31763	3274.68966	2999.3078	2760.57246
0.00266972	594.455882	490.691146	495.794397	382.425987	416.079657	417.016515
0.00106936	4914.7268	4737.13232	4311.65705	3812.22549	3897.33301	4092.33488
6.25E-35	4903.21445	5397.60261	5656.85712	9499.1406	9867.83512	9256.03708
7.04E-10	3287.29916	3640.9283	3708.76367	2660.93606	2643.47134	2469.42959
6.13E-146	7146.02951	6943.27971	6213.58714	22429.4179	23301.2641	24812.4827
1.33E-59	343.27734	354.279007	379.462751	49.4746906	40.9653717	49.0042449
1.46E-51	596.549036	582.941081	632.437918	1387.96565	1522.94793	1437.45785
5.46E-14	418.630903	426.901297	408.084029	220.630377	204.023616	230.608211
1.57E-13	15632.7245	14641.2424	15796.1757	22148.6156	21297.1738	20007.1841
1.51E-43	3498.70777	3734.15962	3924.80816	1886.72401	1836.21254	1664.22259
1.27E-23	9650.48889	9865.83618	9682.30138	6856.92469	6908.68944	6961.48537
0.0135417	96.2851076	125.616933	103.405908	52.1489982	64.2594065	87.4389467
0.025906	1134.48975	1124.66411	1284.26444	1073.7345	1039.3959	892.645951
2.30E-24	238.619615	212.959957	253.898434	60.171921	65.8658917	59.5737879
6.69E-54	12750.4507	12493.978	12946.0503	7561.60474	7572.16782	7525.51462
0.00881941	28.2575859	12.7579698	22.1584088	37.4403064	62.6529214	38.4347019
1.56E-09	954.478458	1184.52843	1188.24467	1719.57979	1751.06883	1588.31405
0.00016496	11.5123498	14.7207344	30.4678121	1.3371538	2.40972774	0
0.00142772	3733.14108	3772.43353	4239.64222	3341.54735	3419.40367	2997.90674
0.00524659	222.920956	198.239223	248.358832	161.79561	171.09067	145.091
3.05E-62	740.976698	744.86916	721.071553	1684.81379	1767.93692	1721.87464

1.52E-20	5136.60118	5085.52304	5932.91396	10499.3316	9875.0643	8546.91683
2.02E-136	1593.93716	1702.69828	1699.73461	4971.53783	5064.44448	4757.25522
0.00857667	2.09315451	0.98138229	0.92326703	8.02292281	14.4583665	7.68694037
2.71E-25	729.464348	758.608512	801.395785	1557.78418	1546.24197	1333.68415
1.12E-74	7728.97304	7811.80304	8365.72259	16972.4932	16870.5039	15979.2273
2.21E-05	1905.81718	1885.23538	2117.05131	2520.53491	2626.60324	2347.39942
1.73E-09	3756.16578	3806.78191	3438.24643	2448.32861	2678.01077	2822.06798
6.94E-42	868.659123	822.398361	926.960102	1831.90071	1811.31202	1765.11368
9.87E-28	1911.05007	1871.49603	2203.83841	1006.87681	1077.1483	996.419646
2.61E-12	2953.44102	2749.83318	2866.74414	2080.61131	2122.97014	2072.5913
0.01199538	43.9562448	41.2180563	47.0866187	28.0802298	21.6875497	19.2173509
0.03237326	3.13973177	6.86967604	11.0792044	21.3944608	14.4583665	22.0999536
0.02942406	11.5123498	19.6276458	13.8490055	5.3486152	5.62269807	3.84347019
1.08E-16	1611.72898	1528.99361	1625.87325	1052.34004	1051.44454	1012.75439
5.40E-154	1950.82001	1939.21141	1937.9375	466.666676	466.68394	490.042449
3.28E-35	874.938587	810.621773	792.163115	334.28845	360.655919	352.63839
0.07476993	2411.314	2396.53556	2782.72684	2369.43654	2294.06081	2095.65212
1.15E-07	743.069852	856.746741	747.846297	1120.53489	1079.55803	1083.85859
0.07705275	53.3754401	42.1994385	48.0098858	57.4976134	76.3080453	73.0259335
1.66E-12	802.724756	948.015294	985.125925	546.895905	559.860079	521.751078
1.86E-50	875.985164	874.411622	954.658113	2401.52823	2432.21854	2041.84354
0.00062564	31.3973177	31.4042333	35.0841473	76.2177666	56.2269807	73.9868011
0.02100856	3965.48123	4084.5131	4331.04566	3801.52826	3755.96231	3425.4928
9.11E-22	250.131964	170.760519	241.895963	537.535828	606.448149	628.407375
1.80E-21	4002.11143	3934.36161	3994.97646	5756.44711	5592.9781	5616.27081
2.29E-19	1968.61182	1980.42946	1973.94492	2961.79567	2847.49495	2857.62008
1.29E-10	432.236407	437.696502	422.856301	270.105068	226.514408	249.825562
0.00507078	3104.14814	2914.70541	3289.60044	3658.4528	3614.59162	3459.12317
3.33E-44	391.419894	405.310887	387.772154	90.9264585	100.405323	95.1258871
1.21E-11	766.094552	740.94363	782.930445	1080.42027	1159.88229	1129.01937
6.28E-26	7448.49034	6964.87013	7229.18087	4811.07938	4851.58519	4813.94641
2.38E-09	3005.76988	3070.74519	2662.70213	2007.06786	2044.25237	2208.07362
0.00324299	3011.00277	3017.75055	3280.36777	3879.08318	3787.28877	3345.7408
8.47E-16	8206.21227	8510.54723	9475.48957	5827.31626	6097.41444	5635.48816
0.00012272	7623.26874	7782.36157	8286.32163	9787.96582	9297.53288	9024.468
7.00E-11	479.332384	442.603414	436.705307	243.361992	273.90572	265.199443
1.76E-06	1902.67745	1985.33638	2402.34082	3099.52251	3024.20832	2682.74219
0.00030701	2873.90115	3158.08822	3736.46169	4562.36877	4520.64925	3781.0138
0.0003629	2230.25613	2256.19789	2304.47452	1938.87301	1959.9119	1796.82231
3.87E-19	2538.99643	2349.42921	2891.67235	5196.17967	4817.849	4220.13026
2.70E-20	2838.31752	2604.5886	2436.5017	4059.59894	4299.75754	4496.86012
5.64E-08	895.870132	861.653652	845.712603	641.833824	563.07305	621.681303
6.47E-07	1727.89905	1594.74622	1460.60845	2024.45085	2180.00037	2437.72097

4.01E-23	2361.07829	2680.15504	2612.84571	4217.38309	4015.40967	4092.33488
7.49E-08	369.441772	386.664623	362.843944	187.201532	238.563047	235.412549
4.94E-05	4298.29279	4294.52891	4035.6002	4819.1023	5098.18067	5252.10201
5.73E-07	35.5836267	47.10635	35.0841473	84.2406895	98.7988375	131.638854
0.00039842	453.167952	450.454472	463.480051	572.301827	593.596268	588.050938
0.00999987	357.929422	354.279007	272.363775	389.111756	403.227776	550.577104
0.00704943	2835.17779	2889.18947	2644.23678	2357.40215	2325.38727	2565.51635
0.02003421	80.5864488	76.5478188	82.170766	110.983765	120.486387	110.499768
8.98E-39	778.653479	744.86916	695.220076	1521.68103	1495.63769	1590.23579
3.48E-21	3079.03029	3210.10148	2906.44462	1885.38686	1967.14108	2005.33057
1.81E-13	458.400839	440.640649	428.395904	242.024838	227.317651	248.864695
1.86E-21	2623.76918	2637.9556	2639.62045	3853.67725	3956.77296	3769.48338
0.00238576	6447.96248	6645.92088	7213.48533	8125.88365	8050.09715	7443.84088
2.51E-07	1299.84895	1334.67992	1341.507	1744.98571	1650.66351	1753.58327
0.07862828	36.630204	24.5345573	23.0816758	50.8118444	46.5880697	38.4347019
9.65E-11	1251.7064	1287.57357	1366.43521	1793.12325	1845.85145	1778.56583
0.00372404	1953.95974	1810.65033	1713.58361	1424.0688	1521.34145	1630.59223
8.63E-128	3737.32738	3977.54243	3993.12992	9672.9706	9634.09152	9636.54062
3.98E-19	920.987986	866.560564	871.56408	508.118444	502.026614	515.985872
1.71E-27	843.541269	742.906395	666.598798	264.756453	318.084062	293.064602
0.00036892	93.1453759	63.789849	95.0965045	33.428845	49.8010401	29.7868939
4.48E-223	2855.06276	2591.83063	2650.69965	9927.02982	9882.29348	9771.06208
1.52E-11	375.721235	363.111448	337.915734	605.730672	587.170327	574.598793
2.92E-73	908.429059	881.281298	708.145815	3044.6992	3090.87745	3740.65736
4.65E-24	844.587846	672.24687	591.814169	1580.51579	1554.2744	1733.40505
1.19E-32	1754.06348	1728.21422	1492.92279	3096.8482	3125.41689	3217.94541
1.15E-05	176.871556	168.797754	182.806873	267.43076	273.102478	278.651588
7.91E-08	1453.69581	1455.38994	1054.37095	1862.65524	2204.90089	2546.299
5.12E-31	1214.02962	1169.80769	1203.94021	605.730672	640.184338	583.246601
0.00107244	59.6549036	52.0132615	53.549488	94.9379199	90.7664117	103.773695
0.04663029	68.0275217	39.2552917	67.3984935	78.8920743	93.1761395	90.3215494
0.00491172	20.9315451	18.6462635	15.6955396	65.5205362	36.9491588	30.7477615
8.88E-10	19.8849679	33.3669979	22.1584088	102.960843	89.1599266	84.5563441
0.0109669	34.5370495	68.6967604	44.3168176	873.161432	111.650719	96.0867546
0.00390457	48.1425538	71.6409073	48.9331528	1089.78035	187.958764	112.421503
0.07863677	3.13973177	0.98138229	2.7698011	4.0114614	9.63891098	11.5304106
0.02108988	11.5123498	19.6276458	13.8490055	44.1260754	35.3426736	21.139086
1.03E-05	15.6986589	25.5159396	12.0024714	133.71538	38.5556439	63.4172581
0.0032395	7.3260408	12.7579698	11.0792044	20.057307	30.5232181	40.356437
3.39E-07	90.0056441	98.1382292	79.4009649	175.167148	161.451759	196.016979
0.00307594	105.704303	96.1754646	84.0173001	141.738303	132.535026	166.230086
4.63E-21	664.576558	705.613868	726.611156	353.008603	357.442949	371.85574
0.0012868	901.103018	1046.15352	1004.51453	1260.93603	1215.30603	1154.96279

0.00641994	10471.0055	10822.6839	11141.0633	12430.1817	12674.3647	11537.1366
2.17E-08	854.007042	909.741385	739.536894	441.260754	510.862282	581.324866
0.01793081	836.215228	735.055337	784.776979	633.810902	626.529214	713.924587
0.01784277	1874.41987	1815.55724	1868.69248	2079.27416	2015.33564	2166.75632
0.02049764	711.672535	763.515423	749.692831	829.035357	876.337657	875.350335
2.68E-14	10803.817	9850.13406	9238.20994	6105.44425	6478.95466	7050.84606
0.00043207	1008.90048	969.605704	953.734846	770.200589	764.686938	828.267825
2.79E-23	1947.68028	1929.39759	1635.10592	800.955127	913.286815	993.537043
1.02E-11	1421.25191	1488.75694	1602.79157	1020.24835	1045.0186	919.550242
4.40E-08	4305.61883	3755.75003	3779.85524	5264.37451	5138.34279	5150.25005
1.91E-06	3510.22012	3208.13871	3364.38507	4221.39455	4069.22692	4150.9478
2.05E-10	3374.16508	3374.9737	3292.37024	2511.17484	2627.40648	2567.43808
4.31E-07	3080.07687	3282.72377	3037.54854	2392.16815	2455.51257	2563.59461
1.06E-09	639.458704	753.7016	752.462633	1077.74596	1094.0164	1036.77608
2.26E-08	1452.64923	1402.3953	1402.44262	1884.04971	1808.09905	1877.53519
0.00347786	1229.72828	1079.52052	1263.0293	1048.32858	967.907311	889.763348
0.00052026	6620.64773	6747.00326	6079.71342	5214.89982	5549.603	5672.96199
1.23E-26	1214.02962	1228.69063	1407.05896	669.914054	649.823249	620.720435
6.89E-05	1031.92518	1089.33434	1215.01942	823.686741	892.402508	845.563441
4.57E-47	2818.43255	3055.04307	2851.97187	1494.93795	1492.42472	1506.64031
3.39E-05	4063.85949	3987.35625	3765.08296	4510.21977	4890.94408	5015.72859
1.51E-28	6226.0881	6348.56205	6363.1564	9723.78244	9597.14237	9179.16767
0.00023827	333.858145	329.74445	279.749911	179.178609	190.368492	245.982092
7.22E-64	107.797457	115.80311	113.561845	513.46706	534.156317	580.363998
9.16E-05	0	0	2.7698011	10.6972304	25.7037626	22.0999536
1.51E-18	1148.09525	1038.30246	1107.92044	627.125133	669.10107	638.016051
6.12E-13	1284.15029	1576.09996	1609.25444	2548.61514	2474.79039	2255.15613
0.00031503	9388.84457	9708.81501	9924.19735	11516.9057	11094.3865	10867.412
4.41E-24	469.913188	495.598057	533.648346	181.852917	208.039829	146.051867
1.25E-10	3128.21942	3071.72657	2778.1105	2033.81093	1937.42111	2244.58659
2.81E-61	1877.5596	1954.91353	2087.50676	706.017207	788.784215	670.685547
3.41E-46	8783.92292	9063.06547	9284.37329	5351.28951	5352.80856	5139.68051
0.03208703	1207.75015	1303.27568	1511.38813	1694.17387	1609.69813	1442.26219
7.53E-20	1040.29779	992.177497	1118.07638	1821.20348	1683.59645	1716.10944
4.60E-05	1846.16228	1724.28869	1801.29398	1433.42887	1466.72095	1481.65776
4.06E-05	139.194775	153.095638	126.487584	72.2063052	66.6691343	86.4780792
1.77E-08	53.3754401	66.7339958	71.0915616	172.49284	140.567452	141.247529
4.54E-06	172.685247	174.686048	219.737554	328.939835	310.854879	289.221131
2.02E-15	237.573037	196.276458	220.660821	85.5778433	57.0302233	75.9085362
0.00773775	212.455183	241.420044	222.507355	176.504302	165.467972	161.425748
6.47E-28	610.154541	566.257582	524.415675	195.224455	215.269012	239.256019
8.85E-30	4522.26033	4610.53401	4620.9515	2759.88544	2985.65268	2791.32022
0.0038384	1618.00844	1562.36061	1411.67529	1133.90642	1255.46816	1369.23625

3.35E-30	5058.10788	5162.07086	5602.38436	9329.32207	9082.26387	8460.43875
2.97E-08	3933.03733	4074.69928	3777.08544	2869.53206	3075.61585	3144.91948
5.37E-10	370.488349	391.571534	390.541955	240.687684	217.67874	196.977847
2.66E-19	1319.73392	1211.02575	1234.40802	724.73736	753.441542	772.537507
6.60E-05	115.123498	97.1568469	104.329175	53.486152	51.4075252	57.6520528
0.00183849	1552.07407	1391.60009	1347.0466	1715.56833	1692.43212	1720.91378
1.86E-05	2062.80377	2132.54372	2595.30363	3320.15289	3341.48914	2742.31598
0.01017252	2573.53347	2386.72173	2341.4052	1980.32478	2139.03499	2209.99536
1.27E-51	478.285806	457.324148	329.606331	1587.20156	1755.08504	2119.67381
3.21E-07	1337.52573	1199.24916	1191.93774	858.45274	925.335454	937.806725
1.04E-25	1930.93504	1955.89491	1900.08356	1175.35819	1187.99578	1182.82795
8.95E-45	1096.81297	1122.70134	1022.97987	447.946523	444.193148	459.294687
3.08E-38	3051.81928	3101.16804	3382.85041	5879.46526	5907.04595	5510.57538
3.28E-06	601.781923	586.866611	496.717664	298.185298	339.771612	417.016515
1.63E-05	196.756524	170.760519	182.806873	128.366765	85.9469562	80.7128739
1.00E-40	29.3041632	21.5904104	32.3143462	280.802298	260.250596	212.351728
9.05E-05	2191.53278	2233.6261	2034.88054	1664.75648	1721.34885	1841.98309
1.40E-14	2851.92303	2877.41288	2932.2961	2132.76031	2028.99076	2070.66956
8.53E-09	2052.338	1902.90026	1668.34353	985.482351	1138.19474	1354.82324
5.21E-26	200.942833	204.127517	223.430622	615.090748	601.628694	481.394641
0.00157997	695.973876	812.584538	826.323995	957.402121	1051.44454	929.158917
2.16E-27	468.866611	502.467733	504.1038	195.224455	199.20416	162.386615
3.41E-07	2131.87787	2222.83089	2220.45722	2808.02298	2747.08963	2684.66392
1.23E-20	2218.74378	2059.92143	2305.39778	3416.42796	3500.53117	3322.67998
0.00996484	899.009864	841.044624	979.586323	1159.31235	1075.54182	1020.44133
2.40E-05	15496.6694	15765.9065	16017.7598	18220.0577	18927.6082	17984.5579
0.00127825	52.3288628	93.2313177	60.9356242	33.428845	33.7361884	28.8260264
0.03639066	854.007042	935.257324	874.333881	778.223512	811.275007	709.120249
0.00025105	3921.52498	4111.01042	4134.38978	3322.8272	3547.11924	3481.22312
3.16E-26	2191.53278	2383.77759	2454.96704	1387.96565	1377.56103	1357.70584
3.48E-49	34993.3572	35233.587	31627.4355	65515.1876	68397.7123	74893.86
1.28E-09	495.031043	552.51823	507.796869	822.349588	763.883695	761.967964
0.00027137	2652.02677	2387.70312	2542.67741	3131.6142	3096.50015	2894.13305
1.51E-05	15.6986589	14.7207344	15.6955396	49.4746906	54.6204955	43.2390396
8.12E-23	1275.77768	1307.20121	1367.35848	759.503359	785.571245	724.49413
0.00421845	535.847556	586.866611	497.640931	421.203447	436.160722	429.507793
2.25E-63	6016.77265	6240.60999	6083.40649	3079.4652	3122.20391	3269.83226
1.95E-26	6433.3104	6917.76378	6772.16369	4421.96762	4452.37363	4379.63428
4.01E-12	3045.53982	2871.52459	3198.197	4345.74985	4169.63224	4077.92187
7.63E-32	536.894133	640.842637	654.596327	1395.98857	1345.43132	1280.83644
0.06855302	2121.4121	2196.33357	2510.36306	2053.86824	2122.97014	1856.3961
9.47E-10	1254.84613	1391.60009	1260.2595	857.115586	934.171122	921.471977
1.36E-11	4086.88419	3800.89362	3992.20665	2997.89882	2919.78678	3000.78935

3.67E-08	4389.34502	4133.58221	3674.60279	5315.18636	5429.91985	5843.03555
1.01E-05	21.9781224	16.683499	16.6188066	44.1260754	67.4723769	59.5737879
0.02650436	1681.84965	1678.16372	1652.64799	1929.51293	1832.99957	1843.90482
7.88E-05	5030.89687	4688.06321	4936.70883	5706.97242	5813.86981	5700.82715
0.07697918	5427.54965	5560.51207	5902.44615	6289.97148	6197.01652	5893.96153
8.66E-43	8146.55737	8325.06598	8849.51452	15865.3298	15305.7874	14425.5045
4.78E-16	1812.67181	1777.28333	1949.93998	2742.50245	2823.39767	2641.42489
4.37E-05	6515.99	6232.75894	6973.43591	8149.95242	8025.19663	7644.6622
2.33E-09	1225.54197	1177.65875	1157.77686	1548.4241	1642.63108	1750.70067
1.14E-19	542.127019	523.076762	546.574084	968.099352	921.319241	914.745904
7.99E-06	7101.02669	7050.25038	6387.16134	5168.09944	5445.18146	5784.42263
0.04320508	8591.3527	8700.9354	8885.52193	8186.05557	8100.70144	7904.09644
9.84E-10	5356.3824	4974.62684	4639.41684	3413.75365	3502.13766	3838.66585
5.95E-08	3164.84962	3169.8648	3570.27362	4738.87307	4481.29036	4143.26086
6.43E-07	200.942833	245.345573	318.527127	117.669534	140.567452	122.991046
0.00119433	847.727578	885.206827	931.576437	748.806128	734.966962	639.937786
1.45E-09	1368.92305	1239.48583	1324.88819	953.39066	938.187335	909.941567
0.00240958	3204.61956	3074.67072	2655.31599	2178.22354	2381.61425	2655.8379
0.00166501	178.964711	163.890843	168.957867	117.669534	122.092872	99.9302248
1.79E-14	6225.04152	6285.75358	5956.9189	4246.80047	4402.57259	4649.63806
0.01108874	6220.85522	6466.32792	6598.58949	7058.83491	7182.59516	7107.53724
0.03819814	13321.8819	12576.4141	12405.0159	13609.5514	13838.2632	14422.6219
1.68E-21	1024.59913	1086.3902	1251.02683	2143.45754	2227.39168	1929.42203
0.08380596	618.527159	576.071405	594.58397	500.095522	532.549832	534.242356
5.25E-21	3240.20319	3050.13616	3225.89502	2160.84054	2045.85886	2048.56961
8.35E-55	857.146773	867.541946	793.086382	283.476606	256.234384	277.690721
0.02384282	744.11643	701.688339	701.682946	620.439364	567.089263	639.937786
7.70E-75	3032.98089	2988.30908	3108.6401	1306.39926	1375.1513	1276.99297
1.59E-06	91.0522214	101.082376	108.94551	49.4746906	36.1459162	42.278172
0.00637824	722.138307	744.86916	756.155701	631.136594	619.30003	611.11176
5.97E-13	419.67748	427.882679	434.858773	744.794667	751.031814	655.311667
0.00246179	79.5398715	98.1382292	91.4034363	49.4746906	42.5718568	60.5346554
0.00116684	141.28793	136.412139	158.80193	101.623689	89.1599266	87.4389467
0.00168295	1256.93929	1382.76765	1434.75697	1621.96756	1646.64729	1601.7662
0.03471712	274.203241	289.507776	281.596445	248.710607	216.072254	219.077801
4.55E-06	2216.65063	2274.84415	2524.21207	3142.31143	3172.00495	2808.61584
0.00393046	681.321794	752.720218	740.460161	891.881585	857.863077	870.545997
2.81E-17	2767.15027	2734.13107	2646.08332	1751.67148	1769.54341	1921.73509
5.07E-10	675.042331	730.148425	815.244791	494.746906	440.980177	394.916562
0.02327455	5502.90322	6002.1341	6017.85453	5332.56936	5325.49832	5100.28494
5.55E-05	2984.83834	3149.25577	3132.64505	2672.97045	2534.23034	2513.6295
0.02276086	1329.15312	1273.83421	1181.7818	1025.59697	1093.21315	1148.23672
2.48E-09	4756.69363	4967.75716	5545.1418	3833.61995	3629.85323	3235.24103

9.47E-46	4362.13401	4288.64062	4602.48616	2429.60846	2445.07042	2321.45599
3.26E-06	1244.38036	1426.92985	1563.09109	2093.98285	2044.25237	1778.56583
0.01833873	3811.63437	3883.32973	4062.37495	3594.26942	3517.39926	3485.06659
0.00725787	11084.2997	10716.6946	12000.6249	13217.7653	13084.8217	12204.9396
0.00266176	4049.20741	4086.47586	3599.81816	4438.01347	4501.37143	4850.45937
0.0829877	764.001398	845.951536	666.598798	663.228285	630.545427	674.529018
2.01E-06	1286.24345	1219.85819	1170.7026	871.824278	903.647904	979.12403
0.0181583	1889.07195	1943.13694	2083.8137	2290.54446	2282.01217	2144.65636
2.10E-10	369.441772	319.930627	380.386018	573.638981	572.711961	620.720435
0.01077432	3769.77128	3626.20757	3624.74637	3246.60943	3355.94751	3266.94966
6.16E-14	752.489048	748.794689	807.858655	1186.05542	1163.09526	1172.25841
8.59E-08	1204.61042	1215.93266	1197.47734	1650.04779	1587.20734	1524.8968
0.0004086	643.645013	724.260131	726.611156	557.593135	542.991985	531.359753
0.00134473	2136.06418	2286.62074	2337.71213	1952.24455	1951.07623	1818.92227
7.40E-21	2688.65697	2784.18156	2764.2615	4158.54832	4421.04717	3985.67858
7.83E-80	4270.03521	4337.70973	4367.05307	8506.97248	8969.00667	8611.29495
1.84E-82	1714.29355	1690.92169	1513.23467	466.666676	496.403915	518.868475
4.16E-35	556.779101	598.643198	559.499822	1188.72973	1183.97957	1143.43238
0.00040321	5544.76631	5446.67172	4680.04059	6098.75849	6341.60018	6838.49433
1.63E-53	429.096675	412.180563	545.650817	1557.78418	1562.30682	1415.3579
0.05849563	837.261806	771.366481	677.678003	561.604596	667.494585	694.707236
4.10E-11	1841.97597	1867.5705	1915.7791	1424.0688	1273.94273	1252.97128
7.00E-25	5798.038	6001.15271	6586.58702	3820.24841	3620.21432	3378.41029
4.27E-08	3038.21378	2848.95279	2619.30857	1785.10032	1961.51838	2202.30842
4.84E-17	976.456581	988.251968	989.74226	1514.99526	1490.81823	1484.54036
0.00028244	1307.17499	1125.64549	1191.93774	1417.38303	1534.19333	1592.15752
7.18E-05	915.7551	868.523328	967.583851	1196.75265	1143.81744	1140.54978
4.07E-16	5503.94979	5614.48809	5835.04765	7625.78813	7846.07354	7616.79704
6.07E-07	2254.32741	2540.79875	2405.11062	1842.59794	1873.96494	1881.37866
1.85E-05	1034.01833	1119.75719	1085.76203	1569.81856	1410.49397	1300.05379
4.13E-07	2847.73672	2787.12571	2832.58326	2271.82431	2312.53539	2129.28248
0.00030683	2969.13968	3087.42869	3282.21431	3867.04879	3751.14286	3549.44472
5.71E-07	962.851076	989.23335	1143.92785	770.200589	723.721566	695.668104
0.08428504	20.9315451	26.4973219	13.8490055	9.36007661	12.0486387	7.68694037
2.99E-16	1549.98092	1661.48022	1427.37083	878.510047	861.076047	993.537043
0.02914185	7705.94834	7300.50287	7315.04471	6681.75754	6670.92964	6991.27227
1.76E-05	5734.19679	5264.13461	5342.94632	4421.96762	4431.48932	4649.63806
1.59E-33	6112.01118	6273.97699	6136.95597	3661.12711	3735.88125	3967.4221
3.46E-35	104.657726	88.3244063	106.175709	457.3066	400.818048	351.677522
5.39E-13	303.507405	288.526394	264.054372	132.378226	105.224778	138.364927
0.08821862	3020.42196	3022.65746	2908.29116	2805.34867	2666.76537	2784.59415
9.66E-28	6437.49671	6272.01423	6621.67117	9541.92952	9671.04068	9479.91921
0.00282395	2332.82071	2313.11806	1899.16029	2475.07169	2682.02698	2958.51118

8.40E-10	1945.58712	2070.71664	2236.15276	1469.53203	1540.61927	1373.07972
2.44E-07	65.9343672	70.659525	81.247499	24.0687684	19.277822	25.9434238
2.49E-10	28.2575859	42.1994385	39.7004825	113.658073	118.879902	109.5389
0.07232942	128.729003	128.56108	121.871248	89.5893047	102.011808	97.0476222
9.14E-14	1668.24415	1470.11067	1432.91044	918.624661	967.104068	1003.14572
6.89E-06	1047.62383	1157.04972	1085.76203	1373.25695	1494.83444	1387.49274
0.00770697	220.827801	252.215249	216.044486	161.79561	138.960967	192.173509
0.00049273	488.751579	540.741643	549.343885	656.542516	698.821046	666.842077
0.02748366	18.8383906	13.7393521	11.0792044	6.685769	2.40972774	4.80433773
3.32E-07	4603.89335	4618.38507	4946.86477	5950.33441	6106.25011	5676.80546
0.00094989	319.206063	350.353478	349.918206	470.678138	422.505598	453.529482
0.00227862	207.222297	243.382808	214.197952	291.499529	347.000795	281.534191
3.86E-06	4088.97734	4136.52636	3986.66705	4762.94184	4972.07158	4944.62439
6.02E-15	29.3041632	40.236674	41.5470165	117.669534	157.435546	166.230086
0.00119675	65.9343672	67.7153781	74.7846297	45.4632292	25.7037626	32.6694966
5.20E-06	175.824979	167.816372	185.576674	94.9379199	100.405323	101.85196
0.00013236	162.219475	237.494515	238.202895	346.322834	342.984582	312.281953
6.61E-21	9454.77894	9110.17182	8309.4033	5583.95427	5558.43866	6002.53956
2.24E-16	406.071976	400.403975	431.165705	889.207278	850.633894	689.902898
2.66E-07	7935.14876	8206.31872	8543.91313	6680.42039	6814.71006	6662.65557
7.36E-41	1796.97315	1888.17953	2014.56867	926.647584	945.416519	901.293759
2.79E-64	1580.33166	1465.20376	1679.42273	533.524367	469.89691	417.016515
0.00640881	32.443895	32.3856156	37.8539484	16.0458456	16.0648516	12.4912781
0.00192755	1389.8546	1312.10812	1484.61339	1724.9284	1735.80722	1574.86191
2.38E-13	2131.87787	1999.07573	2045.95975	2840.11467	2835.44631	2819.18538
1.58E-19	82.6796033	72.6222896	75.7078968	250.047761	242.57926	215.23433
2.08E-162	1524.86306	1526.04946	1505.84853	246.036299	234.546834	278.651588
1.70E-20	9.41919531	17.6648813	6.46286924	106.972304	113.257204	109.5389
0.00092908	13.6055043	20.6090281	26.774744	5.3486152	4.81945549	0.96086755
2.25E-05	45.002822	48.0877323	31.3910791	2.6743076	7.22918323	15.3738807
0.0001292	377.81439	322.874774	307.447922	215.281762	225.711165	243.099489
3.87E-15	242.805924	267.917366	237.279628	489.398291	465.880697	471.785965
1.19E-10	3220.31822	3296.46312	3494.56572	2559.31237	2467.56121	2481.92087
2.11E-05	901.103018	805.714862	979.586323	1303.72496	1348.64429	1089.6238
1.10E-05	967.037385	972.549851	1081.1457	731.423129	795.210156	748.515819
9.89E-14	7719.55385	7696.98131	7156.24278	4863.22837	4932.71269	5613.38821
1.03E-06	640.505281	697.76281	633.361185	486.723984	465.880697	422.78172
0.00474662	5311.37958	5373.06805	4750.20889	3990.06694	4245.13704	4753.41175
6.80E-22	120.356385	108.933434	108.94551	320.916912	297.199755	308.438482
9.45E-05	1050.76357	1210.04437	1015.59374	1310.41072	1457.88529	1502.79684
2.42E-07	6324.46636	5362.27284	6069.55748	8053.67734	8027.60636	7389.07143
1.42E-23	598.642191	535.834731	589.967635	1025.59697	1075.54182	1093.46727
2.06E-14	1117.74451	1105.03646	1221.48229	1703.53394	1725.36507	1703.61816

1.63E-33	1552.07407	1454.40856	1415.36836	2602.1013	2558.32762	2643.34662
0.006942	1529.04937	1641.85257	1894.54395	2099.33147	2098.87287	1901.55687
1.76E-36	373.628081	440.640649	454.247381	1099.14042	1064.29642	981.045765
5.49E-12	1187.86519	1120.73858	1136.54172	1631.32764	1673.1543	1583.50972
1.51E-18	917.848254	982.363674	862.33141	431.900678	444.99639	534.242356
0.0067207	9015.21649	8826.55233	7731.43814	6790.067	7068.53472	7859.89653
0.00022928	328.625259	304.22851	291.752383	403.820448	426.521811	427.586058
5.88E-06	3270.55393	3595.78472	3923.88489	2813.3716	2852.31441	2500.17736
0.000139	3344.86091	3403.43379	3155.72672	3758.73933	4064.40746	4002.01333
6.25E-39	4251.19682	4471.17772	4536.01094	7298.18545	7805.91141	7455.37129
0.00167653	10384.1395	9988.50897	10934.2515	9511.17499	8908.76347	8502.71692
3.58E-05	173.731825	158.983931	157.878663	101.623689	97.995595	82.634609
3.08E-23	1406.59983	1442.63197	1347.96987	667.239747	765.49018	807.128739
0.04028709	749.349316	810.621773	712.76215	641.833824	637.77461	680.294223
1.39E-34	21682.9876	22268.5456	21786.3322	32114.4228	32828.5243	33240.2519
0.01280531	5.23288628	6.86967604	11.0792044	26.743076	18.4745794	21.139086
6.09E-12	13.6055043	19.6276458	14.7722725	86.9149971	80.3242582	73.0259335
4.02E-07	373.628081	320.912009	254.821701	506.781291	506.042826	632.250846
4.86E-21	6897.9907	7253.39652	7352.89866	10435.1483	10107.2014	10128.5048
0.00640493	1343.8052	1316.03365	1183.62834	1063.03727	1084.37749	1105.95855
1.63E-14	3156.47701	3140.42333	3294.21678	2364.08792	2276.38948	2237.86052
2.30E-06	1422.29849	1491.70108	1450.45251	1125.8835	1173.53741	1105.95855
3.07E-23	2736.79953	2685.06195	2568.52889	1609.93318	1575.1587	1734.36592
8.81E-11	4097.34996	3856.83241	4123.31057	2983.19013	3123.00716	3027.69364
1.11E-07	1022.50598	1013.76791	961.120982	756.829051	728.541022	691.824633
4.77E-28	1387.76144	1417.11603	1570.47722	2674.3076	2604.11245	2486.72521
0.01974282	2298.28366	2400.46109	2378.33588	2586.05545	2677.20752	2615.48146
8.20E-12	1206.70358	1334.67992	1429.21737	874.498586	871.518201	770.615772
2.05E-06	6.27946354	7.85105833	13.8490055	45.4632292	40.9653717	43.2390396
0.00055809	1383.57513	1396.507	1484.61339	1211.46134	1174.34065	980.084897
1.46E-05	926.220872	854.783976	937.116039	703.342899	704.443744	647.624726
0.00742053	232.340151	260.066307	262.207838	213.944608	183.139309	156.62141
6.40E-07	4100.48969	4391.68576	3700.45427	2795.9886	3009.74995	3237.16276
0.00217281	1334.386	1544.69573	1734.81876	2069.91408	1959.9119	1787.21364
0.00243481	313.973177	283.619482	324.989996	215.281762	253.824656	176.799629
0.00019727	3087.40291	3021.67608	3209.27621	3933.90648	3611.37865	3592.68376
3.58E-05	2485.62099	2371.01962	2392.18488	2872.20636	2868.37926	2934.48949
2.82E-07	512.822856	525.039526	467.173119	735.43459	682.756194	742.750613
0.01211245	8.37261806	0	1.84653407	17.3829994	11.2453961	20.1782185
2.23E-05	198.849679	153.095638	145.876191	81.5663819	83.5372285	99.9302248
0.04760549	154.893434	146.225961	125.564317	89.5893047	102.81505	121.069311
2.60E-25	989.015508	1014.74929	1117.15311	532.187213	547.811441	494.846786
6.17E-19	940.872954	1041.24661	1010.9774	1666.09364	1603.27219	1564.29237

7.30E-47	1941.40081	1926.45344	2020.10827	3558.16626	3528.64466	3640.72713
4.06E-08	1769.76214	1739.9908	1458.76191	933.333353	1005.65971	1231.83219
3.85E-15	1211.93646	1129.57102	1070.06649	692.645669	704.443744	721.611527
0.02291383	2554.69508	2537.85461	2334.94233	2676.98191	2758.33503	2960.43291
1.47E-54	1840.92939	1764.52536	1912.08603	3678.51011	3728.65206	3560.01426
2.64E-07	39.7699358	33.3669979	34.1608802	94.9379199	85.1437137	100.891092
4.38E-14	4430.16153	4529.07928	4245.18182	3012.60751	3196.10223	3261.18445
8.97E-11	1598.12347	1653.62916	1745.89796	2582.04399	2510.93631	2195.58234
2.39E-07	3064.37821	3179.67863	2687.63034	2036.48524	2161.52579	2328.18207
0.00405716	18.8383906	31.4042333	26.774744	6.685769	8.8356684	9.60867546
4.53E-123	8149.6971	8029.66991	7978.87371	3242.59797	3376.02857	3421.64933
1.37E-05	370.488349	347.409331	372.076615	221.967531	249.0052	259.434238
2.53E-15	4515.98086	4276.86403	4910.85735	7040.11476	6794.629	6444.53863
0.00041275	398.745935	418.068856	502.257266	550.907366	632.151912	608.229157
3.44E-53	212.455183	220.811016	284.366246	973.447967	954.252187	840.759103
0.039317	574.570914	535.834731	607.509708	441.260754	512.468767	491.964184
1.05E-16	891.683823	839.08186	885.413085	501.432675	535.762802	469.86423
9.72E-06	585.036687	522.095379	596.430504	775.549204	788.784215	744.672349
0.01117017	2164.32177	2101.13949	2041.34341	2344.03061	2314.94512	2513.6295
0.00284217	1022.50598	965.680175	979.586323	1198.08981	1176.75038	1140.54978
0.02442507	412.351439	400.403975	439.475108	611.079287	462.667727	489.081581
2.90E-25	1152.28156	1219.85819	1140.23479	655.205362	650.626491	636.094316
3.24E-22	3889.08109	3430.91249	3608.12757	2306.59031	2280.40569	2297.4343
4.48E-10	393.513049	365.074213	324.066729	560.267443	601.628694	611.11176
0.03069952	1538.46857	1623.20631	1403.36589	1211.46134	1314.10486	1412.47529
0.00953058	63.8412127	56.9201729	46.1633517	13.371538	36.1459162	29.7868939
0.03805108	382.000699	383.720476	424.702836	346.322834	322.100275	323.812363
1.57E-22	712.719112	669.302723	734.920559	1274.30757	1253.86167	1193.39749
1.16E-26	1659.87153	1566.28614	1447.68271	2664.94753	2692.46913	2903.74173
0.01433108	73.260408	69.6781427	52.6262209	93.6007661	97.995595	107.617165
9.47E-14	4577.72892	4877.46999	4876.69647	6320.72602	6512.69085	6417.63434
7.46E-06	1378.34225	1254.20657	1246.4105	1678.12802	1600.05922	1669.02693
1.03E-05	106.75088	81.4547302	96.9430385	48.1375368	42.5718568	32.6694966
3.53E-45	574.570914	558.406524	591.814169	1520.34387	1476.35987	1286.60164
5.14E-35	2672.95831	2663.47154	2695.93974	4241.45186	4327.06779	4377.71254
1.01E-13	3334.39514	3350.43914	3058.78368	2095.32001	2186.42631	2357.96896
0.00299424	92.0987986	98.1382292	70.1682946	45.4632292	47.3913123	53.8085826
3.82E-24	148.61397	125.616933	134.796987	350.334296	374.311043	416.055648
6.34E-07	953.431881	1004.93547	924.190301	1243.55303	1253.86167	1302.93639
5.00E-09	1734.17851	1784.15301	1843.76427	1330.46803	1334.18593	1152.08019
1.23E-05	1254.84613	1265.00177	1363.66541	1631.32764	1744.64289	1579.66625
8.13E-34	5076.94627	5128.70386	5465.74084	3123.59128	3034.65047	2819.18538
1.86E-96	16792.3321	16600.0815	16733.2917	8603.24755	8499.913	8532.50381

9.78E-11	4744.13471	4448.60593	4791.75591	3615.66388	3432.25555	3541.75778
1.37E-22	1633.7071	1604.56005	1521.54407	925.31043	902.041419	981.045765
7.70E-06	52.3288628	36.3111448	35.0841473	94.9379199	97.1923524	93.204152
3.35E-05	79.5398715	79.4919656	80.3242319	37.4403064	37.7524013	31.708629
8.27E-85	11698.6406	11271.1756	11304.4816	5523.78235	5752.82337	5459.6494
4.16E-20	2415.50031	2209.09154	2444.81111	3822.92272	3558.36464	3605.17503
2.46E-19	872.845432	875.393004	876.180415	1346.51388	1458.68853	1508.56205
6.19E-53	31960.3763	32753.634	35691.657	17817.5744	17301.042	16497.1349
8.05E-19	7983.29132	8277.95963	8580.84381	5924.92849	5594.58458	5352.9931
4.59E-07	514.91601	583.922464	572.425561	799.617973	822.520404	747.554951
1.43E-05	356.882845	432.789591	404.390961	529.512905	567.892505	613.994362
0.00021077	248.03881	214.922722	259.438036	363.705834	374.311043	312.281953
1.03E-05	1038.20464	1081.48329	1161.46993	1477.55495	1432.98477	1342.33196
0.02790398	322.345795	224.736545	278.826644	224.641839	191.974977	213.312595
4.43E-64	992.15524	968.624322	992.512061	2147.469	2286.02839	2317.61252
4.04E-34	5084.27231	5370.1239	5380.80027	3282.71258	3217.78978	3103.60218
0.00058787	19275.8599	20241.9912	22145.4831	26569.246	24836.2606	23019.5038
1.58E-08	636.318972	650.656459	624.128515	462.655215	403.227776	402.603502
9.92E-41	2091.06136	2171.79901	1744.05143	633.810902	626.529214	798.480931
0.08042944	515.962588	495.598057	481.945392	592.359134	556.647109	560.18578
1.08E-06	1816.85812	1816.53862	1768.05637	2225.02392	2210.52358	2332.9864
9.29E-08	609.107964	656.544753	523.492408	359.694372	368.688345	404.525237
6.20E-14	4593.42758	4390.70437	3947.88984	2725.11945	2688.45292	3045.95012
1.58E-15	845.634424	824.361125	958.351181	480.038215	524.517406	443.920806
0.0001725	5669.309	5371.10528	6283.75543	7621.77666	7424.37118	6577.13836
1.01E-24	11.5123498	9.81382292	12.0024714	116.332381	131.731783	115.304106
0.00168632	1209.84331	1264.02039	1233.48476	1537.72687	1463.50798	1404.78835
1.77E-06	1200.42411	1274.8156	1278.72484	962.750737	988.791618	905.137229
9.85E-09	299.321095	376.8508	359.150876	554.918827	550.221168	559.224912
2.54E-08	1234.96116	1190.41672	1107.92044	1557.78418	1600.86247	1595.04013
0.00194643	586.083264	708.558015	709.992349	553.581674	524.517406	464.099025
7.61E-07	462.587148	448.491707	480.098858	643.170978	671.510798	638.976918
0.00385493	446.888489	427.882679	426.54937	533.524367	540.582257	535.203223
0.00595338	941.919531	1007.87961	949.118511	1111.17481	1126.1461	1162.64973
1.33E-25	2631.09522	2632.06731	2757.79863	1589.87587	1713.31643	1592.15752
0.01078998	1266.35848	1177.65875	986.972459	1255.58742	1396.83885	1628.67049
0.08150699	501.310506	501.486351	540.111215	465.329523	419.292628	456.412085
0.00105651	3866.05639	3678.22083	3704.14734	4119.77086	4424.26014	4593.90774
1.22E-18	1576.14535	1482.86864	1435.68024	2283.85869	2258.71814	2397.36453
1.94E-22	2168.50808	2290.54627	1971.17512	3481.9485	3645.91808	3969.34383
1.96E-13	500.263929	417.087474	421.009767	762.177666	742.999388	812.893944
0.0217568	1400.32037	1367.06553	1179.012	1429.41741	1534.99657	1676.71387
6.36E-54	1265.3119	1301.31292	1227.02189	2738.49098	2562.34384	2821.10712

0.00022439	9785.49735	9285.83925	8010.26479	6990.64007	6823.54573	7743.63156
2.48E-27	40809.187	40087.5039	41278.3458	29152.6272	28721.545	28750.1179
1.39E-31	6726.35203	6866.7319	6666.91125	9985.86458	10274.2759	10399.4695
2.18E-69	1894.30484	1901.91888	2103.2023	4749.5703	4627.48051	4386.36035
5.18E-05	553.639369	522.095379	493.024596	643.170978	714.885898	768.694037
0.04415287	286.762168	283.619482	366.537012	288.825221	228.924136	218.116933
4.44E-22	1612.77555	1567.26752	1639.72225	2468.38592	2477.20012	2434.83836
1.72E-41	208.268874	207.071664	216.044486	18.7201532	21.6875497	18.2564834
0.00032675	450.02822	483.82147	501.333999	348.997142	365.475375	363.207933
6.99E-12	3495.56804	3439.74493	3287.75391	4408.59608	4552.77895	4585.25993
6.37E-12	959.711345	1004.93547	1031.28928	1502.96087	1473.95014	1384.61013
0.00901719	57.5617491	58.8829375	48.0098858	29.4173836	35.3426736	21.139086
2.76E-17	184.197597	215.904104	249.282099	65.5205362	71.4885898	62.4563905
2.78E-06	361.069154	395.497064	363.767211	553.581674	502.829856	562.107515
0.00047801	4086.88419	4227.79491	3911.88242	3415.09081	3554.34842	3505.24481
4.98E-19	683.414949	600.605963	572.425561	255.396376	269.889507	319.008025
2.05E-28	2231.30271	2211.0543	2240.76909	1291.69057	1284.38489	1382.6884
9.07E-25	3834.65907	3744.95483	3657.98399	2368.09938	2431.41529	2502.09909
5.13E-06	11610.7281	12430.1881	12116.9566	9927.02982	10226.0813	10197.6873
3.87E-07	5393.0126	5151.27565	5508.21112	4329.70401	4425.86662	4089.45228
7.34E-09	1113.5582	1051.06043	1004.51453	1500.28656	1398.44533	1476.85342
5.57E-12	1621.14817	1510.34735	1447.68271	1073.7345	1041.00239	995.458778
1.84E-08	506.543392	549.574083	596.430504	374.403064	343.787825	331.499304
5.17E-17	2756.68449	2673.28536	2526.98187	1759.6944	1741.42992	1838.13962
8.08E-16	504.450238	534.853349	526.262209	234.001915	221.694953	293.064602
3.29E-17	59.6549036	65.7526136	78.4776979	193.887301	217.67874	216.195198
3.52E-10	620.620313	605.512874	589.044368	393.123217	397.605078	363.207933
0.06765332	462.587148	451.435854	447.784511	394.460371	371.098073	405.486105
0.02207464	1902.67745	1903.88165	1589.86583	1297.03919	1477.16311	1690.16601
2.74E-33	7673.50445	7827.50516	8311.24984	4988.92083	4747.9669	4867.75499
3.63E-12	2762.96396	2767.49806	2512.2096	1831.90071	1886.81682	1961.13066
1.05E-17	1969.6584	1876.40294	1530.77674	722.063052	877.944142	977.202295
6.70E-09	1742.55113	1598.67175	1889.92762	1290.35342	1212.8963	1186.67142
1.84E-12	3075.89056	3195.38074	3118.79604	2226.36108	2341.45213	2354.12549
1.27E-18	5641.05141	5568.36312	5438.04283	3925.88356	3955.16647	4046.21324
2.81E-24	1014.13336	1025.54449	1071.91303	585.673365	563.876292	557.303177
2.46E-12	2882.27377	2859.748	2679.32093	1916.1414	1963.92811	2099.49559
6.78E-05	735.743812	788.04998	781.083911	950.716352	975.939737	996.419646
0.01735236	31.3973177	38.2739094	39.7004825	26.743076	8.8356684	15.3738807
1.91E-05	1094.71981	910.722767	1128.23232	770.200589	788.784215	732.18107
6.50E-09	3448.47206	3602.65439	3747.54089	2834.76606	2797.69391	2689.46826
6.01E-26	2304.56312	2386.72173	2228.76662	1325.11942	1418.5264	1407.67096
3.28E-06	29.3041632	35.3297625	30.4678121	2.6743076	7.22918323	2.88260264

0.00929421	461.54057	419.050239	482.868659	391.786064	369.491588	289.221131
1.31E-12	822.609724	790.994127	675.831469	406.494755	418.489385	471.785965
2.02E-23	793.305561	799.826568	919.573966	418.52914	412.063444	404.525237
2.79E-14	934.59349	946.052529	844.789336	1402.67434	1392.82264	1374.04059
0.00115908	141.28793	126.598316	127.410851	70.8691514	70.6853472	96.0867546
1.44E-18	317.112909	339.558273	291.752383	121.680996	126.912328	129.717119
0.00370134	3544.75717	3557.51081	3108.6401	3781.47095	3989.7059	4394.04729
1.96E-11	1273.68452	1201.21193	1047.90808	605.730672	730.950749	752.359289
0.00013398	1375.20252	1465.20376	1189.16794	894.555893	1028.95375	1085.78033
0.0288038	1090.5335	1166.86355	1208.55655	1355.87395	1371.13509	1244.32347
0.00116412	2297.23708	2278.76968	2414.34329	1966.95324	2056.30101	1951.52199
1.73E-21	5394.05918	5691.03591	5787.96103	3816.23695	3735.078	3468.73184
1.88E-08	1504.9781	1560.39784	1672.95987	2032.47378	2176.7874	2125.43901
0.08225006	13.6055043	17.6648813	21.2351418	10.6972304	3.21297033	9.60867546
2.37E-07	850.86731	892.076503	870.640813	1152.62658	1192.01199	1134.78457
4.47E-70	2608.07052	2370.03823	2448.50417	903.915969	1005.65971	983.928368
7.69E-08	801.678179	741.925013	677.678003	501.432675	485.961762	516.94674
2.43E-05	1727.89905	1739.00942	1393.20995	1121.87204	1141.40771	1284.67991
0.03389878	1053.9033	1110.92475	886.336352	739.446052	865.895503	925.315447
7.80E-15	1976.98444	1981.41085	2025.64787	1391.97711	1391.21615	1268.34516
5.90E-07	484.56527	469.100736	408.084029	271.442222	296.396513	306.516747
2.97E-11	1376.24909	1244.39275	1172.54913	855.778433	824.930131	832.111295
5.40E-16	1465.20816	1652.64778	1583.40296	964.08789	1028.95375	985.850103
0.01533645	10.4657726	14.7207344	10.1559374	2.6743076	2.40972774	2.88260264
4.70E-20	1473.58078	1489.73832	1607.40791	2505.82622	2441.05421	2306.08211
0.00432694	272.110087	244.364191	315.757326	212.607454	195.187947	200.821317
5.84E-09	851.913887	901.890326	874.333881	1215.4728	1178.35687	1221.26265
2.91E-11	904.24275	856.746741	828.170529	1353.19965	1220.12548	1254.89302
1.56E-12	1010.99363	1077.55776	1121.76945	1628.65333	1579.17492	1516.24899
5.37E-08	2762.96396	2741.98212	3107.71684	2237.05831	2138.23175	2079.31737
0.00273545	627.946354	689.911751	605.663174	462.655215	469.093668	563.068382
0.00038436	38.7233585	52.0132615	34.1608802	13.371538	13.6551239	16.3347483
3.94E-08	1075.88142	1018.67482	942.655641	1317.09649	1457.88529	1459.5578
4.99E-06	633.17924	486.765617	563.192891	383.763141	388.76941	323.812363
1.11E-07	283.622437	297.358834	280.673178	155.109841	172.697155	175.838761
0.03345532	3269.50735	3416.19176	3653.36765	3185.10035	3124.61364	2929.68515
5.72E-05	283.622437	333.669979	242.81923	410.506217	412.063444	473.7077
1.26E-11	30.3507405	28.4600865	24.9282099	109.646612	127.71557	86.4780792
1.71E-16	1611.72898	1593.76484	1589.86583	1020.24835	1055.46075	1091.54553
0.00022532	254.318273	264.973219	248.358832	172.49284	187.155522	156.62141
5.32E-65	7619.08243	8321.14045	8514.36859	3899.14048	3889.30058	3726.24435
9.05E-35	6839.38237	6302.43708	6560.73554	4070.29617	3935.88865	3901.12224
1.77E-12	1113.5582	1239.48583	1182.70507	792.932204	787.980973	767.73317

0.03141436	397.699358	396.478446	399.774626	326.265527	338.968369	328.616701
0.03933396	1642.07972	1411.22774	1425.5243	1283.66765	1281.17192	1351.94064
0.02398033	373.628081	304.22851	275.133576	394.460371	379.130499	434.312131
4.32E-06	27.2110087	17.6648813	19.3886077	60.171921	73.0950749	58.6129203
1.57E-09	131.868734	91.2685531	103.405908	26.743076	36.9491588	35.5520992
1.07E-10	1427.53138	1497.58938	1483.69012	1953.5817	2004.09024	2006.29144
4.72E-07	1113.5582	1049.09767	992.512061	684.622746	734.966962	814.815679
1.67E-09	183.15102	192.350929	184.653407	382.425987	330.132701	310.360218
2.81E-07	441.655602	415.124709	426.54937	584.336211	620.906516	645.702991
1.51E-07	4474.11777	4238.59012	4190.70907	5248.32867	5360.84099	5342.42356
0.00157881	897.963286	898.946179	904.801693	740.783206	746.212358	750.437554
0.00020991	1093.67323	1046.15352	959.274448	1259.59888	1259.48437	1380.76666
6.76E-06	190.477061	199.220605	190.193009	100.286535	113.257204	120.108443
7.46E-14	1324.96681	1517.21702	1467.99458	953.39066	898.025206	809.050474
2.10E-06	1704.87435	1678.16372	1654.49452	2012.41647	2144.65769	2167.71718
6.43E-41	1479.86024	1614.37387	1732.04896	730.085975	684.36268	634.172581
1.86E-13	768.187707	639.861254	749.692831	1208.78704	1130.96555	1165.53233
0.00640553	1150.18841	1082.46467	976.816522	845.081202	843.404711	952.219739
0.03860515	21.9781224	6.86967604	14.7722725	4.0114614	4.81945549	4.80433773
0.03144628	837.261806	810.621773	818.014592	736.771744	703.640501	710.081117
0.01252077	1929.88846	2026.55443	2126.28398	2360.07646	2499.69091	2175.40413
5.93E-36	1447.41635	1405.33944	1492.92279	2814.70875	2606.52218	2573.20329
6.79E-11	139.194775	156.039784	124.64105	290.162375	263.463567	336.303641
0.0006285	721.09173	605.512874	599.200305	488.061137	479.535821	499.651124
1.86E-12	399.792512	356.241772	456.093915	212.607454	202.417131	171.995291
0.00094084	159.079743	152.114255	117.254913	57.4976134	66.6691343	106.656298
1.04E-05	924.127718	839.08186	850.328938	589.684826	656.249189	681.25509
1.43E-28	1792.78684	1715.45625	2000.71966	993.505274	889.189538	860.937322
0.02002455	15.6986589	31.4042333	24.9282099	54.8233058	39.3588865	45.1607747
4.80E-09	31.3973177	33.3669979	35.0841473	82.9035357	116.470174	104.734563
0.00546591	0	0	0	9.36007661	1.60648516	6.72607283
0.00093142	239.666192	239.457279	294.522184	204.584532	160.648516	155.660543
0.00383186	4018.85667	4244.47841	4466.76591	5122.63621	5015.44668	4626.57724
8.28E-07	243.852501	245.345573	221.584088	135.052534	111.650719	148.93447
5.32E-05	38.7233585	48.0877323	45.2400847	12.0343842	17.6713368	8.64780792
4.89E-34	39597.2505	31645.6534	31031.9283	15937.5362	15942.7588	16424.109
5.84E-08	566.198296	629.066049	643.517123	391.786064	428.931539	397.799164
0.00764941	1940.35423	1840.0918	1994.25679	1761.03156	1635.4019	1603.68794
0.05648743	3066.47136	2846.00865	2746.71943	3230.56358	3052.32181	3320.75824
2.46E-06	2252.23426	2304.28562	2403.26409	2869.53206	2918.1803	2807.65497
8.71E-23	1526.95622	1497.58938	1505.84853	881.184355	935.777608	917.628507
1.88E-63	23.0246997	24.5345573	25.8514769	310.219682	327.722973	310.360218
0.07494854	2714.8214	2881.33841	2935.98917	2699.71352	2605.71893	2465.58612

5.07E-179	16619.6468	17182.0412	17094.2891	6118.81579	6355.25531	6369.59097
1.28E-05	704.346494	749.776071	706.299281	476.026753	502.026614	577.481395
1.02E-49	1144.95552	1141.34761	1141.15805	465.329523	489.977975	485.238111
1.15E-05	64.8877899	46.1249677	56.3192891	14.7086918	18.4745794	20.1782185
2.72E-39	670.856022	696.781427	657.366128	1366.57118	1584.79761	1523.93593
6.17E-18	5315.56589	5538.92166	5613.46357	7936.00781	7990.6572	7456.33216
0.00079486	318.159486	303.247128	310.217723	196.561609	195.99119	255.590767
6.20E-30	4403.9971	4209.14865	4489.84759	7312.89414	7208.29893	6729.9163
0.00162315	774.46717	711.502162	628.74485	481.375368	553.434139	580.363998
0.02195924	26.1644314	26.4973219	25.8514769	5.3486152	10.4421536	16.3347483
0.0077142	219.781224	250.252484	260.361304	184.527225	189.565249	169.112688
3.41E-42	4244.91735	4221.90662	4327.35259	2471.06022	2388.84344	2524.19904
0.00050852	6945.08668	7000.19989	7151.62644	6088.06126	6304.65102	6069.80029
1.93E-15	291.995055	341.521038	363.767211	691.308515	626.529214	607.268289
5.21E-08	620.620313	635.935725	552.113686	334.28845	343.787825	430.468661
0.07523104	15.6986589	11.7765875	11.0792044	6.685769	2.40972774	5.76520528
0.07658717	189.430484	207.071664	205.888549	139.063995	163.861487	176.799629
0.00065386	954.478458	879.318533	976.816522	1283.66765	1172.73417	1082.89772
2.33E-06	4728.43605	4186.57686	3849.10026	2663.61037	2939.86785	3421.64933
0.01233049	7041.37178	8095.42253	8723.9502	9584.71844	9397.13496	8846.7075
3.39E-06	3225.55111	3206.17595	3495.48899	4345.74985	4364.01695	3901.12224
0.0117546	1129.25686	938.201471	1073.75956	958.739275	848.224166	702.394176
0.00767395	616.434004	612.38255	616.742379	783.572127	710.869685	721.611527
4.14E-23	279.436128	232.587603	276.056843	85.5778433	60.2431936	72.065066
1.72E-37	1358.45728	1268.9273	1506.7718	580.32475	576.728174	483.316376
1.98E-75	7966.54608	8410.44624	9032.32139	3560.84057	3705.35803	3401.47111
8.24E-07	304.553982	288.526394	340.685535	181.852917	205.630101	161.425748
1.97E-08	933.546913	1004.93547	1070.98976	1496.2751	1474.75338	1317.34941
3.64E-11	552.592792	574.108641	554.883487	808.97805	884.370082	858.054719
2.39E-05	4292.01333	4161.06092	3796.47404	4772.30192	5167.25953	5299.18452
8.27E-54	1051.81014	1088.35296	1052.52442	2143.45754	2241.0468	2228.25184
1.86E-92	3472.54334	3413.24761	3533.34294	7629.79959	7581.80673	7278.57166
0.00540702	3455.7981	3251.31953	2716.25161	3583.57219	3720.61964	4246.07369
1.47E-05	7038.23205	6624.33047	6191.42873	7966.76235	7981.82153	8446.02573
0.00097926	4243.87078	4203.26036	4236.87242	4647.94661	4946.36782	4960.95914
5.95E-18	2801.68732	2916.66817	2891.67235	1905.44417	2012.92591	1978.42628
2.08E-34	6476.22007	6488.89971	6785.08943	10818.9114	10558.6237	10160.2134
9.16E-13	18009.5014	18847.4469	19075.6202	14429.2267	14623.0312	14291.9439
1.68E-30	5131.36829	5251.37664	5197.9934	3322.8272	3368.79939	3426.45367
6.04E-12	1298.80238	1460.29685	1467.99458	2064.56547	2139.03499	1969.77847
0.01747633	1265.3119	1308.1826	1509.5416	1651.38494	1700.46455	1476.85342
2.52E-10	2491.90045	2633.04869	2773.49417	2008.40501	1855.49036	1792.01797
1.14E-14	128.729003	158.983931	140.336589	366.380141	352.623493	289.221131

1.12E-06	2174.78754	2199.27772	2171.52406	2812.03444	2705.32101	2625.09014
9.66E-28	3918.38525	3905.90152	4006.97893	2603.43845	2537.44332	2479.99914
8.99E-08	11.5123498	10.7952052	10.1559374	37.4403064	53.0140104	73.9868011
7.53E-19	2362.12487	2482.8972	2622.07838	4064.94755	3841.10603	3662.82709
6.81E-13	1555.2138	1544.69573	1310.11592	826.361049	840.19174	977.202295
3.14E-14	1267.40506	1213.9699	1139.31152	1775.74025	1767.13368	1906.36121
0.01033802	45.002822	39.2552917	48.9331528	17.3829994	30.5232181	12.4912781
2.48E-14	1178.44599	1196.30501	1239.94763	755.491897	811.275007	790.793991
2.38E-05	3183.68802	3438.76355	3149.26385	3935.24364	3857.17088	4177.85209
2.41E-07	4133.98016	4359.30014	4339.35506	3234.57504	3570.41328	3232.35843
1.28E-07	953.431881	907.77862	804.165586	624.450825	608.054634	621.681303
4.51E-28	9458.96525	9290.74616	9263.13815	13311.3661	13653.5174	13850.9057
2.59E-31	945.059263	903.853091	745.999763	264.756453	280.331661	344.951449
0.05797294	355.836267	316.98648	321.296928	276.790837	279.528418	271.925516
0.00189246	7357.43812	6772.5192	5989.23325	5201.52829	5366.46369	5943.92664
5.94E-06	3390.91031	3114.90739	2371.87301	1656.73356	1767.93692	2274.37348
0.00198802	1518.5836	1608.48558	1828.99199	2009.74216	2037.82643	1949.60025
0.08396756	1678.70992	1510.34735	1692.34847	1881.3754	1728.57804	1814.11793
0.003919	887.497514	849.877065	726.611156	615.090748	629.742184	709.120249
1.68E-15	1251.7064	1312.10812	1198.40061	799.617973	815.29122	789.833123
0.04515539	2411.314	2179.65007	2415.26656	2231.70969	2007.30321	1982.26975
2.43E-18	5279.98226	5091.41133	5228.46121	3762.7508	3748.73313	3712.7922
0.0004615	153.846857	145.244579	148.645992	234.001915	236.956562	198.899582
2.70E-12	749.349316	651.637842	578.88843	279.465144	319.690547	386.268754
0	1545.79461	1610.44834	1598.17524	8017.57419	8221.18782	8009.79187
0.00103629	2534.81012	2507.43176	1993.33353	2730.46806	2991.27537	3324.60171
3.12E-08	2226.06983	2330.78294	2034.88054	2814.70875	2951.11324	3089.18916
0.01695934	579.8038	618.270844	612.126043	744.794667	681.149709	715.846322
6.40E-18	1499.74521	1486.79417	1414.4451	956.064968	934.974365	903.215494
0.0010995	3537.43113	3495.68372	3874.02847	3138.29997	3167.1855	2942.17643
0.04273174	2810.05993	2932.37029	3332.99399	2840.11467	2652.307	2454.05571
3.96E-05	10205.1748	10087.6286	10838.2317	12527.794	12614.9247	11833.0838
2.84E-12	379.907544	381.757712	374.846416	656.542516	596.809238	620.720435
2.79E-08	661.436826	749.776071	805.088853	514.804213	477.929336	438.155601
1.07E-05	3976.99358	3639.94692	3505.64493	2810.69729	2913.36084	3096.8761
0.00765841	767.141129	951.940823	1039.59868	1255.58742	1166.30823	1061.75864
2.47E-19	929.360604	870.486093	881.720017	492.072599	515.681737	517.907608
1.04E-07	322.345795	317.967863	299.138519	196.561609	187.958764	181.603966
2.36E-14	2310.84258	2070.71664	2019.185	3088.82528	3134.25255	3360.15381
0.00105234	1308.22157	1160.97525	1046.98482	879.847201	883.56684	987.771838
0.00143804	2318.16862	2419.10735	2671.01153	3090.16243	3023.40508	2781.71155
2.28E-13	585.036687	714.446308	654.596327	357.020065	377.524013	360.32533
4.88E-05	0	0	0.92326703	25.4059222	11.2453961	20.1782185

1.17E-06	475.146075	421.994385	424.702836	291.499529	264.266809	308.438482
1.88E-05	1228.6817	1130.5524	989.74226	1416.04588	1492.42472	1791.05711
0.01442684	441.655602	419.050239	375.769683	489.398291	505.239584	514.064137
1.04E-109	3746.74658	3740.04791	3572.12015	1397.32572	1424.95234	1357.70584
6.94E-160	1353.22439	1384.73041	1274.10851	4748.23315	4882.91165	5288.61498
8.45E-09	1440.09031	1302.2943	1251.9501	1763.70586	1877.17791	2047.60874
0.0018103	1180.53915	1225.74648	1366.43521	1076.40881	1043.41211	883.037275
1.79E-13	143.381084	168.797754	181.883606	52.1489982	59.439951	50.92598
5.17E-05	2428.05924	2408.31214	2610.0759	3280.03827	2987.25916	2934.48949
3.63E-12	16266.9503	16124.1111	14960.619	10800.1912	11199.6113	12191.4874
0.0399672	3129.266	3100.18666	3452.09544	3872.39741	3737.48773	3344.77993
0.00089932	750.395893	813.56592	786.623513	905.253123	996.020801	997.380513
4.12E-20	271.06351	247.308338	324.989996	664.565439	615.283818	599.581349
2.25E-24	731.557503	657.526136	736.767093	1271.63326	1284.38489	1399.98402
9.28E-11	1913.14323	1896.03059	1875.15535	1389.3028	1400.05182	1419.20137
0.07549073	132.915312	146.225961	145.876191	128.366765	108.437749	89.3606818
1.47E-07	11874.4656	11583.2552	11618.3924	9410.88845	9388.29929	9960.35299
2.04E-07	10374.7203	11457.6383	13135.3201	17464.5658	17181.3588	14814.6558
1.77E-11	3600.22576	3716.49474	3529.64987	2639.5416	2709.33723	2792.28109
0.02596184	6175.85239	6172.89462	6716.76767	7189.87599	7181.79192	6793.33355
4.87E-13	1044.4841	1070.68808	1120.84618	746.131821	666.691343	667.802945
8.30E-18	714.812266	677.153781	637.054253	1080.42027	1169.5212	1178.98448
4.62E-41	1592.89059	1589.83931	1510.46487	2798.66291	3053.9283	2983.49373
0.00033551	219.781224	242.401426	276.98011	339.637065	351.820251	355.520992
0.01250636	868.659123	853.802594	794.009649	1006.87681	938.990578	1006.02832
3.85E-16	442.70218	400.403975	453.324114	738.108898	770.309636	753.320156
0.00065499	1510.21098	1444.59473	1371.97481	1222.15857	1153.45635	1203.96704
1.95E-08	961.804499	912.685531	971.27692	663.228285	677.936739	691.824633
0.06056441	376.767812	346.427949	335.145933	423.877755	411.260202	411.25131
8.20E-16	1420.20534	1343.51236	1481.84359	2099.33147	2175.18091	2068.74783
7.50E-20	939.826377	900.908944	826.323995	1460.17195	1578.37167	1730.52245
2.50E-20	587.129841	599.62458	595.507237	1068.38589	983.16892	1102.11508
7.51E-27	72.2138307	83.4174948	62.7821583	262.082145	269.086265	275.768986
7.87E-08	562.011987	638.879872	582.581498	829.035357	845.814438	856.132984
0.00021269	1251.7064	1231.63478	1294.42038	1067.04873	976.742979	1027.16741
6.38E-17	1179.49257	1180.6029	1262.10604	1928.17578	1828.98336	1797.78318
0.00026034	255.364851	303.247128	299.138519	196.561609	193.581462	199.86045
9.92E-05	1059.13618	991.196115	918.650699	667.239747	630.545427	835.954765
0.04573023	371.534926	349.372096	326.83653	280.802298	304.428938	278.651588
8.07E-07	1620.10159	1666.38713	1455.06885	1145.94081	1175.1439	1229.91046
1.08E-08	28.2575859	26.4973219	33.2376132	97.6122275	84.3404711	89.3606818
3.71E-09	2463.64286	2467.19508	2616.53877	1873.35247	1970.35405	1885.22213
1.13E-117	4518.07402	4593.85051	4254.41449	10945.941	11402.8317	11711.0537

1.97E-20	961.804499	1004.93547	1081.1457	1659.40787	1778.37908	1655.57478
0.02373313	623.760045	620.233608	554.883487	653.868209	713.279412	774.459242
1.42E-88	6997.41554	6772.5192	6501.64645	2872.20636	2886.85384	3050.75446
3.51E-31	2160.13546	2098.19534	2225.99682	3860.36302	3737.48773	3499.4796
2.93E-05	3374.16508	3295.48174	3501.02859	2919.00675	2787.25176	2705.80301
0.0362971	159.079743	147.207344	162.494998	184.527225	202.417131	214.273463
2.62E-07	295.134786	293.433305	328.683064	180.515763	176.713368	194.095244
5.06E-12	1465.20816	1450.48303	1522.46734	1059.02581	1042.60887	1011.79353
0.00734003	111.983766	74.5850542	76.6311638	44.1260754	51.4075252	56.6911852
2.93E-36	1556.26038	1422.02294	1405.21243	714.04013	718.098868	721.611527
0.0242622	483.518693	486.765617	503.180533	401.14614	392.785622	436.233866
1.60E-29	2208.27801	2090.34428	2057.03895	1206.11273	1249.84546	1163.6106
0.00311681	5417.08388	5421.15578	4431.68176	3757.40218	4033.081	4559.31651
1.86E-11	2643.65415	2553.55672	2586.07096	1868.00386	1973.56702	1929.42203
0.00017892	5446.38805	5542.84718	5606.07743	6410.31532	6428.35038	6273.50421
3.53E-10	2835.17779	2733.14968	2390.33835	1595.22448	1740.62667	1929.42203
3.31E-08	1559.40011	1558.43508	1383.05402	2011.07932	2075.57883	2492.49042
4.43E-07	68.0275217	91.2685531	58.1658231	169.818533	147.796635	156.62141
0.00078234	1058.08961	925.443501	795.856183	636.485209	671.510798	761.007097
0.00323993	352.696536	285.582247	234.509827	171.155687	201.613888	219.077801
5.31E-12	984.829199	1001.99132	1071.91303	1619.29325	1637.81162	1412.47529
1.51E-12	3559.40925	3500.59064	3438.24643	2561.98668	2629.81621	2659.68137
9.32E-08	1374.15594	1378.84212	1318.42532	974.785121	1007.2662	1059.8369
4.36E-05	1631.61394	1552.54679	1620.33364	1345.17672	1297.23677	1206.84964
1.14E-08	334.904722	363.111448	351.76474	201.910224	216.072254	158.543145
1.66E-05	131.868734	138.374903	169.881134	92.2636123	65.0626491	69.1824633
0.03670103	666.669713	679.116546	763.541837	621.776517	605.644907	579.403131
0.05843464	998.434703	1036.3397	1109.76697	1203.43842	1154.25959	1178.98448
0.03173674	1927.79531	1935.28588	2011.79887	1781.08886	1768.74016	1734.36592
0.00623879	83.7261806	75.5664365	103.405908	143.075457	140.567452	119.147576
4.77E-05	287.808746	314.042333	280.673178	217.95607	189.565249	160.46488
3.76E-21	2160.13546	2174.74316	2052.42262	1223.49573	1345.43132	1357.70584
2.81E-05	253.271696	199.220605	214.197952	149.761226	128.518813	119.147576
3.43E-05	983.782622	996.103026	1143.92785	1385.29134	1331.7762	1324.07548
0.0543707	235.479883	220.811016	163.418265	141.738303	154.222576	173.917026
7.92E-07	145.474239	136.412139	125.564317	238.013377	228.924136	235.412549
7.29E-13	4879.14317	4924.57634	5281.08743	3726.64764	3750.33961	3539.83604
1.83E-10	251.178542	282.6381	324.989996	147.086918	142.97718	122.991046
0.00134614	2258.51372	2303.30424	2093.04637	1755.68294	1871.55522	1952.48285
0.00459777	16269.0435	15711.9305	14482.3667	17495.3203	17207.0626	18314.1354
9.26E-19	2059.66404	1974.54117	1716.35342	1055.01435	1084.37749	1163.6106
8.32E-16	26068.1463	24634.6583	23830.4454	17140.9746	17557.2763	18530.3306
0.00183295	11332.3385	11599.9387	10251.9571	8932.18739	9400.34793	9937.29217

1.92E-57	287.808746	283.619482	351.76474	1077.74596	1010.47917	988.732705
1.02E-09	5624.30618	5497.7036	5628.23584	4240.1147	4544.74653	4247.99542
2.05E-20	1366.8299	1541.75158	1618.48711	867.812817	866.698746	815.776547
3.65E-08	667.71629	740.94363	749.692831	430.563524	495.600673	501.572859
1.05E-14	1059.13618	1321.92195	1277.80157	647.18244	728.541022	697.589839
4.60E-05	1698.59489	1791.02268	1904.69989	2195.60654	2298.88027	2174.44326
7.02E-25	505.496815	484.802852	594.58397	1133.90642	1130.96555	1033.89348
0.00133413	784.932943	601.587345	692.450275	961.413583	900.434934	841.719971
4.90E-13	589.222996	550.555466	523.492408	278.127991	256.234384	334.381906
0.00031945	612.247695	738.980866	636.130986	829.035357	871.518201	872.467732
3.81E-12	1683.94281	1740.97219	1603.71484	2401.52823	2293.25757	2309.92558
7.78E-08	6361.09657	7185.68114	7158.08931	5307.16344	5441.16525	5329.93228
6.44E-22	3233.92372	3231.69189	3415.16476	5063.80144	4917.45108	4767.82477
5.87E-22	1891.1651	1910.75132	2022.87807	3229.22643	3160.75956	2919.11561
0.0188483	2139.20391	2149.22722	1869.61574	1652.7221	1710.9067	1910.20468
0.00216855	1754.06348	1809.66895	1649.87819	1954.91886	2062.72695	2105.26079
2.70E-33	1748.8306	1872.47741	1676.65293	885.195816	930.958152	933.963255
3.58E-11	2146.52995	2184.55698	2134.59338	2935.05259	2784.03879	3046.91099
1.68E-254	6350.6308	6510.49012	6430.55489	19605.349	19526.0239	19902.4495
4.17E-49	293.041632	299.321599	313.910791	883.858662	806.455552	841.719971
5.85E-08	382.000699	400.403975	377.616217	220.630377	240.972774	254.6299
1.82E-05	3061.23848	3167.90204	3223.12521	2452.34007	2425.7926	2727.90296
5.38E-05	10755.6745	10742.2106	10293.5042	8666.09378	8789.88357	9403.04981
0.02865813	1609.63582	1599.65314	1400.59609	1211.46134	1350.25078	1402.86662
7.93E-06	936.686645	942.127	842.019535	1168.67242	1165.50499	1210.69311
2.79E-14	489.798156	498.542204	506.873602	788.920743	843.404711	796.559196
1.38E-10	3037.1672	3237.58018	3394.85288	4298.94947	4340.72291	4271.05624
5.32E-21	8109.92716	8042.42788	9156.03917	5402.10136	5415.46149	4978.25476
1.62E-36	5384.63999	5579.15833	5282.93397	8668.76809	8591.48265	9018.70279
0.02514354	11492.4649	11237.8086	9404.39801	8834.57516	9016.39798	9937.29217
0.08760064	4256.4297	4543.80001	4696.6594	5134.6706	4935.92566	4686.15102
1.04E-41	263.737469	247.308338	297.291985	894.555893	866.698746	736.985408
6.18E-52	1048.67041	1149.19866	1040.52195	323.59122	326.116488	396.838297
4.89E-13	2081.64216	1988.28052	1709.89055	1170.00958	1134.17853	1284.67991
2.33E-50	2709.58852	2590.84925	2856.5882	5335.24367	5168.06277	5091.63713
5.22E-08	21829.5084	22691.5214	20906.4587	26297.8038	27517.4844	29043.1825
9.00E-09	3445.33233	3495.68372	3255.43956	4241.45186	4341.52615	4493.01665
3.29E-14	5455.80724	5501.62913	5221.99834	7152.43568	7103.07415	7249.74564
1.36E-16	5005.77902	4788.1642	5246.92655	7992.16827	7610.72346	6981.66359
0.00010015	2116.17921	2060.90281	2201.99188	1799.80902	1752.67531	1746.8572
9.15E-05	972.270272	1024.56311	880.79675	1207.44988	1183.97957	1316.38854
0.00255496	1821.04443	2048.14484	1927.78157	2337.34484	2226.58844	2236.89965
0.00147763	2754.59134	2461.30679	2719.02141	3322.8272	3140.67849	2976.76766

0.02537315	11726.8982	11694.1514	12155.7338	12656.1607	13155.507	12948.6511
2.01E-25	2153.85599	2052.07037	1914.85583	3432.47381	3484.46632	3930.90913
0.05723371	3669.29986	3710.60645	3741.07802	4288.25224	4040.31019	3870.37448
0.00026604	4275.26809	4005.02113	3990.36012	4900.66868	4649.9713	4855.26371
2.39E-07	3549.99006	3468.20502	3339.45686	4310.98385	4282.88945	4208.59985
0.0001396	60.7014809	68.6967604	72.9380957	28.0802298	32.1297033	28.8260264
6.42E-10	1736.27167	1662.4616	1686.80887	1268.95896	1253.05843	1247.20608
0.07042253	1897.44457	1829.29659	1823.45239	1973.63901	2050.67831	2051.45221
3.12E-05	1058.08961	1025.54449	1063.60362	1390.63995	1271.53301	1307.74073
3.76E-11	6881.24546	7383.92036	6865.41366	9156.82923	9035.6758	9567.35816
4.28E-38	803.771333	722.297367	857.715074	1794.4604	1734.20073	1653.65305
0.00012288	581.896955	598.643198	573.348828	434.574985	432.947751	463.138157
9.76E-07	3383.58427	3436.80079	3557.34788	4363.13285	4359.19749	4098.10009
4.62E-06	7759.32378	7791.19401	8288.16816	9739.82829	9910.40697	9245.46753
0.00118204	1809.53208	1966.69011	2041.34341	1694.17387	1608.89489	1546.03588
1.99E-05	2758.77765	3061.91275	3170.49899	4004.77563	3906.97192	3535.0317
9.31E-34	8682.40492	8883.47251	9223.43767	15829.2267	15918.6615	14212.1919
1.20E-05	4618.54543	4597.77604	4827.76332	5838.01349	6091.79174	5378.93653
0.00966925	1917.32953	1883.27262	1551.08862	1466.85772	1419.32964	1566.2141
4.11E-16	13413.9807	11967.957	15267.1437	27594.843	24421.7875	21339.9073
9.36E-24	1536.37541	1465.20376	1389.51689	2406.87684	2401.69532	2651.99443
4.60E-05	2519.11146	2570.24022	2828.89019	3205.15766	3269.19731	3199.68893
0.0145863	2888.55323	2795.95815	2663.62539	3079.4652	3057.14127	3283.28441
0.00090666	16.7452361	11.7765875	13.8490055	37.4403064	39.3588865	36.5129668
6.87E-05	534.800978	567.238965	733.997292	895.893047	914.8933	814.815679
2.51E-23	2271.07265	2390.64726	2432.80863	3596.94372	3701.34182	3564.8186
1.52E-07	54.4220174	55.9387906	60.0123572	109.646612	137.354481	144.130132
1.58E-22	3929.8976	3960.85893	3864.7958	2652.91314	2651.50376	2705.80301
2.41E-18	1245.42694	1286.59218	1474.45745	2476.40884	2386.43371	2124.47815
0.00122243	1279.96399	1280.70389	1433.8337	1647.37348	1666.72836	1527.7794
6.33E-10	704.346494	682.060693	702.606213	465.329523	469.093668	401.642634
4.99E-09	118.26323	106.97067	113.561845	228.6533	221.694953	212.351728
3.00E-07	694.927299	689.911751	687.83394	509.455598	495.600673	465.059892
2.24E-17	935.640068	799.826568	1043.29175	493.409753	430.538024	466.02076
0.02870057	9896.43454	10622.4819	9950.04882	9180.898	9358.57932	9278.13703
5.67E-17	3481.96253	3623.26342	3291.44698	1958.93032	2188.83603	2340.67334
4.82E-33	2688.65697	2694.87577	2660.85559	1625.97902	1535.79982	1485.50123
3.79E-15	6059.68232	6292.62326	6239.43861	8133.90657	8400.31092	8204.84798
0.00069396	2135.0176	2005.9454	2214.91761	2650.23883	2458.72554	2471.35133
2.68E-10	1396.13406	1210.04437	1247.33376	1809.16909	1815.32823	1831.41354
4.73E-118	3450.56522	3365.15988	2826.12039	12091.8818	12402.0655	13891.2621
0.01267187	1487.18628	1438.70644	1008.2076	623.113671	742.196145	1005.06745
0.03402149	2088.9682	1966.69011	2088.43003	1884.04971	1830.58984	1804.50925

0.03530604	7173.24052	6922.67069	6581.97068	5974.40318	6187.37761	6522.36891
1.32E-05	3292.53205	3450.54014	2956.30104	2417.57407	2380.81101	2696.19434
0.00262902	3822.10014	3462.31673	3168.65246	3860.36302	4277.26675	4573.72952
6.31E-36	1380.4354	1323.88471	1549.24208	3203.82051	3280.4427	2781.71155
1.57E-24	1032.97175	1108.96199	1056.21749	1794.4604	1842.63848	1736.28766
9.73E-07	2335.96044	2231.66333	2513.13287	2901.62375	3088.46773	3064.20661
0.01205694	2948.20813	3001.06705	2618.38531	3015.28182	3403.33882	3726.24435
3.89E-05	3242.29634	3161.03236	2792.88278	2286.533	2404.10505	2557.82941
2.66E-07	1507.07125	1427.91123	1410.75203	1079.08312	1040.19914	1146.31498
0.00267903	4869.72398	4779.33176	3951.5829	3598.28088	3562.38085	3983.75685
0.00176413	4419.69576	4574.22286	4693.8896	5098.56744	5209.83138	5264.59329
2.31E-24	2578.76636	2688.0061	2678.39766	4191.97717	4081.27556	3925.14393
1.14E-05	3306.13755	3237.58018	3245.28362	2747.85106	2748.69611	2645.26836
0.00023823	6352.72395	6665.54853	6710.3048	7802.29243	7790.6498	7371.77582
0.0001583	1774.99503	1878.36571	1768.97964	2110.0287	2157.50957	2277.25609
2.76E-11	1116.69793	1205.13745	1137.46499	783.572127	773.522606	810.011342
1.61E-08	2851.92303	3043.26649	3318.22172	4569.05454	4616.23512	3886.70923
2.87E-21	1070.64853	1069.7067	1227.02189	1968.29039	2019.35185	1848.70916
3.19E-11	1026.69229	979.419527	1072.83629	1623.30471	1469.93392	1441.30132
9.46E-15	659.343672	681.079311	642.593856	1148.61511	1028.95375	1030.05001
1.84E-09	2684.47066	2907.83573	3191.73414	4097.03925	4249.15326	3896.3179
0.03548076	303.507405	301.284364	355.457808	247.373453	269.086265	258.47337
1.56E-14	2061.7572	2221.84951	2326.63293	3246.60943	3201.72493	3120.89779
1.08E-15	620.620313	620.233608	596.430504	323.59122	355.836464	344.951449
1.17E-84	1317.64077	1477.96173	1454.14558	3645.08126	3883.67788	3624.39239
0.07667007	700.160185	826.32389	805.088853	914.6132	967.907311	810.011342
0.00170988	1334.386	1565.30476	1532.62328	1841.26078	1804.08284	1721.87464
0.00030875	3636.85597	3566.34325	3424.39743	4130.46809	4096.53717	4121.16091
0.04058244	105.704303	120.710022	121.871248	172.49284	167.074457	130.677986
9.16E-09	483.518693	539.760261	570.579027	821.012434	811.275007	760.046229
4.25E-17	2991.1178	2967.70005	3068.93962	4173.25701	4195.336	4148.0652
3.07E-27	564.105141	567.238965	718.301752	1521.68103	1518.93172	1298.13206
3.58E-18	3769.77128	3646.8166	3673.67953	2370.77369	2552.70492	2602.99018
1.66E-09	1444.27661	1649.70363	1818.83606	2564.66099	2519.77198	2266.68654
0.00023078	487.705002	475.970412	518.876073	716.714437	722.918323	578.442263
3.63E-06	1249.61324	1273.83421	1282.41791	1593.88733	1597.64949	1582.54885
0.0018966	1289.38318	1292.48048	1383.97728	1593.88733	1598.45274	1504.71858
3.45E-16	869.705701	887.169592	1055.29422	1684.81379	1673.95754	1508.56205
0.05271346	3881.75505	3895.10632	3694.91467	3909.83771	4286.10242	4592.94687
9.08E-05	1835.69651	1980.42946	2092.1231	2679.65622	2592.86705	2266.68654
1.61E-19	827.84261	867.541946	920.497233	1632.66479	1449.85286	1440.34045
4.63E-14	2283.63157	2241.47715	2141.05625	3080.80236	3182.44711	3500.44047
1.00E-17	1326.01338	1190.41672	1263.95257	1961.60463	1908.50437	1975.54368

1.69E-18	5116.71621	5149.31289	5116.7459	3766.76226	3617.00135	3662.82709
4.00E-05	156.986589	142.300432	182.806873	104.297996	80.3242582	83.5954765
0.00192842	2769.24342	2976.53249	2935.0659	3412.4165	3274.01676	3316.91477
0.00792986	4925.19257	4541.83725	3830.63492	3348.23312	3508.5636	4029.87849
1.69E-34	3141.82493	3225.80359	3424.39743	1846.6094	1812.11526	1657.49652
5.04E-40	6733.67807	6935.42866	6319.76285	11559.6946	12241.4169	13013.99
2.33E-10	129.77558	107.952052	121.871248	45.4632292	38.5556439	39.3955694
4.60E-70	1787.55395	1904.86303	1732.97222	660.553978	689.182135	703.355044
1.44E-05	4512.84113	4570.29733	4100.2289	5285.76897	5333.53074	5910.29628
0.00044887	13239.2023	13594.1075	15673.3812	11976.8866	11949.0366	10402.3521
0.0018346	922.034563	1037.32108	1119.92291	1323.78226	1301.25298	1195.31923
0.00113486	281.529282	302.265746	277.903377	354.345757	375.917528	444.881674
2.33E-15	10688.6935	10294.7002	10329.5116	7936.00781	7740.84876	7947.33548
2.57E-08	4683.43322	4572.2601	3943.2735	2775.93129	2910.95112	3403.39285
6.53E-108	9168.01677	9794.19527	10461.5388	25857.8802	25702.1561	24825.9348
2.91E-07	6876.01258	6484.97418	6112.95103	4915.37737	4878.0922	5338.58009
6.59E-15	3162.75647	3223.84083	3377.31081	4718.81576	4809.01334	4382.51688
2.70E-19	309.786868	267.917366	342.53207	739.446052	730.950749	602.463952
0.00733099	1710.10724	1571.19305	1587.09603	1390.63995	1410.49397	1413.43616
6.99E-08	69005.0249	67111.828	67036.5728	55990.6411	54820.503	57719.3135
8.63E-07	2372.59064	2280.73245	1913.93256	1432.09172	1523.75118	1687.28341
6.81E-07	709.57938	671.265488	747.846297	934.670507	1004.85647	960.867546
3.10E-13	7.3260408	3.92552917	8.3094033	58.8347672	73.8983175	61.495523
0.08088394	55.4685946	63.789849	53.549488	42.7889216	40.1621291	33.6303641
0.00058103	4851.93216	4734.18818	5053.96374	4372.49293	4134.28957	4053.90018
5.43E-15	979.596312	913.666914	898.338824	541.547289	588.776812	567.87272
3.14E-32	2341.19332	2399.4797	2395.87795	1414.70872	1420.13288	1393.25794
0.00116412	760.861666	668.321341	631.514651	530.850059	537.369287	540.007561
0.00136921	1071.69511	1039.28385	1147.62092	933.333353	900.434934	809.050474
0.00220586	1199.37754	1529.97499	1390.44015	1708.88256	1705.284	1671.90953
5.07E-14	2583.99925	2477.99029	2525.13534	3399.04496	3434.66528	3549.44472
2.35E-33	1032.97175	1077.55776	1051.60115	1956.25601	1982.40269	1841.02222
0.00064324	119.309807	144.263197	116.331646	92.2636123	55.4237381	65.3389932
4.75E-10	1982.21732	2137.45063	2078.27409	1556.44702	1539.81603	1466.28388
7.41E-06	3419.1679	3132.57228	3331.14746	2564.66099	2752.71233	2666.40744
0.01169825	1120.88424	1120.73858	1150.39072	1260.93603	1330.97296	1299.09292
6.15E-06	3562.54898	3572.23154	3144.64752	2464.37445	2483.62606	2864.34616
0.09063419	2262.70003	2107.02778	2310.93739	2087.29708	2054.69452	1945.75678
0.00036066	934.59349	1021.61897	1071.91303	841.069741	803.242582	712.963719
9.59E-05	563.058564	564.294818	513.336471	385.100295	391.98238	431.429528
1.39E-29	250.131964	201.18337	212.351418	596.370595	551.024411	588.050938
0.00814759	1223.44881	1064.79979	1071.91303	905.253123	891.599266	1000.26312
3.70E-35	1067.5088	979.419527	1028.51948	434.574985	488.37149	436.233866

0.01350262	13.6055043	8.83244063	19.3886077	25.4059222	35.3426736	35.5520992
0.00250738	31.3973177	40.236674	48.9331528	20.057307	16.8680942	12.4912781
0.000105	24.0712769	17.6648813	18.4653407	4.0114614	1.60648516	1.92173509
1.80E-71	2215.60405	2250.3096	1940.7073	568.290365	556.647109	689.902898
0.02233651	545.266751	521.113997	605.663174	481.375368	475.519608	429.507793
1.69E-12	97.3316849	132.486609	121.871248	246.036299	290.773815	273.847251
0.00036608	5401.38522	5185.62403	6024.3174	7582.9992	6976.16182	6233.14777
5.45E-07	3012.04935	3184.58554	3633.97904	4757.59322	4582.49893	4100.98269
1.37E-66	3227.64426	3196.36212	3608.12757	8321.1081	8226.81052	7460.17563
5.18E-15	3903.73317	4193.44653	4110.38483	5751.0985	5541.57057	5522.10579
8.27E-14	1854.5349	1892.10506	1852.99694	1306.39926	1313.30162	1332.72329
1.04E-06	1037.15806	934.275942	906.648227	1298.37634	1232.97736	1413.43616
0.01041883	2499.22649	2797.92091	2913.83076	3131.6142	3149.51416	3163.17596
0.00038018	327.578681	339.558273	349.918206	276.790837	221.694953	220.038668
0.00261407	1750.92375	1839.11041	1913.93256	2131.42316	2209.72034	2080.27824
0.00187113	3397.18978	3481.94437	3501.02859	3055.39643	3089.27097	2913.3504
1.66E-06	1669.29072	1719.38178	1672.95987	2060.55401	2098.06962	2165.79545
0.00356508	2843.55041	2753.75871	2563.91255	2218.33816	2293.25757	2458.86005
6.76E-09	793.305561	747.813306	807.858655	1259.59888	1102.04882	1074.24992
0.00071428	691.787567	684.023457	585.351299	469.340984	493.190945	532.320621
1.29E-63	1901.63088	2040.29378	2107.81864	4213.37163	4339.91967	4238.38675
3.30E-14	5843.04083	5734.21673	5121.36224	3268.00389	3580.85543	3903.04397
1.67E-20	782.839788	856.746741	953.734846	1581.85295	1592.83004	1486.46209
8.95E-17	2081.64216	2107.02778	2278.62304	3421.77658	3247.50976	3096.8761
0.00080119	175.824979	197.257841	211.428151	262.082145	275.512205	292.103734
4.17E-68	1949.77343	2117.82299	2279.54631	5177.45952	5504.62141	5020.53293
8.21E-33	3194.15379	3128.64675	3152.95692	5017.00106	5268.46809	5558.61876
0.00192697	1058.08961	1122.70134	1205.78675	1335.81665	1441.01719	1319.27114
1.65E-10	541.080442	513.262939	493.947863	728.748821	910.073845	896.489421
0.00917673	1012.04021	802.770715	864.177944	726.074514	723.721566	740.828878
4.78E-32	8576.70062	8682.28914	8458.0493	5561.22266	5742.38122	5648.94031
4.07E-37	2354.79883	2293.49042	2449.42744	1337.1538	1253.86167	1260.65822
1.78E-14	1003.66759	943.108382	858.638341	494.746906	565.482777	562.107515
0.07348119	2060.71062	2199.27772	2129.97705	2309.26461	2331.81321	2337.79074
1.82E-06	477.239229	535.834731	636.130986	358.357219	371.901315	306.516747
0.02138798	795.398715	810.621773	757.078968	873.161432	919.712756	939.72846
0.01154032	1569.86589	1520.16117	1428.2941	1683.47664	1685.20294	1868.88738
1.65E-35	2055.47773	2172.78039	2207.53148	3630.37257	3634.67268	3623.43152
0.00313602	2721.10087	2873.48735	2371.87301	3021.96759	3169.59523	3414.92326
8.15E-15	12.5589271	13.7393521	11.0792044	90.9264585	79.5210156	78.7911388
0.01449353	4657.26879	4696.89565	4940.4019	5522.4452	5387.34799	5087.79366
0.00088729	3566.73529	3677.23945	2959.07084	2540.59222	2624.99676	2936.41122
0.00109386	783.886365	750.757453	753.3859	915.950354	918.909513	946.454533

2.09E-08	703.299917	711.502162	622.281981	442.597908	449.012603	464.099025
0.0516013	403.978821	403.348122	378.539484	310.219682	347.804038	335.342774
5.55E-21	1018.31967	1001.00994	1088.53183	1704.8711	1644.23756	1701.69642
0.00026195	9.41919531	6.86967604	9.23267034	29.4173836	29.7199755	32.6694966
2.29E-24	1361.59701	1404.35806	1445.83617	796.943665	834.569042	810.972209
0.00015851	25.1178542	37.2925271	42.4702836	6.685769	11.2453961	9.60867546
2.51E-06	77.446717	76.5478188	89.5569023	34.7659988	32.9329458	16.3347483
7.10E-08	1044.4841	1179.62151	1315.65552	1861.31809	1759.9045	1585.43145
0.00321696	514.91601	491.672528	453.324114	323.59122	390.375895	401.642634
0.07679598	1556.26038	1581.00687	1732.97222	1543.07549	1461.9015	1344.2537
0.00179057	2289.91104	2176.70592	2672.85806	3181.08889	2993.6851	2694.2726
1.95E-54	4484.58355	4256.255	4397.52088	2223.68677	2312.53539	2349.32115
1.29E-49	252.225119	298.340217	228.970224	10.6972304	10.4421536	12.4912781
2.02E-06	1809.53208	1687.97754	2090.27656	2697.03922	2682.83022	2341.63421
0.0726723	235.479883	217.866869	300.985053	344.985681	291.577057	303.634145
3.33E-06	1211.93646	1104.05508	1174.39567	895.893047	898.828449	880.154673
8.57E-06	483.518693	546.629937	577.041896	722.063052	845.011196	719.689792
9.57E-10	2235.48902	2346.48506	2186.29634	1742.3114	1659.49917	1648.84871
3.58E-31	3407.65555	3702.75539	3126.18218	6164.27902	6415.4985	7051.80692
5.62E-11	863.426237	854.783976	902.031892	530.850059	568.695748	607.268289
0.00026718	1085.30062	1236.54169	1382.13075	1771.72879	1661.9089	1468.20561
1.10E-47	3397.18978	3440.72632	3891.57055	7882.52166	7922.38158	7116.18505
2.63E-12	4207.24057	4522.2096	4496.31045	5866.09372	6026.72909	5768.08788
7.16E-16	3956.06203	3975.57966	4635.72378	6545.36786	6387.38501	6248.52165
3.20E-13	1403.4601	1522.12393	1359.97234	2072.58839	2050.67831	2258.03873
0.0126206	11842.0217	11471.3776	11801.1992	13007.8322	12592.434	12926.5511
1.82E-06	6710.65337	7183.71838	7520.93326	8969.6277	9236.48645	8535.38642
8.67E-19	326.532104	280.675335	348.071672	692.645669	653.036219	615.916097
0.00049376	1708.01408	1602.59728	1706.19748	1434.76603	1399.24858	1350.97977
1.12E-05	1241.24063	1255.18795	1317.50206	948.042045	1025.74078	1004.10659
0.05529531	466.773457	539.760261	462.556784	529.512905	584.760599	613.033495
0.00068938	8015.73521	8448.72015	9300.06883	10785.4826	10545.7719	9648.07103
1.45E-222	3632.66966	3397.54549	3632.13251	12087.8704	11761.0779	11951.2705
2.04E-08	1245.42694	1187.47257	933.422971	1627.31618	1768.74016	2173.48239
8.17E-11	3655.69436	4075.68066	4335.66199	5779.17873	5730.33258	5457.72766
0.01285899	2812.15309	2514.30143	2291.54878	2073.92555	2110.11826	2283.98216
0.01211705	2432.24555	2418.12597	2230.61315	1894.74694	2019.35185	2193.66061
0.00143346	4688.66611	4717.50468	4657.88219	5150.71644	5380.92205	5469.25807
0.0348283	1113.5582	1206.11884	1313.80899	1434.76603	1417.72316	1325.03635
0.0009462	59.6549036	68.6967604	64.6286924	36.1031526	27.3102478	31.708629
0.01026501	315.019754	344.465184	372.076615	268.767914	268.283022	272.886383
5.30E-18	924.127718	854.783976	782.007178	486.723984	417.686142	446.803409
8.77E-80	1110.41847	1123.68272	987.895726	2865.5206	3069.1899	3216.98455

2.62E-05	4288.8736	4259.19915	4180.55313	4976.88645	4913.43487	5163.70219
8.00E-44	2520.15803	2391.62865	2616.53877	4748.23315	4647.56158	4473.7993
0.02638808	2196.76566	2398.49832	2592.53383	2880.22929	2912.5576	2524.19904
0.07896103	27.2110087	31.4042333	17.5420736	9.36007661	10.4421536	18.2564834
4.86E-18	8104.69428	7747.03181	7735.13121	5455.58751	5596.99431	5786.34436
6.55E-16	30416.6748	30761.4279	31452.938	40756.4478	39876.1747	39906.7509
1.72E-06	3746.74658	3976.56105	3745.69436	4742.88453	4577.67947	4681.34669
1.03E-09	2027.22015	1834.2035	1866.84594	2536.58076	2543.86926	2602.02932
1.66E-10	3069.61109	3069.76381	3529.64987	2309.26461	2302.09324	2060.10002
2.51E-05	1186.81861	1306.21983	1345.20007	1755.68294	1664.31863	1541.23154
2.79E-05	803.771333	842.026006	924.190301	685.9599	628.938941	604.385687
0.02899416	91.0522214	120.710022	112.638578	139.063995	155.829061	148.93447
4.05E-09	817.376838	980.40091	1017.44027	1370.58265	1455.47556	1369.23625
5.34E-09	1074.83484	1252.2438	1360.89561	1870.67817	1853.88388	1729.56158
2.99E-18	1026.69229	1001.99132	978.663056	1512.32095	1592.83004	1660.37912
3.96E-37	1106.23216	1080.5019	1203.94021	2238.39546	2229.80141	2106.22166
9.59E-61	698.06703	733.092572	853.098739	2698.37637	2680.42049	2196.54321
0.00024711	162.219475	144.263197	194.809344	271.442222	262.660324	237.334284
0.04039696	97.3316849	89.3057886	100.636107	62.8462286	75.5048027	61.495523
4.75E-16	1406.59983	1403.37668	1438.45004	2104.68008	2079.59504	2008.21317
4.73E-30	910.522214	965.680175	989.74226	1859.98094	1835.4093	1677.67474
3.48E-14	830.982342	798.845186	834.633399	485.38683	502.829856	527.516283
0.02488085	1471.48762	1543.71435	1600.02177	1773.06594	1670.74457	1780.48756
2.62E-05	1563.58642	1543.71435	1448.60598	1921.49001	1801.67311	1979.38715
8.93E-15	351.649958	350.353478	373.923149	609.742133	637.77461	618.7987
7.14E-13	659.343672	668.321341	604.739907	957.402121	1016.90511	1047.34563
2.24E-19	492.937888	542.704407	531.801811	954.727814	969.513796	883.998143
0.02842607	25.1178542	14.7207344	29.5445451	16.0458456	6.42594065	4.80433773
5.17E-08	4518.07402	4885.32105	5398.34235	7141.73845	6956.08076	6248.52165
0.00030432	72.2138307	48.0877323	81.247499	29.4173836	30.5232181	25.9434238
1.87E-25	4335.96958	4136.52636	3922.03836	6457.1157	6456.46387	6956.68104
2.85E-11	2367.35776	2581.03543	2418.03636	3243.93512	3314.98213	3421.64933
1.15E-76	283.622437	258.103543	288.059315	1056.3515	997.627286	962.789282
9.99E-08	916.801677	912.685531	810.628456	1186.05542	1215.30603	1412.47529
1.25E-05	447.935066	469.100736	465.326585	320.916912	330.132701	330.538436
5.59E-05	1332.29285	1443.61335	1581.55643	2067.23978	2020.15509	1703.61816
0.00160562	37.6767812	29.4414688	29.5445451	54.8233058	69.078862	68.2215958
0.00256872	300.367673	360.167301	375.769683	446.609369	475.519608	439.116469
8.74E-85	2684.47066	2867.59906	2765.18477	1127.22065	1138.19474	1119.41069
0.00331137	24.0712769	16.683499	17.5420736	49.4746906	41.7686142	39.3955694
1.03E-10	1244.38036	1104.05508	1319.34859	1847.94655	1914.12707	1717.07031
3.19E-06	171.63867	194.313694	166.188066	296.848144	269.889507	293.064602
1.06E-12	620.620313	648.693695	562.269624	355.682911	361.459162	351.677522

0.00025884	26.1644314	28.4600865	26.774744	58.8347672	65.8658917	58.6129203
3.91E-12	1545.79461	1516.23564	1251.02683	732.760283	812.881493	933.963255
0.00018827	3124.03311	2984.38355	2832.58326	2476.40884	2506.11685	2512.66863
0.0599724	1229.72828	1159.01249	1147.62092	1327.79372	1293.22056	1317.34941
1.32E-11	2233.39587	2577.1099	2856.5882	1659.40787	1679.58024	1513.36639
6.21E-05	326.532104	395.497064	381.309285	534.86152	504.436341	501.572859
0.04711113	479.332384	516.207086	444.091443	336.962758	375.917528	458.33382
1.56E-11	1921.51584	1998.09435	2060.73202	1413.37157	1458.68853	1449.94913
0.00697186	2000.00914	1999.07573	2321.09332	2591.40407	2548.68871	2334.90814
1.24E-08	1667.19757	1841.07318	2090.27656	2845.46329	2827.41389	2509.78603
8.27E-28	326.532104	367.036977	407.160762	929.321892	916.499786	792.715726
0.0041652	2103.62029	2144.32031	2167.831	1913.46709	1871.55522	1817.00053
7.37E-40	1861.86094	1915.65823	2003.48946	3492.64573	3495.71172	3350.54513
1.51E-14	692.834144	695.800045	659.212662	1060.36296	1037.78942	1100.19334
0.00728529	781.793211	722.297367	673.061668	565.616058	554.237381	643.781256
1.21E-26	7567.80014	7443.78468	8382.3414	13808.7873	13161.9329	12357.7175
0.00185834	232.340151	225.717927	232.663293	171.155687	166.271214	156.62141
7.45E-76	2317.12205	2191.42666	1836.37813	6275.26279	6494.21627	7043.15912
5.01E-14	300.367673	330.725832	320.373661	607.067826	561.466565	539.046694
0.00176287	488.751579	533.871967	593.660703	703.342899	694.001591	659.155137
6.33E-65	7611.75639	7425.13842	8566.99481	21255.3968	19857.7631	18185.3792
1.66E-06	118.26323	96.1754646	105.252442	201.910224	187.155522	190.251774
1.06E-37	55.4685946	59.8643198	62.7821583	377.077372	372.704558	270.003781
9.21E-17	398.745935	425.919915	446.861244	751.480436	733.360477	748.515819
1.02E-07	5150.20668	5050.19327	5562.68388	7318.24275	7009.89801	6383.04311
0.00216855	1731.03878	1649.70363	1600.02177	1440.11464	1374.34806	1424.96657
0.00077874	6396.68019	7087.54291	7435.99269	8569.81871	8563.36916	7881.99648
1.03E-13	1088.44035	1124.66411	1055.29422	1547.08695	1632.18893	1597.92273
2.67E-08	2066.99008	1971.59702	1693.27174	1116.52342	1225.74818	1420.16223
7.45E-20	1404.50668	1319.95918	1219.63575	730.085975	739.786418	785.989653
0.00124918	1044.4841	1147.2359	1164.23973	1397.32572	1346.23457	1311.5842
5.95E-05	54.4220174	60.8457021	50.7796869	17.3829994	24.90052	17.2956158
4.75E-11	1800.11288	1951.96938	1970.25185	2638.20445	2612.94812	2560.71201
0.06952507	1641.03314	1693.86584	1907.46969	1603.24741	1571.94573	1503.75771
0.00850095	1878.60618	1909.76994	1932.3979	2238.39546	2381.61425	2034.1566
0.00055142	1335.43258	1349.40065	1923.16523	3205.15766	2955.9327	2253.2344
0.06010117	3556.26952	3515.31137	3189.8876	3595.60657	3786.48553	4006.81767
0.00028114	466.773457	419.050239	426.54937	561.604596	568.695748	585.168336
3.11E-18	3299.85809	3459.37258	3555.50135	4935.43468	4924.68027	4841.81157
1.03E-08	875.985164	902.871708	981.432857	1335.81665	1267.51679	1252.97128
8.66E-43	2881.22719	3095.27975	3521.34047	1405.34864	1397.64209	1264.50169
3.39E-06	2051.29142	2077.58631	2345.09827	2993.88736	2856.33062	2632.77708
7.21E-21	383.047276	362.130066	424.702836	783.572127	731.753992	785.989653

0.00217993	556.779101	613.363932	590.890902	728.748821	722.115081	714.885455
2.53E-06	5813.73666	5790.15552	6236.66881	4987.58368	4871.66626	4685.19016
8.73E-05	205.129142	249.271102	233.58656	342.311373	327.722973	333.421039
0.00432119	347.463649	408.255033	432.088972	528.175751	535.762802	470.825098
4.97E-93	2936.69578	2702.72683	2900.90502	6590.83108	6466.90602	6622.29913
8.09E-05	138.148198	156.039784	204.042014	355.682911	350.213766	213.312595
2.14E-18	168.498938	133.467992	152.339061	382.425987	375.917528	340.147111
4.40E-17	1794.88	1901.91888	1985.94739	3004.58459	2945.49055	2733.66817
2.73E-10	482.472115	517.188468	517.029539	754.154744	816.094463	745.633216
0.05484052	219.781224	189.406782	200.348946	244.699146	236.956562	273.847251
6.87E-10	163.266052	171.741901	201.272213	314.231143	334.952157	344.951449
1.44E-25	428.050098	493.635293	446.861244	984.145197	882.763597	938.767593
5.03E-05	671.902599	626.121902	743.229962	929.321892	947.826246	848.446044
5.67E-67	3824.1933	4073.71789	4117.77097	8267.62195	7908.72646	7910.82251
8.02E-05	232.340151	258.103543	280.673178	410.506217	368.688345	349.755787
0.01179643	522.242051	568.220347	650.903259	762.177666	742.196145	653.389932
4.91E-07	671.902599	632.991578	695.220076	873.161432	949.432731	922.432845
0.00040056	497.124197	491.672528	551.190419	673.925516	649.823249	659.155137
2.56E-27	789.119252	774.310628	761.695303	1365.23403	1359.08645	1371.15799
4.78E-07	546.313328	543.68579	587.197833	770.200589	796.013398	760.046229
0.03626528	325.485527	346.427949	324.989996	387.774602	382.343469	431.429528
5.94E-05	834.122074	765.478188	872.487347	1032.28273	1093.21315	1048.30649
0.02180457	3406.60897	3618.35651	4208.25114	3362.94181	3298.91728	2933.52862
7.93E-06	852.960464	812.584538	872.487347	637.822363	635.364882	649.546461
0.00017479	3016.23565	3039.34096	3478.87018	4080.9934	4067.62043	3682.04444
0.02791488	196.756524	185.481253	225.277156	159.121302	142.97718	162.386615
1.05E-53	615.387427	624.159138	658.289395	1509.64664	1489.21175	1432.65351
5.60E-21	333.858145	281.656718	311.14099	628.462286	616.08706	664.920342
6.57E-09	498.170774	559.387906	558.576555	359.694372	333.345671	336.303641
8.09E-06	301.41425	317.967863	332.376132	223.304685	204.826858	192.173509
0.02638808	322.345795	313.060951	348.071672	411.843371	396.801835	387.229621
0.00200079	906.335904	878.337151	906.648227	1112.51196	1028.95375	1090.58467
6.29E-12	236.52646	268.898748	260.361304	461.318061	472.306638	432.390396
1.25E-08	477.239229	545.648554	559.499822	798.280819	824.930131	752.359289
0.03678345	999.48128	971.568469	977.739789	1080.42027	1154.25959	1097.31074
0.00137962	625.8532	638.879872	660.135929	877.172893	763.080453	772.537507
6.40E-11	375.721235	354.279007	385.002353	621.776517	639.381095	564.02925
7.77E-21	923.081141	853.802594	957.427914	1527.02964	1524.55442	1497.9925
9.78E-17	1668.24415	1592.78346	1736.66529	2425.59699	2458.72554	2467.50786
6.85E-05	750.395893	673.228252	735.843826	984.145197	926.941939	890.724216
3.27E-13	362.115731	356.241772	440.398375	771.537743	699.624289	658.194269
0.0003313	523.288628	528.965055	582.581498	417.191986	416.8829	412.212177
0.00028858	1636.84683	1647.74087	1755.13063	2238.39546	2105.29881	1901.55687

1.90E-11	245.945655	234.550368	276.056843	457.3066	466.68394	427.586058
0.03049241	30.3507405	25.5159396	32.3143462	57.4976134	48.1945549	45.1607747
0.00025519	146.520816	142.300432	145.876191	223.304685	212.859284	216.195198
1.25E-12	856.100196	847.9143	849.405671	1190.06688	1300.44974	1340.41023
0.0001662	2.09315451	0	1.84653407	20.057307	18.4745794	13.4521457
4.70E-17	1363.69017	1543.71435	1496.61586	2258.45277	2434.62826	2232.09531
0.00031993	1486.1397	1573.15581	1605.56137	1230.1815	1334.18593	1261.61909
3.12E-06	936.686645	876.374387	817.091325	572.301827	657.052432	654.350799
1.88E-05	372.581503	414.143327	348.994939	544.221597	514.075252	540.968429
7.03E-34	1783.36765	1727.23283	1626.79651	3043.36205	3283.65567	3536.95344
0.00038018	913.661945	933.29456	974.046721	1199.42696	1178.35687	1100.19334
0.05315379	279.436128	289.507776	286.21278	235.339069	242.57926	226.764741
2.96E-08	256.411428	233.568985	225.277156	109.646612	139.764209	120.108443
1.93E-05	526.42836	529.946438	529.955277	699.331438	714.885898	684.137693
0.00013906	509.683124	475.970412	518.876073	639.159517	681.952952	642.820389
1.85E-11	582.943532	589.810757	670.291867	1025.59697	989.594861	908.019831
1.03E-07	255.364851	263.991837	270.517241	394.460371	406.440746	439.116469
5.61E-10	380.954122	361.148683	376.69295	558.930289	588.776812	603.424819
2.02E-35	457.354261	516.207086	567.809226	1280.99334	1179.16011	1179.94535
8.46E-18	3195.20037	3245.43124	2759.64516	1662.08217	1718.93912	1960.16979
3.60E-09	1066.46222	1023.58173	1245.48723	1797.13471	1768.74016	1518.17072
0.07841746	838.308383	857.728123	882.643284	811.652357	757.457754	722.572395
7.10E-05	36.630204	36.3111448	36.0074143	13.371538	8.8356684	8.64780792
2.93E-19	4924.14599	5017.80766	4858.23113	3559.50342	3473.22092	3382.25376
0.0023234	526.42836	524.058144	538.264681	641.833824	642.594065	671.646415
0.00059668	1665.10442	1537.82605	1490.15299	1846.6094	1837.01578	2024.54792
2.56E-05	388.280162	414.143327	393.311756	287.488067	230.530621	288.260264
3.60E-12	338.044454	305.209893	384.079086	584.336211	634.561639	596.698746
0.01677221	268.970355	308.15404	326.83653	374.403064	392.785622	365.129668
0.00017544	197.803102	226.699309	269.593974	339.637065	423.308841	316.125423
2.18E-05	208.268874	290.489158	310.217723	494.746906	430.538024	394.916562
0.0217568	245.945655	263.991837	276.98011	359.694372	358.246191	286.338529
0.02056698	512.822856	513.262939	484.715193	574.976134	598.415723	613.033495
0.00640881	255.364851	250.252484	282.519712	344.985681	345.39431	326.694966
0.00904534	455.261107	473.026265	529.03201	655.205362	589.580055	564.02925
0.00029871	30.3507405	19.6276458	24.9282099	72.2063052	49.8010401	59.5737879
0.00712501	372.581503	415.124709	372.999882	295.51099	321.297033	293.064602
6.01E-09	56.5151719	75.5664365	55.396022	286.150913	152.616091	148.93447
9.94E-34	268.970355	276.749806	281.596445	44.1260754	57.8334659	67.2607283
2.50E-20	635.272395	574.108641	703.52948	1254.25027	1280.36868	1126.13676
1.83E-22	609.107964	579.015552	657.366128	1163.32381	1138.19474	1077.13252
9.96E-05	335.951299	369.981124	332.376132	490.735445	443.389905	503.494594
8.95E-08	145.474239	136.412139	153.262328	283.476606	249.0052	246.942959

0.01565122	448.981643	492.653911	526.262209	611.079287	644.20055	543.851031
2.01E-49	456.307684	497.560822	561.346357	121.680996	98.7988375	111.460635
0.00018068	162.219475	138.374903	135.720254	92.2636123	86.7501988	75.9085362
0.05707779	261.644314	257.12216	252.0519	239.35053	199.20416	186.408304
8.64E-21	304.553982	277.731189	300.985053	619.10221	594.39951	574.598793
2.66E-15	13.6055043	28.4600865	20.3118747	102.960843	115.666932	111.460635
0.02984713	4.18630903	4.90691146	8.3094033	12.0343842	19.277822	18.2564834
8.76E-15	348.510227	350.353478	381.309285	680.611285	693.198348	594.777011
5.04E-05	993.201817	1116.81305	1311.96245	1589.87587	1647.45053	1442.26219
0.00498359	1779.18134	1624.18769	1603.71484	1268.95896	1350.25078	1529.70113
0.02228093	307.693714	286.563629	281.596445	242.024838	224.10468	233.490814
0.01005898	478.285806	514.244321	551.190419	627.125133	657.855674	595.737879
3.96E-06	838.308383	898.946179	1013.7472	1238.20442	1285.18813	1202.0453
0.02725416	489.798156	486.765617	440.398375	382.425987	383.146711	412.212177
1.66E-08	640.505281	676.172399	724.764622	1045.65427	1038.59266	913.785037
5.77E-08	585.036687	470.082118	433.935506	752.81759	773.522606	859.976454
9.53E-06	106.75088	111.877581	143.10639	240.687684	206.433343	197.938715
5.84E-05	749.349316	791.97551	944.502176	1272.97042	1232.17412	1003.14572
7.39E-12	109.890612	114.821728	116.331646	239.35053	248.201958	238.295152
0.00017095	1284.15029	1292.48048	1269.49217	1580.51579	1484.39229	1683.43994
0.00125375	345.370495	356.241772	309.294456	439.9236	435.357479	447.764277
1.08E-06	294.088209	331.707215	356.381075	504.106983	518.894708	465.059892
0.02780097	161.172898	164.872225	208.65835	238.013377	258.644111	209.469125
1.63E-06	211.408606	238.475897	227.12369	331.614143	355.033221	363.207933
4.75E-16	918.894832	980.40091	1090.37837	1553.77272	1690.82563	1645.00524
5.47E-09	762.95482	762.534041	833.710131	1227.50719	1194.42172	1048.30649
0.00272086	133.961889	126.598316	139.413322	187.201532	182.336066	204.664787
1.48E-17	316.066332	336.614126	372.076615	710.028668	629.742184	649.546461
0.00406403	327.578681	337.595508	376.69295	466.666676	490.781217	401.642634
0.02056163	279.436128	299.321599	280.673178	334.28845	340.574855	398.760032
3.92E-08	182.104443	141.31905	154.185595	335.625604	308.445151	258.47337
0.00238658	90.0056441	103.045141	131.103919	160.458456	180.729581	160.46488
2.00E-06	68.0275217	79.4919656	89.5569023	164.469918	159.042031	144.130132
8.80E-06	53.3754401	55.9387906	65.5519594	17.3829994	19.277822	22.0999536
0.01733415	455.261107	535.834731	407.160762	369.054449	347.804038	391.073091
0.00102835	6.27946354	4.90691146	6.46286924	25.4059222	21.6875497	22.0999536
4.62E-07	1548.93434	1351.36342	1288.88078	970.773659	885.173325	1060.79777
6.66E-19	691.787567	736.036719	770.927973	1176.69534	1261.8941	1285.64078
0.00039626	185.244174	217.866869	213.274685	307.545374	294.790027	280.573324
0.02065766	1697.54831	1705.64242	1811.44992	1544.41264	1584.79761	1469.16648
2.07E-23	497.124197	515.225703	358.227609	1072.39735	1181.56984	1408.63182
0.0054952	214.548338	213.94134	268.670707	294.173836	320.49379	319.968893
4.82E-26	357.929422	361.148683	401.62116	867.812817	835.372285	746.594084

0.06993561	287.808746	279.693953	362.843944	394.460371	371.098073	363.207933
1.31E-06	1674.52361	1683.07063	1874.23208	2614.13568	2488.44552	2114.86947
0.00150335	434.329562	417.087474	496.717664	636.485209	630.545427	521.751078
6.21E-19	178.964711	179.592959	182.806873	406.494755	400.014806	383.386151
6.56E-05	2713.77483	2601.64446	2180.75673	2971.15575	3379.24154	3872.29621
2.38E-10	667.71629	746.831924	713.685417	1113.84912	1159.07905	987.771838
1.40E-08	1957.09947	2025.57305	2088.43003	2574.02107	2665.96213	2600.10758
4.09E-09	1981.17075	2028.5172	2074.58102	2595.41553	2640.25837	2620.2858
0.07707661	1768.71556	1982.39223	2198.29881	2409.55115	2351.09104	2043.76527
1.34E-06	973.316849	810.621773	777.390842	584.336211	604.841664	574.598793
2.53E-07	578.757223	571.164494	500.410732	302.196759	355.033221	385.307886
0.00808619	608.061386	697.76281	782.930445	913.276046	903.647904	792.715726
3.61E-34	2536.90327	2436.77223	2767.0313	4942.12045	4753.5896	4503.58619
2.07E-11	33.4904722	36.3111448	38.7772154	125.692457	115.666932	107.617165
1.38E-09	221.874378	266.935983	247.435565	425.214909	440.176935	398.760032
1.08E-18	204.082565	241.420044	202.19548	454.632292	461.864484	504.455462
7.59E-06	1216.12277	1098.16678	1043.29175	808.97805	767.096665	888.80248
2.29E-09	274.203241	283.619482	259.438036	488.061137	461.061242	416.055648
5.68E-18	226.060687	277.731189	276.056843	620.439364	544.59847	524.63368
2.34E-10	113.030344	140.337668	156.955396	279.465144	289.167329	273.847251
0.00324671	1112.51162	1136.44069	1220.55902	1379.94272	1369.5286	1343.29283
2.91E-07	250.131964	231.606221	201.272213	127.029611	120.486387	125.873649
0.01370755	334.904722	295.39607	317.60386	276.790837	235.350076	228.686476
0.01231297	207.222297	222.77378	171.727668	264.756453	250.611685	283.455926
3.17E-07	492.937888	557.425142	477.329056	755.491897	718.098868	725.454998
7.23E-55	1017.27309	1021.61897	1104.22737	2298.56738	2453.10284	2263.80394
0.00784678	38.7233585	58.8829375	59.0890902	93.6007661	87.5534414	81.6737414
6.56E-09	147.567393	130.523845	168.957867	64.1833824	61.8496788	61.495523
0.0112195	540.033865	597.661816	507.796869	675.262669	642.594065	675.489885
0.01190439	132.915312	101.082376	108.94551	176.504302	138.960967	181.603966
0.01243127	536.894133	657.526136	600.123572	711.365822	758.260997	707.198514
5.69E-06	198.849679	263.991837	240.049429	129.703919	143.780422	130.677986
0.00019913	974.363426	919.555207	852.175472	1182.04396	1169.5212	1104.99768
0.00165339	223.967533	243.382808	250.205366	287.488067	374.311043	330.538436
0.0494624	422.817212	398.44121	436.705307	369.054449	368.688345	313.24282
0.07146207	120.356385	136.412139	130.180652	151.098379	174.30364	165.269218
3.87E-16	1582.42481	1868.55188	1730.20242	2828.08029	2680.42049	2622.20753
1.36E-19	232.340151	235.53175	200.348946	52.1489982	64.2594065	74.9476686
3.31E-12	794.352138	851.839829	704.452747	455.969446	429.734781	491.003316
0.05808431	726.324616	741.925013	831.863597	867.812817	924.532211	843.641706
0.00029735	90.0056441	84.3988771	113.561845	171.155687	191.974977	136.443192
3.90E-05	83.7261806	66.7339958	90.4801693	175.167148	138.157724	139.325794
3.31E-08	728.417771	669.302723	740.460161	980.133736	1050.6413	980.084897

0.00016556	187.337329	170.760519	178.190538	94.9379199	122.092872	109.5389
3.49E-06	196.756524	215.904104	183.73014	287.488067	344.591068	319.968893
6.61E-06	240.712769	259.084925	264.977639	369.054449	370.29483	395.877429
0.00010509	31.3973177	17.6648813	16.6188066	61.5090748	51.4075252	64.3781256
8.62E-05	393.513049	366.055595	409.007296	282.139452	286.757602	217.156066
0.00853553	145.474239	156.039784	151.415794	195.224455	235.350076	191.212642
7.41E-08	730.510925	766.45957	830.94033	1211.46134	1107.67152	1031.97174
0.00166094	129.77558	132.486609	168.0346	244.699146	201.613888	201.782185
0.00214746	294.088209	296.377452	312.987524	395.797525	376.720771	403.56437
0.00021156	196.756524	184.499871	165.264799	121.680996	115.666932	103.773695
6.64E-09	924.127718	942.127	1011.90067	1298.37634	1337.3989	1322.15374
1.82E-08	625.8532	611.401168	732.150758	1088.44319	1073.93533	899.372023
1.23E-05	250.131964	255.159396	305.601388	457.3066	379.933741	406.446972
1.53E-09	871.798855	957.829117	1058.98729	592.359134	662.67513	609.190024
3.34E-14	1234.96116	1252.2438	1218.71248	826.361049	822.520404	828.267825
9.92E-11	68.0275217	70.659525	72.9380957	173.829994	159.845274	173.917026
0.00469886	453.167952	470.082118	521.645874	624.450825	596.005996	577.481395
0.00399825	356.882845	365.074213	400.697893	284.81376	298.002998	285.377661
1.26E-19	3212.99218	3357.30882	3755.85029	6072.01541	5797.80495	5292.45845
0.03723488	2064.89693	2013.79646	1575.09356	1421.39449	1457.88529	1793.93971
0.00026417	718.998576	710.520779	497.640931	842.406895	896.418721	1098.27161
1.92E-16	2282.585	2129.59957	2524.21207	4105.06217	3800.14065	3494.67527
1.38E-64	2158.0423	2177.68731	2125.36071	4222.7317	4159.19009	4171.12602
0.00944385	2201.99855	2364.14994	2238.92256	2527.22068	2544.6725	2639.50315
0.0007315	2468.87575	2857.78523	2746.71943	3215.85489	3165.57901	3267.91053
4.87E-48	265.830623	280.675335	312.987524	874.498586	840.19174	799.441799
1.19E-18	7.3260408	5.88829375	7.38613627	93.6007661	102.81505	73.9868011
0.000881	243.852501	231.606221	248.358832	314.231143	360.655919	317.08629
0.00268702	208.268874	218.848251	162.494998	110.983765	131.731783	145.091
0.00698575	168.498938	161.928078	158.80193	129.703919	104.421536	110.499768
1.47E-08	130.822157	131.505227	127.410851	227.316146	277.118691	235.412549
5.60E-11	341.184186	315.023716	331.452865	529.512905	547.008198	534.242356
0.06507936	643.645013	703.651103	651.826526	747.468975	763.883695	758.124494
0.00039448	364.208885	343.483802	343.455337	457.3066	466.68394	462.17729
0.03555496	330.718413	391.571534	384.079086	328.939835	285.954359	289.221131
3.78E-13	1252.75298	1164.90078	1408.90549	2528.55784	2317.35485	1901.55687
0.00533549	581.896955	531.909202	489.331528	611.079287	659.46216	760.046229
3.31E-14	1456.83554	1568.2489	1609.25444	2360.07646	2243.45653	2200.38668
3.87E-06	5290.44803	4966.77578	4734.51335	3679.84726	4021.03236	4127.88698
0.00805017	93.1453759	120.710022	139.413322	163.132764	168.680942	185.447436
7.52E-05	643.645013	640.842637	647.210191	835.721126	875.534414	784.067918
7.05E-24	8921.02454	8545.877	8162.60385	12773.8303	12602.0729	13431.9674
3.61E-25	1809.53208	1886.21676	2062.57855	3459.21688	3390.48694	3123.78039

	Gene	baseMean	log2FoldChai	lfcSE	stat	pvalue
9	A4GALT	962.337146	-0.6211778	0.08968985	-6.9258422	4.33E-12
12	AAAS	933.727865	-0.7357498	0.08626237	-8.5292091	1.47E-17
21	AADAT	137.815888	-1.0271176	0.18665487	-5.5027637	3.74E-08
23	AAGAB	1869.81102	0.35021693	0.07981912	4.38763232	1.15E-05
25	AAMDC	315.484958	-1.00628	0.16820657	-5.9824063	2.20E-09
28	AAR2	2412.10901	0.30927076	0.06298805	4.90999117	9.11E-07
30	AARS	7953.31604	1.15052222	0.05268377	21.8382662	1.00E-105
31	AARS2	936.527587	0.60235786	0.0822776	7.32104287	2.46E-13
34	AASDHPPT	4297.70535	0.71223667	0.06000001	11.8706091	1.68E-32
35	AASS	1927.76869	-0.4805646	0.08712799	-5.5156166	3.48E-08
36	AATBC	11.2766727	-2.1822999	0.65563969	-3.3285049	0.00087313
37	AATF	7085.06851	0.5942561	0.07238172	8.21002977	2.21E-16
43	ABCA10	164.720535	1.48153769	0.18097543	8.18640258	2.69E-16
44	ABCA11P	369.634047	0.69922029	0.11553863	6.05183122	1.43E-09
48	ABCA2	3824.93311	-0.7671157	0.07649057	-10.028892	1.14E-23
52	ABCA6	75.3349578	-3.1376187	0.29916818	-10.487809	9.83E-26
53	ABCA7	1222.89822	-0.6562546	0.09039944	-7.2594985	3.89E-13
58	ABCB10	1536.24371	-0.3871531	0.07383291	-5.2436393	1.57E-07
62	ABCB6	616.6452	-1.2662436	0.12423194	-10.192577	2.14E-24
72	ABCC3	5824.17242	-0.6119319	0.0540136	-11.32922	9.40E-30
76	ABCC6	46.066864	-1.7279581	0.31265887	-5.5266564	3.26E-08
81	ABCD1	1003.73252	-0.6738838	0.0980979	-6.8695028	6.44E-12
85	ABCE1	8810.12991	0.76762056	0.06358572	12.0722162	1.48E-33
86	ABCF1	9630.14436	0.14781304	0.05664866	2.60929461	0.00907291
87	ABCF2	6315.87116	0.5794769	0.06703942	8.64382382	5.44E-18
88	ABCF3	3017.55372	-0.3174404	0.07037401	-4.5107625	6.46E-06
90	ABCG2	108.750219	0.7973081	0.19821187	4.02250438	5.76E-05
97	ABHD11-AS1	8.46468061	-2.3845971	0.76925522	-3.0998777	0.00193601
98	ABHD12	5844.46795	-0.3283964	0.06882059	-4.7717758	1.83E-06
101	ABHD14A	273.829645	-1.5358831	0.16469148	-9.3258203	1.10E-20
104	ABHD15	673.28481	-0.3518055	0.09371265	-3.7540874	0.00017397
107	ABHD17A	696.924009	-0.9598389	0.10919719	-8.7899598	1.50E-18
109	ABHD17C	2179.27456	-0.246229	0.06514678	-3.7796031	0.00015708
110	ABHD2	3896.34031	-0.310988	0.08724487	-3.564542	0.00036449
111	ABHD3	877.600229	-0.6394494	0.08809447	-7.2586781	3.91E-13
112	ABHD4	2217.01905	-0.5940051	0.0815032	-7.2881198	3.14E-13
120	ABL1	4665.5354	0.36911265	0.07182546	5.1390224	2.76E-07
121	ABL2	7776.58768	1.26077101	0.08304357	15.1820418	4.65E-52
122	ABLIM1	4584.53677	-0.7968494	0.07972465	-9.9950195	1.60E-23
123	ABLIM2	59.7414201	-2.3286662	0.31598063	-7.3696487	1.71E-13
130	ABTB1	1082.32089	-0.881015	0.12055887	-7.3077574	2.72E-13
131	ABTB2	2233.16204	0.70884776	0.0729239	9.72037584	2.47E-22

132	ACAA1	1844.17327	-0.32802	0.10378409	-3.1606004	0.00157444
133	ACAA2	1353.71523	-0.6080328	0.08262552	-7.3588977	1.85E-13
135	ACACB	249.524671	-1.0429477	0.1411968	-7.386482	1.51E-13
136	ACAD10	546.953272	-0.4213312	0.10197208	-4.1318291	3.60E-05
141	ACADM	2241.09664	0.96329646	0.06958795	13.8428629	1.40E-43
142	ACADS	452.31355	-1.6535894	0.14778346	-11.189272	4.60E-29
143	ACADSB	629.957762	-1.3055551	0.10274109	-12.707234	5.39E-37
149	ACAT1	6765.14416	-0.1766023	0.07675718	-2.3007913	0.02140343
150	ACAT2	3031.18782	-0.4217672	0.08208802	-5.1379874	2.78E-07
153	ACBD5	2172.22069	-0.235521	0.0848355	-2.7762078	0.0054997
156	ACCS	210.115764	-1.3291333	0.17205743	-7.7249396	1.12E-14
164	ACHE	75.1667387	1.58177005	0.25407911	6.22550207	4.80E-10
165	ACIN1	6339.48432	-0.4484806	0.06653566	-6.7404544	1.58E-11
172	ACO1	8748.7288	-0.6921303	0.05359695	-12.913613	3.77E-38
173	ACO2	3656.88258	-0.3832675	0.06180494	-6.2012444	5.60E-10
174	ACOT1	31.1958434	-2.4138907	0.51572023	-4.6806205	2.86E-06
175	ACOT11	17.3455323	-1.1636997	0.57358995	-2.0288007	0.0424786
178	ACOT2	402.481249	-0.654856	0.13332268	-4.9118122	9.02E-07
184	ACOX1	1229.69754	-0.3943321	0.07321411	-5.3860122	7.20E-08
185	ACOX2	77.6129028	-1.2915469	0.23024455	-5.609457	2.03E-08
186	ACOX3	837.585844	-0.3452483	0.09811157	-3.5189352	0.00043328
187	ACOXL	98.2544794	0.8946873	0.22349603	4.00314634	6.25E-05
188	ACP1	2779.09185	0.16388852	0.06895062	2.37689694	0.01745897
190	ACP5	17.5629443	-2.6116739	0.57275395	-4.5598531	5.12E-06
200	ACSF2	1356.49528	-0.5473789	0.07994046	-6.8473327	7.52E-12
204	ACSL4	9109.76784	0.8106243	0.07491656	10.8203617	2.76E-27
214	ACSS1	1286.07303	-1.1346234	0.08198676	-13.839106	1.48E-43
215	ACSS2	1869.66227	-0.3899524	0.08205713	-4.7522057	2.01E-06
216	ACSS3	136.684235	0.99317081	0.17940749	5.53583803	3.10E-08
220	ACTB	162197.129	-0.5227065	0.05940203	-8.7994718	1.37E-18
223	ACTG1	107880.658	-0.3296438	0.06125528	-5.381476	7.39E-08
239	ACTN4	29121.4976	-0.7192037	0.07394387	-9.726347	2.33E-22
241	ACTR1A	6084.06317	-0.8793294	0.08642327	-10.174683	2.57E-24
243	ACTR2	10197.4249	0.30447519	0.06613189	4.60405968	4.14E-06
249	ACTR5	642.691252	0.51092518	0.09409824	5.42969981	5.64E-08
250	ACTR6	942.470996	-0.5382146	0.08730949	-6.1644456	7.07E-10
254	ACTRT3	85.1749966	0.61123273	0.22615012	2.70277431	0.00687634
256	ACVR1B	896.923004	0.20498076	0.0812836	2.52179707	0.0116757
258	ACVR2A	524.870071	0.39475345	0.10880117	3.6282095	0.00028539
266	ADA	114.497213	-1.6179876	0.26577058	-6.0879108	1.14E-09
270	ADAM10	15202.1179	-0.2190444	0.07418961	-2.9524948	0.00315217
273	ADAM15	7278.54315	0.70233964	0.07481375	9.38784145	6.12E-21
274	ADAM17	2428.95602	0.27062876	0.09338915	2.89786081	0.00375717

283	ADAM22	389.935355	-1.7947547	0.13238343	-13.557246	7.18E-42
285	ADAM28	896.959822	-1.3875231	0.08312615	-16.691776	1.50E-62
294	ADAM8	7500.21454	-1.6643502	0.06653546	-25.014486	4.25E-138
299	ADAMTS12	2088.66926	0.72009629	0.13481296	5.34144719	9.22E-08
301	ADAMTS14	174.685815	-1.8603945	0.18647788	-9.9764887	1.93E-23
302	ADAMTS15	4336.99362	-0.5139585	0.05942178	-8.6493276	5.18E-18
313	ADAMTS6	299.516947	0.78281755	0.15646185	5.00324873	5.64E-07
314	ADAMTS7	128.28263	-1.4486541	0.19571014	-7.402039	1.34E-13
317	ADAMTS9	1865.82802	0.94401923	0.08480839	11.1312009	8.84E-29
323	ADAMTSL4	649.696675	-0.4372309	0.10008694	-4.368511	1.25E-05
333	ADAT2	323.005013	0.63751851	0.12967987	4.91609469	8.83E-07
336	ADCK2	1654.81328	-0.2907079	0.08851907	-3.2841271	0.00102299
338	ADCK4	548.479924	-0.9861044	0.10667424	-9.2440721	2.37E-20
339	ADCK5	792.476935	-0.6438556	0.13323696	-4.8324097	1.35E-06
353	ADD1	9552.7827	-0.1651924	0.05283795	-3.1263968	0.00176963
355	ADD3	3579.12296	-0.8090906	0.07775237	-10.405993	2.33E-25
365	ADGRD1	407.965974	-0.9176014	0.10972013	-8.3631093	6.11E-17
375	ADGRF5	12287.0133	-0.7629622	0.09297623	-8.2059913	2.29E-16
393	ADH5	4710.45121	-0.3663016	0.05835732	-6.2768757	3.45E-10
397	ADI1	4796.20369	-0.2773186	0.08430362	-3.2895214	0.00100358
403	ADIRF	460.367752	-0.3233411	0.13782808	-2.345974	0.01897743
405	ADM	1283.3623	-0.4995917	0.10193988	-4.9008464	9.54E-07
406	ADM2	277.727757	2.30676097	0.17013278	13.5585918	7.05E-42
408	ADNP	6268.90674	0.29551676	0.06640308	4.45034743	8.57E-06
411	ADO	2428.56386	0.43754582	0.07217311	6.06244911	1.34E-09
412	ADORA1	75.5767084	-1.8498184	0.30642101	-6.0368525	1.57E-09
414	ADORA2A-AS	16.1720119	-1.3891612	0.49605838	-2.8003987	0.00510395
415	ADORA2B	4585.78533	1.61744254	0.06327006	25.5641069	3.83E-144
417	ADPGK	2122.95671	-0.6002946	0.06655608	-9.0193804	1.89E-19
422	ADPRM	320.243438	0.88278764	0.13904005	6.34916085	2.16E-10
429	ADRB1	7.79054584	-2.3889569	0.79380296	-3.0095086	0.00261671
430	ADRB2	2228.99481	0.27206171	0.07746701	3.51196872	0.0004448
432	ADRBK1	3713.39723	-0.209368	0.06571588	-3.1859577	0.00144276
434	ADRM1	8186.07863	0.47289383	0.0702231	6.73416366	1.65E-11
438	ADTRP	103.415099	0.73776681	0.21185065	3.48248554	0.00049678
440	AEBP2	1378.91927	0.32465866	0.07889296	4.11517897	3.87E-05
441	AEN	1430.97676	1.45912834	0.07414092	19.6804717	3.17E-86
444	AFAP1-AS1	1158.96207	-0.4387052	0.0989073	-4.4355191	9.19E-06
446	AFAP1L2	2498.41321	-0.1818628	0.06159103	-2.9527473	0.0031496
447	AFF1	3062.95718	0.29743516	0.09461678	3.14357729	0.00166896
450	AFF4	8722.675	0.77834236	0.09078741	8.57324151	1.01E-17
452	AFG3L2	6362.84823	0.50827295	0.09186137	5.53304324	3.15E-08
454	AFMID	876.942364	-0.3347539	0.09093279	-3.681333	0.00023202

456	AFTPH	1975.89033	0.22319195	0.08095585	2.75695879	0.00583417
460	AGAP11	33.1076387	1.22632962	0.43026089	2.85020008	0.00436917
462	AGAP2-AS1	538.901425	-1.1632886	0.10976667	-10.597831	3.05E-26
468	AGAP9	256.545262	-0.7725892	0.14406074	-5.3629407	8.19E-08
478	AGFG1	3322.5748	0.70116488	0.07710479	9.09366232	9.58E-20
480	AGGF1	1904.65014	-0.5202929	0.08769129	-5.933234	2.97E-09
482	AGL	1445.89821	-0.2802305	0.09315566	-3.0081957	0.00262804
483	AGMAT	180.255193	0.6999173	0.166023	4.21578505	2.49E-05
486	AGO2	1396.08296	0.88953022	0.12120432	7.33909682	2.15E-13
487	AGO3	484.493653	0.72496582	0.11849269	6.11823229	9.46E-10
495	AGPS	5228.50618	0.31804304	0.07359088	4.32177243	1.55E-05
496	AGR2	15.7271976	-7.5176151	1.25990807	-5.9667965	2.42E-09
501	AGTPBP1	3295.61272	0.33989785	0.07948153	4.27643841	1.90E-05
510	AHCYL1	7553.43206	-0.7871452	0.08252208	-9.5386015	1.45E-21
511	AHCYL2	830.017592	-0.4250252	0.09258606	-4.5905961	4.42E-06
513	AHI1	1057.67076	-0.3164331	0.10570856	-2.9934478	0.00275845
516	AHR	7548.32854	0.44667225	0.08171943	5.46592472	4.60E-08
517	AHRR	184.915291	0.80041423	0.17191404	4.65589806	3.23E-06
519	AHSA2	479.555884	-1.3855054	0.1280808	-10.817432	2.85E-27
533	AIMP1	2664.67816	0.67956015	0.06405223	10.6094695	2.69E-26
534	AIMP2	2950.12308	0.90627216	0.10376022	8.73429307	2.45E-18
535	AIP	3898.9551	-0.3004698	0.0894366	-3.3595838	0.0007806
540	AJUBA	16266.4659	0.8422379	0.0573785	14.678632	8.84E-49
541	AK1	3539.82296	-0.3983301	0.10200902	-3.9048517	9.43E-05
542	AK2	5152.49824	0.94532504	0.07206033	13.1185218	2.58E-39
546	AK6	2274.19574	0.6316044	0.09683321	6.52260079	6.91E-11
550	AKAP1	3509.87152	0.6198912	0.06269426	9.88752696	4.72E-23
552	AKAP11	2525.25668	0.56397217	0.0942123	5.98618379	2.15E-09
560	AKAP5	102.669209	-1.2023549	0.22861846	-5.2592207	1.45E-07
564	AKAP8L	2553.85971	0.33776903	0.07350811	4.59499005	4.33E-06
567	AKIRIN1	3445.99362	0.88994899	0.06311852	14.0996495	3.82E-45
568	AKIRIN2	1481.60026	0.50203192	0.07713847	6.50819115	7.61E-11
571	AKR1A1	2237.54974	0.34471749	0.11605197	2.97037175	0.0029744
576	AKR1C2	606.432381	-1.7633845	0.11955103	-14.750057	3.07E-49
592	ALAD	1468.37892	-1.0469906	0.07415127	-14.119657	2.87E-45
597	ALDH16A1	1846.79673	-0.464569	0.14440703	-3.2170798	0.00129503
601	ALDH1A3	1288.0792	-0.9633839	0.08640093	-11.150156	7.15E-29
603	ALDH1L1	105.280632	-2.4540205	0.22938314	-10.698347	1.04E-26
607	ALDH2	3100.59726	-0.5895033	0.07345763	-8.025079	1.01E-15
609	ALDH3A2	2353.68528	-1.0066343	0.07157537	-14.063977	6.32E-45
610	ALDH3B1	1602.05338	-2.3736952	0.07592542	-31.26351	1.46E-214
612	ALDH4A1	527.939106	-0.4206455	0.11098111	-3.7902444	0.0001505
613	ALDH5A1	303.193876	-0.4928103	0.12173991	-4.0480589	5.16E-05

614	ALDH6A1	645.952671	-0.4370522	0.10242828	-4.2669096	1.98E-05
615	ALDH7A1	3129.00427	-0.6138388	0.07363726	-8.3359817	7.69E-17
618	ALDOA	40078.6314	-0.1975585	0.05101147	-3.8728252	0.00010758
620	ALDOC	383.4327	-0.7850586	0.1179597	-6.6553117	2.83E-11
625	ALG12	846.833083	-0.611099	0.09150183	-6.678544	2.41E-11
636	ALG9	1705.87408	-0.1866672	0.07624454	-2.4482697	0.01435441
638	ALKBH1	526.999981	0.86030883	0.10716242	8.02808324	9.90E-16
640	ALKBH3	1131.18376	0.23852177	0.0961949	2.47956781	0.01315417
643	ALKBH5	4559.21214	-0.2678015	0.05853789	-4.5748408	4.77E-06
644	ALKBH6	657.987587	-0.4603505	0.10643284	-4.3252671	1.52E-05
645	ALKBH7	782.252061	-0.7431881	0.13981678	-5.3154426	1.06E-07
646	ALKBH8	848.571984	0.44093774	0.09466142	4.65805111	3.19E-06
648	ALMS1	708.744009	0.46434958	0.13056367	3.55649916	0.00037583
658	ALOX5	372.381242	-1.2235098	0.11579657	-10.566029	4.28E-26
660	ALOXE3	26.5095446	2.05530966	0.45982198	4.46979428	7.83E-06
662	ALPK1	194.911238	-0.9830906	0.15431409	-6.3707117	1.88E-10
663	ALPK2	338.569544	1.60639837	0.15008695	10.7031181	9.84E-27
665	ALPL	4.95138938	2.55717052	1.07296261	2.38328018	0.01715913
668	ALS2	2301.30607	0.4053185	0.07457039	5.43538126	5.47E-08
669	ALS2CL	1394.20444	-0.5720909	0.07307877	-7.8284144	4.94E-15
670	ALS2CR11	200.654639	-0.3924618	0.17380896	-2.2580069	0.02394523
676	AMACR	63.3305881	-1.0924676	0.29262235	-3.7333703	0.00018893
679	AMBRA1	1196.25646	-0.2863152	0.08071255	-3.5473445	0.00038914
680	AMD1	6208.46106	0.413522	0.06746861	6.12910217	8.84E-10
685	AMER1	425.626632	1.19753222	0.14262589	8.3963175	4.61E-17
689	AMH	470.182458	-0.5393465	0.12397007	-4.3506187	1.36E-05
692	AMIGO1	230.329699	-1.0418036	0.18210638	-5.7208521	1.06E-08
693	AMIGO2	8760.26882	1.50383362	0.07010996	21.4496431	4.60E-102
695	AMMECR1	850.792773	1.3602204	0.09219538	14.7536723	2.91E-49
696	AMMECR1L	2455.11418	0.76253787	0.07862302	9.69865894	3.05E-22
697	AMN	97.6249939	0.89399437	0.23488716	3.80605891	0.0001412
703	AMPD2	3162.24642	-0.6245945	0.06909136	-9.0401242	1.56E-19
706	AMT	301.681968	-0.4520282	0.12615775	-3.5830393	0.00033962
712	AMY2B	88.9662018	-1.0629322	0.26751615	-3.9733384	7.09E-05
713	AMZ1	42.2192333	3.90107981	0.47018018	8.29698906	1.07E-16
718	ANAPC11	3616.78148	-0.4237101	0.13838301	-3.0618654	0.00219962
719	ANAPC13	2027.88413	0.29971562	0.07676418	3.90436826	9.45E-05
720	ANAPC15	1133.67533	-0.6158375	0.11689231	-5.2684177	1.38E-07
723	ANAPC2	1843.16037	-0.4153751	0.10706993	-3.8794751	0.00010468
724	ANAPC4	842.215193	-0.5854849	0.08829638	-6.6309054	3.34E-11
730	ANGPT1	251.66874	-0.5061536	0.14943007	-3.3872272	0.00070603
734	ANGPTL2	2707.66926	-0.4522403	0.0730029	-6.1948264	5.83E-10
741	ANK1	759.31846	-0.7227412	0.09915083	-7.2893113	3.12E-13

742	ANK2	546.856348	-1.2409106	0.1184285	-10.478141	1.09E-25
744	ANKAR	74.7216908	-1.1062916	0.24654727	-4.487138	7.22E-06
745	ANKDD1A	58.1978519	-1.10107	0.2642821	-4.1662679	3.10E-05
750	ANKH	1371.19247	-0.6071009	0.08796639	-6.9015098	5.15E-12
762	ANKRD11	6885.20947	0.85499589	0.0814413	10.4983085	8.79E-26
764	ANKRD13A	3113.02818	0.40212249	0.09194338	4.3735883	1.22E-05
765	ANKRD13B	667.062339	1.12955144	0.10044315	11.2456797	2.43E-29
766	ANKRD13C	3330.25788	0.95626187	0.07254342	13.1819247	1.12E-39
767	ANKRD13D	2167.7417	-0.1982815	0.09718286	-2.0402927	0.04132118
769	ANKRD17	4681.96829	0.30239382	0.09940746	3.04196295	0.00235041
774	ANKRD2	28.6578381	-1.8850064	0.40131734	-4.6970469	2.64E-06
791	ANKRD27	2608.21804	0.18379268	0.07219154	2.54590344	0.01089953
792	ANKRD28	7073.201	0.63105246	0.06397422	9.86416737	5.95E-23
799	ANKRD31	9.66849695	1.80975788	0.73240753	2.47097116	0.01347467
802	ANKRD34A	102.358146	1.08460155	0.21441296	5.05847022	4.23E-07
807	ANKRD36	113.237218	-0.5321708	0.19031687	-2.7962353	0.00517017
808	ANKRD36B	62.0545194	-0.8188933	0.27364454	-2.9925441	0.00276663
812	ANKRD37	262.840546	-1.0367087	0.1640871	-6.3180393	2.65E-10
821	ANKRD50	2075.33852	0.34204732	0.09786056	3.49525185	0.00047361
826	ANKRD6	392.490044	-1.1768643	0.11770821	-9.9981497	1.55E-23
832	ANKRD65	57.1968516	-1.8287166	0.33612633	-5.4405634	5.31E-08
842	ANKZF1	2176.48609	-0.9075013	0.08487005	-10.692833	1.10E-26
847	ANO2	50.2023469	4.63636736	0.50320243	9.21372211	3.15E-20
853	ANO8	1107.27747	-1.2913456	0.08977075	-14.384925	6.43E-47
855	ANOS1	1539.46981	1.84146503	0.10853261	16.9669286	1.44E-64
864	ANTXR1	3056.33023	-0.4363986	0.09986475	-4.3698962	1.24E-05
869	ANXA10	23.4005682	2.16547803	0.46628114	4.64414669	3.41E-06
870	ANXA11	6628.71701	-0.5726919	0.05260867	-10.885885	1.35E-27
878	ANXA4	2551.12715	-0.2800053	0.06342211	-4.4149477	1.01E-05
880	ANXA6	10253.6002	-1.055851	0.0519622	-20.3196	8.63E-92
882	ANXA8	255.468569	-1.4574222	0.15223586	-9.5734488	1.03E-21
883	ANXA8L1	162.316188	-1.8365719	0.17222489	-10.663801	1.50E-26
888	AOC2	151.407077	1.9630961	0.20484772	9.58319718	9.41E-22
889	AOC3	35.5409724	1.61858998	0.39555973	4.09189778	4.28E-05
893	AP1AR	2722.29969	0.94167767	0.06583529	14.3035396	2.08E-46
898	AP1M1	4751.20766	-0.3840561	0.0568876	-6.7511385	1.47E-11
901	AP1S2	3979.66049	-0.7465623	0.06166576	-12.106594	9.75E-34
903	AP2A1	5813.12983	-0.6961109	0.06243223	-11.149866	7.17E-29
904	AP2A2	4447.92601	-0.1848872	0.07991167	-2.3136446	0.02068723
907	AP2S1	6748.87585	-0.2573607	0.12012053	-2.1425201	0.03215165
912	AP3M2	1498.53792	0.68243314	0.07454931	9.15411757	5.48E-20
921	AP5M1	2139.17207	-0.4067557	0.07508757	-5.4170841	6.06E-08
922	AP5S1	646.938829	-0.928478	0.096152	-9.656357	4.62E-22

925	APBA1	22.2184216	-1.3736832	0.47082671	-2.9175983	0.00352738
928	APBB1	1853.20859	-0.3161031	0.07541043	-4.1917695	2.77E-05
936	APCDD1L-AS	256.681566	1.61585013	0.14325905	11.2792187	1.66E-29
938	APEH	2328.37802	-1.3356074	0.07970432	-16.757028	5.03E-63
940	APEX1	5456.00687	-0.4356139	0.06111639	-7.127612	1.02E-12
942	APH1A	6647.8823	-0.4310474	0.06192689	-6.9605858	3.39E-12
943	APH1B	702.049626	-0.8982919	0.08966683	-10.018107	1.27E-23
949	APLN	435.959113	-1.7180072	0.11482439	-14.962042	1.30E-50
952	APLP2	51123.6493	-0.4502566	0.05109391	-8.8123337	1.23E-18
953	APMAP	9951.45768	-0.2129787	0.07368961	-2.8902136	0.0038498
965	APOBEC3B	462.282427	-1.0140656	0.11821465	-8.5781714	9.64E-18
967	APOBEC3C	405.647893	-1.0431535	0.11387158	-9.1607887	5.15E-20
984	APOL1	1030.10024	-0.769732	0.07789619	-9.8815105	5.01E-23
995	APOPT1	1124.88282	0.48442809	0.09122103	5.31048701	1.09E-07
997	APPBP2	2180.07243	0.66027595	0.07836697	8.42543727	3.59E-17
1001	APTR	376.676623	0.76184752	0.13503225	5.64196701	1.68E-08
1002	APTX	1644.51431	0.47649171	0.07806403	6.10385704	1.04E-09
1009	AQP3	744.5354	-0.849282	0.0906938	-9.364278	7.66E-21
1012	AQP5	38.0827836	-3.8649315	0.48693864	-7.9372045	2.07E-15
1013	AQP6	15.664928	-1.7833062	0.57404126	-3.106582	0.00189264
1021	ARAF	3235.76342	-0.3999537	0.06181729	-6.4699322	9.80E-11
1022	ARAP1	4555.60042	-0.5741793	0.0780653	-7.355116	1.91E-13
1023	ARAP2	559.41312	1.40283078	0.11276956	12.4397995	1.59E-35
1024	ARAP3	1570.16641	-0.4776559	0.0694521	-6.8774868	6.09E-12
1025	ARC	9.17142098	1.87720733	0.71561943	2.62319223	0.00871101
1026	ARCN1	15771.8584	0.41765573	0.07896626	5.28904046	1.23E-07
1027	AREG	2117.13277	1.26372133	0.08070916	15.6577182	2.94E-55
1028	AREL1	2681.83666	-0.3216271	0.07799027	-4.1239391	3.72E-05
1029	ARF1	18681.1749	-0.1259695	0.04982923	-2.5280232	0.01147068
1030	ARF3	5259.0091	-0.4426134	0.08088446	-5.4721683	4.45E-08
1031	ARF4	17159.6996	0.40298994	0.0573836	7.02273746	2.18E-12
1032	ARF5	4093.98188	-0.3577775	0.09319662	-3.838953	0.00012356
1033	ARF6	12842.0883	0.66529413	0.06928696	9.60201052	7.84E-22
1034	ARFGAP1	5819.68115	0.26870196	0.07947396	3.38100611	0.00072221
1036	ARFGAP3	2905.95836	0.52871615	0.06064023	8.71890046	2.81E-18
1037	ARFGEF1	2401.90642	0.70097538	0.07611211	9.20977492	3.27E-20
1038	ARFGEF2	6772.96957	0.4443843	0.07906655	5.62038278	1.91E-08
1050	ARHGAP11A	2496.41861	-0.2430512	0.07033658	-3.4555447	0.00054918
1063	ARHGAP25	85.4053062	-1.6597994	0.25270987	-6.568004	5.10E-11
1067	ARHGAP27	1658.43874	-0.6267772	0.08028821	-7.8065903	5.88E-15
1069	ARHGAP29	12290.525	0.59758126	0.09196228	6.49811278	8.13E-11
1073	ARHGAP32	4601.43036	0.49383856	0.08272709	5.96949054	2.38E-09
1074	ARHGAP33	317.217801	-0.5738567	0.11941195	-4.805689	1.54E-06

1077	ARHGAP39	1539.47144	1.0916915	0.08004184	13.6390112	2.35E-42
1083	ARHGAP5-AS	365.140736	0.24095276	0.11737954	2.05276623	0.04009525
1087	ARHGDIA	22919.6007	0.22314512	0.05658628	3.94344927	8.03E-05
1088	ARHGDIB	697.808709	-5.1120555	0.15923459	-32.103926	3.89E-226
1090	ARHGEF1	3694.27065	-0.3492931	0.06851899	-5.0977563	3.44E-07
1096	ARHGEF16	168.621654	0.77405263	0.16354768	4.73288665	2.21E-06
1097	ARHGEF17	2093.53675	-0.4218372	0.07844623	-5.377406	7.56E-08
1098	ARHGEF18	5217.74462	0.32590915	0.06072732	5.36676288	8.02E-08
1100	ARHGEF2	3941.24244	0.81474243	0.05809732	14.0237521	1.12E-44
1101	ARHGEF25	92.0492747	-2.4258236	0.24262653	-9.9981794	1.55E-23
1110	ARHGEF37	142.736592	-1.0926513	0.17334195	-6.3034441	2.91E-10
1114	ARHGEF4	1073.97807	-0.888207	0.0780494	-11.380062	5.26E-30
1121	ARHGEF9	457.662052	-0.7994095	0.11321892	-7.0607411	1.66E-12
1123	ARID1A	2745.597	0.4959805	0.1019508	4.86490032	1.15E-06
1127	ARID3B	154.22398	1.46800678	0.17653273	8.31577661	9.12E-17
1133	ARIH1	2733.89753	0.44001679	0.06702135	6.56532271	5.19E-11
1140	ARL13B	1745.57412	0.80850321	0.07986434	10.1234568	4.35E-24
1142	ARL14EP	1353.40123	0.64890181	0.07399569	8.76945383	1.80E-18
1143	ARL14EPL	43.585765	1.21878459	0.32279822	3.77568557	0.00015957
1145	ARL16	1188.19596	0.59927065	0.09904815	6.05029637	1.45E-09
1148	ARL2	4318.47015	-0.2677665	0.07358943	-3.6386547	0.00027407
1151	ARL3	1326.81162	-0.4474149	0.07810151	-5.7286335	1.01E-08
1152	ARL4A	1424.4419	0.51372764	0.07921047	6.48560302	8.84E-11
1154	ARL4D	1288.51181	1.26961557	0.14556908	8.7217394	2.74E-18
1156	ARL5B	874.192999	0.97667499	0.15358616	6.35913418	2.03E-10
1159	ARL6IP1	12802.8439	-0.178667	0.06990302	-2.5559263	0.01059055
1160	ARL6IP4	4479.45186	-0.3750324	0.08874204	-4.2260968	2.38E-05
1161	ARL6IP5	5782.56739	-0.5904466	0.06368689	-9.2710849	1.84E-20
1165	ARL9	2.51245436	4.69559368	1.64126317	2.86096329	0.00422356
1174	ARMC6	3572.32457	0.39272062	0.08978243	4.37413694	1.22E-05
1176	ARMC8	1521.84959	0.20893382	0.07478538	2.79377892	0.00520961
1178	ARMCX1	1593.03538	-1.2401505	0.07522692	-16.485462	4.67E-61
1186	ARMT1	1975.32542	0.41470567	0.07995882	5.1864907	2.14E-07
1189	ARNTL	487.64592	0.79844096	0.12134551	6.57989676	4.71E-11
1193	ARPC1B	8006.61284	-0.2733151	0.08127325	-3.3629163	0.00077124
1195	ARPC3	5671.4054	0.74606628	0.07804738	9.55914628	1.19E-21
1199	ARPC5L	3821.74915	0.74540592	0.09387151	7.94070449	2.01E-15
1200	ARPIN	1131.26037	-0.6085588	0.07438834	-8.1808365	2.82E-16
1204	ARRB1	3082.56894	-0.7989231	0.06339526	-12.602254	2.05E-36
1205	ARRB2	1886.42473	0.61031559	0.06742265	9.0520854	1.40E-19
1211	ARRDC4	570.332568	2.86350472	0.13325087	21.4895757	1.95E-102
1214	ARSB	820.105358	-0.3776157	0.08552316	-4.4153616	1.01E-05
1216	ARSE	262.882789	-0.9870455	0.13167099	-7.4963019	6.56E-14

1220	ARSI	490.145767	-0.831458	0.12500624	-6.6513322	2.90E-11
1221	ARSJ	732.871152	-0.3461055	0.10138038	-3.4139304	0.00064033
1232	ASAH1	2817.33953	-2.6330995	0.06651066	-39.589137	0
1235	ASAP1	7194.36579	0.46632796	0.10521482	4.43215107	9.33E-06
1243	ASB12	6.49645669	-4.3066528	1.26390532	-3.4074172	0.00065581
1244	ASB13	1455.37643	-0.4657298	0.09197977	-5.0633941	4.12E-07
1255	ASB6	2405.4041	0.72611756	0.06266902	11.5865468	4.82E-31
1256	ASB7	737.678552	0.49715639	0.10182205	4.88260075	1.05E-06
1257	ASB8	660.986555	-0.5184537	0.09651292	-5.3718581	7.79E-08
1258	ASB9	183.456384	-0.9905426	0.16944015	-5.8459734	5.04E-09
1261	ASCC2	1165.11209	-0.5854872	0.08189506	-7.1492379	8.73E-13
1262	ASCC3	1708.33321	0.32399055	0.08011671	4.04398232	5.26E-05
1269	ASF1B	2466.25404	-0.8980407	0.07057929	-12.723856	4.36E-37
1270	ASGR1	51.8082408	1.06316097	0.32346428	3.28679558	0.00101334
1272	ASH1L	2759.95587	0.50108429	0.1245961	4.02166931	5.78E-05
1274	ASH2L	1439.11171	-0.3778456	0.08364277	-4.5173732	6.26E-06
1275	ASIC1	674.889989	-0.8503286	0.09768609	-8.7047048	3.18E-18
1284	ASMTL-AS1	278.123021	-0.4125764	0.13831195	-2.9829409	0.00285493
1286	ASNS	5288.44533	2.51654197	0.06052729	41.5769795	0
1287	ASNSD1	3254.07549	-0.2112441	0.06756568	-3.1264996	0.00176901
1292	ASPHD1	868.558785	0.36805049	0.13810406	2.66502295	0.00769831
1293	ASPHD2	301.016652	1.43016542	0.13673067	10.4597263	1.32E-25
1294	ASPM	3797.34778	-0.5382366	0.11285924	-4.7690968	1.85E-06
1299	ASS1	2529.12076	2.09197564	0.0757322	27.6233314	5.84E-168
1303	ASTN2	81.8823054	-0.722149	0.23615717	-3.057917	0.00222881
1306	ASXL1	11606.0969	0.23013901	0.05858743	3.92812943	8.56E-05
1310	ATAD1	2644.81605	0.24522824	0.06961562	3.52260367	0.00042733
1313	ATAD3A	2408.48412	0.35053581	0.10163134	3.44909163	0.00056248
1323	ATF3	751.066815	2.29005085	0.11354119	20.1693403	1.82E-90
1324	ATF4	7319.59076	0.81638041	0.0699648	11.6684449	1.85E-31
1329	ATF7IP	2668.16847	0.20912345	0.08363141	2.50053708	0.01240052
1331	ATG10	151.723553	-1.0222134	0.18156005	-5.6301667	1.80E-08
1332	ATG101	2404.06967	1.05243384	0.10406761	10.1129821	4.84E-24
1333	ATG12	1937.35014	0.2312913	0.07687954	3.00848959	0.0026255
1335	ATG14	1358.44345	0.2840354	0.08229917	3.45125455	0.00055799
1337	ATG16L2	526.938087	-1.5758688	0.14128725	-11.153652	6.87E-29
1339	ATG2B	1559.67098	0.57437635	0.0954664	6.01652863	1.78E-09
1345	ATG5	2740.36777	0.51459525	0.08503217	6.05177159	1.43E-09
1347	ATG9A	4024.13396	-0.5838752	0.06779845	-8.611926	7.18E-18
1349	ATHL1	371.915535	-1.435635	0.13614359	-10.545006	5.36E-26
1350	ATIC	5316.27699	0.46603702	0.06044826	7.70968489	1.26E-14
1351	ATL1	440.065018	-0.5185768	0.12056669	-4.3011619	1.70E-05
1359	ATOH8	241.331483	-2.3128147	0.16677261	-13.868073	9.89E-44

1370	ATP13A1	3079.77218	-0.2851684	0.07096459	-4.0184607	5.86E-05
1372	ATP13A3	11644.4149	0.86393034	0.08667737	9.96719661	2.12E-23
1377	ATP1A1	62521.6825	-0.2655248	0.05160225	-5.1456052	2.67E-07
1390	ATP2B1	19146.1465	1.27188802	0.06409604	19.8434718	1.25E-87
1399	ATP5A1	23520.0427	-0.4640121	0.0726891	-6.3835174	1.73E-10
1401	ATP5C1	6534.82463	-0.2919391	0.06209561	-4.7014449	2.58E-06
1405	ATP5F1	5311.44916	-0.2003051	0.06181801	-3.2402396	0.00119429
1408	ATP5G3	9374.10111	-0.3109627	0.0951407	-3.2684509	0.00108138
1412	ATP5J2	2104.88581	-1.0897397	0.25567397	-4.2622241	2.02E-05
1416	ATP5O	4848.73183	0.19905141	0.08812473	2.25874626	0.02389917
1419	ATP6AP1	5498.61615	-0.1130857	0.05496846	-2.0572825	0.03965906
1423	ATP6V0A2	1731.57046	0.74738334	0.07035961	10.6223348	2.35E-26
1424	ATP6V0A4	951.032655	-0.3760978	0.08027039	-4.6853866	2.79E-06
1425	ATP6V0B	4814.44576	0.44730862	0.08897161	5.02754306	4.97E-07
1428	ATP6V0D1	2488.81519	-0.574507	0.08374232	-6.8604135	6.87E-12
1430	ATP6V0E1	8737.75918	-0.3500719	0.08347399	-4.1937837	2.74E-05
1431	ATP6V0E2	2500.08018	-0.7865507	0.10594938	-7.4238349	1.14E-13
1436	ATP6V1B2	4737.19151	0.27842975	0.07679695	3.6255313	0.00028837
1437	ATP6V1C1	7870.12544	0.31456932	0.05509477	5.70960382	1.13E-08
1439	ATP6V1D	3508.25461	-0.1685443	0.06151885	-2.7397172	0.00614921
1452	ATP8B1	1320.22082	-0.5707015	0.09519982	-5.9947743	2.04E-09
1454	ATP8B3	244.636716	-1.4793698	0.1436366	-10.299393	7.09E-25
1457	ATP9A	774.938641	-0.3280344	0.08860697	-3.702129	0.0002138
1458	ATP9B	902.176047	-0.717444	0.09393691	-7.6375087	2.21E-14
1459	ATPAF1	1591.28274	0.418988	0.08113856	5.16385786	2.42E-07
1460	ATPAF2	342.470395	-0.569848	0.12250183	-4.6517507	3.29E-06
1461	ATPIF1	3232.35047	0.39851827	0.10493218	3.79786519	0.00014595
1462	ATR	1467.74971	0.2281311	0.08257262	2.76279352	0.0057309
1463	ATRAID	2023.95002	-0.3375544	0.07106314	-4.7500633	2.03E-06
1465	ATRN	2898.14929	-0.2876899	0.09776912	-2.9425442	0.00325527
1466	ATRNL1	713.399955	-1.2089211	0.09555511	-12.65156	1.10E-36
1468	ATXN1	1572.70779	0.59458869	0.08670902	6.85728796	7.02E-12
1472	ATXN2L	6499.29934	0.65343796	0.09152387	7.13953617	9.36E-13
1473	ATXN3	817.996071	0.27691529	0.08848795	3.12941236	0.00175156
1475	ATXN7	1757.38534	0.56296672	0.0970684	5.79969102	6.64E-09
1478	ATXN7L3	4341.58807	0.35248143	0.06008801	5.86608612	4.46E-09
1479	ATXN7L3B	2326.08882	0.34895905	0.06272553	5.5632695	2.65E-08
1481	AUH	543.482991	-0.4129961	0.09826205	-4.2030067	2.63E-05
1482	AUNIP	322.210351	0.90869118	0.12233986	7.42759732	1.11E-13
1484	AURKA	5174.41406	-0.4620988	0.05721095	-8.0771048	6.63E-16
1503	AXL	39608.836	0.24204825	0.05570467	4.34520576	1.39E-05
1507	AZIN1	13743.4099	0.64395021	0.05631795	11.4341915	2.82E-30
1513	B3GALNT2	2490.96804	0.66058406	0.07343016	8.99608693	2.34E-19

1516	B3GALT4	47.1046742	-2.0702449	0.32112626	-6.4468255	1.14E-10
1517	B3GALT5	48.7191592	0.67811055	0.31660999	2.14178503	0.03221078
1522	B3GAT3	1459.71087	-0.6377924	0.11980734	-5.3234837	1.02E-07
1523	B3GLCT	1504.4337	-0.4563277	0.08254435	-5.5282725	3.23E-08
1526	B3GNT4	61.3054313	-1.1017934	0.25613537	-4.3016058	1.70E-05
1527	B3GNT5	2225.2275	0.39386918	0.08084517	4.87189512	1.11E-06
1532	B3GNTL1	425.819547	-1.0036876	0.11833055	-8.4820665	2.21E-17
1539	B4GALT2	3494.96226	0.71651402	0.06841381	10.473237	1.15E-25
1546	B4GAT1	1559.48923	-1.3662111	0.09196294	-14.856104	6.35E-50
1554	BACE1	3207.57516	-0.2239148	0.07563766	-2.9603617	0.00307278
1556	BACE2	4241.3027	-0.1939993	0.06003374	-3.2315043	0.0012314
1557	BACH1	3400.90443	0.48996531	0.09419604	5.20154891	1.98E-07
1561	BAG1	3557.92192	-0.356528	0.10242571	-3.4808444	0.00049984
1562	BAG2	3224.54203	0.48423538	0.06045235	8.01019918	1.15E-15
1564	BAG4	1179.31743	0.76441741	0.08048608	9.49751071	2.15E-21
1575	BAIAP2-AS1	463.549182	-0.5911434	0.12121083	-4.8769848	1.08E-06
1578	BAIAP3	269.979283	-2.3718236	0.14804404	-16.021068	9.11E-58
1579	BAK1	1330.91877	0.4653177	0.0737799	6.30683548	2.85E-10
1585	BANP	675.303472	0.49043844	0.09090574	5.39502169	6.85E-08
1586	BAP1	5558.24148	-0.2997679	0.05917021	-5.0661959	4.06E-07
1596	BATF3	491.697492	0.26251789	0.10246761	2.56195972	0.01040834
1598	BAZ1A	4109.65639	0.80762876	0.05779748	13.9734257	2.26E-44
1607	BBS10	1079.56086	0.46290329	0.11114558	4.16483771	3.12E-05
1608	BBS12	408.40858	0.3378885	0.11550199	2.92539112	0.00344024
1609	BBS2	1133.80752	-0.6656641	0.07759816	-8.578348	9.62E-18
1610	BBS4	150.410402	-1.1659073	0.17639665	-6.6095773	3.85E-11
1612	BBS7	1356.96697	0.31409785	0.08126024	3.86533257	0.00011094
1613	BBS9	328.174465	-0.5786775	0.11945798	-4.844193	1.27E-06
1615	BCAM	1967.15296	-0.5230866	0.0824492	-6.3443504	2.23E-10
1620	BCAR3	9603.84391	1.16980779	0.06057322	19.3122917	4.23E-83
1625	BCAS4	566.648228	-0.7186328	0.11070891	-6.491192	8.52E-11
1626	BCAT1	7946.54587	0.53885544	0.05847563	9.21504294	3.11E-20
1627	BCAT2	1591.92465	-0.7330446	0.08089661	-9.0614999	1.29E-19
1631	BCHE	3.75882376	-3.5191158	1.39767704	-2.5178318	0.01180797
1633	BCKDHB	552.872084	-0.9180555	0.10581884	-8.6757285	4.11E-18
1638	BCL2	366.865633	-0.63878	0.13894135	-4.5974793	4.28E-06
1640	BCL2L1	25003.1563	0.51928476	0.05906128	8.79230468	1.47E-18
1642	BCL2L11	1084.704	-0.6244159	0.09380954	-6.6562091	2.81E-11
1643	BCL2L12	1755.73677	-0.4075062	0.11650442	-3.4977746	0.00046916
1650	BCL6	1759.95284	0.82326612	0.08358609	9.84931938	6.90E-23
1653	BCL7B	3408.55378	0.40927919	0.06640711	6.1631832	7.13E-10
1654	BCL7C	725.610582	-1.0279725	0.12327595	-8.3387914	7.51E-17
1660	BCOR	1306.57586	0.74349895	0.09990234	7.44225753	9.90E-14

1663	BCR	2346.58983	-0.4870781	0.0896698	-5.4319077	5.58E-08
1668	BDH1	1131.71921	-0.2106816	0.08131836	-2.5908249	0.00957462
1669	BDH2	504.243112	-1.0011234	0.10783596	-9.2837616	1.64E-20
1670	BDKRB1	128.05833	1.82337896	0.20915932	8.71765575	2.84E-18
1671	BDKRB2	97.6413888	1.00109172	0.21382397	4.68184977	2.84E-06
1685	BEND6	235.799769	0.81612676	0.15534309	5.25370483	1.49E-07
1686	BEND7	1092.86042	0.58232827	0.08370605	6.95682451	3.48E-12
1694	BEX2	535.340417	1.1420773	0.1497187	7.62815399	2.38E-14
1714	BICC1	1367.28182	-0.4409304	0.07825116	-5.6348097	1.75E-08
1717	BID	1796.07807	-0.2595587	0.07180939	-3.6145511	0.00030087
1724	BIRC3	8499.7791	0.77634345	0.06476962	11.9862287	4.20E-33
1725	BIRC5	4393.88239	-0.4315216	0.08124838	-5.3111404	1.09E-07
1726	BIRC6	3070.76065	0.33369017	0.09597086	3.47699463	0.00050707
1728	BIRC7	7.12327988	-3.4441228	0.99262626	-3.4697075	0.00052103
1744	BLOC1S3	592.187717	0.26952082	0.11317232	2.38150822	0.01724191
1750	BLVRB	3138.08581	0.86054707	0.08838142	9.73674232	2.10E-22
1751	BLZF1	1027.71941	0.2688436	0.09580719	2.80609018	0.00501467
1752	BMF	322.0504	-1.4357131	0.12654374	-11.345588	7.80E-30
1753	BMI1	202.5215	1.60571401	0.19985297	8.03447676	9.40E-16
1754	BMP1	2528.28827	-0.4357177	0.06924331	-6.2925603	3.12E-10
1762	BMP6	18.6148587	4.04563735	0.73171599	5.52897216	3.22E-08
1768	BMPR1A	649.966122	-0.3694967	0.10422515	-3.5451779	0.00039235
1771	BMPR2	3207.83385	-0.3254295	0.10397713	-3.1298185	0.00174914
1783	BNIP1	705.262078	0.52252511	0.09850575	5.30451399	1.13E-07
1785	BNIP3	8916.60323	-0.565423	0.05546698	-10.193868	2.11E-24
1789	BOD1	4593.31808	0.85777043	0.06086533	14.0929238	4.20E-45
1794	BOLA1	145.278342	-0.7706019	0.19767011	-3.8984237	9.68E-05
1798	BOLA3-AS1	36.5540209	-1.6542684	0.36088767	-4.5838874	4.56E-06
1800	BOP1	6397.37883	0.7271721	0.10763527	6.7558907	1.42E-11
1804	BPHL	958.797011	-0.3945861	0.07906706	-4.990525	6.02E-07
1821	BRAF	389.245066	0.60017641	0.14704471	4.08159122	4.47E-05
1822	BRAP	1617.00619	0.57899314	0.08432754	6.86600293	6.60E-12
1823	BRAT1	2323.91612	-0.6610823	0.12342113	-5.3563136	8.49E-08
1830	BRD2	11015.8302	0.3623623	0.05148961	7.03758072	1.96E-12
1832	BRD4	4002.26077	0.23119538	0.08563355	2.69982247	0.00693765
1835	BRD8	3246.18155	-0.6560197	0.062795	-10.447005	1.51E-25
1843	BRF2	419.656195	0.77598585	0.11579286	6.70150011	2.06E-11
1850	BRIP1	1286.19265	-0.2979991	0.11604286	-2.5680093	0.01022844
1856	BRPF1	1454.36505	0.30089315	0.08397662	3.58305853	0.00033959
1857	BRPF3	6121.9885	0.62369648	0.09241736	6.74869362	1.49E-11
1861	BRWD1	2117.3939	0.53741362	0.07835828	6.85841545	6.96E-12
1867	BSG	23737.2177	-0.1917279	0.05362697	-3.575215	0.00034994
1874	BST2	5939.93373	-0.3909169	0.11104653	-3.5202978	0.00043106

1878	BTBD10	2690.45251	0.63479644	0.06584239	9.64115139	5.36E-22
1884	BTBD2	2557.00151	-0.7890325	0.06274611	-12.575002	2.90E-36
1890	BTC	53.5915159	-0.7364238	0.33594536	-2.1920941	0.02837271
1891	BTD	924.691548	-0.7520731	0.08993371	-8.3625275	6.14E-17
1893	BTF3L4	1594.3536	0.96165557	0.0709429	13.5553467	7.37E-42
1896	BTG2	44.9114763	1.07625478	0.32680659	3.29324687	0.00099037
1897	BTG3	1900.21302	1.17259104	0.08698513	13.4803615	2.04E-41
1906	BTN3A2	1245.8302	-0.2244356	0.07571504	-2.9642141	0.00303457
1915	BUB1B	2836.47485	-0.3844592	0.09748831	-3.9436438	8.03E-05
1917	BUD13	1362.09717	-0.2039364	0.08042961	-2.5355886	0.01122585
1921	BYSL	3243.47794	0.64800765	0.06342558	10.2168189	1.67E-24
1922	BZRAP1	686.939376	-1.1957954	0.10897889	-10.972725	5.17E-28
1926	C10orf10	234.742592	-1.647852	0.15244375	-10.809574	3.10E-27
1944	C10orf54	2100.10579	-0.7986314	0.07636354	-10.458281	1.34E-25
1952	C10orf88	727.257291	0.22279914	0.08912481	2.49985548	0.0124244
1968	C11orf49	1046.52758	-0.4834313	0.08013068	-6.0330357	1.61E-09
1972	C11orf57	2166.28331	0.49498715	0.06719558	7.36636457	1.75E-13
1973	C11orf58	9841.70329	0.69805825	0.05725794	12.1914653	3.45E-34
1978	C11orf71	221.032294	-1.0678442	0.15340165	-6.9611	3.38E-12
1980	C11orf73	837.539922	0.21079539	0.09981891	2.11177818	0.03470548
1982	C11orf80	942.520823	-0.6294201	0.08730297	-7.2096067	5.61E-13
1985	C11orf86	132.700911	-1.2455505	0.22834865	-5.4545996	4.91E-08
1991	C11orf96	44.4025418	0.81107095	0.39909309	2.03228512	0.0421248
1995	C12orf29	1954.11307	0.69894536	0.07746382	9.02286193	1.83E-19
1996	C12orf4	927.93394	0.39237422	0.08206926	4.78101307	1.74E-06
2001	C12orf49	3501.35228	0.41923186	0.06311218	6.64264539	3.08E-11
2008	C12orf66	155.25252	1.13295745	0.18948452	5.97915578	2.24E-09
2012	C12orf75	5551.75449	0.24083757	0.05924975	4.06478638	4.81E-05
2019	C14orf119	3550.83398	0.21658079	0.07244802	2.98946468	0.00279467
2021	C14orf142	939.507508	0.94503409	0.09009707	10.4890653	9.70E-26
2022	C14orf159	620.319915	-0.5150049	0.10122408	-5.0877707	3.62E-07
2023	C14orf166	6211.43725	0.20227858	0.06948901	2.91094361	0.00360339
2032	C14orf79	250.913787	0.36557378	0.16515033	2.21358185	0.02685756
2034	C14orf93	236.463256	-0.5301734	0.13457306	-3.93967	8.16E-05
2041	C15orf41	321.048647	0.31241761	0.12801482	2.44048	0.01466776
2043	C15orf48	8031.2075	-0.8556381	0.1323382	-6.4655416	1.01E-10
2044	C15orf52	5075.51135	-0.2304259	0.0736082	-3.1304386	0.00174545
2058	C16orf52	488.837014	0.5175413	0.1199028	4.31634041	1.59E-05
2061	C16orf59	436.861282	-0.3799837	0.11507833	-3.3019573	0.00096013
2065	C16orf72	2185.30729	0.78762859	0.0836919	9.41104954	4.91E-21
2076	C16orf95	133.75022	-1.0482984	0.18749189	-5.5911667	2.26E-08
2083	C17orf107	86.597562	0.93877524	0.25083643	3.74257926	0.00018214
2088	C17orf51	2127.09989	1.22006208	0.07980139	15.2887329	9.09E-53

2090	C17orf58	231.946385	-0.7787859	0.13884356	-5.6090888	2.03E-08
2092	C17orf62	1676.1377	-0.3803868	0.10931897	-3.4796048	0.00050215
2099	C17orf80	1198.20923	0.4284186	0.10473254	4.09059704	4.30E-05
2103	C17orf96	1119.19148	0.91991197	0.10877615	8.4569272	2.75E-17
2115	C18orf8	2591.3639	0.53193273	0.06734981	7.89805879	2.83E-15
2124	C19orf44	354.125888	0.60055584	0.13306838	4.51313692	6.39E-06
2127	C19orf48	3267.07935	0.56442227	0.07454093	7.57197789	3.68E-14
2130	C19orf54	982.081486	0.29590399	0.08556919	3.45806682	0.00054407
2132	C19orf60	844.683572	-1.0386913	0.31789255	-3.267429	0.00108529
2133	C19orf66	890.111536	-0.7276939	0.08386744	-8.6767158	4.07E-18
2148	C1QBP	11299.267	0.83515815	0.06138137	13.6060535	3.69E-42
2150	C1QL1	100.499176	-2.3581514	0.2677979	-8.8057126	1.30E-18
2161	C1QTNF6	212.197959	-1.3703966	0.15407019	-8.8946254	5.86E-19
2167	C1R	1356.71318	-2.8405512	0.09112408	-31.172343	2.53E-213
2168	C1RL	217.317988	-0.652219	0.17771645	-3.6699982	0.00024255
2170	C1S	592.070415	-2.3059098	0.11702255	-19.704833	1.96E-86
2174	C1orf106	73.537818	-3.5965712	0.33381777	-10.774055	4.56E-27
2175	C1orf109	1346.69332	1.52954353	0.08686413	17.6084602	2.12E-69
2176	C1orf110	453.935801	1.1663508	0.14922286	7.81616718	5.45E-15
2202	C1orf198	1143.90034	0.54530251	0.08380968	6.50643831	7.70E-11
2204	C1orf21	1528.16624	-0.5548212	0.0739711	-7.5005137	6.36E-14
2206	C1orf216	1191.45725	0.24609581	0.08609427	2.85844589	0.00425722
2208	C1orf226	528.399853	-0.730609	0.10009249	-7.2993393	2.89E-13
2215	C1orf43	9327.03612	0.36895218	0.05983733	6.16591994	7.01E-10
2216	C1orf50	199.54424	0.48419446	0.14599655	3.31647876	0.0009116
2228	C2	2214.92552	-0.4295637	0.06917592	-6.2097294	5.31E-10
2249	C21orf58	305.865207	-0.5424698	0.13673826	-3.967213	7.27E-05
2250	C21orf59	1569.60274	0.51365922	0.09143076	5.61801323	1.93E-08
2253	C21orf91	342.580409	0.78469467	0.12981651	6.04464468	1.50E-09
2265	C2CD2L	2180.67	0.71721725	0.0698277	10.2712431	9.50E-25
2281	C2orf49	1363.01643	0.47988527	0.07616931	6.30024451	2.97E-10
2287	C2orf68	1282.06265	-0.390002	0.08916713	-4.3738317	1.22E-05
2298	C2orf82	46.9197917	-0.6117534	0.30587077	-2.0000388	0.04549607
2302	C3	13870.2363	-0.4235314	0.07682361	-5.5130372	3.53E-08
2303	C3AR1	35.2269138	-2.5297517	0.40713147	-6.213599	5.18E-10
2306	C3orf17	1241.95764	0.49136921	0.08458849	5.80893707	6.29E-09
2307	C3orf18	604.144798	-0.5308355	0.09471072	-5.6048095	2.08E-08
2308	C3orf20	9.23040955	2.12224712	0.82009322	2.58781206	0.00965877
2334	C4orf26	178.651014	-1.6364221	0.17289613	-9.4647696	2.94E-21
2335	C4orf27	935.499422	-0.2168296	0.09806679	-2.2110398	0.02703308
2337	C4orf3	6434.32318	-0.2652529	0.09696168	-2.7356468	0.00622578
2338	C4orf32	1233.7417	0.89759769	0.07834244	11.4573624	2.16E-30
2339	C4orf33	255.217715	-1.1683961	0.16044966	-7.2820108	3.29E-13

2346	C5	409.580222	-0.9116884	0.11245434	-8.107187	5.18E-16
2349	C5orf15	4101.49357	-0.4728305	0.07714098	-6.1294324	8.82E-10
2351	C5orf22	1171.04678	-0.7672237	0.11438088	-6.7076224	1.98E-11
2352	C5orf24	2953.42426	-0.2193555	0.07084563	-3.096246	0.00195988
2355	C5orf34	665.518323	0.78914996	0.10141249	7.78158532	7.16E-15
2358	C5orf45	324.358973	-0.3665049	0.12863674	-2.8491461	0.00438367
2376	C6orf106	8327.07867	0.54397882	0.05323029	10.2193477	1.62E-24
2379	C6orf132	4006.21751	0.79351988	0.0681644	11.6412653	2.54E-31
2388	C6orf223	49.0099189	2.60835287	0.35648082	7.31695143	2.54E-13
2393	C6orf48	12712.0322	1.96376396	0.07323068	26.8161385	2.10E-158
2394	C6orf52	789.305052	0.77696749	0.10462791	7.42600582	1.12E-13
2401	C7orf13	630.884861	0.49778484	0.13851161	3.59381298	0.00032587
2408	C7orf49	2973.15937	0.23412124	0.07559752	3.09694343	0.00195527
2421	C7orf73	2732.02288	-0.2524215	0.08792447	-2.87089	0.00409318
2429	C8orf33	2898.7976	0.44582892	0.06075657	7.33795404	2.17E-13
2437	C8orf46	271.390056	0.79112261	0.13194579	5.99581553	2.02E-09
2441	C8orf59	1326.97971	-0.2323014	0.08714289	-2.6657525	0.00768162
2444	C8orf82	1134.86417	-1.2099057	0.09557172	-12.659662	9.89E-37
2463	C9orf16	2311.78507	-0.7467092	0.13765719	-5.4244109	5.81E-08
2471	C9orf3	2502.38715	-0.1480387	0.06793123	-2.1792434	0.02931359
2472	C9orf40	2209.7049	0.59290431	0.07909969	7.49565864	6.60E-14
2474	C9orf43	33.054334	1.46229699	0.38495295	3.79863819	0.00014549
2481	C9orf69	3818.28895	-0.4800163	0.08125842	-5.90728	3.48E-09
2484	C9orf84	118.573047	0.60909643	0.20055252	3.03709193	0.00238873
2485	C9orf85	315.262983	0.43356532	0.12384661	3.50082504	0.00046382
2486	C9orf89	2232.16841	-0.3425158	0.0866084	-3.9547645	7.66E-05
2488	C9orf91	725.426453	0.40527649	0.09769827	4.1482465	3.35E-05
2492	CA11	192.767932	-0.3388109	0.14767344	-2.2943255	0.02177181
2493	CA12	447.703802	-1.8843287	0.13829459	-13.625469	2.83E-42
2501	CA5B	466.108076	0.30903057	0.11608476	2.66211153	0.00776521
2502	CA5BP1	792.595775	0.88301211	0.08682551	10.1699621	2.70E-24
2506	CA9	230.3845	-0.3734272	0.18547311	-2.0133766	0.04407503
2507	CAAP1	1468.65422	-0.1782375	0.07809127	-2.2824259	0.02246421
2510	CABIN1	1664.36592	-0.5325003	0.09061719	-5.8763722	4.19E-09
2512	CABLES2	1357.34692	-0.6127374	0.08495439	-7.2125452	5.49E-13
2520	CACFD1	867.954304	-0.7196154	0.13602049	-5.2904927	1.22E-07
2538	CACNA2D1	340.041861	0.99729483	0.12848895	7.76171695	8.38E-15
2543	CACNB1	590.664886	0.7911465	0.10276153	7.6988585	1.37E-14
2545	CACNB3	146.287661	-0.6491145	0.19194166	-3.3818325	0.00072004
2552	CACNG6	20.9999468	-2.348147	0.56150527	-4.1818788	2.89E-05
2553	CACNG7	35.1834984	-1.8465567	0.37877006	-4.875139	1.09E-06
2554	CACNG8	13.1046997	-2.1252832	0.59794866	-3.5542904	0.000379
2556	CACTIN-AS1	58.0190582	-1.3405798	0.27188754	-4.9306407	8.20E-07

2559	CAD	5049.20353	0.1704043	0.06439772	2.64612302	0.00814202
2565	CADM4	349.170292	-0.2432293	0.11912606	-2.0417809	0.04117327
2567	CADPS2	291.610766	-0.3166815	0.12413125	-2.5511825	0.01073581
2570	CALB1	49.4747983	3.00139222	0.37036065	8.10397165	5.32E-16
2575	CALCOCO2	4721.15285	0.50609613	0.05661218	8.93970471	3.90E-19
2576	CALCR	32.6695635	-1.1836664	0.3896706	-3.0376076	0.00238464
2578	CALD1	9830.24216	-0.8524634	0.07022577	-12.138898	6.57E-34
2580	CALHM2	867.064334	-0.8259658	0.1008144	-8.192935	2.55E-16
2581	CALHM3	123.096217	-2.2909882	0.21189771	-10.811765	3.03E-27
2582	CALM1	12661.2807	-0.1137434	0.0569372	-1.997699	0.04574931
2584	CALM3	13863.1792	-0.8290944	0.05098463	-16.261653	1.85E-59
2591	CALR	51124.5767	-0.4507089	0.05343385	-8.4348957	3.31E-17
2593	CALU	27869.1663	-0.1786063	0.0869608	-2.0538718	0.0399881
2596	CAMK1D	793.081596	-0.3585026	0.10304293	-3.4791573	0.00050299
2600	CAMK2D	1406.13071	-0.2479892	0.08992332	-2.7577853	0.00581944
2601	CAMK2G	1588.37589	-1.603454	0.07474289	-21.45293	4.29E-102
2602	CAMK2N1	968.299802	0.67572182	0.09044186	7.47133928	7.94E-14
2603	CAMK2N2	843.723317	-0.3116726	0.11434612	-2.7256948	0.00641663
2605	CAMKK1	438.060047	0.45016343	0.11767953	3.82533339	0.0001306
2609	CAMLG	2012.4819	0.16181199	0.06793027	2.38203094	0.01721745
2611	CAMSAP1	3913.9503	1.04096831	0.06682871	15.5766633	1.05E-54
2612	CAMSAP2	3121.8447	0.67303711	0.11089664	6.06904872	1.29E-09
2619	CANT1	5022.04015	-0.373434	0.07249713	-5.1510174	2.59E-07
2621	CAP1	13929.7477	0.2627641	0.07495675	3.50554281	0.00045568
2624	CAPN1	10284.45	-0.5470087	0.05772768	-9.4756747	2.65E-21
2625	CAPN10	602.86747	-0.3786016	0.1127458	-3.358011	0.00078505
2634	CAPN5	933.009016	-1.229773	0.10561465	-11.643963	2.46E-31
2636	CAPN7	2436.59648	0.54969575	0.07315191	7.51444166	5.72E-14
2637	CAPN8	15.3669275	-3.596499	0.72175882	-4.9829651	6.26E-07
2639	CAPNS1	9541.00352	-0.9487472	0.06882464	-13.784993	3.14E-43
2641	CAPRIN1	13049.3585	0.24731228	0.0783213	3.15766291	0.00159039
2643	CAPS	100.024495	-1.3220972	0.21636789	-6.110413	9.94E-10
2650	CARD10	1646.18355	-0.5738579	0.08694111	-6.600536	4.10E-11
2651	CARD11	1048.33184	-1.1853066	0.08654515	-13.695818	1.08E-42
2652	CARD14	20.0023169	-1.4120798	0.52189161	-2.7056956	0.00681615
2657	CARD8	655.965847	-0.674746	0.09324385	-7.2363594	4.61E-13
2659	CARD9	108.947681	2.66465441	0.23474312	11.3513632	7.30E-30
2661	CARHSP1	3705.4142	-0.2030797	0.06394015	-3.1760906	0.00149274
2662	CARKD	1577.59397	0.41732105	0.08076305	5.16722772	2.38E-07
2663	CARM1	4888.69245	0.42616361	0.06377545	6.68225214	2.35E-11
2666	CARS	4824.4648	0.633378	0.05660502	11.1894316	4.59E-29
2682	CASC4	3716.30016	-0.2531288	0.07418567	-3.4120977	0.00064465
2685	CASC8	262.822542	-0.8164359	0.14030858	-5.8188596	5.93E-09

2692	CASP10	330.322364	-1.3109051	0.14668911	-8.9366215	4.01E-19
2698	CASP3	1999.46018	0.79505751	0.06772171	11.740068	7.94E-32
2699	CASP4	1280.71932	0.31968683	0.0843244	3.79115448	0.00014995
2701	CASP6	847.533672	-0.7258043	0.08807043	-8.2411805	1.71E-16
2712	CAT	1473.63708	-1.3922098	0.09690349	-14.366972	8.34E-47
2724	CAV1	36515.481	-0.3604091	0.06842592	-5.2671422	1.39E-07
2727	CBARP	271.141369	2.13369441	0.15699759	13.5906192	4.55E-42
2732	CBLB	810.607129	0.62995823	0.09826913	6.41054023	1.45E-10
2734	CBLL1	1622.01313	0.42341232	0.10328873	4.09930815	4.14E-05
2742	CBR4	621.23754	-0.9119087	0.09972368	-9.1443543	6.00E-20
2744	CBWD1	409.027946	0.57198734	0.12741392	4.489206	7.15E-06
2745	CBWD2	317.881279	0.80292338	0.13919055	5.76851934	8.00E-09
2747	CBWD5	160.150735	0.70407068	0.1739622	4.0472625	5.18E-05
2749	CBX1	5684.5219	0.68638609	0.0646956	10.6094704	2.69E-26
2753	CBX4	1247.00981	0.81036684	0.08068123	10.0440563	9.76E-24
2754	CBX5	3336.94374	-0.4130871	0.07638978	-5.4076225	6.39E-08
2756	CBX7	629.660192	-0.4471183	0.0922124	-4.848787	1.24E-06
2760	CC2D1A	1892.58452	-0.1564709	0.0760162	-2.0583894	0.03955277
2761	CC2D1B	3303.27989	0.4009719	0.06667271	6.01403378	1.81E-09
2762	CC2D2A	703.250258	-0.600237	0.09805635	-6.1213474	9.28E-10
2764	CCAR1	5744.6712	0.30581539	0.06248537	4.89419203	9.87E-07
2768	CCBE1	813.893325	-1.043208	0.09088914	-11.477808	1.71E-30
2769	CCBL1	680.820571	-0.5359683	0.10783169	-4.9704152	6.68E-07
2770	CCBL2	1861.46418	-0.403831	0.06763996	-5.9703017	2.37E-09
2772	CCDC102A	634.267688	-0.8355456	0.13285591	-6.2891116	3.19E-10
2776	CCDC106	663.981023	-0.9005427	0.116938	-7.7010272	1.35E-14
2777	CCDC107	826.754102	-0.9582207	0.1089924	-8.7916284	1.47E-18
2779	CCDC109B	1211.92089	-0.760849	0.08445592	-9.0088306	2.08E-19
2781	CCDC112	774.933715	0.8980164	0.0946404	9.4887213	2.34E-21
2783	CCDC114	47.3225447	-1.1656481	0.31793173	-3.6663473	0.00024604
2784	CCDC115	911.401545	-0.3745589	0.08329637	-4.4967014	6.90E-06
2788	CCDC120	751.056685	-0.5430924	0.11047502	-4.915975	8.83E-07
2789	CCDC121	36.1514796	-2.0666128	0.40916199	-5.0508427	4.40E-07
2790	CCDC122	195.187147	0.49497799	0.15494685	3.19450187	0.00140072
2792	CCDC125	439.389541	-0.5272418	0.11047171	-4.7726413	1.82E-06
2799	CCDC134	96.74706	-0.5298912	0.21097648	-2.5116128	0.01201809
2801	CCDC137	2184.13569	0.48605916	0.08448177	5.7534205	8.75E-09
2812	CCDC146	307.420959	-0.3692205	0.12685438	-2.9105854	0.00360752
2816	CCDC15	666.785321	0.40830608	0.1254504	3.2547213	0.00113504
2819	CCDC152	16.9295709	1.9432247	0.54911908	3.53880385	0.00040194
2821	CCDC154	74.6112228	1.06775974	0.25571667	4.17555776	2.97E-05
2825	CCDC159	67.8624241	-0.9578894	0.25428711	-3.7669602	0.00016525
2830	CCDC167	772.038267	-0.6831469	0.15107936	-4.5217752	6.13E-06

2832	CCDC169	20.7582793	0.96907289	0.44599063	2.17285479	0.02979125
2834	CCDC17	84.1309609	0.54802332	0.22340251	2.45307591	0.01416404
2839	CCDC174	766.964411	0.46288169	0.08941365	5.17685707	2.26E-07
2861	CCDC3	1197.32794	-0.7333472	0.07782233	-9.4233522	4.37E-21
2862	CCDC30	9.24834752	1.46499004	0.67341346	2.17546888	0.029595
2864	CCDC34	870.988517	-0.4939713	0.09314938	-5.3030014	1.14E-07
2873	CCDC43	1568.86535	0.28362992	0.06861393	4.13370757	3.57E-05
2875	CCDC50	8657.62332	0.57352499	0.05984989	9.58272392	9.45E-22
2876	CCDC51	967.780425	0.68894445	0.1018692	6.76302994	1.35E-11
2884	CCDC61	281.207146	-0.8751206	0.16514327	-5.2991598	1.16E-07
2885	CCDC62	28.9344173	1.01890774	0.38181756	2.66857221	0.00761744
2893	CCDC69	4700.61272	-0.6805872	0.07298513	-9.3250131	1.11E-20
2897	CCDC71L	1025.57665	-0.4014869	0.1009628	-3.9765828	6.99E-05
2901	CCDC77	495.115335	-0.376341	0.10118609	-3.7192958	0.00019978
2904	CCDC8	508.741182	-0.7406522	0.10426422	-7.1036085	1.22E-12
2905	CCDC80	3860.29209	-0.5149887	0.05955836	-8.6467906	5.30E-18
2913	CCDC86	3578.79852	0.56229404	0.05871274	9.57703568	9.99E-22
2916	CCDC88B	365.21681	-0.9510235	0.1578171	-6.0261117	1.68E-09
2917	CCDC88C	1447.98352	0.67396335	0.10138401	6.64762945	2.98E-11
2920	CCDC90B	1805.58319	0.42762028	0.07737923	5.52629275	3.27E-08
2923	CCDC93	2985.93625	0.3973261	0.0730333	5.44034183	5.32E-08
2924	CCDC94	785.742763	-0.2673445	0.1017476	-2.6275259	0.00860083
2926	CCDC97	984.911876	0.34258689	0.08606582	3.98052203	6.88E-05
2929	CCHCR1	1845.94955	-0.3479183	0.07711959	-4.5114121	6.44E-06
2945	CCL2	3444.16388	-9.6928804	0.28530919	-33.973249	5.53E-253
2946	CCL20	74.3869763	0.49920362	0.24500544	2.03752053	0.04159791
2954	CCL28	439.537372	-1.1587642	0.12121301	-9.5597348	1.18E-21
2967	CCNA2	4752.52117	-0.6067705	0.07323441	-8.2853198	1.18E-16
2968	CCNB1	8018.82263	-0.6508135	0.0686518	-9.4799191	2.54E-21
2969	CCNB1IP1	1334.74647	0.71252493	0.09346153	7.62372412	2.46E-14
2970	CCNB2	2038.31453	-0.9049617	0.07470428	-12.11392	8.91E-34
2974	CCND2	66.375265	3.8793116	0.37735788	10.2801923	8.66E-25
2978	CCNE1	806.883764	0.49430814	0.09399295	5.25899182	1.45E-07
2980	CCNF	1172.03621	-0.820534	0.08149813	-10.068134	7.64E-24
2982	CCNG2	1657.82453	-1.2814055	0.07024698	-18.241431	2.42E-74
2983	CCNH	2859.08052	1.06142768	0.07110849	14.9268761	2.20E-50
2984	CCNI	6088.15033	-0.2690948	0.09217915	-2.9192584	0.00350865
2988	CCNK	2502.50792	0.25778361	0.09243985	2.78866325	0.00529261
2992	CCNT1	1667.47888	0.63409637	0.08013706	7.91264779	2.52E-15
2993	CCNT2	1664.26405	0.47503261	0.08384145	5.66584472	1.46E-08
3000	CCR1	19.3248504	-2.5955536	0.5170678	-5.0197548	5.17E-07
3010	CCRL2	1994.03391	-1.1627052	0.06929055	-16.780142	3.41E-63
3011	CCS	1219.38952	-0.8108987	0.10539995	-7.6935394	1.43E-14

3015	CCT2	11266.0133	0.4153835	0.06660735	6.23630119	4.48E-10
3017	CCT4	10904.5994	0.3192144	0.06908948	4.6203038	3.83E-06
3019	CCT6A	16414.7686	0.63280952	0.05668303	11.164004	6.12E-29
3021	CCT6P1	149.958245	1.03748411	0.17396272	5.96383004	2.46E-09
3023	CCT7	17263.9924	0.52938828	0.0802533	6.59646784	4.21E-11
3024	CCT8	13556.0297	0.69869379	0.09591932	7.28418227	3.24E-13
3037	CD177	139.122458	0.7171097	0.1834127	3.90981495	9.24E-05
3038	CD180	22.5655313	-1.6428072	0.48708143	-3.3727568	0.0007442
3059	CD274	1206.62761	1.29775259	0.10452101	12.416189	2.13E-35
3060	CD276	2949.85757	-0.4272285	0.06382528	-6.693719	2.18E-11
3062	CD2AP	7717.02319	0.71215407	0.06788256	10.4909729	9.50E-26
3074	CD34	159.520696	-1.1840759	0.17267656	-6.8571896	7.02E-12
3080	CD3EAP	1511.51985	0.80306938	0.07708509	10.4179605	2.05E-25
3086	CD46	10078.0799	-0.3374122	0.07013495	-4.8108998	1.50E-06
3087	CD47	7777.35256	-0.1382054	0.06881724	-2.0082964	0.0446118
3092	CD55	13728.7573	0.61041028	0.05878231	10.3842512	2.92E-25
3093	CD58	2045.87028	-0.3618776	0.07756403	-4.6655342	3.08E-06
3094	CD59	18647.5767	-0.5620732	0.04866039	-11.550939	7.30E-31
3103	CD74	17343.8404	-1.1283484	0.06192538	-18.221099	3.51E-74
3107	CD81	10881.8227	-0.3456208	0.08874077	-3.8947243	9.83E-05
3115	CD9	8677.0228	-0.2377052	0.0639317	-3.7181113	0.00020072
3118	CD99	15525.7105	-0.2660328	0.10104726	-2.6327563	0.00846951
3119	CD99L2	3870.14973	-0.3670565	0.06918696	-5.3052848	1.12E-07
3121	CDA	4978.91703	1.00851399	0.06400853	15.7559301	6.25E-56
3123	CDAN1	589.775797	-0.8424087	0.10292669	-8.1845502	2.73E-16
3124	CDC123	5464.74636	0.41918625	0.06548194	6.40155476	1.54E-10
3125	CDC14A	373.644409	0.63440473	0.12741573	4.97901405	6.39E-07
3129	CDC20	5830.16012	0.48610378	0.06758417	7.19256885	6.36E-13
3132	CDC25A	2034.04777	1.18365939	0.06774807	17.4714857	2.36E-68
3133	CDC25B	7639.80596	-0.3003338	0.06864173	-4.3753827	1.21E-05
3134	CDC25C	689.647209	-0.7863238	0.09302842	-8.4525116	2.85E-17
3137	CDC34	5736.69976	0.91247236	0.09854467	9.25947921	2.05E-20
3141	CDC40	607.568863	-0.410527	0.11896957	-3.4506894	0.00055916
3145	CDC42BPG	345.393233	-1.3661676	0.13556455	-10.077617	6.94E-24
3146	CDC42EP1	4989.05809	0.38022254	0.06797099	5.59389422	2.22E-08
3147	CDC42EP2	5669.64984	0.9957112	0.07652895	13.0109092	1.06E-38
3148	CDC42EP3	9069.60569	0.41855804	0.07805939	5.3620459	8.23E-08
3154	CDC45	1221.45104	-0.4545195	0.08518515	-5.3356656	9.52E-08
3155	CDC5L	4065.62521	0.53439023	0.06592911	8.10552828	5.25E-16
3156	CDC6	5345.54325	0.89720579	0.05819429	15.4174195	1.25E-53
3159	CDCA2	1256.74383	-0.4878261	0.07764137	-6.2830691	3.32E-10
3160	CDCA3	1101.45652	-0.6950225	0.07967331	-8.7234044	2.70E-18
3161	CDCA4	2484.20125	0.23782737	0.07865956	3.02350225	0.00249867

3163	CDCA7	1122.90792	-0.5255107	0.0825966	-6.3623764	1.99E-10
3164	CDCA7L	4731.01928	-0.1834725	0.05975859	-3.0702282	0.00213895
3166	CDCP1	22149.6359	0.33172319	0.09338355	3.55226566	0.00038193
3168	CDH1	249.21943	-5.061942	0.25015989	-20.234827	4.83E-91
3178	CDH2	61.41161	0.69784129	0.26501572	2.63320718	0.00845827
3182	CDH24	933.746802	-0.3735709	0.08534176	-4.377352	1.20E-05
3185	CDH4	1477.10354	0.81171967	0.08169049	9.93652598	2.89E-23
3196	CDIP1	1500.12805	0.81803162	0.08184503	9.994885	1.60E-23
3199	CDK1	4703.58559	-0.6450634	0.06887605	-9.3655686	7.56E-21
3200	CDK10	1551.74423	-0.5897428	0.08474054	-6.9593941	3.42E-12
3206	CDK15	116.584512	0.98110774	0.18892357	5.19314625	2.07E-07
3208	CDK17	2286.71703	0.58364394	0.07607962	7.67148833	1.70E-14
3210	CDK19	802.962025	-0.5374844	0.10600922	-5.0701666	3.97E-07
3212	CDK20	199.682721	1.00149156	0.15948153	6.27967098	3.39E-10
3214	CDK2AP2	2105.50758	-0.185149	0.08446277	-2.1920787	0.02837383
3216	CDK4	8082.26269	0.46851188	0.0790762	5.92481512	3.13E-09
3217	CDK5	1064.15212	-1.2050146	0.12756318	-9.4464138	3.51E-21
3218	CDK5R1	1013.71229	1.47407418	0.085954	17.1495712	6.33E-66
3220	CDK5RAP1	1668.19034	-0.1529493	0.0754777	-2.0264169	0.04272208
3223	CDK6	3140.16703	1.55926149	0.08761412	17.7969195	7.47E-71
3224	CDK7	2342.4872	0.53346257	0.07469995	7.14140448	9.24E-13
3225	CDK8	529.438951	0.45080867	0.11105654	4.05927158	4.92E-05
3228	CDKL1	139.718937	0.59851524	0.17450571	3.42977451	0.00060408
3230	CDKL3	140.087364	0.57057251	0.20213768	2.82269256	0.00476222
3233	CDKN1A	3191.52824	1.84849481	0.06915434	26.7299882	2.11E-157
3235	CDKN1C	617.122163	-1.4107571	0.12555688	-11.236	2.71E-29
3238	CDKN2AIP	1707.09951	1.16208302	0.07106714	16.3519041	4.22E-60
3242	CDKN2C	1349.70242	-0.6411559	0.08218365	-7.8015019	6.12E-15
3247	CDON	1538.88936	-0.688717	0.10108598	-6.8131804	9.55E-12
3251	CDR2L	2635.41688	1.11210384	0.07104865	15.6527095	3.18E-55
3261	CDS2	2749.86305	-0.3573091	0.06240384	-5.7257552	1.03E-08
3264	CDV3	10224.715	0.72521665	0.05374695	13.4931676	1.72E-41
3272	CDYL	4259.68898	1.09499355	0.0611118	17.9178738	8.55E-72
3274	CEACAM1	43.5144639	-3.8574052	0.44740128	-8.6218018	6.59E-18
3288	CEBPA-AS1	2.12246345	3.36307224	1.67301485	2.01018673	0.04441143
3289	CEBPB	5860.33623	0.32574747	0.10234069	3.18297129	0.00145772
3291	CEBPD	1849.53474	-0.997941	0.09612567	-10.381628	3.01E-25
3293	CEBPG	3741.67328	1.86443144	0.06562275	28.4113593	1.46E-177
3294	CEBPZ	4098.90763	0.78490922	0.06717369	11.6847714	1.52E-31
3301	CECR6	68.1380293	-0.9053781	0.2479995	-3.6507255	0.0002615
3309	CELF1	6144.11628	0.46265712	0.05724108	8.08260579	6.34E-16
3314	CELF4	154.51059	1.14622753	0.18098181	6.33338518	2.40E-10
3318	CELSR1	873.514141	-1.5279968	0.09421428	-16.218314	3.74E-59

3319	CELSR2	1184.04339	-1.0064892	0.12277864	-8.1975919	2.45E-16
3322	CEMIP	5219.0134	-0.6540335	0.08840029	-7.3985443	1.38E-13
3325	CENPA	664.684801	-1.0678777	0.10741324	-9.9417697	2.74E-23
3326	CENPB	5882.75981	-0.4614256	0.08957988	-5.1509962	2.59E-07
3329	CENPC	583.19813	-0.6529616	0.11636305	-5.6114172	2.01E-08
3332	CENPH	691.841035	0.38805906	0.11323372	3.42706283	0.00061015
3335	CENPK	2018.40109	-0.1920916	0.07081308	-2.7126572	0.00667461
3337	CENPM	1131.01801	-0.6183284	0.12346176	-5.0082587	5.49E-07
3341	CENPQ	1604.60109	0.43105162	0.07851725	5.48989697	4.02E-08
3343	CENPU	3247.94964	-0.3951345	0.07186089	-5.4986029	3.83E-08
3347	CENPW	907.121792	-0.5426364	0.27559054	-1.9689949	0.04895367
3348	CEP104	1737.19902	1.05886387	0.08681043	12.1974261	3.21E-34
3353	CEP131	654.363587	-0.8152173	0.11818208	-6.8979773	5.27E-12
3362	CEP192	1498.15122	-0.433081	0.08536515	-5.0732763	3.91E-07
3364	CEP290	1263.06844	0.19431431	0.09097935	2.13580683	0.03269516
3369	CEP44	626.943806	-0.3735946	0.09607518	-3.888565	0.00010084
3370	CEP55	4376.87496	-1.1008645	0.07881634	-13.967466	2.46E-44
3374	CEP68	354.662573	-0.7255056	0.12017805	-6.0369224	1.57E-09
3375	CEP70	453.622407	-0.7927429	0.10827148	-7.3218069	2.45E-13
3376	CEP72	800.500665	0.24818347	0.08880799	2.79460731	0.00519628
3378	CEP78	916.748718	-0.2831615	0.08858448	-3.1965133	0.00139099
3379	CEP83	517.630691	0.55412093	0.10373827	5.34152875	9.22E-08
3381	CEP85	1305.70516	0.35449197	0.08932087	3.96874727	7.23E-05
3386	CEPT1	1869.21449	-0.1824881	0.07789946	-2.3426106	0.01914936
3402	CES2	1062.37744	-0.7490972	0.07908874	-9.4716046	2.76E-21
3403	CES3	45.2986096	-3.0257824	0.40997667	-7.380377	1.58E-13
3409	CETN3	1368.24013	-0.3538977	0.09706178	-3.6461074	0.00026624
3413	CFAP20	1205.25413	-0.1868306	0.0849125	-2.2002718	0.02778761
3417	CFAP44	478.588746	-0.3116834	0.10168689	-3.0651284	0.00217577
3434	CFB	6670.14857	-0.550348	0.05888754	-9.3457464	9.12E-21
3439	CFH	46.5474736	-3.8136071	0.4264369	-8.9429574	3.79E-19
3445	CFI	79.8406482	-0.441451	0.22346648	-1.9754686	0.04821499
3448	CFL2	5579.23452	0.40291331	0.05916496	6.80999874	9.76E-12
3451	CFP	331.893723	-1.8307918	0.13795376	-13.271054	3.41E-40
3462	CGNL1	192.901779	-1.6668839	0.17365864	-9.5986235	8.10E-22
3464	CGRRF1	883.389874	0.82816209	0.08860708	9.34645552	9.06E-21
3469	CHAC1	588.307748	2.71941639	0.11969328	22.719875	2.85E-114
3470	CHAC2	459.649591	1.02595983	0.10794278	9.50466338	2.01E-21
3477	CHCHD1	1673.32359	-0.4743614	0.12696365	-3.7361981	0.00018682
3480	CHCHD3	5565.99041	0.4752353	0.07112303	6.68187665	2.36E-11
3481	CHCHD4	1178.73019	0.66608055	0.08211913	8.1111493	5.01E-16
3483	CHCHD6	396.937745	-0.7773479	0.1143367	-6.7987607	1.06E-11
3485	CHD1	3258.28535	1.05194724	0.11907665	8.83420269	1.01E-18

3504	CHI3L2	13.9874355	-3.8138903	0.74833111	-5.0965279	3.46E-07
3507	CHIC1	830.474034	0.6353408	0.0997802	6.36740347	1.92E-10
3508	CHIC2	1640.37881	0.73361806	0.0907917	8.08023288	6.46E-16
3511	CHKA	3413.29299	-0.6243309	0.06637378	-9.4062887	5.14E-21
3518	CHML	1911.09373	1.06170376	0.09896996	10.7275355	7.56E-27
3519	CHMP1A	3163.91596	-0.1707584	0.07454462	-2.2906867	0.02198154
3520	CHMP1B	3254.27755	0.26040859	0.06865001	3.79327845	0.00014867
3522	CHMP2A	2972.52431	-0.4201822	0.11885144	-3.5353561	0.00040723
3523	CHMP2B	2682.69363	0.13471069	0.06727569	2.00236816	0.04524515
3525	CHMP4A	1637.4946	0.6269507	0.09818083	6.38567298	1.71E-10
3526	CHMP4B	8933.013	0.15949886	0.07272759	2.19309986	0.02830019
3527	CHMP4C	392.949458	0.97178753	0.13085975	7.42617594	1.12E-13
3535	CHORDC1	3011.97063	0.98104553	0.07357192	13.3345098	1.46E-40
3539	CHPF2	5731.74848	-0.1734308	0.07065498	-2.4546148	0.01410356
3543	CHRDL1	41.045036	-7.0446031	1.06830363	-6.5941956	4.28E-11
3558	CHRNA5	299.159502	-0.4529543	0.1262576	-3.587541	0.00033381
3574	CHST14	1056.69793	-1.1609801	0.09731956	-11.929566	8.30E-33
3575	CHST15	4542.63073	-0.8198804	0.07243607	-11.318676	1.06E-29
3577	CHST3	3269.52486	-0.3009858	0.07270637	-4.1397449	3.48E-05
3578	CHST4	79.0612831	-2.6295512	0.27105221	-9.7012716	2.98E-22
3581	CHST7	733.066662	-0.9111122	0.12900287	-7.0627285	1.63E-12
3585	CHSY3	10.3789104	-2.4219301	0.81036963	-2.9886733	0.00280192
3586	CHTF18	1339.2794	-0.3800733	0.09946679	-3.8211072	0.00013285
3588	CHTOP	4488.39345	0.78339717	0.07122262	10.9992747	3.85E-28
3592	CIAO1	4918.58026	0.5374504	0.06144373	8.74703382	2.19E-18
3593	CIAPIN1	2377.98627	0.55890361	0.09761059	5.72585028	1.03E-08
3595	CIB1	2329.02217	-0.472099	0.10577028	-4.4634377	8.07E-06
3606	CILP2	253.903074	1.49653536	0.14509686	10.3140439	6.09E-25
3607	CINP	873.941198	-0.3474883	0.09220511	-3.7686449	0.00016414
3608	CIPC	751.689176	0.37870419	0.08980189	4.21710707	2.47E-05
3609	CIR1	1363.71447	-0.7421689	0.08289015	-8.9536441	3.44E-19
3610	CIRBP	5540.45672	0.90035985	0.07997385	11.2581778	2.11E-29
3612	CIRH1A	2875.60149	0.53711875	0.06913827	7.76876195	7.93E-15
3618	CIT	2163.41852	-0.9498665	0.10656036	-8.9138824	4.93E-19
3620	CITED2	6525.3029	-0.2812525	0.05489295	-5.1236551	3.00E-07
3622	CIZ1	6180.67892	-0.3614889	0.05705394	-6.3359154	2.36E-10
3625	CKAP4	12930.7788	-0.142366	0.06073951	-2.3438777	0.01908443
3626	CKAP5	13065.0408	0.35682044	0.07659018	4.65882727	3.18E-06
3628	CKLF	663.954986	-0.6659923	0.10381777	-6.4150128	1.41E-10
3634	CKMT2-AS1	166.625291	0.38853961	0.17220995	2.25619722	0.02405828
3636	CKS2	2288.74269	-1.0709981	0.10912946	-9.8140147	9.80E-23
3639	CLASRP	1495.79423	-0.5093208	0.07276819	-6.9992239	2.57E-12
3645	CLCC1	1679.33134	-0.5126624	0.06818738	-7.5184345	5.54E-14

3648	CLCN2	369.818935	-0.4677158	0.11569051	-4.042819	5.28E-05
3649	CLCN3	3170.47425	-0.3822005	0.08841332	-4.3228841	1.54E-05
3651	CLCN5	404.995252	-0.9217905	0.11521097	-8.0008922	1.24E-15
3652	CLCN6	1054.96092	0.69999432	0.08956815	7.81521468	5.49E-15
3656	CLDN1	2292.98682	0.76358934	0.07906244	9.65805415	4.54E-22
3659	CLDN11	445.541435	-5.0989004	0.1872443	-27.231271	2.77E-163
3662	CLDN15	535.098845	1.08820696	0.10473725	10.3898752	2.76E-25
3667	CLDN2	203.353642	-1.6885975	0.16215806	-10.413281	2.16E-25
3670	CLDN23	668.05732	-0.3034737	0.10663736	-2.8458476	0.00442934
3673	CLDN3	195.002867	-1.2877568	0.16553978	-7.7791383	7.30E-15
3675	CLDN4	8721.92314	0.35731762	0.07884058	4.53215388	5.84E-06
3701	CLEC3B	39.4268894	-1.7908249	0.35388705	-5.060442	4.18E-07
3717	CLIC1	29932.6289	0.25582261	0.08560049	2.9885648	0.00280291
3718	CLIC2	321.137206	-0.3932082	0.12829159	-3.064957	0.00217701
3720	CLIC4	17294.5779	0.79441608	0.07063878	11.2461749	2.42E-29
3721	CLIC5	49.0903971	0.64657883	0.31831142	2.03127754	0.04222685
3722	CLIC6	2633.49818	0.91855894	0.06378979	14.399781	5.19E-47
3723	CLINT1	6001.61684	0.54724995	0.07271219	7.52624726	5.22E-14
3727	CLIP3	68.5380764	-1.2258857	0.26982694	-4.5432297	5.54E-06
3729	CLK1	3037.39587	0.1719803	0.0649664	2.64721911	0.00811568
3733	CLK4	1059.67034	0.33095324	0.08598221	3.84908956	0.00011856
3736	CLMN	603.823194	-0.4345146	0.09881262	-4.3973595	1.10E-05
3738	CLN3	1552.16003	-0.924226	0.09964639	-9.2750571	1.78E-20
3743	CLNS1A	4809.71417	0.46775634	0.07455463	6.27400758	3.52E-10
3753	CLPX	2170.1663	0.4144419	0.06366315	6.50991843	7.52E-11
3762	CLSTN3	502.036171	-0.6530824	0.10836858	-6.0264925	1.68E-09
3766	CLTCL1	504.351737	0.20837288	0.10286782	2.02563711	0.04280199
3767	CLU	5034.38307	-0.3566414	0.062858	-5.6737635	1.40E-08
3768	CLUAP1	662.039183	0.49490451	0.09264059	5.34219929	9.18E-08
3769	CLUH	6437.45098	0.56399516	0.06956343	8.10763825	5.16E-16
3779	CMAS	1965.30296	-0.455252	0.09088114	-5.0093119	5.46E-07
3780	CMBL	1658.84247	-0.5885035	0.10207538	-5.7653813	8.15E-09
3784	CMIP	3215.77079	-0.3079909	0.08114259	-3.7956748	0.00014724
3786	CMPK1	9281.25471	0.84021459	0.06387051	13.1549688	1.59E-39
3794	CMTM6	5334.74795	-0.5093892	0.06530586	-7.8000529	6.19E-15
3795	CMTM7	3155.52467	-0.2960589	0.07891159	-3.7517795	0.00017558
3796	CMTM8	316.611881	-0.3598093	0.12393791	-2.9031415	0.0036944
3798	CMTR2	1619.11679	1.45389326	0.0706311	20.5843218	3.79E-94
3802	CNBP	15577.7713	1.19452273	0.06197543	19.2741335	8.86E-83
3804	CNDP2	7505.13994	0.47277394	0.07316186	6.46202695	1.03E-10
3807	CNGA1	291.991624	-0.8085212	0.124593	-6.4892989	8.62E-11
3811	CNGB1	55.0246759	-1.3897088	0.32015529	-4.3407334	1.42E-05
3820	CNN1	6.71469755	5.1116932	1.37105086	3.72830311	0.00019277

3821	CNN2	8466.80048	-0.5029029	0.06687538	-7.5200008	5.48E-14
3822	CNN3	13348.6108	-0.3155795	0.05286012	-5.9700872	2.37E-09
3824	CNNM2	397.423335	-1.5278994	0.12084334	-12.643637	1.21E-36
3825	CNNM3	1410.18845	-0.4164199	0.0725918	-5.7364585	9.67E-09
3830	CNOT2	2208.08175	0.21537274	0.06545956	3.29016494	0.00100129
3833	CNOT6	2273.76498	-0.173838	0.08321552	-2.0890094	0.03670688
3842	CNPY4	659.742116	-0.7769323	0.12915281	-6.015605	1.79E-09
3846	CNST	1601.12224	0.76330414	0.07348901	10.3866431	2.85E-25
3861	CNTNAP1	837.335638	-1.2526742	0.09731432	-12.872456	6.43E-38
3870	COA1	1943.53961	-0.554728	0.06784305	-8.1766374	2.92E-16
3873	COA5	646.269362	-0.4408374	0.09265568	-4.7578025	1.96E-06
3875	COA7	1004.43292	1.08643809	0.09981374	10.8846548	1.36E-27
3877	COBL	67.0036722	-3.6053784	0.34405853	-10.478968	1.08E-25
3880	COG1	1109.63949	0.26991609	0.08197632	3.29261029	0.00099262
3882	COG3	1376.98655	0.77150239	0.08224068	9.38103164	6.53E-21
3883	COG4	1281.43225	-0.6102031	0.07614572	-8.0136236	1.11E-15
3884	COG5	2084.6491	0.25576979	0.09719316	2.63156158	0.00849935
3885	COG6	777.99922	-0.3519979	0.092238	-3.8161921	0.00013553
3886	COG7	428.052817	-0.9343092	0.13333099	-7.0074423	2.43E-12
3887	COG8	344.607066	-0.868618	0.15157627	-5.730567	1.00E-08
3893	COL13A1	3698.52555	-0.8977366	0.07768343	-11.556346	6.86E-31
3895	COL15A1	62.5310397	1.06910636	0.2551631	4.18989408	2.79E-05
3915	COL4A1	1852.07047	-1.1009113	0.09768098	-11.270477	1.84E-29
3916	COL4A2	6645.23038	-0.5174314	0.07285059	-7.1026385	1.22E-12
3918	COL4A3	21.3826235	-1.3520696	0.45467546	-2.9737026	0.0029423
3919	COL4A3BP	1682.71172	-0.5446741	0.0800325	-6.8056619	1.01E-11
3920	COL4A4	164.555145	-0.3940944	0.17995328	-2.1899819	0.02852555
3927	COL6A2	5722.891	0.27095092	0.05555053	4.87755741	1.07E-06
3933	COL7A1	8499.17406	0.23222119	0.10598459	2.19108453	0.02844568
3934	COL8A1	1511.71763	-0.4986102	0.11404646	-4.3719918	1.23E-05
3938	COL9A3	20.1580598	-1.6149243	0.45851805	-3.5220518	0.00042822
3948	COMMD10	1276.59332	-0.2491064	0.09023468	-2.7606499	0.00576865
3952	COMMD4	1696.73789	-0.7961569	0.12722608	-6.2578123	3.90E-10
3953	COMMD5	1098.6129	-0.4388933	0.10942805	-4.0107931	6.05E-05
3957	COMMD9	1004.13296	-0.5929612	0.08608641	-6.8879775	5.66E-12
3964	COPE	5178.03825	-0.1769196	0.07649877	-2.3127121	0.02073847
3965	COPG1	6079.55448	-0.2644484	0.05882454	-4.4955452	6.94E-06
3969	COPS2	3632.51944	0.4251041	0.0690413	6.15724342	7.40E-10
3970	COPS3	3819.37366	0.42093014	0.07532161	5.58843788	2.29E-08
3976	COPS8	2043.19194	-0.3072731	0.06429079	-4.7794268	1.76E-06
3977	COPZ1	4721.42398	0.18170515	0.06425221	2.82799838	0.004684
3979	COQ10A	452.404062	-0.3510169	0.12817635	-2.7385464	0.00617114
3980	COQ10B	1920.83683	0.20728698	0.07315574	2.83350256	0.00460409

3981	COQ2	1348.70806	-1.0146382	0.08582338	-11.822399	2.99E-32
3987	COQ9	1119.89081	-0.7222829	0.08000107	-9.0284163	1.74E-19
3990	CORO1B	5514.84874	-0.8826688	0.07186943	-12.281562	1.14E-34
3992	CORO2A	1144.5817	-0.5701866	0.07878074	-7.2376402	4.57E-13
3994	CORO6	43.3788693	-0.9082939	0.33400916	-2.7193682	0.00654067
4003	COX15	1221.14015	-0.5565955	0.07807438	-7.129041	1.01E-12
4006	COX18	1090.5177	-0.4164507	0.0911072	-4.5709964	4.85E-06
4007	COX19	748.011476	0.75659272	0.10017342	7.55282923	4.26E-14
4008	COX20	1145.25614	-0.9013846	0.09216305	-9.7803245	1.37E-22
4011	COX5A	2557.87266	-0.830385	0.0826771	-10.043712	9.79E-24
4015	COX6B1	4246.66206	-0.434363	0.08471492	-5.12735	2.94E-07
4023	COX7C	3993.15079	0.69297312	0.1016968	6.81410914	9.48E-12
4024	COX8A	6737.46678	-0.6417505	0.08038548	-7.9834133	1.42E-15
4030	CPA4	695.068686	0.36739691	0.09350851	3.92902125	8.53E-05
4031	CPA5	5.08169831	-3.2182095	1.2124818	-2.6542333	0.00794889
4037	CPD	14228.8953	-0.178536	0.07574828	-2.356965	0.01842499
4038	CPE	2104.38407	-0.3395729	0.08590926	-3.9526925	7.73E-05
4041	CPEB2	2557.74918	0.73769767	0.08712542	8.46707698	2.52E-17
4044	CPEB4	2669.84574	0.96289127	0.09871786	9.75397271	1.77E-22
4050	CPM	291.629219	-1.3966656	0.13963084	-10.002558	1.49E-23
4054	CPNE2	1362.51285	-0.7131882	0.07767421	-9.1817889	4.24E-20
4059	CPNE7	569.10674	0.66884904	0.11287319	5.92566814	3.11E-09
4065	CPQ	295.22311	-0.4557937	0.13702034	-3.3264676	0.00087954
4066	CPS1	105.507183	-2.0442608	0.24362683	-8.390951	4.82E-17
4069	CPSF2	5482.128	0.73114333	0.06577388	11.1160131	1.05E-28
4070	CPSF3	2461.8952	0.22049628	0.06624284	3.32860569	0.00087282
4075	CPSF7	4823.57096	0.39159724	0.08762752	4.46888405	7.86E-06
4076	CPT1A	2926.86217	0.54354575	0.06456764	8.41823771	3.82E-17
4081	CPVL	103.708936	-4.7432637	0.34800958	-13.629693	2.67E-42
4088	CR2	25.3712812	2.46294847	0.47790221	5.15366621	2.55E-07
4090	CRABP2	427.206407	1.17703385	0.12662951	9.29509919	1.47E-20
4091	CRACR2A	243.167694	0.36617993	0.13700748	2.67270021	0.00752435
4092	CRACR2B	613.188748	-0.5484434	0.12493286	-4.3899049	1.13E-05
4094	CRAMP1L	1152.74082	0.36033071	0.10841479	3.32363038	0.00088854
4095	CRAT	1907.12017	-0.4973128	0.06879438	-7.2289738	4.87E-13
4103	CRCP	2879.71304	0.78786079	0.06807014	11.5742492	5.57E-31
4108	CREB3L2	3776.81416	-0.2523982	0.07404023	-3.4089326	0.00065218
4109	CREB3L3	16.9139773	-3.8590122	0.7108323	-5.4288645	5.67E-08
4111	CREB5	80.278261	1.02048186	0.23536581	4.3357268	1.45E-05
4112	CREBBP	2569.15106	0.67498377	0.10280137	6.56590261	5.17E-11
4113	CREBL2	2526.62702	-0.1459716	0.06908432	-2.1129483	0.0346052
4116	CREG1	5245.2723	-0.137974	0.06702124	-2.0586605	0.03952677
4120	CREM	1363.48746	0.73894322	0.07709345	9.58503245	9.24E-22

4121	CRH	3.23712649	-3.3460554	1.44213038	-2.320217	0.02032914
4131	CRIPT	797.225601	-0.5664504	0.10245504	-5.5287702	3.22E-08
4136	CRISPLD2	820.373824	-1.9608755	0.09115101	-21.512384	1.19E-102
4144	CRNDE	540.61483	1.85422399	0.15454925	11.9976251	3.66E-33
4150	CROT	373.867531	-0.5550177	0.1156689	-4.7983311	1.60E-06
4154	CRTAM	78.0793356	-0.5831001	0.2392454	-2.4372467	0.01479958
4155	CRTAP	9118.83245	-0.5350231	0.05556191	-9.6293154	6.01E-22
4156	CRTC1	786.849378	-0.6115146	0.09612777	-6.3614767	2.00E-10
4161	CRY1	2978.39686	0.82127989	0.0635251	12.9284316	3.11E-38
4162	CRY2	467.217893	0.33095582	0.10326315	3.20497504	0.00135074
4179	CRYL1	441.608665	-0.69822	0.11431106	-6.108071	1.01E-09
4181	CRYM-AS1	17.632107	1.37267617	0.49306585	2.78396117	0.00536995
4183	CRYZL1	956.980735	0.63297123	0.09449895	6.69818255	2.11E-11
4193	CSF1	48402.0745	-1.2017571	0.0574725	-20.910122	4.33E-97
4195	CSF2	8952.63469	2.11762734	0.08049202	26.3085385	1.53E-152
4197	CSF2RB	6.05836202	2.23063504	0.93478394	2.38625734	0.01702084
4201	CSGALNACT2	2055.34422	0.35020388	0.06548994	5.34744577	8.92E-08
4215	CSNK1A1	7338.68395	0.31478462	0.05921983	5.3155273	1.06E-07
4223	CSNK1G3	2471.67195	0.39948308	0.07744911	5.15800756	2.50E-07
4225	CSNK2A2	1046.19913	0.64946805	0.08864203	7.32686373	2.36E-13
4227	CSNK2B	7397.30214	0.37194613	0.05543839	6.70918039	1.96E-11
4228	CSPG4	2306.46801	1.26817819	0.08227693	15.4135329	1.33E-53
4231	CSPP1	1022.06769	0.39263095	0.08244339	4.76243113	1.91E-06
4232	CSRNP1	1795.27218	1.93616633	0.07697482	25.1532437	1.30E-139
4233	CSRNP2	976.525755	0.92442903	0.08973335	10.3019562	6.90E-25
4237	CSRP2BP	984.611064	-0.3907755	0.08203777	-4.7633615	1.90E-06
4239	CST1	7616.11219	-1.8012074	0.07278027	-24.748569	3.21E-135
4242	CST2	481.633829	-2.2889182	0.14233865	-16.080791	3.48E-58
4243	CST3	13194.0423	-0.9257052	0.11041782	-8.3836576	5.13E-17
4244	CST4	994.266317	-1.3443396	0.10175876	-13.211046	7.58E-40
4247	CST7	1650.81943	-0.6869044	0.07412879	-9.2663645	1.93E-20
4253	CSTF1	1941.47773	-0.7000107	0.06506294	-10.758977	5.38E-27
4254	CSTF2	1538.95831	0.30920459	0.07324806	4.22133505	2.43E-05
4256	CSTF3	1849.99727	0.28174094	0.06874451	4.09837747	4.16E-05
4293	CTAGE5	2519.98825	0.31420521	0.07067137	4.44600437	8.75E-06
4302	CTBP1	7434.28216	-0.2521318	0.07425487	-3.3954918	0.00068505
4304	CTBP1-AS2	650.77058	-0.5617874	0.11035764	-5.0906075	3.57E-07
4305	CTBP2	3008.85713	0.5097173	0.06786874	7.51033956	5.90E-14
4306	CTBS	2355.86327	-0.614334	0.08224532	-7.4695318	8.05E-14
4320	CTDNEP1	5479.05452	0.25391453	0.05727614	4.43316372	9.29E-06
4322	CTDSP1	2717.99999	-0.1628705	0.07614817	-2.138863	0.03244677
4324	CTDSPL	3982.08275	-0.5354441	0.06153089	-8.7020374	3.26E-18
4327	CTGF	6225.19594	-0.2985444	0.05817494	-5.1318376	2.87E-07

4329	CTH	3044.59266	3.05618304	0.07203792	42.4246425	0
4330	CTHRC1	155.706754	-0.8222975	0.20142919	-4.0823154	4.46E-05
4331	CTIF	2333.61597	-0.1529224	0.06969388	-2.1942018	0.0282209
4339	CTNNBL1	3512.26525	0.67207275	0.08419129	7.98268724	1.43E-15
4343	CTPS1	3535.50983	0.4071671	0.07119676	5.71889931	1.07E-08
4344	CTPS2	742.308944	-0.3720258	0.09381831	-3.9653857	7.33E-05
4345	CTR9	2587.81475	-0.2649727	0.06796438	-3.8987004	9.67E-05
4350	CTSA	9017.8014	-0.796396	0.07246871	-10.989515	4.29E-28
4351	CTSB	21697.1996	-0.6604801	0.06011835	-10.986331	4.45E-28
4353	CTSD	132523.2	-0.3891035	0.06148337	-6.3285967	2.47E-10
4355	CTSF	2651.25422	-0.9781912	0.10846421	-9.0185619	1.91E-19
4357	CTSH	2374.97263	-1.4445524	0.09769973	-14.785634	1.81E-49
4358	CTSK	89.536322	-0.9091513	0.24298856	-3.7415395	0.0001829
4363	CTSO	337.186244	-1.1490215	0.13458706	-8.5373845	1.37E-17
4365	CTSV	268.902686	-1.9201699	0.16136495	-11.899548	1.19E-32
4367	CTSZ	15962.1926	-0.4336205	0.07767977	-5.5821548	2.38E-08
4370	CTTNBP2NL	1581.50736	0.38980247	0.10752229	3.6253178	0.00028861
4373	CTXN1	838.958721	1.47926039	0.17828951	8.29695715	1.07E-16
4378	CUEDC2	2087.0717	-0.9489699	0.075283	-12.605367	1.97E-36
4380	CUL2	3239.48619	0.31243703	0.07341156	4.25596515	2.08E-05
4382	CUL4A	5736.46687	0.49840219	0.05465552	9.11897323	7.58E-20
4392	CWC15	2739.82378	-0.1769555	0.08232755	-2.1494085	0.03160203
4395	CWC27	1371.78854	-0.2123934	0.07692646	-2.7609926	0.0057626
4399	CX3CL1	930.140203	-1.7725205	0.08906074	-19.902379	3.88E-88
4404	CXCL1	9014.16783	-1.1166724	0.06882209	-16.225493	3.33E-59
4410	CXCL16	645.91091	1.3665939	0.09720453	14.0589532	6.79E-45
4416	CXCL8	24657.064	0.34811957	0.05999196	5.80277038	6.52E-09
4422	CXCR4	349.065101	-1.4829112	0.12045931	-12.310473	7.96E-35
4425	CXXC1	1548.9498	-0.3231241	0.0710963	-4.5448786	5.50E-06
4446	CYB561A3	1635.29586	-0.6923976	0.07805327	-8.8708334	7.26E-19
4447	CYB561D1	488.717186	-0.5647693	0.1081791	-5.2206878	1.78E-07
4455	CYB5R3	6747.29073	-0.7026337	0.06959799	-10.095603	5.78E-24
4458	CYBA	3881.70803	-0.5119669	0.12078818	-4.2385511	2.25E-05
4460	CYBRD1	8194.24491	-0.5889521	0.08298417	-7.0971626	1.27E-12
4462	CYCS	8002.26668	0.8432206	0.06812944	12.3767437	3.49E-35
4464	CYFIP1	5047.91112	-0.7044648	0.0630303	-11.176604	5.31E-29
4470	CYLD	4672.48432	1.32184806	0.05887163	22.4530568	1.19E-111
4477	CYP1A1	324.456381	2.0480079	0.13342992	15.3489401	3.60E-53
4479	CYP1B1	10594.1686	-0.6288472	0.06466607	-9.7245303	2.37E-22
4481	CYP20A1	1442.69977	-0.4177821	0.07974292	-5.2391117	1.61E-07
4486	CYP26B1	462.144181	0.67147289	0.10985898	6.11213457	9.83E-10
4488	CYP27A1	405.906916	-1.4167156	0.13847128	-10.231115	1.44E-24
4507	CYP2S1	171.991172	-1.7679384	0.18070027	-9.7838171	1.32E-22

4508	CYP2U1	1792.06464	-0.9093823	0.07859744	-11.570126	5.84E-31
4513	CYP3A5	17.2741822	-5.7622368	1.1035049	-5.2217592	1.77E-07
4535	CYP51A1	3469.42189	-0.3582649	0.0725155	-4.9405292	7.79E-07
4545	CYSTM1	1774.94616	0.58327393	0.07587467	7.68733409	1.50E-14
4547	CYTH2	3818.30322	-0.5503707	0.08246921	-6.6736504	2.50E-11
4549	CYTH4	126.244625	-2.4817551	0.21122049	-11.749594	7.10E-32
4550	CYTIP	6.32869515	5.0288901	1.39677326	3.60036254	0.00031777
4554	D2HGDH	403.604004	-0.9948696	0.15276927	-6.512236	7.40E-11
4560	DAB2IP	3859.10572	-0.2242934	0.06451071	-3.4768395	0.00050736
4565	DACT3	16.3375341	3.19554331	0.67321818	4.74666817	2.07E-06
4568	DAG1	6559.17536	-0.977626	0.09776904	-9.9993417	1.53E-23
4570	DAGLB	2664.802	0.68221667	0.07009319	9.73299545	2.18E-22
4578	DAP	16744.8988	-0.4544936	0.0500392	-9.0827516	1.06E-19
4579	DAP3	6067.4936	0.65122865	0.07347561	8.86319441	7.78E-19
4580	DAPK1	18.8099912	-5.288807	0.92845715	-5.6963394	1.22E-08
4581	DAPK2	284.494791	-1.3946885	0.13160044	-10.597902	3.05E-26
4582	DAPK3	3015.73529	-0.4086237	0.09767323	-4.1835787	2.87E-05
4587	DARS2	1197.60659	-0.529521	0.09249962	-5.7245745	1.04E-08
4594	DAZAP1	7321.7689	0.2940095	0.06030111	4.87568929	1.08E-06
4595	DAZAP2	4208.86949	-0.4622686	0.07391017	-6.2544647	3.99E-10
4601	DBH-AS1	549.096369	-1.2269236	0.11470209	-10.696611	1.06E-26
4602	DBI	2722.91904	-0.8292654	0.07622182	-10.879632	1.44E-27
4605	DBN1	5102.16739	0.78462173	0.06684858	11.737298	8.21E-32
4606	DBNDD1	465.887254	-0.4888408	0.11702497	-4.1772353	2.95E-05
4609	DBP	536.748577	-0.3106443	0.13785985	-2.2533341	0.02423809
4619	DCAF13	4290.39982	1.28687416	0.07479517	17.2053109	2.42E-66
4621	DCAF15	1765.3197	-0.2902886	0.07366879	-3.9404557	8.13E-05
4624	DCAF4	904.550413	0.2193569	0.10021984	2.18875721	0.02861449
4628	DCAF6	3408.03111	-0.3581939	0.08028122	-4.46174	8.13E-06
4630	DCAF8	3572.60072	-0.6720741	0.06176524	-10.881104	1.42E-27
4633	DCAKD	642.434504	-0.5865855	0.10974422	-5.3450247	9.04E-08
4636	DCBLD2	13691.8862	0.38436343	0.05717536	6.72253663	1.79E-11
4644	DCHS1	36.0796043	-1.2886177	0.34789463	-3.704046	0.00021219
4648	DCLK2	365.031449	0.30417339	0.13661497	2.22650112	0.02598063
4651	DCLRE1B	1312.52851	0.16664404	0.07555728	2.20553256	0.02741675
4654	DCP1A	1776.55779	0.76199714	0.08435847	9.03284701	1.67E-19
4657	DCPS	962.558099	-0.6498303	0.08669431	-7.4956515	6.60E-14
4672	DCUN1D2	730.039129	0.87541175	0.0933092	9.3818379	6.48E-21
4673	DCUN1D3	411.801858	0.61157218	0.12372626	4.94294558	7.70E-07
4675	DCUN1D5	4021.98125	0.43330139	0.08608709	5.03329127	4.82E-07
4685	DDHD1	1506.63906	0.58351819	0.09793774	5.95805224	2.55E-09
4686	DDHD2	1670.16515	-0.3527514	0.07468524	-4.7231744	2.32E-06
4688	DDI2	132.193374	1.31094818	0.22942516	5.71405578	1.10E-08

4689	DDIAS	880.906124	0.39737127	0.1063988	3.73473457	0.00018791
4690	DDIT3	791.307023	1.16842964	0.09471893	12.3357563	5.81E-35
4691	DDIT4	7198.48468	-0.2475766	0.07509815	-3.2967076	0.00097825
4693	DDN	41.5141482	0.86280751	0.32534601	2.65196892	0.00800239
4697	DDR2	36.2093821	2.22851167	0.4157671	5.36	8.32E-08
4698	DDRGK1	3021.53512	-0.2648722	0.09409207	-2.8150326	0.00487723
4702	DDX10	3241.50262	0.67787861	0.06176441	10.9752301	5.03E-28
4703	DDX11	1047.92066	-0.2653427	0.0805865	-3.2926448	0.0009925
4711	DDX12P	275.258387	-0.3522516	0.13617437	-2.5867686	0.00968806
4712	DDX17	7259.90436	-0.2100343	0.07275482	-2.8868781	0.00389085
4713	DDX18	5693.86066	0.72852452	0.05438746	13.3950823	6.46E-41
4714	DDX19A	1131.12924	0.18504768	0.08602493	2.15109371	0.0314688
4717	DDX21	14394.5115	0.71872491	0.07074398	10.1595198	3.01E-24
4718	DDX23	2868.33567	-0.3904026	0.0714071	-5.4672801	4.57E-08
4719	DDX24	9503.58916	0.48370837	0.05253398	9.20753319	3.34E-20
4725	DDX31	1294.15071	1.06739785	0.07717295	13.8312441	1.65E-43
4731	DDX41	3004.45665	-0.2574865	0.06546006	-3.9334905	8.37E-05
4735	DDX47	4811.16496	0.63479307	0.06927499	9.16338018	5.03E-20
4739	DDX51	1015.6595	0.33199307	0.10197109	3.25575695	0.00113091
4740	DDX52	2720.81559	0.27169258	0.06108517	4.44776668	8.68E-06
4741	DDX53	21.2120111	-2.7422238	0.54261043	-5.0537617	4.33E-07
4742	DDX54	5176.30156	-0.5140445	0.07033292	-7.3087327	2.70E-13
4744	DDX56	3823.05206	0.17909868	0.07093641	2.52477789	0.01157714
4747	DDX6	6077.81745	0.41390392	0.07928041	5.22075886	1.78E-07
4749	DDX60L	3027.28666	1.13431896	0.0710421	15.9668567	2.17E-57
4750	DEAF1	1632.81071	-0.3124468	0.0723469	-4.3187316	1.57E-05
4753	DECR1	1859.69634	-0.2837862	0.08435753	-3.364088	0.00076797
4754	DECR2	421.232936	-0.9786733	0.11281299	-8.6751826	4.13E-18
4756	DEDD2	1322.46138	-0.2329137	0.07896566	-2.9495567	0.0031823
4757	DEF6	303.383584	-1.7566819	0.1837374	-9.5608291	1.17E-21
4811	DEGS1	8133.28324	-0.3676885	0.05963638	-6.1655068	7.03E-10
4813	DEK	30211.7522	0.92167906	0.05172455	17.8189868	5.03E-71
4814	DENND1A	2056.79616	0.22771818	0.0704673	3.23154385	0.00123123
4815	DENND1B	617.509608	0.52988313	0.11806984	4.48787858	7.19E-06
4818	DENND2C	157.179275	0.90206266	0.18408368	4.90028594	9.57E-07
4820	DENND3	1157.88413	-0.8983773	0.08300629	-10.823002	2.68E-27
4827	DENND6A	1907.42819	-0.3890932	0.08044572	-4.8367171	1.32E-06
4828	DENND6B	94.3390457	-1.9803676	0.23334434	-8.4868896	2.12E-17
4830	DEPDC1	2469.49012	0.26344412	0.08956748	2.94129192	0.00326846
4832	DEPDC1B	944.621262	-0.505931	0.10081937	-5.0181928	5.22E-07
4834	DEPDC5	242.711641	-0.6838538	0.15073851	-4.5366893	5.71E-06
4842	DESI1	1783.21459	0.45605856	0.07066331	6.45396513	1.09E-10
4843	DESI2	3199.54751	0.23635363	0.06290457	3.75733667	0.00017173

4846	DFFA	1747.87691	1.03807999	0.07259498	14.2996122	2.20E-46
4849	DFNB31	445.374869	-0.8393935	0.11425003	-7.3469872	2.03E-13
4851	DGAT1	1989.05529	-0.2129892	0.0904902	-2.3537263	0.01858629
4856	DGCR14	501.239446	-0.725131	0.10642105	-6.8137928	9.51E-12
4857	DGCR2	4179.11796	-0.2874931	0.066504	-4.3229452	1.54E-05
4863	DGKA	473.084729	0.50780845	0.11407964	4.45135029	8.53E-06
4865	DGKD	2604.89133	0.70242417	0.06902652	10.1761489	2.53E-24
4869	DGKI	198.328906	1.62717326	0.19702817	8.25858167	1.47E-16
4871	DGKQ	1044.65309	-0.4770255	0.11333379	-4.2090319	2.56E-05
4875	DHCR24	4538.14152	-0.2989799	0.07201802	-4.1514596	3.30E-05
4876	DHCR7	4054.61468	-0.5935432	0.07153767	-8.2969312	1.07E-16
4877	DHDDS	1329.29091	0.52784112	0.07277158	7.25339659	4.06E-13
4879	DHFR	1159.14582	-0.4233858	0.09759612	-4.3381416	1.44E-05
4882	DHODH	366.655056	0.42299328	0.11848506	3.57001373	0.00035696
4883	DHPS	2482.74825	-0.3030571	0.06940624	-4.3664239	1.26E-05
4884	DHRS1	795.559032	-0.9598483	0.10653314	-9.0098567	2.06E-19
4886	DHRS12	211.318535	-1.3895473	0.16160407	-8.5984674	8.08E-18
4887	DHRS13	917.084028	-0.3290218	0.12116996	-2.715374	0.0066201
4889	DHRS3	1210.97217	-0.7502243	0.08884847	-8.4438625	3.07E-17
4890	DHRS4	176.744485	-0.5800916	0.17156454	-3.381186	0.00072174
4893	DHRS4L2	92.1694844	-0.7221519	0.24230983	-2.9802829	0.00287982
4894	DHRS7	1028.91952	-1.1217205	0.08724821	-12.856659	7.89E-38
4898	DHRSX	1560.0113	-0.4170576	0.09042993	-4.6119419	3.99E-06
4899	DHTKD1	1081.20153	-0.6031092	0.08076163	-7.4677689	8.16E-14
4904	DHX32	3275.14732	-0.7058261	0.06533709	-10.802839	3.34E-27
4905	DHX33	2738.85026	1.13726915	0.08342159	13.6327914	2.56E-42
4906	DHX34	1838.94296	0.54686201	0.07813559	6.99888489	2.58E-12
4908	DHX36	2448.04559	0.83524243	0.06956816	12.0061026	3.30E-33
4909	DHX37	1534.74091	0.5012719	0.07665317	6.53948025	6.17E-11
4911	DHX40	1709.2343	-0.388978	0.0999629	-3.8912232	9.97E-05
4912	DHX57	879.208547	-0.4557549	0.10885755	-4.1867093	2.83E-05
4916	DIABLO	2538.28258	0.35278589	0.0868338	4.06277137	4.85E-05
4917	DIAPH1	11396.4824	0.271726	0.06240249	4.35440934	1.33E-05
4918	DIAPH2	831.801496	0.45521112	0.11732799	3.87981689	0.00010454
4926	DIEXF	2984.90364	0.62237353	0.07054656	8.82216669	1.12E-18
4927	DIMT1	1431.5878	0.40257561	0.09580559	4.2020053	2.65E-05
4935	DIP2B	1841.6697	-0.4233882	0.1126029	-3.7600118	0.00016991
4936	DIP2C	1367.292	-0.5202221	0.09172905	-5.6712905	1.42E-08
4943	DIS3	3184.01543	0.70908337	0.09166058	7.73596875	1.03E-14
4945	DIS3L2	691.917835	-0.4777231	0.09530922	-5.0123492	5.38E-07
4952	DIXDC1	206.989443	-1.103947	0.15636343	-7.0601355	1.66E-12
4953	DKC1	10307.4361	1.39096031	0.07343084	18.9424539	5.10E-80
4971	DLC1	5034.49063	0.76636498	0.06661953	11.5036077	1.27E-30

4974	DLEU1	587.083618	0.51840504	0.09953912	5.20805349	1.91E-07
4983	DLG3	1118.27396	-0.3324173	0.07989569	-4.1606406	3.17E-05
5004	DLL4	3.93251942	2.70662462	1.21019126	2.23652633	0.02531731
5005	DLST	4879.18327	0.44703424	0.06824832	6.55011338	5.75E-11
5010	DLX3	13.0618429	1.2183012	0.57379945	2.12321781	0.0337356
5022	DMKN	80.0272879	0.80975232	0.23328438	3.47109526	0.00051834
5035	DMTN	874.799717	-0.7542381	0.0935509	-8.0623289	7.49E-16
5036	DMWD	1714.89251	0.66475889	0.09195108	7.22948425	4.85E-13
5038	DMXL2	1166.14137	-0.7404882	0.11914312	-6.2151151	5.13E-10
5039	DNA2	932.492134	-0.5939313	0.10308938	-5.7613236	8.35E-09
5041	DNAAF2	1183.7014	1.04594559	0.09014441	11.6030002	3.98E-31
5042	DNAAF3	130.838392	-0.4278624	0.18481391	-2.3150988	0.02060753
5043	DNAAF5	3611.38133	-0.4117728	0.0729965	-5.640993	1.69E-08
5048	DNAH14	611.620909	0.58293076	0.11178169	5.21490361	1.84E-07
5051	DNAH2	5131.94234	-0.9910008	0.07211108	-13.742698	5.63E-43
5072	DNAJB4	1132.77715	0.96368434	0.09108486	10.5800715	3.69E-26
5079	DNAJB9	1680.95153	0.69613844	0.07641165	9.11037059	8.21E-20
5086	DNAJC15	1245.37967	-0.2639131	0.07519032	-3.5099342	0.00044822
5087	DNAJC16	1106.00491	0.44208671	0.08645888	5.11325987	3.17E-07
5089	DNAJC18	605.107333	0.28573612	0.12832902	2.22659008	0.02597468
5093	DNAJC22	66.2893509	-2.010779	0.26534359	-7.5780201	3.51E-14
5103	DNAJC4	491.35351	-0.5754784	0.10552483	-5.4534886	4.94E-08
5107	DNAJC6	629.181559	0.85533397	0.11048923	7.74133326	9.84E-15
5109	DNAJC8	5601.18053	0.6957868	0.05827838	11.9390201	7.41E-33
5116	DNASE1L1	1133.39421	-1.1095681	0.08035269	-13.808724	2.26E-43
5119	DNASE2	1889.23639	-0.5094923	0.06708761	-7.5944312	3.09E-14
5123	DNHD1	1425.30694	0.58235732	0.08339548	6.98307978	2.89E-12
5125	DNM1	869.251816	-0.8153049	0.08961976	-9.0973792	9.25E-20
5130	DNM2	7692.36907	-0.4377266	0.07841138	-5.5824371	2.37E-08
5131	DNM3	327.289121	-2.0907124	0.15054372	-13.887742	7.52E-44
5137	DNMT3A	337.052612	-0.5450153	0.1329389	-4.0997429	4.14E-05
5144	DNTTIP2	5856.93218	0.36131957	0.06931224	5.21292561	1.86E-07
5145	DOC2A	129.596773	-0.9251993	0.19066575	-4.8524671	1.22E-06
5148	DOCK1	1939.03929	-0.5651359	0.0837469	-6.7481409	1.50E-11
5149	DOCK10	241.486274	-2.8638967	0.18031852	-15.882432	8.39E-57
5156	DOCK6	1110.22948	0.70799375	0.10022994	7.06369548	1.62E-12
5157	DOCK7	3193.84853	0.69838902	0.09826486	7.10720985	1.18E-12
5162	DOHH	1524.74041	0.59499019	0.12754499	4.66494353	3.09E-06
5163	DOK1	1189.72887	0.97411825	0.12618152	7.71997554	1.16E-14
5166	DOK4	365.001531	-0.6503258	0.13041131	-4.9867291	6.14E-07
5170	DOLK	1208.43041	-0.3181102	0.09753518	-3.2614917	0.00110828
5173	DOPEY1	425.704426	0.26485804	0.12456455	2.12627136	0.03348067
5186	DPH2	1759.7859	1.053162	0.09497593	11.0887257	1.42E-28

5187	DPH3	2207.66694	0.36331937	0.06578002	5.52324823	3.33E-08
5189	DPH5	1136.56707	0.27128719	0.07712704	3.51740718	0.00043578
5190	DPH6	149.028235	-0.671065	0.18002806	-3.727558	0.00019334
5193	DPM1	2515.39529	0.26380606	0.06706485	3.93359641	8.37E-05
5195	DPM3	797.579801	-0.5951088	0.29994104	-1.984086	0.04724625
5200	DPP4	433.878354	-0.3659533	0.13524404	-2.7058736	0.0068125
5204	DPP9	6475.74255	-0.178579	0.06718905	-2.6578587	0.00786389
5223	DPY30	1286.61469	-0.1890851	0.08951632	-2.1122978	0.03466091
5224	DPYD	2522.42475	-0.2940632	0.07060793	-4.1647338	3.12E-05
5228	DPYSL2	5131.54099	-0.8833308	0.06601713	-13.380327	7.88E-41
5236	DRAM2	1291.60821	-0.4653597	0.0770005	-6.0435923	1.51E-09
5237	DRAP1	18287.2917	1.30563527	0.10826657	12.0594495	1.73E-33
5254	DSC2	18.0318795	-4.0112657	0.6986721	-5.7412708	9.40E-09
5261	DSCC1	762.591506	-0.3308924	0.08969959	-3.6888953	0.00022523
5263	DSCR3	3105.1255	0.29982063	0.06380403	4.69908648	2.61E-06
5278	DST	16945.6774	0.68607601	0.09284602	7.38939625	1.47E-13
5288	DTNBP1	1442.06673	0.82146801	0.09113327	9.01391977	1.99E-19
5289	DTWD1	428.530264	0.27526992	0.11831457	2.32659363	0.0199869
5290	DTWD2	209.135346	-0.6202353	0.16405247	-3.7807128	0.00015638
5292	DTX2	1989.20304	-0.4619922	0.08204722	-5.6308094	1.79E-08
5298	DUOX1	36.137712	-3.2892825	0.45786852	-7.183902	6.77E-13
5303	DUS1L	3744.58973	0.3070644	0.07574481	4.05393305	5.04E-05
5306	DUS4L	572.859231	0.85992226	0.10782888	7.97487881	1.53E-15
5307	DUSP1	1815.97541	1.12299339	0.07964785	14.0994812	3.83E-45
5310	DUSP12	1896.27088	0.48533575	0.0777619	6.24130529	4.34E-10
5312	DUSP14	2371.07266	0.54275955	0.06577412	8.25187153	1.56E-16
5314	DUSP16	793.964948	0.35647113	0.09029647	3.94778601	7.89E-05
5316	DUSP19	19.4649536	-2.2253443	0.53326189	-4.1730796	3.01E-05
5317	DUSP2	69.1981781	1.95392216	0.26775714	7.29736721	2.93E-13
5319	DUSP22	1126.06567	0.42087296	0.0911682	4.61644477	3.90E-06
5324	DUSP3	4421.34128	-0.8537559	0.06217595	-13.731288	6.60E-43
5326	DUSP5	6110.95019	0.9262566	0.06089161	15.2115644	2.96E-52
5328	DUSP6	3207.40791	0.21774929	0.05916846	3.68015779	0.00023309
5330	DUSP8	449.123733	0.71305669	0.13122305	5.43392864	5.51E-08
5337	DVL1	8619.24141	0.78778763	0.06410238	12.2895221	1.03E-34
5339	DVL3	2768.90434	-0.2019924	0.10141289	-1.9917823	0.04639495
5340	DXO	1122.8389	0.29232324	0.09569096	3.05486771	0.0022516
5345	DYNC1H1	26614.9822	0.58319829	0.07796521	7.48023776	7.42E-14
5348	DYNC1LI1	3786.08664	0.67990057	0.06813217	9.97914187	1.88E-23
5358	DYRK1A	2068.79457	0.78315245	0.08012327	9.77434487	1.45E-22
5359	DYRK1B	631.823987	-1.4584102	0.09827085	-14.84072	7.99E-50
5360	DYRK2	683.701093	0.55176523	0.09714455	5.67983713	1.35E-08
5363	DYSF	2215.42023	-1.8307962	0.07447962	-24.58117	2.01E-133

5371	E2F1	4609.49998	-0.138339	0.06845406	-2.0209021	0.0432899
5373	E2F3	3128.85085	0.83996696	0.07493354	11.209492	3.66E-29
5374	E2F4	2622.71233	0.3716123	0.06980568	5.32352535	1.02E-07
5375	E2F5	1488.37956	0.92009057	0.07190557	12.7958173	1.73E-37
5376	E2F6	611.808231	0.74756864	0.10074654	7.42029084	1.17E-13
5377	E2F7	4992.10352	0.88210559	0.07968239	11.0702695	1.75E-28
5378	E2F8	808.568628	-0.5791206	0.10798202	-5.3631203	8.18E-08
5380	EAF1	1482.31055	0.95074212	0.07751829	12.2647453	1.40E-34
5389	EBI3	303.040966	-1.8790306	0.13005514	-14.447954	2.58E-47
5390	EBLN1	7.74634564	-2.9333822	0.87181856	-3.3646705	0.00076635
5392	EBLN3	3398.40163	0.56411283	0.0893192	6.31569476	2.69E-10
5393	EBNA1BP2	7497.15397	1.176301	0.07841561	15.0008524	7.25E-51
5394	EBP	3229.12076	-0.3386851	0.10736178	-3.1546151	0.0016071
5395	EBPL	929.674182	-0.5358486	0.13904583	-3.8537553	0.00011632
5401	ECH1	2638.43548	-0.7326556	0.0830649	-8.8202793	1.14E-18
5404	ECHDC3	1316.36826	-0.2402331	0.1028901	-2.3348512	0.0195512
5405	ECHS1	5677.57482	-0.4585792	0.08502913	-5.3932012	6.92E-08
5407	ECI2	3853.56593	-0.2770555	0.06292227	-4.4031396	1.07E-05
5409	ECM2	9.80207525	1.71284971	0.68468722	2.50165282	0.01236151
5412	ECSIT	1364.77399	-0.243227	0.08343882	-2.9150341	0.0035565
5420	EDC4	2348.00562	-0.3199734	0.07907191	-4.0466131	5.20E-05
5423	EDEM1	4544.07329	-0.4991321	0.07088258	-7.0416754	1.90E-12
5427	EDIL3	6315.05354	-0.2629552	0.09009852	-2.9185292	0.00351687
5428	EDN1	1422.49219	0.8835714	0.07589589	11.6418876	2.52E-31
5429	EDN2	189.060024	-3.1597032	0.19828442	-15.935207	3.61E-57
5434	EDRF1	696.634272	-0.5986065	0.1182962	-5.0602341	4.19E-07
5439	EEF1A2	6144.0258	1.16869649	0.11987972	9.74890867	1.86E-22
5440	EEF1B2	2693.97662	0.50454054	0.0813667	6.20082317	5.62E-10
5445	EEF1G	17963.1741	0.23837724	0.07077576	3.36806315	0.00075698
5450	EEPD1	267.359709	-0.92559	0.13679907	-6.7660551	1.32E-11
5466	EFEMP1	2928.71852	-1.9948678	0.07742638	-25.764703	2.21E-146
5467	EFEMP2	413.055929	-1.4870658	0.12677533	-11.729931	8.95E-32
5469	EFHC1	561.194866	0.24330961	0.10263995	2.37051561	0.01776329
5471	EFHD1	395.532807	-0.5282868	0.10966317	-4.8173584	1.45E-06
5477	EFNA5	1239.68608	-0.5004944	0.09584435	-5.2219504	1.77E-07
5479	EFNB2	2382.35598	0.92853043	0.06979104	13.3044352	2.18E-40
5480	EFNB3	113.081292	-0.7013244	0.22596562	-3.1036773	0.00191132
5487	EGF	147.435531	-1.3817771	0.18765357	-7.3634472	1.79E-13
5495	EGFR	6448.86901	1.27872078	0.22084831	5.79004113	7.04E-09
5497	EGLN1	5115.09439	-0.1982277	0.05936296	-3.3392498	0.00084005
5501	EGR1	397.30839	0.78569809	0.13663858	5.75019213	8.91E-09
5503	EGR3	7.13502539	2.85349829	0.94135512	3.03126655	0.0024353
5508	EHD2	10129.3658	-0.4109995	0.06332571	-6.4902479	8.57E-11

5510	EHD4	3880.85768	0.5033134	0.06374578	7.89563437	2.89E-15
5517	EHMT2	2878.91668	-0.62793	0.06684533	-9.393775	5.79E-21
5518	EI24	7030.97595	-0.3671421	0.05433495	-6.7570158	1.41E-11
5519	EID1	5751.59321	-0.2079729	0.06426212	-3.2363225	0.0012108
5523	EIF1	26030.3676	1.25460748	0.0630304	19.9047999	3.70E-88
5528	EIF1B	1338.66795	0.37206601	0.09008353	4.1302335	3.62E-05
5530	EIF2A	4026.65933	0.55035229	0.08193238	6.71715227	1.85E-11
5531	EIF2AK1	6071.04592	-0.5512191	0.07676584	-7.1805255	6.94E-13
5536	EIF2B2	2115.63624	0.38826142	0.06920549	5.61026954	2.02E-08
5537	EIF2B3	1614.757	0.79675866	0.10241333	7.77983368	7.26E-15
5538	EIF2B4	1404.82241	0.28254428	0.07186756	3.9314577	8.44E-05
5542	EIF2S1	7391.14989	0.25067729	0.05728004	4.37634599	1.21E-05
5544	EIF2S3	9670.39921	0.37234055	0.0574025	6.48648708	8.79E-11
5549	EIF3D	6851.3641	0.12172176	0.05483305	2.21986105	0.0264282
5550	EIF3E	14934.3793	0.46902026	0.05662638	8.28271668	1.20E-16
5552	EIF3G	4357.94893	-0.4602031	0.05950603	-7.7337228	1.04E-14
5553	EIF3H	9130.46991	0.28768806	0.06882777	4.17982543	2.92E-05
5554	EIF3I	8607.49756	0.83266695	0.05925287	14.0527696	7.41E-45
5556	EIF3J	3477.65341	0.79448379	0.07129857	11.1430538	7.74E-29
5559	EIF3L	5286.67914	-0.4124948	0.07193915	-5.7339404	9.81E-09
5565	EIF4E	997.74581	0.58987106	0.0983366	5.99848929	1.99E-09
5569	EIF4EBP1	3190.96992	0.84061326	0.06773562	12.4102091	2.30E-35
5570	EIF4EBP2	3690.6488	-0.7632695	0.07247495	-10.531494	6.18E-26
5574	EIF4G2	29513.7824	0.24967506	0.05633472	4.43199228	9.34E-06
5578	EIF5A	16999.6708	0.33680362	0.08957861	3.75986675	0.00017
5579	EIF5A2	1348.32382	0.65364191	0.09381342	6.96746675	3.23E-12
5580	EIF5AL1	626.297857	0.40216874	0.12443089	3.23206521	0.00122899
5581	EIF5B	16614.5405	0.5920597	0.06452924	9.17506044	4.51E-20
5582	EIF6	12595.8062	0.68801331	0.06262657	10.9859659	4.46E-28
5584	ELAC2	4980.17149	0.49669826	0.05995131	8.28502754	1.18E-16
5591	ELF1	2026.60993	0.72167031	0.08629437	8.36288967	6.12E-17
5593	ELF3	1279.79458	-0.5558892	0.07711189	-7.2088657	5.64E-13
5594	ELF4	2616.33175	0.35746099	0.09599879	3.7235989	0.0001964
5598	ELFN2	1076.80764	-0.4294003	0.08580081	-5.0046181	5.60E-07
5601	ELK3	8728.35663	0.40322283	0.06173672	6.53132861	6.52E-11
5603	ELL	1169.84934	0.50395438	0.08024914	6.27987245	3.39E-10
5604	ELL2	3132.72162	0.36860829	0.0812029	4.53934899	5.64E-06
5608	ELMO2	2824.68468	0.4638226	0.07119944	6.51441397	7.30E-11
5612	ELMOD3	412.583608	-0.6050102	0.10916485	-5.5421706	2.99E-08
5615	ELOF1	1696.10693	0.86268146	0.0946466	9.11476423	7.88E-20
5616	ELOVL1	4089.79002	0.1698955	0.05936869	2.86170221	0.00421373
5619	ELOVL3	14.3430482	-3.4074064	0.70099083	-4.860843	1.17E-06
5623	ELOVL7	956.857298	0.58565512	0.11328137	5.16991537	2.34E-07

5624	ELP2	1933.10355	0.67497345	0.06585939	10.2487052	1.20E-24
5625	ELP3	1202.60064	0.23368049	0.07824165	2.98665091	0.00282052
5626	ELP4	613.726209	-0.6825598	0.10606395	-6.4353613	1.23E-10
5630	EMB	208.172287	-6.1410524	0.36884534	-16.649397	3.06E-62
5632	EMC1	6141.87528	0.82349555	0.06097795	13.5048084	1.46E-41
5633	EMC10	2450.60858	-1.5673906	0.07879053	-19.893134	4.67E-88
5635	EMC3	1510.55539	-0.3909689	0.08380616	-4.6651571	3.08E-06
5636	EMC3-AS1	570.87146	0.97517225	0.12651078	7.70821448	1.28E-14
5637	EMC4	2252.67875	-0.601806	0.07494786	-8.029663	9.77E-16
5638	EMC6	1535.68954	-0.3056842	0.07582807	-4.0312798	5.55E-05
5643	EMD	2574.96548	0.33755906	0.07590587	4.44707434	8.70E-06
5644	EME1	421.349839	-0.5608086	0.11060725	-5.0702696	3.97E-07
5646	EMG1	1358.4206	0.5523397	0.09207707	5.99866737	1.99E-09
5651	EML1	91.082402	-2.0983533	0.25647867	-8.1813951	2.81E-16
5652	EML2	1333.42986	-0.3062993	0.07350312	-4.1671601	3.08E-05
5654	EML3	1376.84876	-0.5955359	0.08260605	-7.2093494	5.62E-13
5655	EML4	3310.2951	0.3539195	0.08050734	4.3961149	1.10E-05
5659	EMP2	929.166447	-1.2140019	0.09275156	-13.088749	3.82E-39
5660	EMP3	7021.30597	-0.3984418	0.0607718	-6.5563597	5.51E-11
5666	ENAH	3343.4506	0.34043259	0.08644376	3.93819735	8.21E-05
5669	ENDOD1	2138.71029	-1.2376449	0.07460456	-16.5894	8.31E-62
5670	ENDOG	985.092351	-0.8917901	0.27197666	-3.2789215	0.00104205
5674	ENGASE	741.914599	-0.4902242	0.10136747	-4.8361097	1.32E-06
5677	ENKUR	14.2204398	1.22622508	0.53695949	2.28364543	0.02239238
5680	ENO2	5759.00554	-0.6545476	0.06003163	-10.903378	1.11E-27
5683	ENOPH1	3837.17622	0.77656507	0.06742279	11.5178424	1.07E-30
5692	ENPP4	643.576089	0.8548225	0.1065673	8.02143365	1.05E-15
5705	ENTPD4	2758.24594	-0.2502783	0.08233662	-3.0396966	0.00236817
5707	ENTPD6	3808.65071	-0.5807502	0.07688411	-7.5535786	4.23E-14
5708	ENTPD7	567.945314	0.34265601	0.09986246	3.43127939	0.00060074
5711	EOGT	505.602205	-0.6092116	0.11470669	-5.3110379	1.09E-07
5715	EP400	2725.65425	0.20871046	0.08644564	2.41435482	0.0157631
5720	EPB41L2	3626.01938	0.21892409	0.10944817	2.00025357	0.04547289
5721	EPB41L3	15.2907934	1.06248522	0.52715456	2.01550986	0.04385125
5722	EPB41L4A	16.9516062	-1.4480555	0.57899586	-2.5009773	0.01238511
5723	EPB41L4A-A!	1020.76498	1.20548994	0.1445543	8.33935744	7.47E-17
5726	EPB41L5	1642.42815	1.00413053	0.08813446	11.3931659	4.52E-30
5729	EPC2	879.499479	0.24339636	0.10342917	2.35326616	0.01860931
5730	EPCAM	1009.29141	0.57564528	0.10912345	5.27517506	1.33E-07
5731	EPDR1	2415.92938	-0.4105204	0.06818841	-6.0203843	1.74E-09
5732	EPG5	6597.34242	0.26395483	0.0656048	4.02340703	5.74E-05
5737	EPHA2	27626.4056	1.40785591	0.05980358	23.5413302	1.54E-122
5746	EPHB2	4361.78286	0.22355831	0.06023309	3.71155294	0.00020599

5747	EPHB3	64.3783002	2.3547262	0.30784106	7.64916214	2.02E-14
5750	EPHX1	5327.42183	-0.7195633	0.06345241	-11.340205	8.30E-30
5751	EPHX2	122.934403	-0.806177	0.2080543	-3.8748392	0.0001067
5753	EPHX4	727.24918	-1.386088	0.10468694	-13.240315	5.13E-40
5756	EPN1	8460.72902	-0.2387473	0.06721681	-3.5518995	0.00038246
5757	EPN2	1857.24888	0.45605664	0.07918879	5.75910633	8.46E-09
5760	EPN3	77.5091473	-1.7578613	0.25319558	-6.9427016	3.85E-12
5762	EPOR	668.611509	-0.7361375	0.09688951	-7.5977009	3.01E-14
5765	EPPK1	40.5954564	2.44723648	0.41132804	5.94959802	2.69E-09
5766	EPRS	9734.30126	0.67239321	0.06590317	10.2027444	1.93E-24
5767	EPS15	5410.77522	1.08781631	0.07296861	14.9080039	2.92E-50
5768	EPS15L1	2754.33541	0.21847705	0.07273769	3.00362928	0.0026678
5769	EPS8	3461.67818	0.42048891	0.07363816	5.71020386	1.13E-08
5770	EPS8L1	412.65901	-0.5957192	0.15189243	-3.9219809	8.78E-05
5771	EPS8L2	6610.695	-0.6654462	0.06348689	-10.481631	1.05E-25
5774	EPT1	2284.30289	0.63358163	0.08413287	7.53072633	5.05E-14
5780	ERAP2	565.374695	-0.8571153	0.12335799	-6.9481944	3.70E-12
5782	ERBB2	3096.00266	-0.4120302	0.08601762	-4.7900667	1.67E-06
5783	ERBB2IP	7277.85592	0.37196053	0.08977036	4.14346709	3.42E-05
5784	ERBB3	301.916464	-0.3070342	0.13524374	-2.2702285	0.02319372
5790	ERCC2	3524.46975	-0.1698934	0.08640381	-1.9662717	0.04926724
5794	ERCC6	740.482478	1.33108849	0.11940542	11.147639	7.35E-29
5796	ERCC6L	1640.6945	-0.3592183	0.07581661	-4.7379888	2.16E-06
5799	EREG	1200.35767	2.80957533	0.09855674	28.5071875	9.54E-179
5804	ERGIC3	8352.01512	-0.2928691	0.097899	-2.9915437	0.00277571
5805	ERH	6584.99835	-0.399287	0.08624676	-4.6295881	3.66E-06
5812	ERICH2	336.182505	0.50158192	0.12474616	4.02082045	5.80E-05
5816	ERICH5	2.17505238	4.4749939	1.68243254	2.65983556	0.00781788
5820	ERLEC1	2823.75378	-0.4857094	0.06653043	-7.3005596	2.87E-13
5826	ERMP1	746.484674	-0.5437418	0.0990328	-5.4905224	4.01E-08
5827	ERN1	263.401544	1.21495041	0.177319	6.85177812	7.29E-12
5829	ERO1A	8358.33327	-0.302254	0.0685153	-4.4114814	1.03E-05
5834	ERRFI1	12035.0245	0.31082063	0.06771631	4.59004113	4.43E-06
5835	ERV3-1	214.441231	1.56730362	0.17570838	8.9199138	4.67E-19
5839	ERVMER34-1	119.93159	0.9815885	0.18873174	5.20097217	1.98E-07
5844	ESCO1	1881.42122	0.46816673	0.08214533	5.69924928	1.20E-08
5846	ESD	2082.16586	0.27997254	0.07354018	3.80706912	0.00014062
5847	ESF1	3567.6863	0.92028762	0.07155416	12.8614125	7.42E-38
5849	ESPL1	1822.04372	-0.5910081	0.07773159	-7.6031903	2.89E-14
5854	ESR2	157.778035	1.16175931	0.18553955	6.26151836	3.81E-10
5857	ESRP2	529.917569	0.44092338	0.11239917	3.92283485	8.75E-05
5858	ESRRA	4471.16169	0.90610233	0.09767186	9.27700529	1.74E-20
5865	ETAA1	591.495974	-0.5012783	0.10208196	-4.9105472	9.08E-07

5866	ETF1	9638.44855	0.60687189	0.05561918	10.9111987	1.02E-27
5867	ETFA	3521.60941	-0.6822397	0.06585644	-10.359498	3.79E-25
5870	ETHE1	627.907667	0.4034018	0.10501829	3.84125294	0.00012241
5872	ETNK2	1026.15387	-0.5375425	0.08667732	-6.2016515	5.59E-10
5874	ETS1	16489.8353	0.78560819	0.0558385	14.0692935	5.87E-45
5875	ETS2	5963.65806	1.56917037	0.06453217	24.3160938	1.32E-130
5880	ETV4	2170.39117	0.40421366	0.06538579	6.18198045	6.33E-10
5881	ETV5	3089.3272	0.40552749	0.07569298	5.35753138	8.44E-08
5884	EVA1A	1088.80885	0.57515096	0.08791976	6.54177124	6.08E-11
5888	EVC	1745.25458	-0.7869345	0.10988548	-7.1614061	7.99E-13
5894	EVL	388.911434	-0.3200911	0.11449764	-2.7956127	0.00518014
5895	EVPL	1882.13064	-0.5664018	0.0811109	-6.9830542	2.89E-12
5908	EXOC2	2671.83019	-0.2161229	0.07631472	-2.831995	0.00462586
5909	EXOC3	2535.29941	-0.3232026	0.10046657	-3.217016	0.00129531
5911	EXOC3L1	14.4600953	-1.2967668	0.55976176	-2.3166405	0.02052332
5912	EXOC3L2	10.9236064	-6.9882872	1.3094743	-5.336712	9.46E-08
5914	EXOC4	3344.49546	-0.4959349	0.05868111	-8.4513554	2.88E-17
5917	EXOC6B	393.543161	0.92300174	0.16106657	5.73056057	1.00E-08
5918	EXOC7	6978.07185	0.2253047	0.05513439	4.08646395	4.38E-05
5920	EXOG	401.19128	0.37009311	0.12342263	2.99858396	0.00271237
5923	EXOSC2	2352.82044	0.49654499	0.07398775	6.71117866	1.93E-11
5925	EXOSC4	1801.6815	1.16176822	0.1346453	8.62836077	6.22E-18
5926	EXOSC5	1989.48025	0.33695443	0.10464734	3.21990444	0.00128233
5928	EXOSC7	1840.09632	0.34433259	0.09854812	3.49405549	0.00047574
5929	EXOSC8	1897.04547	0.21960313	0.08943282	2.45550937	0.01406851
5931	EXPH5	329.75759	0.65811452	0.13580475	4.8460345	1.26E-06
5940	EYA3	635.101275	0.81368416	0.10383891	7.83602357	4.65E-15
5942	EYS	12.9150048	2.66153403	0.66487179	4.0030786	6.25E-05
5952	F12	141.907418	0.75232956	0.17944523	4.19253037	2.76E-05
5957	F2RL1	6524.66236	0.38560113	0.06435668	5.99162566	2.08E-09
5960	F3	29352.2299	0.68676473	0.05297517	12.9638996	1.96E-38
5963	F8	281.013541	-0.3676522	0.13514626	-2.7204025	0.00652025
5964	F8A1	236.327107	-0.8695331	0.14237798	-6.1072165	1.01E-09
5978	FABP3	77.9186624	-0.9681843	0.24185201	-4.0032095	6.25E-05
5985	FADD	3870.57729	-0.5126707	0.07786153	-6.5843897	4.57E-11
5987	FADS2	4723.67567	-0.9187024	0.06558164	-14.008529	1.38E-44
5988	FADS3	3721.1125	0.27649798	0.07524015	3.67487263	0.00023797
5990	FAF1	2047.99156	0.55125907	0.07067117	7.80033845	6.17E-15
5991	FAF2	3897.04235	0.24379077	0.07639194	3.19131514	0.00141627
5992	FAH	1344.58706	-0.3987556	0.10443462	-3.818232	0.00013441
6002	FAM102A	3804.44971	-1.040438	0.07128636	-14.59519	3.01E-48
6003	FAM102B	1986.76906	-0.9676825	0.09907524	-9.7671476	1.56E-22
6005	FAM104A	2358.55683	0.43054311	0.07791968	5.52547331	3.29E-08

6007	FAM105A	672.055275	-0.317935	0.09349582	-3.4005264	0.00067256
6012	FAM107B	5004.64844	1.04498156	0.05845772	17.8758524	1.82E-71
6015	FAM110A	518.727984	-0.3321451	0.11984959	-2.7713492	0.00558245
6019	FAM111A	2847.04903	-0.3009355	0.06219627	-4.8384823	1.31E-06
6021	FAM114A1	5015.07533	-0.4409578	0.0687923	-6.4099875	1.46E-10
6022	FAM114A2	520.120537	0.21656896	0.10062382	2.1522634	0.03137661
6025	FAM118A	807.702202	0.86350285	0.09684047	8.91675611	4.80E-19
6028	FAM120AOS	1067.21806	0.35321332	0.07876237	4.48454389	7.31E-06
6030	FAM120C	522.560708	-0.6406373	0.11589778	-5.5276065	3.25E-08
6032	FAM122B	2583.20524	-0.1677279	0.06248622	-2.6842377	0.00726954
6036	FAM126A	2835.4196	0.44011436	0.08001829	5.50017211	3.79E-08
6038	FAM127A	3647.13841	-0.7522601	0.08797453	-8.5508852	1.22E-17
6039	FAM127B	1998.33345	-0.2914329	0.074804	-3.8959532	9.78E-05
6040	FAM127C	1287.55542	-0.3501554	0.07893573	-4.4359556	9.17E-06
6048	FAM132B	874.055353	0.45279108	0.08769897	5.16301473	2.43E-07
6049	FAM133A	38.5299432	-2.1887386	0.36837659	-5.9415789	2.82E-09
6055	FAM134C	1922.50662	-0.8406834	0.07150978	-11.756201	6.56E-32
6056	FAM135A	906.479008	1.00904022	0.11077475	9.10893715	8.32E-20
6058	FAM136A	3477.71527	0.69536328	0.06093403	11.4117387	3.65E-30
6067	FAM13B	2819.36329	0.70025402	0.08148634	8.59351382	8.43E-18
6068	FAM13C	62.9289148	-1.1435665	0.28514329	-4.0104976	6.06E-05
6069	FAM149A	54.274949	-0.7057346	0.284945	-2.4767398	0.01325885
6090	FAM160B1	1998.14419	-0.4778272	0.08040894	-5.9424643	2.81E-09
6091	FAM160B2	1264.92507	-0.5093188	0.08212486	-6.2017609	5.58E-10
6100	FAM167A	552.062987	0.25560554	0.1032712	2.47509016	0.01332025
6103	FAM168A	2649.53142	-0.2718261	0.09355081	-2.9056518	0.00366489
6105	FAM169A	528.614381	0.76169965	0.14796405	5.14786961	2.63E-07
6111	FAM171A2	309.767123	-0.678933	0.14314521	-4.7429672	2.11E-06
6113	FAM172A	458.626107	-0.7775067	0.10466742	-7.428354	1.10E-13
6115	FAM173A	709.234861	-0.6055869	0.17890052	-3.3850485	0.00071166
6124	FAM179A	88.7702631	-1.5051231	0.28886168	-5.210532	1.88E-07
6125	FAM179B	738.926978	0.31021301	0.11363668	2.72986685	0.00633599
6146	FAM189B	2387.58305	-0.4259107	0.07081318	-6.0145677	1.80E-09
6147	FAM192A	1809.68657	-0.4516831	0.07327963	-6.1638287	7.10E-10
6149	FAM193B	1777.68305	-0.8527789	0.06945846	-12.277539	1.20E-34
6153	FAM196B	189.111062	0.49241441	0.18244116	2.69903141	0.00695416
6157	FAM198B	93.7060085	-1.4356187	0.23085372	-6.2187377	5.01E-10
6164	FAM200A	522.136078	0.72720336	0.10046242	7.23856132	4.53E-13
6165	FAM200B	1362.51038	0.21980733	0.07712241	2.85010986	0.00437041
6173	FAM208A	3521.79539	0.36051569	0.0688354	5.23735865	1.63E-07
6174	FAM208B	5276.52588	1.14518444	0.10646199	10.7567447	5.51E-27
6179	FAM20C	5308.80108	-0.6169032	0.09446988	-6.5301581	6.57E-11
6180	FAM210A	2769.38103	0.32604176	0.0651228	5.00656869	5.54E-07

6183	FAM212B	108.639631	0.46602885	0.19627744	2.3743373	0.01758048
6185	FAM213A	3035.1549	-0.4636006	0.08576474	-5.4054919	6.46E-08
6193	FAM217B	660.486522	-0.6535928	0.10123604	-6.4561282	1.07E-10
6196	FAM219B	1273.74797	-0.380586	0.08069431	-4.7163927	2.40E-06
6200	FAM220A	1467.9781	0.44985345	0.07676154	5.86040184	4.62E-09
6204	FAM222A-AS	3.02825681	2.95009879	1.44674867	2.03912322	0.04143773
6227	FAM24B	257.97227	1.62915387	0.15790586	10.3172478	5.89E-25
6235	FAM26F	61.5927697	-0.9401338	0.28942188	-3.2483162	0.0011609
6241	FAM32A	3017.94392	-0.3352573	0.0660721	-5.0741128	3.89E-07
6247	FAM3C	2712.71408	-0.448995	0.07262427	-6.1824373	6.31E-10
6252	FAM43A	547.035884	-1.656585	0.1047097	-15.820741	2.24E-56
6254	FAM45A	710.674555	-1.3022372	0.09769494	-13.329628	1.56E-40
6255	FAM45B	137.648339	-1.3751	0.18472748	-7.4439383	9.77E-14
6258	FAM46C	513.309337	-0.9361985	0.10599215	-8.8327161	1.02E-18
6268	FAM50B	187.625636	-1.2908308	0.17209161	-7.5008351	6.34E-14
6269	FAM53A	184.593202	0.70518845	0.19207429	3.67143586	0.00024119
6270	FAM53B	739.621899	-0.6327399	0.08771618	-7.2134916	5.45E-13
6273	FAM57A	1369.83469	-0.8451566	0.09559411	-8.8410944	9.48E-19
6276	FAM60A	1328.2236	1.08985696	0.10156793	10.7303261	7.33E-27
6277	FAM63A	457.230277	-1.3585616	0.13455473	-10.096721	5.71E-24
6279	FAM64A	1011.25094	-0.6016284	0.09290118	-6.476004	9.42E-11
6282	FAM65C	154.518828	-1.4084152	0.17845205	-7.8924013	2.96E-15
6299	FAM72A	144.668189	1.23618916	0.18931738	6.52971844	6.59E-11
6300	FAM72B	126.012557	0.36090536	0.18207159	1.98221683	0.04745498
6301	FAM72C	72.2993469	0.76753083	0.29636664	2.5898017	0.00960312
6303	FAM73A	1381.62083	0.57567676	0.08761622	6.5704357	5.02E-11
6310	FAM76A	347.693187	0.34923825	0.12467954	2.80108705	0.00509308
6314	FAM81A	72.5516855	-1.3686787	0.24779936	-5.5233345	3.33E-08
6321	FAM83D	2359.5541	-0.8928375	0.06604673	-13.518269	1.22E-41
6324	FAM83G	1155.69143	0.82885121	0.11662637	7.10689357	1.19E-12
6328	FAM84B	2459.53156	1.22511694	0.06805512	18.0018323	1.88E-72
6335	FAM86EP	83.1559701	0.9402652	0.23299521	4.0355559	5.45E-05
6337	FAM86HP	27.2928545	0.86404598	0.3874383	2.23015118	0.02573741
6341	FAM89A	439.764504	0.35991185	0.1059677	3.39642973	0.00068271
6350	FAM91A1	6511.94892	0.70377133	0.08022511	8.77245726	1.75E-18
6357	FAM96A	1645.34397	-0.2294805	0.0986619	-2.3259283	0.02002238
6359	FAM98A	2313.53208	0.48730747	0.0681323	7.15237023	8.53E-13
6360	FAM98B	1410.6281	-0.2328249	0.10162161	-2.291096	0.02195786
6373	FANCE	1346.04907	0.30955294	0.08616105	3.59272459	0.00032724
6375	FANCG	1804.37901	-0.2808326	0.09065056	-3.0979683	0.00194852
6379	FANK1	114.627255	-0.7099037	0.19540716	-3.6329462	0.0002802
6382	FAR1	5317.98869	0.90808945	0.07132709	12.7313403	3.96E-37
6386	FARP1	1029.46616	-0.8259834	0.08133812	-10.154936	3.15E-24

6388	FARS2	550.270707	-0.7620086	0.13334684	-5.7144854	1.10E-08
6389	FARSA	5144.73518	0.3391617	0.08209026	4.13157052	3.60E-05
6390	FARSB	1964.63554	0.36448173	0.08791576	4.14580661	3.39E-05
6391	FAS	204.036477	-1.2965332	0.15286289	-8.4816741	2.22E-17
6395	FASTK	3566.84081	-0.6395021	0.09212703	-6.9415259	3.88E-12
6397	FASTKD2	1726.25257	0.50955975	0.07694045	6.62278119	3.53E-11
6398	FASTKD3	778.387819	0.2729947	0.10480058	2.60489689	0.00919019
6400	FAT1	6662.87885	-0.628373	0.09917157	-6.3362215	2.35E-10
6402	FAT3	271.206863	1.69516969	0.13713755	12.361091	4.24E-35
6407	FAXDC2	234.35946	-2.1256255	0.16112532	-13.192374	9.71E-40
6409	FBL	11633.7134	0.48155298	0.10232191	4.70625488	2.52E-06
6410	FBLIM1	719.459762	0.34921218	0.10032935	3.48065822	0.00050018
6415	FBLN7	565.515214	-0.442128	0.10781798	-4.1006891	4.12E-05
6419	FBP1	1604.19758	-0.2236137	0.08827369	-2.5331867	0.01130307
6421	FBRS	2751.02416	0.3629191	0.0986541	3.67870274	0.00023442
6422	FBRSL1	1823.38885	0.78409327	0.09462408	8.28640276	1.17E-16
6423	FBXL12	919.421045	0.17668846	0.08661429	2.0399458	0.04135572
6424	FBXL13	240.143942	0.90541449	0.13944829	6.49283326	8.42E-11
6426	FBXL15	601.141366	-1.1950794	0.16720819	-7.1472543	8.85E-13
6428	FBXL17	635.74219	-0.803317	0.1291597	-6.2195636	4.99E-10
6431	FBXL19-AS1	158.400432	0.64990536	0.19260029	3.37437383	0.00073984
6433	FBXL20	538.771406	-0.8283098	0.10370099	-7.9874825	1.38E-15
6438	FBXL5	2475.32184	-0.1729715	0.06519004	-2.6533429	0.00796989
6441	FBXL8	103.081968	-1.4069985	0.20973332	-6.7085121	1.97E-11
6443	FBXO11	2055.36673	0.25304752	0.07888919	3.20763247	0.00133832
6446	FBXO17	1510.00096	0.9207916	0.10637177	8.65635275	4.87E-18
6453	FBXO25	606.670079	-0.4027948	0.0953542	-4.2241956	2.40E-05
6456	FBXO3	1309.48928	-0.4333973	0.07815241	-5.54554	2.93E-08
6458	FBXO30	1123.84397	0.40776318	0.08229386	4.95496503	7.23E-07
6462	FBXO34	2463.50576	0.53384884	0.06331002	8.43229575	3.39E-17
6469	FBXO42	1454.86053	0.92706988	0.0767945	12.0720866	1.48E-33
6470	FBXO43	329.668821	-0.9039188	0.13821512	-6.5399413	6.15E-11
6472	FBXO45	3156.76228	0.95615916	0.06729818	14.2078001	8.20E-46
6477	FBXO6	134.175762	0.58454559	0.19168101	3.04957494	0.00229165
6479	FBXO8	866.824696	-0.8017942	0.09109347	-8.8018846	1.35E-18
6480	FBXO9	1686.80274	-0.4554706	0.06801399	-6.6967188	2.13E-11
6481	FBXW10	32.0563081	2.39440478	0.40045158	5.97926165	2.24E-09
6487	FBXW5	5947.72384	-0.4061023	0.07041877	-5.7669609	8.07E-09
6490	FBXW9	422.552165	-0.7550706	0.12024587	-6.2793891	3.40E-10
6496	FCF1	2177.75945	0.34272534	0.07837304	4.37300067	1.23E-05
6497	FCGBP	411.895618	-1.4003626	0.12180099	-11.497137	1.36E-30
6501	FCGR2A	9.14972809	-2.7561873	0.8087577	-3.4079271	0.00065458
6506	FCGRT	1835.52991	-0.2993125	0.08869943	-3.3744584	0.00073961

6524	FDFT1	3527.58297	-0.2090688	0.06753566	-3.0956803	0.00196362
6525	FDPS	5341.37535	-0.3285366	0.09114764	-3.6044446	0.00031282
6530	FDXR	248.63346	-0.4223681	0.17131444	-2.4654552	0.01368393
6532	FEM1A	1797.67151	0.42595717	0.07051348	6.04079091	1.53E-09
6533	FEM1B	1941.5305	0.36064088	0.0710545	5.07555339	3.86E-07
6534	FEM1C	2178.65672	0.18335612	0.08512141	2.154054	0.03123594
6545	FERMT2	3815.23257	0.2437289	0.07781879	3.13200594	0.00173616
6551	FEZ2	4117.71223	0.51273563	0.06436917	7.9655467	1.64E-15
6558	FFAR4	26.7381974	-3.9132522	0.56923506	-6.8745805	6.22E-12
6561	FGD1	994.0198	-0.4454019	0.08280967	-5.3786218	7.51E-08
6563	FGD3	24.023575	-1.3040453	0.43990148	-2.9644031	0.00303271
6566	FGD5-AS1	5466.1707	-0.2071703	0.06983631	-2.9665129	0.00301198
6597	FGFBP1	45.3044544	-0.9946895	0.30570834	-3.2537204	0.00113904
6600	FGFR1	4573.8929	0.45077198	0.07147187	6.30698464	2.85E-10
6601	FGFR1OP	2104.66909	-0.327934	0.07904871	-4.1485047	3.35E-05
6613	FHAD1	83.4380698	2.32776033	0.26546295	8.76868243	1.81E-18
6614	FHDC1	145.729509	-0.4439232	0.16978625	-2.6146002	0.00893319
6617	FHL2	1893.54712	1.26118552	0.08494398	14.8472625	7.25E-50
6618	FHL3	1164.64073	0.36249082	0.08741493	4.14678397	3.37E-05
6620	FHOD1	1289.03432	-0.6529841	0.08454982	-7.7230695	1.14E-14
6621	FHOD3	2202.31999	-0.2565019	0.07302858	-3.5123505	0.00044416
6625	FICD	233.280746	0.61164839	0.15627913	3.91381995	9.08E-05
6626	FIG4	467.014248	-0.6596277	0.11022652	-5.9842921	2.17E-09
6629	FIGN	516.231692	0.66466518	0.14250225	4.66424329	3.10E-06
6630	FIGNL1	1535.54421	0.19603467	0.08363043	2.34405922	0.01907514
6636	FIS1	2449.38526	-0.7547926	0.10729877	-7.0344943	2.00E-12
6640	FJX1	3873.1636	0.327546	0.08856204	3.69849203	0.00021688
6644	FKBP15	1149.96954	-0.9228607	0.10675639	-8.6445478	5.40E-18
6651	FKBP4	7657.54465	1.04612023	0.06049574	17.2924603	5.36E-67
6654	FKBP7	417.611804	-0.386899	0.11223892	-3.4471019	0.00056663
6656	FKBP9	3866.13436	-0.2879931	0.06044847	-4.7642749	1.90E-06
6657	FKBP9P1	18.4620472	-3.0014714	0.57474724	-5.2222459	1.77E-07
6658	FKBPL	680.702606	0.50019292	0.09633682	5.19212594	2.08E-07
6662	FLAD1	783.135494	0.24752568	0.08431833	2.93560919	0.00332893
6667	FLI1	55.2243929	-2.9641013	0.33953121	-8.7299819	2.55E-18
6669	FLJ10038	325.381776	-0.6720919	0.15421419	-4.3581717	1.31E-05
6709	FLJ42627	39.8223488	0.77518316	0.3470076	2.23390829	0.02548911
6720	FLNB	69601.6797	1.19830533	0.07625609	15.714225	1.21E-55
6721	FLNC	14440.8186	0.79171936	0.0869763	9.10270255	8.81E-20
6723	FLOT2	5082.9544	-0.5122445	0.05837483	-8.7750919	1.71E-18
6738	FMNL1	4380.48077	-1.0573326	0.07088583	-14.915994	2.59E-50
6753	FN3K	430.387679	-1.220162	0.114835	-10.62535	2.27E-26
6755	FNBP1	3655.29275	-0.3338533	0.06772954	-4.9292126	8.26E-07

6757	FNBP4	2582.90709	0.30083608	0.06829671	4.40484007	1.06E-05
6760	FNDC3B	8301.36729	0.34748523	0.09752344	3.56309457	0.00036651
6774	FOLR1	144.60039	-1.4232484	0.19172567	-7.4233583	1.14E-13
6778	FOS	101.567943	0.49743105	0.21873968	2.27407781	0.02296131
6779	FOSB	45.5394055	2.57699294	0.38547573	6.68522749	2.31E-11
6780	FOSL1	12858.3644	0.26110096	0.06317834	4.13276046	3.58E-05
6781	FOSL2	4911.69528	0.19937733	0.0764772	2.6070166	0.00913349
6787	FOXC1	882.305755	0.83710521	0.09179852	9.11894048	7.59E-20
6814	FOXJ3	2837.32989	0.88152925	0.06346311	13.8904209	7.24E-44
6816	FOXK2	6792.00503	0.99463878	0.05602372	17.753888	1.61E-70
6820	FOXM1	5905.90937	-0.5578594	0.08284537	-6.7337427	1.65E-11
6822	FOXN2	1077.70904	0.59040512	0.12390899	4.76482881	1.89E-06
6824	FOXN3-AS1	166.111757	0.85738283	0.17984241	4.76741173	1.87E-06
6828	FOXO3	1245.66604	0.48353768	0.07873405	6.1414052	8.18E-10
6830	FOXO4	354.062822	-0.3621587	0.11862262	-3.0530321	0.00226542
6838	FOXQ1	1127.37134	-0.5327984	0.08615161	-6.1844277	6.23E-10
6842	FOXRED2	1268.53209	-1.8393841	0.09287754	-19.804402	2.73E-87
6847	FPR1	134.589041	-1.6774382	0.18518538	-9.0581569	1.33E-19
6848	FPR2	7.77797218	-1.9736486	0.77799022	-2.5368553	0.01118532
6851	FRAS1	1963.71417	-0.5334103	0.08741785	-6.1018468	1.05E-09
6852	FRAT1	73.172379	-1.6284837	0.26620019	-6.1175154	9.50E-10
6853	FRAT2	634.87581	-0.5039741	0.11100142	-4.5402489	5.62E-06
6857	FRG1	524.643267	-0.4987434	0.1190091	-4.1908008	2.78E-05
6861	FRG1HP	332.16001	-0.6500736	0.13254058	-4.9047133	9.36E-07
6870	FRMD3	584.111288	-0.4993958	0.09745092	-5.1245879	2.98E-07
6872	FRMD4B	276.648286	-0.5497694	0.14787128	-3.7178918	0.00020089
6874	FRMD6	11398.8839	0.93627094	0.07813491	11.9827487	4.38E-33
6883	FRMPD3	25.1369766	1.17013118	0.47464736	2.46526425	0.01369122
6886	FRRS1	72.8555308	-0.7984096	0.30741467	-2.5971746	0.00939941
6890	FRY	71.723544	-1.7996377	0.31236307	-5.761365	8.34E-09
6896	FSCN1	1357.79681	-0.7423322	0.07983522	-9.2983046	1.43E-20
6900	FSD1L	509.872522	0.47522175	0.11059851	4.29681853	1.73E-05
6908	FSTL3	4521.83222	1.12415399	0.11552298	9.73099864	2.22E-22
6917	FTL	99441.5276	0.92960146	0.07910963	11.7507998	7.00E-32
6922	FTSJ1	4455.08882	0.50778819	0.06139266	8.27115491	1.33E-16
6928	FUCA1	1816.99453	-0.8934678	0.07327737	-12.192957	3.39E-34
6930	FUK	233.972869	-0.5761659	0.172097	-3.347914	0.00081422
6935	FURIN	5830.56618	-0.5893142	0.07740405	-7.6134798	2.67E-14
6936	FUS	15892.6228	0.33523525	0.06821814	4.91416582	8.92E-07
6937	FUT1	101.533608	1.0348803	0.21625257	4.78551678	1.71E-06
6938	FUT10	261.045602	-0.324114	0.14465175	-2.2406504	0.02504873
6939	FUT11	607.032019	-0.9674129	0.12146001	-7.9648678	1.65E-15
6946	FUT8	5895.65364	-1.1602924	0.0678205	-17.108286	1.29E-65

6947	FUT8-AS1	123.043324	-1.9499373	0.19884031	-9.8065489	1.06E-22
6952	FXR2	3878.94483	0.13438511	0.05869972	2.28936553	0.02205812
6963	FYN	2727.54439	0.53531812	0.06455572	8.29234229	1.11E-16
6965	FZD1	1587.90245	-1.366522	0.07347174	-18.599286	3.26E-77
6968	FZD2	2209.93179	-1.0423172	0.0749874	-13.899898	6.34E-44
6970	FZD4	561.183382	-0.7631203	0.10664279	-7.1558547	8.32E-13
6972	FZD6	3173.17815	-0.6491287	0.0761286	-8.5267389	1.51E-17
6977	G0S2	8190.24911	0.24482235	0.12114734	2.0208645	0.04329379
6979	G3BP1	11582.7651	0.50175701	0.06303698	7.95972497	1.72E-15
6985	GAA	2076.30035	-0.7502878	0.07212675	-10.402352	2.42E-25
6991	GABARAPL1	1662.17254	0.34573588	0.07631351	4.5304677	5.89E-06
6992	GABARAPL2	1546.25522	-0.205004	0.08586326	-2.3875641	0.01696045
6994	GABBR1	1134.5351	0.64933219	0.09205874	7.05345478	1.75E-12
6997	GABPB1	1272.96968	0.5302216	0.07831342	6.7705072	1.28E-11
6998	GABPB1-AS1	448.90602	1.22960179	0.1262711	9.73779278	2.08E-22
7021	GACAT2	27.3409779	0.9845241	0.3890021	2.53089662	0.01137714
7025	GADD45A	8143.90661	2.87527917	0.10360332	27.7527707	1.61E-169
7053	GAL	198.662322	2.01288793	0.16855707	11.9418777	7.16E-33
7059	GALE	5473.3488	0.47596763	0.08170609	5.82536313	5.70E-09
7060	GALK1	716.067447	-1.1329242	0.12005203	-9.4369431	3.84E-21
7062	GALM	562.395712	-0.4814462	0.10066866	-4.7824837	1.73E-06
7064	GALNT1	4813.642	-0.2760292	0.07735763	-3.5682216	0.00035941
7065	GALNT10	3240.49472	-0.5237102	0.08916645	-5.8733992	4.27E-09
7066	GALNT11	2559.28042	-0.2771754	0.0620012	-4.4704844	7.80E-06
7073	GALNT2	8857.59705	-0.6086452	0.06248127	-9.7412416	2.01E-22
7074	GALNT3	1583.52547	0.49030477	0.07787428	6.29610676	3.05E-10
7076	GALNT5	760.957312	-1.5652941	0.09215597	-16.98527	1.06E-64
7087	GALT	840.285291	-0.4289916	0.10431442	-4.1124859	3.91E-05
7088	GAMT	1543.82862	-0.5618417	0.08855634	-6.3444542	2.23E-10
7089	GAN	333.079621	1.28237496	0.13022428	9.84743378	7.03E-23
7091	GANC	527.122939	-0.4314944	0.10328589	-4.17767	2.95E-05
7093	GAPDH	61149.5984	-0.2082075	0.05690454	-3.6588918	0.00025331
7098	GAR1	2097.64897	0.69585183	0.08678598	8.01802165	1.07E-15
7101	GARNL3	195.43161	1.18601468	0.16402976	7.23048496	4.81E-13
7102	GARS	15478.5437	0.84034481	0.05071579	16.5696887	1.15E-61
7103	GART	5955.67598	0.8883199	0.0576288	15.4145138	1.31E-53
7106	GAS2L1	2229.15513	-0.3433266	0.08401366	-4.0865567	4.38E-05
7109	GAS2L3	1243.69751	0.23393132	0.11543781	2.02647048	0.0427166
7110	GAS5	7780.27731	1.91338237	0.08876338	21.555988	4.65E-103
7112	GAS6	14754.6389	-0.5121668	0.08288708	-6.1790907	6.45E-10
7120	GATA2	752.489093	0.90815645	0.09007694	10.0820086	6.64E-24
7126	GATA6	2746.44984	0.86635385	0.07239473	11.9670845	5.29E-33
7129	GATAD2A	6364.44275	0.52168907	0.06276431	8.31187437	9.42E-17

7130	GATAD2B	2117.64634	0.38733024	0.08271013	4.68298442	2.83E-06
7132	GATC	2998.78838	0.4538225	0.06557236	6.92094254	4.49E-12
7134	GATS	666.902384	-0.265445	0.10111105	-2.6252815	0.00865774
7135	GATSL2	648.605995	-0.3151266	0.10794857	-2.9192285	0.00350899
7136	GATSL3	194.851411	-1.261716	0.15137435	-8.3350712	7.75E-17
7141	GBAS	1853.40185	-0.5793428	0.08842676	-6.5516682	5.69E-11
7146	GBP1	1595.661	-0.4876868	0.07867045	-6.1991112	5.68E-10
7148	GBP2	3891.8593	-1.4809221	0.0632753	-23.404429	3.85E-121
7149	GBP3	2981.35054	-0.2387164	0.08892086	-2.6845937	0.0072618
7150	GBP4	763.46325	-1.6783866	0.13405001	-12.5206	5.76E-36
7157	GCA	776.290955	-0.5765053	0.09731812	-5.9239262	3.14E-09
7159	GCC1	1620.25195	0.78265886	0.07832782	9.99209281	1.65E-23
7165	GCH1	596.035302	0.92634252	0.1109713	8.34758666	6.97E-17
7166	GCHFR	265.717159	-1.569417	0.15267895	-10.279197	8.75E-25
7168	GCKR	39.7574269	0.86707286	0.34563618	2.50862875	0.01212008
7173	GCN1	11007.6534	0.35066069	0.0720505	4.86687375	1.13E-06
7175	GCNT2	3547.86108	0.75434513	0.06388689	11.807511	3.57E-32
7186	GDAP1	139.568552	0.75942738	0.17763491	4.2752148	1.91E-05
7188	GDAP2	1036.68074	0.60497614	0.08582661	7.04881766	1.80E-12
7192	GDF11	380.051616	0.75721405	0.14308685	5.29198896	1.21E-07
7196	GDF5	310.375056	0.5990084	0.13491325	4.43995234	9.00E-06
7208	GDPD5	998.644586	-2.2389162	0.09043087	-24.758319	2.52E-135
7210	GEM	1766.43242	0.9375283	0.07314395	12.8175782	1.31E-37
7213	GEMIN5	3000.89577	1.09130549	0.07084817	15.4034397	1.55E-53
7215	GEMIN7	1573.15314	0.45136526	0.09129258	4.94416172	7.65E-07
7217	GEMIN8P4	36.8297351	-1.0188081	0.3301859	-3.085559	0.0020317
7219	GET4	4684.73245	0.84474485	0.11009617	7.67279087	1.68E-14
7220	GFAP	269.285155	-1.647933	0.14430374	-11.419892	3.33E-30
7222	GFI1	16.601919	-1.1079142	0.52680785	-2.1030708	0.03545958
7224	GFM1	2531.98593	0.34362576	0.07216481	4.76168031	1.92E-06
7226	GFOD1	192.861006	1.41005484	0.16454831	8.56924521	1.04E-17
7227	GFOD2	897.880997	0.2793971	0.08528621	3.27599374	0.00105291
7228	GFPT1	7297.29653	1.34650139	0.05817866	23.1442493	1.66E-118
7236	GGA1	1250.5062	0.20402936	0.07364087	2.77059958	0.00559532
7240	GGCT	2009.69245	0.51061196	0.08397169	6.08076315	1.20E-09
7242	GGH	2062.42012	-0.5804228	0.09228773	-6.2892742	3.19E-10
7245	GGNBP2	4968.86627	0.61234013	0.05923296	10.3378273	4.75E-25
7247	GGT1	455.359801	-2.1460551	0.12974172	-16.540979	1.86E-61
7249	GGT5	626.129543	-2.394433	0.11598692	-20.643992	1.11E-94
7251	GGT7	172.443499	-1.2775597	0.18330423	-6.9696135	3.18E-12
7258	GHDC	1532.14416	-0.7081802	0.07966992	-8.888928	6.17E-19
7270	GIGYF1	3635.13553	-0.3398373	0.06211982	-5.4706738	4.48E-08
7291	GIPR	134.264969	-0.5427384	0.17675395	-3.0705869	0.00213639

7296	GJA3	404.375686	0.93232084	0.1126906	8.27327968	1.30E-16
7304	GJB3	1405.94594	2.20026605	0.0886924	24.807832	7.38E-136
7305	GJB4	89.5475757	5.63550492	0.4940746	11.4061822	3.89E-30
7318	GK5	470.309382	-0.2342516	0.11277368	-2.0771835	0.03778463
7319	GKAP1	467.30541	0.53390589	0.10935182	4.88246009	1.05E-06
7323	GLB1	3706.42213	-0.8371853	0.07837793	-10.681391	1.24E-26
7327	GLCCI1	234.369381	-0.4100736	0.16189437	-2.5329701	0.01131006
7328	GLCE	2689.72211	-0.4519725	0.09062129	-4.987487	6.12E-07
7331	GLE1	2712.21501	-0.3520819	0.06161924	-5.7138303	1.10E-08
7335	GLI3	547.784507	0.44365528	0.107293	4.13498794	3.55E-05
7336	GLI4	397.927299	-0.8180429	0.11453243	-7.1424562	9.17E-13
7341	GLIPR2	356.615676	-1.1573424	0.12019687	-9.6287236	6.05E-22
7347	GLMN	745.553713	-0.3365349	0.10426115	-3.2278074	0.00124743
7349	GLO1	13104.9791	0.52123079	0.0704977	7.39358535	1.43E-13
7359	GLRX	847.474822	-0.5660394	0.09243805	-6.1234455	9.16E-10
7360	GLRX2	913.049696	0.6565057	0.09394392	6.98827247	2.78E-12
7361	GLRX3	3905.60261	0.20409158	0.09461038	2.15717968	0.03099167
7367	GLT8D1	2077.33219	-0.5206234	0.08158117	-6.3816613	1.75E-10
7388	GM2A	1900.17796	-0.9684011	0.0878211	-11.026975	2.83E-28
7393	GMEB1	584.185136	0.62858684	0.10520075	5.97511724	2.30E-09
7394	GMEB2	1958.32969	0.1422344	0.06717796	2.1172778	0.03423628
7396	GMFG	71.6713767	-1.9492605	0.25974896	-7.5044016	6.17E-14
7404	GMPR2	2358.08614	-0.2310076	0.08263271	-2.7955949	0.00518043
7408	GNA13	4850.32864	0.27977942	0.08258851	3.38763153	0.00070499
7413	GNAI2	13126.1836	-0.3435724	0.05738824	-5.9868074	2.14E-09
7424	GNB1	23649.3526	0.75558172	0.05078932	14.8767846	4.66E-50
7426	GNB2	10409.829	-0.2900052	0.05476662	-5.2952919	1.19E-07
7427	GNB2L1	38451.2022	0.16502158	0.05992811	2.75365895	0.00589331
7431	GNE	2969.82627	-0.5845773	0.08588769	-6.8062995	1.00E-11
7434	GNG12	7259.60626	0.49832868	0.08635662	5.77059049	7.90E-09
7437	GNG2	79.0618314	-2.0803539	0.27568528	-7.546119	4.48E-14
7440	GNG5	3777.44454	0.25863898	0.07629349	3.39005306	0.00069879
7441	GNG7	139.722316	-2.1461544	0.19379305	-11.074465	1.67E-28
7445	GNL1	3541.66548	0.48009852	0.06574573	7.30235293	2.83E-13
7446	GNL2	4361.13055	1.31162216	0.06600392	19.8718832	7.13E-88
7447	GNL3	7894.51156	1.09938989	0.06429019	17.100431	1.47E-65
7448	GNL3L	1957.02735	0.14954347	0.06863077	2.17895656	0.0293349
7451	GNPAT	2742.00566	-0.2832868	0.06435261	-4.4021033	1.07E-05
7454	GNPNAT1	4699.98049	1.07891914	0.06845039	15.7620589	5.68E-56
7457	GNRH1	99.8654483	-0.4596233	0.20973448	-2.1914534	0.028419
7461	GNS	9199.97726	-0.3705252	0.06239722	-5.9381676	2.88E-09
7467	GOLGA2P5	197.898125	-0.8589833	0.18094364	-4.7472421	2.06E-06
7471	GOLGA3	4192.87629	-0.1329988	0.05687465	-2.3384555	0.01936363

7479	GOLGA6L10	36.3806302	-0.8184455	0.34633532	-2.363159	0.01811989
7509	GOLIM4	1830.31798	-0.7385126	0.09230639	-8.0006662	1.24E-15
7510	GOLM1	9758.95718	-0.4780418	0.06095224	-7.8428906	4.40E-15
7515	GON4L	1333.82937	0.30336026	0.0798616	3.79857495	0.00014553
7516	GOPC	2232.03129	0.52926146	0.07748454	6.83054243	8.46E-12
7518	GORASP1	1482.32884	0.14232281	0.07136751	1.99422397	0.04612758
7519	GORASP2	6495.76569	0.44071567	0.07784768	5.66125639	1.50E-08
7520	GOSR1	2074.51752	0.33842329	0.06829021	4.95566325	7.21E-07
7524	GOT2	5832.87386	0.4758748	0.05459423	8.71657749	2.87E-18
7532	GPAA1	5638.5287	-0.3027787	0.07655215	-3.9551946	7.65E-05
7533	GPALPP1	576.853798	0.32542777	0.1194542	2.72428908	0.00644401
7534	GPAM	456.959731	-0.5251158	0.11498278	-4.5669083	4.95E-06
7535	GPANK1	1056.21114	0.43373296	0.07772657	5.58024077	2.40E-08
7536	GPAT2	1516.8602	-0.7381408	0.07913226	-9.3279386	1.08E-20
7538	GPAT4	3965.61367	-0.1800476	0.07095251	-2.5375789	0.01116222
7541	GPATCH2	790.634164	0.62905632	0.10451775	6.01865545	1.76E-09
7544	GPATCH4	1942.26946	0.16487448	0.06747226	2.44358907	0.01454198
7547	GPBP1	4982.16567	0.58485238	0.05818683	10.0512845	9.07E-24
7548	GPBP1L1	3825.70439	0.64244146	0.07077373	9.07740031	1.11E-19
7561	GPD1L	1055.62147	-0.437783	0.08169703	-5.3586163	8.39E-08
7563	GPER1	426.187482	-0.7302389	0.12471714	-5.8551608	4.77E-09
7567	GPI	15213.0835	-0.3009642	0.05263825	-5.7175948	1.08E-08
7574	GPN2	1046.80426	0.99349323	0.10557007	9.41074723	4.93E-21
7577	GPR1	798.773358	0.94151909	0.08617981	10.9250544	8.75E-28
7587	GPR137B	193.75535	-0.4843157	0.15040645	-3.2200459	0.0012817
7593	GPR146	75.4134403	-2.2592385	0.26320064	-8.5837122	9.19E-18
7606	GPR160	143.856538	0.56267513	0.18661909	3.01509956	0.00256895
7626	GPR3	249.421247	1.0184165	0.13763361	7.39947505	1.37E-13
7631	GPR35	255.879141	1.58726811	0.14829098	10.70374	9.78E-27
7634	GPR39	441.851521	-0.7318698	0.10963872	-6.675286	2.47E-11
7639	GPR55	55.1197608	-2.6139901	0.34295273	-7.6220127	2.50E-14
7643	GPR63	92.241437	1.86645146	0.24515219	7.61343976	2.67E-14
7645	GPR68	1428.52236	-0.5827295	0.07729619	-7.5389167	4.74E-14
7657	GPRASP1	71.3414342	0.68716135	0.25601141	2.68410434	0.00727244
7661	GPRC5C	725.993951	-0.2617214	0.10900272	-2.4010541	0.01634792
7664	GPRIN1	589.875549	-0.5748033	0.11057398	-5.1983595	2.01E-07
7665	GPRIN2	124.457329	-0.715784	0.19206883	-3.7267056	0.000194
7667	GPS1	2986.58182	-0.5922719	0.07046401	-8.4053108	4.27E-17
7669	GPSM1	1234.76775	0.29055706	0.07945774	3.65674947	0.00025543
7670	GPSM2	3130.26881	-0.2509699	0.05970334	-4.2036158	2.63E-05
7673	GPT2	1810.37393	0.94031411	0.07485598	12.5616427	3.43E-36
7674	GPX1	8029.34971	-0.252345	0.11775238	-2.143014	0.03211197
7677	GPX4	15340.5464	-0.2138886	0.09507262	-2.2497388	0.02446553

7680	GPX7	71.5919262	-2.0281971	0.300352	-6.7527338	1.45E-11
7683	GRAMD1B	266.622266	3.99184152	0.20289746	19.6741815	3.59E-86
7684	GRAMD1C	277.339726	-0.395231	0.15474763	-2.5540358	0.01064823
7687	GRAMD4	1198.11493	-0.4305557	0.08119734	-5.3025836	1.14E-07
7692	GRB10	4882.48297	0.18338848	0.05667207	3.23595849	0.00121235
7700	GRHL1	49.64526	1.46602088	0.30708644	4.77396808	1.81E-06
7703	GRHPR	2673.22858	-0.6067119	0.0801354	-7.5710849	3.70E-14
7718	GRIK4	517.479022	-1.6067494	0.10537434	-15.248015	1.70E-52
7727	GRINA	11050.8	0.48109699	0.06164437	7.80439432	5.98E-15
7730	GRIPAP1	2030.59913	-0.3674652	0.07345545	-5.0025593	5.66E-07
7733	GRK5	905.353116	-1.522891	0.09153349	-16.637528	3.73E-62
7741	GRM5-AS1	3.23523113	4.01252741	1.52453713	2.63196437	0.00848928
7751	GRPEL2	1046.05707	0.60555434	0.08232612	7.35555555	1.90E-13
7756	GRWD1	2624.99996	0.32510229	0.07834765	4.14948377	3.33E-05
7759	GS1-124K5.1	586.787374	0.34257422	0.09493349	3.60857101	0.00030789
7761	GS1-259H13.	160.038832	1.19345879	0.17259769	6.91468558	4.69E-12
7765	GSAP	391.614146	-1.245951	0.12156072	-10.249618	1.19E-24
7770	GSDMC	58.3128134	-1.3131715	0.28908817	-4.5424604	5.56E-06
7771	GSDMD	1922.66339	-0.8047134	0.12895324	-6.2403505	4.37E-10
7772	GSE1	1723.92945	1.16053846	0.07727506	15.0182803	5.57E-51
7778	GSK3B	1329.1682	-0.2964727	0.07788176	-3.8067027	0.00014083
7779	GSKIP	1426.94578	0.71950182	0.07194783	10.0003269	1.52E-23
7780	GSN	13788.1691	-1.0722748	0.07585542	-14.135771	2.29E-45
7782	GSPT1	11352.3983	0.76916642	0.05829734	13.1938507	9.52E-40
7783	GSPT2	602.678347	1.02922154	0.09711115	10.5983871	3.03E-26
7793	GSTK1	3090.88815	-0.7601436	0.08045169	-9.4484479	3.44E-21
7794	GSTM1	464.23381	-1.1643188	0.12299742	-9.4662052	2.90E-21
7795	GSTM2	268.219409	-2.590799	0.15250947	-16.987791	1.01E-64
7798	GSTM4	1230.87703	-1.5823473	0.07987406	-19.81053	2.42E-87
7816	GTF2E1	450.899679	0.29743774	0.11643001	2.55464848	0.01062951
7819	GTF2F2	2204.81066	0.59617684	0.07573432	7.8719511	3.49E-15
7820	GTF2H1	4698.61691	0.42224457	0.063163	6.68499831	2.31E-11
7825	GTF2H3	2495.72603	0.35116944	0.06604122	5.3174279	1.05E-07
7837	GTF3C1	3876.45802	-0.2785298	0.07945223	-3.5056263	0.00045553
7838	GTF3C2	1911.81301	-0.4096622	0.08426302	-4.8617078	1.16E-06
7840	GTF3C3	1064.25681	-0.4310577	0.09190235	-4.6903881	2.73E-06
7841	GTF3C4	1752.0073	0.75232595	0.08597547	8.75047163	2.12E-18
7842	GTF3C5	2479.69586	-0.5919848	0.06927553	-8.5453664	1.28E-17
7844	GTPBP1	1664.09291	0.49120478	0.07223847	6.79976706	1.05E-11
7845	GTPBP10	1853.79298	1.15129311	0.07859531	14.6483689	1.38E-48
7846	GTPBP2	4098.93205	0.64768878	0.06011718	10.7737712	4.58E-27
7848	GTPBP4	7703.24309	0.94461372	0.07328087	12.8903184	5.10E-38
7852	GTSE1	1831.809	-0.7427255	0.08914781	-8.331394	7.99E-17

7861	GUCD1	2465.07974	-0.5421429	0.06557064	-8.2680738	1.36E-16
7871	GUF1	1805.58386	0.43122537	0.08164209	5.28189988	1.28E-07
7872	GUK1	5189.2342	-0.3214801	0.09139054	-3.5176513	0.00043538
7874	GUSB	2800.90567	-0.8333862	0.06716197	-12.408602	2.35E-35
7875	GUSBP1	103.671455	-0.5155184	0.20757681	-2.4835069	0.01300958
7886	GXYLT2	289.127899	-1.5646838	0.14368043	-10.890028	1.29E-27
7888	GYG2	468.071832	-0.623202	0.10409772	-5.9867018	2.14E-09
7890	GYLTL1B	18.1842093	2.14658971	0.55582869	3.86196278	0.00011248
7897	GZF1	1251.16155	0.47305592	0.07553536	6.26270824	3.78E-10
7902	GZMM	21.7418275	3.08587228	0.57110548	5.40333162	6.54E-08
7904	H1F0	11808.1128	0.19199766	0.05639714	3.40438633	0.00066313
7907	H1FX	3695.5012	-0.9373797	0.08327821	-11.256002	2.16E-29
7908	H1FX-AS1	66.2775578	-1.4488329	0.25980851	-5.5765413	2.45E-08
7913	H2AFV	7644.17021	-0.4078393	0.06002213	-6.7948154	1.08E-11
7914	H2AFX	6900.10203	-0.4929186	0.10987045	-4.4863625	7.24E-06
7925	H6PD	3532.70445	-0.5656382	0.07262683	-7.7882816	6.79E-15
7929	HACD1	1765.35301	-0.4637277	0.08453333	-5.4857378	4.12E-08
7930	HACD2	278.183334	-0.6399178	0.23441509	-2.7298489	0.00633634
7931	HACD3	6558.95367	-0.4915005	0.06704103	-7.33134	2.28E-13
7935	HADH	2210.86023	-0.5125138	0.06705369	-7.6433344	2.12E-14
7938	HAGH	1793.71419	-0.2787086	0.08706375	-3.2012015	0.00136856
7940	HAGLR	590.18194	-0.7444445	0.11700318	-6.3626006	1.98E-10
7957	HARBI1	192.438172	0.56149184	0.15876049	3.53672281	0.00040512
7958	HARS	3529.08001	-0.5412516	0.07052058	-7.6750873	1.65E-14
7965	HAUS1	1452.61423	-0.5726771	0.07603355	-7.5319008	5.00E-14
7966	HAUS2	1368.43675	0.40076798	0.07527115	5.32432387	1.01E-07
7968	HAUS4	1444.76145	-0.46782	0.10152396	-4.6079765	4.07E-06
7969	HAUS5	882.874579	-0.5579543	0.08557869	-6.5197801	7.04E-11
7976	HAX1	2610.27548	0.53737426	0.07364235	7.29708219	2.94E-13
7991	HCAR1	142.220985	-1.8200705	0.20990032	-8.6711183	4.28E-18
7996	HCFC1	3800.61522	-0.5272095	0.06638599	-7.9415772	2.00E-15
7998	HCFC1R1	2371.64156	-0.2697848	0.07045565	-3.829144	0.00012859
7999	HCFC2	589.236015	0.68868985	0.11094921	6.20725346	5.39E-10
8002	HCG17	15.6995927	1.86322613	0.57695531	3.22941153	0.00124045
8003	HCG18	2627.92564	0.97471314	0.07881054	12.367802	3.90E-35
8010	HCG4	26.9147284	2.43578219	0.47446056	5.13379273	2.84E-07
8019	HCN4	9.63848709	2.34091998	0.74820581	3.12871129	0.00175575
8029	HDAC2	8275.63236	0.27934481	0.05874224	4.75543355	1.98E-06
8030	HDAC3	4303.4655	-0.2647247	0.06582047	-4.0219199	5.77E-05
8032	HDAC5	987.032526	-0.9579513	0.09487022	-10.097492	5.67E-24
8033	HDAC6	2540.73771	-0.5469944	0.0703978	-7.7700485	7.85E-15
8034	HDAC7	2807.85072	-0.2437705	0.06291688	-3.8744849	0.00010685
8035	HDAC8	1047.57249	-0.3247866	0.08156425	-3.9819723	6.83E-05

8036	HDAC9	4312.10418	0.71780555	0.07891517	9.09591325	9.38E-20
8039	HDDC3	290.540172	-1.0797415	0.13507659	-7.9935502	1.31E-15
8040	HDGF	26603.2119	-0.3803344	0.06862867	-5.5419171	2.99E-08
8042	HDGFRP2	3696.42858	-0.3430488	0.07789578	-4.4039453	1.06E-05
8043	HDGFRP3	1055.3886	-0.3442285	0.08226152	-4.184563	2.86E-05
8049	HEATR1	2129.15207	0.3453626	0.07292984	4.73554557	2.18E-06
8050	HEATR3	735.002817	-0.5741967	0.09804078	-5.8567123	4.72E-09
8053	HEATR5B	1118.56957	0.32122087	0.09691344	3.31451327	0.00091803
8056	HEBP1	3006.97086	-0.1665942	0.07996705	-2.0832851	0.03722525
8065	HECW2	89.3773597	1.24160037	0.23417011	5.30212999	1.14E-07
8067	HEIH	779.384824	0.69536579	0.09519167	7.30490196	2.77E-13
8069	HELLS	1598.57261	-0.6963347	0.07481625	-9.3072664	1.31E-20
8072	HELZ	1154.29966	0.30141697	0.11388761	2.64661777	0.00813012
8073	HELZ2	1892.42109	-1.0061223	0.10566965	-9.5213926	1.71E-21
8085	HERC2P2	901.661039	-0.4482116	0.11196899	-4.0029976	6.25E-05
8091	HERC4	4277.30672	0.16015191	0.06027848	2.65686699	0.00788706
8094	HERPUD1	2235.63066	0.85912396	0.07328521	11.7230198	9.71E-32
8099	HES4	380.809556	0.49556225	0.21527707	2.30197412	0.02133663
8101	HES6	357.427537	-0.3891548	0.18463501	-2.107698	0.03505712
8102	HES7	172.092298	4.30185177	0.26194138	16.422956	1.31E-60
8106	HEXB	7654.45878	-0.5831241	0.05611998	-10.390668	2.73E-25
8107	HEXDC	700.178284	-1.1858159	0.1101412	-10.766324	4.96E-27
8108	HEXIM1	6657.61167	-0.566162	0.06263456	-9.0391302	1.58E-19
8109	HEXIM2	337.24999	-1.372083	0.16296324	-8.4195861	3.78E-17
8110	HEY1	21.6229976	1.89716482	0.45859901	4.13687075	3.52E-05
8113	HFE	643.104521	-0.5226445	0.11820187	-4.4216268	9.80E-06
8121	HGS	6939.02823	0.28408086	0.06561618	4.32943271	1.49E-05
8122	HGSNAT	2427.51638	-0.9579044	0.09883331	-9.6921203	3.26E-22
8126	HHIP	51.357165	-2.048936	0.30206889	-6.783009	1.18E-11
8127	HHIP-AS1	110.0905	-0.899836	0.2056227	-4.3761508	1.21E-05
8132	HHLA3	94.1976722	2.27464143	0.24770843	9.18273727	4.20E-20
8133	HIAT1	4667.82067	-0.3012155	0.06159773	-4.8900422	1.01E-06
8134	HIATL1	4204.1625	-0.2950621	0.06357826	-4.6409272	3.47E-06
8137	HIBCH	894.246922	-0.3579324	0.10417368	-3.4359195	0.00059055
8144	HIF1A-AS2	127.160506	0.69572778	0.19082044	3.64598137	0.00026637
8145	HIF1AN	2386.81739	-0.5130376	0.07186509	-7.1388993	9.41E-13
8147	HIGD1A	3575.46383	0.28042899	0.06804093	4.12147471	3.76E-05
8150	HIGD2A	2483.14681	-0.5165507	0.06340076	-8.1473897	3.72E-16
8154	HINFP	1293.59839	0.20215644	0.07511635	2.69124398	0.00711861
8159	HIP1R	3215.34935	0.37063932	0.07643233	4.84924807	1.24E-06
8160	HIPK1	2871.88552	0.69789974	0.08357184	8.3508962	6.77E-17
8166	HIRIP3	821.253629	-0.3799706	0.09762018	-3.8923363	9.93E-05
8240	HIVEP1	1471.64845	1.07339315	0.11877303	9.03734739	1.61E-19

8241	HIVEP2	1926.95895	0.57957903	0.11881308	4.87807427	1.07E-06
8243	HJURP	2479.42979	-0.6715101	0.07520563	-8.9289868	4.30E-19
8244	HK1	13233.8148	-0.6607077	0.05220893	-12.655071	1.05E-36
8245	HK2	3170.01343	0.53613882	0.06115457	8.76694608	1.84E-18
8248	HKR1	686.587789	0.23088664	0.10422677	2.21523346	0.02674404
8252	HLA-DMA	1389.78773	-0.8087492	0.0870267	-9.2931156	1.50E-20
8253	HLA-DMB	675.40552	-1.2851832	0.09731055	-13.207028	7.99E-40
8255	HLA-DOB	106.156928	1.89604879	0.24019794	7.89369299	2.93E-15
8256	HLA-DPA1	3545.93564	-0.2587465	0.06057111	-4.2717813	1.94E-05
8257	HLA-DPB1	2166.61211	-0.3985661	0.1056803	-3.7714324	0.00016231
8261	HLA-DQB1	282.531278	-0.3394291	0.12915701	-2.6280346	0.00858798
8262	HLA-DQB2	76.9700754	-0.5349442	0.23040172	-2.321789	0.0202443
8263	HLA-DRA	6516.45977	-0.489425	0.06509906	-7.5181588	5.56E-14
8269	HLA-E	16596.8007	0.56704618	0.05031652	11.269582	1.85E-29
8275	HLA-L	266.422654	0.35747936	0.13672434	2.6145992	0.00893322
8277	HLF	23.6992704	1.33989891	0.41782076	3.20687493	0.00134185
8280	HLX	196.592032	1.3945677	0.18101922	7.70397598	1.32E-14
8285	HMBS	2699.6692	-0.3386564	0.09432874	-3.5901718	0.00033046
8286	HMCES	1717.46804	-0.5661865	0.08894307	-6.3657181	1.94E-10
8290	HMG20B	4102.97367	-0.9109989	0.07876946	-11.565382	6.17E-31
8291	HMGA1	58792.6122	0.93621515	0.07198057	13.0064988	1.12E-38
8295	HMGB2	7498.51738	-0.6660911	0.06515871	-10.222595	1.57E-24
8296	HMGB3	1475.75343	-0.3094769	0.09178589	-3.3717269	0.00074698
8302	HMGCS1	1936.86145	-0.210697	0.07543985	-2.7929137	0.00522356
8304	HMGN1	3589.41629	0.4574198	0.09964987	4.59026999	4.43E-06
8305	HMGN2	1936.07054	0.14488423	0.07225081	2.00529564	0.04493145
8307	HMGN3	1235.68174	-0.5831743	0.11849076	-4.9216855	8.58E-07
8309	HMGN4	5405.34678	0.35952103	0.0646906	5.55754694	2.74E-08
8312	HMGXB4	1336.55355	-0.2101164	0.07809116	-2.6906547	0.0071312
8324	HN1	7638.76915	0.22428387	0.09035664	2.48220694	0.01305714
8325	HN1L	6041.59434	0.14487693	0.0613489	2.3615246	0.01819996
8333	HNMT	1350.10491	-1.5358874	0.08331929	-18.433755	7.04E-76
8342	HNRNPAB	11705.61	-0.1521076	0.05851865	-2.5993022	0.00934135
8343	HNRNPC	13843.0483	0.4897735	0.07333472	6.67860346	2.41E-11
8353	HNRNPH3	9220.48858	0.32799503	0.07012093	4.67756221	2.90E-06
8354	HNRNPK	21428.2888	0.42023053	0.06617467	6.35032278	2.15E-10
8357	HNRNPLL	1856.30092	0.51528804	0.06975696	7.38690499	1.50E-13
8359	HNRNPR	10697.5566	0.65046437	0.05310494	12.2486592	1.71E-34
8360	HNRNPU	30273.8353	0.41962934	0.05213841	8.04837307	8.39E-16
8366	HOMER1	490.352231	0.76189167	0.12475609	6.10704975	1.01E-09
8389	HOXA13	21.7983948	-0.9518424	0.42401445	-2.2448347	0.02477874
8407	HOXB8	32.1107444	1.00948778	0.4205274	2.40052794	0.01637144
8408	HOXB9	506.502587	1.48324342	0.11488901	12.9102293	3.94E-38

8425	HOXD11	4.49936634	-3.839455	1.3461501	-2.8521745	0.00434213
8437	HPCAL1	13377.5847	0.58503148	0.05943116	9.8438513	7.29E-23
8451	HPS6	1207.27489	-1.0066404	0.10471106	-9.613506	7.01E-22
8452	HPSE	1250.38945	-0.8544528	0.08728626	-9.7890863	1.25E-22
8463	HRAT17	21.1244653	4.02538893	0.67695571	5.94631061	2.74E-09
8466	HRAT92	18.5426151	-2.2112918	0.50730244	-4.3589221	1.31E-05
8468	HRCT1	13.667595	-2.2973342	0.61423302	-3.7401673	0.0001839
8470	HRH1	2725.65399	1.06523643	0.07826609	13.6104466	3.47E-42
8476	HRSP12	668.515737	-0.3018851	0.11156013	-2.7060302	0.00680929
8483	HS3ST3B1	926.926925	-0.7268034	0.09229988	-7.8743706	3.42E-15
8492	HSBP1L1	1160.0952	0.63514154	0.11750537	5.40521304	6.47E-08
8493	HSCB	318.944838	0.61031844	0.12984449	4.7003801	2.60E-06
8494	HSD11B1	57.4059243	-4.3160082	0.41957566	-10.286603	8.10E-25
8495	HSD11B1L	241.633016	-0.5854763	0.15791556	-3.7075276	0.00020929
8499	HSD17B11	4019.21966	-0.6356419	0.06549646	-9.7049805	2.87E-22
8505	HSD17B4	3803.60011	-0.3986298	0.06444028	-6.1860343	6.17E-10
8512	HSD3B7	1550.1652	-1.1858443	0.0856225	-13.849681	1.28E-43
8515	HSDL1	1555.25287	0.28114908	0.07665097	3.66791308	0.00024454
8517	HSF1	5386.30665	0.24599786	0.06370966	3.86123317	0.00011282
8518	HSF2	1749.56794	0.6193585	0.07033861	8.80538435	1.30E-18
8520	HSF4	329.241668	-0.6805645	0.12099462	-5.6247499	1.86E-08
8529	HSP90AB1	71420.8168	0.6722428	0.06393332	10.5147495	7.39E-26
8535	HSPA13	3365.63249	0.74439687	0.08285617	8.98420572	2.61E-19
8538	HSPA1B	2020.19428	-0.2688876	0.09745242	-2.7591682	0.00579487
8541	HSPA4	17307.7533	1.01471149	0.05594857	18.1365061	1.64E-73
8543	HSPA5	47615.1395	1.1770286	0.06877206	17.1149255	1.15E-65
8547	HSPA9	27209.884	1.07638994	0.05206656	20.6733442	6.02E-95
8555	HSPB8	1003.14312	0.86012711	0.08133691	10.5748686	3.90E-26
8558	HSPBP1	1828.73609	-0.3243586	0.1016241	-3.1917484	0.00141414
8560	HSPE1	3426.3323	-0.2055723	0.09926373	-2.0709706	0.03836155
8562	HSPG2	7224.8087	-0.3393506	0.09482629	-3.5786555	0.00034537
8571	HTR1D	302.141074	-0.5666229	0.13962246	-4.0582504	4.94E-05
8589	HTRA1	1120.44524	-1.5423338	0.09535287	-16.175012	7.57E-59
8590	HTRA2	1843.0531	0.46783174	0.09045981	5.17170837	2.32E-07
8591	HTRA3	164.217065	-2.2013361	0.20040975	-10.984177	4.55E-28
8593	HTT	3181.94623	-0.2967586	0.07308839	-4.0602701	4.90E-05
8598	HUS1B	13.5858644	2.92124008	0.66292536	4.40658973	1.05E-05
8599	HUWE1	21643.4955	0.20771764	0.08423485	2.46593471	0.01366562
8600	HVCN1	156.977365	-1.3530174	0.18297342	-7.3946119	1.42E-13
8602	HYAL2	1210.95804	-0.9336422	0.07819608	-11.939758	7.34E-33
8610	HYLS1	1378.11665	-0.9307999	0.11921918	-7.8074677	5.83E-15
8612	HYOU1	15222.3457	0.82552551	0.05722296	14.4264734	3.53E-47
8617	IARS	18796.7253	0.99852372	0.05596719	17.8412354	3.38E-71

8622	IBTK	4356.59446	0.84478172	0.07842433	10.7719345	4.67E-27
8629	ICAM5	192.822673	1.25672564	0.17454772	7.19989718	6.03E-13
8636	ICT1	1032.5578	0.21086875	0.09013834	2.33939015	0.01931525
8637	ID1	2626.86596	-1.7096214	0.08965731	-19.068399	4.62E-81
8638	ID2	243.655577	-1.0504077	0.13918451	-7.546872	4.46E-14
8640	ID3	856.090632	-0.8627619	0.12937233	-6.6688283	2.58E-11
8643	IDH1	3017.52352	-0.2242892	0.06818624	-3.289361	0.00100415
8644	IDH1-AS1	15.1505355	-1.3620372	0.58119271	-2.3435209	0.01910269
8645	IDH2	888.842481	-1.2363477	0.13103239	-9.4354358	3.89E-21
8647	IDH3B	3118.00703	-0.5106331	0.07749586	-6.5891663	4.42E-11
8648	IDH3G	1579.55328	-0.7111545	0.07768778	-9.1540083	5.49E-20
8652	IDNK	368.615173	0.90650194	0.12563183	7.21554325	5.37E-13
8657	IER2	3111.37167	0.21100224	0.09418641	2.24026202	0.02507392
8658	IER3	60435.0935	1.09514196	0.04859146	22.5377474	1.77E-112
8661	IER5L	1293.05468	-1.4518052	0.0835835	-17.369518	1.40E-67
8668	IFI30	2162.48559	0.17509098	0.08131719	2.15318545	0.03130411
8674	IFIT1	2045.76628	-1.9973373	0.07177496	-27.827773	2.00E-170
8679	IFITM1	599.859187	-2.9684381	0.12944363	-22.932284	2.21E-116
8681	IFITM2	662.697117	-1.4328537	0.13564398	-10.563341	4.41E-26
8682	IFITM3	4847.56162	-0.7698338	0.07481051	-10.290449	7.78E-25
8699	IFNAR1	3194.67058	0.38687251	0.0815211	4.74567313	2.08E-06
8714	IFRD1	3640.9084	0.45245265	0.05813366	7.78297143	7.08E-15
8715	IFRD2	7224.3032	0.57852607	0.09846252	5.87559709	4.21E-09
8716	IFT122	290.854089	-1.1300679	0.13233488	-8.5394557	1.35E-17
8717	IFT140	493.909884	-0.4075685	0.1031575	-3.9509345	7.78E-05
8724	IFT52	2109.11942	0.31397067	0.06847375	4.58527032	4.53E-06
8725	IFT57	2018.63927	0.62325419	0.08605909	7.24216554	4.42E-13
8728	IFT80	958.091267	-0.2207025	0.08309824	-2.6559222	0.00790919
8736	IGF1R	1234.15395	-0.8597187	0.09042887	-9.507127	1.96E-21
8740	IGF2BP2	3295.86976	0.24937992	0.08598525	2.90026395	0.00372849
8745	IGFBP1	422.874121	1.28780671	0.12544562	10.2658566	1.00E-24
8748	IGFBP4	69770.3502	-0.881589	0.09506698	-9.2733457	1.80E-20
8750	IGFBP6	2869.51085	-0.9582151	0.08606853	-11.133166	8.65E-29
8751	IGFBP7	13088.9083	-0.5974121	0.08386823	-7.1232234	1.05E-12
8758	IGFLR1	117.915219	-0.6940194	0.20704625	-3.3520017	0.0008023
8773	IGSF3	624.276563	2.0389077	0.10562082	19.3040316	4.97E-83
8780	IK	3299.41804	-0.5183695	0.06056025	-8.5595668	1.13E-17
8784	IKBKE	2326.31793	-0.9703534	0.06816101	-14.236195	5.46E-46
8787	IKZF2	123.537267	-0.8226343	0.19374519	-4.2459597	2.18E-05
8792	IL10RA	6.22527281	3.39863927	1.12694929	3.01578721	0.00256313
8795	IL11	781.14838	3.86988422	0.13345968	28.9966542	7.25E-185
8796	IL11RA	138.587254	-0.8252048	0.19090725	-4.3225429	1.54E-05
8797	IL12A	183.9953	1.6456863	0.17664074	9.31657247	1.20E-20

8811	IL17D	45.7330645	1.94962652	0.34760206	5.60878867	2.04E-08
8813	IL17RA	967.941349	-0.2191304	0.09039237	-2.4242136	0.01534158
8815	IL17RC	1204.45106	-1.2163503	0.1058471	-11.49158	1.45E-30
8816	IL17RD	1107.79486	-0.8873748	0.09104471	-9.7465824	1.91E-22
8817	IL17RE	241.83685	-1.5193607	0.16618914	-9.1423582	6.11E-20
8824	IL1A	7854.53046	1.43065878	0.065054	21.9918658	3.45E-107
8827	IL1R1	1152.87159	-0.3897942	0.1185607	-3.2877186	0.00101003
8830	IL1RAPL1	331.397429	0.75356739	0.12448867	6.05330079	1.42E-09
8833	IL1RL2	86.0204212	1.69058898	0.24608532	6.86993024	6.42E-12
8834	IL1RN	135.689687	2.22037221	0.2212675	10.0347869	1.07E-23
8838	IL20RB	193.444199	2.75711933	0.1881107	14.6568981	1.22E-48
8848	IL24	1624.38402	2.28521048	0.07698947	29.6821168	1.31E-193
8852	IL27RA	577.324923	-1.1144132	0.09607282	-11.599671	4.14E-31
8855	IL2RG	41.5580251	1.84932014	0.34688121	5.33127791	9.75E-08
8859	IL32	2689.59116	0.8487391	0.09288903	9.13712949	6.41E-20
8873	IL6	5928.82493	1.08945845	0.06772946	16.0854431	3.23E-58
8875	IL6ST	22009.4285	1.14444914	0.09688887	11.8119779	3.39E-32
8877	IL7R	1039.82897	0.5224768	0.09047433	5.77486247	7.70E-09
8881	ILDR2	1131.73108	-0.5486323	0.08762005	-6.2614926	3.81E-10
8882	ILF2	9314.71666	0.21334509	0.0624144	3.41820319	0.00063036
8885	ILK	7192.92285	-0.3901477	0.06816695	-5.7234147	1.04E-08
8887	ILVBL	2419.89008	-0.4380735	0.09242501	-4.7397722	2.14E-06
8891	IMP3	1834.5702	-0.9097187	0.12695282	-7.1658013	7.73E-13
8892	IMP4	2197.71736	0.70450195	0.09322624	7.5569059	4.13E-14
8894	IMPA2	1631.94522	-0.6391417	0.09067905	-7.0483937	1.81E-12
8898	IMPDH2	10201.6057	0.42075311	0.06401369	6.57286182	4.94E-11
8904	INAFM2	682.317293	0.8094821	0.10153779	7.97222516	1.56E-15
8906	INCENP	2572.93924	-0.5129249	0.0876904	-5.8492715	4.94E-09
8909	INF2	6143.91137	-0.2824277	0.0572684	-4.9316494	8.15E-07
8911	ING2	662.053923	-0.2010806	0.09677	-2.0779229	0.03771646
8913	ING4	453.230566	-1.0679113	0.10749957	-9.9340979	2.96E-23
8916	INHA	18.0708091	-1.6773324	0.48128393	-3.4851204	0.00049192
8921	INHBE	10.5020485	5.7637154	1.29534378	4.44956425	8.60E-06
8929	INO80D	489.206725	0.27268659	0.11342021	2.40421522	0.01620723
8932	INPP4A	1378.93313	-0.1684419	0.08367115	-2.0131417	0.04409972
8935	INPP5B	389.256723	1.07157465	0.13399969	7.99684444	1.28E-15
8937	INPP5E	1245.32384	-0.765322	0.08372616	-9.1407745	6.20E-20
8939	INPP5J	63.3864823	-2.0267944	0.27775641	-7.2970214	2.94E-13
8941	INPPL1	6595.56435	-0.1800307	0.07478463	-2.4073229	0.01606995
8945	INSIG1	3111.22824	-0.4035411	0.0644553	-6.2607906	3.83E-10
8953	INSR	1896.15301	-0.2901172	0.06813554	-4.2579429	2.06E-05
8955	INTS1	3398.72686	-0.9118769	0.08057542	-11.317061	1.08E-29
8957	INTS12	754.404826	0.64642946	0.08630786	7.48980993	6.90E-14

8959	INTS3	2851.66191	-0.6283446	0.07759426	-8.0978238	5.60E-16
8960	INTS4	775.688675	-0.3348367	0.09449877	-3.5432921	0.00039516
8962	INTS5	1165.65459	-0.4418785	0.08437852	-5.2368597	1.63E-07
8963	INTS6	973.354664	0.40093952	0.1117817	3.58680827	0.00033475
8965	INTS7	2144.70627	0.45203795	0.0757011	5.97135287	2.35E-09
8966	INTS8	1258.95578	-0.226107	0.07669529	-2.948121	0.00319712
8969	INVS	440.45007	-0.6193989	0.14733747	-4.203947	2.62E-05
8974	IPMK	2278.79929	0.80127463	0.09084907	8.81984419	1.15E-18
8978	IPO4	3011.01912	0.3386206	0.06451887	5.24839663	1.53E-07
8979	IPO5	12923.8816	0.79565356	0.05138789	15.4832878	4.50E-54
8980	IPO5P1	385.568482	0.82170772	0.11979854	6.85907963	6.93E-12
8981	IPO7	10712.4441	0.30682138	0.0867001	3.53888127	0.00040183
8983	IPO9	6708.15976	0.21420599	0.07495897	2.85764313	0.004268
8986	IPPK	969.06544	0.63505906	0.08363378	7.59333173	3.12E-14
8990	IQCB1	727.565985	0.76740522	0.09844582	7.79520408	6.43E-15
8992	IQCD	134.942396	-2.6843705	0.20776708	-12.920096	3.47E-38
8993	IQCE	1482.43725	-0.2007037	0.07267648	-2.7616041	0.00575182
9012	IQSEC2	1476.91241	-0.6895752	0.09116037	-7.5644186	3.90E-14
9016	IRAK1	12258.0325	0.89350533	0.05265425	16.969293	1.39E-64
9018	IRAK2	1794.78828	0.45844785	0.07398746	6.19629158	5.78E-10
9019	IRAK3	365.423589	-2.69918	0.14118296	-19.118313	1.78E-81
9020	IRAK4	511.09434	0.46257664	0.10848609	4.26392594	2.01E-05
9022	IRF1	4777.25343	1.18592484	0.06550651	18.1039225	2.97E-73
9026	IRF2BPL	2310.6058	-0.3000952	0.08282383	-3.6232954	0.00029087
9039	IRS2	2404.60983	0.52688862	0.06970131	7.55923505	4.05E-14
9052	ISG20L2	1183.5549	0.6727049	0.07684163	8.75443242	2.05E-18
9064	IST1	4484.1766	0.59429604	0.0775487	7.66352036	1.81E-14
9068	ISYNA1	569.792801	-1.7391543	0.12027919	-14.459312	2.19E-47
9070	ITFG1	1637.01404	-0.498379	0.07596445	-6.5606868	5.36E-11
9073	ITFG3	2417.45498	-0.5725158	0.0708793	-8.0773347	6.62E-16
9075	ITGA10	90.5909218	-1.0571119	0.21433595	-4.9320326	8.14E-07
9078	ITGA2B	12.9056558	-2.3113889	0.61191581	-3.7772989	0.00015854
9079	ITGA3	72124.1323	1.0378392	0.05817103	17.841171	3.39E-71
9081	ITGA5	6198.9231	0.35197962	0.06706127	5.24862724	1.53E-07
9097	ITGB2-AS1	69.6362216	-1.4359942	0.26964489	-5.3255012	1.01E-07
9098	ITGB3	660.644465	-0.9714281	0.12193793	-7.9665786	1.63E-15
9100	ITGB4	8140.22781	-1.2007007	0.07369857	-16.29205	1.12E-59
9101	ITGB5	9426.89224	-0.9156291	0.0545859	-16.774094	3.78E-63
9102	ITGB6	41.3836009	-1.8735111	0.40481037	-4.6281203	3.69E-06
9117	ITM2B	7531.77449	-0.737137	0.06145483	-11.994776	3.78E-33
9118	ITM2C	3145.42523	-0.8243715	0.06199875	-13.296582	2.42E-40
9123	ITPKB	279.93386	-0.9602116	0.12886399	-7.4513569	9.24E-14
9125	ITPKC	888.562279	0.61798788	0.09748475	6.33932844	2.31E-10

9129	ITPR3	29556.3007	1.15093281	0.08525843	13.4993436	1.58E-41
9130	ITPRIP	3213.2018	0.65268489	0.09543935	6.83873998	7.99E-12
9131	ITPRIPL1	3.21070674	3.00456809	1.46220207	2.05482412	0.039896
9132	ITPRIPL2	2890.39872	-0.3678641	0.07092654	-5.1865504	2.14E-07
9137	IVNS1ABP	4234.50448	0.30937475	0.06611025	4.67967885	2.87E-06
9144	IZUMO4	50.9235417	-1.8163411	0.29828039	-6.0893749	1.13E-09
9147	JADE3	742.442765	0.49472439	0.10550899	4.68893119	2.75E-06
9148	JAG1	2012.00157	0.82052521	0.09333043	8.79161551	1.47E-18
9150	JAGN1	2211.24264	0.43263447	0.08426125	5.13444198	2.83E-07
9151	JAK1	7403.7372	0.49255045	0.07791617	6.32154309	2.59E-10
9153	JAK3	39.6045627	-1.8576882	0.34279334	-5.4192657	5.98E-08
9160	JARID2	2138.00317	0.27442655	0.07876414	3.48415593	0.00049369
9162	JAZF1	328.102442	0.79914441	0.13809913	5.78674459	7.18E-09
9165	JDP2	908.337636	1.33829971	0.10349457	12.931111	3.00E-38
9167	JKAMP	3562.98021	-0.2823937	0.07300789	-3.8679892	0.00010974
9170	JMJD4	1247.2938	0.40572602	0.09236592	4.39259423	1.12E-05
9171	JMJD6	3433.15893	1.50087191	0.08216938	18.2655855	1.56E-74
9172	JMJD7	97.3780868	-0.6910344	0.22233147	-3.1081267	0.00188277
9174	JMJD8	1908.04369	-0.5172863	0.07727775	-6.6938583	2.17E-11
9176	JOSD1	2946.32011	0.27233177	0.08669604	3.14122502	0.00168243
9178	JPH1	129.318909	2.14808474	0.20512397	10.4721295	1.16E-25
9179	JPH2	345.955482	-0.4477486	0.12917942	-3.4660985	0.00052807
9184	JRKL	1569.69005	0.23458473	0.07695458	3.04835302	0.00230099
9188	JUN	7565.5274	0.88268726	0.05748679	15.3546103	3.30E-53
9190	JUND	7570.37315	-0.3934027	0.09384308	-4.1921334	2.76E-05
9191	JUP	4508.63364	-1.1012087	0.06116506	-18.003883	1.82E-72
9193	KALRN	166.921679	1.84640683	0.1935032	9.54199624	1.40E-21
9194	KANK1	587.934828	-0.8979922	0.10169499	-8.83025	1.04E-18
9195	KANK2	4735.13158	-0.2892537	0.06498976	-4.4507579	8.56E-06
9204	KARS	5075.71556	0.22458327	0.06563603	3.42164598	0.00062243
9205	KAT2A	3400.99294	-0.2740315	0.06505708	-4.2121709	2.53E-05
9206	KAT2B	1658.43359	-0.5241307	0.09633931	-5.4404654	5.31E-08
9208	KAT6A	2193.24917	0.37851178	0.10190337	3.71441873	0.00020367
9210	KAT7	1450.45086	0.25733068	0.10001886	2.57282153	0.01008732
9218	KAZALD1	163.267423	-2.097541	0.18454391	-11.36608	6.17E-30
9219	KAZN	524.139351	-0.2871028	0.12331199	-2.3282635	0.01989812
9224	KBTBD2	4066.58519	0.53622884	0.06149861	8.71936465	2.80E-18
9225	KBTBD3	184.980192	-0.5760793	0.15481542	-3.7210719	0.00019838
9226	KBTBD4	688.096529	-0.7527608	0.10592736	-7.1063871	1.19E-12
9227	KBTBD6	545.993613	-0.3356791	0.10728887	-3.1287411	0.00175557
9228	KBTBD7	149.141984	-0.9990911	0.17713137	-5.6403963	1.70E-08
9234	KCMF1	3011.28085	0.56282767	0.077583	7.25452318	4.03E-13
9246	KCNAB2	784.064161	0.51567235	0.09504017	5.4258359	5.77E-08

9247	KCNAB3	69.4269457	0.71126244	0.25079151	2.83607059	0.00456724
9252	KCNC3	45.7422756	1.17836787	0.33044339	3.5660204	0.00036244
9255	KCND1	99.469832	0.49049369	0.21580645	2.27284076	0.02303578
9257	KCND3	9.35041216	-2.4900918	0.7673467	-3.2450674	0.00117423
9262	KCNE3	17.6905331	-4.4422945	0.76792752	-5.7847835	7.26E-09
9286	KCNJ11	20.4980244	1.79313995	0.46927175	3.82111206	0.00013285
9289	KCNJ14	243.559928	1.85289927	0.1540282	12.0296105	2.48E-33
9301	KCNK1	7.85491026	-6.5192693	1.36788945	-4.7659329	1.88E-06
9312	KCNK5	38.0214737	-2.4011076	0.38206091	-6.28462	3.29E-10
9328	KCNN4	1918.4017	-0.6386033	0.08657323	-7.3764522	1.63E-13
9332	KCNQ1OT1	374.110381	-0.5785159	0.13009912	-4.4467317	8.72E-06
9336	KCNQ5	670.677698	1.22558861	0.12979888	9.44221274	3.65E-21
9338	KCNQ5-IT1	19.938745	1.29262997	0.48196912	2.68197672	0.00731886
9352	KCTD12	1072.15368	-0.6416199	0.08628956	-7.435661	1.04E-13
9356	KCTD16	3.12090096	3.95447998	1.52159483	2.59890472	0.00935217
9357	KCTD17	464.912836	0.22202026	0.11153235	1.99063559	0.04652096
9358	KCTD18	618.441555	-0.6063554	0.10122893	-5.9899411	2.10E-09
9360	KCTD2	2048.24415	0.17847189	0.07107185	2.51114751	0.01203394
9366	KCTD5	3918.5062	1.10805	0.07643011	14.4975578	1.26E-47
9370	KCTD9	1749.02565	0.3042323	0.07740105	3.93059683	8.47E-05
9371	KDELC1	247.823227	-0.5991884	0.15196801	-3.9428586	8.05E-05
9372	KDELC2	5671.05892	-0.872558	0.07476807	-11.670196	1.81E-31
9373	KDELR1	7681.12575	-0.5508285	0.06936191	-7.9413687	2.00E-15
9375	KDELR3	832.564266	-1.2341287	0.09389377	-13.143883	1.85E-39
9378	KDM1B	1067.28701	0.6524482	0.10449348	6.2439133	4.27E-10
9381	KDM3A	2744.86101	-0.203065	0.07874758	-2.5786826	0.00991779
9382	KDM3B	3783.19875	-0.2679725	0.07599725	-3.5260815	0.00042176
9383	KDM4A	1695.3079	0.55460834	0.07632849	7.2660727	3.70E-13
9385	KDM4B	2718.71821	-0.2144992	0.08473591	-2.5313846	0.01136132
9390	KDM5B	3814.20229	0.42109754	0.06500995	6.47743232	9.33E-11
9393	KDM6A	1724.12567	0.22774925	0.09140887	2.49154427	0.01271891
9394	KDM6B	1111.67477	1.40259412	0.11320683	12.3896595	2.97E-35
9395	KDM7A	712.726567	1.15138008	0.10165529	11.3263171	9.72E-30
9400	KEL	19.3479697	2.08824175	0.52612394	3.96910609	7.21E-05
9404	KHDC1	580.126276	-0.5352076	0.11928968	-4.4866214	7.24E-06
9407	KHDRBS1	11048.8977	0.6010163	0.06108104	9.83965461	7.60E-23
9409	KHDRBS3	104.170602	1.26478658	0.20862239	6.0625638	1.34E-09
9410	KHK	344.247241	-0.9763319	0.13483366	-7.2410105	4.45E-13
9412	KHSRP	7280.35345	0.29993469	0.11573271	2.59161546	0.00955265
9413	KIAA0020	3662.11341	1.00023938	0.06431856	15.5513338	1.56E-54
9416	KIAA0100	11869.9239	-0.1928132	0.07690827	-2.5070538	0.01217422
9420	KIAA0195	2104.05084	-0.6883772	0.0779359	-8.8326081	1.02E-18
9421	KIAA0196	961.047677	-0.3649722	0.09789469	-3.7282121	0.00019284

9428	KIAA0368	9624.36251	0.46397798	0.05161898	8.98851469	2.51E-19
9431	KIAA0430	1302.91495	-0.3752174	0.08207421	-4.5716844	4.84E-06
9432	KIAA0513	512.514204	0.63642554	0.1041237	6.11220633	9.83E-10
9435	KIAA0753	1077.52065	0.35817661	0.0940541	3.80819782	0.00013998
9439	KIAA0895L	267.306408	-0.6587494	0.13756479	-4.7886479	1.68E-06
9440	KIAA0907	3487.28004	0.73807068	0.06839583	10.7911657	3.79E-27
9442	KIAA0930	2880.7281	-0.3754726	0.06789241	-5.5304061	3.19E-08
9445	KIAA1033	4874.73537	0.48044538	0.08883824	5.408092	6.37E-08
9446	KIAA1107	184.133668	-0.6394818	0.20207974	-3.1645025	0.00155348
9450	KIAA1161	557.722174	-0.2140235	0.10046023	-2.1304304	0.0331361
9454	KIAA1211L	78.3076094	-4.2340779	0.38783296	-10.917272	9.53E-28
9455	KIAA1217	2971.65496	-0.7113292	0.09618605	-7.3953467	1.41E-13
9457	KIAA1279	3299.65814	-0.8224352	0.06439827	-12.771077	2.38E-37
9458	KIAA1324	119.063668	-1.766997	0.22148997	-7.9777745	1.49E-15
9468	KIAA1524	2782.12758	0.32698384	0.08085525	4.0440646	5.25E-05
9472	KIAA1586	1047.12688	0.31209519	0.09729957	3.20756998	0.00133861
9478	KIAA1715	3651.05806	-0.148568	0.06381878	-2.3279665	0.01991388
9481	KIAA1841	486.906044	-0.3326252	0.11166188	-2.9788608	0.00289322
9482	KIAA1875	168.707887	1.15354361	0.16655116	6.92606156	4.33E-12
9490	KIDINS220	2546.28707	0.21999559	0.08441153	2.60622668	0.00915459
9494	KIF13B	1910.89282	-0.2420052	0.07979097	-3.0329893	0.00242144
9496	KIF15	1349.66878	-0.5062803	0.07716175	-6.5612857	5.33E-11
9498	KIF17	116.512231	3.12224411	0.25519967	12.2345148	2.03E-34
9500	KIF18B	1500.43075	-0.6533423	0.06894406	-9.4764122	2.63E-21
9505	KIF20A	2372.16676	-1.0076669	0.09348122	-10.779351	4.31E-27
9509	KIF22	2319.02032	-0.5840045	0.09130942	-6.3958848	1.60E-10
9510	KIF23	4467.03261	-0.1776647	0.05876915	-3.0230944	0.00250204
9517	KIF2A	3581.36201	0.48838761	0.0892266	5.4735649	4.41E-08
9523	KIF4A	3625.15537	-0.1521626	0.0673773	-2.2583654	0.02392289
9528	KIF6	3.09107398	-5.1634629	1.6250214	-3.1774738	0.00148564
9532	KIFAP3	1023.74456	0.50615194	0.08843703	5.72330315	1.04E-08
9533	KIFC1	3458.27951	-0.448346	0.06260243	-7.1617998	7.96E-13
9536	KIN	740.444995	0.35617981	0.08534251	4.17353323	3.00E-05
9553	KIRREL	5234.61921	-0.729803	0.09068188	-8.0479478	8.42E-16
9562	KIZ	339.397356	-0.4042496	0.13743483	-2.9413911	0.00326742
9571	KLF10	5054.85764	2.45892013	0.0674153	36.4742155	2.84E-291
9572	KLF11	1158.57607	0.49977326	0.07574084	6.59846481	4.15E-11
9578	KLF17	45.152064	-0.7491168	0.31945548	-2.3449802	0.01902809
9582	KLF4	3604.30576	1.20139879	0.06592651	18.2233023	3.37E-74
9583	KLF5	2847.97652	1.10239053	0.07641789	14.4258178	3.56E-47
9594	KLHDC7B	4.21223052	3.43304321	1.30258411	2.63556355	0.00839977
9596	KLHDC8B	757.483733	-0.4263629	0.10348627	-4.1199943	3.79E-05
9597	KLHDC9	44.3497724	-1.5798636	0.31863248	-4.9582627	7.11E-07

9606	KLHL18	1357.32428	0.55304696	0.09719487	5.69008364	1.27E-08
9609	KLHL21	4631.518	1.27163463	0.08121354	15.6579143	2.93E-55
9610	KLHL22	755.10816	-0.6923976	0.10138204	-6.8295883	8.52E-12
9612	KLHL24	1292.20123	-0.9877984	0.07384273	-13.377058	8.23E-41
9614	KLHL26	382.164384	-0.4351839	0.11104041	-3.919149	8.89E-05
9616	KLHL29	926.793421	0.88629227	0.090891	9.75115565	1.82E-22
9624	KLHL36	1223.19587	-0.5152517	0.07454562	-6.9118973	4.78E-12
9629	KLHL42	958.787667	-0.6471027	0.08387148	-7.7154089	1.21E-14
9635	KLHL8	1137.84486	0.50224615	0.08998932	5.58117516	2.39E-08
9657	KLRC1	14.4756088	-1.4058459	0.57231834	-2.4564054	0.01403347
9658	KLRC2	140.743821	-0.844714	0.17805397	-4.7441457	2.09E-06
9659	KLRC3	128.864324	-0.5364012	0.2082239	-2.576079	0.00999278
9669	KMT2A	5274.59742	0.49327005	0.09368173	5.26538179	1.40E-07
9676	KNDC1	138.325994	-1.3475463	0.19101751	-7.0545695	1.73E-12
9678	KNOP1	1067.29784	0.28125277	0.07909457	3.55590466	0.00037668
9683	KPNA3	3329.00164	0.62457455	0.07983137	7.82367278	5.13E-15
9684	KPNA4	4092.89732	0.53202984	0.05957797	8.92997655	4.26E-19
9685	KPNA5	123.190643	0.60651066	0.21157958	2.86658409	0.00414928
9686	KPNA6	4810.96864	0.6364585	0.06823712	9.32715986	1.09E-20
9687	KPNA7	9.16440511	-3.7629491	0.96786641	-3.8878807	0.00010112
9688	KPNB1	26660.6598	0.61829677	0.05933696	10.4200954	2.01E-25
9690	KPTN	479.794843	-0.5410976	0.1226546	-4.4115553	1.03E-05
9694	KRBOX1	306.222894	-0.4978216	0.14719309	-3.382099	0.00071934
9696	KRBOX4	376.66256	0.65647872	0.1185756	5.53637255	3.09E-08
9702	KRR1	4130.98717	0.82289713	0.0633932	12.9808429	1.57E-38
9704	KRT10	1499.50981	0.29807508	0.0922059	3.23271143	0.00122621
9708	KRT15	117.874312	-0.9165574	0.21778871	-4.2084706	2.57E-05
9717	KRT19	9344.25117	-0.5659984	0.10745864	-5.2671281	1.39E-07
9746	KRT7	22457.7131	-0.501524	0.08444816	-5.9388383	2.87E-09
9756	KRT79	7.26790631	2.00968015	0.86332803	2.32782915	0.01992118
9757	KRT8	11321.1958	-0.7650287	0.05531138	-13.831307	1.65E-43
9758	KRT80	8455.98633	-0.4228949	0.06421504	-6.5856047	4.53E-11
9759	KRT81	1132.08747	-1.7848045	0.09529197	-18.729852	2.83E-78
9764	KRT86	118.798693	-1.8604487	0.24700895	-7.5319083	5.00E-14
9847	KRTAP5-AS1	3.46714754	4.12095016	1.51339576	2.7229825	0.00646955
9867	KTN1	19540.01	0.44196902	0.05353468	8.2557517	1.51E-16
9868	KTN1-AS1	86.3205358	0.82386689	0.24339167	3.38494285	0.00071193
9873	L1CAM	5385.40976	1.84551005	0.06839332	26.9837782	2.29E-160
9883	LACC1	605.625703	0.28717058	0.11748211	2.44437714	0.01451025
9886	LACTB	2936.84886	-0.328571	0.07391428	-4.4452979	8.78E-06
9898	LAMA3	2139.95831	-0.8520153	0.11205004	-7.6038825	2.87E-14
9899	LAMA4	68.7924815	-3.0179496	0.30125396	-10.017958	1.27E-23
9900	LAMA5	13836.6148	-0.3044627	0.08320238	-3.6593026	0.0002529

9903	LAMB2	13327.2242	-0.1572561	0.06931491	-2.2687191	0.02328541
9905	LAMB3	35156.2373	0.87316862	0.08031267	10.8721159	1.57E-27
9908	LAMC2	7426.40888	0.63723412	0.06316486	10.0884285	6.22E-24
9911	LAMP2	12276.1775	-0.1747971	0.08008158	-2.1827382	0.0290551
9912	LAMP3	239.930989	2.27239239	0.19154627	11.8634122	1.83E-32
9917	LAMTOR3	1520.1264	0.22622012	0.09296681	2.43334281	0.01496013
9918	LAMTOR4	1422.4625	-0.4073587	0.14768367	-2.7583191	0.00580995
9919	LAMTOR5	4174.18632	-0.6442726	0.08884271	-7.2518338	4.11E-13
9921	LANCL1	967.022031	-1.3482637	0.08651189	-15.584722	9.25E-55
9934	LARP4B	5638.41799	0.46683318	0.06002878	7.77682249	7.44E-15
9936	LARP7	2395.80769	-0.3740799	0.0817955	-4.5733553	4.80E-06
9937	LARS	13386.8411	1.17617741	0.05156812	22.8082279	3.80E-115
9940	LAS1L	3115.78177	0.44263078	0.06672125	6.63403014	3.27E-11
9941	LASP1	26742.1377	-0.1354127	0.05091884	-2.6593822	0.00782841
9944	LATS1	1402.74743	0.47024596	0.10340145	4.54776934	5.42E-06
9947	LAYN	4270.75506	-0.6224862	0.0673569	-9.2416094	2.43E-20
9948	LBH	848.431327	-1.6594483	0.09130129	-18.175517	8.07E-74
9955	LBX2-AS1	189.789932	0.76777827	0.15102581	5.08375542	3.70E-07
9957	LCA5	134.370264	1.65075759	0.18699557	8.82778958	1.07E-18
9984	LCMT2	628.690911	0.25988069	0.09386929	2.76853787	0.00563084
9989	LCN2	6546.93551	-0.2563177	0.07898881	-3.2449875	0.00117456
10001	LDB1	1305.35513	-0.6556501	0.07956831	-8.2400913	1.72E-16
10007	LDHB	21646.2097	-0.3160713	0.10505777	-3.0085476	0.002625
10009	LDHD	57.1104404	-1.7231207	0.35742522	-4.8209264	1.43E-06
10013	LDLRAD3	1886.19401	0.256402	0.08132119	3.15295444	0.00161627
10017	LDOC1	329.923971	-5.4439922	0.23876464	-22.800663	4.52E-115
10018	LDOC1L	1925.9471	-0.4154865	0.07551821	-5.5018056	3.76E-08
10022	LEF1	28.3508755	1.3570108	0.40765976	3.32878281	0.00087226
10033	LENG1	400.849924	-0.4682977	0.12710679	-3.6842854	0.00022935
10040	LEPROT	4598.26299	0.62675907	0.06588046	9.51358112	1.84E-21
10046	LFNG	425.943907	-1.2795589	0.1121979	-11.404482	3.97E-30
10055	LGALS3BP	28176.1751	-0.3233872	0.06538088	-4.9462042	7.57E-07
10061	LGALS9	58.9382623	-0.6709136	0.26902961	-2.4938283	0.01263737
10070	LGR4	1871.01789	-0.2080943	0.09126356	-2.2801469	0.02259898
10078	LHFPL2	5039.58049	0.42869762	0.06003549	7.1407361	9.28E-13
10084	LHPP	145.691022	-1.4927338	0.19982065	-7.4703681	8.00E-14
10099	LIG1	2520.47751	-0.7877983	0.07321298	-10.760364	5.30E-27
10101	LIG4	1020.90785	0.96292118	0.09008769	10.68871	1.15E-26
10115	LIMA1	8898.04459	0.19305597	0.07458479	2.5884093	0.00964203
10117	LIMD1	2977.29093	-0.1783947	0.06276764	-2.8421451	0.00448111
10120	LIME1	526.935661	-0.4945656	0.12515865	-3.9515098	7.77E-05
10121	LIMK1	6889.52635	-0.1744431	0.05599562	-3.1152997	0.00183758
10124	LIMS2	1122.67287	-0.383189	0.09658053	-3.9675596	7.26E-05

10134	LIN7B	94.9345986	-0.7021462	0.22733745	-3.0885636	0.00201127
10136	LIN9	744.434036	-0.2956741	0.09649084	-3.0642717	0.00218201
10137	LINC-PINT	1164.49052	0.84212714	0.09509378	8.85575374	8.31E-19
10145	LINC00094	876.152856	-0.4650839	0.09103033	-5.1091091	3.24E-07
10154	LINC00152	5627.64635	0.64440718	0.09656595	6.67323411	2.50E-11
10162	LINC00173	113.379132	-0.9998391	0.19752879	-5.0617387	4.15E-07
10164	LINC00176	80.3646811	0.69768314	0.24681056	2.82679617	0.00470162
10204	LINC00294	424.349297	-0.29778	0.14329047	-2.0781565	0.03769495
10215	LINC00310	35.6474853	0.71771245	0.34691636	2.06883427	0.03856164
10236	LINC00341	39.3522369	-1.0304869	0.3378923	-3.0497496	0.00229032
10239	LINC00346	44.6594288	-1.2817188	0.31928087	-4.0143926	5.96E-05
10297	LINC00460	29.3266204	2.04395153	0.44979868	4.5441475	5.52E-06
10362	LINC00565	22.9031031	1.83653702	0.52530246	3.49615159	0.00047202
10411	LINC00638	154.497477	0.82712117	0.16931818	4.88501097	1.03E-06
10424	LINC00657	12097.2123	-0.1553895	0.0550691	-2.8217179	0.00477672
10428	LINC00662	158.217502	0.9625965	0.18142546	5.30574098	1.12E-07
10431	LINC00665	132.276936	0.68456341	0.2061489	3.32072305	0.00089785
10432	LINC00667	774.028247	0.37143361	0.09671314	3.8405701	0.00012275
10434	LINC00669	87.2222241	-1.3077275	0.21811517	-5.9955824	2.03E-09
10437	LINC00672	36.9705454	-1.5847831	0.347414	-4.561656	5.08E-06
10439	LINC00674	1958.71976	-0.2475624	0.07907008	-3.1309233	0.00174258
10458	LINC00702	30.0238307	1.31046821	0.39647339	3.30531185	0.00094871
10463	LINC00707	26.9799486	2.35277097	0.49375622	4.76504575	1.89E-06
10474	LINC00842	43.0746313	-1.50391	0.37725301	-3.9864759	6.71E-05
10489	LINC00865	40.4827522	0.84221536	0.32769498	2.57011979	0.01016634
10500	LINC00883	693.365929	-0.3505184	0.09823879	-3.5680246	0.00035968
10523	LINC00909	325.927392	1.0120563	0.12753398	7.9355818	2.10E-15
10534	LINC00926	56.9104103	-0.6002093	0.26474733	-2.2671022	0.02338398
10550	LINC00944	22.3446553	1.67991299	0.44124634	3.80719981	0.00014055
10578	LINC00997	231.600974	-0.6924132	0.15737195	-4.3998517	1.08E-05
10579	LINC00998	1040.89119	0.6561001	0.08480547	7.73653086	1.02E-14
10584	LINC01003	380.284753	-0.9437297	0.1447175	-6.5211858	6.98E-11
10639	LINC01088	980.137311	3.35986366	0.10873848	30.8985706	1.25E-209
10662	LINC01116	582.630772	-1.2349403	0.09843921	-12.545208	4.22E-36
10670	LINC01124	84.3704323	-0.6709471	0.23022536	-2.9143057	0.00356481
10674	LINC01128	484.794548	0.79950262	0.11193944	7.14227807	9.18E-13
10681	LINC01138	150.983032	0.41991452	0.18951136	2.21577497	0.02670692
10705	LINC01179	5.3190972	2.37814225	1.04031807	2.2859761	0.02225566
10723	LINC01204	27.6962872	1.31535878	0.4027761	3.26573192	0.00109182
10739	LINC01224	849.980324	1.46878359	0.10446295	14.0603307	6.66E-45
10748	LINC01234	21.8832351	-4.2554395	0.6664136	-6.3855832	1.71E-10
10759	LINC01252	27.4646678	-1.7755292	0.4118267	-4.3113503	1.62E-05
10790	LINC01296	22.424876	3.89615595	0.62345347	6.24931311	4.12E-10

10832	LINC01356	50.1113356	0.74481832	0.3443902	2.16271636	0.030563
10928	LINC01503	84.7570267	-1.333346	0.25461753	-5.2366621	1.64E-07
10945	LINC01521	95.4194343	-1.4762015	0.23625318	-6.2483877	4.15E-10
10952	LINC01530	14.4893805	-1.4618009	0.56919791	-2.5681769	0.0102235
10979	LINC01561	29.0898138	-2.4976026	0.43023872	-5.8051553	6.43E-09
10992	LINC01578	2156.62919	-0.4196971	0.11408772	-3.678723	0.0002344
10996	LINC01583	19.010516	2.60741097	0.56628917	4.60438075	4.14E-06
11030	LIPA	3955.8776	-0.8347465	0.06426602	-12.988924	1.41E-38
11032	LIPE	193.696743	-0.385901	0.16174171	-2.3859091	0.01703696
11036	LIPH	867.022469	-1.1071509	0.09593717	-11.540376	8.26E-31
11042	LIPT1	222.970181	0.55738737	0.14651287	3.80435781	0.00014217
11044	LITAF	9885.17503	-0.3736716	0.07010958	-5.3298222	9.83E-08
11046	LIX1L	1579.66154	-0.8380708	0.07171277	-11.686492	1.49E-31
11051	LLGL1	2559.53814	-1.0111908	0.07596805	-13.310738	2.00E-40
11052	LLGL2	444.292208	-0.8992004	0.11644706	-7.7219671	1.15E-14
11053	LLPH	743.57529	0.50287271	0.10005247	5.0260901	5.01E-07
11057	LMAN2	5693.78038	-0.4116614	0.07881763	-5.2229602	1.76E-07
11061	LMBRD1	1479.09248	-0.3667746	0.07532076	-4.869502	1.12E-06
11063	LMCD1	239.347184	0.44064898	0.13840201	3.1838338	0.00145338
11065	LMF1	141.083835	-0.4175993	0.17433596	-2.3953707	0.01660357
11067	LMF2	3824.93163	-0.3219742	0.08322175	-3.8688704	0.00010934
11068	LMLN	1277.39709	0.36842874	0.08818122	4.17808637	2.94E-05
11071	LMNB1	3909.30389	-0.2458362	0.07481523	-3.2859107	0.00101653
11074	LMNTD2	357.654486	-1.1087603	0.14978124	-7.4025311	1.34E-13
11079	LMO7	2419.59708	-0.5311213	0.07971709	-6.6625776	2.69E-11
11092	LNX1	223.649841	-0.6157737	0.14196647	-4.3374587	1.44E-05
11109	LOC1001283	196.079183	0.83952461	0.1787167	4.6975164	2.63E-06
11138	LOC1001295	228.72128	-1.0251609	0.1475914	-6.9459395	3.76E-12
11151	LOC1001300	132.979598	-1.1594947	0.19735518	-5.8751676	4.22E-09
11156	LOC1001303	22.9569687	1.00899231	0.41522841	2.42996936	0.0151001
11166	LOC1001307	164.608411	1.66093384	0.17782442	9.34030214	9.61E-21
11256	LOC1002886	422.993884	-0.273887	0.13747491	-1.9922693	0.04634152
11259	LOC1002887	83.0165551	0.88292475	0.22224362	3.9727788	7.10E-05
11276	LOC1002941	1573.22326	0.57705238	0.07686055	7.50778314	6.01E-14
11279	LOC1003350	51.9102492	2.50136146	0.33278884	7.51636219	5.63E-14
11280	LOC1003792	79.6696564	1.1347153	0.23727114	4.78235694	1.73E-06
11326	LOC1005061	326.873602	-0.8915662	0.17335317	-5.1430626	2.70E-07
11331	LOC1005061	102.156715	-0.9581408	0.19812836	-4.8359602	1.33E-06
11360	LOC1005064	18.9145315	1.1028439	0.47770952	2.30860776	0.02096536
11363	LOC1005065	1786.84732	1.23508715	0.07534698	16.3919935	2.18E-60
11366	LOC1005066	115.325079	0.42781037	0.20083014	2.13021	0.03315428
11382	LOC1005068	220.165537	0.61502683	0.18129159	3.39247295	0.00069265
11386	LOC1005068	42.65142	-1.2794492	0.36274684	-3.5271132	0.00042012

11389	LOC1005070	403.371364	-0.5388496	0.12318747	-4.3742244	1.22E-05
11390	LOC1005070	6.89945194	-2.7055105	0.90882201	-2.9769421	0.00291139
11403	LOC1005072	12.7533125	1.29637373	0.56833478	2.28100369	0.02254823
11419	LOC1005074	33.3147951	1.16461154	0.37166801	3.13347264	0.00172751
11437	LOC1005076	131.346057	-1.717827	0.20085382	-8.5526229	1.20E-17
11506	LOC1019269	2.44919067	3.58283078	1.60319967	2.2348001	0.02543048
11517	LOC1019270	206.356729	-0.9978254	0.14868856	-6.7108418	1.94E-11
11518	LOC1019270	7.69635755	5.30511922	1.34516803	3.94383385	8.02E-05
11585	LOC1019273	18.7498608	-1.9025509	0.50778421	-3.7467705	0.00017913
11621	LOC1019274	34.8183064	-1.5883381	0.38493251	-4.1262769	3.69E-05
11625	LOC1019274	34.0773039	2.06667158	0.40748803	5.07173571	3.94E-07
11637	LOC1019275	26.4560361	0.77281324	0.39101482	1.97642953	0.04810615
11662	LOC1019276	1.47823421	3.9063227	1.95906476	1.99397323	0.04615498
11675	LOC1019277	173.850596	0.59499126	0.16653407	3.57278998	0.0003532
11683	LOC1019277	187.108873	0.43409681	0.16374578	2.65104117	0.00802441
11721	LOC1019279	10.4357332	-2.2862527	0.75030608	-3.0470934	0.00231066
11745	LOC1019280	119.529117	0.75045624	0.19281024	3.89220109	9.93E-05
11748	LOC1019281	37.2582037	-0.8672692	0.40145794	-2.1602991	0.03074952
11785	LOC1019282	7.86699067	2.45313041	0.85136162	2.88142003	0.00395888
11822	LOC1019284	12.7852672	1.81664399	0.59122135	3.07269689	0.00212134
11888	LOC1019287	11.8770477	1.31928575	0.60860392	2.1677247	0.03017964
11896	LOC1019288	99.2740223	0.81638134	0.2271803	3.59353924	0.00032622
11989	LOC1019293	1.79641624	4.22430054	1.89437923	2.22991283	0.02575323
12029	LOC1019295	90.152276	-1.0960168	0.21470481	-5.104761	3.31E-07
12057	LOC1019296	3.57378325	3.23444553	1.4041127	2.30355122	0.02124785
12063	LOC1019297	81.568939	1.14690989	0.2413655	4.75175563	2.02E-06
12064	LOC1019297	198.820044	1.53538983	0.1727618	8.88732236	6.26E-19
12110	LOC1026064	185.489923	0.7689879	0.16296342	4.71877611	2.37E-06
12132	LOC1027237	18.4040127	1.0677766	0.51644954	2.06753326	0.03868393
12170	LOC1027243	30.9965679	-0.8706194	0.35460446	-2.4551846	0.01408122
12179	LOC1027245	24.8426472	1.0406736	0.42265487	2.46223024	0.0138076
12180	LOC1027245	3.94791663	2.67013396	1.28815944	2.07282879	0.03818822
12205	LOC1027249	102.631995	-1.121831	0.23252709	-4.8245176	1.40E-06
12217	LOC1033449	111.831681	-0.5929112	0.20701607	-2.8640829	0.00418218
12219	LOC1036110	178.934597	-0.414898	0.20270735	-2.0467832	0.04067938
12251	LOC146880	858.858518	-0.7336881	0.09965308	-7.3624231	1.81E-13
12252	LOC148413	673.901355	0.63117561	0.1148356	5.49634086	3.88E-08
12264	LOC152225	250.86272	0.82968773	0.13571557	6.11343068	9.75E-10
12266	LOC153684	33.3283091	-1.171081	0.37208224	-3.1473713	0.00164746
12284	LOC254896	34.1262141	-0.7966415	0.34406731	-2.3153654	0.02059294
12291	LOC283140	35.9991198	0.6531087	0.33271585	1.96296237	0.04965055
12300	LOC283575	7.02448215	1.82151211	0.87634651	2.07852956	0.03766061
12303	LOC283710	5.03091643	-2.8233188	1.04007702	-2.7145286	0.00663702

12310	LOC284080	32.7965972	0.73240818	0.35435305	2.06688832	0.03874468
12385	LOC374443	460.871847	0.78882079	0.14035886	5.62002866	1.91E-08
12409	LOC389906	320.30242	0.49712	0.12555422	3.9594049	7.51E-05
12418	LOC399815	80.6994877	1.35988775	0.23129113	5.87955002	4.11E-09
12430	LOC400706	11.5739034	-2.7114978	0.69489511	-3.9020246	9.54E-05
12513	LOC642846	107.943208	-0.6128269	0.24015198	-2.5518294	0.0107159
12549	LOC646029	15.8191736	4.85651506	0.9417151	5.15709589	2.51E-07
12569	LOC652276	108.77454	1.85799288	0.2304595	8.06212325	7.50E-16
12586	LOC728323	54.312909	0.73736716	0.33748478	2.1848901	0.02889691
12587	LOC728392	229.337677	-0.3889534	0.15865122	-2.4516256	0.01422126
12588	LOC728485	21.2273745	0.89511522	0.43759027	2.04555555	0.04080012
12589	LOC728554	310.657491	-0.8028781	0.15507026	-5.1775117	2.25E-07
12594	LOC728743	288.792477	-1.0802643	0.13565021	-7.9636025	1.67E-15
12617	LOC730101	500.173983	-0.715272	0.10727956	-6.6673651	2.60E-11
12618	LOC730102	299.698821	-1.4672772	0.13549598	-10.828935	2.51E-27
12636	LONP2	1250.25118	-0.5064749	0.09567039	-5.2939568	1.20E-07
12639	LONRF3	458.276645	1.02809928	0.13491385	7.62041335	2.53E-14
12643	LOXL1	267.007905	-1.7037536	0.136759	-12.458073	1.26E-35
12644	LOXL1-AS1	426.789785	0.38333064	0.11249908	3.40741142	0.00065582
12646	LOXL3	85.6150737	0.94712697	0.21876576	4.32941131	1.50E-05
12647	LOXL4	6283.13973	-1.0485667	0.05555547	-18.874228	1.86E-79
12649	LPAL2	104.684238	-0.8191365	0.21514432	-3.8073815	0.00014045
12656	LPCAT1	5653.65041	-0.8218859	0.06058933	-13.564863	6.47E-42
12659	LPCAT4	1769.96829	-0.6508708	0.07054486	-9.2263388	2.80E-20
12663	LPIN3	1159.13662	-0.5892717	0.07844708	-7.5117094	5.84E-14
12670	LPPR2	2265.55352	-0.5525597	0.0782372	-7.0626208	1.63E-12
12677	LRCH1	1068.66181	0.39632322	0.09156497	4.32832781	1.50E-05
12680	LRCH4	2630.52988	-0.653396	0.07124159	-9.1715533	4.66E-20
12684	LRFN3	895.124855	-0.5186638	0.12748513	-4.0684256	4.73E-05
12687	LRG1	166.325866	-3.0663111	0.20025359	-15.31214	6.34E-53
12691	LRIG2	778.9522	0.55926555	0.09780551	5.71813926	1.08E-08
12692	LRIG3	457.781245	0.52616311	0.10683818	4.92486015	8.44E-07
12697	LRP1	3077.79694	-1.201599	0.09908947	-12.126406	7.65E-34
12699	LRP10	9212.53569	-0.4731376	0.08177411	-5.7859095	7.21E-09
12700	LRP11	2400.58131	-0.6059818	0.06959451	-8.7073216	3.11E-18
12704	LRP2BP	138.560173	-0.7941219	0.18057137	-4.3978283	1.09E-05
12705	LRP3	1210.6479	-0.2844359	0.09642466	-2.9498249	0.00317954
12706	LRP4	175.094718	-1.4756273	0.17111311	-8.6236955	6.48E-18
12708	LRP5	9114.52752	-0.7748864	0.07329419	-10.572276	4.01E-26
12713	LRPPRC	11946.8339	0.97675721	0.05445035	17.9384927	5.90E-72
12714	LRR1	2559.87231	0.53804894	0.06886423	7.81318464	5.58E-15
12720	LRRC15	188.771324	-2.0666884	0.21451742	-9.6341286	5.74E-22
12728	LRRC20	1289.46689	-1.4954791	0.07505234	-19.925817	2.43E-88

12730	LRRC24	215.312442	-0.6685745	0.18748097	-3.5660928	0.00036234
12733	LRRC27	265.478973	-1.322104	0.13337721	-9.912518	3.67E-23
12736	LRRC3	137.422535	-0.5014412	0.19416974	-2.5824888	0.00980906
12742	LRRC36	6.00369833	-6.1285476	1.38240468	-4.4332514	9.28E-06
12747	LRRC37A4P	177.013088	-0.941454	0.17946535	-5.2458816	1.56E-07
12749	LRRC37A6P	39.5687108	-1.7478934	0.39690019	-4.4038614	1.06E-05
12758	LRRC40	1316.30414	0.61641196	0.08687369	7.0954962	1.29E-12
12759	LRRC41	2793.82719	0.47665078	0.06669224	7.14701983	8.87E-13
12760	LRRC42	5442.07768	1.60453817	0.07367805	21.7776974	3.78E-105
12762	LRRC45	969.662489	-1.4010651	0.1269646	-11.035085	2.59E-28
12766	LRRC49	203.769813	0.69281057	0.14919396	4.64369043	3.42E-06
12781	LRRC70	21.5655032	1.20021723	0.45375418	2.6450825	0.0081671
12788	LRRC75A-AS	15503.326	0.67243848	0.06858803	9.80402147	1.08E-22
12789	LRRC75B	131.051679	-0.7093049	0.20464801	-3.4659749	0.00052831
12792	LRRC8C	738.030093	0.43972456	0.10011566	4.39216552	1.12E-05
12796	LRRCC1	373.429839	-0.5379062	0.11598636	-4.6376677	3.52E-06
12798	LRRFIP1	6966.8309	0.60610197	0.07386101	8.20598016	2.29E-16
12799	LRRFIP2	3307.60995	0.71073362	0.06784387	10.4760181	1.11E-25
12803	LRRK1	1223.705	-0.3672073	0.10815371	-3.3952355	0.0006857
12805	LRRN1	29.6412021	-4.5256747	0.61617293	-7.3448126	2.06E-13
12818	LRWD1	1745.89824	0.42972769	0.09847602	4.36377998	1.28E-05
12821	LSG1	2371.4915	0.35298568	0.06546972	5.39158717	6.98E-08
12822	LSM1	1453.34016	0.34375557	0.08340979	4.12128555	3.77E-05
12823	LSM10	964.394803	0.38055355	0.08611067	4.41935432	9.90E-06
12827	LSM14B	3535.88366	0.13547469	0.06875675	1.9703475	0.04879856
12828	LSM2	1512.50349	-0.2800771	0.07913109	-3.5394063	0.00040103
12829	LSM3	1986.3564	-0.3410366	0.08509079	-4.007914	6.13E-05
12842	LTA	26.1266909	0.91366669	0.39960254	2.28643866	0.02222861
12848	LTBP2	1535.68588	-1.4324319	0.09122742	-15.701768	1.47E-55
12849	LTBP3	4711.79942	-0.8625157	0.10142073	-8.5043335	1.83E-17
12851	LTBR	4326.45927	-0.2794339	0.06154662	-4.5401989	5.62E-06
12861	LUM	95.1463773	-1.756518	0.22731999	-7.7270721	1.10E-14
12864	LURAP1L	308.09615	0.69042813	0.12550778	5.50107836	3.77E-08
12865	LURAP1L-AS	70.058657	2.83506323	0.30633882	9.25466536	2.15E-20
12866	LUZP1	3563.35176	0.87633549	0.09277712	9.44559917	3.53E-21
12870	LVCAT1	20.369261	-1.0883579	0.44932194	-2.4222229	0.01542589
12874	LXN	319.514624	-1.0506298	0.12921002	-8.1311793	4.25E-16
12899	LYPD3	27.9011867	-1.7347323	0.43014203	-4.032929	5.51E-05
12901	LYPD5	4.35115977	2.33229838	1.1164372	2.0890547	0.0367028
12910	LYRM1	1868.47983	0.6731716	0.06947137	9.68991396	3.33E-22
12912	LYRM4	1581.48875	0.28797654	0.07926517	3.63307794	0.00028006
12913	LYRM5	284.623399	0.33398971	0.13567514	2.46168686	0.01382853
12914	LYRM7	733.815505	0.57181993	0.10214943	5.59787709	2.17E-08

12915	LYRM9	52.358055	-2.034477	0.3149504	-6.4596743	1.05E-10
12918	LYSMD3	2101.07042	0.65742831	0.0887321	7.40913734	1.27E-13
12927	LZIC	1098.92487	0.9868671	0.09396669	10.5023076	8.43E-26
12928	LZTFL1	1076.70981	0.29568885	0.08475741	3.48864899	0.00048547
12932	LZTS2	4005.25977	-0.6810122	0.07624493	-8.9319022	4.19E-19
12949	MAD2L1BP	1607.65165	0.39000011	0.09114719	4.27879454	1.88E-05
12952	MADD	3525.83403	-0.2676916	0.07295332	-3.669354	0.00024316
12959	MAFB	38.5284268	-1.3192616	0.32455334	-4.0648529	4.81E-05
12960	MAFF	5131.20137	1.67501817	0.0693914	24.1387008	9.81E-129
12961	MAFG	6185.88325	1.00819353	0.06766485	14.8998109	3.31E-50
12964	MAFK	3785.50356	0.91493471	0.07565321	12.0937993	1.14E-33
12971	MAGEA12	637.831238	11.71146	1.18155164	9.91193241	3.69E-23
12993	MAGEC2	28.5320776	7.21835261	1.22380139	5.89830396	3.67E-09
12995	MAGED1	3927.61214	-0.6700489	0.08167414	-8.2039295	2.33E-16
12996	MAGED2	7785.98382	-0.3216091	0.07159639	-4.4919741	7.06E-06
12999	MAGEE1	352.536312	-0.8105347	0.12437377	-6.5169262	7.18E-11
13002	MAGEH1	75.2523006	-1.5103402	0.23964353	-6.302445	2.93E-10
13011	MAGOH	1308.51212	0.27586156	0.08736225	3.1576745	0.00159033
13015	MAK	30.9591267	1.7044608	0.41316062	4.12541931	3.70E-05
13016	MAK16	996.655495	0.54585434	0.08487627	6.43117757	1.27E-10
13018	MAL2	2557.65022	4.65921555	0.08939686	52.1183366	0
13024	MAMDC2	528.385042	-1.3055636	0.10589076	-12.329344	6.30E-35
13027	MAML1	2229.48827	0.21768432	0.0792655	2.74626812	0.00602775
13032	MAN1A1	135.232982	-2.7125854	0.24675622	-10.992976	4.13E-28
13037	MAN2A1	2875.95679	0.51216577	0.08594811	5.95901175	2.54E-09
13038	MAN2A2	2320.35993	-0.3299111	0.08236987	-4.0052403	6.20E-05
13040	MAN2B2	1983.30853	-0.7568424	0.07406226	-10.219003	1.63E-24
13041	MAN2C1	1074.34342	-0.7587926	0.07989005	-9.4979616	2.14E-21
13042	MANBA	656.588201	-0.3127501	0.09634328	-3.246206	0.00116954
13044	MANEA	385.869854	-0.8065016	0.15442256	-5.2226928	1.76E-07
13048	MANSC1	96.5419223	-1.3023521	0.21145392	-6.159035	7.32E-10
13054	MAP1B	13463.7025	0.41766995	0.0734072	5.6897681	1.27E-08
13056	MAP1LC3B	3900.938	0.285509	0.06258446	4.56197898	5.07E-06
13058	MAP1LC3C	15.2890052	-2.6485649	0.64972309	-4.0764519	4.57E-05
13059	MAP1S	2229.37895	-0.1929387	0.08285654	-2.3285874	0.01988094
13063	MAP2K3	5718.50762	0.64827534	0.06444075	10.0600217	8.30E-24
13067	MAP2K6	19.0357948	-1.6347537	0.47459298	-3.4445384	0.00057204
13069	MAP3K1	2227.31304	-0.3845833	0.10023486	-3.8368214	0.00012464
13070	MAP3K10	1477.19043	0.78941622	0.09961113	7.92498018	2.28E-15
13071	MAP3K11	4300.00796	-0.7064111	0.06972689	-10.131114	4.02E-24
13072	MAP3K12	328.640868	-1.0029102	0.11867295	-8.4510431	2.89E-17
13074	MAP3K14	1669.17866	0.5573133	0.07036566	7.92024554	2.37E-15
13076	MAP3K15	372.485108	-0.5500125	0.11807234	-4.6582674	3.19E-06

13078	MAP3K2	3091.42919	0.55422205	0.0658	8.42282785	3.68E-17
13081	MAP3K5	644.280072	-0.7972057	0.09594915	-8.3086261	9.68E-17
13082	MAP3K6	607.472762	-0.9004259	0.09473783	-9.5043964	2.01E-21
13085	MAP3K8	623.789073	-0.8404741	0.09805722	-8.5712613	1.02E-17
13089	MAP4K2	710.43249	-1.3872637	0.09097229	-15.249299	1.66E-52
13092	MAP4K5	4745.86826	0.77753129	0.07873876	9.87482321	5.35E-23
13096	MAP7D1	11049.2893	0.60497571	0.06043958	10.0095946	1.38E-23
13100	MAPK1	4735.6271	-0.2199106	0.07661452	-2.8703509	0.00410016
13102	MAPK11	273.237373	-1.0522491	0.14899149	-7.0624778	1.64E-12
13107	MAPK1IP1L	6331.31317	0.35029307	0.06310618	5.55085203	2.84E-08
13108	MAPK3	1364.44258	-0.6304085	0.08494237	-7.4216034	1.16E-13
13110	MAPK6	2359.70014	0.3796451	0.09425239	4.02796259	5.63E-05
13111	MAPK7	1064.95446	-0.2647377	0.08183918	-3.2348527	0.00121706
13112	MAPK8	1763.42219	0.18111721	0.07111477	2.54682966	0.01087065
13115	MAPK8IP3	2559.16218	-0.4319969	0.06691495	-6.4559104	1.08E-10
13117	MAPKAP1	4211.17702	-0.3526757	0.06306261	-5.5924694	2.24E-08
13118	MAPKAPK2	8350.02176	0.28967075	0.05342807	5.42169631	5.90E-08
13121	MAPKAPK5-A	372.983937	-0.333105	0.13475341	-2.4719596	0.01343747
13124	MAPRE2	1353.01482	0.34488712	0.1001385	3.44410126	0.00057296
13129	1-Mar	196.285958	-0.5036102	0.17938957	-2.8073549	0.00499502
13134	2-Mar	518.103508	-1.1020938	0.10146769	-10.861525	1.76E-27
13142	MARCKS	14794.6566	-0.1834525	0.05919512	-3.0991146	0.001941
13145	MARK1	86.3869583	-3.3643492	0.2961488	-11.360334	6.59E-30
13146	MARK2	3182.85624	0.37914582	0.06515738	5.81892389	5.92E-09
13148	MARK3	3710.76036	0.70561207	0.06179182	11.4191833	3.35E-30
13150	MARS	7207.41323	0.9694069	0.05567616	17.4115268	6.75E-68
13151	MARS2	832.065066	0.59095731	0.08577216	6.88984982	5.59E-12
13152	MARVELD1	919.675818	-2.271484	0.09227083	-24.617574	8.19E-134
13158	MASP2	13.3802967	3.50154132	0.86829383	4.03266866	5.51E-05
13160	MAST2	3056.63939	0.33414419	0.08087084	4.13182522	3.60E-05
13166	MAT2B	3614.35606	-0.1734638	0.06088321	-2.8491235	0.00438399
13168	MATN1	17.3596039	2.24031312	0.5677903	3.94566997	7.96E-05
13170	MATN2	7083.37561	-0.3172995	0.06405853	-4.953274	7.30E-07
13171	MATN3	205.098828	1.08857733	0.16943511	6.42474483	1.32E-10
13173	MATR3	15817.0426	0.71163657	0.05710333	12.4622613	1.20E-35
13175	MAVS	5972.58839	-0.343877	0.06516133	-5.2773176	1.31E-07
13176	MAX	3438.26881	0.50628479	0.07232671	6.99996962	2.56E-12
13178	MB	183.157999	-0.9699904	0.16263338	-5.964276	2.46E-09
13179	MB21D1	2480.92832	1.266628	0.07317907	17.308609	4.05E-67
13180	MB21D2	436.695411	0.43341634	0.11761138	3.68515646	0.00022856
13182	MBD2	2650.30813	-0.4230122	0.07271053	-5.817757	5.96E-09
13190	MBD5	291.234122	-0.7019692	0.15422939	-4.5514622	5.33E-06
13195	MBLAC1	96.3881481	-0.9499511	0.24426242	-3.8890597	0.00010063

13201	MBOAT1	137.737139	-1.0011184	0.17557035	-5.7020924	1.18E-08
13202	MBOAT2	1165.48009	-0.4561721	0.0785771	-5.8054073	6.42E-09
13204	MBOAT7	14781.4954	-0.2721475	0.06519978	-4.1740558	2.99E-05
13207	MBTPS1	5164.04749	-0.4189062	0.0551586	-7.5945758	3.09E-14
13216	MCC	841.268357	1.66559513	0.12050309	13.8220121	1.88E-43
13218	MCCC2	2369.60876	-0.4528016	0.07296604	-6.2056489	5.45E-10
13231	MCL1	11751.6495	0.62721141	0.05784495	10.8429759	2.15E-27
13235	MCM3AP	3487.79273	0.42829813	0.06557997	6.5309287	6.54E-11
13236	MCM3AP-AS	235.972452	0.9859373	0.16846802	5.85237074	4.85E-09
13238	MCM5	3049.59168	-0.8332303	0.06621246	-12.584193	2.58E-36
13239	MCM6	5131.29155	-0.2890717	0.06492108	-4.4526633	8.48E-06
13248	MCOLN3	558.194143	-1.3713287	0.10885752	-12.597465	2.18E-36
13249	MCPH1	500.985882	0.50111669	0.1101847	4.54796974	5.42E-06
13251	MCRS1	1623.44177	-0.9772173	0.10086647	-9.6882278	3.38E-22
13255	MCTS2P	186.949566	0.60484273	0.1664819	3.63308397	0.00028005
13256	MCU	1057.2147	-0.9133293	0.0887508	-10.290942	7.74E-25
13260	MDFIC	1953.06519	-0.2919722	0.08614573	-3.3892822	0.00070076
13263	MDH1	6026.1048	-0.3365074	0.10169371	-3.309029	0.0009362
13268	MDM2	2187.22933	0.5682962	0.06937943	8.19113371	2.59E-16
13270	MDN1	2315.89832	0.53862436	0.09160855	5.87962949	4.11E-09
13274	ME2	3028.70011	0.93524896	0.06054282	15.4477267	7.82E-54
13276	MEA1	5990.7993	0.63331657	0.08391279	7.54731914	4.44E-14
13277	MEAF6	1927.33346	0.71316724	0.07417188	9.61506235	6.91E-22
13279	MECOM	3788.34142	1.27967931	0.08785785	14.565339	4.67E-48
13282	MED1	3435.72177	0.25971547	0.1161498	2.23603884	0.02534923
13283	MED10	4259.32759	1.53402574	0.09353871	16.3999034	1.92E-60
13287	MED13	3881.89173	0.47997061	0.09413104	5.09896196	3.42E-07
13288	MED13L	3540.85917	0.67209567	0.08978616	7.48551537	7.13E-14
13289	MED14	7816.01048	-0.2034884	0.10070476	-2.020643	0.04331673
13290	MED14OS	52.4581574	-0.850533	0.30756134	-2.7654094	0.00568514
13291	MED15	3449.89209	0.15877421	0.07015384	2.26322923	0.02362156
13293	MED16	2711.95507	-0.2070283	0.10446714	-1.981755	0.04750666
13294	MED17	2627.42219	0.33777901	0.07984312	4.23053389	2.33E-05
13296	MED19	606.143859	0.2104834	0.10678141	1.97116139	0.04870542
13299	MED22	3236.74559	0.54394764	0.07509421	7.24353631	4.37E-13
13301	MED24	2562.76742	-0.5892385	0.06390545	-9.2204737	2.96E-20
13303	MED26	568.927973	0.29240741	0.11034316	2.64998223	0.0080496
13313	MED8	1596.07918	0.66769178	0.07012353	9.5216504	1.70E-21
13330	MEGF8	1766.06282	-0.7197999	0.08984521	-8.011556	1.13E-15
13331	MEGF9	2410.57596	-0.8727646	0.09789295	-8.9154992	4.86E-19
13332	MEI1	40.2215501	0.94556297	0.32091109	2.94649517	0.00321397
13343	MELK	4631.52015	-0.2343807	0.06558225	-3.5738438	0.00035178
13345	MEN1	2958.37807	-0.2581977	0.0616089	-4.1909161	2.78E-05

13353	MERTK	825.781294	0.62702696	0.0890939	7.03782127	1.95E-12
13358	MEST	1256.46167	-0.3177621	0.08357964	-3.8019074	0.00014359
13366	METTL1	418.630846	0.30656119	0.12164851	2.52005704	0.01173358
13367	METTL10	959.880887	-0.4432899	0.10086441	-4.3949093	1.11E-05
13370	METTL13	2746.96457	0.48078194	0.06173089	7.78835245	6.79E-15
13374	METTL17	1970.83036	-0.4279034	0.06874939	-6.2241051	4.84E-10
13381	METTL22	300.036232	0.34370628	0.1706878	2.01365462	0.04404581
13384	METTL25	116.550309	-0.5585123	0.20036695	-2.7874472	0.00531251
13385	METTL2A	762.047023	0.3784441	0.09073559	4.17084537	3.03E-05
13386	METTL2B	948.018811	0.41146608	0.08955088	4.59477439	4.33E-06
13390	METTL6	737.3375	0.33635513	0.0970222	3.46678518	0.00052672
13391	METTL7A	567.123977	-1.6885631	0.10134536	-16.661473	2.50E-62
13393	METTL8	1336.65093	0.81841242	0.08962433	9.13158801	6.75E-20
13394	METTL9	2405.47846	-0.5744084	0.06340055	-9.0599914	1.30E-19
13395	MEX3A	363.148661	2.5604831	0.13322337	19.2194741	2.54E-82
13397	MEX3C	1748.15635	0.94586671	0.09548955	9.9054472	3.94E-23
13401	MFAP3	2231.55002	0.55937094	0.08881649	6.2980531	3.01E-10
13402	MFAP3L	98.2688591	0.53415667	0.20954251	2.54915654	0.01079838
13405	MFF	1743.20853	-0.4403949	0.08442159	-5.2166138	1.82E-07
13406	MFGE8	3857.05708	-0.3539414	0.05898911	-6.0001141	1.97E-09
13409	MFI2-AS1	57.1825556	-1.0038881	0.28311764	-3.5458337	0.00039137
13411	MFN2	6322.56889	0.2771029	0.06279109	4.41309287	1.02E-05
13418	MFSD2A	379.087917	1.89471445	0.13565755	13.9668927	2.48E-44
13420	MFSD3	1260.88568	-1.4713362	0.12070194	-12.189831	3.52E-34
13423	MFSD6	2840.42144	-0.3220995	0.07538338	-4.2728185	1.93E-05
13425	MFSD7	530.594076	-0.7346695	0.15977889	-4.5980388	4.26E-06
13428	MGA	2126.69848	0.54523065	0.09229806	5.90728189	3.48E-09
13437	MGAT4B	12994.7407	0.25628811	0.07743431	3.30974895	0.0009338
13442	MGAT5B	491.372707	-0.876231	0.11976614	-7.3161834	2.55E-13
13443	MGC12916	250.348892	-2.6175183	0.16216061	-16.141518	1.30E-58
13459	MGC72080	247.270565	0.41793972	0.14729294	2.83747295	0.00454722
13462	MGME1	2044.49794	0.28237497	0.07126068	3.96256371	7.41E-05
13467	MGST2	388.003591	-1.2866548	0.12365633	-10.405086	2.35E-25
13468	MGST3	2649.60332	-0.5188408	0.09579405	-5.4162109	6.09E-08
13475	MIATNB	33.2147197	-0.8430109	0.37745685	-2.2333967	0.02552279
13478	MICA	2018.99416	0.1530268	0.07277461	2.10274996	0.03548763
13483	MICALL1	2972.29186	0.44299398	0.06251009	7.08675942	1.37E-12
13484	MICALL2	653.845729	-0.6246326	0.09638845	-6.4803677	9.15E-11
13486	MICU1	2594.87525	-0.9013621	0.06366513	-14.157861	1.67E-45
13490	MID1IP1	3246.23824	0.78218476	0.06870392	11.3848639	4.97E-30
13493	MIDN	1535.03477	0.78172999	0.07085307	11.0331136	2.65E-28
13496	MIEN1	1756.73996	-0.4452484	0.12081318	-3.6854289	0.00022832
13497	MIER1	2895.55136	0.88452964	0.08338244	10.6081043	2.73E-26

13498	MIER2	2265.79464	0.63151833	0.06699835	9.42587937	4.27E-21
13500	MIF	11351.484	-0.1635961	0.07800303	-2.0973047	0.03596661
13503	MIIP	872.524966	0.60986769	0.10422163	5.85164214	4.87E-09
13506	MINA	2689.78826	0.79944409	0.06803191	11.7510169	6.98E-32
13507	MINK1	4550.49508	-0.5073333	0.07363648	-6.8897006	5.59E-12
13511	MINPP1	1801.5748	-0.8062763	0.07386601	-10.915391	9.73E-28
13513	MIOX	10.3101432	4.20760999	0.99147505	4.24378808	2.20E-05
13516	MIPEPP3	21.7781365	0.91128779	0.45724913	1.99297872	0.04626378
13553	MIR1204	26.3015563	2.17457984	0.43190459	5.03486162	4.78E-07
13734	MIR17HG	252.899543	0.74426501	0.14900749	4.99481606	5.89E-07
13807	MIR210HG	697.666551	-0.8845031	0.115862	-7.6341086	2.27E-14
13829	MIR222	3.08605365	2.96996716	1.39083737	2.13538061	0.03272993
13835	MIR22HG	1852.53079	1.51892286	0.07412989	20.4900203	2.64E-93
13875	MIR30A	108.208864	-6.6231137	0.58495844	-11.322366	1.02E-29
14326	MIR4449	1.76520561	4.18730287	1.84583891	2.26850938	0.02329818
14525	MIR4712	16.0648264	1.41931733	0.56169184	2.52686124	0.0115087
14661	MIR5047	175.256536	-0.9230646	0.16488781	-5.5981373	2.17E-08
15269	MIR7111	27.1179434	1.18595827	0.43617479	2.71899773	0.00654801
15434	MIS12	2027.86754	0.95420554	0.07133623	13.3761702	8.33E-41
15437	MISP	314.195649	0.69513589	0.12312382	5.64582766	1.64E-08
15439	MITF	909.915648	0.69404742	0.09119585	7.61051553	2.73E-14
15441	MKI67	10362.8592	-1.1774233	0.12288643	-9.581394	9.57E-22
15444	MKL2	793.128106	-0.8382218	0.11799124	-7.104102	1.21E-12
15445	MKLN1	8623.29973	1.2026052	0.06844263	17.5709958	4.11E-69
15446	MKLN1-AS	503.872159	0.82804187	0.11696816	7.07920772	1.45E-12
15447	MKNK1	1749.44178	0.65658017	0.07531657	8.71760571	2.84E-18
15449	MKNK2	4686.89242	0.53688913	0.07253023	7.40228037	1.34E-13
15453	MKRN3	32.4999309	7.40479481	1.21913971	6.07378691	1.25E-09
15456	MKS1	275.418577	-1.0799116	0.13295766	-8.1222215	4.58E-16
15457	MKX	321.599613	2.68347174	0.14782562	18.1529538	1.22E-73
15462	MLF1	755.891765	0.42312066	0.09925316	4.2630448	2.02E-05
15463	MLF2	4700.21618	-0.4082941	0.05938546	-6.8753215	6.19E-12
15465	MLH3	1235.93001	0.77080242	0.0790297	9.75332646	1.79E-22
15469	MLKL	545.100596	-1.397999	0.10212623	-13.688932	1.18E-42
15470	MLLT1	4778.3418	0.42008007	0.06231662	6.74105981	1.57E-11
15474	MLLT3	569.533726	-1.0493915	0.10836569	-9.6837982	3.53E-22
15482	MLX	2536.05878	-0.562882	0.08829383	-6.3751	1.83E-10
15487	MMAB	538.849515	-0.5775645	0.09701259	-5.9534999	2.62E-09
15488	MMACHC	418.318161	0.53788564	0.11251949	4.78037739	1.75E-06
15489	MMADHC	6344.15274	0.37767085	0.06671877	5.66063851	1.51E-08
15495	MMP1	56.9252393	0.76334162	0.26806335	2.84761646	0.0044048
15497	MMP11	174.657436	-2.1991442	0.18233699	-12.060878	1.70E-33
15500	MMP14	13783.3635	-0.65012	0.05541361	-11.732135	8.72E-32

15501	MMP15	864.590356	-0.6709969	0.09154448	-7.3297364	2.31E-13
15510	MMP24	147.646829	-1.6983087	0.17977261	-9.4469825	3.49E-21
15511	MMP24-AS1	5958.41523	-0.4443141	0.09857722	-4.5072699	6.57E-06
15516	MMP28	12.4171123	-3.8763997	0.83136072	-4.6627169	3.12E-06
15518	MMP7	9.291288	-1.6181797	0.68543066	-2.3608218	0.01823449
15522	MMRN2	32.2922613	-1.3636501	0.38436974	-3.5477562	0.00038853
15526	MNAT1	1916.40153	0.21749458	0.08807852	2.46932594	0.01353679
15529	MNS1	147.600108	-0.9123069	0.17049599	-5.3508996	8.75E-08
15533	MOAP1	1886.86052	0.35314489	0.06955586	5.07714086	3.83E-07
15537	MOB3A	3607.3983	-0.2350803	0.05989914	-3.9246028	8.69E-05
15539	MOB3C	611.415844	0.59362683	0.09467271	6.27030593	3.60E-10
15542	MOCOS	1016.15362	0.52852221	0.10339844	5.11151053	3.20E-07
15550	MOGS	2463.1465	-0.6097157	0.07420568	-8.2165643	2.09E-16
15552	MON1A	480.365904	0.26913831	0.1097216	2.45292001	0.01417018
15556	MORC2	2447.53117	0.20182416	0.06546904	3.08274223	0.00205103
15558	MORC3	2527.54095	1.02071284	0.08846574	11.5379446	8.49E-31
15559	MORC4	6270.56267	-0.5542587	0.05965706	-9.2907469	1.53E-20
15569	MOSPD1	2573.6169	0.32812269	0.07839684	4.18540712	2.85E-05
15571	MOSPD3	713.849268	-0.9875271	0.09745712	-10.132939	3.95E-24
15572	MOV10	2383.3789	-0.2595458	0.07188688	-3.610475	0.00030564
15574	MOXD1	269.547611	-1.8397158	0.14255222	-12.905557	4.19E-38
15578	MPC2	2994.3801	-0.1623223	0.08217698	-1.9752767	0.04823676
15583	MPHOSPH10	1143.97307	0.40260453	0.07767822	5.18297856	2.18E-07
15584	MPHOSPH6	1216.86845	0.50024403	0.07852057	6.37086588	1.88E-10
15589	MPLKIP	1900.72006	0.6776384	0.09512238	7.12385858	1.05E-12
15590	MPND	274.264228	-0.6439875	0.14281902	-4.5091163	6.51E-06
15594	MPP3	551.632744	0.55920432	0.10439962	5.35638253	8.49E-08
15597	MPP6	1366.76788	0.78669371	0.07685636	10.2358963	1.37E-24
15599	MPPE1	87.0763345	-1.5425347	0.25868976	-5.9628751	2.48E-09
15609	MPZL2	296.601228	-3.9125124	0.1804351	-21.683765	2.92E-104
15610	MPZL3	253.688099	1.91269652	0.14841175	12.8877704	5.27E-38
15611	MR1	836.951554	0.24923639	0.09808306	2.54107479	0.01105123
15614	MRAS	885.509024	0.50799772	0.08674896	5.85595173	4.74E-09
15616	MRC2	2695.2216	-1.0909573	0.06866985	-15.886991	7.80E-57
15617	MRE11A	1705.79781	-0.5448046	0.08603127	-6.3326352	2.41E-10
15632	MRI1	869.469628	-0.4034185	0.1022413	-3.9457492	7.96E-05
15646	MRPL10	1526.38572	0.14854069	0.07225342	2.05582923	0.03979898
15658	MRPL21	1784.15165	0.54264799	0.11611061	4.6735434	2.96E-06
15659	MRPL22	1104.91104	-0.280098	0.09707948	-2.8852444	0.0039111
15660	MRPL23	168.366334	-0.4634722	0.16061861	-2.885545	0.00390737
15662	MRPL24	2286.50407	0.45972821	0.08257751	5.56723249	2.59E-08
15667	MRPL32	1787.58951	0.22083418	0.08500093	2.59802081	0.00937628
15671	MRPL36	1540.82679	0.46398559	0.08580651	5.40734719	6.40E-08

15672	MRPL37	3624.68781	0.25005447	0.0741449	3.37251067	0.00074486
15673	MRPL38	1851.86701	-0.4663079	0.11154755	-4.1803508	2.91E-05
15675	MRPL4	2732.23722	-0.4587978	0.07259344	-6.3200999	2.61E-10
15680	MRPL43	1706.84739	-0.9280982	0.07601634	-12.209194	2.78E-34
15682	MRPL45	2502.36982	0.43530139	0.0889085	4.89606053	9.78E-07
15690	MRPL52	2032.64956	0.74851327	0.11778826	6.35473571	2.09E-10
15693	MRPL55	729.69214	-0.3295161	0.1442966	-2.2836026	0.0223949
15696	MRPS10	4549.77818	0.51398913	0.05774056	8.90170028	5.50E-19
15708	MRPS22	1490.88325	-0.4398069	0.08510017	-5.1681076	2.36E-07
15711	MRPS25	2283.82836	0.85785111	0.0702799	12.2062077	2.88E-34
15713	MRPS27	3077.59912	0.20612523	0.06698656	3.07711344	0.00209016
15715	MRPS30	1638.41469	0.36513557	0.07762847	4.70362934	2.56E-06
15716	MRPS31	686.123194	0.25916553	0.09365107	2.7673525	0.00565136
15720	MRPS35	3407.07834	0.49406906	0.06955436	7.10335176	1.22E-12
15726	MRRF	1066.71977	0.18549743	0.08191908	2.26439825	0.02354963
15729	MRTO4	3968.37752	1.27456298	0.07419623	17.1782721	3.86E-66
15749	MSANTD3	3664.70361	0.24515024	0.06622329	3.70187361	0.00021401
15751	MSANTD4	1925.56532	0.55552014	0.06847471	8.11277871	4.95E-16
15755	MSH2	3358.91518	0.47730374	0.07371205	6.47524751	9.47E-11
15764	MSL2	1241.67544	0.78168049	0.07914825	9.87615661	5.28E-23
15774	MSRB1	500.631296	-0.4048921	0.111837	-3.6203767	0.00029417
15775	MSRB2	1064.76559	-0.2952438	0.10572574	-2.7925446	0.00522953
15777	MSS51	63.7786634	-0.9939042	0.32791808	-3.0309527	0.00243783
15778	MST1	58.228201	-2.0473467	0.29580206	-6.9213404	4.47E-12
15780	MST1P2	18.806779	-2.2408141	0.55164921	-4.0620272	4.86E-05
15781	MST1R	968.803977	-1.1676935	0.08530441	-13.688548	1.19E-42
15786	MSX2	132.571106	2.29545036	0.23233022	9.88011949	5.08E-23
15788	MT1A	94.3960272	-2.0924	0.30396014	-6.8837973	5.83E-12
15792	MT1F	90.4302164	-2.5777257	0.25131435	-10.256978	1.10E-24
15799	MT1M	4.05253584	-4.5406517	1.5053325	-3.0163779	0.00255814
15805	MTA2	6531.65215	0.46609279	0.05889383	7.9141186	2.49E-15
15806	MTA3	1318.76015	-0.1881617	0.08045492	-2.3387218	0.01934984
15809	MTCH1	26628.4132	0.32545994	0.07947902	4.09491617	4.22E-05
15810	MTCH2	6935.14033	-0.2738299	0.08773308	-3.1211707	0.00180134
15816	MTERF3	1363.27856	0.69933954	0.0799467	8.74757244	2.18E-18
15818	MTF1	906.807868	0.92852563	0.11295359	8.22041727	2.03E-16
15820	MTFMT	422.756275	-0.8241243	0.11897875	-6.9266513	4.31E-12
15825	MTG1	894.061018	-1.5760174	0.11620313	-13.562607	6.67E-42
15826	MTG2	2111.58255	0.39389899	0.08114246	4.85441291	1.21E-06
15829	MTHFD2	2592.2157	1.30608742	0.06521745	20.0266557	3.23E-89
15840	MTMR10	798.618646	-0.3796382	0.10293969	-3.6879672	0.00022605
15841	MTMR11	413.86356	-1.653818	0.12086826	-13.682814	1.29E-42
15844	MTMR2	3835.85875	0.19860621	0.07056134	2.81466033	0.00488288

15846	MTMR4	1896.48565	-0.6673213	0.07955764	-8.3878981	4.95E-17
15849	MTMR8	31.0920789	-3.8505912	0.50991875	-7.5513819	4.31E-14
15850	MTMR9	724.391275	0.72448393	0.09011506	8.03954326	9.02E-16
15851	MTMR9LP	122.492473	-0.5425623	0.22492558	-2.412186	0.01585719
15854	MTO1	1983.03887	0.39230292	0.07474509	5.24854451	1.53E-07
15855	MTOR	2563.5094	0.43288807	0.10298454	4.20342762	2.63E-05
15857	MTPAP	1644.76893	0.42645169	0.07516921	5.67322282	1.40E-08
15861	MTRF1L	395.33258	0.41803296	0.132918	3.14504397	0.00166062
15872	MTRR	2888.73018	0.74999384	0.08137854	9.21611342	3.08E-20
15873	MTSS1	35.7962802	1.1100741	0.38187352	2.90691566	0.00365012
15874	MTSS1L	4325.91345	0.17235326	0.06364851	2.7078915	0.00677122
15881	MTX1	567.753984	-0.431871	0.11682568	-3.6967133	0.00021841
15882	MTX2	2894.55737	-0.3352672	0.0841645	-3.9834757	6.79E-05
15884	MUC1	727.962674	-1.3995059	0.10279819	-13.61411	3.30E-42
15892	MUC20	25.9909336	1.39682056	0.43541395	3.20802893	0.00133648
15907	MUSK	27.4572325	1.06937105	0.39272988	2.72291749	0.00647082
15912	MVB12B	594.906605	-0.3254988	0.09904512	-3.2863686	0.00101488
15913	MVD	858.872647	-0.856524	0.13834416	-6.1912554	5.97E-10
15915	MVP	6155.44196	-0.6175755	0.07371297	-8.3781107	5.38E-17
15918	MXD1	666.801467	1.06052579	0.09975789	10.6309969	2.14E-26
15919	MXD3	664.968418	-1.2596663	0.13877067	-9.0773238	1.11E-19
15920	MXD4	1668.4693	-1.331367	0.08191013	-16.253998	2.09E-59
15921	MXI1	2098.65554	-0.5323591	0.10855405	-4.9040924	9.39E-07
15923	MXRA7	3556.30025	-0.1854295	0.06242826	-2.9702809	0.00297528
15924	MXRA8	1528.64807	-3.0656927	0.10126204	-30.274846	2.46E-201
15929	MYBBP1A	4343.84777	0.59187435	0.0735544	8.04675608	8.50E-16
15930	MYBL1	3509.44031	0.57534616	0.07586316	7.58399923	3.35E-14
15937	MYC	7405.7159	0.31924968	0.06408936	4.98132127	6.32E-07
15939	MYCBP2	2284.12816	0.48962828	0.09399807	5.20891813	1.90E-07
15949	MYEF2	1686.42733	-0.3698633	0.07190971	-5.1434408	2.70E-07
15950	MYEOV	6512.29093	0.18945308	0.05701192	3.32304312	0.00089041
15951	MYEOV2	839.64524	-0.3681527	0.15122086	-2.4345364	0.01491088
15959	MYH15	969.03521	-0.6776819	0.08399506	-8.0681161	7.14E-16
15978	MYL6	21085.6651	-0.5467042	0.11390425	-4.7996825	1.59E-06
15981	MYL9	7098.4988	-0.7114533	0.07604704	-9.3554365	8.33E-21
15983	MYLK	4753.27594	-0.2182665	0.09523639	-2.2918392	0.02191492
15987	MYLK3	9.12793292	-1.5086432	0.70863187	-2.128952	0.03325824
15990	MYNN	1241.99803	0.53977771	0.07942556	6.79601974	1.08E-11
15991	MYO10	5810.9494	-0.3851114	0.06745099	-5.7094995	1.13E-08
15993	MYO15B	67.4190246	-2.6253691	0.3028559	-8.6687072	4.37E-18
15996	MYO18A	6145.69433	-0.6473112	0.09435578	-6.8603243	6.87E-12
16002	MYO1D	45.5656859	1.79907609	0.32929407	5.46343293	4.67E-08
16029	MYPN	44.939348	1.0130525	0.32579595	3.10946926	0.00187424

16040	MZF1	606.128249	-0.7824073	0.10464548	-7.4767425	7.62E-14
16042	MZT1	2021.89008	0.66845986	0.07342015	9.10458297	8.66E-20
16043	MZT2A	929.540955	-1.0587253	0.16147732	-6.5564952	5.51E-11
16044	MZT2B	3467.64521	-0.5048193	0.11916804	-4.2361966	2.27E-05
16046	N4BP2	434.129509	0.72965229	0.13071584	5.58197277	2.38E-08
16048	N4BP2L2	1520.97986	0.35878051	0.08382508	4.28010935	1.87E-05
16053	NAA10	2827.03355	0.43905985	0.09085775	4.83238754	1.35E-06
16055	NAA15	8153.36967	1.29872639	0.05673302	22.8918966	5.60E-116
16058	NAA25	4190.9809	1.19467847	0.06845228	17.4527189	3.28E-68
16059	NAA30	1941.30233	0.75192492	0.06860706	10.9598767	5.96E-28
16060	NAA35	1723.09258	0.51948647	0.07304609	7.11176252	1.15E-12
16063	NAA50	8701.80135	1.04318833	0.05834099	17.8808821	1.66E-71
16064	NAA60	1316.34836	0.37114602	0.07884856	4.70707398	2.51E-06
16065	NAAA	767.592047	-0.8785791	0.0904906	-9.7090652	2.76E-22
16076	NACA	9931.93686	0.13151527	0.06645313	1.97906807	0.04780834
16078	NACAD	74.8949533	-2.0423631	0.28371503	-7.1986428	6.08E-13
16084	NADSYN1	1379.54653	-0.5076934	0.07945438	-6.389747	1.66E-10
16086	NAF1	856.873642	1.07482376	0.09688954	11.0932903	1.35E-28
16087	NAGA	891.929607	-0.8070821	0.08715727	-9.2600666	2.04E-20
16090	NAGPA	454.570328	-0.8916253	0.10950632	-8.1422265	3.88E-16
16092	NAGS	360.159453	0.81374275	0.12979019	6.2696782	3.62E-10
16095	NALCN	68.6037792	-1.8968146	0.28679663	-6.6137967	3.75E-11
16098	NAMPT	21041.0794	0.50711895	0.06020306	8.42347454	3.65E-17
16101	NANOS1	1366.40682	-1.2841603	0.10766712	-11.927136	8.55E-33
16104	NANP	909.185826	-0.5082943	0.08692331	-5.8476185	4.99E-09
16105	NANS	2581.90465	0.4760935	0.08119659	5.86346673	4.53E-09
16109	NAP1L4	5793.81084	-0.2445074	0.06094759	-4.0117646	6.03E-05
16115	NAPEPLD	307.021093	-0.6359619	0.13665478	-4.6537843	3.26E-06
16120	NARF	1756.57036	-0.4011082	0.08518131	-4.7088756	2.49E-06
16123	NARS	12474.1182	0.84626241	0.05545848	15.2593878	1.43E-52
16124	NARS2	1057.65578	0.68869035	0.08157229	8.44269962	3.10E-17
16126	NAT1	336.28556	-0.6172602	0.12135846	-5.0862558	3.65E-07
16127	NAT10	3460.89079	0.18400216	0.05781599	3.18254787	0.00145985
16128	NAT14	1356.47097	-1.0733503	0.14993729	-7.1586615	8.15E-13
16130	NAT2	2.93509011	3.85962872	1.54560454	2.49716446	0.01251909
16131	NAT6	271.051373	-1.1715026	0.13099206	-8.9433098	3.78E-19
16135	NAT9	2124.04136	0.56734566	0.08176782	6.93849585	3.96E-12
16136	NATD1	608.024287	0.28430979	0.11338871	2.50739074	0.01216262
16142	NAV3	988.740824	0.33534553	0.10237478	3.27566551	0.00105413
16147	NBEAL2	1701.91647	-0.6499593	0.07284613	-8.9223589	4.56E-19
16150	NBN	2470.00469	-0.4753465	0.07571379	-6.2782023	3.43E-10
16151	NBPF1	433.8025	1.22490334	0.14037957	8.72565248	2.65E-18
16156	NBPF14	213.40547	-0.4876888	0.15817348	-3.0832527	0.00204751

16175	NCAPD2	5297.92022	-0.8472149	0.06477555	-13.07924	4.33E-39
16176	NCAPD3	4922.19197	-0.172744	0.07423041	-2.3271325	0.01995822
16179	NCAPH	2783.91408	-0.6064525	0.06899709	-8.7895378	1.50E-18
16181	NCBP1	3253.4088	0.66221782	0.05927451	11.1720506	5.59E-29
16182	NCBP2	4789.02973	0.45597978	0.06417291	7.10548747	1.20E-12
16183	NCBP2-AS2	699.35502	-0.2381755	0.11265236	-2.1142523	0.03449373
16192	NCK1	1375.07055	0.45350853	0.07830058	5.79189255	6.96E-09
16198	NCKAP5L	1592.96456	0.67389871	0.08013682	8.40935124	4.12E-17
16199	NCKIPSD	1969.84095	-0.5653238	0.07241746	-7.8064569	5.88E-15
16200	NCL	38493.2774	0.71359819	0.06030026	11.8340811	2.60E-32
16201	NCLN	5744.13068	-0.3383931	0.08283407	-4.0851919	4.40E-05
16205	NCOA3	3487.58727	0.68341991	0.08630372	7.91877656	2.40E-15
16208	NCOA6	3706.27081	0.47233988	0.07521677	6.27971508	3.39E-10
16213	NCOR2	4682.8084	0.35595395	0.09834114	3.61958344	0.00029508
16221	NCSTN	5243.04651	-0.4022758	0.0696902	-5.772344	7.82E-09
16224	NDE1	970.483085	-0.5238544	0.082899	-6.3191878	2.63E-10
16225	NDEL1	2121.78915	0.4085163	0.07990528	5.11250665	3.18E-07
16233	NDP	18.572967	-4.4999622	0.77480853	-5.8078376	6.33E-09
16237	NDRG4	63.1432436	-2.3931721	0.29527313	-8.1049436	5.28E-16
16238	NDST1	8048.65553	-0.2386097	0.08450435	-2.8236385	0.00474819
16239	NDST2	621.575894	-1.5609471	0.1046535	-14.915383	2.62E-50
16245	NDUFA12	2461.02039	-0.3588967	0.07353618	-4.8805454	1.06E-06
16247	NDUFA2	1707.52476	-0.432558	0.10565342	-4.0941218	4.24E-05
16248	NDUFA3	517.244344	-0.8673008	0.12010041	-7.2214636	5.14E-13
16249	NDUFA4	2450.72035	-0.8175575	0.07831484	-10.43937	1.64E-25
16252	NDUFA6	1829.82345	0.23845944	0.07806249	3.05472508	0.00225267
16255	NDUFA8	2842.38201	-0.4681565	0.09975184	-4.6932122	2.69E-06
16256	NDUFA9	1927.92129	-0.5060489	0.10093735	-5.0134954	5.35E-07
16261	NDUFAF4	1072.84771	0.75085733	0.07691296	9.76242906	1.63E-22
16263	NDUFAF5	679.641578	0.67390356	0.10246569	6.57687038	4.80E-11
16267	NDUFB10	1469.88726	-0.4918438	0.08306512	-5.9211834	3.20E-09
16272	NDUFB4	2111.01209	-0.2359753	0.08850464	-2.6662475	0.00767032
16273	NDUFB5	2375.62854	-1.1367047	0.07387327	-15.387227	1.99E-53
16274	NDUFB6	2001.34632	-0.7545542	0.08791092	-8.5831677	9.23E-18
16275	NDUFB7	2201.07464	-0.5092151	0.13776432	-3.6962767	0.00021878
16278	NDUFC1	1135.75449	-0.5545706	0.15124672	-3.6666623	0.00024574
16283	NDUFS3	2342.62272	-0.4414241	0.11026957	-4.0031361	6.25E-05
16286	NDUFS6	2221.03809	-0.573284	0.09970079	-5.7500444	8.92E-09
16289	NDUFV1	6186.39252	-0.5221384	0.11784799	-4.4306095	9.40E-06
16292	NDUFV3	1305.84958	0.41461634	0.07784284	5.32632629	1.00E-07
16294	NEB	75.0262621	2.66373642	0.29993785	8.88096127	6.63E-19
16299	NECAB3	1146.66105	-0.4417692	0.08410058	-5.2528668	1.50E-07
16301	NECAP2	4391.86789	0.38278364	0.07254492	5.27650525	1.32E-07

16302	NEDD1	3492.38716	0.59159785	0.06785652	8.71836384	2.82E-18
16304	NEDD4L	13449.9699	0.77205744	0.06175628	12.5016832	7.31E-36
16311	NEGR1	161.959772	1.49726906	0.20247366	7.3948832	1.42E-13
16313	NEIL1	173.951488	-1.71573	0.18902974	-9.0765085	1.12E-19
16319	NEK2	1620.70501	-0.7359081	0.07723642	-9.5279929	1.60E-21
16323	NEK6	4542.82355	-0.5222674	0.06120899	-8.5325283	1.43E-17
16326	NEK9	3025.79441	0.46560234	0.06732972	6.91525678	4.67E-12
16327	NELFA	1318.45725	-0.2629053	0.0908394	-2.8941772	0.00380154
16328	NELFB	5192.61607	-0.6892361	0.07375965	-9.344352	9.25E-21
16330	NELFE	4493.51076	0.34106359	0.07486532	4.55569538	5.22E-06
16338	NES	504.443427	-3.3907171	0.13728051	-24.699188	1.09E-134
16348	NEURL1B	221.648918	-0.4488644	0.15441546	-2.9068616	0.00365075
16359	NEXN	906.144896	0.39038057	0.09693089	4.0274115	5.64E-05
16361	NF1	1842.70481	0.91073272	0.13755749	6.62074251	3.57E-11
16363	NF2	291.553832	0.39650063	0.14940118	2.65393243	0.00795598
16366	NFAT5	3770.28347	0.38524817	0.11268757	3.41872828	0.00062915
16367	NFATC1	86.7270174	1.20358157	0.24844409	4.84447656	1.27E-06
16368	NFATC2	2242.53626	0.94307635	0.08985551	10.4954756	9.06E-26
16370	NFATC3	1254.7391	0.42142687	0.08257822	5.10336602	3.34E-07
16372	NFE2	18.089872	-3.5643589	0.62884623	-5.668093	1.44E-08
16374	NFE2L2	8063.47336	0.24973775	0.06913965	3.61207707	0.00030375
16382	NFIL3	1569.5123	0.97912789	0.08681769	11.2779773	1.69E-29
16384	NFKB1	3464.02948	0.3062016	0.06226492	4.91772248	8.76E-07
16387	NFKBIB	1742.02639	1.34640018	0.11524741	11.6826935	1.56E-31
16389	NFKBIE	3353.24226	1.34070604	0.12762796	10.5047987	8.21E-26
16390	NFKBIL1	1092.3371	0.25843169	0.10380759	2.48952591	0.01279136
16393	NFS1	904.977921	-0.7286643	0.08368841	-8.7068728	3.12E-18
16395	NFX1	2525.25379	0.35057561	0.0754145	4.6486501	3.34E-06
16396	NFXL1	1546.68991	1.24461045	0.08348361	14.9084408	2.90E-50
16398	NFYB	1592.19621	-0.5165328	0.06962781	-7.4184838	1.18E-13
16399	NFYC	1915.05552	0.55927212	0.09912684	5.6419848	1.68E-08
16402	NGDN	1820.53065	0.72548007	0.10358045	7.00402512	2.49E-12
16403	NGEF	468.617329	-0.7794292	0.11675802	-6.6755949	2.46E-11
16404	NGF	342.71091	0.43641199	0.14914441	2.92610351	0.00343237
16406	NGFRAP1	7129.56475	-0.3021466	0.12715712	-2.3761676	0.01749351
16407	NGLY1	1793.86397	0.33510536	0.07490821	4.47354625	7.69E-06
16410	NHEJ1	520.438001	0.41534172	0.10908501	3.807505	0.00014038
16414	NHLRC2	1449.52628	0.2368749	0.10917369	2.16970694	0.03002905
16415	NHLRC3	680.411704	-0.7799952	0.09118795	-8.553709	1.19E-17
16418	NHS	876.493977	0.44974422	0.1053359	4.26961964	1.96E-05
16422	NICN1	828.263549	-0.5344566	0.09568343	-5.5856756	2.33E-08
16425	NIF3L1	1595.62689	-0.3456789	0.07799942	-4.4318144	9.34E-06
16426	NIFK	2786.52699	0.96111948	0.0724203	13.2714102	3.39E-40

16430	NINJ1	1295.6738	-0.7799206	0.08018484	-9.7265342	2.32E-22
16431	NINJ2	237.351101	-0.8512823	0.16926776	-5.0292055	4.93E-07
16432	NINL	1098.83324	-0.7973459	0.09438577	-8.4477333	2.97E-17
16433	NIP7	2239.55915	0.84616721	0.07150526	11.8336351	2.62E-32
16442	NIPSNAP1	1226.02991	-1.1185927	0.0779176	-14.356097	9.76E-47
16443	NIPSNAP3A	1037.95629	-0.6025923	0.10494453	-5.7420081	9.36E-09
16445	NISCH	2879.35483	-0.3732273	0.06266681	-5.9557403	2.59E-09
16448	NKAIN1	11.2938812	3.55390123	0.82157273	4.32572931	1.52E-05
16452	NKAP	781.617356	-0.7368139	0.09851691	-7.4790605	7.49E-14
16454	NKAPP1	22.7373743	-1.0887236	0.45639954	-2.3854617	0.0170577
16459	NKIRAS1	1431.13643	0.92022393	0.0870179	10.575111	3.89E-26
16465	NKX1-2	2.04856912	4.3842774	1.74923082	2.50640301	0.01219665
16471	NKX2-5	20.9225212	-2.4442752	0.50507328	-4.8394467	1.30E-06
16474	NKX3-1	1199.75006	0.46369523	0.09501249	4.88036056	1.06E-06
16482	NLGN2	2315.63481	-0.2258766	0.06862902	-3.2912696	0.00099736
16488	NLN	2254.25772	0.35835357	0.07339533	4.88251168	1.05E-06
16491	NLRC5	1418.5781	-0.753313	0.09053706	-8.3204932	8.76E-17
16494	NLRP11	9.1843792	-3.466459	0.87184057	-3.9760239	7.01E-05
16506	NLRX1	798.514024	-1.0529425	0.09885567	-10.651312	1.72E-26
16507	NMB	327.39316	-1.8405779	0.15814669	-11.638422	2.63E-31
16509	NMD3	2166.16772	0.72418788	0.07730151	9.36835307	7.37E-21
16510	NME1	4419.40762	1.09159511	0.11637523	9.37996056	6.60E-21
16513	NME3	586.40054	-1.0445364	0.13650392	-7.6520621	1.98E-14
16514	NME4	1507.78136	-0.2823356	0.0855252	-3.3011978	0.00096273
16516	NME6	1035.17874	0.32647386	0.08523316	3.83036198	0.00012795
16517	NME7	2138.67121	0.2343039	0.08692733	2.69539985	0.00703042
16520	NMI	1000.72055	-0.2768361	0.08766765	-3.1577907	0.0015897
16521	NMNAT1	532.415245	0.51622683	0.10862841	4.75222652	2.01E-06
16524	NMRAL1	1098.42555	-0.5269989	0.10005266	-5.2672153	1.39E-07
16534	NNMT	3261.18952	-0.2593703	0.1050431	-2.46918	0.01354231
16535	NNT	4694.06675	-0.6943262	0.07659403	-9.0650176	1.25E-19
16536	NNT-AS1	793.054256	0.29468241	0.09953256	2.9606634	0.00306977
16537	NOA1	1182.54307	-0.6122959	0.08376689	-7.3095218	2.68E-13
16540	NOC2L	4895.15036	0.90845301	0.07399407	12.2773761	1.20E-34
16541	NOC3L	1819.24942	0.56196485	0.08546089	6.57569599	4.84E-11
16543	NOCT	1004.79701	1.72324333	0.14383545	11.9806579	4.49E-33
16544	NOD1	338.319737	-0.7283587	0.1300333	-5.6013246	2.13E-08
16545	NOD2	285.868424	1.42443084	0.1374706	10.3617123	3.70E-25
16548	NOL10	2077.06567	0.46166473	0.07235711	6.38036477	1.77E-10
16549	NOL11	2993.28811	0.75476811	0.08003023	9.43103716	4.06E-21
16554	NOL6	4862.02214	0.77347339	0.0659833	11.7222601	9.80E-32
16555	NOL7	6097.82195	0.67090262	0.08823425	7.60365331	2.88E-14
16558	NOLC1	7710.0831	0.22693679	0.06619665	3.42822171	0.00060755

16563	NONO	12482.7056	-0.2069492	0.06360676	-3.2535722	0.00113964
16565	NOP14	6272.63233	0.80255747	0.05401104	14.8591384	6.07E-50
16567	NOP16	2452.50979	1.02226082	0.10258398	9.96511197	2.17E-23
16568	NOP2	2921.17651	0.8874253	0.06573654	13.4997258	1.57E-41
16569	NOP56	6611.30457	0.36138817	0.05842526	6.18547807	6.19E-10
16570	NOP58	4774.81318	0.91574057	0.05935884	15.4271972	1.07E-53
16576	NOSIP	1666.67817	-0.4921399	0.10681767	-4.6072891	4.08E-06
16577	NOSTRIN	54.5046927	-2.4409218	0.31609678	-7.7220712	1.14E-14
16578	NOTCH1	3286.72409	1.20985106	0.08970295	13.4873057	1.86E-41
16585	NOV	612.318408	1.39278588	0.10265257	13.5679587	6.20E-42
16589	NOX3	4.61260419	4.548235	1.42144187	3.19973338	0.00137555
16592	NOXA1	1019.93636	-1.3591298	0.10789711	-12.596536	2.21E-36
16597	NPAS2	3702.79147	-0.4155469	0.0787042	-5.2798562	1.29E-07
16604	NPC1	7739.53707	0.32527892	0.07623904	4.26656627	1.99E-05
16606	NPC2	7605.77014	-1.005504	0.06445009	-15.601283	7.13E-55
16634	NPL	18.1119166	2.29961975	0.5741226	4.00545065	6.19E-05
16636	NPM1	17099.4118	0.66428938	0.06070836	10.942305	7.23E-28
16641	NPPA-AS1	68.2407816	1.12162314	0.25224547	4.44655412	8.73E-06
16644	NPR1	22.5881819	-3.204911	0.57610758	-5.5630425	2.65E-08
16648	NPRL3	1767.59573	-0.2319665	0.07314215	-3.1714481	0.00151681
16652	NPTN	5837.76109	-0.5139481	0.06155699	-8.349144	6.88E-17
16656	NPTXR	358.597138	-0.7907583	0.11738805	-6.7362763	1.62E-11
16669	NR1D1	1650.9802	2.07709079	0.08941657	23.2293716	2.30E-119
16670	NR1D2	4760.7572	0.8828036	0.07702388	11.4614267	2.06E-30
16671	NR1H2	3265.21506	-0.7638596	0.06167917	-12.384402	3.17E-35
16672	NR1H3	437.294915	-1.2551635	0.10625946	-11.812252	3.37E-32
16676	NR2C1	1063.28958	-0.2979549	0.09901707	-3.0091272	0.00261999
16678	NR2C2AP	1049.93285	0.42208908	0.1209957	3.48846353	0.00048581
16683	NR2F2	8690.43439	-0.4843299	0.0530441	-9.130703	6.81E-20
16685	NR2F6	3334.25477	-0.4778827	0.10333721	-4.6244979	3.76E-06
16688	NR4A1	58.2213279	1.10350951	0.26692975	4.13408217	3.56E-05
16701	NRBP2	1460.57245	-0.6689167	0.08980374	-7.4486508	9.43E-14
16703	NRD1	9929.12943	0.67400084	0.0535378	12.5892514	2.42E-36
16705	NREP	602.61592	-0.3661113	0.10567194	-3.4646025	0.00053102
16708	NRG1	4606.30031	1.82142369	0.06331617	28.7671156	5.53E-182
16711	NRG2	20.133672	-2.3741045	0.51698528	-4.592209	4.39E-06
16715	NRGN	999.252643	-0.4905025	0.09598619	-5.1101369	3.22E-07
16716	NRIP1	1658.93883	0.18124967	0.08974162	2.01968359	0.04341622
16722	NRM	1489.7319	-0.4133993	0.08087942	-5.1113045	3.20E-07
16727	NRP2	2947.13554	-0.2320293	0.08921159	-2.6008872	0.0092983
16728	NRROS	284.681816	-1.4108408	0.14467775	-9.7516086	1.82E-22
16730	NRSN2	2572.41517	-0.4530544	0.06795561	-6.6669175	2.61E-11
16732	NRTN	114.787227	-0.6671489	0.20856786	-3.1987137	0.00138042

16736	NSA2	2649.76263	-0.2761087	0.07160742	-3.8558667	0.00011532
16738	NSDHL	1308.38831	-0.2452829	0.08869909	-2.7653368	0.00568641
16744	NSMAF	2164.07139	0.22800082	0.07474705	3.05029865	0.00228614
16746	NSMCE2	209.391158	-0.943801	0.15678537	-6.0197003	1.75E-09
16747	NSMCE4A	1205.83648	-0.7033037	0.0880406	-7.9884024	1.37E-15
16749	NSRP1	2338.43647	0.4979147	0.07331985	6.79099434	1.11E-11
16750	NSUN2	9228.3535	0.8126412	0.05322907	15.266868	1.27E-52
16752	NSUN4	547.44416	0.55091821	0.10968539	5.02271273	5.09E-07
16753	NSUN5	1166.25373	0.42257847	0.1291929	3.27091108	0.00107202
16754	NSUN5P1	231.385078	-0.7505375	0.14487712	-5.180511	2.21E-07
16755	NSUN5P2	308.720542	-0.6132575	0.16196245	-3.7864179	0.00015283
16756	NSUN6	436.451909	0.40905558	0.10516244	3.88974999	0.00010035
16762	NT5C2	7317.71387	-0.1588263	0.08039087	-1.9756759	0.0481915
16763	NT5C3A	2105.51804	0.98133031	0.08617884	11.3871373	4.85E-30
16765	NT5DC1	526.76086	-1.3808191	0.11529811	-11.976078	4.74E-33
16767	NT5DC3	1588.88996	-0.4729563	0.07948559	-5.9502153	2.68E-09
16771	NTF3	118.465943	5.89904729	0.47373278	12.4522676	1.36E-35
16773	NTHL1	635.898744	-0.4422982	0.15821059	-2.7956296	0.00517987
16776	NTMT1	2034.72703	0.4234244	0.07939752	5.33296758	9.66E-08
16793	NUB1	6304.05928	0.4522861	0.06512878	6.94448955	3.80E-12
16795	NUBP2	1642.16553	-0.3853111	0.11762828	-3.275667	0.00105413
16796	NUBPL	387.384268	-0.4485358	0.1142538	-3.9257844	8.64E-05
16797	NUCB1	5999.82618	-0.7654272	0.05543647	-13.807287	2.30E-43
16802	NUDCD1	7722.48772	1.14938012	0.07249615	15.8543598	1.31E-56
16803	NUDCD2	1954.40199	-0.1994294	0.08349059	-2.3886453	0.01691062
16804	NUDCD3	4751.01163	-0.2394793	0.06773015	-3.5357858	0.00040656
16805	NUDT1	756.427883	-0.5866659	0.12543891	-4.6769057	2.91E-06
16808	NUDT12	612.000881	-0.3709604	0.1030019	-3.6014909	0.0003164
16810	NUDT14	990.722704	-0.3953408	0.15185656	-2.6033832	0.00923087
16812	NUDT16	1160.10283	0.36688351	0.07382451	4.96967097	6.71E-07
16813	NUDT16L1	564.145226	-0.6724846	0.12617671	-5.3297047	9.84E-08
16818	NUDT2	465.221043	-0.7305736	0.11218872	-6.5120058	7.42E-11
16820	NUDT22	1422.11617	-0.6417229	0.12197576	-5.2610693	1.43E-07
16822	NUDT4	713.059251	0.40457282	0.09629138	4.2015477	2.65E-05
16825	NUDT5	2852.65458	0.23951865	0.07704386	3.10886094	0.0018781
16826	NUDT6	606.400442	-0.4241815	0.105346	-4.0265556	5.66E-05
16831	NUF2	1770.21137	-0.5095632	0.08018386	-6.3549349	2.09E-10
16832	NUFIP1	595.209773	0.67909642	0.09753571	6.96254173	3.34E-12
16833	NUFIP2	7850.60955	0.88300775	0.08262973	10.6863207	1.18E-26
16835	NUMA1	9190.6722	-0.6371008	0.08100961	-7.8645089	3.71E-15
16837	NUMBL	1001.34938	-0.2410025	0.08677382	-2.7773637	0.00548018
16839	NUP133	3323.91194	-0.1234369	0.05937959	-2.0787773	0.03763783
16840	NUP153	9203.21802	0.92187702	0.07171632	12.8544949	8.12E-38

16842	NUP160	3860.11652	0.58941843	0.07834358	7.52350639	5.33E-14
16845	NUP210	2057.21386	-0.3394737	0.07554243	-4.4938142	7.00E-06
16849	NUP35	1311.51916	0.54825982	0.08334202	6.57843182	4.75E-11
16851	NUP43	2940.10826	0.50748499	0.06158238	8.24074942	1.71E-16
16853	NUP50-AS1	652.179026	0.61178521	0.09330851	6.55658559	5.51E-11
16855	NUP62	3582.6217	-0.3942661	0.06870992	-5.7381246	9.57E-09
16858	NUP88	3300.64087	0.24745313	0.06245608	3.96203416	7.43E-05
16859	NUP93	2588.80286	0.15815712	0.06915499	2.28699519	0.0221961
16860	NUP98	7571.82628	0.52437358	0.0755045	6.94493139	3.79E-12
16861	NUPL1	3010.57015	0.47787771	0.08739637	5.46793554	4.55E-08
16862	NUPL2	985.525019	0.53407315	0.09926755	5.38013834	7.44E-08
16863	NUPR1	1345.07787	1.16709501	0.10974537	10.6345718	2.06E-26
16866	NUSAP1	3724.76701	-0.8933754	0.10839471	-8.2418732	1.70E-16
16872	NUTM2B-AS	85.2441243	0.93028246	0.26939936	3.45317249	0.00055403
16877	NWD1	69.1708862	-0.8036938	0.26378153	-3.0468163	0.00231279
16879	NXF1	3503.13949	0.43451715	0.0636909	6.82228009	8.96E-12
16895	NXPH4	63.1801842	0.63828551	0.28919014	2.20714825	0.0273037
16899	NYAP2	74.6706327	2.23218652	0.26484312	8.42833483	3.51E-17
16903	OAF	3134.68807	0.27409527	0.06388544	4.29041869	1.78E-05
16904	OARD1	2347.82992	0.54004854	0.08515071	6.34226727	2.26E-10
16905	OAS1	75.0871097	-2.1990876	0.27687594	-7.9425015	1.98E-15
16906	OAS2	717.786682	-1.197103	0.09070382	-13.197934	9.02E-40
16909	OAT	6773.98647	-0.7952415	0.06257992	-12.707614	5.36E-37
16911	OAZ2	2615.19292	-0.3309182	0.07363067	-4.4942984	6.98E-06
16913	OBFC1	854.805543	-1.3880204	0.08653635	-16.039738	6.74E-58
16917	OBSL1	3250.50285	-0.3831748	0.09991044	-3.8351829	0.00012547
16920	OCEL1	831.827572	-0.4935715	0.10781609	-4.5779023	4.70E-06
16924	OCLN	234.649713	1.10340578	0.17477926	6.31313909	2.73E-10
16930	ODC1	4846.28659	1.12323101	0.07517183	14.9421804	1.75E-50
16933	ODF2L	1835.6549	0.40888125	0.07996866	5.11301871	3.17E-07
16942	OGFOD1	2183.69709	0.93030028	0.07631071	12.1909524	3.47E-34
16944	OGFOD3	1500.11019	-0.7344685	0.11445753	-6.4169526	1.39E-10
16949	OGG1	741.331736	-0.4947661	0.08844617	-5.5939802	2.22E-08
16951	OGT	14398.5574	1.0891878	0.06365358	17.11118	1.22E-65
16952	OIP5	586.549073	-0.3927842	0.11281784	-3.4815789	0.00049847
16953	OIP5-AS1	809.140813	-0.1783212	0.08486248	-2.1012965	0.03561495
16955	OLA1	6815.44677	0.61369275	0.05947078	10.3192311	5.77E-25
16958	OLFM2	13.9501193	1.56436547	0.57127809	2.73836069	0.00617463
16962	OLFML2A	1841.96126	-1.3781407	0.09353864	-14.733384	3.93E-49
16975	ONECUT2	277.595272	2.39214291	0.17459056	13.7014444	9.95E-43
16985	OPHN1	1208.16555	0.59536263	0.08935628	6.66279594	2.69E-11
16986	OPLAH	635.94795	-0.7053231	0.10058881	-7.0119443	2.35E-12
16996	OPRL1	141.254698	-1.3906118	0.18411097	-7.5531174	4.25E-14

17072	OR1G1	3.85370978	-5.4912144	1.494188	-3.6750492	0.0002378
17402	ORAI3	1103.92856	-1.3530414	0.10064088	-13.444253	3.33E-41
17403	ORAOV1	1415.71353	0.35015253	0.08219437	4.26005499	2.04E-05
17407	ORC4	2758.24117	0.52244691	0.07801216	6.69699347	2.13E-11
17408	ORC5	1139.90559	0.31921217	0.08311788	3.84047535	0.0001228
17409	ORC6	1238.33968	0.88706289	0.07791086	11.3856125	4.93E-30
17416	OSBP	4562.63026	0.61603532	0.07388802	8.33741771	7.59E-17
17418	OSBPL10	2271.84626	0.30247815	0.06884094	4.39387001	1.11E-05
17424	OSBPL5	632.170828	-0.8721888	0.1149367	-7.588427	3.24E-14
17426	OSBPL7	370.406897	-1.649256	0.12126464	-13.600469	3.98E-42
17427	OSBPL8	6743.71261	0.7469705	0.09956754	7.50214851	6.28E-14
17428	OSBPL9	6056.81411	0.75849332	0.07889746	9.61365986	7.00E-22
17433	OSGEP	1146.49496	-0.4228995	0.09452051	-4.4741559	7.67E-06
17434	OSGEPL1	495.4878	-0.6650451	0.10703817	-6.2131584	5.19E-10
17436	OSGIN1	704.071662	0.73178785	0.09643263	7.58859163	3.23E-14
17443	OST4	1784.44792	-1.0071272	0.07776688	-12.950592	2.33E-38
17447	OSTM1	2946.86058	0.18603365	0.07495429	2.48196142	0.01306614
17462	OTUB2	1984.76255	1.22375803	0.06890021	17.7613107	1.41E-70
17465	OTUD4	3871.45268	0.88984166	0.08294653	10.727895	7.53E-27
17466	OTUD5	2204.94916	-0.6954329	0.06967881	-9.9805519	1.85E-23
17468	OTUD6B	1344.56392	1.01311933	0.0890487	11.3771375	5.44E-30
17471	OTUD7B	2143.69658	0.71806577	0.06803379	10.5545454	4.84E-26
17486	OXA1L	3439.9962	-0.2522253	0.07178001	-3.5138656	0.00044164
17487	OXCT1	566.582768	0.68283112	0.10847483	6.29483494	3.08E-10
17496	OXSR1	7322.66686	0.92742499	0.05415922	17.1240477	9.82E-66
17498	OXTR	139.404051	-1.5546965	0.19430711	-8.0012334	1.23E-15
17499	P2RX1	5.32916544	-1.9110269	0.93705652	-2.0393934	0.04141078
17510	P2RY11	133.020184	1.1466436	0.20273263	5.65594012	1.55E-08
17514	P2RY2	680.499642	-0.6544688	0.09487311	-6.8983596	5.26E-12
17516	P2RY6	67.3381592	-1.9602201	0.27139044	-7.2228784	5.09E-13
17519	P3H2	4021.67843	-0.2920484	0.06153877	-4.7457618	2.08E-06
17522	P3H4	440.594165	-0.7829263	0.15184414	-5.1561178	2.52E-07
17523	P4HA1	5191.97753	-0.294863	0.06012638	-4.9040541	9.39E-07
17528	P4HTM	1060.59195	-0.519865	0.10072107	-5.1614318	2.45E-07
17529	PA2G4	5618.67941	0.37817258	0.0698269	5.41585797	6.10E-08
17532	PABPC1	51374.1619	0.32822201	0.05332264	6.1553972	7.49E-10
17533	PABPC1L	1124.8153	0.21677125	0.07987957	2.7137257	0.00665312
17539	PABPC4	10028.9592	0.73379556	0.05375548	13.6506176	2.00E-42
17540	PABPC4L	43.467746	-2.4960737	0.35011548	-7.1292869	1.01E-12
17548	PACRGL	687.207259	0.41404825	0.09344883	4.43074848	9.39E-06
17552	PACSIN2	5075.13481	0.38776121	0.06075564	6.38230755	1.74E-10
17553	PACSIN3	1426.45291	-0.3280978	0.11095721	-2.956976	0.00310672
17554	PADI1	165.301318	-1.2167883	0.16266262	-7.480442	7.41E-14

17563	PAFAH1B3	2895.33509	-0.4333816	0.13258291	-3.2687592	0.0010802
17564	PAFAH2	270.573782	0.51876827	0.12720537	4.07819489	4.54E-05
17571	PAGE5	46.1216077	-3.3527381	0.38432198	-8.7237742	2.69E-18
17574	PAICS	14053.1267	0.5068717	0.06502654	7.79484385	6.45E-15
17576	PAIP2	4079.5788	0.1377565	0.06252959	2.20306103	0.02759045
17577	PAIP2B	73.9280157	1.23671016	0.2412366	5.12654438	2.95E-07
17578	PAK1	1810.46313	-0.2814592	0.06649308	-4.2329094	2.31E-05
17579	PAK1IP1	2709.83452	1.19390725	0.06958053	17.1586388	5.42E-66
17580	PAK2	9319.3669	0.48485858	0.07400361	6.55182381	5.68E-11
17582	PAK4	2870.64502	-0.6251696	0.07279138	-8.5885108	8.81E-18
17588	PALM	231.852844	-1.5269089	0.16298699	-9.3682872	7.37E-21
17592	PALMD	3.19976209	-5.2186301	1.57514809	-3.3131044	0.00092267
17593	PAM	9886.91726	-0.1636881	0.05455807	-3.0002547	0.00269754
17600	PANK1	418.796539	-0.8718277	0.12568849	-6.9364165	4.02E-12
17602	PANK3	2369.10428	1.07074166	0.10115481	10.5851774	3.49E-26
17605	PANX1	2573.18799	0.76495323	0.09061801	8.44151464	3.13E-17
17608	PAOX	461.519324	-1.3400094	0.12330784	-10.867187	1.65E-27
17613	PAPLN	129.294961	-0.6378758	0.1776088	-3.5914649	0.00032882
17620	PAPSS1	2484.04056	-0.2222122	0.06481888	-3.4282012	0.00060759
17621	PAPSS2	3831.79228	-0.9669477	0.07807952	-12.384141	3.18E-35
17622	PAQR3	1734.75641	0.1802307	0.09138473	1.97221898	0.04858461
17623	PAQR4	1193.23678	-0.2741814	0.07516651	-3.6476536	0.00026465
17627	PAQR8	830.445409	-1.3345744	0.09064654	-14.722839	4.60E-49
17633	PARD6A	217.63882	-0.9886818	0.18230666	-5.4231802	5.85E-08
17637	PARG	1145.78377	-0.6608444	0.08451115	-7.819612	5.30E-15
17644	PARP1	6902.27902	-0.380005	0.06073608	-6.2566602	3.93E-10
17645	PARP10	2157.95929	-0.6783898	0.10466539	-6.4815107	9.08E-11
17647	PARP12	4162.86247	0.79139275	0.06185112	12.7951242	1.75E-37
17651	PARP2	1652.28031	-0.5321687	0.07452443	-7.1408632	9.27E-13
17652	PARP3	885.550581	-1.1027219	0.08643092	-12.758419	2.80E-37
17654	PARP6	2624.20912	0.6213075	0.06718546	9.2476487	2.29E-20
17656	PARP9	613.066852	-0.3771675	0.1101493	-3.4241478	0.00061673
17657	PARPBP	1318.03759	-0.4608793	0.07357089	-6.2644247	3.74E-10
17658	PARS2	347.447205	0.67137623	0.1163473	5.77044947	7.91E-09
17661	PARVA	5490.96165	-0.2832346	0.06512249	-4.3492593	1.37E-05
17674	PAWR	3415.30225	0.80719663	0.08753648	9.22126007	2.94E-20
17682	PAX8	3158.87601	1.88572846	0.06725674	28.0377625	5.63E-173
17683	PAX8-AS1	5928.49804	0.36604063	0.0723501	5.05929704	4.21E-07
17685	PAXBP1	1909.41834	0.74328413	0.07261115	10.2365007	1.36E-24
17687	PAXIP1	1810.47399	-0.3175135	0.0848416	-3.742427	0.00018225
17689	PAXIP1-AS2	51.0823015	-1.1382801	0.28888705	-3.9402256	8.14E-05
17690	PBDC1	2966.69002	0.48800375	0.10807105	4.51558268	6.31E-06
17691	PBK	3418.60844	-0.2773496	0.07089465	-3.9121376	9.15E-05

17695	PBX1	774.804911	-0.8761873	0.11200947	-7.8224391	5.18E-15
17697	PBX3	801.695894	-0.373581	0.09443413	-3.9559953	7.62E-05
17699	PBXIP1	1815.00793	-0.8354706	0.0914388	-9.1369377	6.42E-20
17716	PCBP2	10042.8838	-0.2584531	0.11826588	-2.1853567	0.02886271
17720	PCCA	163.865113	-0.7472547	0.19043768	-3.9238807	8.71E-05
17730	PCDH18	11.4006636	-4.0792625	0.89885015	-4.538312	5.67E-06
17755	PCDHB10	114.072554	-0.5714786	0.18901025	-3.0235322	0.00249842
17756	PCDHB11	90.9733863	-0.9372027	0.22809056	-4.1089063	3.98E-05
17757	PCDHB12	43.6109566	-1.9091555	0.3458691	-5.5198787	3.39E-08
17758	PCDHB13	116.944668	-0.822477	0.20381135	-4.0354817	5.45E-05
17759	PCDHB14	259.48984	-0.9317516	0.14172642	-6.5742971	4.89E-11
17761	PCDHB16	29.9662562	-0.8436078	0.36752794	-2.295357	0.02171268
17772	PCDHB9	66.3485139	-0.9503498	0.28678387	-3.313819	0.00092031
17774	PCDHGA10	13.8459074	-1.7511071	0.60723079	-2.8837588	0.0039296
17779	PCDHGA4	58.5755804	-1.268794	0.31811854	-3.9884313	6.65E-05
17782	PCDHGA7	16.942375	-1.2744465	0.5506902	-2.3142712	0.02065285
17785	PCDHGB1	172.627523	-1.1584709	0.19430504	-5.9621249	2.49E-09
17786	PCDHGB2	298.111787	-0.7550697	0.17288773	-4.367399	1.26E-05
17793	PCDHGC3	76.554239	-1.0734166	0.23819781	-4.5064082	6.59E-06
17795	PCDHGC5	13.8487993	-1.4602353	0.55837036	-2.6151734	0.00891822
17796	PCED1A	1830.53836	-0.8189885	0.08676044	-9.4396529	3.74E-21
17799	PCF11	1568.00683	0.58579665	0.08504427	6.8881377	5.65E-12
17804	PCGF5	4801.59287	-0.166389	0.0712688	-2.3346681	0.01956077
17807	PCIF1	2489.21125	-0.6423051	0.06399889	-10.036192	1.06E-23
17809	PCK2	1465.15986	0.85405924	0.0986361	8.65868806	4.77E-18
17812	PCMT1	5226.10083	0.30177319	0.06279516	4.80567586	1.54E-06
17813	PCMTD1	742.283781	-0.4953499	0.09241994	-5.3597729	8.33E-08
17814	PCMTD2	2300.18143	-0.2551649	0.06354881	-4.0152581	5.94E-05
17815	PCNA	10069.272	-0.2377516	0.06758573	-3.5177778	0.00043518
17821	PCNXL2	538.379655	0.80525566	0.13410298	6.0047561	1.92E-09
17823	PCNXL4	8537.37912	0.36723887	0.06265434	5.86134782	4.59E-09
17824	PCOLCE	71.4662634	-0.6912321	0.25029232	-2.7616994	0.00575014
17833	PCSK4	164.095843	-1.2050496	0.1687753	-7.1399641	9.34E-13
17836	PCSK7	1337.95059	0.37934011	0.07973655	4.75741802	1.96E-06
17837	PCSK9	123.115574	1.37013973	0.20762651	6.59905976	4.14E-11
17839	PCYOX1	4021.5022	-0.5499985	0.08224187	-6.6875732	2.27E-11
17840	PCYOX1L	187.450378	-0.9422054	0.16636034	-5.6636422	1.48E-08
17841	PCYT1A	1096.95762	0.46611569	0.10552915	4.41693793	1.00E-05
17844	PCYT2	2263.53118	-0.7106999	0.07057366	-10.070327	7.47E-24
17849	PDCD11	2274.07404	0.16446535	0.07130534	2.30649427	0.02108303
17850	PDCD1LG2	180.604882	0.54313176	0.16511854	3.28934442	0.00100421
17851	PDCD2	3764.40583	0.47502281	0.0676382	7.02299635	2.17E-12
17852	PDCD2L	534.544436	0.45431012	0.13152442	3.45418838	0.00055195

17853	PDCD4	2185.08661	-0.9063636	0.07760616	-11.679017	1.63E-31
17862	PDCL3	754.874488	-0.3894062	0.10674825	-3.6478932	0.0002644
17863	PDCL3P4	79.1246648	1.00779187	0.2671411	3.77250774	0.00016161
17866	PDE11A	93.6068363	-1.7274409	0.22728478	-7.6003369	2.95E-14
17868	PDE1A	13.4826418	-3.5215328	0.76098099	-4.6276225	3.70E-06
17874	PDE4A	2163.31192	-0.2221036	0.07303405	-3.041096	0.00235719
17875	PDE4B	104.634273	-0.5565524	0.21008901	-2.6491266	0.00807001
17881	PDE6B	6.72807833	2.7265967	1.03238607	2.64106306	0.00826463
17887	PDE7B	1145.55986	-0.6569175	0.08639627	-7.6035398	2.88E-14
17888	PDE8A	2721.12756	-0.7801862	0.08510365	-9.1674817	4.84E-20
17889	PDE8B	53.1241713	-2.6973956	0.33996487	-7.9343365	2.12E-15
17892	PDGFA	406.778394	-0.3084652	0.11743253	-2.6267439	0.00862062
17893	PDGFB	128.934075	-4.1779346	0.27573508	-15.151988	7.35E-52
17897	PDGFRB	686.029419	-1.2264093	0.11052678	-11.096037	1.31E-28
17898	PDGFRL	142.593696	-1.5192913	0.17837645	-8.5173308	1.63E-17
17901	PDHB	6203.74474	-0.4918977	0.06746223	-7.2914535	3.07E-13
17902	PDHX	2699.60645	0.56958442	0.068686	8.29258348	1.11E-16
17904	PDIA3	11700.6015	-0.1307599	0.05263519	-2.4842676	0.01298182
17907	PDIA5	1319.45347	-0.3193485	0.08201452	-3.8938046	9.87E-05
17909	PDIK1L	573.267154	0.47267835	0.10785765	4.38242785	1.17E-05
17912	PDK2	1132.689	-0.9049224	0.08305062	-10.896034	1.20E-27
17913	PDK3	889.35231	-0.4077397	0.08833674	-4.6157434	3.92E-06
17915	PDLIM1	5889.18361	-0.5800519	0.08469159	-6.8489907	7.44E-12
17917	PDLIM3	120.125752	-1.7442327	0.21966901	-7.9402764	2.02E-15
17921	PDP1	9454.40637	0.39078253	0.06207047	6.29578831	3.06E-10
17922	PDP2	2097.20492	0.62160824	0.0761836	8.15934443	3.37E-16
17926	PDRG1	1710.10314	0.53509283	0.08651693	6.18483346	6.22E-10
17930	PDSS2	596.832247	-0.3843864	0.10319419	-3.7248842	0.0001954
17938	PDZD11	1550.83367	-0.8484788	0.0738936	-11.48244	1.62E-30
17943	PDZD8	2034.34937	0.33616649	0.0858206	3.91708369	8.96E-05
17951	PEA15	17086.9754	0.49587407	0.0602543	8.22968832	1.88E-16
17953	PEAR1	280.643014	-0.7124988	0.13969932	-5.1002314	3.39E-07
17954	PEBP1	5830.16328	-0.8941263	0.06737891	-13.270121	3.45E-40
17956	PECAM1	79.6751928	-0.8934265	0.24244368	-3.6850889	0.00022862
17957	PECR	382.314242	-1.0332227	0.12681986	-8.1471684	3.73E-16
17958	PEF1	2614.97158	0.58698964	0.09308009	6.30628585	2.86E-10
17959	PEG10	2565.59222	-0.7706369	0.08324916	-9.2569931	2.10E-20
17964	PELI3	943.601286	-0.7517879	0.08843763	-8.5007697	1.88E-17
17965	PELO	3579.72062	0.6625813	0.0580863	11.4068438	3.86E-30
17966	PELP1	2918.1136	0.47464994	0.09430617	5.03307406	4.83E-07
17969	PEPD	4124.0526	-0.5720327	0.10374374	-5.5139012	3.51E-08
17970	PER1	368.613773	0.54643532	0.13968509	3.91190869	9.16E-05
17971	PER2	207.84594	1.27635107	0.16394704	7.78514264	6.96E-15

17972	PER3	1364.27284	1.55093961	0.09661987	16.051973	5.54E-58
17974	PERM1	22.0894049	2.75348912	0.53026389	5.19267701	2.07E-07
17975	PERP	4581.94808	-0.7497299	0.05486061	-13.666089	1.62E-42
17976	PES1	4291.34875	0.30898951	0.08823793	3.50177648	0.00046217
17977	PET100	729.749604	-0.6302028	0.17316206	-3.6393817	0.00027329
17980	PEX10	1154.19212	0.52475823	0.0820473	6.39580137	1.60E-10
17981	PEX11A	172.77383	-0.76474	0.16548349	-4.6212468	3.81E-06
17998	PFAS	2428.12929	0.45063844	0.08336914	5.4053386	6.47E-08
18000	PFDN2	5658.81528	1.58186808	0.09405088	16.8192797	1.76E-63
18001	PFDN4	2465.13995	0.39817294	0.07772605	5.12277363	3.01E-07
18002	PFDN5	2611.66069	-0.4506527	0.09009463	-5.0019925	5.67E-07
18005	PFKFB2	1539.77039	-0.413707	0.08138697	-5.0832092	3.71E-07
18007	PFKFB4	1724.0472	-0.5323067	0.07307192	-7.284696	3.22E-13
18008	PFKL	6211.65927	-0.866691	0.07180761	-12.069627	1.53E-33
18010	PFKP	16474.407	-0.1776035	0.06162674	-2.8819227	0.00395257
18011	PFN1	32343.5791	-0.2720931	0.08475737	-3.2102591	0.00132615
18019	PGAM1	2749.7684	-0.7047197	0.10912341	-6.458007	1.06E-10
18023	PGAM5	1696.75744	0.27067371	0.07124993	3.7989328	0.00014532
18025	PGAP2	423.208536	-0.7297753	0.11172983	-6.5316066	6.51E-11
18026	PGAP3	285.19437	-0.9132114	0.14019676	-6.5137836	7.33E-11
18034	PGD	5590.36091	0.6985734	0.05837831	11.9663181	5.33E-33
18035	PGF	155.451411	-2.4371936	0.19700668	-12.371121	3.75E-35
18036	PGGT1B	659.495933	0.61504864	0.10224418	6.01548803	1.79E-09
18037	PGK1	40508.3825	-0.2293126	0.04939831	-4.6421136	3.45E-06
18045	PGM2	3465.89605	-0.3116785	0.06343835	-4.9130935	8.97E-07
18046	PGM2L1	1057.41856	0.99316273	0.08645128	11.4881202	1.51E-30
18047	PGM3	4124.92287	0.4398087	0.06540117	6.72478365	1.76E-11
18054	PGPEP1	1359.35559	-0.9114803	0.07561248	-12.054628	1.83E-33
18057	PGRMC1	3548.79978	-0.1461567	0.06634948	-2.2028314	0.02760664
18063	PHACTR4	1671.68965	0.40858472	0.0742422	5.50340252	3.73E-08
18068	PHC2	7660.35592	0.84361396	0.05328556	15.8319419	1.87E-56
18077	PHF14	1308.28239	-0.2999365	0.07523155	-3.9868442	6.70E-05
18078	PHF19	5001.16987	-0.6844885	0.05684824	-12.040628	2.17E-33
18084	PHF23	2542.21441	0.48745219	0.06805092	7.1630511	7.89E-13
18085	PHF24	3.42314792	5.12945854	1.53110921	3.3501585	0.00080765
18086	PHF3	7257.79688	0.42333963	0.08691918	4.87049734	1.11E-06
18087	PHF5A	1321.55986	0.70736589	0.08321888	8.50006529	1.89E-17
18097	PHKB	1547.02068	-0.6583167	0.07703849	-8.5452955	1.28E-17
18100	PHLDA1	45717.0421	1.48896377	0.06735322	22.1067953	2.72E-108
18101	PHLDA2	3326.32438	0.2391455	0.06746276	3.54485201	0.00039283
18102	PHLDA3	1477.5092	-0.406779	0.10882599	-3.7378844	0.00018558
18103	PHLDB1	2602.78084	-0.8323873	0.06058972	-13.738093	6.00E-43
18106	PHLPP1	913.623122	-0.2693766	0.10640838	-2.5315352	0.01135644

18107	PHLPP2	915.702416	0.64710077	0.09196305	7.03653043	1.97E-12
18115	PHTF1	1699.02869	-0.4979337	0.07215971	-6.9004389	5.18E-12
18117	PHYH	1026.66804	-0.9970969	0.09306588	-10.713882	8.76E-27
18118	PHYHD1	889.849291	-1.654497	0.08937919	-18.510986	1.68E-76
18123	PI16	115.058788	-1.7625815	0.19700723	-8.9467861	3.66E-19
18126	PI4K2B	2116.72203	-0.9421079	0.07333323	-12.846943	8.95E-38
18134	PIAS3	1520.02825	-0.9036828	0.07620036	-11.859299	1.93E-32
18137	PICALM	12414.2232	0.52954599	0.10734293	4.93321702	8.09E-07
18139	PID1	327.159692	1.60025018	0.1415217	11.3074545	1.21E-29
18140	PIDD1	666.129698	-0.4689247	0.12218409	-3.8378536	0.00012411
18143	PIF1	323.238097	-1.1997342	0.12356897	-9.7090246	2.76E-22
18145	PIGA	887.951893	0.49974947	0.08612304	5.80273813	6.52E-09
18146	PIGB	402.628835	-0.8166602	0.12451374	-6.5587962	5.42E-11
18150	PIGG	2065.56831	-0.5676238	0.07427125	-7.6425777	2.13E-14
18153	PIGL	197.807153	0.54813148	0.1573104	3.48439437	0.00049325
18154	PIGM	322.279871	0.4931114	0.13900995	3.54731	0.00038919
18158	PIGQ	1259.95435	-0.577827	0.08585401	-6.7303445	1.69E-11
18160	PIGS	2431.38677	-0.7218667	0.06843461	-10.54827	5.17E-26
18162	PIGU	1113.52146	-0.5572834	0.07895864	-7.0579155	1.69E-12
18164	PIGW	905.08759	0.34383436	0.08549122	4.02186773	5.77E-05
18165	PIGX	1479.95232	-0.5508414	0.07389112	-7.4547713	9.00E-14
18167	PIGZ	126.298194	-1.2153967	0.19091924	-6.3660254	1.94E-10
18168	PIH1D1	4323.48785	-0.4159727	0.09828172	-4.2324524	2.31E-05
18171	PIK3AP1	151.916559	-0.7180774	0.16633978	-4.3169313	1.58E-05
18172	PIK3C2A	3396.11697	0.72780879	0.10755706	6.76672268	1.32E-11
18173	PIK3C2B	245.97234	-0.8824878	0.13707799	-6.4378519	1.21E-10
18175	PIK3C3	2161.93504	-0.5255143	0.06862699	-7.6575443	1.90E-14
18178	PIK3CD	1030.60622	0.30709668	0.07755058	3.9599533	7.50E-05
18180	PIK3CD-AS2	19.7446131	-1.4821296	0.48902434	-3.0307891	0.00243916
18184	PIK3R1	672.898468	0.91707312	0.11304434	8.1125083	4.96E-16
18185	PIK3R2	3394.9966	-0.693013	0.06617953	-10.471713	1.17E-25
18194	PIM2	1003.73192	-0.7649196	0.08374693	-9.1337032	6.62E-20
18195	PIM3	4835.42123	0.30716284	0.12289733	2.49934502	0.01244231
18196	PIN1	1955.24897	-0.9488466	0.07853087	-12.082467	1.31E-33
18198	PIN4	978.911797	-0.3773894	0.0861648	-4.3798557	1.19E-05
18202	PINLYP	27.8280891	0.75130286	0.37719992	1.99178958	0.04639415
18203	PINX1	522.963242	0.44211965	0.09855408	4.48606116	7.26E-06
18207	PIP4K2C	1101.58702	0.22036739	0.09978326	2.2084606	0.02721218
18208	PIP5K1A	5545.26016	1.04106672	0.07598979	13.7000863	1.01E-42
18215	PIR	123.773607	-0.7406288	0.19348567	-3.8278224	0.00012928
18218	PISD	1123.4315	0.16525047	0.07643533	2.16196442	0.03062092
18220	PITHD1	2379.68749	1.16423184	0.09146663	12.7284881	4.11E-37
18221	PITPNA	3394.86	0.30042948	0.07178839	4.18493111	2.85E-05

18223	PITPNB	4576.43232	0.16473951	0.0594283	2.77207153	0.00557008
18226	PITPNM2	1142.37439	-0.3522688	0.09374395	-3.7577767	0.00017143
18227	PITPNM3	3063.74992	-0.2969293	0.07909708	-3.7539858	0.00017404
18228	PITRM1	5721.87564	0.1655584	0.06553936	2.52609115	0.01153396
18244	PKD1P6	547.183559	0.6391047	0.10254518	6.23242094	4.59E-10
18259	PKMYT1	1934.38936	0.21102869	0.09123822	2.3129419	0.02072583
18263	PKN3	1582.53049	-0.5326479	0.07827323	-6.8049824	1.01E-11
18269	PKP3	1226.8795	-1.1147765	0.09431143	-11.820164	3.07E-32
18275	PLA2G15	1292.82073	-0.7391611	0.08798972	-8.4005392	4.44E-17
18294	PLA2R1	555.777171	-0.7586085	0.09933038	-7.6372255	2.22E-14
18295	PLAA	2777.18885	0.32863086	0.06754899	4.86507476	1.14E-06
18298	PLAC8	3175.15826	-0.2493237	0.08127472	-3.0676656	0.00215738
18303	PLAGL2	1883.09574	0.47129579	0.09903249	4.75900164	1.95E-06
18308	PLBD1	220.724272	-2.2145994	0.16251924	-13.626691	2.78E-42
18313	PLCB3	3676.09425	-0.3704629	0.07069578	-5.2402408	1.60E-07
18314	PLCB4	18.6342319	7.56844797	1.25883445	6.01226633	1.83E-09
18315	PLCD1	403.876777	-0.6248047	0.11779053	-5.3043715	1.13E-07
18317	PLCD4	23.1487309	-2.0100968	0.46498214	-4.322955	1.54E-05
18328	PLCXD1	517.980312	0.4307074	0.09870016	4.36379655	1.28E-05
18329	PLCXD2	42.8606332	2.23322182	0.39993966	5.58389684	2.35E-08
18331	PLCXD3	12.1275423	4.43493613	0.97515617	4.54792398	5.42E-06
18334	PLD2	455.191812	-1.0088255	0.10845174	-9.3020686	1.38E-20
18335	PLD3	6743.12645	-0.4641353	0.07537409	-6.1577559	7.38E-10
18338	PLD6	1204.93272	1.49957753	0.0800967	18.7220896	3.27E-78
18342	PLEKHA1	850.463681	-1.2397843	0.09201616	-13.473549	2.24E-41
18343	PLEKHA2	1832.79538	-0.83315	0.08238464	-10.112928	4.84E-24
18347	PLEKHA6	947.691932	-0.1672678	0.07955912	-2.1024338	0.0355153
18348	PLEKHA7	681.271043	0.73320657	0.12616728	5.81138443	6.20E-09
18351	PLEKHB1	82.5526762	-1.7468908	0.24553005	-7.1147738	1.12E-12
18354	PLEKHF1	676.822262	0.5585091	0.09358048	5.96822192	2.40E-09
18355	PLEKHF2	1305.49472	0.5704481	0.08484839	6.72314584	1.78E-11
18359	PLEKHG4	1296.85241	-0.502291	0.07490335	-6.7058558	2.00E-11
18364	PLEKHH1	331.662164	-1.6340678	0.12654376	-12.913065	3.80E-38
18365	PLEKHH2	171.5821	-0.7002854	0.19125018	-3.6616196	0.00025063
18366	PLEKHH3	908.865681	-0.4499993	0.11049907	-4.0724261	4.65E-05
18370	PLEKHM2	4380.00746	0.4427189	0.06944724	6.37489551	1.83E-10
18371	PLEKHM3	249.780389	-0.6424316	0.16310316	-3.9388055	8.19E-05
18382	PLIN1	15.9866868	-1.7927673	0.52221601	-3.4329995	0.00059694
18383	PLIN2	4926.15173	1.21520363	0.07646195	15.8929189	7.09E-57
18384	PLIN3	6683.16968	-0.2563412	0.06380826	-4.0173662	5.89E-05
18387	PLK1	3243.60856	-1.0509109	0.06356074	-16.533962	2.09E-61
18392	PLLP	421.716298	-1.1917925	0.11126188	-10.711598	8.98E-27
18398	PLP2	17025.6576	0.13978913	0.06017008	2.32323335	0.02016662

18399	PLRG1	4627.58391	1.01491442	0.05647207	17.9719707	3.23E-72
18400	PLS1	1429.29793	0.28951221	0.09202218	3.14611348	0.00165456
18403	PLSCR1	1809.28131	0.28603136	0.06896542	4.14746034	3.36E-05
18410	PLXDC1	20.0250237	-1.1321913	0.47045521	-2.4065868	0.01610238
18411	PLXDC2	4.44876394	3.51000035	1.32033908	2.65840828	0.00785107
18412	PLXNA1	8124.15413	-0.2392589	0.07622065	-3.1390292	0.00169509
18416	PLXNB1	382.048039	-1.0378072	0.11873293	-8.7406855	2.32E-18
18417	PLXNB2	9466.06865	-1.028857	0.06656654	-15.456069	6.87E-54
18418	PLXNB3	1263.70271	-0.8847301	0.09039276	-9.7876215	1.27E-22
18420	PLXND1	1970.87221	-1.0967814	0.0760074	-14.429928	3.35E-47
18423	PMAIP1	11446.2622	1.22622332	0.06679186	18.3588744	2.80E-75
18428	PMEPA1	2592.46786	-0.2654757	0.06599568	-4.0226231	5.76E-05
18429	PMF1	605.200105	-0.5889993	0.13129434	-4.4860984	7.25E-06
18432	PML	3152.67246	-0.1926641	0.06760224	-2.8499663	0.00437239
18434	PMM2	1972.26172	0.52411388	0.06622391	7.91426936	2.49E-15
18438	PMPCB	3831.73653	-0.2392088	0.06255205	-3.8241561	0.00013122
18441	PMS2CL	232.250454	0.69363863	0.15598492	4.44683129	8.71E-06
18452	PNKD	1729.61718	-0.4686594	0.09873042	-4.7468588	2.07E-06
18453	PNKP	1361.5244	-0.49747	0.10932578	-4.550345	5.36E-06
18464	PNMAL1	1363.89018	-0.7387698	0.08107597	-9.1120692	8.08E-20
18468	PNO1	1521.01902	1.10498182	0.0953078	11.5938233	4.43E-31
18470	PNP	2756.57699	0.69816942	0.06526492	10.6974682	1.05E-26
18476	PNPLA6	7484.32631	-0.1512773	0.07496758	-2.0179028	0.04360138
18480	PNPT1	2077.01958	0.82804045	0.06921856	11.9626933	5.57E-33
18482	PNRC2	2197.97438	1.09890484	0.07453446	14.7435809	3.38E-49
18483	POC1A	1783.25848	-0.2519581	0.09273062	-2.7170968	0.00658573
18484	POC1B	1634.02554	0.60740396	0.09100564	6.67435482	2.48E-11
18486	POC5	564.607132	0.80036364	0.11422645	7.00681542	2.44E-12
18488	PODNL1	63.3029578	-1.8333401	0.27109022	-6.7628412	1.35E-11
18489	PODXL	20468.5461	-0.6655777	0.06702546	-9.9302227	3.08E-23
18490	PODXL2	421.557914	-0.6089654	0.11312006	-5.3833544	7.31E-08
18494	POGK	2588.8858	0.2014899	0.0951467	2.11767613	0.03420251
18498	POLA2	1455.42314	-0.4883942	0.07211781	-6.7721726	1.27E-11
18500	POLD1	3295.62636	-0.436684	0.07876472	-5.5441578	2.95E-08
18502	POLD3	1471.85161	-0.2260712	0.08558667	-2.6414301	0.00825568
18505	POLDIP3	3717.77558	0.57831728	0.06425693	9.00007697	2.26E-19
18506	POLE	3342.06895	-0.3474549	0.07412601	-4.6873547	2.77E-06
18507	POLE2	1001.86215	-0.3946199	0.08194958	-4.815399	1.47E-06
18508	POLE3	5060.17329	0.42521583	0.0612798	6.93892367	3.95E-12
18510	POLG	3197.59711	-0.7574261	0.06128042	-12.360003	4.30E-35
18511	POLG2	648.453494	0.3652736	0.12634264	2.8911349	0.00383853
18514	POLK	1498.00058	0.63162446	0.11355202	5.56242363	2.66E-08
18519	POLR1A	3149.87201	0.34450615	0.09900678	3.47962181	0.00050212

18520	POLR1B	2230.82509	0.61359148	0.07853261	7.8132066	5.58E-15
18521	POLR1C	1853.1707	0.49307894	0.10492596	4.69930362	2.61E-06
18523	POLR1E	1389.80758	0.31030376	0.0733942	4.22790595	2.36E-05
18527	POLR2D	1403.36032	0.22090702	0.08412052	2.62607771	0.00863751
18528	POLR2E	7295.4336	-0.3528767	0.07081477	-4.983095	6.26E-07
18529	POLR2F	1683.15123	-0.4127925	0.12485303	-3.3062276	0.00094561
18530	POLR2G	2079.25406	0.2675955	0.09812796	2.72700574	0.00639119
18532	POLR2I	788.879236	-0.5719837	0.11505003	-4.9716079	6.64E-07
18540	POLR3A	1596.90402	0.24223058	0.08130692	2.97921216	0.00288991
18542	POLR3C	1589.24027	0.54760896	0.07649514	7.15874142	8.14E-13
18543	POLR3D	1043.83101	1.60562197	0.08688567	18.4797105	3.01E-76
18544	POLR3E	1510.8833	0.80101821	0.0867886	9.22953268	2.72E-20
18545	POLR3F	737.350304	0.3985944	0.09821207	4.05850704	4.94E-05
18546	POLR3G	451.48483	0.87194379	0.11186302	7.79474592	6.45E-15
18548	POLR3H	2741.36382	0.78113927	0.07784646	10.0343578	1.08E-23
18550	POLRMT	1785.45837	0.32562173	0.0944779	3.44653843	0.00056782
18562	POMGNT2	1166.28398	-0.3641955	0.08332731	-4.3706617	1.24E-05
18565	POMT1	1925.55353	-0.4920225	0.0784941	-6.2682739	3.65E-10
18567	POMZP3	301.162528	-0.4631676	0.12905327	-3.5889647	0.00033199
18569	PON2	954.321353	-0.6322614	0.08487078	-7.4496941	9.36E-14
18573	POP5	358.891559	-0.7376789	0.14207889	-5.1920375	2.08E-07
18577	POR	2575.33711	-0.5207541	0.06166135	-8.4453893	3.03E-17
18580	POT1	1770.8661	0.40157189	0.07534469	5.32979684	9.83E-08
18599	POU2F1	1087.50433	0.42101493	0.09121577	4.6155939	3.92E-06
18621	PP7080	481.923477	-0.6921255	0.11765297	-5.8827712	4.03E-09
18623	PPA2	2436.06914	-0.2225584	0.10766426	-2.067152	0.03871983
18626	PPAP2A	1300.11382	-0.324844	0.08643666	-3.7581739	0.00017116
18629	PPAPDC1A	590.342124	-2.3432848	0.11165787	-20.986294	8.75E-98
18631	PPAPDC2	114.30217	-1.1718099	0.19281837	-6.0772734	1.22E-09
18638	PPAT	2376.21179	1.09617057	0.06655492	16.4701668	6.01E-61
18642	PPCS	1137.66881	0.30855976	0.08625866	3.57714538	0.00034737
18650	PPFIA4	942.706274	-0.8332087	0.08951786	-9.3077371	1.31E-20
18653	PPHLN1	1615.48355	-0.3486866	0.07579427	-4.600435	4.22E-06
18663	PPIC	1353.2427	-0.343188	0.10016379	-3.4262683	0.00061194
18665	PPIE	1723.06386	0.26650921	0.08042669	3.31369129	0.00092073
18668	PPIG	3001.09449	-0.1980824	0.07938294	-2.495277	0.01258588
18670	PPIL1	3284.53046	0.3391115	0.06964997	4.86879582	1.12E-06
18673	PPIL4	1608.4245	0.63133548	0.0822906	7.67202463	1.69E-14
18674	PPIL6	26.5696767	1.20327647	0.48091343	2.50206462	0.01234714
18683	PPM1G	8650.54058	0.19741862	0.07116812	2.7739754	0.00553759
18688	PPM1M	604.150197	-0.557889	0.09216721	-6.0530097	1.42E-09
18689	PPM1N	46.7832943	-0.7925148	0.28570365	-2.7739051	0.00553878
18692	PPP1CA	10536.9441	-0.3068271	0.09868591	-3.1091279	0.0018764

18693	PPP1CB	11476.5124	0.12737191	0.06198836	2.05477138	0.03990109
18694	PPP1CC	11071.8857	-0.1863383	0.05351436	-3.482024	0.00049764
18695	PPP1R10	4763.96132	0.75288697	0.0617796	12.1866601	3.66E-34
18696	PPP1R11	3071.25031	0.27934233	0.07107408	3.93029832	8.48E-05
18701	PPP1R13L	3432.32711	-0.234981	0.0760731	-3.0888838	0.0020091
18706	PPP1R15A	19109.2614	2.51006171	0.08358109	30.0314557	3.81E-198
18707	PPP1R15B	3396.26059	1.19632117	0.07884159	15.1737313	5.28E-52
18711	PPP1R18	10024.2421	0.67318272	0.05146952	13.0792506	4.33E-39
18715	PPP1R2	1832.27319	0.40119859	0.07621872	5.26378043	1.41E-07
18717	PPP1R26	2607.05788	0.34830867	0.06675161	5.21798145	1.81E-07
18729	PPP1R3D	502.065886	-0.5715075	0.10391707	-5.4996494	3.81E-08
18730	PPP1R3E	513.230625	-0.3585658	0.10997735	-3.26036	0.00111271
18732	PPP1R3G	49.5058837	-0.7615351	0.29248288	-2.6036911	0.00922258
18734	PPP1R7	1457.97744	0.36560136	0.08790974	4.1588264	3.20E-05
18735	PPP1R8	2083.80675	0.5024	0.0776212	6.47245891	9.64E-11
18737	PPP1R9B	5869.14781	0.75969431	0.07547354	10.0657041	7.83E-24
18738	PPP2CA	12150.2604	0.39217702	0.05315054	7.37860851	1.60E-13
18739	PPP2CB	4019.2031	0.26885925	0.06471278	4.15465454	3.26E-05
18740	PPP2R1A	11961.6155	-0.3057193	0.06837603	-4.4711474	7.78E-06
18742	PPP2R2A	3729.08165	0.80914416	0.07970672	10.1515174	3.26E-24
18745	PPP2R2C	290.004718	3.02573258	0.16404796	18.4441955	5.81E-76
18746	PPP2R2D	1601.71418	-0.8571939	0.08902148	-9.629068	6.03E-22
18750	PPP2R4	7211.15717	-0.3758116	0.07368199	-5.1004537	3.39E-07
18751	PPP2R5A	1119.84284	-0.3286137	0.09130188	-3.5992	0.0003192
18755	PPP2R5E	2509.95762	0.59876589	0.08908688	6.72114596	1.80E-11
18757	PPP3CB	3201.94495	-0.6241203	0.06230698	-10.016861	1.29E-23
18759	PPP3CC	626.11669	0.37855728	0.09318739	4.062323	4.86E-05
18760	PPP3R1	3692.38537	0.60922416	0.0618884	9.84391579	7.28E-23
18762	PPP4C	3607.70827	-0.5061266	0.07089074	-7.1395309	9.36E-13
18767	PPP4R3A	4309.09714	0.89434216	0.0634911	14.0861035	4.62E-45
18768	PPP4R3B	3154.48041	0.5033905	0.07309237	6.88704539	5.70E-12
18775	PPP6R2	1330.30514	-0.6576959	0.0796895	-8.2532319	1.54E-16
18776	PPP6R3	9281.62457	0.52792851	0.06310934	8.36529954	6.00E-17
18777	PPRC1	2670.38315	0.38282504	0.07754999	4.93649344	7.95E-07
18781	PPTC7	2424.13641	0.95693904	0.09582551	9.98626646	1.75E-23
18787	PQLC2	1186.32351	0.192071	0.0822746	2.33451156	0.01956895
18789	PQLC3	433.290488	-1.3992291	0.12172581	-11.494926	1.40E-30
18821	PRC1	7522.4199	-0.2896358	0.05914879	-4.896733	9.74E-07
18823	PRCAT47	58.1557499	-5.2225717	0.52473415	-9.9527955	2.45E-23
18826	PRCP	5428.95724	-0.3697709	0.05568407	-6.640516	3.13E-11
18835	PRDM2	861.565805	0.78636641	0.14028087	5.60565685	2.07E-08
18836	PRDM4	2877.28486	0.40000388	0.06686539	5.98222597	2.20E-09
18844	PRDX3	12087.2439	-0.5171139	0.0660411	-7.8301829	4.87E-15

18846	PRDX5	8030.41517	-0.3732129	0.10052285	-3.7127175	0.00020505
18847	PRDX6	10993.7027	0.35445711	0.06498988	5.45403585	4.92E-08
18851	PRELP	8.86388582	-6.6895734	1.32952442	-5.0315536	4.87E-07
18852	PREP	1697.39202	-0.4727445	0.07173601	-6.5900578	4.40E-11
18853	PREPL	1010.95389	0.33390851	0.10675926	3.12767733	0.00176193
18870	PRICKLE4	273.788777	-0.5421257	0.14967621	-3.6219895	0.00029235
18871	PRIM1	714.950694	-0.4284557	0.11142434	-3.8452618	0.00012042
18872	PRIM2	1263.81878	0.44491431	0.08040754	5.53324088	3.14E-08
18876	PRKAA2	1233.28082	0.92623073	0.08217501	11.2714403	1.82E-29
18878	PRKAB2	607.78942	0.33357111	0.10242365	3.25677835	0.00112684
18888	PRKAR2A	1134.97353	0.91696034	0.12343625	7.4286145	1.10E-13
18890	PRKAR2B	181.452374	-1.8522028	0.17815755	-10.396432	2.57E-25
18895	PRKCDBP	3266.32987	-1.0816893	0.14309864	-7.5590464	4.06E-14
18896	PRKCE	799.618233	0.50717099	0.09685702	5.23628536	1.64E-07
18897	PRKCG	23.128025	-1.5513028	0.47353523	-3.276003	0.00105287
18898	PRKCH	589.811227	0.48719704	0.09696135	5.02465196	5.04E-07
18902	PRKCSH	12478.1485	-0.4393285	0.05658424	-7.7641483	8.22E-15
18903	PRKCZ	622.372088	1.13181825	0.10126105	11.1772321	5.27E-29
18907	PRKDC	12915.6256	0.64994457	0.09060031	7.17375634	7.30E-13
18909	PRKG1-AS1	53.6936973	0.70257738	0.28159406	2.49500067	0.01259569
18921	PRLR	202.101433	-0.4287235	0.16156247	-2.6536082	0.00796362
18925	PRMT1	8787.81585	0.55358977	0.05974874	9.26529581	1.95E-20
18926	PRMT2	3641.288	-0.1858147	0.07509277	-2.474469	0.01334344
18927	PRMT3	2062.18036	1.16713279	0.06880871	16.961991	1.57E-64
18928	PRMT5	6034.96601	0.71447542	0.05496079	12.9997289	1.23E-38
18930	PRMT6	845.602837	-0.704181	0.09352748	-7.5291345	5.11E-14
18931	PRMT7	874.816464	-0.300058	0.09103862	-3.2959416	0.00098092
18941	PROCA1	19.5265617	-1.6524082	0.46424847	-3.5593187	0.00037182
18955	PROS1	1214.7012	-1.6458033	0.09226659	-17.837478	3.62E-71
18956	PROSC	1014.04988	0.91427564	0.08157799	11.2073817	3.75E-29
18957	PROSER1	1273.7208	0.31614727	0.12776713	2.47440226	0.01334593
18958	PROSER2	3175.84136	0.85175912	0.07802763	10.9161223	9.65E-28
18966	PRPF19	4644.39957	0.32191457	0.06802228	4.7324872	2.22E-06
18968	PRPF31	3908.77074	-0.4128799	0.07178421	-5.7516824	8.84E-09
18983	PRPSAP1	1748.11535	-0.5830011	0.07028964	-8.2942687	1.09E-16
18984	PRPSAP2	1150.27911	0.35337201	0.09775082	3.61502856	0.00030031
18986	PRR12	1967.7661	-0.3246517	0.07526033	-4.3137164	1.61E-05
18988	PRR14	1091.82084	-0.2648011	0.07843933	-3.3758709	0.00073582
18989	PRR14L	1264.22248	0.50096348	0.08827262	5.67518557	1.39E-08
18994	PRR19	210.859756	-0.3505763	0.14321195	-2.447954	0.014367
19012	PRR3	915.809729	0.73175102	0.08594583	8.51409601	1.68E-17
19022	PRR5L	718.776279	-0.3388214	0.09042717	-3.7468979	0.00017903
19028	PRRC2B	9684.32387	0.25211722	0.07963799	3.16579096	0.00154662

19030	PRRG1	1193.27461	0.61861956	0.10041435	6.16066888	7.24E-10
19035	PRRT2	20.8944533	-1.2233845	0.4983347	-2.4549455	0.01409059
19047	PRSS22	128.947155	-0.4213523	0.2148351	-1.9612826	0.04984607
19048	PRSS23	13500.914	-1.4636856	0.05611005	-26.08598	5.26E-150
19071	PRTFDC1	574.814147	-0.6685082	0.09726679	-6.8729339	6.29E-12
19072	PRTG	468.260425	-0.5780016	0.1196083	-4.8324542	1.35E-06
19074	PRUNE	593.790373	0.2589787	0.09908557	2.61368743	0.00895709
19079	PSAP	49091.4121	-0.4390863	0.06687388	-6.5658858	5.17E-11
19081	PSAT1	8538.97765	2.46241275	0.05744234	42.8675538	0
19082	PSCA	18.6615524	1.53912405	0.53765415	2.86266562	0.00420094
19090	PSG1	759.396133	2.03092099	0.11936998	17.0136656	6.50E-65
19091	PSG10P	13.2347588	1.26220839	0.56490499	2.23437289	0.02545855
19092	PSG11	113.985567	3.30986806	0.25136248	13.1677093	1.35E-39
19093	PSG2	48.640357	2.9204657	0.37131428	7.86521246	3.68E-15
19095	PSG4	463.756967	0.99206378	0.12485908	7.94546762	1.93E-15
19096	PSG5	1494.85056	2.51786587	0.08909082	28.261789	1.02E-175
19097	PSG6	182.473325	4.41204707	0.2843005	15.5189562	2.58E-54
19098	PSG7	125.316558	2.40330633	0.25161551	9.55150316	1.28E-21
19099	PSG8	89.7162486	1.82648299	0.23530214	7.76228813	8.34E-15
19100	PSG9	436.760156	1.93940901	0.13495285	14.3710123	7.87E-47
19101	PSIP1	1890.6163	-0.633569	0.07099677	-8.923913	4.50E-19
19102	PSKH1	1344.04146	-1.1554536	0.08550317	-13.513576	1.30E-41
19104	PSMA1	5793.20908	-0.3842378	0.06418997	-5.9859469	2.15E-09
19105	PSMA2	6058.80499	-0.1665927	0.07427384	-2.2429521	0.02489991
19108	PSMA4	4979.92504	-0.3616335	0.06178228	-5.8533532	4.82E-09
19111	PSMA7	15052.1592	0.49271187	0.09958452	4.94767554	7.51E-07
19116	PSMB2	6076.39232	0.49313	0.05954678	8.28138795	1.22E-16
19128	PSMC3IP	1330.56639	0.67373847	0.0838781	8.03235281	9.56E-16
19129	PSMC4	4636.30616	0.35068807	0.05970308	5.87386948	4.26E-09
19130	PSMC5	5759.03949	0.28550817	0.06723466	4.24644345	2.17E-05
19132	PSMD1	6458.82637	-0.2329156	0.05607523	-4.1536281	3.27E-05
19134	PSMD11	6742.14104	0.35897136	0.06593093	5.44465781	5.19E-08
19135	PSMD12	5163.65776	0.88807991	0.06327287	14.0357132	9.42E-45
19137	PSMD14	7781.61771	0.58593841	0.05797999	10.1058726	5.20E-24
19138	PSMD2	23661.6817	0.12299901	0.04869385	2.52596626	0.01153806
19139	PSMD3	5316.70487	-0.5827017	0.06741913	-8.6429738	5.48E-18
19145	PSMD7	4539.2136	0.24209648	0.05847706	4.14002463	3.47E-05
19150	PSME3	8715.26284	0.26395638	0.05214113	5.06234474	4.14E-07
19151	PSME4	4026.25689	0.65946733	0.07184204	9.17940653	4.33E-20
19152	PSMF1	3606.96637	-0.552727	0.05731807	-9.6431551	5.25E-22
19153	PSMG1	2470.37842	0.52102238	0.0767706	6.78674356	1.15E-11
19157	PSMG4	1078.18499	0.45938851	0.11904364	3.85899251	0.00011386
19158	PSORS1C1	245.737506	0.88795961	0.1452005	6.11540329	9.63E-10

19162	PSPH	1613.69821	0.72224824	0.0761504	9.4844973	2.44E-21
19164	PSRC1	1260.33085	-1.1799687	0.07827149	-15.075331	2.35E-51
19170	PTBP1	15115.5469	0.30803754	0.07307585	4.21531231	2.49E-05
19185	PTDSS1	6842.25832	0.43044412	0.05413463	7.95136363	1.84E-15
19187	PTEN	2897.9047	-0.6370024	0.06483183	-9.8254588	8.75E-23
19200	PTGES	3045.39952	-1.2711961	0.0801995	-15.850424	1.40E-56
19201	PTGES2	4296.33559	-0.2913772	0.09213879	-3.1623728	0.00156489
19220	PTK2B	1304.62723	-0.6233914	0.08239958	-7.5654682	3.86E-14
19224	PTMS	5408.36702	-0.5400598	0.11308697	-4.775615	1.79E-06
19225	PTN	144.404704	-0.6633268	0.18936165	-3.5029628	0.00046011
19226	PTOV1	4260.35678	-0.4642909	0.09945968	-4.6681322	3.04E-06
19229	PTP4A1	9963.24266	0.43541178	0.06402767	6.80036893	1.04E-11
19230	PTP4A2	14267.4382	0.78615797	0.06150649	12.7817082	2.07E-37
19236	PTPN12	11833.6754	0.58658236	0.0627135	9.35336652	8.49E-21
19240	PTPN2	1592.38194	0.34394631	0.08411627	4.088939	4.33E-05
19245	PTPN3	1213.45965	-0.3805917	0.07830776	-4.8602036	1.17E-06
19248	PTPN6	172.910658	-1.1872911	0.18359105	-6.4670423	9.99E-11
19250	PTPN9	1705.0177	-0.3947426	0.06980392	-5.6550207	1.56E-08
19251	PTPRA	3324.03604	-1.0080334	0.08131542	-12.396584	2.73E-35
19254	PTPRCAP	62.5478431	-2.1374874	0.33628485	-6.3561812	2.07E-10
19258	PTPRE	2598.77155	-0.9413739	0.08373645	-11.242104	2.53E-29
19259	PTPRF	17792.4676	0.48057011	0.08259869	5.81813216	5.95E-09
19263	PTPRJ	6253.43982	-0.422322	0.08940834	-4.7235189	2.32E-06
19264	PTPRK	5590.89397	0.47879573	0.07023177	6.81736721	9.27E-12
19270	PTPRR	296.386241	0.47721408	0.12611555	3.78394327	0.00015436
19271	PTPRS	2639.04722	-0.3951114	0.07373884	-5.3582533	8.40E-08
19276	PTRF	28446.1122	-0.4556336	0.05759898	-7.9104462	2.56E-15
19278	PTRH2	1635.39088	0.42151936	0.08726221	4.83049144	1.36E-06
19288	PUM1	5347.41536	0.83497029	0.0751135	11.1161141	1.05E-28
19289	PUM2	5418.33027	0.37586854	0.0760482	4.94250429	7.71E-07
19290	PURA	1862.11236	0.5031906	0.06946998	7.24328122	4.38E-13
19293	PUS1	974.064094	0.51776531	0.12352854	4.19146292	2.77E-05
19295	PUS3	545.526234	0.87795379	0.10740401	8.17431125	2.98E-16
19296	PUS7	2138.25809	0.97515564	0.07308609	13.3425616	1.31E-40
19297	PUS7L	711.277729	0.94546047	0.11377379	8.31000269	9.57E-17
19298	PUSL1	1297.96917	1.26037481	0.12101804	10.4147684	2.12E-25
19300	PVR	10377.6397	1.23239819	0.06007025	20.5159498	1.55E-93
19303	PVRL1	450.688023	-1.0113798	0.10374938	-9.7482969	1.88E-22
19308	PVT1	960.341769	1.11419838	0.09148882	12.1785194	4.05E-34
19313	PWP1	3803.66416	0.82890542	0.06098901	13.5910616	4.52E-42
19314	PWP2	169.900371	0.44840257	0.18036634	2.48606567	0.01291641
19321	PXDC1	3534.42866	0.36172507	0.0608559	5.9439608	2.78E-09
19326	PXMP4	464.577063	-0.8877931	0.11398058	-7.7889858	6.75E-15

19327	PXN	18378.4901	0.19393875	0.05440408	3.56478315	0.00036416
19330	PXYLP1	130.969039	-0.4392395	0.18398106	-2.3874168	0.01696724
19331	PYCARD	315.759008	-0.9986297	0.19730098	-5.0614532	4.16E-07
19333	PYCR1	5673.18685	1.12359746	0.06010131	18.6950575	5.43E-78
19338	PYGB	16614.5839	0.7304366	0.0605618	12.0610116	1.70E-33
19339	PYGL	8390.15636	1.27409711	0.05328822	23.9095463	2.44E-126
19341	PYGO1	1021.69525	0.44088697	0.10695386	4.12221672	3.75E-05
19344	PYROXD1	229.921446	0.55820775	0.14366604	3.88545371	0.00010214
19345	PYROXD2	393.813623	-1.4635267	0.11702224	-12.506398	6.89E-36
19350	QARS	5544.79502	-0.9126419	0.06316167	-14.449298	2.53E-47
19352	QKI	5170.80827	0.45575231	0.07298141	6.24477298	4.24E-10
19360	QRSL1	623.04296	0.20216758	0.09368478	2.15795533	0.03093131
19361	QSER1	3594.88926	0.82767108	0.10796529	7.66608473	1.77E-14
19362	QSOX1	17975.2786	0.24613082	0.09263688	2.65694203	0.0078853
19363	QSOX2	7252.34083	0.53302891	0.06614524	8.05846204	7.73E-16
19365	QTRTD1	1110.5329	0.89765461	0.08224802	10.913997	9.88E-28
19366	R3HCC1	868.928339	-0.3827613	0.09882025	-3.8733088	0.00010737
19367	R3HCC1L	836.113579	0.54983438	0.08812	6.2396096	4.39E-10
19372	RAB10	7025.50771	0.18768089	0.05598419	3.3523908	0.00080117
19373	RAB11A	4753.15277	-0.4747073	0.06463187	-7.3447874	2.06E-13
19377	RAB11FIP2	1440.3374	0.36355998	0.09228202	3.93966227	8.16E-05
19378	RAB11FIP3	1299.22802	-0.3608936	0.0827797	-4.359688	1.30E-05
19384	RAB15	388.816205	0.42636751	0.11693085	3.6463217	0.00026602
19389	RAB1B	11780.4183	-0.2954245	0.05576241	-5.2979144	1.17E-07
19390	RAB20	411.512408	-0.6335119	0.11109969	-5.7021934	1.18E-08
19391	RAB21	2090.16971	0.49627178	0.07157267	6.9338164	4.10E-12
19392	RAB22A	4531.61212	-0.2551102	0.05626248	-4.5342869	5.78E-06
19396	RAB26	66.3777324	-1.7742862	0.26894449	-6.5972209	4.19E-11
19397	RAB27A	1236.41334	-0.8705178	0.09285161	-9.3753664	6.89E-21
19398	RAB27B	951.383692	-1.339145	0.11564176	-11.580116	5.20E-31
19399	RAB28	1916.35257	0.89770656	0.08006045	11.2128599	3.53E-29
19400	RAB29	2986.85365	0.43426803	0.06165861	7.04310451	1.88E-12
19404	RAB30-AS1	474.840142	0.27713146	0.11994296	2.31052719	0.02085899
19405	RAB31	4097.53861	-0.5561129	0.06115104	-9.0940873	9.54E-20
19408	RAB33B	475.931905	0.3697182	0.1256184	2.94318494	0.00324854
19410	RAB35	2800.72878	0.17393025	0.08858502	1.96342727	0.04959655
19413	RAB38	267.273048	-1.3277521	0.15742803	-8.4340261	3.34E-17
19416	RAB3A	119.52143	-1.0825168	0.20646431	-5.2431184	1.58E-07
19419	RAB3D	949.628119	-0.4556183	0.08842398	-5.1526561	2.57E-07
19427	RAB40C	613.067655	-0.3861305	0.11264651	-3.4278073	0.00060848
19435	RAB5A	4882.8146	0.39194231	0.08201538	4.77888777	1.76E-06
19436	RAB5B	3682.40163	-0.5237068	0.07358241	-7.1172829	1.10E-12
19443	RAB7B	20.1274299	-2.1586944	0.47660788	-4.5292881	5.92E-06

19445	RAB8B	2223.97708	0.35357972	0.09095439	3.88743988	0.00010131
19446	RAB9A	2172.30208	0.1924897	0.08318267	2.31405997	0.02066443
19450	RABEP1	4500.81461	0.37856427	0.06341584	5.96955377	2.38E-09
19451	RABEP2	341.746386	-1.2746814	0.15002385	-8.496525	1.95E-17
19452	RABEPK	1110.06516	1.14056393	0.08605295	13.2542113	4.27E-40
19455	RABGEF1	4142.38974	0.62507879	0.06209449	10.0665734	7.76E-24
19456	RABGGTA	810.098712	-0.9130708	0.11319637	-8.0662545	7.25E-16
19457	RABGGTB	3895.15932	1.37116673	0.07858776	17.447587	3.59E-68
19458	RABIF	1353.44622	0.40436327	0.0775645	5.21325192	1.86E-07
19464	RAC2	9391.27291	-0.9154846	0.05904112	-15.505881	3.17E-54
19466	RACGAP1	4728.67054	-0.1632531	0.05575799	-2.9278875	0.00341274
19468	RAD1	2627.80911	0.58383446	0.06720994	8.68672814	3.73E-18
19470	RAD18	2434.25122	0.65779862	0.09238782	7.11997093	1.08E-12
19474	RAD23A	8204.19159	0.24026346	0.05396919	4.45186314	8.51E-06
19475	RAD23B	13685.8739	0.62813858	0.06111528	10.2779307	8.86E-25
19476	RAD50	5194.64435	0.63292604	0.0812206	7.79267904	6.56E-15
19478	RAD51-AS1	43.505538	-1.1785327	0.32508725	-3.6252813	0.00028865
19488	RAD54L2	462.417072	0.69338112	0.14127376	4.90806738	9.20E-07
19490	RAD9B	35.7295834	1.28061706	0.34166549	3.74816039	0.00017814
19492	RAE1	2898.36324	0.83201375	0.06651185	12.5092554	6.64E-36
19495	RAET1G	140.641925	-2.0470788	0.20900014	-9.7946288	1.19E-22
19503	RAI14	6379.36231	0.3427255	0.08074595	4.24449173	2.19E-05
19505	RALA	4421.65406	0.49740167	0.06809808	7.30419532	2.79E-13
19512	RALGDS	1964.87852	-0.6251354	0.06593558	-9.4810023	2.52E-21
19515	RALY	6138.19632	-0.3968709	0.06149932	-6.4532571	1.09E-10
19518	RAMP1	1201.42613	-1.0926899	0.10808605	-10.109445	5.02E-24
19525	RANBP17	215.326625	-0.3842023	0.15369061	-2.4998426	0.01242485
19531	RANGAP1	12259.6578	0.27390083	0.09311142	2.94164595	0.00326473
19534	RAP1B	1252.99353	0.38097931	0.0734855	5.18441441	2.17E-07
19535	RAP1GAP	135.717524	0.72104679	0.198796	3.62706894	0.00028666
19538	RAP2A	2173.92161	0.80158423	0.07334138	10.9294949	8.33E-28
19539	RAP2B	3147.71023	0.62414204	0.07264652	8.59149282	8.58E-18
19540	RAP2C	2823.79733	0.5925083	0.06832985	8.67129461	4.27E-18
19543	RAPGEF2	2194.75507	0.59508087	0.11101582	5.36032476	8.31E-08
19545	RAPGEF4	57.964199	-0.6204391	0.26269912	-2.361786	0.01818714
19550	RAPH1	4761.97394	0.38108196	0.09665886	3.94254573	8.06E-05
19552	RARA	1442.0015	0.39559737	0.0878039	4.50546483	6.62E-06
19557	RARRES2	3859.14492	-1.615445	0.12361942	-13.06789	5.02E-39
19558	RARRES3	1725.96458	-0.8660412	0.1308873	-6.6166942	3.67E-11
19563	RASA3	2745.16048	0.21836591	0.06812202	3.20551149	0.00134823
19568	RASAL2	3379.22433	0.56395213	0.10802862	5.22039549	1.79E-07
19572	RASD2	208.17909	-1.3251306	0.14922752	-8.8799343	6.69E-19
19573	RASEF	31.3801648	-1.9570835	0.38529742	-5.0794097	3.79E-07

19582	RASGRP3	348.477258	0.40083244	0.13491106	2.97108656	0.00296748
19587	RASL11A	59.3596964	-1.1796548	0.28259657	-4.174342	2.99E-05
19590	RASSF1	2996.35268	1.22851789	0.10115436	12.1449822	6.10E-34
19593	RASSF2	120.409786	-1.7354795	0.20105782	-8.631743	6.04E-18
19594	RASSF3	2942.06502	-0.4534777	0.06614092	-6.8562342	7.07E-12
19595	RASSF4	683.26756	-2.1053426	0.10014832	-21.022246	4.11E-98
19599	RASSF8	562.649327	0.28036197	0.11112962	2.52283737	0.01164122
19615	RBBP8	3616.97788	0.33261566	0.07523306	4.42113716	9.82E-06
19617	RBBP9	1100.3466	-0.5753359	0.10462274	-5.499148	3.82E-08
19618	RBCK1	3994.26615	0.5677625	0.07226061	7.857151	3.93E-15
19626	RBL2	1377.3083	-0.7709104	0.10082597	-7.6459506	2.07E-14
19637	RBM18	1595.09595	0.72595165	0.07153187	10.1486465	3.36E-24
19639	RBM20	32.0925006	1.73672864	0.43775566	3.96734705	7.27E-05
19640	RBM22	2195.79077	0.58569419	0.07419072	7.89443951	2.92E-15
19641	RBM23	3330.96546	-0.1851925	0.06062846	-3.054548	0.002254
19642	RBM24	523.68624	0.84954037	0.10539375	8.06063323	7.59E-16
19645	RBM26-AS1	87.19818	0.85749443	0.22502453	3.81067099	0.00013859
19646	RBM27	1913.88913	0.3406821	0.11197966	3.04235693	0.00234733
19647	RBM28	1495.03966	0.48291169	0.07306328	6.60949945	3.86E-11
19650	RBM34	2427.08237	0.3722127	0.0760778	4.89252678	9.95E-07
19651	RBM38	1114.373	0.86367521	0.0805295	10.7249537	7.77E-27
19652	RBM39	15285.5237	0.57250821	0.05321555	10.7582886	5.42E-27
19658	RBM45	389.875177	-0.6026877	0.1169877	-5.1517182	2.58E-07
19662	RBM4B	396.686804	-0.8288235	0.11315557	-7.3246373	2.40E-13
19663	RBM5	4290.14996	0.23821704	0.05934195	4.01431081	5.96E-05
19665	RBM6	3075.43522	-0.3207072	0.06181204	-5.1884262	2.12E-07
19675	RBMXL1	1047.65867	-0.217637	0.07997223	-2.7214074	0.00650046
19693	RBP7	15.7177925	-1.1735425	0.50853985	-2.3076708	0.02101746
19696	RBPMS	441.07996	2.20402529	0.12535234	17.5826423	3.35E-69
19697	RBPMS-AS1	17.0528311	2.35297828	0.62114361	3.78813889	0.00015178
19698	RBPMS2	267.000859	-0.3304116	0.14393674	-2.2955334	0.02170257
19700	RBX1	2030.76992	-0.2381777	0.10417543	-2.2863135	0.02223592
19701	RC3H1	1195.79719	0.32763027	0.09277281	3.53153341	0.00041316
19703	RCAN1	2647.25379	0.78159795	0.06659334	11.7368787	8.25E-32
19705	RCAN3	528.757628	-0.3062217	0.10310785	-2.9699161	0.00297881
19708	RCBTB2	83.0778872	-1.3912682	0.25329314	-5.4927196	3.96E-08
19709	RCC1	5613.95021	1.26252727	0.05936327	21.267818	2.26E-100
19710	RCC2	5427.30565	0.82077249	0.06802372	12.065974	1.60E-33
19711	RCCD1	1604.40002	-0.9968608	0.09750473	-10.223717	1.55E-24
19714	RCL1	426.166001	1.0929944	0.11547742	9.46500524	2.94E-21
19717	RCN3	462.425263	-0.5010747	0.12939511	-3.8724394	0.00010775
19721	RCSD1	811.278474	-1.2362847	0.08640683	-14.307721	1.96E-46
19738	RECK	656.614746	-0.607567	0.09472057	-6.4143094	1.41E-10

19740	RECQL4	4459.86119	-0.1746085	0.08407082	-2.0769211	0.03780884
19743	REEP2	449.022221	-0.9503937	0.14128926	-6.7265811	1.74E-11
19744	REEP3	4365.88327	-0.4827105	0.08149893	-5.9229057	3.16E-09
19745	REEP4	1651.30342	-0.4216759	0.09581554	-4.4009136	1.08E-05
19747	REEP6	347.596887	-1.1159631	0.1277234	-8.7373428	2.39E-18
19754	REL	200.445113	0.66754947	0.20607374	3.2393718	0.00119793
19757	RELL1	768.381238	-0.7729203	0.09492376	-8.1425386	3.87E-16
19759	RELN	212.138267	2.49353606	0.18009441	13.8457162	1.35E-43
19766	REPIN1	12740.1489	-0.3565612	0.0739066	-4.8244838	1.40E-06
19767	REPS1	1623.83311	0.25374791	0.07317168	3.46784306	0.00052465
19776	REST	1670.8711	0.59263206	0.09763484	6.06988319	1.28E-09
19782	REV3L	1248.31219	0.3317305	0.11047323	3.00281355	0.00267496
19783	REXO1	2628.64699	0.50232038	0.070341	7.14121788	9.25E-13
19786	REXO4	2155.88714	0.39643505	0.07233641	5.48043596	4.24E-08
19787	RFC1	5242.62677	0.8377701	0.06216516	13.4765203	2.15E-41
19791	RFC5	1564.68715	-0.2408696	0.0806238	-2.9875746	0.00281201
19792	RFESD	304.395224	1.10233718	0.12814135	8.60250956	7.80E-18
19793	RFFL	185.47519	0.58692846	0.16712806	3.51184874	0.000445
19794	RFK	3731.26228	0.9463308	0.06478228	14.6078657	2.50E-48
19795	RFNG	1995.3572	0.55212269	0.07191416	7.67752422	1.62E-14
19804	RFT1	731.598072	-0.3432572	0.09403057	-3.6504846	0.00026175
19805	RFTN1	3216.6905	0.80962944	0.06639169	12.1947405	3.32E-34
19808	RFWD3	2709.40474	0.24066806	0.07135563	3.37279718	0.00074409
19809	RFX1	871.360196	-0.2166354	0.08730089	-2.4814799	0.01308381
19819	RFXAP	217.609212	0.36219987	0.1686171	2.14806131	0.03170889
19823	RGL1	776.066349	0.73529952	0.09177977	8.01156439	1.13E-15
19825	RGL3	198.475373	-0.370673	0.15086039	-2.4570596	0.01400795
19828	RGMB	2190.7771	0.75100923	0.07411245	10.1333753	3.93E-24
19830	RGN	66.5499775	-1.3188362	0.27207496	-4.8473267	1.25E-06
19844	RGS12	950.467626	-0.5886657	0.08256362	-7.1298428	1.00E-12
19846	RGS14	144.517184	-1.7028218	0.22878309	-7.4429534	9.85E-14
19847	RGS16	72.5525528	5.65266951	0.5566025	10.1556667	3.13E-24
19850	RGS19	1067.80702	-0.20313	0.09551547	-2.1266713	0.0334474
19856	RGS4	397.543051	-0.8225072	0.11129968	-7.390023	1.47E-13
19857	RGS5	181.401889	0.61681829	0.15444318	3.99382028	6.50E-05
19862	RGS9	107.9136	-1.6409484	0.20700804	-7.9269791	2.25E-15
19867	RHBDD2	2323.16873	-0.4689176	0.07233843	-6.4822756	9.03E-11
19869	RHBDF1	1617.62376	-0.5135726	0.0682138	-7.5288664	5.12E-14
19872	RHBDL2	426.054077	0.38992877	0.11909146	3.27419581	0.00105963
19885	RHOBTB2	485.17092	-0.784657	0.10943753	-7.1699083	7.50E-13
19890	RHOG	1947.66686	-0.3287798	0.08018742	-4.1001417	4.13E-05
19892	RHOJ	53.1915169	-1.4475533	0.28185452	-5.1358171	2.81E-07
19896	RHOU	706.915134	-0.4355804	0.08905278	-4.8912612	1.00E-06

19897	RHOV	34.306079	-1.8230762	0.3860929	-4.7218589	2.34E-06
19903	RHPN1	798.167815	-0.5412386	0.09992499	-5.4164489	6.08E-08
19907	RIBC2	51.4978622	-1.1608997	0.31734014	-3.6582188	0.00025397
19910	RIC8A	9595.81589	0.35147583	0.0648998	5.4156689	6.11E-08
19911	RIC8B	772.763043	-0.2605865	0.11124174	-2.3425245	0.01915378
19912	RICTOR	3269.37093	0.57444407	0.08813021	6.51812924	7.12E-11
19915	RILP	562.06395	-0.6400616	0.11773193	-5.4366015	5.43E-08
19917	RILPL2	1236.36138	0.45487267	0.09854447	4.61591269	3.91E-06
19919	RIMBP3	6.97037081	-3.2969205	1.05527657	-3.1242241	0.00178275
19922	RIMKLA	23.2860423	-2.750798	0.50332079	-5.4652977	4.62E-08
19925	RIMS2	305.652976	0.43951915	0.13082838	3.35950927	0.00078081
19928	RIN1	1237.50322	-0.7605498	0.08737996	-8.7039388	3.21E-18
19929	RIN2	2573.16848	-0.6904579	0.06357056	-10.861283	1.76E-27
19930	RIN3	2321.59731	0.47071902	0.06995611	6.72877671	1.71E-11
19931	RING1	2330.01238	0.25666431	0.07333266	3.50000007	0.00046526
19934	RIOK1	1266.47641	0.50326166	0.07725898	6.51395685	7.32E-11
19935	RIOK2	1236.65033	0.48290879	0.0844478	5.71842934	1.08E-08
19936	RIOK3	5538.61954	0.85400218	0.06807371	12.5452565	4.22E-36
19937	RIPK1	2627.23525	0.57880516	0.06557226	8.82698238	1.08E-18
19944	RIT1	2192.20001	0.95576393	0.06402686	14.9275466	2.18E-50
19946	RITA1	2376.20436	-0.1688565	0.06912214	-2.4428707	0.01457096
19948	RLF	2048.12197	1.10813795	0.07914378	14.0015804	1.52E-44
19954	RMDN1	1357.54978	-0.6439206	0.07324744	-8.7910319	1.48E-18
19955	RMDN2	110.853167	-1.3829533	0.20303197	-6.8115051	9.66E-12
19957	RMDN3	1298.99581	-0.5200779	0.10305985	-5.0463682	4.50E-07
19958	RMI1	1632.27682	0.33042715	0.08090267	4.08425499	4.42E-05
19959	RMI2	718.369293	-0.6732948	0.09675345	-6.9588713	3.43E-12
19989	RNASE1	23.2986211	-4.8981105	0.75712005	-6.4693975	9.84E-11
19996	RNASE4	384.532568	-1.2050865	0.13298683	-9.0616983	1.28E-19
20002	RNASEH1-AS	432.725557	0.87018702	0.11488699	7.57428659	3.61E-14
20006	RNASEH2C	3608.00655	-0.4123491	0.08987333	-4.5881139	4.47E-06
20010	RNASET2	1989.87781	-1.1778313	0.08688999	-13.555432	7.36E-42
20013	RND3	6465.13661	0.86929123	0.06398961	13.5848815	4.92E-42
20015	RNF103	1949.18359	-0.1988067	0.06651782	-2.9887739	0.00280099
20017	RNF11	3927.75961	0.49211317	0.0646721	7.60935808	2.75E-14
20018	RNF111	1310.16686	0.34548783	0.11051542	3.12615059	0.00177111
20026	RNF123	1655.66845	-0.5198004	0.07763366	-6.6955541	2.15E-11
20028	RNF126	3407.78575	0.3225388	0.07894612	4.08555595	4.40E-05
20032	RNF130	1713.24248	-0.9420603	0.07696298	-12.240433	1.89E-34
20034	RNF135	995.259282	-0.8534784	0.09425834	-9.0546729	1.37E-19
20035	RNF138	1730.35665	0.41361052	0.08210928	5.03731776	4.72E-07
20041	RNF144A	118.509348	-2.068814	0.21456634	-9.6418384	5.32E-22
20043	RNF144B	450.513714	0.31646463	0.11222903	2.81981086	0.0048052

20044	RNF145	5507.05236	-0.6623289	0.05695038	-11.629929	2.90E-31
20047	RNF149	3841.08597	0.16800683	0.0799788	2.10064201	0.0356724
20053	RNF165	32.1458939	-0.8836984	0.39375804	-2.2442677	0.02481518
20055	RNF167	2330.39117	0.36476543	0.06754969	5.3999568	6.67E-08
20057	RNF169	1516.1868	0.7218286	0.07729038	9.33917739	9.71E-21
20059	RNF170	829.521453	-0.4362901	0.08317255	-5.2456019	1.56E-07
20063	RNF182	659.391334	1.05993309	0.10335369	10.2553963	1.12E-24
20067	RNF187	4845.17337	-0.4304071	0.09714836	-4.4304102	9.41E-06
20072	RNF207	690.799713	-0.2911368	0.10449962	-2.7860085	0.00533615
20073	RNF208	339.169255	-0.2702641	0.12472057	-2.1669573	0.03023811
20081	RNF216P1	664.604058	0.50813465	0.10335288	4.91650197	8.81E-07
20082	RNF217	981.031074	0.46767634	0.08609853	5.43187395	5.58E-08
20084	RNF219	826.885452	0.66965862	0.10026517	6.67887581	2.41E-11
20088	RNF223	10.5950555	2.33178912	0.69406657	3.3596044	0.00078054
20093	RNF26	3871.04884	-0.8583093	0.06032719	-14.227571	6.18E-46
20097	RNF38	1489.94268	0.30530709	0.07994042	3.81918294	0.00013389
20101	RNF41	1155.78979	0.19401173	0.08476978	2.28868985	0.02209738
20106	RNF6	2687.94258	0.81372369	0.08137786	9.99932565	1.53E-23
20108	RNF8	3462.20143	0.5278357	0.07496246	7.04133333	1.90E-12
20110	RNFT2	119.096045	-1.1790349	0.24982936	-4.7193609	2.37E-06
20115	RNMTL1	1607.65693	0.52877563	0.09836288	5.37576392	7.63E-08
20116	RNPC3	391.958425	-0.881217	0.11385501	-7.7398176	9.96E-15
20117	RNPEP	3510.60993	-0.4641003	0.0669435	-6.9327163	4.13E-12
20118	RNPEPL1	1909.67372	-0.5702364	0.07410927	-7.6945352	1.42E-14
20119	RNPS1	2975.2158	-0.3685413	0.06184231	-5.9593715	2.53E-09
20129	RNU11	10.782458	2.53041905	0.69772538	3.62666906	0.0002871
20211	ROCK1	4033.61995	0.59399926	0.09029337	6.57854762	4.75E-11
20213	ROCK2	2401.71811	0.47023332	0.13925094	3.37687729	0.00073314
20214	ROGDI	203.635085	-1.1342987	0.15602276	-7.2700851	3.59E-13
20221	ROR1	2820.41374	0.83204473	0.08600835	9.67399991	3.89E-22
20229	RORC	15.6626243	-2.6835165	0.69289486	-3.8729058	0.00010755
20233	RP1L1	44.832262	-0.7293907	0.29266939	-2.4922001	0.01269545
20235	RP9	414.781308	0.57420153	0.12120381	4.73748726	2.16E-06
20238	RPA2	2876.63842	0.73981363	0.06654141	11.1180944	1.02E-28
20239	RPA3	1387.96795	-0.2812013	0.12474619	-2.2541874	0.02418438
20240	RPA4	29.9221865	3.26278674	0.47580791	6.85736131	7.01E-12
20241	RPAIN	1938.1543	0.49491617	0.0851521	5.81214269	6.17E-09
20243	RPAP2	1164.51864	1.25681194	0.07954892	15.7992334	3.15E-56
20244	RPAP3	1921.58544	0.57076187	0.06885356	8.28950408	1.14E-16
20250	RPF2	2088.75813	1.02457031	0.07502485	13.6564136	1.85E-42
20251	RPGR	618.040826	0.4902419	0.12266438	3.99661157	6.43E-05
20256	RPIA	2114.27376	1.09277292	0.12126582	9.01138417	2.03E-19
20257	RPL10	8624.18739	0.6923721	0.0658058	10.5214456	6.88E-26

20258	RPL10A	12187.0565	0.62877426	0.08223808	7.64577965	2.08E-14
20260	RPL11	15779.0842	0.33476856	0.0809235	4.13685224	3.52E-05
20261	RPL12	10241.8725	0.41185083	0.06621377	6.22001781	4.97E-10
20271	RPL15	14224.1697	-0.2515303	0.05772221	-4.3576007	1.31E-05
20282	RPL22L1	1955.124	0.47393993	0.08186635	5.78919098	7.07E-09
20286	RPL23AP53	273.063119	1.0756699	0.14117658	7.61932256	2.55E-14
20288	RPL23AP7	180.763912	0.41258988	0.15445888	2.67119568	0.00755816
20292	RPL24	14498.0152	0.4866945	0.09125406	5.33340101	9.64E-08
20293	RPL26	14587.6722	0.86276706	0.10219616	8.44226481	3.11E-17
20294	RPL26L1	657.104057	0.21900835	0.09978013	2.19490937	0.0281701
20295	RPL27	13726.4009	0.39753942	0.08824808	4.50479388	6.64E-06
20298	RPL29	8540.30687	-0.4456496	0.08583994	-5.1916339	2.08E-07
20301	RPL30	19459.8394	0.33241006	0.06884147	4.82863131	1.37E-06
20314	RPL37	20527.7935	0.72721291	0.10320681	7.04617149	1.84E-12
20320	RPL4	12837.4818	-0.4013083	0.08207901	-4.8892926	1.01E-06
20321	RPL41	128.723237	0.50676137	0.18671211	2.71413235	0.00664496
20322	RPL5	23350.2433	0.178448	0.06272045	2.84513242	0.0044393
20326	RPL7L1	3452.58494	1.30887461	0.08238008	15.8882425	7.64E-57
20327	RPL8	53898.0226	0.79017259	0.08987736	8.79167592	1.47E-18
20335	RPP14	1331.99903	0.53865874	0.07480196	7.20113145	5.97E-13
20337	RPP25	1567.77901	0.27020559	0.08799895	3.07055463	0.00213662
20341	RPP40	544.781982	1.10801526	0.12025261	9.21406415	3.14E-20
20343	RPRD1A	3235.55889	0.36294739	0.08100897	4.48033559	7.45E-06
20366	RPS2	10976.2441	0.60977608	0.10137198	6.01523317	1.80E-09
20368	RPS21	9581.11282	0.31645326	0.12048132	2.62657529	0.00862489
20369	RPS23	8205.25856	0.54724163	0.07510223	7.28662249	3.18E-13
20376	RPS27L	1922.33804	-1.0794103	0.1081051	-9.9848231	1.78E-23
20387	RPS6KA1	2304.8328	0.46229628	0.06588867	7.01632468	2.28E-12
20388	RPS6KA2	1560.2624	0.59618175	0.08057075	7.39948148	1.37E-13
20390	RPS6KA2-IT1	6.19983623	2.59011999	0.92872769	2.78889068	0.00528889
20392	RPS6KA4	5962.08172	-0.2048707	0.07763264	-2.6389756	0.0083157
20393	RPS6KA5	338.169793	0.64793013	0.11925059	5.43334926	5.53E-08
20396	RPS6KB2	2361.57112	-0.4832261	0.08646894	-5.588435	2.29E-08
20401	RPS8	16904.507	0.59998641	0.06651197	9.02072768	1.87E-19
20403	RPSA	4377.29283	0.40268146	0.09027598	4.46056055	8.17E-06
20404	RPSAP52	54.6865311	2.979268	0.37304641	7.98632004	1.39E-15
20412	RPUSD4	1591.78389	-0.2903433	0.08901495	-3.2617367	0.00110732
20415	RRAGA	3341.89125	-0.3606135	0.07766745	-4.6430452	3.43E-06
20416	RRAGB	773.013515	-0.517706	0.09373331	-5.5231803	3.33E-08
20417	RRAGC	4764.17683	0.6975208	0.06169728	11.3055362	1.23E-29
20422	RREB1	1928.54309	0.51360343	0.08149707	6.3021094	2.94E-10
20427	RRN3	2108.55574	1.29392724	0.0895102	14.4556405	2.31E-47
20433	RRP12	2385.30927	0.78764042	0.07023054	11.2150702	3.44E-29

20434	RRP15	2341.22161	0.65694227	0.0746706	8.79787101	1.39E-18
20435	RRP1B	2815.39833	0.45838157	0.07706602	5.9479079	2.72E-09
20436	RRP36	2814.26297	0.13153737	0.0624421	2.10654932	0.03515666
20437	RRP7A	3681.58559	0.42842946	0.08389947	5.106462	3.28E-07
20439	RRP8	573.072813	0.34604355	0.10063785	3.43850299	0.00058494
20440	RRP9	1532.6053	0.76422901	0.09289869	8.22647783	1.93E-16
20441	RRS1	1284.33236	0.71433769	0.08537932	8.36663595	5.93E-17
20444	RSAD1	1444.4455	-0.2272872	0.09198618	-2.4708838	0.01347796
20448	RSC1A1	88.1971125	0.71082628	0.23020581	3.08778596	0.00201654
20451	RSL1D1	7096.18624	0.79155367	0.05992142	13.2098608	7.70E-40
20452	RSL24D1	4690.76824	1.68246813	0.10320474	16.3022367	9.51E-60
20465	RSPRY1	1464.13984	0.54230881	0.08169855	6.63792429	3.18E-11
20466	RSRC1	649.415716	-0.7903276	0.14330911	-5.5148453	3.49E-08
20467	RSRC2	4666.29899	1.01027665	0.06441834	15.6830599	1.98E-55
20475	RTEL1	101.922975	-2.0459105	0.22031737	-9.2861973	1.60E-20
20477	RTF1	2878.58521	-0.4421358	0.0688187	-6.4246466	1.32E-10
20479	RTKN	772.775566	-0.4255792	0.11265358	-3.7777688	0.00015824
20483	RTN2	519.775906	-1.0115003	0.10321208	-9.8002127	1.12E-22
20484	RTN3	11092.1436	-0.1550705	0.05992178	-2.5878828	0.00965678
20485	RTN4	30071.9636	0.58022715	0.06980881	8.31166069	9.44E-17
20486	RTN4IP1	317.84409	-0.570407	0.12050901	-4.7333137	2.21E-06
20496	RUFY1	3889.52343	-0.4664875	0.06646873	-7.0181491	2.25E-12
20500	RUNDC1	1358.61059	0.56198142	0.08087426	6.94882904	3.68E-12
20504	RUNX1	3516.17974	0.42427939	0.06737226	6.29753819	3.02E-10
20512	RUVBL1	2876.83207	0.41791603	0.06876781	6.07720432	1.22E-09
20527	RYBP	4204.42027	0.88464862	0.06878668	12.860754	7.48E-38
20534	S100A11	13138.1572	0.23564773	0.06941103	3.39496105	0.00068638
20537	S100A14	10.5273176	1.45676419	0.64658007	2.25302983	0.02425727
20538	S100A16	10301.4349	0.43813374	0.06610459	6.62788674	3.41E-11
20540	S100A3	1444.27901	-0.9939522	0.08653873	-11.485634	1.56E-30
20543	S100A6	57450.9025	-0.2247109	0.08842172	-2.5413535	0.01104242
20552	S100PBP	1398.11277	0.83798137	0.07182636	11.6667659	1.88E-31
20553	S100Z	12.4341012	3.72069901	0.81029285	4.59179544	4.39E-06
20558	S1PR5	34.5789302	-2.5853551	0.39632223	-6.5233664	6.87E-11
20559	SAA1	9477.87261	-0.5108182	0.12916267	-3.9548435	7.66E-05
20560	SAA2	2379.02749	-0.7899896	0.07354683	-10.741313	6.51E-27
20565	SAC3D1	1810.36583	-0.4437087	0.14385784	-3.0843551	0.00203994
20567	SACS	2726.93832	0.81605441	0.07877144	10.3597756	3.78E-25
20575	SALL2	107.012077	-3.8918536	0.28406311	-13.700665	1.01E-42
20590	SAMD4A	4340.40201	0.59362946	0.07082822	8.38125688	5.24E-17
20595	SAMD9	1436.33819	0.53786767	0.12072028	4.45548721	8.37E-06
20598	SAMM50	1668.68292	-0.2299622	0.07059374	-3.2575441	0.00112381
20604	SAP30	1176.11687	-0.3940801	0.11140527	-3.5373561	0.00040415

20610	SAPCD2	1397.17265	-0.1960294	0.09040367	-2.168379	0.03012986
20611	SAR1A	6318.336	-0.6675001	0.05712858	-11.68417	1.54E-31
20613	SARAF	4937.42878	-0.6902668	0.08358731	-8.2580333	1.48E-16
20614	SARDH	776.896693	-1.4786645	0.09997385	-14.790514	1.69E-49
20617	SARS	16655.8256	1.17296443	0.06850332	17.1227393	1.00E-65
20618	SARS2	590.175879	-1.0509406	0.09939101	-10.573799	3.94E-26
20619	SART1	5814.84478	-0.3030691	0.08908494	-3.4020243	0.00066889
20624	SAT1	5866.33806	1.54822345	0.08821158	17.5512494	5.82E-69
20626	SATB1	463.21213	1.13400585	0.12083403	9.38482156	6.30E-21
20635	SBDS	6967.54644	0.56164992	0.05630904	9.97441811	1.97E-23
20636	SBDSP1	1534.28076	0.45271137	0.07728952	5.85734457	4.70E-09
20651	SCAF4	2173.28321	0.61327109	0.08185471	7.4921908	6.77E-14
20652	SCAF8	2393.49512	0.37246754	0.07951914	4.68399835	2.81E-06
20655	SCAMP1-AS1	243.477221	0.80081208	0.14155524	5.65724058	1.54E-08
20656	SCAMP2	1475.11456	-0.7728671	0.11146806	-6.9335301	4.10E-12
20659	SCAMP5	505.213617	-1.0625721	0.10772969	-9.863318	6.00E-23
20663	SCAPER	481.316282	-0.303353	0.11648786	-2.6041598	0.00920998
20664	SCARA3	630.132685	-1.7580093	0.10277889	-17.104771	1.37E-65
20665	SCARA5	19.5462026	-1.4693132	0.50555163	-2.9063563	0.00365665
20666	SCARB1	2355.03648	0.24843026	0.06560779	3.78659711	0.00015272
20667	SCARB2	6824.76681	-0.2169249	0.07569993	-2.8655886	0.00416235
20668	SCARF1	176.852893	-0.7052199	0.18301309	-3.8533853	0.0001165
20669	SCARF2	515.524857	-1.5830769	0.13018212	-12.160478	5.05E-34
20696	SCD	19068.3251	-0.9236755	0.06773957	-13.635686	2.46E-42
20700	SCFD1	3943.29345	0.8347774	0.06500126	12.8424793	9.48E-38
20702	SCG2	268.983254	-1.1817424	0.15537536	-7.6057259	2.83E-14
20725	SCMH1	2621.35985	0.51815202	0.08263816	6.27012995	3.61E-10
20726	SCML1	2375.00906	1.00060764	0.0847126	11.8117923	3.39E-32
20727	SCML2	618.592423	1.44001985	0.11839217	12.1631344	4.88E-34
20732	SCN1B	259.908159	-2.2977524	0.14543692	-15.798962	3.16E-56
20739	SCN5A	792.750314	-2.3711379	0.11143467	-21.278278	1.80E-100
20742	SCN9A	90.0631287	1.13021297	0.22280858	5.07257376	3.92E-07
20744	SCNN1A	1165.01217	-1.4930622	0.08782642	-17.000149	8.19E-65
20748	SCO1	1673.86924	0.69058647	0.07495542	9.2132954	3.16E-20
20754	SCPEP1	2146.54662	-0.4706917	0.07269941	-6.4744916	9.51E-11
20758	SCRN2	841.950024	-1.0126639	0.11170962	-9.0651449	1.24E-19
20767	SCX	149.631584	-1.2111187	0.21289798	-5.6887278	1.28E-08
20769	SCYL2	1980.32372	0.78043151	0.10114014	7.71633812	1.20E-14
20770	SCYL3	486.942274	0.30993508	0.099728	3.10780394	0.00188483
20771	SDAD1	3841.9054	0.72886878	0.06542406	11.1406848	7.95E-29
20773	SDC2	2730.30279	-0.4707294	0.06975228	-6.7485878	1.49E-11
20774	SDC3	3240.82949	-0.173177	0.06229954	-2.7797478	0.00544011
20775	SDC4	16409.8705	-0.1212571	0.05705913	-2.1251129	0.0335772

20777	SDCBP	14028.9976	0.29547471	0.05682615	5.19962536	2.00E-07
20780	SDCCAG3	3748.10137	0.30926197	0.07766617	3.98193927	6.84E-05
20782	SDE2	1495.36031	0.6819161	0.07797954	8.74480847	2.23E-18
20783	SDF2	2142.00845	-0.5077742	0.10743874	-4.7261742	2.29E-06
20786	SDHA	7791.13339	-0.5969958	0.07239859	-8.2459591	1.64E-16
20789	SDHAF3	671.718753	-0.2212135	0.09896975	-2.2351625	0.02540668
20796	SDHD	4254.33938	-0.2778401	0.06346591	-4.3777838	1.20E-05
20797	SDK1	103.139598	-3.3224547	0.28548337	-11.637997	2.64E-31
20799	SDPR	2510.49685	-0.3829685	0.07962507	-4.8096473	1.51E-06
20800	SDR16C5	20.6407918	-4.1754998	0.68129221	-6.1287942	8.85E-10
20808	SEC11A	3954.12035	-0.4284941	0.07783865	-5.5049014	3.69E-08
20809	SEC11C	667.737561	0.44415317	0.09979347	4.450724	8.56E-06
20815	SEC14L4	30.4110852	-1.3675503	0.36886681	-3.7074365	0.00020937
20818	SEC16A	5593.0696	0.24438767	0.06626675	3.6879381	0.00022608
20819	SEC16B	63.8172548	-1.8165218	0.27154172	-6.6896601	2.24E-11
20823	SEC22C	2834.11781	-0.2884701	0.06273828	-4.5979915	4.27E-06
20825	SEC23B	4566.92272	0.40816154	0.05993945	6.8095649	9.79E-12
20830	SEC24C	5400.40204	-0.645836	0.05660742	-11.409036	3.77E-30
20833	SEC31B	467.352788	-0.8753544	0.10364137	-8.445994	3.01E-17
20835	SEC61A2	814.506802	0.31491948	0.08883343	3.54505579	0.00039253
20839	SEC63	4592.03268	0.80620676	0.07080798	11.3858175	4.92E-30
20840	SECISBP2	1948.41346	0.34463502	0.06849047	5.03186795	4.86E-07
20843	SEH1L	5711.53609	0.69965088	0.05943026	11.7726379	5.40E-32
20848	SELENBP1	507.254933	-1.5970675	0.11352153	-14.068411	5.94E-45
20852	SELO	983.33052	-1.0348748	0.11866595	-8.7209082	2.76E-18
20858	SEMA3B	2233.35294	-1.365003	0.1150936	-11.859938	1.91E-32
20861	SEMA3D	81.8959144	2.56950912	0.28226863	9.10306314	8.78E-20
20863	SEMA3F	199.112655	-1.0229461	0.16089865	-6.3577051	2.05E-10
20864	SEMA3G	34.8663825	-2.9338659	0.40757763	-7.1982996	6.10E-13
20866	SEMA4B	2979.61335	-1.0066349	0.06651918	-15.133002	9.81E-52
20869	SEMA4F	661.469133	-0.834479	0.09346552	-8.9282019	4.33E-19
20870	SEMA4G	93.5151956	-2.3268336	0.25982548	-8.9553713	3.39E-19
20875	SEMA6B	2809.15306	-1.9414355	0.08534089	-22.749182	1.46E-114
20876	SEMA6C	118.201625	-1.1984126	0.19786925	-6.0565886	1.39E-09
20877	SEMA6D	3.79932992	-5.4619491	1.53201791	-3.5651992	0.00036358
20878	SEMA7A	2212.43796	1.36166167	0.08070775	16.8715109	7.29E-64
20887	SENP6	4239.02845	0.14302363	0.06449158	2.21771015	0.0265746
20890	15-Sep	11703.5268	-0.4059869	0.05367878	-7.5632657	3.93E-14
20892	SEPHS2	2744.13589	0.2111786	0.06989023	3.02157552	0.00251463
20893	SEPN1	3141.11827	-0.6798234	0.10108938	-6.7249734	1.76E-11
20894	SEPP1	32.2715753	-3.3395823	0.47325628	-7.0566044	1.71E-12
20897	1-Sep	108.135191	-0.7797151	0.21083073	-3.6982993	0.00021705
20903	3-Sep	72.2387581	-0.9844038	0.28853024	-3.4117871	0.00064538

20904	4-Sep	155.279221	-2.6179127	0.1954781	-13.392357	6.70E-41
20908	6-Sep	794.735826	-0.5815189	0.08821751	-6.5918762	4.34E-11
20909	7-Sep	7452.76351	-0.4135607	0.06982007	-5.9232355	3.16E-09
20911	SEPT7P2	258.316391	0.44990181	0.14755418	3.04906177	0.00229557
20913	8-Sep	2983.87506	-0.4091591	0.06181699	-6.6188779	3.62E-11
20916	SERAC1	810.867483	0.38898955	0.08768879	4.43602374	9.16E-06
20917	SERBP1	13246.3001	1.17360018	0.05255234	22.3320242	1.81E-110
20922	SERGEF	660.13493	-0.4071623	0.09253504	-4.4000882	1.08E-05
20924	SERHL2	14.9267533	-1.5512517	0.5366679	-2.8905244	0.003846
20926	SERINC2	3871.20382	0.54929959	0.07296599	7.52815962	5.15E-14
20932	SERPINA1	6428.2813	-0.8839332	0.05632051	-15.694694	1.64E-55
20938	SERPINA3	152.892548	-0.9436038	0.17287466	-5.4583118	4.81E-08
20940	SERPINA5	51.8458921	-3.6463877	0.40162588	-9.0790655	1.10E-19
20944	SERPINB1	4761.80103	-0.1736779	0.06464852	-2.6864953	0.0072206
20949	SERPINB2	2.35591061	3.52170229	1.63703408	2.15126999	0.0314549
20952	SERPINB5	4.18860839	3.44588807	1.38232144	2.49282691	0.01267306
20960	SERPINE1	35594.6142	0.73137555	0.04988108	14.6623833	1.12E-48
20961	SERPINE2	1141.6463	1.07319671	0.08714161	12.3155477	7.47E-35
20970	SERTAD2	4959.02734	0.76882783	0.06495472	11.8363666	2.53E-32
20975	SESN1	263.796805	-0.5183408	0.13759169	-3.7672393	0.00016506
20976	SESN2	600.70118	1.46646323	0.10548708	13.9018275	6.17E-44
20977	SESN3	42.6266116	-0.6638916	0.31809804	-2.0870661	0.03688216
20978	SESTD1	818.125434	-0.1992244	0.09672808	-2.0596337	0.03943357
20979	SET	22918.407	0.87292344	0.06781519	12.8720921	6.46E-38
20985	SETD4	783.184678	0.55335447	0.08868809	6.23933259	4.39E-10
20986	SETD5	5593.49745	0.23378444	0.079612	2.9365478	0.00331888
20989	SETD8	1624.83995	0.77317449	0.07850677	9.8485072	6.96E-23
20999	SF1	14336.5869	0.38810787	0.07805029	4.97253561	6.61E-07
21000	SF3A1	4392.17939	0.4876117	0.06905606	7.06109884	1.65E-12
21001	SF3A2	3869.02764	0.38005504	0.06986947	5.43950103	5.34E-08
21002	SF3A3	4689.57302	1.04739993	0.0733217	14.2849916	2.71E-46
21006	SF3B4	3249.14749	0.44617716	0.09342507	4.77577542	1.79E-06
21008	SF3B6	2985.51633	0.46950209	0.0964155	4.86957053	1.12E-06
21009	SFI1	582.903769	-0.7080282	0.09948857	-7.1166787	1.11E-12
21010	SFMBT1	1131.60126	0.41628242	0.07686954	5.41544022	6.11E-08
21012	SFN	3951.56087	0.4539372	0.10012664	4.53363038	5.80E-06
21020	SFT2D1	3740.79527	-0.4258049	0.0792053	-5.375965	7.62E-08
21023	SFTA1P	723.147951	1.51228225	0.09706436	15.5802012	9.92E-55
21033	SFXN3	2329.48463	-1.560722	0.07852764	-19.874811	6.72E-88
21035	SFXN5	431.590405	-0.6818972	0.11041118	-6.1759801	6.58E-10
21053	SGOL2	2586.22232	-0.5018706	0.07542434	-6.6539611	2.85E-11
21054	SGPL1	2783.85384	-1.086834	0.06920611	-15.704307	1.41E-55
21059	SGSM2	3419.99481	-0.2621329	0.06473352	-4.0494159	5.13E-05

21060	SGSM3	1623.16701	-1.1972264	0.08358611	-14.323269	1.57E-46
21061	SGTA	5823.15477	0.62571233	0.07095094	8.81894407	1.16E-18
21062	SGTB	1300.54354	1.0776184	0.10063573	10.7081095	9.32E-27
21063	SH2B1	1381.40305	-0.3754361	0.0834897	-4.4967947	6.90E-06
21065	SH2B3	4042.23751	0.71425066	0.07721706	9.24990707	2.25E-20
21069	SH2D3A	1295.79266	-1.2487618	0.09948691	-12.552022	3.87E-36
21073	SH2D5	1695.14405	1.45701437	0.1055745	13.8008176	2.52E-43
21077	SH3BGRL	2231.16478	-2.2553817	0.07928747	-28.445627	5.52E-178
21080	SH3BP1	446.922414	-1.3676838	0.11366057	-12.033055	2.38E-33
21083	SH3BP5	1120.27233	1.02721452	0.08276081	12.4118467	2.25E-35
21085	SH3BP5L	1914.96093	-0.4215901	0.09108972	-4.6282954	3.69E-06
21093	SH3GLB1	7190.67283	-0.3667643	0.07777371	-4.7157879	2.41E-06
21094	SH3GLB2	3308.14561	-0.6817829	0.08086093	-8.4315491	3.41E-17
21095	SH3KBP1	12183.7534	0.84539068	0.07942349	10.6440886	1.86E-26
21099	SH3RF1	1753.44994	0.60196628	0.07786368	7.73102774	1.07E-14
21103	SH3TC1	160.894787	-0.7001476	0.16261895	-4.3054487	1.67E-05
21104	SH3TC2	1467.04888	-1.2518696	0.10122168	-12.367603	3.91E-35
21105	SH3YL1	427.800344	0.67080818	0.13963509	4.80400872	1.56E-06
21112	SHB	2403.84791	-0.4309444	0.07047542	-6.1148185	9.67E-10
21116	SHC3	537.397004	-1.4071532	0.10249008	-13.729653	6.75E-43
21123	SHFM1	2647.37521	-0.5591815	0.1034169	-5.4070607	6.41E-08
21124	SHH	86.3387392	-1.9475706	0.239482	-8.13243	4.21E-16
21127	SHISA4	425.183601	-0.5882581	0.10863534	-5.4149796	6.13E-08
21133	SHKBP1	2568.24285	-0.6629213	0.06362085	-10.419875	2.01E-25
21134	SHMT1	1182.52522	-0.7433881	0.07997419	-9.2953504	1.47E-20
21139	SHPK	546.006007	-0.4844664	0.10680672	-4.535917	5.74E-06
21142	SHROOM1	766.966882	-0.3904116	0.11824187	-3.3018048	0.00096065
21148	SIAE	1416.35932	-0.8520102	0.0851018	-10.01166	1.35E-23
21149	SIAH1	1083.0876	0.49020882	0.09025492	5.4313805	5.59E-08
21150	SIAH2	916.625658	0.45435882	0.08604026	5.28077013	1.29E-07
21154	SIGIRR	250.306829	-0.796458	0.16413379	-4.8524925	1.22E-06
21171	SIK2	1721.95986	0.61868401	0.08760829	7.06193447	1.64E-12
21177	SIMC1	789.553848	0.46102882	0.10348668	4.45495824	8.39E-06
21180	SIPA1	3082.10243	-0.7872488	0.09713499	-8.1046882	5.29E-16
21182	SIPA1L2	64.4123	4.11465141	0.3976311	10.3479115	4.28E-25
21185	SIRPB1	25.4540553	-5.7406015	0.90571894	-6.3381709	2.33E-10
21191	SIRT2	1280.93429	-0.3663757	0.07356365	-4.9803907	6.35E-07
21192	SIRT3	757.227511	-0.6439837	0.10310753	-6.2457481	4.22E-10
21204	SIX5	1076.73875	-0.3873847	0.08859952	-4.3723117	1.23E-05
21207	SKA2	3565.01743	0.30904468	0.06134143	5.03810668	4.70E-07
21213	SKIL	2634.41762	0.75222956	0.06960584	10.8069899	3.19E-27
21216	SKIV2L2	1761.48668	0.38085001	0.07164277	5.31595852	1.06E-07
21221	SKP2	1089.47408	-0.602267	0.09022889	-6.6748803	2.47E-11

21236	SLC10A5	31.2351805	2.03587185	0.40517431	5.02468145	5.04E-07
21240	SLC11A2	2672.8395	0.34034249	0.06800912	5.00436561	5.60E-07
21244	SLC12A4	2257.19368	-0.3353338	0.06803686	-4.9287073	8.28E-07
21247	SLC12A7	3069.67389	0.47972192	0.06854442	6.99870117	2.58E-12
21249	SLC12A9	997.949544	-0.6833007	0.09025766	-7.5705568	3.72E-14
21263	SLC16A1	102.555331	0.79753484	0.20405739	3.90838503	9.29E-05
21271	SLC16A2	112.312831	-0.7733673	0.19008938	-4.0684402	4.73E-05
21272	SLC16A3	15985.8318	-0.4178133	0.06477739	-6.4499874	1.12E-10
21273	SLC16A4	110.507984	-0.4964619	0.1903724	-2.607846	0.00911139
21283	SLC17A5	4286.69651	-1.0013122	0.05932128	-16.879475	6.37E-64
21287	SLC17A9	2736.61499	0.14423863	0.0655957	2.19890355	0.02788478
21291	SLC18B1	819.669175	-0.7288664	0.11653629	-6.254416	3.99E-10
21298	SLC1A4	836.875684	1.08075235	0.08624919	12.5305796	5.08E-36
21299	SLC1A5	12537.2216	1.44583309	0.05124061	28.2165452	3.66E-175
21301	SLC1A7	21.2674324	-1.4630853	0.45087983	-3.2449563	0.00117469
21302	SLC20A1	11362.5052	0.48520669	0.05880764	8.25074262	1.57E-16
21310	SLC22A15	636.950373	1.55469618	0.09717238	15.9993627	1.29E-57
21313	SLC22A18	401.931198	-1.0995762	0.11726127	-9.3771471	6.78E-21
21316	SLC22A20	42.8278281	-0.9629465	0.35143152	-2.7400686	0.00614264
21330	SLC23A3	95.5154514	1.3114795	0.21628671	6.06361567	1.33E-09
21336	SLC25A1	2882.97511	-0.4815729	0.08924983	-5.3957851	6.82E-08
21337	SLC25A10	2014.25586	-0.7862531	0.07961867	-9.8752354	5.33E-23
21338	SLC25A11	1931.8553	-0.207291	0.09773649	-2.120917	0.03392879
21339	SLC25A12	906.014589	-0.6369234	0.08302521	-7.6714456	1.70E-14
21346	SLC25A19	444.517193	0.67635941	0.10979431	6.16024071	7.26E-10
21351	SLC25A22	4018.7214	0.7787322	0.08125893	9.58334257	9.40E-22
21352	SLC25A23	1971.34006	-0.7274223	0.09116709	-7.9790007	1.48E-15
21354	SLC25A25	1644.24176	0.89002538	0.10088941	8.82179225	1.13E-18
21364	SLC25A32	3924.70857	1.1647129	0.06444156	18.0739405	5.11E-73
21365	SLC25A33	418.319611	0.95833029	0.11271798	8.50201793	1.86E-17
21367	SLC25A35	116.531587	-1.1203923	0.19434093	-5.7650867	8.16E-09
21370	SLC25A38	2218.01712	0.5582552	0.07658016	7.28981488	3.10E-13
21373	SLC25A4	2322.13306	-0.2368067	0.09014041	-2.6270876	0.00861192
21376	SLC25A42	230.246898	-0.7636863	0.16514972	-4.6242058	3.76E-06
21378	SLC25A44	1760.97387	0.20116049	0.08069016	2.49299901	0.01266692
21380	SLC25A46	2410.78622	-0.2681156	0.07289468	-3.6781235	0.00023496
21393	SLC26A2	1335.05719	-0.9634524	0.10451148	-9.2186278	3.01E-20
21398	SLC26A6	1304.18156	-0.6003596	0.07714049	-7.7826774	7.10E-15
21399	SLC26A7	38.8280967	0.76949237	0.32044371	2.40133397	0.01633542
21402	SLC27A1	1207.50934	-0.9205044	0.08221498	-11.196309	4.25E-29
21404	SLC27A3	181.721227	-1.880606	0.19000114	-9.8978668	4.25E-23
21411	SLC29A1	6904.00841	0.3075875	0.05792757	5.30986406	1.10E-07
21417	SLC2A10	162.471892	-1.0597769	0.17213297	-6.1567337	7.43E-10

21419	SLC2A12	407.629806	-1.6927743	0.16197541	-10.45081	1.45E-25
21420	SLC2A13	149.597152	-2.1838565	0.19987526	-10.926097	8.65E-28
21425	SLC2A4RG	5717.72823	-0.443386	0.09048887	-4.8998956	9.59E-07
21426	SLC2A5	49.6795491	-1.0142073	0.30608995	-3.313429	0.00092159
21440	SLC30A9	3479.07879	-0.2851274	0.08607729	-3.3124583	0.0009248
21442	SLC31A2	2692.23311	0.35366672	0.07156988	4.94155787	7.75E-07
21451	SLC35A4	4829.97766	-0.1630599	0.05815361	-2.8039515	0.00504805
21453	SLC35B1	2020.06714	-0.2842047	0.08101587	-3.5080122	0.00045147
21454	SLC35B2	3927.91772	-0.5997807	0.06777447	-8.8496551	8.78E-19
21455	SLC35B3	1433.86551	0.19254872	0.07986079	2.4110546	0.01590647
21464	SLC35E2B	2602.67306	0.27025016	0.08546694	3.16204332	0.00156666
21468	SLC35F2	5961.84419	0.71138247	0.07096501	10.0244119	1.19E-23
21479	SLC36A1	942.461183	0.27617338	0.10759153	2.56686915	0.01026213
21483	SLC37A1	380.740202	-0.85874	0.11959343	-7.1804947	6.95E-13
21485	SLC37A3	3285.38271	-0.2098198	0.06865278	-3.056247	0.00224127
21486	SLC37A4	2110.80518	-1.2561168	0.07465641	-16.825305	1.59E-63
21487	SLC38A1	16901.4259	0.96854272	0.06493733	14.9150372	2.63E-50
21490	SLC38A2	53126.2011	1.77109782	0.07741579	22.8777343	7.74E-116
21492	SLC38A4	1037.6847	-0.271863	0.1074682	-2.529706	0.01141581
21494	SLC38A6	473.048464	-1.6636814	0.10920316	-15.234737	2.08E-52
21498	SLC39A1	12198.7729	0.15417502	0.07848204	1.96446253	0.04947648
21499	SLC39A10	4726.25862	-0.3597236	0.08232016	-4.369812	1.24E-05
21500	SLC39A11	429.467666	-0.9768912	0.12233158	-7.9856012	1.40E-15
21503	SLC39A13	4259.00317	-0.5288927	0.08449119	-6.2597384	3.86E-10
21504	SLC39A14	8676.0491	1.01259305	0.06855772	14.7699348	2.29E-49
21512	SLC39A9	3452.378	-0.3196589	0.07110716	-4.4954531	6.94E-06
21514	SLC3A2	12529.4326	1.27832751	0.08692973	14.7052966	5.96E-49
21515	SLC40A1	255.172839	-1.6760378	0.15273158	-10.973748	5.11E-28
21519	SLC43A1	708.285784	0.22442894	0.09278185	2.41888832	0.01556802
21520	SLC43A2	328.221837	-1.1451487	0.1242877	-9.2136932	3.15E-20
21521	SLC43A3	17396.7301	0.13266001	0.05642771	2.3509728	0.0187244
21522	SLC44A1	3424.6278	-0.5074224	0.06855923	-7.4012265	1.35E-13
21523	SLC44A2	8953.74696	-0.5102494	0.05164509	-9.8799194	5.09E-23
21527	SLC45A1	93.5076503	-0.7477928	0.22337164	-3.3477518	0.0008147
21531	SLC46A1	1204.06408	-0.1755308	0.08668283	-2.0249779	0.04286963
21534	SLC47A1	155.953867	-2.1392623	0.1860371	-11.499117	1.33E-30
21541	SLC4A2	12637.533	-0.2568207	0.06393831	-4.0166953	5.90E-05
21544	SLC4A5	36.8788326	1.16645277	0.38620384	3.02030336	0.00252522
21545	SLC4A7	2353.99821	1.54814199	0.09541751	16.2249258	3.36E-59
21572	SLC6A14	11.9305351	-2.5878074	0.70449604	-3.6732746	0.00023946
21583	SLC6A6	3954.59035	-0.2028673	0.0860975	-2.3562512	0.01846043
21585	SLC6A8	178.385912	-1.0533415	0.17658765	-5.9649782	2.45E-09
21586	SLC6A9	1581.85281	1.57470449	0.07175168	21.9465858	9.34E-107

21587	SLC7A1	8492.16154	0.93950867	0.08579013	10.9512442	6.55E-28
21589	SLC7A11	4579.08079	2.0305251	0.06319393	32.1316502	1.59E-226
21590	SLC7A11-AS1	5.9209037	2.86354599	1.01338359	2.8257276	0.00471734
21593	SLC7A2	1386.46878	1.23808611	0.09042955	13.6911678	1.15E-42
21596	SLC7A5	14850.7351	1.29161185	0.06277175	20.5763251	4.47E-94
21599	SLC7A6	2549.54288	0.48014538	0.07975693	6.02010864	1.74E-09
21608	SLC8B1	3369.90622	-0.5140596	0.09325974	-5.5121275	3.55E-08
21609	SLC9A1	1068.13404	0.33736247	0.08799776	3.83376205	0.0001262
21616	SLC9A6	1999.50221	-0.2280937	0.08264349	-2.7599721	0.00578063
21617	SLC9A7	2883.52352	-0.2041469	0.06505733	-3.1379548	0.00170131
21618	SLC9A7P1	37.7817128	-0.7695744	0.31676884	-2.4294512	0.0151217
21620	SLC9A9	14.4059404	1.45579861	0.61427043	2.36996367	0.01778983
21625	SLC9C2	11.5139739	-1.2279149	0.6069637	-2.0230451	0.0430685
21633	SLCO3A1	1428.82781	-0.5920036	0.07676351	-7.712044	1.24E-14
21634	SLCO4A1	1312.3006	-1.9998999	0.07677644	-26.048352	1.40E-149
21635	SLCO4A1-AS1	567.625352	-1.8774212	0.10476988	-17.919474	8.31E-72
21641	SLF2	2530.59868	-0.2338255	0.08211619	-2.847496	0.00440647
21643	SLFN12	1101.39815	0.68135928	0.09975278	6.83047894	8.46E-12
21644	SLFN12L	67.7445884	0.69874501	0.24366218	2.86767938	0.00413494
21645	SLFN13	880.517143	-0.348552	0.10389392	-3.3548831	0.00079399
21647	SLFN5	1121.18879	0.37747776	0.08954208	4.21564669	2.49E-05
21649	SLFNL1-AS1	53.756728	1.0303964	0.27434897	3.75578741	0.0001728
21663	SLMAP	4190.63379	-0.1866453	0.06408432	-2.9124962	0.00358553
21664	SLMO1	431.95238	1.38945294	0.1434176	9.68816222	3.39E-22
21665	SLMO2	6099.08264	0.87806589	0.06460664	13.5909542	4.53E-42
21670	SLU7	2426.73467	0.34987168	0.06188502	5.65357657	1.57E-08
21680	SMAD1	391.481817	-0.5497678	0.12099209	-4.5438327	5.52E-06
21683	SMAD2	3767.64903	0.31807711	0.08103633	3.9251175	8.67E-05
21688	SMAD6	302.499989	-1.2633264	0.13593361	-9.2937015	1.49E-20
21693	SMAP2	1084.15919	0.70162079	0.0926291	7.57451829	3.60E-14
21696	SMARCA4	7221.44882	-0.2306457	0.05644323	-4.0863305	4.38E-05
21701	SMARCB1	2766.23983	-0.4020367	0.08564811	-4.6940519	2.68E-06
21702	SMARCC1	3559.49232	0.16614235	0.07243058	2.29381506	0.02180112
21708	SMC1A	8958.88317	-0.1543154	0.07644247	-2.0187126	0.0435171
21713	SMC4	9272.83277	0.22957965	0.06997123	3.28105799	0.00103418
21721	SMCO4	439.366095	-0.3267355	0.11353491	-2.8778421	0.00400406
21725	SMCR8	2552.02665	0.32367757	0.1026018	3.15469679	0.00160665
21727	SMG1	4922.53888	0.84539759	0.10676602	7.91822731	2.41E-15
21734	SMG6	2171.08819	-0.3686098	0.06313058	-5.8388478	5.26E-09
21735	SMG7	4085.90299	0.93747782	0.10242238	9.15305617	5.53E-20
21738	SMG9	3079.84174	0.22670043	0.07722538	2.93556915	0.00332936
21741	SMIM10L1	791.251795	-0.5428737	0.09029734	-6.0120674	1.83E-09
21745	SMIM12	2289.80509	0.72973471	0.08832229	8.26218043	1.43E-16

21746	SMIM13	4133.50521	0.99781612	0.0693441	14.3893437	6.04E-47
21747	SMIM14	309.131648	-0.9106993	0.12675027	-7.1849893	6.72E-13
21748	SMIM15	5802.38548	0.63283833	0.06137031	10.3117992	6.23E-25
21753	SMIM2-AS1	79.14919	1.45460805	0.25911917	5.61366438	1.98E-08
21760	SMIM3	652.745808	0.72439758	0.09631166	7.52139017	5.42E-14
21761	SMIM4	476.005086	0.75480475	0.16425211	4.59540353	4.32E-06
21764	SMIM7	2819.55289	-0.1997051	0.09319777	-2.1428097	0.03212839
21767	SMKR1	105.85694	0.54536347	0.2247348	2.42669789	0.01523694
21775	SMOX	886.465606	0.28555268	0.08632483	3.30788562	0.00094003
21776	SMPD1	2369.87929	-1.2158345	0.0731539	-16.620229	4.97E-62
21780	SMPDL3A	309.613028	-1.7551194	0.13462959	-13.036654	7.57E-39
21791	SMURF1	3615.35003	0.62211262	0.07310467	8.50988884	1.74E-17
21792	SMURF2	8558.52226	0.42139711	0.07047956	5.97899743	2.25E-09
21797	SMYD5	1550.4593	0.21963759	0.07267578	3.02215655	0.00250981
21798	SNAI1	43.1513212	0.89159502	0.3167419	2.81489449	0.00487933
21805	SNAP29	1588.6852	0.32985939	0.08001208	4.12261994	3.75E-05
21806	SNAP47	1826.93445	-0.2317153	0.07691684	-3.0125428	0.00259069
21808	SNAPC1	6393.01724	1.02214564	0.06260551	16.3267685	6.37E-60
21813	SNAPIN	697.761931	-1.1318859	0.09596116	-11.79525	4.13E-32
21846	SNCG	438.07838	-3.6734769	0.16986014	-21.626479	1.01E-103
21849	SNED1	63.6165858	-1.3076873	0.27695005	-4.7217443	2.34E-06
21851	SNHG1	7357.21891	2.01584821	0.07073262	28.4995557	1.19E-178
21854	SNHG12	560.890335	0.9223082	0.12407685	7.43336257	1.06E-13
21855	SNHG15	2173.79942	1.93859547	0.11850517	16.3587415	3.77E-60
21857	SNHG17	1691.73088	1.78984779	0.12922434	13.8507015	1.26E-43
21865	SNHG3	3352.03981	1.45452217	0.07761606	18.7399634	2.34E-78
21866	SNHG4	255.279057	0.74709824	0.14031242	5.32453384	1.01E-07
21870	SNHG8	2725.1841	1.49379644	0.23771103	6.28408552	3.30E-10
21873	SNN	907.865927	-1.301119	0.09669006	-13.456596	2.82E-41
21947	SNORA67	81.2503456	0.79458113	0.23871688	3.32855026	0.00087299
21950	SNORA70	79.4666133	0.61126079	0.27742487	2.20333814	0.02757092
21957	SNORA71A	36.3427526	1.41619993	0.36527502	3.87707847	0.00010572
21959	SNORA71C	139.710294	3.21185737	0.25131691	12.7801085	2.12E-37
21963	SNORA73A	72.8381385	0.7988735	0.28588203	2.79441666	0.00519934
21964	SNORA73B	85.0711056	0.84581752	0.32757711	2.58204102	0.00982179
21967	SNORA75	6.49285146	2.06654155	0.91009918	2.27067731	0.02316652
21981	SNORA9	22.9695942	0.86282787	0.43774374	1.97107986	0.04871474
22141	SNORD17	35.4826569	1.43905625	0.48245895	2.98275376	0.00285668
22155	SNORD24	40.9639524	2.65196069	0.40495475	6.5487828	5.80E-11
22189	SNORD47	151.866873	1.11024373	0.18545395	5.98662768	2.14E-09
22230	SNORD76	168.442094	1.18744286	0.16876731	7.03597684	1.98E-12
22261	SNPH	554.246259	-1.0993762	0.11254368	-9.7684395	1.54E-22
22262	SNRK	1227.78948	0.3871296	0.08416626	4.59958198	4.23E-06

22264	SNRNP200	12918.8837	0.27587192	0.0660653	4.17574575	2.97E-05
22265	SNRNP25	787.41385	-0.4215428	0.1236496	-3.4091724	0.0006516
22267	SNRNP35	794.059054	-0.1974058	0.09159348	-2.155239	0.03114314
22268	SNRNP40	2379.34072	0.46078721	0.06289277	7.32655326	2.36E-13
22269	SNRNP48	1046.18038	0.68599845	0.08055574	8.51582322	1.65E-17
22270	SNRNP70	9781.23488	-0.2924632	0.08343022	-3.5054827	0.00045578
22272	SNRPA1	927.812714	-0.4018039	0.08118531	-4.9492199	7.45E-07
22284	SNTA1	1547.05151	-0.8419847	0.10235693	-8.2259672	1.94E-16
22286	SNTB2	1478.77383	-0.289113	0.0829595	-3.4849898	0.00049216
22290	SNU13	4694.80312	0.26457457	0.0770952	3.43179064	0.00059961
22293	SNW1	4695.41998	0.66494597	0.06151558	10.8093905	3.11E-27
22294	SNX1	2843.54758	-0.805704	0.06177486	-13.042586	7.00E-39
22299	SNX14	2925.77494	-0.4585116	0.06703161	-6.8402294	7.91E-12
22301	SNX16	1083.01671	0.84890398	0.09729823	8.72476264	2.67E-18
22309	SNX24	2583.89511	1.24469138	0.06617433	18.8092767	6.34E-79
22311	SNX27	1100.25573	-0.495801	0.09684969	-5.1192832	3.07E-07
22315	SNX3	5941.63281	-0.5228616	0.07021967	-7.4460848	9.62E-14
22319	SNX33	1082.61	-0.8348665	0.10332985	-8.079626	6.50E-16
22320	SNX4	1116.51893	-0.2223039	0.09226974	-2.4092825	0.01598392
22323	SNX7	2452.81273	-0.8347318	0.07738853	-10.786247	4.00E-27
22324	SNX8	4579.49698	0.20344013	0.07784311	2.6134636	0.00896296
22326	SOAT1	8008.93185	0.43020807	0.05188991	8.29078512	1.13E-16
22329	SOCS1	271.352926	-0.7416619	0.16495083	-4.4962607	6.92E-06
22330	SOCS2	352.007312	2.26039681	0.13057626	17.310933	3.89E-67
22331	SOCS2-AS1	11.6251474	4.39885982	1.00465588	4.37847418	1.20E-05
22332	SOCS3	1020.00701	-0.4762148	0.08465271	-5.6255119	1.85E-08
22333	SOCS4	2915.12252	1.38874858	0.09256524	15.0029167	7.03E-51
22344	SON	14498.3159	0.8260081	0.0623534	13.2472025	4.68E-40
22346	SORBS2	486.951751	-0.3192623	0.10735427	-2.9739135	0.00294028
22347	SORBS3	2935.90849	-0.2944345	0.08427916	-3.4935627	0.00047662
22353	SORL1	1676.01592	-0.8084522	0.07670868	-10.539253	5.69E-26
22354	SORT1	8279.7675	-0.5273983	0.0588429	-8.9628201	3.16E-19
22355	SOS1	1603.08268	0.27635254	0.11121689	2.48480733	0.01296216
22361	SOWAHC	1619.34184	0.88951256	0.08255247	10.7751176	4.51E-27
22367	SOX13	1757.09251	-0.2942333	0.06953562	-4.2314035	2.32E-05
22378	SOX4	88.6585698	-2.5020062	0.26288847	-9.5173677	1.78E-21
22391	SP2-AS1	98.6897378	0.89669671	0.21368865	4.19627675	2.71E-05
22393	SP4	287.949101	0.85876135	0.14593541	5.88453041	3.99E-09
22394	SP5	184.602149	-0.8242432	0.17703718	-4.6557634	3.23E-06
22395	SP6	207.362817	-0.5152289	0.1475785	-3.4912193	0.00048082
22405	SPACA6P	505.684472	-0.6160703	0.12674462	-4.8607215	1.17E-06
22414	SPAG5	4060.42722	-0.6464018	0.06044369	-10.694281	1.08E-26
22417	SPAG7	1368.06883	-0.6114408	0.09473102	-6.4544939	1.09E-10

22419	SPAG9	6672.42266	0.42282572	0.07087173	5.96606998	2.43E-09
22445	SPATA20	3658.43665	-0.4674285	0.08179802	-5.7144231	1.10E-08
22484	SPC24	312.127421	-0.9840796	0.13903883	-7.0777324	1.47E-12
22485	SPC25	1080.40678	-0.7510874	0.10638827	-7.0598702	1.67E-12
22489	SPDEF	62.2423954	-3.4768558	0.33925236	-10.248582	1.20E-24
22503	SPECC1L	1942.10479	0.59807765	0.07849078	7.61971868	2.54E-14
22509	SPEN	4018.64279	1.19178836	0.1140588	10.4488946	1.48E-25
22516	SPG7	2287.52567	-0.4353467	0.07539312	-5.774355	7.72E-09
22518	SPHK1	642.199476	0.86297638	0.16619188	5.19265072	2.07E-07
22519	SPHK2	1038.71534	-0.8719936	0.08910047	-9.7866334	1.29E-22
22525	SPIDR	1819.47389	-0.425116	0.06849083	-6.2069043	5.40E-10
22541	SPINT1	769.195058	-1.6569011	0.09349333	-17.722131	2.83E-70
22545	SPIRE1	4121.44588	0.48415242	0.07746533	6.2499238	4.11E-10
22546	SPIRE2	539.970952	-0.3605985	0.1367327	-2.6372514	0.00835809
22549	SPNS2	122.809976	-2.0821498	0.2133046	-9.7613918	1.65E-22
22553	SPOCK1	96.6184164	2.4391585	0.26287004	9.27895209	1.71E-20
22563	SPPL2B	2123.68066	-0.2775074	0.08791946	-3.1563819	0.0015974
22565	SPPL3	2609.43845	-0.5736104	0.06459455	-8.8801674	6.68E-19
22566	SPR	1629.07121	-0.7162865	0.11721686	-6.1107806	9.91E-10
22569	SPRED3	365.979104	1.15457287	0.13288478	8.68852593	3.67E-18
22583	SPRTN	912.064831	0.22279452	0.09757696	2.28326976	0.02241448
22584	SPRY1	394.555948	-1.0491868	0.11385875	-9.2148103	3.12E-20
22585	SPRY2	2939.62701	0.56722926	0.06804915	8.33558229	7.71E-17
22587	SPRY4	3295.11913	0.82999687	0.07516678	11.0420703	2.39E-28
22592	SPSB1	1161.06415	0.45353407	0.09214985	4.92170189	8.58E-07
22597	SPTAN1	19584.9954	0.37764892	0.05458389	6.91868836	4.56E-12
22598	SPTB	57.018698	-0.9109278	0.31039555	-2.934732	0.00333836
22600	SPTBN2	901.527849	-0.1890831	0.09570781	-1.9756287	0.04819685
22603	SPTLC1	4080.27422	-0.2151079	0.05906704	-3.6417594	0.00027078
22604	SPTLC2	2001.18773	-0.7861226	0.07411281	-10.607108	2.76E-26
22614	SQSTM1	49542.6143	0.76533466	0.07969983	9.602714	7.79E-22
22616	SRBD1	649.570981	0.39767736	0.09412135	4.22515587	2.39E-05
22617	SRC	2961.86423	0.22427889	0.07171127	3.12752619	0.00176284
22619	SRCIN1	46.4930492	2.19243717	0.36099351	6.07334243	1.25E-09
22620	SRD5A1	1224.05168	-0.4722491	0.09731202	-4.852937	1.22E-06
22623	SRD5A3	441.206733	-0.9636608	0.12477629	-7.7231078	1.14E-14
22625	SREBF1	5196.36507	-0.8042859	0.07811298	-10.296444	7.31E-25
22626	SREBF2	6560.84556	-0.3048073	0.06570658	-4.6389162	3.50E-06
22629	SRF	4333.19304	0.71269903	0.06620053	10.7657597	4.99E-27
22630	SRFBP1	1046.34969	1.11559762	0.09772446	11.4157462	3.49E-30
22632	SRGAP2	2286.72822	-0.2200437	0.08564146	-2.569359	0.01018868
22639	SRGN	777.204011	-3.2602319	0.11187265	-29.142351	1.04E-186
22640	SRI	3537.81847	-0.6142735	0.06759952	-9.0869502	1.02E-19

22642	SRM	6570.31699	0.99296856	0.08455646	11.7432609	7.65E-32
22645	SRP14-AS1	38.6255751	1.53647813	0.35039799	4.38495133	1.16E-05
22646	SRP19	2197.80984	0.52044667	0.07596903	6.8507739	7.35E-12
22648	SRP68	6146.91486	0.40959242	0.05751414	7.12159513	1.07E-12
22649	SRP72	6364.2391	0.12623811	0.06312108	1.99993589	0.04550719
22651	SRPK1	11136.5967	0.52733492	0.0639747	8.24286669	1.68E-16
22652	SRPK2	2440.03058	0.53050165	0.07875195	6.7363622	1.62E-11
22654	SRPR	9078.82653	0.63722744	0.07156872	8.90371464	5.40E-19
22655	SRPRB	1841.94436	0.90467157	0.07814156	11.5773424	5.37E-31
22659	SRRD	692.558434	0.47245941	0.10204032	4.63012487	3.65E-06
22674	SRSF5	6875.66253	-0.220397	0.06730426	-3.2746362	0.00105798
22675	SRSF6	8723.1107	-0.2349833	0.05210928	-4.5094316	6.50E-06
22676	SRSF7	4663.41493	-0.4553287	0.07500034	-6.0710221	1.27E-09
22681	SS18	3826.73964	0.19467449	0.08082309	2.40864932	0.01601167
22686	SSBP2	227.909708	-0.6172338	0.18836167	-3.2768544	0.0010497
22694	SSH2	842.027801	-0.4091466	0.09644579	-4.242244	2.21E-05
22695	SSH3	1193.74989	-0.5463836	0.08179481	-6.6799301	2.39E-11
22697	SSNA1	2812.64094	-0.4197141	0.09786676	-4.2886277	1.80E-05
22698	SSPN	146.207786	-0.7721145	0.16935087	-4.5592593	5.13E-06
22701	SSR2	6366.51339	-0.1303082	0.05846514	-2.2288181	0.02582601
22702	SSR3	9017.25532	0.67369828	0.05474165	12.3068682	8.32E-35
22705	SSRP1	14983.5301	0.2284009	0.05565127	4.10414564	4.06E-05
22736	ST3GAL1	2255.36071	1.44465347	0.08495405	17.0051169	7.53E-65
22738	ST3GAL3	588.176655	-0.278123	0.09793515	-2.8398686	0.00451321
22739	ST3GAL4	2685.77702	-0.8187437	0.06889129	-11.884575	1.42E-32
22741	ST3GAL5	636.676469	-1.3090332	0.10424618	-12.557133	3.63E-36
22743	ST3GAL6	598.786472	-0.8691885	0.09796807	-8.8721613	7.17E-19
22745	ST5	2310.12897	-1.3060173	0.06809504	-19.179333	5.51E-82
22749	ST6GALNAC2	71.4869915	-1.6710184	0.26621025	-6.2770626	3.45E-10
22754	ST7	738.678408	-0.246114	0.09090936	-2.7072459	0.0067844
22759	ST7L	508.952708	0.26554735	0.10353834	2.56472485	0.01032577
22760	ST8SIA1	63.2269113	-1.7240175	0.26720507	-6.452039	1.10E-10
22763	ST8SIA4	115.578441	-1.0807764	0.2011458	-5.3730994	7.74E-08
22772	STAG1	1759.71892	0.48180247	0.07624431	6.31919218	2.63E-10
22780	STAG3L5P-P\	260.687876	-0.4897601	0.12856392	-3.8094673	0.00013927
22781	STAM	3127.38456	0.55970258	0.0779428	7.18094007	6.92E-13
22790	STARD13	909.757107	0.40124016	0.08732495	4.59479425	4.33E-06
22792	STARD3	2469.62184	-0.550488	0.07396281	-7.4427682	9.86E-14
22801	STARD9	641.965325	-0.7294282	0.13127869	-5.5563338	2.75E-08
22802	STAT1	5857.93448	-0.2252431	0.06256595	-3.6000906	0.00031811
22803	STAT2	2691.91216	-0.7079484	0.06419011	-11.028933	2.77E-28
22806	STAT5A	1062.35292	-0.8403494	0.08463891	-9.9286419	3.12E-23
22814	STC1	3725.21118	-1.4977843	0.09232227	-16.223434	3.44E-59

22820	STEAP3	3480.42421	-1.1281663	0.06612377	-17.061433	2.87E-65
22824	STIL	1802.78919	0.44590812	0.10662192	4.182143	2.89E-05
22825	STIM1	3797.4655	-0.3355117	0.06400301	-5.2421231	1.59E-07
22827	STIP1	14467.9476	0.46054703	0.08096538	5.68819678	1.28E-08
22829	STK11	5335.25422	0.60741428	0.098828	6.14617609	7.94E-10
22830	STK11IP	683.567856	-0.5847371	0.11638113	-5.0243294	5.05E-07
22831	STK16	1107.98428	-0.5704494	0.08817983	-6.4691599	9.85E-11
22833	STK17B	2473.37412	0.40096016	0.06756813	5.93416124	2.95E-09
22834	STK19	431.366344	0.30928241	0.11334585	2.72866108	0.0063592
22836	STK25	3507.92529	-0.3825503	0.06053672	-6.3193102	2.63E-10
22837	STK26	1162.86931	0.84420545	0.08091787	10.4328673	1.76E-25
22844	STK35	1847.39446	0.87552834	0.06864472	12.7544886	2.94E-37
22845	STK36	591.19509	-0.8205164	0.10348383	-7.9289338	2.21E-15
22848	STK39	2238.20613	-0.256156	0.06966741	-3.6768412	0.00023614
22849	STK4	3907.03424	0.70687426	0.06177495	11.4427331	2.56E-30
22851	STK40	6424.79647	0.80611348	0.05356947	15.0480011	3.56E-51
22855	STMN3	1221.82872	-1.3995195	0.08807099	-15.890811	7.34E-57
22858	STOM	793.490694	0.64801284	0.08932315	7.25470201	4.03E-13
22860	STOML2	6510.10498	0.3851063	0.08677214	4.43813314	9.07E-06
22867	STPG1	882.660389	1.338984	0.11404071	11.7412804	7.83E-32
22873	STRADA	676.701783	-0.636084	0.10592703	-6.0049264	1.91E-09
22874	STRADB	1665.79748	-0.6349182	0.06873333	-9.2374135	2.53E-20
22883	STS	5787.82274	-0.4202439	0.07485567	-5.6140559	1.98E-08
22887	STX10	2871.27005	-0.1941311	0.09056799	-2.1434853	0.03207415
22889	STX12	1280.5953	0.4920578	0.074669	6.58985346	4.40E-11
22894	STX18	1537.65364	0.43948802	0.08238853	5.334335	9.59E-08
22898	STX1A	1074.23866	0.22272208	0.08001657	2.78344938	0.00537843
22901	STX3	7442.77251	0.5226445	0.06043652	8.64782545	5.25E-18
22909	STXBP3	2212.56385	-0.503331	0.07291271	-6.9032005	5.08E-12
22916	STYX	1354.37279	0.40113622	0.08068785	4.9714577	6.65E-07
22921	SUCLG2	2250.35408	-1.0743071	0.07151828	-15.021434	5.31E-51
22924	SUCO	3648.32884	0.22403207	0.07312173	3.06382362	0.00218528
22926	SUFU	789.389012	-1.2689693	0.09998577	-12.691499	6.59E-37
22935	SULT1A1	13.2353693	-2.2902509	0.63489899	-3.6072682	0.00030944
22949	SUMF1	1371.57596	-0.6405823	0.08529466	-7.5102278	5.90E-14
22950	SUMF2	6993.40317	-0.4357599	0.05788333	-7.5282452	5.14E-14
22951	SUMO1	5465.83924	-0.2225059	0.06593031	-3.3748647	0.00073852
22958	SUN2	5827.63789	-0.4220234	0.07449484	-5.6651353	1.47E-08
22959	SUN3	159.663278	0.97093201	0.19415513	5.00080539	5.71E-07
22961	SUOX	229.642896	-1.0274605	0.14403578	-7.1333698	9.79E-13
22967	SUPT4H1	3047.36261	-0.1682441	0.06177917	-2.7233145	0.00646305
22968	SUPT5H	7866.23127	0.33189675	0.05629982	5.89516574	3.74E-09
22975	SURF6	2901.51087	0.54692167	0.10744363	5.09031287	3.57E-07

22976	SUSD1	2042.43591	-0.2992551	0.08020491	-3.7311315	0.00019062
22977	SUSD2	45.1734097	-2.7625653	0.36307854	-7.6087264	2.77E-14
22979	SUSD4	99.4095207	1.99869336	0.22842066	8.7500552	2.13E-18
22980	SUSD5	76.4710392	-3.0639766	0.28324921	-10.817247	2.85E-27
22982	SUV39H1	1435.73342	-0.4258525	0.09890628	-4.3056167	1.67E-05
22983	SUV39H2	1454.67574	0.54060358	0.08247982	6.55437423	5.59E-11
22985	SUV420H2	210.179953	-0.5254795	0.15665506	-3.354373	0.00079545
22996	SVIP	724.397844	0.62850696	0.09156759	6.86385825	6.70E-12
22998	SVOPL	10.200075	-1.7117276	0.68585635	-2.4957524	0.01256903
22999	SWAP70	6287.19774	0.54885133	0.06043315	9.08195813	1.07E-19
23000	SWI5	404.385037	0.26557227	0.11226352	2.36561495	0.01800015
23002	SWT1	265.393972	0.28139214	0.13662719	2.05956177	0.03944045
23003	SYAP1	4855.42029	0.27146538	0.05651529	4.80339713	1.56E-06
23004	SYBU	95.853666	1.91615954	0.22924756	8.35847295	6.35E-17
23011	SYCP2	49.5989673	-1.6597904	0.30844342	-5.3811827	7.40E-08
23012	SYCP2L	158.39081	-0.5960465	0.16249297	-3.668137	0.00024432
23015	SYDE2	391.90985	1.26443067	0.16142558	7.83290162	4.77E-15
23033	SYNGR2	8109.30897	-0.5670058	0.07430627	-7.6306593	2.34E-14
23036	SYNJ1	548.276225	0.54221965	0.10949954	4.95179862	7.35E-07
23037	SYNJ2	8387.61704	-0.1748651	0.06883743	-2.5402616	0.01107696
23041	SYNM	1795.55912	-0.4183513	0.07425949	-5.6336414	1.76E-08
23042	SYNPO	1045.91562	-2.1260183	0.09088788	-23.391658	5.20E-121
23043	SYNPO2	27.8511971	-0.9833647	0.40293025	-2.4405332	0.0146656
23047	SYNRG	1881.0542	0.57902449	0.07584101	7.63471506	2.26E-14
23052	SYS1	2354.9963	0.15939323	0.06509781	2.44851896	0.01434449
23054	SYT1	159.016414	1.49667853	0.17231735	8.68559388	3.77E-18
23057	SYT12	1509.98428	-0.2533902	0.0792635	-3.1968089	0.00138957
23059	SYT14	89.5833497	3.79348763	0.32683573	11.6067104	3.81E-31
23061	SYT15	15.396514	-1.2942114	0.5511329	-2.3482746	0.01886061
23070	SYT8	26.1499498	-2.5633461	0.47503473	-5.3961236	6.81E-08
23072	SYTL1	252.218994	-1.3560185	0.16518716	-8.2089821	2.23E-16
23073	SYTL2	401.026548	0.98151283	0.11477394	8.55170463	1.21E-17
23077	SYVN1	3139.31746	-0.4339151	0.07199914	-6.0266707	1.67E-09
23079	SZT2	1265.90381	0.69108037	0.10691862	6.46361085	1.02E-10
23088	TAB1	911.432896	-0.567917	0.08663727	-6.5551122	5.56E-11
23096	TACC3	7399.57899	-0.2868518	0.07079885	-4.0516453	5.09E-05
23102	TADA1	659.095291	-0.2244908	0.09439468	-2.3782143	0.01739671
23105	TADA3	4713.21127	-0.5247459	0.08090918	-6.4856166	8.84E-11
23109	TAF12	158.209551	0.6798373	0.17112913	3.97265676	7.11E-05
23110	TAF13	1838.33925	1.08050651	0.09278421	11.645371	2.42E-31
23111	TAF15	7020.93151	0.25746806	0.086815	2.96570923	0.00301986
23112	TAF1A	950.094505	1.0427364	0.10444666	9.9834345	1.80E-23
23114	TAF1B	1616.88001	0.68286154	0.07392136	9.23767534	2.52E-20

23116	TAF1D	2096.53398	0.70697186	0.07695737	9.18653825	4.06E-20
23118	TAF2	2295.45242	0.59885549	0.09338214	6.41295552	1.43E-10
23121	TAF4B	506.153944	0.27897914	0.11881467	2.34801932	0.01887354
23123	TAF5L	1433.5094	0.39006996	0.07720059	5.05268094	4.36E-07
23125	TAF6L	839.758546	-0.5391452	0.10003609	-5.3895064	7.07E-08
23137	TALDO1	8397.06987	-0.2816123	0.09426909	-2.987324	0.00281431
23138	TAMM41	364.414894	0.24917214	0.11953599	2.08449485	0.03711518
23139	TANC1	3364.18746	-0.4534814	0.08703796	-5.2101563	1.89E-07
23147	TAP1	4082.10789	0.36488809	0.0637983	5.71940168	1.07E-08
23148	TAP2	6624.64775	0.82625339	0.05887571	14.0338579	9.67E-45
23149	TAPBP	10351.6024	-0.2594591	0.06525788	-3.9759049	7.01E-05
23150	TAPBPL	147.224174	-0.5805578	0.17193193	-3.3766726	0.00073368
23153	TARBP1	1283.80337	-0.5188642	0.0746897	-6.9469314	3.73E-12
23154	TARBP2	695.781262	-0.5163145	0.09788251	-5.2748395	1.33E-07
23159	TARS	33261.1123	0.85476769	0.04998423	17.1007483	1.47E-65
23170	TAS2R19	13.9776284	1.21427672	0.5914725	2.05297242	0.04007525
23184	TAS2R5	41.5694207	1.92438655	0.34159434	5.63354348	1.77E-08
23193	TATDN1	477.902957	0.84671182	0.13934671	6.0762958	1.23E-09
23196	TAX1BP1	9995.01062	0.65966801	0.0594041	11.1047561	1.19E-28
23198	TAZ	1259.54621	-0.2868599	0.08559792	-3.3512485	0.00080448
23200	TBC1D1	3041.86013	-0.3951988	0.06377047	-6.1972075	5.75E-10
23204	TBC1D12	1381.20654	-0.4022597	0.07266212	-5.5360299	3.09E-08
23209	TBC1D17	2185.81593	-0.9473965	0.06880936	-13.768426	3.95E-43
23211	TBC1D2	4049.75329	-0.2151788	0.06437944	-3.3423528	0.00083071
23214	TBC1D22A	927.682572	-0.4774681	0.09479442	-5.0368795	4.73E-07
23216	TBC1D22B	2154.87424	0.79324166	0.08041601	9.8642253	5.95E-23
23217	TBC1D23	3108.89294	0.52434277	0.06845102	7.66011578	1.86E-14
23223	TBC1D2B	1099.86396	-0.898049	0.0978653	-9.176378	4.46E-20
23225	TBC1D30	23.3138306	1.84605488	0.45423265	4.06411755	4.82E-05
23240	TBC1D5	1349.65621	-0.4037676	0.11239564	-3.5923772	0.00032768
23242	TBC1D8	789.264852	-0.7108336	0.0959023	-7.4120596	1.24E-13
23249	TBCCD1	235.496433	-0.4557434	0.14730477	-3.0938808	0.00197557
23250	TBCD	3661.71014	-0.5846246	0.08587702	-6.8076955	9.92E-12
23252	TBCEL	1810.28155	0.23508987	0.10012529	2.34795707	0.0188767
23253	TBCK	256.803607	-0.8743646	0.13413941	-6.5183271	7.11E-11
23257	TBL1XR1	5913.86541	1.3351179	0.05778011	23.1068785	3.95E-118
23259	TBL2	2901.7037	0.29014993	0.06839335	4.24237076	2.21E-05
23262	TBPL1	637.478288	0.43545288	0.10263131	4.24288557	2.21E-05
23278	TBX4	9.11275859	2.01053013	0.83800405	2.39918903	0.01643143
23282	TBXA2R	121.642137	-1.4796492	0.21250072	-6.9630315	3.33E-12
23283	TBXAS1	110.261926	-1.2065358	0.20795714	-5.8018483	6.56E-09
23285	TCAF1	1056.88633	-0.1842951	0.08876868	-2.0761278	0.03788213
23288	TCAIM	1234.87907	0.36663693	0.08298071	4.41833948	9.95E-06

23291	TCEA1	3529.83817	1.2185994	0.06217413	19.5997809	1.55E-85
23292	TCEA2	1622.34221	-0.2983499	0.11999936	-2.4862624	0.01290927
23294	TCEAL1	1083.51716	-0.3893007	0.08430422	-4.6178072	3.88E-06
23304	TCEB1	3210.95487	0.48572048	0.07158362	6.7853581	1.16E-11
23306	TCEB3	2822.63733	0.87907135	0.06970029	12.6121622	1.81E-36
23307	TCEB3-AS1	52.9060077	0.79741481	0.27734995	2.87512147	0.00403872
23317	TCF19	4307.76584	-0.3026997	0.06673789	-4.5356505	5.74E-06
23318	TCF20	2325.24056	0.66157379	0.07329221	9.02652268	1.77E-19
23322	TCF25	2444.24376	-0.9458414	0.08924481	-10.598279	3.04E-26
23329	TCHH	19.5586267	-1.3279975	0.56490882	-2.3508175	0.01873222
23332	TCIRG1	6198.66202	-1.2370089	0.0726392	-17.029494	4.96E-65
23337	TCN2	245.29824	-2.0014285	0.14656066	-13.655973	1.86E-42
23338	TCOF1	6511.62513	0.71160926	0.07377657	9.64546362	5.14E-22
23345	TCP11L1	556.081628	0.42125673	0.11703482	3.59941357	0.00031894
23346	TCP11L2	333.38224	0.65487341	0.14474357	4.52436952	6.06E-06
23354	TCTN1	547.958931	-0.375597	0.09941805	-3.7779563	0.00015812
23355	TCTN2	667.277589	-1.2677858	0.12019008	-10.548174	5.18E-26
23357	TDG	818.076583	0.73582714	0.08999804	8.17603485	2.93E-16
23363	TDP2	2439.48259	0.19896334	0.06987277	2.84750873	0.00440629
23375	TDRP	1560.33926	0.93992811	0.07126708	13.1888125	1.02E-39
23379	TEAD4	498.050554	0.27325428	0.10262497	2.66264912	0.00775282
23381	TECPR1	983.977857	-0.8492946	0.0891715	-9.5242825	1.66E-21
23383	TECR	2986.24867	-0.9520025	0.07656763	-12.433485	1.72E-35
23388	TEF	539.658118	0.82413713	0.11734477	7.02321132	2.17E-12
23397	TELO2	1482.22926	-0.3175152	0.10569953	-3.0039418	0.00266506
23405	TEPP	46.4605789	-0.8611246	0.31415835	-2.7410526	0.00612427
23407	TERF1	392.555152	-0.2684207	0.11105918	-2.4169161	0.01565262
23408	TERF2	1046.37723	0.80183797	0.08798007	9.11385963	7.95E-20
23409	TERF2IP	2169.41038	0.65752381	0.09216804	7.13396753	9.75E-13
23410	TERT	92.2438871	0.69131442	0.23538772	2.93691788	0.00331492
23411	TES	8620.59585	1.2254428	0.05915266	20.7166132	2.45E-95
23414	TESK1	1774.25816	0.62267964	0.07880533	7.90149172	2.76E-15
23417	TET1	78.6977358	-0.9183114	0.24748142	-3.7106278	0.00020675
23420	TET3	1072.39687	1.29246248	0.08726849	14.8101849	1.26E-49
23421	TEX10	4127.03089	0.88923949	0.05664772	15.6977104	1.57E-55
23436	TEX264	3177.56631	-0.303248	0.08538552	-3.5515155	0.00038302
23446	TEX40	77.7959426	-0.6006915	0.28622655	-2.0986577	0.03584709
23458	TFAP2E	323.059117	1.7621119	0.13281451	13.2674649	3.57E-40
23461	TFB2M	1149.62928	0.28119803	0.09401847	2.99088059	0.00278174
23465	TFDP2	1779.09832	-0.2497073	0.07797747	-3.2023001	0.00136335
23474	TFIP11	1509.40686	0.21010585	0.07376224	2.84842002	0.00439369
23475	TFPI	4783.46564	-0.5264632	0.06869807	-7.6634361	1.81E-14
23476	TFPI2	15689.8622	-0.4392623	0.05443348	-8.0697076	7.05E-16

23484	TGFB1	4188.54782	-0.5938237	0.06243427	-9.5111818	1.89E-21
23485	TGFB1I1	1376.04246	-0.7247658	0.0887457	-8.1667713	3.17E-16
23487	TGFB2-AS1	71.3116113	1.14183671	0.26029215	4.38675045	1.15E-05
23489	TGFB3	54.1709716	-1.9611699	0.28714375	-6.829924	8.50E-12
23490	TGFBI	10005.61	-0.6886328	0.05407755	-12.734173	3.82E-37
23496	TGIF1	2795.45287	0.25714093	0.0775156	3.3172796	0.00090899
23497	TGIF2	1172.1453	0.56417803	0.07477003	7.54551015	4.51E-14
23502	TGM2	23537.137	-1.7308725	0.06853334	-25.25592	9.75E-141
23508	TGOLN2	7707.53791	-0.469766	0.0611357	-7.6839881	1.54E-14
23509	TGS1	717.992993	0.46771206	0.09743563	4.80021572	1.58E-06
23513	THAP1	498.037818	0.39469669	0.11012977	3.5839237	0.00033847
23514	THAP10	295.823187	0.34465778	0.14556871	2.36766395	0.01790079
23519	THAP5	1605.17304	0.77774145	0.07545827	10.3069078	6.56E-25
23523	THAP8	232.889697	-0.8187563	0.16331532	-5.0133468	5.35E-07
23525	THAP9-AS1	1760.8169	1.20779681	0.08044327	15.0142683	5.92E-51
23526	THBD	4868.5612	-0.5015314	0.06302149	-7.9581016	1.75E-15
23527	THBS1	28684.4013	0.66188727	0.07551496	8.76498148	1.87E-18
23529	THBS3	435.97713	-1.913483	0.11663008	-16.406428	1.72E-60
23538	THEM6	1732.6664	-0.7346659	0.12548535	-5.8545947	4.78E-09
23542	THNSL1	583.06946	0.22242977	0.09546902	2.32986338	0.01981337
23544	THOC1	2175.67166	0.3063586	0.07148664	4.28553624	1.82E-05
23546	THOC3	555.241195	-0.4697841	0.11616558	-4.0440902	5.25E-05
23551	THOP1	4158.4766	-0.3492672	0.09469199	-3.6884553	0.00022562
23553	THRA	863.057288	-0.2835407	0.10427219	-2.7192358	0.0065433
23555	THRAP3	7161.59354	0.37270862	0.08618849	4.32434307	1.53E-05
23560	THSD1	30.9398577	2.04828909	0.40290291	5.08382794	3.70E-07
23567	THUMPD1	1437.79441	0.20378155	0.07794262	2.61450713	0.00893563
23573	TIA1	1225.79307	-0.3016148	0.08304878	-3.6317793	0.00028147
23576	TIAM1	482.925504	0.66488172	0.12399208	5.362292	8.22E-08
23578	TICAM1	1485.19485	0.4151212	0.07916001	5.2440774	1.57E-07
23580	TICRR	652.24289	-0.3143694	0.11398565	-2.7579735	0.00581609
23584	TIGAR	638.086114	0.62805559	0.10269834	6.1155381	9.62E-10
23586	TIGD2	528.921198	0.33099628	0.10997124	3.00984401	0.00261382
23589	TIGD5	1121.91286	0.20008848	0.09957125	2.0095006	0.04448407
23594	TIMELESS	2301.93501	-0.7548814	0.0687101	-10.986469	4.44E-28
23595	TIMM10	1730.39363	0.84867397	0.11368816	7.46492849	8.33E-14
23596	TIMM10B	480.805782	-0.4573347	0.1064756	-4.2952069	1.75E-05
23598	TIMM17A	4432.44924	0.24835655	0.06610981	3.75672761	0.00017215
23601	TIMM22	1901.68212	0.43247732	0.07754676	5.57698752	2.45E-08
23604	TIMM44	2938.28095	0.61978545	0.07751517	7.99566694	1.29E-15
23606	TIMM8A	819.667356	1.2655684	0.11236152	11.2633612	1.99E-29
23607	TIMM8B	1621.62154	0.35838387	0.09254591	3.87249811	0.00010773
23608	TIMM9	1844.39785	0.76489578	0.08070216	9.47800873	2.59E-21

23610	TIMP1	8393.57565	-0.4730435	0.08822824	-5.3615886	8.25E-08
23611	TIMP2	37654.9731	-0.4923559	0.04955877	-9.9347879	2.94E-23
23615	TINAGL1	12088.6291	1.20916373	0.06668272	18.1330901	1.75E-73
23618	TIPARP	2678.8353	0.60415387	0.07635107	7.91284136	2.52E-15
23620	TIPIN	674.919266	0.47385209	0.09275099	5.10886278	3.24E-07
23622	TIRAP	261.755995	-0.8603811	0.16143218	-5.3296754	9.84E-08
23624	TJAP1	2130.63706	0.5395854	0.06451223	8.36407933	6.06E-17
23627	TJP3	132.102722	-2.6329535	0.20450218	-12.874941	6.23E-38
23630	TKFC	420.159869	-0.6800374	0.11740954	-5.7920114	6.95E-09
23638	TLE1	4676.08342	0.62211914	0.06967453	8.9289318	4.30E-19
23644	TLK2	1493.6227	0.82100712	0.07594489	10.8105649	3.07E-27
23649	TLR1	43.7552643	-1.0777544	0.31723872	-3.3972978	0.00068055
23652	TLR3	193.236256	-0.6254836	0.18128909	-3.4502002	0.00056017
23658	TLR8-AS1	468.951844	0.40399589	0.11664538	3.46345389	0.00053329
23667	TM4SF1	3362.20334	-0.925157	0.06793759	-13.617747	3.14E-42
23678	TM7SF2	499.743975	-1.0156859	0.11341464	-8.955509	3.38E-19
23679	TM7SF3	1865.37877	-0.8639471	0.06753249	-12.793059	1.79E-37
23680	TM9SF1	3475.99629	-0.4794975	0.06454867	-7.4284648	1.10E-13
23682	TM9SF3	11976.2338	-0.2542197	0.05639012	-4.5082316	6.54E-06
23683	TM9SF4	5255.17017	-0.2914302	0.06155373	-4.7345664	2.20E-06
23684	TMA16	1604.09887	0.85697936	0.07698581	11.1316536	8.80E-29
23687	TMBIM4	1381.83068	-0.5667576	0.08934883	-6.3432013	2.25E-10
23694	TMC5	413.103029	-1.3729882	0.12507145	-10.977631	4.90E-28
23695	TMC6	2158.62651	-0.9835409	0.08048516	-12.220152	2.43E-34
23697	TMC8	339.306234	-2.3016957	0.13398712	-17.178486	3.85E-66
23699	TMCC1-AS1	121.518015	1.20618662	0.19954591	6.04465726	1.50E-09
23700	TMCC2	520.187483	-0.7608631	0.09963411	-7.636573	2.23E-14
23708	TMCO6	384.444289	-0.818208	0.10955516	-7.4684577	8.11E-14
23709	TMED1	1760.90761	-0.3011759	0.1029823	-2.9245402	0.00344966
23710	TMED10	7955.93075	-0.226498	0.05998925	-3.7756432	0.0001596
23714	TMED3	2694.59148	-0.2172806	0.0748849	-2.9015279	0.00371348
23725	TMEM101	1453.17514	-0.9984859	0.11797423	-8.4635938	2.59E-17
23727	TMEM104	1710.06376	-0.2958884	0.08592466	-3.443579	0.00057407
23731	TMEM106C	2841.78378	-0.5559195	0.06652062	-8.3571007	6.43E-17
23735	TMEM109	4784.84903	-0.7475358	0.07197381	-10.386219	2.86E-25
23737	TMEM110	1008.94078	-0.3214938	0.08496476	-3.7838482	0.00015442
23740	TMEM115	2785.86791	-0.257644	0.07981056	-3.2281936	0.00124575
23741	TMEM116	1151.61209	0.82608713	0.07740151	10.6727525	1.37E-26
23742	TMEM117	29.288923	-1.0543269	0.37385026	-2.8201851	0.0047996
23744	TMEM120A	858.516731	-0.9410714	0.11713912	-8.0337925	9.45E-16
23751	TMEM127	3702.85797	-0.1467273	0.0625179	-2.3469641	0.01892708
23753	TMEM129	1815.81224	-1.1319617	0.0801622	-14.120892	2.82E-45
23754	TMEM130	17.5574497	-5.2259076	0.93652504	-5.5801045	2.40E-08

23761	TMEM133	385.193017	-0.8169743	0.13044057	-6.2631916	3.77E-10
23762	TMEM134	666.51579	-0.6969919	0.12145879	-5.738505	9.55E-09
23763	TMEM135	768.091611	-0.5225188	0.09718085	-5.3767672	7.58E-08
23764	TMEM136	1065.323	0.22714995	0.09082066	2.5010824	0.01238144
23766	TMEM139	86.7284922	-2.214846	0.26958319	-8.2158164	2.11E-16
23769	TMEM143	236.615646	-1.3840501	0.14938777	-9.2648153	1.95E-20
23772	TMEM147	4000.75415	0.23267152	0.08128758	2.86232557	0.00420545
23774	TMEM14A	962.916857	-0.9504629	0.09712459	-9.7860177	1.29E-22
23789	TMEM161A	1248.2019	-0.4743273	0.11236164	-4.2214346	2.43E-05
23790	TMEM161B	1495.3329	0.47924969	0.07673955	6.24514569	4.23E-10
23793	TMEM164	2124.64078	-0.541651	0.06560968	-8.2556568	1.51E-16
23794	TMEM165	5812.18604	-0.1330777	0.05964575	-2.2311337	0.02567227
23796	TMEM167B	2048.69067	0.48464581	0.07232288	6.70114056	2.07E-11
23798	TMEM169	11.5343373	-2.2133464	0.7076762	-3.1276259	0.00176224
23800	TMEM170A	1111.07281	0.44289589	0.08196146	5.40370888	6.53E-08
23803	TMEM173	1849.65563	-1.3848659	0.0751738	-18.422189	8.72E-76
23805	TMEM175	609.089668	-0.9351342	0.12225393	-7.6491131	2.02E-14
23812	TMEM179B	1572.25146	-0.3294554	0.12804032	-2.5730599	0.01008038
23813	TMEM18	889.014174	-0.6948521	0.11064751	-6.279871	3.39E-10
23815	TMEM181	2022.66447	-0.1905759	0.06409512	-2.9733301	0.00294587
23825	TMEM187	445.549301	-0.2916895	0.12769715	-2.2842288	0.02235809
23826	TMEM189	1181.13321	-0.4550741	0.09193581	-4.9499109	7.42E-07
23828	TMEM19	1382.15496	-0.6641047	0.08494373	-7.8181716	5.36E-15
23830	TMEM191A	7.91257046	-2.0471219	0.75018108	-2.728837	0.00635581
23833	TMEM192	1965.43814	0.47101081	0.07155189	6.58278634	4.62E-11
23836	TMEM198B	224.563562	-1.0131422	0.18474985	-5.4838594	4.16E-08
23842	TMEM201	1603.24437	1.25418963	0.07705917	16.275671	1.47E-59
23847	TMEM206	1199.35195	0.65191211	0.078772	8.27593721	1.27E-16
23850	TMEM209	1562.12619	0.76567451	0.07384943	10.3680491	3.47E-25
23856	TMEM214	2390.26571	-0.8885823	0.08423782	-10.548496	5.16E-26
23860	TMEM218	584.623811	-0.5409453	0.10142794	-5.3332971	9.64E-08
23864	TMEM221	33.1210327	-1.0746663	0.36288365	-2.9614624	0.00306182
23865	TMEM222	1290.53096	0.4432027	0.08875265	4.99368421	5.92E-07
23866	TMEM223	450.884233	-1.0374562	0.12428295	-8.3475342	6.97E-17
23869	TMEM229B	275.775951	-0.3680084	0.1286086	-2.8614605	0.00421694
23870	TMEM230	3499.94078	-0.1863947	0.07690699	-2.4236383	0.0153659
23871	TMEM231	344.307704	0.28847812	0.14422561	2.00018644	0.04548014
23876	TMEM236	41.137532	0.78541462	0.31777901	2.47157488	0.01345194
23877	TMEM237	1374.38569	-0.7583398	0.07094632	-10.688924	1.15E-26
23882	TMEM242	219.613491	-0.7694068	0.16292442	-4.7224765	2.33E-06
23885	TMEM245	7790.37048	-0.3806758	0.06642211	-5.7311607	9.97E-09
23889	TMEM248	6161.01701	-0.4424804	0.06053382	-7.3096404	2.68E-13
23891	TMEM25	1078.72167	-0.5301804	0.08682447	-6.1063474	1.02E-09

23898	TMEM255B	398.299893	-0.2327482	0.10825962	-2.1499074	0.03156254
23902	TMEM258	1329.76277	-0.6256589	0.10837126	-5.7732914	7.77E-09
23907	TMEM261	395.59554	0.38679959	0.14312488	2.70253209	0.00688135
23911	TMEM27	38.0943158	1.20105517	0.36897242	3.25513537	0.00113338
23918	TMEM37	80.5194518	-1.4440606	0.24980184	-5.7808244	7.43E-09
23920	TMEM38B	2156.3772	0.77848349	0.07373136	10.5583771	4.65E-26
23928	TMEM44	962.520769	-0.5278274	0.09888504	-5.3377885	9.41E-08
23931	TMEM45B	354.525199	1.329153	0.12165112	10.9259411	8.66E-28
23933	TMEM5	579.378912	0.59284631	0.0981808	6.0383119	1.56E-09
23935	TMEM50A	5358.04289	0.38422794	0.0636977	6.03205394	1.62E-09
23936	TMEM50B	800.561567	-0.6323206	0.08363963	-7.56006	4.03E-14
23937	TMEM51	1384.20783	0.56630116	0.09228642	6.13634372	8.44E-10
23941	TMEM53	164.456349	-0.8065585	0.16426316	-4.9101605	9.10E-07
23943	TMEM55A	1178.13407	-0.9521676	0.09191055	-10.35972	3.78E-25
23944	TMEM55B	2141.92502	0.44447025	0.07081704	6.27631817	3.47E-10
23945	TMEM56	1520.90951	-0.4164492	0.08333815	-4.997102	5.82E-07
23947	TMEM57	1140.39364	0.95177111	0.09556352	9.95956515	2.29E-23
23950	TMEM60	1024.26824	-0.3733077	0.09459407	-3.9464175	7.93E-05
23951	TMEM61	8.88420128	-2.8007227	0.98236575	-2.8509979	0.00435823
23952	TMEM62	753.672475	-0.5204897	0.08606551	-6.0475998	1.47E-09
23956	TMEM64	2643.01306	0.49693179	0.06826819	7.27911154	3.36E-13
23960	TMEM69	2688.59783	1.28361575	0.07080309	18.129376	1.87E-73
23968	TMEM79	205.588648	0.78324801	0.1699649	4.60829268	4.06E-06
23969	TMEM80	562.035705	-0.6876816	0.10913563	-6.3011649	2.95E-10
23980	TMEM8B	312.491319	-1.2155226	0.13748245	-8.841293	9.46E-19
23983	TMEM91	127.158899	-0.6166488	0.23016202	-2.6791945	0.00737995
23984	TMEM92	873.09867	-0.2261106	0.08618552	-2.623534	0.00870227
23987	TMEM97	1540.20148	-0.8575268	0.09167817	-9.3536634	8.47E-21
23991	TMEM9B-AS	44.4669408	1.27371826	0.34547922	3.68681589	0.00022708
23993	TMIE	51.3089427	0.88735866	0.27583222	3.21702317	0.00129528
23997	TMLHE	2.55221017	4.69861842	1.63573343	2.87248419	0.00407258
24000	TMOD2	248.479835	-0.3516778	0.14956293	-2.3513701	0.01870442
24001	TMOD3	6420.49761	0.84724762	0.07190746	11.7824713	4.81E-32
24004	TMPO-AS1	194.204826	-0.9528329	0.14991117	-6.3559831	2.07E-10
24023	TMPRSS9	28.743937	-2.2694952	0.40829187	-5.5585119	2.72E-08
24027	TMSB4X	29052.0201	-0.7933727	0.09725042	-8.1580388	3.41E-16
24032	TMTC4	611.943551	-0.2414332	0.09918005	-2.4342924	0.01492094
24034	TMUB2	1849.42673	-0.3625906	0.0696081	-5.2090281	1.90E-07
24036	TMX2	3300.52245	0.15893374	0.07445118	2.13473759	0.03278245
24038	TMX3	3433.56358	0.79737991	0.06199848	12.8612825	7.43E-38
24039	TMX4	1225.10166	-0.9970014	0.08184299	-12.18188	3.88E-34
24041	TNF	78.1160407	2.29152775	0.25938502	8.83446441	1.01E-18
24042	TNFAIP1	2702.28521	-0.4005425	0.0679439	-5.8951947	3.74E-09

24043	TNFAIP2	15156.0688	-0.6151631	0.06469896	-9.5080842	1.94E-21
24046	TNFAIP8	705.416417	-0.2996157	0.08875184	-3.3758821	0.00073579
24047	TNFAIP8L1	866.119649	-1.310807	0.09452744	-13.866948	1.00E-43
24050	TNFAIP8L3	45.9803284	-0.9145854	0.30948024	-2.9552305	0.00312436
24051	TNFRSF10A	1517.81759	1.6602227	0.08593604	19.3192827	3.70E-83
24052	TNFRSF10B	7384.24458	0.57401918	0.0578259	9.92667918	3.19E-23
24055	TNFRSF11A	255.292087	-0.9448503	0.13957169	-6.7696413	1.29E-11
24057	TNFRSF12A	4995.3919	0.15860384	0.0614225	2.58217825	0.00981789
24060	TNFRSF14	654.168129	-0.477537	0.12399418	-3.8512858	0.0001175
24062	TNFRSF18	20.961176	-1.0563657	0.42732641	-2.4720346	0.01343465
24063	TNFRSF19	212.505473	-0.7049013	0.14753733	-4.7777828	1.77E-06
24064	TNFRSF1A	4404.05812	-0.1346505	0.05947411	-2.2640185	0.02357298
24066	TNFRSF21	6759.14217	-0.3617926	0.05950588	-6.0799468	1.20E-09
24071	TNFRSF9	412.74453	0.37115022	0.1304773	2.84455792	0.00444731
24074	TNFSF12	482.007759	-1.0625584	0.12230983	-8.6874325	3.71E-18
24077	TNFSF13B	8.70998938	-1.936568	0.73284446	-2.6425362	0.00822877
24087	TNIP3	149.722175	-1.3991471	0.17129212	-8.1681931	3.13E-16
24090	TNKS	1265.91401	0.5844899	0.09825917	5.94845143	2.71E-09
24102	TNNT1	4246.29405	-0.2394574	0.10155284	-2.3579586	0.01837574
24107	TNPO1	10002.9734	0.40792464	0.08502982	4.79743024	1.61E-06
24109	TNPO3	4533.77108	0.61804335	0.06467832	9.55564864	1.23E-21
24114	TNRC6B	1014.29574	-0.3357086	0.10594625	-3.1686687	0.00153139
24115	TNRC6C	793.398439	0.47095527	0.08589247	5.48307974	4.18E-08
24117	TNS1	186.659188	-1.6644533	0.16927118	-9.8330579	8.11E-23
24118	TNS2	977.586637	-1.6776547	0.10887423	-15.409107	1.42E-53
24119	TNS3	7821.09061	-0.497339	0.07179923	-6.9268024	4.30E-12
24120	TNS4	213.038145	-1.9149816	0.16067109	-11.918645	9.46E-33
24125	TOB2	1483.69789	0.79463192	0.08758701	9.07248603	1.16E-19
24127	TOE1	813.77957	0.75381588	0.08458444	8.91199234	5.01E-19
24128	TOLLIP	4983.81801	0.33158789	0.07267574	4.56256647	5.05E-06
24131	TOM1L1	1427.62525	0.56763506	0.07315494	7.75935372	8.54E-15
24136	TOMM34	4685.39281	0.58135421	0.0554736	10.4798355	1.07E-25
24139	TOMM5	4224.35948	0.57409459	0.10653348	5.38886566	7.09E-08
24140	TOMM6	7888.55213	0.27001438	0.08081833	3.34100425	0.00083476
24142	TOMM70A	5311.60817	0.40560364	0.05486713	7.39247118	1.44E-13
24143	TONSL	2678.57434	-0.4613342	0.07562581	-6.1002219	1.06E-09
24145	TOP1	7948.47155	0.30324311	0.05802031	5.22649948	1.73E-07
24149	TOP2A	18028.4493	-0.34174	0.05388537	-6.3419812	2.27E-10
24150	TOP2B	4929.89127	-0.4033143	0.05712134	-7.0606591	1.66E-12
24151	TOP3A	2301.96833	1.01521707	0.08805675	11.5291227	9.41E-31
24155	TOPORS	1585.71628	0.2078853	0.09572271	2.17174469	0.02987493
24158	TOR1AIP1	2540.43874	-0.349239	0.07127139	-4.9001288	9.58E-07
24164	TOX	189.848569	0.54650243	0.16384439	3.33549675	0.00085147

24167	TOX4	2576.78044	0.24548128	0.06353971	3.86343107	0.00011181
24168	TP53	3361.08039	-0.7943427	0.06982026	-11.376966	5.45E-30
24169	TP53AIP1	33.7068972	2.26055685	0.40363376	5.60051482	2.14E-08
24171	TP53BP2	3798.96401	0.86674338	0.07347363	11.7966589	4.06E-32
24173	TP53I13	1414.71108	-0.3565281	0.12927773	-2.7578458	0.00581836
24177	TP53RK	1598.18582	0.53738077	0.0835442	6.43229278	1.26E-10
24184	TP63	31.0443373	-1.7473656	0.37998181	-4.5985507	4.25E-06
24186	TP73-AS1	1115.14112	-0.4902847	0.08048087	-6.0919417	1.12E-09
24187	TPBG	5537.8071	1.10118757	0.07011642	15.7051318	1.39E-55
24189	TPCN1	3906.10686	-0.5367413	0.06234052	-8.6098303	7.32E-18
24192	TPD52L1	869.353114	-0.3016486	0.09448335	-3.1926115	0.00140993
24193	TPD52L2	11791.0897	0.41851059	0.0570679	7.33355529	2.24E-13
24195	TPGS1	676.031474	-1.1633981	0.15915123	-7.3100163	2.67E-13
24202	TPK1	264.614712	-1.0660316	0.14347645	-7.4300108	1.09E-13
24203	TPM1	6500.89789	-0.3331866	0.09554147	-3.4873509	0.00048783
24204	TPM2	2887.92605	-0.3113017	0.13709698	-2.2706676	0.02316711
24211	TPP2	1973.43965	0.27457057	0.08438237	3.25388538	0.00113838
24220	TPRG1L	1938.81927	0.26914334	0.07421991	3.62629559	0.00028752
24222	TPRN	808.292198	-0.2818657	0.1186207	-2.3761933	0.0174923
24229	TPST1	1247.39659	-0.2505032	0.08017087	-3.1246165	0.00178037
24241	TRA2A	2144.1901	-0.5162802	0.07832357	-6.5916328	4.35E-11
24242	TRA2B	5205.83779	-0.2300272	0.05781609	-3.9786013	6.93E-05
24244	TRABD2A	233.576377	0.92134319	0.13912042	6.62263105	3.53E-11
24246	TRADD	555.923046	-0.8379673	0.12427567	-6.7428106	1.55E-11
24247	TRAF1	2156.35778	0.60145568	0.06652879	9.04053283	1.56E-19
24248	TRAF2	3026.26042	0.4727586	0.10532376	4.48862274	7.17E-06
24254	TRAF4	3225.98953	0.77973485	0.08212207	9.49482712	2.21E-21
24256	TRAF6	786.203791	0.49400432	0.10984393	4.49732934	6.88E-06
24258	TRAFD1	1429.52272	-0.3162175	0.07199373	-4.3922921	1.12E-05
24260	TRAK1	3679.5051	-0.1966484	0.06837805	-2.8759002	0.00402877
24261	TRAK2	2955.51224	-0.3086696	0.09196936	-3.3562215	0.00079015
24264	TRAM2	13751.4901	0.53799344	0.0691447	7.78068907	7.21E-15
24265	TRAM2-AS1	444.115141	0.22433949	0.10337611	2.17012901	0.02999707
24266	TRANK1	564.879612	-1.2751824	0.12873398	-9.905562	3.94E-23
24268	TRAPPC1	3274.9313	-0.6560278	0.07029379	-9.3326566	1.03E-20
24269	TRAPPC10	1364.81477	0.80236055	0.12207703	6.57257597	4.95E-11
24271	TRAPPC12	697.284442	-1.1756133	0.09473186	-12.409904	2.31E-35
24274	TRAPPC2B	300.827244	-0.3593053	0.12632324	-2.8443321	0.00445046
24276	TRAPPC3	3126.81341	0.77622729	0.08020601	9.67791886	3.74E-22
24279	TRAPPC5	1035.09126	-0.6547818	0.11572401	-5.6581331	1.53E-08
24282	TRAPPC8	2972.22143	0.2944596	0.08739668	3.36923092	0.00075378
24283	TRAPPC9	546.556417	-0.8580804	0.11145637	-7.6988015	1.37E-14
24291	TREML2	3.97689135	4.32780022	1.46587199	2.95237254	0.00315342

24297	TREX1	342.377185	-1.1841991	0.13916309	-8.5094341	1.75E-17
24304	TRIAP1	1676.41046	0.83209172	0.10965182	7.58849006	3.24E-14
24305	TRIB1	505.508305	0.34483895	0.10759495	3.20497352	0.00135075
24306	TRIB2	3765.24427	-0.1681799	0.05916212	-2.8426953	0.00447338
24307	TRIB3	2411.21148	1.22439747	0.09060922	13.5129458	1.31E-41
24310	TRIM11	1930.67354	0.7518289	0.10299355	7.29976675	2.88E-13
24312	TRIM14	1159.41026	-0.5041113	0.08805883	-5.7247102	1.04E-08
24319	TRIM22	151.346733	-0.518719	0.18274373	-2.8385048	0.00453254
24321	TRIM24	3104.47497	0.39418668	0.06379757	6.17870988	6.46E-10
24325	TRIM28	16069.4249	-0.1783496	0.08564205	-2.0825008	0.03729675
24331	TRIM33	3912.86686	0.31413458	0.0836377	3.75589704	0.00017272
24333	TRIM35	899.090145	0.16638386	0.08371377	1.98753278	0.04686339
24334	TRIM36	447.214419	0.95673944	0.12762984	7.49620501	6.57E-14
24337	TRIM39	932.604008	0.52170706	0.08539273	6.10950196	9.99E-10
24348	TRIM47	1620.68016	-0.7796154	0.08142109	-9.5751037	1.02E-21
24359	TRIM52	136.568762	-0.3558533	0.17340924	-2.0521009	0.04015985
24387	TRIM8	10096.5484	-0.7422841	0.051813	-14.326214	1.50E-46
24391	TRIO	17367.7262	0.81308452	0.10057353	8.08447807	6.24E-16
24392	TRIOBP	3333.50804	-0.502352	0.06343707	-7.9189032	2.40E-15
24395	TRIP12	8060.0838	0.42920936	0.06801971	6.31007354	2.79E-10
24399	TRIQK	1075.78257	-0.2438707	0.07993686	-3.0507916	0.00228239
24400	TRIT1	1058.91066	0.92494789	0.08511835	10.8666091	1.66E-27
24401	TRMT1	2273.19367	0.75609273	0.07510232	10.0675022	7.69E-24
24402	TRMT10A	333.090944	0.58130447	0.14586341	3.98526578	6.74E-05
24405	TRMT11	1059.92637	0.42209672	0.09079224	4.64903937	3.33E-06
24408	TRMT13	663.344462	0.87825989	0.10334714	8.49815378	1.93E-17
24410	TRMT2A	1312.79698	-0.5459887	0.08160078	-6.6909733	2.22E-11
24411	TRMT2B	880.243986	-0.4808426	0.08980476	-5.3543109	8.59E-08
24412	TRMT44	443.314167	0.40790072	0.1067154	3.82232273	0.0001322
24414	TRMT6	1752.50016	0.3688989	0.08618452	4.280338	1.87E-05
24415	TRMT61A	1512.70738	1.10423235	0.08800606	12.5472301	4.12E-36
24418	TRNAU1AP	790.703172	0.54838418	0.08358444	6.56084073	5.35E-11
24419	TRNP1	230.713539	2.2832851	0.16830603	13.5662702	6.35E-42
24420	TRNT1	888.574957	0.82061997	0.09394098	8.73548477	2.43E-18
24422	TROAP	1045.3045	-0.8988824	0.08070874	-11.137361	8.25E-29
24423	TROVE2	2094.46716	1.14319717	0.06815192	16.7742472	3.77E-63
24438	TRPM4	225.161459	-1.1327332	0.14924652	-7.5896793	3.21E-14
24444	TRPT1	864.867994	-0.6945409	0.12068538	-5.7549716	8.67E-09
24445	TRPV1	337.140624	-0.3497902	0.12601398	-2.7758049	0.00550652
24453	TRUB2	1321.67996	-0.8693391	0.08485854	-10.244567	1.25E-24
24455	TSACC	47.6696979	1.00038159	0.32526889	3.07555269	0.00210113
24457	TSC2	2231.00226	-0.6429208	0.07272769	-8.84011	9.56E-19
24458	TSC22D1	7742.19297	1.1475908	0.06227288	18.4284197	7.77E-76

24460	TSC22D2	1564.51289	0.88560037	0.0826454	10.715665	8.59E-27
24461	TSC22D3	1237.70476	1.49660081	0.09838505	15.2116688	2.96E-52
24462	TSC22D4	6080.23259	-0.5885483	0.06413014	-9.1774058	4.42E-20
24463	TSEN15	2815.622	0.4959888	0.06462313	7.67509755	1.65E-14
24469	TSG101	3058.31937	-0.578967	0.0711816	-8.1336606	4.17E-16
24470	TSGA10	201.364009	0.46904549	0.15304696	3.06471614	0.00217877
24475	TSHZ1	278.387756	-0.5475318	0.13709435	-3.9938323	6.50E-05
24480	TSKU	1453.66625	-0.2901919	0.07124605	-4.0730943	4.64E-05
24488	TSPAN1	54.9850032	-2.6457252	0.33239752	-7.9595215	1.73E-15
24494	TSPAN15	978.060665	-2.0668592	0.09383304	-22.026988	1.59E-107
24499	TSPAN2	469.428838	-0.3788867	0.10415205	-3.637823	0.00027495
24509	TSPAN9	2110.65433	-0.2457116	0.07066785	-3.4769931	0.00050707
24513	TSPO	4423.17625	-0.7184691	0.104926	-6.8473883	7.52E-12
24522	TSPYL1	2560.55247	-0.2748024	0.06778833	-4.0538312	5.04E-05
24527	TSR1	7458.6222	0.57973291	0.05392478	10.75077	5.88E-27
24528	TSR2	2515.37042	-0.4061092	0.09191303	-4.418407	9.94E-06
24532	TSSC4	2324.51016	0.90110058	0.11004713	8.18831505	2.65E-16
24537	TSSK6	108.179561	0.81327401	0.2379969	3.41716217	0.00063278
24538	TST	907.357144	-0.2983779	0.14453336	-2.0644222	0.0389777
24540	TSTD1	251.559297	-0.7304815	0.21487561	-3.3995554	0.00067496
24541	TSTD2	1345.22198	0.52607251	0.07901025	6.65828214	2.77E-11
24545	TTC1	3150.24208	-0.5864168	0.069361	-8.4545611	2.80E-17
24546	TTC12	1298.32091	-0.3778733	0.08105017	-4.6622141	3.13E-06
24547	TTC13	1633.67825	-0.1721592	0.06886404	-2.4999872	0.01241978
24566	TTC30A	278.088817	-1.085195	0.13137877	-8.2600488	1.46E-16
24567	TTC30B	118.383407	-1.021483	0.19909185	-5.1307123	2.89E-07
24568	TTC31	553.569894	-1.1304988	0.10396846	-10.873478	1.54E-27
24570	TTC33	1460.40766	0.65749979	0.0802617	8.19194914	2.57E-16
24574	TTC38	1908.89674	-0.3152772	0.07356122	-4.2859159	1.82E-05
24577	TTC39B	121.40542	0.64447993	0.20141592	3.19974671	0.00137548
24583	TTC5	249.595502	-0.8193019	0.16989219	-4.8224813	1.42E-06
24585	TTC7A	1954.95212	-0.5171931	0.07906644	-6.5412472	6.10E-11
24588	TTC9	184.346292	-0.8965433	0.16703303	-5.367461	7.99E-08
24592	TTF2	1468.89852	0.68594762	0.09537927	7.19178958	6.39E-13
24597	TTLL1	173.320386	-0.8590209	0.2065432	-4.1590376	3.20E-05
24599	TTLL11	172.524846	0.43535824	0.15622124	2.78680572	0.00532304
24652	TTYH3	4864.63892	-0.3404653	0.06999651	-4.8640328	1.15E-06
24653	TUB	222.681838	-1.1884556	0.15460985	-7.6868038	1.51E-14
24655	TUBA1A	1766.40206	-1.0784034	0.07480891	-14.41544	4.14E-47
24657	TUBA1C	17729.7001	0.16111014	0.06741678	2.38976303	0.01685925
24670	TUBB3	1681.1186	-0.6821808	0.09305871	-7.330649	2.29E-13
24672	TUBB4B	24589.251	-0.2665147	0.06114595	-4.3586643	1.31E-05
24673	TUBB6	10726.4009	-0.3325557	0.07441395	-4.4689967	7.86E-06

24677	TUBE1	464.475562	0.8400757	0.1201082	6.9943244	2.67E-12
24680	TUBGCP2	4311.48464	-1.2219996	0.05731841	-21.319497	7.49E-101
24681	TUBGCP3	1400.63246	-0.4803086	0.0867681	-5.5355439	3.10E-08
24683	TUBGCP5	620.540222	-0.7487835	0.10085662	-7.4242373	1.13E-13
24684	TUBGCP6	964.52727	-1.1128045	0.12329857	-9.0252829	1.79E-19
24686	TUFT1	2503.33271	0.65552916	0.07040162	9.31128009	1.26E-20
24690	TULP3	805.71899	0.86365193	0.11284434	7.65348036	1.96E-14
24691	TULP4	884.381876	0.44569643	0.10707232	4.16257392	3.15E-05
24693	TUSC1	542.941973	-0.2984085	0.13145925	-2.2699699	0.02320941
24701	TVP23B	1165.42154	1.17260719	0.09173648	12.7823428	2.06E-37
24708	TWISTNB	2464.40785	0.7827819	0.06810377	11.4939586	1.41E-30
24709	TWSG1	7070.21612	-0.1594563	0.07214091	-2.2103454	0.0270812
24711	TXLNA	4319.54679	0.51217691	0.06467096	7.91973629	2.38E-15
24713	TXLNG	2868.3984	0.7931667	0.07822263	10.1398621	3.68E-24
24716	TXN2	2033.47019	-0.2630917	0.10663513	-2.4672137	0.01361691
24718	TXNDC12	2320.72094	0.55697792	0.07018248	7.9361385	2.09E-15
24720	TXNDC15	1633.67612	-0.489807	0.0814486	-6.0136943	1.81E-09
24726	TXNDC9	2731.03577	0.42747981	0.06490631	6.58610579	4.52E-11
24727	TXNIP	15221.2647	1.75682034	0.05157689	34.0621617	2.68E-254
24730	TXNL4B	505.078668	1.06620142	0.10364045	10.2875031	8.02E-25
24733	TXNRD3	305.877995	-1.1043517	0.13004698	-8.4919441	2.03E-17
24735	TYK2	2759.40263	-0.6892953	0.06982576	-9.8716476	5.52E-23
24737	TYMS	10171.4769	-0.3658136	0.05712449	-6.4037955	1.52E-10
24744	TYSND1	1243.70722	-1.006027	0.11236002	-8.9536025	3.44E-19
24747	TYW3	1238.56412	0.61185029	0.08473481	7.22076691	5.17E-13
24748	TYW5	628.507121	0.41644032	0.09557208	4.35734299	1.32E-05
24752	U2SURP	3956.07825	0.34561617	0.06618439	5.22201928	1.77E-07
24753	UACA	8138.60269	-0.3491603	0.07890637	-4.4249955	9.64E-06
24754	UAP1	8497.74394	0.92851195	0.05609277	16.5531478	1.52E-61
24760	UBA52	10454.5943	-0.3114994	0.08785017	-3.5458027	0.00039142
24761	UBA6	6119.58739	0.6006007	0.06421547	9.35289737	8.53E-21
24762	UBA6-AS1	376.314523	0.6628168	0.14130247	4.69076576	2.72E-06
24763	UBA7	813.140255	-1.3452011	0.10877831	-12.366445	3.97E-35
24764	UBAC1	1905.78599	-0.267405	0.09184776	-2.9113936	0.0035982
24769	UBAP1	3979.29267	0.77197258	0.07273886	10.6129322	2.59E-26
24776	UBC	33090.8084	0.85378561	0.06161345	13.8571314	1.15E-43
24779	UBE2B	4342.33828	0.44810842	0.06924983	6.47089561	9.74E-11
24782	UBE2D2	7282.09009	0.5835479	0.06279073	9.29353575	1.49E-20
24786	UBE2E1	5838.57689	0.20779208	0.07716442	2.69284824	0.00708445
24789	UBE2E3	1957.41923	-0.5076837	0.07013324	-7.2388455	4.53E-13
24791	UBE2F	1321.06734	0.63194089	0.09649486	6.54895913	5.79E-11
24793	UBE2G1	2712.64503	0.67511674	0.07375269	9.1537919	5.50E-20
24794	UBE2G2	3032.92566	0.16605459	0.06951368	2.3888044	0.0169033

24795	UBE2H	13849.3702	0.21509034	0.05411343	3.97480534	7.04E-05
24798	UBE2J2	3546.96942	1.14905448	0.07971789	14.4140095	4.22E-47
24800	UBE2L3	4328.85216	0.21483173	0.05574207	3.85403175	0.00011619
24802	UBE2M	4763.75952	0.20836873	0.06256458	3.33045849	0.00086703
24807	UBE2Q1	4027.36317	0.21224248	0.06423837	3.30398281	0.00095322
24811	UBE2Q2P1	54.2580257	-1.0383043	0.27198545	-3.8174993	0.00013481
24816	UBE2T	1678.06559	-0.2652533	0.0762059	-3.4807456	0.00050002
24819	UBE2V2	2242.22181	0.31399569	0.06595576	4.76070133	1.93E-06
24820	UBE2W	1211.20823	0.1854908	0.07684327	2.41388473	0.01578345
24821	UBE2Z	8334.51572	0.25008362	0.06395804	3.91011995	9.23E-05
24829	UBIAD1	1373.80607	1.13600926	0.09505621	11.9509211	6.42E-33
24839	UBOX5	534.936299	-0.5260145	0.10878301	-4.8354475	1.33E-06
24841	UBP1	4171.55042	0.29975766	0.05926122	5.0582429	4.23E-07
24842	UBQLN1	9673.3745	0.32468011	0.07043312	4.60976463	4.03E-06
24843	UBQLN2	1928.31304	-0.2530013	0.08495909	-2.9779196	0.00290212
24848	UBR2	3606.8348	0.29397351	0.08372459	3.51119672	0.00044609
24850	UBR4	14223.7437	0.96123441	0.06587919	14.5908652	3.21E-48
24851	UBR5	6375.73623	0.60369206	0.0692522	8.71729758	2.85E-18
24854	UBTD1	1654.27497	-0.4818606	0.10812864	-4.4563645	8.34E-06
24868	UCA1	18381.534	0.59520259	0.12184816	4.8847895	1.04E-06
24871	UCHL3	1974.46963	0.58216681	0.08012283	7.2659292	3.70E-13
24872	UCHL5	3484.27025	0.52816445	0.07359979	7.17616759	7.17E-13
24874	UCK2	3327.82919	0.27845904	0.06289308	4.42749878	9.53E-06
24876	UCKL1-AS1	105.550478	3.68431483	0.28264962	13.0349186	7.74E-39
24884	UEVLD	773.464237	0.41852715	0.14072354	2.97410907	0.00293841
24888	UFM1	3605.0306	0.86386699	0.06395046	13.5083777	1.40E-41
24889	UFSP1	76.5423903	0.57535851	0.22903652	2.51208199	0.01200212
24897	UGP2	3688.46346	-0.4305646	0.06231373	-6.9096266	4.86E-12
24922	UHRF1BP1	1616.49447	0.30875476	0.09707319	3.18063897	0.00146951
24923	UHRF1BP1L	1569.75573	0.23924415	0.08529502	2.80490163	0.00503319
24930	ULK2	666.998024	-0.3752009	0.09531117	-3.9365889	8.26E-05
24932	ULK4	156.932504	0.65305821	0.16782674	3.89126437	9.97E-05
24936	UMAD1	638.122184	-0.4983547	0.09416956	-5.2920994	1.21E-07
24941	UNC119	784.811403	-0.815589	0.11090793	-7.3537481	1.93E-13
24946	UNC13D	8839.20191	-0.712516	0.07307928	-9.7499037	1.85E-22
24947	UNC45A	3107.80113	-0.601184	0.09156049	-6.5659763	5.17E-11
24959	UNC93B1	2173.83001	-1.0006788	0.0647799	-15.447366	7.86E-54
24967	UPF1	8208.83642	0.53666383	0.06013505	8.9243102	4.48E-19
24968	UPF2	2611.02961	0.35670777	0.07275107	4.90312725	9.43E-07
24970	UPF3B	2093.42463	1.01234994	0.07808522	12.9646809	1.94E-38
24978	UPP1	7965.47008	1.84238913	0.10099729	18.2419655	2.40E-74
24983	UQCC3	1150.79658	-0.6813148	0.25799574	-2.6407988	0.00827108
24984	UQCR10	1893.97239	-0.4915062	0.08139944	-6.0382015	1.56E-09

24988	UQCRC1	6476.03901	-0.4372676	0.07349619	-5.949527	2.69E-09
24990	UQCRFS1	3293.09807	-0.1766138	0.08880708	-1.9887354	0.04673042
24991	UQCRH	4808.16419	0.63587535	0.08740813	7.27478518	3.47E-13
24996	URB1	2004.28512	0.69110113	0.09603352	7.19645739	6.18E-13
24998	URB2	1828.34107	1.11896153	0.07404122	15.1126844	1.34E-51
24999	URGCP	3486.8196	0.79363761	0.0666831	11.9016307	1.16E-32
25004	UROD	3606.61436	0.41890166	0.09388844	4.4616959	8.13E-06
25005	UROS	2592.68207	-0.8232497	0.08395749	-9.8055541	1.07E-22
25008	USF1	1385.09597	-0.3770917	0.0782136	-4.8213063	1.43E-06
25014	USMG5	3925.25251	-0.7320637	0.09179362	-7.9751047	1.52E-15
25016	USP1	7680.76557	1.08040501	0.05624278	19.2096645	3.07E-82
25017	USP10	3752.34952	0.69691091	0.06056135	11.5075195	1.21E-30
25018	USP11	3356.71092	-0.1466643	0.05967253	-2.4578193	0.01397835
25023	USP14	8392.15711	0.44461874	0.05750288	7.73211303	1.06E-14
25024	USP15	2245.69322	0.3771703	0.06620513	5.69699503	1.22E-08
25058	USP21	1088.78322	-0.4204292	0.07963374	-5.279536	1.30E-07
25060	USP24	5637.34142	1.26038503	0.08144369	15.4755385	5.07E-54
25061	USP25	1846.5277	1.0302587	0.08321781	12.3802665	3.34E-35
25067	USP3	1344.04711	0.50873008	0.08983424	5.66298643	1.49E-08
25071	USP31	1122.39177	1.09852007	0.08465955	12.9757368	1.68E-38
25072	USP32	3883.64556	0.53959232	0.08266572	6.5274009	6.69E-11
25074	USP32P2	276.626766	-0.7420871	0.14823168	-5.0062648	5.55E-07
25075	USP33	3315.75932	0.83476067	0.07723734	10.8077351	3.16E-27
25077	USP35	614.834728	-0.2224585	0.09625033	-2.3112488	0.02081912
25078	USP36	1992.30083	0.67885766	0.08176485	8.3025611	1.02E-16
25079	USP37	1339.0372	1.1276711	0.09250728	12.1900795	3.51E-34
25080	USP38	1992.67659	0.57948179	0.08420566	6.88174421	5.91E-12
25081	USP39	4233.40051	0.27656593	0.06231275	4.4383525	9.07E-06
25086	USP44	176.420536	0.85136645	0.18588663	4.58003066	4.65E-06
25087	USP45	680.807151	0.45464037	0.09824667	4.62753959	3.70E-06
25090	USP47	3944.80504	0.51127999	0.05786939	8.83506849	1.00E-18
25092	USP49	845.473636	0.61841973	0.12400571	4.9870261	6.13E-07
25093	USP5	3116.2849	-0.836878	0.06035197	-13.866623	1.01E-43
25096	USP53	2415.20372	0.78878877	0.09264553	8.51405077	1.68E-17
25104	USPL1	611.236418	0.36461443	0.11701404	3.11598883	0.00183329
25108	UTP11L	1741.93342	0.62406688	0.07913456	7.88614806	3.12E-15
25109	UTP14A	1542.72849	0.21202073	0.07636859	2.77628192	0.00549845
25111	UTP15	1271.2994	0.59365747	0.09645427	6.15480736	7.52E-10
25112	UTP18	5361.72405	0.67320694	0.07388352	9.11173331	8.11E-20
25113	UTP20	3063.87149	0.908708	0.07645978	11.8847851	1.42E-32
25114	UTP23	1407.48105	0.98996856	0.08717902	11.355583	6.96E-30
25116	UTP6	3174.89695	0.71760734	0.07120601	10.0779047	6.92E-24
25122	UVRAG	1442.42519	0.16124819	0.07494334	2.15160125	0.03142877

25124	UXS1	4883.37241	-0.3916431	0.05853436	-6.6908246	2.22E-11
25129	VAMP1	127.738041	-1.0977325	0.21387422	-5.1326076	2.86E-07
25134	VAMP7	3296.61809	0.14763066	0.06367219	2.31860514	0.02041646
25135	VAMP8	4392.58861	-0.2623111	0.09424951	-2.7831563	0.00538329
25136	VANGL1	2793.01445	-0.7786531	0.07515774	-10.360252	3.76E-25
25140	VARS	8184.94791	0.34801046	0.0802224	4.33807114	1.44E-05
25142	VASH1	84.8906244	-1.6837782	0.23152402	-7.2725852	3.53E-13
25144	VASN	1524.47463	-0.8366298	0.09977741	-8.3849625	5.07E-17
25145	VASP	5209.34492	0.25523177	0.08576468	2.97595427	0.00292078
25157	VCL	12372.1915	-0.699662	0.09268179	-7.5490775	4.38E-14
25159	VCPIP1	1339.7766	0.4995036	0.09685832	5.15705402	2.51E-07
25160	VCPKMT	369.943303	0.46754099	0.114404	4.08675398	4.37E-05
25167	VDAC1	10020.3755	-0.3649363	0.05732128	-6.3665061	1.93E-10
25169	VDAC3	4438.06905	-0.2065843	0.08769885	-2.35561	0.01849232
25171	VEGFA	32582.6354	2.36355491	0.06516513	36.2702382	4.77E-288
25172	VEGFB	6231.20062	-0.3651208	0.07184271	-5.0822245	3.73E-07
25179	VEZT	3881.18365	0.27042125	0.0710054	3.80846045	0.00013983
25183	VGLL3	448.203819	0.76658743	0.1313043	5.83825072	5.28E-09
25184	VGLL4	1593.08213	-0.2920889	0.07265516	-4.020209	5.81E-05
25189	VIM	59528.7333	-0.6744732	0.04906948	-13.74527	5.44E-43
25198	VKORC1	1975.35968	-0.5779831	0.1066221	-5.4208568	5.93E-08
25200	VLDLR	1578.95444	1.63949593	0.07831692	20.934121	2.62E-97
25201	VLDLR-AS1	13.1147292	1.45726568	0.61119918	2.38427295	0.0171129
25203	VMAC	49.4464121	-0.7747876	0.29523046	-2.6243485	0.00868149
25205	VMP1	4621.21979	-0.411156	0.06205196	-6.6259955	3.45E-11
25224	VPS16	738.923135	-1.088667	0.09465711	-11.501165	1.30E-30
25228	VPS26B	2198.03564	-0.4949099	0.06474822	-7.643606	2.11E-14
25232	VPS33B	622.828289	-0.5578791	0.11285171	-4.9434705	7.67E-07
25234	VPS36	944.185596	-0.716393	0.08834974	-8.1086029	5.12E-16
25235	VPS37A	1810.55577	0.52438401	0.09403464	5.57649824	2.45E-08
25236	VPS37B	2872.8273	0.14007255	0.06285031	2.22866908	0.02583593
25237	VPS37C	1515.15864	0.731297	0.07479622	9.77719191	1.41E-22
25238	VPS37D	115.113015	-0.5503328	0.20577224	-2.6744755	0.00748462
25240	VPS41	2003.90036	-0.3324548	0.07056627	-4.7112418	2.46E-06
25242	VPS4A	3279.21751	-0.2475451	0.0686621	-3.6052652	0.00031183
25244	VPS50	1330.03106	0.23414736	0.07268	3.22162027	0.00127468
25245	VPS51	2930.3938	-0.7798157	0.08900936	-8.7610527	1.93E-18
25247	VPS53	2163.21221	-0.3270712	0.07405807	-4.4164162	1.00E-05
25248	VPS54	988.331956	-0.3023966	0.09235335	-3.2743436	0.00105908
25251	VPS9D1	479.829117	-0.6814965	0.10497742	-6.4918388	8.48E-11
25252	VPS9D1-AS1	297.31471	0.57450846	0.13532039	4.24554261	2.18E-05
25255	VRK3	1085.95252	-0.3311238	0.08769322	-3.7759335	0.00015941
25258	VSIG10	833.851577	-0.987014	0.08904652	-11.084251	1.50E-28

25269	VSTM4	61.8340789	2.84136137	0.32231879	8.81537613	1.19E-18
25271	VSX1	26.9664956	-2.0548662	0.44162231	-4.6529945	3.27E-06
25277	VTN	13.3363404	-2.049782	0.67166111	-3.0518099	0.00227466
25282	VWA1	1607.29438	-1.3488311	0.10721078	-12.581115	2.68E-36
25290	VWA8	540.729306	-0.331035	0.10835324	-3.0551465	0.00224951
25297	VWDE	269.692176	1.69877877	0.15395426	11.0343081	2.61E-28
25299	WAC	6870.87577	0.37421598	0.07973688	4.69313571	2.69E-06
25301	WAPL	4131.36613	0.41405383	0.08486918	4.87872991	1.07E-06
25302	WARS	5536.86558	1.04507191	0.08389054	12.4575663	1.27E-35
25307	WASF2	5807.05938	0.71363446	0.06317011	11.2970265	1.36E-29
25319	WBP1L	1492.58811	-1.0604254	0.06999626	-15.149744	7.61E-52
25322	WBP4	1296.33075	0.58679421	0.08427658	6.96271996	3.34E-12
25329	WDFY1	3245.99659	0.25601022	0.07551758	3.39007439	0.00069874
25330	WDFY2	325.670687	-0.3645764	0.11703394	-3.1151344	0.00183861
25334	WDHD1	2776.1684	0.84733707	0.06544014	12.9482762	2.40E-38
25337	WDR11	2970.79019	-0.7646211	0.06536826	-11.697131	1.32E-31
25339	WDR12	2186.34628	0.48340182	0.0655238	7.37749999	1.61E-13
25340	WDR13	2336.14328	-0.7628808	0.07808536	-9.7698303	1.52E-22
25344	WDR20	1055.54512	0.57945275	0.07829474	7.40091533	1.35E-13
25345	WDR24	582.390071	-0.623513	0.13708125	-4.5484922	5.40E-06
25349	WDR3	3804.16098	1.31565257	0.06934974	18.97127	2.95E-80
25352	WDR34	4695.44025	-0.8342702	0.10143454	-8.2247151	1.96E-16
25353	WDR35	1101.01355	0.43828836	0.09444766	4.64054233	3.47E-06
25354	WDR36	3360.88792	0.91375199	0.06858999	13.3219445	1.73E-40
25357	WDR4	329.967848	1.09342788	0.15243159	7.17323657	7.32E-13
25359	WDR43	3786.03005	1.20795954	0.07877349	15.3345939	4.49E-53
25363	WDR46	4931.98803	0.91795152	0.06327582	14.5071453	1.09E-47
25364	WDR47	1489.59084	0.52644861	0.08528335	6.17293546	6.70E-10
25368	WDR53	825.707939	0.97257457	0.08844731	10.9960905	3.99E-28
25369	WDR54	706.618142	-1.106844	0.1106252	-10.005351	1.44E-23
25373	WDR6	8536.65589	-0.2471011	0.06195526	-3.9883805	6.65E-05
25374	WDR60	2303.40408	-0.3200516	0.06716705	-4.7650094	1.89E-06
25375	WDR61	733.444356	-1.1483524	0.09773318	-11.749872	7.07E-32
25376	WDR62	2465.28274	0.1907434	0.0709292	2.68920824	0.00716217
25380	WDR7	493.770322	-0.5981371	0.1308022	-4.5728365	4.81E-06
25383	WDR73	933.9006	0.25636218	0.08674622	2.9553122	0.00312353
25384	WDR74	1726.11064	0.16526142	0.0765048	2.16014446	0.03076149
25385	WDR75	2822.54092	0.5203899	0.06496886	8.00983544	1.15E-15
25387	WDR77	3409.45348	0.46011008	0.09579826	4.80290661	1.56E-06
25388	WDR78	43.8614246	2.46970864	0.35876861	6.88384814	5.83E-12
25390	WDR82	5858.47319	0.35030323	0.06254458	5.60085686	2.13E-08
25392	WDR83OS	3380.2739	-0.2533516	0.09677376	-2.6179788	0.00884523
25397	WDR89	1023.59342	0.5705559	0.09262209	6.16004153	7.27E-10

25398	WDR90	676.405449	-0.246321	0.10630978	-2.3170113	0.02050311
25399	WDR91	370.799316	-0.4453602	0.11660796	-3.8192953	0.00013383
25402	WDSUB1	1264.01513	0.33146191	0.08035762	4.12483496	3.71E-05
25407	WEE2-AS1	16.648804	1.38483062	0.50670325	2.733021	0.00627563
25423	WFS1	1204.3539	-0.7693296	0.07784771	-9.8824952	4.96E-23
25426	WHAMMP2	25.8878374	-1.4293562	0.41574559	-3.438055	0.00058591
25427	WHAMMP3	71.5038656	-0.6772432	0.25028917	-2.705843	0.00681313
25429	WHSC1L1	1574.30588	0.55320762	0.11068541	4.99801775	5.79E-07
25431	WIBG	416.738358	-0.7699029	0.12891807	-5.9720323	2.34E-09
25433	WIPF1	1416.49768	-0.7036146	0.08533956	-8.2448819	1.65E-16
25434	WIPF2	2952.87945	0.3737585	0.09727587	3.84225294	0.00012191
25437	WIPI2	3622.19948	-0.9147149	0.06019144	-15.19676	3.72E-52
25439	WISP2	169.434441	-2.2570007	0.24491194	-9.2155601	3.10E-20
25441	WIZ	2202.93056	0.24849046	0.11232255	2.21229368	0.02694638
25445	WNK3	390.707694	0.90381942	0.14166675	6.37989787	1.77E-10
25461	WNT7B	982.242924	-0.8323068	0.08707611	-9.558382	1.20E-21
25469	WRN	788.985742	0.78399721	0.10059895	7.79329401	6.53E-15
25492	XAB2	1957.9804	-0.7228502	0.07231182	-9.9962937	1.58E-23
25500	XBP1	7487.36862	1.61344102	0.08937767	18.0519479	7.62E-73
25527	XPC	739.866045	-0.8201666	0.09244465	-8.8719753	7.19E-19
25532	XPO4	1675.95715	0.59473332	0.10317198	5.76448512	8.19E-09
25533	XPO5	6306.31746	1.17769767	0.07324246	16.079438	3.56E-58
25534	XPO6	5276.08611	0.27969485	0.06218087	4.4980851	6.86E-06
25536	XPOT	6272.3579	0.82395776	0.08363953	9.85129581	6.77E-23
25540	XRCC3	2733.87652	1.34558909	0.08236375	16.337152	5.37E-60
25543	XRCC6	13915.6926	0.26291201	0.05017558	5.23983956	1.61E-07
25547	XRN2	8844.66139	0.37000646	0.06535742	5.66127738	1.50E-08
25548	XRRA1	411.806638	0.47731481	0.13155753	3.62818306	0.00028542
25549	XXYLT1	1563.18291	-0.4550646	0.07355325	-6.186873	6.14E-10
25554	XYLT2	1228.48099	-0.3432389	0.07604639	-4.5135466	6.38E-06
25555	YAE1D1	743.796255	0.85728803	0.10340726	8.29040457	1.13E-16
25557	YAP1	11526.3317	0.56871141	0.07151871	7.95192448	1.84E-15
25558	YARS	7556.38272	1.55311296	0.0589131	26.3627789	3.66E-153
25565	YDJC	1511.96705	0.57913009	0.13461068	4.30225948	1.69E-05
25568	YES1	5514.08282	0.61919867	0.07511781	8.24303469	1.68E-16
25569	YIF1A	2228.61299	-0.6802684	0.08685806	-7.8319546	4.80E-15
25570	YIF1B	2184.69099	-0.489456	0.07709936	-6.3483796	2.18E-10
25579	YKT6	6540.63724	0.66150494	0.05971004	11.078621	1.59E-28
25582	YOD1	1888.09691	0.91312979	0.08305702	10.9940107	4.08E-28
25583	YPEL1	42.8052194	-2.1165629	0.32880195	-6.4371968	1.22E-10
25584	YPEL2	329.737085	-0.3685853	0.12344834	-2.9857455	0.00282888
25585	YPEL3	670.195236	-1.1406536	0.10898166	-10.466473	1.23E-25
25588	YRDC	2817.59035	1.94675974	0.09375273	20.7648331	9.00E-96

25591	YTHDF1	5087.97043	0.2859551	0.06357499	4.49791787	6.86E-06
25592	YTHDF2	4282.6505	1.10948377	0.07879238	14.0811054	4.96E-45
25593	YTHDF3	2773.89275	0.19109298	0.07949528	2.40382789	0.01622441
25594	YTHDF3-AS1	20.0076138	-1.1212076	0.45968771	-2.4390638	0.01472537
25600	YWHAH	7724.55094	-0.2896818	0.05742746	-5.0443083	4.55E-07
25602	YWHAZ	38162.9131	0.36387294	0.05008416	7.26522973	3.72E-13
25603	YY1	5985.66015	0.92346928	0.06464718	14.2847569	2.72E-46
25604	YY1AP1	2257.19492	0.2483177	0.06996244	3.5493001	0.00038626
25609	ZAK	3162.34831	-0.294733	0.08357651	-3.5265052	0.00042108
25620	ZBED4	1896.20809	0.79964925	0.08807343	9.0793469	1.09E-19
25622	ZBED5-AS1	713.37806	-0.8856298	0.10547916	-8.3962533	4.61E-17
25626	ZBED9	143.24835	0.53252769	0.19013407	2.80080095	0.0050976
25629	ZBTB10	1129.58905	0.29363566	0.09948451	2.95157178	0.00316161
25630	ZBTB11	2118.7177	1.1289275	0.09620229	11.7349339	8.44E-32
25635	ZBTB17	1455.91907	0.75427656	0.07415524	10.1715875	2.66E-24
25637	ZBTB2	1413.7317	0.39384552	0.08307492	4.74084759	2.13E-06
25642	ZBTB21	1908.49593	1.86166859	0.09377725	19.852029	1.06E-87
25646	ZBTB26	220.946947	0.52954211	0.16610518	3.1879928	0.00143264
25647	ZBTB3	76.8516657	-1.0566141	0.2485857	-4.2505025	2.13E-05
25655	ZBTB40	2069.00004	0.76760338	0.06831049	11.2369773	2.68E-29
25658	ZBTB43	1128.41849	0.24388608	0.08617545	2.83011106	0.00465318
25660	ZBTB45	756.867181	-0.5050414	0.10449054	-4.8333692	1.34E-06
25663	ZBTB47	1814.91191	0.24159426	0.0758462	3.18531791	0.00144595
25669	ZBTB7B	2475.2859	1.01163432	0.08951923	11.3007482	1.30E-29
25671	ZBTB8A	646.631355	1.22235999	0.09607297	12.7232452	4.39E-37
25673	ZBTB8OS	1139.30517	1.18598646	0.08937282	13.2701015	3.45E-40
25674	ZBTB9	631.671537	0.30458976	0.0962403	3.16488789	0.00155143
25681	ZC3H12B	15.3082501	-2.1202188	0.59512024	-3.562673	0.0003671
25682	ZC3H12C	6532.9216	0.53565565	0.08131324	6.58755742	4.47E-11
25683	ZC3H12D	45.2295751	-2.0326066	0.36034659	-5.6406987	1.69E-08
25686	ZC3H15	5833.29325	0.68960108	0.07196214	9.58283125	9.44E-22
25691	ZC3H7A	3839.02079	0.92026694	0.06822662	13.4883852	1.83E-41
25693	ZC3H8	613.571129	1.63388024	0.10459349	15.6212424	5.22E-55
25696	ZC3HC1	1188.38068	0.58487797	0.09104459	6.4240829	1.33E-10
25697	ZC4H2	410.801103	-0.6267592	0.11809746	-5.3071356	1.11E-07
25699	ZCCHC11	2378.61144	1.02039101	0.09783194	10.4300392	1.81E-25
25705	ZCCHC18	101.732701	2.28336311	0.24177574	9.44413668	3.58E-21
25706	ZCCHC2	540.301347	0.92018287	0.11216176	8.20406953	2.32E-16
25707	ZCCHC24	2332.13382	-0.8463065	0.06816421	-12.415702	2.15E-35
25710	ZCCHC5	45.409225	1.73656785	0.32659172	5.317244	1.05E-07
25713	ZCCHC8	1459.21237	0.27254769	0.10346754	2.63413715	0.00843514
25716	ZCWPW1	219.500544	0.36543584	0.14664749	2.49193389	0.01270497
25719	ZDHHC1	467.898961	-1.2597611	0.12002655	-10.495687	9.04E-26

25720	ZDHHC11	58.1515605	1.56959075	0.28069654	5.59177094	2.25E-08
25721	ZDHHC12	1225.6006	-0.7918148	0.12092855	-6.5477903	5.84E-11
25725	ZDHHC16	2170.18344	-1.5247816	0.07216235	-21.129877	4.23E-99
25727	ZDHHC18	1563.12212	0.5406963	0.07598983	7.11537681	1.12E-12
25729	ZDHHC2	2431.41694	-0.3961365	0.09550227	-4.1479281	3.35E-05
25731	ZDHHC21	620.358829	1.08229921	0.10584618	10.2252079	1.53E-24
25734	ZDHHC24	431.338751	-0.5939531	0.11776979	-5.0433404	4.57E-07
25738	ZDHHC6	1711.78314	-0.7642927	0.07320723	-10.440126	1.63E-25
25742	ZDHHC9	2556.45389	0.31600805	0.08354056	3.78268991	0.00015514
25743	ZEB1	2589.93888	0.64790788	0.10451256	6.19933058	5.67E-10
25745	ZEB2	603.735211	1.02954652	0.11776857	8.74211591	2.29E-18
25747	ZER1	2032.4139	-0.3988379	0.06600792	-6.0422728	1.52E-09
25748	ZFAND1	2763.08589	0.724893	0.06990707	10.369381	3.42E-25
25749	ZFAND2A	953.290904	0.66511419	0.0853159	7.79589928	6.40E-15
25750	ZFAND2B	702.933669	-0.3330176	0.12063575	-2.7605214	0.00577092
25751	ZFAND3	10098.004	0.48136167	0.07335182	6.56236863	5.30E-11
25752	ZFAND4	179.276461	-1.187093	0.15787919	-7.5189964	5.52E-14
25755	ZFAS1	5124.4536	1.80086208	0.0988797	18.2126577	4.10E-74
25763	ZFP1	379.055475	0.27550195	0.11325058	2.43267589	0.01498771
25768	ZFP30	706.955518	0.51197155	0.11028986	4.64205474	3.45E-06
25770	ZFP36L1	9548.82547	0.31693248	0.0824356	3.84460695	0.00012075
25778	ZFP69	134.140113	0.41160041	0.17800787	2.3122596	0.02076338
25779	ZFP69B	175.313231	2.15443683	0.18165542	11.8600199	1.91E-32
25780	ZFP82	659.349277	0.90708713	0.09793421	9.26220894	2.00E-20
25782	ZFP91	6035.28119	0.17023836	0.07099108	2.39802455	0.01648376
25785	ZFPL1	1453.49828	-0.6908744	0.07652978	-9.027524	1.76E-19
25789	ZFR	8918.4119	0.44998817	0.08082434	5.56748348	2.58E-08
25794	ZFYVE1	1306.86157	0.2864577	0.08041807	3.56210623	0.00036789
25796	ZFYVE19	1610.40352	-0.8366087	0.10749293	-7.7829186	7.09E-15
25797	ZFYVE21	1318.02874	-0.2246332	0.08382102	-2.6799152	0.00736408
25801	ZFYVE9	1734.58048	0.90105562	0.07723837	11.6659072	1.90E-31
25803	ZG16B	31.232466	-4.4437692	0.57904523	-7.6743042	1.66E-14
25807	ZHX1	3096.29102	1.00189802	0.06778204	14.7811729	1.94E-49
25810	ZHX3	1772.03303	-0.191312	0.08120253	-2.3559862	0.0184736
25820	ZKSCAN1	2262.31798	0.25648786	0.06607973	3.88149096	0.00010382
25826	ZKSCAN8	1985.73363	0.47753261	0.23836892	2.00333417	0.04514143
25828	ZMAT2	4024.73687	0.23574157	0.07661683	3.07689002	0.00209172
25831	ZMAT5	524.146753	0.28500951	0.10023794	2.84332967	0.00446449
25835	ZMPSTE24	5134.15942	0.81754488	0.0623815	13.1055672	3.06E-39
25836	ZMYM1	1374.7756	0.81856797	0.09122564	8.97300283	2.89E-19
25838	ZMYM3	2646.52712	-0.8721316	0.09492965	-9.1871356	4.03E-20
25839	ZMYM4	2819.40898	0.50275561	0.08123942	6.18856746	6.07E-10
25840	ZMYM5	548.941371	0.68161518	0.11143942	6.11646354	9.57E-10

25841	ZMYM6	723.248114	0.35352972	0.0950608	3.71898517	0.00020002
25848	ZMYND8	5576.42918	-0.4442254	0.07064837	-6.2878365	3.22E-10
25849	ZNF10	281.936588	0.34529085	0.14336499	2.40847392	0.01601937
25850	ZNF100	487.457844	0.35104561	0.11534366	3.04347564	0.00233862
25855	ZNF114	5224.10247	1.26055043	0.06220467	20.2645615	2.64E-91
25858	ZNF121	268.634299	0.99014434	0.17871248	5.54043193	3.02E-08
25859	ZNF124	271.786382	1.21539591	0.13829678	8.78831701	1.52E-18
25860	ZNF131	2496.5198	0.52401671	0.06999488	7.48650057	7.07E-14
25863	ZNF134	670.969541	0.53960829	0.09653761	5.58961753	2.28E-08
25865	ZNF136	289.839286	0.71363331	0.13089002	5.45215974	4.98E-08
25868	ZNF14	342.6503	1.34753748	0.12910991	10.4371345	1.68E-25
25869	ZNF140	630.678517	0.64056556	0.105859	6.05112029	1.44E-09
25871	ZNF142	836.46991	0.34803654	0.11461789	3.03649395	0.00239347
25873	ZNF146	7453.9338	1.28874095	0.05984475	21.5347353	7.36E-103
25878	ZNF16	338.667024	0.5214459	0.12262981	4.25219521	2.12E-05
25884	ZNF175	752.232128	0.48253103	0.10360402	4.65745449	3.20E-06
25889	ZNF182	790.186655	0.25479784	0.08602376	2.96194736	0.003057
25892	ZNF189	740.05828	0.73599034	0.09540103	7.71469992	1.21E-14
25895	ZNF195	1051.08024	0.59411388	0.08356911	7.10925204	1.17E-12
25896	ZNF197	708.098221	0.42414657	0.09786522	4.33398661	1.46E-05
25900	ZNF200	414.499432	0.38990796	0.11566713	3.37094858	0.0007491
25901	ZNF202	999.01601	0.31390076	0.08789912	3.57114778	0.00035542
25905	ZNF207	3358.94406	-0.6000811	0.09717373	-6.1753427	6.60E-10
25907	ZNF211	859.645577	-0.1839528	0.08891808	-2.0687894	0.03856585
25913	ZNF217	4064.89138	0.45017173	0.08666921	5.19413683	2.06E-07
25918	ZNF223	192.797083	-0.3959713	0.16734881	-2.3661436	0.01797447
25924	ZNF23	738.778199	0.21524841	0.08732776	2.4648338	0.01370768
25926	ZNF232	489.894341	0.95358141	0.10737104	8.88117926	6.62E-19
25933	ZNF248	543.430539	-0.2093026	0.10257711	-2.0404419	0.04130633
25934	ZNF25	311.657245	-0.2954033	0.12932714	-2.2841553	0.02236241
25935	ZNF250	416.433266	0.43325345	0.10831822	3.99982059	6.34E-05
25936	ZNF251	1132.87616	0.4181298	0.0756755	5.52529928	3.29E-08
25943	ZNF26	378.235413	0.79992398	0.12228147	6.54166133	6.08E-11
25944	ZNF260	696.971704	0.52857057	0.10023037	5.27355699	1.34E-07
25945	ZNF263	1130.26357	0.17464661	0.08540788	2.04485343	0.04086931
25946	ZNF264	842.493946	0.5139916	0.08761165	5.86670407	4.45E-09
25948	ZNF267	467.757461	0.40882758	0.10550868	3.87482419	0.0001067
25950	ZNF271P	1129.81808	0.37182732	0.09109173	4.08189994	4.47E-05
25952	ZNF274	1992.29754	0.27831229	0.0707277	3.93498318	8.32E-05
25954	ZNF276	935.507838	0.49029386	0.08481039	5.7810591	7.42E-09
25956	ZNF28	527.993447	0.61131283	0.12461574	4.90558294	9.32E-07
25960	ZNF280D	478.864547	-0.6752218	0.10694451	-6.3137586	2.72E-10
25961	ZNF281	2041.78044	0.31986809	0.0990259	3.23014584	0.00123727

25966	ZNF286A	365.139969	0.74547375	0.13416058	5.55657807	2.75E-08
25967	ZNF286B	45.870936	0.92608679	0.32904024	2.81450922	0.00488518
25971	ZNF296	194.973709	0.57344969	0.14672838	3.90823986	9.30E-05
25972	ZNF3	1050.50143	0.36060758	0.07702076	4.68195311	2.84E-06
25975	ZNF300	9.26571891	3.58986411	0.93387727	3.84404271	0.00012102
25981	ZNF317	2015.20544	0.62175333	0.07832822	7.93779469	2.06E-15
25982	ZNF318	1572.53808	-0.2008464	0.08618503	-2.3304089	0.01978455
25984	ZNF32	768.693366	-0.6826543	0.0942752	-7.2410809	4.45E-13
25991	ZNF324	464.470649	0.34512461	0.11248009	3.06831725	0.00215268
25995	ZNF330	2657.52694	0.90485664	0.09511259	9.51353119	1.84E-21
25996	ZNF331	1144.05316	0.32180899	0.08073871	3.98580792	6.73E-05
25998	ZNF334	235.871146	-0.9105915	0.15881248	-5.733753	9.82E-09
26006	ZNF341	233.389986	-0.2989832	0.13817908	-2.1637373	0.03048452
26008	ZNF343	626.543725	0.25093138	0.11041225	2.27267696	0.02304565
26010	ZNF346	613.810685	0.33668275	0.10010344	3.36334847	0.00077003
26011	ZNF347	798.813034	0.48922058	0.10824496	4.51956915	6.20E-06
26012	ZNF35	489.827817	1.28623724	0.10808082	11.9006989	1.17E-32
26013	ZNF350	460.805199	0.36467673	0.10295547	3.54208201	0.00039698
26015	ZNF354A	720.905265	0.67373262	0.10397796	6.47957128	9.20E-11
26018	ZNF358	2794.71415	-0.5434341	0.10851125	-5.0080896	5.50E-07
26023	ZNF37A	1521.90228	0.61544228	0.11481459	5.36031436	8.31E-08
26024	ZNF37BP	740.167299	-0.7541991	0.0915395	-8.2390562	1.74E-16
26029	ZNF385B	22.9091801	-2.5940365	0.47585096	-5.4513633	5.00E-08
26036	ZNF395	4990.27998	-0.1984405	0.05891009	-3.3685317	0.0007557
26042	ZNF408	658.962487	0.38195993	0.09406843	4.06044756	4.90E-05
26044	ZNF410	2102.05197	0.57269229	0.08092751	7.07660869	1.48E-12
26045	ZNF414	316.89177	-1.0839614	0.15429467	-7.0252681	2.14E-12
26050	ZNF419	461.407365	0.5768573	0.13921825	4.14354644	3.42E-05
26051	ZNF420	362.556902	0.2927064	0.12023788	2.43439424	0.01491674
26054	ZNF426	345.649631	0.81458237	0.14240126	5.72033113	1.06E-08
26058	ZNF430	386.831502	0.72262827	0.14699133	4.91612847	8.83E-07
26059	ZNF431	378.708535	0.73902919	0.11947507	6.18563524	6.19E-10
26062	ZNF436	714.012389	0.69833916	0.0912682	7.65150561	1.99E-14
26066	ZNF44	331.836744	0.4215347	0.13237226	3.184464	0.00145022
26067	ZNF440	649.019591	0.55701737	0.11969034	4.65382057	3.26E-06
26069	ZNF442	38.2737337	0.88966854	0.35991163	2.47190827	0.0134394
26073	ZNF446	365.224649	-0.422705	0.12056799	-3.5059475	0.00045498
26078	ZNF460	127.664708	1.47114883	0.23799023	6.1815514	6.35E-10
26081	ZNF467	235.321326	-0.7884339	0.13971436	-5.6431844	1.67E-08
26082	ZNF468	935.167369	0.77668756	0.12446862	6.24002687	4.37E-10
26086	ZNF473	1034.46932	1.07675859	0.09106327	11.8242908	2.92E-32
26089	ZNF48	532.267319	0.8844831	0.10229759	8.64617767	5.33E-18
26092	ZNF484	202.579933	0.65907603	0.15878266	4.15080605	3.31E-05

26094	ZNF486	649.055852	0.53832282	0.1042334	5.1645902	2.41E-07
26096	ZNF488	363.040044	-1.6025851	0.14495002	-11.056122	2.05E-28
26102	ZNF497	130.391104	-0.6003677	0.17978967	-3.3392782	0.00083996
26103	ZNF500	233.004146	-0.5601119	0.1419758	-3.9451222	7.98E-05
26105	ZNF502	442.72603	0.83609257	0.11292214	7.40415114	1.32E-13
26106	ZNF503	47.1189772	1.67037262	0.33216968	5.02867271	4.94E-07
26108	ZNF503-AS2	11.4199051	1.40005026	0.61919922	2.26106593	0.02375517
26109	ZNF506	488.38976	0.59233372	0.10706271	5.53258682	3.16E-08
26110	ZNF507	1676.45684	0.78073038	0.11021786	7.0835199	1.41E-12
26112	ZNF511	1363.44415	-0.9656339	0.07970681	-12.114822	8.82E-34
26115	ZNF513	277.475124	-0.3996511	0.12784344	-3.126098	0.00177143
26117	ZNF516	701.834186	0.60473898	0.10272013	5.8872489	3.93E-09
26120	ZNF518B	1410.73457	0.8834711	0.09291141	9.50874735	1.93E-21
26123	ZNF524	441.582056	-0.4499914	0.13793072	-3.262445	0.00110456
26127	ZNF528	804.03469	0.24428767	0.10621644	2.29990437	0.02145364
26128	ZNF528-AS1	669.613996	0.58466597	0.13512559	4.32683371	1.51E-05
26130	ZNF529-AS1	175.612059	0.75610463	0.17071318	4.42909344	9.46E-06
26132	ZNF532	1006.33941	0.3168338	0.12510636	2.53251548	0.01132474
26137	ZNF542P	285.962578	1.86954064	0.13977216	13.3756294	8.39E-41
26139	ZNF544	1670.61867	0.4977417	0.07320235	6.79953204	1.05E-11
26143	ZNF549	448.852611	0.5512753	0.10908804	5.05348965	4.34E-07
26144	ZNF550	434.790212	0.53997203	0.11681853	4.62231486	3.79E-06
26145	ZNF551	226.367148	0.42797989	0.15949443	2.68335322	0.0072888
26148	ZNF555	281.105713	0.38411921	0.1491629	2.57516586	0.0100192
26151	ZNF558	1429.99527	0.72537247	0.08788473	8.2536801	1.54E-16
26157	ZNF562	1039.30267	0.52952464	0.08938439	5.92412864	3.14E-09
26158	ZNF563	164.410185	0.36144095	0.16198003	2.23139206	0.02565517
26161	ZNF566	447.441039	0.50921462	0.11426713	4.45635266	8.34E-06
26162	ZNF567	412.559543	0.25741749	0.11874356	2.16784375	0.03017058
26163	ZNF568	367.235001	0.45624419	0.11235028	4.0609082	4.89E-05
26165	ZNF57	210.504173	0.53359337	0.19033859	2.80339034	0.00505684
26166	ZNF570	158.840807	0.77821673	0.18043782	4.31293584	1.61E-05
26167	ZNF571	120.558661	0.86309355	0.19333721	4.46418747	8.04E-06
26172	ZNF575	40.8009967	-1.7765269	0.34488347	-5.1510934	2.59E-07
26173	ZNF576	459.198887	-0.2834615	0.12975874	-2.1845269	0.02892355
26175	ZNF578	11.2784073	1.34483786	0.59989264	2.24179757	0.02497446
26177	ZNF580	1333.96713	-0.3801544	0.10879767	-3.4941408	0.00047559
26182	ZNF584	1111.04623	0.85113114	0.08215703	10.3598091	3.78E-25
26185	ZNF586	261.01969	0.42894634	0.14074998	3.04757652	0.00230695
26189	ZNF592	1750.2202	-0.2133975	0.07096481	-3.0070891	0.00263762
26190	ZNF593	810.067186	1.19117198	0.14211336	8.3818437	5.21E-17
26191	ZNF594	321.802318	0.64079378	0.13889285	4.61358372	3.96E-06
26197	ZNF600	494.965485	0.53626741	0.11263493	4.76111094	1.93E-06

26200	ZNF607	385.740949	0.37850082	0.12867592	2.94150466	0.00326622
26202	ZNF609	2082.81042	0.27381556	0.09307002	2.94203815	0.0032606
26206	ZNF614	601.02185	0.55925425	0.10334395	5.41158169	6.25E-08
26208	ZNF616	276.191689	0.8697666	0.14611485	5.95262268	2.64E-09
26213	ZNF622	4221.07262	1.08828135	0.10266862	10.5999417	2.98E-26
26214	ZNF623	1000.19498	0.68453767	0.08593898	7.96539242	1.65E-15
26224	ZNF638	3033.29271	0.82583034	0.06445344	12.8128195	1.39E-37
26225	ZNF639	3075.97597	0.85253235	0.06486626	13.1429243	1.87E-39
26227	ZNF644	2412.08012	0.32174041	0.095187	3.3800877	0.00072463
26233	ZNF652	779.289502	-0.5362795	0.10534609	-5.0906447	3.57E-07
26234	ZNF653	526.154235	-0.3769807	0.10842315	-3.476939	0.00050717
26235	ZNF654	862.274834	0.36731823	0.10650596	3.44880453	0.00056307
26236	ZNF655	3440.32035	0.5526001	0.08468298	6.52551552	6.78E-11
26244	ZNF665	52.5243965	0.74406665	0.28444167	2.61588481	0.00889966
26248	ZNF669	339.904509	0.64012238	0.13471425	4.75170497	2.02E-06
26249	ZNF670	261.761654	0.31713976	0.14568582	2.17687466	0.02948992
26252	ZNF672	1084.17	-0.3358557	0.08867298	-3.7875764	0.00015212
26254	ZNF674-AS1	324.260733	0.26600489	0.12092214	2.19980296	0.02782088
26255	ZNF675	459.38157	1.18036229	0.11996155	9.83950545	7.61E-23
26261	ZNF681	220.214499	0.98065745	0.1670058	5.87199622	4.31E-09
26265	ZNF687	1334.82199	0.18307845	0.08154683	2.24507133	0.02476355
26266	ZNF688	211.34284	-0.4790672	0.15563581	-3.0781297	0.00208304
26268	ZNF69	282.343812	-0.61018	0.12936458	-4.7167472	2.40E-06
26269	ZNF691	329.543897	1.03037091	0.14073906	7.3211437	2.46E-13
26272	ZNF696	682.086662	0.56304253	0.11206437	5.02427771	5.05E-07
26273	ZNF697	1357.75061	0.48296511	0.0785942	6.14504783	7.99E-10
26274	ZNF699	81.6104311	0.91090262	0.24620075	3.69983695	0.00021574
26281	ZNF704	84.5775494	-4.369621	0.36054975	-12.119329	8.34E-34
26288	ZNF707	677.342742	0.36214044	0.10057365	3.60074878	0.0003173
26299	ZNF720	155.10491	0.59487397	0.18314527	3.24809901	0.00116179
26300	ZNF721	909.399565	0.85705116	0.09688372	8.84618328	9.06E-19
26311	ZNF737	199.039522	-0.8096917	0.16940885	-4.7795123	1.76E-06
26315	ZNF746	1287.39902	0.68073647	0.0811747	8.38606707	5.03E-17
26317	ZNF749	348.152308	0.75605737	0.13559883	5.57569256	2.47E-08
26327	ZNF767P	405.07512	-0.3476399	0.12500129	-2.78109	0.00541767
26329	ZNF77	168.201328	0.49487832	0.16135887	3.06694217	0.00216261
26330	ZNF770	3336.54303	1.36363064	0.07624926	17.8838535	1.58E-71
26331	ZNF771	169.471782	-1.2980176	0.20677808	-6.2773462	3.44E-10
26335	ZNF775	656.509054	-0.8617454	0.11600663	-7.428415	1.10E-13
26336	ZNF776	988.212758	0.46780369	0.1099524	4.25460174	2.09E-05
26338	ZNF778	171.147417	1.19769305	0.1906627	6.28173768	3.35E-10
26342	ZNF782	107.178476	0.54872978	0.25403705	2.16003843	0.03076969
26343	ZNF783	834.977022	0.22257792	0.09020579	2.46744621	0.01360807

26344	ZNF784	154.003484	-0.7508144	0.18693462	-4.0164545	5.91E-05
26350	ZNF79	307.279042	0.89683108	0.12771498	7.02212888	2.19E-12
26351	ZNF790	347.818978	0.60764669	0.11594448	5.24084205	1.60E-07
26352	ZNF790-AS1	36.299744	1.06128565	0.3638927	2.91647963	0.00354006
26354	ZNF792	387.553414	-0.2477341	0.11445685	-2.1644327	0.03043116
26358	ZNF8	186.585343	0.35835459	0.16178871	2.21495427	0.0267632
26360	ZNF800	1083.66856	0.66255078	0.09378101	7.06487126	1.61E-12
26366	ZNF81	186.539585	0.48593461	0.17318756	2.80582859	0.00501874
26375	ZNF823	435.062247	0.74309321	0.11080623	6.7062405	2.00E-11
26385	ZNF837	158.709736	-0.703976	0.17262876	-4.0779765	4.54E-05
26387	ZNF84	1309.25437	0.60752494	0.07917988	7.67271906	1.68E-14
26388	ZNF841	956.893483	0.76156502	0.10748461	7.08533987	1.39E-12
26394	ZNF85	337.698216	0.39143055	0.12782698	3.06219035	0.00219724
26399	ZNF862	900.647113	-0.454005	0.09609182	-4.7246997	2.30E-06
26400	ZNF865	1205.86001	-0.31287	0.08648878	-3.6174635	0.0002975
26403	ZNF879	108.050763	0.87020357	0.19929809	4.36634168	1.26E-05
26410	ZNF91	581.330796	0.30044271	0.11400187	2.63541914	0.00840334
26411	ZNF92	369.059439	-0.2746491	0.12428823	-2.2097758	0.02712073
26415	ZNFX1	4215.5294	0.33987764	0.08497873	3.99956106	6.35E-05
26416	ZNHIT1	1841.95468	-0.3061756	0.14403202	-2.1257471	0.03352432
26417	ZNHIT2	881.20679	0.62200007	0.16963865	3.66661777	0.00024578
26419	ZNHIT6	3171.22692	0.62076247	0.08326445	7.45531197	8.97E-14
26436	ZPR1	3346.38144	0.90691635	0.06168605	14.7021294	6.25E-49
26437	ZRANB1	2850.70715	0.40629517	0.06576679	6.17781622	6.50E-10
26438	ZRANB2	3928.52504	0.76570221	0.0737331	10.3847822	2.91E-25
26445	ZSCAN12	429.173414	0.82178048	0.12411768	6.62097813	3.57E-11
26446	ZSCAN12P1	12.9466446	1.30984906	0.56511195	2.31785767	0.02045706
26447	ZSCAN16	298.01419	0.36399893	0.1247133	2.91868581	0.0035151
26448	ZSCAN16-AS	165.475881	-0.8389498	0.18303596	-4.5835245	4.57E-06
26450	ZSCAN2	138.03341	-0.8201576	0.17207823	-4.7661902	1.88E-06
26451	ZSCAN20	196.547235	0.84791012	0.15388513	5.51001999	3.59E-08
26452	ZSCAN21	434.834378	0.53095675	0.11091034	4.78726087	1.69E-06
26455	ZSCAN25	794.782681	0.26998784	0.08852312	3.04991317	0.00228908
26464	ZSCAN9	458.354395	0.50677762	0.10318965	4.91112844	9.06E-07
26465	ZSWIM1	337.400067	-0.5318933	0.12433947	-4.2777513	1.89E-05
26470	ZSWIM6	1572.97851	0.35777294	0.12325528	2.90269861	0.00369962
26471	ZSWIM7	674.282232	0.41985078	0.12444049	3.37390825	0.00074109
26476	ZWILCH	2050.94546	0.54189986	0.07141154	7.58840778	3.24E-14
26477	ZWINT	4114.56269	-0.9333036	0.068224	-13.67999	1.34E-42
26478	ZXDA	154.359973	0.50446054	0.19157361	2.63324655	0.00845729
26479	ZXDB	966.918528	0.82922431	0.09041238	9.17157911	4.66E-20
26483	ZYX	10848.259	0.43248742	0.06130502	7.05468156	1.73E-12
26485	ZZZ3	2875.40569	0.8247782	0.07374873	11.1836265	4.90E-29

padj	CtrlA_1	CtrlA_2	CtrlA_3	TrtC_1	TrtC_2	TrtC_3
2.53E-11	1147.37137	1200.59133	1152.74881	758.008737	697.751604	817.551028
1.30E-16	1230.93975	1146.26111	1123.88032	718.11354	665.682241	717.490229
1.53E-07	206.662362	167.251853	181.174684	79.7903933	102.038884	89.977153
3.58E-05	1645.39378	1699.15099	1589.75808	1999.07283	2013.5673	2271.92311
1.00E-08	485.600086	409.074597	371.308555	184.380504	182.698192	259.847813
3.23E-06	2140.02829	2141.24984	2183.05539	2599.65728	2697.71372	2710.94957
2.05E-103	4983.61205	4938.72352	4897.68925	10981.9616	11106.6895	10811.2203
1.59E-12	729.529432	778.733151	723.70327	1120.30025	1112.70973	1154.18969
3.64E-31	3132.68521	3348.23296	3292.00373	5279.10494	5425.55321	5308.65203
1.42E-07	2213.43295	2057.09127	2465.76754	1595.80787	1694.81727	1539.69525
0.00211544	22.5860505	12.7835811	19.9093059	5.39124279	3.88719557	3.10266045
1.80E-15	5749.27916	5895.36151	5295.87537	8561.29356	7993.04588	9015.5556
2.18E-15	68.8874541	92.6809633	98.5510643	227.510446	255.583108	245.110175
6.65E-09	273.291211	301.479455	270.76656	462.568632	441.196697	468.501728
1.49E-22	4579.32174	4860.95673	5013.16323	3062.22591	2638.43399	2795.49706
1.46E-24	143.421421	119.313424	143.347003	10.7824856	20.4077767	14.7376371
2.47E-12	1506.48957	1518.05026	1464.32945	1026.49263	845.465035	976.562376
6.04E-07	1670.23844	1747.08942	1804.77858	1380.15816	1293.46432	1321.73335
2.92E-23	946.355517	810.692104	857.095619	391.404227	300.285857	394.037877
1.76E-28	7038.94265	6950.00695	7133.50431	4636.4688	4634.50891	4551.60288
1.34E-07	76.7925718	59.656712	75.6553625	23.7214683	20.4077767	20.1672929
3.71E-11	1177.86253	1269.83573	1251.29988	866.911841	756.059537	700.425596
3.38E-32	6083.55271	6713.5107	6762.19575	11025.0915	11007.566	11268.8628
0.01853208	9498.56355	8800.43032	9113.48478	10164.6492	10023.1338	10180.6046
4.90E-17	4736.2948	5101.71418	5352.61689	7544.50517	7781.19372	7378.90221
2.09E-05	3558.43226	3289.64155	3196.43906	2789.42902	2617.05441	2654.32601
0.00016474	76.7925718	86.2891727	75.6553625	127.23333	136.051845	150.479032
0.00445807	15.8102354	17.0447749	9.95465295	2.15649712	1.94359778	3.87832556
6.27E-06	6730.64306	6530.27937	6262.47217	5107.66342	4907.5844	5528.16526
1.20E-19	479.953574	383.507434	357.372041	144.485307	157.43142	120.228092
0.00046473	748.727575	777.667853	739.630715	590.88021	556.840765	625.961746
1.40E-17	918.122954	991.792837	851.122828	524.0288	425.647914	470.828723
0.000422	2338.78553	2311.69759	2442.87184	2003.38582	2012.5955	1966.31106
0.00093169	3983.05001	4164.25156	4794.16086	3522.63804	3676.31521	3237.62618
2.48E-12	1062.67368	1012.03351	1131.84404	705.174557	658.879648	694.995941
2.01E-12	2577.06836	2555.65093	2865.94459	1799.59684	1860.99488	1642.85871
1.03E-06	3845.2751	4052.39522	4315.34206	5565.91906	5155.39312	5058.88786
2.31E-50	4260.85843	4589.30563	4885.74367	11398.1655	11629.5173	9895.9355
2.08E-22	5592.30611	5634.36339	6228.62635	3377.07449	3594.6841	3080.16616
1.12E-12	102.76653	109.725738	87.600946	12.9389827	17.49238	27.923944
1.75E-12	1521.1705	1461.58944	1228.40417	742.913257	661.795045	878.052907
2.96E-21	1619.41982	1732.17525	1732.10961	2899.41037	2791.00642	2624.85074

0.0036766	2182.94178	2143.38044	1834.64254	1646.48555	1440.20596	1817.38336
1.21E-12	1684.91937	1715.13047	1506.13899	1043.7446	1067.03518	1105.32278
9.90E-13	333.144245	314.263036	360.358437	153.111295	178.810996	157.460018
0.00010575	679.840121	591.240628	608.229296	464.725129	468.407066	469.277393
5.15E-42	1505.36027	1539.35623	1515.09818	2894.01913	2870.69392	3122.05208
8.26E-28	726.141524	727.598827	606.238365	242.605926	170.064806	241.23185
1.44E-35	856.011315	895.915979	938.723774	341.804793	397.465747	349.824966
0.04061415	7532.44785	7405.95468	6600.93037	6093.18261	6157.31778	6801.0317
1.04E-06	3761.70671	3475.00347	3179.51615	2506.9279	2515.01553	2748.95716
0.01169832	2159.22643	2285.06513	2601.15082	2021.71605	2037.86228	1928.30347
8.14E-14	355.730296	271.651099	275.743887	108.903104	113.70047	134.96573
2.33E-09	33.8790758	34.0895497	44.7959383	117.529093	98.151688	122.555088
8.82E-11	7168.81244	7235.50693	7543.63601	5432.21624	5637.40537	5019.32894
1.07E-36	10578.1768	11083.3649	10761.9753	6748.75773	6623.78124	6696.31691
2.70E-09	3988.69652	4206.8635	4221.76832	3222.88494	3214.71073	3086.37148
9.60E-06	59.8530339	55.3955183	42.8050077	11.8607341	0.97179889	16.2889674
0.07536231	18.0688404	25.5671623	28.8684936	3.23474568	9.71798891	18.6159627
3.21E-06	511.574044	502.820858	463.886828	319.161573	261.413902	356.030287
2.87E-07	1386.7835	1412.58572	1391.66048	1039.43161	1064.11979	1083.60416
8.53E-08	111.80095	109.725738	109.501183	42.0516938	43.7309501	48.8669021
0.00109703	956.51924	959.833884	896.914231	759.086985	658.879648	794.281075
0.0001776	83.5683869	57.5261152	65.7007095	121.842087	116.615867	144.273711
0.0337298	2563.51673	2765.51472	2536.44557	2820.69823	2995.08418	2993.29167
1.67E-05	31.6204707	34.0895497	24.8866324	2.15649712	8.74619002	3.87832556
4.31E-11	1697.3417	1525.50735	1610.66285	1051.29234	1141.8637	1112.30377
4.53E-26	5954.81222	7010.72896	6879.66066	11927.5856	11478.8885	11406.9311
5.41E-42	1766.22915	1750.28532	1785.86474	835.642633	737.595359	840.820982
6.87E-06	2199.88132	2111.42149	2052.64944	1664.81577	1470.33172	1718.87389
1.28E-07	80.1804794	99.0727539	94.5692031	186.537001	183.66999	176.07598
1.29E-17	200225.338	192418.463	181146.811	133618.718	129911.048	135862.398
2.94E-07	123388.723	121255.463	115815.419	95195.3305	91005.079	100623.932
2.79E-21	35497.3663	34056.5255	39143.6863	22544.0209	22908.2153	20579.1711
3.51E-23	7902.85908	8553.28108	7194.22769	4364.75017	3906.63154	4582.62948
1.37E-05	8582.6992	9158.37059	9632.1222	11516.7729	11485.6911	10808.8933
2.27E-07	499.151717	549.693989	540.537655	780.651957	737.595359	748.516833
3.37E-09	1099.94066	1139.86932	1110.93927	745.069754	729.820967	829.186005
0.01437833	58.7237314	79.8973822	63.7097789	93.8076246	107.869677	107.041785
0.02337293	807.451306	837.324565	855.104689	958.562969	946.53212	976.562376
0.00074191	433.65217	475.123099	452.936709	542.359025	600.571715	644.577708
5.36E-09	206.662362	182.166031	128.415023	62.5384164	69.9695202	37.2319254
0.00699794	15020.8529	16338.482	17700.3684	13992.4315	14639.1785	13521.3942
6.80E-20	5635.21961	5667.38764	5321.75747	9345.18026	8187.40566	9514.30827
0.00823478	1931.10732	2248.84498	2422.96253	2756.00332	2728.81129	2486.00668

2.37E-40	577.073591	595.501822	641.07965	200.554232	174.9238	150.479032
1.07E-60	1324.67186	1294.33759	1274.19558	497.072586	504.363625	487.117691
1.51E-135	11198.1639	11661.8219	11349.2998	3743.679	3335.21379	3713.10889
3.63E-07	1246.74999	1666.12674	1818.71509	2844.4197	2756.99345	2199.01059
2.49E-22	267.644699	252.475728	300.630519	93.8076246	62.195129	71.3611903
4.68E-17	5159.78324	5021.81679	5120.67348	3700.54905	3548.03775	3471.10138
2.04E-06	181.817707	232.235057	245.879928	340.726545	437.309501	359.132947
8.86E-13	204.403757	181.100733	178.188288	74.3991506	56.3643357	75.2395159
1.56E-27	1145.11276	1335.88423	1345.86908	2572.70106	2399.37146	2396.02953
3.89E-05	773.57223	749.970094	717.730478	604.897441	516.025211	535.984593
3.14E-06	232.63632	269.520502	255.834581	369.839256	432.450507	377.74891
0.00245286	1875.7715	1894.10061	1694.28193	1547.28668	1345.94146	1571.49752
2.53E-19	725.012222	745.7089	715.739547	401.108464	329.439824	373.870584
4.70E-06	975.717383	1035.47007	889.945974	626.462413	496.589233	730.676536
0.00410213	10085.8009	10032.9806	10176.6417	9235.19891	8955.12678	8830.9473
3.37E-24	4186.32446	4672.39891	4811.08377	2573.77931	2748.24726	2482.90402
5.18E-16	527.38428	547.563392	524.610211	292.205359	287.652472	268.380129
1.86E-15	14067.7216	15049.4709	17267.341	9005.53196	10003.6978	8328.31631
1.70E-09	5416.13492	5361.64699	5138.59186	4061.76232	4199.14301	4085.42815
0.00240998	5408.2298	5436.21788	4925.56228	4306.52474	3931.89831	4768.78911
0.0364204	546.582423	503.886157	486.782529	390.325978	345.960405	488.669021
3.38E-06	1634.10076	1530.83384	1347.86001	1022.17963	989.291271	1175.90831
2.33E-40	94.8614122	85.2238743	99.5465295	483.055354	522.827804	380.85157
2.72E-05	5496.3154	5543.81302	5845.37221	6801.59191	7333.19443	6593.15345
6.24E-09	1923.2022	2171.0782	2093.46352	2870.29766	2766.71144	2746.63016
7.26E-09	99.3786223	161.925361	94.5692031	24.7997169	30.1257656	42.6615812
0.01091935	25.9739581	22.371267	21.9002365	9.70423703	7.77439113	9.30798135
1.45E-141	2284.57901	2289.32632	2189.02818	7218.8741	6828.83081	6704.07357
1.89E-18	2474.30183	2547.12854	2652.91501	1682.06775	1703.56346	1677.76364
1.09E-09	248.446556	202.406701	224.975157	384.934735	467.435267	393.262212
0.00588818	11.2930253	14.914178	12.9410488	2.15649712	3.88719557	1.55133022
0.00112456	2119.70084	1886.64352	2050.65851	2509.0844	2515.01553	2292.86607
0.00338782	3878.02488	4008.71799	4058.51201	3634.77589	3298.28544	3402.06718
9.20E-11	7329.1734	6771.03681	6470.52442	9729.03675	9023.15271	9793.54771
0.00124673	75.6632693	75.6361884	81.6281542	105.668359	143.826236	138.06839
0.00011312	1131.56113	1268.77043	1272.20465	1517.09572	1544.18844	1539.69525
4.27E-84	760.0206	763.818973	765.512812	2104.74119	2156.42174	2035.34525
2.90E-05	1244.49138	1285.8152	1469.30678	974.736697	1071.89418	907.528181
0.00699311	2699.03304	2646.2013	2622.05559	2365.67734	2308.02237	2349.48963
0.00388434	2470.91393	2688.81323	3082.95602	3383.54398	3624.80986	3126.70607
8.96E-17	5682.65031	6454.64318	7135.49524	11301.1231	11591.6172	10170.521
1.30E-07	5317.8856	5618.38391	4826.01575	7556.3659	6624.75304	8233.68517
0.00061009	942.967609	1039.73127	953.655753	737.522014	760.918532	826.85901

0.01235379	1737.99659	1804.61554	1927.22081	1988.29034	2282.7556	2114.4631
0.00947691	15.8102354	9.58768586	33.84582	40.9734452	49.5617435	48.8669021
4.67E-25	741.95176	789.386136	704.789429	345.039539	299.314059	352.927626
3.24E-07	352.342388	329.177215	290.675866	183.302255	175.895599	207.87825
9.75E-19	2376.05252	2482.14534	2731.55677	4141.55271	4304.09729	3900.04418
1.33E-08	2014.67571	2278.67334	2438.88997	1519.25222	1641.36833	1535.04126
0.00590833	1395.81792	1675.71443	1684.32728	1323.01098	1350.80046	1245.71817
7.47E-05	144.550723	150.207078	117.464905	242.605926	207.964963	218.737562
1.40E-12	800.675491	1071.69022	1062.16147	1872.91775	1964.97736	1604.07545
4.47E-09	342.178665	393.09512	359.362972	619.992921	654.020654	538.311588
4.75E-05	4376.04729	4524.32243	5060.94556	5824.69871	5982.39397	5602.62911
1.10E-08	32.7497733	28.7630576	32.8503548	0	0	0
5.77E-05	2638.0507	2941.28896	3146.6658	3604.58493	3823.05684	3620.02908
1.67E-20	8815.33552	9470.50303	10404.6033	5565.91906	5927.00144	5137.23004
1.45E-05	904.571324	930.005528	1018.361	699.783315	750.228744	677.155643
0.00619189	1041.21693	1157.97939	1318.99152	915.433026	1020.38884	892.014879
1.86E-07	5677.0038	6522.82228	6965.27067	8669.11841	9005.66033	8450.09573
1.08E-05	121.964673	135.2929	146.333398	254.46666	253.639511	197.794604
4.67E-26	645.961045	731.860021	704.789429	260.936151	227.400941	306.387719
4.15E-25	1995.47756	2052.83007	2097.44538	3193.77223	3283.70845	3364.83526
2.26E-17	2198.75202	2172.1435	1789.8466	3785.73069	3458.63225	4295.63339
0.00190292	4592.87337	4484.90639	3834.53232	3581.94171	3201.10555	3698.37126
3.87E-47	11253.4997	11605.3611	12084.9487	21539.0932	20658.5008	20457.3917
0.00026113	4250.69471	4297.41386	3529.91994	3151.72054	2683.13674	3326.052
7.61E-38	3541.49272	3645.45122	3381.59561	6853.34784	6297.25682	7195.84525
3.63E-10	1811.40125	1923.92896	1619.62204	2609.36151	2585.95685	3094.9038
5.95E-22	2834.54934	2749.53524	2716.62479	4400.33237	4252.59195	4105.59544
9.79E-09	1821.56497	2124.20507	2165.13702	3182.98975	3150.57201	2707.07124
5.57E-07	130.999093	138.488796	159.274447	57.1471736	79.6875091	50.4182323
1.43E-05	2306.03576	2270.15095	2194.00551	2908.03636	2646.20838	2998.72132
1.49E-43	2381.69903	2413.96624	2450.83556	4338.8722	4668.52187	4422.06681
3.97E-10	1265.94813	1192.06894	1220.44045	1734.90193	1827.95371	1648.28836
0.00663164	2089.20967	2125.27037	1702.24566	2447.62423	2177.80132	2883.14722
1.37E-47	892.148996	985.401047	931.755517	263.092648	322.637232	243.558845
1.13E-43	1971.76221	2058.15656	1908.30697	938.076246	954.306511	979.665037
0.00306146	2269.89808	2258.43267	1897.35685	1745.68442	1140.8919	1768.51646
1.27E-27	1548.27376	1795.02785	1763.9645	878.772575	893.083181	849.353298
1.63E-25	199.886547	159.794764	174.206427	34.5039539	33.0411623	30.2509394
7.95E-15	3952.55884	3797.7889	3426.39155	2489.67592	2490.72056	2446.44776
2.44E-43	3118.00427	3286.44565	3027.20996	1492.29601	1568.48341	1629.6724
1.96E-211	2714.84327	2698.40092	2645.94676	516.48106	497.561032	539.087253
0.0004052	559.00475	631.721968	623.161275	450.707898	412.04273	490.996016
0.00014865	336.532153	362.201466	364.340298	250.153666	258.498505	247.437171

6.01E-05	680.969423	785.124942	762.526416	525.107048	589.881927	532.106267
6.48E-16	3968.36908	3810.57248	3578.69774	2332.25163	2481.00257	2603.13212
0.00029551	43795.4813	43137.1293	41523.8439	37434.6335	37101.3381	37479.3626
1.54E-10	526.254977	476.188398	452.936709	291.127111	285.708874	268.380129
1.32E-10	1018.63088	1033.33948	1019.35646	695.47032	608.346106	705.855252
0.02824096	1843.02172	1764.1342	1840.61533	1539.73894	1710.36605	1537.36825
7.76E-15	368.152624	383.507434	372.304021	616.758176	708.441392	712.836238
0.02605936	1071.7081	1103.64917	939.719239	1248.61183	1123.39952	1300.01473
1.56E-05	5168.81766	4855.63024	4919.58949	4141.55271	4133.06068	4136.62204
4.69E-05	762.279205	808.561507	713.748617	584.410719	584.051134	494.874342
4.15E-07	1088.64764	1015.2294	836.190848	591.958459	478.125055	683.360964
1.07E-05	678.710818	707.358157	772.481069	948.858732	1048.571	935.452125
0.00095898	556.746145	608.285403	620.174879	850.738113	949.447517	667.071997
6.51E-25	525.125675	504.951455	534.564864	228.588694	209.908561	231.148203
2.50E-05	12.4223278	5.32649214	12.9410488	52.8341794	43.7309501	31.8022696
9.53E-10	234.894925	265.259309	276.739352	122.920336	133.136448	136.51706
1.55E-25	156.973051	171.513047	173.210961	472.272869	604.45891	452.988426
0.03319852	3.38790758	0	0.9954653	8.62598847	9.71798891	6.98098601
2.20E-07	1839.63382	2061.35246	2037.71746	2626.61349	2716.1779	2526.34127
3.69E-14	1700.7296	1694.8898	1606.68099	1094.42229	1136.0329	1132.47106
0.04492704	239.412136	216.255581	226.966087	164.972029	212.823957	143.498046
0.00050238	90.3442021	104.399246	63.7097789	50.6776823	32.0693634	38.7832556
0.00099073	1273.85325	1280.48871	1388.67409	1113.83076	1030.10682	1090.58515
4.18E-09	5092.02509	5356.3205	5526.82332	6930.98174	6819.11282	7525.50292
3.94E-16	264.256791	224.777968	285.69854	563.923996	689.977213	525.125281
4.20E-05	570.297776	573.130555	528.592072	407.577955	322.637232	418.859161
4.57E-08	264.256791	304.675351	359.362972	172.519769	163.262214	117.901097
8.51E-100	4260.85843	4952.5724	4488.55302	12969.1737	12711.1295	13179.3259
1.30E-47	461.884733	492.167874	475.832411	1277.72454	1254.59237	1142.55471
3.64E-21	1787.6859	1762.0036	1911.29337	3112.90359	3295.37004	2861.4286
0.00038134	72.2753617	73.5055916	59.7279177	128.311578	99.1234869	152.806027
1.57E-18	3701.85368	3875.55568	3929.10152	2643.86547	2386.73808	2436.36412
0.00087209	348.954481	372.85445	324.521686	241.527677	262.385701	259.847813
0.00019996	111.80095	161.925361	87.600946	59.3036707	49.5617435	63.6045392
8.94E-16	3.38790758	6.39179057	5.97279177	94.8858732	65.1105257	77.5665112
0.00501592	4643.69199	4464.66572	3325.84955	3170.05076	2504.32574	3592.10513
0.00026157	1841.89242	1920.73307	1693.28647	2187.76633	2186.54751	2337.07898
5.31E-07	1505.36027	1411.52042	1201.52661	883.08557	785.213504	1015.34563
0.000288	2142.28689	2194.51476	1983.96233	1719.80645	1311.9285	1706.46325
1.81E-10	975.717383	1031.20888	1025.32925	701.939812	628.753883	690.34195
0.00173477	259.739581	314.263036	310.585172	226.432197	216.711153	182.281301
2.81E-09	3015.23775	3151.15275	3217.34383	2270.79146	2438.24342	2153.24635
1.99E-12	907.959231	938.527916	988.497038	575.78473	623.894888	521.246955

1.62E-24	676.452213	809.626806	818.272473	321.318071	353.734796	301.733729
2.31E-05	83.5683869	107.595141	114.478509	48.5211851	51.5053412	42.6615812
9.18E-05	82.4390844	72.4402932	83.6190848	33.4257053	36.9283579	40.3345858
2.99E-11	1705.24681	1564.92339	1695.2774	1056.68359	1190.45364	1014.56997
1.32E-24	4589.48547	4748.0351	5367.54887	9387.23195	8993.02694	8225.92852
3.81E-05	2627.88698	2520.49608	2895.80854	3454.70838	3953.27789	3225.9912
4.43E-28	432.522868	381.376838	440.991126	963.954212	885.30879	898.2202
3.32E-38	2115.18363	2252.04088	2426.94439	4314.07248	4541.21622	4332.08965
0.07349654	2421.22462	2505.5819	2023.78095	2098.2717	1849.33329	2108.25778
0.00533037	4024.8342	3973.56314	4578.14489	5366.44308	5703.48769	4445.33676
8.90E-06	53.0772187	44.742534	37.8276812	9.70423703	12.6333856	13.961972
0.0219405	2365.88879	2411.83564	2546.40023	2895.09738	2823.07578	2607.01044
7.44E-22	5260.29117	5586.42496	5802.56721	8563.45005	8888.07266	8338.39996
0.02663658	4.5172101	5.32649214	2.98639589	24.7997169	8.74619002	11.6349767
1.55E-06	58.7237314	62.8526073	74.6598972	139.094064	155.487823	123.330753
0.01105422	127.611185	137.423497	136.378745	101.355365	83.5747047	93.0798135
0.00620945	83.5683869	91.6156649	62.7143136	37.7386996	52.4771401	44.2129114
1.32E-09	397.514489	357.940272	306.603311	159.580787	150.628828	204.77559
0.00119203	1678.14355	1822.72561	1987.9442	2387.24231	2522.78992	2053.18555
2.02E-22	507.056834	543.302199	581.351733	243.684174	250.724114	228.821208
2.14E-07	125.35258	73.5055916	69.6825707	20.4867226	22.3513745	31.8022696
1.73E-25	2951.9968	2898.67702	2669.83792	1511.70448	1383.84162	1642.85871
3.31E-19	2.25860505	3.19589529	5.97279177	102.433613	98.151688	89.2014879
2.64E-45	1582.15284	1585.16406	1547.94853	707.331055	585.022933	636.045392
1.08E-62	600.788944	632.787267	779.449326	2462.71971	2567.49267	2193.58094
3.87E-05	3417.26944	3292.83744	3832.54139	2630.92648	2901.79149	2262.61513
1.14E-05	10.1637227	10.6529843	4.97732648	37.7386996	34.9847601	41.8859161
2.24E-26	7920.92792	7889.60016	7970.69062	5350.26935	5374.04787	5266.76611
3.18E-05	2897.79028	2751.66584	2745.49328	2280.4957	2301.21977	2330.098
1.34E-89	13932.2053	13790.2882	13817.0583	6791.88767	6533.40395	6656.75799
1.20E-20	368.152624	331.307811	423.072751	144.485307	140.910839	124.882083
2.34E-25	264.256791	246.083937	250.857254	73.320902	64.1387268	75.2395159
1.10E-20	47.4307061	57.5261152	79.6372236	236.136434	260.442103	227.269878
0.00012462	24.8446556	9.58768586	17.9183753	63.616665	55.3925368	41.8859161
8.38E-45	1789.9445	1879.18643	1922.24349	3648.79312	3475.15284	3618.47775
8.22E-11	5455.6605	5219.9623	5464.10901	4103.81401	4145.69407	4118.00608
2.25E-32	4814.21667	5087.8653	5058.95463	2890.78439	3059.22291	2966.91905
1.28E-27	7134.93336	7415.54236	7016.0394	4611.66909	4231.21237	4469.38238
0.03936107	4808.57016	4933.39702	4456.69813	4360.43717	3767.6643	4360.78926
0.0586275	7994.33258	7845.92293	6209.71251	6201.00746	5165.11111	7077.16848
5.69E-19	1097.68206	1173.95887	1179.62638	1792.0491	1909.58482	1838.32632
2.43E-07	2248.44133	2541.80205	2524.49999	1869.683	1842.5307	1808.07538
5.46E-21	852.623407	839.455162	852.118293	475.507614	412.04273	449.885765

0.00777035	37.2669834	34.0895497	24.8866324	7.54773991	19.4359778	10.0836465
8.26E-05	2028.22734	2147.64163	1991.92606	1654.03329	1560.70902	1736.71419
3.06E-28	128.740488	135.2929	115.473974	376.308747	373.170774	411.10251
3.60E-61	3363.06292	3500.57064	3143.6794	1348.88895	1222.52301	1391.54321
6.26E-12	6469.77417	6137.18425	6215.6853	4438.07107	4756.95557	4718.37088
2.00E-11	7974.00514	7369.73453	7558.56799	5734.12584	5452.76358	5798.09671
1.66E-22	921.510861	896.981277	921.800864	500.307331	502.420027	469.277393
6.19E-49	678.710818	651.962638	674.92547	219.962706	184.641789	205.551255
1.16E-17	58371.389	60294.8258	58444.763	44155.3567	42227.5772	43247.984
0.00842717	11344.9732	10894.807	9816.28328	9326.85003	8739.38743	9586.44512
8.59E-17	589.495919	580.587644	683.884658	304.066094	329.439824	286.220426
5.35E-19	530.772187	573.130555	533.569398	284.65762	260.442103	251.315496
6.30E-22	1274.98255	1325.23125	1296.09581	746.148003	764.805727	773.338117
4.26E-07	869.562945	955.572691	988.497038	1205.48189	1375.09543	1355.08695
3.09E-16	1632.97145	1604.33943	1830.66068	2603.97027	2792.95001	2615.54276
7.12E-08	268.774001	321.720125	248.866324	458.255637	441.196697	521.246955
4.87E-09	1424.05049	1376.36557	1327.9507	1861.05701	1833.78451	2043.87757
8.44E-20	920.381559	964.095078	986.506108	546.672019	546.150977	503.406658
1.58E-14	71.1460592	70.3096963	72.6689666	0	9.71798891	4.65399067
0.00436341	14.6809328	37.285445	20.9047712	5.39124279	8.74619002	6.98098601
5.06E-10	3823.81835	3577.27212	3643.40298	2818.54173	2767.68324	2783.86209
1.24E-12	5319.0149	5668.45294	5363.56701	3897.86854	3300.22903	3784.47008
4.02E-34	281.196329	325.981319	312.576103	882.007321	804.649482	750.068164
3.52E-11	1783.16869	1852.55397	1847.58359	1321.93273	1284.71813	1331.04133
0.01785994	6.77581516	2.13059686	2.98639589	14.0172313	13.6051845	15.5133022
4.76E-07	13233.167	13000.902	14277.9587	17815.9009	19783.8818	16519.3399
1.65E-53	1260.30162	1302.85998	1173.65358	2875.68891	2873.60932	3216.68322
0.00010916	2825.51492	2928.50538	3181.50708	2496.14541	2432.41262	2226.93454
0.02300757	19730.0444	19321.3176	19437.4554	17696.2153	18172.6393	17729.3775
1.80E-07	5974.01036	5751.54622	6450.61511	4405.72361	4861.90985	4110.24943
1.30E-11	14362.4695	14897.1332	15076.3219	18742.1164	20189.122	19691.0345
0.00033637	4921.50041	4811.953	4065.48027	3681.14058	3253.58269	3830.23432
9.18E-21	10338.7646	9950.95262	9509.67997	15938.6702	14654.7273	16659.7353
0.00177152	5399.19538	5357.3858	5082.8458	6563.29898	5695.7133	6819.64767
2.58E-17	2335.39762	2437.40281	2365.22554	3446.08239	3398.38072	3453.26108
3.43E-19	1782.03939	1797.15845	1907.31151	3053.59992	3114.61545	2756.71381
8.03E-08	5190.27441	5903.88389	6117.13424	8040.4995	8043.57942	7342.44595
0.00136933	2606.43023	2645.136	2865.94459	2248.14824	2367.3021	2245.5505
2.71E-10	123.093975	145.945885	120.451301	47.4429366	28.1821678	47.3155718
4.36E-14	2003.38268	2102.8991	1934.18907	1339.18471	1205.03063	1365.94626
4.24E-10	8723.86202	9808.20263	10807.7667	15129.9838	15859.7579	13413.5768
1.08E-08	3665.716	3726.4139	4069.46213	5590.71878	5716.12108	4840.1503
5.34E-06	364.764716	395.225717	378.276812	252.310163	261.413902	251.315496

8.11E-41	1020.88948	957.703287	970.578663	2242.757	2039.80587	2005.09432
0.0715137	337.661455	305.740649	360.358437	402.186712	391.634953	393.262212
0.00022467	21898.3053	20773.3194	20781.3335	25153.3824	23775.0599	25136.2036
5.63E-223	1378.87838	1286.8805	1405.597	29.1127111	39.8437545	46.5399067
1.27E-06	4161.47981	4269.7161	3988.82944	3250.9194	3074.77169	3419.90748
7.52E-06	136.645606	115.05223	121.446766	224.2757	215.739354	198.570269
3.00E-07	2247.31203	2337.26475	2606.12814	1834.1008	1768.67398	1767.74079
3.18E-07	4556.73569	4541.3672	4792.16993	5967.02752	5847.31393	5601.85344
4.24E-43	2900.04889	2829.43263	2841.05795	5127.0719	5013.51048	4936.33277
2.02E-22	162.619564	161.925361	141.356072	31.2692082	23.3231734	31.8022696
1.45E-09	193.110732	198.145508	191.129337	100.277116	88.4336991	85.3231624
9.96E-29	1394.68862	1393.41034	1395.64234	770.947719	722.046576	767.132796
9.97E-12	554.48754	548.628691	641.07965	323.474568	332.355221	345.94664
4.03E-06	2198.75202	2081.59313	2552.37302	3208.86771	3563.58653	2868.40959
7.66E-16	86.9562945	86.2891727	72.6689666	221.040955	241.977924	216.410566
2.76E-10	2322.9753	2220.08193	2415.99427	3185.14624	3210.82354	3048.36389
5.86E-23	1195.93138	1279.42341	1328.94617	2303.13892	2285.67099	2080.33383
1.67E-17	1023.14809	1095.12678	1044.24309	1643.2508	1626.79134	1687.84728
0.00042829	22.5860505	28.7630576	26.877563	70.0861563	65.1105257	48.091237
6.71E-09	1002.82064	905.503664	928.769121	1301.44601	1385.78522	1604.85112
0.00071367	5102.18881	4678.7907	4375.06997	3924.82475	3822.08504	4007.86164
4.37E-08	1616.03192	1474.37303	1504.14806	1090.10929	1112.70973	1163.49767
4.59E-10	1173.34532	1188.87305	1159.71707	1655.11154	1580.145	1789.45941
2.52E-17	805.192701	872.479413	590.31092	1838.41379	1525.72426	2098.94979
1.02E-09	423.488447	657.289131	684.880123	1211.95138	1227.382	1040.16692
0.02137053	13592.2852	14437.9896	12754.8968	12233.8081	11344.7803	12453.3034
7.15E-05	5512.12563	5043.12276	4622.94083	3825.62589	3608.28928	4264.60679
1.98E-19	6884.2282	7238.70282	6728.34993	4548.05242	4468.3313	4827.73966
0.0091817	0	0	0	3.23474568	4.85899446	6.98098601
3.80E-05	3190.27964	3255.552	2823.13958	4168.50893	3628.69706	4367.77025
0.01111938	1380.00769	1417.91221	1438.44735	1628.15532	1553.90643	1712.66857
3.19E-59	2292.48413	2281.86923	2142.24132	958.562969	905.716567	977.338041
8.10E-07	1558.43749	1767.33009	1754.00985	2285.88694	2139.90116	2346.38696
2.51E-10	316.204707	397.356314	353.39018	643.71439	652.077056	563.132872
0.00188116	9281.73746	9020.9471	7988.609	7114.28399	6804.53584	7829.56364
1.38E-20	4291.3496	4479.57989	3942.04257	6806.98315	6820.08462	7688.39259
1.54E-14	3098.80613	2906.13411	2565.31407	4607.35609	4445.00813	5307.87636
2.28E-15	1370.97327	1376.36557	1351.84187	892.789807	902.80117	892.790544
5.39E-35	3992.08443	3945.86538	3806.65929	2319.31265	2175.85772	2255.63415
1.41E-18	1542.62725	1459.45885	1478.26596	2299.90418	2239.99644	2298.29573
3.68E-100	116.31816	167.251853	129.410488	1069.62257	950.419316	988.973018
3.17E-05	891.019693	945.985005	943.7011	718.11354	688.033615	733.779196
4.46E-13	341.049363	337.699602	369.317625	181.145758	172.008404	176.07598

1.58E-10	645.961045	640.244356	597.279177	381.69999	292.511466	383.178565
0.00158387	763.408508	824.540984	871.032134	664.201112	686.090017	587.954155
0	4787.11341	4898.24218	4870.81169	779.573708	806.59308	761.70314
2.95E-05	5739.11544	5650.34287	6733.32726	8291.73142	9529.45993	7222.21786
0.00161896	7.90511768	19.1753717	9.95465295	0	1.94359778	0
1.52E-06	1707.50542	1795.02785	1563.87598	1245.37709	1108.82253	1311.6497
9.63E-30	1777.52218	1821.66031	1837.62894	3038.50444	2974.67641	2982.43236
3.70E-06	542.065213	638.113759	654.020699	852.89461	906.688366	832.288665
3.09E-07	778.089441	820.27979	738.635249	511.089817	550.038172	567.786862
2.22E-08	282.325632	222.647372	227.961553	109.981353	132.164649	125.657748
5.39E-12	1429.697	1339.08013	1427.49723	873.381333	970.827092	950.189763
0.00015111	1410.49886	1513.78907	1625.59483	1923.59543	1955.25937	1821.26168
1.17E-35	3352.8992	3024.38224	3253.18059	1743.52792	1674.40949	1749.12483
0.00243141	48.5600086	26.6324607	25.8820977	73.320902	57.3361346	79.1178415
0.00016524	1937.88314	2221.14722	2694.72455	3426.67392	3529.57357	2749.73282
2.03E-05	1530.20492	1773.72188	1574.8261	1291.74177	1225.4384	1238.73718
2.91E-17	817.615029	872.479413	912.841676	490.603094	504.363625	451.437095
0.00638949	354.600993	307.871246	291.671332	217.806209	243.921522	252.866827
0	1601.35098	1544.68272	1574.8261	8765.08253	9229.17407	9015.5556
0.00410125	3357.41641	3582.59862	3534.89726	3135.54681	2860.97594	3053.01788
0.01597592	878.597365	733.990617	663.975352	1025.41438	797.84689	1111.52811
1.95E-24	164.878169	171.513047	153.301656	398.951967	441.196697	476.258379
6.35E-06	4159.2212	4287.82618	5043.02719	3148.48579	3539.29156	2606.23478
3.07E-165	1016.37227	989.66224	878.995856	4060.68407	4069.89376	4159.11633
0.00508048	123.093975	94.8115602	88.5964113	58.2254222	58.3079335	68.2585299
0.00023828	10451.6949	10468.6877	11124.3247	12645.6991	12836.4916	12109.6837
0.00108307	2265.38087	2503.45131	2491.64963	2845.49795	2938.71985	2824.19667
0.00140047	2272.15668	2216.88603	1864.5065	2810.99399	2383.82268	2902.53885
2.73E-88	293.618657	218.386178	253.84365	1191.46466	1300.26692	1248.82083
3.77E-30	5315.62699	5541.68243	5050.99091	9350.5715	8774.37219	9884.30053
0.02471001	2373.79391	2315.95878	2733.5477	2888.62789	2981.479	2715.60356
7.60E-08	194.240035	207.733194	208.052247	85.1816361	120.503063	94.6311437
6.51E-23	1702.98821	1597.94764	1393.65141	3194.85048	2890.1299	3644.85036
0.00590338	1689.43658	1753.48121	1903.32964	2022.7943	2189.4629	2065.59619
0.00138994	1190.28486	1261.31334	1223.42685	1424.36635	1613.18616	1438.08312
1.22E-27	860.528525	807.496209	698.816637	313.770331	215.739354	265.277468
8.20E-09	1124.78532	1297.53349	1334.91896	1955.94289	1952.34397	1692.50127
6.65E-09	2215.69156	2443.7946	2112.37736	3214.25895	2987.30979	3468.77438
6.45E-17	4689.99339	5021.81679	4771.26516	3304.83183	3030.06894	3326.82767
8.09E-25	579.332196	503.886157	545.514982	229.666943	169.093007	203.999925
9.13E-14	4547.70127	4566.93436	4282.4917	6039.27018	6123.30481	6337.95963
5.19E-05	527.38428	511.343246	515.651023	339.648296	415.929925	330.433338
3.68E-42	402.031699	378.180942	427.054612	72.2426534	69.9695202	98.5094693

0.00016728	3285.14105	3300.29453	3561.77483	2937.14907	2680.22134	2714.05223
2.72E-22	7457.91388	8236.88745	9077.64803	15773.6982	15554.6131	13765.7287
9.98E-07	68091.2958	68832.1274	67851.91	58742.9815	55479.9987	56131.7815
1.74E-85	10474.2809	11477.5253	11686.7626	27690.5012	27347.3926	26200.4162
8.79E-10	27800.0403	28686.3561	25325.6326	20235.4907	18425.307	20647.4296
8.72E-06	7354.01805	7271.72708	6958.30242	5583.17104	5996.97096	6044.75822
0.00283504	5837.36476	5700.41189	5503.92762	4779.87586	4895.92281	5151.19201
0.00258395	11246.7239	10788.2772	9109.50292	8037.26476	7796.74251	9266.09543
6.13E-05	2816.4805	2573.761	3201.41639	1236.7511	1775.47657	1025.42928
0.04485032	4823.25109	4673.46421	4050.54829	4980.43009	4904.669	5660.02832
0.07083023	5749.27916	5610.92682	5781.66244	5343.79986	5198.15227	5307.87636
3.63E-25	1290.79279	1313.51296	1273.20011	2214.72254	2213.75787	2083.43649
9.39E-06	1047.99274	1081.27791	1093.02089	851.816361	803.677683	828.41034
1.81E-06	4508.17568	4060.91761	3655.34856	5541.11934	5190.37788	5930.73545
3.95E-11	3173.3401	3043.55761	2719.61119	1947.3169	1910.55662	2138.50871
8.20E-05	10584.9526	9929.64665	8866.60939	7475.49726	7274.8865	8294.96271
7.56E-13	3376.61455	3358.88595	2761.42073	1934.37791	1596.66558	1972.51638
0.00074878	4397.50404	4475.3187	3972.90199	5376.14731	4810.40451	5390.87253
4.86E-08	6840.1854	7134.30358	7072.78092	8572.07604	8674.2769	8927.12978
0.0129829	3829.46487	3637.99413	3673.26694	3261.70189	3245.8083	3401.29152
9.31E-09	1442.11933	1579.83757	1709.21391	1120.30025	1101.04814	968.805725
9.94E-24	339.92006	349.417885	390.222396	138.015816	125.362057	124.882083
0.00056495	826.649449	851.173445	908.859815	698.705066	698.723403	665.520666
1.57E-13	1167.69881	1067.42903	1129.85311	667.435858	745.36975	635.269727
9.10E-07	1326.93047	1458.39355	1300.07768	1903.10871	1720.08404	1839.10198
1.10E-05	444.945195	385.638031	397.190653	268.483891	292.511466	266.053134
0.00039344	3076.22008	2898.67702	2394.09404	3424.51742	3504.3068	4096.28746
0.01215735	1290.79279	1344.40662	1418.53805	1600.12086	1677.32489	1475.31504
6.94E-06	2338.78553	2282.93453	2159.16423	1775.87538	1730.77383	1856.16661
0.00721116	2889.88516	3247.02961	3418.42782	2740.90784	2811.41419	2281.2311
2.90E-35	927.157374	1008.83761	1052.20682	422.673435	426.619713	442.904779
4.03E-11	1199.31928	1222.9626	1335.91443	1815.77057	2061.18545	1801.09439
5.76E-12	4953.12088	4591.43623	5609.44694	8150.48086	8495.46591	7195.84525
0.0040651	710.331289	736.121214	771.485604	894.946304	938.757729	856.334284
2.91E-08	1335.96489	1348.66781	1569.84877	2065.92424	2304.13517	1919.77115
1.98E-08	3843.0165	3820.16017	3777.7908	5054.82924	4699.61944	4854.11227
1.10E-07	2001.12408	2069.87485	2066.58595	2625.53524	2611.22362	2582.18916
7.89E-05	618.857784	621.068984	622.16581	487.368349	438.2813	473.155718
7.36E-13	230.377715	213.059686	228.957018	406.499707	413.014529	441.353449
5.26E-15	5976.26897	6120.13947	5893.15455	4234.28209	4432.37474	4390.26454
4.30E-05	35059.1969	37092.626	36728.6875	44190.9389	41529.8256	43051.7407
5.43E-29	10425.7209	10892.6764	10860.5264	16703.1484	16248.4775	17329.9099
2.32E-18	1778.65148	1977.19388	2033.7356	3075.16489	3077.68709	3003.37531

5.86E-10	76.7925718	69.2443979	81.6281542	21.5649712	19.4359778	13.961972
0.05872916	29.3618657	47.9384293	34.8412853	58.2254222	73.8567157	48.091237
3.98E-07	1935.62453	1874.92523	1523.0619	1160.19545	977.629685	1286.82842
1.33E-07	1696.21239	1665.06144	1858.53371	1245.37709	1349.82866	1211.58891
5.18E-05	83.5683869	79.8973822	87.600946	39.8951967	34.9847601	41.8859161
3.89E-06	1736.86729	1986.78157	2044.68572	2610.43976	2462.53839	2510.0523
1.93E-16	603.047549	544.367497	557.460565	305.144342	251.695913	293.201412
1.70E-24	2779.21352	2636.61361	2519.52266	4369.06316	4171.93264	4493.428
2.91E-48	2230.37249	2424.61922	2087.49072	912.198281	790.072499	912.182172
0.00683653	3359.67502	3282.18446	3724.03567	2969.49653	3091.29227	2818.76702
0.00291755	4451.71056	4524.32243	4600.04513	4112.44	3879.42117	3879.87689
7.50E-07	2420.09531	2919.98299	3144.67487	3991.67616	4059.20397	3869.79325
0.00125385	4210.03982	4254.80192	3522.95168	3220.72844	2722.98049	3416.02915
8.95E-15	2618.85256	2742.07816	2704.67921	3699.47081	3758.91811	3823.25334
2.44E-20	822.132239	901.242471	897.909697	1518.17397	1432.43157	1504.01465
3.80E-06	562.392658	519.865633	587.324524	398.951967	391.634953	321.125356
5.57E-56	449.462405	439.968251	466.873224	90.5728789	96.2080902	76.7908461
1.42E-09	1088.64764	1166.50178	1098.99369	1559.14742	1532.52685	1539.69525
2.73E-07	561.263356	543.302199	579.360802	815.15591	787.157102	765.581466
1.50E-06	6130.98341	6025.32791	6242.56287	5192.84506	4868.71245	4889.0172
0.02102238	451.72101	470.861906	419.090889	538.046031	530.602195	539.862918
8.55E-43	3035.56519	3009.46806	2921.69064	5158.3411	5235.08063	5297.79272
9.23E-05	796.158281	934.266722	991.483434	1211.95138	1403.2776	1140.22772
0.00759378	362.506111	334.503707	384.249604	477.664112	466.463468	425.064482
8.58E-17	1363.06815	1411.52042	1398.62874	838.877379	905.716567	885.033893
2.08E-10	208.920967	189.62312	225.970622	83.025139	101.067085	93.8554786
0.0003043	1111.23369	1251.72565	1265.23639	1498.7655	1540.30124	1474.53938
4.44E-06	376.057741	394.160419	408.140771	273.875134	266.272896	250.539831
1.12E-09	2416.70741	2163.62111	2377.17113	1676.67651	1680.24028	1488.50135
5.41E-81	5610.37495	6113.74768	6006.63759	12923.8872	13467.189	13501.2269
4.43E-10	722.753617	693.509277	700.807568	405.421458	392.606752	484.790695
3.28E-19	6267.62902	6649.59279	6522.28862	9187.75597	9316.63597	9735.37282
1.30E-18	2071.14083	1998.49985	1894.37046	1257.23782	1107.85074	1222.44822
0.02361322	2.25860505	9.58768586	8.95918766	0	0.97179889	0.77566511
3.74E-17	657.25407	775.537256	736.644319	376.308747	379.001568	392.486547
1.41E-05	391.867977	449.555937	497.732648	278.188128	325.552629	258.296482
1.38E-17	20902.2605	20829.7802	19924.2379	29244.2574	28264.7708	30853.6312
1.53E-10	1252.3965	1253.85625	1439.44282	856.129356	906.688366	799.710731
0.00118167	2149.06271	2121.00917	1738.08241	1539.73894	1284.71813	1701.80926
8.60E-22	1242.23278	1209.11372	1359.80559	2265.40022	2385.76628	2097.39846
3.40E-09	3034.43589	3006.27217	2745.49328	3894.63379	3854.1544	3916.33315
6.33E-16	1041.21693	1030.14358	852.118293	460.412135	423.704317	546.068239
6.62E-13	976.846685	950.246198	1002.43355	1733.82368	1762.84319	1413.26183

2.24E-07	2473.17253	2917.8524	2823.13958	2123.07141	1916.38741	1825.91567
0.01946974	1272.72395	1217.6361	1153.74428	1048.0576	1010.67085	1087.48249
1.76E-19	677.581516	679.660398	661.984421	327.787562	310.003846	368.440928
2.60E-17	54.2065213	50.0690262	64.7052442	181.145758	231.288136	186.935292
9.54E-06	58.7237314	77.7667853	58.7324524	132.624573	125.362057	132.638734
5.74E-07	150.197236	185.361927	176.197357	292.205359	340.129612	270.707124
2.05E-11	936.191794	838.389863	852.118293	1265.86381	1357.60305	1306.99571
1.68E-13	343.307968	373.919749	285.69854	711.644049	617.092296	880.379902
7.41E-08	1477.1277	1581.96817	1664.41797	1145.09997	1162.27147	1172.80565
0.00077915	2006.77059	1992.10806	1874.46115	1628.15532	1580.145	1694.82827
9.33E-32	5889.31267	6300.17491	6607.89863	10657.4088	11036.72	10507.1596
4.25E-07	5303.20466	5251.92125	4586.10862	3667.12335	3550.95315	4003.98331
0.00126998	2546.5772	2704.79271	2897.79948	3463.33437	3813.33885	2998.72132
0.00130211	15.8102354	9.58768586	13.9365141	1.07824856	0	2.32699534
0.03334382	578.202893	517.735036	515.651023	697.626818	567.530553	676.379978
2.53E-21	2437.03485	2242.45319	2009.84443	4008.92814	3791.95927	4338.29497
0.01074156	892.148996	922.548439	979.537851	1237.82935	1108.82253	1025.42928
1.46E-28	475.436364	461.27422	474.836946	156.346041	187.557186	176.851646
7.39E-15	88.085597	131.031707	81.6281542	327.787562	254.61131	331.984668
1.54E-09	2944.09169	2874.17516	2900.78587	2228.73977	2205.98348	2015.95363
1.33E-07	1.12930253	5.32649214	0	31.2692082	35.956559	38.0075905
0.00099819	712.589894	752.100691	731.666992	583.33247	615.148698	504.957988
0.00406007	3054.76333	3679.54077	3967.92467	2823.93298	3110.72825	2610.1131
4.40E-07	592.883826	604.024209	541.533121	801.138679	796.875091	895.11754
2.88E-23	10683.2019	10838.3462	10406.5942	7003.22439	7181.59381	7386.65886
1.64E-43	3392.42479	3141.56507	3267.1171	5799.899	5986.28117	5972.62136
0.00026778	203.274455	201.341403	145.337933	115.372596	92.3208947	114.022772
1.50E-05	70.0167566	43.6772356	52.7596607	16.1737284	20.4077767	16.2889674
7.96E-11	5046.85299	5160.30559	4249.64135	8345.64384	6746.2279	8835.60129
2.18E-06	1114.62159	1075.95141	1077.09345	836.720882	811.452074	836.942656
0.00012976	274.420514	319.589529	332.485409	527.263545	498.532831	383.178565
3.80E-11	1400.33513	1234.68088	1255.28174	1941.92565	2048.55206	1821.26168
3.35E-07	2906.8247	3079.77776	2556.35488	1937.61266	1431.45977	2031.46693
1.17E-11	9630.69194	9579.16347	9707.77756	12477.4923	12561.4725	12138.3833
0.0144978	3529.07039	3473.93818	4041.5891	4301.1335	4660.74748	4007.08597
2.22E-24	4040.64444	3853.18442	4019.68886	2565.15332	2563.60548	2434.81279
1.14E-10	284.584237	312.13244	331.489943	529.420042	499.50463	560.805876
0.02068548	1246.74999	1417.91221	1589.75808	1076.09206	1325.53369	1061.10987
0.00087209	1245.62069	1265.57453	1396.63781	1609.8251	1696.76086	1511.7713
8.35E-11	4347.81473	4648.96234	5457.14075	7808.67606	7598.49553	6870.84156
4.00E-11	1652.1696	1731.10995	1796.81486	2585.64004	2620.94161	2317.68736
0.00089766	25209.4203	24587.0877	26138.9277	22049.1048	22579.7472	21859.0185
0.00109205	7495.18087	6892.48083	5834.4221	5056.98574	4492.62627	5867.90657

6.32E-21	2006.77059	2158.29462	2157.1733	3289.73635	3326.4676	3204.27258
7.53E-35	3268.20151	3299.22923	3150.64766	1893.40447	1854.19228	1876.33391
0.05246003	59.8530339	47.9384293	93.5737378	33.4257053	34.0129612	52.7452276
5.21E-16	1176.73323	1203.78722	1098.99369	720.270037	708.441392	639.923718
2.42E-40	1096.55275	1051.44955	1097.00276	2102.58469	2158.36534	2060.16654
0.00238156	21.456748	34.0895497	30.8594242	56.0689251	75.8003135	51.1938974
6.53E-40	1088.64764	1283.68461	1131.84404	2595.34428	2502.38215	2799.37539
0.00675758	1382.26629	1302.85998	1343.87815	1127.84799	1128.25851	1189.87028
0.00022456	2877.46284	3217.20125	3539.87459	2408.80728	2719.0933	2256.40981
0.02256078	1549.40307	1374.23497	1451.3884	1231.35985	1319.70289	1246.49384
2.28E-23	2572.55115	2419.29273	2589.20523	4019.71063	3867.75959	3992.34833
8.77E-27	933.933189	942.789109	993.474365	433.455921	359.56559	458.418081
5.07E-26	376.057741	366.46266	325.517152	121.842087	99.1234869	119.452427
1.98E-24	2814.2219	2600.39346	2589.20523	1486.90476	1477.13431	1632.77506
0.02474147	670.805701	662.615623	680.898262	782.808454	747.313347	819.102359
7.43E-09	1253.5258	1191.00364	1216.45859	898.181049	831.859851	888.136554
1.15E-12	1769.61706	1763.0689	1861.5201	2481.04993	2613.16722	2509.27664
8.18E-33	7531.31855	7554.03116	7435.13029	11843.4822	11956.0418	12730.2158
1.99E-11	289.101447	300.414157	309.589707	118.607341	152.572426	155.908688
0.06280397	809.709911	816.018596	705.784894	832.407887	897.942176	963.376069
3.52E-12	1129.30253	1208.04842	1096.00729	780.651957	733.708163	707.406582
1.98E-07	196.49864	220.516775	142.351537	99.1988674	67.0541235	70.5855252
0.07480338	42.913496	37.285445	16.92291	64.6949135	34.0129612	70.5855252
1.83E-18	1386.7835	1483.96071	1596.72633	2432.52875	2482.94617	2341.73297
6.01E-06	778.089441	837.324565	792.390375	1027.57088	1071.89418	1060.33421
1.67E-10	2879.72144	3065.92888	3041.14648	4036.9626	4099.04772	3885.30655
1.02E-08	81.3097819	87.3544712	122.442231	215.649712	206.021365	218.737562
0.00013905	5263.67907	5068.68992	4937.50787	5854.88967	6145.65619	6040.10423
0.00626753	3258.03779	3259.81319	3336.79967	3570.08098	4121.3991	3758.87313
1.45E-24	661.77128	664.74622	602.256504	1170.97793	1230.2974	1306.99571
1.34E-06	745.339667	744.643602	699.812103	479.820609	559.756161	492.547346
0.00791871	5985.30339	5782.43987	5564.651	6175.1295	6979.45964	6781.64008
0.04991156	210.05027	253.541026	195.111198	284.65762	237.118929	325.003682
0.00022796	284.584237	269.520502	283.707609	199.475983	195.331577	186.159627
0.02881475	274.420514	263.128712	321.53529	342.883042	360.537389	363.786938
5.20E-10	10704.6586	11764.0905	8569.96073	5488.28516	4787.08134	6873.16856
0.00405259	5442.10887	5157.10969	5838.40396	4716.2592	4898.83821	4400.34818
4.87E-05	348.954481	413.33579	442.982056	553.141511	620.007693	554.600555
0.00231202	485.600086	527.322722	470.855085	346.117787	380.945165	410.326844
5.47E-20	1582.15284	1661.86555	1566.86238	2773.25529	2518.90273	3008.80497
9.44E-08	188.593522	193.884314	159.274447	84.1033876	79.6875091	96.958139
0.0004855	55.3358238	52.199623	70.678036	86.2598847	134.108247	121.003758
4.63E-51	1237.71557	1218.7014	1374.73757	3022.33071	3101.98206	2807.13204

8.55E-08	294.747959	311.067141	273.752956	167.128527	172.008404	172.97332
0.00125875	2055.3306	2016.60993	1617.63111	1452.40081	1317.7593	1597.09447
0.00012524	936.191794	1017.36	1109.9438	1298.21126	1553.90643	1273.64211
2.39E-16	756.632693	845.846952	719.721409	1596.88612	1267.22575	1528.83594
2.14E-14	2163.74364	2185.99238	2008.84897	3024.48721	3029.09714	3136.01405
2.07E-05	236.024228	289.761173	317.553429	426.986429	440.224898	414.20517
2.56E-13	2782.60142	2622.76473	2504.59068	3951.78097	3649.10484	4091.63347
0.00135735	871.82155	850.108146	922.796329	1040.50986	1147.69449	1059.55854
0.00259187	1300.95651	1248.52976	860.082015	531.576539	387.747758	739.208852
3.71E-17	1118.0095	1064.23313	1147.77149	653.418627	687.061816	670.174657
1.25E-40	8369.26102	8237.95275	7746.71093	14269.5414	14086.2249	15085.9108
1.23E-17	193.110732	194.949612	116.46944	33.4257053	34.0129612	31.0266045
5.68E-18	291.360052	303.610052	323.526221	122.920336	103.010682	128.760409
3.14E-210	2388.47484	2470.42706	2287.57925	304.066094	327.496226	362.235607
0.0006359	267.644699	245.018639	283.707609	147.720053	214.767555	145.049376
2.66E-84	1051.38065	1022.68649	879.991321	212.414966	194.359778	191.589283
7.38E-26	111.80095	150.207078	146.333398	8.62598847	8.74619002	15.5133022
1.87E-67	622.245692	706.292858	748.589902	2036.81153	1976.63894	1989.58101
4.06E-14	271.032606	229.039162	338.4582	603.819193	706.497794	574.767848
4.01E-10	907.959231	874.61001	1007.41088	1360.74968	1349.82866	1362.8436
4.32E-13	1905.13336	1746.02412	1803.78312	1241.06409	1265.28216	1207.71058
0.00924832	1081.87182	1143.06521	1047.22949	1249.69008	1232.24099	1394.64587
1.86E-12	683.228028	630.65667	664.970817	380.621741	409.127333	401.794528
3.35E-09	8512.68244	8126.09641	7785.53408	10287.5695	10716.0264	10534.3079
0.00220279	160.360959	175.774241	163.256308	225.353949	231.288136	241.23185
2.56E-09	2463.00881	2618.50354	2546.40023	1883.70023	1812.40493	1965.53539
0.00020473	400.902397	343.026094	343.435527	280.344625	233.231734	234.250864
8.14E-08	1372.10257	1351.86371	1157.72614	1754.31041	1807.54594	1974.06771
6.93E-09	222.472598	251.410429	279.725748	452.864395	444.112093	404.897189
1.32E-23	1626.19564	1649.08197	1672.3817	2843.34145	2582.06965	2710.94957
1.47E-09	1133.81974	1081.27791	1199.53568	1573.16465	1610.27076	1580.02983
3.80E-05	1544.88586	1390.21445	1428.4927	1032.96212	1209.88962	1085.93116
0.07998918	71.1460592	54.3302199	44.7959383	40.9734452	33.0411623	37.2319254
1.44E-07	15330.2818	15321.122	17020.4656	12055.8971	12626.583	10867.0682
2.50E-09	54.2065213	58.5914136	66.6961748	11.8607341	14.5769834	5.42965579
2.77E-08	968.941568	1100.45328	1028.31565	1372.61042	1513.09087	1468.33406
8.75E-08	696.779659	726.533528	717.730478	521.872302	481.040451	480.91237
0.01961995	2.25860505	0	7.96372236	12.9389827	13.6051845	18.6159627
3.32E-20	251.834463	258.867518	299.635054	84.1033876	99.1234869	78.3421763
0.05022164	1051.38065	1046.12306	921.800864	834.564384	814.367471	944.760107
0.013135	7635.21438	7337.77558	6102.20226	5726.5781	5396.39924	6407.76949
4.16E-29	848.106197	865.022324	871.032134	1674.52001	1614.15796	1529.6116
2.10E-12	311.687497	360.070869	386.240535	188.693498	145.769834	138.844055

4.13E-15	512.703347	531.583916	559.451496	302.987845	269.188293	281.566436
4.17E-09	4300.38402	4996.24963	5006.19497	3337.17929	3480.01183	3488.94168
1.10E-10	1290.79279	1467.98123	1665.41344	843.190373	970.827092	788.075754
0.00450647	2992.65169	3165.00163	3372.63642	2787.27252	2757.96525	2645.01803
5.27E-14	449.462405	498.559665	514.655558	820.547153	904.744768	805.140387
0.0095031	400.902397	365.397361	329.499013	301.909596	276.962684	271.482789
2.23E-23	6664.01421	6777.4286	6883.64252	9944.68646	9993.008	9699.69223
5.14E-30	2920.37633	2788.95129	3082.95602	5299.59167	5033.91826	4911.51149
1.64E-12	16.9395379	14.914178	9.95465295	69.0079078	81.6311069	101.61213
9.84E-156	5664.58147	5064.42873	4836.96587	19583.1503	20148.3064	20974.7603
7.44E-13	661.77128	549.693989	535.560329	960.719466	982.488679	1045.59657
0.0008394	547.711725	566.738764	455.923105	787.121448	598.628117	829.186005
0.00449707	2844.71306	2808.12666	2545.40476	3344.72703	3020.35095	3275.63377
0.0089217	3044.59961	3021.18634	2849.02168	2308.53016	2362.4431	2806.35638
1.41E-12	2469.78462	2453.38228	2439.88544	3420.20443	3267.18787	3342.34097
9.26E-09	201.01585	198.145508	197.102129	366.60451	318.750036	346.722305
0.0159432	1487.29143	1460.52415	1355.82373	1131.08274	1206.97422	1320.18202
2.63E-35	1697.3417	1529.76854	1527.04376	737.522014	613.2051	704.303922
2.34E-07	3176.72801	3215.07066	2301.51576	1695.00673	1462.55733	2019.83195
0.05400216	2728.3949	2648.33189	2517.53173	2299.90418	2372.16109	2447.99909
4.48E-13	1792.20311	1846.16218	1649.48599	2615.831	2521.81812	2832.72899
0.00039233	16.9395379	11.7182827	23.8911671	58.2254222	41.7873523	45.7642416
1.55E-08	4589.48547	4602.08921	4153.08121	3299.44059	2893.0453	3372.59191
0.00540951	88.085597	88.4197696	104.523856	145.563555	162.290415	122.555088
0.00116924	280.067027	282.304084	242.893532	361.213267	360.537389	364.562603
0.00021496	2616.59395	2590.80578	2282.60192	1965.64712	1822.12292	2115.23876
9.89E-05	638.055927	654.093235	581.351733	773.104217	881.421594	824.532014
0.04127261	216.826085	219.451476	210.043177	158.502538	178.810996	172.97332
9.65E-41	692.262449	663.680921	758.544555	155.267792	226.429142	190.037953
0.016109	417.841935	378.180942	451.941244	525.107048	548.094575	475.482714
3.67E-23	563.521961	562.47757	546.510447	1041.58811	987.347674	1054.12889
0.07784721	256.351673	298.28356	226.966087	184.380504	164.234013	252.091161
0.04240934	1487.29143	1630.97189	1560.88958	1310.072	1383.84162	1438.85878
1.87E-08	1880.28871	2001.69575	2018.80362	1494.4525	1377.03903	1213.9159
3.45E-12	1604.73889	1530.83384	1787.85567	1053.44884	1102.99174	1064.21253
4.72E-07	1114.62159	1170.76297	956.642149	663.122864	527.686798	774.889447
6.13E-14	205.53306	237.56155	236.92074	451.786146	488.814842	419.634826
9.91E-14	408.807514	452.751832	436.013799	713.800546	806.59308	726.022545
0.00176745	169.395379	176.839539	189.138406	103.511862	141.882638	96.958139
8.60E-05	45.172101	43.6772356	16.92291	4.31299423	5.83079335	10.0836465
3.83E-06	66.628849	50.0690262	48.7777995	9.70423703	20.4077767	15.5133022
0.00096607	25.9739581	20.2406701	17.9183753	3.23474568	5.83079335	5.42965579
2.92E-06	91.4735046	86.2891727	71.6735013	35.5822024	32.0693634	31.0266045

0.01680041	4651.59711	4805.56121	4795.15633	5660.80493	5106.80317	5275.29843
0.07325595	400.902397	360.070869	375.290416	324.552816	298.34226	335.862994
0.02164102	326.36843	304.675351	339.453666	259.857903	255.583108	263.726138
4.24E-15	12.4223278	12.7835811	7.96372236	72.2426534	103.010682	88.4258228
3.83E-18	3843.0165	3871.29449	3988.82944	5554.05833	5612.1386	5457.57973
0.00540237	37.2669834	43.6772356	55.7460565	12.9389827	18.4641789	27.923944
1.53E-32	12147.9073	12307.3927	13501.4958	6863.05208	7434.26152	6727.34352
2.07E-15	1137.20764	992.858136	1195.55382	604.897441	667.625838	604.243122
4.96E-26	220.213993	193.884314	198.097594	48.5211851	43.7309501	34.1292649
0.0803689	13591.1559	13277.8796	12613.5408	12051.5841	12014.3497	12419.1741
1.19E-57	17988.6599	17716.9782	17515.2119	9996.44239	10131.0034	9830.77963
2.86E-16	60023.5586	59888.9471	57228.3044	42822.6415	42353.9111	44430.0976
0.07133723	27353.9658	28629.8953	32789.6314	26266.1349	28241.4476	23933.9227
0.00126068	879.726668	812.822701	979.537851	687.92258	745.36975	653.110025
0.01232561	1369.84396	1545.74802	1663.42251	1255.08132	1335.25168	1267.43679
8.01E-100	2416.70741	2313.82819	2438.88997	822.70365	757.031336	781.094768
5.35E-13	747.598272	781.929047	706.78036	1280.95929	1128.25851	1164.27333
0.01350158	998.303433	982.205151	825.24073	731.052523	679.287425	846.250637
0.00035448	400.902397	361.136167	350.403784	476.585863	483.955848	555.37622
0.03330394	1961.59849	1922.86366	1815.7287	2101.50644	2148.64735	2124.54674
5.73E-53	2555.61162	2509.8431	2613.0964	5410.65127	5441.10199	4953.39741
6.00E-09	2161.48504	2249.91028	2806.21667	3815.92165	4261.33814	3436.19645
9.71E-07	5610.37495	5812.26823	5583.56484	4529.7222	3995.06524	4601.24545
0.00114988	12247.2859	12211.5159	13533.3507	14852.8739	16426.3167	14307.143
3.00E-20	12098.218	12539.6278	11995.3568	8610.89299	8024.14345	8438.46076
0.00191298	666.28849	730.794722	648.047907	555.298008	456.745479	560.030211
5.00E-30	1221.90533	1242.13797	1458.35666	573.628233	594.740921	507.284983
3.90E-13	1832.858	2002.76105	2096.44991	2906.95811	2933.86085	2846.69096
2.26E-06	27.1032606	29.828356	28.8684936	0	0.97179889	5.42965579
1.13E-41	13010.6944	11977.1502	12720.0555	6412.34418	6956.13646	6169.6403
0.00371092	11525.6616	11440.2398	12832.5431	14074.3784	15318.4659	13104.8621
4.68E-09	160.360959	136.358199	132.396884	48.5211851	65.1105257	57.3992183
2.21E-10	2068.88223	1931.38605	1908.30697	1404.95787	1187.53825	1376.02991
3.80E-41	1360.80954	1486.09131	1521.07097	628.61891	670.541235	622.859085
0.01426105	21.456748	39.4160419	26.877563	4.31299423	11.6615867	16.2889674
2.91E-12	789.382466	807.496209	823.249799	478.74236	533.517591	503.406658
1.37E-28	31.6204707	26.6324607	30.8594242	195.162989	170.064806	199.345934
0.00349763	4071.13561	3870.22919	3957.97001	3422.36093	3314.80602	3595.98346
8.94E-07	1412.75746	1367.84318	1273.20011	1881.54373	1688.01467	1842.20464
1.29E-10	4163.73841	4255.86722	4094.34876	5926.05408	5466.36876	5425.77746
8.25E-28	3870.11976	3746.65457	3731.00393	5793.42951	5845.37033	5960.21072
0.00159387	3822.68905	3914.97173	4385.02463	3373.83974	3506.2504	3295.0254
2.61E-08	367.023321	340.895497	298.639589	178.989261	198.246974	193.140613

3.94E-18	441.557288	449.555937	519.632884	196.241238	216.711153	158.235683
1.65E-30	1442.11933	1482.89541	1461.34305	2572.70106	2443.10241	2594.5998
0.00040378	1141.72485	1087.6697	1188.58556	1346.73245	1529.61145	1389.99188
1.40E-15	1062.67368	1002.44582	1104.96648	616.758176	633.612877	664.745001
3.40E-45	2114.05433	2038.98119	2250.74703	713.800546	906.688366	817.551028
5.34E-07	39390.0721	40106.3552	43660.1124	31729.6203	33856.5016	30350.2245
1.53E-40	111.80095	92.6809633	97.555599	494.916088	395.522149	434.372463
7.39E-10	622.245692	681.790994	604.247434	1000.61466	1049.5428	905.201186
0.00012088	1302.08581	1279.42341	1573.83063	1821.16182	2039.80587	1715.77123
6.21E-19	815.356424	836.259267	783.431188	436.690666	396.493948	459.193746
2.29E-05	322.980523	338.7649	326.512617	460.412135	443.140294	562.357206
3.48E-08	212.308875	224.777968	257.825512	407.577955	359.56559	445.231774
0.00014911	115.188858	121.444021	129.410488	175.754515	193.387979	225.718548
4.15E-25	4588.35616	4121.63962	4363.12439	6815.60914	7085.38572	7133.01637
1.29E-22	866.175038	942.789109	908.859815	1551.59968	1540.30124	1672.33398
2.56E-07	3692.81926	3617.75346	4122.22179	2815.30699	3014.52016	2759.0408
4.35E-06	736.305247	702.031665	741.621645	514.324563	542.263781	541.414248
0.07066219	2050.81339	2015.54463	1918.26162	1910.65645	1694.81727	1765.4138
8.32E-09	2771.3084	2914.6565	2854.99447	3909.72927	3559.69934	3809.29137
4.38E-09	781.477348	857.565235	902.887023	536.967782	589.881927	550.72223
3.49E-06	5063.79253	5123.02014	5229.1792	6009.07922	6438.16765	6604.78843
3.30E-29	1120.26811	1032.27418	1133.83497	556.376256	532.545792	508.060649
2.41E-06	823.261542	847.977549	747.594437	571.471736	492.702038	601.916127
1.07E-08	2116.31293	2148.70693	2097.44538	1544.05194	1620.96055	1641.30738
1.58E-09	869.562945	856.499937	714.744082	457.177389	380.945165	526.676611
9.75E-14	924.898769	873.544712	798.363167	438.847163	416.901724	531.330602
1.38E-17	1206.0951	1081.27791	989.492504	560.689251	510.194418	612.775439
2.07E-18	1542.62725	1474.37303	1555.91226	846.425119	975.686087	876.501577
2.66E-20	527.38428	532.649214	562.437892	1015.71014	1072.86598	938.554786
0.00064436	67.7581516	76.7014869	51.7641954	36.660451	22.3513745	28.6996092
2.22E-05	1068.32019	1029.07828	989.492504	795.747436	816.311069	769.459791
3.14E-06	860.528525	861.826429	947.682961	704.096309	563.643357	568.562527
1.61E-06	38.3962859	54.3302199	82.6236195	11.8607341	12.6333856	17.0646325
0.00329535	173.912589	145.945885	166.242704	216.72796	248.780516	219.513227
6.24E-06	504.798229	534.779811	517.641954	327.787562	382.888763	368.440928
0.0240113	112.930253	120.378722	109.501183	72.2426534	93.2926936	72.1368554
3.80E-08	1949.17616	1852.55397	1659.44065	2533.88411	2408.11765	2701.64159
0.00792679	356.859598	318.52423	363.344833	279.266377	275.990885	250.539831
0.0027025	470.919153	633.852565	613.206622	740.75676	829.916253	712.060573
0.00102126	2.25860505	8.52238743	9.95465295	28.0344625	27.210369	25.5969487
8.82E-05	48.5600086	43.6772356	52.7596607	84.1033876	95.2362913	123.330753
0.00044265	89.2148996	89.485068	89.5918766	45.2864395	56.3643357	37.2319254
1.99E-05	1058.15647	1028.01298	770.490139	562.845748	497.561032	715.163234

0.05480656	12.4223278	11.7182827	17.9183753	29.1127111	22.3513745	31.0266045
0.02788511	67.7581516	73.5055916	63.7097789	93.8076246	113.70047	92.3041484
8.51E-07	685.486633	630.65667	619.179414	880.929072	914.462757	871.071921
4.88E-20	1525.68771	1427.49989	1533.01656	905.728789	873.647203	918.387493
0.05445129	4.5172101	3.19589529	6.96825707	14.0172313	13.6051845	13.1863069
4.43E-07	1099.94066	948.115602	1009.40181	684.687835	737.595359	746.189838
0.00010494	1400.33513	1417.91221	1427.49723	1721.96295	1690.93007	1754.55448
1.10E-20	6598.51466	7183.30731	7093.6857	10558.2099	10259.2809	10252.7415
7.59E-11	756.632693	779.79845	687.866519	1220.57737	1064.11979	1297.68773
4.52E-07	403.161002	405.878701	282.712144	216.72796	189.500784	189.262287
0.01581566	15.8102354	25.5671623	15.9274447	40.9734452	38.8719557	36.4562603
1.21E-19	5551.65122	5571.51078	6242.56287	3597.03719	3774.46689	3466.44739
0.00019741	1224.16394	1243.20327	1035.28391	933.763252	813.395672	903.649856
0.00053008	559.00475	550.759288	567.415218	431.299423	451.886484	410.326844
7.39E-12	642.573137	646.636146	621.170344	351.50903	408.155534	382.4029
4.78E-17	4623.36454	4566.93436	4434.79789	3219.6502	3224.42872	3092.5768
1.16E-20	2883.10935	2847.5427	2938.61355	4277.41203	4335.19485	4190.9186
7.74E-09	562.392658	480.449591	402.167979	269.56214	206.021365	270.707124
1.62E-10	1059.28577	1107.91037	1177.63544	2016.3248	1748.26621	1578.4785
1.34E-07	1547.14446	1632.03719	1442.42921	2004.46407	2049.52386	2157.90034
2.14E-07	2424.61252	2558.84683	2746.48875	3395.40471	3524.71458	3265.55012
0.01765695	920.381559	840.52046	816.281542	694.392072	657.907849	784.973094
0.00019426	810.839214	882.067099	911.846211	1070.70082	1149.63809	1084.37983
2.08E-05	2150.19201	2123.13977	1930.20721	1658.34628	1546.13204	1667.67999
9.62E-250	6853.73703	6783.82039	7003.09835	6.46949135	7.77439113	10.0836465
0.07392063	70.0167566	55.3955183	59.7279177	74.3991506	103.010682	83.7718321
1.37E-20	589.495919	573.130555	658.00256	258.779654	304.173053	253.642492
9.82E-16	5359.66979	5681.23652	6169.8939	3707.01854	3933.84191	3663.46633
2.88E-20	9998.84457	10116.0739	9280.72295	6092.10436	5982.39397	6642.79602
1.74E-13	1015.24297	971.552167	1047.22949	1604.43386	1847.38969	1522.63062
2.06E-32	2653.86094	2563.10802	2756.4434	1330.55872	1491.7113	1434.20479
1.21E-23	7.90511768	7.457089	9.95465295	101.355365	127.305655	144.273711
5.58E-07	690.003844	684.98689	634.111393	1014.63189	930.983338	886.585223
1.02E-22	1461.31747	1506.33198	1520.07551	910.041784	819.226465	815.224033
2.53E-72	2278.9325	2369.22371	2399.07136	977.971443	951.391115	970.357055
1.04E-48	1786.5566	1971.86739	1798.80579	3737.2095	4009.64223	3850.40162
0.00773473	6234.87925	6361.96222	7363.45679	5531.41511	6058.19429	4978.99436
0.01128544	2313.94088	2156.16402	2367.21647	2628.76999	3077.68709	2471.26905
1.91E-14	1226.42254	1306.05587	1385.68769	2137.08864	2009.68011	1939.93845
6.23E-08	1290.79279	1393.41034	1492.20248	1910.65645	2052.43926	1846.08297
1.88E-06	32.7497733	34.0895497	32.8503548	5.39124279	3.88719557	6.98098601
2.47E-61	2868.42842	2698.40092	2702.68828	1235.67285	1257.50777	1201.50526
1.03E-13	1715.41054	1506.33198	1441.43375	814.077662	841.57784	997.505334

2.17E-09	9927.69851	9926.45076	9114.48025	12584.2389	12434.1668	13609.0444
1.27E-05	9824.93198	10209.8201	9076.65256	12096.8706	11537.1964	12682.1246
1.09E-27	13193.6414	12965.7472	12457.2527	19678.0362	19518.5807	20675.3536
1.12E-08	110.671648	90.5503664	93.5737378	210.258469	203.105968	191.589283
2.26E-10	14833.3887	14583.9355	12979.872	20517.9918	18723.6492	21945.1174
2.07E-12	10739.667	11152.6093	9118.46211	16565.1326	15155.2037	18605.1034
0.00025607	111.80095	102.268649	101.53746	164.972029	198.246974	155.908688
0.00181929	32.7497733	25.5671623	44.7959383	6.46949135	8.74619002	17.0646325
5.38E-34	682.098726	629.591371	780.444792	1659.42453	1887.23345	1600.97279
1.20E-10	3446.63131	3351.42886	3351.73165	2615.831	2422.69464	2510.82797
1.42E-24	5433.07445	6177.66559	5937.95049	9890.77403	9370.08491	9492.58964
4.03E-11	207.791665	249.279832	207.056781	103.511862	97.1798891	92.3041484
2.98E-24	1176.73323	1039.73127	1088.04357	1926.83017	1947.48498	1890.29588
5.21E-06	10282.2995	11521.2025	11948.5699	8941.9153	8978.44996	8796.04237
0.07864895	7941.25536	8481.90609	8027.43214	7412.95884	6926.0107	7874.55222
4.18E-24	10765.641	10813.8444	11020.7963	16099.3292	17485.5775	16187.3552
1.03E-05	2201.01062	2336.19945	2364.23008	1772.64063	1920.27461	1680.8663
1.44E-29	22421.1724	22246.6271	22037.6107	14961.777	15088.1496	15130.1237
3.63E-72	23891.5242	23211.7875	24296.3215	10674.6607	11557.6042	10431.1444
0.00027146	13356.261	12351.07	10832.6533	9463.7876	8948.32419	10338.8403
0.00053233	10012.3962	9263.83514	8895.47788	7986.58707	7984.29969	7919.5408
0.01740999	18391.8209	17771.3084	14699.0406	13854.4157	12683.9191	15753.7584
4.38E-07	4439.28823	4417.79258	4223.75925	3470.88211	3147.65661	3521.51961
3.62E-54	3488.4155	3274.72737	3157.61592	6491.05632	6721.93293	6739.75416
2.21E-15	702.426171	762.753675	806.326889	434.534169	397.465747	435.148128
7.83E-10	4810.82876	4754.42689	4466.65278	5959.47978	6276.84904	6520.24093
2.30E-06	248.446556	302.544754	326.512617	455.020892	454.801881	454.539756
3.97E-12	5084.11997	4849.23845	4639.86374	6915.88626	6429.42146	7062.43085
2.04E-66	1261.43092	1263.44394	1205.50847	2883.23665	2830.85017	2759.81647
3.78E-05	8729.50853	8599.08892	7969.69516	6975.18993	6432.33686	7133.01637
2.47E-16	877.468063	902.307769	839.177244	531.576539	480.068652	507.284983
2.20E-19	4321.84077	4101.39895	3520.96075	7597.33934	6648.07622	8230.58251
0.0013926	633.538717	657.289131	787.413049	548.828516	549.066374	469.277393
9.25E-23	426.876355	536.910408	529.587537	187.615249	190.472583	200.897264
9.30E-08	4494.62405	4364.52766	4149.09935	5685.60465	5328.37332	5912.11949
3.07E-37	3914.16256	3951.19187	3498.06505	7749.37239	7021.24699	7883.8602
3.26E-07	7389.02643	7466.67669	8431.59105	10174.3534	11148.4769	9807.50968
3.74E-07	1442.11933	1466.91594	1328.94617	1056.68359	958.193707	1075.84751
4.19E-15	3333.70106	3193.76469	3435.35073	4677.44225	5051.41064	4702.08191
6.60E-52	3680.39693	3874.49039	3650.37124	6978.42467	6855.06938	7034.5069
1.64E-09	1421.79188	1487.15661	1491.20701	1103.04828	1008.72725	1028.53194
2.48E-17	1305.47372	1404.06333	1376.7285	816.234159	836.718845	869.520591
0.00564231	2317.32878	2371.3543	2153.19143	2668.66518	2513.07193	2881.59589

1.00E-09	1374.36117	1313.51296	1287.13663	977.971443	870.731807	913.733502
0.00489105	5059.27532	5138.99962	4896.69379	4514.62672	4276.88692	4499.63332
0.0009734	18531.8545	18756.7094	21551.8236	24870.8813	27395.0107	21791.5357
7.30E-89	431.393565	515.60444	504.700905	12.9389827	17.49238	13.1863069
0.01739101	38.3962859	56.4608167	45.7914036	71.1644049	72.8849168	83.7718321
3.75E-05	1087.51833	1006.70702	1067.1388	855.051107	775.495515	810.570042
3.68E-22	1086.38903	1054.64544	1074.10705	1937.61266	1978.58254	1731.28453
2.08E-22	1051.38065	1130.28163	1076.09798	1983.97735	1768.67398	1990.35668
8.34E-20	5508.73772	5734.50144	5972.79177	3448.23889	3712.27176	3844.97196
2.01E-11	1915.29708	1881.31703	1798.80579	1263.70731	1123.39952	1327.93867
7.83E-07	77.9218743	73.5055916	83.6190848	158.502538	151.600627	154.357357
1.22E-13	1691.69518	1894.10061	1903.32964	2772.17704	2831.82197	2627.17774
1.47E-06	929.415979	949.1809	972.569594	628.61891	749.256945	588.72982
1.67E-09	144.550723	131.031707	124.433162	253.388411	246.836918	297.855403
0.05246003	2368.1474	2326.61177	2026.76734	2077.78497	1873.62826	1960.10574
1.40E-08	7123.64034	6956.39874	6266.45403	9365.66698	8686.91029	10094.5058
3.94E-20	1536.98074	1623.51481	1294.10488	681.453089	526.714999	722.14422
5.14E-64	534.160095	509.212649	565.424288	1534.3477	1503.37288	1435.75612
0.07574788	1828.34079	1705.54278	1738.08241	1497.68725	1581.1168	1658.37201
6.72E-69	1485.03282	1538.29093	1748.03706	4665.58151	5053.35423	4350.70561
5.69E-12	1830.5994	2038.98119	1874.46115	2654.64795	2756.02166	2900.21185
0.000142	452.850313	400.552209	487.777995	618.914673	639.44367	577.094844
0.00149869	115.188858	105.464544	112.487578	169.285024	180.754594	155.133022
0.01023615	115.188858	135.2929	88.5964113	152.033047	159.375018	190.037953
9.65E-155	1418.40397	1339.08013	1404.60153	5109.81992	4734.6042	5142.65969
4.93E-28	901.183416	943.854408	848.136432	326.709313	285.708874	397.140537
2.80E-58	1006.20855	1066.36373	1090.0345	2366.75559	2338.14813	2375.08657
4.54E-14	1675.88495	1686.36741	1572.83517	1095.50054	974.714288	1092.91214
5.43E-11	1805.75474	1850.42337	2038.71293	1257.23782	1264.31036	1016.89696
1.78E-53	1634.10076	1640.55958	1725.14136	3819.1564	3463.49125	3530.05193
4.44E-08	3149.62475	3041.42701	3075.98776	2341.95587	2465.45379	2424.72914
5.52E-40	7701.84323	7542.31288	7879.10781	12480.7271	12820.9428	12923.3564
8.03E-70	2630.14558	2763.38412	2754.45247	5857.04617	5931.86043	5621.24507
5.92E-17	101.637227	73.5055916	68.6871054	7.54773991	5.83079335	3.87832556
0.07833783	0	1.06529843	0	2.15649712	4.85899446	4.65399067
0.00342018	5512.12563	5417.04251	4676.69596	6977.34642	5541.19728	7037.60956
4.29E-24	2673.05908	2540.73675	2182.05993	1277.72454	1132.14571	1291.48241
9.78E-175	1561.82539	1703.41219	1571.8397	5937.91481	5748.19044	5926.85712
3.13E-30	2900.04889	3141.56507	2991.37321	5036.49902	5067.93122	5456.0284
0.0006831	82.4390844	100.138052	83.6190848	53.9124279	43.7309501	44.9885765
5.03E-15	5323.53211	5058.03694	5121.66895	7129.37947	6960.99546	7271.08476
1.20E-09	80.1804794	100.138052	107.510252	209.18022	232.259935	197.794604
2.37E-57	1287.40488	1291.1417	1310.03233	487.368349	458.689077	406.448519

1.99E-15	1433.08491	1579.83757	1727.13229	861.520598	861.985617	640.699383
9.08E-13	5826.07173	6156.35962	7161.37734	4155.56995	4226.35338	3788.34841
3.50E-22	958.777845	833.063371	908.859815	391.404227	473.26606	422.737486
9.71E-07	7223.01896	6847.7383	6377.94615	5120.60241	4344.91284	5382.34021
8.44E-08	629.021507	703.096963	805.331424	459.333886	474.237859	428.167142
0.00151244	675.322911	591.240628	533.569398	739.678511	758.003135	853.231624
0.0139968	2101.632	2082.65843	2272.64727	1855.66577	1933.87979	1863.92326
1.99E-06	1431.9556	1494.6137	1184.6037	899.259298	758.003135	1017.67263
1.63E-07	1268.20674	1405.12863	1425.5063	1803.90984	1918.33101	1806.52405
1.56E-07	3698.46577	3742.39338	3629.46647	2644.94371	3028.12535	2744.30317
0.08531659	1244.49138	1148.39171	835.195383	696.548569	565.586955	952.516758
7.64E-33	1027.6653	1177.15476	1173.65358	2524.17988	2278.8684	2241.67217
3.06E-11	929.415979	781.929047	791.39491	535.889534	423.704317	463.847737
1.44E-06	1618.29052	1726.84875	1816.72416	1276.64629	1364.40564	1185.99196
0.05953734	1111.23369	1209.11372	1212.47673	1324.08923	1478.10611	1243.39117
0.00027796	668.547096	726.533528	728.680596	529.420042	534.48939	573.992183
9.28E-43	5913.02803	5647.14697	6348.08219	2766.7858	3000.91498	2585.29182
7.26E-09	439.298683	422.923476	462.891362	265.249145	288.624271	248.988501
1.58E-12	549.97033	589.110031	587.324524	307.300839	345.960405	342.068315
0.01109638	752.115482	732.925319	709.766756	924.059015	838.662443	845.474972
0.00327335	979.10529	1037.60067	1000.44262	884.163818	828.944454	770.235456
3.63E-07	389.609372	433.576461	434.022869	622.149418	638.471872	587.954155
0.00020362	1087.51833	1094.06149	1255.28174	1434.07058	1557.79362	1405.50518
0.03670575	1966.1157	1895.1659	2101.42724	1642.17255	1816.29213	1794.1134
3.12E-20	1321.28396	1286.8805	1387.67862	802.216928	788.128901	788.075754
1.03E-12	75.6632693	91.6156649	73.6644319	17.2519769	9.71798891	3.87832556
0.00069465	1503.10166	1645.88607	1459.35212	1127.84799	1124.37132	1348.88163
0.05150224	1384.5249	1211.24431	1254.28627	1096.57878	1178.79206	1106.09845
0.00496869	507.056834	529.453319	553.478704	422.673435	431.478708	427.391477
1.00E-19	8275.52891	7754.30726	7752.68372	5324.39138	5516.90231	5397.07785
3.72E-18	97.1200173	80.9626806	82.6236195	3.23474568	10.6897878	4.65399067
0.08414739	94.8614122	86.2891727	94.5692031	64.6949135	75.8003135	62.8288741
5.54E-11	4648.2092	5000.51082	4767.2833	6247.37215	6366.25454	6445.77708
1.05E-38	555.616843	540.106303	459.904967	141.250561	137.023644	157.460018
9.47E-21	266.515396	273.781696	340.449131	81.9468905	96.2080902	98.5094693
9.95E-20	644.831742	646.636146	619.179414	1056.68359	1190.45364	1142.55471
6.60E-112	172.783287	145.945885	147.328864	1060.99658	945.560321	1057.23155
2.29E-20	277.808421	315.328335	314.567033	613.52343	623.894888	612.775439
0.00049707	2203.26923	2032.5894	1604.69006	1307.9155	1271.11295	1620.36442
1.29E-10	4946.34506	4691.57428	4337.24229	6233.35492	6439.13945	6748.28648
4.01E-15	878.597365	910.830157	943.7011	1408.19262	1536.41405	1394.64587
5.97E-11	538.677305	497.494366	467.868689	296.518354	296.398662	284.669096
9.61E-18	1928.84871	1919.66777	2510.56348	4392.78463	4955.20255	3842.64497

1.28E-06	27.1032606	21.3059686	29.8639589	2.15649712	1.94359778	1.55133022
9.73E-10	583.849406	657.289131	708.77129	1071.77907	969.855294	991.300013
5.13E-15	1253.5258	1339.08013	1104.96648	2075.62848	1925.1336	2143.93837
5.72E-20	4280.05657	4182.36163	3961.95188	2615.831	2639.40579	2800.15106
1.20E-25	1106.71648	1276.22752	1328.94617	2608.28326	2824.04758	2322.34135
0.04162477	3555.04435	3352.49416	3146.6658	3055.75642	2806.5552	3066.97985
0.00040059	3080.73729	3051.0147	2753.45701	3557.142	3543.17876	3540.13557
0.00103377	3801.2323	3472.87288	2935.62716	2481.04993	2191.4065	2952.95708
0.07960397	2502.5344	2464.03527	2704.67921	2828.24597	2825.99118	2770.67578
8.68E-10	1396.94722	1295.40289	1171.66265	1924.67368	1831.84091	2204.44025
0.05234058	9232.04815	8076.02739	8013.49563	9185.59947	9367.16951	9723.73785
7.44E-13	260.868884	287.630576	248.866324	486.2901	482.984049	591.056816
4.55E-39	1923.2022	2034.72	2119.34561	3823.46939	3925.09572	4245.99082
0.02777233	5858.82151	6273.54245	6095.234	5613.362	4971.72313	5577.80782
2.30E-10	63.2409415	74.57089	106.514787	0	1.94359778	0
0.00085844	355.730296	334.503707	347.417388	260.936151	230.316337	266.053134
1.82E-31	1452.28305	1516.98496	1414.55618	628.61891	594.740921	733.003531
1.98E-28	5547.13401	5640.75518	6208.71705	3325.31856	3418.7885	3115.07109
0.0001024	3618.28529	3405.75908	3801.68196	2991.0615	3032.01254	2768.34879
3.55E-21	154.714446	128.90111	125.428627	20.4867226	18.4641789	26.3726138
9.85E-12	1047.99274	989.66224	834.199918	566.080493	423.704317	536.760258
0.00628216	13.5516303	26.6324607	11.9455835	4.31299423	5.83079335	0
0.00036026	1588.92865	1514.85437	1439.44282	1312.2285	1029.13503	1151.08703
6.62E-27	3522.29458	3280.05386	3096.89253	5477.50268	5646.15156	5907.4655
2.03E-17	4092.59236	4080.09298	3867.38267	5877.53289	5615.05399	5978.82669
4.44E-08	1983.05524	2126.33566	1661.43158	2830.40247	2665.64436	3001.04832
2.57E-05	2972.32425	2823.04084	2327.39786	1894.48272	1795.88435	2161.003
8.58E-24	143.421421	136.358199	119.455835	387.091233	346.932204	390.159551
0.00043994	1008.46716	1034.40477	893.927835	783.886702	739.538956	783.421763
7.43E-05	630.15081	660.485026	668.952679	855.051107	887.252388	808.243047
3.39E-18	1793.33241	1650.14727	1678.35449	955.328223	1091.33015	1013.7943
3.86E-28	4095.98026	3926.69001	3576.70681	7138.00546	6727.76372	7777.59408
5.81E-14	2385.08694	2274.41215	2378.16659	3454.70838	3549.98135	3211.25356
4.79E-18	2741.94653	2643.0054	3164.58417	1586.10363	1582.0886	1262.7828
1.12E-06	7273.83757	7122.58529	7082.73558	5926.05408	5749.16224	5997.44265
1.18E-09	7155.26081	6878.63195	6820.9282	5269.40071	5479.00215	5480.84968
0.03658937	13428.5363	13861.6632	13416.8813	12202.5389	11719.8946	12955.1587
1.06E-05	10893.2522	11448.7622	12027.2117	15483.6493	15241.6938	13295.6757
7.19E-10	869.562945	816.018596	758.544555	480.898857	560.72796	497.977002
0.04512941	128.740488	145.945885	157.283517	197.319486	206.021365	164.441004
1.21E-21	2985.87588	2988.16209	3328.83595	1341.34121	1754.097	1334.14399
1.53E-11	1804.62544	1773.72188	1692.291	1272.3333	1226.4102	1205.38358
3.79E-13	1974.02082	1943.10433	2005.86257	1418.9751	1359.54665	1374.47858

0.00015181	459.626128	422.923476	405.154375	322.396319	303.201254	305.612054
4.73E-05	3268.20151	3608.16578	3885.30105	2735.51659	2981.479	2544.18157
9.63E-15	530.772187	504.951455	553.478704	305.144342	267.244695	268.380129
4.08E-14	842.459685	772.341361	796.372236	1306.83725	1399.3904	1212.36457
5.37E-21	1570.85981	1737.50174	1789.8466	2830.40247	3056.30751	2773.00278
1.37E-160	872.950853	871.414115	853.113758	25.8779654	24.2949723	25.5969487
3.96E-24	342.178665	333.438408	350.403784	777.417211	723.018375	684.136629
3.13E-24	291.360052	328.111916	310.585172	99.1988674	107.869677	82.996167
0.00958706	763.408508	764.884272	685.875589	652.340378	534.48939	607.345783
5.37E-14	303.78238	266.324607	259.816442	128.311578	96.2080902	115.574102
1.89E-05	7977.39305	7911.97143	7054.86255	9876.7568	9032.87069	10477.6843
1.54E-06	71.1460592	50.0690262	62.7143136	11.8607341	21.3795756	19.3916278
0.00628358	29010.6526	28559.5856	24289.3532	32498.4116	30198.6505	35039.1201
0.00497088	359.118203	354.744377	380.267743	249.075417	309.032047	274.58545
4.41E-28	11524.5323	13191.5904	13230.7292	22455.6045	22052.0604	21312.9503
0.07496161	35.0083783	26.6324607	52.7596607	67.9296592	56.3643357	55.8478881
2.14E-45	1866.73708	1832.3133	1768.94183	3477.3516	3368.25496	3487.39034
3.58E-13	4615.45942	4762.94928	5250.08397	6993.52015	7509.09003	6878.59822
1.80E-05	79.0511768	118.248126	90.5873419	47.4429366	34.0129612	41.8859161
0.01675401	2770.1791	2990.29269	2810.19853	3142.0163	3220.54153	3291.14707
0.00032362	902.312718	952.376795	959.628545	1190.38641	1252.64877	1100.66879
3.43E-05	734.046642	676.464502	671.939074	493.83784	547.122776	499.528332
1.91E-19	2214.56225	2051.76477	1835.638	1025.41438	998.037461	1187.54329
1.73E-09	4063.23049	4224.97357	3824.57767	5437.60748	5293.38856	6014.50728
3.93E-10	1822.69428	1864.27225	1894.37046	2462.71971	2499.46675	2477.47437
7.73E-09	609.823364	635.983162	594.292781	431.299423	365.396383	375.421914
0.07586632	485.600086	431.445864	486.782529	545.593771	538.376586	538.311588
5.96E-08	5631.8317	5886.83912	5443.20424	4438.07107	4263.28174	4543.07056
3.62E-07	557.875448	557.151078	533.569398	811.921165	741.482554	770.235456
4.12E-15	5015.23252	5317.96976	5251.07943	8057.75148	7105.79349	7876.87922
1.98E-06	2385.08694	2330.87296	2105.4091	1616.29459	1522.80886	1831.34533
3.55E-08	1872.38359	1824.85621	2278.62006	1324.08923	1419.79818	1233.30753
0.0003968	3382.26107	3625.21055	3662.31682	3020.17421	3015.49196	2589.17014
4.73E-38	6388.46439	6841.34651	6728.34993	11772.3178	11409.8908	12547.1589
4.58E-14	5879.14895	6327.87267	6591.97119	4463.94903	4384.7566	4360.78926
0.00046867	3721.05182	3570.88033	3144.67487	2777.56829	2801.6962	2917.27649
0.00810537	385.092161	338.7649	343.435527	288.970614	278.906282	264.501803
6.16E-92	863.916433	872.479413	861.077481	2426.05926	2355.64051	2335.52765
1.12E-80	9700.7087	9541.87803	9180.18095	21548.7974	20663.3598	22831.7026
5.32E-10	6566.89419	6467.42676	5826.45837	8895.55061	8184.49026	9090.01945
4.48E-10	378.316346	357.940272	379.272278	202.710729	219.626549	214.083571
4.38E-05	95.9907147	56.4608167	86.6054807	35.5822024	21.3795756	34.1292649
0.00051235	0	1.06529843	0	11.8607341	8.74619002	18.6159627

3.75E-13	9924.3106	9500.33139	10355.8255	7105.658	7317.64565	6597.03178
1.08E-08	14656.0882	15027.0996	14724.9227	11691.4491	12240.7788	11751.3265
3.21E-35	583.849406	573.130555	613.206622	223.197452	181.726393	209.42958
4.19E-08	1630.71285	1587.29466	1618.62657	1247.53358	1162.27147	1214.69157
0.00240547	2091.46828	2033.6547	2005.86257	2334.40813	2446.01781	2337.07898
0.0660739	2353.46646	2327.67707	2548.39116	2133.8539	2316.76856	1962.43273
8.24E-09	901.183416	891.654785	708.77129	476.585863	429.53511	550.72223
4.09E-24	1138.33695	1208.04842	1213.4722	2058.3765	2052.43926	1936.06012
1.80E-36	1137.20764	1135.60813	1265.23639	474.429366	538.376586	473.155718
2.36E-15	2321.84599	2258.43267	2358.25728	1522.48696	1620.96055	1579.25417
6.69E-06	721.624314	746.774199	763.521882	542.359025	574.333145	529.003607
2.27E-26	617.728482	622.134282	687.866519	1390.94064	1480.04971	1227.87787
1.60E-24	125.35258	141.684691	104.523856	7.54773991	13.6051845	9.30798135
0.00238597	991.527618	987.531644	1038.2703	1195.77765	1286.66173	1158.06801
7.23E-20	979.10529	1008.83761	1063.15694	1671.28527	1860.99488	1678.5393
8.71E-15	1605.86819	1541.48683	1497.1798	1040.50986	1022.33243	981.216367
0.01746306	1749.28961	1888.77411	2060.61316	2281.57395	2502.38215	2025.26161
0.00036711	868.433643	827.736879	919.809933	687.92258	717.187582	646.904704
1.44E-11	542.065213	547.563392	595.288247	276.031631	349.847601	257.520817
4.33E-08	378.316346	537.975707	420.086355	223.197452	257.526706	250.539831
1.36E-29	4880.84552	4928.07053	4634.88642	2533.88411	2391.59707	2821.86968
8.32E-05	41.7841935	38.3507434	40.8140771	90.5728789	84.5465035	79.1178415
3.37E-28	2382.82833	2492.79832	2699.70188	1339.18471	1132.14571	1065.76386
7.44E-12	7446.62086	7551.90056	8471.40966	5686.6829	5490.66374	5224.10453
0.00656642	25.9739581	27.6977591	38.8231465	8.62598847	11.6615867	15.5133022
5.71E-11	1937.88314	1890.90471	2160.15969	1407.11437	1326.50549	1373.70291
0.05270689	177.300497	158.729466	223.979691	152.033047	137.995443	137.292725
3.79E-06	5099.93021	5264.70484	5196.32884	6344.41452	6116.50222	6315.46534
0.05257049	7351.75945	8503.21206	7595.4002	9870.28731	7598.49553	10075.8898
3.83E-05	1509.87748	1749.22002	2049.66304	1320.85448	1301.23872	1139.45205
0.00108517	31.6204707	25.5671623	33.84582	11.8607341	8.74619002	9.30798135
0.01222995	1297.5686	1437.08758	1422.51991	1200.09065	1241.95898	1060.33421
1.91E-09	2320.71669	2328.74237	1812.7423	1252.92483	1038.85301	1426.44814
0.0001723	1342.7407	1347.60251	1104.96648	930.528506	839.634242	1026.20494
3.28E-11	1202.70719	1280.48871	1138.8123	816.234159	820.198264	766.357131
0.03943851	5706.36567	5717.45667	5062.93649	5014.93405	4532.47003	5034.06658
2.23E-05	6842.44401	6501.51631	6562.10723	5402.02528	5713.20568	5456.0284
3.52E-09	2911.34191	3157.54454	3233.27128	4080.09255	4335.19485	4077.6715
9.57E-08	3309.9857	3093.62664	3394.53666	4138.31797	4715.16822	4264.60679
6.05E-06	2266.51017	2290.39162	2222.874	1851.35278	1803.65874	1824.36434
0.010083	4368.14217	4491.29818	4415.88405	4717.33744	5162.19571	5173.6863
0.01302726	571.427078	493.233173	458.909501	368.761007	376.086171	446.00744
0.00992451	1728.96217	1804.61554	1816.72416	1932.22142	2123.38058	2119.11709

6.40E-31	1784.29799	1924.99426	1706.22752	841.033876	886.280589	949.414097
1.75E-18	1443.24863	1417.91221	1322.97338	858.285853	822.141862	854.782954
2.75E-33	7369.82829	7407.01998	6677.5812	4008.92814	3644.24584	3981.48902
2.89E-12	1383.39559	1348.66781	1369.76025	972.5802	909.603762	883.482563
0.01373929	59.8530339	61.7873089	48.7777995	29.1127111	20.4077767	40.3345858
6.19E-12	1448.89514	1440.28348	1474.2841	941.310992	979.573282	1042.49391
1.59E-05	1305.47372	1268.77043	1168.67626	907.885286	878.506198	1013.7943
2.94E-13	565.780566	568.869361	535.560329	921.902518	865.872812	1030.08327
1.67E-21	1412.75746	1523.37675	1539.98481	724.583031	872.675404	798.159401
1.29E-22	3442.1141	3105.34492	3275.08082	1755.38865	2023.28529	1746.02217
1.10E-06	5194.79162	5069.75522	4382.03823	3408.34369	3564.55833	3860.48526
5.40E-11	3368.70944	3039.29642	2751.46608	4449.9318	4756.95557	5592.54546
1.10E-14	8848.08529	8393.48632	7396.30715	5166.96709	5070.84661	5549.10821
0.00023744	614.340574	641.309654	565.424288	804.373425	779.382711	765.581466
0.0164429	4.5172101	12.7835811	9.95465295	3.23474568	0	0
0.03544632	13725.5429	15519.2675	16076.7645	13864.12	13538.1304	12649.5467
0.00021676	2304.90646	2431.01101	2318.43867	1888.01323	1661.7761	2022.15895
2.19E-16	1708.63472	1980.38978	2061.60863	3311.30132	3282.73665	3001.82398
2.15E-21	1628.45424	1760.9383	2039.70839	3661.73211	3739.48213	3188.75928
1.94E-22	416.712632	429.315267	420.086355	183.302255	162.290415	138.06839
4.43E-19	1707.50542	1707.67338	1660.43611	1071.77907	1061.20439	966.47873
1.40E-08	505.927532	412.270492	401.172514	713.800546	684.146419	697.322936
0.00213067	319.592615	325.981319	379.272278	222.119203	265.301097	259.072147
4.12E-16	154.714446	181.100733	174.206427	46.364688	26.2385701	50.4182323
1.84E-27	3871.24906	4205.7982	4286.47356	6980.58117	6937.67229	6610.99375
0.00211527	2316.19948	2321.28528	2186.04179	2674.05643	2567.49267	2706.29558
2.51E-05	3963.85187	4048.13403	4504.48046	5434.37274	6030.98392	4959.60273
3.28E-16	2346.69065	2312.76289	2484.68138	3540.96827	3480.98363	3395.0862
9.14E-41	204.403757	187.492523	207.056781	9.70423703	9.71798891	3.87832556
9.58E-07	7.90511768	6.39179057	8.95918766	57.1471736	33.0411623	38.7832556
1.59E-19	276.679119	287.630576	222.984226	560.689251	586.96653	628.288741
0.01562984	193.110732	221.582073	221.988761	283.579371	275.019086	263.726138
3.55E-05	769.05502	745.7089	670.943609	545.593771	411.070931	536.760258
0.00215126	879.726668	1035.47007	1110.93927	1383.3929	1344.96967	1161.94634
3.07E-12	2251.82924	2189.18827	2255.72436	1649.72029	1528.63966	1567.61919
1.11E-29	2018.06361	2154.03342	2165.13702	3636.93239	3511.10939	3793.0024
0.00161133	4025.96351	3954.38777	4336.24683	3465.49087	3655.90743	3222.88854
2.28E-07	24.8446556	34.0895497	35.8367506	1.07824856	4.85899446	0.77566511
4.48E-05	54.2065213	44.742534	59.7279177	112.13785	114.672269	96.1824739
2.75E-10	2012.4171	1782.24427	2140.25039	3199.16347	3517.91199	2762.91913
0.0626543	2533.02557	2730.35987	2697.71095	2509.0844	2340.09173	2349.48963
0.07063027	5395.80747	5783.50517	5309.81189	5023.56004	4755.01198	5203.93724
1.08E-20	1066.06158	1002.44582	998.451691	1645.4073	1689.95827	1778.6001

0.03875766	5.64651263	4.26119372	7.96372236	0	0	1.55133022
1.33E-07	981.363895	1029.07828	846.145501	640.479644	612.233302	674.052983
2.28E-100	1287.40488	1303.92528	1323.96884	329.944059	356.650193	320.349691
8.15E-32	255.222371	242.888042	206.061316	754.773991	737.595359	1047.1479
5.53E-06	428.005657	471.927204	434.022869	306.222591	316.806439	286.220426
0.02905728	110.671648	89.485068	80.6326889	63.616665	68.9977213	55.072223
7.08E-21	10804.0373	10583.7399	10981.9731	7485.20149	7641.25468	7216.7882
1.01E-09	957.648542	903.373068	990.487969	681.453089	598.628117	589.505485
8.85E-37	2100.5027	2208.36364	2150.20504	3774.9482	3712.27176	3924.0898
0.00318555	416.712632	406.944	417.099959	538.046031	529.630396	494.874342
4.74E-09	559.00475	568.869361	510.673697	336.41355	364.424584	310.266045
0.01143912	9.03442021	8.52238743	11.9455835	19.4084741	28.1821678	28.6996092
1.17E-10	776.960138	787.255539	688.861984	1094.42229	1148.66629	1245.71817
7.38E-95	64589.3287	68396.4203	69425.7406	30355.9317	28689.4469	28955.5786
6.65E-150	3518.90667	3443.04452	3099.87893	14103.4911	13670.295	15880.1918
0.03296032	3.38790758	1.06529843	1.99093059	11.8607341	4.85899446	13.1863069
3.52E-07	1763.97055	1821.66031	1834.64254	2304.21717	2348.83792	2258.73681
4.15E-07	6272.14623	6738.01256	6612.87596	8272.32294	7904.61218	8232.13384
9.37E-07	2002.25338	2148.70693	2242.78331	2704.24739	3004.80217	2727.23853
1.53E-12	807.451306	885.262994	751.576298	1255.08132	1315.8157	1262.00714
1.08E-10	6604.16117	6436.53311	6308.26358	8223.80176	8270.98036	8540.07289
6.97E-52	1316.76675	1336.94953	1404.60153	3574.39397	3059.22291	3146.87336
6.55E-06	913.605744	855.434638	881.982252	1155.88245	1214.74861	1110.75244
4.71E-137	752.115482	757.427183	722.707805	2916.66235	2686.05214	2936.66812
9.70E-24	639.18523	728.664125	653.025234	1326.24573	1293.46432	1218.56989
6.52E-06	1084.13043	1170.76297	1097.00276	858.285853	824.08546	873.398916
1.07E-132	12814.1958	11775.8088	10919.2588	3341.49228	3439.19628	3406.72117
2.16E-56	787.123861	720.141738	890.941439	147.720053	197.275175	146.600706
4.38E-16	18539.7596	18544.715	14780.6687	9089.63535	7886.148	10323.327
2.29E-38	1536.98074	1460.52415	1285.1457	521.872302	552.953569	608.121448
2.07E-19	2141.15759	2036.8506	1933.1936	1248.61183	1253.62057	1291.48241
8.64E-26	2409.93159	2379.87669	2420.9716	1454.55731	1500.45749	1483.07169
7.29E-05	1398.07653	1393.41034	1331.93257	1781.26662	1659.83251	1669.23132
0.00012135	1645.39378	1681.04092	1683.33181	1965.64712	2111.71899	2012.85097
2.77E-05	2095.98549	2302.1099	2340.33891	2789.42902	2849.31435	2742.75184
0.00168705	8414.43312	8281.62999	7552.5952	6937.45123	6286.56703	7133.01637
1.32E-06	755.50339	769.145466	800.354098	580.097725	544.207379	455.315421
4.02E-13	2485.59486	2396.92146	2565.31407	3662.81035	3360.48057	3582.02149
5.42E-13	2575.93906	2891.21994	3080.96509	1892.32622	1888.20525	1806.52405
2.93E-05	5088.63718	4848.17315	5056.9637	5957.32329	6074.71487	5848.51495
0.0591284	2903.43679	2861.39158	2850.01714	2653.5697	2333.28914	2706.29558
2.97E-17	4582.70965	4834.32427	4722.48736	3134.46856	3292.45464	3326.052
1.07E-06	6746.45329	7034.16553	6821.92367	5556.21482	5420.69422	5771.7241

0	648.21965	640.244356	671.939074	5209.01879	5487.74834	5610.38576
0.00012942	187.464219	227.973864	182.170149	115.372596	85.5183024	135.741395
0.05220506	2470.91393	2386.26848	2514.54534	2272.94796	2087.42402	2269.59612
1.11E-14	2921.50564	2771.90651	2434.90811	4292.50751	4090.30153	4562.46219
4.62E-08	3162.04707	3087.23485	2872.91284	3962.56345	3870.67498	4257.6258
0.00020627	770.184323	890.589486	851.122828	659.888118	633.612877	648.456034
0.00026751	2710.32606	2866.71807	2894.81308	2426.05926	2377.99189	2250.98016
7.35E-27	11252.3704	11302.8163	11778.3454	6575.15971	7130.08847	6068.02817
7.60E-27	26362.4382	25877.1641	27494.7515	16696.6789	17620.6575	16131.5073
1.24E-09	155842.619	152027.673	142991.621	117220.714	109286.56	117770.01
1.90E-18	3778.64625	3726.4139	3049.1102	1746.76267	1589.86299	2016.72929
8.18E-48	3679.26763	3626.27585	3117.79731	1291.74177	1137.0047	1397.74853
0.00048729	137.774908	92.6809633	119.455835	75.4773991	58.3079335	53.5208927
1.21E-16	452.850313	418.662283	522.61928	219.962706	193.387979	215.634901
2.59E-31	490.117296	400.552209	385.245069	105.668359	130.221051	101.61213
9.91E-08	19766.1821	18404.0957	16861.1912	13810.2075	12661.5678	14269.9111
0.00074928	1284.01697	1297.53349	1524.05737	1754.31041	2032.03148	1597.09447
8.94E-16	450.591708	534.779811	344.430992	1294.97652	913.490958	1495.48234
5.18E-35	2868.42842	2774.03711	2609.11454	1414.66211	1357.60305	1498.585
6.29E-05	2757.75677	2877.37106	3033.18276	3540.96827	3820.14144	3407.49684
7.80E-19	4658.37292	4801.30002	4806.10645	6727.19276	6644.18902	6781.64008
0.05774043	3027.66007	2880.56695	2818.16225	2336.56463	2570.40807	2805.58071
0.01221862	1508.74818	1460.52415	1450.39294	1192.54291	1306.09771	1312.42537
5.62E-86	1468.09328	1416.84691	1432.47456	442.081909	387.747758	433.596798
2.14E-57	12593.9818	12291.4133	12133.7265	5220.87952	5871.6089	5973.39703
2.60E-43	343.307968	363.266764	375.290416	970.423703	924.180746	898.995865
2.87E-08	20913.5535	22573.6737	21603.5878	27390.7481	26679.7668	28781.054
1.94E-33	514.961952	516.669738	509.678231	196.241238	184.641789	172.197655
1.79E-05	1693.95379	1785.44017	1686.31821	1376.92341	1347.88506	1403.17819
6.98E-18	1997.73617	2093.31141	1969.03035	1333.79347	1172.96126	1244.94251
6.79E-07	598.530339	582.718241	568.410684	420.516938	355.678394	406.448519
7.72E-23	8298.11496	8077.09269	8697.38029	5256.46172	5386.68125	4768.01345
6.78E-05	5057.01671	4832.19367	3802.67743	3285.42336	2716.1779	3596.75913
7.74E-12	8982.47229	9906.21009	10640.5285	6563.29898	7009.5854	6063.37418
8.64E-34	5578.75448	5880.44733	5726.91184	9702.08053	10100.8777	11024.5282
9.49E-28	6116.30248	6472.75325	6179.84855	3974.42419	3662.71002	3881.42822
2.66E-109	2656.11954	2670.70316	2683.77444	6726.11451	6457.60363	6840.59063
1.86E-51	114.059555	128.90111	135.38328	530.498291	534.48939	503.406658
2.84E-21	11975.124	13461.1109	13164.0331	8466.40768	8216.55963	8281.7764
6.18E-07	1696.21239	1663.99615	1592.74447	1201.16889	1178.79206	1323.28468
4.63E-09	319.592615	371.789152	377.281347	574.706482	565.586955	563.908537
1.98E-23	595.142431	657.289131	520.62835	224.2757	191.444382	246.661506
1.61E-21	275.549816	233.300356	287.68947	90.5728789	75.8003135	69.034195

1.16E-29	2167.13155	2389.46438	2458.79928	1231.35985	1292.49253	1213.14024
6.76E-07	29.3618657	37.285445	34.8412853	2.15649712	0	0
2.78E-06	3784.29277	4185.55753	3724.03567	3142.0163	2914.42488	3066.20419
1.08E-13	1503.10166	1418.97751	1342.88268	2081.01972	2088.39582	2215.29956
1.36E-10	4811.95806	4620.19929	4183.94064	3122.60783	2855.14514	3315.96835
1.49E-30	222.472598	189.62312	229.952483	39.8951967	39.8437545	35.6805952
0.00081999	0	0	0.9954653	10.7824856	6.80259224	19.3916278
3.87E-10	595.142431	562.47757	453.932175	324.552816	234.203533	251.315496
0.00127034	3989.82583	4171.70865	4311.36019	3689.76657	3540.26336	3451.70975
7.05E-06	4.5172101	5.32649214	0	23.7214683	34.9847601	29.4752743
2.00E-22	8248.42565	7969.49755	9880.98852	4297.89876	4881.34583	4076.89583
2.62E-21	2120.83014	2106.09499	1913.2843	3298.36234	3204.02094	3346.21929
1.08E-18	19339.3058	19521.5937	19223.4303	13951.4581	14043.4658	14390.1392
7.47E-18	4827.7683	4916.35225	4420.86138	7313.75997	7010.5572	7915.66247
5.24E-08	29.3618657	41.5466387	38.8231465	2.15649712	0.97179889	0
4.67E-25	408.807514	400.552209	428.050077	143.407058	163.262214	162.889674
8.54E-05	3619.4146	3506.96243	3196.43906	2547.90134	2284.69919	2938.99511
4.47E-08	1317.89605	1366.77788	1557.90319	1022.17963	997.065662	923.817149
3.82E-06	6938.43472	6399.24766	6398.85092	7962.86561	8088.28217	8142.93235
1.95E-09	4687.73479	4668.13772	5275.96607	3410.50019	3698.66658	3512.21163
1.66E-25	854.882012	727.598827	726.689666	340.726545	300.285857	344.39531
2.39E-26	3661.19879	3424.93445	3370.64549	1807.14458	2035.91868	2037.67225
1.71E-30	3918.67977	3803.11539	3522.95168	6676.51508	6254.49766	6437.24477
8.76E-05	517.220557	521.99623	592.301851	366.60451	429.53511	367.665263
0.04545198	625.6336	626.395476	532.573933	483.055354	393.578551	559.254546
2.00E-64	2560.12883	2594.00167	2331.37972	5955.16679	5805.52658	6496.19532
0.00022735	1988.70175	1984.65097	1852.56091	1664.81577	1564.59622	1536.59259
0.05284875	913.605744	804.300314	791.39491	987.67568	886.280589	1044.04524
2.59E-05	3739.12066	3618.81876	4126.20365	3050.36517	3147.65661	2766.02179
2.36E-26	4228.10866	4419.92318	4522.39884	2701.01264	2772.54224	2791.61874
3.56E-07	857.140617	777.667853	679.902797	521.872302	493.673837	524.349616
9.93E-11	11466.9379	11855.7062	12310.9193	15782.3242	15675.1161	15060.3138
0.00056103	59.8530339	42.6119372	50.7687301	24.7997169	21.3795756	17.0646325
0.04838541	351.213086	296.152963	331.489943	439.925412	428.563311	342.84398
0.0508637	1217.38812	1224.02789	1267.22732	1430.83584	1410.08019	1325.61168
1.68E-18	1255.78441	1284.74991	1411.56979	2226.58327	2392.56887	2088.09048
4.48E-13	1182.37974	1221.8973	1121.88939	798.982182	721.074777	729.125206
7.18E-20	526.254977	496.429068	521.623815	997.379917	946.53212	892.014879
2.75E-06	293.618657	338.7649	343.435527	547.750268	492.702038	454.539756
1.76E-06	3556.17366	3622.01466	3091.91521	4483.35751	4357.54623	5020.88027
1.15E-08	1140.59555	1132.41223	1342.88268	1854.58752	1928.049	1641.30738
7.87E-06	1780.91008	1904.75359	1935.18453	1399.56663	1492.6831	1507.89298
4.74E-08	58.7237314	73.5055916	94.5692031	219.962706	198.246974	148.152036

0.00049982	713.719197	702.031665	864.063876	971.501951	1077.72497	956.395083
1.42E-33	499.151717	495.363769	468.864154	1018.94489	1102.01994	1163.49767
0.00235371	8135.4954	7795.8539	7514.76752	6687.29756	6010.57614	7046.91755
0.01654177	21.456748	33.0242513	33.84582	52.8341794	46.6463468	61.2775439
3.29E-07	9.03442021	14.914178	13.9365141	75.4773991	65.1105257	38.7832556
0.01046782	3657.81088	3292.83744	2945.58181	2808.8375	2513.07193	2911.07117
8.56E-27	2438.16415	2580.15279	2462.78114	3921.59001	4064.06296	3982.26469
0.00238597	1182.37974	1153.7182	1097.00276	916.511275	981.51688	956.395083
0.01967279	293.618657	301.479455	329.499013	267.405643	240.034326	219.513227
0.00851381	7786.54092	7502.89683	8070.23715	6797.27891	7201.02978	6201.44257
2.04E-39	4261.98773	4286.76088	4308.3738	7138.00546	7193.25539	6974.78069
0.05752725	1019.76018	1117.49805	1040.26123	1167.74319	1151.58169	1289.93108
4.08E-23	10248.4204	10856.4563	11536.4473	18028.3159	18849.9831	16847.4462
1.85E-07	3073.96148	3306.68632	3381.59561	2525.25812	2356.61231	2565.90019
3.50E-19	8023.69445	8000.3912	7751.68826	11219.1763	10972.5813	11054.0035
6.01E-42	787.123861	851.173445	869.041203	1758.6234	1748.26621	1750.67616
0.00023348	3424.04526	3171.39342	3221.3257	2758.15981	2657.86997	2793.94573
5.23E-19	3948.04163	3847.85792	3514.98796	5720.1086	5746.24684	6089.7468
0.00269451	919.252256	948.115602	831.213522	1209.79488	998.037461	1187.54329
2.75E-05	2488.98277	2403.31326	2503.59522	2971.65303	2995.08418	2962.26506
1.59E-06	22.5860505	38.3507434	49.7732648	6.46949135	3.88719557	6.2053209
1.74E-12	6221.32762	6156.35962	5891.16362	4301.1335	3957.16509	4530.65992
0.02319701	3761.70671	3549.57436	3449.28725	4040.19735	3843.46462	4294.08206
6.79E-07	4750.97573	5148.58731	5733.8801	6953.62496	7175.76301	6704.07357
1.32E-55	1774.13427	1901.5577	2006.85804	4245.06458	4204.9738	4031.13159
4.82E-05	1829.47009	1780.11367	1818.71509	1450.24431	1392.58781	1525.73328
0.00187398	2195.36411	2037.91589	1895.36592	1561.30391	1715.22504	1753.00315
3.75E-17	591.754524	562.47757	522.61928	294.361857	269.188293	286.996092
0.00705851	1465.83468	1407.25922	1413.56072	1284.19403	1139.9201	1223.99955
1.36E-20	466.401943	506.016754	433.027404	156.346041	93.2926936	165.216669
3.35E-09	8970.04997	9234.00678	9287.69121	6900.79078	7459.52829	6947.63241
4.56E-69	20754.3218	21252.7037	20624.05	39206.1958	38792.2681	40640.9736
0.00291755	1900.61615	1813.13793	1969.03035	2264.32197	2257.48882	2136.18172
2.31E-05	447.2038	478.318995	589.315455	717.035292	765.777526	707.406582
3.39E-06	119.706068	86.2891727	122.442231	205.945475	218.654751	190.037953
4.40E-26	1435.34351	1594.75175	1490.21155	847.503367	785.213504	794.281075
4.61E-06	2022.58082	2219.01663	2244.77424	1755.38865	1653.02991	1549.77889
1.85E-16	150.197236	145.945885	154.297121	47.4429366	37.9001568	30.2509394
0.00723577	2025.96873	2197.71066	2508.57254	2638.47422	2837.65276	2608.56177
1.90E-06	1028.7946	1173.95887	1124.87578	740.75676	729.820967	869.520591
1.86E-05	297.006564	278.04289	321.53529	182.224006	213.795756	163.665339
5.60E-10	1526.81702	1459.45885	1526.0483	1979.66435	2079.64963	2127.6494
0.00045902	2868.42842	2968.98672	2975.44577	3434.22166	3590.7969	3359.4056

8.85E-45	1132.69043	1136.67342	1163.69893	2469.1892	2317.74036	2267.26912
1.32E-12	540.93591	575.261152	596.283712	353.665527	305.144852	300.958064
0.03572894	2194.23481	2091.18082	2123.32748	1867.5265	1625.81955	2032.24259
5.41E-11	622.245692	637.04846	615.197553	391.404227	342.07321	399.467533
4.73E-05	4557.865	4646.83175	4577.14943	3960.40696	3524.71458	3807.74004
2.71E-05	399.773094	428.249968	344.430992	552.063262	569.47415	544.516909
3.46E-23	1987.57245	1930.32075	2029.75374	3405.10895	3104.89746	3171.69464
1.22E-15	71.1460592	131.031707	88.5964113	293.283608	308.060249	297.855403
7.69E-05	1278.37046	1211.24431	1158.7216	943.467489	722.046576	954.068088
9.76E-05	4910.20738	4793.84293	5313.79375	4071.46656	4291.4639	3848.07462
8.94E-16	4835.67342	4662.81122	5129.63267	3378.15273	3288.56745	3032.85059
2.58E-12	1075.096	1117.49805	1074.10705	1537.58244	1597.63738	1573.82451
4.43E-05	1253.5258	1274.09692	1455.37026	942.38924	1089.38656	940.106116
0.00091459	325.239128	313.197738	301.625985	445.316655	384.832361	429.718472
3.92E-05	2834.54934	2794.27778	2601.15082	2169.4361	2193.3501	2303.72538
2.05E-18	1105.58717	1054.64544	995.465295	490.603094	515.053412	611.999774
7.23E-17	334.273548	280.173487	302.62145	134.78107	102.038884	114.022772
0.01389436	1042.34623	1057.84134	963.610406	916.511275	658.879648	863.31527
2.66E-16	1647.65239	1496.74429	1414.55618	878.772575	875.590801	952.516758
0.00177086	221.343295	231.169759	183.165614	154.189544	122.44666	148.152036
0.00643856	111.80095	127.835811	105.519321	67.9296592	51.5053412	88.4258228
2.19E-36	1462.44677	1425.3693	1343.87815	643.71439	609.317905	688.79062
1.32E-05	1909.65057	1830.1827	1614.64471	1277.72454	1294.43612	1433.42913
5.49E-13	1341.6114	1298.59878	1270.21372	890.63331	871.703606	814.448368
5.43E-26	4133.24725	4163.18626	3884.30558	2565.15332	2496.55135	2408.44017
8.78E-41	1577.63563	1703.41219	1852.56091	3796.51318	3981.46006	3521.51961
1.53E-11	1582.15284	1420.04281	1481.25236	2305.29542	2077.70603	2167.20832
7.37E-32	1701.85891	1871.72934	1702.24566	3158.19003	3086.43328	3167.81632
3.25E-10	1250.1379	1301.79468	1259.2636	1913.89119	1717.16864	1766.18946
0.00027515	1795.59102	1910.08008	2106.40457	1519.25222	1620.96055	1303.11739
8.43E-05	903.442021	1016.2947	1129.85311	711.644049	814.367471	699.649931
0.00014019	2406.54368	2264.82446	2019.79908	2737.67309	2738.52928	3062.32586
4.13E-05	9669.08823	10725.4246	10582.7916	12665.1076	12466.2362	12270.2464
0.00028765	651.607558	693.509277	757.54909	1036.19686	1039.82481	812.121373
1.06E-17	2289.09622	2439.5334	2323.416	3512.9338	3849.29541	3495.147
7.92E-05	1254.65511	1136.67342	1307.04593	1575.32114	1809.48954	1506.34165
0.00045449	1810.27195	2134.85805	2382.14845	1656.18979	1681.21208	1385.33789
6.04E-08	1466.96398	1658.66965	1705.23205	1215.18613	1102.01994	1055.68022
7.48E-14	2099.3734	2499.19011	2649.92862	4020.78888	4084.47074	3750.34082
1.95E-06	762.279205	853.304041	799.358632	593.036707	596.684519	546.843904
1.00E-11	278.937724	269.520502	297.644123	145.563555	137.023644	113.247106
6.06E-78	5839.62336	5965.6712	5268.99781	14907.8646	14078.4505	15784.0094
2.48E-29	3501.96713	3815.89897	3863.40081	6506.1518	6415.81628	6103.70877

7.25E-07	447.2038	497.494366	502.709974	678.218343	722.046576	674.828648
9.39E-05	1238.84487	1198.46073	1303.06407	953.171726	999.981059	1016.1213
0.0472971	0	1.06529843	1.99093059	5.39124279	9.71798891	5.42965579
3.04E-10	3916.42116	4060.91761	4407.92033	5740.59533	5706.40309	5442.84209
0.06123941	10.1637227	8.52238743	4.97732648	15.0954798	23.3231734	16.2889674
0.00129596	54.2065213	58.5914136	61.7188483	86.2598847	114.672269	104.71479
5.91E-15	1103.32857	1150.5223	1041.2567	694.392072	597.656318	661.642341
3.06E-12	1296.4393	1312.44766	1369.76025	2344.11237	1886.26165	2080.33383
2.48E-09	1338.22349	1433.89169	1602.69913	866.911841	1013.58624	741.535847
3.63E-08	990.398316	1136.67342	1236.3679	759.086985	773.551917	698.874266
7.99E-30	798.416886	747.839497	772.481069	1578.55589	1471.30352	1733.61153
0.03922813	153.585144	148.076482	149.319794	92.7293761	123.418459	117.901097
7.16E-08	4006.76536	4366.65826	3996.79316	3211.02421	2895.9607	3191.08627
7.00E-07	457.367523	449.555937	561.442427	708.409303	759.946733	733.003531
2.02E-41	6556.73047	6599.52377	7325.62911	3563.61149	3486.81442	3259.3448
5.64E-25	697.908961	780.863748	824.245265	1517.09572	1415.91098	1560.63821
8.41E-19	1235.45696	1365.71259	1248.31348	2114.44542	1994.13132	2127.6494
0.00113237	1346.12861	1345.47192	1384.69223	1111.67426	1179.76385	1104.54712
1.18E-06	863.916433	991.792837	956.642149	1269.09855	1327.47729	1227.10221
0.04837952	505.927532	516.669738	612.211157	637.244898	770.636521	587.954155
2.44E-13	112.930253	107.595141	98.5510643	23.7214683	26.2385701	28.6996092
2.00E-07	618.857784	598.697717	546.510447	396.79547	407.183735	380.075905
7.18E-14	439.298683	447.42534	455.923105	816.234159	897.942176	718.265894
1.63E-31	4286.83239	4396.48662	4146.11296	6760.61846	7048.45736	6968.57537
8.19E-42	1548.27376	1565.98869	1531.02562	764.478228	690.949012	699.649931
2.17E-13	2240.53621	2244.58379	2174.09621	1513.86098	1594.72198	1567.61919
1.71E-11	1094.29415	1145.19581	1182.61277	1798.5186	1755.0688	1576.15151
9.44E-19	1181.25044	1082.3432	1061.166	648.027384	642.359067	600.364797
9.90E-08	8370.39032	8459.53482	9718.72768	6656.02835	6797.73324	6151.80001
2.80E-42	521.737767	532.649214	533.569398	136.937567	141.882638	96.958139
0.00012067	423.488447	387.768628	388.231465	258.779654	315.83464	248.212836
7.07E-07	5120.25765	4947.2459	5313.79375	6313.14531	7064.97794	6382.17254
4.27E-06	195.369337	144.880586	169.2291	89.4946304	89.405498	89.2014879
8.38E-11	2191.9762	2366.02781	2381.15299	1613.05984	1661.7761	1420.24282
4.99E-55	450.591708	360.070869	462.891362	66.8514106	49.5617435	58.9505485
9.79E-12	773.57223	881.001801	873.023064	1539.73894	1284.71813	1309.32271
7.21E-12	2236.019	2358.57072	2709.65653	3999.2239	4334.22306	3525.39794
1.03E-05	1262.56022	1329.49244	1053.20228	1967.80362	1486.8523	2048.53156
8.44E-14	832.295962	869.283518	707.775825	1627.07708	1299.29512	1802.64572
2.22E-06	426.876355	490.037277	422.077285	278.188128	255.583108	317.247031
0.00264422	1344.99931	1422.1734	1258.26813	1066.38782	968.883495	1189.87028
0.06082748	355.730296	383.507434	419.090889	505.698574	479.096853	411.10251
2.49E-27	1105.58717	1216.57081	1111.93474	2493.98892	2086.45222	2544.18157

1.37E-07	1876.9008	1943.10433	1972.01675	2496.14541	2539.3105	2418.52382
0.00110289	1059.28577	1046.12306	985.510643	1222.73387	1258.47956	1247.2695
0.00051371	205.53306	168.317152	176.197357	114.294347	101.067085	128.760409
0.00023341	2367.01809	2260.56327	2232.82866	2651.41321	2730.75488	2849.79362
0.0826976	1106.71648	1055.71074	717.730478	596.271453	475.209658	833.839996
0.01425644	466.401943	458.078324	540.537655	349.352533	446.055691	342.84398
0.01628646	6504.78255	6976.63941	7143.45896	6289.42384	6181.61275	5758.53779
0.06274864	1440.99002	1414.71631	1259.2636	1149.41296	1162.27147	1293.03374
9.23E-05	2598.52511	2908.26471	2828.1169	2306.37367	2224.44766	2268.82045
2.48E-39	7116.86452	6417.35774	6431.70127	3632.61939	3610.23288	3580.47016
6.98E-09	1518.9119	1421.1081	1553.92133	1073.93556	1113.68153	1068.09086
3.91E-32	11390.1453	11393.3667	8821.81345	25807.8793	23319.2862	28991.2592
4.07E-08	31.6204707	29.828356	40.8140771	1.07824856	0.97179889	3.87832556
0.00059371	835.683869	874.61001	839.177244	692.235575	632.641078	701.201261
8.81E-06	2718.23118	2770.84121	2860.96726	3354.43127	3560.67114	3365.61092
9.69E-13	12517.1892	12429.9021	14022.1242	21950.9842	22704.1375	18049.7272
1.98E-18	1145.11276	1029.07828	954.651218	1825.47481	1743.40721	1954.67608
0.03814704	361.376808	398.421612	402.167979	475.507614	510.194418	423.513151
0.00042025	210.05027	285.499979	263.798303	169.285024	174.9238	151.254697
7.57E-08	2464.13811	2314.89349	2138.25945	1662.65928	1585.97579	1769.29212
4.22E-12	49.6893112	75.6361884	71.6735013	2.15649712	10.6897878	6.98098601
0.00014516	3494.06202	3332.25349	3218.3393	4210.56062	3810.42345	4401.89951
1.18E-14	406.548909	363.266764	450.945779	721.348286	743.426152	751.619494
1.49E-43	1101.06996	1215.50551	1112.9302	2445.46773	2378.96369	2641.91537
2.11E-09	1665.72123	1572.38048	1505.14353	2152.18412	2131.15497	2351.04096
1.29E-15	1924.3315	1947.36553	1920.25255	2691.3084	2871.66572	2871.51225
0.00022096	704.684776	657.289131	726.689666	870.146587	937.78593	867.193596
8.91E-05	29.3618657	24.5018639	42.8050077	3.23474568	6.80259224	10.0836465
1.88E-12	31.6204707	31.9589529	21.9002365	105.668359	114.672269	109.368781
1.29E-05	1040.08763	928.94023	920.805398	1345.6542	1187.53825	1333.36833
2.35E-41	5477.11725	5884.70852	5715.96173	3211.02421	3050.47672	3188.75928
1.48E-50	4398.63334	4171.70865	4072.44852	8208.70628	7793.82711	8020.37726
0.00061266	2989.26379	2922.11359	2985.40042	3516.16855	3410.04231	3421.45881
2.22E-07	362.506111	336.634304	321.53529	646.949135	484.927647	542.189913
2.50E-33	6143.40574	6653.85399	6170.88937	11255.8367	10415.7405	11075.7221
0.08138792	2935.05727	2784.69009	3164.58417	2727.96885	2815.30139	2185.82429
0.00512771	1037.82902	1087.6697	904.877954	1190.38641	1195.31264	1320.95769
5.01E-13	20101.585	20903.2858	22914.6156	33908.7607	32644.6684	29216.9778
2.42E-22	2702.42095	3032.90463	2993.36414	4710.86795	4677.26806	4599.69412
1.77E-21	1606.99749	1444.54467	1511.11632	2479.97169	2810.44239	2559.69487
3.64E-48	921.510861	895.915979	960.62401	342.883042	352.762998	317.247031
5.76E-08	514.961952	590.17533	557.460565	857.207604	784.241705	798.159401
6.34E-131	3297.56338	3513.35422	3561.77483	1021.10139	975.686087	923.041484

0.07659739	4966.67251	4919.54814	4607.01339	4400.33237	4150.55306	4612.88042
6.61E-28	2040.64966	2302.1099	2384.13938	4027.25837	4047.54238	3971.40537
3.98E-07	2380.56973	2277.60804	2203.96016	2973.80952	2820.16038	3080.16616
4.74E-36	1028.7946	1033.33948	1026.32472	1878.30899	1978.58254	1984.92702
7.76E-13	495.763809	447.42534	428.050077	779.573708	732.736364	787.300089
3.04E-27	3234.32244	3455.8281	3842.49604	6637.69813	6668.48399	6113.79241
3.24E-07	970.07087	883.132397	1052.20682	611.366933	724.961973	609.672778
3.37E-33	944.096912	1037.60067	1049.22042	1972.11661	1995.10312	1895.72553
1.09E-45	482.212179	477.253696	469.859619	136.937567	122.44666	129.536074
0.00187029	15.8102354	9.58768586	15.9274447	1.07824856	0.97179889	3.10266045
1.34E-09	2468.65532	2720.77219	3035.17369	4076.8578	4302.15369	3786.79708
3.46E-49	4876.32831	4746.9698	4177.96785	10115.0497	9997.86699	11068.7412
0.00374738	3827.20626	3831.87845	3162.59324	2841.18495	2489.74876	3222.11288
0.00031796	1208.3537	1192.06894	902.887023	728.896026	653.048855	892.790544
1.08E-17	3479.38108	3427.06505	2978.43216	1995.83808	1876.54366	2073.35285
0.03740584	1532.46353	1445.60997	1301.07314	1222.73387	1066.06338	1330.26567
2.76E-07	7025.39102	6747.60025	5946.90968	4746.45016	4440.14913	5158.94866
3.35E-05	4201.0054	4347.48289	4121.22632	3355.50951	3483.89903	3612.27243
0.02464641	5.64651263	2.13059686	5.97279177	11.8607341	18.4641789	14.7376371
0.00782754	1542.62725	1493.5484	1404.60153	1239.98584	1172.96126	1334.91966
0.0001495	2472.04323	2666.44197	2680.78804	2269.71322	2000.93392	1998.11333
1.14E-11	4894.39715	5520.37646	5550.71449	3777.1047	3836.66202	3685.18495
0.00774916	6241.65506	6760.38383	7662.09638	5851.65493	6111.64323	5262.88779
5.11E-30	1008.46716	1043.99246	948.678427	1797.44035	1867.79747	1868.57726
2.19E-55	329.756338	383.507434	307.598776	32.3474568	40.8155534	40.3345858
1.54E-06	836.813172	847.977549	830.218056	586.567216	619.035894	459.193746
2.25E-21	4291.3496	4020.43627	3040.15101	7934.83114	8005.67927	9571.70749
2.71E-09	2405.41438	2228.60431	2051.65397	2993.218	3153.4874	3331.48166
0.0018482	17323.5008	17095.9092	15030.5305	19175.5724	19075.4404	20078.0914
7.44E-11	363.635413	372.85445	314.567033	190.849995	176.867398	185.383962
8.71E-144	4493.49475	4596.76272	4955.42624	1161.2737	1258.47956	1106.87412
1.85E-30	679.840121	549.693989	596.283712	229.666943	219.626549	203.224259
0.03428343	469.789851	548.628691	522.61928	611.366933	626.810285	587.954155
5.05E-06	460.755431	468.731309	472.846015	309.457336	321.665433	339.741319
6.76E-07	1594.57517	1385.95326	1377.72397	968.267206	1123.39952	988.197353
6.74E-39	1587.79935	1601.14354	1733.10508	3083.79088	3220.54153	3067.75552
0.00440472	105.025135	149.14178	165.247239	100.277116	75.8003135	82.996167
1.17E-12	197.627942	235.430953	207.056781	80.8686419	68.9977213	94.6311437
3.08E-08	3011.84984	3707.23853	4573.16757	9994.28589	10015.3594	7391.31285
0.00203955	5268.19628	5578.96787	5548.72356	4817.61456	4838.58668	4638.47737
3.87E-08	351.213086	255.671623	268.77563	506.776823	522.827804	478.585374
0.00550565	1.12930253	1.06529843	2.98639589	7.54773991	10.6897878	19.3916278
4.45E-10	11630.6867	11323.057	11730.563	9252.45088	8557.66104	8281.7764

2.18E-14	3042.34101	3251.2908	3336.79967	4624.60807	4507.20326	4522.90327
6.43E-20	3396.942	3448.37101	3639.42112	2355.9731	2236.10925	2196.6836
7.90E-11	8147.91773	7826.74756	7788.52047	6134.15605	6135.9382	6152.57567
0.00287188	6132.11272	6484.47154	5881.20897	5340.56511	5178.71629	5492.48466
5.40E-86	16197.5861	15142.1519	14788.6324	35231.7717	36563.9333	38258.1303
0.00010639	1173.34532	1151.5876	1175.64451	1422.20985	1685.09928	1424.12115
1.03E-10	3320.14943	3020.12105	3462.2283	4610.59084	5206.89846	4539.9679
4.32E-12	7242.2171	6767.84092	7638.20521	4972.88235	5245.77042	4559.35953
8.49E-08	1919.81429	1796.09315	1781.88288	2472.42395	2377.99189	2345.6113
5.34E-14	1267.07743	1214.44021	1060.17054	2033.57678	1836.6999	2276.5771
0.00023531	1265.94813	1274.09692	1264.24093	1504.15674	1525.72426	1594.76747
3.76E-05	6849.21982	6567.56481	6834.86472	7887.38821	8306.93692	7900.92483
4.56E-10	8718.2155	8242.21394	8330.05359	10631.5308	10850.1346	11250.2468
0.04917151	6700.15189	6547.32414	6440.66046	7045.27608	7287.51989	7087.25213
1.00E-15	12118.5454	12604.611	12859.4207	17046.0315	17939.4075	17038.2599
7.61E-14	5167.68836	5040.99217	4934.52147	3554.9855	3747.25652	3702.24958
8.67E-05	8308.27869	7786.26622	8574.93806	9520.93477	10642.1697	9950.23206
2.83E-43	6348.9388	5911.34098	6311.24997	10725.3384	11235.9388	11112.1784
1.37E-27	2453.97439	2530.08377	2645.94676	4205.16938	4702.53484	4328.21133
4.25E-08	5937.87268	5805.87644	6366.99603	4397.09762	4863.85345	4348.37862
9.11E-09	830.037357	774.471958	785.422118	1108.43952	1343.99787	1144.10604
5.77E-34	2376.05252	2292.52222	2194.00551	4010.00639	3989.23445	4283.99842
9.31E-25	4516.0808	4480.64519	4935.51693	2721.49936	2886.24271	2603.90778
2.95E-05	26292.4214	26944.5932	27659.9987	31779.2198	33347.279	31059.1824
0.00045469	16200.974	15614.0791	13260.5932	18647.2306	17655.6423	20619.5057
1.91E-11	996.044828	1047.18836	1098.99369	1640.01606	1815.32033	1492.37968
0.00291263	613.211272	526.257424	480.809738	722.426534	627.782084	787.300089
4.71E-19	14003.3513	12831.5196	12925.1214	19010.6003	20193.981	20722.6691
7.62E-27	10255.1962	9422.5646	9268.77737	15193.6004	15507.9667	15926.7318
9.83E-16	4292.4789	4113.11723	3989.8249	5821.46397	5728.75446	5935.38944
5.19E-16	1376.61978	1579.83757	1631.56762	2539.27536	2651.06738	2381.29189
3.54E-12	1591.18726	1458.39355	1520.07551	1068.54432	1013.58624	1026.98061
0.00052144	2213.43295	2195.58006	2469.7494	3116.13833	3149.60021	2553.48955
2.03E-06	1270.46534	1201.65663	1233.3815	971.501951	936.814131	847.026303
3.43E-10	7163.16592	7524.2028	7860.19397	9863.81782	10250.5347	9708.22455
1.67E-09	999.432736	927.874931	976.551455	1360.74968	1318.7311	1435.75612
1.83E-05	2451.71578	2866.71807	2884.85843	3390.01347	3655.90743	3547.11656
3.82E-10	2281.1911	2378.81139	2460.79021	3447.16064	3279.82126	3100.33345
1.23E-07	507.056834	467.66601	518.637419	328.86581	327.496226	325.779347
8.09E-19	1323.54256	1187.80775	1101.98008	2069.15898	2102.001	2392.15121
0.00916261	3906.25744	3745.58928	3895.2557	4309.75949	4442.09273	4239.7855
4.10E-06	16.9395379	28.7630576	32.8503548	3.23474568	1.94359778	2.32699534
8.82E-07	658.383373	803.235015	832.208987	1173.13443	1244.87438	1029.3076

1.66E-23	1474.8691	1466.91594	1525.05283	2362.44259	2366.3303	2403.01052
0.00631816	1128.17322	1077.01671	1109.9438	1318.69799	1346.91326	1234.85886
6.31E-10	741.95176	728.664125	797.367702	447.473152	524.771401	442.129114
2.16E-60	346.695876	419.727581	465.877758	4.31299423	3.88719557	8.53231624
4.74E-40	4341.03891	4430.57617	4531.35802	8220.56701	7727.74478	7599.96677
6.70E-86	3789.93928	3715.76092	3487.11493	1334.87172	1149.63809	1226.32654
1.03E-05	1705.24681	1690.62861	1745.05066	1257.23782	1437.29056	1227.87787
9.23E-14	415.58333	337.699602	401.172514	731.052523	861.013818	678.706973
7.67E-15	2837.93725	2712.2498	2600.15535	1743.52792	1717.16864	1905.03352
0.00015902	1740.25519	1670.38794	1682.33635	1447.00957	1293.46432	1380.6839
2.76E-05	2321.84599	2115.68268	2386.13031	2924.21009	2978.5636	2723.36021
1.46E-06	525.125675	517.735036	463.886828	348.274284	337.214215	335.862994
9.11E-09	1075.096	1136.67342	1094.01636	1617.37284	1451.86754	1775.49744
2.27E-15	176.171194	149.14178	117.464905	33.4257053	40.8155534	29.4752743
9.14E-05	1491.80864	1514.85437	1417.54258	1187.15166	1180.73565	1208.48624
3.52E-12	1758.32403	1663.99615	1550.93493	1080.40506	1056.34539	1151.08703
3.45E-05	2717.10188	2834.75912	3164.58417	3740.44425	3906.63154	3498.24966
1.12E-37	1297.5686	1198.46073	1398.62874	562.845748	575.304944	542.189913
2.92E-10	8412.17452	7778.80913	7764.6293	5951.93204	6151.48698	6068.80384
0.00022924	2700.16234	2961.52963	3188.47534	3699.47081	4040.73979	3470.32571
5.80E-60	2914.72982	2917.8524	3176.52976	1273.41155	1330.39268	1219.34556
0.00249683	1359.68024	1437.08758	1044.24309	780.651957	479.096853	809.794377
4.62E-06	884.243878	873.544712	843.159105	653.418627	543.23558	653.88569
0.04230131	7.90511768	10.6529843	6.96825707	19.4084741	23.3231734	17.0646325
1.86E-26	7324.65619	6918.048	6889.61531	4467.18378	4359.48983	4595.04013
2.11E-29	2793.89445	2885.89344	2808.2076	4594.41711	4806.51732	5134.12738
8.19E-15	432.522868	421.858178	519.632884	836.720882	811.452074	839.269651
0.00536714	2856.00609	2906.13411	3226.30302	2505.84965	2710.34711	2344.83563
2.93E-13	4766.78596	4559.47728	4371.08811	3095.65161	2798.78081	3260.12047
0.00149061	474.307061	493.233173	533.569398	658.809869	636.528274	611.224108
4.25E-07	535.289397	640.244356	657.007095	384.934735	400.381143	415.7565
0.03079246	2383.95763	2491.73302	2707.6656	3131.23381	2998.97138	2640.36404
0.07997691	3251.26197	3101.08373	3700.1445	3778.18295	4541.21622	3384.22688
0.07749687	12.4223278	5.32649214	11.9455835	22.6432197	18.4641789	20.942958
0.02468498	13.5516303	18.1100733	42.8050077	6.46949135	10.6897878	10.0836465
6.31E-16	649.348953	715.880544	488.77346	1392.01889	1222.52301	1656.04501
8.62E-29	993.786223	1097.25738	1185.59917	2182.37508	2316.76856	2078.7825
0.03576924	729.529432	810.692104	874.018529	990.910425	1007.75545	864.090935
5.12E-07	832.295962	863.957026	737.639784	1130.00449	1116.59693	1375.25424
8.01E-09	2617.72326	2831.56322	2822.14411	2113.36718	2016.4827	2094.2958
0.00016419	5744.76195	5960.34471	6278.39962	7424.81958	7335.13803	6840.59063
4.32E-120	14597.3645	15501.1574	15270.4376	42137.9537	38794.2117	39457.3086
0.00054531	3943.52442	3962.91016	4166.02226	4640.7818	4829.84049	4627.61806

1.44E-13	15.8102354	21.3059686	25.8820977	107.824856	87.4619002	127.984744
1.55E-28	6869.54727	6717.77189	6300.29985	4135.08322	3903.71615	4038.11257
0.00029328	155.843749	160.860063	153.301656	92.7293761	67.0541235	107.817451
1.56E-38	1103.32857	1107.91037	946.687496	379.543493	399.409344	426.615812
0.00097461	9335.94398	9209.50492	8932.3101	8174.20232	7195.19899	7917.2138
3.67E-08	1473.7398	1591.55585	1630.57215	2233.05277	2207.92708	2006.64565
2.25E-11	115.188858	126.770513	116.46944	45.2864395	27.210369	34.1292649
2.11E-13	842.459685	884.197696	781.440257	490.603094	487.843043	525.125281
1.21E-08	4.5172101	13.8488796	18.9138406	83.025139	57.3361346	65.9315345
2.64E-23	7087.50265	7509.28862	7918.92643	12031.0974	12372.9435	11486.049
1.36E-48	3193.66754	3487.78706	3703.1309	7391.39387	7605.29812	7083.37381
0.00599539	2468.65532	2509.8431	2657.89234	2968.41828	3131.13603	2790.06741
4.85E-08	2705.80885	3048.8841	3125.76103	3966.87645	4035.8808	3886.85788
0.0002441	544.323818	504.951455	441.986591	346.117787	258.498505	380.075905
1.56E-24	8286.82194	7797.9845	8240.46172	5358.89534	4890.09202	5089.91447
3.47E-13	1609.2561	1828.0521	1932.19814	2895.09738	2770.59864	2670.61498
2.17E-11	740.822457	665.811518	778.453861	368.761007	468.407066	369.992259
5.75E-06	3431.95038	3677.41018	3496.07412	2850.88919	2342.03533	2777.65677
0.00010087	5917.54524	5988.04247	7126.53605	8195.76729	8834.62372	7604.62076
0.04368214	313.946102	363.266764	323.526221	304.066094	250.724114	255.969487
0.08582426	3460.18294	3940.53889	3793.71824	3571.15923	2981.479	3399.74019
1.31E-27	372.669834	389.899225	499.723578	1021.10139	1126.31492	1033.18593
7.34E-06	1725.57426	1875.99053	1930.20721	1399.56663	1449.92395	1462.9044
7.21E-176	257.480976	319.589529	320.539825	2062.68949	2177.80132	2064.04486
0.00622823	9929.95711	9499.26609	8163.81089	7553.13115	6650.01981	8315.90567
1.22E-05	8118.55586	7234.44163	7120.56326	5106.58517	6168.97936	5760.86479
0.00016578	271.032606	280.173487	284.703075	359.05677	391.634953	430.494137
0.01620664	0	0	0	4.31299423	4.85899446	3.87832556
1.84E-12	3165.43498	3444.10982	3275.08082	2350.58186	2293.44538	2413.86983
1.63E-07	823.261542	877.805905	953.655753	616.758176	640.415469	567.011197
4.18E-11	126.481883	176.839539	171.220031	425.908181	379.001568	300.958064
3.23E-05	8930.52438	8837.71577	9921.8026	7488.43624	7742.32177	7229.19885
1.46E-05	10687.7191	10374.9414	11167.1297	12830.0796	14302.9361	12847.3413
4.55E-18	90.3442021	111.856335	121.446766	326.709313	362.480986	273.809785
7.52E-07	81.3097819	77.7667853	82.6236195	172.519769	148.68523	156.684353
5.15E-08	1436.47281	1655.47376	1642.51774	2210.40955	2263.31962	2080.33383
0.00037997	1968.3743	1819.52972	1856.54278	2156.49712	2317.74036	2374.31091
2.07E-36	2636.9214	2344.72184	2420.9716	4467.18378	4717.11182	4819.20734
2.03E-13	2091.46828	2163.62111	2312.46588	1513.86098	1481.02151	1369.82459
1.87E-09	103.895832	75.6361884	112.487578	229.666943	218.654751	206.32692
0.00024331	479.953574	439.968251	428.050077	629.697158	654.992453	546.843904
1.88E-19	3337.08897	3155.41395	2844.04435	5887.23713	5103.88778	6499.29798
3.23E-06	648.21965	705.22756	724.698735	495.994337	517.968809	456.866751

1.71E-26	7404.83666	7902.38374	7614.31405	11488.7384	11806.3847	11614.0337
5.37E-24	4527.37383	4378.37654	4113.2626	2638.47422	2739.50107	2732.66819
0.0003335	599.659641	486.841382	535.560329	731.052523	723.990174	690.34195
2.69E-09	1258.04301	1187.80775	1197.54475	892.789807	843.521438	777.216442
2.27E-43	11766.203	12058.1129	12497.0713	21061.4291	21302.8035	20253.3917
4.11E-128	2972.32425	2914.6565	3130.73835	8833.01219	9336.07195	8595.14511
3.03E-09	1875.7715	1803.55024	1925.22988	2471.3457	2479.05897	2467.39072
3.33E-07	2421.22462	2764.44942	2786.30736	3547.43776	3600.51489	3416.02915
3.21E-10	896.666206	906.568963	821.258869	1359.67143	1209.88962	1338.79798
4.95E-12	2084.69246	2093.31141	2447.84916	1441.61832	1308.04131	1096.0148
0.01106798	456.238221	414.401089	426.059146	341.804793	326.524427	368.440928
1.71E-11	2182.94178	2192.38417	2363.23461	1646.48555	1428.54437	1479.19337
0.00996895	2852.61818	2798.53897	2961.50925	2462.71971	2662.72896	2292.86607
0.00306146	2898.91958	2872.04456	2685.76537	2253.53949	1934.85159	2566.67586
0.03908498	27.1032606	17.0447749	17.9183753	4.31299423	8.74619002	11.6349767
3.72E-07	27.1032606	24.5018639	13.9365141	0	0	0
2.50E-16	3873.50766	3971.43254	3895.2557	2795.89851	2803.6398	2727.23853
4.33E-08	217.955388	281.238785	314.567033	543.437274	578.22034	425.840147
0.00012732	6364.74904	6337.46035	6599.93491	7633.9998	7374.98179	7557.30519
0.00609162	322.980523	402.682806	324.521686	455.020892	441.196697	460.745077
1.07E-10	2044.03757	1993.17336	1820.70603	2681.60417	2711.31891	2866.08259
5.60E-17	1195.93138	1236.81148	907.864349	2616.90925	2044.66487	2807.90771
0.00303284	1913.03848	1792.89726	1570.84424	2226.58327	1959.14656	2474.37171
0.00119704	1726.70356	1612.86182	1527.04376	2082.09797	1803.65874	2288.21208
0.02771901	1761.71194	1900.4924	1598.71726	1986.13385	1933.87979	2201.33759
4.41E-06	222.472598	259.932817	283.707609	416.203944	428.563311	367.665263
3.48E-14	433.65217	470.861906	475.832411	828.094893	862.957415	739.208852
0.00017761	2.25860505	3.19589529	4.97732648	28.0344625	20.4077767	18.6159627
8.24E-05	118.576765	108.66044	90.5873419	174.676267	172.008404	186.935292
9.49E-09	5456.78981	5627.9716	5886.18629	7507.84471	7647.08548	7022.09626
5.61E-37	21795.5388	22862.3696	22827.0147	35371.944	36620.2976	36636.2146
0.0136997	316.204707	283.369382	350.403784	244.762423	236.147131	255.193822
4.77E-09	325.239128	306.805947	283.707609	174.676267	173.952002	153.581692
0.0001776	111.80095	98.0074555	100.541995	42.0516938	51.5053412	63.6045392
2.44E-10	4743.07061	4625.52578	4287.46903	3188.38099	2951.35323	3427.66413
5.24E-43	6039.50991	5948.62643	6546.17978	3339.33579	3286.62385	3181.77829
0.00062454	3385.64897	3514.41952	3198.42999	4136.16147	3775.43869	4316.57635
4.58E-14	1649.91099	1743.89353	1590.75354	2487.51943	2362.4431	2453.42875
0.00332876	3516.64807	3508.02773	3680.2352	4005.6934	4633.53711	4038.11257
0.0003642	1663.46262	1498.87489	1427.49723	1169.89969	1015.52984	1292.25808
1.30E-46	5162.04185	5047.38396	5149.54197	2636.31773	2289.55819	2541.85457
1.89E-21	2320.71669	2718.64159	2847.03075	1276.64629	1483.93691	1273.64211
1.35E-07	2107.27851	1945.23493	1974.00768	2757.08156	2863.89133	2503.84698

0.0016584	715.977802	784.059644	736.644319	627.540661	576.276743	591.832481
1.68E-69	3169.95219	3302.42513	3329.83141	6698.08005	6629.61204	6897.98984
0.01186126	589.495919	564.608167	582.347198	439.925412	406.211937	529.779272
4.57E-06	3167.69359	3139.43447	3120.7837	2537.11886	2637.46219	2479.80136
7.42E-10	5623.92658	5585.35966	6115.14331	4264.47305	4447.92353	4053.62588
0.05738891	460.755431	487.90668	494.746252	570.393488	530.602195	576.319178
4.68E-18	588.366616	555.020481	576.374406	1053.44884	951.391115	1121.61175
2.34E-05	941.838307	928.94023	939.719239	1228.12511	1220.57941	1144.10604
1.33E-07	609.823364	585.914136	712.753152	438.847163	403.29654	384.729896
0.01513493	2682.0935	2714.3804	2803.23027	2387.24231	2463.51019	2448.77476
1.55E-07	2156.96783	2476.81885	2583.23244	3283.26686	3288.56745	3223.66421
1.08E-16	4862.77668	4638.30936	4232.71844	2713.95162	2459.62299	2975.45137
0.00027018	2273.28599	2115.68268	2208.93749	1899.87396	1691.90187	1800.31873
2.90E-05	1468.09328	1460.52415	1402.6106	1058.84008	1159.35608	1175.90831
9.13E-07	723.882919	780.863748	709.766756	1027.57088	962.080902	1040.16692
1.27E-08	56.4651263	72.4402932	60.723383	9.70423703	19.4359778	12.4106418
1.38E-30	2580.45627	2346.85244	2474.72672	1361.82793	1409.10839	1362.06794
8.51E-19	502.539624	642.374953	658.998026	1196.8559	1273.05655	1165.049
7.00E-29	2699.03304	2582.28339	2685.76537	4186.83915	4377.95401	4334.41665
7.54E-17	2042.90827	2089.05022	2310.47495	3526.95104	3712.27176	3234.52352
0.00017249	74.5339667	90.5503664	95.5646684	25.8779654	43.7309501	47.3155718
0.02626075	66.628849	71.3749947	63.7097789	35.5822024	53.448939	34.9049301
1.26E-08	2103.89061	2440.5987	2432.91718	1651.87679	1691.90187	1667.67999
2.69E-09	1529.07562	1429.63049	1499.17073	1093.34404	967.911696	1070.41785
0.02637034	488.987994	468.731309	551.487774	599.506199	606.402508	597.262136
0.00804524	2612.07674	2848.608	3232.27581	2401.25954	2571.37987	2231.58853
9.87E-07	297.006564	433.576461	444.972987	637.244898	729.820967	629.064406
7.17E-06	357.988901	408.009298	377.281347	276.031631	201.162371	238.129189
7.32E-13	579.332196	589.110031	568.410684	351.50903	343.045009	320.349691
0.0017481	1005.07925	915.09135	649.043373	589.801962	420.78892	675.604313
7.16E-07	171.653984	111.856335	111.492113	40.9734452	33.0411623	63.6045392
0.01335041	601.918246	684.98689	690.852915	810.842916	921.265349	723.69555
8.29E-09	2761.14468	2782.5595	2668.84246	2128.46265	1909.58482	2074.90418
3.39E-09	2222.46737	2008.08754	2042.69479	1497.68725	1545.16024	1542.02224
2.89E-33	2319.58739	2236.0614	2307.48855	1299.28951	1282.77454	1220.89689
0.01452881	132.128396	159.794764	178.188288	244.762423	239.062527	180.729971
2.43E-09	118.576765	132.097005	160.269913	47.4429366	45.6745479	58.1748834
2.87E-12	397.514489	384.572733	398.186118	617.836424	671.513034	663.193671
0.00947841	1296.4393	1278.35811	1203.51754	1396.33188	1507.26008	1493.15534
6.24E-07	3031.04798	3047.8188	3170.55697	4109.20526	4069.89376	3702.24958
8.83E-26	2951.9968	3237.44193	3665.30322	7693.30347	7910.44298	6200.66691
3.45E-10	6670.79002	6571.82601	6039.48795	4414.3496	3586.90971	4569.44318
2.01E-06	2469.78462	2525.82257	2379.16206	3073.00839	2988.28159	3180.22696

0.03394192	95.9907147	79.8973822	97.555599	130.468076	129.249253	118.676762
2.58E-07	3656.68158	3663.5613	3238.24861	2545.74485	2339.11993	2767.57312
5.53E-10	752.115482	782.994345	886.959578	503.542077	531.573994	505.733653
8.12E-06	1436.47281	1460.52415	1425.5063	1048.0576	1191.42544	1080.5015
2.05E-08	1239.97417	1244.26856	1240.34976	1632.46832	1644.28372	1806.52405
0.07366614	0	0	1.99093059	6.46949135	5.83079335	3.87832556
8.30E-24	125.35258	144.880586	108.505717	397.873718	346.932204	424.288816
0.00276106	102.76653	80.9626806	59.7279177	42.0516938	35.956559	48.091237
1.44E-06	3501.96713	3316.27401	3284.04001	2572.70106	2770.59864	2662.08267
3.03E-09	3050.24612	3100.01843	3244.2214	2164.04486	2448.93321	2268.82045
1.31E-54	828.908054	859.695832	804.335959	264.170897	245.86512	279.23944
4.85E-39	963.295055	1059.97194	1012.38821	380.621741	411.070931	436.699458
6.55E-13	194.240035	201.341403	201.08399	63.616665	86.4901013	79.1178415
9.74E-18	696.779659	689.248083	636.102324	350.430782	376.086171	331.209003
4.31E-13	283.454934	270.585801	243.888997	131.546324	100.095286	96.1824739
0.00063262	136.645606	159.794764	124.433162	277.10988	180.754594	228.821208
3.42E-12	935.062492	863.957026	898.905162	583.33247	576.276743	580.197504
9.06E-18	1874.64219	1753.48121	1653.46786	1011.39715	868.788209	1057.23155
1.17E-25	745.339667	879.936502	919.809933	1891.24797	1890.14884	1642.85871
7.64E-23	612.081969	639.179057	719.721409	296.518354	259.470304	216.410566
4.87E-10	1264.81883	1274.09692	1118.90299	842.112124	745.36975	822.205019
2.24E-14	231.507018	223.71267	218.0069	97.0423703	69.9695202	86.8744926
3.46E-10	92.6028072	98.0074555	68.6871054	201.63248	191.444382	215.634901
0.08302112	102.76653	106.529843	121.446766	135.859318	150.628828	138.844055
0.01952085	44.0427985	79.8973822	36.8322159	88.4163818	84.5465035	100.060799
2.67E-10	993.786223	1128.15104	1205.50847	1635.70306	1674.40949	1652.16669
0.01089877	278.937724	325.981319	311.580637	390.325978	417.873523	361.459942
1.36E-07	110.671648	88.4197696	114.478509	40.9734452	42.7591512	38.0075905
3.98E-40	3066.05636	2991.35799	3144.67487	1673.44176	1594.72198	1687.07162
7.23E-12	718.236407	842.651057	934.741912	1675.59826	1360.51845	1402.40252
1.83E-70	1475.9984	1512.72377	1432.47456	3583.01996	3328.4112	3424.56147
0.00015635	56.4651263	53.2649214	60.723383	116.450844	120.503063	91.5284832
0.04798902	13.5516303	23.4365654	20.9047712	35.5822024	36.9283579	33.3535998
0.00168176	392.997279	373.919749	389.226931	469.038123	509.222619	504.182323
1.63E-17	4483.33103	4977.07426	5400.39923	8071.76871	8435.21438	7703.9059
0.03821054	1753.80682	1944.16963	1631.56762	1520.33047	1361.49025	1660.69901
5.27E-12	1894.96964	1886.64352	1996.90338	2648.17846	2813.35779	2641.13971
0.04158899	1479.38631	1385.95326	1706.22752	1236.7511	1427.57257	1227.87787
0.00084266	1180.12114	1294.33759	1132.83951	1528.95646	1392.58781	1547.4519
0.00448333	2055.3306	2078.39723	1804.77858	1751.07566	1486.8523	1649.83969
0.00072867	143.421421	124.639916	158.278982	90.5728789	89.405498	81.4448368
1.06E-35	3369.83874	3844.66203	3876.34186	6893.24304	7001.81101	6922.03546
4.27E-23	1351.77512	1271.96632	1323.96884	762.321731	757.031336	709.733578

4.73E-08	769.05502	728.664125	581.351733	361.213267	409.127333	452.21276
0.00010585	4731.77758	4678.7907	4219.77739	5847.34193	5219.53185	6171.19163
9.99E-05	1735.73798	1605.40473	1811.74684	2075.62848	2436.29982	2122.99541
1.94E-16	273.291211	304.675351	292.666797	105.668359	125.362057	122.555088
2.27E-11	4502.52917	4525.38773	4008.73874	2791.58552	2481.00257	3091.80114
1.91E-10	1322.41326	1507.39728	1443.42468	2036.81153	1975.66715	2071.80151
0.01874506	654.995465	664.74622	793.38584	869.068338	889.195986	798.935066
1.18E-09	7605.85251	7771.35204	8893.48695	5521.71087	5693.7697	4491.101
1.04E-33	128.740488	126.770513	128.415023	401.108464	437.309501	404.897189
2.91E-38	359.118203	382.442136	403.163445	71.1644049	101.067085	89.2014879
8.52E-06	10902.2866	9936.03845	8294.21684	13126.598	12598.4008	14944.7397
0.00125418	629.021507	616.80779	651.034303	894.946304	760.918532	764.030136
0.00012022	687.745238	673.268607	595.288247	463.64688	455.77368	517.36863
0.02270809	1827.21149	1766.26479	1593.73994	1459.94855	1380.92622	1597.09447
0.00061589	2376.05252	2190.25357	2651.91955	3176.52025	3370.19856	2741.20051
9.73E-16	1401.46444	1361.45139	1257.27267	2454.09372	2024.25709	2441.79377
0.07354292	895.536903	894.85068	800.354098	951.015229	987.347674	987.421688
4.38E-10	166.007471	157.664167	177.192823	324.552816	320.693634	294.752743
5.46E-12	921.510861	923.613738	666.961748	369.839256	282.793477	442.129114
2.41E-09	700.167566	850.108146	872.027599	530.498291	460.632674	401.018863
0.00180965	106.154437	107.595141	155.292586	197.319486	207.964963	176.07598
1.07E-14	636.926625	695.639874	734.653388	387.091233	397.465747	380.85157
0.01647654	2577.06836	2615.30764	2677.80164	2267.55672	2419.77924	2294.4174
1.09E-10	159.231656	148.076482	141.356072	65.7731621	50.5335423	53.5208927
0.00315839	1861.09056	1807.81143	1956.08931	2222.27028	2398.39966	2086.53915
4.41E-17	1075.096	1139.86932	917.819002	2059.45475	1746.32261	2121.44408
7.21E-05	719.365709	675.399204	676.916401	533.733037	537.404787	497.201337
1.21E-07	1492.93794	1550.00921	1471.29771	1069.62257	1181.70745	1091.36081
2.60E-06	947.48482	952.376795	996.460761	1360.74968	1268.19755	1217.79423
2.92E-16	1998.86547	2052.83007	1985.95326	2974.88777	2920.25567	2848.24229
3.38E-32	992.656921	988.596942	1026.32472	1889.09147	2009.68011	1822.81301
3.24E-10	431.393565	399.486911	456.918571	227.510446	263.3575	199.345934
3.25E-44	2070.01153	2181.73118	2191.01912	4045.58859	4076.69635	4375.5269
0.00521073	118.576765	92.6809633	111.492113	150.954798	146.741633	184.608297
1.27E-17	1026.536	1139.86932	1136.82137	669.592355	620.007693	608.121448
1.18E-10	1923.2022	2000.63045	1929.21174	1399.56663	1440.20596	1427.99947
1.02E-08	9.03442021	10.6529843	10.9501183	58.2254222	51.5053412	51.9695625
3.51E-08	7057.01149	6729.49017	6551.15711	5445.15522	4772.50436	5131.02472
1.67E-09	559.00475	542.2369	490.764391	342.883042	280.84988	319.574026
3.82E-05	1792.20311	2014.47933	1955.09384	2368.91208	2345.92252	2589.94581
2.66E-29	556.746145	650.89734	584.338128	242.605926	207.964963	228.821208
0.00161682	18.0688404	19.1753717	10.9501183	1.07824856	0.97179889	4.65399067
0.00180935	2149.06271	2079.46253	1849.57452	1634.62482	1529.61145	1770.84345

0.00451448	3905.12814	3845.72733	3599.60251	3255.2324	3147.65661	3412.15083
0.00080805	6283.43926	6177.66559	5382.48085	4689.30298	4286.60491	5228.75852
0.02701029	286.842842	317.458932	250.857254	225.353949	166.17761	245.110175
7.09E-09	1561.82539	1536.16033	1505.14353	1991.52509	2154.47814	2036.89658
1.43E-06	1610.3854	1738.56704	1750.02799	2248.14824	2134.07037	2167.98399
0.05716772	1893.84034	1979.32448	2245.76971	2344.11237	2416.86384	2192.02961
0.00403317	3274.97733	3463.28519	3740.95858	4059.60582	4424.60035	3927.96813
1.27E-14	3497.44992	3348.23296	3336.79967	4863.97925	4617.98833	5041.82323
3.59E-11	58.7237314	39.4160419	52.7596607	2.15649712	1.94359778	5.42965579
2.98E-07	1157.53509	1129.21633	1151.75335	883.08557	790.072499	852.455958
0.0067543	25.9739581	34.0895497	42.8050077	12.9389827	9.71798891	18.6159627
0.00670985	5611.50425	5718.52197	6241.5674	5072.08122	5306.99375	4846.35562
0.00271129	65.4995465	67.113801	48.7777995	26.956214	30.1257656	33.3535998
1.42E-09	3635.22483	3780.74412	4176.97238	5377.22556	5339.06311	5134.12738
9.88E-05	2167.13155	2523.69198	2338.34798	1839.49204	1824.06652	1935.28446
1.68E-17	18.0688404	29.828356	34.8412853	131.546324	138.967241	147.376371
0.01827025	163.748866	173.643644	167.23817	112.13785	123.418459	134.190064
3.31E-48	1094.29415	1226.15849	1025.32925	2630.92648	2593.73124	2790.84307
9.95E-05	1084.13043	989.66224	984.515177	1354.28019	1210.86142	1364.39493
8.25E-14	1538.11004	1703.41219	1487.22515	997.379917	967.911696	1040.16692
0.00112327	2339.91483	2294.65282	2557.35034	1968.88187	2072.84704	1980.27303
0.00025202	156.973051	202.406701	193.120267	312.692082	280.84988	253.642492
9.90E-09	601.918246	561.412272	553.478704	331.022308	389.691355	364.562603
1.04E-05	334.273548	393.09512	468.864154	679.296592	677.343827	544.516909
0.03657822	1373.23187	1394.47564	1524.05737	1684.22425	1716.19684	1521.07929
1.19E-11	3427.43317	3105.34492	2697.71095	1770.48413	1645.25552	2050.08289
0.00057258	3663.4574	3493.11355	3150.64766	4579.32163	3859.0134	4493.428
4.87E-17	1433.08491	1460.52415	1621.61297	774.182465	907.660164	702.752592
4.46E-65	5038.94787	5136.86902	4816.0611	10395.3944	9925.95388	10632.0417
0.00141042	451.72101	463.404817	503.70544	360.135019	383.860562	342.84398
6.49E-06	4195.35888	4336.8299	4219.77739	3573.31572	3358.53697	3512.98729
6.75E-07	38.3962859	29.828356	29.8639589	6.46949135	3.88719557	2.32699534
7.87E-07	590.625221	567.804063	534.564864	791.434442	755.087739	844.699307
0.00736026	714.848499	707.358157	726.689666	832.407887	846.436834	871.071921
2.35E-17	91.4735046	96.942157	105.519321	15.0954798	6.80259224	15.5133022
4.06E-05	422.359145	420.792879	357.372041	266.327394	199.218773	286.220426
0.04758222	32.7497733	25.5671623	29.8639589	37.7386996	68.0259224	44.9885765
6.98E-54	40268.6695	41312.2731	45168.2423	101305.765	101225.488	88329.6403
8.99E-19	10195.3432	9986.10747	11540.4292	19889.3729	18597.3154	16436.3437
1.59E-17	5961.58804	5964.6059	6000.6648	4342.10695	4045.59878	4183.16195
1.22E-48	5847.52848	5816.52942	6084.28389	3032.03495	2862.91953	2639.58838
3.52E-25	618.857784	586.979434	599.270108	286.814117	250.724114	239.68052
2.94E-06	4212.29842	3986.34672	4029.64352	3180.83325	3428.50649	3094.12813

3.32E-05	2350.07856	2296.78341	2295.54297	2876.76715	2980.5072	2697.76326
0.0009367	6562.37698	7218.46215	8135.93786	9345.18026	10238.8731	8307.37335
7.59E-13	223.6019	209.86379	200.088524	63.616665	75.8003135	94.6311437
0.04328195	85.826992	100.138052	66.6961748	131.546324	112.728671	112.471441
1.27E-10	16.9395379	9.58768586	12.9410488	52.8341794	89.405498	91.5284832
0.00010535	11663.4365	11395.4973	12033.1845	13886.7632	14838.3973	13332.9076
0.01864035	4273.28076	4498.75526	4943.48066	5495.8329	5299.21935	4959.60273
7.80E-19	641.443835	593.371225	663.975352	1138.63048	1191.42544	1064.9882
2.71E-42	1970.63291	1962.27971	2055.63584	3638.01064	3781.26949	3616.15075
1.43E-68	4579.32174	4411.40079	4625.92723	9207.16444	9040.64509	8887.57086
9.22E-11	6802.91842	6919.1133	7377.3933	4593.33886	5332.26052	4410.43183
6.48E-06	722.753617	838.389863	1017.36553	1357.51494	1364.40564	1165.82466
6.40E-06	123.093975	120.378722	111.492113	225.353949	175.895599	240.456185
3.88E-09	1008.46716	1065.29843	1041.2567	1533.26945	1425.62897	1400.07553
0.00515715	404.290304	366.46266	424.068216	313.770331	299.314059	316.471366
2.99E-09	1442.11933	1274.09692	1285.1457	904.650541	897.942176	960.273409
3.73E-85	1900.61615	1889.83941	2157.1733	540.202528	596.684519	526.676611
1.34E-18	206.662362	193.884314	214.025039	67.9296592	66.0823246	58.9505485
0.02248452	13.5516303	8.52238743	14.9319794	5.39124279	1.94359778	2.32699534
4.92E-09	2102.7613	2382.00729	2480.69952	1641.09431	1680.24028	1495.48234
4.49E-09	137.774908	100.138052	93.5737378	38.8169481	36.9283579	31.8022696
1.83E-05	753.244785	765.94957	714.744082	577.941227	452.858283	544.516909
8.29E-05	653.866163	650.89734	540.537655	404.34321	422.732518	475.482714
3.32E-06	435.910775	406.944	374.294951	286.814117	228.372739	260.623478
1.11E-06	644.831742	689.248083	718.725943	470.116372	486.871245	494.874342
0.00053263	284.584237	337.699602	362.349368	239.37118	238.090728	197.794604
9.71E-32	7061.5287	8088.81097	8321.0944	15195.7569	15597.3722	14128.74
0.02702162	14.6809328	8.52238743	22.8957018	35.5822024	46.6463468	22.4942883
0.01913137	77.9218743	95.8768586	102.532925	72.2426534	58.3079335	30.2509394
3.63E-08	82.4390844	116.117529	136.378745	18.3302255	39.8437545	37.2319254
1.54E-19	1752.67752	1633.10249	1711.20484	1084.71805	989.291271	975.786711
5.29E-05	387.350766	462.339518	429.045542	632.931904	582.107536	565.459867
2.67E-21	3005.07402	3100.01843	2428.93532	6464.10011	5230.22163	6902.64383
1.47E-30	70725.9586	72261.323	62419.6559	130583.448	120933.569	139725.211
1.10E-15	3714.27601	3750.91577	3573.72041	5204.70579	5065.01582	5421.89913
8.04E-33	2418.96601	2274.41215	2394.09404	1216.26437	1336.22348	1262.00714
0.00197989	269.903304	301.479455	269.771095	196.241238	143.826236	222.615887
1.88E-13	6695.63468	6927.63568	7388.34342	4860.7445	4877.45864	4233.58018
3.17E-06	15140.559	13535.6818	13489.5502	16984.5713	18034.6438	18170.7309
5.88E-06	57.5944288	75.6361884	66.6961748	136.937567	119.531264	152.806027
0.0468558	249.575858	317.458932	302.62145	241.527677	241.006125	214.083571
1.27E-14	718.236407	830.932774	858.091085	441.003661	440.224898	353.703291
1.02E-63	8037.24608	7959.90986	8439.55477	3627.22815	3874.56218	3435.42078

1.30E-21	201.01585	188.557822	196.106663	58.2254222	48.5899446	45.7642416
0.04175614	3674.75042	3673.14898	3747.92684	4071.46656	3950.36249	4156.01367
9.28E-16	2282.32041	2210.49424	2188.03272	3335.02279	3227.34412	3122.05208
3.70E-75	2258.60505	2386.26848	2219.88761	905.728789	868.788209	888.136554
2.38E-42	2941.83308	2810.25726	3172.5479	1431.91409	1493.6549	1409.38351
5.14E-12	684.357331	681.790994	753.567229	411.890949	384.832361	450.66143
1.33E-16	3681.52624	3822.29076	4119.23539	2495.06716	2606.36463	2314.5847
0.07659739	8187.44332	8133.5535	6171.88483	9061.60089	7549.90559	10037.1066
1.33E-14	9207.2035	10007.4134	9551.48951	13795.1121	12922.9817	14012.3903
3.49E-24	2575.93906	2595.06697	2640.96943	1638.93781	1454.78294	1552.10589
1.91E-05	1474.8691	1438.15288	1480.25689	1728.43244	1933.87979	1917.44416
0.03285803	1689.43658	1715.13047	1565.86691	1387.7059	1351.77226	1567.61919
1.05E-11	884.243878	810.692104	954.651218	1337.02821	1481.02151	1339.57365
7.22E-11	1069.44949	1025.88239	1031.30205	1423.2881	1517.94987	1569.94619
2.50E-21	272.161909	231.169759	301.625985	648.027384	581.135737	659.315345
0.02283313	16.9395379	19.1753717	18.9138406	42.0516938	28.1821678	38.7832556
8.76E-167	2176.16597	1965.4756	1721.1595	14349.3318	12654.7652	15996.5416
1.57E-31	84.6976895	80.9626806	71.6735013	286.814117	330.411623	337.414324
2.51E-08	4849.22505	4640.43996	4248.64588	6325.00605	5872.5807	6904.19516
4.30E-20	1034.44111	1009.90291	907.864349	472.272869	373.170774	498.752667
5.97E-06	688.874541	661.550324	616.193018	451.786146	493.673837	462.296407
0.0009206	4852.61296	5454.32796	5508.90495	4352.88943	4619.93193	4093.1848
1.90E-08	3795.58579	3490.98295	4177.96785	2690.23015	2850.28615	2437.91545
2.49E-05	2852.61818	2832.62852	2728.57037	2303.13892	2318.71215	2320.01435
2.42E-21	10098.2232	10900.1335	11095.4562	7169.27467	7065.94974	6816.54501
1.51E-09	1274.98255	1286.8805	1387.67862	1829.7878	1949.42858	1772.39478
7.94E-63	1127.04392	1103.64917	1181.61731	371.995753	405.240138	376.197579
0.00011439	1017.50158	1034.40477	841.168175	752.617494	687.061816	708.957913
1.12E-09	1942.40035	1873.85994	1707.22298	1288.50703	1137.0047	1313.9767
8.75E-22	191.981429	211.994387	178.188288	508.93332	445.083892	462.296407
8.75E-05	617.728482	557.151078	641.07965	442.081909	458.689077	446.00744
0.0006625	68043.8651	63941.3423	64678.3666	55691.5381	58982.3619	55560.1163
8.41E-15	1773.00497	1501.00549	1532.02109	2430.37225	2685.08034	2664.40966
3.04E-12	133.257698	102.268649	122.442231	266.327394	295.426863	252.866827
8.02E-60	11090.8801	11172.8499	11018.8054	19766.4526	19552.5937	20269.6807
6.88E-52	4107.27329	4179.16574	4245.65949	7796.81533	7923.07636	7482.06567
0.00012729	2581.58557	2533.27966	2364.23008	2133.8539	1787.13816	1974.84338
0.07574788	931.674584	1188.87305	1307.04593	1383.3929	1348.85686	1302.34172
9.08E-101	3539.23412	3336.51468	2919.69971	11799.274	11639.2353	13447.7061
3.09E-09	17954.7809	17854.4017	16232.0571	12609.0386	10820.0089	13057.5465
8.85E-23	517.220557	552.889885	499.723578	995.22342	956.250109	993.627009
1.17E-31	1954.82267	1884.51292	1995.90792	3778.18295	3508.194	3357.07861
7.91E-16	5333.69583	5013.29441	5329.72119	7632.92155	7716.0832	7160.94032

9.50E-06	1866.73708	1684.23682	1953.10291	2369.99033	2549.02849	2282.78243
2.62E-11	2456.23299	2467.23116	2667.84699	3503.22957	3486.81442	3411.37516
0.01776329	726.141524	664.74622	792.390375	607.053939	627.782084	583.300164
0.00773473	811.968516	644.505549	701.803033	569.315239	579.192139	584.851495
6.52E-16	275.549816	288.695874	260.811907	113.216099	117.587666	113.247106
3.01E-10	2127.60596	2234.9961	2297.5339	1438.38358	1655.94531	1365.94626
2.73E-09	1750.41892	1862.14165	1975.00315	1294.97652	1386.75702	1304.66872
1.06E-118	5791.06335	5494.8093	5905.10013	2044.35927	2102.9728	2012.85097
0.01512244	2989.26379	3259.81319	3432.36434	2551.13609	3040.75873	2614.76709
1.48E-34	1024.27739	1130.28163	1332.92803	398.951967	392.606752	301.733729
1.41E-08	924.898769	934.266722	930.760051	614.601678	567.530553	685.687959
2.14E-22	1193.67277	1139.86932	1240.34976	2085.33271	1926.1054	2136.18172
5.89E-16	382.833556	394.160419	455.923105	766.634725	732.736364	843.923642
1.22E-23	389.609372	430.380565	374.294951	123.998584	118.559465	157.460018
0.02420116	37.2669834	21.3059686	25.8820977	51.7559308	61.2233302	41.110251
3.99E-06	9274.96165	9924.32016	9828.22886	13337.9347	11473.0577	12207.4175
7.58E-31	2534.15487	2618.50354	2768.38899	4456.40129	4521.78024	4387.93754
5.79E-05	101.637227	104.399246	104.523856	196.241238	162.290415	168.319329
1.08E-11	819.873634	823.475685	824.245265	1174.21268	1344.96967	1233.30753
4.69E-07	254.093068	256.736921	335.471805	478.74236	524.771401	430.494137
2.85E-05	251.834463	257.80222	231.943414	369.839256	334.298819	416.532165
8.59E-133	1590.05796	1605.40473	1748.03706	362.291516	340.129612	345.94664
3.59E-36	1181.25044	1185.67715	1267.22732	2319.31265	2404.23046	2240.89651
8.10E-52	1783.16869	1960.14911	2005.86257	4171.74367	4114.59651	3969.85404
2.74E-06	1430.8263	1302.85998	1255.28174	1843.80504	1649.14272	1957.00308
0.0046599	48.5600086	53.2649214	45.7914036	26.956214	26.2385701	20.1672929
1.21E-13	3664.5867	3489.91765	2900.78587	6123.37357	5184.54709	6745.18382
6.39E-29	442.68659	399.486911	384.249604	115.372596	131.19285	142.722381
0.06402773	23.7153531	17.0447749	26.877563	12.9389827	13.6051845	5.42965579
6.57E-06	2088.08037	2268.02035	2337.35251	2871.37591	2899.84789	2727.23853
9.27E-17	98.2493198	95.8768586	121.446766	297.596602	281.821678	262.174808
0.00252112	796.158281	827.736879	809.313285	1032.96212	929.03974	992.075679
4.37E-116	3955.94675	4189.81872	4210.8182	10686.5215	10269.9707	10470.7034
0.01188714	1171.08672	1153.7182	1162.70347	1333.79347	1304.15411	1377.58124
5.60E-09	1819.30637	1596.88234	1558.89865	2366.75559	2241.94004	2474.37171
1.58E-09	2557.87022	2674.96435	2184.05086	1671.28527	1551.96283	1734.38719
6.72E-24	4048.54956	3863.8374	3879.32826	5892.62837	5936.71943	6192.13459
1.28E-59	778.089441	680.725696	771.485604	166.050278	145.769834	190.037953
1.81E-92	1080.74252	1066.36373	1010.39727	212.414966	168.121208	218.737562
1.88E-11	224.731203	219.451476	287.68947	114.294347	88.4336991	100.060799
5.97E-18	2029.35664	1836.57449	1837.62894	1184.99517	1105.90714	1198.4026
1.82E-07	4031.61002	4081.15828	4071.45306	3286.50161	3058.25111	3281.83909
0.00488699	164.878169	143.815288	169.2291	106.746607	106.897878	114.022772

1.08E-15	286.842842	285.499979	261.807373	542.359025	543.23558	506.509318
2.56E-133	505.927532	459.143623	541.533121	2300.98242	2415.89204	2212.1969
7.44E-29	3.38790758	3.19589529	3.98186118	173.598018	170.064806	183.056966
0.06783118	485.600086	487.90668	552.483239	415.125695	413.014529	467.726063
3.70E-06	398.643792	372.85445	375.290416	511.089817	554.897167	591.056816
1.95E-25	4595.13198	4685.18249	4974.34008	2709.63863	2850.28615	2423.95348
0.02271687	277.808421	245.018639	278.730283	193.006492	241.006125	170.646325
2.21E-06	2815.3512	3025.44754	3480.14667	2261.08723	2398.39966	2157.90034
4.75E-08	2955.38471	3088.30015	3080.96509	2367.83383	2377.02009	2403.78618
0.00010441	469.789851	427.18467	494.746252	629.697158	673.456632	591.832481
5.65E-12	492.375901	480.449591	550.492308	283.579371	290.567869	290.098752
7.11E-21	500.281019	520.930932	455.923105	225.353949	221.570147	215.634901
0.0029531	853.75271	846.912251	798.363167	589.801962	649.161659	735.330526
9.41E-13	10334.2474	10610.3724	11342.3316	15261.5301	16532.2427	14549.1505
4.33E-09	1084.13043	979.009256	973.565059	638.323147	701.6388	708.182248
1.65E-11	752.115482	702.031665	673.930005	1114.90901	1036.90942	1198.4026
0.05678047	3772.99974	3815.89897	3302.95385	4146.94396	3734.62314	4660.19599
8.90E-10	2508.18091	2457.64348	2382.14845	1567.7734	1685.09928	1863.1476
4.88E-27	2457.3623	2409.70505	2675.81071	1269.09855	1410.08019	1179.01097
1.04E-08	458.496826	427.18467	489.768925	724.583031	747.313347	657.764015
0.06204455	1814.78916	1886.64352	1883.42034	2111.21068	1996.07492	2057.83954
4.20E-13	126.481883	112.921633	102.532925	24.7997169	33.0411623	30.2509394
0.01106798	2589.49069	2575.8916	2476.71766	2130.61915	1988.30053	2387.49722
0.0017327	3915.29186	4430.57617	4796.15179	5504.45889	5316.71173	5138.78137
9.76E-09	15190.2483	14367.6799	14487.0064	11969.6373	11230.108	11512.4216
2.15E-48	17635.1882	17665.8438	17480.3706	30170.4729	30038.3037	28905.9361
4.61E-07	11576.4802	11209.0701	11574.275	9202.85145	9233.06127	9663.23597
0.01247143	37885.8412	36117.8779	34762.6436	40035.369	39642.5922	42262.8893
5.68E-11	3333.70106	3510.15832	3844.48697	2312.84316	2598.59024	2219.17789
3.44E-08	5376.60933	6128.66186	6547.17525	8277.71419	9158.23275	8069.24416
3.09E-13	126.481883	140.619393	115.473974	34.5039539	37.9001568	19.3916278
0.00171844	3530.1997	3275.79267	3513.99249	3814.8434	4438.20554	4091.63347
2.91E-27	236.024228	213.059686	233.934344	60.3819193	47.6181457	47.3155718
1.82E-12	2999.42751	3020.12105	2855.98993	4029.41486	4014.50122	4330.53832
1.01E-85	2649.34373	2493.86362	2373.18926	6220.41594	6091.23545	6338.7353
1.15E-63	4927.14692	5219.9623	4926.55775	10662.8	10290.3785	11340.2239
0.05403567	1817.04776	1869.59874	1878.44301	2121.99316	2082.56502	1972.51638
3.36E-05	3072.83217	3078.71246	2880.87657	2457.32847	2449.90501	2512.3793
3.30E-54	2808.57538	3125.58559	3124.76556	6529.87327	6173.83836	6437.24477
0.05253236	135.516303	100.138052	111.492113	86.2598847	79.6875091	86.0988275
1.30E-08	9843.00082	10583.7399	10695.2791	8175.28057	8189.34926	7713.21388
7.03E-06	303.78238	222.647372	238.911671	161.737284	117.587666	142.722381
0.0370674	4396.37473	4314.45864	4447.73894	3994.91091	3941.6163	4062.15819

0.0349135	39.5255884	57.5261152	41.8095424	31.2692082	27.210369	20.942958
9.65E-15	2141.15759	2185.99238	2536.44557	1447.00957	1406.193	1265.1098
3.30E-14	11305.4476	11108.932	11666.8533	8140.77662	8544.05585	7787.67773
0.00039237	1156.40579	1184.61185	1239.35429	1569.9299	1432.43157	1420.24282
4.83E-11	1690.56588	1924.99426	1864.5065	2759.23806	2525.70532	2627.17774
0.08100067	1408.24025	1388.08385	1432.47456	1531.11295	1523.78066	1610.28077
6.39E-08	5393.54887	5249.79066	5888.17722	7467.94952	8048.43842	6926.68945
2.59E-06	1783.16869	1798.22375	1914.27976	2372.14683	2274.00941	2305.27671
2.62E-17	4927.14692	4779.99405	4931.53507	6716.41027	6862.84377	6779.31308
0.00021471	6460.73975	6401.37826	5823.47198	5226.27076	4635.48071	5283.83074
0.0135559	444.945195	507.082052	582.347198	673.905349	657.907849	594.935141
1.62E-05	483.341481	558.216377	576.374406	369.839256	357.621992	396.364872
1.00E-07	926.028072	877.805905	892.93237	1193.62115	1208.91782	1237.96152
1.18E-19	1961.59849	1916.47187	1813.73777	1142.94347	1068.00698	1198.4026
0.02244068	4282.31518	4368.78886	3988.82944	3771.71346	3497.50421	3884.53088
8.09E-09	546.582423	626.395476	688.861984	985.519183	910.575561	985.870358
0.02858058	1893.84034	1760.9383	1839.61987	2067.00249	2039.80587	2052.40989
1.20E-22	4076.78212	3851.05382	4030.63898	5872.14165	6037.78651	6024.59093
1.13E-18	2830.03213	2970.05202	3161.59778	4535.11344	4902.72541	4554.70554
3.31E-07	1226.42254	1168.63238	1249.30895	851.816361	933.898735	903.649856
2.11E-08	534.160095	492.167874	567.415218	366.60451	297.370461	299.406733
4.65E-08	17274.9407	16643.1574	16464.0005	13764.9211	13714.026	13417.4551
5.49E-20	668.547096	789.386136	642.075116	1491.21776	1266.25396	1423.34548
1.47E-26	544.323818	555.020481	541.533121	1034.04037	1088.41476	1029.3076
0.00303176	210.05027	231.169759	235.925275	173.598018	157.43142	154.357357
8.20E-17	129.869791	122.509319	121.446766	33.4257053	20.4077767	24.8212836
0.00578746	116.31816	110.791037	120.451301	201.63248	168.121208	145.825041
9.02E-13	154.714446	164.055958	175.201892	352.587279	322.637232	327.330677
1.54E-25	108.413043	133.162304	141.356072	377.386996	369.283579	405.672854
1.35E-10	570.297776	523.061529	560.446961	336.41355	349.847601	311.04171
1.76E-13	84.6976895	90.5503664	109.501183	17.2519769	7.77439113	20.942958
1.88E-13	27.1032606	42.6119372	48.7777995	146.641804	152.572426	135.741395
3.26E-13	1723.31566	1797.15845	1620.6175	1113.83076	1137.9765	1178.23531
0.01513915	45.172101	67.113801	51.7641954	75.4773991	100.095286	88.4258228
0.03179189	818.744332	776.602555	778.453861	755.85224	587.938329	638.372387
7.62E-07	653.866163	755.296586	709.766756	460.412135	436.337702	523.573951
0.00051537	155.843749	159.794764	149.319794	92.7293761	79.6875091	109.368781
3.66E-16	3715.40531	3652.90831	3406.48224	2431.4505	2257.48882	2455.75575
0.00066776	1150.75927	1066.36373	1114.92113	1427.60109	1303.18231	1345.77897
7.87E-05	3372.09734	3461.1546	3371.64096	2916.66235	2817.24499	2842.81264
8.90E-35	1290.79279	1263.44394	1167.68079	2465.95445	2303.16337	2371.20825
0.05856744	9305.45282	9617.51422	7267.89212	7701.92946	6211.73851	8071.57116
0.04584385	17715.3687	17525.2245	14188.3669	14382.7575	13030.8513	15200.7092

8.13E-11	123.093975	86.2891727	135.38328	22.6432197	38.8719557	23.2699534
4.80E-84	40.6548909	23.4365654	30.8594242	491.681343	546.150977	466.950398
0.02147444	281.196329	323.850722	338.4582	286.814117	223.513745	210.205245
4.44E-07	1360.80954	1425.3693	1341.88722	1025.41438	960.137305	1075.07185
0.00287515	4574.80453	4569.06496	4573.16757	5336.25212	5064.04402	5177.56462
6.21E-06	18.0688404	33.0242513	27.8730283	71.1644049	74.8285146	72.9125206
2.57E-13	3365.32153	3356.75535	2960.51379	2191.00107	1985.38513	2180.39463
8.55E-51	769.05502	749.970094	817.277008	266.327394	255.583108	246.661506
4.44E-14	9263.66862	8815.3445	9597.28091	12696.3768	12533.2903	13398.8391
2.05E-06	2363.63019	2134.85805	2365.22554	1732.74543	1816.29213	1770.84345
2.62E-60	1418.40397	1261.31334	1350.84641	444.238406	488.814842	468.501728
0.01744856	0	0	0.9954653	7.54773991	3.88719557	6.98098601
1.24E-12	867.30434	807.496209	814.290612	1246.45533	1320.67469	1220.12122
9.84E-05	2435.90555	2368.15841	2188.03272	3079.47788	2708.40351	2970.02171
0.00079614	545.45312	500.690262	506.691835	649.105632	661.795045	656.98835
2.73E-11	101.637227	93.7462617	96.5601337	247.997169	203.105968	217.186231
1.65E-23	521.737767	547.563392	583.342663	214.571463	257.526706	224.942883
1.81E-05	71.1460592	89.485068	89.5918766	28.0344625	28.1821678	43.4372463
2.12E-09	2715.97258	2551.38974	2070.56781	1461.0268	1143.8073	1593.21614
2.68E-49	1054.76856	1051.44955	1089.03903	2524.17988	2384.79448	2239.34518
0.00038041	1418.40397	1456.26295	1519.08004	1215.18613	1235.15639	1130.91973
1.98E-22	1059.28577	1073.82082	1101.98008	1728.43244	1801.71514	1796.4404
9.01E-44	18914.688	19497.0918	17656.5679	8996.90597	8126.18233	9537.57822
2.86E-38	7967.22932	8553.28108	8665.5254	14446.3742	14167.856	14314.124
4.66E-25	398.643792	402.682806	387.236	844.268621	811.452074	771.786787
3.87E-20	4125.34213	3998.065	3539.87459	2223.34853	2233.19385	2425.50481
3.28E-20	678.710818	695.639874	552.483239	279.266377	287.652472	291.650082
7.64E-63	500.281019	428.249968	451.941244	75.4773991	75.8003135	77.5665112
3.33E-85	1932.23662	1834.44389	1770.93276	603.819193	617.092296	626.737411
0.02144167	405.419607	431.445864	378.276812	446.394903	514.081614	529.779272
2.63E-14	1806.88404	1766.26479	1696.27286	2483.20643	2652.03917	2824.19667
1.27E-10	3863.34394	4092.87656	4090.3669	5268.32246	5620.88479	5255.9068
4.11E-07	2149.06271	2270.15095	2161.15516	2733.3601	2896.9325	2763.6948
0.00114969	3846.4044	4349.61348	4550.27187	3673.59284	3408.09871	3430.76679
4.09E-06	2098.24409	2134.85805	2310.47495	1549.44318	1797.82795	1580.02983
9.17E-06	1169.95742	1152.6529	1341.88722	919.746021	938.757729	862.539605
1.97E-17	1221.90533	1332.68833	1358.81013	2299.90418	2294.41718	2004.31865
1.13E-16	3052.50473	3016.92515	2875.89924	2023.87254	1873.62826	2035.34525
5.93E-11	1375.49048	1382.75736	1390.66502	2045.43752	1919.30281	1870.90425
5.99E-47	1093.16485	1177.15476	1181.61731	2486.44118	2731.72668	2452.65309
7.40E-26	3075.09078	3264.07439	3241.235	5079.62896	5017.39768	4916.16548
1.44E-36	5317.8856	5545.94362	4941.48973	10077.311	9541.12151	10795.707
6.73E-16	2046.29618	2371.3543	2459.79475	1394.17539	1420.76998	1298.4634

1.13E-15	3032.17728	2877.37106	2858.97633	1993.68159	2061.18545	1967.08672
4.95E-07	1409.36955	1552.13981	1649.48599	2159.73186	2055.35466	2007.42131
0.00110203	6143.40574	6061.54806	5091.80499	4751.8414	4200.11481	4886.69021
5.87E-34	3774.12904	3551.70496	3438.33713	2051.90701	2002.87751	1986.47835
0.0257935	129.869791	110.791037	125.428627	74.3991506	100.095286	81.4448368
2.14E-26	432.522868	441.03355	422.077285	171.441521	123.418459	144.273711
9.77E-09	595.142431	561.412272	546.510447	361.213267	381.916964	362.235607
0.00030809	7.90511768	2.13059686	9.95465295	29.1127111	35.956559	24.0456185
1.85E-09	1019.76018	1063.16783	1061.166	1399.56663	1477.13431	1486.17436
2.61E-07	2.25860505	5.32649214	5.97279177	52.8341794	29.1539667	34.9049301
0.00163653	11421.7658	10801.0608	10849.5763	12233.8081	12835.5198	12706.9459
3.95E-28	5358.54049	4623.39518	4586.10862	2595.34428	2373.13289	2636.48572
1.02E-07	110.671648	85.2238743	95.5646684	31.2692082	36.9283579	38.0075905
6.13E-11	8843.56808	8351.93968	8956.20126	6489.97808	6793.84605	6429.48812
2.32E-05	8811.94761	8111.18224	7280.83317	6161.11226	4739.46319	6296.07372
5.01E-14	4016.92909	4132.29261	4497.5122	2911.27111	2935.80445	2702.41725
1.67E-07	2152.45062	2113.55208	1876.45208	1448.08781	1420.76998	1580.8055
0.01335041	249.575858	279.108188	486.782529	226.432197	256.554907	170.646325
1.48E-12	7536.96506	7608.36138	7849.24385	5370.75607	5835.65234	5152.74334
1.50E-13	2613.20605	2567.36921	2620.06466	1747.84091	1820.17932	1896.5012
0.00322448	2027.09803	2089.05022	1783.87381	1691.77199	1512.11907	1658.37201
1.00E-09	710.331289	671.13801	835.195383	423.751684	481.040451	419.634826
0.00102889	147.938631	171.513047	146.333398	239.37118	243.921522	205.551255
1.19E-13	4503.65847	4053.46052	3994.80223	2857.35868	2834.73737	2930.46279
3.44E-13	1802.36683	1715.13047	1696.27286	1106.28302	1185.59465	1210.03758
3.97E-07	1165.44021	1147.32641	1226.41324	1497.68725	1582.0886	1591.66481
1.34E-05	1804.62544	1643.75548	1584.78075	1219.49912	1063.14799	1352.75996
3.69E-10	1045.73414	1056.77604	1051.21135	758.008737	673.456632	712.060573
1.88E-12	2199.88132	2150.83753	2040.70386	3029.87845	2944.55064	3295.80106
3.88E-17	207.791665	211.994387	244.884463	51.7559308	82.6029058	54.2965579
1.53E-14	4329.74589	4513.66944	4615.97258	3177.5985	3217.62613	2949.07876
0.00034941	2700.16234	2605.71996	2474.72672	2111.21068	2093.25481	2244.77483
2.60E-09	430.264262	426.119372	495.741717	736.443766	781.326309	665.520666
0.00293739	7.90511768	6.39179057	5.97279177	36.660451	15.5487823	21.7186231
9.63E-34	1670.23844	1770.52599	1874.46115	3515.0903	3687.97679	3249.26116
1.06E-06	3.38790758	11.7182827	9.95465295	34.5039539	51.5053412	50.4182323
0.00407322	2.25860505	5.32649214	1.99093059	10.7824856	20.4077767	17.0646325
6.77E-06	7722.17067	7221.65805	7488.88542	8781.25626	9150.45836	9289.36538
0.00016512	4840.19063	4779.99405	4473.62104	3896.79029	3761.83351	4068.36351
7.59E-23	1389.04211	1304.99058	1217.45406	672.827101	618.064095	719.817224
5.76E-14	2912.47122	3025.44754	3109.83358	2079.94147	2161.28073	1955.45175
0.00029364	2965.54843	3057.40649	3110.82905	2582.4053	2501.41035	2629.50473
0.00019317	1198.18998	1169.69767	1128.85765	878.772575	950.419316	959.497744

9.56E-19	2973.45355	3363.14714	3444.30992	5290.96568	5693.7697	5106.9791
1.02E-14	398.643792	430.380565	354.385645	194.084741	178.810996	186.935292
1.24E-07	30370.3328	31115.2365	28785.8699	23343.003	21472.8683	24531.9605
3.34E-05	4313.93565	4125.90081	3963.94281	3298.36234	2980.5072	3495.92266
8.51E-05	1155.27648	1153.7182	1231.39057	943.467489	964.996299	883.482563
7.43E-06	1838.50451	1865.33755	1920.25255	2484.28468	2437.27162	2229.26153
2.09E-08	797.287584	937.462617	902.887023	574.706482	615.148698	582.524499
0.00221741	893.278298	999.249926	1089.03903	1268.02031	1302.21051	1159.61934
0.06694456	3343.86478	3259.81319	2940.60448	2648.17846	2836.68096	3012.6833
4.45E-07	39.5255884	61.7873089	57.7369871	118.607341	136.051845	122.555088
1.78E-12	582.720104	629.591371	574.383475	916.511275	936.814131	1036.28859
1.42E-19	1899.48685	1979.32448	2053.6449	1182.83867	1200.17163	1275.96911
0.01677984	985.881105	963.02978	1151.75335	1261.55081	1448.95215	1114.63077
1.96E-20	2274.41529	2491.73302	2811.19399	1426.52284	1193.36904	1157.29235
0.00017762	963.295055	1042.92716	1112.9302	805.451673	833.803449	651.558694
0.01632861	4059.84258	4069.44	3990.82037	4434.83632	4705.45023	4403.45084
2.01E-30	1625.06634	1627.776	1516.09364	2823.93298	2776.42943	3044.48557
0.04049625	343.307968	370.723853	234.92981	491.681343	297.370461	546.843904
0.06336028	466.401943	399.486911	351.399249	325.631065	221.570147	380.075905
8.86E-59	15.8102354	19.1753717	14.9319794	338.570047	285.708874	358.357282
3.94E-24	9332.55608	9343.73252	8867.60485	6033.87893	6120.38942	6228.59085
8.01E-26	1051.38065	977.943958	887.955044	476.585863	394.55035	412.65384
1.59E-18	7960.45351	8231.56096	7652.14173	5389.0863	5144.70333	5567.72418
3.24E-16	528.513582	546.498094	385.245069	199.475983	161.318616	202.448594
0.00010362	7.90511768	8.52238743	10.9501183	35.5822024	31.0975645	35.6805952
3.09E-05	705.814079	741.447706	825.24073	607.053939	504.363625	474.707049
4.61E-05	6300.37879	6426.94542	6046.4562	8039.42125	7200.05799	7620.90973
3.88E-21	3081.86659	2975.37851	3554.80657	1730.58894	1765.75859	1456.69908
6.64E-11	77.9218743	92.6809633	77.646293	20.4867226	18.4641789	20.942958
3.76E-05	144.550723	139.554094	145.337933	74.3991506	92.3208947	64.3802043
4.40E-19	37.2669834	35.1548482	24.8866324	154.189544	139.93904	173.748985
3.56E-06	4901.17296	5324.36155	5234.15652	4126.45723	4232.18417	4188.59161
1.15E-05	4396.37473	4765.07987	4734.43295	3810.53041	3839.57742	3678.97963
0.00146678	1111.23369	969.42157	935.737378	733.20902	754.11594	861.76394
0.00069481	92.6028072	115.05223	83.6190848	162.815532	154.516024	154.357357
5.78E-12	2985.87588	2736.75166	2697.71095	2033.57678	1915.41561	1951.57342
0.00011026	3252.39128	3335.44938	3101.86986	3783.57419	3840.54922	4138.94904
2.99E-15	2907.95401	2966.85612	2895.80854	1980.7426	2056.32645	2091.19314
0.01484917	1244.49138	1159.04469	1205.50847	1382.31465	1419.79818	1350.43296
4.34E-06	2783.73073	2868.84867	2761.42073	3823.46939	3293.42644	3761.20013
5.73E-16	2035.00315	2133.79275	2400.06683	3660.65386	3681.1742	3320.62235
0.00027402	1027.6653	906.568963	853.113758	722.426534	690.949012	726.79821
1.61E-18	881.985273	934.266722	1026.32472	2009.85531	2312.88136	1664.57733

3.78E-06	1375.49048	1481.83011	1775.91009	2234.13101	2663.70076	2030.69126
4.21E-18	3041.2117	2932.76657	3163.58871	1858.90052	2057.29825	1822.81301
2.78E-35	16287.9303	16261.7805	16086.7192	10490.2802	9986.20541	10289.9734
1.71E-17	2557.87022	2618.50354	2585.22337	3866.59933	3734.62314	3657.261
0.04971657	670.805701	561.412272	663.975352	697.626818	753.144141	772.562452
1.62E-19	1893.84034	1819.52972	1595.73087	1021.10139	993.178467	1015.34563
2.41E-38	1024.27739	903.373068	946.687496	388.169481	385.80416	404.121523
2.21E-14	44.0427985	57.5261152	33.84582	150.954798	157.43142	193.140613
5.88E-05	3958.20535	3742.39338	3888.28744	3212.10246	3294.39824	3180.22696
0.00043526	2671.92978	2596.13227	2126.31387	1844.88328	1694.81727	2065.59619
0.01763474	318.463312	290.826471	337.462735	241.527677	259.470304	247.437171
0.03861286	88.085597	103.333948	81.6281542	66.8514106	62.195129	59.7262136
3.80E-13	8050.79771	7293.03304	7489.88088	5375.06907	5620.88479	5269.09311
3.40E-28	13539.208	13321.5569	13269.5524	19930.3464	19462.2164	20057.9241
0.01827025	258.610279	229.039162	214.025039	297.596602	282.793477	316.471366
0.00316586	13.5516303	11.7182827	14.9319794	37.7386996	30.1257656	34.1292649
9.54E-14	114.059555	88.4197696	122.442231	310.535585	240.034326	304.060724
0.00085033	3093.15962	3252.3561	2701.69281	2407.72903	2159.33714	2583.74049
9.83E-10	2098.24409	2168.9476	1886.40673	1275.56804	1384.81342	1490.82835
1.23E-29	5814.77871	5355.2552	4901.67111	2940.38382	2728.81129	2876.9419
3.25E-37	40578.0984	37697.7155	42799.0349	75615.4149	82533.908	73531.5013
2.16E-23	9667.95893	9130.67283	8802.89961	5547.58883	5822.04716	6019.93694
0.00182559	1828.34079	1535.09504	1537.99388	1264.78556	1378.98263	1309.32271
0.01114642	1960.46919	2157.22932	2114.36829	1820.08357	1864.88207	1704.13625
1.46E-05	3123.65079	3252.3561	2701.69281	4050.97984	3737.53854	4670.27964
0.07911615	1814.78916	1835.50919	1866.49743	1938.69091	2157.39354	2003.54299
3.06E-06	1588.92865	1518.05026	1341.88722	960.719466	846.436834	1158.06801
1.13E-07	4931.66413	4756.55748	4520.40791	5923.89758	5960.0426	6339.51096
0.01487364	1424.05049	1443.47937	1432.47456	1222.73387	1320.67469	1175.90831
0.02588485	7692.80881	6989.42299	6459.5743	7832.39753	7694.70362	9163.70764
0.03506	5785.41684	5469.24213	5960.84619	6201.00746	6485.7858	6347.26761
7.69E-74	1913.03848	2063.48306	2046.67665	656.653372	744.397951	676.379978
0.0190221	12806.2906	11955.8443	12206.3955	10698.3822	11273.8389	11292.9084
1.32E-10	11670.2123	12120.9655	10757.9934	16037.8691	15197.9629	17273.2864
9.74E-06	8759.9997	7681.86697	8090.14646	9820.68787	10677.1544	10293.076
1.08E-09	17817.006	17838.4222	19330.9406	23920.9443	26036.4359	23625.9837
9.87E-13	1507.61887	1558.5316	1518.08458	2270.79146	2100.0574	2182.72163
4.11E-33	8250.68426	8169.77365	8557.01968	12932.5132	13207.7187	13067.6301
6.61E-15	25561.7627	25604.4477	26537.1138	35052.7824	35135.3889	33751.516
4.77E-09	301.523775	396.291016	392.213326	645.870887	619.035894	587.17849
0.04637573	33.8790758	23.4365654	28.8684936	15.0954798	15.5487823	13.961972
0.03182695	29.3618657	11.7182827	22.8957018	34.5039539	59.2797324	34.9049301
1.11E-36	265.386094	260.998115	273.752956	721.348286	824.08546	693.44461

0.00942294	10.1637227	5.32649214	9.95465295	0	0	1.55133022
9.05E-22	11192.5173	10624.2212	10290.1248	16286.9445	15555.5849	16316.1156
8.22E-21	1697.3417	1662.93085	1477.2705	853.972859	691.920811	860.21261
1.54E-21	1486.16212	1587.29466	1756.00078	906.807038	883.365192	882.706898
1.23E-08	0	2.13059686	4.97732648	35.5822024	39.8437545	44.2129114
4.05E-05	31.6204707	27.6977591	31.8548895	8.62598847	6.80259224	4.65399067
0.00048981	23.7153531	25.5671623	18.9138406	2.15649712	7.77439113	3.87832556
1.18E-40	1680.40216	1733.24054	1872.47022	3708.09679	3913.43414	3446.28009
0.01425356	813.097819	719.076439	684.880123	549.906765	580.163938	663.969336
2.58E-14	1166.56951	1132.41223	1167.68079	652.340378	771.60832	670.950322
2.59E-07	991.527618	976.878659	759.54002	1441.61832	1261.39496	1529.6116
8.76E-06	250.705161	259.932817	247.870859	361.213267	363.452785	430.494137
1.13E-23	101.637227	104.399246	122.442231	4.31299423	3.88719557	7.75665112
0.00055363	319.592615	299.348858	251.85272	203.788978	164.234013	210.980911
3.43E-21	4939.56925	5018.6209	4714.52364	3225.04144	2985.36619	3232.19652
2.96E-09	4290.2203	4181.29633	4503.485	3342.57053	3347.84718	3156.18134
4.70E-42	2152.45062	2091.18082	2218.89214	965.03246	854.211225	1019.22396
0.00064072	1350.64582	1443.47937	1416.54712	1810.37933	1667.6069	1642.85871
0.00030896	5046.85299	4924.87464	4814.07017	5981.04476	5517.8741	6033.12324
1.23E-17	1368.71466	1331.62304	1438.44735	2126.30616	2080.62143	2151.69502
7.83E-08	421.229842	410.139895	386.240535	244.762423	243.921522	269.155794
1.11E-24	56659.3663	56762.2962	51807.9958	87562.409	84169.4456	91563.3882
2.58E-18	2295.87204	2487.47183	2762.41619	4314.07248	4343.94104	3990.02134
0.01227956	2513.82742	2140.18454	1972.01675	1731.66719	1844.4743	1918.99549
1.66E-71	11617.1351	11631.9935	11133.2839	23116.5709	22433.0056	23914.5311
9.15E-64	29321.2108	30590.0444	27695.8355	66956.0008	61614.9651	69512.7804
9.96E-93	18051.9009	17210.9614	17254.4	36426.4711	37421.0599	36894.5111
5.95E-25	693.391751	694.574576	749.585367	1291.74177	1277.91554	1311.6497
0.00332423	2180.68318	2105.0297	1815.7287	1726.27594	1426.60077	1718.09822
0.06876029	4025.96351	3694.45495	3292.9992	2829.32422	3199.16195	3516.08995
0.00088632	7466.9483	7801.18039	8940.27382	7138.00546	6202.02052	5800.42371
0.00014255	374.928439	313.197738	393.208792	244.762423	263.3575	223.391552
4.76E-57	1673.62634	1722.58756	1609.66738	595.193204	503.391826	618.205094
8.74E-07	1614.90261	1643.75548	1383.69676	2137.08864	2003.84931	2275.02577
7.76E-27	282.325632	284.43468	242.893532	67.9296592	41.7873523	65.9315345
0.00014144	3267.07221	3533.59489	3721.04927	2970.57478	2827.93477	2771.45145
3.30E-05	2.25860505	3.19589529	3.98186118	25.8779654	21.3795756	24.8212836
0.02697722	19320.1076	19607.8829	21332.8213	24622.8841	24536.9502	20440.327
9.34E-13	212.308875	200.276105	263.798303	88.4163818	93.2926936	83.7718321
1.61E-31	1565.2133	1617.12301	1587.76715	837.79913	789.1007	868.744926
4.33E-14	1750.41892	1638.42898	2033.7356	873.381333	1115.62513	857.109949
1.48E-45	10540.9098	11301.751	11102.4244	20018.7627	19041.4275	19328.7989
3.10E-69	12231.4757	12649.3535	12735.983	25586.8383	25523.3261	24053.3751

7.54E-26	2795.02375	3226.78894	3324.85409	5707.16962	5660.72854	5425.0018
3.77E-12	105.025135	127.835811	108.505717	296.518354	227.400941	291.650082
0.03699925	1019.76018	950.246198	903.882488	1027.57088	1127.28671	1166.60033
5.62E-79	4248.4361	4098.20306	3726.0266	1264.78556	1098.13275	1325.61168
3.08E-13	322.980523	334.503707	327.508082	160.659035	172.008404	144.273711
1.41E-10	1160.923	1224.02789	930.760051	618.914673	520.884206	681.033969
0.00241083	3124.78009	3385.51841	3243.22593	2757.08156	2911.50948	2683.02562
0.03662035	35.0083783	12.7835811	17.9183753	5.39124279	9.71798891	10.0836465
4.36E-20	1313.37884	1249.59506	1184.6037	443.160158	490.75844	651.558694
2.37E-10	3908.51604	3770.09114	3314.89943	2575.93581	2554.85929	2583.74049
5.69E-19	2089.20967	1956.95321	1838.6244	1179.60392	1202.11523	1210.81324
3.37E-12	249.575858	287.630576	232.938879	472.272869	459.660876	509.611979
0.04688777	3041.2117	2953.00724	2659.88327	3521.55979	2914.42488	3578.14316
4.00E-110	39279.4005	37852.1838	38484.6883	82658.5345	81943.0543	82392.6996
1.18E-65	1899.48685	1911.14538	1873.46569	677.140095	646.246263	750.843829
0.05726835	2223.59667	1972.93269	1898.35232	2272.94796	2388.68167	2218.40222
1.12E-167	3305.46849	3255.552	3258.15791	780.651957	811.452074	863.31527
5.66E-114	1049.12205	967.290973	1176.63998	116.450844	143.826236	145.825041
6.69E-25	1079.61322	886.328293	933.746447	364.448013	418.845322	293.201412
1.09E-23	6336.51647	6064.74396	5931.9777	3396.48296	3916.34953	3439.29911
7.08E-06	2520.60324	2865.65277	2918.70425	3532.34228	3869.70319	3461.01773
5.22E-14	3015.23775	3127.71619	3080.96509	4153.41345	4243.84576	4224.2722
1.87E-08	6063.22526	6208.55924	5114.70069	8951.61953	7604.32632	9403.38816
1.19E-16	373.799136	405.878701	419.090889	166.050278	185.613588	194.691943
0.00021825	548.841028	576.32645	563.433357	455.020892	407.183735	412.65384
1.49E-05	1964.9864	1816.33382	1861.5201	2275.10446	2370.2175	2366.55426
2.80E-12	1652.1696	1674.64913	1443.42468	2317.15615	2408.11765	2616.31842
0.01637248	988.13971	1030.14358	1075.10252	857.207604	918.349952	879.604237
2.23E-20	1540.36865	1548.94392	1682.33635	889.555061	945.560321	798.159401
0.00817397	2999.42751	2797.47367	3236.25768	3584.09821	3878.44938	3279.51209
1.40E-23	258.610279	260.998115	219.002365	562.845748	577.248541	658.53968
1.94E-19	95697.0961	94811.5602	80838.7502	49127.1608	43472.4516	54675.0824
1.53E-27	4095.98026	3864.9027	3407.47771	1980.7426	1831.84091	2036.12092
6.45E-12	16648.1778	16368.3104	14268.0041	10696.2257	9488.64437	11064.0872
0.00195253	153.585144	169.38245	114.478509	89.4946304	91.3490958	89.2014879
6.30E-81	248.446556	265.259309	219.99783	1018.94489	993.178467	999.83233
1.01E-16	3992.08443	3799.9195	3866.38721	2664.35219	2758.93705	2714.82789
2.18E-44	2998.29821	3019.05575	3222.32116	1616.29459	1552.93463	1549.00323
6.57E-05	160.360959	147.011183	166.242704	76.5556477	104.95428	86.0988275
0.00577585	3.38790758	0	0	9.70423703	8.74619002	15.5133022
6.00E-182	121.964673	93.7462617	85.6100154	1535.42595	1343.02607	1507.11731
4.74E-05	177.300497	185.361927	168.233635	121.842087	82.6029058	96.1824739
1.31E-19	79.0511768	90.5503664	97.555599	243.684174	313.891042	279.23944

8.56E-08	18.0688404	25.5671623	12.9410488	81.9468905	58.3079335	77.5665112
0.03003658	1003.94995	1071.69022	1048.22496	943.467489	813.395672	926.919809
2.83E-29	1739.12589	1771.59129	1542.97121	761.243482	622.923089	788.851419
2.30E-21	1299.82721	1483.96071	1529.03469	809.764668	764.805727	759.376145
6.32E-19	371.540531	384.572733	318.548895	153.111295	106.897878	116.349767
7.30E-105	4080.17003	4213.25529	4456.69813	11256.915	12021.1523	11098.9921
0.00242412	1241.10348	1215.50551	1465.32491	920.824269	1170.04587	904.425521
6.59E-09	224.731203	270.585801	244.884463	419.438689	401.352942	427.391477
3.70E-11	41.7841935	47.9384293	32.8503548	115.372596	129.249253	148.927702
1.41E-22	37.2669834	61.7873089	44.7959383	191.928243	241.006125	237.353524
5.30E-47	51.9479162	54.3302199	43.800473	302.987845	339.157813	368.440928
1.26E-190	569.168473	544.367497	546.510447	2607.20502	2696.74192	2782.31076
8.29E-30	781.477348	771.276062	816.281542	365.526261	370.255378	359.132947
3.83E-07	14.6809328	24.5018639	14.9319794	73.320902	58.3079335	63.6045392
6.63E-19	2091.46828	1961.21441	1711.20484	3414.81319	3214.71073	3744.1355
2.01E-56	3700.72438	3712.56502	3959.96095	7589.7916	8162.13889	8447.76874
7.21E-31	12053.0459	13789.2229	15286.3651	30759.1966	32892.4771	27276.2637
3.36E-08	839.071777	819.214492	900.896092	1227.04686	1314.8439	1137.90072
1.87E-09	1273.85325	1302.85998	1454.3748	941.310992	944.588522	873.398916
0.00155987	8702.40527	9014.5553	8166.79728	9882.14804	9812.25341	10310.1407
4.50E-08	8599.63874	8163.38186	7718.8379	6121.21707	6004.74535	6549.71621
7.28E-06	2936.18657	2872.04456	2547.39569	2085.33271	1840.5871	2237.79385
4.80E-12	2737.42932	2510.9084	1937.17546	1283.11578	1122.42772	1416.3645
2.86E-13	1794.46171	1720.45696	1501.16167	2843.34145	2465.45379	2861.4286
1.09E-11	2119.70084	2012.34873	1832.65161	1303.60251	1161.29968	1362.06794
2.63E-10	9008.44625	8928.26613	8239.46625	11309.7491	11552.7452	12170.9613
1.20E-14	540.93591	439.968251	506.691835	867.99009	872.675404	865.642265
2.18E-08	2811.96329	3082.97365	3178.52069	2208.25305	2255.54523	1900.37953
2.91E-06	6644.81606	6767.84092	6816.94634	5690.99589	5344.8939	5597.97512
0.0677158	735.175945	681.790994	707.775825	579.019476	662.766844	605.794453
3.77E-22	638.055927	614.677193	588.31999	306.222591	278.906282	293.201412
0.00123559	25.9739581	28.7630576	27.8730283	9.70423703	6.80259224	9.30798135
2.73E-05	0	0	0.9954653	18.3302255	24.2949723	19.3916278
0.031543	438.16938	400.552209	489.768925	529.420042	564.615156	512.714639
0.07788052	1360.80954	1456.26295	1558.89865	1315.46324	1348.85686	1233.30753
9.94E-15	223.6019	300.414157	228.957018	536.967782	511.166217	534.433262
6.41E-19	1543.75655	1594.75175	1564.87144	993.066923	849.352231	926.144144
1.88E-12	88.085597	108.66044	108.505717	22.6432197	29.1539667	23.2699534
0.03131091	6600.77327	6746.53495	7670.0601	6241.98091	6358.48015	5955.55673
1.87E-09	3514.38946	3685.93256	3429.37794	2761.39456	2637.46219	2638.81271
6.24E-05	2134.38177	2031.5241	2091.47259	1728.43244	1740.49181	1650.61536
2.01E-28	4060.97188	4736.31681	4517.42151	2463.79796	2210.84248	2403.01052
4.67E-13	584.978709	582.718241	596.283712	918.667772	945.560321	898.2202

4.45E-15	3199.31406	3536.79078	3650.37124	2281.57395	2327.45834	2114.4631
0.00100492	828.908054	840.52046	925.782725	659.888118	732.736364	666.296331
6.25E-07	1278.37046	1395.54094	1355.82373	971.501951	937.78593	1054.90455
0.00086073	747.598272	826.671581	940.714704	1106.28302	1220.57941	998.280999
1.07E-08	1727.83287	1853.61927	1850.56998	2538.19711	2584.98505	2313.03336
0.00708776	1331.44768	1335.88423	1404.60153	1123.535	1211.83322	1146.43304
7.86E-05	509.315439	510.277947	579.360802	349.352533	415.929925	278.463775
1.08E-17	1498.58445	1645.88607	1838.6244	2890.78439	3078.65889	2720.25755
5.89E-07	2586.10279	2747.40465	2643.95582	3449.31714	3241.9211	3397.41319
2.43E-52	9269.31514	9467.30714	9605.24464	16495.0465	16516.694	16189.6822
3.99E-11	295.877262	257.80222	282.712144	457.177389	525.7432	494.098677
0.00102111	8757.74109	9314.96946	10657.4515	11950.2288	12617.8368	10976.437
0.00927019	6206.64668	5934.77755	6489.43826	7272.78653	7718.02679	6627.28272
2.18E-13	802.934096	759.55778	715.739547	1192.54291	1166.15867	1177.45964
4.76E-14	582.720104	494.298471	538.546725	904.650541	964.0245	881.155568
9.84E-37	234.894925	226.908565	239.907136	31.2692082	34.0129612	42.6615812
0.01219725	1556.17888	1563.85809	1635.54948	1349.9672	1432.43157	1356.63828
2.70E-13	1835.11661	1642.69018	1990.93059	1189.30816	1086.47116	1116.95776
1.04E-62	8616.57828	8529.84452	8590.8655	16165.1024	15477.8409	16167.9636
2.78E-09	1570.85981	1510.59317	1455.37026	2141.40164	1969.83635	2120.66842
2.17E-79	662.900583	584.848837	651.034303	113.216099	87.4619002	93.0798135
6.08E-05	423.488447	418.662283	447.959383	535.889534	631.669279	608.897113
2.95E-71	3052.50473	2896.54643	2805.2212	6523.40378	6457.60363	6928.24078
0.00075484	2458.4916	2719.70689	2470.74486	2244.9135	1921.24641	2048.53156
2.81E-13	1953.69337	1991.04276	1965.04849	3010.46998	2714.2343	2793.17007
1.91E-17	902.312718	887.393591	946.687496	1485.82651	1459.64193	1419.46716
1.29E-13	3657.81088	3742.39338	3321.86769	5475.34618	5001.84889	5705.79257
9.30E-46	875.209458	935.33202	820.263403	290.048862	222.541946	275.361115
2.84E-10	1913.03848	1862.14165	1975.00315	1316.54149	1446.03675	1309.32271
5.25E-15	2947.47959	2917.8524	2809.20306	1964.56887	1827.95371	2037.67225
2.90E-06	118.576765	133.162304	115.473974	54.9906765	63.1669279	58.1748834
0.00042573	21.456748	17.0447749	25.8820977	4.31299423	4.85899446	3.87832556
3.10E-69	45804.5105	47903.2744	48027.2186	101913.897	94153.7074	94942.1854
5.89E-07	5169.94697	5403.19363	5763.74406	7018.31987	7173.81942	6664.51464
3.94E-07	123.093975	80.9626806	101.53746	31.2692082	39.8437545	41.110251
1.26E-14	795.028978	861.826429	965.601337	478.74236	481.040451	381.627235
7.31E-58	10765.641	11329.4488	11935.6289	5342.72161	4743.35039	4724.5762
2.71E-61	12240.5101	12670.6595	12055.0847	6568.69022	6409.98549	6616.42341
1.23E-05	70.0167566	54.3302199	69.6825707	30.1909596	15.5487823	8.53231624
8.43E-32	9073.9458	9447.06647	9723.70501	5490.44166	5897.84747	5557.64053
7.48E-39	4163.73841	3974.62844	3924.12419	2261.08723	2246.79904	2302.17405
6.20E-13	360.247506	372.85445	375.290416	204.867226	185.613588	180.729971
1.16E-09	668.547096	719.076439	716.735013	1045.9011	995.122065	1185.99196

5.09E-40	16783.6941	18415.8139	19861.5236	43195.7155	42347.1085	36733.9484
4.56E-11	2309.42367	2418.22743	2765.40259	3997.06741	4272.99973	3516.08995
0.07120216	0	2.13059686	0	4.31299423	9.71798891	3.10266045
8.09E-07	3058.15124	3265.13968	3447.29632	2524.17988	2457.6794	2589.94581
9.64E-06	3761.70671	3919.23292	3667.29415	4692.53773	4462.50051	4903.75484
5.31E-09	88.085597	69.2443979	80.6326889	21.5649712	24.2949723	21.7186231
9.23E-06	560.134053	638.113759	649.043373	890.63331	930.983338	785.748759
1.38E-17	1306.60302	1473.30773	1581.79435	2760.31631	2584.98505	2365.00293
1.06E-06	1998.86547	1945.23493	1704.23659	2564.07507	2394.51247	2660.53134
1.29E-09	5704.10706	6185.12268	6564.09816	8760.76954	9178.64053	8029.68524
2.40E-07	58.7237314	55.3955183	71.6735013	19.4084741	18.4641789	13.961972
0.00123915	1870.12498	1902.62299	2030.7492	2371.06858	2483.91797	2169.53532
3.14E-08	234.894925	252.475728	230.947949	382.778238	482.01225	385.505561
8.56E-37	553.358238	446.360042	545.514982	1235.67285	1299.29512	1369.82459
0.00030105	3750.41369	3971.43254	4008.73874	3097.80811	3469.32204	3080.16616
3.50E-05	1068.32019	1149.457	1002.43355	1461.0268	1295.40792	1507.11731
1.66E-72	1914.16778	1852.55397	1613.64924	5049.438	4835.67128	5333.47331
0.0043424	124.223278	125.705215	110.496648	91.6511275	59.2797324	72.9125206
1.20E-10	2391.86275	2198.77596	2150.20504	1599.04261	1484.90871	1623.46708
0.00391462	2722.74839	2495.99422	2787.30283	3116.13833	3573.30452	2982.43236
1.71E-24	41.7841935	51.1343246	49.7732648	217.806209	190.472583	224.942883
0.00131877	368.152624	457.013026	372.304021	297.596602	290.567869	290.098752
0.00522923	1334.83559	1507.39728	1484.23876	1677.75476	1707.45065	1706.46325
1.71E-51	5418.39352	5195.46044	5347.63957	10154.9449	9557.6421	9719.08386
8.25E-05	8990.37741	8912.28666	7888.067	6713.17553	5701.5441	7216.7882
1.77E-70	6267.62902	6072.20104	6109.17052	2998.60924	2809.47059	2794.7214
1.62E-20	77.9218743	64.9832042	74.6598972	273.875134	294.455064	215.634901
9.94E-18	747.598272	748.904795	797.367702	421.595186	434.394104	377.74891
2.72E-05	5226.41209	5066.55933	5328.72573	4468.26203	4282.71771	4038.11257
0.00154092	4762.26875	4517.93064	4764.2969	5241.36624	5786.0906	5382.34021
7.58E-05	3713.14671	3802.05009	3655.34856	3104.2776	2926.08646	3205.04824
2.14E-07	1779.78078	1993.17336	2093.46352	1409.27087	1458.67014	1216.2429
0.00053967	1767.35845	1860.01106	2091.47259	2593.18778	2695.77012	2151.69502
0.02042909	1201.57789	1294.33759	1466.32038	1575.32114	1720.08404	1445.0641
1.16E-28	277.808421	282.304084	234.92981	53.9124279	63.1669279	67.4828648
0.0380068	564.651263	529.453319	632.120463	513.246314	496.589233	408.775514
2.57E-17	3328.05454	3429.19564	3202.41186	4792.81484	4712.25282	4934.78144
0.00052645	232.63632	230.104461	202.079455	139.094064	155.487823	150.479032
7.25E-12	848.106197	887.393591	854.109224	499.229083	578.22034	461.520742
0.00407322	552.228935	650.89734	623.161275	512.168065	475.209658	462.296407
7.18E-08	187.464219	185.361927	223.979691	95.9641217	94.2644925	107.817451
2.56E-12	2595.13721	2286.13043	2412.01241	3587.33295	3803.62086	3383.45122
2.32E-07	609.823364	620.003686	704.789429	953.171726	930.011539	886.585223

0.00984994	62.1116389	50.0690262	45.7914036	87.3381333	95.2362913	76.015181
0.00092727	19.1981429	25.5671623	38.8231465	72.2426534	61.2233302	57.3992183
0.04341291	88.085597	71.3749947	88.5964113	105.668359	136.051845	107.041785
0.00279046	23.7153531	13.8488796	9.95465295	4.31299423	1.94359778	2.32699534
3.17E-08	31.6204707	29.828356	39.8186118	2.15649712	1.94359778	0.77566511
0.00036026	7.90511768	11.7182827	7.96372236	28.0344625	34.0129612	33.3535998
5.59E-32	112.930253	110.791037	93.5737378	405.421458	346.932204	391.710882
6.44E-06	7.90511768	21.3059686	17.9183753	0	0	0
1.62E-09	60.9823364	76.7014869	54.7505913	7.54773991	12.6333856	15.5133022
1.06E-12	2450.58648	2312.76289	2247.76064	1495.53075	1362.46205	1641.30738
2.76E-05	443.815893	418.662283	481.805203	269.56214	343.045009	287.771757
4.09E-20	380.574951	350.483183	472.846015	946.702235	1034.96582	838.493986
0.01522665	6.77581516	11.7182827	15.9274447	26.956214	34.9847601	23.2699534
6.95E-13	1216.25882	1324.16595	1377.72397	862.598847	853.239427	798.935066
0.01904191	0	0	0.9954653	6.46949135	5.83079335	5.42965579
0.08156779	443.815893	449.555937	395.199722	508.93332	464.51987	527.452276
9.58E-09	704.684776	795.777926	737.639784	503.542077	512.138016	456.866751
0.02404021	1909.65057	1984.65097	1872.47022	2153.26237	2087.42402	2282.00676
5.35E-46	2502.5344	2539.67145	2410.02148	5390.16455	4908.5562	5760.08912
0.000236	1530.20492	1513.78907	1650.48146	1903.10871	2046.60847	1849.96129
0.00022519	298.135867	344.091393	253.84365	189.771746	204.077767	197.018939
3.70E-30	7116.86452	7138.56477	7748.70186	3987.36317	4307.01269	3727.84653
1.53E-14	9052.48905	8606.54601	9729.6778	6086.71311	6536.31934	6075.00916
5.46E-38	1202.70719	1086.6044	1214.46766	507.855071	516.025211	467.726063
2.08E-09	748.727575	803.235015	935.737378	1365.06268	1355.65945	1195.29994
0.02010872	2846.97167	2883.76285	3079.96962	2559.76208	2745.33187	2353.36795
0.00106941	3951.42954	4018.30567	4427.82963	3550.6725	3549.00955	3201.94558
2.36E-12	1286.27558	1437.08758	1395.64234	2092.88045	1953.31577	2006.64565
0.02280665	2752.11026	3113.86731	2893.81761	2749.53382	2321.62755	2481.35269
4.83E-10	3317.89082	3147.95686	3316.89036	4452.0883	4493.59807	4156.78934
0.02527489	1596.83377	1478.63422	1688.30914	1797.44035	2055.35466	1728.18187
7.41E-34	658.383373	526.257424	645.061511	1690.69374	1727.85843	1421.79415
1.82E-28	414.454027	454.882429	456.918571	988.753928	1055.3736	905.976851
0.00020334	6.77581516	6.39179057	8.95918766	19.4084741	38.8719557	35.6805952
2.32E-05	753.244785	722.272335	585.333594	473.351117	447.02749	499.528332
9.43E-22	8422.33824	9140.26052	8777.01751	13739.0431	12815.112	13399.6148
6.24E-09	68.8874541	51.1343246	63.7097789	152.033047	141.882638	147.376371
2.82E-12	456.238221	492.167874	421.08182	255.544908	199.218773	241.23185
0.01942961	6224.71552	5848.48837	7503.8174	7780.6416	9349.67713	6974.78069
8.49E-53	2531.89626	2452.31698	2341.33437	4748.60665	4874.54324	5023.98293
0.02429809	11796.6942	12381.9636	13804.1173	11376.6005	11466.2551	10393.9125
9.74E-18	2443.81067	2648.33189	2696.71549	1704.71097	1511.14728	1619.58875
0.00051246	979.10529	1071.69022	1193.56289	836.720882	882.393393	802.813391

2.48E-18	8054.18562	8037.67665	8176.75194	11246.1325	11257.3184	10974.11
1.58E-05	1401.46444	1436.02228	1575.82156	1106.28302	1185.59465	1112.30377
4.63E-09	377.187044	428.249968	398.186118	640.479644	595.71272	635.269727
0.00037847	911.347139	890.589486	1030.30658	1167.74319	1307.06951	1158.06801
5.79E-06	368.152624	306.805947	307.598776	204.867226	203.105968	213.307906
6.16E-26	2596.26651	2521.56138	2723.59305	4283.88152	4592.72156	4205.65624
1.32E-07	3316.76152	3350.36356	3094.9016	2523.10163	2413.94845	2585.29182
2.55E-07	3663.4574	4053.46052	4491.53941	5962.71453	5849.25753	5227.98286
0.00363211	173.912589	232.235057	265.789234	136.937567	174.9238	121.003758
0.06026448	588.366616	588.044733	619.179414	528.341794	542.263781	480.136704
1.60E-26	161.490261	131.031707	152.30619	17.2519769	3.88719557	3.87832556
9.30E-13	3552.78575	3390.8449	4123.21725	2277.26096	2445.04601	2040.77491
6.44E-36	4092.59236	4395.42132	4160.04947	2320.3909	2383.82268	2445.6721
1.15E-14	190.852127	198.145508	164.251774	52.8341794	40.8155534	67.4828648
0.00015108	2238.27761	2573.761	2590.2007	3156.03353	3207.90814	2926.58447
0.00315864	870.692248	928.94023	1002.43355	1087.9528	1278.88734	1113.8551
0.03802856	3647.64716	3984.21612	3883.31012	3521.55979	3456.68866	3412.92649
0.00646602	540.93591	502.820858	584.338128	453.942643	396.493948	442.904779
2.53E-11	112.930253	106.529843	94.5692031	250.153666	221.570147	226.494213
0.01867682	2202.13993	2296.78341	2556.35488	2743.06433	2925.11466	2554.26521
0.00547613	1975.15012	2112.48678	2124.32294	1841.64854	1608.32717	1803.42139
2.83E-10	1522.29981	1586.22936	1641.52227	1155.88245	1126.31492	1065.76386
4.87E-33	16.9395379	27.6977591	26.877563	238.292931	203.105968	186.159627
2.98E-20	1852.05614	1823.79091	1827.67428	1156.9607	1181.70745	1160.39501
6.98E-26	3167.69359	3009.46806	3325.84955	1564.53866	1767.70218	1397.74853
8.12E-10	2902.30749	2807.06136	2638.9785	1896.63921	1634.56574	2034.56959
0.00564919	4654.98501	4823.67129	4745.38306	4169.58718	4321.58967	4086.97948
1.79E-07	2647.08512	3118.1285	3175.53429	4112.44	4510.11865	3924.86547
0.04488997	3619.4146	4051.32992	3777.7908	3445.00415	3369.22676	3488.16601
0.00348146	10.1637227	6.39179057	1.99093059	0	0	0
4.50E-08	840.20108	782.994345	914.832607	1170.97793	1192.39724	1241.06418
4.94E-12	3920.93837	4040.67694	4010.72968	3055.75642	2837.65276	2883.92289
8.90E-05	653.866163	641.309654	653.025234	834.564384	841.57784	818.326693
6.63E-15	6077.9062	6350.24393	7162.3728	3944.23323	4315.75888	3557.2002
0.00723437	398.643792	387.768628	374.294951	245.840671	338.186014	291.650082
7.06E-288	1492.93794	1667.19204	1507.13446	8485.81616	8722.86685	8453.19839
2.23E-10	954.260635	943.854408	980.533316	1393.09714	1351.77226	1327.93867
0.03650553	65.4995465	50.0690262	53.755126	45.2864395	29.1539667	27.1482789
3.51E-72	2060.97711	2244.58379	2246.76517	5038.65551	5131.09815	4903.75484
1.49E-45	1801.23753	1724.71816	1902.33418	3848.26911	4148.60947	3662.69066
0.01728656	1.12930253	1.06529843	0	7.54773991	7.77439113	7.75665112
0.00011088	851.494105	904.438366	851.122828	660.966367	569.47415	707.406582
2.56E-06	67.7581516	71.3749947	59.7279177	25.8779654	24.2949723	17.0646325

5.43E-08	1111.23369	997.119329	1190.57649	1713.33696	1669.5505	1462.12874
1.65E-53	2577.06836	2919.98299	2641.96489	6822.07863	5976.56318	6851.44994
4.85E-11	1005.07925	915.09135	878.995856	613.52343	523.799602	594.159476
2.59E-39	1681.53146	1724.71816	1747.04159	865.833593	886.280589	847.801968
0.00024679	426.876355	432.511162	457.914036	332.100556	329.439824	314.14437
2.21E-21	635.797322	648.766743	665.966283	1265.86381	1239.04359	1105.32278
2.78E-11	1424.05049	1475.43832	1417.54258	1023.25788	1018.44524	980.440702
8.74E-14	1220.77603	1111.10626	1179.62638	752.617494	723.018375	765.581466
9.96E-08	958.777845	873.544712	991.483434	1361.82793	1404.2494	1237.18585
0.02765626	24.8446556	17.0447749	20.9047712	7.54773991	12.6333856	3.87832556
7.13E-06	197.627942	169.38245	176.197357	97.0423703	93.2926936	110.920111
0.02025132	120.83537	145.945885	190.133871	111.059602	105.926079	99.2851344
5.39E-07	3990.95513	4336.8299	4814.07017	6383.23147	6635.44283	5487.055
1.04E-11	189.722824	213.059686	192.124802	94.8858732	68.0259224	72.1368554
0.00096072	980.234593	934.266722	976.551455	1132.16099	1214.74861	1165.82466
3.83E-14	2502.5344	2508.7778	2845.03981	4001.3804	4295.3511	3820.92634
4.18E-18	3276.10663	3383.38781	3379.60468	4777.71936	4995.0463	4745.51916
0.00903597	76.7925718	94.8115602	120.451301	167.128527	145.769834	134.190064
1.19E-19	3961.59326	3583.66391	3754.89509	5728.73459	6154.40238	5682.52261
0.00027865	20.3274455	13.8488796	16.92291	0	3.88719557	0
2.92E-24	20404.238	20690.2261	22003.7649	32418.6212	33314.2378	31132.8706
3.23E-05	606.435457	569.934659	531.578468	388.169481	342.07321	440.577784
0.00176598	392.997279	383.507434	300.630519	227.510446	256.554907	276.13678
1.27E-07	266.515396	310.001843	300.630519	435.612418	481.040451	466.174732
4.52E-37	2837.93725	3086.16955	3026.2145	5211.17528	5415.83522	5208.59123
0.00290644	1480.51561	1260.24804	1295.10035	1591.49487	1791.99716	1577.70284
7.70E-05	180.688404	156.598869	125.428627	69.0079078	97.1798891	78.3421763
5.34E-07	12024.8133	11816.2902	9623.16301	7733.19866	6499.39099	8368.6509
1.29E-08	27885.8673	27048.9924	24034.5141	18953.4532	16788.7976	20034.6542
0.03803412	5.64651263	3.19589529	0	11.8607341	9.71798891	13.1863069
6.01E-42	14561.2268	14158.8814	14044.0244	8547.27633	8095.08476	8520.68126
2.42E-10	9489.52913	9561.0534	10005.4217	7497.06223	7397.33316	6785.5184
3.27E-76	1832.858	1818.46442	1614.64471	522.950551	464.51987	539.087253
3.44E-13	199.886547	216.255581	143.347003	56.0689251	35.956559	61.2775439
0.01360304	0	1.06529843	0	3.23474568	8.74619002	7.75665112
1.25E-15	16615.4281	16174.426	16919.9236	22214.0768	23160.883	22155.3226
0.00174853	70.0167566	73.5055916	43.800473	106.746607	117.587666	106.26612
1.11E-157	2245.05342	2329.80766	2456.80835	8644.3187	8670.38971	7966.0807
0.02852145	535.289397	500.690262	600.265573	608.132187	750.228744	639.148053
2.78E-05	3422.91596	3218.26655	3171.55243	2501.53666	2512.10013	2794.7214
2.02E-13	2612.07674	2694.13973	2952.55007	1668.05052	1665.6633	1247.2695
1.66E-22	108.413043	137.423497	121.446766	14.0172313	17.49238	13.961972
0.00066154	14248.41	15382.9093	16240.0208	13652.7833	11340.8931	12154.6723

0.04382158	13125.8833	14662.7676	14367.5506	13343.3259	12118.3322	12345.4859
2.59E-26	25037.7663	26313.9365	23141.5817	47169.0615	41003.1106	48271.9669
8.30E-23	5603.59914	5711.06488	6120.12064	9231.96416	9186.41492	8705.28956
0.05356571	12441.5259	12711.1409	13902.6683	11022.935	12717.9321	10860.8629
3.96E-31	83.5683869	69.2443979	93.5737378	383.856487	482.01225	327.330677
0.02933273	1285.14627	1340.14542	1576.81703	1609.8251	1713.28145	1595.54314
0.01231	1833.9873	1760.9383	1273.20011	1164.50844	1036.90942	1465.2314
2.61E-12	5410.4884	5257.24775	4609.00432	2986.74851	3224.42872	3557.2002
5.08E-53	1322.41326	1385.95326	1457.36119	527.263545	568.502351	540.638583
5.46E-14	4750.97573	4688.37839	4761.31051	6560.06423	6805.50764	6264.27145
1.57E-05	2828.90283	2674.96435	2613.0964	1882.62198	2177.80132	2197.45926
9.30E-113	8191.96053	8345.54789	8102.09204	18857.489	18539.9792	18283.978
1.77E-10	2776.95491	2649.39719	2499.61336	3612.13267	3533.46077	3623.13174
0.01622073	27946.8496	27320.6435	28720.1692	25611.638	25558.3108	25295.215
1.77E-05	1012.98437	1199.52603	1314.01419	1624.92058	1730.77383	1534.26559
2.59E-19	5104.44742	5130.47723	5295.87537	3484.89934	3471.26564	3137.56538
8.24E-72	1280.62906	1318.83945	1267.22732	429.142926	375.114372	419.634826
1.37E-06	143.421421	141.684691	136.378745	228.588694	253.639511	235.026529
1.02E-17	59.8530339	67.113801	67.6916401	204.867226	193.387979	213.307906
0.01195823	543.194515	597.632419	575.378941	703.01806	665.682241	687.239289
0.00279078	7119.12313	7017.12075	7244.00096	5617.67499	6657.7942	5625.89906
1.41E-15	1524.55841	1585.16406	1679.34995	1062.07483	991.234869	989.748683
0.00590338	26169.3274	25194.3078	20662.8731	19030.0088	17107.5477	21713.1935
4.96E-06	107.28374	98.0074555	57.7369871	35.5822024	14.5769834	29.4752743
0.00376674	1660.07471	1616.05772	1880.43394	1982.8991	2109.77539	2067.92319
1.09E-112	686.615936	620.003686	628.138601	12.9389827	19.4359778	12.4106418
1.53E-07	2208.91574	2084.78903	2308.48402	1676.67651	1708.42245	1568.39486
0.00211422	16.9395379	11.7182827	18.9138406	40.9734452	50.5335423	31.0266045
0.00060355	535.289397	449.555937	412.122632	326.709313	340.129612	341.292649
2.11E-20	3473.73457	3587.92511	3780.77719	5588.56228	5802.61118	5355.9676
7.57E-29	575.944288	601.893612	631.124997	251.231914	260.442103	235.026529
2.71E-06	31674.6773	32368.0275	29920.7004	26045.0939	23432.9867	25615.5647
0.02513297	72.2753617	67.113801	77.646293	39.8951967	56.3643357	40.3345858
0.04263134	1782.03939	2081.59313	2151.2005	1776.95362	1812.40493	1621.91575
5.71E-12	4143.41097	4285.69558	4457.69359	5879.68939	5809.41377	5661.57965
5.39E-13	254.093068	189.62312	201.08399	88.4163818	64.1387268	76.7908461
8.52E-26	3186.89173	3267.27028	3123.7701	1870.76125	1717.16864	1957.00308
1.80E-25	629.021507	697.770471	748.589902	1355.35844	1379.95443	1314.75237
0.01959306	7771.85999	8382.83334	8754.12181	9257.84213	10218.4653	9003.14496
0.00968464	3051.37543	3180.98111	3250.19419	2839.02846	2736.58568	2805.58071
0.00021776	711.460592	588.044733	550.492308	458.255637	396.493948	456.866751
0.0042438	7420.6469	7202.48268	7293.77422	6605.35067	6265.18745	6549.71621
0.0002045	1359.68024	1248.52976	1204.51301	1059.91833	873.647203	989.748683

0.00461669	142.292118	116.117529	94.5692031	77.6338962	66.0823246	72.9125206
0.00498032	789.382466	818.149193	852.118293	671.748852	720.102978	615.102434
7.97E-18	805.192701	857.565235	840.172709	1472.88753	1356.63125	1654.49368
1.20E-06	1020.88948	1003.51112	1024.33379	684.687835	803.677683	719.817224
1.37E-10	4732.90689	4620.19929	3823.5822	6442.53514	6576.1631	7570.4915
1.53E-06	168.266076	152.337675	133.39235	71.1644049	76.7721124	78.3421763
0.01011968	65.4995465	53.2649214	64.7052442	123.998584	85.5183024	89.2014879
0.06768417	516.091254	414.401089	472.846015	361.213267	451.886484	329.657673
0.06909044	23.7153531	31.9589529	24.8866324	53.9124279	43.7309501	35.6805952
0.00520838	62.1116389	56.4608167	39.8186118	25.8779654	30.1257656	21.7186231
0.00016997	60.9823364	57.5261152	70.678036	34.5039539	23.3231734	20.942958
1.80E-05	12.4223278	17.0447749	4.97732648	58.2254222	43.7309501	39.5589207
0.00118853	18.0688404	3.19589529	8.95918766	31.2692082	45.6745479	30.2509394
3.65E-06	107.28374	102.268649	124.433162	189.771746	203.105968	200.121599
0.0102635	12376.0264	12726.055	13142.1328	11452.0779	11223.3054	11663.6763
4.37E-07	101.637227	121.444021	98.5510643	205.945475	237.118929	184.608297
0.00217228	120.83537	106.529843	77.646293	173.598018	142.854437	172.197655
0.00033437	685.486633	702.031665	638.093254	904.650541	797.84689	916.060498
9.27E-09	132.128396	119.313424	121.446766	48.5211851	51.5053412	50.4182323
1.66E-05	60.9823364	51.1343246	53.755126	23.7214683	17.49238	14.7376371
0.00404645	2114.05433	1993.17336	2269.66087	1882.62198	1779.36377	1713.44423
0.00228579	20.3274455	11.7182827	19.9093059	37.7386996	36.9283579	53.5208927
6.47E-06	10.1637227	3.19589529	12.9410488	54.9906765	49.5617435	31.0266045
0.00018979	64.370244	72.4402932	53.755126	20.4867226	34.9847601	12.4106418
0.02057425	22.5860505	37.285445	26.877563	54.9906765	55.3925368	45.7642416
0.00092116	788.253163	755.296586	789.403979	548.828516	646.246263	632.167067
1.60E-14	241.670741	202.406701	205.065851	417.282192	439.253099	449.885765
0.04398801	79.0511768	63.9179057	62.7143136	47.4429366	45.6745479	42.6615812
0.00037983	12.4223278	8.52238743	10.9501183	35.5822024	34.0129612	32.5779347
3.40E-05	282.325632	266.324607	308.594242	176.832764	205.049566	150.479032
7.45E-14	837.942474	813.888	774.472	1248.61183	1221.55121	1348.88163
3.66E-10	522.86707	533.714513	443.977522	307.300839	218.654751	255.193822
1.45E-206	177.300497	183.23133	162.260843	1663.73753	1782.27917	1912.0145
1.09E-34	825.520147	818.149193	811.304216	337.491799	333.32702	369.992259
0.00784384	93.7321097	113.986932	102.532925	78.7121448	58.3079335	58.9505485
5.66E-12	326.36843	348.352586	385.245069	634.010153	638.471872	576.319178
0.04965286	162.619564	110.791037	114.478509	173.598018	176.867398	167.543664
0.04207506	0	1.06529843	3.98186118	8.62598847	5.83079335	12.4106418
0.00260673	18.0688404	9.58768586	19.9093059	38.8169481	41.7873523	38.0075905
2.56E-43	391.867977	453.817131	506.691835	1231.35985	1225.4384	1290.70675
8.68E-10	40.6548909	50.0690262	33.84582	3.23474568	1.94359778	1.55133022
4.97E-05	53.0772187	41.5466387	32.8503548	14.0172313	11.6615867	11.6349767
2.01E-09	2.25860505	3.19589529	2.98639589	42.0516938	39.8437545	44.2129114

0.05608974	45.172101	19.1753717	47.7823342	72.2426534	61.2233302	55.072223
6.26E-07	124.223278	145.945885	94.5692031	46.364688	40.8155534	56.6235532
2.02E-09	134.387001	156.598869	131.401419	37.7386996	49.5617435	62.8288741
0.02068029	15.8102354	15.9794764	31.8548895	9.70423703	5.83079335	7.75665112
2.83E-08	55.3358238	44.742534	48.7777995	6.46949135	6.80259224	12.4106418
0.00061589	2671.92978	2573.761	2161.15516	1827.63131	1590.83479	2114.4631
1.37E-05	9.03442021	5.32649214	1.99093059	25.8779654	36.9283579	34.9049301
4.07E-37	4839.06132	5166.69738	5200.3107	2868.14117	2914.42488	2746.63016
0.03298419	202.145152	209.86379	245.879928	184.380504	147.713431	172.197655
1.63E-29	1097.68206	1200.59133	1255.28174	553.141511	512.138016	583.300164
0.00038386	191.981429	191.753717	158.278982	253.388411	270.160092	272.258454
3.86E-07	10927.1312	10654.0496	11891.8284	8452.39045	9131.02238	8254.62812
3.07E-30	2086.95107	2004.89164	1984.9578	1158.03895	1148.66629	1094.46347
6.22E-39	3224.15871	3603.90458	3435.35073	1789.89261	1604.43997	1699.48226
8.31E-14	589.495919	582.718241	564.428823	313.770331	274.047287	341.292649
1.82E-06	662.900583	602.958911	581.351733	801.138679	928.067941	885.033893
6.73E-07	6507.04116	6795.53868	6201.74879	4906.03094	4453.75432	5298.56838
3.94E-06	1577.63563	1685.30211	1734.10054	1329.48047	1264.31036	1283.72576
0.00341093	206.662362	215.190283	188.142941	265.249145	269.188293	291.650082
0.03221693	142.292118	167.251853	174.206427	119.68559	124.390258	118.676762
0.00030001	4456.22777	4292.08737	4002.76595	3579.78522	3047.56132	3571.16218
8.74E-05	1095.42345	1051.44955	1196.54929	1491.21776	1495.59849	1334.14399
0.00243839	4394.11613	4389.02953	3943.03804	3667.12335	3351.73438	3710.7819
8.84E-13	516.091254	500.690262	448.954848	268.483891	179.782795	231.923869
1.47E-10	2740.81723	2750.60054	3085.94242	2031.42028	2053.41106	1855.39095
4.45E-05	260.868884	268.455204	282.712144	163.893781	191.444382	174.52465
8.88E-06	125.35258	137.423497	158.278982	232.901689	302.229455	220.288892
2.21E-11	324.109825	284.43468	312.576103	132.624573	160.346817	158.235683
1.88E-08	162.619564	173.643644	214.025039	84.1033876	91.3490958	72.1368554
0.02958713	16.9395379	13.8488796	14.9319794	29.1127111	34.9847601	27.923944
1.05E-19	79.0511768	68.1790994	89.5918766	269.56214	240.034326	241.23185
0.08133524	487.858691	374.985047	525.605676	401.108464	376.086171	372.319254
0.00020036	63.2409415	56.4608167	55.7460565	99.1988674	111.756873	111.695776
4.10E-13	1195.93138	1280.48871	1311.02779	1817.92707	1974.69535	1859.26927
3.85E-13	20.3274455	9.58768586	16.92291	91.6511275	84.5465035	88.4258228
5.97E-06	56.4651263	40.4813403	52.7596607	101.355365	117.587666	109.368781
1.01E-06	494.634506	453.817131	327.508082	214.571463	203.105968	267.604464
4.62E-06	135.516303	129.966408	139.365141	67.9296592	68.0259224	72.1368554
0.03983074	15.8102354	7.457089	12.9410488	21.5649712	26.2385701	29.4752743
1.46E-58	1106.71648	989.66224	1099.98915	2497.22366	2557.77468	2469.71772
0.06029123	101.637227	90.5503664	102.532925	154.189544	112.728671	130.311739
0.0017043	188.593522	178.970136	155.292586	266.327394	209.908561	321.901022
0.00106556	54.2065213	44.742534	81.6281542	29.1127111	29.1539667	17.0646325

3.80E-05	474.307061	448.490639	510.673697	298.674851	369.283579	318.798361
0.00650411	10.1637227	13.8488796	11.9455835	0	3.88719557	1.55133022
0.04255871	6.77581516	7.457089	7.96372236	17.2519769	14.5769834	22.4942883
0.00401361	19.1981429	26.6324607	15.9274447	51.7559308	35.956559	50.4182323
1.07E-16	178.429799	224.777968	202.079455	51.7559308	61.2233302	69.8098601
0.04748808	0	1.06529843	0	4.31299423	3.88719557	5.42965579
1.07E-10	261.998186	281.238785	280.721213	146.641804	141.882638	125.657748
0.00022442	0	1.06529843	0	18.3302255	9.71798891	17.0646325
0.00047761	39.5255884	26.6324607	22.8957018	5.39124279	8.74619002	9.30798135
0.00010811	66.628849	43.6772356	46.7868689	19.4084741	10.6897878	21.7186231
1.45E-06	19.1981429	10.6529843	9.95465295	45.2864395	49.5617435	69.8098601
0.08400807	23.7153531	19.1753717	15.9274447	34.5039539	28.1821678	37.2319254
0.0810324	0	0	0	3.23474568	4.85899446	0.77566511
0.00090548	158.102354	132.097005	125.428627	208.101972	225.457343	193.916278
0.01658333	160.360959	161.925361	156.288051	186.537001	210.880359	246.661506
0.00524914	10.1637227	24.5018639	16.92291	5.39124279	4.85899446	0.77566511
0.00027413	95.9907147	96.942157	74.6598972	155.267792	147.713431	146.600706
0.0563844	50.8186137	54.3302199	38.8231465	24.7997169	40.8155534	13.961972
0.00864633	0	3.19589529	3.98186118	14.0172313	9.71798891	16.2889674
0.00485396	4.5172101	6.39179057	5.97279177	23.7214683	17.49238	18.6159627
0.05545062	11.2930253	4.26119372	4.97732648	16.1737284	17.49238	17.0646325
0.00084015	80.1804794	73.5055916	61.7188483	148.798301	128.277454	103.16346
0.04801337	0	0	0	1.07824856	1.94359778	7.75665112
1.23E-06	125.35258	119.313424	124.433162	50.6776823	58.3079335	62.8288741
0.04033655	0	0	1.99093059	4.31299423	5.83079335	9.30798135
6.88E-06	48.5600086	42.6119372	60.723383	107.824856	127.305655	102.387795
6.05E-18	109.542345	111.856335	84.6145501	323.474568	301.257656	262.174808
8.03E-06	125.35258	129.966408	155.292586	250.153666	238.090728	214.083571
0.06927563	19.1981429	9.58768586	6.96825707	32.3474568	21.3795756	20.942958
0.02773778	44.0427985	38.3507434	37.8276812	24.7997169	18.4641789	22.4942883
0.02723584	14.6809328	21.3059686	12.9410488	24.7997169	38.8719557	36.4562603
0.0684708	0	3.19589529	0	10.7824856	5.83079335	3.87832556
4.88E-06	168.266076	121.444021	131.401419	73.320902	70.9413191	50.4182323
0.00910108	134.387001	109.725738	159.274447	88.4163818	88.4336991	90.7528181
0.07244398	211.179572	180.035434	220.993296	163.893781	186.585387	110.920111
1.18E-12	1092.03554	1112.17156	1016.37007	615.679927	598.628117	718.265894
1.58E-07	594.013129	500.690262	493.750787	749.382748	798.818689	906.752516
4.60E-09	191.981429	166.186555	184.16108	312.692082	319.721835	330.433338
0.00383685	39.5255884	46.8731309	51.7641954	28.0344625	13.6051845	20.1672929
0.03920896	45.172101	50.0690262	34.8412853	22.6432197	27.210369	24.8212836
0.08644439	28.2325632	28.7630576	26.877563	51.7559308	36.9283579	43.4372463
0.0676365	1.12930253	1.06529843	6.96825707	7.54773991	14.5769834	10.8593116
0.01392651	6.77581516	10.6529843	8.95918766	1.07824856	1.94359778	0.77566511

0.06936116	21.456748	28.7630576	23.8911671	38.8169481	34.9847601	48.8669021
8.04E-08	392.997279	311.067141	310.585172	628.61891	493.673837	628.288741
0.00021117	283.454934	267.389906	245.879928	391.404227	383.860562	349.824966
1.83E-08	41.7841935	50.0690262	43.800473	115.372596	121.474861	111.695776
0.00026407	14.6809328	27.6977591	17.9183753	2.15649712	3.88719557	3.10266045
0.02160339	147.938631	95.8768586	147.328864	81.9468905	103.982481	70.5855252
9.42E-07	0	1.06529843	1.99093059	33.4257053	28.1821678	30.2509394
5.92E-15	41.7841935	59.656712	39.8186118	149.87655	192.41618	169.094994
0.05333633	35.0083783	49.0037277	37.8276812	63.616665	96.2080902	44.2129114
0.0279914	276.679119	262.063413	242.893532	190.849995	170.064806	233.475199
0.07263667	14.6809328	18.1100733	11.9455835	22.6432197	28.1821678	31.8022696
8.48E-07	479.953574	361.136167	343.435527	240.449429	230.316337	208.653915
1.29E-14	413.324725	384.572733	377.281347	210.258469	175.895599	171.42199
1.42E-10	583.849406	644.505549	636.102324	364.448013	409.127333	363.011273
4.14E-26	440.427985	400.552209	478.818807	161.737284	166.17761	150.479032
4.64E-07	1398.07653	1409.38982	1592.74447	1040.50986	1121.45592	939.330451
1.78E-13	261.998186	317.458932	323.526221	692.235575	620.007693	534.433262
3.21E-34	409.936817	401.617508	413.118098	135.859318	124.390258	117.125432
0.00161896	396.385187	344.091393	370.31309	497.072586	497.561032	455.315421
4.61E-05	63.2409415	57.5261152	54.7505913	118.607341	107.869677	111.695776
2.20E-77	8511.55314	8446.75124	8454.48675	4022.94537	4223.43798	4039.6639
0.00037961	134.387001	125.705215	140.360607	69.0079078	94.2644925	64.3802043
2.15E-40	7351.75945	7240.83342	7072.78092	4237.51684	3905.65974	4113.35209
2.97E-19	2225.85528	2097.57261	2166.13248	1327.32398	1385.78522	1417.14016
3.98E-13	1358.55094	1405.12863	1412.56525	975.814946	899.885773	902.874191
9.86E-12	2746.46374	2782.5595	2552.37302	1979.66435	1732.71742	1799.54306
4.63E-05	877.468063	902.307769	986.506108	1245.37709	1281.80274	1118.50909
4.86E-19	3207.21917	3137.30387	3302.95385	2167.2796	1922.21821	2046.20457
0.00013701	1053.63926	1173.95887	935.737378	816.234159	605.430709	785.748759
3.25E-51	306.040985	289.761173	295.653193	30.1909596	43.7309501	32.5779347
4.63E-08	579.332196	627.460775	680.898262	952.093477	962.080902	871.847586
3.01E-06	355.730296	393.09512	377.281347	525.107048	527.686798	567.786862
1.78E-32	3843.0165	4433.77206	4590.09048	2068.08074	1880.43085	1651.39102
3.15E-08	10709.1759	11172.8499	10247.3198	8328.39187	6859.92837	7957.54839
2.85E-17	2736.30002	3051.0147	2904.76773	1904.18695	1895.97964	1911.23884
3.43E-05	186.334917	164.055958	176.197357	104.59011	111.756873	88.4258228
0.00705329	1347.25791	1381.69206	1259.2636	1209.79488	973.742489	1092.13648
5.83E-17	233.765623	255.671623	282.712144	103.511862	82.6029058	92.3041484
6.11E-25	10973.4326	11836.5308	11702.69	7306.21223	6295.31322	6572.98616
5.60E-70	7806.86836	8236.88745	8106.0739	16152.1634	15577.9362	15801.074
4.15E-14	2140.02829	2139.11925	1986.94873	2941.46207	3004.80217	3146.87336
6.76E-21	294.747959	258.867518	361.353902	50.6776823	93.2926936	73.6881857
3.58E-86	1892.71103	1953.75732	1864.5065	677.140095	679.287425	669.398992

0.00092715	271.032606	307.871246	214.025039	195.162989	133.136448	170.646325
4.65E-22	391.867977	372.85445	372.304021	159.580787	153.544225	142.722381
0.0199115	134.387001	176.839539	171.220031	132.624573	100.095286	109.368781
2.93E-05	11.2930253	12.7835811	11.9455835	0	0	0
5.97E-07	241.670741	227.973864	229.952483	91.6511275	138.967241	131.863069
3.34E-05	49.6893112	61.7873089	70.678036	20.4867226	26.2385701	8.53231624
7.82E-12	932.803887	1081.27791	1102.97555	1597.96436	1553.90643	1628.89674
5.47E-12	2233.7604	2340.46065	2433.91265	3203.47647	3351.73438	3199.61859
7.63E-103	2723.87769	2809.19196	2549.38662	7791.42409	7979.4407	8799.14503
4.47E-27	1521.1705	1494.6137	1204.51301	594.114956	445.083892	558.478881
1.14E-05	159.231656	153.402974	155.292586	228.588694	258.498505	267.604464
0.01684616	10.1637227	13.8488796	14.9319794	36.660451	32.0693634	21.7186231
1.33E-21	12381.6729	12327.6334	11156.1796	18865.0368	18222.201	20067.2321
0.00131919	161.490261	182.166031	143.347003	123.998584	88.4336991	86.8744926
3.51E-05	582.720104	602.958911	691.84838	891.711558	844.493237	814.448368
1.17E-05	421.229842	433.576461	470.855085	310.535585	315.83464	288.547422
1.86E-15	5299.81676	5363.77759	5908.08653	8245.36673	8996.91414	7987.02366
1.65E-24	2527.37905	2494.92892	2506.58161	3977.65893	3965.91128	4373.1999
0.00168839	1234.32766	1380.62676	1518.08458	1133.23924	1138.9483	937.003456
1.34E-12	54.2065213	57.5261152	58.7324524	0	5.83079335	1.55133022
3.97E-05	1468.09328	1594.75175	1401.61514	2079.94147	1748.26621	2182.72163
2.78E-07	2018.06361	2095.44201	2133.28213	2688.07366	2710.34711	2583.74049
0.00011033	1331.44768	1334.81893	1178.63091	1608.74685	1560.70902	1705.68758
3.12E-05	894.407601	823.475685	797.367702	1085.7963	1054.4018	1130.91973
0.08507186	3559.56156	3316.27401	3236.25768	3694.07956	3558.72754	3850.40162
0.00101923	1742.5138	1653.34316	1582.78982	1283.11578	1410.08019	1403.17819
0.00017422	2334.26832	2077.33194	2248.7561	1645.4073	1914.44382	1697.93093
0.04204222	20.3274455	15.9794764	17.9183753	43.1299423	29.1539667	30.2509394
8.41E-54	2081.30456	2319.15468	2319.43414	917.589523	805.621281	771.011122
1.60E-16	6230.36204	6321.48088	5688.0887	3659.57561	2793.92181	3577.3675
1.83E-05	4919.2418	4676.6601	4637.87281	3949.62447	3778.35409	3997.00232
8.01E-14	155.843749	145.945885	139.365141	33.4257053	50.5335423	45.7642416
1.54E-07	236.024228	226.908565	244.884463	364.448013	368.31178	407.999849
2.30E-19	9.03442021	21.3059686	20.9047712	125.076833	121.474861	122.555088
3.97E-20	2398.63857	2368.15841	2770.37992	4753.9979	4963.94874	4124.98707
0.03018803	29.3618657	29.828356	23.8911671	10.7824856	17.49238	10.8593116
3.40E-15	443.815893	393.09512	455.923105	197.319486	224.485544	202.448594
0.00015798	38.3962859	51.1343246	38.8231465	11.8607341	19.4359778	7.75665112
0.06607341	3.38790758	0	0.9954653	7.54773991	8.74619002	5.42965579
3.96E-21	1439.86072	1418.97751	1462.33852	2209.3313	2382.85088	2297.52006
0.00072841	1521.1705	1414.71631	1337.90536	1678.83301	1806.57414	1729.7332
0.02727094	247.317253	283.369382	224.975157	309.457336	332.355221	310.266045
9.09E-08	531.90149	595.501822	642.075116	860.44235	932.926936	840.045317

5.40E-10	77.9218743	77.7667853	97.555599	15.0954798	19.4359778	26.3726138
8.42E-13	1521.1705	1560.6622	1807.76498	2523.10163	2747.27547	2446.44776
1.26E-24	711.460592	672.203309	826.236195	1486.90476	1454.78294	1441.96144
0.0012201	901.183416	977.943958	1019.35646	1210.87313	1213.77682	1137.12505
4.11E-18	5096.5423	4984.53135	4718.5055	3316.69257	2832.79377	3082.49316
5.71E-05	1507.61887	1420.04281	1249.30895	1828.70956	1714.25324	1925.97647
0.00063741	3539.23412	3985.28142	4029.64352	3298.36234	3148.62841	3153.85435
0.00013903	57.5944288	56.4608167	50.7687301	24.7997169	21.3795756	20.1672929
2.99E-126	2458.4916	2435.27221	2448.84463	8036.18651	7232.12735	8176.28595
1.53E-48	4013.54118	4280.36909	4031.63445	8443.76446	7738.43457	8607.55575
2.61E-32	2691.12792	2638.74421	2542.41836	5188.53206	4511.09045	5141.10836
4.67E-22	0	1.06529843	0	1241.06409	1254.59237	1330.26567
1.64E-08	0	1.06529843	0	54.9906765	63.1669279	51.9695625
1.89E-15	4938.43995	5059.10224	4474.6165	3152.79879	2758.93705	3181.77829
2.26E-05	8805.1718	8853.69524	8294.21684	6978.42467	6395.4085	7388.98586
3.76E-10	408.807514	472.992502	463.886828	273.875134	257.526706	238.129189
1.45E-09	107.28374	112.921633	114.478509	36.660451	35.956559	44.2129114
0.00371092	1211.74161	1144.13051	1197.54475	1297.13302	1469.35992	1531.16293
0.0001085	11.2930253	18.1100733	13.9365141	59.3036707	50.5335423	32.5779347
6.48E-10	764.53781	813.888	851.122828	1186.07341	1226.4102	1137.90072
0	193.110732	193.884314	197.102129	4965.33461	4764.72996	5031.73958
1.54E-33	711.460592	746.774199	797.367702	320.239822	305.144852	289.323087
0.0127435	2018.06361	1956.95321	2207.94203	2392.63355	2536.39511	2264.94213
7.08E-27	191.981429	270.585801	239.907136	50.6776823	31.0975645	27.1482789
1.15E-08	2114.05433	2421.42333	2574.27325	3511.85556	3427.53469	3206.59957
0.00017614	2485.59486	2498.12482	2767.39352	2069.15898	2199.18089	1902.70652
2.24E-23	2407.67299	2418.22743	2647.93769	1492.29601	1511.14728	1422.56982
2.44E-20	1387.9128	1340.14542	1324.96431	795.747436	765.777526	831.513
0.0027799	708.072684	727.598827	747.594437	587.645465	544.207379	624.410415
6.73E-07	421.229842	468.731309	581.351733	288.970614	316.806439	238.129189
3.48E-09	124.223278	138.488796	149.319794	57.1471736	53.448939	56.6235532
5.44E-08	12294.7166	10821.3014	11468.7557	15044.8021	16481.7092	14670.9299
1.66E-05	3620.5439	3485.65646	3444.30992	4107.04876	4385.7284	4362.34059
0.0001325	22.5860505	30.8936544	25.8820977	5.39124279	0	6.98098601
0.03797816	2373.79391	2391.59497	2367.21647	2312.84316	1931.9362	1998.88899
1.10E-22	4757.75154	4303.80565	4304.39194	7025.86761	6932.81329	6986.41567
0.00142345	22.5860505	29.828356	33.84582	9.70423703	9.71798891	8.53231624
0.00033915	2271.02738	2472.55765	2821.14865	1969.96012	2089.36762	1739.81685
1.74E-14	1145.11276	1051.44955	1051.21135	2129.5409	1694.81727	1791.01074
5.42E-23	5594.56471	5186.93805	5214.24722	3463.33437	3101.01026	3239.95317
2.50E-16	453.979616	428.249968	433.027404	219.962706	222.541946	214.083571
1.80E-14	1311.12023	1331.62304	1408.58339	1973.19486	2016.4827	1974.06771
1.07E-05	448.333103	455.947728	424.068216	279.266377	329.439824	297.855403

3.16E-16	2428.00043	2536.47556	2548.39116	3670.35809	3833.74663	3531.60326
8.11E-16	836.813172	762.753675	854.109224	461.490383	482.01225	468.501728
2.29E-20	789.382466	804.300314	778.453861	438.847163	430.506909	403.345858
9.11E-17	850.364802	772.341361	780.444792	424.829932	447.02749	467.726063
8.40E-51	991.527618	1031.20888	1061.166	388.169481	392.606752	397.916203
6.71E-22	3251.26197	3520.81131	3717.06741	5899.09786	6447.88564	5639.08537
1.81E-22	9130.41092	8645.96205	8522.17839	13783.2513	12807.3376	13406.5958
0.00893581	4789.37201	5087.8653	5407.36748	4454.2448	4618.96013	4055.95287
9.86E-12	385.092161	411.205194	310.585172	173.598018	172.008404	186.935292
1.18E-07	5610.37495	5538.48653	5550.71449	6790.80942	7530.46961	6967.02404
7.68E-13	1677.01425	1637.36369	1656.45425	1172.05618	1056.34539	987.421688
0.0001612	1862.21987	2000.63045	2287.57925	2747.37733	2816.27319	2444.12077
0.00288592	1107.84578	1208.04842	1169.67172	1010.3189	959.165506	934.67646
0.0218925	1622.80773	1612.86182	1721.1595	1917.12594	1896.95144	1809.62671
5.53E-10	2944.09169	2851.80389	3020.24171	2263.24372	2179.74491	2095.84713
9.37E-08	4817.60458	4518.99593	4831.98854	3726.42702	3793.90287	3578.14316
2.37E-07	7357.40596	7519.94161	7666.07824	9052.9749	9282.62301	9221.10685
0.02657514	448.333103	445.294743	355.38111	321.318071	306.116651	361.459942
0.00142555	1123.65601	1110.04096	1340.89175	1479.35702	1646.22732	1417.91583
0.01070079	261.998186	230.104461	200.088524	130.468076	168.121208	186.935292
2.91E-26	715.977802	712.684649	690.852915	343.96129	333.32702	311.817375
0.00446898	15807.9768	15513.941	15879.6624	14432.357	13119.285	14014.7172
1.24E-28	137.774908	154.468272	179.183753	20.4867226	15.5487823	10.8593116
2.61E-08	2754.36886	2734.62107	2810.19853	3791.12193	3506.2504	3500.57665
6.44E-29	2906.8247	2745.27405	2811.19399	4516.78321	4745.29399	4539.19224
5.72E-66	4811.95806	4962.16008	4844.92959	9746.28872	9278.73581	9600.40709
3.24E-11	667.417793	687.117487	637.097789	1018.94489	1014.55804	967.254395
2.64E-131	1443.24863	1546.81332	1579.80342	331.022308	310.003846	307.163384
0.00015813	6.77581516	0	0	24.7997169	14.5769834	34.1292649
0.00010575	2625.62837	2544.99795	2939.60902	3509.69906	3537.34796	3182.55396
0.0095031	3840.75789	3773.28703	3881.31919	3268.17138	3477.09643	3445.50443
0.00022282	7.90511768	8.52238743	1.99093059	22.6432197	32.0693634	31.0266045
2.62E-06	7938.99676	8232.62626	7407.25726	6353.04051	6134.9664	6433.36644
6.75E-10	108.413043	148.076482	136.378745	313.770331	251.695913	272.258454
3.05E-34	11401.4383	12357.4618	12219.3365	19852.7125	19498.173	19573.1334
5.07E-07	6735.16027	6315.08909	6991.15277	5294.20042	5413.89162	5086.03614
1.52E-11	2799.54096	2732.49047	2991.37321	3796.51318	4246.76116	4062.93386
1.11E-08	245.058648	254.606324	227.961553	135.859318	105.926079	129.536074
3.38E-65	1350.64582	1520.18086	1498.17527	3604.58493	3412.95771	3499.02532
0.00060176	380.574951	343.026094	391.217861	528.341794	451.886484	525.125281
2.63E-08	3173.3401	2912.5259	3023.2281	2153.26237	2399.37146	2240.12084
1.74E-05	327.497733	324.916021	429.045542	210.258469	247.808717	207.87825
0.00027744	123.093975	153.402974	104.523856	78.7121448	49.5617435	69.034195

5.07E-08	184.076312	171.513047	195.111198	93.8076246	94.2644925	87.6501577
2.82E-08	1329.18907	1418.97751	1296.09581	1002.77116	974.714288	971.132721
8.88E-05	16598.4885	16409.857	15508.3538	13592.4013	12560.5007	14019.3712
2.16E-13	5848.65778	5972.06299	5904.10467	4406.80186	4500.40067	4352.25694
6.82E-42	341.049363	422.923476	443.977522	1328.40222	1378.01083	1133.24673
2.63E-09	2610.94744	2758.05763	2845.03981	1952.70814	2119.49338	1931.40613
3.55E-26	8818.72343	9567.44519	9322.53249	14507.8344	14025.0016	14268.3597
3.44E-10	2868.42842	3045.68821	3005.30973	4194.38689	3933.84191	3879.10123
2.14E-08	140.033513	143.815288	190.133871	309.457336	354.706595	277.68811
6.72E-35	3900.61093	3949.06128	3869.3736	2295.59118	2073.81883	2209.09424
2.70E-05	5489.53958	5850.61897	5592.52403	4594.41711	4411.96697	4848.68262
5.71E-35	796.158281	789.386136	832.208987	276.031631	314.862841	340.516984
1.77E-05	374.928439	409.074597	459.904967	582.254222	580.163938	599.589132
4.02E-21	2180.68318	2260.56327	2019.79908	1141.86522	937.78593	1199.95393
0.00072841	138.904211	136.358199	169.2291	210.258469	253.639511	213.307906
1.08E-23	1286.27558	1394.47564	1463.33398	689.000829	757.031336	753.170824
0.00172304	1934.49523	2156.16402	2358.25728	1727.35419	1804.63054	1737.48985
0.00225817	7006.19287	7213.13566	5960.84619	5116.28941	4792.91213	6067.25251
2.10E-15	1778.65148	1670.38794	1835.638	2655.7262	2624.82881	2558.14354
1.83E-08	1815.91846	1730.04465	2117.35468	2870.29766	2799.75261	2562.02187
4.18E-52	2057.5892	2108.22559	2073.55421	3979.81543	4047.54238	3905.47384
3.07E-13	5009.58601	4892.91568	4189.91343	7101.34501	6976.54424	7774.49142
8.11E-21	1466.96398	1523.37675	1392.65595	2351.66011	2317.74036	2511.60363
2.01E-46	2108.40782	2139.11925	2381.15299	5519.55437	5777.34441	4804.46971
0.04735163	2932.79866	2891.21994	3555.80204	3711.33154	4335.19485	3187.98361
1.28E-58	2343.30274	2290.39162	1928.21628	6271.09362	5828.84975	6894.11152
1.27E-06	3006.20332	3156.47924	3560.77936	4642.93829	4908.5562	4016.39395
4.82E-13	2542.05999	2729.29457	2917.70878	4688.22473	4500.40067	3867.46625
0.07663017	7847.52325	7901.31845	9347.41912	7342.87268	8109.66175	6347.26761
0.01206764	57.5944288	76.7014869	68.6871054	36.660451	26.2385701	48.8669021
0.04437727	3403.71781	3106.41022	3269.10803	3741.5225	3742.39753	3436.19645
0.08309481	2758.88607	3143.69566	2816.17132	2754.92507	2116.57799	2681.47429
7.01E-05	2197.62272	2294.65282	2469.7494	2859.51518	3162.23359	2780.75943
0.08495211	588.366616	498.559665	599.270108	662.044615	616.120497	672.501652
2.77E-12	2651.60233	2709.0539	2540.42743	4085.48379	3539.29156	3894.61453
3.13E-19	3020.88426	3159.67514	3056.07846	2090.72396	1988.30053	2060.9422
0.01663144	466.401943	504.951455	561.442427	635.088401	664.710442	580.973169
1.96E-20	1279.49976	1213.37491	1207.4994	1963.49063	1937.76699	1974.84338
8.85E-15	2035.00315	2179.60059	2376.17566	1455.63555	1258.47956	1291.48241
4.73E-18	2818.73911	2993.48858	3539.87459	1783.42312	1757.98419	1569.94619
0.00712331	29.3618657	21.3059686	31.8548895	50.6776823	51.5053412	56.6235532
0.00090224	4893.26785	4898.24218	5228.18373	4143.70921	4475.13389	4150.58402
8.29E-05	3259.16709	3217.20125	3189.47081	2784.03778	2668.55976	2631.83173

1.17E-11	635.797322	644.505549	665.966283	1067.46607	954.306511	986.646023
0.00038737	1374.36117	1390.21445	1417.54258	1070.70082	1218.63581	1067.31519
0.02347527	406.548909	388.833927	328.503548	475.507614	431.478708	480.91237
3.47E-05	1163.1816	1092.99619	1064.1524	829.173142	716.215783	893.566209
5.01E-14	2321.84599	2265.88976	2293.55204	3189.45924	3133.07963	3277.96076
2.35E-09	2285.70831	2288.26103	2210.92842	1658.34628	1628.73494	1753.00315
0.07781329	304.911682	287.630576	202.079455	335.335302	292.511466	377.74891
0.01132371	135.516303	141.684691	140.360607	76.5556477	94.2644925	110.920111
9.00E-05	658.383373	651.962638	676.916401	844.268621	919.321751	821.429354
1.43E-05	798.416886	813.888	827.231661	1106.28302	1148.66629	993.627009
0.0013156	686.615936	643.440251	627.143136	792.512691	782.298108	892.014879
1.77E-60	871.82155	849.042848	877.004925	270.640388	252.667712	281.566436
6.96E-19	938.450399	918.287246	1043.24763	1785.57961	1762.84319	1571.49752
1.32E-18	2883.10935	2885.89344	2865.94459	1957.02113	1870.71287	1970.18939
3.18E-80	100.507925	106.529843	108.505717	604.897441	651.105257	607.345783
4.98E-22	1093.16485	1145.19581	1343.87815	2305.29542	2448.93321	2152.47069
1.49E-09	1629.58355	1793.96255	1986.94873	2693.4649	2786.14742	2499.19299
0.02175958	75.6632693	80.9626806	84.6145501	100.277116	118.559465	129.536074
6.94E-07	2082.43386	2103.9644	1837.62894	1385.5494	1509.20368	1540.47091
9.03E-09	4324.09937	4340.0258	4321.31485	3322.08381	3334.242	3500.57665
0.00099597	71.1460592	92.6809633	64.7052442	40.9734452	41.7873523	31.8022696
3.21E-05	5754.92567	5918.79807	5479.04099	7058.21507	6632.52743	7091.90612
9.33E-43	129.869791	182.166031	169.2291	587.645465	612.233302	593.383811
8.31E-33	2032.74455	1922.86366	1605.68552	711.644049	561.699759	730.676536
5.86E-05	3031.04798	3152.21805	3282.04908	2632.00473	2615.11082	2330.098
1.41E-05	752.115482	623.199581	614.202087	438.847163	294.455064	460.745077
1.55E-08	1552.79097	1790.76666	1842.60626	2602.89202	2674.39055	2296.7444
0.00225268	12355.6989	12190.2099	10986.9505	14332.0798	12999.7538	15103.7511
1.65E-12	573.685683	613.611895	719.721409	362.291516	350.8194	328.106342
8.17E-57	437.040078	446.360042	410.131702	57.1471736	69.9695202	81.4448368
0.00981165	237.153531	198.145508	201.08399	255.544908	279.878081	311.817375
0.00020859	1880.28871	1799.28905	1855.54731	2167.2796	2374.10469	2190.47828
3.39E-24	540.93591	586.979434	521.623815	242.605926	234.203533	201.672929
2.44E-07	3279.49454	3296.03334	2790.28922	2106.89768	2008.70831	2416.19682
0.04763999	40.6548909	47.9384293	39.8186118	23.7214683	14.5769834	32.5779347
0.06406506	1885.93522	1932.45135	1919.25709	1981.82085	2196.26549	2198.23493
8.33E-12	2543.18929	2566.30391	2449.84009	3466.56912	3327.4394	3480.40936
4.74E-10	732.91734	826.671581	820.263403	502.463828	514.081614	526.676611
6.61E-44	3300.95128	3461.1546	3378.60921	1767.24939	1849.33329	1811.9537
9.44E-29	2449.45718	2424.61922	2288.57471	4213.79537	3881.36477	4219.61821
4.56E-27	1147.37137	1129.21633	1110.93927	1975.35136	1883.34625	1963.98406
0.00060121	2184.07109	2136.98865	1757.99171	1520.33047	1243.90258	1697.15527
4.21E-25	1979.66733	1927.12486	2195.00098	3867.67758	3959.10868	3444.72876

4.77E-20	1744.7724	1767.33009	1819.71056	2858.43693	2665.64436	2738.87351
0.0648238	12635.766	12298.8704	11049.6648	11229.9587	9845.29457	11049.3495
2.15E-08	732.91734	711.61935	629.134067	1093.34404	942.644925	1125.49008
1.47E-30	1920.9436	1936.71254	2030.7492	3310.22308	3583.02251	3357.07861
3.24E-11	5098.80091	5156.0444	5769.71685	3867.67758	3835.69022	3575.0405
1.63E-26	2268.76878	2362.83192	2245.76971	1352.12369	1230.2974	1349.6573
6.63E-05	0	2.13059686	0.9954653	20.4867226	20.4077767	17.8402976
0.081207	13.5516303	13.8488796	17.9183753	34.5039539	17.49238	33.3535998
1.75E-06	11.2930253	7.457089	9.95465295	40.9734452	40.8155534	47.3155718
2.13E-06	180.688404	172.578345	214.025039	315.926828	286.680673	347.49797
1.61E-13	1011.85506	895.915979	808.31782	517.559308	437.309501	515.041635
0.05958192	0	1.06529843	0.9954653	5.39124279	4.85899446	6.2053209
4.17E-91	939.579702	990.727539	945.692031	2719.34287	2641.34939	2878.49323
1.90E-28	239.412136	237.56155	165.247239	4.31299423	1.94359778	0.77566511
0.04384085	0	0	0	1.07824856	4.85899446	4.65399067
0.0230785	3.38790758	9.58768586	12.9410488	29.1127111	24.2949723	17.0646325
9.08E-08	222.472598	255.671623	211.038643	109.981353	124.390258	127.984744
0.01375137	11.2930253	24.5018639	13.9365141	40.9734452	26.2385701	45.7642416
2.61E-39	1317.89605	1464.78534	1358.81013	2671.89993	2710.34711	2643.4667
6.97E-08	233.765623	248.214534	237.916206	400.030215	363.452785	401.794528
1.92E-13	645.961045	719.076439	718.725943	1161.2737	1160.32788	1054.12889
1.11E-20	14123.0574	12752.6875	16236.039	6443.61339	7452.7257	5169.03231
7.37E-12	924.898769	1012.03351	1112.9302	561.767499	649.161659	497.977002
3.59E-67	5160.91254	4953.63769	5554.69635	12241.3559	12402.0975	11427.0984
8.78E-12	311.687497	383.507434	393.208792	668.514106	613.2051	653.110025
2.60E-17	1335.96489	1450.93646	1287.13663	2172.67085	2156.42174	2093.52014
8.85E-13	3906.25744	3884.07807	3685.21252	5707.16962	5104.85958	5833.77731
5.84E-09	1.12930253	0	0	71.1644049	66.0823246	56.6235532
3.66E-15	369.281926	401.617508	350.403784	183.302255	178.810996	169.094994
1.24E-71	77.9218743	96.942157	84.6145501	588.723713	561.699759	519.695625
6.11E-05	671.935003	644.505549	623.161275	804.373425	839.634242	951.741093
3.57E-11	5403.71259	5176.28507	5502.93215	3960.40696	4121.3991	4036.56124
2.16E-21	872.950853	922.548439	943.7011	1532.1912	1627.76314	1516.42529
4.16E-41	833.425264	753.165989	784.426653	305.144342	293.483265	300.958064
8.78E-11	4125.34213	3983.15083	4152.08575	5711.48262	5445.96099	5252.02848
4.20E-21	755.50339	752.100691	794.381306	379.543493	405.240138	330.433338
9.27E-10	3027.66007	2863.52218	3180.51162	2027.10729	2265.26322	1852.28829
1.18E-08	669.676398	638.113759	628.138601	431.299423	439.253099	426.615812
6.02E-06	322.980523	330.242513	369.317625	514.324563	497.561032	475.482714
6.42E-08	5608.11635	5664.19175	5285.92072	6954.7032	6935.72869	7616.25574
0.00954007	41.7841935	47.9384293	36.8322159	78.7121448	68.0259224	68.2585299
3.85E-32	311.687497	288.695874	259.816442	72.2426534	53.448939	62.053209
1.81E-30	16303.7406	16915.8738	17293.2231	10741.5121	10474.0485	10971.783

1.49E-12	1014.11367	1132.41223	1039.26577	703.01806	627.782084	670.950322
3.92E-20	222.472598	224.777968	228.957018	79.7903933	67.0541235	62.8288741
2.12E-05	7180.10546	7189.6991	6238.58101	5178.82783	4357.54623	5605.73177
1.04E-05	21.456748	18.1100733	29.8639589	2.15649712	2.91539667	0
0.03511483	11.2930253	14.914178	15.9274447	2.15649712	6.80259224	4.65399067
0.00098934	44.0427985	49.0037277	45.7914036	26.956214	15.5487823	12.4106418
0.02675024	1688.30728	1897.2965	1733.10508	1940.84741	1970.80815	2268.04479
3.45E-07	206.662362	183.23133	188.142941	103.511862	107.869677	96.1824739
1.41E-06	1600.22168	1643.75548	1726.13682	2097.19345	2171.97052	2081.88516
0.00024161	3767.35323	3967.17135	3966.9292	3284.34511	3352.70618	3305.88471
1.77E-09	498.022414	477.253696	486.782529	718.11354	771.60832	716.714564
1.19E-06	761.149903	807.496209	925.782725	1191.46466	1304.15411	1106.87412
1.71E-15	2929.41075	3074.45127	2923.68157	2067.00249	1809.48954	1974.84338
0.02789404	475.436364	435.707057	397.190653	510.011568	520.884206	542.965579
0.00470175	2371.53531	2208.36364	2249.75157	2663.27394	2617.05441	2575.20817
1.67E-29	1494.06724	1706.60808	1803.78312	3443.9259	3564.55833	3152.30302
1.65E-19	7223.01896	7556.16176	7599.38207	5252.14873	5083.48	4909.1845
8.48E-05	2167.13155	2223.27782	2454.81742	2776.49004	3048.53312	2771.45145
5.33E-23	1010.72576	942.789109	894.923301	483.055354	448.971088	502.630993
0.00079067	2626.75768	2666.44197	2496.62696	2302.06067	2131.15497	2077.23117
1.18E-36	416.712632	446.360042	401.172514	107.824856	131.19285	114.022772
0.08417692	3288.52896	3260.87849	2940.60448	2865.98467	2589.84405	3020.43995
8.25E-07	1012.98437	993.923434	950.669357	1275.56804	1285.68993	1345.0033
9.52E-10	1009.59646	1012.03351	1003.42902	1421.1316	1355.65945	1499.36066
6.43E-12	1486.16212	1581.96817	1320.98245	2285.88694	2164.19613	2565.12453
2.10E-05	364.764716	334.503707	305.607846	185.458752	226.429142	228.821208
3.35E-07	441.557288	411.205194	484.791599	643.71439	683.174621	645.353373
1.89E-23	1026.536	943.854408	1038.2703	1740.29317	1759.92779	1691.72561
1.12E-08	130.999093	122.509319	136.378745	26.956214	54.4207379	51.1938974
5.83E-102	538.677305	564.608167	567.415218	31.2692082	31.0975645	46.5399067
1.48E-36	102.76653	110.791037	105.519321	437.768915	379.001568	386.281226
0.02223298	767.925718	703.096963	822.254334	905.728789	971.798891	850.904628
2.10E-08	762.279205	690.313382	741.621645	1048.0576	998.037461	1072.74485
4.66E-55	3515.51876	3827.61725	3660.32589	1789.89261	1679.26848	1698.7066
1.21E-09	1878.0301	2007.02224	2186.04179	1341.34121	1485.8805	1336.47099
0.00022278	931.674584	1036.53537	1001.43809	830.25139	667.625838	749.292498
0.07106552	1494.06724	1413.65102	1436.45642	1559.14742	1661.7761	1593.21614
9.92E-06	1605.86819	1482.89541	1271.20918	2026.02904	1871.68466	2447.22343
0.00855044	1269.33604	1219.7667	1147.77149	891.711558	1101.04814	999.83233
0.00854457	205.53306	192.819016	188.142941	131.546324	137.023644	155.133022
1.08E-07	2019.19292	1937.77784	1821.70149	2470.26745	2590.81584	2879.2689
0.01908653	1701.85891	1609.66593	1643.5132	1725.19769	2045.63667	1999.66466
2.56E-07	1385.6542	1257.05215	1245.32708	1660.50278	1781.30737	1915.11716

0.00182066	3410.49363	3440.91393	3084.94695	3903.25978	3750.17192	4158.34067
8.66E-05	2329.75111	2288.26103	1829.66521	1569.9299	1380.92622	1712.66857
1.31E-09	3207.21917	3243.83372	3041.14648	2225.50503	2213.75787	2461.96107
6.63E-33	2320.71669	2249.91028	2145.22771	1124.61325	1160.32788	1240.28851
3.46E-06	2211.17435	2182.79648	1991.92606	2731.2036	2688.96753	3208.1509
1.05E-09	1684.91937	1579.83757	1288.13209	2471.3457	2257.48882	2914.17383
0.04230148	918.122954	868.218219	653.025234	672.827101	542.263781	723.69555
5.34E-18	3733.47415	3748.78517	3760.86789	5245.67924	5523.7049	5286.15774
8.90E-07	1701.85891	1840.83568	1606.68099	1324.08923	1182.67925	1289.15542
6.86E-33	1731.22077	1562.7928	1579.80342	2925.28834	2926.08646	2977.77837
0.00478703	2791.63584	2802.80017	2978.43216	3257.3889	3445.02707	3190.31061
8.63E-06	1375.49048	1511.65847	1409.57886	1890.16972	1742.43541	1901.15519
0.01199887	635.797322	615.742492	623.161275	697.626818	792.016096	752.395159
7.40E-12	2743.07584	3009.46806	2736.5341	3947.46797	3863.87239	4142.0517
0.04427086	983.6225	959.833884	1051.21135	1092.26579	1173.93306	1139.45205
3.17E-64	2388.47484	2417.16213	2160.15969	5503.38064	5392.51205	5948.57575
0.00056543	3496.32062	3237.44193	3329.83141	3806.21741	4125.28629	3993.124
3.96E-15	1520.0412	1620.31891	1536.99842	2299.90418	2335.23274	2240.89651
4.90E-10	2647.08512	2815.58375	2960.51379	3826.70413	4148.60947	3754.99481
6.64E-22	912.476441	891.654785	935.737378	1613.05984	1482.96511	1614.1591
0.00076272	629.021507	558.216377	525.605676	416.203944	417.873523	456.866751
0.01115778	1291.92209	1206.98312	1023.33832	922.980766	908.631963	1034.73726
0.00550994	127.611185	62.8526073	64.7052442	34.5039539	49.5617435	43.4372463
2.61E-11	75.6632693	101.203351	104.523856	23.7214683	19.4359778	24.8212836
0.00014059	28.2325632	45.8078324	18.9138406	8.62598847	5.83079335	5.42965579
4.17E-41	1285.14627	1376.36557	1358.81013	628.61891	593.769123	570.113858
6.39E-22	28.2325632	44.742534	60.723383	228.588694	224.485544	208.653915
3.37E-11	188.593522	168.317152	102.532925	38.8169481	22.3513745	45.7642416
1.53E-23	167.136774	165.121256	132.396884	28.0344625	24.2949723	25.5969487
0.00576536	4.5172101	13.8488796	4.97732648	0	0.97179889	0
1.89E-14	5394.67817	5331.81864	5729.89824	7598.41759	7449.8103	7685.28993
0.03704902	1431.9556	1335.88423	1444.42014	1256.15957	1283.74634	1160.39501
0.00012307	25054.7058	24236.6046	21623.4971	30068.0393	27267.7051	31519.9275
0.00416841	8027.08236	7878.94718	6870.70147	6231.19842	5737.50065	6865.41191
2.02E-17	963.295055	1091.93089	1062.16147	1658.34628	1669.5505	1734.38719
1.65E-15	571.427078	585.914136	714.744082	1215.18613	1280.83094	1072.74485
2.52E-11	511.574044	559.281675	551.487774	296.518354	277.934483	339.741319
2.21E-40	1392.43001	1388.08385	1237.36336	487.368349	371.227176	487.893356
4.24E-06	1876.9008	1875.99053	1724.14589	2429.294	2208.89888	2554.26521
4.79E-87	1498.58445	1469.04653	1511.11632	3759.85272	3559.69934	3754.99481
0.00059568	790.511768	965.160376	953.655753	666.357609	701.6388	714.387568
4.50E-41	613.211272	595.501822	673.930005	214.571463	202.134169	183.832632
0.01047736	3384.51967	3535.72549	3794.71371	4044.51034	4286.60491	3969.07838

4.23E-16	2188.5883	2340.46065	2450.83556	1483.67002	1539.32944	1376.02991
2.98E-13	56.4651263	66.0485026	51.7641954	4.31299423	4.85899446	3.10266045
7.09E-15	546.582423	520.930932	570.401614	928.372009	895.026779	885.033893
0.0309519	134.387001	153.402974	148.324329	112.13785	68.0259224	118.676762
5.89E-07	1636.35936	1838.70509	1670.39077	2187.76633	2307.05057	2257.96114
7.87E-05	2079.04595	1967.6062	2496.62696	2966.26179	3196.24655	2675.26897
5.98E-08	1447.76584	1352.929	1408.58339	1907.4217	1964.00556	1787.90808
0.00386543	295.877262	318.52423	400.177049	437.768915	467.435267	452.21276
3.25E-19	2088.08037	2090.11552	2282.60192	3660.65386	3891.08276	3319.84668
0.00801632	22.5860505	19.1753717	25.8820977	53.9124279	62.195129	31.0266045
0.0141807	4120.82492	3874.49039	4206.83634	4733.51117	4502.34426	4517.47361
0.00057643	648.21965	721.207036	587.324524	512.168065	433.422306	504.182323
0.00019199	3408.23502	3203.35238	3079.96962	2441.15474	2404.23046	2830.40199
1.12E-40	1097.68206	975.813361	1092.02543	407.577955	428.563311	366.113933
0.00315446	21.456748	9.58768586	11.9455835	46.364688	34.0129612	32.5779347
0.01360383	18.0688404	19.1753717	15.9274447	32.3474568	46.6463468	32.5779347
0.00243476	691.133146	635.983162	657.007095	566.080493	515.053412	504.182323
2.87E-09	1151.88858	1164.37118	1005.41995	624.305916	478.125055	729.125206
4.58E-16	8044.02189	7193.96029	7122.55419	5019.24704	4576.20098	4976.66736
3.32E-25	403.161002	415.466387	476.827877	899.259298	916.406355	889.687884
1.13E-18	1010.72576	979.009256	827.231661	371.995753	328.468025	472.380053
1.35E-57	2485.59486	2415.03154	2264.68355	974.736697	875.590801	995.178339
3.33E-06	2281.1911	2360.70132	2801.23934	1617.37284	1963.03376	1568.39486
0.00663234	3856.56813	3759.43816	3739.96312	3185.14624	3364.36776	3432.31812
2.67E-198	2760.01537	2796.40838	2635.9921	370.917504	275.019086	333.535998
6.70E-15	3660.06949	3336.51468	3398.51852	5577.77979	4913.41519	5176.78896
2.34E-13	2601.91302	2832.62852	3019.24624	4307.60299	4290.49211	4004.75897
2.28E-06	6460.73975	6539.86705	6766.17761	8214.09752	8670.38971	7783.02374
7.22E-07	1812.53055	1839.77039	2045.68118	2788.35077	2869.72213	2348.71396
1.01E-06	1914.16778	1895.1659	1894.37046	1438.38358	1553.90643	1422.56982
0.0021549	6146.79365	5997.63015	6110.16598	7199.46563	6789.95885	6829.73131
0.02925266	1108.97508	981.139853	750.580833	698.705066	629.725682	868.744926
5.65E-15	1146.24206	1224.02789	1208.49487	704.096309	761.890331	769.459791
5.49E-06	27342.6728	26696.3786	21065.0411	16355.9524	15250.44	19803.506
9.17E-20	9414.99516	9003.90232	8026.43668	5168.04534	5359.47089	5618.14241
0.04151672	4632.39896	5095.32238	5605.46508	4642.93829	4659.77568	3883.75522
0.06046129	11.2930253	18.1100733	10.9501183	4.31299423	7.77439113	2.32699534
6.08E-11	996.044828	988.596942	1053.20228	1410.34911	1458.67014	1545.1249
4.86E-08	6322.96484	6439.729	6980.20265	5185.29732	5129.15455	4808.34803
3.96E-17	112.930253	123.574618	112.487578	9.70423703	23.3231734	22.4942883
3.95E-11	6782.59097	7318.60021	8400.73163	5243.52274	4722.94261	4405.77784
1.89E-07	16.9395379	19.1753717	24.8866324	62.5384164	66.0823246	83.7718321
0.00432444	18.0688404	34.0895497	36.8322159	59.3036707	63.1669279	58.1748834

5.14E-13	830.037357	744.643602	724.698735	478.74236	418.845322	439.802119
8.85E-19	1497.45515	1523.37675	1663.42251	2412.04203	2552.91569	2482.12836
2.92E-10	1395.81792	1385.95326	987.501573	662.044615	458.689077	687.239289
6.85E-05	4573.67523	4289.95677	3342.77246	2953.3228	2498.49495	3147.64903
9.92E-08	277.808421	328.111916	372.304021	580.097725	546.150977	500.303997
5.68E-05	1375.49048	1233.61558	1389.66955	1615.21634	1812.40493	1699.48226
4.70E-06	2472.04323	2486.40653	2244.77424	3084.86913	3014.52016	3659.588
1.41E-113	4560.1236	4808.75711	4768.27877	11494.1296	11885.1004	11403.8285
2.82E-66	2363.63019	2586.54459	2693.72909	5809.60323	5864.80631	5827.57199
1.01E-26	1385.6542	1487.15661	1466.32038	2460.56321	2387.70988	2460.40974
6.99E-12	1372.10257	1420.04281	1454.3748	2126.30616	2016.4827	1949.24643
1.54E-69	5420.65213	5837.83539	5798.58535	11786.335	11516.7887	11850.6116
8.49E-06	1151.88858	1081.27791	1209.49033	1531.11295	1472.27532	1452.04509
3.30E-21	1020.88948	1024.81709	936.732843	559.611002	538.376586	525.125281
0.08355519	10021.4306	9182.87246	9235.92701	9898.32177	10894.8374	10358.2319
3.80E-12	134.387001	110.791037	117.464905	26.956214	19.4359778	40.3345858
8.45E-10	1667.97983	1617.12301	1575.82156	1154.80421	1063.14799	1198.4026
2.37E-27	518.34986	589.110031	546.510447	1165.58669	1241.95898	1079.72584
2.19E-19	1098.81136	1133.47753	1171.66265	643.71439	688.033615	615.878099
3.11E-15	594.013129	610.416	566.419753	342.883042	315.83464	297.855403
1.78E-09	252.963766	262.063413	267.780164	519.715805	410.099132	448.334435
2.02E-10	99.3786223	122.509319	102.532925	21.5649712	40.8155534	24.8212836
3.14E-16	16438.1276	18110.0733	17593.8536	24130.1245	24703.1278	25271.1694
1.87E-31	1969.50361	2115.68268	1727.13229	861.520598	693.864408	830.737335
2.20E-08	1035.57042	1065.29843	1101.98008	783.886702	699.695202	768.684126
2.01E-08	2272.15668	2213.69014	1995.90792	2918.81885	2864.86313	3225.9912
0.00017167	6390.723	6422.68423	6038.49248	5394.47754	5107.77497	5408.71283
1.09E-05	327.497733	395.225717	397.190653	263.092648	222.541946	236.577859
8.42E-06	2036.13245	2128.46626	1834.64254	1524.64346	1420.76998	1594.76747
7.22E-51	8633.51781	9262.76984	8854.6638	15962.3917	15900.5735	16230.7925
2.68E-16	779.218743	810.692104	840.172709	1264.78556	1290.54893	1360.51661
1.35E-06	402.031699	400.552209	420.086355	247.997169	258.498505	288.547422
0.00342426	3242.22755	3219.33185	3259.15338	3745.83549	3646.18944	3652.60701
5.05E-12	2065.49432	1922.86366	1530.03016	945.623986	661.795045	1013.01864
0.02491485	0	1.06529843	0	4.31299423	6.80259224	5.42965579
3.71E-18	355.730296	377.115644	393.208792	164.972029	170.064806	165.216669
2.32E-11	1739.12589	1773.72188	1623.6039	2599.65728	2319.68395	2688.45528
0.02427783	493.505204	555.020481	594.292781	715.957043	689.005414	600.364797
0.00252336	841.330382	857.565235	921.800864	1166.66494	1181.70745	963.376069
4.46E-18	2074.52874	2037.91589	2123.32748	1392.01889	1260.42316	1323.28468
1.69E-09	2709.19676	2809.19196	3099.87893	2054.0635	2134.07037	2013.62663
2.44E-17	225.860505	273.781696	278.730283	707.331055	577.248541	539.862918
0.00469431	214.56748	248.214534	284.703075	163.893781	183.66999	185.383962

1.27E-37	6571.4114	6790.21219	7065.81267	3937.76374	3816.25425	3606.06711
0.03809647	4781.4669	5275.35782	5590.5331	4666.65976	4744.32219	4474.81203
1.41E-17	3244.48616	3368.47363	3466.21016	2235.20926	2296.36078	2092.74447
9.98E-28	2467.52602	2558.84683	2531.46825	4030.49311	3982.43186	3949.68675
7.30E-12	4212.29842	4004.45679	3901.22849	5390.16455	5448.87638	5777.15376
0.0624655	746.46897	833.063371	692.843846	638.323147	577.248541	708.182248
3.05E-08	1156.40579	1152.6529	1174.64905	1530.0347	1540.30124	1696.3796
3.54E-16	1188.02626	1214.44021	1278.17744	2105.81944	1887.23345	1884.09056
4.37E-14	2367.01809	2315.95878	2369.2074	1537.58244	1695.78907	1533.48993
5.59E-31	30417.7635	28700.205	28372.7519	47777.1936	45839.7537	49851.9968
0.00012789	6467.51557	6698.59652	6079.30656	5304.98291	4514.97765	5399.40485
1.82E-14	2494.62928	2639.80951	2892.82215	4413.27135	4584.94717	3900.04418
1.67E-09	3057.02194	2937.02777	3318.8813	4281.72503	4556.765	4086.20381
0.00076495	4051.93746	3780.74412	4488.55302	5736.28233	5479.97395	4559.35953
3.41E-08	5742.50335	5859.14136	6304.28172	4462.87078	4778.33515	4311.14669
1.31E-09	1118.0095	1134.54283	1180.62184	788.199696	836.718845	764.805801
1.18E-06	1848.66824	1759.873	1859.52917	2500.45841	2553.88749	2208.31857
2.78E-08	35.0083783	40.4813403	30.8594242	4.31299423	0	0.77566511
4.21E-15	110.671648	106.529843	101.53746	12.9389827	26.2385701	20.942958
0.01021135	8191.96053	8332.76431	9610.22196	7729.96392	7665.54965	6761.47278
1.23E-48	856.011315	948.115602	979.537851	332.100556	315.83464	297.855403
3.73E-06	2886.49726	2800.66957	2611.10547	2049.75051	2255.54523	2162.55433
0.00012345	2117.44224	2026.19761	1744.0552	1387.7059	1324.56189	1645.1857
3.24E-12	730.658734	699.901068	575.378941	347.196036	370.255378	380.075905
2.40E-24	3331.44245	3072.32067	2979.42763	1657.26803	1873.62826	1790.23508
0.00512947	1796.72032	1628.8413	1613.64924	1897.71746	1964.00556	2078.00684
9.06E-06	3667.97461	3405.75908	2827.12144	2352.73836	2256.51703	2544.18157
1.94E-06	2364.75949	2330.87296	2094.45898	1613.05984	1381.89802	1782.47843
1.98E-21	799.546189	814.953298	785.422118	1338.10646	1332.33628	1366.72193
2.56E-10	510.444742	528.388021	533.569398	794.669188	786.185303	924.592814
1.43E-08	1868.99568	1676.77973	1609.66738	1186.07341	1250.70517	1227.10221
0.01592164	2319.58739	2283.99983	2247.76064	1715.49346	2139.90116	1959.33007
1.04E-51	3279.49454	3401.49788	3119.78824	1426.52284	1451.86754	1574.60018
8.23E-17	2783.73073	2474.68825	2282.60192	1429.75759	1524.75246	1512.54697
0.00057733	2813.09259	2848.608	2096.44991	1908.49995	1466.44453	2073.35285
0.00064376	1438.73142	1547.87862	1070.12519	786.043199	853.239427	1118.50909
0.0001776	2825.51492	2955.13784	2316.44774	1945.1604	1780.33557	2233.13986
3.87E-08	2997.1689	2641.9401	2333.37065	1687.45899	1747.29441	1918.99549
2.97E-05	7969.48793	7787.33151	6127.08889	5036.49902	4371.15141	5826.79632
3.93E-07	1107.84578	1116.43275	1133.83497	1438.38358	1585.00399	1453.59642
6.40E-18	21.456748	11.7182827	27.8730283	132.624573	140.910839	115.574102
5.76E-07	1385.6542	1295.40289	1282.1593	1011.39715	904.744768	1000.60799
5.09E-07	4011.28257	3851.05382	3577.70227	5001.99506	4696.70404	5212.46955

2.59E-17	2594.0079	2875.24046	2887.84482	4200.85638	4245.78936	4150.58402
1.86E-34	9850.90594	9649.47317	10302.0703	16647.0795	17855.8328	16394.4578
9.32E-13	68.8874541	82.027979	102.532925	221.040955	284.737075	212.532241
1.14E-18	255.222371	298.28356	247.870859	78.7121448	65.1105257	98.5094693
1.85E-20	1941.27104	2086.91962	2049.66304	1139.70873	1236.12819	1270.53945
1.26E-16	5314.49769	5431.95669	5319.76654	3801.90442	3834.71843	3554.09754
2.72E-11	2434.77625	2550.32444	2637.98303	3542.04652	3620.92267	3368.71358
0.00832886	1491.80864	1529.76854	1295.10035	1180.68217	1146.72269	1266.66113
1.01E-19	6496.87743	6587.80548	6146.00273	4150.1787	3633.55605	4141.27603
1.71E-05	4150.18678	3958.64896	3788.74091	4817.61456	4808.46091	5437.41244
3.58E-132	862.78713	1000.31522	900.896092	91.6511275	74.8285146	96.1824739
0.00801669	286.842842	231.169759	248.866324	210.258469	179.782795	172.97332
0.00016155	790.511768	731.860021	830.218056	963.954212	1124.37132	995.954004
1.93E-10	1070.57879	1228.28909	1537.99388	2484.28468	2734.64208	2000.44032
0.01645561	223.6019	286.565277	243.888997	378.465244	319.721835	297.079738
0.00155709	3044.59961	3122.38969	3640.41659	4247.22107	4949.37175	3617.70208
4.44E-06	47.4307061	47.9384293	61.7188483	146.641804	97.1798891	119.452427
1.35E-24	1450.02444	1544.68272	1606.68099	3099.96461	3135.99502	2617.86975
1.24E-06	1017.50158	1047.18836	1151.75335	1492.29601	1426.60077	1393.09454
6.15E-08	32.7497733	35.1548482	31.8548895	4.31299423	2.91539667	1.55133022
0.0007861	7199.3036	7068.25508	7833.31641	8449.15571	9269.01783	8561.79151
3.10E-28	1069.44949	1023.75179	1076.09798	1945.1604	2294.41718	2008.19698
3.12E-06	3036.69449	3082.97365	3171.55243	3917.27701	3888.16736	3687.51194
3.20E-30	1018.63088	1062.10253	871.032134	2544.6666	2125.32418	2830.40199
1.23E-24	2031.61524	2011.28343	1653.46786	5182.06257	3799.73367	5441.29076
0.02540723	1054.76856	1039.73127	891.936905	1165.58669	1086.47116	1315.52803
2.85E-17	1175.60393	1102.58387	1108.94834	657.731621	701.6388	683.360964
1.11E-05	2164.87294	2176.40469	2316.44774	2837.95021	3011.60476	2644.24237
1.35E-48	904.571324	949.1809	899.900627	2338.72112	2017.4545	2170.31098
7.86E-13	1880.28871	1818.46442	1924.23442	1294.97652	1311.9285	1323.28468
7.12E-08	1579.89423	1384.88796	1679.34995	2226.58327	2527.64892	2091.96881
1.48E-11	1526.81702	1395.54094	1196.54929	2224.42678	2096.17021	2483.67969
1.35E-10	635.797322	594.436523	547.505913	354.743776	310.003846	369.216593
0.00757738	345.566573	251.410429	276.739352	394.638972	437.309501	350.600631
0.03378527	8582.6992	8571.39116	6468.53349	6307.75407	5378.90686	7468.1037
2.46E-05	1609.2561	1621.38421	1531.02562	1892.32622	2000.93392	2108.25778
0.00037948	413.324725	457.013026	467.868689	545.593771	634.584676	604.243122
0.05520897	1119.1388	1374.23497	1497.1798	1565.61691	1669.5505	1471.43672
1.06E-16	833.425264	904.438366	842.16364	500.307331	499.50463	502.630993
5.94E-05	718.236407	726.533528	776.46293	1015.71014	1129.23031	892.790544
9.72E-08	998.303433	971.552167	971.574128	682.531338	610.289704	735.330526
2.95E-05	1801.23753	1851.48867	1707.22298	1316.54149	1430.48797	1466.78273
1.04E-38	2028.22734	1824.85621	1822.69696	3575.47222	3653.96383	3813.94536

2.79E-21	1726.70356	1567.05399	1620.6175	948.858732	916.406355	994.402674
1.80E-06	297.006564	345.156691	274.748422	136.937567	201.162371	169.094994
2.57E-16	1435.34351	1455.19765	1295.10035	846.425119	723.990174	836.942656
5.61E-31	1634.10076	1657.60436	1513.10725	2772.17704	2898.87609	2961.4894
3.97E-45	1707.50542	1609.66593	1718.1731	784.964951	788.128901	747.741168
4.06E-08	1331.44768	1303.92528	1121.88939	804.373425	753.144141	912.957837
1.17E-08	3160.91777	3248.09491	3338.7906	2571.62281	2468.36918	2488.33368
4.68E-05	1.12930253	3.19589529	0.9954653	22.6432197	20.4077767	19.3916278
5.05E-13	1016.37227	1000.31522	916.823537	549.906765	565.586955	640.699383
0.03302066	39.5255884	22.371267	30.8594242	19.4084741	8.74619002	15.5133022
5.94E-25	979.10529	1030.14358	960.62401	1886.93498	1713.28145	2016.72929
0.02434007	0	0	0	6.46949135	1.94359778	3.87832556
4.54E-06	47.4307061	28.7630576	29.8639589	6.46949135	6.80259224	6.2053209
3.74E-06	949.743425	983.27045	1091.02996	1370.45392	1517.94987	1286.05276
0.00239637	2565.77534	2453.38228	2468.75393	2240.60051	2113.66259	2051.63422
3.70E-06	1899.48685	1923.92896	2101.42724	2622.30049	2545.1413	2433.26146
7.37E-16	1697.3417	1890.90471	1752.01892	1167.74319	983.460478	1019.99962
0.00019784	14.6809328	17.0447749	18.9138406	0	2.91539667	1.55133022
2.68E-25	1118.0095	1097.25738	1018.361	546.672019	465.491669	545.292574
5.31E-30	538.677305	531.583916	467.868689	126.155081	122.44666	177.627311
8.13E-20	1562.9547	1691.6939	1646.4996	2754.92507	2504.32574	2836.60732
7.30E-20	3324.66664	2821.97554	2324.41146	5905.56736	5560.63326	6579.19148
1.41E-13	836.813172	862.891727	669.948144	431.299423	322.637232	394.813542
0.00231765	1808.01334	1552.13981	1605.68552	1378.00166	1293.46432	1409.38351
0.00034774	901.183416	924.679036	928.769121	1131.08274	1237.09999	1088.25815
0.01467578	1980.79663	2115.68268	1801.79218	2264.32197	2178.77311	2490.66068
0.00371029	1162.0523	1095.12678	1034.28844	874.459581	880.449796	957.946414
6.87E-06	487.858691	403.748105	424.068216	607.053939	620.979492	650.783029
5.34E-07	1348.38722	1360.38609	1182.61277	952.093477	800.762286	946.311437
0.02675811	3699.59508	3810.57248	3152.63859	3001.84399	2577.21066	3325.27634
1.26E-18	5363.0577	5871.92494	6168.89844	3684.37533	3715.18716	3360.95693
0.00683159	638.055927	707.358157	790.399445	876.616078	879.477997	866.41793
1.73E-12	1517.7826	1409.38982	1363.78745	883.08557	971.798891	949.414097
2.89E-33	3532.4583	3489.91765	3188.47534	6417.73542	5982.39397	6759.92145
2.58E-10	1348.38722	1466.91594	1590.75354	2157.57537	2292.47358	2059.39087
9.94E-32	395.255884	471.927204	532.573933	1622.76408	1774.50478	1231.7562
8.92E-08	423.488447	446.360042	395.199722	287.892365	230.316337	246.661506
5.27E-24	158.102354	140.619393	166.242704	447.473152	407.183735	395.589207
8.97E-10	1808.01334	1715.13047	1721.1595	2265.40022	2494.60775	2458.08274
4.54E-20	2067.75293	2220.08193	2394.09404	3571.15923	4002.83963	3703.80091
2.02E-30	3421.78665	3658.2348	3685.21252	6434.9874	5908.53726	6063.37418
2.02E-13	5029.91345	4788.51644	4299.41461	7208.09162	6959.05186	8301.9437
0.00150654	6964.40868	6855.19539	7493.86274	8114.89865	8755.90801	8076.22515

0.00271234	12985.8497	13135.1296	14006.1967	11512.4599	12163.0349	11093.5624
2.79E-48	4555.60639	4630.85227	4528.37163	8059.90798	7845.33245	8015.72327
2.78E-22	1712.02263	1688.49801	1456.36573	3211.02421	2927.05826	3720.08988
5.07E-40	1997.73617	1995.30396	2156.17783	3825.62589	3718.10256	3834.11265
2.97E-09	5933.35547	5842.09658	5589.53763	7246.90856	7426.48713	7629.44204
5.71E-52	3393.55409	3305.62102	3227.29849	6060.83515	6374.02893	6287.5414
1.35E-05	2145.6748	2011.28343	1690.30007	1338.10646	1278.88734	1535.81692
8.31E-14	90.3442021	99.0727539	87.600946	12.9389827	14.5769834	22.4942883
5.95E-40	1839.63382	1969.73679	2140.25039	5085.0202	4418.76956	4266.93378
2.07E-40	328.627035	327.046618	356.376576	921.902518	911.54736	828.41034
0.00323963	0	0	0.9954653	8.62598847	8.74619002	9.30798135
5.77E-35	1532.46353	1543.61742	1329.94163	547.750268	518.940608	646.904704
5.00E-07	4159.2212	4053.46052	4482.58023	3116.13833	3443.08347	2962.26506
6.02E-05	6774.68585	6582.47899	7251.96468	8993.67123	8993.99874	7840.42295
3.95E-53	10671.9089	10012.7399	9778.4556	4865.0575	5270.06539	5036.39357
0.00017601	11.2930253	5.32649214	1.99093059	26.956214	35.956559	27.1482789
1.22E-26	13490.648	13461.1109	12742.9512	20875.9703	20082.2241	21943.566
2.76E-05	51.9479162	39.4160419	37.8276812	90.5728789	98.151688	91.5284832
1.10E-07	41.7841935	52.199623	27.8730283	8.62598847	1.94359778	3.10266045
0.00355162	1977.40872	1893.03531	1857.53824	1702.55447	1550.01923	1625.01841
5.81E-16	6764.52213	6716.70659	7116.5814	4842.41428	4968.80773	4617.53441
9.07E-11	473.177758	436.772356	452.936709	281.422874	249.752315	257.520817
6.15E-117	651.607558	576.32645	668.952679	2762.47281	2770.59864	2475.92304
3.98E-29	3057.02194	3372.73483	3611.54809	6359.51	6263.24385	5900.48451
7.90E-34	4094.85096	4133.3579	4102.31248	2463.79796	2322.59935	2474.37171
7.19E-31	619.987087	624.264879	605.2429	255.544908	256.554907	262.174808
0.00589405	1112.36299	1132.41223	1271.20918	959.641217	1040.79661	863.31527
0.00122077	968.941568	930.005528	795.376771	1182.83867	1033.99402	1388.44055
7.01E-19	10319.5665	9892.36121	10194.5601	7308.36873	7219.49396	7208.25589
1.25E-05	4156.9626	4041.74224	3446.30085	3049.28692	2407.14585	2904.09018
0.00010479	31.6204707	37.285445	41.8095424	81.9468905	76.7721124	79.8935066
6.32E-13	1700.7296	1716.19577	1960.07117	1196.8559	1137.0047	1052.57756
6.31E-35	7559.55111	7577.46772	7814.40257	12357.8067	12335.0433	11930.5051
0.00132575	685.486633	626.395476	722.707805	534.811285	560.72796	485.56636
4.37E-179	2006.77059	1983.58567	2104.41363	7262.00404	6911.43372	7369.59423
1.44E-05	28.2325632	34.0895497	38.8231465	4.31299423	10.6897878	4.65399067
1.20E-06	1198.18998	1238.94207	1067.1388	829.173142	771.60832	890.463549
0.07679053	1479.38631	1498.87489	1684.32728	1754.31041	1908.61302	1628.12107
1.19E-06	1681.53146	1608.60063	1813.73777	1264.78556	1341.08247	1228.65354
0.01894111	2871.81632	3196.96058	3479.15121	2829.32422	2809.47059	2496.09033
2.20E-21	448.333103	410.139895	383.254139	168.206775	132.164649	165.992334
1.43E-10	3096.54753	2866.71807	2954.541	2255.69598	2153.50634	2107.48211
0.00325067	153.585144	140.619393	129.410488	85.1816361	72.8849168	107.041785

0.00031557	3098.80613	2852.86919	2756.4434	2378.61632	2465.45379	2346.38696
0.01206885	1527.94632	1376.36557	1354.82827	1118.14376	1288.60533	1184.44063
0.00520023	1894.96964	2061.35246	2023.78095	2219.03553	2472.25638	2313.03336
8.04E-09	271.032606	292.957068	263.798303	119.68559	154.516024	154.357357
1.06E-14	1482.77422	1585.16406	1415.55165	933.763252	847.408633	970.357055
6.29E-11	2037.26176	1836.57449	1944.14372	2601.81377	2826.96297	2783.86209
6.46E-51	6813.08214	6615.50324	6658.66736	12013.8454	11659.6431	11609.3797
1.85E-06	421.229842	458.078324	451.941244	654.496875	709.413191	589.505485
0.00256369	1060.41507	1072.75552	858.091085	1417.89685	1091.33015	1497.03367
8.35E-07	315.075405	298.28356	257.825512	174.676267	167.149409	175.300315
0.00041124	448.333103	336.634304	336.46727	243.684174	207.964963	279.23944
0.00027669	372.669834	377.115644	375.290416	479.820609	518.940608	494.874342
0.08413174	7206.07942	7489.04795	8462.45048	7010.77213	7279.74549	6458.18772
9.22E-29	1399.20583	1521.24616	1328.94617	2627.69174	2720.0651	3035.95325
1.05E-31	723.882919	727.598827	831.213522	281.422874	322.637232	273.809785
1.21E-08	1870.12498	1765.1995	1903.32964	1381.2364	1377.03903	1236.41019
3.44E-34	9.03442021	0	2.98639589	241.527677	251.695913	205.551255
0.01106798	844.71829	803.235015	551.487774	530.498291	470.350663	615.102434
3.79E-07	1885.93522	1718.32637	1612.65378	2283.73045	2299.27618	2408.44017
2.23E-11	5164.30045	5202.91753	5602.47868	7252.29981	7596.55193	7005.80729
0.00252336	1928.84871	2010.21814	1642.51774	1530.0347	1180.73565	1560.63821
0.00024054	430.264262	474.057801	436.013799	345.039539	329.439824	309.49038
8.34E-42	7652.15392	7452.82781	7559.56345	4553.44366	4356.57443	4424.3938
7.78E-55	4365.88357	4929.13583	5100.76417	10567.9141	10923.0195	10448.2091
0.03277612	2229.24319	2131.66216	1909.30244	1782.34487	1750.2098	1923.64948
0.00103224	5219.63628	5298.79438	4916.60309	4414.3496	4109.73751	4546.94889
9.77E-06	1008.46716	895.915979	822.254334	594.114956	518.940608	698.874266
0.00081693	624.504297	736.121214	708.77129	556.376256	533.517591	512.714639
0.01881919	1241.10348	1233.61558	903.882488	867.99009	683.174621	1014.56997
2.41E-06	998.303433	1016.2947	1025.32925	1304.68076	1309.01311	1306.99571
3.86E-07	720.495012	762.753675	598.274643	430.221175	390.663154	482.4637
3.88E-10	599.659641	607.220104	535.560329	360.135019	322.637232	366.113933
5.52E-07	1829.47009	1891.97001	1480.25689	1132.16099	944.588522	1254.25049
7.93E-05	622.245692	581.652942	638.093254	750.460997	847.408633	838.493986
0.0043322	2741.94653	2650.46249	2459.79475	2918.81885	3043.67413	3301.23072
0.00016208	669.676398	664.74622	750.580833	475.507614	555.868966	522.022621
1.05E-09	2048.55478	1940.97374	2249.75157	1422.20985	1459.64193	1500.13633
1.97E-11	437.040078	445.294743	489.768925	731.052523	748.285146	719.817224
1.85E-25	4967.80181	5615.18802	5975.77817	10186.2141	10786.9677	9571.70749
2.78E-14	10464.1172	10908.6559	12186.4861	7676.05149	7257.39412	6651.32834
0.01165965	1150.75927	1080.21261	1023.33832	965.03246	880.449796	908.303846
0.06760256	3497.44992	3451.56691	3448.29178	3268.17138	3123.36164	3154.63001
2.25E-36	5980.78618	6481.27564	6611.88049	12515.231	12441.9412	11188.1936

3.65E-13	2992.65169	3022.25164	3229.28942	4914.65693	4786.10954	4215.73989
2.25E-05	2239.40691	2220.08193	2432.91718	1822.24006	1902.78223	1725.85487
2.53E-10	1027.6653	1054.64544	1112.9302	1479.35702	1673.43769	1521.07929
1.40E-15	2376.05252	2425.68452	2482.69045	3386.77872	3524.71458	3444.72876
2.92E-10	523.996372	510.277947	512.664627	820.547153	797.84689	747.741168
4.15E-08	4024.8342	3869.16389	4312.35566	3050.36517	3206.93634	3032.07492
0.00020902	2989.26379	3015.85985	3048.11473	3593.80245	3711.29997	3445.50443
0.04198989	2413.3195	2580.15279	2348.30263	2768.9423	2637.46219	2784.63775
2.22E-11	5893.82988	6173.40439	6562.10723	9102.57433	9480.86998	8218.17186
1.84E-07	2351.20786	2420.35803	2775.35724	3560.37674	3720.04616	3236.07485
2.96E-07	785.994558	778.733151	850.127362	1086.87455	1306.09771	1105.32278
3.19E-25	906.829929	850.108146	731.666992	1836.2573	1663.7197	2081.88516
1.39E-15	4703.54502	4533.91011	5288.90711	2401.25954	3081.57428	2339.40598
0.00138083	44.0427985	47.9384293	83.6190848	99.1988674	123.418459	113.247106
0.00525329	89.2148996	71.3749947	102.532925	57.1471736	54.4207379	40.3345858
5.10E-11	3018.62565	3093.62664	2827.12144	4042.35385	4018.38842	4018.72095
0.05067563	54.2065213	46.8731309	47.7823342	72.2426534	56.3643357	101.61213
3.02E-16	25.9739581	28.7630576	23.8911671	135.859318	111.756873	121.779423
5.44E-05	2901.17819	2826.23673	2786.30736	3538.81177	3287.59565	3467.99872
1.14E-09	2066.62362	1958.01851	1718.1731	2689.15191	2730.75488	2924.25747
1.52E-14	110.671648	108.66044	150.31526	32.3474568	21.3795756	27.1482789
2.71E-38	958.777845	1025.88239	1013.38367	435.612418	447.999289	425.064482
1.43E-35	8232.61542	9027.33889	8526.16026	4954.55213	4821.0943	5082.15782
2.24E-05	2929.41075	2841.15091	2973.45484	2240.60051	2208.89888	2497.64166
4.14E-56	1244.49138	1194.19954	1273.20011	464.725129	463.548071	488.669021
0.00034131	3858.82673	3841.46613	3339.78607	3012.62647	2423.66643	3026.64527
1.54E-05	1063.80298	965.160376	890.941439	704.096309	616.120497	750.843829
1.36E-09	133.257698	139.554094	173.210961	328.86581	369.283579	263.726138
8.31E-49	3093.15962	3242.76842	2814.18039	6778.94869	6277.82084	6870.84156
1.18E-06	1511.00678	1564.92339	1653.46786	2144.63638	2199.18089	1940.71411
8.22E-33	1485.03282	1402.99803	1619.62204	2904.80162	2902.76329	2786.96475
7.10E-10	1997.73617	2013.41403	1611.65831	1200.09065	970.827092	1206.93491
9.29E-08	861.657828	896.981277	844.154571	604.897441	599.599916	640.699383
9.72E-64	8676.43131	9248.92096	9694.83651	19831.1475	20000.593	18939.415
0.00125059	674.193608	730.794722	593.297316	529.420042	466.463468	525.125281
0.06425343	828.908054	878.871204	870.036668	739.678511	757.031336	780.319103
8.15E-24	5540.35819	5250.85596	5371.53073	7995.21306	8557.66104	8177.06161
0.01303183	7.90511768	7.457089	5.97279177	16.1737284	17.49238	28.6996092
1.74E-47	2706.93816	2532.21437	2738.52503	1084.71805	1098.13275	891.239214
3.54E-41	103.895832	67.113801	95.5646684	441.003661	530.602195	427.391477
1.47E-10	937.321097	1001.38052	946.687496	1507.39149	1538.35764	1317.85503
1.40E-11	806.322004	800.03912	757.54909	531.576539	454.801881	465.399067
2.94E-13	195.369337	191.753717	226.966087	69.0079078	80.659308	83.7718321

0.0006242	6.77581516	6.39179057	9.95465295	0	0	0
1.05E-39	1687.17797	1578.77227	1494.19341	679.296592	544.207379	639.923718
6.19E-05	1307.73233	1214.44021	1214.46766	1491.21776	1572.37061	1694.05261
1.17E-10	2059.84781	2288.26103	2442.87184	3222.88494	3323.55221	3212.02923
0.00033445	1012.98437	1084.4738	945.692031	1263.70731	1287.63353	1244.94251
9.36E-29	918.122954	850.108146	840.172709	1583.94713	1641.36833	1596.3188
6.40E-16	3610.38018	3368.47363	3828.55953	5527.10211	5805.52658	5235.73951
3.49E-05	2013.5464	2064.54836	2023.78095	2564.07507	2590.81584	2374.31091
2.26E-13	749.856877	852.238743	848.136432	494.916088	457.717278	390.159551
1.34E-40	554.48754	584.848837	544.519517	193.006492	178.810996	166.767999
4.27E-13	4413.31427	5105.97537	5585.55577	8878.29863	8978.44996	7500.68164
8.21E-21	4740.81201	4139.74969	4619.95444	7431.28907	8203.92624	7205.15323
2.45E-05	1425.17979	1307.12117	1208.49487	1029.72737	900.857572	1007.58898
2.51E-09	614.340574	592.305926	618.183948	348.274284	388.719557	411.10251
2.26E-13	517.220557	535.84511	534.564864	921.902518	806.59308	908.303846
6.66E-37	2467.52602	2475.75355	2207.94203	1152.64771	1190.45364	1212.36457
0.02589973	2560.12883	2929.57068	2782.3255	3076.24314	3049.50492	3283.39042
1.26E-68	1149.62997	1220.832	1198.54022	2844.4197	2784.20382	2710.94957
1.20E-25	2498.01719	2670.70316	2970.46844	5217.64478	5180.65989	4691.2226
2.39E-22	2813.09259	2678.16025	2685.76537	1737.05843	1716.19684	1599.42146
1.03E-28	828.908054	955.572691	888.950509	1662.65928	1850.30509	1880.9879
7.33E-25	1558.43749	1672.51853	1630.57215	2744.14258	2643.29298	2613.21576
0.00111705	3751.54299	3593.2516	3873.35546	3101.04285	3350.76258	2970.02171
1.52E-09	396.385187	419.727581	487.777995	683.609586	720.102978	691.89328
7.94E-64	4959.8967	5091.06119	5088.81859	9516.62178	9798.64822	9480.95467
9.61E-15	196.49864	242.888042	185.156545	69.0079078	66.0823246	76.7908461
0.07362575	5.64651263	7.457089	11.9455835	3.23474568	2.91539667	0.77566511
6.58E-08	100.507925	67.113801	80.6326889	204.867226	179.782795	165.216669
3.05E-11	832.295962	788.320837	877.004925	518.637557	512.138016	554.600555
3.20E-12	92.6028072	107.595141	121.446766	28.0344625	23.3231734	31.0266045
7.08E-06	4246.1775	4565.86907	4470.63464	3556.06375	3601.48669	3689.83894
9.46E-07	575.944288	607.220104	489.768925	347.196036	252.667712	370.767924
3.33E-06	5530.19447	5958.21411	5674.15218	4672.05101	4573.28558	4743.96783
9.21E-07	1328.05977	1291.1417	1131.84404	865.833593	794.931493	951.741093
2.44E-07	4988.12926	4620.19929	5050.99091	6072.69588	6749.1433	6230.91785
3.56E-09	43726.5938	46375.6365	46561.8937	57400.562	57641.2794	56539.0057
0.01395525	1023.14809	1057.84134	1041.2567	1139.70873	1257.50777	1229.4292
6.93E-41	7609.24042	7576.40243	7411.23912	12706.081	12178.5837	12692.2082
6.19E-12	64.370244	80.9626806	76.6508278	9.70423703	13.6051845	15.5133022
2.96E-05	607.564759	593.371225	566.419753	791.434442	822.141862	742.311512
8.86E-10	4347.81473	4432.70676	4409.91126	6008.00097	5521.7613	5730.61385
0.00690498	1753.80682	1601.14354	1410.57432	1314.38499	1094.24555	1384.56223
5.01E-13	232.63632	221.582073	238.911671	98.1206188	105.926079	94.6311437

0.00258149	3713.14671	3538.92138	2730.56131	2569.46632	2009.68011	2810.2347
0.00013156	222.472598	219.451476	224.975157	331.022308	316.806439	308.714715
2.47E-17	84.6976895	86.2891727	81.6281542	4.31299423	9.71798891	10.0836465
4.77E-14	11191.388	11395.4973	12240.2413	16028.1648	17385.4822	16077.9864
0.05116956	3861.08534	3983.15083	3812.63208	4200.85638	4153.46846	4466.27972
1.10E-06	40.6548909	40.4813403	50.7687301	107.824856	99.1234869	104.71479
6.94E-05	2013.5464	1972.93269	1973.01222	1665.89402	1597.63738	1639.75605
4.42E-64	1688.30728	1636.29839	1622.60843	3562.53324	3814.31065	3934.94911
3.01E-10	7071.69242	7858.70651	8370.86767	11089.7864	10986.1865	10538.9619
7.87E-17	3680.39693	3465.41579	3301.95839	2350.58186	2276.9248	2148.59236
8.13E-20	353.471691	334.503707	346.421923	114.294347	98.151688	144.273711
0.00222737	9.03442021	3.19589529	6.96825707	0	0	0
0.00605909	10431.3674	10593.3276	10317.0023	9533.87376	9023.15271	9422.77978
2.35E-11	539.806608	533.714513	552.483239	304.066094	250.724114	331.984668
5.34E-25	1291.92209	1600.07824	1690.30007	3350.11827	3271.07507	3011.13197
2.70E-16	1744.7724	1946.30023	2025.77188	3491.36883	3312.86242	2918.05215
2.74E-26	691.133146	705.22756	589.315455	273.875134	234.203533	275.361115
0.00084637	151.326539	167.251853	153.301656	105.668359	102.038884	96.1824739
0.00150654	2682.0935	2656.85428	2684.7699	2283.73045	2383.82268	2212.97257
7.92E-34	4692.252	5150.7179	5363.56701	2667.58693	2699.65732	2416.97249
0.08476693	1457.92956	1652.27786	1766.9509	1903.10871	1912.50022	1715.77123
0.00069101	1304.34442	1279.42341	1334.91896	1101.97003	1046.62741	1092.13648
2.03E-47	1172.21602	1175.02417	1218.44952	489.524846	490.75844	436.699458
2.35E-07	306.040985	315.328335	247.870859	154.189544	111.756873	170.646325
3.95E-14	1316.76675	1408.32452	1485.23422	863.677096	931.955137	868.744926
1.92E-09	7451.13807	7851.24942	8114.03762	5993.98374	6136.91	5866.35524
4.71E-10	2764.53258	2850.7386	2354.27542	1768.32764	1438.26236	1771.61912
4.78E-36	3066.05636	3071.25537	3009.29159	5467.79844	5049.46704	5313.30602
5.71E-12	1977.40872	1958.01851	1925.22988	1302.52426	1441.17776	1309.32271
7.56E-36	1178.99184	1176.08947	1270.21372	544.515522	581.135737	562.357206
2.45E-19	2027.09803	2009.15284	2166.13248	3110.74709	3279.82126	3152.30302
0.00152745	648.21965	687.117487	741.621645	516.48106	594.740921	490.220351
1.83E-09	1505.36027	1530.83384	1544.96214	1109.51777	1071.89418	1145.65737
3.45E-08	276.679119	258.867518	268.77563	434.534169	409.127333	436.699458
4.23E-05	5830.58894	5986.97717	6264.4631	5001.99506	5157.33672	4704.40891
3.11E-19	2180.68318	2541.80205	2727.57491	4506.00073	4225.38158	4310.37103
3.37E-170	1360.80954	1276.22752	1398.62874	5096.88094	4934.79477	4885.91454
1.55E-06	5352.89397	4997.31493	5190.35605	6501.83881	7230.18375	6298.40071
1.88E-23	1394.68862	1492.4831	1396.63781	2371.06858	2495.57955	2306.05238
0.00048572	1942.40035	1954.82262	2127.30934	1606.59035	1746.32261	1485.39869
0.00022753	59.8530339	79.8973822	70.678036	36.660451	29.1539667	30.2509394
2.04E-05	2627.88698	2614.24234	2169.11888	3427.75217	3030.06894	3931.07079
0.00025374	3628.44902	3901.12285	3711.09462	3037.42619	2941.63524	3291.92274

3.86E-14	970.07087	923.613738	1113.92567	543.437274	604.45891	493.323011
0.00021406	845.847592	933.201424	934.741912	677.140095	752.172342	667.071997
6.64E-19	2477.68974	2413.96624	2088.48619	1352.12369	1201.14343	1356.63828
0.05327886	11117.9834	9650.53847	12050.1074	8692.83988	10939.5401	7806.29369
0.0002423	203.274455	205.602597	208.052247	128.311578	91.3490958	146.600706
1.84E-05	16.9395379	22.371267	24.8866324	3.23474568	0.97179889	0
0.00564231	130.999093	138.488796	139.365141	88.4163818	101.067085	86.0988275
0.00011616	109.542345	120.378722	128.415023	53.9124279	77.7439113	55.8478881
1.39E-07	71.1460592	64.9832042	69.6825707	22.6432197	22.3513745	10.8593116
0.00015638	127.611185	142.749989	177.192823	87.3381333	88.4336991	78.3421763
2.60E-10	298.135867	350.483183	372.304021	178.989261	181.726393	175.300315
0.04116949	39.5255884	30.8936544	44.7959383	22.6432197	23.3231734	18.6159627
0.00222261	65.4995465	78.8320837	117.464905	50.6776823	43.7309501	41.8859161
0.0085867	12.4223278	22.371267	28.8684936	9.70423703	5.83079335	3.87832556
0.00018842	67.7581516	96.942157	82.6236195	45.2864395	37.9001568	20.942958
0.0393058	30.4911682	25.5671623	15.9274447	5.39124279	16.5205812	7.75665112
1.13E-08	188.593522	249.279832	276.739352	101.355365	124.390258	95.4068088
3.91E-05	320.721917	373.919749	427.054612	219.962706	270.160092	176.851646
2.13E-05	90.3442021	113.986932	106.514787	56.0689251	46.6463468	45.7642416
0.01824601	13.5516303	23.4365654	23.8911671	8.62598847	5.83079335	7.75665112
4.19E-20	2426.87113	2260.56327	2322.42053	1424.36635	1180.73565	1368.27326
3.27E-11	1214.00022	1235.74618	1310.03233	1923.59543	2005.79291	1718.87389
0.03742004	4723.87247	5086.8	5421.304	4631.07756	4581.05997	4365.44325
1.39E-22	2997.1689	3080.84306	3026.2145	1875.07424	1987.32873	1968.63805
4.32E-17	1088.64764	1091.93089	951.664822	1992.60334	1669.5505	1996.562
5.34E-06	4806.31155	4708.61906	4532.35349	5589.64053	5677.24912	6042.43122
3.29E-07	815.356424	859.695832	929.764586	608.132187	628.753883	611.999774
0.00016941	2486.72416	2565.23862	2457.80381	2101.50644	2066.04444	2123.77108
0.00110167	11314.482	11101.4749	10278.1792	9145.70428	8824.90573	9750.88613
8.79E-09	389.609372	365.397361	419.090889	753.695743	742.454353	560.030211
2.03E-08	7009.58078	7653.10391	7704.90139	9840.09635	9602.34485	9414.24747
0.01219518	103.895832	84.1585759	76.6508278	52.8341794	63.1669279	48.091237
5.74E-12	242.800043	233.300356	211.038643	103.511862	88.4336991	105.490455
6.70E-06	1168.82811	1149.457	1169.67172	1623.84233	1438.26236	1477.64204
2.23E-10	70.0167566	53.2649214	82.6236195	180.067509	164.234013	188.486622
1.25E-10	4342.16821	4742.70861	5250.08397	3231.51093	3411.01411	3151.52735
6.31E-08	220.213993	254.606324	265.789234	109.981353	132.164649	141.946716
3.15E-05	808.580609	1021.62119	931.755517	1380.15816	1261.39496	1178.23531
9.95E-23	2669.67117	2974.31321	2786.30736	1713.33696	1701.61986	1735.93852
0.04003241	2166.00225	2185.99238	2080.52247	2554.37084	2327.45834	2330.098
0.00241083	126.481883	155.533571	158.278982	211.336718	234.203533	197.794604
1.29E-11	3096.54753	3305.62102	3049.1102	4468.26203	4164.15825	4502.73598
0.00137584	486.729389	462.339518	405.154375	657.731621	515.053412	680.258303

3.34E-30	2940.70378	2835.82442	2775.35724	1511.70448	1411.05199	1635.87772
0.00069047	954.260635	859.695832	755.558159	665.279361	621.95129	672.501652
0.00043345	48.5600086	63.9179057	45.7914036	88.4163818	92.3208947	135.741395
2.07E-13	147.938631	157.664167	125.428627	44.2081909	47.6181457	38.7832556
1.23E-05	13.5516303	31.9589529	28.8684936	1.07824856	3.88719557	1.55133022
0.00534435	2336.52693	2279.73864	2370.20287	2015.24656	2108.80359	1869.35292
0.01666964	128.740488	133.162304	111.492113	100.277116	71.913118	82.2205019
0.0170352	0	5.32649214	0	10.7824856	8.74619002	15.5133022
2.02E-13	1350.64582	1398.73684	1456.36573	833.486136	963.052701	871.071921
5.04E-19	3278.36523	3342.90647	3695.16718	1958.09938	2191.4065	1860.8206
1.62E-14	82.4390844	82.027979	111.492113	18.3302255	9.71798891	14.7376371
0.0176934	470.919153	469.796607	411.127167	343.96129	354.706595	390.159551
3.63E-50	232.63632	230.104461	270.76656	10.7824856	14.5769834	14.7376371
2.30E-27	874.080155	947.050303	1061.166	405.421458	446.055691	382.4029
1.44E-16	207.791665	224.777968	202.079455	67.9296592	77.7439113	75.2395159
1.96E-12	7463.5604	7442.17482	6850.79216	5078.55071	4960.06154	5427.32879
9.26E-16	2145.6748	2242.45319	2134.27759	3096.72986	3182.64137	3395.86186
0.02574728	12479.9222	12231.7566	11981.4203	11136.1511	11008.5378	11365.8209
0.00027242	1505.36027	1535.09504	1355.82373	1160.19545	1144.77909	1215.46723
3.67E-05	477.694969	463.404817	498.728113	640.479644	739.538956	619.756425
2.01E-26	1564.084	1412.58572	1452.38387	820.547153	787.157102	759.376145
1.30E-05	1051.38065	1001.38052	988.497038	782.808454	800.762286	711.284908
4.26E-11	7654.41252	7257.8782	6262.47217	4610.59084	4568.42659	4981.32135
1.54E-14	198.757245	175.774241	182.170149	43.1299423	49.5617435	71.3611903
1.51E-09	7751.53254	8285.89118	8506.25095	10547.4274	11090.1689	10545.1672
2.71E-15	1514.39469	1721.52226	1719.16857	2572.70106	2569.43627	2486.00668
2.98E-09	1490.67933	1401.93273	1299.08221	2027.10729	1878.48726	2163.33
0.00051903	609.823364	727.598827	689.85745	524.0288	505.335423	524.349616
3.13E-29	2027.09803	1985.71627	1969.03035	1085.7963	1170.04587	1067.31519
0.00024883	1654.4282	1798.22375	1940.16186	2303.13892	2396.45607	2113.68743
1.53E-15	14049.6527	13809.4635	14677.1403	19409.5523	21004.4612	19571.5821
1.26E-06	308.29959	341.960796	395.199722	207.023723	215.739354	215.634901
1.06E-38	8031.59957	7214.20096	7499.83554	3902.18153	4163.18645	4169.97564
0.0006018	108.413043	101.203351	101.53746	42.0516938	68.9977213	55.8478881
2.99E-15	476.565666	503.886157	561.442427	244.762423	230.316337	276.912445
1.42E-09	2187.45899	2191.31887	1893.37499	3262.78014	2801.6962	3353.20028
2.25E-19	3047.98752	3149.02216	3505.03331	1945.1604	1990.24413	1756.10581
1.65E-16	1137.20764	1165.43648	1250.30441	700.861563	664.710442	743.087178
7.39E-29	2816.4805	2766.58002	2733.5477	4342.10695	4378.9258	4440.68277
1.76E-06	2615.46465	2467.23116	2246.76517	3377.07449	3037.84333	3764.30279
1.44E-07	5243.35163	5112.36716	4439.77522	3398.63946	2838.62456	3711.55756
0.00025394	326.36843	264.19401	307.598776	444.238406	490.75844	378.524575
5.14E-14	125.35258	103.333948	135.38328	280.344625	326.524427	276.13678

3.41E-56	653.866163	692.443979	734.653388	2113.36718	2177.80132	1813.50503
7.85E-07	5.64651263	8.52238743	2.98639589	45.2864395	39.8437545	30.2509394
5.64E-41	5753.79637	5719.58726	5765.73499	3402.95245	3445.99887	3403.61851
0.00116541	4161.47981	3862.7721	3479.15121	4650.48603	4419.74136	5174.46196
0.00071187	1002.82064	972.617465	686.871054	499.229083	483.955848	733.003531
8.13E-10	963.295055	931.070827	944.696565	1451.32256	1286.66173	1348.10597
1.26E-05	229.248413	214.124984	209.047712	118.607341	147.713431	117.901097
2.59E-07	2003.38268	2115.68268	2035.72653	3076.24314	2550.00029	2787.74041
1.29E-61	3098.80613	2950.87665	2454.81742	7922.97041	8432.29898	9093.12211
1.12E-06	2125.34735	2270.15095	1987.9442	2682.68241	2768.65504	2956.05974
2.06E-06	3246.74476	3119.1938	2685.76537	2063.76774	2201.12449	2353.36795
1.37E-06	1608.1268	1817.39912	1850.56998	1348.88895	1289.57713	1324.06035
2.06E-12	1987.57245	2058.15656	2071.56328	1385.5494	1353.71586	1487.72569
3.48E-32	8248.42565	8112.24754	7710.87418	4532.95694	4041.71159	4623.73973
0.00863363	18152.4088	17667.9744	16642.1888	15866.4275	14814.1023	15703.3402
0.00313222	38640.2152	35980.4544	31535.3451	29567.732	27344.4772	30993.2509
5.46E-10	3741.37927	3616.68817	2868.93098	2100.42819	1889.17704	2282.00676
0.00039199	1570.85981	1540.42153	1503.1526	1916.04769	1774.50478	1875.55824
3.42E-10	528.513582	488.971979	566.419753	319.161573	315.83464	320.349691
3.83E-10	368.152624	418.662283	331.489943	198.397735	186.585387	207.87825
1.18E-31	4304.90123	4096.07246	4387.01556	6957.93795	6826.88721	6969.35103
9.26E-34	280.067027	281.238785	225.970622	52.8341794	43.7309501	48.8669021
8.25E-09	496.893112	575.261152	491.759856	766.634725	787.157102	839.269651
1.15E-05	43885.8255	43762.4595	43516.7654	36559.0956	37201.4334	38124.7159
3.19E-06	3672.49182	3903.25344	3941.0471	3008.31348	3138.91042	3131.36006
2.94E-29	669.676398	711.61935	738.635249	1424.36635	1472.27532	1327.93867
9.78E-11	3412.75223	3455.8281	3632.45286	4745.37191	4960.06154	4543.07056
4.14E-32	1850.92684	1695.9551	1777.90102	949.93698	951.391115	930.02247
0.05118866	3592.31134	3797.7889	3796.70464	3198.08523	3411.98591	3495.92266
1.52E-07	1367.58536	1456.26295	1484.23876	1902.03046	1982.46974	1837.55065
1.10E-54	5594.56471	5455.39325	5398.4083	9759.22771	9815.1688	9939.37275
0.00018959	1452.28305	1376.36557	1502.15713	1180.68217	1173.93306	1164.27333
4.90E-32	6214.5518	6139.31485	6144.0118	3898.94679	3717.13076	3893.0632
4.89E-12	2102.7613	2188.12297	2060.61316	2980.27902	2840.56816	3080.94183
0.00196474	0	0	0	5.39124279	9.71798891	5.42965579
3.92E-06	6072.25968	5901.7533	6624.82154	8605.50175	8989.13974	7353.30526
1.66E-16	1045.73414	985.401047	982.524247	1593.65137	1559.73722	1762.31114
1.13E-16	1934.49523	1900.4924	1845.59266	1200.09065	1272.08475	1129.3684
5.91E-106	24895.4742	24447.5336	22713.5316	68270.3857	63195.1101	70780.2172
0.00099913	3175.5987	3029.70873	2950.55914	3594.88069	3445.99887	3761.20013
0.00049405	1845.28033	1725.78345	1484.23876	1289.58528	1118.54052	1401.62686
2.15E-41	3329.18385	3343.97177	3327.84048	1865.37001	1860.02308	1890.29588
0.02279949	912.476441	977.943958	1102.97555	906.807038	829.916253	751.619494

1.18E-11	649.348953	726.533528	763.521882	1152.64771	1126.31492	1075.84751
3.01E-11	1918.68499	1984.65097	2062.60409	1450.24431	1419.79818	1358.18961
1.39E-25	1417.27467	1345.47192	1343.87815	609.210436	714.272185	729.900871
1.89E-74	1370.97327	1372.10438	1310.03233	436.690666	399.409344	449.885765
3.60E-18	178.429799	172.578345	182.170149	51.7559308	53.448939	51.9695625
2.47E-36	2639.18	2761.25353	2950.55914	1491.21776	1440.20596	1417.91583
4.15E-31	1940.14174	1983.58567	2019.79908	1004.92766	1125.34312	1046.37224
2.89E-06	9753.78592	9295.79409	11431.9235	14243.6635	16926.7931	12833.3793
2.24E-28	150.197236	189.62312	147.328864	452.864395	525.7432	497.201337
0.00033778	867.30434	746.774199	707.775825	574.706482	482.01225	618.205094
3.30E-21	479.953574	434.641759	437.009265	198.397735	188.528985	200.897264
2.87E-08	734.046642	745.7089	728.680596	980.12794	1044.68381	1094.46347
2.88E-10	485.600086	545.432796	511.669162	269.56214	273.075488	330.433338
1.51E-13	2405.41438	2638.74421	2355.27089	1712.25871	1648.17092	1633.55073
0.00123823	171.653984	165.121256	145.337933	225.353949	261.413902	217.961897
0.00099073	261.998186	237.56155	302.62145	377.386996	412.04273	342.068315
9.43E-11	1551.66167	1513.78907	1462.33852	1053.44884	917.378153	1061.10987
7.82E-25	3129.2973	2978.57441	2976.44123	1803.90984	1776.44837	1923.64948
1.02E-11	1372.10257	1296.46819	1310.03233	866.911841	935.842332	899.77153
0.00016513	796.158281	841.585759	756.553625	994.145171	998.037461	1044.04524
6.05E-13	1831.7287	1744.95883	1702.24566	1159.1172	1217.66401	1223.99955
9.81E-10	196.49864	171.513047	161.265378	79.7903933	79.6875091	69.034195
6.96E-05	5219.63628	5205.04812	4404.93393	3779.2612	3261.35708	4070.69051
4.85E-05	195.369337	174.708942	197.102129	112.13785	112.728671	119.452427
7.40E-11	2287.96692	2487.47183	2893.81761	4277.41203	4720.02722	3710.00623
6.21E-10	330.88564	310.001843	315.562499	187.615249	160.346817	171.42199
1.35E-13	2467.52602	2558.84683	2627.03291	1732.74543	1846.41789	1739.04118
0.00021072	901.183416	934.266722	927.773655	1166.66494	1128.25851	1125.49008
0.00551221	23.7153531	35.1548482	28.8684936	6.46949135	8.74619002	15.5133022
3.96E-15	412.195422	474.057801	509.678231	925.137263	921.265349	795.05674
1.72E-24	4072.26491	4365.59296	4145.11749	2711.79513	2563.60548	2511.60363
6.83E-19	1230.93975	1270.90103	1291.11849	696.548569	754.11594	778.767773
0.0247734	4577.06314	4636.17876	3755.89056	5753.53431	4284.66131	6005.1993
2.99E-32	2688.86931	2390.52967	2646.94222	1363.98443	1372.18003	1268.98812
3.71E-05	1166.56951	1101.51858	1052.20682	804.373425	878.506198	870.296256
0.08138792	18.0688404	22.371267	21.9002365	29.1127111	35.956559	39.5589207
2.32E-05	449.462405	425.054073	455.923105	588.723713	615.148698	603.467457
0.05052195	948.614122	976.878659	1124.87578	1263.70731	1215.72041	1079.72584
3.59E-41	3429.69177	3551.70496	3897.24663	7506.76647	7891.9788	6994.17232
0.00035112	138.904211	155.533571	169.2291	97.0423703	102.038884	79.8935066
0.05617823	1073.9667	1070.62492	1032.29751	1213.02963	1197.25623	1153.41402
1.10E-35	1574.24772	1502.07078	1330.9371	3329.63155	2997.99958	3543.23823
8.50E-05	3055.89264	2966.85612	3103.86079	3686.53182	4025.19101	3530.82759

0.01183642	4306.03053	4265.45491	4373.07904	4870.44874	4983.38471	4660.19599
0.00045829	1294.1807	1191.00364	1355.82373	1091.18754	999.00926	923.041484
0.00046485	3156.40056	3401.49788	3572.72495	2955.4793	2702.57272	2593.82414
0.02312381	5389.03166	5275.35782	5514.87774	6170.8165	6311.8338	5669.33631
2.23E-09	398.643792	437.837654	445.968452	689.000829	680.259224	631.391401
0.03941879	1874.64219	1826.98681	1679.34995	2154.34062	1842.5307	2228.48587
5.73E-11	1850.92684	1937.77784	1824.68789	1376.92341	1211.83322	1293.03374
6.56E-31	1642.00587	1764.1342	1629.57669	839.955627	685.118218	800.486396
3.81E-16	1625.06634	1724.71816	1502.15713	990.910425	901.829371	1012.24297
1.57E-13	676.452213	699.901068	719.721409	422.673435	387.747758	428.167142
4.02E-06	2499.14649	2438.4681	2448.84463	3009.39173	3273.99046	2993.29167
0.00492994	3515.51876	3633.73294	3200.42092	2732.28185	2829.87837	3139.11671
6.66E-06	1516.65329	1448.80586	1766.9509	2255.69598	2337.17633	1973.29205
9.50E-41	379.445649	362.201466	349.408319	63.616665	83.5747047	86.0988275
6.15E-07	4090.33375	3981.02023	4363.12439	3395.40471	3072.82809	3153.85435
8.40E-09	0	0	0	46.364688	39.8437545	25.5969487
4.40E-07	502.539624	474.057801	494.746252	312.692082	288.624271	350.600631
4.73E-05	50.8186137	28.7630576	31.8548895	8.62598847	8.74619002	10.0836465
3.97E-05	439.298683	455.947728	429.045542	586.567216	585.022933	611.999774
9.82E-08	9.03442021	10.6529843	24.8866324	77.6338962	80.659308	54.2965579
1.77E-05	2.25860505	1.06529843	0	22.6432197	28.1821678	18.6159627
1.49E-19	627.892205	565.673466	631.124997	304.066094	294.455064	307.93905
3.51E-09	8022.56515	8027.02366	7407.25726	5906.6456	5166.08291	5929.18412
3.76E-76	613.211272	630.65667	644.066046	1856.74402	1721.05584	1763.86247
7.12E-40	1110.10438	1196.33014	1278.17744	500.307331	508.25082	509.611979
6.51E-23	2205.52783	2351.11363	2485.67684	1265.86381	1418.82638	1269.76379
0.06410284	988.13971	986.466345	1032.29751	900.337547	900.857572	878.052907
2.73E-08	461.884733	488.971979	582.347198	947.780483	869.760008	736.881857
6.85E-12	129.869791	120.378722	132.396884	28.0344625	38.8719557	45.7642416
1.09E-08	535.289397	532.649214	573.38801	847.503367	775.495515	796.60807
9.89E-11	1011.85506	1132.41223	1008.40634	1586.10363	1472.27532	1621.91575
1.11E-10	1517.7826	1531.89914	1510.12085	1126.76974	1042.74021	1051.80189
1.07E-36	514.961952	508.147351	482.800668	146.641804	172.980203	164.441004
0.00065588	191.981429	197.080209	246.875393	138.015816	151.600627	103.939125
0.00013475	1076.22531	1120.69395	953.655753	754.773991	682.202822	865.642265
9.28E-10	3593.44064	3591.121	3952.99269	5033.26427	5261.3192	4847.90695
0.00022873	286.842842	262.063413	364.340298	177.911012	216.711153	190.813618
0.00148204	24.8446556	25.5671623	23.8911671	6.46949135	9.71798891	5.42965579
4.28E-55	2919.24703	3240.63782	2739.52049	6782.18343	6726.79193	7148.52967
0.00016801	7384.50922	7448.56661	6994.13917	6238.74616	5765.68282	6267.37411
1.43E-59	4486.71894	4361.33177	4276.51891	2197.47056	2074.79063	2064.82053
1.42E-25	595.142431	610.416	554.47417	250.153666	267.244695	252.866827
0.03847314	16966.6412	15453.219	16185.2702	17459.0007	18380.6042	17709.2102

3.12E-70	3041.2117	3108.54082	3042.14194	6085.63487	6271.01825	6216.95587
0.00385184	1153.01788	1309.25177	1394.64688	1603.35561	1639.42473	1476.09071
9.92E-05	1652.1696	1601.14354	1639.53134	1960.25588	1937.76699	2064.82053
0.03136705	32.7497733	22.371267	27.8730283	8.62598847	10.6897878	17.8402976
0.0162638	2.25860505	0	0	7.54773991	6.80259224	10.0836465
0.00394091	8258.58937	9001.77172	9125.43036	8124.60289	7099.9627	7134.5677
2.14E-17	490.117296	541.171602	508.682766	270.640388	249.752315	231.923869
3.68E-52	12106.1231	12914.6129	13092.3596	6617.2114	5971.70419	6094.40079
1.56E-21	1626.19564	1636.29839	1653.46786	996.301668	826.029058	843.923642
1.41E-45	2551.09441	2705.85801	2799.24841	1236.7511	1328.44908	1203.83225
3.01E-73	6710.31561	7110.86701	6745.27284	15694.986	15263.0734	17153.0583
0.00016468	2932.79866	2816.64905	2743.50235	2330.09514	2299.27618	2432.48579
2.32E-05	839.071777	752.100691	591.306386	456.09914	494.645636	497.977002
0.00948032	3409.36433	3268.33558	3408.47317	3109.66884	2911.50948	2808.68337
1.89E-14	1631.84215	1656.53906	1565.86691	2334.40813	2310.93776	2333.97632
0.00035606	4341.03891	4067.3094	4039.59817	3453.63013	3501.39141	3587.45114
2.76E-05	145.680026	178.970136	207.056781	280.344625	294.455064	286.996092
7.04E-06	2171.64876	2014.47933	1840.61533	1447.00957	1298.32332	1605.62678
1.75E-05	1651.04029	1707.67338	1423.51537	1239.98584	971.798891	1175.13265
8.28E-19	1578.76493	1746.02412	1790.84207	1026.49263	1043.71201	997.505334
8.87E-30	903.442021	907.634261	1084.06171	1966.72537	2223.47586	2040.77491
1.65E-25	2159.22643	2130.59686	2018.80362	3312.37957	3445.02707	3473.42837
0.07707879	7674.73997	8324.24192	7630.24149	7471.18426	6488.7012	7316.849
1.23E-31	1470.35189	1465.85064	1552.92586	2648.17846	2747.27547	2577.53517
1.50E-47	1344.99931	1478.63422	1374.73757	2889.70614	2947.46604	3152.30302
0.01382559	2082.43386	1963.345	1771.92823	1654.03329	1477.13431	1750.67616
1.36E-10	1146.24206	1312.44766	1424.51084	1985.0556	2030.08788	1905.80918
1.45E-11	390.738674	384.572733	459.904967	663.122864	776.467314	712.836238
7.60E-11	90.3442021	101.203351	105.519321	25.8779654	24.2949723	32.5779347
3.91E-22	24234.8322	24698.9441	26387.7941	15740.2725	16786.854	14962.58
2.91E-07	529.642885	486.841382	511.669162	347.196036	304.173053	349.824966
0.06198981	2277.8032	2322.35057	2623.05105	2708.56038	3096.15127	2505.39831
7.15E-11	1705.24681	1659.73495	1734.10054	1213.02963	1173.93306	1246.49384
1.22E-07	3760.57741	3949.06128	3661.32136	3034.19144	2564.57727	2804.02938
0.01701878	1465.83468	1591.55585	1701.25019	1374.76691	1417.85458	1279.84744
2.24E-18	2863.91121	3102.14902	2981.41856	4476.88802	4312.84348	4569.44318
9.30E-06	3568.59598	3660.3654	3995.7977	3047.13043	3006.74577	2773.77844
5.10E-06	1116.8802	1146.26111	1149.76242	860.44235	912.519159	825.307679
2.31E-11	4222.46215	4496.62467	4241.67762	5700.70013	5704.45949	5995.11565
1.06E-33	4044.03235	3976.75903	4032.62991	2461.64146	2314.82496	2355.69495
0.00840462	621.116389	592.305926	488.77346	720.270037	645.274464	822.980684
1.10E-07	1007.33785	1180.35066	1335.91443	1823.31831	2002.87751	1638.20472
0.00125875	2516.08603	2706.92331	3100.8744	3685.45357	3760.86171	3129.03306

4.15E-14	1700.7296	1741.76293	1845.59266	2747.37733	2854.17334	2495.31467
8.80E-06	1562.9547	1706.60808	1350.84641	2150.02763	1961.09016	2387.49722
7.09E-05	1225.29324	1296.46819	1201.52661	1533.26945	1524.75246	1557.53555
0.01772388	1236.58627	1281.55401	1368.76478	1478.27877	1623.87595	1431.10213
2.26E-06	8479.93267	8207.0591	7865.1713	6450.08288	5948.38101	6821.97466
0.00227865	2149.06271	2056.02597	1563.87598	1435.14883	1279.85914	1614.93476
0.01345457	2056.4599	1975.06329	1631.56762	2242.757	2141.84476	2427.8318
2.39E-06	1008.46716	990.727539	832.208987	644.792638	559.756161	697.322936
0.00645944	1339.3528	1486.09131	1562.88051	1699.31973	1780.33557	1713.44423
5.04E-12	1305.47372	1295.40289	1271.20918	1961.33413	1939.71059	1762.31114
3.35E-74	495.763809	528.388021	524.610211	1610.90335	1479.07791	1624.24275
2.88E-19	1120.26811	997.119329	1188.58556	1834.1008	1942.62598	1982.60003
0.00014242	605.306154	646.636146	656.01163	788.199696	917.378153	810.570042
4.77E-14	293.618657	329.177215	334.476339	568.23699	563.643357	619.756425
1.42E-22	2130.99387	2027.26291	1894.37046	3467.64736	3253.58269	3674.32564
0.00141316	1579.89423	1673.58383	1501.16167	2154.34062	1744.37901	2059.39087
3.85E-05	1332.57698	1234.68088	1370.75571	1054.52709	964.996299	1040.16692
1.79E-09	2289.09622	2285.06513	2180.069	1614.13809	1476.16252	1708.79024
0.00085415	350.083783	345.156691	351.399249	281.422874	228.372739	250.539831
6.28E-13	1203.83649	1097.25738	1180.62184	718.11354	755.087739	771.011122
7.87E-07	522.86707	445.294743	379.272278	257.701406	260.442103	287.771757
2.62E-16	3050.24612	3078.71246	2976.44123	2105.81944	2145.73195	2095.07147
3.86E-07	1508.74818	1512.72377	1556.90772	1892.32622	2136.98576	2017.50496
1.30E-05	971.200173	875.675308	943.7011	1147.25647	1343.02607	1244.16684
1.80E-08	552.228935	586.979434	648.047907	349.352533	346.932204	407.999849
0.06932382	2643.69721	2798.53897	2430.92625	2221.19203	1925.1336	2596.9268
0.00045763	1469.22259	1538.29093	1331.93257	1135.39573	1108.82253	1217.01856
1.52E-95	997.174131	1009.90291	950.669357	189.771746	217.682952	176.851646
5.72E-09	144.550723	162.99066	167.23817	70.0861563	71.913118	69.034195
4.10E-59	1461.31747	1576.64167	1505.14353	3288.6581	3185.55677	3239.95317
0.00089132	1038.95832	1069.55962	942.705635	1227.04686	1220.57941	1327.16301
1.42E-19	1193.67277	1216.57081	1212.47673	720.270037	615.148698	698.098601
1.39E-05	1742.5138	1760.9383	1924.23442	1467.49629	1398.4186	1399.29986
0.00151666	1602.48028	1600.07824	1339.89629	1181.76042	1099.10455	1296.1364
0.00222332	1696.21239	1528.70325	1470.30224	1834.1008	1855.16408	1953.90042
0.02504205	3324.66664	2914.6565	3380.60014	2726.89061	2938.71985	2721.03321
3.95E-06	2895.53168	2872.04456	2934.63169	3409.42194	3844.43641	3751.11648
1.21E-13	1167.69881	1255.98685	1361.79652	1871.8395	1998.99032	1994.235
0.02462059	18.0688404	5.32649214	24.8866324	30.1909596	35.956559	44.9885765
0.01177457	8579.31129	8093.07216	7508.79472	9253.52913	8816.15954	9652.37666
6.60E-09	720.495012	725.46823	712.753152	497.072586	474.237859	494.874342
0.01177568	56.4651263	59.656712	61.7188483	36.660451	32.0693634	34.1292649
0.00432886	12461.8534	12214.7118	10285.1474	9679.43731	8260.29058	10320.2243

0.07120394	10561.2372	11528.6596	10821.7032	11973.9502	11576.0684	12397.4555
0.0012487	11428.5416	11944.126	11983.4112	10464.4023	10391.4455	10219.3879
8.63E-33	3435.33828	3501.63594	3706.1173	6143.86029	5913.39625	5883.41988
0.00023626	2754.36886	2955.13784	2616.0828	3390.01347	3259.41348	3452.48541
0.00461294	3892.70581	3675.27958	3564.76122	3408.34369	2961.07122	3091.80114
3.90E-195	5840.75267	5875.12083	5407.36748	32977.1539	28918.7914	35636.3823
2.61E-50	1905.13336	2091.18082	2192.01458	4882.30947	4891.06382	4415.86148
1.27E-37	7757.17905	7662.6916	7760.64744	12533.5612	12176.6401	12254.7331
5.44E-07	1540.36865	1649.08197	1547.94853	2171.5926	1952.34397	2132.30339
6.89E-07	2309.42367	2233.93081	2336.35705	3042.81743	2917.34027	2802.47805
1.55E-07	564.651263	612.546597	623.161275	389.24773	425.647914	397.140537
0.0026537	634.66802	519.865633	575.378941	459.333886	451.886484	438.250788
0.0188045	65.4995465	68.1790994	53.755126	32.3474568	33.0411623	44.2129114
9.46E-05	1373.23187	1260.24804	1191.57196	1596.88612	1559.73722	1766.18946
4.98E-10	1768.48776	1621.38421	1784.86927	2285.88694	2550.00029	2492.21201
1.04E-22	4699.02781	4131.22731	4245.65949	7858.2755	7021.24699	7259.44979
1.05E-12	10708.0466	10651.919	10167.6825	13840.3985	13734.4337	13799.0823
9.63E-05	3767.35323	3691.25906	3480.14667	4455.32304	4225.38158	4495.75499
2.48E-05	13403.6917	13572.9673	12698.1553	10877.3715	9926.92567	11290.5814
4.42E-23	2667.41257	2590.80578	2869.92645	4655.87728	5162.19571	4428.27213
6.42E-74	65.4995465	72.4402932	52.7596607	502.463828	555.868966	490.996016
7.09E-21	2180.68318	2155.09872	1857.53824	1164.50844	1071.89418	1180.5623
1.26E-06	8302.63217	8387.09453	7748.70186	6170.8165	5866.74991	6790.94806
0.00082318	1147.37137	1353.9943	1237.36336	1043.7446	994.150266	942.433111
1.00E-10	1808.01334	1959.08381	2219.88761	3068.6954	3157.3746	2846.69096
1.68E-22	3813.65463	3893.66576	3946.02443	2488.59767	2455.7358	2613.99143
0.00014043	568.039171	520.930932	544.519517	697.626818	725.933772	699.649931
9.05E-22	2807.44608	2917.8524	3046.1238	4439.14932	4475.13389	4468.60671
5.76E-12	4553.34779	4181.29633	3968.92013	2977.04427	2974.67641	2990.96467
1.80E-43	2964.41913	2923.17889	3154.62952	5632.77047	5745.27505	5434.30978
3.30E-11	2404.28508	2661.11548	2761.42073	3796.51318	3688.94859	3614.59942
1.27E-15	1674.75565	1603.27414	1609.66738	997.379917	992.206668	1104.54712
5.10E-16	7197.045	7709.56473	7897.02619	11196.533	11151.3923	10538.1862
2.84E-06	2176.16597	2357.50542	2417.9852	3150.64229	3113.64365	2806.35638
2.26E-22	1451.15375	1771.59129	1720.16403	3483.82109	3157.3746	2960.71373
0.03743157	1110.10438	1145.19581	1067.1388	1329.48047	1184.62285	1281.39877
2.72E-29	577.073591	696.705172	610.220226	254.46666	233.231734	228.045543
3.45E-06	8033.85817	8185.75313	8602.81108	6894.32128	6854.09758	6563.67818
3.14E-22	123.093975	115.05223	102.532925	1.07824856	0.97179889	6.2053209
1.70E-10	5985.30339	6256.49767	6121.1161	4679.59874	4780.27875	4750.94881
8.71E-08	560.134053	584.848837	750.580833	1075.01381	1275.00015	923.817149
1.00E-08	2495.75858	2369.22371	2576.26418	3328.5533	3332.2984	3161.611
3.64E-14	14068.8509	14844.9336	13781.2216	9642.77686	9587.76786	10597.9124

0.00054306	10133.2316	9132.80343	7926.89015	6832.86112	6391.52131	7765.18344
1.99E-07	9760.56173	9806.07204	9386.24227	11845.6387	11965.7597	13197.9419
1.78E-06	15.8102354	23.4365654	13.9365141	0	0	0
2.35E-10	1935.62453	1998.49985	1984.9578	1465.33979	1347.88506	1452.04509
0.00408703	788.253163	945.985005	947.682961	1100.89178	1245.84618	1037.06426
0.00075843	276.679119	351.548482	346.421923	197.319486	226.429142	244.33451
0.0003284	801.804794	834.12867	825.24073	539.124279	704.554196	584.851495
1.30E-07	1049.12205	1064.23313	1097.99822	1380.15816	1550.99103	1440.41011
3.33E-28	828.908054	828.802178	891.936905	1711.18046	1585.97579	1552.88155
0.00268557	485.600086	581.652942	545.514982	672.827101	698.723403	662.418006
7.32E-13	686.615936	782.994345	885.964113	1613.05984	1590.83479	1250.37216
3.71E-24	239.412136	295.087665	317.553429	79.7903933	81.6311069	75.2395159
2.81E-13	4985.87065	4680.9213	3644.39845	2224.42678	1619.01695	2443.3451
6.27E-07	604.176852	651.962638	723.70327	956.406472	960.137305	901.32286
0.00252112	35.0083783	35.1548482	32.8503548	11.8607341	18.4641789	5.42965579
1.84E-06	494.634506	497.494366	480.809738	731.052523	660.823246	674.052983
6.02E-14	14977.9394	13942.6258	14169.453	10735.0427	10638.2825	10405.5475
9.43E-28	413.324725	364.332063	393.208792	803.295176	888.224187	871.847586
4.53E-12	9148.47977	10066.0049	10946.1364	16626.5928	16696.4768	14010.0633
0.02505869	36.1376808	37.285445	48.7777995	67.9296592	73.8567157	58.1748834
0.01646947	204.403757	223.71267	266.784699	170.363272	187.557186	159.787013
2.09E-19	7463.5604	6833.88942	7069.79453	10326.3864	10682.0134	10351.2509
0.02641323	4018.05839	3945.86538	3664.30775	3471.96036	3155.431	3592.10513
1.17E-62	1211.74161	1282.61931	1317.00059	2816.38524	2882.35551	2862.97993
3.54E-37	4472.038	4631.91757	4607.01339	7535.87918	7384.69978	7578.24815
3.51E-13	1096.55275	1038.66597	1009.40181	672.827101	585.994731	670.174657
0.00235981	926.028072	993.923434	977.54692	781.730205	728.849168	840.820982
0.00094944	29.3618657	30.8936544	28.8684936	8.62598847	7.77439113	11.6349767
3.29E-69	1715.41054	1877.05583	1930.20721	554.219759	644.302665	567.011197
6.76E-28	665.159188	710.554052	732.662457	1337.02821	1310.9567	1327.93867
0.02641516	1058.15647	992.858136	1351.84187	1335.94996	1619.01695	1284.50143
1.62E-26	2302.64785	2354.30953	2138.25945	4215.95186	3756.00271	4287.87674
7.53E-06	4320.71147	4188.75342	3878.33279	5008.46456	5104.85958	5365.27558
3.84E-08	4689.99339	4358.13587	4348.19241	3154.95528	3341.04459	3560.30287
9.13E-16	2109.53712	2132.72745	2049.66304	1354.28019	1385.78522	1456.69908
0.00077783	1052.50995	1062.10253	917.819002	1260.47257	1196.28444	1412.48617
4.92E-05	2181.81248	2065.61365	2315.45228	1778.03187	1791.02536	1674.66098
0.00180085	1236.58627	1137.73872	1201.52661	980.12794	991.234869	1003.71066
5.91E-08	1058.15647	1030.14358	1051.21135	1472.88753	1617.07336	1355.86262
0.02825586	232.63632	239.692146	236.92074	191.928243	170.064806	193.916278
1.48E-16	730.658734	689.248083	646.056977	1162.35195	1126.31492	1140.22772
0.00047752	776.960138	823.475685	808.31782	644.792638	600.571715	658.53968
0.00361752	8488.96709	8470.18781	9558.45777	11070.378	10903.5836	9614.36907

3.45E-09	805.192701	976.878659	1040.26123	1450.24431	1464.50093	1422.56982
0.02774994	37.2669834	18.1100733	32.8503548	6.46949135	13.6051845	17.0646325
0.08674134	173.912589	144.880586	124.433162	127.23333	84.5465035	118.676762
2.23E-147	20036.0854	19844.3791	19571.8432	6904.02552	7451.7539	7197.39658
3.63E-11	693.391751	693.509277	730.671527	419.438689	459.660876	452.21276
4.70E-06	503.668927	544.367497	633.115928	383.856487	387.747758	356.805952
0.01831476	507.056834	529.453319	584.338128	660.966367	643.330866	637.596722
2.75E-10	55562.8136	59517.1579	54436.0242	42296.4562	39045.9077	43690.1131
0	2562.38743	2580.15279	2724.58851	14265.2284	14577.9552	14523.5536
0.00913823	5.64651263	13.8488796	8.95918766	31.2692082	35.956559	16.2889674
4.98E-63	298.135867	265.259309	331.489943	1182.83867	1366.34924	1112.30377
0.04753073	9.03442021	5.32649214	8.95918766	17.2519769	23.3231734	15.5133022
4.00E-38	23.7153531	20.2406701	18.9138406	198.397735	214.767555	207.87825
2.77E-14	12.4223278	11.7182827	9.95465295	78.7121448	106.897878	72.1368554
1.48E-14	299.265169	314.263036	316.557964	599.506199	704.554196	548.395234
6.56E-173	458.496826	425.054073	449.950314	2385.08581	2736.58568	2513.93063
1.40E-52	25.9739581	15.9794764	7.96372236	297.596602	413.014529	334.311663
1.48E-20	32.7497733	39.4160419	46.7868689	213.493215	260.442103	159.011348
6.11E-14	31.6204707	40.4813403	45.7914036	148.798301	138.967241	132.638734
3.22E-45	179.559102	180.035434	182.170149	636.16665	802.705884	639.923718
4.40E-18	2313.94088	2191.31887	2393.09857	1445.93132	1490.7395	1508.66864
4.24E-40	1826.08218	1881.31703	1859.52917	857.207604	751.200543	888.912219
9.81E-09	6634.65234	6398.18236	6647.71724	4814.37981	5330.31692	4934.00578
0.04659246	6761.13422	6392.85587	6074.32923	5393.39929	5587.84363	6143.26769
2.13E-08	5859.95081	5577.90257	5366.55341	4309.75949	4325.47687	4439.9071
2.69E-06	13468.0619	13117.0196	10937.1772	17184.0473	15863.6451	19743.0041
1.01E-15	4814.21667	5232.74588	5095.78685	7075.46704	7177.70661	7062.43085
7.51E-15	1121.39741	956.637989	999.447157	1616.29459	1625.81955	1663.80167
1.89E-08	4146.79888	4141.88029	3940.05164	5065.61173	5203.98306	5319.51134
6.56E-05	5555.03913	4981.33545	5039.04533	6175.1295	6278.79264	6524.89492
9.68E-05	7146.22639	7036.29612	6755.2275	6000.45323	5871.6089	5943.14609
2.09E-07	5844.14057	5783.50517	6093.24307	7568.22663	8014.42546	7149.30534
3.60E-43	3564.07877	3557.03145	3746.93137	6412.34418	6976.54424	6725.01652
6.98E-23	6348.9388	6174.46969	6146.00273	9006.61021	9687.86315	9325.82164
0.02312936	22852.5659	22490.5804	22616.9715	24890.2897	24776.9845	24342.6982
4.94E-17	6418.95556	5998.69545	6709.43609	4203.01288	4418.76956	4151.35968
0.00010231	4263.11704	4095.00716	4121.22632	4780.95411	4980.46932	4994.50766
1.52E-06	7848.65256	7883.20837	8027.43214	9574.8472	9597.48585	9359.95091
4.53E-19	2891.01447	3162.87104	3308.92664	5012.77755	5031.00286	4750.94881
6.20E-21	4343.29752	4252.67133	4273.53251	2891.86263	2940.66345	2939.77078
6.48E-11	2153.57992	1971.86739	1962.0621	2836.87196	3111.70005	2786.18908
0.00031171	1026.536	938.527916	760.535486	1269.09855	1122.42772	1351.98429
4.54E-09	195.369337	171.513047	151.310725	312.692082	310.003846	333.535998

2.76E-20	1224.16394	1268.77043	1162.70347	1966.72537	1952.34397	2107.48211
1.15E-49	1664.59192	1829.1174	1752.01892	776.338962	777.439113	762.478805
7.48E-05	12854.8507	13083.9953	14582.5711	16669.7227	17677.0218	15825.1196
1.42E-14	5913.02803	5801.61524	5771.70778	7969.3351	7909.47118	7688.39259
1.08E-21	3604.73366	3381.25721	3595.62065	2331.17338	2220.56047	2254.08282
8.25E-55	4560.1236	4261.19372	4100.32155	1820.08357	1632.62214	1898.05253
0.00365681	5125.90417	4781.05935	4281.49624	3917.27701	3475.15284	4197.12392
2.68E-13	1523.42911	1584.09876	1636.54495	1053.44884	1077.72497	952.516758
6.16E-06	7225.27756	6602.71966	5401.39469	4385.23689	3896.91355	4938.65977
0.00116057	203.274455	181.100733	147.328864	115.372596	99.1234869	120.228092
1.02E-05	5181.23999	5123.02014	4517.42151	3769.55696	3059.22291	3911.67916
5.91E-11	8001.1084	8408.4005	9002.98813	11250.4455	11449.7345	11666.779
5.64E-36	10565.7544	10900.1335	9956.64389	17966.8557	17514.7314	18700.5102
9.34E-20	9228.66024	9227.61499	9923.79353	13959.0058	14880.1846	13782.7934
0.00012611	1404.85234	1352.929	1452.38387	1670.20702	1946.51318	1727.40621
4.12E-06	1346.12861	1338.01483	1432.47456	1046.97935	1097.16095	1019.99962
5.15E-10	285.713539	223.71267	212.034108	92.7293761	118.559465	104.71479
6.62E-08	1899.48685	1951.62672	1959.0757	1435.14883	1533.49865	1451.26943
6.81E-34	4308.28914	4261.19372	4750.36039	2100.42819	2411.03305	2112.91177
1.04E-09	133.257698	87.3544712	85.6100154	26.956214	12.6333856	29.4752743
4.61E-28	3367.58013	3424.93445	3457.25097	1856.74402	1907.64122	1578.4785
2.62E-08	14031.5839	14362.3534	16171.3337	21990.8794	21417.4758	18781.1794
7.86E-06	6919.23658	6712.4454	7852.23025	5437.60748	5797.75219	4801.36704
5.28E-11	4457.35707	4632.98287	4921.58042	6654.9501	6768.57928	6109.91409
0.00041528	226.989808	264.19401	251.85272	341.804793	353.734796	339.741319
3.32E-07	2989.26379	2997.74978	3004.31426	2442.23299	2288.58639	2112.1361
1.94E-14	33765.0162	32827.1711	32112.715	24364.1044	22884.8921	24722.7741
4.74E-06	1488.42073	1427.49989	1281.16384	1779.11012	1827.95371	2008.19698
1.84E-27	3609.25087	3781.80942	4132.17644	6761.69671	7262.25311	6537.30557
2.76E-06	4472.038	4558.41198	5116.69162	6102.88684	6451.77284	5808.18036
2.78E-12	1565.2133	1548.94392	1507.13446	2277.26096	2130.18317	2143.93837
8.27E-05	924.898769	792.582031	687.866519	1136.47398	1020.38884	1282.17443
2.40E-15	371.540531	377.115644	404.15891	673.905349	771.60832	674.828648
4.09E-39	1420.66258	1462.65474	1441.43375	2806.681	3009.66117	2688.45528
8.02E-16	424.61775	555.020481	477.823342	998.458165	929.03974	882.706898
3.08E-24	779.218743	823.475685	691.84838	1934.37791	1507.26008	2051.63422
2.47E-91	6038.38061	6158.49022	6389.89173	15004.9069	14770.3713	13903.7971
2.26E-21	607.564759	602.958911	597.279177	299.753099	292.511466	304.060724
9.49E-33	652.73686	607.220104	561.442427	1376.92341	1257.50777	1306.22005
1.52E-40	2756.62747	2723.96808	2740.51596	4884.46597	4690.87325	5025.53426
0.02563218	144.550723	119.313424	167.23817	173.598018	217.682952	197.018939
1.25E-08	3150.75405	3104.27962	3027.20996	3852.5821	4070.86556	4000.88065
4.99E-14	626.762902	556.08578	626.147671	314.848579	355.678394	307.93905

0.00093097	17376.578	16931.8532	17126.9804	20097.4749	18857.7575	19880.2968
0.03286753	129.869791	156.598869	165.247239	118.607341	106.897878	108.593116
1.53E-06	491.246599	435.707057	337.462735	199.475983	158.403219	272.258454
6.21E-76	3661.19879	3457.9587	3589.64786	7807.59781	7525.61061	7997.10731
3.85E-32	12841.299	12634.4394	12014.2707	21211.3056	19735.2919	21250.8971
7.30E-124	4983.61205	4921.67874	4822.03389	11692.5274	11965.7597	11955.3264
0.00010992	849.2355	758.492481	991.483434	1162.35195	1240.01539	1128.59274
0.00028127	176.171194	177.904838	204.070386	260.936151	267.244695	293.201412
1.76E-34	587.237314	584.848837	563.433357	203.788978	196.303376	227.269878
1.07E-45	7366.44038	7391.0405	6972.23893	3816.9999	3702.55378	4019.49661
2.07E-09	4030.48072	4431.64146	4617.96351	5988.5925	6234.08989	5722.08153
0.05668184	598.530339	567.804063	572.392545	684.687835	634.584676	680.258303
1.27E-13	2309.42367	2495.99422	2965.49112	4646.17304	5093.19799	4059.05553
0.01632692	16135.4745	17704.1946	15498.3992	20471.6271	16853.9082	21188.0682
6.10E-15	5802.35638	6193.64507	5786.63976	8959.16727	8086.33857	8685.89793
1.66E-26	726.141524	778.733151	821.258869	1471.80928	1421.74178	1443.51277
0.00029502	1046.86344	1017.36	887.955044	756.930488	705.525995	798.935066
2.13E-09	683.228028	654.093235	697.821172	993.066923	1043.71201	944.760107
0.00195033	6391.8523	6676.22525	6637.76259	7673.89499	7390.53057	7382.78054
1.34E-12	5352.89397	5458.58915	5772.70325	3838.56487	4149.58127	3946.58409
0.00022796	1165.44021	1312.44766	1299.08221	1703.63272	1701.61986	1459.80174
4.04E-05	1501.97236	1461.58944	1417.54258	1233.51635	1089.38656	1091.36081
0.00069418	332.014943	302.544754	360.358437	459.333886	429.53511	449.1101
4.55E-07	13156.3744	12798.4953	12993.8085	10639.0785	10179.5934	10915.1595
5.07E-08	508.186137	525.192125	468.864154	313.770331	318.750036	334.311663
2.40E-11	1706.37612	1661.86555	1833.64707	2388.32056	2519.87453	2430.93446
1.88E-05	4921.50041	4946.1806	4927.55321	4054.21458	4113.62471	4226.5992
2.25E-10	108.413043	105.464544	93.5737378	34.5039539	33.0411623	23.2699534
7.62E-20	1511.00678	1576.64167	1708.21845	798.982182	953.334712	870.296256
1.04E-29	1154.14718	1374.23497	1561.88505	533.733037	560.72796	523.573951
6.37E-28	1338.22349	1430.69579	1248.31348	2509.0844	2370.2175	2601.58079
1.13E-11	2535.28417	2566.30391	2521.51359	3317.77082	3501.39141	3478.85803
0.03965033	481.082876	420.792879	387.236	540.202528	475.209658	544.516909
9.72E-19	4784.8548	4970.68247	4874.79355	3424.51742	3340.07279	3190.31061
0.00719717	352.342388	442.098848	449.950314	544.515522	573.361346	493.323011
0.08635903	2566.90464	2409.70505	2917.70878	2989.98325	3144.74121	2775.32977
2.88E-16	370.411229	410.139895	367.326694	119.68559	177.839197	158.235683
6.06E-07	164.878169	150.207078	171.220031	75.4773991	93.2926936	62.053209
9.63E-07	1149.62997	1133.47753	1013.38367	792.512691	780.35451	828.41034
0.00150852	758.891298	656.223832	670.943609	503.542077	494.645636	594.159476
6.06E-06	4063.23049	4172.77395	4432.80696	5433.29449	6116.50222	5078.27949
6.73E-12	4232.62587	4236.69185	4560.22652	2901.56687	3254.55449	2908.74417
1.92E-05	32.7497733	35.1548482	30.8594242	6.46949135	7.77439113	7.75665112

0.00027912	1783.16869	1977.19388	2096.44991	2400.18129	2744.36007	2342.50864
0.03932353	2101.632	2069.87485	1914.27976	2183.45333	2231.25025	2533.32226
1.08E-08	3811.39603	3824.42136	4103.30795	5096.88094	5230.22163	4938.65977
1.71E-16	494.634506	514.539141	442.982056	207.023723	159.375018	231.923869
1.30E-38	757.761995	661.550324	659.993491	1523.56521	1548.07563	1509.44431
1.03E-22	3149.62475	3207.61357	3417.43236	5096.88094	5003.79249	4978.99436
5.73E-15	1062.67368	1140.93462	970.578663	630.775407	487.843043	567.786862
3.07E-66	2259.73435	2244.58379	2013.82629	5491.51991	5323.51433	6037.77723
7.06E-07	1097.68206	1196.33014	1199.53568	1550.52143	1585.00399	1491.60401
1.71E-52	12607.5334	12568.3909	11649.9304	6430.6744	6549.92453	6541.18389
0.00753499	4916.9832	4979.20486	5091.80499	4424.05384	4451.81072	4508.16563
3.40E-17	2077.91665	2103.9644	2129.30027	3050.36517	3085.46148	3319.84668
6.60E-12	1770.74636	1854.68456	2038.71293	2939.30557	3297.31364	2704.74425
2.70E-05	7658.92973	7373.99572	7534.67682	8833.01219	9068.82725	8755.70779
1.23E-23	10442.6605	10489.9936	11324.4132	16171.5719	17188.207	16498.3969
4.85E-14	3810.26672	4003.3915	4402.943	6432.8309	6702.49695	5815.93701
0.00074928	50.8186137	52.199623	77.646293	29.1127111	27.210369	24.0456185
3.26E-06	285.713539	401.617508	371.308555	628.61891	575.304944	511.938974
0.00047519	19.1981429	23.4365654	19.9093059	52.8341794	44.702749	54.2965579
1.70E-34	2193.10551	2059.22186	2003.87164	3772.79171	3624.80986	3736.37885
1.45E-21	248.446556	222.647372	210.043177	45.2864395	47.6181457	69.8098601
6.61E-05	5193.66232	5655.66936	6023.5605	7169.27467	7635.42389	6598.58311
1.79E-12	3632.96623	3624.14525	3742.94951	4966.41286	5547.99987	5015.45062
2.86E-20	2382.82833	2360.70132	2407.03508	1577.47764	1558.76542	1502.46332
5.63E-10	7194.78639	6953.20284	6783.10052	5522.78912	5100.00058	5275.29843
6.74E-23	1745.90171	1719.39166	1444.42014	763.39998	686.090017	849.353298
0.02474147	241.670741	222.647372	266.784699	174.676267	210.880359	175.300315
0.00723026	11688.2811	11543.5738	10068.136	13885.6849	11747.105	14625.1657
8.18E-07	1107.84578	1061.03724	1097.00276	1392.01889	1438.26236	1421.79415
0.00074478	91.4735046	115.05223	100.541995	158.502538	202.134169	146.600706
1.41E-26	1466.96398	1633.10249	1653.46786	2787.27252	2787.11922	2715.60356
7.67E-17	2536.41347	2552.45504	2344.32077	3897.86854	3583.02251	3972.18104
3.88E-17	2133.25247	2270.15095	2350.29356	3497.83832	3387.69094	3303.55771
3.28E-07	1565.2133	1666.12674	2011.83536	2621.22225	2948.43784	2355.69495
0.03503918	72.2753617	64.9832042	73.6644319	38.8169481	51.5053412	46.5399067
0.00022545	3822.68905	3953.32247	4631.90002	5283.41794	5977.53498	4902.97917
2.14E-05	1294.1807	1126.02044	1315.00966	1710.10221	1656.91711	1549.77889
1.47E-37	6446.05882	6250.10588	4763.30144	1924.67368	1626.79134	2143.93837
1.98E-10	2450.58648	2457.64348	1780.88741	1192.54291	1082.58396	1391.54321
0.00318046	2399.76787	2601.45876	2610.11	3012.62647	2990.22519	2856.77461
6.80E-07	2521.73254	2633.41772	3023.2281	4307.60299	4393.50279	3395.86186
6.45E-18	292.489354	311.067141	288.684936	118.607341	127.305655	110.920111
1.40E-06	47.4307061	49.0037277	52.7596607	16.1737284	13.6051845	9.30798135

0.00661921	311.687497	331.307811	257.825512	421.595186	406.211937	362.235607
8.87E-05	94.8614122	71.3749947	81.6281542	26.956214	37.9001568	43.4372463
1.42E-32	1910.77987	1844.03158	1624.59936	4205.16938	3682.146	4711.38989
5.44E-17	185.205614	186.427225	184.16108	61.4601678	44.702749	60.5018788
4.06E-11	3309.9857	3425.99975	3463.22376	2595.34428	2481.97437	2375.86224
7.20E-96	1060.41507	1117.49805	1148.76695	243.684174	274.047287	255.193822
0.02331465	467.531246	498.559665	556.4651	650.183881	638.471872	564.684202
3.10E-05	3107.84055	3199.09118	3296.98106	3946.38972	4377.95401	3773.61077
1.56E-07	1264.81883	1230.41969	1453.37933	885.242067	974.714288	793.50541
2.95E-14	3254.64988	3288.57625	3112.81998	4913.57868	4398.36178	4997.61032
1.48E-13	1568.60121	1782.24427	1856.54278	1071.77907	1082.58396	902.098526
4.55E-23	1246.74999	1151.5876	1208.49487	1960.25588	1992.18773	2011.29964
0.00020465	15.8102354	7.457089	20.9047712	52.8341794	62.195129	33.3535998
2.20E-14	1707.50542	1708.73868	1850.56998	2596.42253	2781.28843	2530.2196
0.00513183	3534.71691	3630.53705	3469.19655	3120.45133	3047.56132	3183.32962
5.99E-15	348.954481	381.376838	390.222396	660.966367	715.243984	645.353373
0.000375	64.370244	60.7220104	60.723383	112.13785	128.277454	96.958139
0.0053241	1505.36027	1671.45323	1888.39767	2034.65503	2460.59479	1922.87381
2.08E-10	1274.98255	1267.70513	1198.54022	1755.38865	1784.22276	1689.39861
3.52E-06	2205.52783	2099.7032	2044.68572	2548.97959	2754.07806	2909.51984
1.24E-25	779.218743	814.953298	777.458396	1378.00166	1502.40109	1434.20479
8.70E-26	12388.4487	12090.0719	12396.5293	18013.2204	18855.8139	17969.058
9.68E-07	447.2038	464.470115	497.732648	306.222591	334.298819	289.323087
1.55E-12	529.642885	501.75556	490.764391	306.222591	274.047287	277.68811
0.00016998	4077.91142	3926.69001	3806.65929	4620.29507	4676.29627	4633.04772
8.02E-07	3521.16528	3312.01282	3415.44143	2724.73411	2701.60092	2777.65677
0.01365977	1150.75927	1089.80029	1139.80776	927.293761	1000.95286	977.338041
0.03992099	24.8446556	24.5018639	15.9274447	10.7824856	9.71798891	8.53231624
2.94E-67	155.843749	148.076482	167.23817	769.869471	741.482554	663.969336
0.00040859	4.5172101	2.13059686	9.95465295	18.3302255	41.7873523	25.5969487
0.04115483	287.972144	299.348858	303.616915	245.840671	264.329298	200.897264
0.0420469	2425.74183	2258.43267	1912.28883	1836.2573	1702.59166	2049.30723
0.00104822	1140.59555	947.050303	1095.01183	1261.55081	1377.03903	1353.53562
1.71E-30	2030.48594	1947.36553	1865.50196	3323.16206	3288.56745	3428.4398
0.00663852	549.97033	632.787267	571.39708	473.351117	462.576272	482.4637
1.61E-07	100.507925	134.227602	125.428627	45.2864395	57.3361346	35.6805952
4.04E-98	3249.00337	3381.25721	3279.06268	8105.19442	7636.39569	8032.7879
3.63E-32	4090.33375	3947.99598	3734.98579	7012.92863	6557.69892	7219.89087
2.13E-23	2355.72507	2124.20507	1933.1936	1150.49121	977.629685	1085.15549
3.31E-20	292.489354	266.324607	257.825512	553.141511	620.979492	566.235532
0.00029584	619.987087	541.171602	464.882293	410.812701	357.621992	380.075905
7.91E-45	1154.14718	1135.60813	1128.85765	461.490383	488.814842	498.752667
7.22E-10	761.149903	826.671581	791.39491	524.0288	492.702038	543.741244

0.06786062	4732.90689	4987.72724	4468.64371	4397.09762	3737.53854	4435.25311
9.67E-11	617.728482	629.591371	529.587537	313.770331	249.752315	353.703291
1.42E-08	4694.5106	5067.62463	5503.92762	3652.02787	3873.59038	3403.61851
3.38E-05	1943.52965	1980.38978	1749.03252	1535.42595	1245.84618	1453.59642
2.20E-17	469.789851	452.751832	502.709974	243.684174	219.626549	197.018939
0.00284321	129.869791	125.705215	208.052247	247.997169	276.962684	214.083571
3.10E-15	976.846685	921.483141	1011.39274	520.794054	587.938329	591.832481
4.96E-42	57.5944288	64.9832042	68.6871054	406.499707	358.593791	316.471366
4.88E-06	14225.8239	15046.275	13648.8247	11538.3378	10223.3243	11758.3074
0.00131099	1507.61887	1409.38982	1526.0483	1793.12735	1798.79975	1708.01458
5.97E-09	1253.5258	1248.52976	1494.19341	1889.09147	2195.2937	1944.59244
0.00601071	1016.37227	1186.74245	1110.93927	1478.27877	1516.97807	1180.5623
5.70E-12	2130.99387	2291.45692	2103.41817	3225.04144	3002.85857	3018.11295
1.72E-07	1948.04686	1878.12113	1759.98264	2472.42395	2356.61231	2520.13595
6.87E-40	3618.28529	3697.65085	3967.92467	6699.1583	6807.45123	6665.29031
0.00630202	1657.81611	1677.84503	1747.04159	1455.63555	1525.72426	1324.06035
6.99E-17	202.145152	190.688419	188.142941	415.125695	389.691355	440.577784
0.0011249	137.774908	157.664167	148.324329	255.544908	219.626549	193.916278
1.08E-46	2447.19857	2594.00167	2607.12361	4979.35184	4714.19642	5045.70156
1.17E-13	1616.03192	1614.99242	1624.59936	2365.67734	2247.77084	2503.07132
0.00068364	815.356424	819.214492	819.267938	627.540661	701.6388	606.570118
7.88E-33	2298.13064	2336.19945	2375.18019	4121.06599	4278.83052	3890.7362
0.00181928	2359.11298	2484.27594	2606.12814	2969.49653	3038.81513	2798.59973
0.02593179	924.898769	922.548439	960.62401	861.520598	792.987895	765.581466
0.05789912	154.714446	191.753717	223.979691	235.058186	272.10369	228.045543
8.85E-15	549.97033	569.934659	626.147671	998.458165	964.0245	947.862767
0.02761221	223.6019	226.908565	221.988761	163.893781	161.318616	193.140613
5.31E-23	1514.39469	1693.8245	1689.30461	2839.02846	2710.34711	2697.76326
4.38E-06	108.413043	85.2238743	91.5828072	44.2081909	27.210369	42.6615812
6.16E-12	1131.56113	1199.52603	1094.01636	765.556477	758.974934	753.170824
6.59E-13	238.282833	248.214534	176.197357	89.4946304	50.5335423	64.3802043
4.24E-23	4.5172101	2.13059686	1.99093059	140.172313	124.390258	162.114008
0.06077657	1136.07834	1242.13797	1051.21135	1024.33613	914.462757	1038.61559
9.65E-13	500.281019	485.776084	537.55126	286.814117	284.737075	290.098752
0.00018422	146.809328	135.2929	147.328864	214.571463	234.203533	210.205245
1.71E-14	154.714446	175.774241	160.269913	45.2864395	56.3643357	55.072223
4.69E-10	2689.99862	2793.21248	2611.10547	1938.69091	1847.38969	2058.61521
3.51E-13	1914.16778	1893.03531	1899.34778	1357.51494	1340.11067	1301.56606
0.00253477	396.385187	347.287288	364.340298	438.847163	482.01225	527.452276
4.66E-12	586.108011	651.962638	603.251969	343.96129	386.775959	338.965654
0.00012048	2195.36411	2086.91962	2222.874	1656.18979	1883.34625	1641.30738
1.05E-06	76.7925718	86.2891727	70.678036	26.956214	28.1821678	30.2509394
3.54E-06	791.641071	804.300314	842.16364	615.679927	581.135737	606.570118

7.92E-06	60.9823364	60.7220104	38.8231465	18.3302255	10.6897878	16.2889674
2.44E-07	971.200173	963.02978	905.873419	658.809869	581.135737	708.957913
0.00066404	86.9562945	52.199623	74.6598972	23.7214683	38.8719557	32.5779347
2.45E-07	8493.4843	8660.87623	8144.89705	11026.1698	10081.4417	11168.0263
0.03671017	814.227121	800.03912	910.850745	710.5658	784.241705	616.653764
3.73E-10	2413.3195	2611.04645	2853.999	3946.38972	4211.77639	3579.69449
2.19E-07	704.684776	718.011141	633.115928	444.238406	382.888763	489.444686
1.30E-05	1167.69881	983.27045	980.533316	1345.6542	1393.55961	1547.4519
0.00412924	13.5516303	4.26119372	19.9093059	2.15649712	1.94359778	0
1.87E-07	38.3962859	46.8731309	36.8322159	5.39124279	2.91539667	9.30798135
0.00190317	245.058648	266.324607	265.789234	367.682759	374.142573	314.920036
2.93E-17	1565.2133	1644.82077	1459.35212	962.875963	850.32403	942.433111
2.91E-26	3121.39218	3147.95686	3262.13977	2012.01181	1922.21821	1973.29205
9.53E-11	1864.47847	2007.02224	1965.04849	2815.30699	2679.24954	2598.47813
0.00117236	2173.90736	2206.23305	1991.92606	2478.89344	2477.11537	2651.99902
3.83E-10	1060.41507	1024.81709	1058.17961	1432.99233	1562.65262	1459.80174
4.63E-08	1016.37227	959.833884	1117.90753	1412.50561	1479.07791	1434.20479
1.09E-34	3648.77646	4080.09298	4106.29434	7048.51083	7301.12507	7046.91755
1.02E-17	2006.77059	2140.18454	2174.09621	3105.35585	3150.57201	3186.43228
1.03E-48	1475.9984	1511.65847	1487.22515	2855.20218	2891.1017	2932.01412
0.02863106	2645.95582	2452.31698	2448.84463	2223.34853	2176.82952	2309.9307
5.77E-43	1331.44768	1247.46446	1314.01419	2873.53241	2941.63524	2580.63783
1.39E-17	1604.73889	1701.28159	1661.43158	1041.58811	1053.43	1082.8285
5.49E-11	140.033513	165.121256	175.201892	63.616665	62.195129	58.9505485
1.65E-06	1653.2989	1543.61742	1397.63327	1038.35336	964.996299	1196.0756
0.00012838	1315.63744	1544.68272	1477.2705	1815.77057	1829.89731	1810.40237
2.02E-11	940.709004	850.108146	860.082015	512.168065	568.502351	578.646174
5.08E-10	53.0772187	45.8078324	36.8322159	0	0.97179889	3.10266045
1.30E-18	604.176852	523.061529	483.796134	210.258469	232.259935	253.642492
2.51E-13	328.627035	287.630576	302.62145	518.637557	591.825525	567.011197
1.47E-05	4452.83986	4207.92879	3701.13997	3217.4937	2816.27319	3252.36382
2.42E-40	2951.9968	2804.93076	2524.49999	1234.5946	1134.08931	1289.15542
1.64E-40	4328.61658	4814.0836	4580.13582	8174.20232	8323.4575	8570.32383
0.00628091	2033.87385	2089.05022	2125.31841	1853.50927	1820.17932	1773.17045
1.94E-13	3072.83217	3369.53893	3349.74072	4601.96485	4524.69564	4647.78535
0.00410447	1071.7081	1080.21261	1308.0414	1492.29601	1614.15796	1294.58507
1.18E-10	1888.19382	1907.94949	2054.64037	1436.22708	1293.46432	1353.53562
0.00012777	3224.15871	3038.23112	2824.13504	3949.62447	3508.194	3902.37118
4.54E-33	2341.04414	2304.2405	2117.35468	1119.222	1195.31264	1202.28092
1.38E-18	1341.6114	1327.36184	1177.63544	667.435858	695.808006	761.70314
1.73E-06	1391.30071	1427.49989	1631.56762	2013.09006	1964.00556	1954.67608
6.28E-21	190.852127	174.708942	209.047712	48.5211851	35.956559	51.9695625
0.01031831	368.152624	414.401089	420.086355	506.776823	526.714999	466.950398

5.85E-30	6944.08123	6654.91928	6648.71271	4277.41203	4333.25126	4183.93762
0.06433845	3763.96532	3736.00159	3354.71805	4182.52616	3730.73594	4278.56876
0.04643893	32.7497733	34.0895497	57.7369871	24.7997169	27.210369	16.2889674
2.66E-07	2051.94269	1945.23493	2114.36829	2589.95304	2687.02393	2593.82414
1.06E-19	1138.33695	1116.43275	1177.63544	1898.79571	1983.44154	1782.47843
5.98E-07	947.48482	940.658513	974.560524	687.92258	703.582397	722.919885
1.55E-23	387.350766	438.902953	455.923105	891.711558	841.57784	940.881781
2.97E-05	6051.93224	5610.92682	5026.10428	4406.80186	3604.40209	4370.87291
0.0113713	771.313625	706.292858	801.349563	644.792638	661.795045	559.254546
0.05554043	402.031699	371.789152	338.4582	328.86581	298.34226	295.528408
3.14E-06	533.030792	501.75556	610.220226	798.982182	792.016096	751.619494
2.24E-07	774.701533	842.651057	851.122828	1167.74319	1171.01766	1078.95017
1.32E-10	556.746145	675.399204	681.893727	1030.80562	990.26307	1026.20494
0.00190292	3.38790758	2.13059686	4.97732648	18.3302255	14.5769834	20.1672929
2.46E-44	5137.19719	4866.28322	4966.37636	2699.93439	2815.30139	2741.20051
0.00036291	1315.63744	1259.18274	1422.51991	1636.78131	1716.19684	1589.33782
0.04182134	1023.14809	1094.06149	1115.9166	1218.42087	1315.8157	1167.37599
2.00E-22	1847.53893	1924.99426	2073.55421	3406.1872	3688.94859	3186.43228
1.14E-11	2888.75586	2918.91769	2701.69281	4112.44	3796.81827	4354.58394
8.01E-06	219.08469	148.076482	129.410488	72.2426534	57.3361346	88.4258228
3.03E-07	1401.46444	1366.77788	1181.61731	2026.02904	1696.76086	1973.29205
7.26E-14	477.694969	518.800335	526.601141	284.65762	281.821678	262.174808
2.41E-11	4074.52351	4152.53328	3985.84304	2991.0615	2776.42943	3083.26882
1.02E-13	2238.27761	2384.13788	2223.86947	1608.74685	1454.78294	1548.22756
1.14E-08	3430.82107	3379.12662	3251.18965	2559.76208	2586.92865	2643.4667
0.00074583	3.38790758	3.19589529	2.98639589	19.4084741	15.5487823	20.1672929
2.53E-10	2923.76424	3254.4867	3463.22376	5065.61173	5144.70333	4349.92995
0.00179528	1654.4282	1995.30396	2389.11671	2985.67026	3126.27703	2259.51247
2.29E-12	293.618657	271.651099	274.748422	109.981353	143.826236	127.984744
4.60E-21	1849.79754	2043.24239	2191.01912	3773.86996	3734.62314	3329.93033
0.00029546	25.9739581	15.9794764	38.8231465	9.70423703	1.94359778	1.55133022
0.02523693	58.7237314	57.5261152	51.7641954	33.4257053	31.0975645	36.4562603
7.36E-06	362.506111	297.218262	340.449131	469.038123	535.461189	484.01503
1.80E-27	2272.15668	2128.46626	2065.59049	3542.04652	3600.51489	3651.05568
0.04535615	1575.37702	1648.01667	1346.86454	1277.72454	1030.10682	1449.71809
4.03E-11	5.64651263	5.32649214	5.97279177	49.5994337	56.3643357	56.6235532
2.72E-08	1753.80682	1625.6454	1449.39747	2241.67875	2212.78608	2345.6113
1.84E-54	712.589894	649.832042	698.816637	1642.17255	1665.6633	1618.03742
9.49E-16	1490.67933	1534.02974	1613.64924	2265.40022	2327.45834	2298.29573
6.42E-41	1348.38722	1427.49989	1355.82373	2660.03919	2763.79605	2977.0027
0.00018221	475.436364	495.363769	569.406149	748.3045	796.875091	622.859085
2.02E-18	1340.4821	1511.65847	1198.54022	2882.1584	2415.89204	3336.91131
1.04E-24	6746.45329	6406.70475	6628.8034	9981.34691	11238.8542	10742.9618

1.48E-13	10153.559	10039.3724	8527.15572	14693.2931	14028.8888	15680.0702
0.00010362	15209.4464	13451.5233	13210.8199	16153.2417	18813.0547	17836.4193
2.41E-09	9363.04725	8766.34077	8242.45265	11362.5833	11845.2567	11871.5545
4.07E-05	16029.3201	15564.01	14791.6188	12993.9734	12716.9603	13249.1358
3.10E-08	1688.30728	1665.06144	1559.89412	2115.52367	2239.99644	2461.96107
1.80E-13	159.231656	167.251853	200.088524	353.665527	393.578551	364.562603
0.01569632	141.162816	158.729466	165.247239	205.945475	200.190572	213.307906
3.79E-07	12761.1185	12789.9729	10678.3562	15950.5309	16123.1154	18684.9969
2.69E-16	11434.1881	10810.6485	8811.8588	17443.9052	18175.5547	20849.8782
0.05211663	640.314532	568.869361	614.202087	654.496875	720.102978	744.638508
2.14E-05	12382.8022	11329.4488	11829.1141	14343.9406	17793.6377	14679.4622
7.89E-07	10651.5814	9456.65415	9438.00647	7005.38089	8028.03064	6662.18765
4.78E-06	18080.1334	17314.2954	16291.785	20273.2294	22557.3959	22242.1971
1.10E-11	15743.6065	17119.3458	13522.4006	23878.8926	23719.6673	29182.8485
3.58E-06	14948.5775	15454.2843	13434.7996	10815.9113	10270.9425	12100.3758
0.01393982	100.507925	118.248126	100.541995	155.267792	134.108247	163.665339
0.00960622	22687.6878	21626.6234	21412.4585	23276.1516	25515.5517	25582.9867
4.58E-55	1798.97892	2091.18082	2064.59502	5255.38348	4836.64308	4668.72831
1.38E-17	42920.2718	40758.3179	34812.4168	67594.3239	63225.2359	74077.5696
3.74E-12	1119.1388	1086.6044	1054.19775	1561.30391	1540.30124	1630.44807
0.00488699	1514.39469	1445.60997	1305.055	1802.83159	1585.00399	1753.77882
3.31E-19	367.023321	314.263036	354.385645	705.174557	840.606041	687.239289
2.38E-05	2605.30093	2850.7386	3033.18276	3788.96544	3746.28473	3388.88088
8.26E-09	9366.43515	9232.94148	7473.95344	13827.4595	11732.528	14224.1468
0.01770008	9765.07894	8844.10756	6997.12556	9746.28872	9894.85631	12239.2198
2.03E-12	7159.77802	6712.4454	6133.06169	9268.62461	9760.74806	10196.8936
2.30E-22	2752.11026	2423.55393	2652.91501	1122.45675	1456.72654	1126.26574
1.35E-11	1924.3315	1959.08381	1932.19814	2762.47281	2569.43627	2681.47429
9.02E-13	1197.06068	1194.19954	1333.9235	1917.12594	1932.90799	1786.35675
0.01128028	1.12930253	2.13059686	1.99093059	10.7824856	12.6333856	8.53231624
0.01713232	6508.17046	6503.64691	6143.01634	5855.96792	4977.55392	5784.13474
2.23E-07	250.705161	254.606324	284.703075	410.812701	413.986328	414.20517
9.57E-08	2913.60052	2883.76285	2464.77207	1976.42961	1862.93847	2067.92319
1.87E-18	13797.8183	12991.3143	13529.3688	19121.6599	21726.5078	20260.3727
2.60E-05	3883.67139	3843.59673	3586.66146	4724.88518	4558.7086	5666.23365
1.08E-14	5.64651263	20.2406701	10.9501183	90.5728789	87.4619002	113.247106
0.0026423	1814.78916	1784.37487	1657.44972	1423.2881	1303.18231	1567.61919
1.14E-05	3997.73094	3742.39338	3534.89726	2880.0019	2765.73964	3130.58439
1.37E-07	843.588987	959.833884	925.782725	668.514106	626.810285	613.551104
2.29E-28	3811.39603	3590.05571	3503.04237	5816.07273	5998.91456	5865.57958
1.46E-09	1592.31656	1480.76482	1692.291	2225.50503	2413.94845	2166.43266
9.79E-46	1177.86253	1316.70886	1170.66719	3158.19003	2695.77012	3132.13572
6.22E-28	1751.54822	1769.46069	1729.12322	3110.74709	2836.68096	3114.29543

1.31E-17	1854.31475	1739.63233	1858.53371	2678.36942	3015.49196	2900.98752
1.22E-08	2208.91574	2360.70132	2543.41383	3428.83042	3213.73893	3136.78971
0.06352698	2779.21352	2640.87481	2637.98303	2971.65303	2961.07122	2894.7822
1.22E-06	3262.555	3259.81319	2896.80401	4185.76091	3894.96996	4589.61047
0.00145369	481.082876	524.126827	507.687301	648.027384	676.372028	601.140462
1.58E-15	1202.70719	1120.69395	1086.05264	1949.47339	1730.77383	2105.93078
5.04E-16	1027.6653	976.878659	915.828072	1500.92199	1593.75018	1690.94994
0.02664005	1577.63563	1677.84503	1419.53351	1375.84516	1223.4948	1392.31888
0.00462757	79.0511768	62.8526073	58.7324524	113.216099	121.474861	93.8554786
2.32E-38	5199.30883	5110.23656	5280.94339	8696.07463	9425.47745	8865.07657
6.21E-58	2343.30274	2354.30953	1989.93513	6988.12891	6316.69279	8152.24033
1.73E-10	1132.69043	1183.54655	1258.26813	1749.99741	1826.01012	1634.32639
1.43E-07	754.374088	814.953298	897.909697	457.177389	585.022933	387.056891
1.12E-53	3199.31406	2982.8356	3106.84719	6011.23571	6510.08077	6187.4806
1.72E-19	166.007471	153.402974	172.215496	42.0516938	43.7309501	34.1292649
6.76E-10	3349.51129	3259.81319	3339.78607	2322.5474	2597.61844	2402.23485
0.00042499	968.941568	865.022324	823.249799	746.148003	587.938329	645.353373
1.38E-21	697.908961	675.399204	712.753152	331.022308	332.355221	369.216593
0.01961845	11340.456	12127.3573	11597.1707	10298.352	10241.7885	10947.7374
7.92E-16	25035.5077	24589.2183	22691.6314	37342.9823	33474.5846	37297.8569
7.51E-06	396.385187	384.572733	359.362972	250.153666	253.639511	262.950473
1.34E-11	4758.88085	4426.31497	4356.15613	3151.72054	3276.90586	3367.16225
2.16E-11	1107.84578	1098.32268	1084.06171	1725.19769	1626.79134	1509.44431
1.50E-09	2906.8247	2924.24419	3175.53429	4036.9626	4153.46846	3900.04418
5.72E-09	2595.13721	2403.31326	2392.10311	3167.89427	3409.07051	3293.47407
2.08E-36	2806.31678	2902.93822	3151.64313	5430.05974	5627.68738	5307.87636
0.00168984	12447.1724	11920.6894	11836.0824	13229.0316	15299.0299	14096.9378
0.04548303	3.38790758	5.32649214	7.96372236	18.3302255	16.5205812	11.6349767
1.84E-10	9227.53094	8775.92846	8246.43451	11783.1002	11440.9883	12334.6266
3.02E-29	1990.96035	1911.14538	1870.47929	885.242067	951.391115	1056.45588
0.02221784	68431.2159	59116.6057	58203.8604	47514.101	54311.8964	57127.7355
3.84E-30	1022.01879	1013.09881	974.560524	1808.22283	1785.19456	1785.58109
1.45E-05	1.12930253	1.06529843	2.98639589	22.6432197	20.4077767	26.3726138
3.61E-10	53.0772187	64.9832042	59.7279177	7.54773991	13.6051845	8.53231624
0.00021496	12366.992	12026.154	9024.88837	7557.44415	6683.06098	9208.69621
1.04E-25	3139.46102	2973.24791	2933.63623	1623.84233	1803.65874	1800.31873
0.00467818	2247.31203	2266.95506	1747.04159	1620.60758	1180.73565	1799.54306
5.36E-24	1784.29799	2054.96067	2085.49979	3584.09821	3469.32204	3383.45122
3.57E-41	207.791665	180.035434	214.025039	14.0172313	10.6897878	15.5133022
4.46E-16	3416.14014	3381.25721	3580.68867	5094.72444	5609.2232	4960.37839
2.66E-05	1063.80298	1088.73499	1360.80106	1628.15532	1956.23117	1520.30362
0.00267907	1852.05614	1798.22375	1755.00532	1549.44318	1481.99331	1575.37584
0.00102658	1417.27467	1397.67154	1194.55835	1009.24065	889.195986	1148.76003

0.05538259	1517.7826	1530.83384	1427.49723	1398.48838	1160.32788	1348.10597
3.15E-30	7644.2488	7923.68971	7696.93766	4720.57219	4984.35651	4940.2111
1.23E-15	6311.67182	6347.04804	5634.33357	3664.96685	3515.96839	4150.58402
7.63E-48	1110.10438	1153.7182	1164.6944	460.412135	376.086171	396.364872
8.08E-64	10511.5479	10707.3145	9487.77973	22899.8429	22234.7586	24093.7097
6.01E-25	757.761995	852.238743	778.453861	379.543493	386.775959	386.281226
0.00165004	7049.10637	6496.18982	5726.91184	5284.49619	4810.40451	5521.95993
5.05E-67	3101.06474	3152.21805	2717.62026	8451.3122	8112.57714	9663.23597
6.99E-20	257.480976	300.414157	310.585172	667.435858	664.710442	578.646174
2.54E-22	5614.89216	5686.56301	5584.56031	8010.30854	8429.38358	8479.57101
2.08E-08	1334.83559	1283.68461	1270.21372	1693.92849	1736.60462	1886.41755
4.59E-13	1651.04029	1688.49801	1812.7423	2677.29117	2797.80901	2412.3185
9.45E-06	1899.48685	2155.09872	2201.96923	2733.3601	2788.09102	2582.96482
6.54E-08	167.136774	172.578345	193.120267	284.65762	320.693634	322.676687
2.40E-11	1762.84124	1678.91032	2139.25492	1103.04828	1192.39724	974.235381
7.50E-22	714.848499	646.636146	686.871054	346.117787	334.298819	302.509394
0.01878101	555.616843	483.645487	555.469635	411.890949	477.153256	404.121523
1.07E-63	962.165752	925.744335	1029.31112	306.222591	271.131891	286.220426
0.00802864	36.1376808	31.9589529	17.9183753	9.70423703	14.5769834	6.98098601
0.000411	2141.15759	2098.6379	2216.90121	2630.92648	2548.05669	2494.539
0.00906224	7129.28685	7143.89126	7734.76535	6339.02328	6791.90245	5809.73169
0.00031839	250.705161	215.190283	193.120267	129.389827	113.70047	159.011348
1.18E-32	800.675491	802.169717	718.725943	246.91892	220.598348	304.060724
8.47E-41	23757.1372	24559.39	26597.8372	13046.8076	13832.5854	12616.1931
2.61E-36	2790.50654	2782.5595	2925.6725	4935.14365	5308.93734	4916.94115
1.99E-13	364.764716	372.85445	380.267743	194.084741	170.064806	131.863069
1.77E-09	2071.14083	2076.26664	2317.44321	3062.22591	3326.4676	2874.61491
7.21E-31	1470.35189	1578.77227	1698.26379	3010.46998	3431.42189	3060.77453
1.14E-32	287.972144	335.569005	374.294951	929.450258	954.306511	829.96167
1.84E-54	450.591708	423.988775	422.077285	83.025139	87.4619002	92.3041484
3.27E-98	1329.18907	1238.94207	1415.55165	295.440105	236.147131	241.23185
1.45E-06	60.9823364	50.0690262	58.7324524	108.903104	128.277454	133.414399
6.21E-63	1649.91099	1673.58383	1831.65614	646.949135	613.2051	574.767848
3.32E-19	1284.01697	1310.31707	1249.30895	2047.59401	1970.80815	2181.1703
4.92E-10	2653.86094	2476.81885	2351.28903	1809.30108	1774.50478	1813.50503
1.26E-18	1169.95742	1204.85252	1004.42448	569.315239	492.702038	610.448443
5.47E-08	260.868884	194.949612	171.220031	104.59011	73.8567157	92.3041484
8.68E-14	1343.87001	1422.1734	1603.69459	2586.71829	2724.92409	2200.56192
0.00434599	429.13496	442.098848	433.027404	553.141511	526.714999	537.535923
1.41E-27	2727.2656	2959.39904	2987.39135	4667.73801	4868.71245	4840.92597
8.35E-11	3150.75405	3313.07811	3052.0966	2354.89485	2173.91412	2337.07898
0.01157723	3317.89082	3416.41206	3570.73401	3020.17421	3042.70233	3077.0635
0.06099009	17142.8123	17501.7879	16654.1344	15879.3665	15085.2342	16195.8875

7.57E-07	12151.2952	12958.2901	12683.2233	14973.6377	15673.1725	15734.3668
0.00019317	3452.27782	3439.84863	3153.63406	4188.99565	3824.02864	4429.82346
2.07E-17	1089.77694	1212.30961	1141.79869	1924.67368	1809.48954	1794.1134
7.76E-06	2684.3521	2640.87481	2222.874	1718.7282	1572.37061	2012.85097
1.35E-15	9844.13012	9648.40787	8650.59342	6329.31904	5877.43969	6396.91018
0.04744875	762.279205	690.313382	718.725943	577.941227	613.2051	667.847662
3.74E-05	4569.15802	4851.36904	4569.18571	3686.53182	3935.78551	3914.00616
5.33E-30	170.524681	200.276105	193.120267	7.54773991	23.3231734	24.0456185
5.24E-06	2643.69721	2951.94195	2926.66797	2276.18271	2243.88364	2020.60762
4.19E-09	39.5255884	47.9384293	29.8639589	1.07824856	3.88719557	1.55133022
1.51E-07	4789.37201	4589.30563	4235.70483	3264.93664	3213.73893	3631.66406
2.72E-05	612.081969	559.281675	527.596607	725.66128	777.439113	804.364721
0.00055375	47.4307061	36.2201466	47.7823342	17.2519769	17.49238	16.2889674
0.00059568	4822.12179	5217.8317	5318.77107	6284.0326	6071.79947	5843.86096
1.23E-10	93.7321097	90.5503664	113.483044	30.1909596	30.1257656	24.8212836
1.41E-05	3096.54753	3039.29642	3213.36197	2512.31914	2608.30822	2534.87359
5.56E-11	3777.51695	4081.15828	3916.16047	5246.75749	5180.65989	5199.28325
7.21E-29	6728.38445	6419.48833	6619.84421	4196.54339	4283.68951	4154.46234
2.61E-16	595.142431	594.436523	625.152206	337.491799	316.806439	335.087329
0.00099851	697.908961	702.031665	777.458396	882.007321	923.208947	904.425521
9.35E-29	3364.19223	3151.15275	3508.0197	5653.25719	6180.64095	5694.93325
1.77E-06	1691.69518	1708.73868	1749.03252	2150.02763	2271.09401	2119.89275
1.14E-30	4230.36726	4286.76088	4540.31721	7148.78794	7130.08847	6932.89477
2.29E-43	802.934096	743.578303	741.621645	265.249145	222.541946	267.604464
2.53E-17	1349.51652	1412.58572	1202.52208	733.20902	537.404787	664.745001
4.12E-31	3383.39037	3369.53893	2901.78134	1293.89827	1028.16323	1423.34548
8.97E-19	24.8446556	13.8488796	31.8548895	141.250561	147.713431	131.863069
1.03E-09	230.377715	274.846995	294.657727	141.250561	126.333856	127.209078
3.81E-12	66.628849	55.3955183	62.7143136	9.70423703	7.77439113	6.98098601
4.82E-50	3901.74023	3985.28142	4046.56643	2085.33271	1963.03376	1895.72553
4.24E-18	810.839214	845.846952	884.968648	480.898857	487.843043	458.418081
3.34E-18	146.809328	139.554094	180.179218	38.8169481	34.0129612	21.7186231
3.48E-112	4731.77758	4532.84481	4109.28074	1217.34262	1058.28899	1205.38358
6.47E-09	151.326539	180.035434	163.256308	69.0079078	64.1387268	81.4448368
0.00092977	12.4223278	6.39179057	3.98186118	0	0	0
5.37E-62	1206.0951	1327.36184	1185.59917	3284.34511	2963.01482	3308.2117
0.04942273	3843.0165	4135.4885	4106.29434	4569.61739	4504.28786	4275.4661
2.73E-13	13140.5642	13579.3591	13300.4118	9904.79126	9934.70007	10361.3346
0.005676	2624.49907	2602.52406	2405.04415	3011.54822	2816.27319	3004.92664
9.77E-11	3759.44811	3565.55384	4275.52344	2488.59767	2653.98277	2103.60378
1.03E-11	48.5600086	52.199623	75.6553625	3.23474568	7.77439113	6.2053209
0.00057293	154.714446	134.227602	120.451301	93.8076246	75.8003135	69.8098601
0.00159546	68.8874541	106.529843	111.492113	63.616665	45.6745479	37.2319254

2.11E-39	255.222371	273.781696	272.757491	45.2864395	34.9847601	49.6425672
2.33E-10	1000.56204	909.764858	947.682961	658.809869	620.979492	630.615736
1.42E-08	8221.32239	8570.32586	8747.15355	6319.6148	6867.70276	5990.46166
0.00521896	220.213993	211.994387	223.979691	252.310163	307.08845	334.311663
1.95E-10	3386.77828	3308.81692	3515.98342	2583.48355	2569.43627	2538.75191
2.90E-05	683.228028	709.488754	712.753152	924.059015	963.052701	872.623251
3.97E-108	7966.10002	8111.18224	8333.03999	18274.1566	18626.4693	18166.8526
3.39E-05	784.865256	758.492481	715.739547	561.767499	555.868966	584.07583
0.0084199	22.5860505	24.5018639	19.9093059	4.31299423	9.71798891	8.53231624
3.53E-13	3173.3401	2963.66023	3290.0128	4705.47671	4785.13774	4309.59536
9.34E-54	8588.34571	8315.71954	8112.04669	4483.35751	4494.56987	4575.6485
1.94E-07	224.731203	185.361927	194.115733	101.355365	100.095286	111.695776
1.11E-18	117.447463	70.3096963	100.541995	6.46949135	7.77439113	8.53231624
0.01504204	5075.08555	5006.90262	5064.92742	4197.62164	4556.765	4669.50398
0.05751393	0	0	0.9954653	3.23474568	6.80259224	3.10266045
0.0251953	0	1.06529843	0.9954653	5.39124279	14.5769834	3.10266045
4.90E-47	26435.8428	27227.9625	26617.7465	45109.6067	43630.8548	44545.6717
1.82E-33	760.0206	772.341361	674.92547	1530.0347	1617.07336	1495.48234
5.44E-31	3699.59508	3496.30944	3806.65929	6347.64927	6415.81628	5988.13467
0.00044222	278.937724	337.699602	314.567033	222.119203	225.457343	203.999925
2.32E-42	306.040985	307.871246	343.435527	898.181049	824.08546	924.592814
0.06636191	56.4651263	46.8731309	53.755126	23.7214683	40.8155534	34.1292649
0.07048547	841.330382	856.499937	923.791794	773.104217	818.254666	695.771606
1.81E-36	16214.5257	16712.4018	15641.7462	29608.7054	27668.0862	31664.9769
2.13E-09	665.159188	607.220104	633.115928	911.120032	914.462757	968.03006
0.00734082	4928.27622	4972.81307	5519.85506	6202.08571	6409.01369	5528.94092
8.66E-22	1220.77603	1238.94207	1140.80323	2018.4813	1958.17477	2171.86231
2.38E-06	12492.3445	11670.3443	13094.3505	16390.4563	17519.5904	14852.4356
9.95E-12	3567.46668	3653.97361	3746.93137	5201.47105	5401.25824	4781.97542
2.15E-07	3523.42388	3163.93633	3398.51852	4577.16513	4332.27946	4218.84255
1.09E-44	3184.63312	2908.26471	3082.95602	6461.94361	5859.94731	6639.69336
6.16E-06	2689.99862	2629.15652	2930.64983	3793.27843	4158.32746	3293.47407
3.94E-06	2566.90464	2703.72741	2243.77878	3306.98833	3223.45692	3868.24191
6.76E-12	739.693155	703.096963	725.6942	477.664112	416.901724	434.372463
2.45E-07	938.450399	1007.77231	961.619475	1301.44601	1305.12591	1275.19344
1.88E-05	3470.34666	3645.45122	2890.83122	4665.58151	4111.68111	4925.47346
3.03E-07	4443.80544	4473.1881	3951.99722	3184.06799	2991.19699	3400.51585
5.44E-53	368.152624	376.050345	382.258673	996.301668	1126.31492	1089.80948
9.58E-86	3305.46849	3345.03707	3786.74998	1200.09065	1172.96126	1166.60033
3.15E-09	505.927532	533.714513	556.4651	315.926828	324.58083	352.927626
1.55E-10	2839.06655	3160.74044	3092.91067	2140.32339	2253.60163	2030.69126
8.10E-54	3817.04254	3720.02211	3816.61394	1821.16182	1859.05128	1669.23132
0.00014784	3713.14671	3632.66764	3842.49604	3240.13692	2987.30979	3104.21178

6.34E-45	2347.81995	2233.93081	2198.98284	1067.46607	901.829371	988.973018
1.09E-17	4627.88175	4766.14517	4346.20148	7354.73342	6582.96569	7261.00112
1.48E-25	761.149903	825.606282	919.809933	1793.12735	1905.69763	1597.87013
2.22E-05	1644.26448	1528.70325	1508.12992	1257.23782	1109.79433	1240.28851
2.40E-19	3146.23684	2879.50165	3157.61592	5025.71653	5385.70946	4658.64466
1.00E-34	1837.37521	1958.01851	1678.35449	784.964951	682.202822	833.839996
9.10E-42	924.898769	940.658513	851.122828	2577.01406	2116.57799	2760.59213
3.84E-175	3501.96713	3764.76465	3799.69103	769.869471	827.000857	723.69555
5.37E-32	649.348953	598.697717	684.880123	253.388411	236.147131	259.072147
5.66E-34	722.753617	737.186513	751.576298	1593.65137	1429.51617	1486.95002
1.22E-05	2261.99296	2270.15095	2046.67665	1758.6234	1452.83934	1699.48226
8.14E-06	7702.97253	7987.60762	8606.79294	6119.06057	6843.40779	5884.19554
2.94E-16	4189.71237	4210.05939	3828.55953	2624.45699	2306.07877	2690.00661
2.89E-25	8460.73453	8433.96766	9241.8998	15223.7914	17238.7405	14503.3863
7.77E-14	1344.99931	1401.93273	1429.48816	2171.5926	2207.92708	1964.75973
5.10E-05	202.145152	207.733194	188.142941	113.216099	129.249253	124.882083
9.64E-34	1939.01244	2028.32821	2228.8468	894.946304	953.334712	757.824815
5.38E-06	322.980523	274.846995	391.217861	563.923996	526.714999	487.117691
4.56E-09	2828.90283	2646.2013	2804.22574	2157.57537	1971.77995	2014.4023
2.40E-41	748.727575	769.145466	823.249799	291.127111	301.257656	290.874417
2.56E-07	3635.22483	3012.66396	2817.16679	1967.80362	2143.78835	2307.60371
3.37E-15	136.645606	148.076482	126.424093	42.0516938	29.1539667	35.6805952
2.45E-07	528.513582	517.735036	485.787064	354.743776	324.58083	339.741319
2.93E-24	3246.74476	3048.8841	3148.65673	1991.52509	2001.90572	1971.74072
1.59E-19	1455.67096	1453.06706	1534.01202	887.398564	839.634242	925.368479
1.86E-05	584.978709	631.721968	692.843846	476.585863	435.365903	454.539756
0.00231296	912.476441	858.630534	840.172709	712.722297	547.122776	730.676536
1.77E-22	1770.74636	1801.41964	1893.37499	1049.13585	1065.09158	918.387493
2.25E-07	924.898769	951.311497	828.227126	1216.26437	1231.2692	1346.55463
4.98E-07	776.960138	774.471958	770.490139	1018.94489	1027.19143	1131.6954
4.27E-06	370.411229	307.871246	275.743887	190.849995	154.516024	202.448594
9.90E-12	1241.10348	1383.82266	1447.40654	2195.31407	2131.15497	1932.95746
2.67E-05	722.753617	662.615623	609.224761	843.190373	991.234869	908.303846
4.21E-15	3988.69652	4061.98291	3657.3395	2515.55389	1943.59778	2325.44401
6.06E-24	2.25860505	7.457089	10.9501183	125.076833	120.503063	120.228092
1.17E-09	40.6548909	52.199623	56.7415218	2.15649712	0.97179889	0
2.29E-06	1472.61049	1423.2387	1431.47909	1154.80421	1105.90714	1097.56613
2.06E-09	991.527618	953.442094	825.24073	629.697158	565.586955	577.870509
3.83E-05	1268.20674	1240.00737	1153.74428	977.971443	857.126622	963.376069
1.72E-06	3150.75405	3290.70685	3113.81544	3864.44283	3926.06752	4044.3179
5.20E-26	1887.06452	1953.75732	2046.67665	3192.69398	3457.66046	3268.65278
4.14E-07	1511.00678	1555.33571	1524.05737	1950.55164	2098.11381	1929.8548
1.35E-10	1200.44859	1369.97378	1369.76025	902.494044	815.33927	878.828572

1.84E-06	7.90511768	10.6529843	17.9183753	49.5994337	48.5899446	52.7452276
2.03E-06	2266.51017	2328.74237	2479.70405	3075.16489	2976.62	2910.2955
2.95E-06	2566.90464	2493.86362	2494.63603	2058.3765	1894.03604	2035.34525
1.53E-11	2442.68136	2615.30764	2633.00571	3694.07956	3405.18332	3627.78573
2.58E-13	1208.3537	1276.22752	1205.50847	808.686419	693.864408	795.05674
0.00025755	81.3097819	75.6361884	67.6916401	136.937567	135.080046	118.676762
0.00013701	134.387001	147.011183	143.347003	84.1033876	87.4619002	77.5665112
5.74E-10	19197.0136	17126.8028	18529.591	14125.0561	13259.2241	13677.3029
0.01859962	127.611185	126.770513	133.39235	99.1988674	84.5465035	91.5284832
4.71E-62	5559.55634	5719.58726	5871.25431	2867.06292	2901.79149	2800.92672
0.05167131	2568.03394	2507.7125	2722.59758	2927.44484	2861.94773	2831.95332
1.95E-09	939.579702	1101.51858	1027.32018	632.931904	524.771401	691.89328
1.30E-34	516.091254	536.910408	558.456031	1110.59602	1161.29968	1137.90072
2.27E-172	6796.1426	6699.66182	6702.46783	18668.7955	18213.4548	18142.807
0.00279078	35.0083783	31.9589529	26.877563	7.54773991	14.5769834	11.6349767
1.30E-15	9915.27618	9167.95828	9327.50982	12900.1658	13396.2477	13467.8733
7.87E-56	328.627035	319.589529	322.530756	916.511275	991.234869	943.208777
7.50E-20	540.93591	545.432796	558.456031	266.327394	227.400941	273.03412
0.01297218	60.9823364	57.5261152	50.7687301	44.2081909	19.4359778	24.0456185
6.20E-09	58.7237314	53.2649214	52.7596607	125.076833	150.628828	132.638734
2.72E-07	3587.79413	3455.8281	3037.16462	2485.36293	2187.5193	2544.18157
6.69E-22	2712.58467	2587.60988	2351.28903	1483.67002	1420.76998	1529.6116
0.06153865	2110.66642	2177.46999	1925.22988	1800.67509	1578.2014	1998.88899
1.22E-13	1137.20764	1106.84507	1064.1524	733.20902	691.920811	702.752592
3.46E-09	343.307968	347.287288	335.471805	589.801962	516.025211	535.208927
1.10E-20	3023.14286	3071.25537	2786.30736	5201.47105	4600.49595	5429.65579
1.14E-14	2487.85347	2290.39162	2593.18709	1532.1912	1599.58098	1324.83601
1.07E-17	1258.04301	1155.8488	1046.23403	2037.88978	1977.61074	2389.82421
5.05E-71	2302.64785	2551.38974	2409.02602	5412.80776	5472.19956	5400.18051
1.63E-16	302.653077	280.173487	270.76656	558.532753	523.799602	573.992183
3.55E-08	153.585144	166.186555	158.278982	79.7903933	77.7439113	63.6045392
1.99E-12	1856.57335	1839.77039	1688.30914	2608.28326	2515.01553	2800.15106
0.01767763	2686.61071	2526.88787	2326.4024	2052.98526	1983.44154	2356.47061
1.25E-05	263.127489	344.091393	262.802838	148.798301	186.585387	176.07598
0.02518886	1577.63563	1701.28159	1634.55402	1889.09147	2016.4827	1746.79783
0.0006172	2448.32788	2702.66211	2749.47515	2231.97452	2206.95528	2125.32241
3.18E-19	1517.7826	1817.39912	1958.08024	908.963535	953.334712	854.782954
5.23E-14	1588.92865	1553.20511	1570.84424	1076.09206	1060.23259	975.786711
0.03177114	28.2325632	29.828356	27.8730283	57.1471736	45.6745479	44.2129114
7.65E-28	1645.39378	1512.72377	1581.79435	882.007321	790.072499	833.064331
5.37E-22	308.29959	314.263036	235.925275	76.5556477	66.0823246	89.2014879
4.27E-07	6400.88672	6018.93612	6092.24761	7829.16278	7567.39797	7515.41927
3.53E-09	224.731203	198.145508	234.92981	118.607341	103.010682	95.4068088

2.14E-24	575.944288	570.999958	719.721409	180.067509	240.034326	159.011348
1.46E-26	212.308875	257.80222	265.789234	58.2254222	43.7309501	59.7262136
3.39E-06	6939.56402	6760.38383	6070.34737	5034.34252	4261.33814	5240.3935
0.0022251	68.8874541	70.3096963	59.7279177	32.3474568	42.7591512	24.0456185
0.00223221	3518.90667	3795.6583	4148.10389	3157.11178	3365.33956	2889.35254
2.77E-06	2397.50926	2413.96624	2281.60646	2836.87196	3183.61317	3039.83158
0.01080907	5141.7144	4951.5071	5214.24722	4637.54705	4461.52871	4573.3215
0.00113976	2325.2339	2292.52222	2037.71746	1911.73469	1728.83023	1824.36434
8.42E-18	4795.01853	4669.20301	4735.42841	3206.71121	2905.67869	3255.46648
0.03103648	1252.3965	1345.47192	1416.54712	1473.96578	1585.97579	1528.83594
0.00366046	2228.11388	2319.15468	2528.48185	3041.73918	2912.48128	2586.06748
1.56E-22	4509.30499	4341.0911	4711.53724	7203.77862	7952.23033	7053.12287
0.02074396	776.960138	824.540984	953.655753	1113.83076	1053.43	932.349465
4.32E-12	473.177758	461.27422	537.55126	264.170897	283.765276	264.501803
0.00510752	3431.95038	3449.43631	3688.19892	3038.50444	3192.35936	2911.84683
1.17E-61	3051.37543	2989.22739	2889.83575	1209.79488	1190.45364	1334.14399
1.23E-48	10962.1396	11402.9544	11928.6606	22259.3632	23575.8411	21279.5967
1.92E-113	21942.3481	24151.3807	26132.9549	81335.5235	86872.9901	78322.009
0.02290281	1088.64764	1091.93089	1222.43138	975.814946	1035.93762	811.345707
1.04E-50	705.814079	700.966366	750.580833	216.72796	240.034326	224.167217
0.0861586	11852.03	11901.514	10891.3858	13023.0861	11644.0943	13880.5272
3.87E-05	4792.75992	5451.13206	5691.07509	4154.4917	4371.15141	3896.94152
1.08E-14	641.443835	532.649214	534.564864	307.300839	273.075488	287.771757
1.89E-09	5303.20466	5142.19552	4648.82293	3674.67109	3141.82582	3643.29903
1.03E-47	5290.78234	5859.14136	6099.21587	11624.5977	11753.9076	11428.6498
2.23E-05	3547.13923	3952.25717	3998.78409	3073.00839	3146.68481	2996.39433
2.62E-47	7710.87765	7536.98638	6699.48144	18514.606	15782.9858	18931.6584
8.68E-27	370.411229	384.572733	409.136236	135.859318	130.221051	100.836465
0.03044561	643.70244	632.787267	681.893727	806.529922	758.003135	726.79821
3.31E-19	451.72101	463.404817	439.995661	225.353949	193.387979	195.467608
0.03597056	16868.3918	15893.1873	17031.4157	17771.6927	18487.5021	18328.1909
8.91E-13	3775.25835	4053.46052	4231.72297	2840.1067	2884.29911	2762.91913
6.40E-22	10413.2986	10709.4451	10439.4446	7405.4111	7387.61517	7367.26724
0.00198077	103.895832	136.358199	111.492113	60.3819193	80.659308	68.2585299
0.07595523	1224.16394	1220.832	1384.69223	1156.9607	1164.21507	1073.52052
2.60E-29	257.480976	231.169759	273.752956	53.9124279	65.1105257	54.2965579
0.00016846	13758.2927	13562.3143	13958.4144	11837.0127	10715.0546	11994.1096
0.00569707	30.4911682	13.8488796	23.8911671	43.1299423	68.0259224	41.8859161
2.15E-57	1029.9239	1285.8152	1281.16384	3621.83691	3606.34569	3298.90372
0.00062827	12.4223278	15.9794764	32.8503548	4.31299423	2.91539667	3.10266045
0.03551058	4028.22211	4095.00716	4571.17664	3603.50668	4044.62699	3385.00255
1.11E-08	240.541438	215.190283	267.780164	100.277116	110.785074	135.741395
1.95E-104	799.546189	808.561507	777.458396	2366.75559	2407.14585	2331.64933

1.11E-26	5542.6168	5520.37646	6396.85999	11357.1921	11979.3649	10156.559
2.52E-223	1719.92775	1848.29277	1832.65161	7497.06223	7418.71274	7157.83766
0.01015098	2.25860505	1.06529843	0.9954653	15.0954798	6.80259224	9.30798135
4.04E-41	787.123861	823.475685	864.063876	2044.35927	2039.80587	1759.98414
7.20E-92	8339.89916	8479.77549	9019.91104	21753.6647	21452.4605	20058.6998
8.03E-09	2056.4599	2046.43828	2282.60192	2954.40105	3168.06439	2789.29174
1.45E-07	4053.06677	4132.29261	3707.11276	3012.62647	2407.14585	2907.19284
0.00034323	937.321097	892.720083	999.447157	1256.15957	1206.97422	1116.1821
0.01225088	2062.10641	2031.5241	2376.17566	1888.01323	1855.16408	1784.02976
0.00395485	3186.89173	2984.9662	3090.91974	2586.71829	2753.10626	2698.53893
0.02962612	47.4307061	44.742534	50.7687301	28.0344625	26.2385701	29.4752743
0.03433084	3.38790758	7.457089	11.9455835	24.7997169	27.210369	11.6349767
0.07625315	12.4223278	21.3059686	14.9319794	4.31299423	6.80259224	9.30798135
8.96E-14	1739.12589	1659.73495	1753.01439	1164.50844	1180.73565	1075.84751
5.81E-147	2105.01991	2105.0297	2089.48166	507.855071	541.291982	525.125281
7.85E-70	944.096912	879.936502	854.109224	239.37118	236.147131	252.091161
0.00954112	2601.91302	2601.45876	3000.3324	2323.62564	2440.18702	2216.07523
4.83E-11	801.804794	930.005528	806.326889	1443.77482	1222.52301	1403.95385
0.00900934	57.5944288	45.8078324	51.7641954	78.7121448	86.4901013	86.0988275
0.00193366	866.175038	1029.07828	1062.16147	778.495459	821.170063	726.022545
7.47E-05	945.226214	949.1809	1029.31112	1335.94996	1307.06951	1160.39501
0.00046166	33.8790758	34.0895497	37.8276812	78.7121448	68.9977213	69.034195
0.00788344	4278.92727	4433.77206	4669.7277	3889.24255	4038.79619	3833.33698
4.02E-21	269.903304	185.361927	260.811907	653.418627	579.192139	643.026378
1.52E-40	4318.45286	4270.7814	4307.37833	7772.01561	7524.63882	8401.22883
6.67E-08	2124.21805	2149.77223	2128.3048	2668.66518	2748.24726	2741.20051
1.80E-05	466.401943	475.123099	455.923105	280.344625	319.721835	351.376296
0.00024113	3349.51129	3163.93633	3546.84285	4114.5965	4551.90601	3879.10123
1.61E-19	422.359145	439.968251	418.095424	203.788978	155.487823	175.300315
2.51E-13	826.649449	804.300314	844.154571	1394.17539	1429.51617	1206.15925
0.00012737	8037.24608	7560.42295	7794.49326	6764.93146	6556.72712	6614.87208
9.02E-06	3243.35686	3333.31878	2870.92191	2551.13609	2201.12449	2397.58086
0.04131465	3249.00337	3275.79267	3536.88819	3826.70413	3941.6163	3526.94927
0.07694545	8854.86111	9238.26797	10216.4603	8702.54412	8831.70832	7909.45715
0.00247868	8225.8396	8447.81654	8934.30103	9761.3842	10754.8983	9512.75694
0.00873841	517.220557	480.449591	470.855085	355.822024	390.663154	421.186156
0.00374684	2053.07199	2155.09872	2590.2007	2902.64512	3051.44852	2559.69487
1.83E-14	3101.06474	3428.13034	4028.64805	6653.87186	6774.41007	5549.10821
2.32E-08	2406.54368	2449.12109	2484.68138	1904.18695	1890.14884	1891.84721
5.73E-19	2739.68793	2550.32444	3117.79731	5306.06116	6043.61731	4757.9298
0.00736027	3062.66845	2827.30203	2627.03291	3349.04002	3176.81058	3436.19645
8.41E-09	966.682962	935.33202	911.846211	691.157326	625.838486	616.653764
1.19E-15	1864.47847	1731.10995	1574.8261	2857.35868	2647.18018	3063.87719

2.48E-45	2547.7065	2909.33001	2817.16679	5466.72019	5564.52045	5495.58732
4.19E-12	398.643792	419.727581	391.217861	224.2757	225.457343	195.467608
8.77E-24	4638.04548	4661.74592	4351.17881	7100.26676	6830.77441	7232.30151
8.34E-08	38.3962859	51.1343246	37.8276812	95.9641217	112.728671	138.844055
3.71E-13	488.987994	488.971979	499.723578	781.730205	785.213504	871.847586
1.42E-05	386.221464	384.572733	293.662262	544.515522	502.420027	744.638508
0.05859122	3059.28054	3136.23857	2851.01261	2433.607	2446.98961	2990.18901
0.02983843	86.9562945	83.0932774	88.5964113	93.8076246	151.600627	131.087404
0.00226603	840.20108	808.561507	749.585367	966.110709	955.27831	999.056665
3.48E-60	3322.40803	3484.59116	3133.72475	1452.40081	1366.34924	1459.80174
2.21E-37	494.634506	478.318995	461.895897	132.624573	131.19285	159.011348
1.53E-16	2831.16143	2863.52218	2846.03528	4710.86795	4383.7848	4056.72854
1.02E-08	6957.63286	7214.20096	7777.57035	9952.2342	10233.0423	9216.45286
0.00566598	1402.59374	1448.80586	1446.41107	1716.57171	1588.89119	1699.48226
0.01047102	39.5255884	26.6324607	24.8866324	53.9124279	53.448939	60.5018788
0.00010975	1350.64582	1397.67154	1473.28864	1776.95362	1873.62826	1659.92334
0.00583493	2108.40782	1965.4756	1847.58359	1689.61549	1631.65034	1718.87389
4.17E-58	4032.73932	4317.65453	4305.3874	8227.0365	8641.23574	8834.04996
8.75E-31	993.786223	940.658513	939.719239	425.908181	472.294261	414.20517
2.00E-101	910.217836	806.430911	718.725943	69.0079078	76.7721124	47.3155718
7.92E-06	100.507925	69.2443979	102.532925	32.3474568	35.956559	41.110251
8.59E-176	3080.73729	2813.45315	2857.98086	11429.4347	12601.3162	11360.3912
7.07E-13	405.419607	394.160419	364.340298	711.644049	644.302665	845.474972
2.51E-58	980.234593	956.637989	763.521882	3382.46573	3012.57656	3947.35976
4.65E-42	911.347139	729.729424	638.093254	2465.95445	2396.45607	3008.80497
2.73E-76	1892.71103	1875.99053	1609.66738	4875.83998	4973.66673	4884.36321
3.96E-07	190.852127	183.23133	197.102129	321.318071	349.847601	289.323087
1.63E-09	1568.60121	1579.83757	1136.82137	3860.12984	3208.87994	4996.83465
8.94E-40	1309.99093	1269.83573	1298.08675	486.2901	496.589233	586.402825
0.00211539	64.370244	56.4608167	57.7369871	109.981353	82.6029058	116.349767
0.05113881	73.4046642	42.6119372	72.6689666	77.6338962	120.503063	89.977153
0.00029072	22.5860505	20.2406701	16.92291	51.7559308	39.8437545	66.7071997
5.75E-36	21.456748	36.2201466	23.8911671	290.048862	206.021365	260.623478
0.0111002	37.2669834	74.57089	47.7823342	91.6511275	78.7157102	107.041785
0.01993037	51.9479162	77.7667853	52.7596607	143.407058	57.3361346	127.209078
0.04363675	3.38790758	1.06529843	2.98639589	16.1737284	10.6897878	4.65399067
0.08495984	12.4223278	21.3059686	14.9319794	33.4257053	34.0129612	21.7186231
0.00639175	16.9395379	27.6977591	12.9410488	57.1471736	21.3795756	76.7908461
3.06E-10	7.90511768	13.8488796	11.9455835	73.320902	49.5617435	89.2014879
9.77E-09	97.1200173	106.529843	85.6100154	186.537001	197.275175	238.129189
1.18E-11	114.059555	104.399246	90.5873419	231.82344	221.570147	248.212836
1.87E-21	717.107104	765.94957	783.431188	391.404227	310.003846	357.581617
1.40E-05	972.329475	1135.60813	1083.06624	1404.95787	1403.2776	1367.49759

8.82E-05	11298.6718	11748.1111	12012.2797	14743.9708	14541.0268	13169.2423
0.00161038	921.510861	987.531644	797.367702	691.157326	571.417748	755.497819
0.0570219	902.312718	797.908523	846.145501	708.409303	738.567157	771.011122
1.53E-12	2022.58082	1970.80209	2014.82176	2699.93439	2798.78081	2769.12445
1.46E-16	767.925718	828.802178	808.31782	1304.68076	1317.7593	1249.5965
0.00114997	11657.79	10692.4003	9960.62575	8685.29214	8128.12593	9563.17517
2.67E-06	1088.64764	1052.51485	1028.31565	776.338962	814.367471	806.691717
1.58E-15	2101.632	2094.37671	1762.96904	1186.07341	981.51688	1155.74102
0.00123583	1533.59283	1616.05772	1728.12775	1308.99375	1416.88278	1268.98812
0.00148802	4645.95059	4076.89709	4075.43492	4855.35326	5385.70946	5129.47339
5.07E-26	3787.68067	3482.46056	3627.47554	5645.70945	5793.86499	5835.32864
2.05E-37	3640.87134	3663.5613	3549.82924	2050.82876	2033.97508	2122.21975
4.52E-11	3323.53733	3563.42324	3275.08082	2420.66801	2432.41262	2539.52758
2.46E-17	690.003844	818.149193	811.304216	1291.74177	1475.19072	1411.7105
7.44E-77	1567.47191	1522.31145	1512.11178	3656.34086	3499.44781	3745.68683
1.14E-06	1326.93047	1171.82827	1361.79652	935.919749	972.77069	832.288665
6.44E-13	7143.96778	7323.9267	6555.13897	4728.11993	4755.98377	5142.65969
5.15E-15	1309.99093	1333.75363	1517.08911	742.913257	873.647203	718.265894
0.03116425	1113.49229	1182.48126	1310.03233	996.301668	1100.07634	996.729669
6.49E-26	3041.2117	3316.27401	3074.9923	1697.16323	1689.95827	1897.27686
0.01832461	4385.08171	4328.30752	4059.50747	5075.31597	4441.12093	5187.64827
9.39E-16	6718.22073	6891.41554	6860.74682	9165.11275	9231.11767	9186.97759
2.22E-05	360.247506	357.940272	301.625985	215.649712	158.403219	234.250864
3.27E-65	116.31816	125.705215	122.442231	607.053939	554.897167	585.62716
3.73E-05	0	0	2.98639589	26.956214	24.2949723	15.5133022
7.80E-08	1238.84487	1127.08574	1194.55835	888.476812	808.536678	862.539605
3.36E-49	1385.6542	1710.86928	1735.09601	4246.14282	4381.8412	4031.13159
1.43E-38	10130.973	10538.9974	10700.2565	19001.9744	19162.9023	17454.792
0.00656275	507.056834	537.975707	575.378941	443.160158	450.914686	407.224184
0.00119908	3375.48525	3334.38408	2995.35507	2701.01264	2397.42786	2811.78603
8.58E-25	2025.96873	2122.07447	2250.74703	1265.86381	1218.63581	1172.80565
3.13E-18	9478.2361	9838.03099	10010.399	6914.80801	6923.0953	6514.03561
0.02571121	1303.21512	1414.71631	1629.57669	1834.1008	1917.35921	1519.52795
7.31E-26	1122.52671	1077.01671	1205.50847	2044.35927	2250.68623	2015.95363
6.99E-05	1992.08966	1871.72934	1942.15279	1610.90335	1589.86299	1535.81692
2.04E-20	150.197236	166.186555	136.378745	17.2519769	34.0129612	27.923944
8.11E-05	57.5944288	72.4402932	76.6508278	117.529093	139.93904	127.984744
1.78E-08	186.334917	189.62312	236.92074	379.543493	399.409344	335.862994
1.08E-05	256.351673	213.059686	237.916206	159.580787	108.841476	131.863069
0.00120894	229.248413	262.063413	239.907136	183.302255	169.093007	160.562678
4.11E-06	658.383373	614.677193	565.424288	374.15225	357.621992	463.847737
1.70E-25	4879.71622	5004.77202	4982.3038	3038.50444	3250.66729	3206.59957
5.58E-10	1745.90171	1695.9551	1522.06644	1045.9011	1004.84005	1193.74861

1.10E-08	5457.91911	5603.46974	6040.48341	7821.61505	7957.08932	7153.95933
4.73E-08	4243.91889	4423.11908	4072.44852	3163.58127	2754.07806	3293.47407
8.87E-12	399.773094	425.054073	421.08182	183.302255	199.218773	244.33451
1.00E-11	1424.05049	1314.57826	1330.9371	745.069754	734.679962	933.12513
1.66E-23	124.223278	105.464544	112.487578	11.8607341	11.6615867	7.75665112
1.79E-13	1674.75565	1510.59317	1452.38387	2263.24372	2347.86612	2403.78618
2.18E-24	2225.85528	2314.89349	2798.25295	5963.79278	6107.75603	4701.30625
3.37E-08	2776.95491	2590.80578	2524.49999	1952.70814	1834.75631	2045.4289
7.85E-07	516.091254	496.429068	355.38111	925.137263	639.44367	920.714488
1.57E-21	1443.24863	1301.79468	1285.1457	773.104217	692.89261	736.106192
2.61E-09	2083.56316	2123.13977	2048.66758	1603.35561	1554.87823	1503.23899
2.51E-68	1183.50905	1218.7014	1102.97555	361.213267	366.368182	382.4029
2.00E-09	3293.04617	3366.34304	3647.38484	4765.85863	5175.8009	4480.24169
0.0172115	649.348953	637.04846	535.560329	521.872302	384.832361	511.163309
2.00E-21	212.308875	185.361927	197.102129	56.0689251	49.5617435	36.4562603
1.84E-19	31.6204707	23.4365654	34.8412853	184.380504	175.895599	129.536074
0.00372676	2364.75949	2424.61922	2194.00551	1985.0556	1714.25324	2059.39087
6.44E-18	3077.34938	3123.45499	3161.59778	2116.60192	2163.22433	2014.4023
4.67E-09	2214.56225	2065.61365	1798.80579	1183.91692	1071.89418	1439.63445
3.35E-17	216.826085	221.582073	240.902602	555.298008	519.912407	441.353449
0.04232466	750.98618	882.067099	890.941439	968.267206	1040.79661	939.330451
3.29E-19	505.927532	545.432796	543.524051	264.170897	266.272896	242.007515
6.50E-16	2300.38925	2412.90094	2394.09404	3337.17929	3684.0896	3509.10897
4.15E-27	2394.12136	2236.0614	2485.67684	4312.99423	4406.13617	3935.72478
3.06E-06	970.07087	912.960753	1056.18868	1293.89827	1451.86754	1281.39877
2.66E-11	16721.5825	17114.0193	17270.3274	22812.5048	22236.7022	21354.8362
0.00737828	56.4651263	101.203351	65.7007095	44.2081909	31.0975645	43.4372463
0.08413262	921.510861	1015.2294	942.705635	922.980766	827.972655	778.767773
0.00070575	4231.49657	4462.53512	4457.69359	3753.38323	3754.05912	3822.47767
4.25E-25	2364.75949	2587.60988	2646.94222	1442.69657	1505.31648	1459.80174
9.12E-21	37759.3593	38246.3442	34100.6592	63325.5379	56555.7801	67268.0055
7.18E-05	534.160095	599.763015	547.505913	748.3045	738.567157	729.125206
0.0040875	2861.6526	2591.87108	2741.51142	3365.21375	3140.85402	3070.08251
5.85E-09	16.9395379	15.9794764	16.92291	101.355365	71.913118	55.8478881
4.27E-06	1376.61978	1418.97751	1474.2841	1001.69291	919.321751	1153.41402
8.25E-14	578.202893	637.04846	536.555794	283.579371	275.990885	335.862994
1.02E-23	6492.36022	6774.23271	6559.12083	4006.77164	3382.83194	3962.87306
1.16E-05	6941.82263	7509.28862	7301.73794	6148.17328	5571.32304	5892.72786
8.05E-26	3286.27035	3117.0632	3448.29178	5517.39788	5198.15227	5431.98278
6.69E-29	579.332196	695.639874	705.784894	1441.61832	1493.6549	1362.06794
0.02061467	2289.09622	2384.13788	2706.67014	2151.10587	2205.98348	1983.37569
9.07E-184	1354.03373	1510.59317	1358.81013	148.798301	135.080046	155.908688
1.04E-18	4409.92636	4125.90081	4304.39194	2667.58693	2944.55064	2774.55411

1.60E-30	4736.2948	4487.03698	3961.95188	9016.31445	8054.26921	9166.03463
3.62E-05	23.7153531	18.1100733	17.9183753	52.8341794	48.5899446	70.5855252
4.21E-11	1814.78916	1821.66031	1781.88288	2384.00756	2739.50107	2645.01803
6.53E-12	5428.55724	5088.93059	5322.75293	7011.85038	7113.56788	6915.83014
0.08000019	5856.5629	6035.9809	6364.00963	6511.54305	6917.26451	6500.07364
1.38E-15	8790.49086	9036.92657	9541.53486	12970.2519	13827.7264	12652.6493
9.07E-11	1955.95198	1929.25545	2102.4227	2829.32422	3110.72825	2712.5009
5.24E-18	7031.03753	6765.71032	7518.74938	10934.5186	11787.9206	10435.0228
1.07E-29	1322.41326	1278.35811	1248.31348	2456.25022	2225.41946	2520.91161
1.21E-05	584.978709	567.804063	589.315455	755.85224	763.833929	893.566209
0.00253152	7662.31764	7653.10391	6886.62891	6176.20774	6316.69279	6559.02419
2.10E-05	9270.44444	9444.93587	9580.358	7941.30064	8064.959	8036.66623
5.93E-09	5779.77033	5399.99774	5002.21311	3950.70272	3745.31293	4102.49278
0.03120694	3415.01084	3440.91393	3849.4643	3928.0595	4432.37474	3894.61453
0.00251414	216.826085	266.324607	343.435527	184.380504	202.134169	154.357357
6.67E-05	914.735046	960.899183	1004.42448	723.504783	786.185303	662.418006
1.31E-10	1477.1277	1345.47192	1428.4927	1015.71014	922.237148	973.459716
5.48E-05	3457.92434	3337.57998	2862.95819	2489.67592	2136.98576	2590.72147
1.68E-05	193.110732	177.904838	182.170149	115.372596	100.095286	108.593116
0.0481284	6717.09143	6823.23644	6422.74209	6087.79136	5925.05784	6223.1612
2.02E-33	6712.57422	7019.25135	7114.59047	11171.7333	11075.592	11009.7906
0.00011848	14374.8919	13651.7994	13375.0717	16035.7126	16056.0613	16407.6441
5.73E-63	1105.58717	1179.28536	1348.85548	3286.50161	3386.71914	3225.21554
0.00974553	667.417793	625.330178	641.07965	562.845748	499.50463	532.881932
3.09E-31	3496.32062	3310.94752	3478.15574	1842.72679	2031.05968	1955.45175
9.41E-35	924.898769	941.723811	855.104689	342.883042	403.29654	352.151961
6.91E-18	802.934096	761.688377	756.553625	417.282192	450.914686	403.345858
6.79E-80	3272.71872	3243.83372	3351.73165	1388.78414	1271.11295	1332.59266
1.70E-09	98.2493198	109.725738	117.464905	38.8169481	39.8437545	24.8212836
0.01420488	779.218743	808.561507	815.286077	686.844332	708.441392	633.718397
0.02086773	452.850313	464.470115	468.864154	539.124279	598.628117	529.779272
5.67E-10	85.826992	106.529843	98.5510643	32.3474568	28.1821678	27.923944
3.07E-07	152.455841	148.076482	171.220031	62.5384164	73.8567157	85.3231624
1.31E-09	1356.29233	1501.00549	1546.95307	2071.31548	2054.38286	2028.36427
0.00037677	295.877262	314.263036	303.616915	210.258469	220.598348	219.513227
4.31E-12	2391.86275	2469.36176	2721.60212	3664.96685	3963.96768	3552.54621
1.43E-05	735.175945	817.083895	798.363167	1079.32681	1034.96582	993.627009
6.60E-13	2985.87588	2967.92142	2853.00354	2058.3765	1851.27689	2101.27679
1.14E-07	728.400129	792.582031	878.995856	528.341794	528.658597	394.813542
0.00082061	5937.87268	6515.36519	6488.4428	5434.37274	5391.54025	5380.01322
4.78E-27	3220.7708	3418.54266	3377.61375	2054.0635	2081.59323	1998.88899
3.97E-22	1434.21421	1382.75736	1274.19558	736.443766	775.495515	771.011122
2.19E-57	5132.67998	5392.54065	5978.76456	1912.81294	2169.05513	1765.4138

2.22E-63	4706.93293	4655.35413	4962.3945	2258.93073	2210.84248	2088.09048
8.59E-05	1342.7407	1548.94392	1685.32275	2076.70672	2306.07877	1856.94228
6.09E-07	4112.9198	4215.38588	4380.0473	3475.1951	3376.02935	3225.21554
5.48E-08	11960.4431	11633.0588	12939.0579	16229.7973	18539.0074	15506.3213
3.77E-09	4369.27147	4435.90266	3881.31919	6838.25236	5492.60733	6994.17232
1.84E-06	824.390844	918.287246	718.725943	570.393488	495.617435	573.992183
5.08E-10	1387.9128	1324.16595	1262.24999	903.572292	822.141862	947.862767
1.33E-08	2038.39106	2109.29089	2246.76517	2802.368	2838.62456	2804.80505
0.01339047	398.643792	347.287288	410.131702	480.898857	458.689077	492.547346
1.31E-09	4067.7477	3936.27769	3908.19675	3058.99116	2962.04302	3114.29543
2.56E-24	811.968516	812.822701	871.032134	1430.83584	1555.85002	1494.70667
7.94E-36	1299.82721	1319.90475	1291.11849	2310.68666	2460.59479	2402.23485
1.69E-14	694.521054	786.19024	783.431188	449.629649	401.352942	432.045468
0.00062012	2304.90646	2482.14534	2520.51813	2045.43752	2087.42402	1988.80535
4.93E-29	2901.17819	3022.25164	2980.42309	4975.03885	4904.669	4658.64466
1.73E-49	4607.55431	4708.61906	4708.55085	8268.00995	8092.16937	8163.87531
4.41E-55	1849.79754	1835.50919	1631.56762	686.844332	634.584676	692.668945
2.56E-12	600.788944	649.832042	603.251969	984.440934	998.037461	924.592814
2.87E-05	5983.04478	5912.40628	5046.01358	7331.01195	6841.46419	7946.68908
1.63E-30	463.014036	447.42534	588.31999	1277.72454	1326.50549	1192.97294
8.78E-09	903.442021	837.324565	730.671527	536.967782	511.166217	540.638583
2.69E-19	1987.57245	2027.26291	2065.59049	1288.50703	1288.60533	1337.24665
8.32E-08	6256.336	6514.29989	7101.64942	4921.12642	5255.4884	4678.03629
0.0585046	3278.36523	3092.56134	2824.13504	2665.43044	2426.58183	2940.54644
2.36E-10	1053.63926	1072.75552	1067.1388	1436.22708	1537.38585	1516.42529
3.77E-07	1410.49886	1221.8973	1285.1457	1716.57171	1739.52002	1852.28829
0.01145578	988.13971	942.789109	1043.24763	1156.9607	1147.69449	1166.60033
4.74E-17	5939.00199	6094.57231	6291.34067	9144.62603	8712.17706	8474.91702
2.96E-11	2432.51764	2758.05763	2593.18709	1808.22283	1851.27689	1832.121
2.39E-06	1115.7509	1215.50551	1170.66719	1459.94855	1631.65034	1532.71426
2.56E-49	3072.83217	3025.44754	3054.08753	1456.7138	1532.52685	1360.51661
0.00498713	3203.83127	3351.42886	3538.87913	4012.16289	4107.79391	3675.87697
1.75E-35	1038.95832	1073.82082	1233.3815	472.272869	466.463468	451.437095
0.00079979	22.5860505	28.7630576	14.9319794	3.23474568	2.91539667	6.98098601
4.02E-13	1672.49704	1803.55024	1538.98935	1040.50986	1067.03518	1106.87412
3.53E-13	8315.0545	7924.75501	7887.07154	5916.34984	5806.49838	6110.68975
0.00180693	6187.44854	5714.26077	5760.75766	4826.24055	5042.66445	5263.66345
6.26E-08	6595.12675	6810.45286	6616.85782	5233.8185	4499.42887	5210.14256
2.07E-06	112.930253	95.8768586	114.478509	196.241238	258.498505	179.954306
6.01E-12	327.497733	313.197738	284.703075	143.407058	147.713431	161.338343
0.01359215	3259.16709	3281.11916	3135.71568	2793.74202	2909.56588	2904.86585
1.67E-08	6946.33984	6808.32226	7139.4771	8993.67123	8765.626	8543.95121
1.32E-06	2517.21533	2510.9084	2047.67211	3563.61149	2992.16879	3777.4891

0.00050671	2099.3734	2247.77968	2411.01695	1856.74402	1907.64122	1732.0602
1.95E-13	71.1460592	76.7014869	87.600946	16.1737284	11.6615867	7.75665112
1.98E-17	30.4911682	45.8078324	42.8050077	157.42429	155.487823	164.441004
4.67E-26	138.904211	139.554094	131.401419	14.0172313	19.4359778	15.5133022
5.10E-05	1800.10823	1595.81705	1544.96214	1200.09065	1113.68153	1359.74094
2.96E-10	1130.43183	1255.98685	1170.66719	1617.37284	1734.66102	1818.93469
0.00193695	238.282833	273.781696	232.938879	170.363272	152.572426	193.140613
3.86E-11	527.38428	586.979434	592.301851	872.303084	878.506198	888.912219
0.02501138	20.3274455	14.914178	11.9455835	4.31299423	1.94359778	7.75665112
1.08E-18	4967.80181	5013.29441	5333.70305	7422.66308	7678.18304	7307.54102
0.03470588	344.437271	380.311539	377.281347	415.125695	457.717278	451.437095
0.07049053	223.6019	264.19401	230.947949	276.031631	312.919243	284.669096
5.39E-06	4412.18497	4490.23288	4298.41915	5232.74026	5354.61189	5344.33262
5.38E-16	31.6204707	43.6772356	44.7959383	157.42429	144.798035	152.806027
2.94E-07	71.1460592	73.5055916	80.6326889	28.0344625	27.210369	17.0646325
0.00064025	189.722824	182.166031	200.088524	130.468076	117.587666	130.311739
3.57E-14	175.041892	257.80222	256.830046	601.662696	586.96653	473.155718
1.65E-13	10202.119	9889.16531	8959.18766	6551.43824	6154.40238	6899.54117
2.64E-06	438.16938	434.641759	464.882293	686.844332	684.146419	580.973169
0.02227443	8562.37175	8908.02546	9212.03584	8169.88933	8168.94148	7304.43836
7.46E-08	1939.01244	2049.63418	2172.10527	1575.32114	1516.97807	1520.30362
1.41E-118	1705.24681	1590.49055	1810.75137	380.621741	389.691355	398.691868
0.02881376	35.0083783	35.1548482	40.8140771	23.7214683	10.6897878	21.7186231
1.60E-13	1499.71375	1424.304	1600.7082	2214.72254	2347.86612	2199.01059
0.02822756	2300.38925	2170.0129	2205.95109	2423.90276	2515.01553	2514.70629
3.43E-17	89.2148996	78.8320837	81.6281542	250.153666	221.570147	232.699534
0.00327044	1645.39378	1656.53906	1623.6039	1485.82651	1289.57713	1358.96528
7.66E-30	10.1637227	19.1753717	6.96825707	158.502538	187.557186	155.133022
0.03621221	14.6809328	22.371267	28.8684936	6.46949135	6.80259224	13.1863069
2.72E-07	48.5600086	52.199623	33.84582	7.54773991	3.88719557	10.8593116
1.81E-15	407.678212	350.483183	331.489943	141.250561	115.644068	166.767999
1.08E-16	261.998186	290.826471	255.834581	538.046031	549.066374	510.387644
7.72E-09	3474.86387	3578.33742	3767.83614	2696.69965	2810.44239	2507.72531
5.27E-10	972.329475	874.61001	1056.18868	1670.20702	1656.91711	1365.1706
2.94E-10	1043.47553	1055.71074	1165.68986	746.148003	723.018375	734.554861
0.00014646	8329.73543	8355.13558	7715.85151	6712.09728	6217.56931	7067.08484
0.03362448	691.133146	757.427183	682.889193	585.488967	620.979492	616.653764
4.59E-10	5731.21032	5832.5089	5121.66895	3894.63379	3566.50193	4132.74372
0.00020043	129.869791	118.248126	117.464905	170.363272	203.105968	210.205245
4.92E-30	1133.81974	1313.51296	1095.01183	2463.79796	2340.09173	2683.80129
0.00672654	6824.37517	5820.79061	6544.18885	6965.48569	8327.3447	7643.40402
2.33E-22	645.961045	581.652942	636.102324	1300.36776	1130.20211	1406.28085
2.68E-19	1206.0951	1199.52603	1317.00059	1978.58611	2025.22889	1974.84338

4.25E-19	1674.75565	1578.77227	1526.0483	2482.12818	2772.54224	2544.95723
7.28E-10	1649.91099	1782.24427	2042.69479	2826.08947	2906.65048	2565.12453
0.03623303	403.161002	478.318995	489.768925	526.185297	611.261503	528.227941
1.60E-06	1281.75837	1216.57081	1225.41778	1536.5042	1706.47885	1634.32639
2.82E-07	990.398316	1066.36373	929.764586	670.670604	633.612877	747.741168
0.00630508	9727.81196	9581.29407	8336.02638	7390.31562	6820.08462	8526.88658
0.06676044	354.600993	330.242513	314.567033	376.308747	427.591512	383.178565
7.17E-07	3529.07039	3903.25344	4230.72751	2899.41037	3000.91498	2621.74808
4.60E-08	3609.25087	3694.45495	3402.50038	4718.41569	4468.3313	4599.69412
3.69E-43	4587.22686	4853.49964	4890.721	8671.27491	8459.50935	8285.65473
0.00019791	11204.9397	10842.6074	11789.2955	9648.1681	9735.48129	8889.12219
0.00179637	187.464219	172.578345	170.224566	104.59011	120.503063	127.984744
2.19E-11	1517.7826	1565.98869	1453.37933	1055.60534	1046.62741	1063.43687
5.13E-07	808.580609	879.936502	768.499208	597.349702	539.348385	580.973169
1.15E-63	23396.8897	24172.6866	23489.9946	42127.1712	43138.1528	43241.7787
0.07148536	5.64651263	7.457089	11.9455835	17.2519769	29.1539667	12.4106418
7.46E-08	14.6809328	21.3059686	15.9274447	64.6949135	73.8567157	58.9505485
5.75E-09	403.161002	348.352586	274.748422	591.958459	589.881927	659.315345
2.09E-27	7443.23295	7873.62069	7927.88561	12244.5906	12647.9626	11832.7713
0.00195757	1450.02444	1428.56519	1276.18651	1134.31748	1080.64037	1187.54329
2.77E-09	3405.97642	3408.95497	3551.82017	2583.48355	2732.69848	2568.22719
1.28E-07	1534.72213	1619.25361	1563.87598	1161.2737	1216.69221	1191.42161
1.42E-41	2953.12611	2914.6565	2769.38445	1520.33047	1436.31876	1521.07929
0.00201858	4421.21939	4186.62283	4445.74801	3856.89509	3577.19172	3810.8427
1.73E-06	1103.32857	1100.45328	1036.27937	819.468905	692.89261	813.672703
7.44E-22	1497.45515	1538.29093	1693.28647	2699.93439	2905.67869	2594.5998
1.33E-13	2479.94835	2605.71996	2564.3186	3511.85556	3886.22377	3605.29144
4.65E-19	1302.08581	1448.80586	1540.98028	816.234159	789.1007	701.976927
0.0001394	6.77581516	8.52238743	14.9319794	36.660451	34.9847601	38.0075905
0.00084366	1492.93794	1515.91966	1600.7082	1174.21268	1343.02607	971.132721
8.24E-13	999.432736	927.874931	1010.39727	615.679927	634.584676	547.619569
0.00453991	250.705161	282.304084	282.712144	214.571463	208.936762	173.748985
5.63E-11	4424.6073	4767.21047	3989.8249	3061.14766	2701.60092	3025.8696
0.03623508	1439.86072	1676.77973	1870.47929	2009.85531	2023.28529	1841.42898
3.72E-10	338.790758	307.871246	350.403784	176.832764	184.641789	182.281301
1.02E-115	3331.44245	3280.05386	3460.23737	8457.78169	8312.76772	8640.90935
6.67E-05	2682.0935	2573.761	2579.25058	3102.1211	3090.32047	3382.67555
6.65E-05	553.358238	569.934659	503.70544	677.140095	782.298108	738.433187
0.03192214	9.03442021	0	1.99093059	17.2519769	11.6615867	14.7376371
1.96E-11	214.56748	166.186555	157.283517	62.5384164	56.3643357	72.9125206
2.88E-08	167.136774	158.729466	135.38328	77.6338962	58.3079335	64.3802043
0.0679711	1067.19089	1101.51858	1204.51301	993.066923	930.983338	1044.04524
3.13E-05	1015.24297	1130.28163	1090.0345	1356.43669	1472.27532	1345.0033

2.06E-83	2094.85619	2091.18082	2178.07807	4942.69139	5074.73381	4797.48872
0.02562094	1909.65057	1888.77411	1572.83517	1372.61042	1272.08475	1718.09822
1.29E-05	1307.73233	1226.15849	1153.74428	946.702235	905.716567	961.049074
6.54E-11	2756.62747	2754.86174	2517.53173	3575.47222	3735.59494	3925.64113
4.77E-35	1986.44314	1915.40657	2061.60863	3681.14058	3812.36705	3478.85803
0.00880853	42.913496	36.2201466	36.8322159	63.616665	75.8003135	62.053209
1.86E-05	4780.33759	4916.35225	4577.14943	4036.9626	3659.79462	3875.99857
1.77E-18	1724.44496	1795.02785	1882.42487	2841.18495	2995.08418	2713.27656
4.66E-25	3306.5978	3451.56691	2897.79948	1670.20702	1552.93463	1786.35675
0.0359816	20.3274455	34.0895497	28.8684936	18.3302255	12.6333856	3.10266045
3.82E-63	8793.87877	8716.27174	8602.81108	3945.31148	3358.53697	3775.1621
6.45E-41	399.773094	377.115644	401.172514	101.355365	88.4336991	103.939125
6.07E-21	4872.9404	4642.57055	5294.87991	8129.99413	8494.49411	7634.8717
0.00082262	430.264262	453.817131	541.533121	664.201112	588.910128	657.764015
1.96E-05	229.248413	239.692146	306.603311	424.829932	431.478708	368.440928
0.00042474	619.987087	581.652942	655.016164	470.116372	480.068652	480.91237
7.82E-25	962.165752	910.830157	954.651218	367.682759	465.491669	342.84398
2.37E-15	631.280112	566.738764	643.070581	1046.97935	1009.69905	1010.69164
0.00954112	2335.39762	2280.80394	2200.97377	2511.24089	2558.74648	2749.73282
3.04E-38	1103.32857	1048.25365	1056.18868	2076.70672	2074.79063	2002.76732
0.01608521	444.945195	434.641759	473.841481	538.046031	526.714999	570.113858
1.91E-20	1243.36208	1324.16595	1229.39964	734.287268	644.302665	728.34954
4.34E-34	4196.48819	3724.28331	3890.27837	2002.30757	2164.19613	1939.93845
1.29E-11	424.61775	396.291016	349.408319	679.296592	633.612877	754.722154
0.00599001	1660.07471	1762.0036	1513.10725	1394.17539	1128.25851	1435.75612
0.01293655	68.8874541	61.7873089	49.7732648	30.1909596	26.2385701	41.8859161
0.03060073	412.195422	416.531686	457.914036	355.822024	353.734796	359.132947
8.15E-19	769.05502	726.533528	792.390375	1286.35053	1429.51617	1274.41778
5.99E-12	1791.07381	1700.21629	1560.88958	2701.01264	2377.02009	2886.24988
0.00733487	79.0511768	75.6361884	56.7415218	133.702821	113.70047	94.6311437
4.10E-93	4939.56925	5294.53319	5258.04769	12247.8254	12252.4404	11731.1592
2.09E-14	1487.29143	1361.45139	1343.87815	2224.42678	2023.28529	2205.21591
0.00054706	115.188858	88.4197696	104.523856	58.2254222	63.1669279	42.6615812
5.73E-48	619.987087	606.154806	638.093254	1538.66069	1611.24256	1420.24282
8.93E-54	2884.23865	2891.21994	2906.75866	5271.5572	5397.37104	5411.03982
0.00097589	3597.95785	3636.92884	3297.97652	2903.72337	2550.97209	3077.83917
0.06462668	99.3786223	106.529843	75.6553625	74.3991506	37.9001568	72.9125206
1.09E-38	160.360959	136.358199	145.337933	470.116372	499.50463	526.676611
0.00623935	1028.7946	1090.86559	996.460761	1174.21268	1208.91782	1398.5242
0.00321308	1871.25429	1936.71254	1987.9442	1630.31182	1732.71742	1515.64963
0.00951939	1354.03373	1373.16967	1470.30224	1658.34628	1615.12976	1585.45949
1.29E-13	5478.24655	5567.24959	5893.15455	3814.8434	4176.79163	3770.50811
5.58E-15	18119.659	18019.5229	18041.813	12903.4005	13837.4444	13217.3335

2.15E-20	5119.12835	4828.99778	5166.46488	3442.84765	3267.18787	3306.66037
2.55E-15	1762.84124	1741.76293	1640.52681	1075.01381	930.011539	1106.09845
3.60E-05	56.4651263	39.4160419	37.8276812	91.6511275	111.756873	90.7528181
4.84E-11	85.826992	86.2891727	86.6054807	20.4867226	23.3231734	22.4942883
1.03E-35	12623.3436	12234.9525	12188.4771	7671.7385	7830.75547	7484.39267
0.0021971	2606.43023	2397.98676	2635.9921	2927.44484	3283.70845	2921.15481
3.11E-13	941.838307	950.246198	944.696565	1434.07058	1390.64421	1371.37592
3.60E-138	34486.6405	35554.3351	38482.6974	11017.5438	11396.2856	10285.3194
1.11E-13	8614.31967	8985.79225	9251.85446	6466.25661	6697.63796	6229.36652
5.48E-06	555.616843	633.852565	617.188483	830.25139	881.421594	789.627084
0.00086965	385.092161	469.796607	436.013799	546.672019	574.333145	576.319178
0.03452593	267.644699	233.300356	279.725748	360.135019	351.791199	282.342101
9.22E-24	1120.26811	1173.95887	1252.29534	2081.01972	1996.07492	2007.42131
1.94E-06	347.825178	243.95334	300.630519	157.42429	172.980203	174.52465
2.84E-49	1070.57879	1051.44955	1070.12519	2303.13892	2658.84177	2410.76717
1.34E-14	5486.15167	5829.313	5801.57174	4209.48237	3917.32133	3967.52705
1.74E-17	20799.4939	21972.8454	23877.2306	35468.9863	37296.6696	32691.1818
1.16E-58	686.615936	706.292858	672.93454	202.710729	172.008404	175.300315
2.12E-08	2256.34645	2357.50542	1880.43394	1441.61832	1051.4864	1408.60784
0.03785742	556.746145	537.975707	519.632884	653.418627	617.092296	613.551104
5.55E-05	1960.46919	1971.86739	1906.31604	2254.61774	2461.56659	2499.19299
0.00015108	657.25407	712.684649	564.428823	497.072586	443.140294	456.866751
0.00059465	4956.50879	4766.14517	4256.6096	3910.80752	3189.44396	3871.34458
0.01374313	912.476441	894.85068	1033.29298	869.068338	735.651761	733.003531
4.71E-05	6117.43178	5830.3783	6775.1368	7956.39611	8876.41107	7413.80714
1.37E-06	12.4223278	10.6529843	12.9410488	51.7559308	58.3079335	39.5589207
0.01827303	1305.47372	1372.10438	1329.94163	1569.9299	1607.35537	1441.96144
0.00073187	1295.31	1383.82266	1378.71943	1163.43019	1108.82253	1024.65361
3.25E-07	322.980523	409.074597	387.236	613.52343	628.753883	535.984593
6.03E-07	1332.57698	1292.20699	1194.55835	1771.56238	1627.76314	1692.50127
0.01232152	632.409415	769.145466	765.512812	611.366933	608.346106	526.676611
4.54E-09	499.151717	486.841382	517.641954	733.20902	853.239427	738.433187
0.00588245	482.212179	464.470115	459.904967	524.0288	639.44367	603.467457
0.07844765	1016.37227	1094.06149	1023.33832	1232.4381	1050.5146	1314.75237
7.59E-27	2839.06655	2857.13039	2973.45484	1789.89261	1712.30965	1639.75605
5.61E-13	1366.45606	1278.35811	1064.1524	2208.25305	1965.94916	2499.19299
5.33E-05	540.93591	544.367497	582.347198	417.282192	423.704317	376.197579
0.00046007	4171.64353	3992.73851	3993.80677	4524.33095	4907.5844	5004.5913
1.02E-07	1700.7296	1609.66593	1547.94853	2172.67085	2066.04444	2313.03336
1.00E-14	2339.91483	2486.40653	2125.31841	3539.89002	3442.11167	3696.04426
3.64E-28	539.806608	452.751832	453.932175	1107.36127	1071.89418	1292.25808
0.00029584	1511.00678	1483.96071	1271.20918	1743.52792	1756.0406	1963.98406
2.94E-20	1365.32675	1412.58572	1322.97338	2141.40164	2483.91797	2340.18164

3.26E-07	10558.9786	10079.8537	8636.6569	6732.584	6751.0869	7602.29377
3.74E-22	44034.8934	43515.3102	44506.2579	30714.9884	31890.5524	31267.8363
1.75E-71	7258.02734	7453.89311	7188.2549	17368.4278	15609.0338	17654.138
1.91E-14	2044.03757	2064.54836	2267.66994	3177.5985	3434.33728	3084.82015
1.21E-06	597.401036	566.738764	531.578468	782.808454	796.875091	774.113782
3.86E-07	309.428892	307.871246	395.199722	163.893781	215.739354	178.402976
5.14E-16	1740.25519	1701.28159	1767.94636	2553.29259	2537.36691	2483.67969
1.75E-36	224.731203	224.777968	232.938879	39.8951967	33.0411623	37.2319254
3.05E-08	485.600086	525.192125	540.537655	353.665527	292.511466	323.452352
4.21E-18	3771.87044	3733.87099	3544.85192	5886.15888	5272.00899	5847.73928
5.02E-26	1035.57042	1090.86559	1111.93474	1858.90052	1998.99032	1865.4746
0.0016769	62.1116389	63.9179057	51.7641954	28.0344625	34.9847601	21.7186231
0.00139493	198.757245	234.365654	268.77563	168.206775	165.205812	124.106418
0.00133123	389.609372	429.315267	392.213326	574.706482	473.26606	554.600555
1.07E-40	4409.92636	4589.30563	4217.78646	2234.13101	2301.21977	2420.85082
3.34E-18	737.43455	651.962638	617.188483	345.039539	310.975645	335.862994
4.90E-36	2407.67299	2400.11736	2415.99427	1283.11578	1368.29284	1317.07936
7.32E-13	4137.76446	4065.1788	3944.0335	2919.8971	2772.54224	3016.56162
2.11E-05	12528.4822	13493.0699	13064.4865	10965.7878	10742.2649	11063.3115
7.46E-06	5819.29592	5591.75145	5938.94595	4876.91823	4761.81457	4542.2949
1.55E-27	1201.57789	1140.93462	1083.06624	2021.71605	2019.3981	2157.90034
1.13E-09	1749.28961	1639.49428	1560.88958	1192.54291	1011.64265	1137.12505
8.34E-27	546.582423	596.56712	643.070581	245.840671	241.006125	205.551255
5.80E-33	2974.58285	2901.87292	2724.58851	1559.14742	1337.19527	1454.37209
3.17E-64	544.323818	580.587644	567.415218	105.668359	129.249253	108.593116
6.93E-09	64.370244	71.3749947	84.6145501	150.954798	177.839197	179.954306
1.58E-13	669.676398	657.289131	635.106859	403.264961	381.916964	373.870584
5.47E-13	499.151717	490.037277	482.800668	277.10988	279.878081	277.68811
0.00761361	2053.07199	2066.67895	1714.19124	1610.90335	1403.2776	1717.32256
0.0004283	8280.04612	8496.82027	8961.17859	7121.83173	7394.41776	7481.29001
0.00814312	2981.35867	3004.14157	2708.66107	2378.61632	2468.36918	2626.40207
2.26E-16	2125.34735	2036.8506	1650.48146	979.049691	844.493237	1082.8285
0.0014279	1880.28871	1735.37114	2037.71746	1499.84375	1632.62214	1474.53938
5.45E-16	3319.02012	3468.61168	3362.68177	2217.95729	2266.23501	2416.19682
4.11E-24	6086.94062	6044.50329	5863.29059	3613.21092	3285.65205	3815.49669
0.00041538	1094.29415	1113.23686	1155.73521	912.198281	835.747047	942.433111
0.00294952	3110.09916	3104.27962	2888.84029	2632.00473	2303.16337	2676.8203
2.13E-25	793.899676	855.434638	842.16364	1479.35702	1451.86754	1486.95002
0.01031011	33.8790758	41.5466387	42.8050077	23.7214683	17.49238	16.2889674
7.43E-15	1181.25044	988.596942	1216.45859	552.063262	670.541235	542.189913
0.03632778	3721.05182	3910.71053	4040.59363	3467.64736	3558.72754	3518.41695
1.11E-43	2486.72416	2590.80578	2403.05322	1217.34262	1050.5146	1146.43304
1.00E-07	31.6204707	38.3507434	32.8503548	0	0.97179889	1.55133022

1.85E-09	498.022414	454.882429	520.62835	263.092648	321.665433	252.866827
4.14E-08	887.631786	858.630534	728.680596	520.794054	440.224898	563.132872
3.01E-07	856.011315	868.218219	991.483434	631.853655	655.964252	605.018788
0.02468048	1008.46716	1026.94769	910.850745	1166.66494	1065.09158	1213.9159
1.72E-15	152.455841	137.423497	137.374211	30.1909596	42.7591512	20.1672929
2.09E-19	342.178665	368.593256	314.567033	144.485307	131.19285	118.676762
0.0091469	3824.94766	3861.7068	3351.73165	4389.54988	4020.33201	4556.25687
1.58E-21	1374.36117	1303.92528	1129.85311	664.201112	640.415469	664.745001
7.29E-05	1483.90352	1590.49055	1282.1593	1052.37059	914.462757	1165.82466
2.06E-09	1176.73323	1266.63983	1303.06407	1718.7282	1806.57414	1700.25793
1.25E-15	2478.81905	2473.62295	2603.14175	1735.98018	1748.26621	1708.01458
0.04788814	5820.42522	6177.66559	6240.57194	5526.02386	5665.58754	5442.84209
1.14E-10	1623.93703	1693.8245	1803.78312	2401.25954	2450.8768	2318.46302
0.0040872	14.6809328	19.1753717	22.8957018	2.15649712	8.74619002	1.55133022
2.61E-07	918.122954	968.356272	938.723774	1340.26296	1204.05883	1296.91207
9.41E-74	2814.2219	2572.69571	2639.97396	1014.63189	988.319472	1068.09086
1.44E-13	865.045735	805.365612	730.671527	432.377672	355.678394	465.399067
0.02041931	1864.47847	1887.70882	1502.15713	1483.67002	1130.20211	1565.2922
1.67E-09	1137.20764	1205.91782	955.646684	678.218343	624.866687	732.227866
0.00657355	2133.25247	2150.83753	2184.05086	1899.87396	1862.93847	1905.03352
0.04225491	522.86707	509.212649	439.995661	428.064678	344.988606	428.167142
2.66E-06	1485.03282	1350.79841	1264.24093	961.797714	959.165506	1065.76386
3.99E-14	1581.02354	1793.96255	1707.22298	1142.94347	1040.79661	1026.98061
0.01338658	11.2930253	15.9794764	10.9501183	3.23474568	2.91539667	3.10266045
2.47E-10	1590.05796	1617.12301	1733.10508	2338.72112	2306.07877	2207.54291
1.69E-07	293.618657	265.259309	340.449131	160.659035	174.9238	112.471441
9.52E-58	919.252256	979.009256	942.705635	2367.83383	2141.84476	2268.82045
1.06E-15	975.717383	930.005528	892.93237	1492.29601	1488.7959	1416.3645
4.93E-24	1090.90624	1169.69767	1209.49033	1976.42961	1959.14656	1967.08672
7.81E-25	2981.35867	2976.44381	3350.73618	1767.24939	1726.88663	1538.91958
3.79E-07	677.581516	748.904795	653.025234	478.74236	457.717278	491.771681
0.00681476	41.7841935	56.4608167	36.8322159	17.2519769	22.3513745	24.0456185
2.14E-06	1160.923	1105.77977	1016.37007	1518.17397	1375.09543	1566.84353
5.89E-16	683.228028	528.388021	607.23383	299.753099	301.257656	285.444761
0.00916846	306.040985	322.785424	302.62145	251.231914	247.808717	224.167217
0.03007742	3527.94109	3708.30383	3939.05617	3202.39822	3524.71458	3097.23079
0.07997691	306.040985	362.201466	261.807373	411.890949	346.932204	376.973245
0.0265977	32.7497733	30.8936544	26.877563	62.5384164	45.6745479	48.091237
1.80E-25	1739.12589	1730.04465	1714.19124	1002.77116	1026.21963	1033.96159
7.90E-06	274.420514	287.630576	267.780164	150.954798	197.275175	139.61972
4.31E-08	8221.32239	9032.66538	9180.18095	6901.86902	6873.53356	6532.65158
1.73E-12	7379.99201	6841.34651	7073.77639	5185.29732	5377.93506	5107.75476
4.79E-09	1201.57789	1345.47192	1275.19104	908.963535	913.490958	827.634675

0.05768218	429.13496	430.380565	431.036473	383.856487	354.706595	360.684277
3.39E-08	1771.87566	1531.89914	1536.99842	973.658449	1206.97422	957.170749
0.01438708	403.161002	330.242513	296.648658	449.629649	397.465747	496.425672
0.0026993	29.3618657	19.1753717	20.9047712	39.8951967	71.913118	47.3155718
3.25E-08	142.292118	99.0727539	111.492113	48.5211851	43.7309501	38.0075905
7.05E-25	1540.36865	1625.6454	1599.71273	2711.79513	2575.26706	2885.47422
3.70E-07	1201.57789	1138.80402	1070.12519	844.268621	699.695202	820.653689
1.46E-26	197.627942	208.798492	199.093059	531.576539	474.237859	515.8173
7.20E-09	476.565666	450.621235	459.904967	658.809869	699.695202	730.676536
7.47E-09	4827.7683	4601.02391	4518.41698	5858.12442	6355.56475	5987.359
2.79E-13	968.941568	975.813361	975.55599	610.288684	637.500073	635.269727
4.00E-09	1180.12114	1135.60813	1034.28844	1634.62482	1521.83706	1798.7674
3.23E-06	205.53306	216.255581	205.065851	133.702821	117.587666	108.593116
5.36E-24	1429.697	1646.95137	1582.78982	768.791222	868.788209	771.786787
1.70E-09	1839.63382	1821.66031	1783.87381	2476.73694	2338.14813	2591.49714
2.11E-06	1596.83377	1752.41592	1867.49289	1290.66352	1269.16935	1348.88163
2.93E-22	828.908054	694.574576	808.31782	1453.47906	1611.24256	1445.83977
0.00022219	1241.10348	1175.02417	1053.20228	918.667772	829.916253	927.695474
0.00945552	23.7153531	7.457089	15.9274447	0	0	6.2053209
6.82E-09	903.442021	879.936502	881.982252	616.758176	601.543514	638.372387
2.14E-12	2082.43386	2199.84126	2292.55658	3185.14624	3066.0255	3032.07492
1.87E-71	1561.82539	1525.50735	1609.66738	3614.28917	4012.55762	3807.74004
1.34E-05	150.197236	169.38245	134.387815	268.483891	218.654751	292.425747
1.47E-09	778.089441	657.289131	646.056977	425.908181	426.619713	438.250788
9.05E-18	431.393565	386.70333	491.759856	194.084741	189.500784	181.505636
0.01534274	171.653984	165.121256	126.424093	86.2598847	81.6311069	131.863069
0.01784413	997.174131	910.830157	916.823537	818.390656	775.495515	819.878024
9.32E-20	1934.49523	1862.14165	2157.1733	1045.9011	1198.22803	1043.26958
0.00059813	16.9395379	34.0895497	26.877563	72.2426534	47.6181457	69.034195
0.00306146	33.8790758	36.2201466	37.8276812	65.7731621	68.9977213	65.1558694
0.00887904	0	0	0	7.54773991	3.88719557	3.87832556
0.03594012	258.610279	259.932817	317.553429	194.084741	234.203533	226.494213
1.02E-30	4336.5217	4607.4157	4816.0611	8499.83339	8573.20982	7689.94392
1.04E-09	263.127489	266.324607	238.911671	134.78107	130.221051	131.863069
1.13E-07	41.7841935	52.199623	48.7777995	9.70423703	10.6897878	9.30798135
2.74E-15	42727.1611	34351.6131	33458.584	19866.7297	21056.9384	22851.0942
0.02926579	610.952667	682.856293	693.839311	582.254222	562.671558	539.087253
7.22E-07	2093.72688	1997.43455	2150.20504	1599.04261	1659.83251	1596.3188
0.05966502	3308.8564	3089.36544	2961.50925	3292.9711	3468.35024	3682.08229
2.07E-36	2430.25904	2501.32071	2591.19616	4461.79254	4311.87168	4304.94137
9.13E-33	1647.65239	1625.6454	1623.6039	777.417211	884.336991	791.95408
9.60E-18	24.8446556	26.6324607	27.8730283	141.250561	118.559465	129.536074
1.67E-08	2929.41075	3127.71619	3165.57964	2379.69457	2371.18929	2240.12084

2.22E-20	17933.3241	18651.2449	18431.0399	12791.2627	11295.2185	11834.3226
0.00180085	760.0206	813.888	761.530951	622.149418	640.415469	634.494062
3.73E-42	1235.45696	1238.94207	1230.39511	516.48106	444.112093	531.330602
0.0069415	70.0167566	50.0690262	60.723383	24.7997169	33.0411623	37.2319254
4.76E-81	723.882919	756.361884	708.77129	2433.607	2110.74719	2373.53524
4.05E-22	5735.72753	6012.54433	6052.429	8995.82773	8942.4934	8566.4455
7.27E-11	343.307968	329.177215	334.476339	178.989261	191.444382	154.357357
0.01992477	4752.10503	4569.06496	4840.94773	5468.87669	5097.08518	5244.27182
0.00032103	835.683869	772.341361	677.911866	561.767499	468.407066	608.897113
0.02657259	28.2325632	28.7630576	27.8730283	12.9389827	15.5487823	12.4106418
6.10E-06	237.153531	271.651099	280.721213	164.972029	166.17761	154.357357
0.04430516	4580.45105	4582.91384	4665.74584	4229.9691	4041.71159	4323.55734
5.63E-09	7494.05156	7598.77369	7710.87418	6074.85238	6042.64551	5633.65571
0.00962116	315.075405	370.723853	392.213326	510.011568	474.237859	414.20517
3.38E-17	669.676398	690.313382	595.288247	349.352533	274.047287	313.368705
0.0169653	16.9395379	12.7835811	11.9455835	1.07824856	4.85899446	4.65399067
2.52E-15	204.403757	224.777968	221.988761	81.9468905	84.5465035	80.6691717
1.22E-08	1029.9239	954.507392	1053.20228	1485.82651	1700.64806	1371.37592
0.03536725	5102.18881	4544.5631	4150.09482	3696.23606	3562.61474	4422.06681
5.55E-06	7597.9474	8787.64674	9406.15158	11744.2833	11698.5151	10783.2964
1.42E-20	3480.51039	3480.32997	3768.83161	5527.10211	5632.54637	5313.30602
0.00358335	1218.51743	1018.4253	1157.72614	863.677096	1002.89646	824.532014
1.70E-07	665.159188	664.74622	664.970817	902.494044	964.0245	898.995865
1.00E-21	301.523775	252.475728	297.644123	79.7903933	100.095286	88.4258228
7.44E-52	1465.83468	1377.43087	1624.59936	433.455921	530.602195	433.596798
2.51E-11	8596.25083	9129.60753	9738.63699	6760.61846	6590.74008	6110.68975
2.07E-31	328.627035	313.197738	367.326694	99.1988674	88.4336991	81.4448368
1.18E-18	1007.33785	1090.86559	1154.73974	1975.35136	1934.85159	1739.04118
4.87E-18	596.271734	623.199581	598.274643	1002.77116	1043.71201	1018.44829
1.65E-05	4631.26966	4516.86534	4093.35329	5804.21199	5281.72697	5575.48083
6.24E-14	1134.94904	1181.41596	1134.83044	1657.26803	1764.78679	1692.50127
1.59E-24	3747.02578	3705.10794	3809.64569	5563.76256	5657.81315	5629.00172
2.83E-07	3728.95694	3529.33369	2928.6589	4856.43151	4590.77796	5711.99789
0.00202756	7594.55949	7190.76439	6675.59027	8117.05515	8293.33174	9460.01171
9.48E-13	4579.32174	4562.67317	4568.19024	6062.99165	5937.69123	6158.78099
4.97E-09	3023.14286	3166.06693	3117.79731	2455.17197	2150.59095	2158.67601
6.60E-07	6988.12403	7043.75321	7315.67446	8605.50175	9119.3608	8618.41506
1.14E-09	19433.0379	20459.0563	20567.3085	16045.4168	16030.7945	15635.0817
9.98E-12	5536.97029	5700.41189	5604.46961	4367.98491	4201.08661	4168.42431
1.85E-29	1401.46444	1585.16406	1582.78982	3386.77872	2940.66345	2914.94949
0.05495468	1365.32675	1420.04281	1627.58576	1691.77199	1843.5025	1566.06786
3.39E-06	2688.86931	2858.19568	2990.37775	2233.05277	2311.90956	2160.22734
0.00206526	138.904211	172.578345	151.310725	207.023723	253.639511	215.634901

0.00030634	2346.69065	2387.33378	2341.33437	2694.54315	2854.17334	2836.60732
1.03E-28	4228.10866	4239.88775	4320.31938	2483.20643	2604.42103	2290.53908
8.96E-08	12.4223278	11.7182827	10.9501183	52.8341794	43.7309501	70.5855252
8.62E-31	2548.8358	2695.20502	2827.12144	4775.56287	5237.99602	4709.0629
0.01232483	1678.14355	1676.77973	1412.56525	1302.52426	986.375875	1431.8778
6.44E-10	1367.58536	1317.77416	1228.40417	1888.01323	1766.73038	2020.60762
1.40E-05	48.5600086	42.6119372	52.7596607	11.8607341	12.6333856	17.8402976
5.23E-09	1271.59464	1298.59878	1336.90989	981.206188	887.252388	915.284832
8.02E-54	3435.33828	3732.80569	3395.53212	7932.67465	7151.46804	7579.02381
6.56E-17	4460.74498	4732.05562	4678.68689	3302.67534	3123.36164	3139.11671
0.0033152	1028.7946	985.401047	867.050272	759.086985	774.523716	801.262061
1.45E-12	10206.6362	10085.1802	9987.50331	13714.2434	12872.4481	13880.5272
1.72E-12	1019.76018	981.139853	804.335959	462.568632	305.144852	483.239365
7.24E-13	383.962859	344.091393	346.421923	156.346041	196.303376	160.562678
0.00122568	7938.99676	7351.62446	6457.58337	5165.88884	5792.89319	6298.40071
0.04363675	3658.94019	3381.25721	2557.35034	2381.85107	2271.09401	3077.0635
0.00271009	1638.61797	1746.02412	1972.01675	2189.92282	2198.20909	2095.84713
0.00074668	1811.40125	1639.49428	1824.68789	2067.00249	2179.74491	2110.58477
0.03378527	957.648542	922.548439	783.431188	670.670604	679.287425	836.166991
0.00412429	1350.64582	1424.304	1292.11395	1158.03895	1086.47116	1172.80565
2.33E-10	2601.91302	2366.02781	2604.13721	1661.58103	1874.60006	1756.88148
0.00019577	5697.33124	5526.76825	5637.31997	4627.84281	4893.97922	4851.78528
1.91E-10	166.007471	157.664167	160.269913	319.161573	309.032047	289.323087
8.68E-11	808.580609	707.358157	624.15674	410.812701	359.56559	425.064482
1.57E-18	1667.97983	1748.15472	1723.15043	2661.11744	2519.87453	2617.86975
2.30E-05	2735.17072	2721.83749	2149.20957	3599.19369	3170.00798	3782.14309
2.51E-20	2402.02647	2530.08377	2194.00551	4127.53548	3772.5233	4329.76266
2.22E-05	625.6336	671.13801	659.993491	974.736697	996.093864	789.627084
3.51E-05	1618.29052	1613.92712	1525.05283	1280.95929	1257.50777	1281.39877
0.00878793	3817.04254	3794.593	4176.97238	3359.82251	3556.78394	3371.81624
0.00192486	3032.17728	3183.11171	3593.62972	2651.41321	2873.60932	2399.13219
5.31E-14	11011.8289	10950.2025	11685.7671	16450.8383	17298.0203	15112.2834
0.05515601	409.936817	414.401089	404.15891	493.83784	476.181457	466.174732
4.98E-22	713.719197	813.888	868.045738	363.369764	351.791199	278.463775
1.13E-19	4291.3496	3951.19187	3779.78173	2514.47564	2546.1131	2566.67586
2.63E-10	827.778752	1033.33948	1120.89392	1959.17763	1694.81727	1552.88155
5.78E-34	1002.82064	944.919706	950.669357	458.255637	419.817121	407.224184
0.00962559	347.825178	345.156691	322.530756	252.310163	251.695913	285.444761
4.44E-21	2493.49998	2247.77968	2177.0826	3930.216	3711.29997	4201.00225
6.51E-08	1411.62816	1260.24804	1128.85765	768.791222	724.961973	916.060498
0.00184143	2501.4051	2625.96063	2879.8811	3325.31856	3531.51717	2969.24605
9.91E-14	631.280112	775.537256	705.784894	406.499707	377.05797	383.178565
0.00699982	0	0	0.9954653	5.39124279	9.71798891	7.75665112

1.53E-16	512.703347	458.078324	457.914036	191.928243	188.528985	245.110175
2.26E-13	1325.80117	1227.22379	1067.1388	2065.92424	1932.90799	2439.46678
0.00318555	476.565666	454.882429	405.154375	588.723713	542.263781	565.459867
0.00967034	4042.90304	4059.85231	3851.45523	3540.96827	3534.43257	3561.8542
4.27E-40	1460.18817	1503.13608	1373.74211	3499.99482	2981.479	3648.72869
1.85E-12	1553.92028	1413.65102	1349.85094	2554.37084	2075.76243	2636.48572
4.47E-08	1273.85325	1330.55774	1473.28864	988.753928	978.601484	911.406507
0.0097812	154.714446	183.23133	196.106663	143.407058	108.841476	121.779423
3.09E-09	2619.98186	2614.24234	2814.18039	3492.44708	3529.57357	3556.42454
0.06706621	17552.7492	17502.8532	16130.5196	15627.0564	13221.3239	16382.0472
0.00046153	3376.61455	3365.27774	3722.04474	4203.01288	4782.22234	4028.02893
0.08207707	809.709911	883.132397	848.136432	980.12794	938.757729	934.67646
4.46E-13	292.489354	268.455204	350.403784	583.33247	635.556475	553.049225
4.70E-09	789.382466	713.749947	794.381306	1075.01381	1122.42772	1100.66879
1.18E-20	2064.36502	2058.15656	2021.79002	1278.80279	1085.49936	1215.46723
0.07159952	143.421421	158.729466	157.283517	122.920336	125.362057	111.695776
6.09E-45	12813.0665	12573.7174	12526.9353	7685.75573	7468.27448	7511.54095
4.96E-15	11194.7759	12437.3592	14162.4848	23392.6025	23612.7695	19406.3654
1.82E-14	3884.80069	4034.28515	3805.66382	2812.07224	2657.86997	2806.35638
1.39E-09	6664.01421	6700.72712	7242.01002	9516.62178	9552.7831	8684.3466
0.00519238	1127.04392	1162.24059	1208.49487	1025.41438	976.657886	954.843753
2.75E-26	771.313625	735.055916	686.871054	1327.32398	1434.37516	1398.5242
1.02E-22	1718.79844	1725.78345	1628.58122	2964.10529	2652.03917	2949.85442
0.00019067	237.153531	263.128712	298.639589	445.316655	413.014529	341.292649
1.11E-05	937.321097	926.809633	856.100154	1134.31748	1189.48184	1315.52803
1.69E-16	477.694969	434.641759	488.77346	898.181049	895.026779	785.748759
1.22E-10	1629.58355	1568.11929	1479.26143	1054.52709	1017.47344	1127.81707
3.39E-07	1037.82902	990.727539	1047.22949	791.434442	685.118218	729.125206
0.00035866	406.548909	376.050345	361.353902	501.38558	499.50463	515.041635
5.67E-05	1532.46353	1458.39355	1597.7218	1851.35278	2173.91412	1901.15519
1.06E-34	1014.11367	977.943958	890.941439	2113.36718	1901.81043	2178.06764
2.84E-10	633.538717	650.89734	642.075116	935.919749	945.560321	936.227791
2.11E-40	77.9218743	90.5503664	67.6916401	412.969198	344.988606	390.159551
2.24E-17	606.435457	693.509277	628.138601	1115.98726	1079.66857	1207.71058
1.46E-27	1350.64582	1336.94953	1395.64234	733.20902	696.779805	758.60048
2.71E-61	1272.72395	1281.55401	1360.80106	2922.05359	2900.81969	2828.85066
2.24E-13	275.549816	329.177215	322.530756	150.954798	133.136448	139.61972
3.76E-08	1142.85416	1075.95141	990.487969	672.827101	550.038172	757.04915
0.01171139	400.902397	379.246241	352.394715	312.692082	310.003846	267.604464
1.73E-23	1748.16031	1808.87673	1568.85331	941.310992	901.829371	961.049074
0.00481025	30.4911682	28.7630576	35.8367506	54.9906765	85.5183024	50.4182323
9.14E-18	2658.37815	2678.16025	2821.14865	1856.74402	1682.18388	1689.39861
8.44E-74	4875.19901	4986.66195	4587.10408	10489.202	10443.9227	11071.0681

1.36E-25	1037.82902	1090.86559	1165.68986	2166.20135	1987.32873	1939.16278
1.48E-50	673.064306	673.268607	598.274643	1840.57029	1649.14272	1991.90801
4.61E-19	7550.51669	7351.62446	7010.06661	4937.30015	4617.98833	5013.89929
1.19E-13	2330.88041	2277.60804	2400.06683	3219.6502	3417.8167	3247.70983
3.34E-15	3640.87134	3577.27212	3774.8044	2284.8087	2586.92865	2485.23102
0.00497358	171.653984	159.794764	175.201892	216.72796	261.413902	223.391552
0.00018422	318.463312	318.52423	354.385645	210.258469	254.61131	214.083571
0.00013438	1581.02354	1574.51108	1641.52227	1343.4977	1287.63353	1293.80941
1.33E-14	120.83537	80.9626806	82.6236195	16.1737284	14.5769834	14.7376371
3.41E-105	1679.27286	1543.61742	1515.09818	394.638972	347.904003	387.832556
0.00071587	521.737767	528.388021	542.528586	411.890949	383.860562	428.167142
0.00126998	2382.82833	2269.08565	2217.89668	2012.01181	1852.24869	1929.8548
4.31E-11	5845.26988	5884.70852	4778.23342	3554.9855	2900.81969	3575.0405
0.0001452	2852.61818	2771.90651	2788.29829	2186.68808	2404.23046	2359.57327
9.41E-26	5876.89035	6016.80553	6044.46527	9007.68846	9053.27847	8752.60513
3.13E-05	3059.28054	2966.85612	2577.25965	2183.45333	1973.72355	2331.64933
2.14E-15	1682.66076	1691.6939	1491.20701	3063.30416	2550.97209	3467.22305
0.00156563	73.4046642	99.0727539	62.7143136	169.285024	116.615867	127.984744
0.06972085	1141.72485	1004.57642	858.091085	827.016645	650.133458	962.600404
0.00166359	380.574951	310.001843	252.848185	174.676267	139.93904	251.315496
1.51E-10	1062.67368	1087.6697	1155.73521	1615.21634	1641.36833	1508.66864
2.43E-16	3840.75789	3799.9195	3707.11276	2344.11237	2629.6878	2579.86216
1.05E-05	1482.77422	1496.74429	1421.52444	1213.02963	1068.97878	1106.87412
0.02473988	1760.58264	1685.30211	1747.04159	1522.48696	1569.45521	1517.20096
1.21E-15	361.376808	394.160419	379.272278	163.893781	183.66999	186.159627
1.08E-06	142.292118	150.207078	183.165614	83.025139	70.9413191	80.6691717
2.56E-26	719.365709	737.186513	823.249799	347.196036	343.045009	351.376296
2.08E-15	1077.35461	1124.95514	1196.54929	1855.66577	1688.98647	1818.93469
5.54E-05	2080.17525	2100.7685	2169.11888	1757.54515	1588.89119	1756.88148
0.00323963	90.3442021	82.027979	111.492113	161.737284	129.249253	153.581692
4.93E-06	310.558195	340.895497	302.62145	228.588694	165.205812	149.703367
3.22E-10	2330.88041	2360.70132	2212.91935	1723.0412	1484.90871	1617.26176
3.17E-07	273.291211	216.255581	230.947949	125.076833	118.559465	141.946716
3.99E-12	1061.54437	1081.27791	1233.3815	1847.03978	1946.51318	1643.63437
9.46E-05	254.093068	239.692146	176.197357	153.111295	98.151688	118.676762
0.01134476	156.973051	148.076482	135.38328	197.319486	197.275175	200.121599
4.04E-06	5264.80838	5345.66752	5694.06149	4554.52191	4279.80232	4048.97189
1.09E-13	271.032606	306.805947	350.403784	135.859318	137.023644	134.96573
1.72E-45	2437.03485	2500.25541	2256.71982	1111.67426	1129.23031	1163.49767
0.03268749	17555.0078	17055.4278	15614.8686	18257.9828	18265.932	19628.9813
1.48E-12	2222.46737	2143.38044	1850.56998	1262.62906	1221.55121	1386.11356
4.06E-05	28128.6673	26741.1212	25693.9547	22701.4452	21418.4476	22851.8699
2.51E-05	12228.0878	12591.8274	11053.6466	9643.85511	8874.46748	9966.52103

1.58E-11	310.558195	307.871246	379.272278	588.723713	632.641078	567.786862
1.37E-98	6068.87178	5967.8018	6068.35644	2639.55247	2587.90045	2536.42492
1.28E-07	1474.8691	1673.58383	1745.05066	1167.74319	1206.97422	1135.57372
7.54E-13	720.495012	804.300314	808.31782	491.681343	439.253099	459.193746
1.79E-18	1142.85416	1434.95698	1377.72397	708.409303	534.48939	588.72982
1.37E-19	1832.858	1944.16963	2053.6449	3089.18212	3110.72825	2989.41334
1.39E-13	545.45312	526.257424	641.07965	1062.07483	1130.20211	929.246805
9.32E-05	846.976895	653.027937	746.598972	1077.17031	983.460478	999.056665
0.04370221	635.797322	597.632419	564.428823	514.324563	395.522149	549.946565
5.60E-36	660.641978	802.169717	685.875589	1622.76408	1585.97579	1635.10206
2.75E-29	1817.04776	1889.83941	1729.12322	3165.73777	3030.06894	3154.63001
0.0502949	6863.90075	7800.1151	7717.84244	6675.43683	6403.18289	6960.81872
1.81E-14	3489.54481	3508.02773	3682.22613	5201.47105	5213.70105	4822.31
4.97E-23	2040.64966	2074.13604	2181.06446	3587.33295	3944.5317	3382.67555
0.02689022	2308.29436	2333.00356	2015.81722	1884.77848	1580.145	2078.7825
1.59E-14	1892.71103	1964.4103	1778.89648	2759.23806	2690.91113	2838.15865
8.33E-09	1887.06452	2032.5894	1807.76498	1284.19403	1354.68765	1435.75612
2.41E-10	2316.19948	2371.3543	2301.51576	3150.64229	3005.77397	3240.72884
5.18E-251	6852.60773	7067.18978	6933.41578	23636.2867	23087.9981	23750.0901
1.12E-23	316.204707	324.916021	338.4582	650.183881	702.610598	698.098601
1.78E-16	412.195422	434.641759	407.145306	187.615249	178.810996	214.859236
6.92E-22	3303.20989	3438.78333	3475.16935	2241.67875	2008.70831	2088.86615
7.72E-10	11605.8421	11660.7566	11098.4426	8579.62378	8987.19615	9097.00044
3.39E-18	1736.86729	1736.43644	1510.12085	861.520598	695.808006	921.490153
3.25E-12	1010.72576	1022.68649	907.864349	1422.20985	1507.26008	1560.63821
4.08E-05	528.513582	541.171602	546.510447	672.827101	728.849168	753.170824
6.76E-07	3277.23593	3514.41952	3660.32589	4493.06174	4350.74364	4440.68277
3.04E-05	8750.96528	8730.12062	9872.02934	7262.00404	7610.15712	6606.33976
1.05E-59	5810.2615	6056.22157	5696.05242	10942.0664	11210.672	11271.1897
0.00099597	12400.871	12198.7323	10139.8095	9024.94044	8984.28075	9978.93167
9.37E-20	4592.87337	4932.33173	5063.93196	7519.70545	7477.99247	7130.68938
9.16E-06	284.584237	268.455204	320.539825	416.203944	542.263781	425.840147
9.77E-34	1131.56113	1247.46446	1121.88939	502.463828	394.55035	480.91237
0.00790931	2246.18272	2158.29462	1843.60173	1682.06775	1664.6915	1839.87765
4.01E-25	2923.76424	2812.38785	3079.96962	5054.82924	5305.05015	4699.75492
4.26E-42	23554.9921	24631.8303	22541.3162	42003.1726	41284.9323	44528.6071
5.03E-10	3717.66392	3794.593	3510.01063	4948.08264	4773.47615	5310.20336
1.61E-19	5887.05407	5972.06299	5630.35171	8652.94468	8381.76544	9168.36163
0.01478146	5401.45398	5197.59103	5657.22927	5838.71595	6852.15398	6084.31714
2.86E-12	2283.44971	2237.1267	2374.18473	1565.61691	1687.04288	1597.09447
3.06E-10	1049.12205	1112.17156	949.673892	1519.25222	1527.66786	1768.51646
5.70E-19	1964.9864	2223.27782	2078.53154	3476.27335	3209.85174	3322.94934
0.03276558	2972.32425	2671.76846	2931.6453	3152.79879	3301.20083	3167.81632

0.00019879	12653.8348	12694.0961	13106.2961	15196.8352	15016.2365	14428.9224
1.75E-45	2324.1046	2227.53901	2064.59502	4863.97925	4548.01881	5253.57981
0.00031765	3959.33466	4027.89336	4033.62538	4634.31231	4707.39383	4610.55343
0.00210212	4613.20082	4347.48289	4302.40101	5081.78546	4960.06154	5277.62542
0.0022957	3830.59417	3764.76465	3600.59797	4173.90017	4514.97765	4279.34442
0.00036523	65.4995465	74.57089	78.6417583	35.5822024	37.9001568	33.3535998
0.00125395	1873.51289	1804.61554	1818.71509	1425.44459	1618.04515	1528.06027
6.60E-06	2047.42548	1985.71627	1966.04396	2454.09372	2429.49723	2570.55418
0.03082529	1141.72485	1113.23686	1146.77602	1250.76833	1264.31036	1350.43296
0.00025579	7425.16411	8015.30538	7402.27994	9151.09552	8658.72812	9354.52125
1.41E-31	867.30434	784.059644	924.787259	1952.70814	1995.10312	1718.87389
4.63E-06	627.892205	649.832042	618.183948	426.986429	400.381143	486.342025
1.55E-06	3651.03507	3730.6751	3835.52778	4697.92897	4619.93193	4494.20366
1.33E-05	8372.64893	8457.40423	8936.29196	10584.0879	11594.5326	10095.2814
0.00648507	1952.56407	2134.85805	2200.97377	1781.26662	1881.40265	1618.81309
0.0011275	2976.84146	3323.7311	3418.42782	4115.67475	4181.65063	3624.68307
1.38E-46	9368.69376	9643.08138	9944.6983	19938.9723	18809.1675	17637.849
2.61E-17	4983.61205	4990.92314	5205.28803	8136.46362	7811.31949	7126.81105
2.65E-05	2068.88223	2044.30768	1672.3817	1428.67934	1216.69221	1494.70667
3.66E-06	14474.2705	12991.3143	16461.0141	20693.7463	26779.862	18888.9968
2.36E-12	1657.81611	1590.49055	1498.17527	2334.40813	2230.27846	2535.64925
4.46E-12	2718.23118	2790.01659	3050.10567	4114.5965	4314.78708	3917.88448
3.01E-05	3116.87497	3035.03522	2871.91738	3670.35809	3636.47145	3636.31805
[truncated: 53,585 more chars]
