# Supplementary material for: ASAH1 facilitates TNBC by DUSP5 suppression-driven activation of MAP kinase pathway and represents a therapeutic vulnerability
Source: Cell Death Dis. 2024 Jun 26;15(6):452. doi: 10.1038/s41419-024-06831-2 (PMC11208621; doi:10.1038/s41419-024-06831-2)

Supplemental Data 1

Raw blots for Fig. 1D

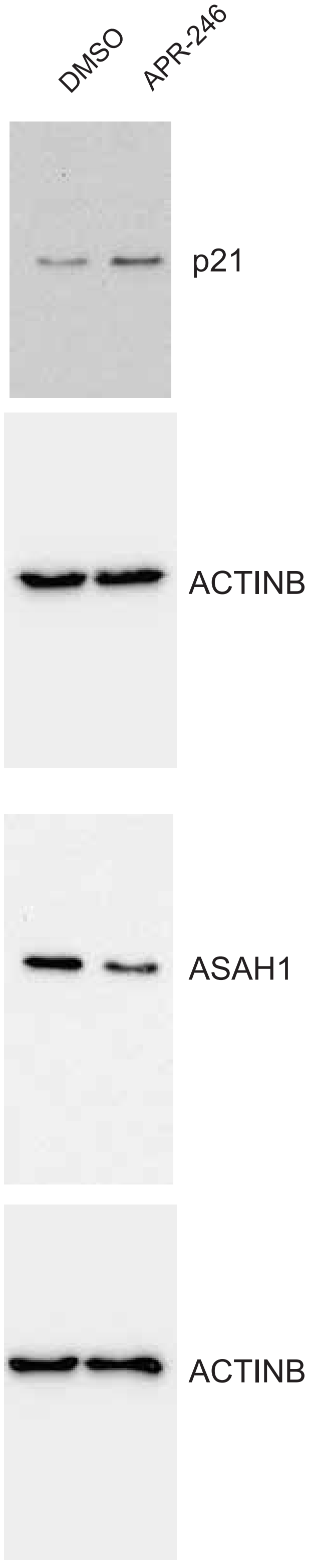

Raw blots for Fig. 1H

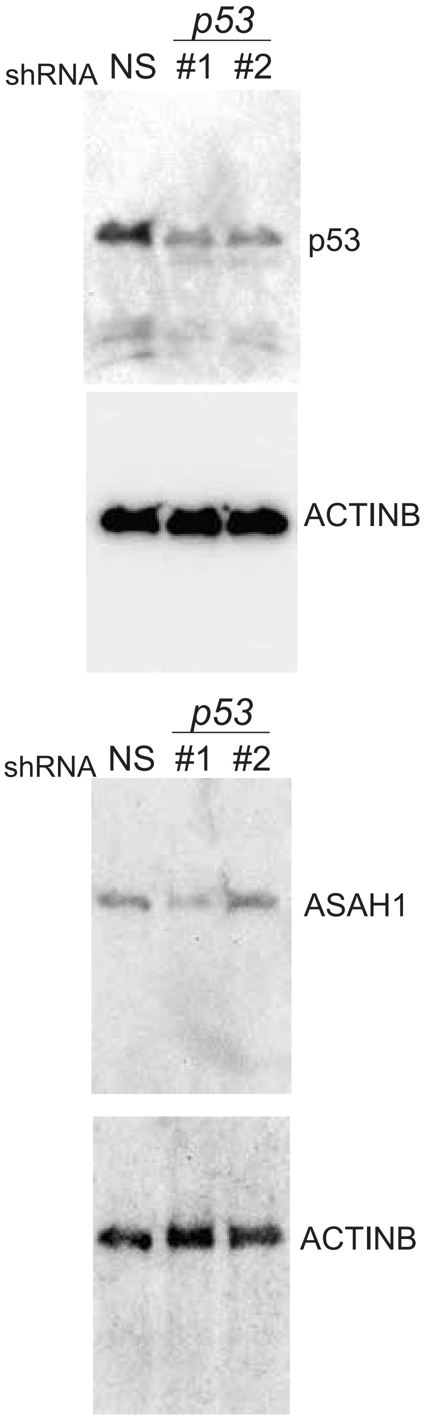

Raw blots for Fig. 1L

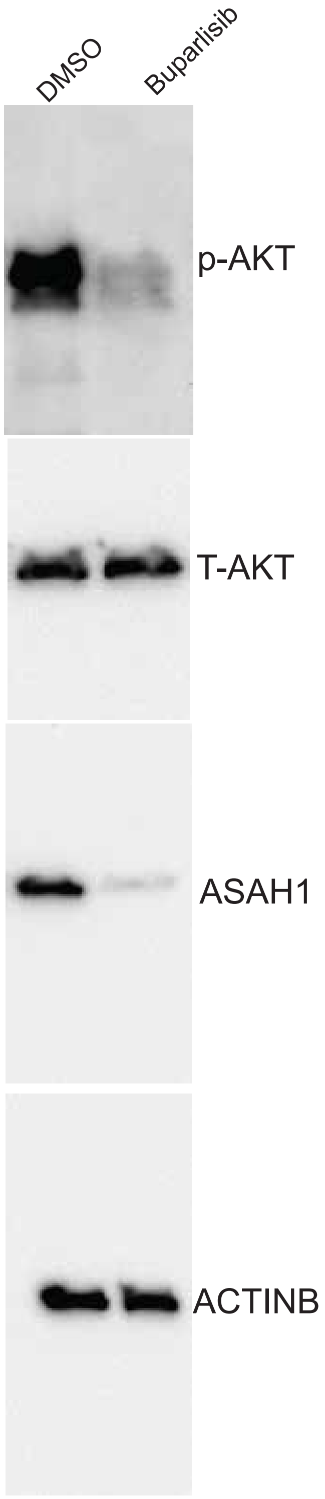

Supplemental Data 2

Raw blots for Fig. 2A

MDA-MB-231

ASAH1
shRNA NS #1 #2

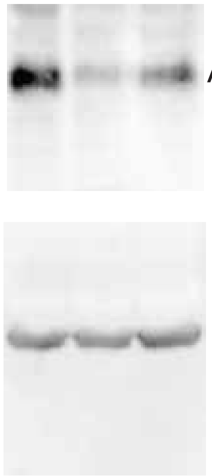

MDA-MB-468

ASAH1
shRNA NS #1 #2

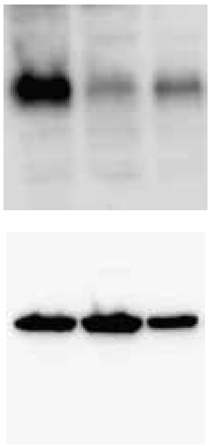

Raw blots for Supplementary Fig. 2A

BT-549

ASAH1
shRNA NS #1 #2

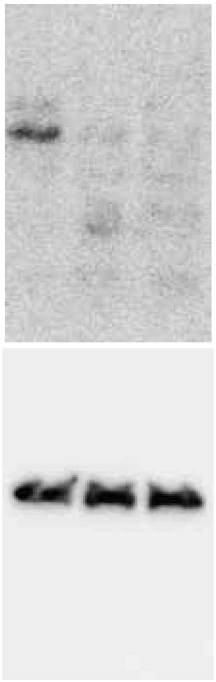

Raw blots for Supplementary Fig. 2D

DU-445

ASAH1
shRNA NS #1 #2

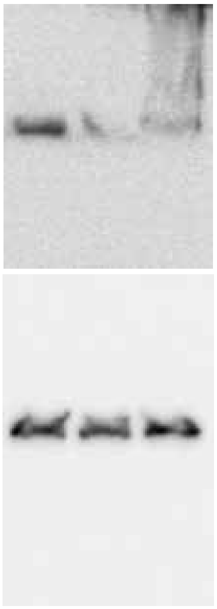

Raw blots for Supplementary Fig. 2G

MDA-MB-453

ASAH1
shRNA NS #1 #2

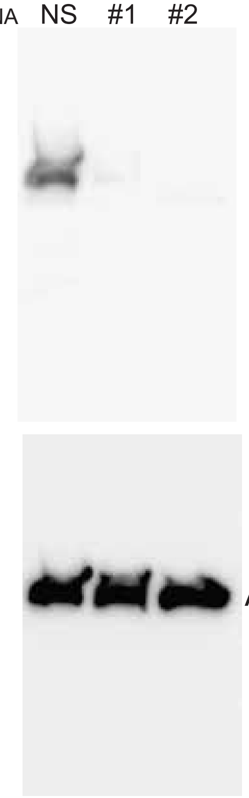

Raw blots for
Supplementary Fig. 5

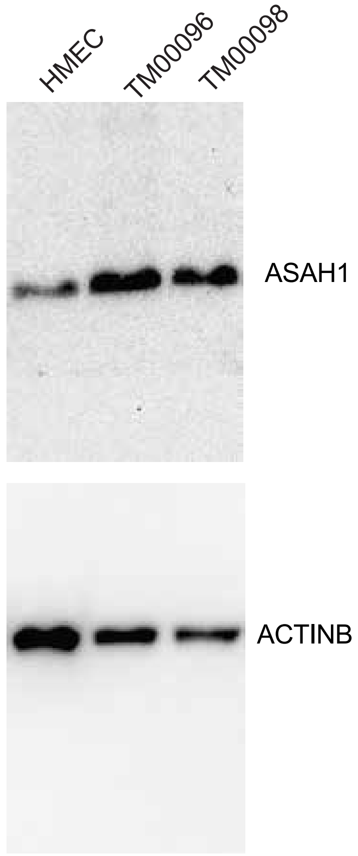

Supplemental Data 4

Raw blots for Fig. 6F

MDA-MB-231

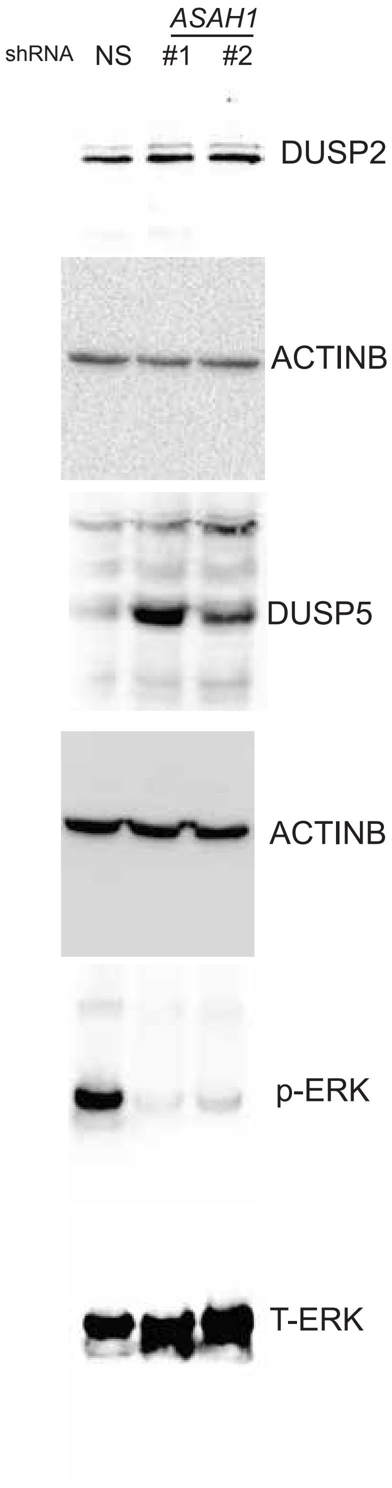

Raw blots for Fig. 6G

MDA-MB-468

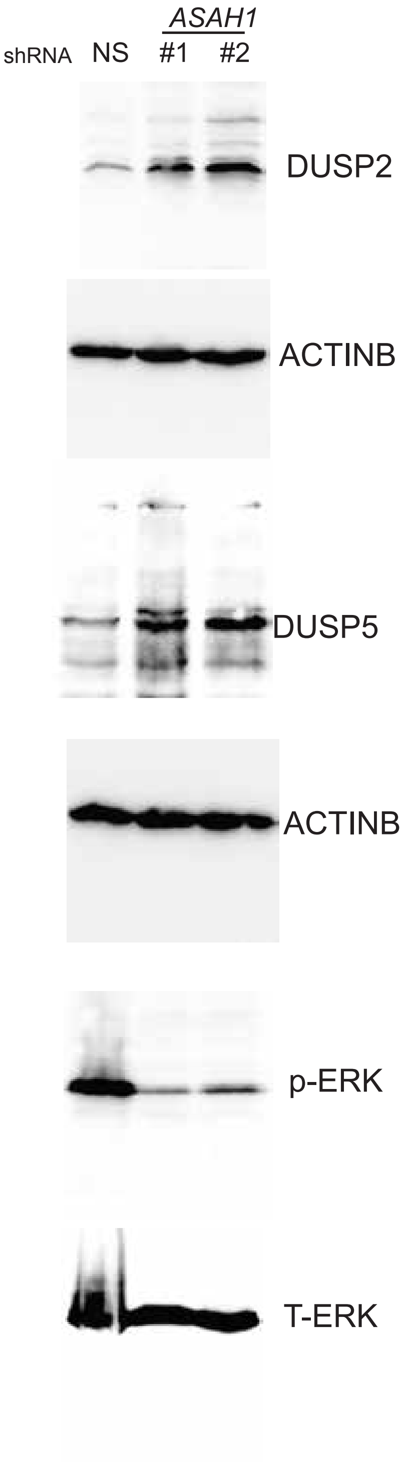

Raw blots for Fig. 6H

MDA-MB-231

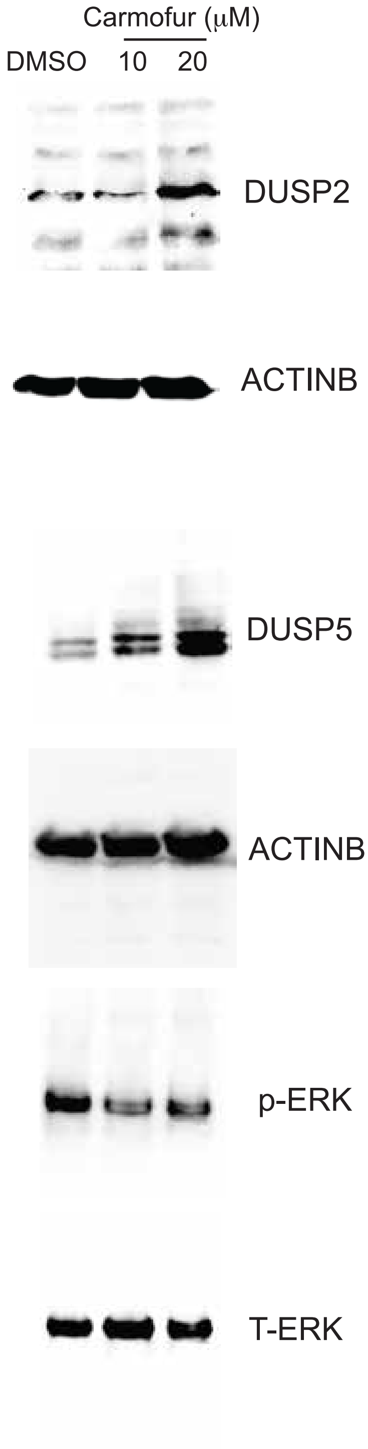

Raw blots for Fig. 6I

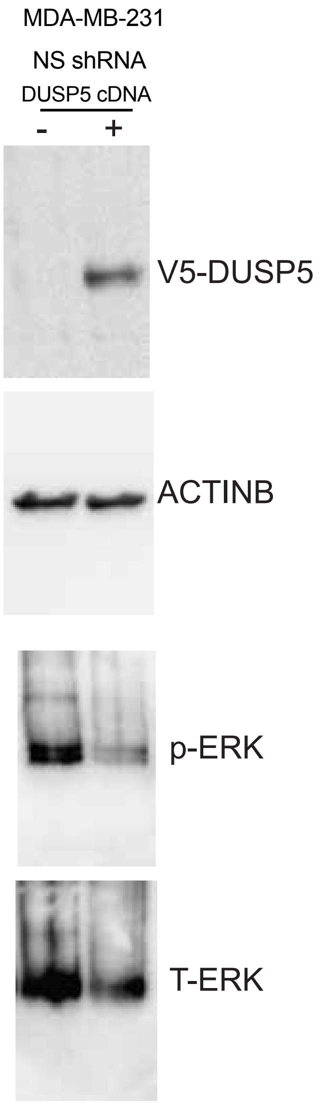

Raw blots for Fig. 6J

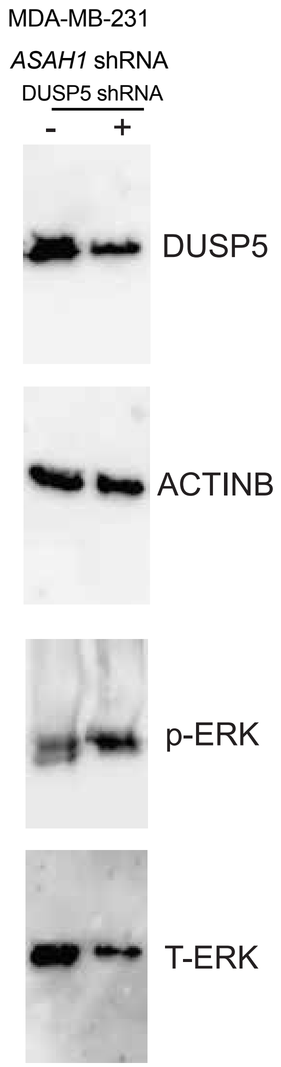

Raw blots for Supplementary Fig. 7A

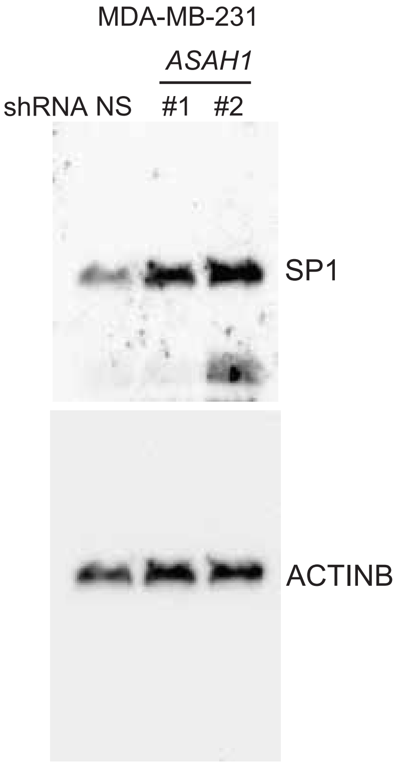

Raw blots for Supplementary Fig. 7B

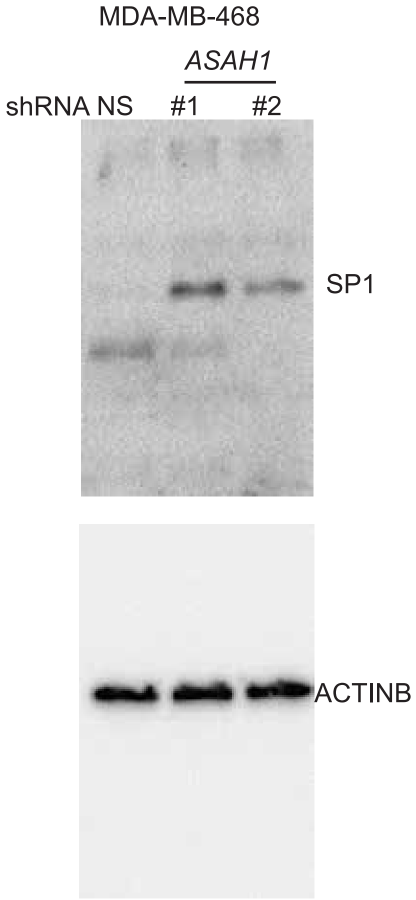

Raw blots for Supplementary Fig. 7C

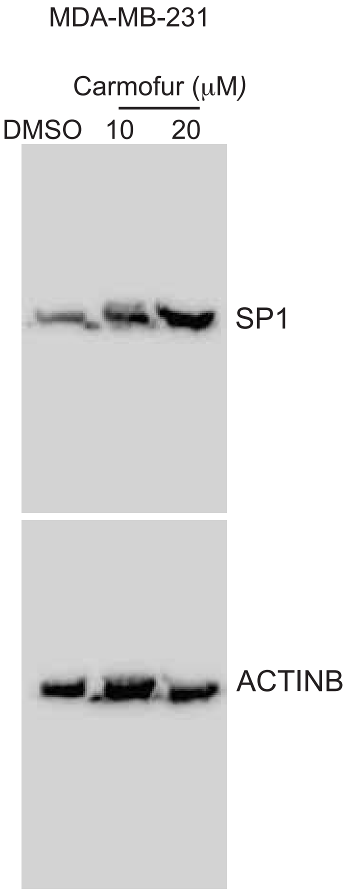

Raw blots for Supplementary Fig. 7D

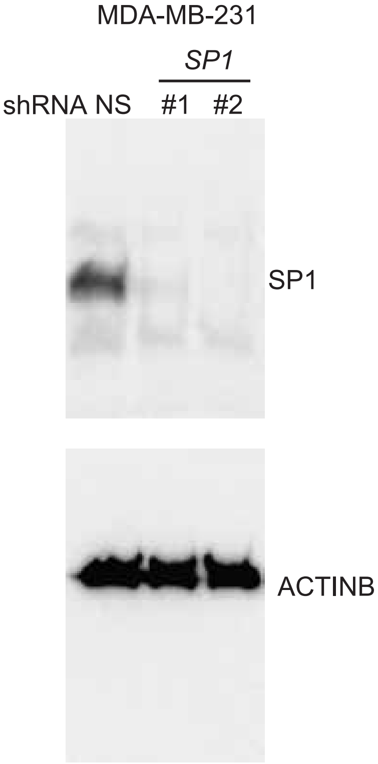

Raw blots for Supplementary Fig. 7D

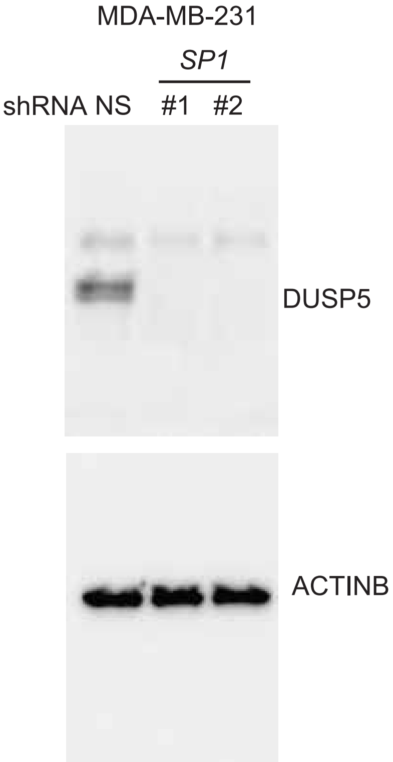

Raw blots for Supplementary Fig. 7E

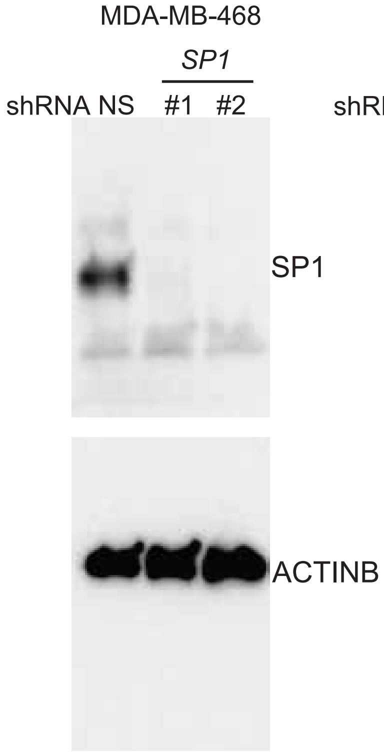

Raw blots for Supplementary Fig. 7E

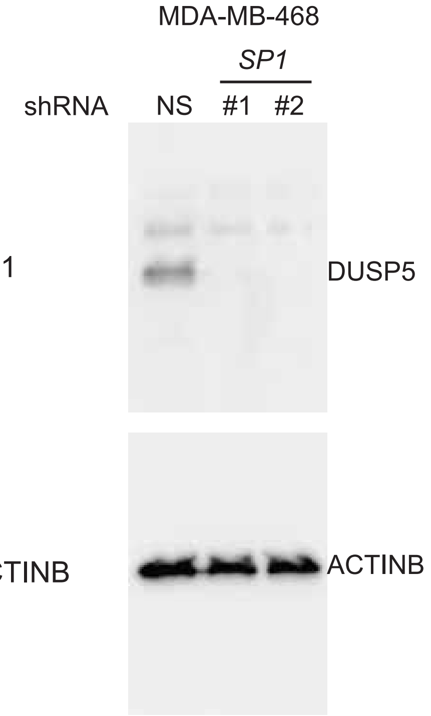

Supplement: Supplementary file 2 — Raw western blot data [file 41419_2024_6831_MOESM2_ESM.pdf]
